# Proteomic analysis in primary T cells reveals IL-7 alters T cell receptor thresholding via CYTIP/cytohesin/LFA-1 localisation and activation

**DOI:** 10.1042/BCJ20210313

**Published:** 2022-02-04

**Authors:** Rayner M. L. Queiroz, Siân C. Piper, Johanna S. Rees, Sam Strickson, Emmanuel Briend, Choon Pei Low, G. John Ferguson, Kathryn S. Lilley, Antony P. Jackson, Donna K. Finch

**Affiliations:** 1 Department of Biochemistry, University of Cambridge, Tennis Court Road, Cambridge CB2 1QW, U.K.; 2 Cambridge Centre for Proteomics, University of Cambridge, Tennis Court Road, Cambridge CB2 1QR, U.K.; 3 Bioscience Asthma, Research and Early Development, Respiratory and Immunology (R&I), BioPharmaceuticals R&D, AstraZeneca, Cambridge, U.K.; 4 BioPharmaceutical R&D, AstraZeneca, Cambridge, U.K.; 5 Biologic Therapeutics 3, Antibody Discovery & Protein Engineering, R&D, AstraZeneca, Cambridge, U.K.; 6 Translational Science and Experimental Medicine, Research and Early Development, Respiratory and Immunology (R&I), BioPharmaceuticals R&D, AstraZeneca, Cambridge, U.K.; 7 Research and Early Development, Respiratory and Immunology (R&I), BioPharmaceuticals R&D, AstraZeneca, Cambridge, U.K.

**Keywords:** CYTIP/cytohesin, IL7, LFA, phosphoproteomics, SPPLAT, TCR

## Abstract

The ability of the cellular immune system to discriminate self from foreign antigens depends on the appropriate calibration of the T cell receptor (TCR) signalling threshold. The lymphocyte homeostatic cytokine interleukin 7 (IL-7) is known to affect TCR thresholding, but the molecular mechanism is not fully elucidated. A better understanding of this process is highly relevant in the context of autoimmune disease therapy and cancer immunotherapy. We sought to characterise the early signalling events attributable to IL-7 priming; in particular, the altered phosphorylation of signal transduction proteins and their molecular localisation to the TCR. By integrating high-resolution proximity- phospho-proteomic and imaging approaches using primary T cells, rather than engineered cell lines or an *in vitro* expanded T cell population, we uncovered transduction events previously not linked to IL-7. We show that IL-7 leads to dephosphorylation of cytohesin interacting protein (CYTIP) at a hitherto undescribed phosphorylation site (pThr280) and alters the co-localisation of cytohesin-1 with the TCR and LFA-1 integrin. These results show that IL-7, acting via CYTIP and cytohesin-1, may impact TCR activation thresholds by enhancing the co-clustering of TCR and LFA-1 integrin.

## Introduction

Homeostatic cytokines such as interleukin-7 (IL-7) have been implicated in the aetiology of autoimmune disease. For example, elevated levels of IL-7 are associated with rheumatoid arthritis [1,2] and Sjorgren's disease [3], and an IL-7 receptor polymorphism has been implicated in susceptibility to multiple sclerosis [4,5]. Insights from autoimmune models such as the non-obese diabetic mouse [6,7] or collagen-induced arthritis mouse model [8] suggest that IL-7 is an important mediator of T cell effector function, and recombinant IL-7 increases T cell responses [9]. IL-7 priming lowers the activation threshold of T cells by promoting activation of the ERK pathway [10]. This role in tuning T cell activation, in addition to its better-known role in maintaining T cell number [11], has made manipulation of IL-7 signalling an attractive aim for immunotherapies, including in cancer where treatment strategies that change T cell responsiveness in tumours have had a major impact on survival in recent years. However, more work is needed to describe and understand the signalling transduction, which leads to this effect.

Molecular localisation and phosphorylation events determine the TCR activation threshold and subsequent T cell proliferation [12]. Antigen-presenting cells (APCs) present antigen on MHC complexes to TCRs on T cells, forming a highly ordered, stable, *trans*-cellular interaction called the immune synapse (IS) [13]. On the T cell surface, the micro-clustering of TCR molecules promotes the *cis-*mediated recruitment and activation of additional T cell proteins, including the surface integrin, lymphocyte function-associated antigen 1 (LFA-1). The *trans*-mediated interaction of LFA-1 on the T cell, with intercellular adhesion molecule-1 (ICAM-1), on the opposing APC, is essential for the formation of a stable and productive IS. Activated integrins recruit cytoskeletal-binding proteins bound to F-actin on the intracellular face of the plasma membrane. This results in concentric supramolecular clusters surrounded by an ‘actin cloud’ [14], promoting the prolonged association of APCs and T cells and facilitating signal transduction into the activated T cell. The formation of the IS is regulated both by antigen affinity and the presence of co-stimulatory factors. Once this activation is complete, the T cell and APC disengage, allowing the activated T cells to proliferate [15].

When considering how IL-7 could impact this activation threshold process, it is interesting to note that IL-7 has been reported to increase the adhesion of LFA-1 to ICAM-1 [16,17]. Hence, IL-7 could influence the *cis*-interactions within the T cell-associated IS. Here, we used two quantitative proteomic methods to investigate whether IL-7 modulates TCR organisation in primary human T cells, in the presence or absence of cross-linking anti-CD3. Firstly, we investigated total and phospho-proteomic changes and secondly, we used selective proteomic proximity labelling assay using tyramide (SPPLAT), a proteomic proximity labelling method [18,19], to assess changes in the immediate molecular neighbourhood of the TCR under these conditions. We identified a novel phosphorylation site (Thr280) on the integrin-binding, cytosolic adaptor, cytohesin interacting protein (CYTIP) and show that IL-7 stimulated the dephosphorylation of this threonine residue over a time course that correlated with concomitant changes in CYTIP/cytohesin localisation, LFA-1 activation and actin cytoskeletal rearrangement. Taken together, these IL-7-induced changes would be expected to result in the increased stability of the IS.

## Results

### IL-7 priming alters TCR activation threshold

**Figure 1.**
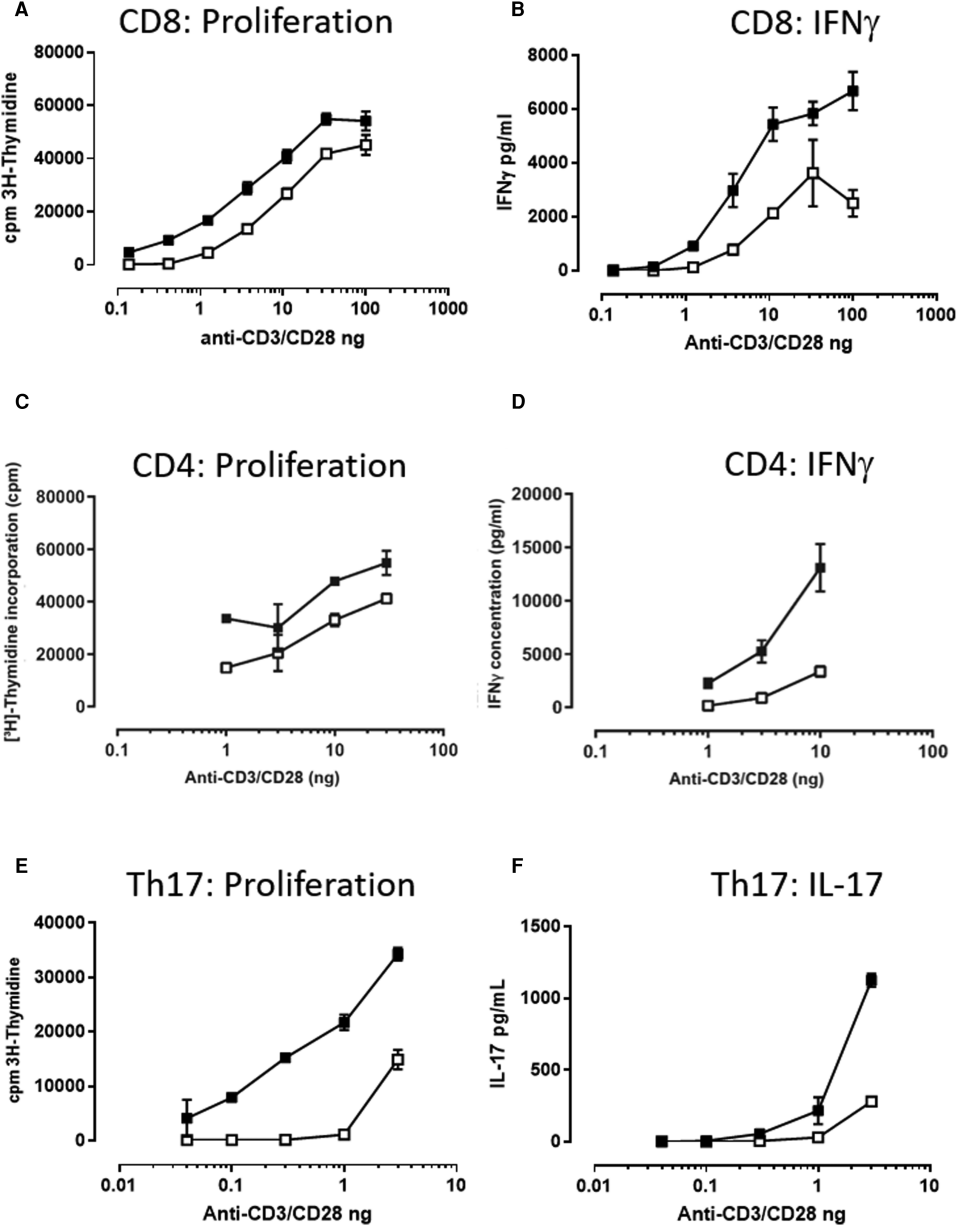
IL-7 increases the functional response of sub-optimally stimulated CD4^+^, CD8^+^ and Th17 T cell populations. Cells were co-stimulated with plate-coated anti-CD3 and soluble anti-CD28 in the presence (closed symbols) or absence of (open symbols) 10 ng/ml IL-7 for 72 h. Total CD8^+^ T cells (isolated by CD8 negative selection) proliferated more (**A**) and produced more IFNγ (**B**) in response to sub-maximal TCR stimulation, in the presence of IL-7. Total CD4^+^ T cells (isolated by CD4 negative selection) proliferated more (**C**) and produced more IFNγ **(D**) in response to sub-maximal TCR simulation in the presence of IL-7. Th17 cells, isolated by negative CD4 selection followed by positive CCR6 isolation, were stimulated with anti-CD3/CD28 proliferated to a larger degree (**E**) and produced more IL-17 (**F**) in the presence of 10 ng/ml IL-7. Th17 data representative of two independent experiments in two donors. CD4^+^ and CD8^+^ T cell data representative of four independent experiments in four donors.

It has previously been observed that a 24 h period of IL-7 priming rendered T cells more responsive to TCR activation by CD3 cross-linking and induced both increased ERK phosphorylation and the up-regulation of activation markers [10]. This suggests a common IL-7 priming impact on T cells. A similar increase in the proliferative response of CD4^+^ T cells was observed with the addition of IL-7 to phytohaemagglutinin (PHA)-stimulated cultures [9]. We were particularly interested in whether T-helper 17 cells (Th17) — a subset of CD4^+^ cells — had an increased response, since this T cell subtype exhibits higher expression of the IL-7 receptor, IL-7R α and has a key role in autoimmunity [20]. In common with previous observations, we showed that IL-7 mediated lowering of the activation threshold for TCR stimulation occurred across multiple T cell subtypes and this response was of a similar magnitude when measuring both proliferation and cytokine release (Figure 1). In the presence of IL-7, a lower concentration of CD3/CD28 stimulation was required to induce activation of total CD8^+^ cells (Figure 1A,B), total CD4^+^ (Figure 1C,D) and Th17 cells (Figure 1E,F). All T cell subsets tested responded similarly. So to investigate the signalling mechanism of this IL-7/T cell priming response we used total T cell populations in subsequent experiments. We undertook an untargeted evaluation of the total and phosphoproteomes after IL-7 stimulation.

The canonical IL-7 signalling pathway involves the phosphorylation of receptor proteins and associated proteins such as JAK1 and JAK3, culminating in the phosphorylation of residue Tyr-694 in the transcription factor STAT5A (or Tyr-699 in STAT5B). IL-7 induced phosphorylation on STAT5A and B reached a peak after 5–10 min of stimulation and decreased, until disappearing after 45 min [21]. However, the priming effect of IL-7 can be observed with an IL-7 incubation period of 24 h prior to TCR stimulation [10]. This suggests that the canonical IL-7 pathway is not directly involved. To observe signalling events associated with longer IL-7 stimulations, we incubated T cells in the presence or absence of IL-7 for 24 h prior to phosphoproteome analysis. We confirmed that T cell viability was minimally affected by their extended culture in the absence of IL-7, where it remained above 90% after 24 h (Supplementary Figure S1).

### Total and phospho-proteomic analysis of IL-7 priming in primary human T cells

To investigate the molecular mechanisms through which IL-7 priming lowers the activation threshold for TCR activation, we performed a large-scale undirected proteomic and phospho-proteomic characterisation of responses of human primary T cells to IL-7 stimulation. Briefly, total T cells isolated from PBMCs from five healthy individuals were incubated for 24 h in the presence or absence of 50 ng/ml IL-7. Using mass spectrometry, we measured the total proteome and phosphoproteome relative abundance changes in response to IL-7. Over 4100 protein identifications passed our initial high stringency for reliability criteria (false discovery rate (FDR) at peptide level less than 1%, and quantitation in at least three out of five donor samples) (Supplementary Table S1). In addition, over 2100 phosphopeptides presented high-reliability phospho-site assignment (pRS score above 75%, FDR at peptide level less than 1%, and quantitation in at least three out of five donor samples) (Supplementary Table S2). However, from these, only five proteins and 14 phosphorylation sites had significant abundance change between 24 h after IL-7 priming and unstimulated cells (Supplementary Table S3). As a control, we also assessed the total proteome in parallel, in the same samples. None of the proteins carrying IL-7-regulated phosphorylation sites presented statistically significant changes in the total proteome following the same trend, therefore, all were considered as reliable phosphorylation site regulations.

**Figure 2.**
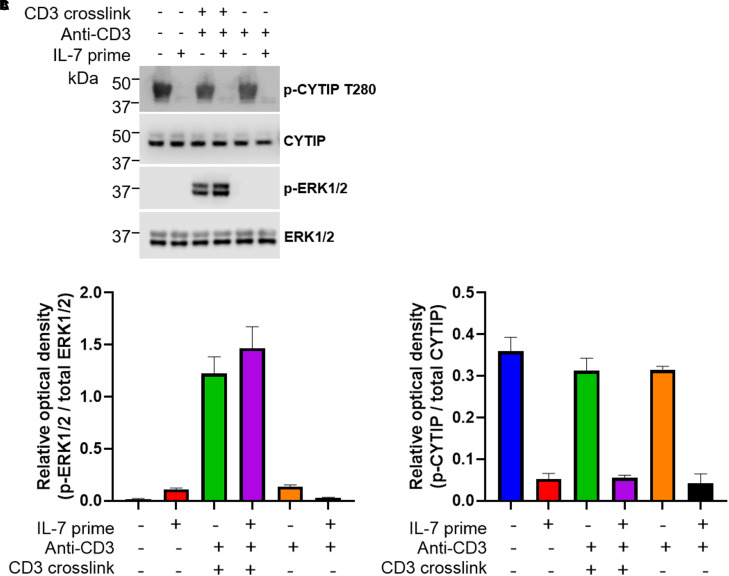
IL-7 priming causes dephosphorylation of CYTIP in primary human T cells. (**A**) Isolated T cells (CD3 negative isolation) were incubated overnight in the presence (+) or absence (−) of IL-7 before activation of the TCR (T cell receptor) by CD3 cross-linking for 8 min at 37°C. Cells were rapidly chilled and lysed. Cell lysates were subjected to SDS–PAGE before immunoblotting with antibodies recognising the total and phosphorylated (p-) forms of CYTIP and ERK1/2. One representative donor shown from two analysed. (**B**) Quantification of the relative p-ERK1/2/total ERK1/2, mean ± SEM of data from two donors plotted. (**C**) Quantification of the relative p-ERK1/2/total ERK1/2, mean ±  of data from two donors plotted.

None of the phosphorylation sites identified with differential abundance upon IL-7 stimulation has previously been associated with IL-7 signalling. Some of these phosphorylation sites occur in proteins with a potential link to TCR thresholding and/or molecular localisation to TCR. For example, the phosphorylation at Ser231 in the T cell surface glycoprotein CD8-α, a co-receptor of the TCR, and at Ser51 in RAS protein activator like-3 (RASAL3), a Ras GTPase-activating protein which represses TCR-stimulated ERK phosphorylation and is required for *in vivo* survival of peripheral naive T cells [22]. A novel phosphorylation site at position Thr280 within cytohesin interacting protein (CYTIP) was observed, which is dephosphorylated upon IL-7 stimulation. CYTIP is linked to the IS via its binding partner, cytohesin-1. This was further confirmed using Western blotting of cell lysates, with a novel phospho-specific antibody to CYTIP pThr280 (Figure 2; for generation and characterisation of this reagent see Experimental Procedures and Supplementary Figure S2). Analysis of the total proteomics data revealed that IL-7 stimulation did not affect total CYTIP abundance. We confirmed this with a Western blot of total CYTIP (Figure 2A).

### Combined IL-7 priming and TCR stimulation caused recruitment of activated integrin β2 to the TCR complex

Given that CYTIP influences the intracellular localisation of cytohesin-1 [23], and cytohesin-1 impacts LFA-1 interaction and cytoskeletal rearrangements during leukocyte activation [24], we sought to investigate if IL-7 impacts the recruitment of these proteins to the TCR complex in anti-CD3 stimulated T cells. We used the proteomic proximity technique of SPPLAT to identify proteins within a few tens to hundreds of nanometres from CD3. In brief, the SPPLAT methodology utilises a target-specific antibody conjugated with horseradish peroxidase (HRP). This enzyme converts biotin-tyramide into an unstable free radical that covalently labels exposed aromatic protein residues such as tyrosine. Thus, proteins lying within a limited vicinity of the targeted surface protein — here CD3 — are selectively biotinylated. The biotinylated proteins are then enriched through streptavidin affinity pulldown and identified through mass spectrometry [18,19,25,26]. To our knowledge, this current work is the first use of such a technique in primary cells.

As with the functional experiments measuring proliferation and cytokine release (Figure 1), we optimised the conditions of CD3 cross-linking by measurement of p-ERK by flow cytometry (data not shown) for sub-maximal TCR activation and a clear IL-7 priming effect. We determined the optimal biotinylation time for the SPPLAT experiment by performing the tyramide-biotin incubations for different time points. We assessed the degree of and cellular localisation of resulting biotinylated proteins by subsequent staining with Alexa-fluor conjugated streptavidin and imaging by confocal microscopy (Supplementary Figure S3) and compared with SPLATT negative controls (Supplementary Figure S4). The resulting stimulation time was 8 min of CD3 cross-linking, followed by 2 min tyramide-biotin incubations, allowing for measurements in changes occurring very early in the TCR signalling pathway.

The method was made quantitative by comparison with an internal standard to report the relative abundance of proteins, as described by Geiger *et al.* [27]. For these experiments, we used stable isotope labelling with amino acids in cell culture (SILAC). Ideally, here the spiked-in heavy-SILAC control would be a SILAC-labelled T cell extract. This, however, requires large numbers of cells, which could only be achieved by large-scale *in vitro* expansion of primary T cells by antigenic stimulation, and which is known to induce changes in protein expression, such as loss of IL-7R α expression [28–31]. Therefore, each of the SPPLAT T cell lysates were spiked with an equivalent total protein amount of heavy-SILAC Jurkat (cultured T cell line) cell lysate. Proteins that bound non-specifically to the neutravidin resin were identified by their light/heavy isotope ratio ∼1 whereas proteins with light/heavy ratios significantly greater than 1 were considered noteworthy. There were limitations using SILAC-labelled Jurkat quantification in this manner, since about half of the identified peptides per condition were absent in the Jurkat control after neutravidin enrichment (Supplementary Table S4). Nonetheless, we were able to identify 48 biotinylated proteins that were reliably enriched (Supplementary Table S5), from which 19 were not identified in SPPLAT controls (named conditions E and F, which comprise of non-specific surface biotinylation and endogenous biotinylation). Nine proteins were identified in all three donors in T cells stimulated with both IL-7 and by anti-CD3, TCR cross-linking, and not detected in any other stimulated condition or negative controls. These proteins were therefore attributable to the IL-7 priming when the TCR is sub-maximally stimulated (Supplementary Table S5). Four of these proteins presented over 12-fold enrichment compared with the SILAC Jurkat control, namely Calcium load-activated calcium channel, activating transcription factor 7-interacting protein 2, CD44 antigen and Integrin β2 (CD18).

We further investigated Integrin β2 since T cells can express 12 of the 24 known integrin heterodimers, including four containing β2 integrin (αLβ2, αMβ2, αXβ2, αDβ2). We note in particular that αLβ2 (LFA-1) is the most abundant and widespread in expression [32] and plays a crucial role in the formation of the IS [33,34]. The detection of Integrin β2 in proximity to the TCR by SPPLAT only after IL-7 priming and TCR activation therefore suggests that in this condition, the integrin is activated. In the inactive form, the α subunit is bent around the β subunit, which restricts its availability for the SPPLAT technique [35]. We confirmed from published 3D structures (PDB: 5ES4) that two surface-exposed tyrosine residues on integrin β2 are accessible to SPPLAT biotinylation only following integrin activation (data not shown).

### IL-7 priming facilitates LFA-1 activation for enhanced T cell activation

**Figure 3.**
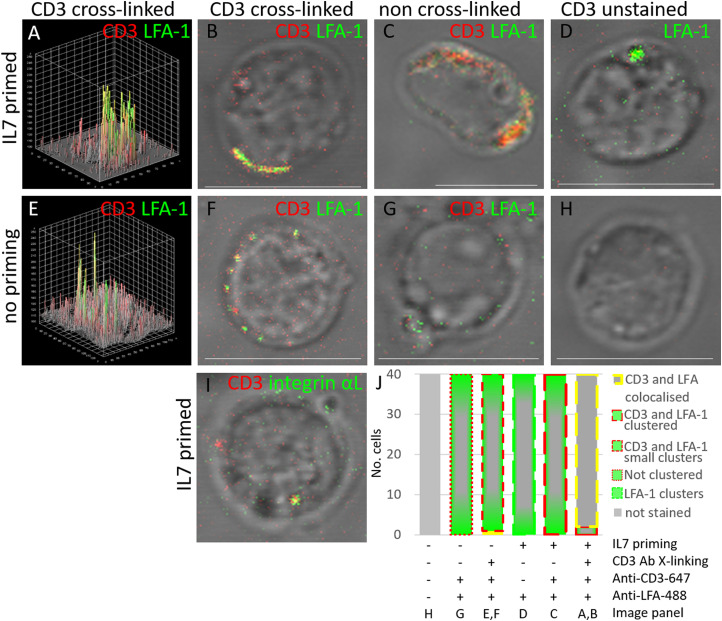
Co-stimulation with IL-7 and CD3 cross-linking is required for clustering of CD3 and LFA-1 on T cell membranes. Isolated T cells (CD3 negative isolation) were incubated overnight in the presence (+) or absence (−) of IL-7 before activation of the TCR (T cell receptor) by CD3 cross-linking for 8 min, 37°C. (**A**–**H**) Cells were stained for CD3 (red) and/or the activated form of LFA-1 (green). (**A**,**B**) Cells primed with IL-7 and subjected to CD3 cross-linking. The 3D cell surface plot in (**A**) is for the image shown in (**B**). (**C**) Cells primed with IL-7 but without subsequent CD3 cross-linking. (**D**) Cells were primed with IL-7 but without subsequent CD3 cross-linking and stained only for LFA-1. (**E**,**F**) Cells stimulated by CD3 cross-linking, but without IL-7 priming, (**E**) 3D cell surface plot for the image shown in (**F**). (**G**) Cells not treated with IL-7 or CD3 cross-linking, but were stained for CD3 and LFA-1. (**H**) Untreated, unstained control samples imaged with both channels. (**I**) Cells were primed with IL-7 and CD3 cross-linked and stained for CD3 (red) and Integrin αL (green). (**J**) Histogram quantifying the proportions of cells exhibiting different phenotypes of membrane localisation and clustering of CD3 and LFA-1 in cells imaged under the conditions in (**A–H**): LFA-1 (green–grey bar), with either membrane-bound CD3 in large clusters of greater than 1 µm in diameter (long red dashed) (represented by **C**), or small dispersed clusters of less than 1 µm in diameter (medium red dash) (represented by **E**,**F**), or no clustering with LFA-1 (small red dash) (**G**), LFA-1 only clustering at the membrane (large green dash) (**D**) or co-localised CD3 and LFA-1 at the membrane (grey filled bar with yellow dash boarder) (**A**,**B**). 40 cells were manually counted from three frames imaged from each of two donors. Scale bars are 10μm.

We performed confocal microscopy experiments using the same IL-7 priming and sub-maximal CD3 cross-linking conditions as used for the SPPLAT experiments, but utilising fluorophore-conjugated antibodies rather than HRP-conjugated antibodies, and pelleting cells onto coverslips rather than proceeding with proteomic sample preparation. We stained the T cells with an antibody that recognises only the activated form of LFA-1, via the β2 subunit identified in our quantitative MS analysis [31], but also confirmed the presence of the Integrin αL subunit using and antibody recognising both active and inactive Integrin αL. Upon IL-7 priming and CD3 cross-linking, we observed co-localisation of LFA-1 with CD3 on the plasma membrane, together with a few regions of large CD3 and LFA-1 clusters (Figure 3A,B,J). Upon IL7 priming, but in absence of CD3 cross-linking, CD3 was uniformly dispersed, or in many large clusters (Figure 3C,J), rather than co-localised with the less abundant LFA-1 clusters, apparent in absence of CD3 staining (Figure 3D,J). With sub-maximal CD3 cross-linking, but without IL-7 priming, CD3 and LFA-1 were both located at the membrane but often scattered in several discrete small clusters (Figure 3E,F,J). In untreated cells, active LFA-1 was neither detected in abundance at the plasma membrane nor associating with CD3 (Figure 3G,J). Unstained control cells showed no fluorescent signals (Figure 3H,J). Finally, we used an antibody specific for the intracellular region of integrin αL, and which detects both inactive and active LFA-1, to show that in cells treated with both IL-7 and cross-linking anti-(CD3) antibody, integrin αL co-localised with the TCR complex (Figure 3I).

### IL-7 priming results in dephosphorylation of CYTIP, but does not affect interaction with cytohesin-1

**Figure 4.**
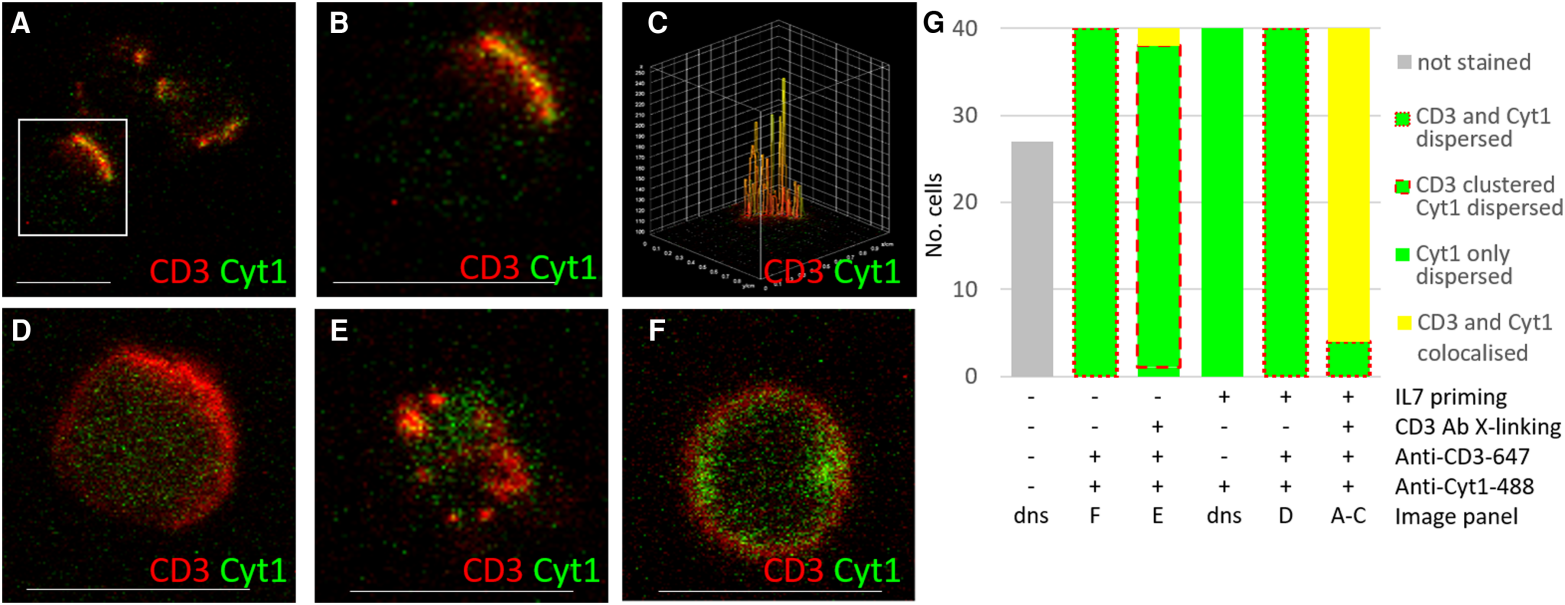
Co-stimulation with IL-7 and CD3 cross-linking is required for clustering of CD3 and cytohesin-1 on T cell membranes. Isolated T cells (CD3 negative isolation) in the presence (+) or absence (−) of IL-7 before activation of the TCR by CD3 cross-linking for 8 min, 37°C. Distribution of cytohesin-1 (green) and CD3 (red) when primed with IL7 and activated with anti-CD3, (**A**) showing three cells, (**B**) zoomed image of (**A**) and (**C**) 3D surface plot of (**B**) showing the extent of co-localisation, (**D**) IL7 primed but not cross-linked, (**E**) not IL7 primed but cross-linked, and (**F**) neither IL7 primed nor cross-linked, (**G**) Histogram to show the different cell phenotypes observed, typically dispersed cytoplasmic cytohesin (green fill bar) with membrane localised CD3 either continuous around the plasma membrane (peripheral dashed red) (represented by **D**,**F**), or in small clusters (medium dashed red) (represented by **E**), or not dispersed Cyt1, instead large areas of clustering co-localised with CD3 (yellow fill bar) (**A**–**C**) in the six different conditions indicated. dns (data not shown). Cells counted using two donors, and imaging three frames per donor. Scale bars are 10 μm.

**Figure 5.**
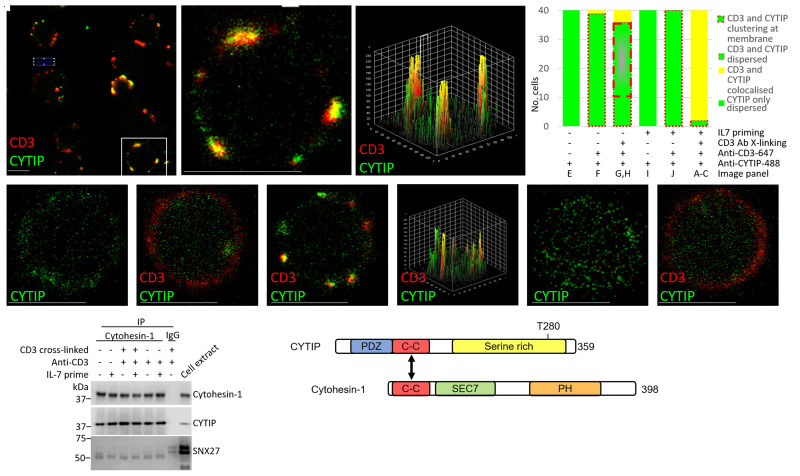
IL-7 mediated dephosphorylation of CYTIP does not impact on its interaction with cytohesin-1 or cellular localisation. (**A**–**C**) Co-localisation and clustering of membrane-bound CD3 (red) and cytoplasmic CYTIP (green) upon 24 h IL-7 priming and 8 min CD3 cross-linking (TCR activation) at low magnification, *n* = 9 cells (**A**), inset digitally zoomed (**B**) and (**C**) surface plot of co-localisation of (**B**). (**D**) Histogram to show the different cell phenotypes observed, typically dispersed cytoplasmic CYTIP (green fill), represented by (**E**,**I**), and, with either ubiquitous membrane localised CD3 (peripheral dashed red), represented by (**F**,**J**), or CD3 and translocated CYTIP clustering at the membrane upon cross-linking (grey bar with green border and thick peripheral dashed red), represented by (**G**,**H)**, or large clusters of co-localised CD3 with translocated CYTIP at the membrane (yellow fill) (**A**–**C**,**G**) under the different conditions: ±L7, ±TCR activation by cross-linking using CD3 Antibody, ±anti CD3-647. (**E**,**F**) No IL-7 priming or TCR activation (E also negative control for anti CD3-647), (**G**) No IL-7 priming, but with TCR activation by CD3 cross-linking, (**H**) showing the surface plot of co-localisation of (**G**). (**I**) IL-7 primed, but no TCR activation (also negative control for anti CD3-647) and CD3 located to membrane (**J**). (**K**) Western blot showing cytohesin-1 immunoprecipitated from the cell extracts to detect association with CYTIP and SNX27, alongside IgG acting as a negative control. (**L**) Schematic of the functional domains comprising CYTIP and cytohesin. Forty cells were manually counted from three frames imaged from each of two donors. Scale bars are 5 μm.

Given the dephosphorylation of CYTIP, we hypothesised that cytohesin-1 may also be implicated in IL-7/TCR pathway cross-talk. We explored whether IL-7 mediated dephosphorylation of CYTIP at Thr280 affects its ability to interact with cytohesin-1. We found strong membrane clustering of cytohesin-1 and co-localisation with CD3 in cells stimulated with IL-7 and CD3 cross-linking (Figure 4A–C,G). However, in cells stimulated with IL-7 alone; CD3 cross-linking alone or unstimulated, cytohesin-1 was largely located in the cytoplasm (Figure 4D–G), while a few cells showed accumulation of cytohesin-1 at the plasma membrane (Figure 4E,G). Controls showed no non-specific staining (Figure 4G). CYTIP also co-localised with CD3 at the membrane forming a few large clusters after both IL-7 priming and CD3 cross-linking (Figure 5A–C,D) compared with no priming or cross-linking (Figure 5D,E,F). In unstimulated cells, CD3 cross-linking alone resulted in a proportion of CYTIP translocating to the plasma membrane, from its cytoplasmic location, as observed by small membrane localised clusters, some of which co-localised with CD3 (Figure 5D,G,H). However, IL-7 alone caused no membrane translocation or clustering of CYTIP (Figure 5D,I,J).

These results were unexpected as CYTIP has been implicated in the sequestration of cytohesin-1 to the cytoplasm in inactivated cells [36]. In co-immunoprecipitation experiments using CYTIP and cytohesin antibodies, we confirmed that under all evaluated conditions, CYTIP remains bound to cytohesin-1, despite stringent washing with detergents and salt to ensure no non-specific binders (Figure 5K). The phosphorylation site maps remotely from the known binding interface between CYTIP and cytohesin-1 (Figure 5L), so we hypothesised that CYTIP dephosphorylation could result in a conformational change that allowed the CYTIP–cytohesin-1 complex to disengage from another cytoplasmic protein. Sorting Nexin 27 (SNX27) is the best described CYTIP binding partner, which could act in this capacity [37]. However, we could not demonstrate clear co-immunoprecipitation of SNX27 with the CYTIP/cytohesin-1 complex, with our stringent procedures, in any of the stimulation conditions, although it could be detected in the total cell extract (Figure 5K).

### Combined IL-7 priming and CD3 cross-linking promotes actin polarisation and IS formation

**Figure 6.**
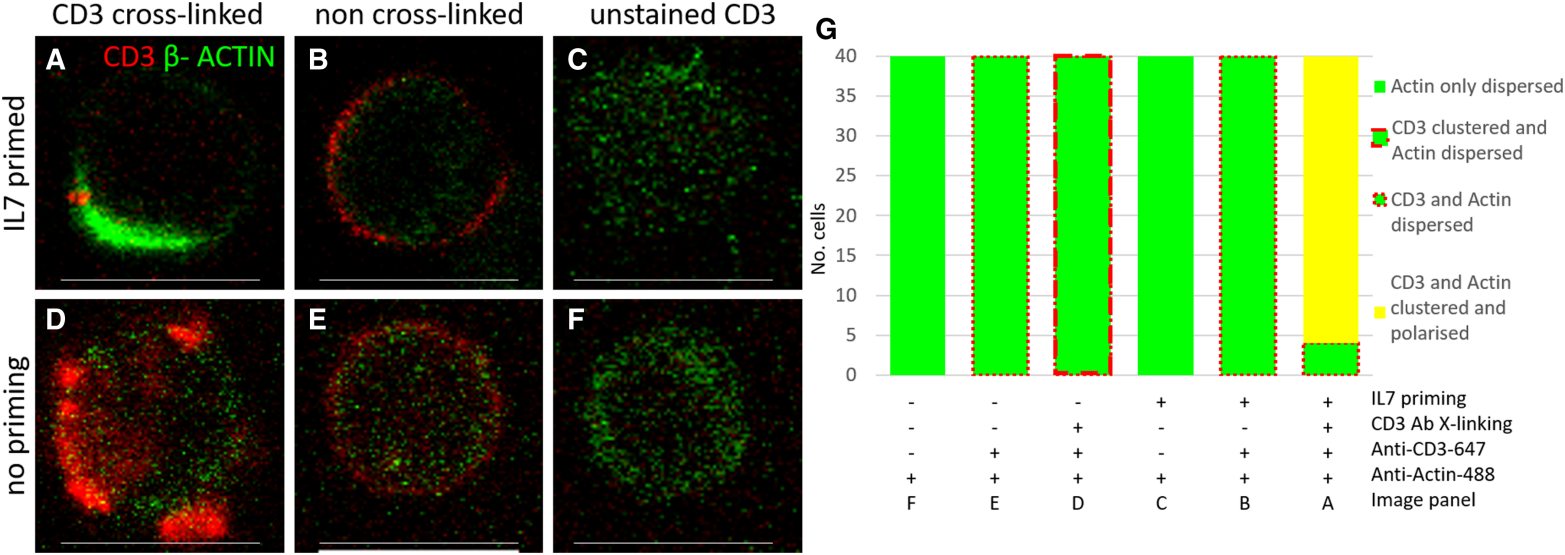
Co-stimulation with IL-7 and CD3 results in actin polarisation. (**A**) Polarisation and/or clustering of β-Actin (green) with CD3 (red) when primed with IL7 for 24 h and CD3 activation by cross-linking with anti-CD3 for 10 min, as shown by dense green areas that obscure the CD3 signal, (**B**) IL7 primed, but not CD3 activated, (**C**) no anti-CD3-647 control, (**D**) CD3 activation but no IL7 priming, (**E**) no priming or activation, (**F**) no anti-CD3-647 control. (**G**) Histogram indicating the proportion of different cell phenotypes observed, typically dispersed cytoplasmic actin (green fill) with either membrane localised CD3 (peripheral dashed red), represented by (**B**,**E**), or CD3 cross-linked induced clustered CD3 (thick peripheral dashed red), represented by (**D**), or polarisation (yellow bar) where cytoplasmic actin (green) recruits to the membrane, represented by (**A**), under the different conditions: ±IL7, ±TCR activation by cross-linking using CD3 antibody, ±anti CD3-647 as indicated below. Forty cells were counted manually from three frames imaged from each of two donors. Quantitation was performed using single channel images as the Actin-488 was dominant in some conditions. Scale bars are 10 μm.

We investigated cytoskeletal appearance, as the formation of the actin cloud within the IS contributes to lowering of the T cell activation threshold [38]. We observed strong polarity of actin only after combined IL-7 priming and sub-maximal TCR activation (Figure 6A,G), whereas polarity, or actin clustering, was absent without CD3 cross-linking or IL-7 priming (Figure 6B–G), however, as expected CD3 clustering was seen with cross-linking alone (Figure 6D,G). It is known that the stimulation of T cells with immobilised anti-TCR antibody results in T cell spreading and actin rearrangement [39–41]. Our results demonstrated that actin cloud formation as early as 10 min post CD3 cross-linking is observed upon IL-7 priming at sub-maximal TCR activation, but not with TCR sub-maximal activation alone, which strongly indicates a sensitisation of the process by IL-7.

## Discussion

The ability of the immune system to discriminate self from foreign antigens depends on the appropriate calibration of the TCR signalling threshold. Understanding these events is highly relevant in the context of autoimmune diseases and cancer immunotherapy. IL-7 priming of human T cells is known to strengthen TCR signalling and T cell activation, and this stimulation occurs upstream of induction of ERK phosphorylation [10,42,43], but many of the biochemical mechanisms underlying this early sensitisation are unclear. Given these uncertainties, we used a comprehensive high-throughput quantitative proteomic workflow to examine how IL-7 priming impacts events at time points earlier than the ERK signalling. We used sub-maximal TCR activation conditions with and without IL-7 priming to identify close molecular neighbours of the CD3 component of the TCR. Importantly, all work was done in uncultured human primary T cells, rather than tissue culture-adapted or over-expressing engineered cell lines. We employed highly stringent criteria for identification, quantitation and interpretation of the proteomics data, which encompassed several donors per condition and then validated findings using standard biochemical methods. Since direct SILAC-labelling could not be used with primary cells [18], we used a SILAC-labelled Jurkat cell line to provide relative quantitation of selected proteins. The success of this new approach illustrates the potential of SPPLAT as a general proteomic tool in primary cells, where a model cell line with overlapping total proteome exists.

These experiments identified changes in the immediate neighbours of the TCR receptor that are induced by IL-7 priming combined with sub-maximal TCR stimulation. The integrin β-subunit 2 (ITGB2 or CD18) passed our high stringency criteria for abundance change in all three donors in T cells stimulated with both IL-7 and TCR cross-linking compared with TCR cross-linking in the absence of IL-7 priming. ITGB2 provides the specific β-subunit for LFA-1 [32]. We confirmed by confocal microscopy that IL-7 priming induced an increase in the membrane clustering of activated LFA-1 induced by CD3 cross-linking. Whilst the involvement of LFA-1 in TCR activation, and IL-7 altering adhesion via LFA-1 are not new concepts [16,44] the use of the SPPLAT methodology enabled us to demonstrate this mechanism of IL-7 priming of LFA-1 activation occurs in cells within 10 min of TCR activation.

When comparing the phosphoproteomes of cells incubated with and without IL-7 we observed abundance changes in 14 phosphorylation sites within 5 proteins, which have not previously been ascribed to T cell activation or IL-7. Here, however, we have focussed on CYTIP. Although this protein is not regarded as a core member of either the TCR, IL-7 or LFA-1 pathways, IL-7 has been shown to increase the mRNA expression of CYTIP [45]. Furthermore, CYTIP is known to be a key regulator of cytohesin-1, which in its phosphorylated state stimulates ERK1/2 and the transcription factor activator protein 1 (AP-1) [23,37,38,46,47] and thus activates the ‘outside-in’ LFA-1 signalling pathway, lowering the T cell activation threshold [36,45]. We add to this understanding by showing that IL-7 stimulation did not lead to increased total protein abundance of CYTIP in primary T cells, despite previous reports of increased gene expression in haematopoietic cells [46]. Crucially, we show that IL-7 stimulated the dephosphorylation of CYTIP at position Thr280. To our knowledge, this residue has not been previously identified as a phosphorylation site in CYTIP. Using immunoprecipitation, we did not observe any changes in the association of CYTIP with cytohesin-1 that could be linked with Thr280 dephosphorylation. However, this dephosphorylation event did correlate with subsequent changes in CYTIP, cytohesin-1 and LFA-1 localisation in IL-7 primed CD3 cross-linked cells. CYTIP contains an N-terminal PDZ domain that can bind to cytoskeletal components, a helical coil-coiled domain and a C-terminal serine-rich region, containing the Thr280 residue. Cytohesin-1 contains an N-terminal coiled-coil domain, with which it binds to CYTIP, and a C-terminal PH domain that can bind to phosphatidylinositol lipids in the inner leaflet of the plasma membrane. Between these two domains lies a Sec7 domain that acts as a guanine nucleotide exchange factor for members of the Arf family of small molecular mass G proteins [23] (Figure 5L). Guanine nucleotide exchange catalysed by the Sec 7 domain is critical for the trans-cellular interaction of the T cell integrin LFA-1 with its trans-binding ICAM-1 partner [24].

**Figure 7.**
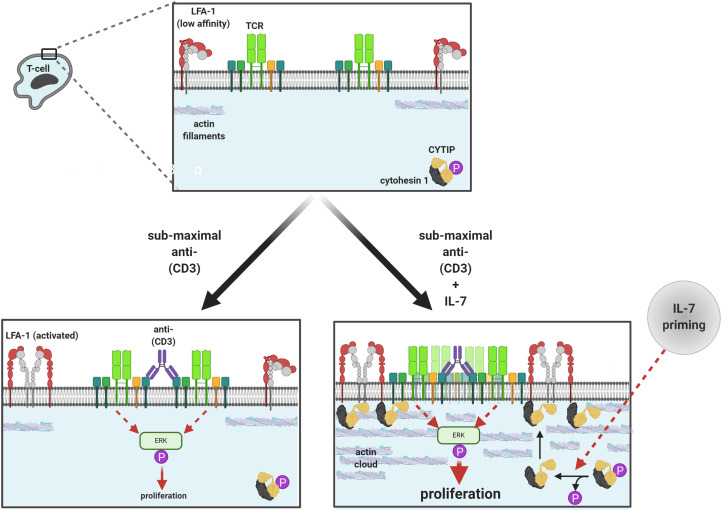
Proposed mechanism of the effects of IL7 priming and TCR activation by sub-maximal CD3 antibody cross-linking. TCR cross-linking activates proliferative signals via phospho-ERK. IL-7 priming stimulates the dephosphorylation of the CYTIP–cytohesin complex, which traffics to the plasma membrane (potentially due to disengagement from a cytoskeletal protein) and increases the efficiency of TCR and LFA-1 cross-linking, thus enhancing ERK signalling and T cell proliferation (Image created with BioRender.com).

Therefore, we hypothesise that dephosphorylation of Thr280 may trigger a conformational change in the CYTIP/cytohesin-1 complex, which confers a different exposure of PDZ, PH and/or Sec7 domains within the complex. Hence, pThr-280 may represent an important control switch for the CYTIP/cytohesin-1 complex in LFA-1 mediated T cell activation [23,47]. We speculate that the decreased phosphorylation at pThr280 not only could alter exposed domains in the CYTIP/cytohesin complex but alternatively or additionally, it could also impact the interaction of CYTIP with an unidentified mediator or scaffold protein, which in turn impacts the localisation of cytohesin-1 and LFA-1 and increases the efficiency of TCR and LFA-1 cross-linking (Figure 7). We attempted to co-immunoprecipitate SNX27 with the CYTIP/cytohesin 1 complex but antibodies failed. These hypotheses could be the basis of further studies.

It has also been shown that in dendritic cells (DCs), CYTIP controls the contact duration with T cells and therefore their ability to proliferate and expand [48] and that some viruses control DC-T cell interactions via degradation of CYTIP [49,50]. We have not addressed whether IL-7 has the same impact on this pathway in DCs (which can express IL-7R), nor whether this important cytokine-mediated priming mechanism can be impacted by viruses. This could be important to understand the overall impact of IL-7 on T cells and antigen processing under various scenarios. Additionally, it will now be interesting to identify the phosphatases that dephosphorylate pThr280 residues. These could be potential drug targets to modulate the effect of IL-7 on lowering the TCR threshold, without impacting haematopoiesis. We also note that in non-small cell lung cancer, hypomethylation of the promoter region of the *CYTIP* gene is associated with the prediction of responsiveness to programmed cell death protein 1 (PD1) [51]. Given PD1 is a checkpoint molecule associated with T cell activation, our data provides a complementary position as to how the IL-7 modification of CYTIP may influence TCR activation, which could theoretically impact PD1 responsiveness. Hence, our results provide new insight into the control of TCR signal strength and suggest new avenues for therapeutic intervention in autoimmune diseases and cancer.

## Experimental procedures

All antibodies used in this study are detailed in Supplementary Table S6.

### T cell isolation

PBMC were purified from blood (leukocyte cones) from healthy consented donors (UK National Blood Service) using Ficoll-Paque Plus (GE Healthcare). CD4^+^, CD8^+^ or total T cells were isolated using Easysep Human CD4^+^ or CD8^+^ T cell enrichment kit (negative selection) from resultant PBMCs (StemCell Technologies). Th17 cells were isolated from CD4^+^ T cells by positive selection using an anti-CCR6 antibody (BD Biosciences) and Easysep human ‘Do it yourself’ selection kit (StemCell Technologies).

### Generation of recombinant human IL-7

Human IL-7 (Uniprot sequence identity P13232-1) was tagged with carboxy-terminal His10 and expressed from a stably transfected Chinese Hamster Ovary (CHO) cell line. Purification used immobilised metal affinity chromatography from culture supernatant using HisTrap Excel (GE Healthcare), followed by size-exclusion chromatography using HiLoad Superdex75 16/60 PG (GE Healthcare) in 2× Dulbecco′s phosphate-buffered saline (DPBS).

### T cell functional assay

Flat 96-well plates (Corning) were coated with anti-CD3 (BD Bioscience) in PBS, 2 h, 37°C. After washing to remove unbound antibody, 1 × 10^5^ cells/well Th17, CD4^+^ or CD8^+^ T cells were seeded in X-VIVO-15 medium (VWR), with anti-CD28 (BD Bioscience) and IL-7 (Humanzyme or MedImmune) and cultured for ∼72 h. Further details are given in Supplementary Table S6. Supernatants were harvested and analysed with Human IL-17 tissue culture kit (Meso Scale Discovery) was used according to the manufacturer's instructions. Detection of IFNγ was performed by enzyme-linked immunosorbent assay (ELISA), as described previously [52]. For proliferation the cells were pulsed with 10 µCi/ml ^3^H-thymidine (PerkinElmer) for the final 6 h of culture, then filter harvested and read with scintillant (PerkinElmer; Topcount) according to standard procedures.

### Generation and testing of pCYTIP antibody

An anti-(phosphor-CYTIP) Thr280 polyclonal antibody was raised, by immunising a sheep with peptide SRQTST*DDE (where the asterisk indicates the phosphorylated residue; SA512, 4th bleed). To test specificity, a dot blot was performed. One microliter of decreasing concentrations of the phosphopeptides and non-phosphopeptides were pipetted onto nitrocellulose membranes (Thermo) and air-dried for 10 min before blocking in 5% milk/PBS-Tween (1 h, RT). The p-CYTIP antibody (1 µg/ml) was incubated in 5% BSA/PBS-Tween with 10 µg/ml of the non-phosphorylated peptide overnight at 4°C. The membrane was washed 5× PBS-Tween before incubation with anti-sheep HRP in 5% milk/PBS-Tween (1 h, RT), followed by 5× washes in PBS-Tween. Membranes were visualised using the ECL technique (Bio-Rad) on a LI-COR C-DiGit (Supplementary Figure S2).

### TCR stimulation and selective proteomic proximity labelling assay using tyramide (SPPLAT) of CD3 molecules

T cells were incubated in serum-free RPMI 1640 media, 3 h on ice, then incubated in RPMI 1640 media/10% foetal bovine serum (FBS) ± IL-7 overnight, 37°C, 5% CO_2_. After washing, the cells were incubated with anti-CD3 for 30 min on ice, washed, and then incubated with HRP-conjugated cross-linking antibody, 2 h on ice. Controls without anti-CD3 or without cross-linking antibody were also included; see Supplementary Tables S6 and S7 for further details. After washing, cells were resuspended in 1 ml pre-warmed (37°C) PBS and incubated for 8 min room temperature, to allow for cross-linked CD3 stimulation. After 8 min, 4ml tyramide labelling solution (80 µg/ml biotin-tyramide/0.3% H_2_O_2_/50 mM Tris–HCl) were added and left to biotinylate for 2 min. Biotin-tyramide reagent was prepared as previously reported [25] with EZ-Link NHS-LC-biotin (PIERCE). These conditions were previously optimised (Supplementary Figures S3 and S4). H_2_O_2_ was quenched by adding 100 U catalase, 5 ml cold PBS, incubated 5 min on ice. Cells were pelleted and resuspended in lysis buffer (5 mM ethylenediaminetetraacetic acid (EDTA), 1% Triton in PBS with protease inhibitors) and stored at −80°C until further processing.

### Neutravidin affinity purification of CD3-SPPLAT T cell proteins

SDS was added to each lysate to a final concentration of 1% before cold bath sonication for 15 min. Debris was pelleted by centrifugation (12 000×***g***, 10 min) and the supernatant was transferred to a new microtube with 10 U DNAse and quantified with a bicinchoninic acid assay (BCA).

As an internal standard for quantitation, and to control for non-specific proteins binding to downstream Neutravidin purification resins, an equal amount of heavy SILAC labelled (Arg6–Lys6) Jurkat cells (from ATCC/ECAC) soluble protein extract was added to the T cell protein extract. This enabled the quantification of the relative abundance of proteins identified and the elimination of non-specific proteins that appeared in a 1 : 1 ratio in the combined sample.

Neutravidin beads (50 µl bed volume per sample) were washed three times with 1 ml Lysis Buffer and added to each mixed sample/SILAC Jurkat control. Samples were incubated with the beads for 1 h under mild agitation at 4°C. The unbound fraction was discarded and the loaded beads were washed three times with Lysis Buffer. Proteins labelled during the SPPLAT protocol were recovered using three rounds of elution with 300 µl of 0.1 M glycine, pH 2, 10 mM biotin. Eluates were ethanol/acetone precipitated as per Queiroz *et al.* [53].

### Proteomics materials

Modified trypsin and Lysyl endopeptidase (Lys-C) were obtained from Promega (Madison, WI). Protein deglycosylation kit was purchased from New England BioLabs (Hitchin, England). PorosOligoR3 reversed-phase material, trifluoroacetic acid, TMT-10plex™ label reagent and Qubit Protein Assay Kit were acquired from Life Technologies (Carlsbad, CA). Titanium dioxide TitanSphere beads were from GL Sciences Inc. (Tokyo, Japan). A complete cocktail of phosphatase inhibitors (PhosStop) was from Roche (Meylan, France). Neutravidin beads were obtained from Thermo. All other reagents were purchased from Sigma–Aldrich (St. Louis, MO) unless stated otherwise. All chemicals and solvents used for mass spectrometry and its sample preparation were of the highest purity available, and all sample preparation procedures were performed in low-binding polypropylene microtubes from Sorenson Bioscience (Salt Lake City, UT).

### Sample digestion

Each sample was resuspended in 100 µl of 8 M urea in 100 mM triethylamonium bicarbonate (TEAB), reduced with 20 mM dithiothreitol (DTT) at room temperature for 60 min, alkylated with 40 mM iodoacetamide at room temperature in the dark for at least 60 min, and digested overnight at room temperature with Lys-C (1 µg per sample). After digestion, the solution was diluted to a final urea concentration of 1 M, 1 µg of modified trypsin was added (1 : 50 (w/w) trypsin: substrate ratio), and the samples were incubated for 3 h at room temperature. The samples were acidified with trifluoroacetic acid (TFA) (0.1% (v/v) final concentration) and debris was pelleted by centrifugation at 21 000×***g*** for 10 min and supernatant frozen at −80°C until to peptide concentration and clean-up. Poros Oligo R3 resin was equilibrated in 0.1% TFA.

Each sample was desalted by sequentially incubating for 5 min with occasional vortexing with 20 µl of slurry (∼10 µl resin) being added, spun down and the supernatant transferred to new microtube with another 20 µl slurry in a total of 3–6 steps. Then peptide-loaded resins were combined and packed into p200 tips over a 3 M Empore C8 ‘plug’ (∼1 cm long), washed twice with 50 µl 0.1% TFA and eluted with 200 µl 60% acetonitrile (adapted from [54]) and quantified with Qubit^TM^. For samples primed or not with IL-7 for 24 h, 100 µg per sample/conditions were used TMT labelling. Samples generated by SPPLAT were lower in abundance than Qubit detection limit, and, thereafter, were directly applied to LC–MS/MS analysis after digestion and desalting.

The analysis of samples generated using the SPPLAT protocol was also performed by the Orbitrap Fusion Lumos coupled to a nanoLC Dionex Ultimate 3000 UHPLC. Samples were trapped on a 100 μm × 2 cm, C18, 5 μm, 100 trapping column (Acclaim PepMap 100) in μl-pickup injection mode at 15 μl/min flow rate for 10 min. Samples were then loaded on a Rapid Separation Liquid Chromatography, 75 μm × 50 cm nanoViper C18 3 μm 100 column (Acclaim, PepMap) at 50°C retrofitted to an EASY-Spray source with a flow rate of 300 nl/min (buffer A, HPLC H_2_O, 0.1% formic acid; buffer B, 100% acetonitrile, 0.1% formic acid; 0–15 min: at 2% buffer B, 15–215 min: linear gradient 2–40% buffer B, 215–215.3 min: 40–90% buffer B, 215.3–225 min: at 90% buffer B, 225–225.3 min: 90–2% buffer B, 225.3–240 min: at 2% buffer B). Mass spectra were acquired using CHarge Ordered Parallel Ion aNalysis (CHOPIN) acquisition in positive ion mode as previously reported [55]. MS scans were acquired at a resolution of 120 000 between 400 and 1500 *m*/*z* and an automatic gain control (AGC) target of 4 × 10^5^. MS/MS spectra were acquired in the linear ion trap (rapid scan mode) after collision-induced dissociation (CID) fragmentation at a collision energy of 35% and an AGC target of 4 × 10^3^ for up to 250 ms, employing a maximal duty cycle of 3 s, prioritising the most intense ions and injecting ions for all available parallelizable time. Selected precursor masses were excluded for 60 s. For precursor selection, we prioritised the least abundant signals. Doubly charged ions were scheduled for CID/ion trap analysis with the same parameters applied as above. Charge states 3–7 with precursor intensity >500 000, however, were scheduled for analysis by a fast HCD/Orbitrap scan of maximal 40 ms (15 000 resolution). The remaining charge-state 3–7 ions with intensity <500 000 were scheduled for analysis by CID/ion trap, as described above.

### Phosphoproteomics methods

T cells stimulated 24 h in RPMI 1640 media/10% FBS with or without IL-7 (50 ng/ml) were reduced, alkylated with iodoacetamide, digested with Lys-C/Trypsin (Promega), before TMT-10plex labelling according to manufacturer's instructions (ThermoScientific). Briefly, TMT labels (0.8 mg) were resuspended in acetonitrile. Each desalted sample digest was resuspended in 150 mM TEAB before TMT labelling at 1 h, room temperature. Each TMT reaction was quenched with hydroxylamine (15 min) and samples were subsequently combined in equal proportions (multiplex). Ten percentage of each multiplex was dried and stored at −80°C for pre-fractionation alkaline-RP chromatography for total proteome analysis. The remaining was dried down and stored (−80°C) before phosphopeptide enrichment. Phosphopeptide enrichment used SIMAC (sequential elution from IMAC) methodology [56], a strategy that separates monophosphorylated from polyphosphorylated peptides. Briefly, each lyophilised multiplexed sample was resuspended in 1 M glycolic acid in 80% acetonitrile/5% TFA (v/v), and 0.6 mg of TiO_2_ beads were added per 100 µg of peptide sample before incubation under vigorous shaking for 10–15 min. Beads were pelleted, and the supernatant was transferred to new microtubes. The addition of TiO_2_ beads to the supernatants (using 0.3 mg TiO_2_ per 100 g of peptide) was repeated two more times. The TiO_2_ beads from the three rounds of enrichment were combined and washed first with 80% acetonitrile/1% TFA (v/v) and then with 10% acetonitrile/0.1% TFA (v/v) to remove non-phosphorylated peptides bound to TiO_2_. Phosphopeptides were then eluted with fresh ammonia solution (0.28%), pH 11, and lyophilised. The pre-enriched phosphopeptides were dissolved in 150 μl SIMAC loading buffer (0.2% TFA, 50% acetonitrile). For each multiplex, 80 μl of iron-coated PHOS-select^TM^ Iron Affinity gel (metal chelate (IMAC) beads) (Sigma) was used. The beads were washed twice with 1 ml SIMAC loading buffer and incubated with the 150 μl sample solution under vigorous agitation for 30 min at room temperature. After incubation, the IMAC beads were packed in the constricted end of a 200 μl GELoader tip by air pressure. The IMAC flow-through was collected in a new microtube. The IMAC column was washed using 50 μl of loading buffer, and the washing flow-through was collected together with the IMAC flow-through. The mono-phosphopeptides were eluted twice from the IMAC column with 70 μl of 1% TFA, 20% acetonitrile, and collected together with the IMAC flow-through. The multi-phosphorylated peptides were subsequently eluted from the same IMAC microcolumn using 80 μl of fresh ammonia solution (0.28% ammonia solution, pH 11). The multi-phosphorylated peptides were acidified with 10 μl 100% formic acid and purified by Oligo R3 reverse-phase microcolumns. The multi-phosphopeptide fraction and the mixture (pooled IMAC flow-through and the mono-phosphopeptides elution) were dried by vacuum centrifugation, respectively. The dried mono-phosphopeptide mixture was dissolved in 70% ACN, 2%TFA and subjected to a second round of TiO_2_ chromatography, and loaded onto TiO_2_ beads, then washed with 50% acetonitrile, 0.1% TFA, re-eluted with 100 μl of fresh ammonia solution and desalted using Poros R3 microcolumns.

### Alkaline reverse-phase pre-fractionation and LC–MS/MS analysis

For total proteome analysis, aliquots of multiplexed samples of T cells, with or without IL-7 priming for 24 h were pre-fractionated using High pH Reversed-phase Peptide Fractionation Kit from Pierce. For the total proteomes, 10% of each fraction was used for the LC–MS/MS analysis, while for the phosphoproteomes both mono- and multi-phosphorylated fractions were each analysed twice with 50% of the phospho-enriched fraction applied in each analysis.

Both the total and the phosphoproteome samples of T cells with and without IL-7 priming for 24 h were analysed in an Orbitrap Fusion Lumos tribrid mass spectrometer (Thermo Scientific), coupled to a nanoLC (Dionex Ultimate 3000 UHPLC). Samples were trapped on a 100 μm × 2 cm, C18, 5 μm, 100 trapping column (Acclaim PepMap 100) in μl-pickup injection mode at 15 μl/min flow rate for 10 min. Samples were then loaded on a Rapid Separation Liquid Chromatography, 75 μm × 50 cm nanoViper C18 3 μm 100 column (Acclaim, PepMap) at 50°C retrofitted to an EASY-Spray source with a flow rate of 300 nl/min (buffer A, HPLC H_2_O, 0.1% formic acid; buffer B, 100% acetonitrile, 0.1% formic acid; 0–15 min: at 2% buffer B, 15–215 min: linear gradient 2–40% buffer B, 215-215.3 min: 40–90% buffer B, 215.3–225 min: at 90% buffer B, 225–225.3 min: 90–2% buffer B, 225.3–240 min: at 2% buffer B). Mass spectra were acquired in positive ion mode applying data acquisition using synchronous precursor selection MS^3^ (SPS-MS^3^) acquisition mode [57]. Each MS scan in the Orbitrap analyzer (mass range = *m*/*z* 380–1500, resolution = 120 000) The most intense ions above a threshold of 2 × 10^4^ were selected for collision-induced dissociation (CID)-MS^2^ fragmentation, with an AGC target and maximum accumulation time of 1 × 10^4^ and 50 ms. Mass filtering was performed by the quadrupole with 0.7 *m*/*z* transmission window, followed by CID fragmentation in the linear ion trap with 35% normalised collision energy. SPS was applied to co-select 10 fragment ions for HCD-MS^3^ analysis. SPS ions were all selected within the 400–1200 *m*/*z* range and were set to preclude selection of the precursor ion and TMT ion series. AGC targets and maximum accumulation times were set to 1 × 10^5^ and 120 ms. Co-selected precursors for SPS-MS^3^ underwent HCD fragmentation with 65% normalised collision energy and were analysed in the Orbitrap with nominal resolution of 5 × 10^4^. The number of SPS-MS^3^ spectra acquired between full scans was restricted to a duty cycle of 3 s. Selected fragmented ions were dynamically excluded for 70 s.

Raw data were viewed in Xcalibur v.2.4 (Thermo Scientific), and data processing was performed using Proteome Discoverer v.2.4 (Thermo Scientific). The Raw files were submitted to a database search using Proteome Discoverer with SequestHF algorithm against the *Homo sapiens* database, downloaded in February 2019 containing 93 273 human protein sequences from UniProt/Swiss-Prot and UniProt/TrEMBL. Common contaminant proteins (several types of human keratins, BSA, and porcine trypsin) were also added to the database, and all contaminant proteins identified were removed from the result lists before statistical analysis. The spectra identification was performed with the following parameters: MS accuracy, 10 ppm; MS/MS accuracy, 0.5 Da; up to two missed cleavage sites allowed; carbamidomethylation of cysteine and TMT6plex tagging of lysine and peptide N-terminus as a fixed modification; and oxidation of methionine and deamidated asparagine and glutamine as variable modifications. For phospho-enriched fractions, phosphorylation of S, T, and Y residues were also added as variable modifications. For SPPLAT generated samples, arginine-6 and lysine-6 were also set as variable modification and no TMT tagging or phosphorylations were searched. Percolator node was used for FDR estimation and only peptide identifications of high confidence (FDR < 1%) were accepted. A minimum of two high-confidence peptides per protein was accepted for identification using Proteome Discoverer. In the identification lists, repeated protein groups were removed and only Master Proteins were considered for biological interpretation of the results. For phosphopeptide identifications, only high-confidence phosphopeptides with phosphorylation site probability scores (pRS) above 75% were accepted. Peptide lists filtered for the abovementioned stringencies were exported and further processed using RStudio software. Due to low protein amounts in the samples recovered by the SPPLAT protocol, Master Proteins with one high confidence and unique peptide identifications were considered. Also, only peptides/proteins identified with higher abundance in ‘light’ sample than in ‘heavy’ SILAC Jurkat spike-in control were accepted.

### Statistical/bioinformatic data analysis of TMT-labelled samples

Statistical analysis of TMT-labelled samples was performed with RStudio software. The data from T cells with and without IL-7 for 24 h consisted of a single multiplex comprised of five biological replicates of the two conditions. Identification results from technical replicates and/or fractions were merged and any repeated protein groups were removed. PSMs from peptides or phosphopeptides containing any missing condition was removed. The intensity values of each sample and each TMT label were log_2_-transformed and median-normalised. Multiple measurements of the same peptide were merged using the RRollup function of the DanteR package [58]; we allowed only one peptide measurement and chose the ‘mean’ instead of the ‘median’ option. The values of non-phosphorylated peptides were converted into protein quantitation (Rrollup, ‘mean’), requiring a minimum of one peptide per protein for total proteome quantitation. Phosphorylated peptides were filtered for pRS > 75% and the correspondent phospho-sites were assigned to the due residue in the sequence before all PSMs from the same sequences and high-confidence phosphorylation site were averaged using the same Rrollup function. All proteins and phosphorylations that were not detected in at least two biological replicates were then removed before statistical analysis.

For more robust statistical testing we applied Limma [59] and rank products [60] approaches to provide sufficient power to deal with low replicate numbers and additional missing values [61]. We, therefore, carried out both sets of analyses on all phosphopeptides and protein ratios against condition 1 (non-stimulated sample) and corrected them for multiple testing [62]. All phosphopeptides/proteins with smaller *q*-values (from both tests) below 0.05 (5% FDR) against either condition 1 (**NS**) were considered regulated.

For data generated by SPPLAT, only Master Proteins, which were detected/identified from/with the ‘light’ SILAC label and Light/Heavy-SILAC ratio above 1 were accepted.

All R scripts and raw files are available at https://github.com/RaynerQueiroz/IL7_analysis accessible upon request

### Confocal microscopy experiments

T cells were incubated as described for the SPPLAT experiments (Supplementary Table S7). For each condition, two biological donors were used. Cells were incubated with anti-CD3, 1 h, 4°C, washed, then incubated with secondary antibody, 1 h, 4°C. Cells were washed, resuspended in warm media for 8 min, during which 1 × 10^6^ cells were spun onto poly-l-lysine-coated coverslips in 24-well plates. Cells were fixed with cold 4% PFA, 10 min, RT, washed, stored in 10% BSA/PBS. Staining of selected proteins was performed by incubation of coverslips with antibody (1 h, RT), either directly conjugated with AF488 or followed by an AF488 secondary, raised against the primary origin for 1 h, RT. Staining for intracellular proteins was performed by permeabilization (10% BSA, 0.5% Saponin/PBS) for 10 min. Cells were washed, mounted with Duolink mounting media onto glass slides and imaged using an Olympus Fluoview IX81-FVSF-2 laser scanning microscope with a UPLANAPO-N 60× magnification lens and numerical aperture set to 2, at room temperature. Acquisition was performed using Fluoview v 5.0 FV300. Cells were counted manually by eye and three frames per donor were imaged, and images were imported into GIMP2 for cropping and collation and Fiji for 3D analysis. Co-localisation was determined by observing regions of yellow pixels generated in an overlay of the green and red fluorescent channels, and clustering of proteins on the plasma membrane was determined by identifying punctate fluorescent signals, of either colour, on the perimeter of the cell that was larger than 1 µm in diameter, as measured using the Fluoview scalebar. Regions where groups of pixels measured less than 1 µm were defined as small clusters and where pixels were scattered, either around the membrane or intracellularly, this was classified as dispersed. Statistical analysis was performed using Excel.

### Western blot and immunoprecipitation

T cells were incubated and antibodies pre-incubated at 4°C as for the SPPLAT experiments (Supplementary Table S7). Cells were resuspended in pre-warmed RPMI 1640 media for 8 min, 37°C with agitation. Cell signalling was terminated by exchanging cells into ice-cold PBS. For each condition, 2 × 10^6^ cells were lysed in lithium dodecyl Sulfate (LDS) sample buffer and heated at 90°C for 5 min for SDS–PAGE, or into immunoprecipitation lysis buffer for IPs. For immunoprecipitation, 0.5 mg of protein extract (equalised to 3 mg/ml with 1× lysis buffer) per condition was incubated with 0.5 µg of anti-cytohesin, or with goat IgG control (AB-108-C, R&D) (2.5 h, 4°C), with agitation; 1.5 mg protein A/G magnetic beads per tube were incubated (1 h, 4°C). Beads were washed ×3 (50 mM Tris–HCl (pH 7.5), 0.2 M NaCl and 1% Triton X-100) and once more (10 mM Tris–HCl (pH 7.5)) before elution into LDS reducing sample buffer. For blotting, cell lysate or immunoprecipitates were run on a 4–12% Bis–Tris gel in MES running buffer, followed by transfer onto polyvinylidene fluoride (PVDF) blotting membrane, before antibody detection using standard techniques, following the antibody manufacturers’ recommendations. For detection of p-CYTIP, an additional high salt wash for 30 min (PBS, 0.5 M NaCl and 0.2% SDS) was performed to minimise background and visualised using the ECL technique as before.

## Data Availability

The mass spectrometry proteomics data have been deposited to ProteomeXchange Consortium PRIDE [63] partner repository, with the dataset identifier PXD013520.

## Open Access

Open access for this article was enabled by the participation of University of Cambridge in an all-inclusive *Read & Publish* pilot with Portland Press and the Biochemical Society under a transformative agreement with JISC.

## Abbreviations

AGC: automatic gain control
AP-1: activator protein 1
APC: antigen-presenting cell
BCA: bicinchoninic acid
BSA: bovine serum albumin
CHOPIN: charge ordered parallel ion analysis
CID: collision-induced dissociation
CYTIP: cytohesin interacting protein
DC: dendritic cells
DPBS: Dulbecco′s phosphate-buffered saline
DTT: dithiothreitol
EDTA: ethylenediaminetetraacetic acid
ELISA: enzyme-linked immunosorbent assay
ERK: extracellular signal-regulated kinase
FBS: foetal bovine serum
FDR: false discovery rate
HCD: higher-energy C-trap dissociation
HPLC: high-performance liquid chromatography
HRP: horseradish peroxidase
ICAM-1: intercellular adhesion molecule-1
IL-7: interleukin 7
IL-7R: IL-7 receptor
IMAC: immobilised metal affinity chromatography
IS: immune synapse
ITGB2: integrin β-subunit 2
JAK: Janus kinase
LC–MS/MS: liquid chromatography with tandem mass spectrometry
LDS: lithium dodecyl Sulfate
LFA-1: lymphocyte function-associated antigen 1
MES: 2-(*N*-morpholino)ethanesulfonic acid
MHC: major histocompatibility complex
PBMC: peripheral blood mononuclear cell
PD1: programmed cell death protein 1
PHA: phytohaemagglutinin
pRS: phospho-site assignment
PVDF: polyvinylidene fluoride
RASAL3: RAS protein activator like 3
RPMI 1640: Roswell Park Memorial Institute media
SDS–PAGE: sodium dodecyl Sulfate–polyacrylamide gel electrophoresis
SILAC: stable isotope labelling with amino acids in cell culture
SIMAC: sequential immobilised metal affinity chromatography
SNX27: Sorting Nexin 27
SPPLAT: selective proteomic proximity labelling assay using tyramide
SPS: synchronous precursor selection
STAT: signal transducer and activator of transcription
TCR: T cell receptor
TEAB: triethylamonium bicarbonate
TFA: trifluoroacetic acid
Th17 cells: T-helper 17 cells
UHPLC: ultra high-performance liquid chromatograph
