# Supplementary material for: Proteomic analysis in primary T cells reveals IL-7 alters T cell receptor thresholding via CYTIP/cytohesin/LFA-1 localisation and activation
Source: Biochem J. 2022 Feb 4;479(3):225–43. doi: 10.1042/BCJ20210313 (PMC8883493; doi:10.1042/BCJ20210313)
Supplement: Supplementary Material [file BCJ-479-225-s1.pdf]

Supplemental Information

Rayner M. L. Queiroz[1-2*], Siân C. Piper[3*], Johanna S. Rees[1-2* #], Sam Strickson[3], Emmanuel Briend[4], Choon Pei Low[5], G. John Ferguson[6], Kathryn S. Lilley[1,2], Antony P. Jackson[1], and Donna K. Finch[4]

Supplemental Information Figures

Supplemental Information Figure S1

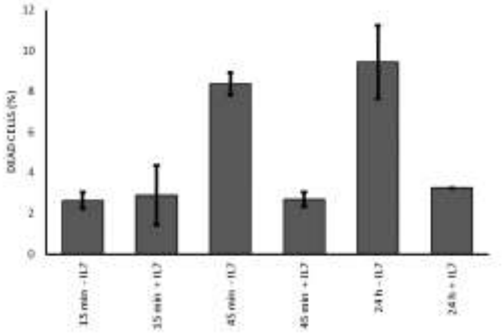

Supplemental Information Figure S1. Total T cell viability is maintained after 24h culture without IL-7 even in absence of FBS. The percentage of viable total T cells measured through flow-cytometry with a fixable Viability Dye eFluor® 780 and was superior to 90% when primed or not with IL-7 with and without FBS. Representative of data from 3 independent experiments in 3 donors.

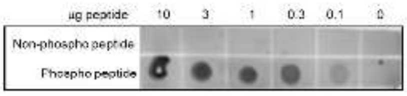

Description	Sequence
Phospho peptide	SRQTST*DDE
Non-phospho peptide	SRQTSTDDE

Supplemental Information Figure S2

Supplemental Information Figure 2. Specificity of anti-p-CYTIP^{T280} Dot blot analysis demonstrating that anti-p-CYTIP^{T280} recognises the phosphorylated CYTIP peptide but not the non-phosphorylated peptide.

Supplemental Information Figure S3

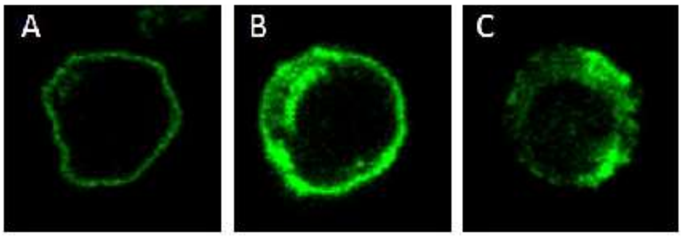

Supplemental Information Figure S3. Analysis of incubation times for SPPLATing of CD3 molecules on the surface of primary human T cells in absence of IL-7 priming or TCR activation. T cells were incubated with anti-CD3-HRP to label surface CD3 molecules. The labelled cells were incubated with biotin-tyramide for 1 min (**A**), 2 min (**B**) or 5 min (**C**) to allow biotinylation of CD3 associated proteins. To assess the degree of biotinylation achieved by each labelling time the cells were incubated with streptavidin-AF488, mounted in Duolink mounting media, and assessed by confocal microscopy for the degree of staining of the cell surface. A good degree of surface staining was observed after 2 min labelling, whereas 5min labelling resulted in some intracellular labelling. Confocal microscopy was performed using an Olympus Fluoview IX81-FVSF-2 laser scanning microscope with a UPLANAPO-N 60x magnification lense and numerical aperture set to 2, at room temperature. Acquisition was performed using Fluoview v 5.0 FV300 and images imported into GIMP2 for cropping and collation.

Supplemental Information Figure S4

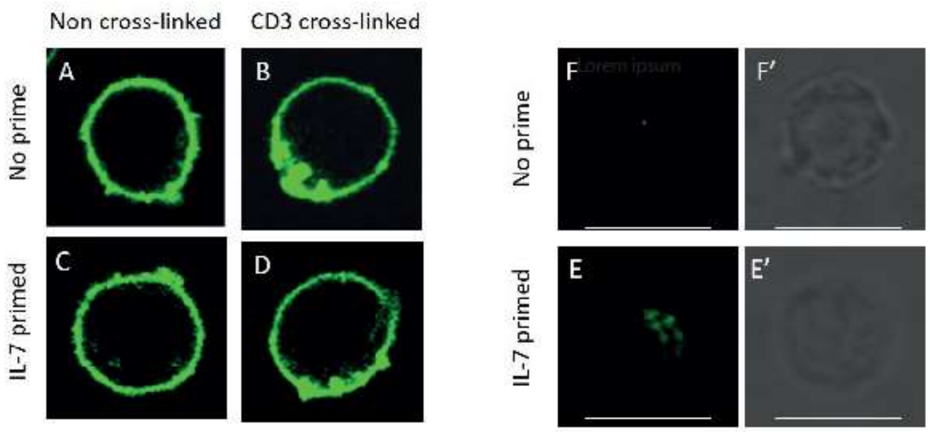

Supplemental Information Figure S4. Analysis of IL-7 and/or TCR stimulation conditions for CD3 SPPLAT analysis on the surface of primary human T cells within 2 min biotinylation. The Biotinylation profile of CD3 SPPLATed primary human T cells upon IL-7 and TCR stimulation was assessed. Following stimulation with CD3-HRP or HRP-tagged cross-linking antibody, the cells were incubated with biotin-tyramide for 2 min. After staining with streptavidin-AF488 and mounting using Duolink media the cells were assessed by confocal microscopy. (**A**) IL-7 unprimed and non-crosslinked CD3; (**B**) IL-7 unprimed and crosslinked CD3; (**C**) IL-7 primed and non-crosslinked CD3; (**D**) IL-7 primed and crosslinked CD3; (**E**) IL-7 primed and non-crosslinked CD3 Negative SPPLAT control; (**E'**) brightfield view; (**F**) IL-7 unprimed and non-crosslinked CD3Negative SPPLAT control; (**F'**) brightfield view. Confocal microscopy was performed using an Olympus Fluoview IX81-FVSF-2 laser scanning microscope with a UPLANAPO-N 60x magnification lense and numerical aperture set to 2, at room temperature. Acquisition was performed using Fluoview v 5.0 FV300 and images imported into GIMP2 for cropping and collation.

Supplemental Information Tables

As separate .pdf files

Supplemental Information Table S1. Log2/median normalized TMT reporter intensities of 24 h IL-7 stimulation of human primary T cell total proteome

Supplemental Information Table S2. Log2/median normalized TMT reporter intensities of phosphorylation sites of 24 h IL-7 stimulation of human primary T cell phosphoproteome

Supplemental Information Table S3. Log2/median normalized TMT reporter intensities of regulated proteins (tab3.1) and phosphorylation sites (tab 3.2) of 24 h IL-7 stimulation of human primary T cell total proteome

Supplemental Information Table S4. Raw Proteome discoverer output from the three replicates of Conditions A-F (tabs 4.1(A)-4.6(F))

Supplemental Information Table S5. Master protein identifications from CD3 SPPLATed primary human T cells with SILAC abundance ratio Light/Heavy > 1 (tab 5.1) and not in controls (tab 5.2)

Supplemental Information Table S6. Antibody sources, concentrations and applications

Supplemental Information Table S7. Summary of stimulation condition and antibody conjugation state per experimental design

Supplemental Table 1. Log2/median normalized TMT reporter intensities of 24 h IL-7 stimulation of human primary T cell total proteome

	Protein nar	Gene name	Control_1	Primed_1	Control_2	Primed_2	Control_3	Primed_3	Control_4	Primed_4	Control_5	Primed_5
A0A0B4J2F	Protein PIG	PIGBOS1	-0.17404	-0.08743	0.056501	-0.11431	0.224811	0.084632	0.033934	-0.00448	-0.00047	0.00861
A0A5B9	T cell recep	TRBC2 TCR	-0.5025	-0.5427	-0.0516	0.137504	-0.53382	-0.24998	-0.5697	-0.47743	-0.28584	-0.18382
A0AVT1	Ubiquitin-li	UBA6 MOP	1.675038	2.056396	1.850161	1.703332	1.859519	1.352211	1.743092	1.719688	1.746833	1.813663
A0FGR8	Extended s	ESYT2 FAM	0.173037	0.329956	0.158866	-0.07977	0.013274	0.147424	0.218001	-0.1193	-0.18593	-0.1008
A1L0T0	2-hydroxya	ILVBL AHAS	-0.13993	-0.23676	-0.09259	-0.16601	-0.06075	-0.24349	-0.30666	-0.13808	-0.21299	-0.1008
A2A288	Probable ri	ZC3H12D C	-1.32496	-1.91667	-1.93147	-2.9386	-4.42999	-3.34395	-3.09516	-1.88982	-3.45003	-4.34873
A2RRP1	Neuroblast	NBAS NAG	-1.60942	-1.69849	-0.88753	-0.91475	-2.07635	-1.40054	-1.85415	-3.07039	-2.08746	-1.70487
A4D1P6	WD repeat	WDR91 HS	0.082462	0.165586	0.26628	0.231326	0.859519	0.447459	0.010196	0.207595	0.719892	0.843565
A5YKK6	CCR4-NOT	CNOT1 CD	-0.53882	-0.41773	-0.71058	-0.74443	-1.04532	-0.58496	-0.83936	-0.79737	-0.6325	-0.56133
A6NC98	Coiled-coil	CCDC88B B	-1.26546	-0.91667	-0.93545	-1.01808	-0.81382	-0.99604	-0.53222	-0.74846	-0.96989	-0.63691
A6NDG6	Glycerol-3-	PGP	-0.15384	-0.27551	-1.52524	-1.6235	-1.26914	-1.71652	-1.77644	-0.81978	-1.04807	-1.0838
A6NHR9	Structural r	SMCHD1 KI	0.138605	0.236803	0.137504	0.329436	0.31895	0.390234	0.213595	0.326501	0.331327	0.323697
A6NI79	Coiled-coil	CCDC69	-0.0661	-0.05331	0.141087	-0.11126	0.272583	0.275658	0.15004	0.21672	0.05391	-0.13967
A6NIH7	Protein unc	UNC119B	-2.27442	-2.03297	-1.65509	-1.25437	-1.46069	-1.3626	-1.58971	-1.35213	-1.52441	-1.44274
A6NJ78	12S rRNA N	METTL15 N	-3.13993	-2.42286	-2.08844	-2.9386	NA	NA	-3.81762	-3.1193	-2.06756	-2.06333
A8MPS7	Carbohydra	YDJC	-3.13993	-1.16506	-1.13906	-1.54171	-1.47063	-1.5366	-1.43911	-2.04654	-0.95361	-0.53495
A8MVM7	Putative ur	0	1.314636	1.549436	1.489598	1.430634	1.345531	1.381871	1.564352	1.417183	1.632876	1.695666
B0I1T2	Unconvent	MYO1G HA	0.03592	0.144226	0.09423	0.373678	-0.17365	0.264767	0.110029	0.410737	0.130629	0.017594
B7ZAP0	Rab GTPase	RABGAP1L	1.829696	1.842571	2.358123	2.245945	2.519028	2.195204	2.191366	2.363683	2.777411	2.634978
B9A064	Immunoglo	IGLL5	2.486725	2.489598	0.704633	0.942413	0.648015	1.117997	1.103623	0.880701	1.591235	1.114796
C9J7I0	UBAP1-MV	UMAD1 RP	-2.53727	-1.96153	-1.91667	-1.56009	-1.31451	-1.81558	-2.11718	-1.86876	-1.58496	-2.1589
E9PRG8	Uncharacte	C11orf98	-0.08746	-0.29776	0.348202	0.294061	0.47155	0.595851	0.66458	0.312739	0.523972	0.287896
G9CGD6	CNK3/IPCE	CNK3/IPCE	0.48158	0.510426	0.555037	0.462109	0.496765	0.58502	0.737699	0.796327	1.121857	0.938304
I3L1I5	Rho guanin	ARHGEF18	-0.84828	-0.63268	-0.5475	-0.82854	-0.56485	-0.42609	-0.75046	-0.7384	-0.41926	-0.3252
L0R819	ASNSD1 up	ASDURF	1.102362	1.225175	1.553427	1.049328	1.564994	1.35435	1.301318	1.162271	1.938599	1.744029
O00116	Alkyldihydr	AGPS AAG5	-0.30006	-0.62118	-0.3085	-0.30521	-0.73766	-0.15654	-0.53723	-0.47746	-0.53175	-0.24073
O00139	Kinesin-like	KIF2A KIF2	0.029995	0.418124	-0.39569	-0.28325	-0.66918	-0.45194	-0.26919	0.093774	-0.32624	-0.21945
O00148	ATP-depen	DDX39A DD	-0.22147	-0.06153	-0.14851	-0.19257	-0.71499	-0.75982	-0.65105	-0.73835	-0.93139	-0.9967
O00151	PDZ and LII	PDLIM1 CLI	-0.04307	0.137504	-0.10932	0.198904	-1.10806	-0.75899	-0.19118	-0.15073	-1.36257	-0.77887
O00154	Cytosolic a	ACOT7 BAC	-0.85641	-0.66307	-0.67911	-0.65337	-1.75056	-1.65392	-0.80009	-1.1414	-1.21287	-1.53673
O00160	Unconvent	MYO1F	0.422595	0.513013	0.730713	0.7507	0.637154	0.536806	0.67423	0.303491	-0.68793	-0.61156

O00161	Synaptosor	SNAP23	-0.05069	-0.01971	-0.04274	-0.09678	-0.04534	0.109011	-0.02914	-0.06148	-0.26303	-0.47836
O00170	AH recepto	AIP XAP2	0.420777	0.320159	0.836096	0.632658	0.652134	0.676414	0.576364	0.601265	0.697449	0.490772
O00178	GTP-bindin	GTPBP1	-0.60251	-0.44072	-0.23702	-0.38008	-0.77607	-0.82565	-0.24625	-0.76134	-0.75796	-0.70188
O00182	Galectin-9	LGALS9	-0.31739	-0.5541	-0.29776	-0.2986	-1.53382	-0.80599	-0.65574	-0.50967	-0.37474	-1.1589
O00186	Syntaxin-bi	STXBP3	-0.65883	-0.42205	-0.03639	-0.10823	0.006167	0.11306	0.027255	0.123071	-0.16801	-0.07757
O00193	Small acidi	SMAP C11c	-0.24788	-0.05933	0.254952	-0.06156	-0.03354	0.244629	-0.14268	0.126833	0.74624	0.818325
O00203	AP-3 comp	AP3B1 ADT	-0.0743	0.128499	0.08755	0.268252	0.20045	0.204766	0.215758	0.145534	0.098896	0.30007
O00217	NADH dehy	NDUFS8	0.531514	0.440463	0.429074	0.55864	0.408839	0.32349	0.367039	0.811582	0.757536	0.581997
O00221	NF-kappa-E	NFKBIE IKB	-1.72489	-1.44392	-1.10512	-1.89129	-1.39046	-1.37197	-0.9898	-1.76783	-1.23502	-1.6763
O00231	26S protea	PSMD11	0.514197	0.512131	0.251549	0.464503	0.031533	0.328819	0.142443	0.089293	0.149982	0.053883
O00232	26S protea	PSMD12	0.248934	0.158874	0.311748	0.276226	0.299827	0.124173	0.152168	0.228204	0.105859	0.288276
O00233	26S protea	PSMD9	-0.40332	0.027039	0.437285	0.005705	-0.19903	-0.4975	0.507907	0.22376	0.025609	0.224919
O00244	Copper tra	ATOX1 HAH	0.530844	0.601144	0.759674	0.714568	0.751444	0.71719	0.649962	0.59119	0.877513	0.581644
O00255	Menin	MEN1 SCG	-1.60942	-1.75002	-1.68589	-1.11757	-0.89072	-0.78858	-1.5697	-1.29078	-1.20211	-1.2402
O00257	E3 SUMO-p	CBX4	-0.47433	-0.34094	-0.58782	-0.75982	-0.31256	-0.25144	-0.41483	-0.65361	-0.05582	-0.27438
O00264	Membrane	PGRMC1 H	-0.23971	-0.03955	-0.89478	-0.68242	-0.45031	-0.6116	-0.33401	-0.66743	-0.9198	-1.07994
O00267	Transcripti	SUPT5H SP	-0.51623	-0.56899	-0.33171	-0.52862	-0.53182	-0.36572	-0.66755	-0.58828	-0.33814	-0.34446
O00268	Transcripti	TAF4 TAF2	-1.73498	-1.95595	-1.61021	-1.51996	-1.43054	-1.41533	-1.65342	-1.47746	-1.3473	-1.28352
O00273	DNA fragm	DFFA DFF1	-0.25848	-0.14768	0.203328	0.222184	0.123112	-0.10188	-0.25081	-0.06165	0.38102	0.422576
O00287	Regulatory	RFXAP	-0.58193	-0.4761	-0.09675	0	-0.06853	-0.37906	-0.80863	-0.33342	-0.22396	-0.25597
O00299	Chloride in	CLIC1 G6 N	0.213707	0.52661	0.648528	0.626185	0.325083	0.090032	0.491982	0.207595	0.181835	-0.0223
O00303	Eukaryotic	EIF3F EIF3S	0.402343	0.429922	-0.32518	0.102163	-0.45672	-0.6158	-0.09777	-0.56003	-0.59614	-0.05669
O00308	NEDD4-like	WWP2	-1.70499	-2.32193	-1.66102	-1.63604	-1.19052	-1.2115	-1.55459	-1.80735	-2.39941	-1.0268
O00329	Phosphatid	PIK3CD	-0.13771	0.063166	-0.88178	-0.7635	-0.85024	-0.96884	-0.91612	-0.96105	-0.67693	-0.52302
O00330	Pyruvate d	PDHX PDX1	0.302882	0.451423	0.257337	0.304418	0.185371	0	0.15545	0.197646	0.409399	0.139912
O00410	Importin-5	IPO5 KPNB	-0.04763	0.194538	-0.44147	-0.22483	-0.69077	-0.71923	-0.66843	-0.87959	-0.76857	-0.35257
O00422	Histone de	SAP18 GIG	0.632659	0.67379	0.747068	0.726167	0.552102	0.750022	0.820451	0.681206	0.725606	0.602557
O00429	Dynamin-1	DNM1L DLI	0.612977	0.716909	0.38001	0.386687	0.610396	0.215332	0.545188	0.294289	0.12846	0.455803
O00442	RNA 3'-terr	RTCA RPC F	-0.30993	-0.34736	0.187234	0.134285	0.258791	0.190649	0.131934	0.003038	-0.0287	0.100832
O00478	Butyrophili	BTN3A3 BT	-2.53727	-1.37151	-1.63656	-1.71621	-2.84503	-1.79647	-1.87276	-1.55102	-1.57089	-1.39453
O00479	High mobil	HMGN4 HN	-0.9418	-0.86605	0.102158	-0.04521	-0.13355	-0.32845	-0.08672	-0.69858	0.377942	0.394768
O00481	Butyrophili	BTN3A1 BT	-0.84215	-1.17943	-1.08789	-1.29221	-0.89575	-1.62795	0.811733	1.008562	-1.98201	-1.55437
O00483	Cytochrom	NDUFA4	0.86119	0.777453	0.716257	0.687544	0.59172	0.371161	0.670992	0.68033	0.277017	0.051823

O00487	26S protea	PSMD14 P(	1.550878	1.408852	1.955593	1.416054	1.892075	1.737929	1.730361	1.845299	2.103099	1.90714
O00488	Zinc finger	ZNF593 ZT8	-2.89482	-2.28345	-1.73697	-2.75803	-1.2076	-2.83494	-1.39808	-3.02308	-2.52947	-1.93934
O00499	Myc box-de	BIN1 AMPH	0.394431	0.539103	0.555603	0.343729	0.494375	0.425023	0.588724	0.593949	0.768112	1.029475
O00505	Importin su	KPNA3 QIP	-1.13993	-1.35147	-0.12624	-1.15899	-0.39046	-0.68684	-0.95964	-0.9886	-0.47603	-1.1589
O00522	Krev intera	KRIT1 CCM	-0.13771	0.034718	-0.10412	-0.32297	-0.30281	0.019119	0.047187	-0.12772	-0.33663	-0.1483
O00541	Pescadillo h	PES1	-1.50897	-1.32219	-1.54091	-1.70337	-0.65478	-0.5579	-0.43137	-0.56589	-1.45511	-0.98893
O00560	Syntenin-1	SDCBP MD.	-2.47996	-1.73702	-0.78124	-0.57573	-1.42031	-1.44678	-1.34286	-2.6933	-1.28038	-1.17547
O00562	Membrane	PITPNM1 D	-0.45618	-0.23561	-0.18273	-0.19512	-0.42396	-0.3107	-0.48099	-0.54275	-0.60796	-0.77164
O00566	U3 small nu	MPHOSPH1	0.840607	0.67319	0.854481	0.716752	0.871762	0.686925	1.116113	0.912724	1.046393	1.250126
O00567	Nucleolar p	NOP56 NO	0.80215	0.779231	0.59754	0.515567	0.85271	0.706921	0.779312	0.624671	0.462926	0.441565
O00571	ATP-depen	DDX3X DBX	0.889817	1.01165	-0.66955	-1.76304	-0.89681	-0.44964	-0.5723	-1.12317	0.827421	0.656896
O00584	Ribonuclea	RNASET2 R	1.105182	0.942324	1.103791	1.012491	1.55467	1.539888	1.608398	1.645691	1.013826	0.955818
O00602	Ficolin-1 (C	FCN1 FCNM	-0.91754	-1.72403	-1.31221	-0.39428	-0.38074	-0.56866	-0.0897	-0.32623	-0.81444	-0.45998
O00629	Importin su	KPNA4 QIP	-0.13829	-0.0048	-1.21916	-1.03802	-1.05759	-1.05076	-1.01551	-0.96087	-0.89321	-0.945
O00635	E3 ubiquitin	TRIM38 RN	-0.15614	-0.2106	-0.24172	-0.06812	-0.34949	0.209426	-0.12491	-0.08506	-0.11476	-0.3921
O00743	Serine/thre	PPP6C PPP	0.806734	0.808469	0.387482	0.88243	0.174874	0.923122	0.874469	0.738628	0.693897	0.400385
O00757	Fructose-1,	FBP2	-1.87245	-1.32193	-1.7632	-1.69574	-2.69302	-1.5366	-1.28157	-1.24936	-0.65748	-0.89752
O00764	Pyridoxal k	PDXK C21o	-1.13993	-1.27399	-1.39183	-1.30259	-1.66918	-1.47454	-2.16829	-1.62619	-2.09925	-0.97285
O14497	AT-rich inte	ARID1A BA	0.531203	0.294985	0.279988	0.377195	0.199811	0.335881	0.372196	0.445012	0.279359	0.474414
O14498	Immunoglo	ISLR UNQ18	-0.74507	-0.69849	-0.46529	-0.56009	0.43351	0.179608	-0.40484	-0.15073	0.060025	0.330752
O14526	F-BAR dom	FCHO1 KIA.	-0.30236	-0.26459	-0.58301	-0.34564	-0.58869	-0.51301	-0.60039	-0.47743	-1.04793	0.090064
O14545	TRAF-type	TRAFD1 FLI	-1.98973	-1.5793	-1.05565	-1.4521	-1.71748	-1.61813	-1.76739	-1.85632	-1.1281	-1.08194
O14561	Acyl carrier	NDUFAB1	1.933319	1.751707	0.781501	1.276043	0.594844	1.676705	1.36626	1.246722	0.540274	0.155098
O14579	Coatomer s	COPE	0.40439	0.488209	0.203328	0.043253	0.086588	0.189226	0.191366	-0.18286	0.069042	0.291517
O14602	Eukaryotic	EIF1AY	-1.38702	-0.93147	0.301509	0.148863	-2.31451	-1.83494	-0.16227	-0.10692	0.606798	0.555154
O14617	AP-3 comp	AP3D1 PRC	1.03592	0.91983	1.254888	1.157325	1.236138	1.315355	0.860449	1.144254	1.195883	1.444062
O14618	Copper cha	CCS	-0.83722	-1.20365	-0.4195	-0.03532	-0.21492	-0.17061	-0.77051	-0.35651	-0.08001	0.178701
O14639	Actin-bindi	ABLIM1 AB	-0.59104	-0.78583	0.285602	-0.36639	-0.07635	-0.48714	-0.30666	-0.09464	0.387063	0.656896
O14641	Segment po	DVL2	-1.87245	-1.69849	-0.73697	-1.20163	-1.24236	-1.35789	-1.46713	-0.80735	-1.5025	-1.1788
O14646	Chromodom	CHD1	-1.25096	-1.13866	-0.84813	-0.37637	-0.77036	-0.56589	-0.47528	-0.53167	-0.503	-0.56106
O14647	Chromodom	CHD2	-1.51978	-1.28345	-1.4761	-1.50564	-1.22487	-1.52083	-1.48134	-1.65535	-1.33852	-0.97369
O14653	Golgi SNAP	GOSR2 GS2	-0.77588	-0.53703	-0.47069	-0.86821	-0.97056	-0.2962	-0.40484	-0.73888	-0.61353	-1.06333
O14662	Syntaxin-16	STX16	-3.37126	-2.4021	-2.42286	-1.63604	-3.01495	-3.04439	-3.89162	-3.58496	-3.00946	-2.28264

O14672	Disintegrin	ADAM10 K	0.24984	0.2824	0.399356	-0.0906	0.294905	0.326293	0.611993	0.453173	0.546743	0.50614
O14683	Tumor prot	TP53I11 PI(	-0.34023	-0.74348	-0.6734	-0.31411	-0.58869	-0.66069	-0.96963	-0.96605	-1.62803	-1.55431
O14737	Programme	PDCD5 TFA	-0.70482	-0.77767	-0.45534	-0.83861	-0.58665	-0.62189	-0.71681	-0.80361	-0.09946	0.00454
O14744	Protein arg	PRMT5 HRI	-1.64689	-1.94642	-3.62449	-1.8011	-2.59991	-2.23704	-1.51019	-3.07039	-3.39941	-3.1788
O14745	Na(+)/H(+)	SLC9A3R1 I	-0.08542	-0.03953	-0.44076	-0.45824	-0.54156	-0.73866	-0.76214	-0.67733	-0.66724	-0.38248
O14773	Tripeptidyl	TPP1 CLN2	0.856726	0.934185	0.416986	0.176878	-0.07635	0.189226	0.500694	0.308122	-0.04307	-0.56737
O14776	Transcripti	TCERG1 CA	0.20847	0.116077	0.360402	0.283793	0.427993	0.459432	0.460361	0.353637	0.382857	0.471451
O14787	Transportir	TNPO2	-2.85042	-2.42286	NA	NA	-3.35199	-3.65208	-2.9301	-3.97728	-2.30319	-3.82517
O14802	DNA-direct	POLR3A	-3.43539	-3.36146	-2.2276	-2.56009	-2.55552	-2.45943	-2.23266	-2.04654	-3.04793	-2.21945
O14818	Proteasom	PSMA7 HSF	0.506813	0.58598	-0.10512	-0.1186	0.425352	0.207984	0.243817	0.522494	0.258312	-0.09606
O14827	Ras-specifi	RASGRF2 G	-0.54241	-0.73688	-1.35385	-1.23433	-1.04166	-0.7216	-1.38728	-1.0145	-0.98178	-0.9281
O14828	Secretory c	SCAMP3 C1	-1.02445	-1.25526	-1.16506	-1.0126	-1.31451	-1.37197	-1.3322	-1.09464	-1.11784	-1.03585
O14867	Transcripti	BACH1	-3.08746	-2.5541	NA	NA	NA	NA	-4.43911	-3.80735	-3.25739	-3.1008
O14879	Interferon-	IFIT3 CIG-4	-0.04052	0.079818	-0.25551	-0.16614	-0.7138	-0.38413	-0.5133	-0.02843	0.36548	0.619357
O14893	Gem-assoc	GEMIN2 SII	-0.54609	-0.2185	-0.20046	-0.19444	-0.07635	-0.24349	-0.5175	-0.33342	-0.3445	-0.26658
O14908	PDZ domai	GIPC1 C19c	0.242581	0.377869	0.230051	0.507171	0.49552	0.215762	0.275743	0.419128	0.231722	0.111088
O14920	Inhibitor of	IKBKB IKKB	-0.15046	-0.17383	-0.44923	-0.46836	-0.74193	-0.67807	-0.29406	-0.946	-0.86166	-0.90379
O14929	Histone ace	HAT1 KAT1	-0.88359	-0.78991	-0.93892	-0.85681	-1.51245	-0.59318	-1.18536	-1.6735	-1.2916	-1.6763
O14933	Ubiquitin/I	UBE2L6 UB	0.584396	0.49932	0.902116	1.098471	0.596414	0.561816	0.909352	0.957812	0.811906	0.945695
O14949	Cytochrom	UQCRQ	1.440573	1.642296	0.812914	1.037153	0.954676	0.573991	1.259192	0.968164	0.293359	0.552139
O14950	Myosin reg	MYL12B M	0.131244	-0.01391	-0.13505	-0.24842	0.007546	-0.15264	-0.03928	-0.08311	-0.17102	0.054923
O14964	Hepatocyte	HGS HRS	-0.6133	-0.70574	-0.54868	-0.2168	-0.79256	-0.55034	-0.52712	-1.11311	-0.74733	-0.55431
O14966	Ras-relatec	RAB29 RAB	-1.60942	-1.35147	-2.03953	-2.06413	-2.55552	-2	-2.81762	-2.04654	-3.1281	-3.44184
O14972	Vacuolar pı	VPS26C DC	-3.30986	-2.20945	-2.26459	-3.20163	-3.01495	-1.52083	-2.13955	-2.04654	-3.97818	-3.24036
O14974	Protein pho	PPP1R12A	-0.25162	-0.09087	-0.11496	-0.14081	-0.3554	-0.35443	-0.32831	-0.32944	-0.16375	-0.18348
O14976	Cyclin-G-as	GAK	-1.49133	-1.08961	-0.85788	-0.80257	-1.1351	-1.29991	-1.01966	-0.98681	-0.88265	-1.35839
O14979	Heterogen	HNRNPDL I	0.029995	0.295167	0.584963	0.613942	0.397242	0.81201	0.426503	0.680728	1.108934	0.79593
O14980	Exportin-1	XPO1 CRM	-0.02445	0.396397	-0.73276	-0.41495	-0.53866	-0.52773	-0.31824	-0.37967	-0.56987	-0.67679
O14981	TATA-bindi	BTAF1 TAF	-1.18057	-0.61849	-1.1305	-1.54171	-1.84503	-1.83494	-0.97968	-1.60224	-1.4892	-1.26127
O15015	Zinc finger	ZNF646 KIA	-1.18057	-0.70483	-0.64264	-0.85681	-0.63413	-0.45194	-1.23266	-0.65535	-0.71049	-0.44786
O15027	Protein tra	SEC16A KIA	-1.85042	-1.50697	-1.04328	-1.17306	-0.55552	-0.70454	-1.17585	-1.28273	-0.98126	-0.48301
O15042	U2 snRNP-a	U2SURP KIA	-0.23661	-0.29298	0.101614	-0.18015	-0.04435	-0.28951	0.104926	-0.1734	-0.03115	-0.08029
O15047	Histone-lys	SETD1A KIA	-1.14131	-1.02364	-1.16776	-1.38013	-0.57712	-0.29547	-0.51012	-0.42212	-0.72123	-0.89875

O15050	TPR and an	TRANK1 KI/	-0.90613	-0.7632	-0.44923	-0.5881	-0.42	-0.41504	-0.77323	-0.93289	-0.79796	-0.64137
O15056	Synaptojan	SYNJ2 KIAA	-1.25096	-1.26459	-1.18265	-1.02531	-0.83173	-0.87447	-1.3322	-1.36257	-0.62803	-0.8893
O15067	Phosphorib	PFAS KIAA(	-1.76553	-1.20945	-1.09675	-1.45317	-1.15697	-0.88452	-1.66382	-1.13179	-1.24616	-0.60054
O15111	Inhibitor of	CHUK IKKA	1.059379	0.899071	0.812914	0.705257	0.703868	0.778973	0.899368	0.63005	-1.65571	-1.86584
O15117	FYN-bindin	FYB1 FYB S	-0.47763	-0.61752	-0.08972	-0.20648	-0.2974	-0.27621	-0.48208	-0.55658	-0.28431	-0.30626
O15118	NPC intrace	NPC1	-0.5639	-0.27872	0	0.4899	-0.25118	0.074547	0.595158	0.177538	-1.07748	-0.41209
O15120	1-acyl-sn-g	AGPAT2	-0.58193	-0.78319	-0.65486	-0.36976	-0.71727	-1.06711	-0.80863	-0.83773	-1.04793	-1.28264
O15126	Secretory c	SCAMP1 SC	-0.76553	-1.26459	-1.10512	-0.69574	-0.81855	-1.11366	-0.97968	-1.24936	-1.10764	-1.31531
O15127	Secretory c	SCAMP2	-0.07089	-0.03559	-0.13906	-0.03171	-0.37479	-0.16625	-0.25858	-0.23261	-0.24097	-0.24553
O15143	Actin-relate	ARPC1B AR	0.348629	0.502131	0.380265	0.276041	0.225613	0.209782	0.302385	0.30714	0.404034	0.305333
O15144	Actin-relate	ARPC2 ARC	1.114016	1.195949	0.453986	0.513883	0.137986	0.424454	0.600639	0.464913	0.19587	0.047718
O15145	Actin-relate	ARPC3 ARC	-0.77849	0.261602	-0.86014	-0.98998	-1.14002	-0.90797	-0.4957	-0.39516	-0.71902	-1.18453
O15160	DNA-direct	POLR1C PC	0.22785	-0.05743	-0.1151	-0.07469	0.09876	-0.01066	-0.28287	-0.04404	0.153078	-0.09643
O15164	Transcripti	TRIM24 RN	-2.76553	-2.03953	-0.82981	-0.42042	-0.67131	-1.10155	-0.87462	-0.66826	-0.83995	-0.69342
O15173	Membrane	PGRMC2 D	-0.98718	-1.01626	-1.71483	-1.50685	-0.74193	-1.23704	-1.92224	-1.99837	-1.59918	-1.56737
O15212	Prefoldin s	PFDN6 HKE	0.446021	0.711643	0.552244	0.803819	0.617662	0.416166	0.678895	0.495682	0.600958	0.985254
O15213	WD repeat	WDR46 BIN	-0.38646	-0.43273	-1.0593	-0.83171	-0.6668	-0.75004	-0.67608	-1.18058	-0.92867	-0.65894
O15231	Zinc finger	ZNF185	1.693897	0.566651	1.478447	1.476438	0.875821	0.871485	1.094068	0.988504	0.625255	2.833045
O15234	Protein CA	CASC3 MLN	-1.49044	-1.36271	-2.06935	-1.25212	-2.01998	-1.77143	-1.21838	-1.04785	-1.34775	-0.71739
O15270	Serine paln	SPTLC2 KIA	-2.85042	-2.00782	-2.44392	-3.59756	NA	NA	-3.3322	-2.97728	-2.02857	-2.39453
O15294	UDP-N-ace	OGT	-0.02524	-0.21834	-0.58379	-0.52678	-0.58414	-0.36935	-0.36375	-0.79155	-0.876	-1.03019
O15305	Phosphoma	PMM2	-0.27479	-0.64526	0.034215	-0.2018	0.115313	0.02652	-0.32613	-0.26305	0.034389	0.243942
O15320	Melanoma	MIA2 CTAG	0.215872	0.215015	0.442748	0.52527	0.303639	0.347252	0.444941	0.373427	0.149152	0.014847
O15327	Inositol pol	INPP4B	-1.94137	-1.48228	-1.42833	-1.77715	-2.07977	-1.09048	-1.86707	-1.50064	-0.84516	-1.03508
O15347	High mobil	HMGB3 HM	1.088176	1.12683	1.657164	1.498763	1.60011	1.684201	1.742293	1.900633	1.721311	1.483215
O15355	Protein pho	PPM1G PPI	-0.73495	-0.57719	-0.31845	-0.42047	-0.24731	-0.47594	-0.25718	-0.34024	-0.08559	-0.64982
O15357	Phosphatid	INPPL1 SHI	-1.57289	-1.96153	-1.45457	-1.35364	-2.55552	-2.16175	-1.81762	-1.46949	-1.95361	-1.41799
O15371	Eukaryotic	EIF3D EIF3S	0.449307	0.687218	0.357615	0.412452	0.301434	0.275135	0.459283	0.347692	0.361852	0.396141
O15372	Eukaryotic	EIF3H EIF3S	0.135087	0.196539	0.23017	0.411898	0.107446	-0.14958	0.113114	0.340133	0.1133	0.174834
O15379	Histone dea	HDAC3	0.290058	0.548103	-1.91099	-1.62247	0.168271	0.259387	-0.15656	0.067114	-1.80207	-2.01606
O15381	Nuclear val	NVL NVL2	0.484561	0.618366	0.817347	0.812721	0.83054	0.986273	0.820451	1.005714	0.776035	0.31305
O15382	Branched-c	BCAT2 BCA	-0.18052	0.002446	0.206636	0.390348	0.276487	-6.21E-05	0.001051	-0.3753	0.066931	0.357375
O15400	Syntaxin-7	STX7	-1.45666	-1.31381	-1.33536	-1.09358	-1.23002	-1.18406	-1.52065	-1.25426	-1.2709	-1.09741

O15427	Monocarbo	SLC16A3 M	-0.79679	-1.15634	-0.57138	-0.04459	-0.42999	-0.2962	-0.88216	0.764187	-0.46297	-0.0268
O15446	DNA-direct	POLR1G AS	-0.32605	-0.28354	-0.82443	-1.07165	-1.47993	-0.96878	-0.57385	-0.94747	-1.21857	-0.71165
O15498	Synaptobre	YKT6	-0.79637	-0.22563	-0.36866	-0.47702	-0.72926	-0.48429	-0.22337	-0.87779	-0.86	-0.34205
O15511	Actin-relate	ARPC5 ARC	-0.20655	-0.04626	0.459427	0.287587	0.441837	0.14095	0.172147	0.213796	0.622524	0.801453
O15514	DNA-direct	POLR2D	-0.00169	0.391273	0.055413	0.112864	0.32929	0.03414	0.106649	0.190103	0.551424	0.446715
O15523	ATP-depen	DDX3Y DBY	0.712513	0.749389	0.27919	0.516454	-0.71727	-0.77761	0.173332	0.045088	0.173065	0.9063
O15530	3-phosphoi	PDPK1 PDK	0.573582	0.679137	0.750122	0.221643	0.658737	0.680986	0.578467	0.232832	0.370352	0.11649
O15533	Tapasin (TF	TAPBP NGS	-0.21963	-0.32435	-0.98297	-0.73967	-1.16724	-0.07562	-0.77412	-0.87682	-0.05275	-0.10273
O15541	E3 ubiquiti	RNF113A R	-0.71659	-0.66011	-0.75619	-0.9378	-0.73872	-1.08324	-0.85246	-1.04703	-0.42051	-0.27409
O43143	Pre-mRNA-	DHX15 DBF	0.456039	0.613963	0.157625	0.226841	0.200929	0.431339	0.743092	0.63743	0.326258	0.137436
O43148	mRNA cap	RNMT KIAA	0.68451	1.032273	0.498523	0.653618	0.706673	0.601254	0.604851	0.714766	0.543182	0.609827
O43149	Zinc finger	ZZEF1 KIAA	0.149576	-0.39183	-0.24503	0.003899	-0.43814	-0.11084	-0.10661	-0.13686	-0.45095	-0.18046
O43159	Ribosomal	RRP8 KIAA(	0.525265	0.377365	0.726244	0.798257	0.523854	0.489467	0.623341	0.595297	0.833092	0.850663
O43169	Cytochrom	CYB5B CYB	-2.02846	-1.8852	-2.50081	-2.84201	-2.91526	-3.61753	-2.96311	-1.64608	-2.07238	-2.55459
O43172	U4/U6 sma	PRPF4 PRP4	-1.09029	-1.30907	-1.58303	-1.23289	-0.77973	-1	-1.26645	-1.3975	-1.51372	-1.54201
O43175	D-3-phospl	PHGDH PG	-0.43539	-0.20945	-0.03154	0.018696	0.168271	0.140481	-0.48134	-0.1838	0.055495	0.034976
O43181	NADH dehy	NDUFS4	0.634165	0.553125	0.204335	0.302131	0.127464	0.090584	0.222186	-0.06078	0.234157	0.194858
O43189	PHD finger	PHF1 PCL1	-0.88589	-0.93222	-0.67273	-0.97718	-0.27	-0.56331	-0.83762	-0.63469	-0.6325	-0.43959
O43237	Cytoplasmi	DYNC1LI2 I	0.329184	-0.18265	0.625965	0.563901	0.239291	0.263945	0.31988	-0.07039	0.214287	0.190431
O43242	26S protea	PSMD3	-0.02828	0.155119	0.530614	0.645806	0.614406	0.543053	0.318649	0.552667	0.696808	0.516905
O43252	Bifunctiona	PAPSS1 ATI	0.080346	0.408446	-0.401	-0.13251	-0.3404	-0.81085	-0.45921	-0.12956	-0.05128	-0.19458
O43290	U4/U6.U5 t	SART1	-1.06193	-0.95395	-0.99624	-1.21613	-0.95375	-0.66575	-0.8687	-0.83144	-0.62209	-0.4524
O43314	Inositol hex	PPIP5K2 HI	-0.25429	0.146285	0.255165	0.069805	0.213355	0.044559	0.073802	-0.17348	-0.06922	0.176786
O43318	Mitogen-ac	MAP3K7 TA	-0.19438	0.049819	0.393431	-0.48794	-0.10806	-0.07285	-0.03626	-0.2091	0.023383	0.217326
O43324	Eukaryotic	EEF1E1 AIM	-0.08542	-0.07084	-0.31338	-0.10985	-0.31841	-0.25651	-0.30515	-0.25296	-0.09459	-0.32811
O43325	LYR motif-c	LYRM1	0.229304	0.620903	0.465798	0.958641	0.903913	0.983512	0.893872	0.677356	0.218346	0.570135
O43390	Heterogene	HNRNPR HI	0.636564	0.672493	0.565889	0.395284	0.45812	0.297247	0.554387	0.407919	0.569231	0.783271
O43395	U4/U6 sma	PRPF3 HPR	0.549851	0.632828	0.438638	0.17177	0.48755	0.453329	0.589689	0.711347	0.473831	0.416201
O43396	Thioredoxir	TXNL1 TRP3	-0.27063	-0.22004	-0.06209	-0.07615	-0.18233	-0.01202	-0.09153	0.121146	-0.22237	0.020529
O43399	Tumor prot	TPD52L2	0.655929	0.988255	0.801774	0.783867	1.022139	1.040414	0.950561	0.917538	1.102362	1.056413
O43402	ER membra	EMC8 C16c	-2.80735	-1.58887	-1.13906	-1.2909	-1.15697	-1.48981	-1.20881	-1.65535	-0.54314	-0.96502
O43422	52 kDa rep	THAP12 DA	-0.0183	0	0.504792	0.772895	0.739937	0.451461	0.522227	0.427862	0.390071	0.052151
O43426	Synaptojan	SYNJ1 KIAA	-2.80735	-1.97679	-3.48699	-1.82312	-2.55552	-2.83494	-2.09516	-2.04654	-2.16993	-1.66223

O43427	Acidic fibro	FIBP	0.984765	1.137504	1.01744	0.906054	1.19125	1.08231	0.880039	0.75743	0.949626	1.293324
O43432	Eukaryotic	EIF4G3	-0.49427	-0.3843	-0.21876	-0.08386	-0.40054	-0.53688	-0.2757	-0.32339	-0.68881	-0.61343
O43447	Peptidyl-pr	PPIH CYP2(	0.165878	-0.24136	1.021288	0.63429	0.851298	1.142958	1.072337	1.389042	0.922521	0.798477
O43488	Aflatoxin B	AKR7A2 AF	-0.94062	-0.68589	-2.43078	-2.0126	-0.39046	-0.72247	-0.17377	-0.19592	-2.0287	-2.07842
O43491	Band 4.1-lil	EPB41L2	-0.83954	-0.7176	-0.50901	-1.10402	-0.53382	-0.57679	-0.7997	-0.69188	-0.52268	-0.89752
O43493	Trans-Golg	TGOLN2 TG	0.026991	0.17552	0.016045	0.223672	-0.32093	0.102375	0.406704	0.066926	0.024683	-0.2301
O43513	Mediator o	MED7 ARC:	0.777608	0.88436	1.130819	0.439438	0.950834	0.68012	0.709072	0.908416	0.971431	0.938984
O43516	WAS/WASI	WIPF1 WAS	-0.6002	-0.69218	-0.81028	-0.98751	-1.06075	-0.80814	-0.7997	-0.53434	-0.65748	-0.63448
O43521	Bcl-2-like p	BCL2L11 BI	-1.80735	-2.15634	-2.26459	-1.84549	-3.27798	-2.83494	-1.55459	-1.58496	-1.78165	-1.82517
O43561	Linker for a	LAT	-0.86581	-0.84174	-0.94699	-1.27433	-0.6986	-1.25669	-1.28813	-1.13121	-0.74151	-0.5153
O43566	Regulator c	RGS14	0.04182	-0.13478	0.049819	-0.02531	0.181447	0.254814	-0.02581	0.110183	0.069042	0.122947
O43583	Density-reg	DENR DRP1	0.426519	0.440771	0.802778	0.791523	0.96961	0.783902	0.790363	0.740278	0.94082	0.899783
O43586	Proline-ser	PSTPIP1 CD	-0.41482	-0.5484	-0.12528	0.017725	-0.31964	-0.30926	-0.10579	0.048813	-0.31444	-0.63197
O43598	2'-deoxynu	DNPH1 C6c	0.135214	0.323039	-0.70275	-0.35542	-0.15402	-0.30249	-0.0495	-0.29173	0.113885	-0.23677
O43615	Mitochond	TIMM44 M	0.793983	0.827261	0.545874	0.47793	0.32871	0.404558	0.49794	0.460463	0.420409	0.390712
O43617	Trafficking	TRAPPC3 B	0.711848	0.822611	0.647107	0.363881	0.416368	0.343655	0.216853	0.344817	0.577996	0.497464
O43633	Charged m	CHMP2A B	-2.01612	-1.97993	-1.53922	-1.72212	-1.66918	-2.04439	-1.55271	-1.55514	-1.11283	-1.13874
O43660	Pleiotropic	PLRG1	-2.07357	-1.52333	-1.43231	-2.07012	-2.1655	-2.54123	-2.35391	-1.93041	-1.77847	-1.27927
O43665	Regulator c	RGS10	-0.00607	-0.22304	0.253253	0.638326	0.554905	1.239088	0.712198	1.172467	1.407948	1.131052
O43670	BUB3-inter	ZNF207 BU	0.616393	0.677489	1.060775	1.338286	0.84717	0.927149	1.075532	0.931543	1.1133	1.67364
O43674	NADH dehy	NDUFB5	-0.16011	-0.5427	-1.64874	-0.86821	-1.69302	-0.86449	-0.38466	-1.72935	-2.04793	-1.79414
O43676	NADH dehy	NDUFB3	0.26	-0.08844	-0.69849	-0.0973	-0.45017	0.52166	0.108376	0.251539	-1.33852	-0.97369
O43678	NADH dehy	NDUFA2	-0.2949	-0.11353	1.329628	1.224613	1.15831	1.328471	1.210736	1.237579	1.23246	1.140138
O43681	ATPase GE	GET3 ARSA	0.326217	0.476081	-0.02488	-0.1235	-0.05057	-0.33428	0.045145	-0.57149	-0.32278	-0.11919
O43684	Mitotic che	BUB3	0.486494	0.657397	0.124933	0.068901	0.069083	0.190847	0.237236	0.427157	0.31809	0.236376
O43704	Sulfotransf	SULT1B1 S	-1.14049	-0.85422	-0.64662	-0.92226	-0.39287	-0.04722	-0.85084	-0.79864	0.021344	-0.02909
O43707	Alpha-actir	ACTN4	0.937045	0.795048	0.101614	0.572362	-0.47914	0.100264	0.368243	0.406657	-0.74188	-0.62797
O43715	TP53-regul	TRIAP1 15E	-1.1669	-1.09675	-1.04757	-1.2909	-1.57755	-1.85457	-1.52484	-1.08246	-0.9906	-1.66223
O43716	Glutamyl-tl	GATC 15E1	-1.60942	-1.72403	-1.53138	-1.15899	-1.76702	-1.74061	-3.01027	-1.50155	-1.54314	-1.63448
O43719	HIV Tat-spe	HTATSF1	-0.48543	-0.152	-0.18791	-0.36167	-0.13992	-0.0383	-0.30035	-0.78746	0.001308	-0.09471
O43747	AP-1 comp	AP1G1 ADT	-1.1004	-1.35347	-0.04757	-0.51457	-0.46036	-0.11958	-0.30035	0.146841	-0.13327	-0.2402
O43752	Syntaxin-6	STX6	-1.25096	-0.97679	-1.37151	-1.5787	-1.64572	-0.59318	-1.31937	-0.69188	-1.2687	-1
O43765	Small gluta	SGTA SGT S	0.142817	0.173343	0.110783	0.251619	0.538431	0.104136	0.329113	0.145561	0.357247	0.389281

O43768	Alpha-endc	ENSA	1.072623	1.191905	0.989138	1.103796	1.080779	0.782676	1.177615	1.068921	1.132294	1.219239
O43772	Mitochond	SLC25A20 (	1.547871	1.292214	-0.15791	0.071017	-0.58054	-0.01998	0.309926	0.096323	-1.01177	-1.38041
O43776	Asparagine	NARS1 NAF	0.324351	0.288798	0.378512	0.231326	0.007417	0.125531	0.31988	0.246722	0.649503	0.929721
O43809	Cleavage ai	NUDT21 CF	0.827215	0.955961	-0.01233	0.019169	0.107446	0.155278	0.300624	0.207059	-0.03339	0.03065
O43813	Glutathion	LANCL1 GP	0.214445	0.251318	0.440794	0.373646	0.523634	0.284621	0.501558	0.633098	0.800388	0.816135
O43815	Striatin	STRN	0.620882	0.377651	0.569776	0.563901	0.286657	0.420894	0.428253	0.615177	0.688056	0.954977
O43818	U3 small nu	RRP9 RNU3	0.535635	0.53216	0.661756	0.759291	1.009388	0.634893	1.189124	0.895868	0.532314	0.735921
O43819	Protein SC(	SCO2	-3.25096	-2.48699	-1.90202	-2.84549	-2.89947	-3.04439	-1.73016	-3.33342	-2.21299	-3.26127
O43823	A-kinase ar	AKAP8 AKA	-0.0563	0.250779	-0.22037	-0.27308	0.225949	-0.31116	0.231942	-0.10752	-0.17255	-0.03521
O43824	Putative G1	GTPBP6 PG	0.812541	0.618366	0.641046	0.630243	0.354283	0.095157	0.981982	1.064379	1.018737	0.843565
O43829	Zinc finger	ZBTB14 ZFI	-2.19545	-2.69208	-2.72081	-2.95317	-1.17365	-0.66069	-1.11718	-1.02308	-2.53695	-2.35155
O43847	Nardilysin (	NRDC NRD	-2.68536	-1.4761	-1.5427	-1.02531	-1.45017	-1.68684	-3.61599	-2.04654	-1.46297	-2.13927
O43852	Calumenin	CALU	-0.85042	-0.69082	0.680507	0.752071	-1.14459	-1.24998	-1.20881	-1.22239	-0.97199	-0.69015
O43865	S-adenosyl	AHCYL1 DC	-1.18057	-0.42286	0.529314	0.542527	0.178819	0.367793	-1.84989	-1.73588	-1.53475	-1.15607
O43914	TYRO prote	TYROBP DA	-1.45187	-3.4021	-1.91667	-1.73697	-1.76702	-1.72247	-3.09516	-1.88982	-2.10764	-2.19898
O43920	NADH dehy	NDUFS5	0.376645	0.240108	0.034673	0.15451	-0.50188	0.475242	0.471473	0.251539	-0.30319	-0.56737
O60216	Double-stra	RAD21 HR2	-0.9395	-0.67755	-0.7733	-0.75803	-0.58908	-1.02032	-0.67492	-0.85834	-0.71049	-0.29754
O60220	Mitochond	TIMM8A D	-1.45035	-1.74279	-0.5225	-1.46368	-2.2571	-1.53234	-1.41653	-1.90304	-0.38065	-0.55186
O60231	Pre-mRNA-	DHX16 DBF	-0.50434	-0.50607	-0.53691	-0.52359	-0.69911	-0.37447	-0.52525	-0.58175	-0.606	-0.57432
O60232	Protein ZNI	ZNRD2 SSS	0.171271	0.023207	0.440162	0.176878	-0.12418	0.203533	0.31988	0.067114	0.018737	0.174834
O60234	Glia matura	GMFG	0.183209	0.128843	0.773351	0.590666	-0.18039	-0.20968	-0.4402	-0.28697	-0.14535	-0.07334
O60256	Phosphorib	PRPSAP2	0.002867	0.145752	0.046929	-0.18594	0.247333	0.355663	0.207441	0.110309	0.513322	0.605679
O60262	Guanine nu	GNG7 GNG	0.730891	0.930098	-0.19153	-0.71621	-0.59991	-0.33015	0.222392	0.152003	-0.22948	-0.42392
O60264	SWI/SNF-re	SMARCA5 S	0.272896	0.214093	-0.22496	-0.07757	-0.18588	0.022783	0.305197	0.158638	-0.13198	-0.19797
O60271	C-Jun-amin	SPAG9 HSS	-0.83502	-0.50768	-0.32462	-0.55318	-0.18206	-0.1315	-0.71246	-0.4122	-0.58524	-0.45265
O60282	Kinesin hea	KIF5C KIAA	-0.48136	-0.80502	-0.09394	-0.10984	0.020483	-0.00912	-0.08041	-0.30986	-0.44699	-0.49386
O60306	RNA helica:	AQR KIAA0	0.187331	0.477047	0.141044	0.170242	-0.38315	-0.13728	-0.02429	0.160964	0.081251	0.487322
O60307	Microtubul	MAST3 KIA	-1.33251	-0.97108	-1.09264	-0.88811	-0.51998	-0.65377	-0.79107	-0.73248	-1.06304	-1.07263
O60308	Centrosom	CEP104 KIA	-0.08104	-0.16506	0.101614	-0.15899	-0.02248	0.169925	0.368243	0.340133	-0.05772	-0.45998
O60313	Dynamin-li	OPA1 KIAA	0.1385	0.181961	0.105794	0.049328	0.291151	0.250227	0.244153	0.360809	0.519575	0.382484
O60318	Germinal-c	MCM3AP G	-2.72489	-1.63656	-0.97679	-0.72655	-0.92749	-0.52869	-0.65574	-0.76783	-0.89984	-0.64829
O60333	Kinesin-like	KIF1B KIAA	-3.1429	-2.68116	-2.30243	-2.19962	-2.89947	-2.04439	-1.30666	-2.02308	-3.20313	-2.01784
O60341	Lysine-spec	KDM1A AO	-0.21138	0.104095	0.363436	0.476438	0.57516	0.483078	0.677854	0.63005	0.115835	0.090973

O60449	Lymphocyt	LY75 CD20!	0.419348	0.822918	0.839694	0.971604	0.559967	0.848211	0.657327	0.583654	0.279892	0.166088
O60488	Long-chain	ACSL4 ACS	-1.06193	-1.19153	-0.70483	-1.03814	-1.87199	-0.86449	-0.83577	-1.05842	-1.54314	-1.50324
O60493	Sorting nex	SNX3	0.667425	0.668291	0.954448	0.948926	0.652753	0.595851	0.415037	0.561879	0.905003	1.024137
O60496	Docking pr	DOK2	-0.0747	0.210086	0.06084	0.192021	0.100527	0.19878	0.288683	0.23756	0.081513	0.064991
O60502	Protein O-(	OGA HEXC	-0.23456	-0.12758	-0.0096	0.011337	-0.0067	0.203252	-0.16992	-0.01395	0.058655	0.124491
O60504	Vinexin (SH	SORBS3 SC	-4.08746	-3.32193	-3.24598	-2.56009	-2.84503	-2.83494	-2.89162	-1.848	-3.5025	-2.30433
O60506	Heterogen	SYNCRIP HI	0.996966	1.107054	0.577143	0.709099	0.862154	0.559227	0.913017	0.780235	0.728454	0.925067
O60508	Pre-mRNA-	CDC40 EHB	-0.38702	-0.42809	-0.6734	-0.73936	-0.5225	-0.40777	-0.77323	-0.2978	-0.06263	-0.19391
O60524	Nuclear ex	NEMF SDC	1.47587	1.025125	1.443033	1.220599	1.391609	1.358618	1.209177	0.988504	1.014075	2.122947
O60547	GDP-mann	GMDS	-0.09597	-0.24485	-0.55403	-1.15472	-0.03694	-0.35047	-0.18785	-0.33827	-0.14981	0.036707
O60551	Glycylpepti	NMT2	0.043124	0.23101	0.274822	0.480687	0.827296	0.789827	0.68109	0.688056	0.562332	0.790379
O60573	Eukaryotic	EIF4E2 EIF4	-1.55497	-1.42286	-1.5541	-0.06413	-1.06075	-1.24998	-1.30666	-1.60224	-1.848	-1.69052
O60610	Protein dia	DIAPH1 DIA	0.532267	0.604053	0.628095	0.487539	0.464303	0.661484	0.453377	0.666486	0.597041	0.701015
O60645	Exocyst cor	EXOC3 SEC	0.290058	0.369485	0.648528	0.646363	1.054723	0.909802	0.788227	0.436348	0.643479	0.733421
O60664	Perilipin-3	PLIN3 M6P	-0.88359	-0.20046	-0.19153	-0.35364	-0.28703	-0.35091	0.055206	-0.05252	-0.44361	-0.08663
O60669	Monocarbo	SLC16A7 M	-0.87245	-0.96153	-0.77649	-0.74746	-0.5231	-0.54455	-0.66382	-0.60224	-0.67996	-1.06333
O60675	Transcripti	MAFK	-0.94062	-1.19153	-0.43862	-1.35364	-1.53382	-1	-0.81762	-0.82753	-1.00946	-1.22979
O60684	Importin su	KPNA6 IPO	-0.23311	-0.1607	-1.68589	-1.3377	-0.6293	-0.72955	-0.45305	-1.21001	-0.75756	-0.60058
O60711	Leupaxin	LPXN LDLP	-0.70499	-1.11471	-1.04757	-0.97513	-0.85845	-1.11366	-0.67195	-0.72935	-1.03822	-0.84093
O60725	Protein-S-is	ICMT PCCM	-0.16011	-0.90933	-0.69218	0.555389	-0.59991	0.105353	-0.81762	0.498454	-0.63534	0.127005
O60749	Sorting nex	SNX2 TRG9	0.409646	0.511434	0.488253	0.256449	0.519481	0.386825	0.340625	0.429775	0.642288	0.590313
O60759	Cytohesin-i	CYTIP PSCD	-1.40296	-1.57719	-1.43335	-1.2909	-1.22487	-0.95693	-1.20881	-1.93289	-1.07748	-1.0726
O60763	General ve	USO1 VDP	0.124706	0.390349	0.41632	0.262087	0.407729	0.135515	0.384011	-0.02302	-0.01228	0.291517
O60783	28S ribosor	MRPS14	-1.38702	-1.43335	-1.42286	-2.89129	-1.51245	-1.40054	-1.39808	-1.74846	-1.46297	-1.56737
O60784	Target of M	TOM1	-0.87681	-0.78665	-0.23948	-0.3677	-0.50382	-0.56682	-0.81041	-0.73706	-0.32914	-0.33594
O60826	Coiled-coil	CCDC22 CX	0.280108	0.847997	0.633525	0.84101	0.766409	0.377671	0.736966	0.367016	0.197939	0.622815
O60828	Polyglutam	PQBP1 NPV	1.07338	1.132739	-1.67311	-1.65141	0.717422	0.144686	-1.66238	-1.43553	-1.819	-1.20132
O60832	H/ACA ribo	DKC1 NOLA	0.741807	0.713292	0.317241	0.266511	0.544664	0.39151	0.446467	0.311862	0.731297	1.094215
O60841	Eukaryotic	EIF5B IF2 K	0.334001	0.332805	0.589594	0.629247	0.534513	0.498638	0.608945	0.51056	0.457226	0.445688
O60869	Endothelial	EDF1	0.833103	0.903247	0.830562	0.765535	1.061865	0.835189	0.929227	0.603921	0.819428	1.302324
O60879	Protein dia	DIAPH2 DIA	-0.13327	-0.03154	-0.16069	0.339385	-0.49139	0.021695	-0.33865	-0.25618	-0.38087	-0.12473
O60880	SH2 domai	SH2D1A DS	1.211278	1.369451	1.119104	1.026506	1.14478	1.294029	1.257914	1.432382	0.82937	0.737403
O60884	DnaJ homo	DNAJA2 CP	0.895716	0.986288	1.314381	1.044232	1.207442	1.138	1.180121	1.336644	1.289507	1.004419

O60885	Bromodom	BRD4 HUNI	0.199882	0.396174	0.651384	0.511511	0.792405	0.330645	0.654865	0.644772	0.781531	0.945893
O60888	Protein Cut	CUTA ACHA	-0.69348	-1.14147	-1.42036	-1.12402	-1.86002	-1.3767	-1.19333	-1.29617	-1.08316	-1.02287
O60907	F-box-like/'	TBL1X TBL1	-3.64689	-3.28345	-0.87317	-0.71621	-1.31451	-1.07861	-0.87276	-0.63743	-0.76553	-0.32082
O60925	Prefoldin su	PFDN1 PFD	0.712503	1.018038	1.211761	1.015173	0.684392	0.882111	0.845738	0.921981	1.24312	1.074703
O60927	E3 ubiquitin	PPP1R11 H	-2.29713	-2.54346	-2.14419	-2.58764	-2.00655	-1.72642	-2.056	-1.58496	-2.20215	-1.65823
O60934	Nibrin (Cell	NBN NBS N	0.495952	0.430625	0.682606	0.745693	0.723985	0.673954	0.493403	0.461626	0.617586	0.775789
O60942	mRNA-capp	RNGTT CAP	-0.54709	-0.53559	-1.24027	-1.05269	-1.06054	-1.4829	-1.31987	-1.30562	-1.48177	-1.45252
O75027	ATP-bindin	ABCB7 ABC	-1.80735	-1.93147	-2.75002	-1.69574	-2.59991	-1.91511	-1.66382	-1.74846	-3.3505	-3.54137
O75044	SLIT-ROBO	SRGAP2 AR	-2.04114	-1.48769	-1.88763	-2.71376	-1.90941	-2.33074	-2.06464	-2.72579	-3.24655	-3.11022
O75083	WD repeat	WDR1	0.341114	0.507225	0.412639	0.30566	0.411314	0.250578	0.489805	0.141661	0.789125	0.484592
O75113	NEDD4-bin	N4BP1 KIA	-0.46855	-0.18709	0.151613	-0.19444	-0.269	0.110424	-0.22666	-0.19592	0.023383	-0.73402
O75116	Rho-associa	ROCK2 KIA	-1.10879	-1.24846	-0.62203	-0.99761	-1.26271	-0.78014	-1.34114	-1.53003	-1.06956	-1.07423
O75122	CLIP-associ	CLASP2 KIA	-0.63743	-0.68306	-0.5242	-0.77344	-0.85249	-0.6372	-0.4957	-0.50164	-0.51592	-0.69052
O75131	Copine-3 (C	CPNE3 CPN	-0.37126	-0.29298	-0.10932	-0.04459	-0.02248	-0.06711	0	-0.26303	-0.04307	-0.296
O75143	Autophagy	ATG13 KIA	-2.92356	-3.07232	-1.72403	-1.47045	-1.12418	-1.38618	-1.83577	-1.07039	-1.4245	-1.06333
O75146	Huntingtin-	HIP1R HIP1	-1.05097	-0.25282	-0.07195	-0.2986	-0.88234	-1.09604	-0.78189	-0.86569	-1.38702	-1.04495
O75150	E3 ubiquitin	RNF40 BRE	-0.81503	-0.54033	-0.79426	-0.5283	-0.75732	-0.93413	-1.08486	-0.60402	-0.46953	-0.58801
O75151	Lysine-spec	PHF2 CENP	-0.9523	-0.74348	-0.98449	-0.85681	-1.03006	-1.03317	-1.24473	-0.95491	-0.67996	-0.73402
O75152	Zinc finger	ZC3H11A K	-0.52293	-0.43818	-0.51668	-0.68872	-0.24103	-0.59218	-0.62405	-0.63359	-0.25256	-0.4064
O75157	TSC22 dom	TSC22D2 K	-1.13163	-1.55256	-0.98628	-0.98489	-1.04467	-1.15643	-2.16995	-0.77272	-1.12605	-1.16968
O75167	Phosphatas	PHACTR2 C	0.446899	0.362589	-1.52025	-1.85494	-1.78875	-1.801	-1.53083	-1.39262	-2.05005	-1.64582
O75170	Serine/thre	PPP6R2 KIA	-2.77868	-1.77394	-1.89199	-2.91865	-1.72358	-2.40895	-2.93095	-2.52858	-2.44084	-2.54092
O75175	CCR4-NOT	CNOT3 KIA	-1.94591	-1.40449	-1.45026	-1.96586	-1.90745	-2.03047	-1.76598	-1.72172	-1.98274	-1.48514
O75190	DnaJ homo	DNAJB6 HS	-0.66935	-0.81809	-0.70826	-0.716	-1.34784	-0.25724	-0.95671	-1.01998	-0.98716	-1.03779
O75208	Ubiquinone	COQ9 C16c	-1.96017	-1.71905	-1.09485	-1.41767	-1.19523	-1.14658	-1.84199	-1.71983	-1.06506	-0.97411
O75223	Gamma-glu	GGCT C7or	1.508501	1.662391	1.106863	1.373335	1.376589	1.567105	1.371712	1.308822	1.209941	0.848888
O75251	NADH dehy	NDUFS7	-0.18057	-0.27399	0.291986	-0.28325	-0.34252	-0.30293	-0.28157	-0.52607	-0.05282	-0.26127
O75306	NADH dehy	NDUFS2	0.187331	-0.03154	0.348202	0.383329	-0.19903	0.250227	0.846291	0.222392	0.230452	0.265982
O75312	Zinc finger	ZPR1 ZNF2!	-1.20893	-1.20813	-0.19519	-0.15733	-0.27869	-0.39076	-0.6029	-0.03369	-0.11317	-0.01881
O75319	RNA/RNP c	DUSP11 PII	-0.30986	-0.23217	0.199938	0.079322	0.360089	0.290996	0.030374	0.172467	0.230452	-0.04495
O75323	Protein Nip	NIPSNAP2 (	-0.69515	-1.18265	-0.73048	0.182416	-0.89947	-0.64351	-1.02061	-1.74846	-0.85651	-0.59384
O75347	Tubulin-spe	TBCA	0.469096	0.482639	0.717626	0.424288	0.524208	0.419825	0.510276	0.648429	0.955568	0.881982
O75348	V-type prot	ATP6V1G1	0.006049	-0.53703	-0.13906	0.304257	0.762031	0.525461	0.244153	0.534473	0.382857	0.056413

O75351	Vacuolar pı	VPS4B SKD	0.843081	0.749307	0.958013	0.520826	0.848939	0.590571	0.96407	0.802677	0.797578	0.784784
O75367	Core histor	MACROH2/	1.128947	0.948399	0.675633	0.653816	0.800967	0.810474	0.958481	0.962299	0.725606	0.821197
O75368	SH3 domai	SH3BGRL	1.085361	1.352068	0.673246	0.899344	0.917143	0.895852	0.82717	0.630761	0.760868	0.662391
O75369	Filamin-B (I	FLNB FLN1I	-0.25819	-0.26929	-0.61375	-0.55813	-0.56649	-0.61813	-0.68834	-0.54287	-0.02713	0.0399
O75376	Nuclear rec	NCOR1 KIA	-1.25821	-1.02367	-0.53288	-0.32201	-0.87728	-0.31284	-0.84461	-0.94786	-0.44873	-0.44576
O75380	NADH dehy	NDUFS6	1.471504	1.174251	1.515742	1.220599	1.348454	1.217701	1.428405	1.197646	1.489126	1.518551
O75390	Citrate synt	CS	1.151365	1.28613	1.0275	0.819758	0.530983	0.871144	0.86022	0.832276	0.891398	0.881027
O75391	Sperm-assc	SPAG7	-1.60942	-1.08459	-1.84488	-2.0906	-1.81855	-2.02203	-2.05209	-1.848	-1.64268	-1.66223
O75394	39S ribosor	MRPL33 C2	1.796926	1.545434	1.70941	2.120294	2.18799	2.044394	2.224583	2.177538	2.244449	2.156091
O75396	Vesicle-tra	SEC22B SE(	0.481856	0.591764	0.348407	-0.0409	0.270452	0.06755	0.124233	-0.02409	-0.18224	-0.13919
O75398	Deformed (	DEAF1 SPN	-1.60234	-1.70233	-1.16689	-0.9371	-1.04696	-1.33494	-1.19895	-1.29248	-0.75607	-1.03405
O75400	Pre-mRNA-	PRPF40A FI	0.052591	0.215179	0.153059	0.099434	0.231675	0.195257	0.025347	0.272648	0.334905	0.082081
O75410	Transformi	TACC1 KIA/	-0.25819	-0.75002	0.083328	-0.36976	-0.28703	-0.33015	-0.51019	-0.71989	-0.58496	-0.68339
O75420	GRB10-inte	GIGYF1 CD!	-0.72712	-0.56196	-0.12996	-0.52165	-0.47907	-0.58254	-0.4057	-0.49914	-0.24612	-0.39834
O75427	Leucine-ric	LRCH4 LRN	-0.96094	-0.91549	-0.65232	-0.65634	-0.61883	-0.69863	-1.16291	-0.82439	-0.75756	-0.56383
O75431	Metaxin-2 (	MTX2	-0.98556	-1.01689	-1.11104	-1.4354	-1.33234	-0.83782	-0.87932	-1.1705	-1.10099	-1.03824
O75436	Vacuolar pı	VPS26A VP	-0.99169	-0.86459	-1.19844	-0.77691	-1.55784	-1.70027	-1.24438	-1.42473	-1.08746	-1.05267
O75438	NADH dehy	NDUFB1	0.093867	-0.25992	-0.63656	-0.44461	-0.51245	-0.10776	-0.32577	-0.60224	-1.57089	-1.45391
O75439	Mitochond	PMPCB MP	-0.60865	-0.62861	-0.26984	-0.24146	-0.8308	-0.33552	-0.53803	-0.28771	-0.30941	-0.38299
O75446	Histone de	SAP30	-2.80735	-1.7112	-1.53138	-2.9386	-1.15697	-2.02203	-2.01027	-1.88982	-2.1913	-1.6763
O75448	Mediator o	MED24 AR(	-1.39844	-1.75229	-1.24456	-1.01929	-1.81214	-1.96493	-1.66391	-1.53596	-0.83357	-0.46969
O75475	PC4 and SF	PSIP1 DFS7	0.633852	0.6277	0.569281	0.421228	0.816551	0.554334	0.808816	0.608778	0.353892	0.377742
O75489	NADH dehy	NDUFS3	-0.06428	0.139013	0.276358	0.365509	0.173775	0.346081	0.509757	0.201548	0.338045	0.502909
O75494	Serine/argi	SRSF10 FUS	0.996727	1.271794	1.184581	1.38938	1.284396	0.949227	1.35121	1.278805	1.155721	1.37749
O75506	Heat shock	HSBP1 HSF	-0.78629	-0.41764	-1.19153	-0.7794	-1.35199	-0.59318	-0.52484	-0.41504	-0.37474	-1.09134
O75521	Enoyl-CoA (	ECI2 DRS1	-0.6002	-0.54839	-0.25526	-0.32979	-0.12418	-0.58496	-0.43219	-0.20249	-0.24616	-0.01781
O75528	Transcripti	TADA3 ADA	-3.30986	-1.58887	-2.58359	-2.77062	-1.07635	-1.07861	-1.01027	-1.26303	-2.75493	-2.06309
O75529	TAF5-like R	TAF5L PAF(	-1.76553	-1.93147	-1.63656	-3.67557	-3.69302	-1.72247	-1.41163	-2.848	-2.16993	-1.80957
O75531	Barrier-to-a	BANF1 BAF	1.109262	1.105458	1.262208	1.10693	1.259691	1.484954	1.240434	1.14689	0.726568	0.622022
O75533	Splicing fac	SF3B1 SAP1	0.244734	0.558731	0.206711	0.216116	0.43351	0.389848	0.302796	0.289507	0.371969	0.611274
O75534	Cold shock	CSDE1 D1S	-0.21444	-0.02008	0.094309	-0.01416	0.116327	0.370901	0.207456	0.097306	0.271073	-0.20142
O75554	WW domai	WBP4 FBP2	0.165878	0.034673	0.763239	0.981058	0.534513	0.778973	0.564352	0.607683	0.751041	0.725413
O75558	Syntaxin-1:	STX11	-3.03684	-1.68589	-1.5427	-1.89129	-3.2076	-1.87447	-1.782	-3.07039	-2.21299	-4.44184

O75564	Jerky prote	JRK JH8	-1.43539	-0.8519	-0.79667	-0.91475	-1.07635	-0.40777	-0.60817	-1.07039	-0.74959	-0.93088
O75569	Interferon-	PRKRA PAC	0.018072	0.342063	0.597902	1.085247	-0.09212	0.577658	0.584963	-0.25618	0.060025	0.202019
O75582	Ribosomal	RPS6KA5 M	1.149576	1.149857	0.970456	1.215206	1.294905	1.337149	1.204745	1.005714	0.853748	0.803557
O75592	E3 ubiquitii	MYCBP2 KI	-1.34023	-1.00782	-0.61252	-0.87971	-0.59996	-0.66069	-0.80863	-0.85834	-0.50603	-1.01781
O75608	Acyl-protei	LYPLA1 APT	-0.22992	-0.20042	0.089228	-0.21195	-0.07601	0.124556	-0.03	0.132904	0.156392	-0.12114
O75643	U5 small nu	SNRNP200	0.239609	0.437285	0.068531	-0.00629	-0.12418	0.134476	0.017687	0.157064	0.165126	0.186547
O75663	TIP41-like p	TIPRL	0.099436	0.111753	-0.28668	-0.50663	-0.18853	-0.52973	-0.30095	-0.17735	-0.15454	-0.25441
O75683	Surfeit locu	SURF6 SUR	-0.27277	-0.16506	-0.14336	-0.32193	-0.1161	-0.24998	-0.0897	-0.50155	-0.14886	0.127005
O75688	Protein pho	PPM1B PP2	0.314636	0.437285	0.852365	0.613942	0.67155	0.595851	0.445411	0.733527	1.069042	1.047877
O75694	Nuclear po	NUP155 KIA	-0.68619	-0.47078	-0.55029	-0.74217	-0.45188	-0.613	-1.782	-1.33342	-1.54314	-1.66223
O75695	Protein XRI	RP2	-2.64689	-1.78991	-3.28345	-3.32193	-3.69302	-2.75899	-2.18536	-1.78746	NA	NA
O75746	Calcium-bir	SLC25A12 A	0.007676	0.154702	-0.29904	-0.18087	-0.3234	-0.1144	-0.37404	-0.49748	-0.55014	-0.29729
O75764	Transcriptio	TCEA3 TFIIS	-0.23661	-0.30256	-0.31706	-0.22344	-0.42	-0.52083	-0.08427	-0.0115	0.360998	0.419456
O75791	GRB2-relat	GRAP2 GAD	1.229304	1.393431	0.206711	0.263034	0.007417	0.369234	0.136573	0.227291	0.368321	0.284267
O75792	Ribonuclea	RNASEH2A	0.461282	0.573412	0.53061	0.527871	0.254026	0.389662	0.55388	0.554222	0.348312	0.257272
O75808	Calpain-15	CAPN15 SC	-3.89482	-4.83094	-4.07195	-4.03814	NA	NA	NA	NA	-3.79796	-3.49075
O75817	Ribonuclea	POP7 RPP2	0.235256	0.201912	0.167147	0.073373	0.17121	-0.00582	-0.32898	0.077635	0.46596	0.646069
O75818	Ribonuclea	RPP40 RNA	-2.00127	-1.57214	-2.22518	-1.96763	-1.91131	-1.47175	-2.48995	-2.73135	-1.83907	-2.04877
O75821	Eukaryotic	EIF3G EIF3S	0.79518	0.690675	1.040371	0.783867	0.50935	1.013598	0.863264	0.662965	1.037233	0.767616
O75822	Eukaryotic	EIF3J EIF3S	0.584005	0.67319	-0.12326	-0.2909	0.181447	-0.52083	-0.1452	0.190694	-0.24058	0.021959
O75828	Carbonyl re	CBR3	-0.20243	-0.15729	-0.64874	-0.69574	0.489342	0.525052	-0.06289	-0.0734	0.869896	0.938938
O75832	26S protea	PSMD10	0.03592	-0.34156	-0.01568	0.286914	0.128008	0.021695	0.090429	-0.0803	-0.24058	-0.2507
O75843	AP-1 comp	AP1G2	-2.32793	-2.08549	-2.58189	-2.64292	-0.8055	-1.12553	-0.62385	-0.95491	-0.96277	-0.89752
O75844	CAAX preny	ZMPSTE24	-1.23661	-1.44392	-0.78319	-1.27563	-1.55552	-0.56866	-1.19704	-0.74846	-1.64268	-1.74882
O75874	Isocitrate d	IDH1 PICD	-0.57289	-0.48699	-0.44491	-0.52376	-0.63413	-0.25649	-0.15333	-0.1193	-0.53629	-0.62324
O75880	Protein SCO	SCO1 SCOD	-0.76553	-1.31221	-1.32193	-1.45317	-1.57755	-1.18641	-1.19704	-1.1193	-1.3505	-1.6763
O75884	Serine hydr	RBBP9 BOG	0.024045	0.158617	-0.13478	-0.11078	-0.18206	0.074547	-0.09516	-0.32623	0.082462	0.351712
O75909	Cyclin-K	CCNK CPR4	-0.42721	-0.04757	-0.998	-1.61783	-0.32379	-0.33015	-0.65574	-0.24257	-2.01397	-2.39135
O75915	PRA1 famil	ARL6IP5 DE	-0.46855	-0.75659	1.208399	0.816288	1.011112	0.862496	0.893872	0.461512	0.443052	0.642794
O75934	Pre-mRNA-	BCAS2 DAM	0.385953	0.342063	0.584963	0.435281	0.570012	0.490881	0.601916	0.561879	0.773279	0.518551
O75935	Dynactin su	DCTN3 DCT	-0.49394	-0.61252	-0.57719	-0.44461	-0.40024	-0.88452	-0.36476	-0.50155	-0.36864	-0.10555
O75937	DnaJ homo	DNAJC8 SP	1.054199	0.960096	1.061376	1.068411	1.105487	1.056912	0.997813	1.04125	1.364664	1.182482
O75940	Survival of	SMNDC1 SI	-0.43539	-0.19153	-0.10932	-0.30633	-0.1161	-0.24349	-0.50293	-0.36995	0.073529	0.202019

O75947	ATP syntha	ATP5PD AT	0.432039	0.378534	0.123255	0.433481	-0.15288	0.578216	0.242789	0.288435	-0.32278	-0.22761
O75955	Flotillin-1	FLOT1	-0.6002	-0.44923	-0.51994	-0.49235	-0.71727	-0.48215	-0.40484	-0.64636	-0.06263	0.139112
O75956	Cyclin-depe	CDK2AP2 D	-0.83705	-0.89665	-0.44723	-0.05539	0.046194	-0.22217	-0.08428	0.079103	-0.12168	-0.03022
O75964	ATP syntha	ATP5MG AT	2.236636	1.704686	0.587837	0.998744	-0.28166	1.370641	1.47767	0.895583	-0.12259	-0.28919
O75995	SAM and SI	SASH3 CXo	-0.09862	-0.04302	0.241466	0.103431	0.132769	-0.33068	-0.01579	-0.06635	0.042659	0.418927
O76003	Glutaredox	GLRX3 PICO	0.818138	1.025834	0.675284	0.758093	0.439902	0.627381	0.661334	0.477139	0.68156	0.506383
O76021	Ribosomal	RSL1D1 CA	-0.21505	-0.22675	-0.25061	-0.47917	-0.45017	-0.16788	-0.73769	-0.43039	-0.51592	-0.22768
O76038	Secretagog	SCGN SECR	1.27761	1.271135	0.52661	-0.32193	1.362983	0.880418	-0.45305	-0.58496	0.973833	0.665292
O76071	Probable cy	CIAO1 CIA1	-0.88359	-0.97679	-1.2276	-1.06413	-0.9852	-0.86329	-0.96963	-0.885	-1.33852	-1.40621
O76094	Signal reco	SRP72	-0.88572	-0.86367	0.055702	0.196903	0.24266	0.059469	0.147079	0.211917	-0.22073	-2.72E-05
O94763	Unconvent	URI1 C19or	-1.59208	-0.95001	-1.35271	-1.18312	-0.88653	-1.33983	-1.53604	-1.20456	-0.50437	-0.87931
O94766	Galactosylg	B3GAT3	-2.45964	-2.53209	-1.85896	-1.96285	-3.79256	-1.37197	-2.64237	-3.25892	-1.93546	-1.59384
O94768	Serine/thre	STK17B DR	-0.11313	0.036072	-0.06601	-0.22675	0.156704	-0.02188	0.15994	-0.05111	0.250276	-0.14335
O94776	Metastasis	MTA2 MTA	-0.53373	-0.51694	-0.4074	-0.36567	-0.64121	-0.3643	-0.23718	-0.51181	-0.38892	-0.25276
O94804	Serine/thre	STK10 LOK	0.497016	0.445728	0.766844	0.471923	0.580991	0.52646	0.582891	0.652279	0.87241	0.872859
O94817	Ubiquitin-li	ATG12 APG	1.95224	2.050761	2.166455	1.945673	1.968043	2.130532	1.860449	2.086115	2.257326	2.35604
O94819	Kelch repea	KBTBD11 C	-2.57923	-1.86278	-2.42286	-1.73697	-1.10009	-1.14708	-2.23266	-2.04596	-1.00028	-1.00592
O94826	Mitochond	TOMM70 K	0.099536	0.27275	0.206089	0.225972	0.372969	0.23173	0.204745	0.019814	0.238467	0.239986
O94842	TOX high m	TOX4 C14o	-3.37126	-4.07195	-2.72403	-3.45317	-3.2076	-3.40054	-3.3322	-3.45372	-3.5025	-2.2402
O94874	E3 UFM1-p	UFL1 KIAA	-0.73495	-0.53138	-0.06378	-0.15899	-0.30529	0.203533	-0.35819	-0.26303	-0.4892	-0.30981
O94880	PHD finger	PHF14 KIAA	-0.00866	-0.10426	-0.05311	-0.25544	0.157198	-0.23054	-0.24473	-0.07039	-0.2687	-0.62081
O94886	CSC1-like p	TMEM63A	-2.68536	-2.15634	-3.28345	-4.38606	-3.79256	-3.65208	-2.89162	-3.88982	-2.97199	-3.44184
O94888	UBX domai	UBXN7 KIA	-3.72489	-3.72403	-4.07195	-3.84549	-3.69302	-4.45943	-3.81762	-3.80735	-3.04793	-2.59384
O94901	SUN domai	SUN1 KIAA	0.357848	-0.0843	-0.04354	0.043253	-0.22487	0.100264	0.010196	0	-0.16993	-0.52218
O94903	Pyridoxal p	PLPBP PRO	-1.68536	-1.05565	-0.65486	-1.0906	-0.69302	-1.18641	-1.06274	-0.55943	-0.96277	-0.65524
O94905	Erlin-2 (Enc	ERLIN2 C8o	-0.39552	-0.56471	-0.59725	-0.47192	-0.62263	-0.72869	-0.42286	-0.55736	-0.32274	-0.53013
O94906	Pre-mRNA-	PRPF6 C20o	-0.06193	0.094327	-0.03991	-0.17464	-0.05477	-0.16335	-0.08836	-0.34148	-0.16588	0.008824
O94913	Pre-mRNA	PCF11 KIAA	-0.60942	-0.5427	-0.18709	-0.21613	-0.48097	-0.11958	-0.04679	-0.51785	-0.00472	0.047877
O94915	Protein fur	FRYL AF4P1	-0.5583	-0.30218	-0.25169	-0.5444	-0.55349	-0.28821	-0.58358	-0.76274	-0.67737	-0.71717
O94919	Endonuclea	ENDOD1 KI	-1.406	-1.57864	-1.11163	-1.23939	-1.40027	-1.75215	-1.35225	-1.34954	-1.64088	-1.38299
O94925	Glutaminas	GLS GLS1 K	-0.55354	-0.26997	-0.89392	-0.71327	-0.87521	-0.69374	-0.70611	-0.73035	-0.9484	-1.14544
O94952	F-box only	FBXO21 FB	-2.98793	-3.88753	-2.26459	-3.03814	NA	NA	-3.28157	-2.72935	-2.04793	-2.30433
O94966	Ubiquitin c	USP19 KIAA	0.213707	-0.21397	-0.07195	0.006259	-0.22487	0.090032	-0.35819	-0.06439	-0.02377	0.309483

O94973	AP-2 comp	AP2A2 ADT	0.730891	0.923946	-1.83128	-1.30708	0.800967	0.443446	0.489805	0.312739	-1.25633	-1.05789
O94979	Protein tra	SEC31A KIA	0.477822	0.69984	0.183387	0.480596	0.365687	0.300687	0.407338	0.61106	0.297196	0.168209
O94992	Protein HE	HEXIM1 CL	-0.411	-0.45457	-0.7112	-0.82312	-0.38074	-0.5366	-0.48134	-0.6644	-0.50919	-0.41209
O95071	E3 ubiquiti	UBR5 EDD	0.35311	0.600476	0.27919	0.453718	0.694708	0.525461	0.186878	0.402098	0.63743	0.368948
O95081	Arf-GAP do	AGFG2 HRE	-0.90613	-1.35147	-0.86605	-0.37788	-0.74193	-0.9783	-1.16227	-1.1193	-0.9445	-0.6763
O95104	SR-related	SCAF4 KIAA	-0.57357	-0.26929	-0.24136	-0.07731	-0.22487	-0.41504	-0.15087	-0.18938	-0.09832	0.027044
O95140	Mitofusin-2	MFN2 CPRI	0.659771	0.513013	0.064808	0.283793	0.072512	-0.06711	0.159657	-0.36995	-0.13327	-0.12473
O95155	Ubiquitin c	UBE4B HDN	0.543974	0.733061	0.960472	0.701404	0.84717	0.603064	0.290901	0.592576	0.565979	0.385981
O95163	Elongator c	ELP1 IKAP I	-1.40046	-1.11133	-1.54715	-1.60195	-1.62218	-1.1621	-1.03418	-1.15108	-1.81179	-1.48394
O95168	NADH dehy	NDUFB4	0.866808	0.896256	0.116169	0.028795	0.149361	0.218811	0.371786	-0.02415	-0.26638	0.00301
O95169	NADH dehy	NDUFB8	0.592994	0.437219	-0.52302	-0.56437	-0.64067	-0.70768	-0.09108	-0.27192	-0.94682	-0.78721
O95182	NADH dehy	NDUFA7	0.853375	0.90741	1.170791	0.865316	1.225234	1.087463	1.208279	0.929611	1.628477	1.326258
O95183	Vesicle-ass	VAMP5 HSI	-0.666	-0.68589	-0.97679	-0.32979	-0.55552	-0.43706	-0.62385	-0.42269	-2.67745	-2.41355
O95202	Mitochond	LETM1	0.065184	0.288798	-0.1475	-0.13934	-0.39455	0.029208	0.069507	0.164771	-0.65493	-0.37145
O95218	Zinc finger	ZRANB2 ZIS	-1.26453	-0.85103	-0.90623	-1.3996	-0.75317	-1.06966	-0.90856	-0.79156	-0.87854	-0.73415
O95219	Sorting nex	SNX4	-1.01217	-1.7112	-1.20945	-0.85681	-0.54463	-1.22422	-1.23266	-1.29078	-1.18057	-0.88112
O95232	Luc7-like pr	LUC7L3 CRI	0.419912	0.682378	0.580073	1.320489	0.857467	0.906891	0.769684	0.663902	0.700418	0.678635
O95248	Myotubula	SBF1 MTM	-1.71866	-1.63906	-1.5541	-1.59756	-1.49139	-1.61813	-1.24478	-1.61887	-1.83073	-1.60745
O95249	Golgi SNAP	GOSR1 GS2	2	1.931121	2.256521	1.782044	1.813715	1.905433	1.910297	1.916022	1.943511	1.694299
O95251	Histone ace	KAT7 HBO1	-1.28011	-0.72403	-0.64874	-1.02531	-0.57755	-0.71348	-0.82667	-0.46158	-0.83954	-0.40036
O95260	Arginyl-tRN	ATE1	0.121991	0.256521	0.462972	0.171318	0.524208	0.676705	0.767339	0.985616	0.956931	0.968684
O95292	Vesicle-ass	VAPB UNQ	-1.41908	-1.43335	0.141044	-0.26053	0.022139	0.174774	-0.04152	-0.36257	0.312468	0.365517
O95295	SNARE-asso	SNAPIN BL	-0.69125	-0.89645	-1.12199	-1.0126	-1.03575	-1.27846	-1.03597	-1.00086	-1.31487	-1.18886
O95298	NADH dehy	NDUFC2 HL	1.946491	1.844975	1.1318	1.118792	0.718852	1.311177	1.491528	1.367956	0.516349	-0.00289
O95299	NADH dehy	NDUFA10	-0.13995	0.07944	-0.15592	-0.01563	-0.04532	-0.00411	-0.08114	-0.12553	-0.09078	-0.11083
O95319	CUGBP Elav	CELF2 BRU	1.2726	1.094327	1.346669	1.309328	1.022139	0.84739	1.2158	0.929611	1.308666	1.479408
O95336	6-phospho	PGLS	0.045357	0.530399	0.431025	0.335482	0.291765	0.402268	0.233181	-0.14244	0.505743	0.781745
O95347	Structural r	SMC2 CAPE	0.032437	-0.07351	0.317646	0.407413	0.18085	0.125855	-0.00414	0.024464	0.007411	0.152612
O95363	Phenylalan	FARS2 FARS	0.183066	0.456075	0.169592	-0.15474	0.210655	0.028433	-0.7456	-0.51376	-0.69614	-0.68935
O95365	Zinc finger	ZBTB7A FBI	-1.88086	-1.93147	-0.84488	-0.74746	-0.77973	-0.84472	-1.12832	-1.29078	-0.96277	-0.95641
O95372	Acyl-protei	LYPLA2 APT	0.940692	0.813302	0.771216	0.623009	1.03912	1.018109	0.943972	1.108819	1.163261	1.223664
O95373	Importin-7	IPO7 RANB	0.53981	0.680507	0.068531	-0.10402	0.371631	0.313158	0.31166	-0.04064	-0.09248	0.155098
O95379	Tumor nec	TNFAIP8	1.917609	1.881477	1.762003	1.486233	1.925415	1.800194	1.72321	1.774582	1.975051	1.794866

O95391	Pre-mRNA-	SLU7	-1.42586	-0.62685	-0.34278	0.01292	-0.7571	-0.49591	-0.36436	-0.45705	-0.40237	-0.42932
O95394	Phosphoac	PGM3 AGN	-2.32696	-1.99968	-1.69252	-0.75429	-1.42004	-1.25373	-1.21838	-0.62153	-1.60778	-0.9781
O95396	Adenylyltra	MOCS3 UB	-0.72489	-0.6672	-0.88033	-1.2909	-1.12418	-0.90484	-0.63176	-1.0115	-0.96277	-0.64829
O95400	CD2 antige	CD2BP2 KI/	-1.02445	-0.75002	-0.70483	-0.68562	-0.9852	-0.66935	-0.94973	-0.75811	-0.55695	-0.1738
O95425	Supervillin	SVIL	-1.31346	-1.2167	-1.11353	-1.10402	-0.83173	-0.9783	-1.41163	-1.29078	-1.06756	-1.04495
O95429	BAG family	BAG4 SODI	-3.25096	-1.87317	-1.7632	-1.69574	-1.14048	-1.37197	-1.53964	-1.46949	-1.1913	-1.37145
O95433	Activator o	AHSA1 C14	-1.1514	-1.33171	-0.85543	-1.0126	-0.99809	-0.97847	-1.17863	-1.31245	-1.18045	-0.87692
O95453	Poly(A)-spe	PARN DAN	-0.43539	-0.39696	-0.06378	-0.61667	-1.24236	-0.82523	-0.53964	-0.79737	-0.49584	-0.89752
O95456	Proteasom	PSMG1 C21	-0.35566	-0.44923	-1.28345	-0.76867	-0.79256	-0.77761	-0.59266	-0.90046	-0.85651	-0.68339
O95466	Formin-like	FMNL1 C17	0.226434	0.233023	0.591432	0.179379	0.253313	0.288926	0.296786	0.236836	0.101896	0.077511
O95470	Sphingosin	SGPL1 KIA/	-1.12234	-1.58596	-1.69517	-1.9386	-1.05156	-2.06548	-2.26211	-1.83472	-1.71316	-1.12232
O95479	GDH/6PGL	H6PD GDH	0.018072	-0.12199	-0.25526	0.231326	-0.44004	-0.51301	-0.4885	-0.39232	-0.3505	-0.45391
O95486	Protein tra	SEC24A	-3.19438	-1.93147	-1.33171	-2.06413	-1.84503	-1.85457	-2.11718	-2.848	-2.02857	-1.85688
O95487	Protein tra	SEC24B	0.661789	0.59786	0.905416	0.87323	0.71507	0.761073	0.678036	0.567897	0.468602	0.530442
O95551	Tyrosyl-DN	TDP2 EAP2	0.51154	0.620266	0.231295	0.381097	0.425989	0.61516	0.570782	0.555975	0.612338	0.171358
O95571	Persulfide (	ETHE1 HSC	0.716207	0.279437	0.302694	0.431864	0.18085	0.778973	0.431751	0.355555	0.35135	0.230695
O95573	Long-chain	ACSL3 ACS3	-0.09392	0.151613	0.116077	0.171318	0.029444	0.048363	-0.01027	-0.08246	-0.25176	-0.52218
O95602	DNA-direct	POLR1A	-0.57289	-0.64874	-0.83094	-0.54171	0.086588	-0.05571	-0.7997	-0.79737	-0.71823	-0.76377
O95613	Pericentrin	PCNT KIAA(	-0.94779	-0.82138	-0.46517	-0.75816	-0.6674	-0.56754	-1.15603	-1.12592	-0.72237	-0.76066
O95625	Zinc finger	ZBTB11	-1.47766	-1.76435	-1.67745	-1.29227	-1.08177	-1.04918	-1.16329	-1.36457	-1.61226	-1.51787
O95630	STAM-bind	STAMBP AI	-0.10692	-0.16506	0.354315	0.278631	0.154975	0.043069	0.141219	0.280108	0.023383	0.276981
O95639	Cleavage ai	CPSF4 CPSI	-0.19438	0.101614	0.087004	0.231326	0.043943	-0.05571	-0.06274	-0.4459	-0.24616	-0.41799
O95644	Nuclear fac	NFATC1 NF	0.523035	0.479846	0.416986	0.278631	0.154975	0.115477	-0.12274	-0.10692	0.360998	0.984427
O95671	Probable b	ASMTL	-0.41194	-0.35299	0.085503	0.185828	0.05706	-0.08912	-0.45124	-0.19934	-0.1318	-0.27834
O95674	Phosphatid	CDS2	0.095442	0.214122	-0.13457	0.208037	-0.4724	-0.35374	-0.19604	-0.60368	-0.95286	-0.65217
O95684	Centrosom	CEP43 FGFI	-1.31268	-1.50399	-1.04194	-1.35186	-0.83422	-1.95693	-0.99451	-0.95803	-1.27838	-1.38013
O95696	Bromodom	BRD1 BRL E	0.338802	0.304669	0.291986	0.288936	0.449936	0.299902	0.060121	0.217477	-0.04793	-0.06796
O95721	Synaptosor	SNAP29	-0.46021	-0.2853	-0.16923	0.000654	-0.36312	-0.62617	-0.23855	-0.49751	-0.5229	-0.4819
O95747	Serine/thre	OXSR1 KIA/	1.052331	1.302535	0.123066	0.298738	0.156088	0.49016	0.523206	0.240067	0.064752	-0.25603
O95749	Geranylger	GGPS1	-3.43539	-3.24598	-3.88753	-3.59756	-3.27798	-3.87447	-4.05209	-3.80735	-3.86507	-3.21945
O95758	Polypyrimi	PTBP3 ROD	0.46062	0.407158	0.496073	0.226585	0.013054	0.230075	0.434382	0.166135	0.07075	0.100658
O95777	U6 snRNA-a	LSM8	-1.52852	-1.63677	-2.02359	-1.8011	-2.89947	-2.2115	-2.13955	-1.34792	-1.82086	-2.26738
O95782	AP-2 comp	AP2A1 ADT	0.776875	0.792095	0.329222	0.225663	0.300528	0.517416	0.729389	0.588839	0.468741	0.455722

O95785	Protein Wi:	WIZ ZNF80	-0.54609	-0.60657	0.196539	0.15451	0.708426	0.282035	0.209177	0.094182	0.640458	0.87044
O95786	Antiviral in	DDX58	-1.40028	-1.63612	-1.08348	-1.1805	-1.67092	-1.48518	-0.93203	-1.35095	-1.19349	-1.30201
O95793	Double-stra	STAU1 STA	-0.72489	-0.82402	-0.44923	-0.5787	-0.39046	-0.61813	-0.5175	-0.27684	-0.50919	-0.56083
O95816	BAG family	BAG2	-0.77748	-0.97311	-0.67963	-0.5737	-0.54893	-0.27947	-0.61635	-0.82682	-0.18057	-0.44039
O95817	BAG family	BAG3 BIS	-1.62803	-1.00782	-0.92405	-1	-0.69613	-0.85531	-1.06835	-1.16454	-0.25302	0.039128
O95819	Mitogen-ac	MAP4K4 H(	-1.23661	-1.78991	-1.53138	-1.8011	-2.34899	-2.50599	-3.28243	-2.78327	-2.55802	-2.6257
O95825	Quinone ox	CRYZL1 4P1	-0.11498	-0.10333	-0.16697	-0.02956	0.183456	-0.10088	-0.1673	-0.05004	-0.11001	0.04015
O95831	Apoptosis-i	AIFM1 AIF	0.035907	-0.23972	0.189165	0.138219	0.215154	0.223908	0.178144	0.063004	0.162982	-0.25234
O95833	Chloride in	CLIC3	-0.06193	0.451612	0.169058	-0.11757	-0.5231	-0.15565	0.108376	0.120752	-0.7028	-0.50952
O95834	Echinoderm	EML2 EMA	0.203214	0.158617	-0.12199	-0.05107	-0.48097	-0.1315	-0.49942	-0.3374	-0.3445	-0.07727
O95861	3'(2'),5'-bis	BPNT1	0.877053	1.413768	1.287694	1.219232	1.518255	1.417415	1.2538	1.302364	1.616807	1.623718
O95865	N(G),N(G)-(	DDAH2 DD.	-2.02051	-1.71403	-1.87728	-1.8855	-2.81879	-2.0887	-1.6777	-2.59567	-2.44408	-2.21915
O95870	Phosphatid	ABHD16A E	-1.06193	-0.60657	-1.1305	-1.56009	-1.27798	-1.1989	-1.06274	-1.10692	-1.36257	-0.85688
O95881	Thioredoxi	TXNDC12 T	0.612045	0.415074	1.000741	0.854841	0.586316	0.609958	0.667595	0.812212	0.206137	0.50614
O95926	Pre-mRNA-	SYF2 CBPIN	-0.28011	-0.42286	0.144576	-0.41086	0.072512	0.217701	-0.12832	-0.13179	0.046393	-0.01781
O95989	Diphosphoi	NUDT3 DIP	-0.36409	-0.1261	-1.01879	-0.74244	-0.79798	-0.29231	-0.50601	-0.73292	-0.83071	-0.73113
O95999	B-cell lymp	BCL10 CIPE	-0.88398	-0.04219	0.149548	0.140708	0.222579	0.233028	-0.30069	0.132862	0.217981	0.257871
O96000	NADH dehy	NDUFB10	1.523035	1.510278	0.424236	0.453718	0.248231	1.771066	1.178232	1.043713	-0.05678	-0.25649
O96006	E3 SUMO-p	ZBED1 ALTI	-1.15335	-1.25526	-0.68589	-0.18015	-0.71727	-0.81558	-0.53222	-1.03477	-1.02857	-0.78649
O96019	Actin-like p	ACTL6A BA	-0.34152	-0.14747	0.235032	0.37389	-0.17177	-0.5064	-0.09987	-0.40663	-0.46826	-0.20839
P00338	L-lactate de	LDHA PIG1!	1.024045	0.970981	0.998049	0.861869	0.662182	0.844349	1.025356	0.896164	0.934905	1.147127
P00367	Glutamate	GLUD1 GLU	0.723568	0.859523	0.725958	0.885829	0.636052	0.883384	1.082537	1.088809	0.597481	0.412823
P00387	NADH-cyto	CYB5R3 DIA	-0.28749	-0.11775	0.744349	0.411898	0.270452	0.723099	0.387926	0.629865	0.145717	-0.28803
P00390	Glutathion	GSR GLUR (	0.560509	0.808469	0.565049	0.803465	0.796692	0.841473	0.78229	0.893085	0.538005	0.376811
P00395	Cytochrom	MT-CO1 CC	-2.98793	-2.34156	-2.5541	-1.3377	-2.35199	-1.91511	-3.05209	-1.14439	-3.16993	-1.8893
P00403	Cytochrom	MT-CO2 CC	3.017942	2.771871	0.843825	1.18897	0.545655	1.231205	1.962003	1.33926	0.206165	-0.06164
P00451	Coagulatio	F8 F8C	-0.666	-0.82402	-0.62449	-0.89129	-1.10806	-0.84472	-0.94973	-0.49346	-0.61353	-0.44786
P00491	Purine nucl	PNP NP	0.079116	0.533398	-0.1939	0.370027	-0.10584	0.700773	0.270639	-0.05737	-0.66797	-0.66557
P00492	Hypoxanth	HPRT1 HPR	1.211091	1.167323	0.984318	1.193539	0.971103	0.782154	1.235488	1.220313	1.007055	1.305711
P00505	Aspartate a	GOT2	0.637069	0.535681	0.585028	1.241144	0.243337	0.481492	0.975858	0.552054	0.667461	0.598823
P00558	Phosphogly	PGK1 PGKA	0.734539	0.765272	0.900945	0.666263	0.988802	0.895185	0.743092	0.850304	1.227762	1.230272
P00747	Plasminoge	PLG	-2.89411	-2.05702	-0.47321	-0.84708	-1.66821	-1.16103	-0.98148	-1.24949	-1.24898	-1.16762
P00813	Adenosine	ADA ADA1	0.144101	0.437285	-0.27872	-0.30633	-1.04532	-0.66935	-0.39808	-0.33342	0.390071	-0.50324

P01009	Alpha-1-an	SERPINA1 /	-0.47697	-0.09259	-0.23676	-0.23816	-0.85845	-0.74977	-0.15087	-0.13179	-1.20211	-1.71937
P01111	GTPase NR	NRAS HRAS	0.13317	0.273266	0.036454	0.101609	0.10839	0.275793	0.070791	0.046281	-0.08302	-0.0854
P01116	GTPase KR	KRAS KRAS	0.448371	0.816331	0.679421	0.55429	0.466186	0.769344	0.818388	0.809662	0.253742	0.337755
P01591	Immunoglo	JCHAIN IGC	-0.55497	-0.40726	-1.75002	-1.86821	-2.74193	-1.44448	-3.18536	-2.04654	-1.10764	-1.47836
P01730	T-cell surfa	CD4	0.186152	0.435568	0.026156	0.024374	0.047433	0.154268	0.215825	-0.08267	0.398983	0.207553
P01732	T-cell surfa	CD8A MAL	1.735046	1.874191	1.311397	1.594191	1.001847	1.298218	2.173299	1.949043	0.753482	0.656632
P01834	Immunoglo	IGKC	0.149083	0.166755	-1.09659	-1.08544	-0.73783	-0.7964	-1.17737	-0.94839	-1.0531	-1.40028
P01848	T cell recep	TRAC TCRA	1.766495	1.965945	1.007355	1.202394	1.400291	1.097562	1.442627	1.39063	1.675598	1.657445
P01857	Immunoglo	IGHG1	-1.24772	-0.8422	-1.05009	-1.83778	-2.09851	-1.73337	-0.93505	-0.71758	-0.47179	-0.71623
P01876	Immunoglo	IGHA1	-0.51111	-0.90933	-2.26459	-1.71621	-2.59991	-2.0902	-2.89162	-3.45372	-2.04793	-1.69052
P01892	HLA class I	HLA-A HLA	-2.15949	-2.27773	-1.57864	-1.21151	-2.0957	-2.2571	0.113641	0.399407	-1.78668	-1.75645
P02545	Prelamin-A	LMNA LMN	0.578585	0.528991	0.608571	0.818658	0.336725	0.342811	0.792602	0.712848	-0.40601	-0.23868
P02656	Apolipopro	APOC3	0.561434	0.611988	1.554573	1.527016	1.328993	1.417355	0.902019	1.210342	1.500234	1.443063
P02671	Fibrinogen	FGA	-1.80735	-2.48699	-2.50901	-1.9386	-2.64572	-1.91511	-1.782	-1.74846	-2.08746	-1.63448
P02675	Fibrinogen	FGB	-0.63531	-0.58012	-0.80149	-0.98291	-0.63445	-0.45974	-0.93036	-0.49491	-0.89109	-0.87575
P02679	Fibrinogen	FGG PRO20	0.925361	0.338983	0.019365	-0.37788	0.18799	1.383966	1.393778	1.104869	-1.57089	-1.60726
P02686	Myelin bas	MBP	-1.26546	-0.91667	-1.32127	-0.97534	-1.88469	-1.24595	-1.36268	-1.25866	-1.37082	-0.7121
P02749	Beta-2-glyc	APOH B2G1	0.80215	0.749389	1.52661	1.691728	1.339666	1.227069	1.340229	1.149425	0.609891	0.178749
P02751	Fibronectir	FN1 FN	-0.98793	-1.37151	-1.42627	-1.22671	-0.91341	-0.56058	-0.35165	-0.69188	-0.98126	-0.96502
P02771	Alpha-feto	AFP HPAFP	-1.03684	-1.66102	-0.86605	-1.30633	-0.83173	-0.74977	-0.75585	-0.61096	-0.98126	-0.97369
P02774	Vitamin D-l	GC	-1.04818	-1.34169	-0.86071	-1.51594	-1.49215	-0.95231	-0.87618	-1.6933	-1.74603	-1.32858
P02775	Platelet ba	PPBP CTAP	-0.07707	-0.16837	0.355269	0.383529	0.424394	0.188835	0.642482	0.676227	0.834372	0.829571
P02794	Ferritin hea	FTH1 FTH F	-0.53859	-0.10033	-0.18691	0.110443	-0.29089	-0.51055	-0.45757	-0.13114	-0.75471	-0.08273
P03928	ATP syntha	MT-ATP8 A	1.912537	1.87376	1.756331	1.695607	1.441746	1.631563	1.680628	1.655715	1.025701	1.196236
P03989	HLA class I	HLA-B HLAI	-0.69792	-0.6636	-0.53837	0.068454	-1.55552	-1.45943	-3.09516	-3.16993	-1.76553	-1.99118
P04075	Fructose-bi	ALDOA ALD	0.260155	0.517886	0.599813	0.220234	0.603034	0.456551	0.514725	0.502478	0.743979	0.668357
P04080	Cystatin-B	CSTB CST6	0.000263	0.179362	-0.04895	0.046271	-0.09614	0.153746	0.221911	0.167903	0.378285	0.443049
P04083	Annexin A1	ANXA1 ANX	0.836521	1.089004	1.359039	1.16055	0.599822	0.847093	0.491355	0.846031	0.401879	0.128588
P04150	Glucocortic	NR3C1 GRL	-0.56616	-0.61357	-0.72702	-0.67313	-1.30884	-0.86424	-0.61276	-1.45645	-1.72149	-0.97931
P04156	Major prior	PRNP ALTP	-3.13993	-3.53138	-2.12199	-2.75803	-4.27798	-2.83494	-3.13955	-3.97728	-3.21299	-1.87299
P04179	Superoxide	SOD2	-1.36144	-0.36216	-2.73702	-2.5798	-2.44234	-1.50757	-1.30866	-1.37515	-1.37412	-1.50822
P04181	Ornithine a	OAT	-0.30236	-0.23217	-0.65486	-1.40255	-0.59991	-1.14958	-0.68012	-0.922	-0.83954	-0.61402
P04234	T-cell surfa	CD3D T3D	0.088176	0.216811	-1.32166	-1.3116	-0.49139	-0.90484	-0.63971	-0.66434	-0.24058	-0.79414

P04350	Tubulin bet	TUBB4A TU	0.090392	0.103584	1.533885	1.259953	1.357467	1.801476	1.564813	1.944883	1.49565	1.175646
P04406	Glyceraldel	GAPDH GA	0.25853	0.564776	0.727676	0.626803	0.958134	0.785579	0.760979	0.691605	1.00347	1.038424
P04424	Argininosu	ASL	-3.19438	-1.48699	-2.44392	-2.8011	-2.51245	-2.75899	-2.13955	-1.61973	-2.02857	-1.79414
P04439	HLA class I	HLA-A HLA	0.846649	0.52661	-1.37112	-1.18884	-4.59991	-3.72247	-3.81314	-4.17008	-0.06365	-0.15998
P04632	Calpain sm	CAPNS1 CA	0.33579	0.405256	0.067552	0.023166	-0.13226	0.074168	0.049337	0.060481	-0.23047	-0.35221
P04733	Metallothic	MT1F PRO	-3.98793	-4.13906	NA	NA	-2.74193	-3.52083	NA	NA	-2.89984	-3.39453
P04792	Heat shock	HSPB1 HSP	-0.32496	0.030861	1.409666	1.263034	1.388772	1.546193	0.336182	0.440573	0.902483	0.728087
P04839	Cytochrom	CYBB NOX2	-0.04307	-0.02359	-0.52576	-0.66558	-0.06075	-0.37906	0.060121	0.415037	-0.38087	-0.12956
P04843	Dolichyl-di	RPN1	-0.16369	-0.28113	0.24753	-0.14998	0.023457	0.210076	-0.15091	0.056143	-0.46447	-0.42414
P04844	Dolichyl-di	RPN2	0.628744	0.80624	-0.20945	0.160134	-0.39046	0	0.098853	-0.42269	-0.91754	-0.06796
P04899	Guanine nu	GNAI2 GNA	0.006049	0.072245	-0.15906	-0.05107	-0.42999	-0.1375	-0.35712	-0.05247	-0.2916	-0.38294
P04921	Glycophori	GYPC GLPC	-0.88359	-1.24598	-3.83094	-3.20163	-3.2076	-3.34395	-2.03103	-3.07039	-3.30319	-3.0268
P05023	Sodium/po	ATP1A1	0.336128	0.068531	-0.24589	-0.17986	-0.39097	-0.11892	-0.01518	-0.04359	-0.2687	-0.63448
P05091	Aldehyde d	ALDH2 ALD	1.110807	1.256521	1.032768	1.193429	0.638492	0.471306	0.752232	0.777608	0.218346	0.166972
P05106	Integrin be	ITGB3 GP3	0.093116	0.13186	0.325626	0.032944	-0.26819	-0.1575	0.478582	0.00471	-0.97998	-0.84983
P05107	Integrin be	ITGB2 CD1	0.399803	0.317241	0.23349	0.006259	-0.1161	0.174774	0.069902	0.172467	-0.25739	-0.23499
P05112	Interleukin	IL4	-3.37126	-2.44392	-1.91667	-1.59756	-3.2076	-2.95693	-3.81762	-3.16993	-2.14886	-2.41799
P05114	Non-histon	HMGN1 HN	0.796899	0.878009	0.776348	0.525197	0.865821	0.72379	0.738746	0.688056	0.843274	0.704829
P05141	ADP/ATP tr	SLC25A5 AI	1.462112	1.483495	0.285173	0.044639	-0.20845	0.249696	0.715318	0.201101	-0.68803	-0.85321
P05161	Ubiquitin-li	ISG15 G1P2	-0.63195	-0.4269	-0.40329	-0.14733	-1.16586	-0.94957	-0.43808	-0.66259	0.065175	0.07668
P05165	Propionyl-(	PCCA	-0.00473	-0.03019	-0.00721	-0.08518	0.269412	-0.24998	-0.05436	-0.34066	-0.09581	0.346726
P05166	Propionyl-(	PCCB	-0.17617	0.187381	-0.62449	-0.34573	-0.44004	0.169115	-0.04217	0.031603	-0.19709	-0.53495
P05198	Eukaryotic	EIF2S1 EIF2	0.100367	0.409689	-0.24638	-0.33491	-0.05622	-0.30484	-0.05765	-0.07965	-0.05666	-0.19955
P05204	Non-histon	HMGN2 HN	0.703295	0.911562	0.584002	1.000654	0.908437	0.60853	0.996891	0.815344	0.396306	0.607138
P05362	Intercellula	ICAM1	-1.1669	-0.4761	-1.51769	-1.97623	-2.42776	-2.23383	-2.03727	-2.02009	-2.91589	-2.53931
P05387	60S acidic r	RPLP2 D11	0.916098	1.046981	0.289052	0.523145	0.468242	0.508357	0.634514	0.500524	0.463744	0.502737
P05388	60S acidic r	RPLP0	1.016199	0.978917	0.88013	0.805562	0.84717	0.520036	0.93459	0.740396	1.071287	1.269657
P05412	Transcripti	JUN	-2.48633	-1.75553	-1.81727	-2.40112	-2.08183	-1.19304	-1.23236	-1.23261	-1.88245	-1.30632
P05455	Lupus La pr	SSB	0.48333	0.60817	0.490986	0.374945	0.299108	0.486987	0.290901	0.490326	0.649503	0.384884
P05534	HLA class I	HLA-A HLA	-1.20832	-0.51457	0.105243	-0.30633	-3.2076	-1.28951	-1.89162	-1.63743	-3.04793	-1.74882
P05556	Integrin be	ITGB1 FNRI	-0.818	-0.92331	-0.43862	-0.74746	-0.8055	-0.85457	-1.2012	-1.24808	-0.97199	-1.42986
P05771	Protein kin	PRKCB PKC	1.179048	1.314109	1.153078	0.99872	0.871762	0.924273	1.117836	0.982722	0.745427	0.880091
P06127	T-cell surfa	CD5 LEU1	-0.61869	-0.88753	-0.04131	-0.51644	-0.17365	-0.23704	-0.39808	-0.59358	-0.14294	-0.18886

P06239	Tyrosine-pı	LCK	0.003108	-0.07986	-0.2748	-0.17456	0.021507	0.084261	0.013843	-0.18638	-0.35078	-0.29492
P06241	Tyrosine-pı	FYN	-1.04933	-0.92405	-0.55984	-0.56937	-1.37109	-1.1989	-0.93988	-1.29078	-1.38702	-1.56737
P06400	Retinoblast	RB1	-2.0646	-1.4018	-1.46726	-1.80708	-1.85349	-1.13391	-2.09317	-2.34859	-2.46514	-1.8489
P06454	Prothymos	PTMA TMS	-0.02577	-0.10512	0.040043	0.312236	0.161638	-0.28284	0.108376	-0.07039	-0.05282	0.201871
P06576	ATP syntha	ATP5F1B A	0.22955	0.253294	0.464667	0.255193	0.229392	0.370571	0.410589	0.459977	0.608072	0.625341
P06702	Protein S10	S100A9 CA	2.833103	2.997073	-1.1305	-1.47045	-1.71727	-0.89465	0.164229	0.141661	-1.3505	-1.09134
P06703	Protein S10	S100A6 CA	2.620731	2.74618	0.321919	0.857862	0.834142	1.330284	1.216673	1.008483	0.36108	0.503554
P06729	T-cell surfa	CD2 SRBC	1.118352	1.222909	1.213452	1.27117	1.232408	1.145291	0.994146	0.855256	0.868843	0.775456
P06730	Eukaryotic	EIF4E EIF4E	-0.38962	0.044048	1.461557	1.363963	-0.52124	-0.56503	-0.10491	-0.24889	-0.19851	-0.17525
P06733	Alpha-enol	ENO1 ENO1	0.233236	0.493758	0.548756	0.426985	0.547819	0.631352	0.54909	0.657439	1.002355	0.901345
P06744	Glucose-6-p	GPI	0.906082	1.000924	0.337974	0.49436	0.557379	0.906891	0.765355	0.778122	0.636706	0.461843
P06746	DNA polym	POLB	-0.83954	-0.63656	-0.65486	-0.97513	-1.14048	-0.28951	-0.7217	-0.42269	-0.4892	-0.7414
P06748	Nucleopho	NPM1 NPM	-0.57334	-0.45201	0.313618	0.333921	0.488914	0.375011	0.372423	0.549227	0.563599	0.514408
P06753	Tropomyos	TPM3	0.565698	0.549384	0.765535	0.483413	0.528919	0.490102	0.499986	0.42696	0.665725	0.751817
P06865	Beta-hexos	HEXA	0.566416	0.726838	-1.08865	-0.96357	-0.54725	-1.00599	-0.48995	-0.85995	-1.26625	-0.39486
P07108	Acyl-CoA-b	DBI	1.84632	2.278822	0.659627	1.263531	0.026789	0.478187	1.042943	1.08292	0.160409	-0.04945
P07195	L-lactate de	LDHB	1.335198	1.046047	1.287201	1.151292	1.445505	1.333993	1.328868	1.16383	1.762203	1.81869
P07196	Neurofilam	NEFL NF68	-1.15335	-1.19153	-0.83789	-1.52356	0.927564	0.447459	-1.45305	-1.97728	-0.75754	-0.68339
P07199	Major cent	CENPB	-0.12003	-0.20945	-0.94642	-0.9386	-0.33313	-0.41504	-0.54709	-0.69188	-1.13845	-0.12956
P07203	Glutathion	GPX1	0.445032	0.557795	-0.38163	0.055377	0.377367	0.529253	0.248466	0.321928	0.407948	0.122947
P07237	Protein dis	P4HB ERBA	0.259557	0.539087	0.889091	1.130291	0.593625	0.688526	0.631298	0.630141	0.243201	0.286073
P07305	Histone H1	H1-0 H1F0	0.460269	1.044158	0.116077	-0.04459	1.273531	1.19162	1.824422	1.799153	1.462001	1.726751
P07355	Annexin A2	ANXA2 ANX	0.36963	0.479846	0.842384	0.842802	0.572755	0.330645	0.546949	0.473931	-0.61353	-0.56083
P07384	Calpain-1 c	CAPN1 CAN	0.490082	0.457495	0.084517	0.420203	0.13846	0.591964	0.394422	0.402887	0.124795	-0.14456
P07437	Tubulin bet	TUBB TUBE	-0.666	-0.45992	0.425721	0.407175	0.31895	0.242193	0.380085	0.644772	0.43644	0.198166
P07602	Prosaposin	PSAP GLBA	0.870054	0.847997	0.907018	0.840458	0.634487	0.807355	0.702799	0.749633	0.588239	0.512195
P07686	Beta-hexos	HEXB HCC7	-0.16011	-0.18265	0.247739	0.425973	0.138001	0.079727	0.529334	0.519842	0.042477	-0.11276
P07737	Profilin-1 (I	PFN1	0.441699	0.504792	0.685364	0.453362	0.22279	0.41913	0.273574	0.473908	0.604383	0.473763
P07738	Bisphosphc	BPGM	-3.64689	-3.57719	-2.23143	-2.14933	-1.91593	-1.24767	-2.07346	-2.07039	-3.04793	-2.11991
P07741	Adenine ph	APRT	1.467851	1.788291	0.866236	1.33416	1.22936	1.59095	1.561697	1.38711	1.05042	0.694298
P07766	T-cell surfa	CD3E T3E	0.070967	0.023207	-0.23217	-0.02531	0.221113	0	-0.05209	-0.16993	-0.08246	-0.00888
P07814	Bifunctiona	EPRS1 EPRS	0.115666	-0.06786	0.285602	0.085211	0.133571	0.290996	0.212198	0.167378	0.429954	0.384226
P07858	Cathepsin I	CTSB CPSB	0.041363	0.410629	0.446478	0.325382	0.813821	0.48189	-0.10554	-0.09319	0.353962	0.15887

P07900	Heat shock	HSP90AA1	-0.22101	-0.21919	0.498431	0.287442	0.550008	0.345783	0.601439	0.482801	0.578493	0.660049
P07902	Galactose-1	GALT	-0.25932	-0.25516	0.398767	0.366013	0.288715	0.242699	0.074118	-0.04122	0.088745	0.182211
P07910	Heterogene	HNRNPC HI	0.395201	0.46014	0.345135	0.079322	0.474107	0.430195	0.387926	0.256219	0.360998	0.228701
P07919	Cytochrom	UQCRH	-0.71204	-1.12727	-0.92219	-1.13987	-1.00015	-0.852	-0.2308	-0.27147	-1.19283	-0.68006
P07948	Tyrosine-pr	LYN JTK8	0.018072	0.09067	-0.08844	-0.15899	0.594844	0.053638	0.715317	0.028345	-0.37474	-0.40036
P07951	Tropomyos	TPM2 TMS	0.095609	-0.00314	0.405677	-0.4598	0.289626	0.002521	0.141033	0.237881	0.014823	0.015803
P07954	Fumarate h	FH	-0.15412	0.378063	-0.03106	0.137504	-0.28703	0.130532	-0.15656	-0.07641	0.193823	0.251185
P07996	Thrombosp	THBS1 TSP	-0.68536	-0.30738	0.675633	0.794755	0.822152	0.656046	1.02032	0.760812	0.379237	0.419456
P08047	Transcripti	SP1 TSFP1	-1.58014	-1.47524	-0.83234	-1.0012	-0.76836	-0.5819	-0.58811	-0.48334	-1.4119	-0.71937
P08069	Insulin-like	IGF1R	-0.22239	-0.16069	0.030861	0.363963	0.513829	0.41913	0.411195	0.5025	0.546743	0.406159
P08133	Annexin A6	ANXA6 ANX	1.006049	1.009294	0.641044	0.943379	0.810463	0.686601	0.820032	0.685102	0.675135	0.714604
P08134	Rho-relatec	RHOC ARHS	1.348356	1.569281	1.733061	1.662305	1.673883	1.534922	1.821898	1.733527	1.014075	0.920399
P08174	Compleme	CD55 CR DA	-1.26546	-1.19153	-2.15634	-1.82312	-1.29613	-1.28951	-1.20881	-1.29078	-1.59918	-1.11991
P08195	4F2 cell-sur	SLC3A2 MD	-0.60942	-0.28345	-0.30738	-0.32193	-0.6574	-0.18021	-0.13393	-0.48543	-0.25739	-0.62081
P08238	Heat shock	HSP90AB1	-0.08062	-0.19329	-0.1537	-0.2011	-0.03307	-0.02715	0.142141	0.001683	0.08245	0.038895
P08240	Signal reco	SRPRA SRP	-0.90613	-0.17276	-0.33201	-0.74715	-0.34441	-0.07922	-0.98752	-0.39412	-1.19299	-0.3868
P08514	Integrin alp	ITGA2B GP2	-1.02146	-1.00131	-0.89953	-1.10807	-1.85174	-1.16362	-0.68686	-0.9701	-1.72224	-1.44894
P08559	Pyruvate de	PDHA1 PHE	0.141922	0.307127	-0.15634	0.091148	0.16378	-0.07229	0.34396	0.003807	0.082007	0
P08567	Pleckstrin (	PLEK P47	-0.89482	-0.82402	-0.73048	-1.41923	-2.79256	-3.04439	-1.51019	-1.56789	-3.3505	-2.44184
P08574	Cytochrom	CYC1	1.462734	1.46014	0.648528	0.743225	-0.269	0.369234	0.937264	0.432111	-0.18057	-0.14906
P08575	Receptor-ty	PTPRC CD4	0.759818	0.87376	0.381508	0.339385	0.537185	0.488714	0.649974	0.393407	0.226428	0.190431
P08579	U2 small nu	SNRPB2	0.193795	0.416465	0.336717	0.598644	0.268077	0.619926	0.285701	0.485474	0.686984	0.669254
P08621	U1 small nu	SNRNP70 R	0.381177	0.624608	0.600079	0.36954	0.526401	0.49589	0.448409	0.484198	0.587935	0.860717
P08648	Integrin alp	ITGA5 FNRA	-0.47837	-0.17418	0.335507	0.026349	-0.13003	-0.65302	-0.18237	-0.20069	-0.49094	-0.34543
P08670	Vimentin	VIM	0.180056	0.2824	0.213452	0.319417	-0.00481	0.015476	-0.16227	-0.0378	0.113033	0.134666
P08708	40S ribosor	RPS17 RPS1	-0.1669	0.445898	1.741248	0.563901	0.84717	0.360747	1.250618	0.056143	2.167828	2.157083
P08754	Guanine nu	GNAI3	-0.64689	-0.52576	-0.64874	-0.72655	-1.47063	-1.12553	-1.05209	-1.39232	-1.52947	-1.21945
P08865	40S ribosor	RPSA LAMB	-0.02181	0.070699	-0.0986	0.008159	0.040381	0.010956	-0.03952	0.05293	0.342195	0.551067
P09012	U1 small nu	SNRPA	0.390584	0.597902	0.440162	0.309328	1	0.533035	0.089267	0.557995	1.071287	1.58053
P09104	Gamma-en	ENO2	-0.09738	-0.17237	0.082083	0.151826	0.237869	0.237467	0.235449	0.42339	0.778221	0.741894
P09110	3-ketoacyl-	ACAA1 ACA	0.678829	0.763239	1.0925	1.037153	1.22981	0.886343	1.110747	0.790904	1.475627	1.409495
P09132	Signal reco	SRP19	-0.55486	-0.72519	0.020095	0.254951	0.309143	-0.03843	0.049596	-0.19774	-0.2664	0.055228
P09234	U1 small nu	SNRPC	-0.10697	-0.23676	-0.2811	-0.04182	-0.44142	-0.18081	-0.236	-0.43864	-0.03436	0.245943

P09326	CD48 antig	CD48 BCM1	0.662745	0.695963	0.53082	0.367628	0.706269	0.312775	0.474176	0.515993	0.437079	0.595044
P09382	Galectin-1	LGALS1	1.570154	1.791174	1.661839	1.686502	1.199012	0.949349	1.57347	1.576475	0.567896	0.725969
P09429	High mobil	HMGB1 HN	1.178228	1.200712	1.216811	1.16853	1.054723	1.090032	0.826233	1.219937	1.377073	1.198166
P09467	Fructose-1,	FBP1 FBP	-0.53509	-0.12366	-2.9979	-1.41459	-1.08514	-0.39493	-0.21316	-0.55328	0.472874	0.213614
P09496	Clathrin lig	CLTA	0.64796	0.690782	0.257576	0.192918	0.474564	0.064623	0.287402	0.156406	0.341835	0.20688
P09497	Clathrin lig	CLTB	0.130255	0.206026	0.781501	0.516454	0.194503	0.014995	0.177861	-0.04819	0.514102	-0.00443
P09525	Annexin A4	ANXA4 AN)	0.239609	0.310969	-0.2185	-0.152	-0.61123	0.130532	0.387926	-0.37737	-1.07748	-1.14906
P09543	2',3'-cyclic-	CNP	0.498011	0.335815	0.417216	0.525006	0.498628	0.529149	0.429805	0.345662	0.454277	0.380323
P09564	T-cell antig	CD7	-1.78629	-2.2276	-2.20945	-1.82312	-3.07635	-1.45943	-1.46713	-1.63743	-1.46297	-1.55431
P09622	Dihydrolipo	DLD GCSL L	0.781139	0.483276	0.730713	0.589138	0.699295	0.625931	0.779312	0.923587	0.840644	0.513302
P09651	Heterogen	HNRNPA1 I	-0.01863	0.042266	0.227365	0.001008	-0.04166	-0.19265	0.049833	-0.04492	0.262382	0.265601
P09661	U2 small nu	SNRPA1	0.018072	-0.18265	0.01165	0.049328	0.154975	-0.27621	-0.1452	0.136462	0.736966	0.749304
P09669	Cytochrom	COX6C	2.530398	1.930098	1.633525	1.105795	0.907882	1.938599	2.078406	2.039528	0.925007	0.385981
P09693	T-cell surfa	CD3G T3G	-1.3769	-0.91667	-1.10113	-1.65553	-1.60062	-0.98622	-1.16569	-1.59526	-0.97377	-0.74461
P09874	Poly [ADP-r	PARP1 ADF	1.047697	1.231615	0.959731	1.012491	1.026556	0.868495	0.99744	0.973816	0.91003	0.665292
P09913	Interferon-	IFIT2 CIG-4	-0.6002	-1.08017	-1.19153	-1.35364	-0.92749	-1.14958	-1.06274	-1.1193	-0.24616	-0.43584
P09914	Interferon-	IFIT1 G10P	0.121991	0.269518	-1.20717	-1.76335	-0.12418	-0.33015	-1.78286	-1.96783	-1.64901	-1.33668
P09960	Leukotrien	LTA4H LTA	0.431612	0.399356	-0.0041	0.006259	0.251836	0.217701	0.26984	0.246722	0.297135	0.444457
P09972	Fructose-bi	ALDOC ALD	-0.27913	-0.12562	-0.33751	-0.28325	0.017346	-0.0176	-0.24593	-0.4243	0.124783	0.256405
P0C6T2	Dolichyl-di	OST4	-0.60853	-0.90501	-1.01201	0.3101	-0.3344	-0.33555	-0.88327	0.598199	-0.90668	-0.22083
P0C7P0	CDGSH iror	CISD3	1.663557	1.784179	1.130016	1.321284	1.62117	1.630134	1.725488	1.763651	1.510144	1.20582
P0C7X5	Zinc finger	ZNF806	1.436099	1.290393	0.26628	0.823395	0.594844	0.529253	0.826233	0.331059	-0.1913	-0.11511
P0C870	Bifunctiona	JMJD7	-0.63743	-0.52576	-0.67963	-0.44461	-1.12418	-0.42234	-0.3322	-0.19592	-0.38702	-0.60726
P0DJD0	RANBP2-lik	RGPD1 RAI	-3.80735	-3.36146	-4.00782	-3.38606	-2.89947	-3.65208	-2.96963	-2.76783	-4.3505	-3.1788
P0DMV9	Heat shock	HSPA1B HS	0.674193	0.726884	1.262459	1.193809	1.237574	1.178924	1.046075	1.116544	0.973229	0.994294
P0DP25	Calmodulin	CALM3 CAI	1.207477	1.213452	-0.01507	0.182768	0.258055	0.208527	0.270413	0.344493	0.174488	-1.04495
P0DPB6	DNA-direct	POLR1D	-0.34023	-0.16069	-0.03953	0.079322	-0.10806	0.321928	0.195839	0.4236	0.470533	0.530855
P10109	Adrenodox	FDX1 ADX	-2.64689	-1.45457	-1.41244	-2.03814	-2.84503	-3	-2.16227	-1.56789	-2.00946	-1.64829
P10124	Serglycin (I	SRGN PRG	-0.37467	0.019678	-0.39696	-0.10661	-0.56982	-0.37093	-0.51782	-0.35486	-0.93254	-1.96505
P10155	60 kDa SS-/	RO60 SSA2	0.093867	0.094572	0.101614	0.165737	-0.28703	-0.1989	0.02032	-0.23581	-0.04793	-0.04952
P10253	Lysosomal	GAA	-1.94062	-1.57719	-1.5427	-1.0126	-1.71727	-2.06711	-1.81762	-1.78746	-2.14886	-2.1008
P10398	Serine/thre	ARAF ARAF	-1.64689	-2.17383	1.153367	0.765535	0.996277	1.388148	1.171061	1.261125	1.250407	1.944743
P10412	Histone H1	H1-4 H1F4	0.082462	0.852323	1.208399	0.961865	1.348454	1.032421	1.744619	1.344648	1.320041	1.479408

P10415	Apoptosis r	BCL2	-0.3072	-0.46341	-0.48241	-0.24617	-0.46294	0.132877	0.049732	-0.38461	-0.71275	-0.15539
P10515	Dihydrolipo	DLAT DLTA	1.093229	1.073348	1.336925	0.969451	0.880919	1.359673	1.42614	1.296762	1.176031	0.753419
P10606	Cytochrom	COX5B	2.217622	2.055458	1.990219	1.705257	1.662182	1.915608	1.904843	1.790904	1.548356	1.342409
P10619	Lysosomal	CTSA PPGB	-1.02445	-1.10512	-1.28345	-1.3377	-2.84503	-0.95693	-1.04152	-1.72935	-1.78165	-1.70487
P10644	cAMP-depe	PRKAR1A P	0.144101	0.186295	0.462964	0.330748	0.23969	0.636609	-0.02675	-0.13808	0.218067	0.426574
P10720	Platelet fac	PF4V1 CXC	0.138605	0.108863	0.335897	0.165737	0.699295	0.402689	1.005107	0.926602	0.778786	0.744029
P10746	Uroporphy	UROS	0.484775	0.274743	0.360322	0.020594	0.249656	-0.22186	0.216927	0.099447	0.609093	0.747794
P10768	S-formylglu	ESD	0.297001	0.463601	0.069805	0.238435	-0.2076	0.010521	0.29969	0.047538	0.434414	0.354794
P10809	60 kDa hea	HSPD1 HSP	0.466058	0.559448	0.961899	0.686987	0.756324	0.785329	0.882816	0.844239	1.12674	0.981668
P11021	Endoplasm	HSPA5 GRP	0.648214	0.686416	0.810693	0.863347	0.411314	0.600183	0.384986	0.606153	0.426247	0.276981
P11142	Heat shock	HSPA8 HSC	0.489946	0.396397	0.571906	0.498806	0.697526	0.399121	0.325095	0.554102	0.675708	0.898579
P11169	Solute carr	SLC2A3 GLU	0.296866	-0.02617	-0.97898	-1.45964	-0.621	-0.31654	-0.49479	-0.52018	-0.73383	-0.72169
P11171	Protein 4.1	EPB41 E41L	-0.13878	-0.11037	-0.22584	0.022499	0.448151	0.053996	0.273768	0.197244	0.208137	0.166063
P11177	Pyruvate de	PDHB PHE1	-0.15567	0.118218	-0.17508	-0.66393	-0.15998	-0.05249	-0.22352	-0.20146	-0.03949	-0.2665
P11182	Lipoamide	DBT BCATE	-0.58193	-0.53703	-1.39183	-1.24558	-1	-1.14958	-0.69661	-0.848	-0.95361	-0.85688
P11216	Glycogen p	PYGB	-0.04307	0.218756	0.186295	0.435281	0.160197	0.192871	0.079617	-0.04654	-0.03339	0.194304
P11274	Breakpoint	BCR BCR1 D	-0.86422	-0.53856	-0.16152	-0.3517	-1.26006	-0.98911	-1.22069	-1.45372	-1.15936	-0.50952
P11279	Lysosome-a	LAMP1	0.343924	0.733487	0.280182	0.37778	0.393608	0.342115	0.263873	0.370072	-0.07334	-0.31774
P11310	Medium-ch	ACADM	0.110807	-0.04354	0.582361	0.646363	0.043041	0.31755	0.740032	0.684498	0.082462	0.039289
P11387	DNA topois	TOP1	0.479567	0.471434	0.209171	0.329436	0.589912	0.189226	0.411195	0.212545	0.002983	0.013216
P11413	Glucose-6-p	G6PD	-0.15808	-0.19342	-0.43831	-0.5021	-0.37386	-0.37167	-0.353	-0.474	-0.34112	-0.38589
P11441	Ubiquitin-li	UBL4A DXS	-0.76546	-0.19837	-0.38404	-0.66954	-0.85681	-0.92437	-0.27644	-1.25322	-0.9376	-0.03365
P11586	C-1-tetrahy	MTHFD1 M	0.105182	0.223338	0.101614	-0.08264	-0.06853	-0.46697	0.030374	-0.12553	-0.21847	-0.0223
P11717	Cation-inde	IGF2R MPR	-0.11769	0.017938	-0.75107	-0.62919	-0.94581	-0.3045	0.005782	0.028498	-0.7577	-1.21431
P11766	Alcohol del	ADH5 ADH	1.611568	1.513071	1.240491	1.258532	1.212845	1.358349	1.471576	1.188235	1.401242	1.30017
P11908	Ribose-pho	PRPS2	1.165878	1.288798	0.721284	0.638326	0.91184	1.172352	1.209177	1.185111	1.252387	1.14112
P11940	Polyadenyl	PABPC1 PA	0.089111	0.518467	0.019365	0.4899	0.365871	0.334984	0.449163	0.294183	0.04182	0.198166
P12004	Proliferatin	PCNA	-0.23724	-0.22625	0.69262	0.525197	0.616374	0.321478	-0.06571	0.130904	0.565979	0.770213
P12074	Cytochrom	COX6A1 CO	0.180805	0.169215	0.741918	0.6067	0.924949	0.637939	0.925524	0.875081	0.688328	1.141956
P12110	Collagen al	COL6A2	-3.08746	-2.38163	-2.4021	-1.21613	-2.42999	-3.1375	-3.05209	-2	-1.81444	-1.8893
P12235	ADP/ATP tr	SLC25A4 AI	-0.86713	-0.5063	-0.74853	-0.36985	-0.80867	-1.0366	-0.96122	-2.20625	-1.19234	-1.62622
P12268	Inosine-5'-r	IMPDH2 IM	0.035013	0.25748	0.759542	0.803948	0.346311	0.392196	0.576328	0.644772	0.962905	0.825281
P12270	Nucleoprot	TPR	-0.22948	-0.1002	0.23183	0.13213	0.342411	0.078976	0.190084	0.232165	0.160465	0.257843

P12544	Granzyme /	GZMA CTL/	0.682611	0.852797	-0.16944	-0.11078	0.065423	0.577658	0.168788	0.406424	-1.05935	-1.64829
P12694	2-oxoisova	BCKDHA	-0.13993	-0.15634	-0.74727	-0.58405	-0.8055	-0.5366	-0.30666	-0.5764	-0.43608	-0.79823
P12814	Alpha-actir	ACTN1	0.909313	0.944389	-0.31336	0.275623	-0.24034	0.857848	0.452803	0.280108	0.393624	0.608932
P12955	Xaa-Pro dip	PEPD PRD	0.078635	0.427028	-0.37239	0.011337	-0.64717	-0.38484	-0.2165	-0.42271	-0.93964	-1.00767
P12956	X-ray repai	XRCC6 G22	0.227606	0.741847	0.565311	0.366298	0.447208	0.346747	0.611896	0.486043	0.580232	0.883213
P13010	X-ray repai	XRCC5 G22	0.543086	0.775554	0.527501	0.516454	0.67155	0.37933	0.417172	0.479946	0.649475	0.845421
P13073	Cytochrom	COX4I1 CO	0.93879	0.582915	-0.37656	-0.41044	-0.38495	0.343623	-0.22687	0.010213	-0.87575	-0.89932
P13489	Ribonuclea	RNH1 PRI F	0.208432	0.350432	0.60817	0.50763	0.520278	0.559232	0.460361	0.367016	0.816754	0.622815
P13501	C-C motif c	CCL5 D17S	-0.5915	-0.2111	-0.72728	-0.5516	-1.33666	-1.13826	-0.79256	-0.76582	-2.69146	-2.7669
P13612	Integrin alp	ITGA4 CD4	-0.30236	-0.07195	-0.09259	-0.0906	-0.34252	0.533035	0.380085	0.308122	-0.32075	-0.46609
P13639	Elongation	EEF2 EF2	1.044351	1.072721	0.584963	0.693669	0.778899	0.459432	0.755266	0.648429	0.803308	0.895753
P13667	Protein dis	PDIA4 ERP	0.612977	0.852323	0.627347	0.62595	0.399746	0.435386	0.489805	0.44907	0.459121	0.295324
P13693	Translation	TPT1	0.391369	0.458315	0.484899	0.296713	0.607774	0.266622	0.347845	0.413683	0.690193	0.593642
P13716	Delta-amin	ALAD	-0.21534	-0.28821	-0.89476	-0.86821	-0.63413	-0.635	-0.21474	-1	-0.32075	-0.04952
P13747	HLA class I	HLA-E HLA-	0.04182	-0.09675	0.892785	1.052355	0.377367	0.402689	0.418869	0.573467	0.301033	0.527789
P13796	Plastin-2 (L	LCP1 PLS2	1.119203	1.315676	-0.08961	0.00971	-0.35202	0.665948	0.391809	0.179365	0.055495	-0.54091
P13798	Acylamino-	APEH D3F1	-0.17763	-0.02024	-0.18607	0.174698	-0.22676	-0.12285	-0.21225	0.001676	-0.08356	-0.3796
P13804	Electron tr	ETFA	0.554081	0.468619	0.618366	0.701404	0.394441	0.470045	0.680628	0.733527	0.80185	0.383708
P13807	Glycogen [s	GYS1 GYS	-2.81741	-1.98608	-2.56306	-2.32227	-2.39489	-2.18727	-2.24335	-2.18667	-2.31721	-1.85661
P13861	cAMP-depe	PRKAR2A P	-0.09768	-0.04248	-0.28545	-0.05913	-0.28694	-0.38618	-0.43731	-0.22947	-0.78165	-0.31582
P13984	General tra	GTF2F2 RA	-0.27644	-0.36512	-0.25759	-0.34573	-0.4175	-0.53269	-0.49213	-0.48303	-0.46977	-0.18886
P13987	CD59 glyco	CD59 MIC1	-0.00607	0.019365	-0.45457	0.097024	-0.57755	-0.03877	-0.17955	-0.34066	-0.57089	-0.3375
P14151	L-selectin (	SELL LNHR	0.031057	0.377226	-0.22108	-0.28622	-0.29659	-0.6506	-0.62837	-0.72002	0.573049	0.305201
P14174	Macrophag	MIF GLIF M	0.275107	0.223506	-0.00782	0.324435	-1.9721	-1.89022	-1.71368	-1.75892	0.722752	0.474639
P14209	CD99 antig	CD99 MIC2	0.552998	0.630846	0.089183	0.395104	-0.27795	-0.05499	0.100879	0.063514	-0.21787	-0.64352
P14210	Hepatocyte	HGF HPTA	0.38514	0.335639	-0.29075	-0.51482	0.150633	-0.01498	0.107548	-0.0763	-0.34354	-0.44699
P14222	Perforin-1 (	PRF1 PFP	-1.34023	-1.13906	-1.20665	-0.5142	-1.93811	-1.47574	-1.14458	-1.84437	-1.54915	-1.78877
P14314	Glucosidas	PRKCSH G1	0.074165	0.175107	0.10341	0.314901	-0.11345	0.048024	0.173474	-0.12176	0.075724	-0.00694
P14317	Hematopoi	HCLS1 HS1	-0.07928	0.032032	0.213448	-0.09983	0.062566	-0.12437	0.26984	0.303491	0.422092	0.353976
P14324	Farnesyl py	FDPS FPS K	0.181093	-0.03773	-0.14487	-0.42617	-0.46818	-0.3427	-0.51367	-0.07792	-0.01463	0.074139
P14406	Cytochrom	COX7A2 CC	1.099536	0.97443	0.758637	1.082288	0.411314	1.386058	1.161945	1.053387	0.494156	0.320157
P14550	Aldo-keto r	AKR1A1 AL	0.953496	0.966975	1.142568	1.27552	1.134125	1.354191	1.305897	1.237881	0.517399	0.676411
P14618	Pyruvate ki	PKM OIP3 I	0.598184	0.392677	0.314109	0.320563	0.410334	0.467359	0.355583	0.383728	0.592202	0.559495

P14625	Endoplasm	HSP90B1 G	-0.23793	0.357349	0.411133	0.204358	0.072512	-0.00898	0.057345	-0.01728	-0.01896	-0.26658
P14678	Small nucle	SNRPB COD	1.828207	1.870221	1.057783	1.523198	1.225823	1.697018	1.642561	1.829716	1.539069	1.274486
P14854	Cytochrom	COX6B1 CC	-1.23661	-1.10512	-0.70867	-1.40453	-1.28654	-1.22914	-1.0721	-1.07784	-0.96536	-0.84304
P14866	Heterogen	HNRNPL HI	-0.06193	0.364521	0.426786	0.314372	0.31527	0.236414	0.261328	0.251507	0.56277	0.505048
P14868	Aspartate--	DARS1 DAF	0.630638	0.569645	-0.21403	-0.16434	-0.06075	0.053628	0.02878	-0.02961	-0.06947	-0.08044
P14921	Protein C-e	ETS1 EWSR	2.370797	2.301509	0.434403	0.84101	0.245577	1.441435	1.222392	0.644772	1.039528	0.555154
P14927	Cytochrom	UQCRB UQ	0.41743	0.355909	-0.88028	-0.09417	-0.47122	-0.14354	0.384947	-0.31018	-0.49561	-1.10018
P15121	Aldo-keto r	AKR1B1 AL	0.931731	1.133954	0.834616	0.991599	1.018487	1.308478	1.22928	1.241889	1.127635	1.121665
P15153	Ras-relatec	RAC2	1.674476	2.012046	1.465205	1.341444	1.374704	1.157902	1.600629	1.143581	1.167171	1.404784
P15170	Eukaryotic	GSPT1 ERF:	0.074007	-0.25061	-0.18265	-0.31312	-0.32379	-0.21628	-0.15846	-0.12942	-0.07748	0.03065
P15289	Arylsulfata:	ARSA	-1.70499	-1.27399	-1.41244	-3.14505	-1.87199	-1.31647	-1.69661	-1.76783	-2.04793	-3.1008
P15311	Ezrin (Cyto	EZR VIL2	0.080232	0.521187	0.801774	0.840993	0.652753	0.484898	0.730813	0.743357	0.341715	0.50614
P15336	Cyclic AMP	ATF2 CREB:	-0.68536	-0.58301	-0.51457	-0.66558	-0.62263	-0.71348	-0.68834	-0.848	-0.67996	0.155098
P15374	Ubiquitin c	UCHL3	0.292727	0.388639	0.168283	-0.08964	0.216925	0.150833	0.120512	-0.09183	0.372318	0.613379
P15498	Proto-onco	VAV1 VAV	-2.59115	-2.0157	-1.68589	-2.98307	-1.81855	-2.02203	-3.24549	-3.02609	-2.1913	-2.11991
P15529	Membrane	CD46 MCP	0.366718	0.406457	0.86406	0.858567	0.584528	0.65272	0.731472	0.791652	0.555238	0.579767
P15531	Nucleoside	NME1 NDP	0.955551	0.934185	0.797293	0.707955	0.907882	0.915608	0.785261	0.926602	1.108934	0.524716
P15586	N-acetylglu	GNS	-2.05993	-1.90195	-2.1965	-2.2574	-2.57363	-2.64922	-1.44199	-2.51934	-2.98201	-2.25536
P15848	Arylsulfata:	ARSB	-3.19438	-3.53138	-4.94642	-4.03814	-3.01495	-4.1375	NA	NA	-4.3505	-3.26127
P15880	40S ribosor	RPS2 RPS4	1.050626	1.032768	1.32546	1.125045	1.444481	1.280674	1.290155	1.152003	1.370146	1.321928
P15927	Replication	RPA2 REPA	-0.12363	0.124492	0.226842	0.15451	0.294905	0.053638	0.278301	0.005714	0.289507	0.50614
P15954	Cytochrom	COX7C	1.624818	1.451612	0.80624	0.809146	0.544745	1.217701	1.274077	0.662965	0.218346	-0.42986
P16070	CD44 antig	CD44 LHR I	0.762164	0.354315	0.927939	0.499896	0.794089	0.995338	0.993402	0.808328	0.844613	0.7015
P16104	Histone H2	H2AX H2AF	0.963163	2.020327	1.775819	2.020243	2.151631	1.856473	2.194722	2.112832	1.436099	1.576084
P16150	Leukosialin	SPN CD43	-0.19025	-0.29321	-0.92706	-1.5991	-1.35472	-1.19016	-1.69076	-0.90823	-0.64228	-0.50428
P16152	Carbonyl re	CBR1 CBR (	-1.39977	-0.88741	-1.5541	-1.3377	-1.2076	-0.93224	-1.38466	-1.02158	-0.58651	-0.85244
P16219	Short-chair	ACADS	-0.18746	-0.10093	-0.36146	-0.39428	-0.19903	0.14543	-0.19704	-0.35523	-1.13845	-1.28264
P16220	Cyclic AMP	CREB1	-1.07289	-1.01577	-1.05515	-1.38606	-1.22205	-0.99501	-1.36828	-1.15761	-1.09879	-0.59482
P16278	Beta-galact	GLB1 ELNR	1.588998	1.717732	0.60561	0.38813	0.134797	0.494765	0.605282	-0.1635	-0.67996	-0.69052
P16284	Platelet en	PECAM1	-1.70499	-1.50901	-1.88753	-2.84549	-1.76702	-1.48981	-1.7997	-2.88982	-1.55695	-1.66223
P16298	Serine/thre	PPP3CB CA	-0.02445	0.015513	0.030861	-0.05759	0.427993	0.394398	-0.53222	-0.07039	-0.46949	0.034976
P16333	Cytoplasmi	NCK1 NCK	-1.12584	-1.06405	-0.7112	-0.43368	-0.61978	-0.85752	-1.65892	-0.7087	-0.85998	-0.8267
P16383	Intron Larg	GCFC2 C2o	-0.89482	-0.78991	-1.34156	-0.95067	-2.69302	-1.03317	-0.97968	-0.96605	-1	-0.93088

P16401	Histone H1	H1-5 H1F5	0.395201	1.009715	0.781501	0.810659	0.624613	-0.23136	0.784601	0.515624	0.14919	-0.22979
P16402	Histone H1	H1-3 H1F3	0.265054	2.604969	-0.05206	0.407431	0.652753	0.028492	0.374618	0.090184	0.018737	0.655828
P16403	Histone H1	H1-2 H1F2	0.921655	0.919793	1.191344	0.948172	1.275375	0.451461	1.613665	0.878069	0.512294	0.521011
P16455	Methylatec	MGMT	-0.14609	-0.21185	-0.19103	0.000641	-0.44902	-0.48359	-0.29256	-0.58068	-0.08903	-0.0726
P16615	Sarcoplasm	ATP2A2 AT	-0.666	-0.83094	-0.86982	-0.92663	-0.87341	-1.07861	-0.89162	-1.10182	-1.55695	-1.31434
P16871	Interleukin	IL7R	-1.96408	-0.93147	-2.44143	-2.37901	-2.01777	-2.5887	-2.80688	-2.15086	-1.22396	-0.93088
P16949	Stathmin (L	STMN1 C1c	0.931164	0.97103	0.701932	0.882085	0.429918	0.412775	0.593104	0.720784	0.791433	0.435903
P16989	Y-box-bindi	YBX3 CSDA	0.826281	0.595323	0.718917	0.525197	0.923649	0.475242	0.730813	0.7022	0.882164	0.964155
P17010	Zinc finger	ZFX	-1.4221	-0.84159	-0.55229	-0.45584	-0.30884	-0.06792	-0.38059	-0.72512	-0.99659	-1.04495
P17026	Zinc finger	ZNF22 KOX	-0.36112	-0.55285	-0.14984	-0.61789	-0.37456	0.203182	0.74925	-0.75659	-0.87924	-1.45142
P17028	Zinc finger	ZNF24 KOX	0.76696	1.005837	0.151613	-0.20887	0.365871	0.079727	0.282513	-0.18286	0.168877	0.309483
P17050	Alpha-N-ac	NAGA	-1.04933	-0.85896	0.01165	0.061401	-0.34252	-0.03317	0.191366	-0.39232	-0.57089	-0.09134
P17066	Heat shock	HSPA6 HSP	-0.55497	-0.17823	-0.37151	-0.8011	-0.72955	-0.48981	-1.08427	-0.52607	-0.71049	-0.83303
P17096	High mobil	HMGA1 HN	0.225541	0.182864	0.706612	0.685554	0.59539	0.485827	0.467475	0.302274	0.432995	0.437541
P17174	Aspartate a	GOT1	-0.47217	-0.34307	-0.68576	-0.67689	-0.68179	-0.47949	-0.85712	-0.8511	-0.75449	-0.64296
P17252	Protein kin	PRKCA PKC	0.133088	0.545434	0.203328	0.209792	1.011112	0.915608	0.553936	0.371448	0.776035	0.846029
P17480	Nucleolar t	UBTF UBF U	0.640458	0.589375	0.367839	0.520832	0.735478	0.451461	0.752232	0.534473	0.537335	0.400728
P17535	Transcripti	JUND	-1.05897	-1.07683	-0.42257	-0.2612	-0.74606	-0.44055	-0.63408	-0.33276	-0.80356	-0.36125
P17568	NADH dehy	NDUFB7	-0.20406	0.002	-0.66539	-0.40555	-0.55925	-0.27536	-0.0677	-0.3643	-1.19436	-0.7857
P17612	cAMP-depe	PRKACA PK	0.053549	0.451612	0.521187	0.650365	0.7175	0.68012	0.471473	0.754039	0.917538	1.114796
P17655	Calpain-2 c	CAPN2 CAN	-0.10428	0.155119	-0.34156	-0.13813	-0.30529	-0.26303	-0.16801	-0.17638	-0.36257	-0.41799
P17676	CCAAT/enh	CEBPB TCF5	-2.76553	-2.5541	-3.48699	-2.9386	-3.69302	-3.45943	-3.38466	-3.33342	-4.08746	-3.13927
P17706	Tyrosine-pr	PTPN2 PTP	0.234925	0.05009	-0.66267	-0.48723	0.093877	-0.26042	-0.62159	-0.57085	-0.85355	-0.90884
P17812	CTP syntha	CTPS1 CTPS	0.105182	0.243406	-0.88033	-0.05107	-0.46036	-1.12553	-0.5175	-0.6735	-0.78165	-0.72668
P17844	Probable A	DDX5 G17P	0.609008	0.71792	0.65597	0.618865	0.443735	0.454784	0.703864	0.582267	0.508226	0.642099
P17858	ATP-depen	PFKL	0.379705	0.332536	0.384498	0.735711	0.519028	0.555519	0.667804	0.569615	0.510796	0.70384
P17931	Galectin-3	LGALS3 MA	1.667425	1.663375	1.027039	0.948926	1.261174	1.032421	1.709072	1.805718	0.756633	0.90157
P17980	26S protea	PSMC3 TBP	0.105864	0.030861	0.486931	0.359081	0.439006	0.471306	0.340229	0.303491	0.614398	0.582769
P17987	T-complex	TCP1 CCT1	0.316165	0.551433	0.401911	0.120294	0.096902	0.338586	0.36212	0.115158	0.480629	0.487153
P18031	Tyrosine-pr	PTPN1 PTP	0.143642	-0.08779	0.211985	-0.07237	-0.4861	-0.31278	-0.08496	-0.26397	0.050951	-0.04039
P18074	General tra	ERCC2 XPD	-1.37126	-2.26459	-2.00782	-3.32193	-1.81855	-3.04439	-2.3322	-1.91119	-2.14886	-2.34873
P18077	60S ribosor	RPL35A GIC	2.631681	2.736576	2.388227	2.501024	2.003714	2.509235	2.963736	2.417183	1.748237	1.809882
P18085	ADP-ribosy	ARF4 ARF2	1.572434	1.710435	0.581705	0.74592	0.497456	0.990058	0.954898	0.650525	0.500462	0.123971

P18124	60S ribosor	RPL7	1.504045	1.462561	0.886471	0.664121	0.847076	0.679293	0.796606	0.76362	0.897428	1.047862
P18206	Vinculin (V	VCL	0.059379	0.172522	0.249978	0.693669	0.043943	0.222392	0.06502	-0.18938	-0.00946	-0.23499
P18433	Receptor-t	PTPRA PTP.	-2.57289	-2.44392	-3.6734	-2.98751	-3.89947	-3.58496	-2.9301	-3.65535	-3.61353	-2.37145
P18583	Protein SOI	SON C21or	-0.41668	-0.12056	-0.09259	-0.08394	-0.34252	-0.19265	-0.50293	-0.24936	-0.14886	-0.02024
P18615	Negative el	NELFE RD F	-2.78041	-2.02359	-2.46529	-2.7168	-2.79256	-2.87447	-2.22342	-2.32398	-3.08746	-1.98424
P18621	60S ribosor	RPL17	0.650259	0.482639	0.443033	0.503944	0.699295	0.250227	0.63111	0.24492	0.146	0.295128
P18669	Phosphogly	PGAM1 PG	0.254929	0.597902	0.629657	0.241973	0.703976	0.362835	0.588353	0.232173	0.544994	0.680935
P18754	Regulator c	RCC1 CHC1	-0.25096	-0.18255	-0.20757	-0.5881	-0.37657	-0.61876	-0.41845	-0.62907	-0.21299	-0.14856
P18858	DNA ligase	LIG1	-1.10218	-1.16692	-0.92369	-1.19289	-0.58118	-1.12192	-1.02589	-1.31962	-0.78523	-0.78503
P18859	ATP syntha	ATP5PF ATI	-0.34593	0.025123	-0.21129	-0.20691	-0.25584	-0.02982	-0.3235	-0.08902	-0.13819	-0.22768
P18887	DNA repair	XRCC1	-0.97897	-1.13017	-1.20771	-1.16165	-1.14722	-1.10002	-1.85843	-1.11226	-1.3478	-0.8785
P19174	1-phosphat	PLCG1 PLC:	-0.2838	0.184506	-0.34423	-0.25388	-0.24403	-0.02327	-0.30256	-0.30497	-0.06512	0.064599
P19338	Nucleolin (I	NCL	0.791683	0.976413	0.749236	0.829634	0.797936	0.592067	0.74844	0.794074	1.18369	0.831648
P19367	Hexokinase	HK1	0.905127	0.826516	0.796999	0.525191	0.766403	0.803359	0.782843	0.726233	0.492753	0.080851
P19387	DNA-direct	POLR2C A-:	-0.64689	-0.22304	-0.41768	-0.4581	-0.09636	-0.32344	-0.34233	-0.40563	-0.13878	-0.21486
P19388	DNA-direct	POLR2E	0.633649	0.735375	0.375932	0.718084	0.640782	0.822881	0.874199	0.746076	0.722706	0.409845
P19404	NADH dehy	NDUFV2	-1.1004	-0.60293	-0.40816	0.044805	-0.19322	-0.29581	-0.26681	-0.31572	-0.45216	-0.42788
P19447	General tra	ERCC3 XPB	-0.8974	-0.58351	-0.24485	-0.16698	-0.21264	-0.25745	-0.63417	-0.24831	-0.16719	-0.28474
P19474	E3 ubiquitii	TRIM21 RN	-0.03737	-0.24206	0.007694	0.484947	-0.2426	-0.19277	-0.09089	-0.1103	-0.19922	-0.05954
P19525	Interferon-	EIF2AK2 PK	0.477126	0.224896	0.049509	-0.02398	0.176182	0.239584	0.120482	-0.00775	0.38161	0.163863
P19623	Spermidine	SRM SPS1 S	-0.46019	-0.45992	-0.41244	-0.52356	-0.37109	-0.24998	-0.46713	-0.30485	-0.22948	-0.21945
P19784	Casein kina	CSNK2A2 C	0.029995	-0.42286	-1.04757	-0.79021	-0.83173	-0.74061	-0.60039	-1.03477	-0.93546	-0.60054
P19838	Nuclear fac	NFKB1	0.286305	0.599102	0.148099	0.257002	0.200987	0.588601	0.117836	0.072569	0.281771	0.31564
P19971	Thymidine	TYMP ECGI	-2.68536	-1.52015	-1.7632	-3.14505	-3.2076	-2.0902	-2.16227	-2.848	-2.28011	-2.08194
P20020	Plasma me	ATP2B1 PM	-1.89482	-1.57719	-1.46529	-1.69574	-3.51245	-1.65208	-1.41163	-1.93289	-2.32665	-3.70487
P20042	Eukaryotic	EIF2S2 EIF2	0.147752	0.273691	-0.60962	-0.81908	-0.57071	-0.89361	-0.2878	-0.81371	-0.848	-0.40664
P20073	Annexin A7	ANXA7 ANX	-0.56393	-0.35534	-0.4736	-0.42825	-0.73574	-0.36021	-0.49658	-0.39608	-0.5332	-0.36981
P20290	Transcripti	BTF3 NACB	-1.14216	-0.92361	-0.58301	-0.62632	-0.46404	-0.71359	-0.41696	-0.64334	-0.77397	-0.53396
P20292	Arachidona	ALOX5AP F	1.160465	1.057333	0.826171	1.176878	0.007417	0.135515	0.654865	0.157146	-0.59918	-0.53495
P20339	Ras-relatec	RAB5A RAE	-1.01404	-0.52246	-2.42286	-2.8011	-2.95606	-3.0902	-2.96963	-3.1193	-3.08746	-3.34873
P20585	DNA mism:	MSH3 DUC	0.529628	0.429292	0.466353	0.580982	0.640679	0.603961	0.487889	0.608191	0.409714	0.498138
P20591	Interferon-	MX1	0.090729	0.208319	0.161423	0.327663	-0.42	-0.30293	0.303392	0.393407	0.104309	-0.33209
P20592	Interferon-	MX2	0.471504	0.393431	0.521187	0.425973	0.427993	0.529253	0.702799	0.893085	0.353637	0.676411

P20618	Proteasom	PSMB1 PSC	0.324351	0.550733	0.133954	-0.10402	-0.02651	0.037755	0.222392	0.120752	0.218346	0.323697
P20645	Cation-dep	M6PR MPR	0.80215	1.227686	0.408198	0.685891	0.043342	0.189226	0.47483	0.384664	0.104556	-0.05591
P20674	Cytochrom	COX5A	1.473159	1.143723	1.239651	1.215003	0.594768	1.602831	1.518142	1.427468	0.692338	0.515352
P20700	Lamin-B1	LMNB1 LM	0.498119	0.586946	0.542092	0.626666	0.342602	0.369234	0.480658	0.622633	0.845769	0.422761
P20701	Integrin alp	ITGAL CD1:	0.154817	0.23469	0.030452	-0.00651	-0.1161	-0.16175	-0.19704	-0.01728	-0.47603	-0.36573
P20718	Granzyme I	GZMH CGL	0.925361	1.158617	0.310969	0.043253	-1.07635	-0.61813	0.546949	0.061639	-1.4245	-1.2402
P20810	Calpastatin	CAST	0.889813	0.735405	0.66799	0.853443	0.765813	0.841186	0.642877	0.502551	0.402078	0.590848
P20839	Inosine-5'-r	IMPDH1 IM	0.245876	0.393312	0.218253	0.378867	0.079808	-0.19394	0.276968	0.181426	0.055467	0.172283
P20933	N(4)-(beta-	AGA	-0.43539	-0.90202	-1.26545	-1.90934	-0.15697	-0.36491	-1.50718	-1.39798	-0.67243	-1.0268
P20962	Parathymo	PTMS	0.378109	0.816953	0.217778	0.180571	-0.01214	-0.56664	0.228669	0.378715	-0.04331	-0.44651
P20963	T-cell surfa	CD247 CD3	-0.08104	-0.20945	0.090652	-0.27784	0.322995	0.18619	0.514867	0.026497	0.48614	0.650699
P21127	Cyclin-depe	CDK11B CD	-0.95987	-1.01976	-0.85329	-1.22212	-0.97451	-1.25634	-1.13105	-1.15949	-0.57417	-0.18779
P21266	Glutathion	GSTM3 GST	-1.76877	-1.17286	-2.33663	-1.7688	-2.15943	-1.99264	-2.73875	-1.95501	0.377547	0.451165
P21281	V-type prot	ATP6V1B2	1.491045	1.381508	1.591447	1.306795	1.59238	1.473275	1.26984	1.419325	1.591235	1.780555
P21283	V-type prot	ATP6V1C1	-0.43539	-0.37656	-1.43335	-1.65567	-0.97056	-1.74061	-1.29406	-0.6644	-0.4245	-1.8893
P21291	Cysteine ar	CSRP1 CSRI	-0.59104	-0.33241	-0.16593	-0.44461	-0.24648	-0.47107	-0.5252	-0.11697	-0.55014	-0.37718
P21333	Filamin-A (I	FLNA FLN F	0.659946	0.795445	0.44734	0.546827	0.072356	0.243316	0.30953	0.25634	-0.13402	-0.11276
P21399	Cytoplasmi	ACO1 IREB:	-0.74908	-0.74537	-0.75694	-0.81708	-0.64572	-0.85579	-0.95964	-0.95491	-0.52795	-0.63441
P21580	Tumor neci	TNFAIP3 OT	-0.04261	-0.02586	-0.54516	-0.96605	-0.86104	-0.87157	-0.1285	0.412964	-0.83628	-0.62677
P21675	Transcriptio	TAF1 BA2R	-1.04933	-0.78319	-0.94642	-0.46179	-0.31451	-1.22422	-1.03103	-1.22239	-0.64208	-1.04097
P21796	Voltage-de	VDAC1 VDA	0.431818	0.500558	-0.58717	-0.24943	-1.425	-0.0586	-0.24349	-0.19736	-1.15936	-1.55431
P21912	Succinate c	SDHB SDH :	-0.07309	0.054415	0.462972	0.299168	-0.28995	0.5025	0.415037	0.494395	0.149745	0.053972
P21953	2-oxoisova	BCKDHB	-0.41918	0.166921	0.248901	0.069817	-0.83094	0.198143	0.286006	-0.53269	0.007998	0.351144
P21964	Catechol O	COMT	1.280788	1.141785	0.545159	1.032325	0.55431	0.317336	0.844672	0.217838	0.224241	0.077439
P22033	Methylmal	MMUT MU	-0.48663	-0.49176	-0.52887	-0.38549	-0.35211	0.012009	-0.75573	-0.3414	-0.34055	-0.39775
P22059	Oxysterol-k	OSBP OSBF	-0.26269	-0.49462	-0.38805	-0.46022	-0.5544	-1.18684	-1.32499	-1.23135	-0.79875	-0.32608
P22061	Protein-L-is	PCMT1	0.852646	0.95367	0.680845	0.528307	1.271867	1.426221	0.7096	0.814857	1.003277	0.828863
P22087	rRNA 2'-O-r	FBL FIB1 FL	0.580995	0.672423	0.345512	0.148863	0.905713	0.557784	0.60253	0.577263	0.553431	0.673021
P22090	40S ribosor	RPS4Y1 RP!	0.556393	0.590152	-0.05971	0.176878	-4.83813	-2.54439	0.159657	-0.22239	0.143492	0.155098
P22102	Trifunction	GART PGFT	-0.57366	-0.68077	-0.59704	-0.78402	-1.00541	-0.7857	-0.52502	-0.72512	-1.1609	-1.22291
P22234	Multifuncti	PAICS ADE:	0.605512	0.686134	-0.29816	-0.57499	0.319759	0.305824	-0.51073	-0.38828	-0.15202	0.012791
P22304	Iduronate :	IDS SIDS	-1.38702	-1.14768	-0.63051	-0.83426	-1.74193	-1.75899	-1.71329	-1.07039	-2.06756	-2.06333
P22307	Non-specifi	SCP2	0.550639	0.365286	0.874229	0.755397	0.274891	0.423004	0.324436	0.576762	0.658043	0.580755

P22314	Ubiquitin-li	UBA1 A1S9	0.26	0.079643	-0.2276	0.165737	0.364716	0.020811	0.071448	0.028345	0.100164	0.27594
P22392	Nucleoside	NME2 NM2	1.42711	1.541421	1.009715	1.334419	1.117764	1.016302	1.141219	1.091498	1.080234	0.941291
P22570	NADPH:adr	FDXR ADXR	-1.10944	-1.10327	-0.03221	-0.20808	-0.28455	-0.20203	-0.27323	-0.07722	-0.22023	0.412371
P22626	Heterogene	HNRNPA2B	0.222797	0.322918	0.589621	0.481137	0.541594	0.497335	0.475158	0.611435	0.943163	0.748573
P22681	E3 ubiquiti	CBL CBL2 R	-1.7553	-1.6502	-1.8765	-3.06519	-1.30108	-1.57169	-2.32984	-1.6775	-1.2523	-1.35168
P22694	cAMP-depe	PRKACB	0.167112	0.132449	0.026397	0.07986	0.20912	0.136577	0.120837	-0.06137	0.057849	0.082645
P22695	Cytochrom	UQCRC2	0.926752	0.882029	0.010931	0.197276	0.118612	0.699016	0.888956	0.753946	0.489554	-0.27867
P22749	Granulysin	GNLY LAG2	-0.59104	-0.2185	-0.07195	-0.55087	-0.84503	-0.50524	-0.74723	-0.51785	-1.37474	-1.31531
P22830	Ferrochelat	FECH	-0.51207	-0.62968	-0.69855	-0.82848	-1.42999	-0.33821	-0.75038	-0.41199	-1.17659	-0.25722
P23025	DNA repair	XPA XPAC	0.993925	0.670742	1.009715	0.638326	1.261174	0.932886	1.025356	1.110183	-0.85487	-1.58597
P23193	Transcripti	TCEA1 GTF	-0.08104	0.123525	0.220019	0.305486	0.411314	-0.07424	0.027713	0.213981	0.219744	0.455165
P23229	Integrin alp	ITGA6	-0.12609	-0.34872	-0.11065	-0.39024	0.094634	-0.01668	-0.3002	-0.40814	-0.14652	0.043219
P23246	Splicing fac	SFPQ PSF	0.591392	0.735788	0.571906	0.496377	0.549834	0.468013	0.656414	0.654753	0.758815	0.763343
P23258	Tubulin gar	TUBG1 TUE	-0.21018	0.023302	-0.08767	-0.13686	-0.50943	-0.08558	-0.11878	0.024929	-0.02854	-0.00817
P23284	Peptidyl-pr	PPIB CYPB	1.009064	1.328153	1.388971	1.603659	1.3249	1.319741	1.224583	1.251539	1.301033	1.137101
P23368	NAD-deper	ME2	0.596233	0.579754	0.449956	0.328597	0.517917	0.532718	0.580376	0.620094	0.366224	0.606593
P23381	Tryptophar	WARS1 IFI5	-0.98793	-0.92405	-1.08844	-1.27563	-0.9852	-0.92545	-1.66382	-1.1571	-1.22396	-1.16882
P23396	40S ribosor	RPS3 OK/SW	1.690145	1.624702	1.10282	1.179918	1.119822	1.118794	1.369402	1.086858	1.221612	1.147755
P23434	Glycine cle	GCSH	-0.78629	-1.02359	-2.58911	-2.51907	-2.53785	-1.84049	-2.28178	-2.31575	-2.13938	-2.00068
P23458	Tyrosine-pr	JAK1 JAK1A	-1.39563	-0.49697	-0.82363	-0.77584	-1.0893	-0.66792	-0.57192	-1.40715	-0.66982	-0.90822
P23497	Nuclear au	SP100	-0.818	-0.48434	-0.54594	-0.51963	-0.50245	-0.59736	-0.76451	-0.28042	-0.41202	-0.49698
P23511	Nuclear tra	NFYA	0.219141	0.245208	0.123197	-0.41086	0.164848	-0.04586	0.197214	0.047134	0.540707	0.968268
P23526	Adenosylho	AHCY SAHH	0.218924	0.332805	0.133954	0.430634	0.28273	0.005454	0.497073	0.312739	0.540274	0.628552
P23528	Cofilin-1 (1	CFL1 CFL	0.486724	0.843747	1.158308	0.993342	1.210659	0.96917	0.942872	1.136462	1.211563	1.151118
P23588	Eukaryotic	EIF4B	-0.69968	-0.41543	-0.36357	-0.24455	0.065423	0.177806	0.339385	0.070159	0.089812	-0.05871
P23634	Plasma me	ATP2B4 AT	-0.91046	-0.65293	-0.73658	-0.36167	-0.90759	-0.55254	-0.51027	-0.52607	-1.13179	-1.41552
P23743	Diacylglyce	DGKA DAG	1.298518	1.413604	0.788821	0.796506	1.060596	1.090555	1.043474	1.059482	1.122233	0.946195
P23786	Carnitine O	CPT2 CPT1	-1.51978	-1.17383	-2.17383	-2.84549	-2.69302	-2.04439	-2.03103	-2.72935	-3.00946	-2.34873
P23919	Thymidylat	DTYMK CD	0.650343	0.626272	0.596788	0.49306	0.316475	0.3712	0.721892	0.673501	0.242445	-0.02914
P23921	Ribonucleo	RRM1 RR1	-1.43539	-2.12199	-1.87317	-2.75803	-3.51245	-2.2115	-1.69661	-3.02308	-2.37474	-1.90578
P24390	ER lumen p	KDELR1 ER	0.648214	0.507538	0.878009	1.636309	0.91184	0.915608	0.532874	2.123382	0.973833	0.831181
P24534	Elongation	EEF1B2 EEI	-0.07464	0.165586	0.291986	0.331316	0.365871	0.551796	0.344264	0.251531	0.684936	0.809625
P24539	ATP syntha	ATP5PB AT	1.941233	1.959469	1.235147	1.51426	1.183553	1.804229	1.821898	1.731804	1.048402	0.63016

P24557	Thromboxa	TBXAS1 CYI	-0.7626	-1.04399	-0.91102	-0.53681	-1.60132	-1.5127	-1.01856	-1.34396	-2.48926	-1.91877
P24666	Low molec	ACP1	-2.68536	-1.40239	-1.35147	-1.26053	-2.74193	-1.68684	-1.51019	-1.61973	-1.30319	-1.13927
P24723	Protein kin	PRKCH PKC	-0.30986	-0.38672	-0.35709	-0.31815	-0.37502	-0.25149	-0.65574	-0.43039	-0.67243	-0.74882
P24752	Acetyl-CoA	ACAT1 ACA	0.337965	-0.01906	0.93826	0.781877	0.492844	0.539996	0.708956	0.690956	0.688056	0.706146
P24928	DNA-direct	POLR2A PC	-0.42721	-0.75659	-0.58887	-0.76867	-0.41743	-0.10603	-0.89162	-0.47743	-0.31487	-0.22979
P24941	Cyclin-depe	CDK2 CDKN	-0.6054	-0.22715	-0.78884	-0.52695	-0.71206	-1.10365	-0.66289	-0.95919	-0.51542	-0.26609
P25098	Beta-adren	GRK2 ADRE	0.208094	0.205552	0.341443	0.417645	0.319787	0.374586	0.326853	0.398656	0.292073	0.092564
P25205	DNA replica	MCM3	-0.00306	-0.02822	-0.25151	-0.61092	-1.2067	-0.58217	-0.43971	-0.6029	-0.28778	-0.28904
P25208	Nuclear tra	NFYB HAP3	-1.08746	-0.78991	-0.60657	-0.47917	-0.89947	-0.44448	-0.66382	-0.91119	-0.66493	-0.37145
P25325	3-mercapto	MPST TST2	0.14632	0.090885	-0.20535	-0.13567	-0.3109	-0.53454	0.10334	0.061414	-0.29339	-0.35178
P25391	Laminin sul	LAMA1 LAN	1.265054	1.263034	1.624702	1.402437	1.609541	1.673282	1.134244	1.253941	1.507484	1.485743
P25398	40S ribosor	RPS12	1.129668	1.194051	1.116357	1.120294	1.248734	1.269921	1.148945	1.253941	1.477322	1.370145
P25440	Bromodom	BRD2 KIAA	0.060258	0.150241	0.342033	0.656862	-0.11941	0.43452	-0.08541	-1.08175	0.175153	0.070677
P25445	Tumor neci	FAS APT1 F	-0.12797	0.049873	-0.48835	-0.90298	-0.59921	-1.09888	-0.89128	-0.86626	-0.90866	-1.23376
P25490	Transcripti	YY1 INO80	-0.13135	0.210086	0.425721	0.961865	0.748815	0.892244	0.560888	0.193184	0.736966	0.662499
P25685	DnaJ homo	DNAJB1 DN	0.633607	0.580629	1.005857	0.499659	0.974107	0.833627	0.944618	1.026472	0.673337	0.923335
P25686	DnaJ homo	DNAJB2 HS	0.523035	0.655971	-0.50669	-0.2986	-0.28615	-0.62904	-0.31369	-0.60478	-0.93546	-1.00888
P25705	ATP syntha	ATP5F1A A	0.63342	0.461935	0.649406	0.815636	0.584963	0.743494	0.984473	0.881451	0.624863	0.558442
P25774	Cathepsin S	CTSS	0.883259	0.819558	0.220163	-0.01894	0.28883	0.455452	0.761315	0.626346	0.254365	-0.27191
P25786	Proteasom	PSMA1 HC	0.377468	0.379705	0.363373	0.273407	0.196272	0.471306	0.566461	0.616609	0.468861	0.592808
P25787	Proteasom	PSMA2 HC	0.596835	0.711529	0.314664	0.694794	0.137352	0.442796	0.700051	0.753779	0.410848	0.137493
P25788	Proteasom	PSMA3 HC	1.130362	1.381508	0.625886	0.551115	0.482236	0.603064	0.841959	0.519875	0.556304	0.844519
P25789	Proteasom	PSMA4 HC	0.021539	0.024094	-0.75001	-0.64943	-0.13676	0.286175	-0.09431	-0.63723	-0.45396	-0.6836
P26010	Integrin be	ITGB7	-0.49394	-0.75002	-1.39183	-1.52356	-0.46036	-1.27621	-0.45305	-0.45372	-0.66493	-0.26127
P26038	Moesin (M	MSN	0.363894	0.585719	0.848689	0.7507	0.789283	0.676928	0.761291	0.709	0.983403	0.902396
P26196	Probable A	DDX6 HLR2	0.665413	0.299925	0.546341	0.204266	0.54711	0.415288	0.404284	0.476424	0.618185	0.66876
P26358	DNA (cytos	DNMT1 AIN	0.171271	0.351262	0.068531	0.108706	0.28883	0.299902	0.195839	0.110183	-0.30319	-0.05871
P26368	Splicing fac	U2AF2 U2A	0.076726	0.2824	-0.62449	-0.30633	-0.02248	-0.21785	-0.63971	-0.15073	-0.65748	-0.27191
P26373	60S ribosor	RPL13 BBC	0.042577	0.021184	0.537397	1.031027	1.151631	0.652879	0.5115	0.607683	0.606586	1.069124
P26440	Isovaleryl-C	IVD	1.148751	1.207241	0.884421	0.608561	0.671907	1.107854	1.055176	1.041697	0.074252	-0.31969
P26447	Protein S1(	S100A4 CA	1.290851	1.379358	1.662003	1.71426	1.669214	0.817366	1.135161	1.071386	0.609891	0.80609
P26583	High mobil	HMGB2 HN	0.357095	0.854481	0.711793	0.425973	1.137106	0.439422	0.286713	0.826851	0.689796	0.844603
P26599	Polypyrimi	PTBP1 PTB	0.067171	-0.19599	-1.13906	-0.67557	-0.79256	-0.635	0.030374	-1.35995	0.182637	0.207329

P26639	Threonine-	TARS1 TAR	0.82737	0.873722	0.938821	0.742372	0.232977	-0.11366	1.092153	0.929611	1.273994	0.896825
P26640	Valine--tRN	VARS1 G7A	-0.18619	-0.12763	-0.36858	-0.26706	-0.37109	-0.282	-0.13206	-0.29197	-0.15938	0.319203
P26641	Elongation	EEF1G EF1(	1.033632	1.053405	0.962212	0.943682	1.247857	1.057117	1.352969	1.370664	1.189446	1.21974
P26842	CD27 antig	CD27 TNFR	0.127977	0.268473	-0.65911	-0.82612	-0.69543	-0.80627	-0.49566	-0.75672	-0.5571	-0.55437
P26885	Peptidyl-pr	FKBP2 FKBI	-0.9706	-0.91942	-0.46855	-0.86486	-0.5943	-0.81673	-1.02387	-0.96281	-1.01962	-0.89851
P27105	Stomatin (E	STOM BND	-0.10428	-0.18122	-0.67794	-1.33778	-1.91675	-1.3767	-0.26645	-0.75039	-2.15388	-2.25505
P27348	14-3-3 prot	YWHAQ	-0.41136	0.062066	-0.11545	-0.08535	-0.521	-0.33793	-0.24033	-0.26454	-0.31452	-0.50557
P27361	Mitogen-ac	MAPK3 ERI	0.083382	0.189718	0.405256	0.309905	0.239016	0.217701	0.160674	0.375482	0.349069	0.636037
P27449	V-type prot	ATP6V0C A	-3.01988	-2.92422	-3.09968	-1.92549	-1.9828	-1.51135	-3.00149	-1.30472	-3.00349	-2.46629
P27635	60S ribosor	RPL10 DXS(	0.455348	0.172485	-0.23467	-0.34614	-0.03464	0.272404	0.202064	0.118156	-0.40498	-0.16043
P27694	Replication	RPA1 REPA	0.310954	0.228487	-0.18709	-0.20025	0.093574	0.268489	0.113114	0.212545	-0.04307	0.047877
P27695	DNA-(apuri	APEX1 APE	1.428658	1.468619	1.023091	1.162229	1.410951	1.123476	1.041404	0.960829	1.42702	1.486519
P27707	Deoxycytid	DCK	-0.39429	-0.44756	-0.58116	-0.42228	-0.77114	-0.38595	-0.74721	-0.83107	-0.70886	-0.71429
P27708	CAD protei	CAD	-0.18057	-0.08017	-0.33662	-0.32979	-0.62263	-0.38618	-0.23326	-0.43812	-0.30945	-0.39453
P27797	Calreticulin	CALR CRTC	1.306312	1.414353	1.208695	1.201634	0.783146	0.58512	0.989067	0.919413	0.682713	0.506559
P27816	Microtubul	MAP4	0.966269	1.027039	0.968464	0.674146	0.503375	0.606658	0.692992	0.659345	0.600593	0.759796
P27824	Calnexin (IF	CANX	1.229304	1.288798	0.687751	0.416605	0.405712	0.115031	1.029952	0.265894	-0.35281	-0.26829
P27986	Phosphatid	PIK3R1 GRI	-0.15455	-0.09477	-0.17253	-0.2321	-0.40658	-0.1394	0.045324	0.237039	-0.25955	-0.30832
P27987	Inositol-tris	ITPKB	-0.96408	-0.77649	-0.63051	-0.86839	-0.40914	-0.72247	-0.75585	-0.3994	-0.30319	-0.47489
P28062	Proteasom	PSMB8 LM	-0.09075	-0.24267	-1.04281	-0.63008	-0.69198	-0.7104	-0.52246	-0.74036	-0.31373	-0.18213
P28065	Proteasom	PSMB9 LM	1.152306	1.179425	0.140172	0.350669	0.276604	0.174774	0.527812	0.328261	0.442635	0.703535
P28066	Proteasom	PSMA5	0.529469	0.489876	0.90116	0.453718	0.557857	1.279786	0.48631	0.842899	1.216318	1.202019
P28070	Proteasom	PSMB4 PR(	1.138605	1.23017	-0.19599	0.225972	-0.05302	0.494765	0.758294	0.280108	-0.06263	0.396106
P28161	Glutathion	GSTM2 GS	-0.94062	-0.73048	-1.53138	-1.5787	-1.12418	-0.05004	-0.74723	-0.29078	0.540274	0.783129
P28331	NADH-ubiq	NDUFS1	0.672857	0.738118	1.004431	0.918245	0.859519	0.753267	0.927025	0.872235	0.966614	0.9732
P28340	DNA polym	POLD1 POL	-0.41908	-0.29776	0.049819	0.024875	-0.06853	-0.24998	-0.378	-0.52607	-0.07251	0.131052
P28482	Mitogen-ac	MAPK1 ERI	1.809218	1.810547	1.658443	1.299168	1.315967	1.273018	1.435988	1.5025	1.441317	1.385981
P28715	DNA repair	ERCC5 ERC	-1.59104	-1.24598	-1.58887	-2.8011	-1.47063	-1.45943	-1.46713	-1.23581	-1.31487	-1.84093
P28799	Progranulir	GRN	-2.40048	-1.87042	-0.0039	0.334419	-1.62902	-1.19293	-1.64539	-1.70588	0.218346	-0.08194
P28838	Cytosol am	LAP3 LAPEI	-0.27876	-0.11762	0.256385	0.567888	-0.00778	0.087606	0.337008	0.47014	-0.4177	-0.55767
P28907	ADP-ribosy	CD38	0.234465	0.288798	-0.40726	-0.62632	-0.04532	0.135515	-0.15656	-0.09464	0.549967	0.452172
P29083	General tra	GTF2E1 TF2	-0.74392	-0.81368	-0.49187	-0.79202	-1.15402	-0.9558	-0.24361	-0.20034	-0.56799	-1.28007
P29084	Transcripti	GTF2E2 TF2	-1.38702	-1.20945	-1.12199	-1.75803	-1.31451	-1.1989	-1.31937	-1.08246	-0.67243	-1.11032

P29144	Tripeptidyl	TPP2	-0.08746	-0.25526	0.072245	-0.06413	-0.1161	0.179608	-0.12274	-0.22689	0.397248	0.429349
P29218	Inositol mo	IMPA1 IMP	0.04182	-0.17823	-0.28345	-0.08394	0.342602	0.140481	0.239827	0.241889	0.453419	0.422761
P29350	Tyrosine-pr	PTPN6 HCP	0.741807	1.079643	1.052896	1.15952	1.040332	1.214798	1.26559	1.192432	0.736966	0.76131
P29353	SHC-transfo	SHC1 SHC S	-1.26546	-1.60064	-1.75002	-1.75803	-1.42999	-1.03317	-2.03103	-2.14439	-1.78165	-1.8893
P29372	DNA-3-met	MPG AAG A	0.029787	0.05142	-0.22475	0.029078	-0.02298	-0.1208	-0.25691	-0.33342	0.117652	-0.22461
P29374	AT-rich inte	ARID4A RB	-2.80735	-1.73697	-1.10512	-1.47045	-0.71727	-1	-1.13955	-0.848	-1.21299	-1.12956
P29401	Transketola	TKT	0.87564	1.021616	-0.18709	-0.12439	-0.22487	0	0.51763	0.083416	0.130629	0.127005
P29466	Caspase-1 (	CASP1 IL1B	0.663437	0.656435	0.521496	0.276643	0.318845	0.341389	0.563478	0.384169	0.170519	0.070923
P29590	Protein PM	PML MYL P	-0.38923	-0.35856	0.193132	0.055377	-0.03471	0.016907	0.072532	-0.01903	0.137608	-0.02553
P29692	Elongation	EEF1D EF1D	-1.22992	-0.78991	-0.5656	-0.50564	-0.64576	-1	-0.9898	-0.69188	-0.8514	-0.47836
P29728	2'-5'-oligoa	OAS2	-3.19438	-2.07195	-1.66827	-2.41884	-2.6163	-1.56875	-1.74438	-2.53139	-2.71101	-1.50198
P30040	Endoplasm	ERP29 C12c	-0.44409	-0.60032	0.038657	-0.06881	-0.42688	-0.28001	-0.38332	-0.36312	-0.18921	-0.3693
P30041	Peroxiredo	PRDX6 AOF	0.338802	0.45446	-0.58301	-0.49011	-0.51495	-0.55435	-0.38863	-0.24257	-0.01421	-0.11276
P30042	Glutamine	GATD3B HE	0.371969	0.600476	1.280796	1.160134	1.154975	1.465381	1.31166	1.571542	1.10953	0.898899
P30043	Flavin redu	BLVRB FLR	-0.19145	-0.07713	-8.43E-05	-0.03338	0.152	0.224882	0.206722	0.042276	-0.02544	-0.15654
P30044	Peroxiredo	PRDX5 ACR	1.528647	1.485154	1.624696	1.566896	1.733879	1.793936	1.647699	1.871683	1.987807	1.991757
P30046	D-dopachro	DDT	0.212351	0.287479	-0.13299	0.344842	-0.08786	0.153122	0.206974	0.16977	0.29989	0.098527
P30048	Thioredoxi	PRDX3 AOF	0.876672	0.881005	0.81247	0.78805	0.914051	0.93012	1.12858	1.061639	1.066793	0.761413
P30049	ATP syntha	ATP5F1D A	-0.44845	-0.43878	-1.00395	-0.89204	-1.02966	-1.33758	-0.82841	-1.12091	-0.90274	-1.03132
P30050	60S ribosor	RPL12	0.719892	0.707023	0.800644	0.725603	0.59976	0.06413	0.729495	0.690489	0.952502	1.043781
P30084	Enoyl-CoA	ECHS1	-0.09023	0.488929	0.551072	0.633724	0.785819	1.047135	0.82909	0.827093	-0.06191	-0.03068
P30085	UMP-CMP	CMPK1 CM	-0.12003	-0.36146	0.219121	-0.47874	0.47155	0.163444	0.149311	0.141064	0.494909	0.181694
P30086	Phosphatid	PEBP1 PBP	0.440573	0.58756	0.186295	0.584963	0.014797	0.730393	0.364274	0.490326	0.455622	0.419547
P30101	Protein disu	PDIA3 ERP5	0.382492	0.406301	0.852297	0.512062	0.258068	0.544321	0.336091	0.349122	0.509794	0.365517
P30153	Serine/thre	PPP2R1A	0.720746	0.707098	0.695031	0.399183	0.603887	0.559232	0.68455	0.51056	0.609891	0.269657
P30154	Serine/thre	PPP2R1B	-0.71491	-0.87317	-1.45457	-1	-0.55552	-0.90484	-1.18536	-0.54265	-0.51592	-0.54137
P30203	T-cell differ	CD6	-0.24377	-0.2185	0.01165	0.25861	0.148	-0.37906	-0.13391	-0.04102	-0.39941	0.036126
P30260	Cell divisior	CDC27 ANA	-3.43539	-3.32193	-2.32193	-3.75803	NA	NA	NA	NA	-3.08746	-3.44184
P30405	Peptidyl-pr	PPIF CYP3	-0.57289	-0.54839	-0.93147	-0.56937	-1.74193	-0.46697	-0.41163	-0.12553	-0.92647	-0.73402
P30414	NK-tumor r	NKTR	-1.42659	-0.63779	-0.49693	-0.58273	-0.52345	-0.67662	-0.65248	-0.68433	-0.37082	-0.2056
P30419	Glycylpepti	NMT1 NMT	-0.28444	-0.09472	-0.11231	0.202651	0.22623	0.166499	0.136858	0.19863	-0.0677	0.049666
P30464	HLA class I	HLA-B HLAI	0.12755	-0.08844	NA	NA	-0.75442	-0.65208	1.235488	1.227291	-0.90866	-1.34873
P30485	HLA class I	HLA-B HLAI	-1.34023	-1.42286	0.795405	0.440164	0.519084	0.892015	0.911042	0.598199	-1.58496	-1.93934

P30501	HLA class I	HLA-C HLA(	-2.14739	-2.10375	-0.52576	-0.5787	-1.44888	-0.91009	-1.5697	-3.02308	-1.76553	-1.58054
P30511	HLA class I	HLA-F HLA-	-0.6958	-0.51975	-0.75605	-0.61837	-2.10575	-1.62334	-0.03815	-0.27005	-1.21572	-1.49788
P30519	Heme oxyg	HMOX2 HC	1.614957	1.696234	0.730713	0.780219	1.033082	0.447459	0.817551	0.436348	0.600593	0.499895
P30520	Adenylosuc	ADSS2 ADS	-0.03532	-0.30256	0.307822	0.439912	0.121186	0.330645	-0.06274	0.16506	0.193823	0.243729
P30533	Alpha-2-ma	LRPAP1 A2	0.809086	0.057333	1.842571	1.212502	0.843031	1.212994	0.888355	0.902303	0.375607	2.387674
P30566	Adenylosuc	ADSL AMPS	0.055616	0.479748	0.463859	0.658648	0.675708	0.53804	0.591524	0.425441	0.576688	0.704712
P30622	CAP-Gly do	CLIP1 CYLN	-0.57521	-0.62825	-0.39932	-0.71371	-0.90981	-0.64612	-0.79082	-0.62355	-0.63936	-0.49075
P30626	Sorcin (22 l	SRI	-0.91867	-0.65841	0.525633	0.89331	0.537887	0.889549	0.951933	0.770655	0.983475	0.947659
P30740	Leukocyte (	SERPINB1 E	0.277894	0.242649	0.038574	-0.14903	0.114333	0.004951	0.099581	0.131585	0.100901	0.235941
P30837	Aldehyde d	ALDH1B1 A	-1.12974	-0.86785	-0.29048	-0.25932	-1.92114	-1.65782	-0.32798	-0.21049	0.600731	0.3161
P30876	DNA-direct	POLR2B	0.08253	0.007777	0.112475	0.316635	0.342602	0.356384	0.274077	0.232173	0.297201	0.468255
P31040	Succinate c	SDHA SDH2	0.413514	0.44062	0.543576	0.323141	0.429231	0.375552	0.60661	0.553159	0.50357	0.362013
P31146	Coronin-1A	CORO1A C(	0.345639	0.612032	0.331765	0.131682	0.21374	0.333978	0.332041	0.273314	0.295485	0.469598
P31150	Rab GDP di	GDI1 GDIL (	-0.99822	-0.50612	0.23349	0	0.45537	0.189226	-1.32379	-1.25788	-1.28586	-0.9766
P31153	S-adenosyl	MAT2A AM	-0.17005	0.078434	0.027039	0.034064	-0.1487	0.061512	-0.01235	0.039389	0.079267	0.094215
P31321	cAMP-depe	PRKAR1B	-1.96486	-1.18456	-1.21086	-1.435	-0.73979	-0.63685	-1.04688	-1.55228	-0.64366	-0.77672
P31483	Nucleolysir	TIA1	-1.73668	-1.88178	-1.81616	-2.00575	-1.53527	-2.18727	-1.97972	-1.89514	-0.98654	-1.68713
P31629	Transcripti(	HIVEP2	-0.6429	-0.44196	-0.63217	-0.39855	-0.53398	-0.57094	0.122543	-0.26303	-0.55162	-0.2691
P31689	DnaJ homo	DNAJA1 DN	-0.86139	-0.90202	-0.73048	-0.90298	-0.83173	-0.81558	-0.83577	-0.69126	-0.73897	-0.52218
P31751	RAC-beta s	AKT2	-0.13986	-0.31708	-0.424	-0.35487	-0.42987	-0.35078	-0.36365	-0.13154	-0.08355	-0.25826
P31930	Cytochrom	UQCRC1	0.865558	0.750658	0.118866	0.347468	0.083358	0.599738	0.637346	0.637715	0.222477	-0.03491
P31937	3-hydroxyi:	HIBADH	0.371236	0.432556	0.8379	0.879145	0.700795	0.780513	0.859952	0.925201	1.382796	1.096057
P31939	Bifunctiona	ATIC PURH	0.26	0.507538	0.179425	-0.0906	0.239291	0.415037	0.218001	0.326501	0.285644	0.412823
P31942	Heterogen(	HNRNPH3 I	-0.86139	-1.2276	-0.84115	-0.82189	-0.71989	-0.83436	-0.67195	-1.14439	-0.82033	-0.63128
P31943	Heterogen(	HNRNPH1 I	-1.01988	-1.17673	-0.67798	-0.47303	-0.54391	-0.73108	-0.57094	-0.45965	-0.58264	-0.47847
P31946	14-3-3 prot	YWHAB	-0.79575	-0.80228	-0.00152	-0.10495	0.08652	-0.50362	0.303965	0.16451	0.352187	0.2095
P31948	Stress-indu	STIP1	1.073849	1.021288	1.231831	1.142803	1.15831	1.084889	1.060121	1.135606	1.323813	1.152314
P31949	Protein S1(	S100A11 M	0.20847	0.114424	0.307822	0.314381	-0.14048	0.048363	0.050273	0.217477	-0.28011	-0.3431
P32119	Peroxiredo	PRDX2 NKE	1.189788	1.274528	1.215277	1.301136	1.138236	1.223063	1.198431	1.506461	1.585219	1.567687
P32121	Beta-arrest	ARRB2 ARE	0.177567	0.138277	-0.12894	-0.45042	-0.02119	0.137479	0.359484	0.083681	-0.03807	-0.05993
P32249	G-protein c	GPR183 EB	-1.666	-1.6734	0.493758	0.439912	0.051139	0.443446	0.966357	0.839704	-0.07748	-0.09134
P32455	Guanylate-	GBP1	0.14609	0.301509	0.687761	0.455859	0.244472	0.319659	0.446799	0.52654	0.51176	0.598171
P32456	Guanylate-	GBP2	0.412803	0.585747	0.472001	0.483044	0.519028	0.282035	0.570511	0.427862	0.541784	0.465797

P32519	ETS-relatec	ELF1	-0.82571	-0.73911	-0.39193	-0.66045	-0.77055	-0.73969	-1.25666	-1.29419	-0.54078	-0.79676
P32780	General tra	GTF2H1 BT	0.381307	0.068531	0.175978	0.288936	0.207442	0.19878	0.040358	-0.37737	0.266174	0.008824
P32942	Intercellula	ICAM3	-0.411	-0.37151	-0.95395	-1.00268	-0.74193	-1.03317	-0.9255	-1.1571	-0.9445	-1.11032
P32969	60S ribosor	RPL9 OK/S\	1.813383	2.17025	1.519827	1.591221	1.537078	1.612031	1.509705	1.207595	1.451696	1.952768
P33176	Kinesin-1 h	KIF5B KNS	-0.47697	-0.64874	-0.18709	-0.39428	-0.37109	-0.33703	-0.60817	-0.43812	-0.20754	-0.31838
P33240	Cleavage st	CSTF2	-1.93427	-1.65061	-2.92088	-2.7761	-1.4555	-1.42069	-2.53999	-2.73196	-2.88946	-2.43344
P33241	Lymphocyt	LSP1 WP34	0.737845	0.904155	0.997049	0.826149	0.862297	0.745035	0.636667	0.985401	1.055495	1.010799
P33316	Deoxyuridi	DUT	-0.32122	0.158617	-0.43062	-0.41689	-0.6574	-1.02203	-1.34566	-1.33342	-0.7028	-0.62081
P33527	Multidrug r	ABCC1 MRI	-1.13752	-1.29755	-0.67911	-0.54408	-0.19017	-0.50499	-1.00044	-0.10418	-0.50241	-0.44251
P33991	DNA replica	MCM4 CDC	-0.91754	-0.83094	-1.06378	-1.23078	-0.90597	-0.79845	-0.21951	-0.41891	-1.1281	-0.82517
P33992	DNA replica	MCM5 CDC	-0.34993	-0.7632	-0.63098	-0.62285	-1.17815	-1.00091	-0.98513	-0.86569	-0.87643	-0.8478
P33993	DNA replica	MCM7 CDC	0.313899	0.21283	-0.53223	-0.25199	-1.18229	-0.78597	-0.21615	-0.50373	-0.71159	-0.4475
P34896	Serine hydr	SHMT1	-0.76553	-0.7632	-1.4761	-0.52356	-1.42999	-1.42968	-1.08427	-3.80735	-1.43721	-1.21945
P34897	Serine hydr	SHMT2	0.013986	0.11614	-0.74561	-0.51907	-0.87844	-0.82721	-0.37161	-0.8867	-0.77639	-0.76306
P34910	Protein EVI	EVI2B EVDI	0.137522	0.407776	-0.31518	-0.14699	-0.70164	-0.48602	-0.2234	-0.51543	-0.54087	-0.27588
P34913	Bifunctiona	EPHX2	-0.08104	0.123255	-0.08844	0.006259	-0.48097	-0.74977	-0.1452	-0.52607	-0.10764	-0.38294
P34932	Heat shock	HSPA4 APG	0.318341	0.409827	0.119559	-0.02221	0.584963	0.3988	0.131912	0.289507	0.409429	0.531308
P34947	G protein-c	GRK5 GPRK	-0.83954	-1.23676	-0.87317	-1.52356	-0.68105	-0.47454	-0.9898	-1.27684	-0.29739	-0.69768
P34949	Mannose-6	MPI PMI1	0.105182	0.256521	-0.87317	-0.73697	-0.59991	-0.30627	-0.66382	-0.28842	-0.37038	-0.11083
P35080	Profilin-2 (I	PFN2	-3.39482	-2.27402	-2.76204	-2.84831	-2.4855	-2.07167	-3.11735	-2.35867	-2.03851	-2.00373
P35226	Polycomb c	BMI1 PCGF	-1.20832	-0.91397	-0.59533	-0.92373	-1.13946	-0.59434	-1.60248	-0.51808	-0.33259	-0.73443
P35232	Prohibitin	PHB PHB1	0.994272	1.238277	1.189716	1.157325	1.264273	1.251042	1.30797	1.375251	1.219133	1.152836
P35236	Tyrosine-pr	PTPN7	-0.04544	-0.32803	-0.43464	-0.56629	-0.46376	0.043673	-0.53964	-0.23922	-0.31487	-0.1322
P35237	Serpin B6 (	SERPINB6 F	0.314092	0.395091	0.460271	0.399183	0.465935	0.589685	0.652754	0.677227	0.487599	0.423075
P35241	Radixin	RDX	-1.13993	-0.68589	-0.98449	-0.75803	-0.6574	-1.06711	-1.18536	-0.37737	-0.64268	-0.45998
P35244	Replication	RPA3 REPA	0.109091	0.419024	-0.44282	-0.20621	0.038047	-0.2421	0.166748	-0.00091	0.287151	0.465104
P35249	Replication	RFC4	0.018072	-0.00782	-0.42286	-0.26806	-0.26006	-0.07285	0.074768	0.25634	0.100164	-0.14906
P35250	Replication	RFC2	-3.03684	-2.03953	-2.30256	-3.14505	-1.51245	-2.79647	-1.782	-2.39232	-3.08746	-2.2402
P35251	Replication	RFC1 RFC14	-1.12017	-1.0342	-1.14168	-1.12382	-1.00377	-1.07012	-1.24795	-1.76205	-1.05369	-1.05566
P35268	60S ribosor	RPL22	-0.55497	-0.47069	-0.81589	-0.68562	-1.05304	-0.81558	-1.20881	-1.05842	-0.96277	-0.87685
P35269	General tra	GTF2F1 RA	-0.38279	-0.44048	-0.35032	-0.51469	-0.54119	-0.41928	-0.69005	-0.80653	-0.61526	-0.38374
P35573	Glycogen d	AGL GDE	-0.04933	0.015513	-0.30778	-0.3851	-0.1161	-0.19265	-0.17377	-0.18584	-0.05282	0.273324
P35579	Myosin-9 ((	MYH9	0.299939	0.246804	0.615823	0.462849	0.572512	0.455402	0.500694	0.522566	0.725606	0.711674

P35580	Myosin-10	MYH10	-1.18057	-1.43335	-0.98449	-0.56009	-1.41009	-0.94636	-1.22069	-1.04654	-0.76553	-0.51584
P35606	Coatomer s	COPB2	0.155031	0.27275	0.064808	-0.08394	0.226636	0.032421	0.257053	0.389042	0.277888	0.302324
P35609	Alpha-actir	ACTN2	1.582941	1.60817	1.717732	1.697542	1.054723	1.208271	1.473317	1.434231	0.773279	0.803557
P35610	Sterol O-ac	SOAT1 ACA	1.856726	2.074098	1.682938	1.867037	1.96233	1.647349	1.833428	1.81551	1.416804	1.370661
P35611	Alpha-addu	ADD1 ADD.	0.118066	0.045395	-0.21366	-0.05759	-0.26031	-0.20769	-0.54709	-0.14306	0.06454	0.368948
P35613	Basigin (5F	BSG UNQ6!	-3.89482	-1.84488	-0.63051	-0.13813	-0.13231	-0.18021	0.055206	-0.05247	-0.78165	-0.50324
P35637	RNA-bindir	FUS TLS	-0.07464	0.27919	-0.1936	-0.12092	-0.02603	0.263945	-0.03485	0.115477	0.173871	0.228098
P35658	Nuclear po	NUP214 CA	0.217786	0.441118	0.3046	0.157794	0.503995	0.273111	0.175831	0.267566	0.144753	-0.12959
P35659	Protein DEI	DEK	0.661904	0.317903	0.609532	0.329436	0.274789	0.122607	0.336182	0.26362	0.105681	0.480081
P35749	Myosin-11	MYH11 KIA	-0.37912	-0.85896	-0.10512	-0.95067	-0.35199	-0.28284	-0.58496	-0.74846	-0.24058	-0.44184
P35754	Glutaredox	GLRX GRX	0.440212	0.462729	-0.32126	0.102633	-0.16024	-0.21134	-0.10602	-0.16414	0.229412	0.123717
P35813	Protein pho	PPM1A PPI	0.116409	-0.69849	-0.34651	0.085247	-0.02248	-0.37197	-0.48134	-0.95491	0.023383	-0.28264
P35914	Hydroxyme	HMGCL	-0.92411	-0.7393	-0.48288	-0.50049	-0.24392	0.093714	-0.4449	-0.58531	-0.96243	-1.24711
P35998	26S protea	PSMC2 MS	-0.0559	0.176306	-0.28512	-0.26952	-0.28789	-0.26527	-0.28938	-0.46908	-0.336	-0.20147
P36402	Transcriptic	TCF7 TCF1	-1.46855	-0.78319	-0.55984	-0.65567	-0.13231	-0.28951	-0.58496	-0.86876	0	0.228701
P36405	ADP-ribosy	ARL3 ARFL:	-0.23848	0.062527	0.213225	0.616881	0.550978	0.447926	0.371286	0.255695	-0.07871	0.249688
P36507	Dual specif	MAP2K2 M	0.719892	0.804009	1.288798	0.765535	0.903913	0.727095	0.500481	0.474661	0.462844	0.244097
P36542	ATP syntha	ATP5F1C A	0.794625	0.889593	0.040968	0.480992	0.509829	1.079334	1.100329	0.978795	0.037637	-0.2602
P36543	V-type prot	ATP6V1E1 .	-0.02445	-0.04354	0.622904	0.402437	0.609541	0.33931	0.274077	0.261125	0.643479	0.815026
P36551	Oxygen-de	CPOX CPO	0.86007	0.968464	0.491285	0.445169	0.27497	0.745617	0.508575	0.613209	0.456056	0.170908
P36578	60S ribosor	RPL4 RPL1	1.368131	1.426713	1.207667	0.834466	1.399608	1.324112	1.022479	1.34915	1.404274	1.449638
P36639	7,8-dihydrc	NUDT1 MT	-1.40537	-0.64352	-1.20144	-0.57237	-1.20515	-1.00017	-0.58996	-0.56774	-0.89252	-1.00003
P36776	Lon protea	LONP1 PRS	0.816429	0.652435	-0.00209	-0.152	-0.34722	0.395433	0.301693	0.120771	-0.12844	-0.0406
P36871	Phosphoglu	PGM1	0.234465	0.395882	-0.20881	-0.14156	-0.42999	-0.26303	-0.22004	-0.33342	-0.26303	-0.18886
P36873	Serine/thre	PPP1CC	0.055309	0.178257	0.185418	0.231612	-0.08127	0.094364	0.065954	0.018097	0.279172	0.123831
P36915	Guanine nu	GNL1 HSR1	0.925841	0.90004	0.697437	0.467393	0.809478	0.743508	0.811733	0.698677	0.480704	0.372371
P36954	DNA-direct	POLR2I	-0.89152	-0.5008	-0.41278	-0.40044	-0.8108	-0.50499	-0.2549	-0.24492	0.030818	-0.09308
P36957	Dihydrolipc	DLST DLTS	-0.93403	-1.14014	-0.01963	-0.41086	-0.61488	-0.29626	-1.11293	-0.97728	-0.55349	-1.14403
P36969	Phospholip	GPX4	0.798669	1.0925	0.625965	0.626185	0.306978	0.871485	0.546949	0.554102	0.312468	0.251185
P37108	Signal reco	SRP14	-0.3347	-0.22584	0.038399	-0.34356	-0.00777	0.260715	0.209313	-0.02338	-0.01671	0.118463
P37173	TGF-beta re	TGFBR2	-3.30986	-1.36146	-1.44392	-1.67557	-1.45017	-1.26303	-0.6477	-0.39985	-0.55003	-0.88112
P37198	Nuclear po	NUP62	-0.97615	-0.46825	-0.59474	-0.38606	-0.47063	-0.77761	-0.95964	-0.50967	-0.23502	-0.42392
P37802	Transgelin-	TAGLN2 KI/	-0.81545	-0.79387	1.248338	1.061401	0.899933	1.142958	0.791186	1.36257	0.500835	0.798477

P37837	Transaldola	TALDO1 TA	0.998944	1.069543	0.946712	1.148852	1.389133	1.324665	1.438055	1.500596	1.537498	1.69026
P38117	Electron tra	ETFB FP585	0.535635	0.53123	0.657741	0.580195	0.408533	0.569904	0.704171	0.48811	0.563758	0.639184
P38159	RNA-bindir	RBMX HNR	0.705095	0.767827	-1.13017	-1.937	-0.09212	-0.58496	-1.09404	-0.90344	-1.22224	-0.86307
P38432	Coilin (p80	COIL CLN80	-3.80735	-4.72403	-4.36146	-3.9386	-3.59991	-4.34395	NA	NA	-4.3505	-4.0268
P38606	V-type prot	ATP6V1A A	0.514573	0.767827	0.858787	0.635342	0.778191	0.787235	0.955846	0.861924	0.871858	0.540619
P38646	Stress-70 p	HSPA9 GRF	0.051549	-0.0552	0.291986	0.176878	-0.06075	-0.05858	0.035374	0.017639	0.110496	0.043589
P38919	Eukaryotic	EIF4A3 DD)	1.028693	1.070866	0.450754	0.632403	0.31009	0.430758	0.760848	0.705309	0.439424	0.298081
P38935	DNA-bindir	IGHMBP2 S	-2.21002	-1.70378	-1.45231	-1.19161	-1.12921	-1.25633	-1.07616	-1.31238	-1.31292	-1.51263
P39019	40S ribosor	RPS19	1.566662	1.716546	1.31177	1.225277	1.361225	1.423945	1.566924	1.482424	1.664123	1.725237
P39023	60S ribosor	RPL3 OK/S\	1.474168	1.337591	1.377943	1.366398	1.537078	1.471818	1.51797	1.344535	1.38426	1.371003
P39656	Dolichyl-di	DDOST KIA	-1.38702	-1.57719	-2.32193	-1.14505	-1.74193	-1.28951	-1.45988	-0.98583	-1.92985	-1.24157
P39687	Acidic leuci	ANP32A C1	0.737129	0.881495	1.369486	1.119379	1.118085	1.093332	1.126429	1.091121	1.390008	1.290742
P39748	Flap endon	FEN1 RAD2	0.316477	0.407882	0.503406	0.235588	-0.00512	0.380003	0.641167	0.557471	-0.13686	0.282035
P39880	Homeobox	CUX1 CUTL	-1.07464	-1.96153	-1.32193	-1.27563	-1.2076	-0.98911	-0.87404	-0.76362	-0.95361	-1
P40121	Macrophag	CAPG AFCP	0.556246	0.572569	1.484034	1.3186	1.411314	1.308753	1.564352	1.588774	1.254587	1.088123
P40189	Interleukin	IL6ST	-1.60942	-1.4021	-1.45457	-1.20163	-2.69302	-1.27621	-1.46713	-1.65535	-1.39941	-1.37145
P40222	Alpha-taxili	TXLNA TXLI	-0.70277	-0.58887	-0.7112	-0.9386	-0.58869	-0.56866	-0.81223	-0.87925	-0.7028	-0.82517
P40227	T-complex	CCT6A CCT	0.808357	0.722601	0.925575	0.946632	1.034888	0.746769	0.800085	0.939503	1.171954	0.9922
P40306	Proteasom	PSMB10 LN	-0.05561	0.552592	0.612326	0.055377	0.744909	0.459367	0.089267	0.515732	0.858429	0.524716
P40429	60S ribosor	RPL13A	0.224123	0.513013	0.932354	0.692542	0.801951	0.951317	1.023183	0.755675	0.784897	1.129535
P40763	Signal trans	STAT3 APR	-0.05706	0.320296	0.045433	0.021594	-0.00051	-0.09465	0.170274	0.073734	-0.00163	-0.17687
P40818	Ubiquitin c	USP8 KIAA(	0.105006	0.257086	-0.07498	0.249953	-0.05651	0.134519	0.233135	0.052139	-0.22669	-0.17866
P40855	Peroxisoma	PEX19 HK3	-0.54681	-0.15586	0.135103	-0.08042	0.188122	-0.0436	-0.2701	-0.06457	0.248764	0.140221
P40925	Malate deh	MDH1 MDI	0.41515	0.758637	0.748705	0.779587	0.855054	0.820823	0.727307	0.716143	0.927835	0.943646
P40926	Malate deh	MDH2	0.684487	0.864178	0.735532	0.789033	0.529039	0.552311	0.906378	0.914501	0.796691	0.445117
P40937	Replication	RFC5	-2.23668	-1.41878	-1.31407	-0.80292	-1.17404	-0.85868	-1.11697	-1.09812	-1.79017	-1.63128
P40938	Replication	RFC3	-1.60516	-1.72495	-1.98204	-1.7475	-2.04057	-1.07167	-1.09491	-1.29455	-1.34608	-1.29131
P40939	Trifunction	HADHA HA	0.239609	0.369485	0.69262	0.430634	0.306978	0.373458	0.445411	0.303491	0.379237	0.202019
P41091	Eukaryotic	EIF2S3 EIF2	0.41834	0.506446	0.468579	0.397684	0.177448	0.330304	0.514569	0.371683	0.565554	0.513842
P41208	Centrin-2 ((	CETN2 CAL	0.396482	0.462593	-0.79807	-0.51199	-0.0826	0.429117	-0.04883	0.054343	-0.4718	-0.54392
P41212	Transcripti	ETV6 TEL T	-1.40296	-1.17383	-1	-0.75803	-1.71727	-1.16175	-1.30666	-1.58496	-1.68753	-1.50324
P41214	Eukaryotic	EIF2D HCA!	0.553443	0.598465	0.141044	0.480939	0.498119	0.313158	0.677251	0.637453	0.453419	0.496762
P41218	Myeloid ce	MNDA	0.727234	0.704633	-0.60657	-0.42764	-0.40024	-0.2962	0.71843	0.265894	-0.64268	-0.37718

P41223	Protein BU	BUD31 EDC	-0.40754	-0.16129	-0.05638	-0.05991	0.041378	-0.20262	-0.45305	-0.40742	-0.4119	-0.30433
P41226	Ubiquitin-li	UBA7 UBE1	-0.97305	-0.68048	-0.24632	-0.3159	-1.53046	-0.73901	-0.64455	-0.83585	-0.40798	-0.36914
P41227	N-alpha-ac	NAA10 ARD	-0.3693	-0.39696	-0.26929	-0.60709	-0.33313	-0.02203	-0.33865	-0.17638	-0.55003	-0.19898
P41236	Protein pho	PPP1R2 IPP	-0.90967	-0.99813	-1.06181	-1.5029	-0.88212	-1.11225	-1.21416	-0.92269	-0.56304	-0.75068
P41240	Tyrosine-pr	CSK	0.082462	0.150989	0.304669	0.288936	0.031923	0.29366	0.349798	0.157146	-0.07269	0.000733
P41250	Glycine--tR	GARS1 GAF	0.694203	0.729862	0.52661	0.806659	0.312976	0.479168	0.6019	0.623533	0.944	0.611274
P41252	Isoleucine-	IARS1 IARS	0.70221	0.534708	0.216811	0.422588	0.507664	0.730393	0.605282	0.506536	0.830075	0.518551
P41567	Eukaryotic	EIF1 SUI1	-0.73245	-1.10273	-0.12275	0.091568	0.081539	0.026979	-0.43274	-0.1653	0.062502	-0.09242
P42025	Beta-centra	ACTR1B CT	-0.48458	-0.73697	-0.70008	-0.43837	-0.81831	-0.39008	-0.46007	-0.73079	-0.23	-0.18103
P42126	Enoyl-CoA	ECI1 DCI	2.003028	2.295167	0.92805	0.801968	1.054723	1.548063	1.465928	1.154577	1.289507	0.803557
P42166	Lamina-ass	TMPO LAP2	-1.28011	-1.02359	-0.57138	-0.68965	-1.01495	-0.9783	-1.06274	-0.94828	-0.68701	-0.4841
P42167	Lamina-ass	TMPO LAP2	0.335226	0.411205	0.182864	0.076323	0.200987	0.335978	0.657873	0.308122	0.193711	0.442305
P42224	Signal trans	STAT1	1.203205	1.251514	1.171156	1.364023	0.377945	0.746769	1.149793	1.123382	1.160898	1.125989
P42226	Signal trans	STAT6	-1.49255	-1.25385	-1.40801	-1.55113	-1.66127	-1.33107	-1.23447	-1.63184	-1.40295	-1.48026
P42229	Signal trans	STAT5A STA	0.686383	0.523901	-0.51457	0.108706	-0.36151	-0.15565	0.103623	-0.02308	-0.2916	-0.50952
P42285	Exosome R	MTREX DO	0.207716	0.221699	0.424524	0.133325	0.270702	0.403973	0.275987	0.041105	0.386842	0.353747
P42331	Rho GTPase	ARHGAP25	0.287215	0.564016	0.4859	0.394258	0.345908	0.528709	0.450597	0.200086	0.063835	0.0859
P42338	Phosphatid	PIK3CB PIK	-0.97595	-1.53138	-1.11353	-1.10402	-0.89947	-0.85457	-1.04152	-1.2091	-0.83954	-0.8893
P42345	Serine/thre	MTOR FRAI	-0.83429	-1.02673	-0.89314	-0.58949	-0.8099	-0.62787	-0.86328	-0.68129	-0.5677	-0.31408
P42566	Epidermal g	EPS15 AF1P	-0.41181	-0.48061	-0.26348	-0.29598	-0.71981	-0.81337	-0.66953	-0.39691	-0.69614	-0.31531
P42574	Caspase-3 (	CASP3 CPP	-0.18367	-0.16313	-0.92128	-0.96098	-0.70647	-0.25474	-0.93137	-0.96063	-0.43701	-0.87081
P42575	Caspase-2 (	CASP2 ICH1	-0.00838	0.094575	0.586505	0.936849	0.238784	0.63588	0.543209	0.157844	0.077033	0.310728
P42677	40S ribosor	RPS27 MPS	1.371969	1.60689	1.682938	0.9874	1.327865	1.459432	1.456638	1.380272	1.628309	1.256751
P42679	Megakaryo	MATK CTK	-1.55036	-1.38955	-1.061	-2.10459	-1.0832	-2.08088	-2.03422	-1.89719	-0.86507	-0.93934
P42696	RNA-bindir	RBM34 KIA	-0.9112	-0.80386	-1.23676	-1.56009	-1	-1.26303	-1.11718	-1.1193	-1.01898	-0.82517
P42704	Leucine-ric	LRPPRC LRI	0.584963	0.608419	-0.09095	-0.13525	-0.26105	-0.07183	0.051844	-0.23321	-0.20228	-0.50204
P42765	3-ketoacyl-	ACAA2	0.228784	0.368334	-0.13607	-0.42389	-0.30977	-0.17522	-0.23952	-0.17297	-0.23779	-0.27735
P42766	60S ribosor	RPL35	0.452614	0.286472	0.453516	0.321679	0.368486	0.436033	0.210411	0.395415	-0.01533	0.434495
P42768	Wiskott-Ald	WAS IMD2	-0.0525	-0.03886	-0.27363	-0.40255	-0.9286	-0.48817	-0.25691	-0.60985	-0.32879	-0.2301
P42858	Huntingtin	HTT HD IT1	-1.18057	-1.03953	-0.83094	-0.7794	-0.22487	-0.55254	-1.22069	-0.96605	-0.97199	-0.90578
P43034	Platelet-act	PAFAH1B1	-0.24578	0.045708	-0.52491	-0.51732	-0.73164	-0.30726	-0.54071	-0.59733	-0.1786	-0.1738
P43116	Prostagland	PTGER2	-2.57289	-2.10512	-2.05565	-1.48794	-3.89947	-1.93587	-2.03103	-2.97728	NA	NA
P43243	Matrin-3	MATR3 KIA	0.4975	0.655382	0.516873	0.277212	0.871762	0.635086	0.705939	0.73163	0.583369	0.645109

P43246	DNA mism:	MSH2	0.610097	0.552765	0.694419	0.800122	0.15819	0.330664	0.067072	-0.156	-0.17623	-0.09337
P43250	G protein-c	GRK6 GPRK	-0.46673	-0.48285	-0.77535	-0.26508	-0.12313	-0.15586	-0.45388	-0.3939	-0.73795	-1.08977
P43304	Glycerol-3-	GPD2	-1.46673	-1.11067	-1.17242	-1.06278	-0.77195	-0.68435	-0.98209	-1.13011	-1.69221	-1.72714
P43307	Translocon	SSR1 TRAP/	0.331874	0.540004	-1.06465	-0.92618	-1.44745	-0.98659	-0.63814	-1.53434	-2.32221	-1.89896
P43403	Tyrosine-pı	ZAP70 SRK	-0.04482	-0.01864	0.162105	-0.30035	-0.84527	-0.41511	-0.01518	-0.04417	-0.69727	-0.3832
P43487	Ran-specifi	RANBP1	0.850404	1.059672	0.723647	0.61137	0.742694	0.570316	0.692839	0.626346	1.014075	0.977701
P43490	Nicotinami	NAMPT PB	0.286836	0.284102	0.061177	0.252734	0.151182	0.177175	0.326637	0.221716	0.190163	0.359473
P43686	26S protea	PSMC4 MIF	0.598947	0.577207	0.925817	0.706154	0.888884	1.036388	0.690035	0.804452	0.978668	0.90182
P43897	Elongation	TSFM	0.005607	-0.04994	0.497871	0.781406	-0.21118	0.055442	0.001658	-0.17393	-0.32915	-0.50899
P45880	Voltage-de	VDAC2	0.607387	0.542921	-0.06592	0.333561	-0.60726	0.404688	0.432931	0.223142	-0.51977	-0.64829
P45954	Short/bran	ACADSB	0.929157	0.780102	0.820945	0.912002	0.614864	1.091383	1.064166	1.020559	0.848511	0.560263
P45973	Chromobo:	CBX5 HP1A	0.339502	0.258667	0.163191	0.361423	0.479155	0.613747	0.362726	0.167054	0.850604	0.684248
P45974	Ubiquitin c	USP5 ISOT	0.25443	0.509365	0.729582	0.783024	0.709079	0.831551	0.592869	0.557995	0.691198	0.694291
P45984	Mitogen-ac	MAPK9 JNK	-0.71491	-0.72403	-0.53703	-0.82312	-0.91341	-0.27621	-0.38466	-0.62855	-0.44361	-0.41799
P45985	Dual specif	MAP2K4 JN	-1.04933	-1.57719	-0.93892	-1.2909	-1.2076	-0.90484	-1.11718	-1.26303	-1.33852	-0.8893
P46019	Phosphoryl	PHKA2 PHK	-1.22684	-0.90979	-0.9145	-0.60629	-1.04174	-0.49771	-0.7467	-0.92212	-0.74512	-0.7453
P46060	Ran GTPas	RANGAP1 I	-0.30986	-0.53703	-0.34156	-0.36167	-0.56649	-0.21785	-0.52484	-0.64636	-0.39941	-0.63329
P46063	ATP-depen	RECQL REC	0.5752	0.660911	0.178837	0.182416	0.152	0.095157	0.234309	0.272104	-0.01421	0.102654
P46087	Probable 2	NOP2 NOL1	-0.68536	-0.20043	-0.15507	-0.51711	0.133667	-0.06659	-0.14904	-0.37737	-0.74169	-0.69768
P46100	Transcripti	ATRX RAD5	-0.5025	-0.6672	-1.36146	-1	-0.92749	-0.57679	-0.75298	-0.6605	-0.74227	-0.80202
P46109	Crk-like prc	CRKL	-0.39345	-0.27264	-0.31829	-0.19075	-0.34884	-0.2871	-0.24048	-0.08989	-0.22017	-0.05224
P46199	Translation	MTIF2	-1.6696	-2.05574	-1.91787	-1.63815	-1.7275	-1.20553	-1.96023	-1.11375	-1.21084	-1.35893
P46379	Large proliı	BAG6 BAT3	-0.42763	-0.1315	-0.18648	-0.01701	-0.31391	-0.27718	-0.23546	-0.39946	-0.34684	-0.34337
P46459	Vesicle-fusi	NSF	-0.22239	-0.19153	0.351262	-0.18728	-0.02248	-0.04439	-0.06809	0.050626	-0.11273	-0.05365
P46527	Cyclin-depe	CDKN1B KII	-0.14662	-0.1766	-0.51577	-0.34413	-0.21732	-0.53767	0.001341	-0.43039	-0.35193	-0.10555
P46734	Dual specif	MAP2K3 M	-1.12318	-0.78687	-0.97679	-1.3377	-1.87199	-1.26303	-0.94973	-1.10692	-1.14886	-1.45391
P46736	Lys-63-spe	BRCC3 BRC	0.388685	0.358299	0.094997	-0.0024	0.099125	0.002418	0.044727	-0.11388	0.034917	0.245682
P46776	60S ribosoı	RPL27A	-0.19162	0.008845	0.694693	0.879024	0.875821	0.991779	1.374176	0.61891	0.900813	0.937233
P46777	60S ribosoı	RPL5 MSTP	0.667425	0.655971	0.939188	0.261081	1.002535	0.737733	0.966495	0.911001	1.175074	1.010772
P46778	60S ribosoı	RPL21	0.640232	1.032768	1.567966	0.783867	1.470989	1.249495	1.303863	0.914504	1.423851	1.295128
P46779	60S ribosoı	RPL28	0.727811	0.492537	0.223205	0.315813	0.903912	0.645222	0.434897	0.347272	0.259004	0.115693
P46781	40S ribosoı	RPS9	1.839892	1.904289	-0.01175	0.220599	0.549834	0.053638	0.62532	0.565752	0.082462	-0.05411
P46782	40S ribosoı	RPS5	0.418065	0.843658	0.735405	-0.00629	0.411314	0.459432	0.62532	0.603921	0.751041	0.87044

P46783	40S ribosor	RPS10	0.831689	0.632559	0.692533	0.634086	0.480787	0.826952	0.989143	0.828945	0.498427	0.433823
P46926	Glucosamir	GNPDA1 GI	0.453374	0.91845	-0.60639	-0.27984	-0.43754	-0.13869	-0.02467	-0.4273	0.212298	0.154144
P46939	Utrophin (I	UTRN DMD	-2.74106	-1.7112	-0.98449	-1.35364	-1.64647	-1.37197	-1.4957	-1.42269	-1.47603	-1.28264
P46940	Ras GTPase	IQGAP1 KIA	0.833208	0.588963	0.375801	0.396852	0.401658	0.171712	0.321857	0.267944	-0.07917	-0.08243
P46976	Glycogenin	GYG1 GYG	-0.41993	-0.45694	-0.75538	-0.70525	-0.4471	-0.59732	-0.55271	-0.64954	-0.50873	-0.65563
P46977	Dolichyl-di	STT3A ITM	-0.40065	-0.52576	-0.72403	-0.8011	-1.33313	-1.38618	-0.80863	-0.60224	-1.02857	-1.13927
P47224	Guanine nu	RABIF MSS	-0.411	-0.43862	0.363436	0.304257	-0.22487	-0.09603	-0.26303	-0.61973	-0.29739	0.034976
P47755	F-actin-cap	CAPZA2	0.117004	0.219373	-0.30666	-0.04016	-0.53427	-0.02322	-0.17022	0.043689	-0.20165	-0.30409
P47756	F-actin-cap	CAPZB	0.766855	0.942907	0.868672	0.836153	0.826527	0.791478	0.845052	0.73475	0.766664	1.016931
P47813	Eukaryotic	EIF1AX EIF	-0.01961	0.087424	0.035792	-0.20627	0.845866	0.673083	0.166494	0.078502	0.283063	0.486005
P47897	Glutamine-	QARS1 QAI	-0.63688	-0.35564	-0.64352	-0.50243	-0.84626	-0.98769	-0.93638	-0.894	-0.79084	-0.48842
P47914	60S ribosor	RPL29	2.057923	1.908449	2.204207	1.815219	2.022361	2.053638	1.985862	2.106199	1.987834	2.217326
P47985	Cytochrom	UQCRFS1	0.572199	0.54518	0.686731	0.876978	0.622774	1.250535	1.184759	1.152376	0.118004	-0.18382
P48047	ATP syntha	ATP5PO AT	0.498819	0.377229	0.989945	0.924616	0.794816	1.109624	0.922721	1.122959	1.154395	0.849127
P48059	LIM and se	LIMS1 PINC	1.622852	1.548103	0.751707	0.844508	0.950834	0.676705	1.263461	1.164827	0.762203	0.882493
P48147	Prolyl endo	PREP PEP	1.195295	1.332805	0.819558	0.909397	1.138179	1.288761	-0.35165	0.265894	-0.18057	0.026311
P48382	DNA-bindir	RFX5	-1.4199	-1.05663	0.121079	-0.30333	-0.53483	-0.55683	-0.43325	-0.43737	-0.19159	-0.08304
P48426	Phosphatid	PIP4K2A PI	0.30402	0.144571	0.357798	0.223266	-0.04704	0.031223	0.107142	0.311405	-0.12341	0.111169
P48444	Coatomer s	ARCN1 COI	0.438391	0.614693	0.60561	0.701404	0.676212	0.551796	0.368243	0.670241	0.418569	0.391967
P48454	Serine/thre	PPP3CC CA	0.761624	0.977034	0.755969	0.665347	0.341158	0.610664	0.561959	0.631036	0.626126	0.268082
P48506	Glutamate-	GCLC GLCL	0.284031	0.194991	-0.04286	-0.29281	-0.72394	-0.44248	-0.03396	-0.59956	-0.17101	-0.16461
P48507	Glutamate-	GCLM GLCI	0.20847	0.27275	0.432172	0.396276	-0.01495	-0.22422	0.278301	-0.45372	-1.08746	-0.69768
P48553	Trafficking	TRAPPC10	-1.19438	-1.6734	-1.66102	-1.23078	-1.15697	-0.86449	-1.46713	-1.29078	-1.10764	-1.66223
P48556	26S protea	PSMD8	-0.98873	-0.67064	-0.9337	-1.06056	0.796692	0.455452	-1.32362	-1.06143	-1.74111	-1.38785
P48634	Protein PRI	PRRC2A BA	-1.8766	-1.44049	-1.49548	-1.29352	-1.40914	-1.45951	-1.68434	-1.71104	-1.48298	-1.00526
P48637	Glutathion	GSS	0.351985	0.276275	-0.25111	0.287006	-0.07851	0.209255	0.152754	0.170452	0.019637	0.033863
P48643	T-complex	CCT5 CCTE	0.588998	0.67319	0.817191	0.697542	0.632743	0.657769	0.651612	0.584963	0.898947	0.764788
P48735	Isocitrate d	IDH2	0.882417	1.251435	0.665835	0.781628	0.258068	0.735115	1.042482	1.019847	0.193823	-0.14416
P48736	Phosphatid	PIK3CG	-3.37126	-2.15634	-2.60064	-4.52356	-4.14048	-2.06711	-2.96963	-3.27684	-3.86507	-3.06333
P48739	Phosphatid	PITPNB	0.621264	0.751148	0.667494	0.643134	0.625445	0.552281	0.480424	0.492562	0.485489	0.556916
P48960	Adhesion G	ADGRE5 CI	-0.43284	-0.39433	-0.21029	-0.38061	-0.46351	-0.06055	-0.23999	-0.35593	-0.54492	-0.72457
P49005	DNA polym	POLD2	-0.44769	-0.45561	-0.87443	-0.49516	-0.70515	-0.39211	-0.75381	-0.36739	-0.52066	-0.60456
P49006	MARCKS-re	MARCKSL1	-0.77588	-0.61252	-0.58887	-0.49676	-0.58869	-0.30968	-0.61599	-0.46949	-0.06756	0.098355

P49023	Paxillin	PXN	-0.15014	-0.21019	-0.06916	0.101338	0.354272	0.047959	0.060712	-0.06417	0.140984	0.012889
P49116	Nuclear rec	NR2C2 TAK	-0.63337	-0.14446	0.001915	-0.22227	0.110561	0.193029	-0.01263	0.076982	0.164622	0.170646
P49137	MAP kinase	MAPKAPK2	-0.03063	0.175978	-0.16069	-0.4361	-0.22487	-0.42968	-0.05209	-0.13808	-0.5639	-0.19898
P49189	4-trimethyl	ALDH9A1 A	0.568319	0.659403	0.159952	0.365148	0.294905	0.574239	0.466854	0.389042	0.41727	0.069069
P49207	60S ribosor	RPL34	0.668692	0.688452	0.516621	0.50357	0.520697	0.664301	0.937235	0.757235	0.532156	0.79593
P49257	Protein ER(	LMAN1 ER(	0.848784	0.624209	0.13282	0.395323	-0.26017	0.317123	0.441088	0.310017	-0.49585	-0.5867
P49321	Nuclear au	NASP	0.250075	0.330278	0.52661	0.367478	0.45594	0.646675	0.074768	0.419325	0.959668	0.714306
P49327	Fatty acid s	FASN FAS	-3.37126	-1.83094	-2.02359	-3.14505	-2.42999	-2.87447	-2.01027	-1.86876	-3.39941	-1.85688
P49368	T-complex	CCT3 CCTG	0.263033	0.440162	0.520897	0.775353	0.641412	0.655541	0.740032	0.581141	0.69963	0.820221
P49407	Beta-arrest	ARRB1 ARR	-1.26849	-1.06178	-1.35543	-1.2478	-0.98697	-1.22772	-1.26669	-0.9593	-0.78974	-0.80038
P49411	Elongation	TUFM	0.390584	0.618366	0.810693	0.713271	0.768909	0.954893	1.013266	0.935609	1.033691	0.594837
P49441	Inositol pol	INPP1	-0.54912	-0.59071	-0.91792	-0.89635	-0.89648	-0.92986	-0.76948	-0.76332	-0.81364	-0.58471
P49454	Centromer	CENPF	0.671236	0.869498	0.502042	0.368829	0.935363	0.659509	0.574694	0.684498	0.699714	0.846029
P49458	Signal reco	SRP9	1.238335	1.324795	0.841902	0.921996	1.203752	1.035798	0.858263	1.100692	1.172183	1.210777
P49585	Choline-ph	PCYT1A CT	-0.15335	-0.17563	-0.152	-0.39704	-0.31451	-0.47454	-0.15545	-0.26523	-0.20021	-0.36116
P49588	Alanine--tR	AARS1 AAR	0.272123	0.298341	0.299396	0.247267	0.057662	0.348073	0.286011	0.122901	0.320041	0.294529
P49589	Cysteine--t	CARS1 CAR	0.070967	0.498167	0.930929	0.682988	0.864198	0.496743	0.757514	0.717569	0.903928	0.943654
P49591	Serine--tRN	SARS1 SARS	0.756234	0.93826	0.804009	0.855891	0.764021	0.369234	0.854802	0.726624	0.922521	0.70112
P49593	Protein pho	PPM1F KIA	-0.39497	-0.61252	-0.38672	-0.64582	-0.84503	-0.60145	-0.7217	-0.64636	-0.80618	0.166972
P49662	Caspase-4 (	CASP4 ICH2	-0.42569	-0.17238	-0.63336	-0.5012	-0.7296	-0.56433	-0.63564	-0.56256	-0.75551	-0.68807
P49711	Transcriptio	CTCF	0.601037	0.732403	0.256502	0.211054	0.309552	0.296211	0.3355	-0.13809	0.00998	0.101016
P49720	Proteasom	PSMB3	-0.25096	-0.31706	-1.45457	-1.05107	-1.69302	-1.1989	-1.15087	-1.18286	-1.15936	-0.93088
P49721	Proteasom	PSMB2	0.012073	0.141044	-0.16506	-0.52356	-0.71727	0.313158	-0.11165	0.192645	-0.24058	-0.30981
P49736	DNA replica	MCM2 BM	-0.35192	-0.35762	-0.5427	-0.37788	-1.0721	-0.93587	-0.69661	-1.04654	-0.82276	-0.66925
P49748	Very long-c	ACADVL VL	-1.10953	-1.20046	-0.63051	-0.65567	-1.04532	-0.73151	-0.83577	-0.77761	-0.83954	-0.95641
P49750	YLP motif-c	YLPM1 C14	-0.5285	-0.64264	-0.12199	-0.72655	0	-0.32329	-0.4957	-0.5764	-0.20211	-0.34873
P49755	Transmeml	TMED10 TM	0.654877	0.759616	0.099602	0.495472	0.20045	0.360503	0.507889	0.304825	-0.27995	-0.25387
P49756	RNA-bindir	RBM25 RN	0.280108	0.165297	0.515742	0.555389	0.549834	0.791656	0.730813	0.82362	0.699714	0.911015
P49757	Protein nur	NUMB C14	-1.70499	-1.33171	-1.5656	-1.75803	-2.26105	-2.51472	-1.68012	-1.53434	-2.63402	-2.89566
P49770	Translation	EIF2B2 EIF2	-4.30986	-2.60064	NA	NA	NA	NA	-3.01027	-2.80735	-2.06756	-4.1008
P49773	Histidine tr	HINT1 HINT	0.595782	0.528464	1.348202	0.777152	1.029444	1.153365	1.263793	1.268273	1.467126	1.927366
P49790	Nuclear po	NUP153	-0.14662	-0.25061	-0.10093	-0.40255	-0.21621	-0.12553	-0.31937	-0.25618	0.037233	-0.24544
P49792	E3 SUMO-p	RANBP2 NU	-0.55566	-0.42029	-0.4447	-0.79021	-0.48157	-0.4565	-0.52507	-0.27691	-0.44398	-0.48574

P49796	Regulator c	RGS3	-1.32496	-1.35147	-1.97679	-2.11757	-1.51245	-2.0902	-1.45305	-1.29078	-1.88235	-2.11991
P49815	Tuberin (Tu	TSC2 TSC4	-0.71491	-0.94642	-0.62449	-0.8011	-1	-0.50524	-0.46007	-0.52607	-0.65006	-0.66223
P49821	NADH dehy	NDUFV1 UQ	0.20847	-0.151	-0.07195	-0.52356	-0.87935	-0.35523	0.235488	-0.27408	0.014075	-0.14906
P49841	Glycogen sy	GSK3B	-1.09719	-0.76545	-1.55029	-0.83037	-0.92086	-1.15474	-1.05702	-0.94491	-0.87224	-1.28356
P49848	Transcriptic	TAF6 TAF2I	-0.31848	-0.49765	-0.62867	-0.62831	-0.45635	-0.56092	-0.26634	-0.20458	0.087983	-0.28145
P49863	Granzyme	GZMK TRYP	0.37002	-0.06327	0.225243	0.176536	0.494411	2.017212	1.220066	1.158335	-2.24154	-1.87747
P49903	Selenide, w	SEPHS1 SEL	0.886542	0.980371	1.170791	1.10254	1.117764	1.090032	1.005107	1.115477	1.479014	1.31927
P49915	GMP synth	GMPS	-0.86139	0.401202	-0.53138	-0.01711	-0.21591	-0.28231	-0.24473	-0.24936	-0.00208	-0.17218
P49916	DNA ligase	LIG3	-1.25168	-1.08303	-1.14768	-0.71584	-1.71727	-1.83494	-0.73106	-0.73888	-1.4245	-1.41799
P49959	Double-stra	MRE11 HN	-0.31739	-0.07195	-0.14336	-0.10402	-0.05376	0.23317	0.079617	-0.1571	0.164677	-0.15397
P50148	Guanine nu	GNAQ GAQ	0.088176	0.465798	0.220163	0.209792	0.657475	0.415037	0.295077	0.152003	0.104556	0.232472
P50213	Isocitrate d	IDH3A	-0.49647	-0.34672	0.266247	0.128876	0.245577	0.23173	0.015739	0.172467	0.121991	0.0649
P50219	Motor neu	MNX1 HLXB	-4.19438	-3.77649	-3.36146	-2.98751	-3.51245	-3.28951	-3.43911	-3.51785	-3.79796	-2.2402
P50238	Cysteine-ri	CRIP1 CRIP	1.021062	1.027039	1.70941	1.444529	1.348454	1.208271	0.992306	1.215013	0.843274	0.194304
P50395	Rab GDP di	GDI2 RABG	-0.02981	0.021576	-0.03154	0.06735	-0.07635	-0.37534	-0.0897	0.157146	0.1533	0.319478
P50402	Emerin	EMD EDMD	-0.14357	-0.12894	-0.77608	-1.21613	-1.29659	-1.05571	-0.70072	-0.88982	-1.14886	-1.00551
P50416	Carnitine O	CPT1A CPT	-0.409	-0.37716	-0.03909	-0.68562	-0.72955	-0.56816	-0.33166	-0.95491	-1.57198	-0.97369
P50453	Serpin B9 (	SERPINB9 P	0.51333	0.523901	-0.10512	0.051704	0.004204	0.115477	0.286713	0.255639	0.372314	0.200591
P50502	Hsc70-inter	ST13 AAG2	0.333833	0.400775	0.520395	0.347246	0.466324	0.380363	0.293677	0.414559	0.718177	1.113717
P50552	Vasodilator	VASP	-0.31827	-0.26096	0.114977	0.225559	-0.22487	-0.03877	0.136573	0.023094	0.007291	-0.11991
P50570	Dynamin-2	DNM2 DYN	0.254929	0.203328	0.01165	0.198904	-0.27956	0.194169	0.195728	-0.19652	-0.67111	-0.39428
P50579	Methionine	METAP2 M	-0.49394	-0.48153	-0.77438	-0.48794	-0.32499	-0.36491	-0.38466	-0.34792	-0.03339	-0.15843
P50747	Biotin--pro	HLCS	-3.19438	-2.57719	-2.46529	-3.59756	-3.27798	-3.28951	-3.55459	-2.30485	-3.79796	-3.26127
P50749	Ras associa	RASSF2 CEI	-0.76553	-0.32894	-0.21464	-0.43903	-0.18406	-0.24822	-0.34016	-0.06791	-0.39302	-0.53647
P50750	Cyclin-depe	CDK9 CDC2	-1.91265	-1.37441	-1.49297	-1.72569	-1.83179	-1.77627	-1.37918	-1.989	-1.49499	-1.6028
P50851	Lipopolysac	LRBA BGL C	-0.36515	-0.24866	-0.4047	-0.40255	-0.37113	-0.48525	-0.3173	-0.40376	-0.52346	-0.35271
P50897	Palmitoyl-p	PPT1 CLN1	-1.08551	-1.46425	-0.70514	-0.65797	-1.91675	-1.84824	-0.78297	-0.81829	-1.70182	-0.98074
P50914	60S ribosor	RPL14	1.934905	1.778095	1.32074	1.046719	1.716045	1.388291	1.944811	1.711421	0.873235	1.278806
P50990	T-complex	CCT8 C21or	0.448796	0.547012	0.495892	0.468067	0.554735	0.655496	0.578056	0.624551	0.820611	0.919223
P50991	T-complex	CCT4 CCTD	0.537227	0.710275	0.422815	0.558288	0.633707	0.470681	0.467779	0.559168	0.67925	0.741513
P50995	Annexin A1	ANXA11 AN	-0.08545	-0.05942	-0.11161	-0.17763	0.370175	0.130195	0.147905	0.297798	0.228448	0.30063
P51003	Poly(A) pol	PAPOLA PA	-1.77801	-1.17431	-1.79187	-2.0018	-1.30333	-1.65577	-1.02759	-1.96644	-1.50238	-0.89547
P51114	Fragile X m	FXR1	-1.11346	-1.03953	-0.41277	-0.15673	-0.32445	-0.42351	-0.07893	-0.25671	-0.55148	-0.49081

P51116	Fragile X m	FXR2 FMR1	-0.08104	-0.42286	-0.41244	-0.53261	-0.89734	-0.49576	-0.44606	-0.51785	-0.49584	-0.40395
P51124	Granzyme	GZMM MET	0.95425	0.980217	-0.74246	-0.70279	-0.83058	-1.08935	0.132002	-0.45259	-3.1055	-3.305
P51148	Ras-relatec	RAB5C RAB	-0.25096	-0.04757	-0.80346	-1.07731	-1.41178	-0.52083	-0.77323	-0.62855	-1.75125	-1.96546
P51149	Ras-relatec	RAB7A RAB	1.899862	1.997688	1.302632	1.088966	1.331236	1.207754	1.419754	1.209306	0.929956	0.953946
P51151	Ras-relatec	RAB9A RAB	-0.13327	-0.35147	-0.66102	-0.40255	-0.81855	-0.91511	-0.87276	-0.81741	-0.71049	-0.65524
P51159	Ras-relatec	RAB27A RA	0.073777	-0.27898	-0.48132	-0.39456	-0.29158	-0.47606	-0.30561	-0.35332	-0.7448	-0.67693
P51178	1-phosphat	PLCD1	-2.49518	-2.65519	-1.88436	-2.9034	-2.76724	-1.8397	-2.1777	-2.58007	-2.87164	-2.20127
P51398	28S ribosor	DAP3 MRPS	-2.80735	-2.00782	-2.34156	-4.14505	-3.59991	-2.87447	-3.61599	-3.39232	-2.1281	-3.44184
P51531	Probable gl	SMARCA2 I	-1.25096	-1.1305	-0.87317	-1.05107	-1.31451	-1.06711	-0.6477	-0.93289	-1.1281	-0.56083
P51532	Transcripti	SMARCA4 I	-1.46855	-1.5656	-1.41244	-1.47045	-1.71727	-1.07861	-1.19704	-1.93289	-0.59205	-1.66223
P51553	Isocitrate d	IDH3G	-0.93129	-0.37186	-1.06642	-1.29916	-0.8671	-0.25766	-1.18023	-1.19322	-1.44537	-1.24848
P51570	Galactokin	GALK1 GAL	-0.54906	-0.29061	-0.4855	-0.21925	-0.61455	-0.39051	-0.41536	-0.47543	-0.50197	-0.53042
P51571	Translocon	SSR4 TRAPI	0.012073	-0.2185	-0.85896	-0.5787	-0.68105	-0.78701	-0.46007	0.177538	-1.30319	-0.6763
P51572	B-cell recep	BCAP31 BA	0.904844	0.483146	0.601307	0.559651	0.519028	0.51755	0.494528	0.481639	-0.08545	-0.21945
P51580	Thiopurine	TPMT	-1.06193	-1.4761	-1.19153	-1.11757	-1.74193	-1.50524	-1.43911	-1.65535	-1.76553	-1.95641
P51608	Methyl-CpG	MECP2	0.545249	0.371884	0.848306	0.654742	1.14931	0.961132	0.966203	0.994997	0.794679	0.661735
P51610	Host cell fa	HCFC1 HCF	-0.28199	-0.24398	0.148853	0.081311	0.161638	-0.25649	0.092722	0.236638	0.19901	0.376942
P51648	Aldehyde d	ALDH3A2 A	-1.3913	-1.03063	-1.16028	-1.48153	-1.77798	-0.92032	-1.37612	-1.61332	-1.40999	-1.36296
P51649	Succinate-s	ALDH5A1 S	0.525676	0.662164	0.770113	0.62818	0.539639	0.700279	0.811722	0.822222	1.525342	1.291034
P51659	Peroxisoma	HSD17B4 E	-0.50712	-0.55666	-0.53723	-0.67727	-0.58226	-0.25326	-0.76305	-0.53627	-0.4184	-0.47996
P51665	26S protea	PSMD7 MC	-0.21632	-0.1699	-0.27051	-0.31079	-0.46214	-0.03695	-0.46394	-0.24348	-0.22196	-0.16616
P51668	Ubiquitin-c	UBE2D1 SF	0.866733	1.141044	0.338983	0.314381	-0.07635	0.479168	0.368243	0.094182	0.308666	0.143125
P51692	Signal trans	STAT5B	1.292534	1.240108	1.357362	1.371256	1.31895	1.433364	1.478834	1.432111	1.199993	1.273324
P51808	Dynein ligh	DYNLT3 TC	0.440573	0.668291	0.133954	0.143195	-0.01495	-0.10776	-0.15656	0.115477	0.15624	0.213514
P51809	Vesicle-ass	VAMP7 SYB	0.540978	0.663594	0.468007	0.170361	0.638175	0.512375	0.376116	0.40601	0.53477	0.261088
P51812	Ribosomal	RPS6KA3 IS	1.091024	1.223506	0.704633	0.879024	0.739937	0.736966	0.820451	0.146841	0.480704	0.503021
P51858	Hepatoma-	HDGF HMG	0.221087	0.26628	0.416986	0.576575	0.726517	0.286523	0.360295	0.39776	0.699714	0.685291
P51948	CDK-activa	MNAT1 CA	-0.41908	-0.14336	0.291986	0.268252	0.086588	0.140481	-0.05209	-0.38482	-0.06756	0.026311
P51970	NADH dehy	NDUFA8	1.195295	0.950418	0.615823	1.108706	0.258068	1.671568	1.292991	1.395585	0.714156	0.127005
P51991	Heterogen	HNRNPA3 I	0.570709	0.880905	0.577027	0.07214	0.573136	0.326424	0.657612	0.418207	0.685381	0.759166
P52209	6-phospho	PGD PGDH	-0.4224	0.045434	-0.57719	-0.66045	-0.36591	-0.08331	-0.44606	-0.37683	-0.08246	0.070431
P52272	Heterogen	HNRNPM H	0.184056	0.395225	0.483613	0.165965	0.513922	0.259387	0.471473	0.53842	0.696296	0.670796
P52294	Importin su	KPNA1 RCH	-0.688	-0.57403	-1.29338	-1.52751	-1.43338	-1.50826	-1.49164	-1.40075	-1.07634	-1.21691

P52298	Nuclear ca	NCBP2 CBP	-1.00434	-0.58959	-0.18462	-0.43051	-0.18953	-0.38095	-0.23166	-0.37099	-0.33865	-0.21434
P52306	Rap1 GTPa	RAP1GDS1	0.53981	0.826171	0.737745	0.76184	0.365871	0.588601	0.588369	0.622633	0.329674	0.471508
P52333	Tyrosine-p	JAK3	0.155031	-0.17383	-0.43335	-0.26806	0.264273	-0.35091	0.127235	0.317341	-0.36257	-0.44786
P52434	DNA-direct	POLR2H	-0.40098	-0.23016	-0.96743	-0.5687	-0.61029	-0.48019	-0.505	-0.71558	-0.45126	-0.46203
P52564	Dual specif	MAP2K6 M	-1.28524	-1.14373	-0.47599	-0.71539	-0.85252	-0.70724	-0.70696	-0.91252	-0.78642	-0.61077
P52565	Rho GDP-d	ARHGDIA G	0.548126	0.366464	0.67754	0.22728	0.694708	0.105854	0.437553	0.358228	0.565979	0.727434
P52566	Rho GDP-d	ARHGDIB G	0.572039	0.38143	1.223852	1.025151	1.39332	1.126343	1.033162	1.240082	1.59847	1.49474
P52594	Arf-GAP do	AGFG1 HRE	0.319501	0.58756	0.723647	0.678072	0.614406	0.844349	0.776328	0.730079	0.652505	0.899199
P52597	Heterogen	HNRNPF HI	0.922166	1.210217	0.740082	0.365016	0.67155	0.402689	0.616359	0.592576	0.480226	0.425577
P52630	Signal tran	STAT2	-3.43539	-2.03953	-3.20945	-1.84549	-3.59991	-1.83494	-2.07346	-3.45372	-3.93546	-2.00888
P52701	DNA mism	MSH6 GTBI	-0.14789	0.084379	-0.01027	0.284942	0.176182	0.280487	-0.18221	-0.11328	-0.07184	-0.13581
P52739	Zinc finger	ZNF131	0.053549	0.493758	0.399356	0.925999	0.422455	0.259387	0.290901	0.045088	0.028014	0.284267
P52747	Zinc finger	ZNF143 SBI	-0.90345	-0.88007	-0.93017	-0.93363	-1.31451	-0.78286	-1.28232	-0.89275	-0.89577	-1.03331
P52756	RNA-bindi	RBM5 H37	-0.36317	-0.51216	0.096514	-0.05221	0.133212	-0.21251	-0.10465	-0.20444	0.109264	0.281178
P52788	Spermine s	SMS	0.588998	0.618366	0.490986	0.378512	0.676212	0.377671	0.31166	0.626346	0.185556	0.429349
P52815	39S ribosor	MRPL12 M	1.015923	1.250944	1.511892	1.504133	1.26772	1.292081	1.370264	1.508082	1.646294	1.419515
P52824	Diacylglyce	DGKQ DAG	0.488887	0.646038	0.269518	-0.13813	0.28883	-0.24349	0.108376	-0.29078	-0.39941	-0.25597
P52888	Thimet olig	THOP1	-0.21534	-0.03953	-0.48699	-0.67557	-0.62263	-0.32329	-0.5175	-0.4459	-0.14365	-0.04495
P52907	F-actin-cap	CAPZA1	0.03823	-0.10512	0.362426	0.631654	0.437398	0.356498	0.356049	0.46305	0.324958	0.360346
P52943	Cysteine-ri	CRIP2 CRP2	-0.41193	-0.76326	-2.60064	-1.27563	-1.06075	-1.14958	-0.4636	-0.41123	-1.74959	-1.01781
P52948	Nuclear po	NUP98 ADA	-0.07464	0	0.102698	0.352043	0.107892	0.254858	-0.02382	-0.04881	0.210218	0.42432
P53004	Biliverdin r	BLVRA BLV	1.135235	1.009421	0.959525	0.98349	0.662389	0.571844	1.222066	1.142186	0.78485	0.801971
P53007	Tricarboxyl	SLC25A1 SL	1.676935	1.688997	1.393431	1.061401	0.757639	1.138	1.633588	0.666576	0.995278	0.759796
P53041	Serine/thre	PPP5C PPP	-0.33258	-0.81714	-0.37656	-0.17306	-0.39158	-0.37906	-0.25691	-0.21573	-0.3932	-0.54783
P53384	Cytosolic F	NUBP1 NBI	-1.40798	-1.65315	-0.82775	-0.76751	-1.68046	-1.07542	-0.94648	-1.41897	-0.89509	-0.84627
P53396	ATP-citrate	ACLY	0.488887	0.182864	1.086401	0.833592	0.510352	0.300782	0.953791	0.921047	0.690054	0.724516
P53567	CCAAT/enh	CEBPG	-3.89482	-2.03953	-1.20945	-1.10402	-0.89947	-1.26303	-1.60039	-1.43812	-1.31487	-1.36004
P53582	Methionine	METAP1 KI	-0.03423	0.371836	0.466458	0.813711	0.648358	0.607407	0.432798	0.309766	0.601446	0.528393
P53597	Succinate--	SUCLG1	1.835146	1.737189	2.201043	2.177488	2.153841	2.355945	2.163436	2.095413	2.574469	2.339061
P53602	Diphospho	MVD MPD	-0.28749	-0.44392	-0.5427	-1.07731	-0.97056	-0.88452	-0.70492	-0.64636	-1.05772	-0.89752
P53611	Geranylger	RABGGTB (	-1.29017	-1.63661	-1.39237	-1.59769	-2.03656	-1.03321	-1.75608	-1.87012	-1.2718	-1.8007
P53618	Coatomer s	COPB1 COF	0.878264	0.947775	0.743773	0.637105	0.439193	0.311513	0.715263	0.259783	0.307426	0.558706
P53621	Coatomer s	COPA	0.611953	0.515144	0.753974	0.529549	0.543763	0.613459	0.521395	0.526546	0.449971	0.416566

P53634	Dipeptidyl	CTSC CPPI	1.003028	1.21177	-0.03154	0.476438	0.319381	0.56308	0.78229	0.367016	-0.70498	-0.57521
P53803	DNA-direct	POLR2K	-1.23245	-1.627	-0.92301	-1.73591	-1.43041	-0.82195	-1.31745	-1.56242	-0.80001	-0.77181
P53990	IST1 homol	IST1 KIAA0	1.100941	1.128374	1.00943	0.868755	1.06447	0.839633	1.049717	1.048033	1.114784	1.127496
P53992	Protein tra	SEC24C KIA	-0.67682	-0.65019	-0.3225	-0.44641	-0.3448	-0.59794	-0.39434	-0.39136	-0.53243	-0.49399
P53999	Activated F	SUB1 PC4 F	1.352193	1.250323	0.974997	0.820671	0.889529	0.630938	0.843397	0.381325	1.187627	0.926056
P54098	DNA polym	POLG MDP	-0.59104	-0.36647	-0.07605	0.143195	-0.83173	-0.71348	-0.68834	-1.22239	-0.89107	-0.74882
P54105	Methylosor	CLNS1A CL(	-0.95964	-0.79048	-1.04136	-1.1841	-1.1525	-1.16852	-0.87746	-1.17814	-0.64292	-0.52601
P54136	Arginine--t	RARS1 RAR	0.098637	0.140311	0.370577	0.134963	0.251836	0.398549	0.125743	0.317341	0.317421	0.320157
P54252	Ataxin-3 (E	ATXN3 ATX	-1.44708	-1.73096	-0.81297	-1.47314	-0.98101	-1.47497	-1.35544	-1.22003	-0.76931	-0.85618
P54577	Tyrosine--t	YARS1 YAR	1.262529	1.0925	1.248338	1.027954	1.083082	1.024384	1.26559	1.241889	1.141356	0.877684
P54578	Ubiquitin c	USP14 TGT	0.405319	0.421888	-0.31498	-0.10259	-0.04014	0.113059	0.022283	-0.19881	-0.32134	-0.06333
P54619	5'-AMP-act	PRKAG1	1.390295	1.274556	0.391498	0.295104	0.525708	0.73685	0.70441	0.32723	0.271106	0.00877
P54709	Sodium/po	ATP1B3	0.59702	0.256763	0.006126	0.161893	-0.82324	0.35058	0.367049	0.321592	-0.44881	-0.64696
P54725	UV excisior	RAD23A	-0.99114	-0.7307	-1.43335	-0.89129	-2.04696	-2.15371	-1.12793	-1.72761	-1.68825	-1.21596
P54727	UV excisior	RAD23B	0.155698	0.036022	0.692303	0.528959	1.018711	0.943935	1.352441	1.033497	0.379315	0.192379
P54819	Adenylate I	AK2 ADK2	0.529335	0.471434	0.124599	0.223266	0.51172	0.465684	0.511291	0.304698	0.648901	-0.11072
P54886	Delta-1-pyr	ALDH18A1	-0.71849	-0.74204	-0.05554	-0.32998	-0.46053	-0.28617	-0.48273	-0.41603	-0.57994	-0.48832
P54920	Alpha-solul	NAPA SNAF	0.028318	0.029805	-0.31812	-0.17275	-0.44434	-0.0137	-0.39747	-0.05684	-0.0076	0.017428
P55010	Eukaryotic	EIF5	-0.17372	-0.16944	-0.07195	0.055377	-0.01495	-0.28284	-0.25691	-0.07641	-0.38702	0.198166
P55036	26S protea	PSMD4 MC	0.741807	0.921242	0.42862	0.439912	0.036712	0.160177	0.344264	0.375867	0.393664	0.599639
P55039	Developme	DRG2	0.678829	0.786031	0.625965	0.559651	0.594844	0.394398	0.543443	0.534473	0.285644	0.320157
P55060	Exportin-2	CSE1L CAS	0.832139	0.745922	0.327109	0.231622	0.219913	0.133909	0.420319	-0.02477	0.039716	0.253147
P55072	Transitiona	VCP	0.035907	0.147046	0.115412	0.005646	-0.04532	-0.12464	0.114098	-0.00795	0.225717	0.218064
P55081	Microfibrill	MFAP1	-1.48568	-1.3814	-1.32282	-0.9926	-1.09021	-0.89669	-1.19516	-0.88322	-0.91024	-0.84751
P55084	Trifunction	HADHB MS	0.868653	0.811395	0.708451	0.806525	0.431773	0.533944	0.735017	0.626346	0.590077	0.34127
P55145	Mesenceph	MANF ARM	-0.21594	-0.19415	0.293008	0.25996	-0.40759	0.136792	-0.26219	-0.04959	-0.25763	-0.40517
P55160	Nck-associa	NCKAP1L H	0.560511	0.717244	-0.06528	0.046902	-0.05509	0.020681	0.019658	-0.21211	0.015682	0.070214
P55196	Afadin (ALL	AFDN AF6 I	-3.89482	-2.24598	-1.97679	-1.61667	-1.66918	-1.60145	-2.28157	-1.80735	-2.39941	-2.00888
P55198	Protein AF-	MLLT6 AF1	-0.80735	-0.79667	-1.13906	-2.0126	-0.68105	-1.30293	-1.46713	-1.18286	-1.07748	-1.27191
P55199	RNA polym	ELL C19orf1	-1.56563	-1.59841	-2.05147	-1.58906	-1.58355	-1.25718	-1.34795	-1.44607	-1.95751	-1.58277
P55201	Peregrin (B	BRPF1 BR14	-0.818	-1.07195	-1.80346	-2.03814	-1.12418	-1.27621	-1.35819	-1.53434	-1.32665	-2.26127
P55209	Nucleosom	NAP1L1 NR	-0.22302	-0.03522	0.353884	-0.11598	0.351979	0.425086	0.09211	0.21486	0.514142	0.190502
P55210	Caspase-7 (	CASP7 MCI	-0.15398	-0.41069	0.10029	0.015433	-0.45192	-0.4104	-0.18893	-0.16542	-0.65903	-0.97857

P55263	Adenosine	ADK	0.409613	0.424861	0.786031	0.739472	1.079567	0.905476	1.041636	1.071874	0.73646	0.455133
P55265	Double-stra	ADAR ADAI	-0.19438	-0.03154	0.023207	-0.12439	0.160396	0.120434	0.159657	0.088809	0.202044	0.450553
P55290	Cadherin-1	CDH13 CDH	0.953805	1.384498	0.96647	1.128925	1.168271	1.058894	0.622	1.099536	0.664453	0.445688
P55327	Tumor prot	TPD52	-1.02445	-1.30256	-1.04757	-1.3377	-0.63876	-0.57401	-0.7161	-0.66146	-0.72992	-0.84884
P55735	Protein SEC	SEC13 D3S1	-0.42969	-0.52032	-1.00391	-0.81908	-0.98859	-0.76042	-0.5549	-0.8819	-1.05858	-1.04469
P55769	NHP2-like p	SNU13 NHP	-0.12003	0.269518	-0.19599	0.043253	0.200987	-0.0614	0.050273	-0.02308	0.147754	0.194304
P55786	Puromycin-	NPEPPS PSA	0.160465	0.23349	-0.20722	-0.00685	0.02457	0.142967	0.169196	0.045088	0.189695	0.319582
P55789	FAD-linked	GFER ALR H	-0.67588	-0.05971	0.304669	0.309328	0.003671	-0.03317	-0.03929	-0.21671	0.254365	0.365517
P55795	Heterogene	HNRNPH2 I	-0.70499	-0.61252	-0.87168	-0.79126	-0.85124	-0.81862	-0.99589	-0.65651	-0.62399	-0.88939
P55809	Succinyl-Co	OXCT1 OXC	1.239609	1.20488	1.153367	1.16853	0.964356	1.255795	1.156341	1.283654	1.54653	1.148552
P55854	Small ubiqu	SUMO3 SM	1.966269	2.136617	1.299926	1.453718	1.669214	1.299902	1.590069	1.775937	1.96782	1.81869
P55884	Eukaryotic	EIF3B EIF3S	-1.11346	-0.41764	-0.0843	-0.13813	-0.00746	-0.26303	-0.44606	-0.22239	-0.10257	-0.12956
P56182	Ribosomal	RRP1 D21S	-0.94062	-1.15634	-1.06378	-0.90298	-0.79256	-0.9783	-0.96963	-1.2091	-1.57089	-1.2402
P56192	Methionine	MARS1 MA	0.413062	0.467677	0.105243	0.073373	0.121186	0.356485	0.574694	0.110183	0.301033	0.135088
P56211	cAMP-regu	ARPP19	0.488887	0.440162	1.894883	1.705257	1.666874	1.975197	1.715317	1.860348	1.981017	2.042515
P56270	Myc-associ	MAZ ZNF80	0.110807	0.595323	1.27275	1.102877	-0.54463	0.160177	1.241992	1.128629	0.584963	0.03065
P56377	AP-1 comp	AP1S2 DC2	-0.01524	0.057456	0.111164	0.068912	0.097932	0.120398	-0.15467	0.156632	-0.24184	0.029553
P56378	ATP syntha	ATP5MPL C	0.246256	0.022794	-0.68562	-0.359	-1.28115	0.424431	0.045571	0.061044	-1.46005	-1.14776
P56385	ATP syntha	ATP5ME AT	0.989243	0.785965	0.503771	0.356948	0.205803	0.869908	0.591511	0.343595	0.148087	-0.27062
P56537	Eukaryotic	EIF6 EIF3A	-0.51911	-0.44659	-0.70367	-0.9242	-1.1914	-0.98205	-0.94758	-0.64671	-0.56459	-0.40587
P56556	NADH dehy	NDUFA6 LY	2.338802	2.275973	1.285602	2.083768	1.757639	2.361811	2.316803	2.226068	1.920032	1.067014
P56715	Oxygen-reg	RP1 ORP1	0.218924	1.142811	1.360402	0.809146	1.072512	0.431339	1.042843	0.534473	1.472233	2.059602
P57076	Cilia- and fl	CFAP298 C2	-1.5915	-1.71228	-2.31587	-1.99665	-3.10193	-2.67034	-2.88646	-2.57863	-1.34045	-1.24157
P57105	Synaptojan	SYNJ2BP OI	-2.07199	-1.44205	-1.97884	-3.37277	-2.69302	-2.63072	-2.24234	-2.30153	-2.24725	-2.41047
P57682	Krueppel-li	KLF3 BKLF	-2.15695	-2.56398	-2.19605	-1.95686	-3.07635	-2	-2.96963	-2.97728	-3.1281	-2.2402
P57721	Poly(rC)-bii	PCBP3 PCB	1.751963	1.731882	1.907995	1.830055	1.69213	1.805838	1.67509	1.621532	1.942818	1.773514
P57737	Coronin-7 (	CORO7	0.503912	0.44795	0.905542	0.818819	0.934161	1.011509	0.815934	0.851137	0.495234	0.450225
P57740	Nuclear po	NUP107	-0.61869	-0.75002	-1.20945	-1.36976	-1.12418	-1.48981	-1.10613	-1.27684	-1.25739	-1.1788
P57764	Gasdermin	GSDMD DF	0.568707	0.690206	0.641046	0.716752	0.748815	0.90105	0.384011	0.877588	0.449971	0.527789
P57772	Selenocyste	EEFSEC SEL	-0.90613	-0.82402	-0.95918	-0.82261	-1.1038	-0.74806	-0.63966	-1.02426	-0.98129	-0.96691
P58107	Epiplakin (4	EPPK1 EPIP	-0.63993	-0.41764	0.361919	0.131176	0.350497	0.693698	-0.61886	-0.12096	0.549234	0.368842
P58546	Myotrophi	MTPN	0.652724	0.716759	0.868802	0.52088	0.754057	0.206835	0.348033	0.18041	0.747447	0.996133
P59768	Guanine nu	GNG2	1.499645	1.522544	0.934185	0.95541	1.348454	1.138	1.411195	1.253941	1.147754	1.006623

P59998	Actin-relate	ARPC4 ARC	0.883555	0.976413	0.850224	0.801968	0.592728	0.489934	0.631938	0.789106	0.637542	0.909203
P60002	Transcripti	ELOF1	0.189969	0.295653	0.305522	0.052783	0.043059	0.311963	0.356115	0.369709	0.185073	0.180712
P60033	CD81 antig	CD81 TAPA	0.234465	0.387482	-0.5427	-0.24558	-1.06075	-0.42968	-0.39808	-0.43812	-0.93546	-1.04495
P60059	Protein tra	SEC61G	-1.3023	-1.24267	-1.13053	-0.17161	-0.75907	-0.44096	-0.95677	0.5048	-0.90309	-0.80707
P60174	Triosephos	TPI1 TPI	0.675647	0.941955	0.805799	0.968434	0.524273	1.258291	0.880757	0.709459	0.246936	0.417298
P60228	Eukaryotic	EIF3E EIF3S	0.839892	0.921889	1.005713	0.858414	0.503375	1.000409	0.661349	0.655715	0.540274	0.815592
P60468	Protein tra	SEC61B	0.471504	-0.2185	0.186295	0.966729	0.643261	0.481778	0.103623	1.589353	0.619129	0.116548
P60510	Serine/thre	PPP4C PPP	-2.79253	-2.33254	-2.84658	-2.49181	-3.07635	-3.28951	-2.71681	-3.06083	-2.19146	-2.90817
P60520	Gamma-an	GABARAPL	1.059379	0.998049	0.847997	1.009378	0.895942	0.975197	0.929227	0.688056	0.889817	0.86316
P60660	Myosin ligh	MYL6	-0.1617	0.172522	0.647807	0.319802	0.434539	0.409767	0.458633	0.370605	0.791302	0.948834
P60709	Actin, cyto	ACTB	0.067241	0.038474	-0.2276	0.220599	-0.23359	-0.26961	0.122543	-0.0115	-0.20211	0.073337
P60842	Eukaryotic	EIF4A1 DD	1.00028	1.030932	0.618366	0.609837	0.604658	0.656046	1.094291	0.622633	0.382857	0.235716
P60866	40S riboso	RPS20	0.849314	0.912816	1.320387	0.893282	0.882173	0.764756	1.240253	0.700378	1.395773	1.393591
P60891	Ribose-pho	PRPS1	0.66797	0.596029	0.623437	0.912733	0.409752	0.786415	1.149775	1.110183	1.119823	1.245596
P60900	Proteasom	PSMA6 PR	0.065184	0.030861	-0.49246	-0.27563	-0.46036	-0.40054	-0.13955	-0.22909	0.100164	0.066195
P60903	Protein S1	S100A10 A	0.78681	1.333235	1.823324	2.204045	1.447993	1.261387	1.920129	1.800717	0.330123	0.221871
P60953	Cell divisio	CDC42	1.316165	1.365465	0.855061	1.347462	0.68085	1.427473	1.155967	1.376689	0.266504	0.64726
P60981	Destrin (Ac	DSTN ACTD	0.389322	0.186231	0.825047	0.804073	0.855414	0.704092	0.57467	0.517356	0.539779	0.135128
P60983	Glia matur	GMFB	-0.28663	-0.30669	-0.34413	-0.36985	-0.41043	-0.31742	-0.55123	-0.70685	-0.48263	0.092283
P61006	Ras-relatec	RAB8A ME	-0.51111	-0.09675	0.393431	0.397684	0.141554	0.41913	0.426503	0.280108	0.15624	-0.18886
P61009	Signal pept	SPCS3 SPC	-0.0502	0.174242	-0.69079	-0.53192	-1.17511	-0.43583	-0.40658	-0.43887	-1.34055	-1.40037
P61011	Signal reco	SRP54	0.15874	0.275441	0.359692	0.039532	0.477773	0.428385	-0.07459	0.331059	0.141354	-0.06796
P61018	Ras-relatec	RAB4B PP1	1.073849	1.072245	0.384498	0.299168	0.022139	0.410933	0.418869	0.321928	0.028014	0.155098
P61019	Ras-relatec	RAB2A RAE	0.903903	0.902554	0.450603	0.554568	-0.07363	0.521112	0.428719	0.531929	-0.025	-0.05536
P61020	Ras-relatec	RAB5B	-1.71807	-1.49327	-1.40619	-2.59201	-1.82757	-2.11537	-1.90649	-1.7077	-1.58778	-1.5321
P61026	Ras-relatec	RAB10	0.672285	0.798975	0.725954	0.521812	0.567014	1.014417	1.036272	0.890531	0.23204	0.256489
P61081	NEDD8-cor	UBE2M UB	0.216032	0.170574	-0.14463	-0.19472	-0.18853	0.17008	0.068534	0.013615	0.135045	-0.04126
P61086	Ubiquitin-c	UBE2K HIP	0.31054	0.666072	0.134812	0.39766	0.214965	0.110388	0.19935	0.203623	0.343856	0.579746
P61106	Ras-relatec	RAB14	1.342289	1.254682	1.198095	1.147364	0.891718	1.079143	1.031586	0.946873	0.460297	0.58938
P61129	Zinc finger	ZC3H6 KIA	-1.72489	-1.64874	-1.93147	-1.40255	-3.35199	-3.04439	-1.42531	-1.30485	-2.32723	-2.28958
P61158	Actin-relate	ACTR3 ARP	0.686383	0.693039	0.285173	0.19066	-0.03138	0.193689	0.195667	0.406127	0.08815	0.198161
P61160	Actin-relate	ACTR2 ARP	0.760305	0.595323	0.638246	0.49436	0.481342	0.510195	0.57614	0.688056	0.530515	0.645625
P61163	Alpha-cent	ACTR1A CT	-1.46894	-0.9799	-0.21749	-0.03955	-0.37109	-0.58324	-0.35995	-0.18286	-0.74033	-0.42986

P61165	Transmeml	TMEM258	-1.23661	-1.58887	-1.6734	-0.41086	-1.41009	-1.18641	-1.68012	0.045088	-1.83112	-1.18886
P61201	COP9 signa	COPS2 CSN	0.012073	0.378434	-0.48699	-0.03814	0.147718	0.180857	-0.46713	-0.37737	-0.44361	0.170908
P61204	ADP-ribosy	ARF3	1.209514	1.885416	0.77468	0.520329	1.043943	0.447459	0.755266	0.67776	1.082462	0.968684
P61221	ATP-bindin	ABCE1 RLI I	-0.87245	-0.68589	0.351262	-0.05107	-0.34252	0.19878	-0.23868	0.045088	0.147754	0.509253
P61224	Ras-relatec	RAP1B OK/	1.726323	2.024124	-1.61797	-1.17094	0.852299	1.351136	-1.15649	-1.15829	-1.41411	-1.97327
P61225	Ras-relatec	RAP2B	0.47587	0.76094	-0.04757	0.102877	-0.17365	-0.635	0.164229	0.115477	-0.50919	-0.49075
P61244	Protein ma	MAX BHLH	-0.79775	-0.57719	-1.72403	-1.8011	-1.14929	-1.17728	-1.36893	-1.15083	-0.74169	-0.57965
P61247	40S ribosor	RPS3A FTE:	0.105996	0.348202	0.80637	0.462849	0.973734	0.792388	0.856874	0.546282	0.862411	0.598394
P61254	60S ribosor	RPL26	1.221526	1.394915	1.113508	0.822768	1.220194	1.24283	1.25156	1.201298	1.096539	1.151885
P61289	Proteasom	PSME3	-0.09392	0.108863	0.146083	0.098085	-0.269	-0.07861	-0.04679	-0.08854	0.13493	0.056413
P61313	60S ribosor	RPL15 EC4!	0.663603	0.808469	0.685364	0.324435	0.604658	0.016302	0.522227	-0.04654	0.494156	0.596716
P61326	Protein ma	MAGOH M	0.454697	0.537362	-0.30917	-0.09808	-0.36249	-0.05254	-0.3069	-0.27484	-0.12778	-0.31507
P61353	60S ribosor	RPL27	-0.28338	-0.52665	-0.02014	-0.31414	0.157516	0.083472	0.064756	-0.11676	0.028548	0.242679
P61421	V-type prot	ATP6V0D1	-1.80735	-1.5541	-1.69849	-1.54171	-1.53382	-1.30293	-2.16227	-1.43812	-1.95361	-1.20918
P61513	60S ribosor	RPL37A	1.200095	1.023731	0.826918	0.72075	1.200856	0.812648	0.898306	0.912503	0.713474	0.87775
P61586	Transformi	RHOA ARH	-0.28011	-0.07195	-0.44923	-0.152	-0.39046	-0.24998	-0.5697	-0.22239	-0.45003	-0.66223
P61604	10 kDa hea	HSPE1	1.456679	1.526079	1.908746	1.452976	1.67155	1.741876	1.469627	1.780943	2.003532	1.669985
P61619	Protein tra	SEC61A1 SI	-1.21302	-1.30836	-1.61776	-0.90594	-0.9466	-1.21668	-1.35498	-0.49818	-1.14985	-1.26447
P61758	Prefoldin s	VBP1 PFDN	-0.30367	-0.10165	-0.07717	-0.2369	0.139909	0.026411	-0.24105	0.130369	-0.01055	-0.01644
P61916	NPC intrace	NPC2 HE1	0.070967	-0.20495	1.369485	1.165737	1.291871	1.402689	1.405413	1.698177	0.932437	0.619939
P61923	Coatomer s	COPZ1 COF	-0.39497	-0.59474	0.477047	0.288936	0	0.514027	0.094068	0.126008	0.301033	0.455403
P61927	60S ribosor	RPL37	1.242173	1.184581	-1.12179	-1.28789	1.342602	0.819792	1.175598	1.175005	-1.34178	-1.56043
P61956	Small ubiqu	SUMO2 SM	1.837443	2.132674	1.514862	1.507434	1.600243	1.456135	1.468881	1.559861	1.665346	1.565232
P61962	DDB1- and	DCAF7 HAN	-2.44175	-1.74758	-1.85543	-1.33778	-2.19102	-2.1154	-2.21485	-2.0199	-2.22975	-2.42013
P61964	WD repeat	WDR5 BIG3	0.973608	1.012174	0.232422	0.674762	0.115313	0.840799	0.842393	0.377477	-0.10297	-0.16856
P61966	AP-1 comp	AP1S1 AP1	-1.12663	-1.41244	-1.5656	-1.50564	-3.51245	-3.18641	-1.9898	-1.65535	-2.06756	-1.20918
P61970	Nuclear tra	NUTF2 NTF	0.980536	0.905773	0.698177	0.893337	0.800564	0.814621	0.976348	0.920601	1.154763	0.975536
P61978	Heterogen	HNRNPK HI	0.934905	0.917767	0.931041	0.716752	0.931764	0.609962	1.00607	0.988724	1.104556	0.894446
P61981	14-3-3 prot	YWHAG	-0.18057	-0.01287	0.075778	0.25916	0.167527	0.091066	0.079788	0.375867	0.246317	0.333664
P62070	Ras-relatec	RRAS2 TC2	-1.30986	-1.32193	-0.70483	-0.62632	-0.77973	-0.89465	-0.82667	-1.80735	-1.68753	-1.63448
P62072	Mitochond	TIMM10 TI	0.008431	0.125643	0.113396	0.395649	0.476714	0.587733	-0.313	-0.69188	0.438903	0.376342
P62081	40S ribosor	RPS7	0.767661	0.782436	1.119069	0.990126	1.134529	1.271709	1.026021	1.100177	1.159995	1.013808
P62136	Serine/thre	PPP1CA PP	0.350372	0.536112	0.685984	0.640509	0.414378	0.550049	0.633453	0.642163	0.609253	0.232313

P62140	Serine/thre	PPP1CB	0.097667	0.326131	0.389506	0.503883	-0.32888	0.335237	0.290858	-0.13	-0.03837	0.292365
P62191	26S protea	PSMC1	0.236854	0.344701	0.363347	0.161978	0.116782	0.223729	0.091764	0.224601	0.447908	0.321918
P62195	26S protea	PSMC5 SU(	-0.2949	-0.25252	-0.31507	-0.13792	-0.19903	0.016302	-0.26039	-0.18286	-0.06756	0.205861
P62241	40S ribosor	RPS8 OK/S\	1.044943	0.929373	0.91465	0.554606	1.122045	1.213517	0.713423	0.888413	0.972052	0.838138
P62244	40S ribosor	RPS15A OK	1.027023	1.232085	1.206711	1.380922	1.210659	1.358618	1.553936	1.399931	1.275942	0.982189
P62249	40S ribosor	RPS16	1.247289	1.488209	0.399356	0.4899	0.405712	0.31755	0.332124	0.380272	0.230452	0.351712
P62258	14-3-3 prot	YWHAE	-0.46877	-0.71985	-0.55345	-0.42215	-0.41799	-0.24376	-0.44248	-0.74905	-0.79767	-0.40858
P62263	40S ribosor	RPS14 PRO	0.746032	0.688627	0.413723	0.195472	0.237264	0.146472	0.455274	0.078392	0.215616	0.60907
P62266	40S ribosor	RPS23	1.464549	1.456425	1.276534	1.027295	1.452745	1.367098	1.544934	1.356984	1.426482	1.246789
P62269	40S ribosor	RPS18 D6S:	0.501685	0.506948	0.819399	0.627643	1.179126	0.916523	1.099835	0.821994	0.857059	0.974841
P62273	40S ribosor	RPS29	0.74228	0.907656	0.361549	0.563876	0.52011	0.525936	0.603552	0.014339	0.653932	0.515793
P62277	40S ribosor	RPS13	1.662137	1.920648	1.159962	0.720564	1.497158	1.326334	1.521237	1.240615	0.89743	1.069593
P62280	40S ribosor	RPS11	0.969355	0.952074	0.551614	0.845586	1.005304	0.809009	0.663355	0.659304	0.869317	0.982262
P62304	Small nucle	SNRPE	0.770518	1.034673	0.220163	-0.18728	-0.34252	0.208271	-0.07346	0.056143	-0.11273	0.287896
P62306	Small nucle	SNRPF PBS(	-1.8892	-2.62209	-1.85896	-1.26053	-0.92749	-1.0902	-1.12832	-0.63743	-1.46297	-1.54137
P62308	Small nucle	SNRPG PBS	1.105182	1.032768	-0.52015	-0.32979	-0.33313	-0.01097	0.286713	0.083416	0.032631	-0.21945
P62310	U6 snRNA-a	LSM3 MDS(	-1.06193	-1.28345	-0.22304	-0.41086	-0.66918	-0.36491	-0.54709	-0.36257	0.164677	0.008824
P62312	U6 snRNA-a	LSM6	0.996966	0.90116	0.996096	1.15451	0.875821	1.07195	0.945257	0.956411	1.23246	1.186547
P62314	Small nucle	SNRPD1	-0.68966	-0.637	-0.46413	-0.57956	-0.83727	-0.85416	-1.03156	-0.59791	-0.59502	-0.68704
P62316	Small nucle	SNRPD2 SN	1.612977	1.532014	0.847563	0.823395	1.027006	0.736966	1.454773	1.411521	0.751041	0.634265
P62318	Small nucle	SNRPD3	-0.38125	-0.14545	-0.66028	-0.61554	-0.55552	-0.35091	-0.44075	-0.52607	-0.94923	-0.60726
P62328	Thymosin b	TMSB4X TE	0.518916	0.534472	1.054681	0.944418	0.901193	0.589049	0.727843	1.113296	0.944909	0.537874
P62330	ADP-ribosy	ARF6	0.782083	0.82887	0.332688	0.369341	0.164876	0.233349	0.16071	0.403148	-0.08565	-0.05241
P62333	26S protea	PSMC6 SU(	0.080073	0.125166	0.203185	0.278177	0.291842	0.547835	0.255317	0.362385	0.329392	0.097763
P62424	60S ribosor	RPL7A SUR	1.786304	1.501432	0.89219	0.723491	1.072512	1.012947	1.174365	1.018736	0.767752	0.753904
P62487	DNA-direct	POLR2G RP	-0.97232	-0.48396	-1.2276	-1.35364	-0.98595	-0.91338	-1.35819	-1.18286	-0.98126	-1.52855
P62495	Eukaryotic	ETF1 ERF1	0.224123	0.485427	-0.2576	-0.21652	-0.29798	0.044938	-0.20726	-0.36002	-0.20503	-0.11619
P62633	Cellular nu(	CNBP RNF1	0.245015	0.470284	0.099643	-0.17306	-0.05302	0.189226	0.143539	-0.65792	0.254365	0.135854
P62701	40S ribosor	RPS4X CCG	1.130888	1.316678	0.754021	0.731939	1.297428	1.022942	1.082036	0.761089	0.773279	0.887286
P62714	Serine/thre	PPP2CB	-1.17029	-1.10633	-0.71739	-0.79153	-0.65126	-0.71144	-0.83996	-1.0869	-0.67337	-0.66067
P62750	60S ribosor	RPL23A	1.377789	1.297797	1.688096	1.53172	1.772952	1.313158	1.51866	1.417183	1.547789	1.429807
P62753	40S ribosor	RPS6 OK/S\	1.083688	1.070389	1.030861	0.654356	1.033082	0.93002	1.045324	1.017074	0.902483	0.882493
P62805	Histone H4	H4C1 H4/A	2.006049	2.349245	0.019365	0.344335	0.251836	1.150363	0.93459	0.146841	-0.62076	-0.41914

P62820	Ras-relatec	RAB1A RAE	-1.34023	-1.07195	-0.90933	-0.10402	-1.53382	-1.04439	-1.08427	0.25634	-0.92647	-1.20918
P62826	GTP-bindin	RAN ARA24	0.941233	0.948399	0.582361	0.383329	0.919723	0.23173	0.631938	0.202629	0.685127	1.026311
P62829	60S ribosor	RPL23	1.467019	1.390459	1.202067	0.847315	1.513829	1.297685	1.254911	0.991387	1.456218	1.541536
P62834	Ras-relatec	RAP1A KRE	-1.11978	-0.76561	-2.20595	-1.78174	-0.8335	-1.58299	-0.88473	-0.79738	-1.77309	-1.75635
P62841	40S ribosor	RPS15 RIG	1.605028	1.406728	1.519827	0.885829	1.650386	1.52166	1.524007	1.561879	1.388271	1.224919
P62847	40S ribosor	RPS24	1.601436	1.663631	1.54672	0.937936	1.40099	1.124526	1.679604	1.079031	1.607865	1.798038
P62851	40S ribosor	RPS25	0.982117	0.930643	1.679642	1.702852	1.881754	1.351709	1.6474	1.479825	2.088845	2.199514
P62854	40S ribosor	RPS26	1.073849	1.11067	0.189718	-0.0126	0.584963	0.259387	0.543443	0.246722	0.407948	0.416143
P62857	40S ribosor	RPS28	-0.4765	-0.55364	-0.31464	0.055401	-0.03495	-0.35406	-0.16879	-0.12836	0.104323	0.192868
P62873	Guanine nu	GNB1	0.224123	0.437285	0.2824	0.471923	0.300954	0.548063	0.340229	0.61891	0.658492	0.163024
P62875	DNA-direct	POLR2L	1.590944	1.610981	1.503058	1.474322	1.735156	1.651865	1.699248	1.659436	1.713668	1.82793
P62877	E3 ubiquitii	RBX1 RNF7	0.285091	0.830562	0.641046	0.64235	0.923649	0.6386	0.702799	0.152003	1.183481	1.632839
P62879	Guanine nu	GNB2	0.13975	0.123836	-0.42948	-0.31358	-0.53499	-0.13256	-0.04804	-0.26451	-0.45594	-0.31671
P62888	60S ribosor	RPL30	0.080027	0.481374	0.078807	0.25458	0.126481	0.109784	0.3739	0.088769	-0.11545	0.058963
P62899	60S ribosor	RPL31	1.029995	1.269042	1.604473	1.225077	1.62399	1.77259	1.595873	1.490326	1.266124	1.281711
P62906	60S ribosor	RPL10A NE	1.27604	1.23619	0.622057	0.486054	0.935063	0.5366	0.686697	0.572547	0.8096	1.061478
P62910	60S ribosor	RPL32 PP9!	1.149576	0.828368	0.474036	0.471845	1.466176	1.508275	1.127235	1.421464	0.406217	0.631001
P62913	60S ribosor	RPL11	0.488524	0.797231	0.740735	1.041913	0.919723	0.791656	0.801472	0.86507	0.60005	0.73608
P62917	60S ribosor	RPL8	0.7663	0.77468	0.918731	0.820696	1.087336	1.153808	0.989145	0.824301	0.944504	1.210956
P62942	Peptidyl-pr	FKBP1A FKI	-1.25096	-1.34034	-1.95318	-1.36278	-1.26569	-1.64314	-1.04325	-1.31449	-1.17055	-1.5046
P62993	Growth fac	GRB2 ASH	0.381307	0.46014	0.116077	0.354182	0.422455	0.467359	0.261328	0.321928	0.304855	0.353514
P62995	Transforme	TRA2B SFR!	0.834547	0.744007	1.634782	1.289197	1.232977	1.150363	0.900967	1.100544	1.515751	1.648451
P63010	AP-2 comp	AP2B1 ADT	-0.90923	-1.05337	-0.83392	-0.84202	-0.65826	-0.83504	-0.7963	-0.95236	-0.87082	-0.89486
P63027	Vesicle-ass	VAMP2 SYE	-2.11036	-1.28658	-1.4018	-1.56151	-1.43839	-1.60921	-1.50616	-1.42106	-0.97225	-0.80173
P63092	Guanine nu	GNAS GNA!	0.178098	0.220072	0.407479	0.333921	0.504902	0.684218	0.403548	0.462671	0.3132	0.248457
P63098	Calcineurin	PPP3R1 CN	1.270059	1.290466	1.073631	1.051185	0.857163	1.350882	0.841236	0.771303	0.222907	0.374874
P63104	14-3-3 prot	YWHAZ	0.008437	0.0846	0.265989	0.263034	0.172645	0.445787	0.065807	0.141661	0.581244	0.471394
P63146	Ubiquitin-c	UBE2B RAD	-0.42351	-0.84897	-1.12562	-0.44312	-1.02061	-0.62795	-0.84519	-0.9413	-0.80244	-0.7525
P63151	Serine/thre	PPP2R2A	-1.04818	-1.21824	-1.27674	-1.68594	-1.19906	-1.36404	-1.68606	-1.38246	-1.69151	-1.79065
P63167	Dynein ligh	DYNLL1 DL	0.012073	-0.10932	2.207555	2.253858	2.197749	1.90105	2.158511	2.16355	1.788372	1.687445
P63173	60S ribosor	RPL38	0.950672	0.754021	0.648528	0.819846	1.276604	0.544321	1.198071	0.652077	0.667425	0.911015
P63208	S-phase kir	SKP1 EMC1	-0.37467	-0.49362	-0.30646	-0.35932	-0.38818	-0.43898	-0.42657	-0.80222	-0.09061	-0.16665
P63218	Guanine nu	GNG5 GNG	1.612977	1.782635	2.240933	2.020243	1.977516	1.568474	1.82479	1.882255	1.696808	1.392739

P63220	40S ribosor	RPS21	1.464793	1.467835	1.421645	0.859992	1.410932	1.229053	1.36603	1.349228	1.77373	1.572837
P63241	Eukaryotic	EIF5A	1.839892	1.742414	1.258152	1.196873	1.488875	1.00273	1.393778	1.031149	1.5099	1.417801
P63244	Receptor o	RACK1 GNE	0.135817	0.11967	-0.56927	-0.2738	-0.38528	0.273446	-0.11971	-0.19689	-0.45885	-0.23614
P63272	Transcripti	SUPT4H1 S	-2.47996	-1.52577	-1.95461	-2.68735	-2.52631	-1.70631	-2.2129	-2.40145	-1.26875	-1.92052
P63313	Thymosin b	TMSB10 PT	0.098814	0.291553	-0.01933	0.225045	0.2856	0.039001	0.057211	0.203957	0.584438	0.15269
P67775	Serine/thre	PPP2CA	-0.80986	-0.72472	-0.17832	-0.4386	-0.98708	-0.45631	-0.39645	-0.72079	-0.42985	-0.4636
P67809	Y-box-bindi	YBX1 NSEP	-0.73603	-0.56198	-1.1518	-1.05102	-0.9791	-1.47143	-1.40753	-1.13969	-0.88135	-0.6706
P67812	Signal pept	SEC11A SEC	-0.22264	-0.7047	1.250064	1.433444	0.548079	1.009388	1.726165	1.146939	0.489047	0.177312
P67870	Casein kina	CSNK2B CK	1.059379	0.923946	0.636037	0.9874	0.239291	0.92139	0.891116	0.419325	0.490805	0.455403
P67936	Tropomyos	TPM4	0.116409	0.295167	0.807953	0.88243	0.31895	0.289447	0.574694	0.29762	0.069042	0.429349
P68032	Actin, alpha	ACTC1 ACT	0.029946	0.236799	0.382724	0.236659	0.18799	0.205851	0.215596	0.217477	0.242458	0.392739
P68036	Ubiquitin-c	UBE2L3 UB	1.268031	1.370857	1.328463	1.432735	1.261174	1.116645	1.299811	1.427102	1.393825	1.311496
P68104	Elongation	EEF1A1 EEF	0.842983	1.040807	1.016948	1.14647	1.028984	1.202088	1.128007	1.112772	0.947683	1.064949
P68366	Tubulin alp	TUBA4A TU	0.338802	0.152155	1.226842	1.204358	0.946564	1.047041	0.926538	0.916091	0.863213	0.797523
P68371	Tubulin bet	TUBB4B TU	-1.55778	-2.11309	0.166965	0.101259	-0.14292	-0.13182	-0.08789	-0.10246	-0.27213	-0.09795
P68400	Casein kina	CSNK2A1 C	-0.20383	-0.1031	-1.05116	-1.05319	-1.34165	-0.6723	-1.20399	-0.94323	-0.74838	-0.86838
P68402	Platelet-act	PAFAH1B2	2.395201	2.60817	2.262222	2.236659	2.009266	2.279786	2.435042	2.306966	2.301989	2.353445
P68431	Histone H3	H3C1 H3FA	-2.94062	-1.90202	-2.03953	-1.71621	-2.84503	-2.42968	-1.58496	-3.27684	-4.16993	-4.0268
P78316	Nucleolar p	NOP14 C4o	-2.85042	-2.24598	-1.36146	-1.2909	-2.64572	-1.95693	-2.18536	-1.72935	-1.45003	-1.1008
P78318	Immunoglo	IGBP1 IBP1	0.47587	0.462972	1.030861	0.650365	0.96233	0.610242	0.854802	1.00286	1.086908	1.087983
P78332	RNA-bindin	RBM6 DEF3	-0.72824	-0.45738	-0.54839	-0.79021	-0.40024	-0.33015	-0.41163	-0.59358	-0.45003	-0.16385
P78344	Eukaryotic	EIF4G2 DAI	0.42711	0.548103	0.468619	0.439912	0.220266	0.690316	0.231136	0.498454	0.055495	-0.11032
P78346	Ribonuclea	RPP30 RNA	-0.72489	-0.82402	-0.33171	-0.35364	-1.19052	-0.72247	-0.60039	-1.07039	-0.53629	-0.10555
P78347	General tra	GTF2I BAP1	-1.64689	-1.46529	-2.04849	-2.28732	-1.09212	-1.85457	-1.53964	-1.63743	-1.83112	-1.38294
P78356	Phosphatid	PIP4K2B PI	-1.3399	-1.18562	0.130357	0.180221	-0.65643	-1.15402	-0.84225	-0.75642	-0.50707	-0.61059
P78362	SRSF protei	SRPK2	-0.90603	-0.59804	-0.43628	-0.89661	-0.96936	-0.43299	-0.34847	-0.46497	-0.36453	-0.52781
P78364	Polyhomec	PHC1 EDR1	-4.08746	-4.72403	-4.07195	-3.9386	NA	NA	-3.81762	-3.58496	-4.00946	-3.76377
P78371	T-complex	CCT2 99D8	0.326551	0.431515	0.221862	0.291435	0.336655	0.408564	0.36262	0.379016	0.676491	0.493938
P78406	mRNA expo	RAE1 MRN	0.742976	0.7325	1.016912	1.024539	0.754198	1.2643	1.141492	1.012427	0.837775	0.431352
P78410	Butyrophili	BTN3A2 BT	-0.82873	-0.76983	0.2824	0.666263	-0.23359	-0.45943	0.407343	-0.05247	-0.77357	-0.52218
P78417	Glutathion	GSTO1 GST	2.019836	2.108378	0.64638	1.130209	0.423883	0.282378	1.423611	1.226825	1.160761	1.064084
P78527	DNA-deper	PRKDC HYR	0.028784	0.112475	-0.29879	-0.31411	-0.47478	-0.30286	-0.0548	-0.23581	-0.33942	-0.23755
P78537	Biogenesis	BLOC1S1 B	-0.51238	-0.52867	-0.72466	-0.76951	-0.63427	-0.4733	-0.70639	-0.84594	-0.30076	-0.52086

P78563	Double-stra	ADARB1 AD	-2.40296	-2.2847	-2.28345	-2.75803	-3.35399	-2.75332	-2.13955	-3.72935	-2.64028	-3.17936
P80217	Interferon-	IFI35 IFP35	-0.19965	0.590067	0.15686	0.464264	0.424257	0.27733	0.345623	0.345305	0.585867	0.54598
P80297	Metallothio	MT1X	-2.48873	-1.74338	-1.46751	-1.84075	-2.87844	-2.14455	-1.94622	-1.87194	-1.71391	-1.94184
P80303	Nucleobinc	NUCB2 NEF	0.044639	0.239991	0.221206	0.100046	-0.29475	-0.0436	-0.13435	0.206496	0.519337	0.459891
P80723	Brain acid s	BASP1 NAP	0.643934	0.921557	0.11307	-0.12463	0.34901	-0.01913	-0.36148	-0.43204	-0.21523	-0.24627
P81877	Single-stran	SSBP2 SSDI	-1.82873	-2.02359	-2.05565	-1.27563	-1.24236	-1.70454	-1.42531	-1.6735	-1.62803	-1.26127
P82094	TATA elem	TMF1 ARA1	-0.76249	-1.05642	-0.39193	-0.57544	-0.61223	-0.63166	-0.62132	-0.72201	-0.32774	-0.77971
P82650	28S riboson	MRPS22 C3	-0.30935	-0.36407	-0.45849	-0.32886	-1.07244	-0.35664	-0.38351	-0.29514	-0.80965	-0.50987
P82663	28S riboson	MRPS25 RF	1.009064	0.896979	0.847997	0.9874	1.018473	0.759737	1.122543	0.950498	1.06454	1.178749
P82673	28S riboson	MRPS35 M	0.777608	0.744743	0.482639	0.294061	0.3249	0.471306	0.661349	0.767554	0.756633	0.499895
P82909	28S riboson	MRPS36 KC	-0.22948	-0.33171	0.144576	-0.32193	-0.16528	-0.1315	0.094068	0.380272	0.046393	0.047877
P82912	28S riboson	MRPS11 RF	-0.65404	-0.64014	-0.69277	-1.2432	-1.04778	-0.46887	-0.26517	-0.51506	-0.44602	-0.63655
P82930	28S riboson	MRPS34	-1.242	-1.67051	-1.23749	-2.28992	-1.94177	-1.21803	-1.01796	-1.88283	-1.195	-2.11748
P82933	28S riboson	MRPS9 RPM	-0.63743	-0.48699	-1.26459	-0.79405	-1.00377	-0.65924	-1.0409	-0.83773	-0.76736	-0.82653
P82979	SAP domain	SARNP HCC	0.759818	1.068531	0.432422	0.227949	0.256013	0.261754	0.591652	0.584959	0.371969	0.424817
P83111	Serine beta	LACTB MRF	0.438197	0.472483	0.747075	0.75937	0.833823	0.797432	0.744483	0.999071	0.719116	0.669343
P83436	Conserved	COG7 UNQ	-0.35566	-0.34156	-0.48699	-0.53261	-0.79256	-0.89465	-0.47422	-0.11309	-0.66493	-0.76377
P83731	60S riboson	RPL24	0.727234	0.46014	0.269518	0.126054	0.342602	0.566631	0.680628	0.680932	0.371969	0.258602
P83881	60S riboson	RPL36A RPI	1.467519	1.212223	1.714796	1.351783	2.006175	1.600478	1.824495	1.657482	1.455855	1.52048
P83916	Chromobox	CBX1 CBX	0.360408	0.454741	0.970902	0.585951	0.910566	0.738573	0.703418	0.696522	1.303035	1.161047
P84074	Neuron-spe	HPCA BDR2	-0.9579	-0.33329	-0.86459	-0.67064	-0.83361	-0.25673	-0.24606	-0.13661	-0.92029	-0.28293
P84085	ADP-ribosy	ARF5	1.728281	1.91369	1.241553	1.060602	1.063644	1.220306	1.380955	1.348919	1.066387	0.969811
P84090	Enhancer o	ERH	0.763394	1.074098	0.133937	0.609837	-0.45017	0.529253	0.399607	0.770913	0.281771	0.57905
P84095	Rho-relatec	RHOG ARH	-0.1669	-0.01568	0.217428	0.109456	-0.1467	-0.29202	-0.22666	-0.58496	-0.07748	0.0051
P84098	60S riboson	RPL19	0.018072	0.410579	1.227881	0.755619	0.899076	0.891045	1.005588	1.012293	1.087112	1.009258
P84103	Serine/argi	SRSF3 SFRS	-0.03684	-0.09675	0.349832	0.309328	0.643917	0.139723	0.23105	0.098446	0.382857	0.221127
P84157	Matrix-rem	MXRA7	-2.72489	-2.57719	-3.72403	-1.07731	-3.27798	-1.10188	-2.05209	-1.36257	-1.81444	-1.51584
P84243	Histone H3	H3-3A H3.3	-0.95869	-0.59337	-1.62057	-0.80144	-0.91602	-0.77476	-1.21554	-0.74855	-1.76698	-1.74892
P85037	Forkhead b	FOXK1 MN	-1.09649	-0.78998	-1.37119	-0.574	-1.4855	-1.02425	-0.89966	-1.38113	-1.10725	-0.68383
P86790	Vacuolar fu	CCZ1B C7o	-0.2949	-0.41244	0.223506	-0.01894	-0.14048	0.135515	0.387926	0.321928	0.160465	-0.07727
P98170	E3 ubiquitin	XIAP API3 E	-0.83484	-1.0786	-1.52418	-1.53118	-0.98312	-1.65722	-1.01466	-0.72242	-0.96694	-1.53303
P98171	Rho GTPase	ARHGAP4 K	0.299939	0.419903	0.595323	0.597862	0.466176	0.540568	0.155069	0.251539	0.277888	0.447683
P98175	RNA-bindin	RBM10 DXS	-0.99069	-0.93379	-0.4752	-0.95763	-0.48059	-0.30183	-0.16418	-0.44021	-0.58606	-0.32126

P98179	RNA-bindir	RBM3 RNP	-1.01967	-0.68816	-0.36999	-0.24761	-0.65156	-0.42852	-0.32958	-0.52196	-0.33379	-0.17466
Q00059	Transcripti	TFAM TCF6	0.554361	0.840178	0.645166	0.612374	1.130707	0.607075	0.896881	0.764124	0.541871	0.653946
Q00169	Phosphatid	PITPNA PIT	0.872763	0.817472	-0.10107	0.293508	-0.78364	0.615129	0.472553	0.425693	-0.66024	-0.81388
Q00325	Phosphate	SLC25A3 PI	0.972831	0.954033	0.980373	1.063064	0.433517	1.203339	1.239673	1.238118	0.273883	-0.40959
Q00341	Vigilin (Higl	HDLBP HBF	0.467126	0.462972	0.348202	0.299168	0.919723	0.743508	0.286713	0.633745	0.578663	0.536968
Q00403	Transcripti	GTF2B TF2I	-0.31335	-0.21423	-0.01963	-0.7857	-0.09212	-0.04974	0.030374	-0.10128	-0.44245	-0.14416
Q00535	Cyclin-depe	CDK5 CDKN	-1.55497	-1.62449	-2.05565	-1.9386	-1.33313	-1.79647	-2.46713	-2.24936	-2.06756	-2.06333
Q00577	Transcripti	PURA PUR1	-1.74507	-1.46529	-1.88753	-1.45317	-2.27889	-1.40823	-1.53964	-1.50155	-0.89984	-1.28276
Q00610	Clathrin he	CLTC CLH17	0.155031	0.301509	0.112475	0.137504	0.18799	-0.05571	-0.01027	0.06882	0.206137	0.094215
Q00613	Heat shock	HSF1 HSTF1	-0.32496	0.030861	-0.12199	-0.30633	-0.24236	0.105353	-0.02061	-0.01728	0.139217	-0.15397
Q00653	Nuclear fac	NFKB2 LYT1	-0.87365	-0.86073	-0.90202	-0.86821	-0.73606	-1.06711	-0.97465	-1.25348	-0.81444	-0.93934
Q00688	Peptidyl-pr	FKBP3 FKBI	1.204081	1.30852	1.079719	1.062773	1.199289	1.01764	1.031024	1.13546	1.35018	1.346137
Q00722	1-phosphat	PLCB2	-0.06171	-0.03967	-0.16757	-0.16644	-0.32813	-0.44072	-0.30251	-0.3101	-0.40852	-0.49556
Q00765	Receptor e	REEP5 C5or	1.241754	1.395465	2.025552	1.816947	1.801505	1.63866	1.503299	1.461949	1.271161	0.963288
Q00796	Sorbitol de	SORD	-1.90243	-1.10534	-1.04194	-1.02255	-1.10958	-1.14886	-1.2237	-1.22017	-0.89157	-0.76793
Q00839	Heterogen	HNRNPU C	0.47587	0.656134	0.740082	0.643753	1.094432	0.869228	1.085776	0.82918	0.645983	0.70213
Q01081	Splicing fac	U2AF1 U2A	0.164906	0.90146	0.443563	0.630903	0.923649	0.344234	0.870222	0.60989	0.375786	0.528083
Q01082	Spectrin be	SPTBN1 SPT	0.299939	0.471434	-0.16086	0.008971	-0.16528	0.130532	-0.06274	-0.08246	0.297201	0.379192
Q01085	Nucleolysir	TIAL1	-0.15335	-0.73697	0.574527	0.589138	0.264273	0.555519	0.050273	0.061639	0.281771	0.362078
Q01105	Protein SET	SET	0.410039	0.931197	0.111175	0.136962	0.438495	0.093814	0.106657	0.358083	0.172574	0.321897
Q01130	Serine/argi	SRSF2 SFRS	1.56487	1.582427	-0.02885	-0.16216	0.206133	-0.03457	0.768878	0.629334	-0.11311	0.382491
Q01196	Runt-relate	RUNX1 AM	-0.43756	-0.32654	0.011337	0.069463	-0.13952	0.113737	-0.33607	-0.16793	0.327025	0.354048
Q01201	Transcripti	RELB	-1.76142	-1.20552	-1.17051	-1.49159	-1.27956	-1.62391	-1.62009	-1.66158	-1.3284	-2.15485
Q01415	N-acetylgal	GALK2 GK2	0.4975	0.414062	0.42862	0.685891	0.383081	0.825971	0.372201	0.050626	0	0.429349
Q01432	AMP deam	AMPD3	0.674441	0.683969	0.5155	0.704338	0.776012	0.829198	0.453891	0.553185	1.032787	0.737766
Q01433	AMP deam	AMPD2	-0.64689	-0.57864	-0.2548	-0.41923	-0.01495	-0.2962	-0.46146	-0.23462	-0.18311	-0.31531
Q01459	Di-N-acetyl	CTBS CTB	0.644341	0.850161	0.944352	1.301715	0.460783	0.880418	0.866073	0.581141	0.108934	0.174834
Q01469	Fatty acid-l	FABP5	-0.22533	-0.36995	-0.61252	-0.89129	-0.37563	-0.63055	-0.24233	-0.12663	-0.14378	-0.07036
Q01518	Adenylyl cy	CAP1 CAP	1.270089	1.349576	1.014153	1.246793	1.138365	1.43334	1.300601	1.574446	1.104496	1.022745
Q01543	Friend leuk	FLI1	-0.44597	-0.36985	0.159876	-0.07458	0.114333	0.043443	0	-0.19283	-0.04683	0.11054
Q01650	Large neut	SLC7A5 CD	0.334001	0.763239	0.574527	0.609837	0.348454	0.652573	-0.02061	0.241889	-0.29739	-0.37145
Q01658	Protein Dr1	DR1	-0.6787	-0.71227	-0.97145	-1.0085	-1.01619	-1.19989	-1.34894	-1.09901	-0.73241	-0.57711
Q01780	Exosome c	EXOSC10 P	0.383323	0.287044	0.423038	0.011834	0.355057	0.472145	0.055206	0.039528	0.474598	0.320744

Q01804	OTU domai	OTUD4 HIN	-1.54709	-1.21747	-0.98975	-1.7356	-0.88744	-1.34636	-2.15252	-2.20916	-1.82811	-1.70854
Q01813	ATP-depen	PFKP PFKF	-0.64889	-0.56825	-0.29502	-0.42115	-0.47226	-0.50228	-0.54727	-0.86528	-1.09459	-1.09134
Q01826	DNA-bindir	SATB1	-1.58836	-1.84765	-2.13906	-1.47045	-1.57823	-1.70454	-1.76861	-2.97728	-1.76553	-1.28264
Q01831	DNA repair	XPC XPCC	1.070967	0.97443	-0.27399	0.091148	0.564994	0.352211	0.384011	0.207595	-0.45003	0.178749
Q01844	RNA-bindir	EWSR1 EW	0.980427	1.033832	0.853387	0.785569	0.635373	0.534157	0.755243	0.420045	0.95809	0.67832
Q02040	A-kinase ar	AKAP17A C	-0.14662	-0.0039	-0.57138	-0.13813	-0.00746	-0.4975	-0.07886	-0.36995	-0.37474	-0.52218
Q02083	N-acyletha	NAAA ASAH	0.575735	0.47173	0.546739	0.693825	0.104849	0.047394	0.478044	0.307648	0.197916	0.420317
Q02086	Transcripti	SP2 KIAA0C	0.51881	0.414062	0.817347	0.942413	0.859519	0.994525	0.749192	0.784271	0.907519	1.052151
Q02127	Dihydroorc	DHODH	-0.94065	-0.88144	-1.30646	-1.35553	-1.33258	-0.71043	-1.09053	-1.51532	-1.39225	-1.59077
Q02218	2-oxoglutaı	OGDH	0.626127	0.988117	0.939717	0.885904	0.525595	0.739145	0.861411	1.001219	0.576908	0.303306
Q02252	Methylmal	ALDH6A1 N	0.26	0.414062	0.049819	-0.10402	-0.5231	-0.1315	0.426503	0.039528	0.470533	-0.00888
Q02338	D-beta-hyd	BDH1 BDH	0.761643	0.735501	0.123626	0.197356	0.216032	0.684918	0.361745	0.294443	0.157502	-0.03148
Q02446	Transcripti	SP4	-1.75821	-1.82009	-0.4195	-0.82116	-0.5015	-0.70851	-0.23266	0.061639	-0.55072	-0.6811
Q02447	Transcripti	SP3	0.716207	0.808469	0.697437	0.765535	1.079567	0.90105	0.877257	0.889966	0.676303	0.706554
Q02543	60S ribosoı	RPL18A	1.329184	1.403784	0.381508	0.171318	0.232977	-0.01097	0.51866	0.187627	0.014075	0.251185
Q02750	Dual specif	MAP2K1 M	0.875751	0.893778	1.279805	1.083762	1.449936	1.519574	0.979609	1.196866	1.279831	1.105178
Q02790	Peptidyl-pr	FKBP4 FKBI	0.115621	0.175978	0.317241	0.646363	0.476903	0.661188	0.421575	0.416914	0.661475	0.690191
Q02818	Nucleobinc	NUCB1 NU	0.224123	-0.17823	0.246696	0.220599	-0.50188	0.095157	-0.32577	-0.26992	0.117652	0.060663
Q02878	60S ribosoı	RPL6 TXREI	1.40439	1.467123	1.236803	1.155874	1.411314	1.486987	1.350297	1.404021	1.692998	1.438722
Q02880	DNA topois	TOP2B	0.103242	0.165586	0	0	0.174874	0.037755	0.131912	0.126008	0.086908	-0.03132
Q02978	Mitochond	SLC25A11 S	-0.77588	-0.46529	-1.08017	-0.87971	-1.35199	-1.40054	-1.01027	-1.33342	-1.45003	-1.56737
Q03164	Histone-lys	KMT2A ALL	-1.34023	-1.94642	-1.23451	-1.82312	-0.10806	-0.47454	-0.73773	-1.80365	-1.64127	-1.48633
Q03188	Centromer	CENPC CEN	0.749038	1.01165	-1.16307	-1.76147	-1.05892	-1.3352	-1.83312	-1.30153	-1.47627	-1.46894
Q03252	Lamin-B2	LMNB2 LM	0.226797	0.054945	0.693946	0.493208	0.766409	0.570147	0.680628	0.713038	0.88404	0.897172
Q03518	Antigen pe	TAP1 ABCB	-0.11289	0.251125	0.040788	-0.30041	-1.34552	-0.91401	-0.12493	-0.32768	-1.26376	-0.70939
Q03519	Antigen pe	TAP2 ABCB	0.395166	0.302933	0.278585	-0.06003	-0.90112	-0.19878	0.156798	0.025111	-0.48892	-0.59485
Q03701	CCAAT/enh	CEBPZ CBF	0.542457	0.630238	-0.27135	-0.53492	0.01073	-0.13376	-0.20134	-0.43214	-0.01517	0.018653
Q04206	Transcripti	RELA NFKB	-0.9803	-0.69054	-0.46188	-0.23816	-0.37109	-0.12553	-0.36476	-0.25618	-0.37534	-0.41201
Q04323	UBX domai	UBXN1 SAK	-0.24762	0.037194	0.220163	0.120294	0.036712	0.208271	0.074768	-0.07641	0.250407	0.0649
Q04446	1,4-alpha-g	GBE1	0.49599	0.39756	0.339141	0.568665	0.380122	0.680462	0.166437	0.281847	0.561993	0.589362
Q04637	Eukaryotic	EIF4G1 EIF4	0.019433	-0.12143	0.273059	0.175477	-0.10027	0.110424	0.272617	-0.11781	-0.08277	-0.0396
Q04726	Transducin	TLE3 KIAA1	-1	-0.89476	-0.35646	-0.91475	-0.63413	-0.91511	-0.73016	-0.848	-0.59918	-0.48454
Q04760	Lactoylglut	GLO1	1.531026	1.587577	1.718913	1.584035	1.406642	1.393385	1.586696	1.032168	1.615979	1.535629

Q04837	Single-strar	SSBP1 SSBF	0.292917	-0.00639	0.176811	0.595802	0.52937	1.232015	0.38873	0.994639	0.969022	0.490132
Q04864	Proto-onco	REL	-1.69225	-1.62529	-1.26047	-1.74627	-0.05302	0.069348	-0.97929	-1.76441	-1.62803	-1.10355
Q04917	14-3-3 prot	YWHAH YW	0.552195	0.615891	0.10024	-0.06344	0.172397	0.327339	0.130741	0.284148	-0.04644	0.00132
Q04941	Proteolipid	PLP2 A4	-0.5285	-0.32193	-0.81251	-0.65519	-1.56134	-0.36989	-0.4566	-0.45814	-1.26203	-1.26236
Q05048	Cleavage st	CSTF1	-1.87245	-1.38163	-2.11454	-1.61667	-1.15697	-1.37197	-1.69661	-1.63743	-1.68753	-1.3375
Q05086	Ubiquitin-p	UBE3A E6A	-0.39497	-0.45457	-0.57274	-0.69574	-0.72955	-0.47454	-0.57447	-0.46212	-0.4635	-0.68339
Q05193	Dynamin-1	DNM1 DNM	2.031478	2.164717	2.264658	2.409539	2.18799	2.404755	2.287761	2.284815	2.175154	2.104542
Q05209	Tyrosine-pr	PTPN12	0.229304	0.298341	0.034673	0.187933	-0.01495	-0.30968	-0.07886	-0.0115	-0.22948	-0.40036
Q05315	Galectin-10	CLC LGALS1	2.211091	2.364951	2.079643	2.148863	3.370193	3.271322	1.931911	1.688056	2.382857	2.168941
Q05519	Serine/argi	SRSF11 SFR	-0.03722	0.255244	0.881646	0.512711	0.356727	0.458199	0.318422	0.275529	0.626305	0.49198
Q05655	Protein kin	PRKCD	0.969368	0.670742	-2.05029	-1.88364	-1.17404	-1.52425	-1.20293	-1.9661	-1.8875	-2.3291
Q05D32	CTD small p	CTDSPL2 H	-1.28011	-1.61252	-0.73748	-0.9586	-0.87346	-1.11037	-0.68052	-0.57868	-2.42428	-2.18593
Q06124	Tyrosine-pr	PTPN11 PT	0.192645	0.396397	0.2824	0.314381	0.065423	0.165059	0.235488	0.289507	0.304855	0.077537
Q06203	Amidophos	PPAT GPAT	-0.666	-0.36146	0.172522	0.476438	0.14828	0.459432	-0.0574	0.152003	0.523972	0.889677
Q06210	Glutamine-	GFPT1 GFA	-1.1004	-0.81028	-0.60064	-0.74746	-0.19903	-0.88452	-0.66382	-0.65535	-0.89984	-1.0268
Q06265	Exosome co	EXOSC9 PM	0.899598	0.956459	0.682938	0.421296	0.154975	0.250227	0.204745	0.067114	0.342525	0.67364
Q06323	Proteasom	PSME1 IFI5	1.036362	1.209464	1.235746	1.373678	0.887927	0.686925	1.235488	1.207595	1.210218	1.31305
Q06330	Recombinin	RBPJ IGKJR	-3.37126	-2.48699	-3.88753	-4.38606	NA	NA	-3.43911	-2.88982	-3.1281	-3.64829
Q06546	GA-binding	GABPA E4T	-0.27277	-0.01175	-0.36146	-0.11078	-0.03767	-0.5366	-0.3322	-0.36995	-0.2916	0.081724
Q06547	GA-binding	GABPB1 E4	-1.34869	-1.26359	-1.79873	-1.44121	-1.38888	-1.85227	-0.99454	-1.7549	-1.73076	-1.25761
Q06587	E3 ubiquitin	RING1 RNF	-0.70516	-0.54556	-0.66307	-0.44661	-0.09221	-0.53762	-0.42821	-0.35025	-0.89107	-1.08194
Q06787	Synaptic fu	FMR1	-2.35109	-1.28345	-1.1305	-1.27563	-1.26006	-1.33015	-1.03103	-1.2091	-0.87368	-0.73402
Q07002	Cyclin-depe	CDK18 PCT	-1.06193	-1.10512	-0.6734	-1.65567	-0.9852	-0.57679	-0.65574	-1.05842	-0.78165	-0.89752
Q07020	60S ribosor	RPL18	-0.40143	-0.475	1.013583	1.024875	1.040332	0.991779	1.200299	0.833292	0.688056	1.021959
Q07021	Compleme	C1QBP GC1	0.139487	0.308651	-0.25703	-0.27465	0.640419	0.667685	0.428123	0.598872	0.447146	0.636875
Q07065	Cytoskeleto	CKAP4	-0.85616	-0.99222	-1.1305	-1.26178	-1.06075	-0.94456	-1.20514	-0.80472	-1.22502	-0.90578
Q07666	KH domain	KHDRBS1 S	-0.88359	0.286237	0.451736	0.286456	0.462471	0.356485	0.518523	0.177538	0.009399	0.448933
Q07812	Apoptosis r	BAX BCL2L4	0.677641	0.119244	-0.39726	-0.43486	-0.73528	0.089024	-0.0274	-0.23811	-0.73537	-0.66411
Q07866	Kinesin ligh	KLC1 KLC K	0.839892	0.794397	1.10343	0.758136	0.970342	1.040363	0.898086	0.913221	0.976232	0.863439
Q07889	Son of seve	SOS1	-0.02445	-0.23676	-0.23217	-0.2909	-0.23359	-0.62654	-0.3322	-0.71049	-0.53629	-0.15397
Q07955	Serine/argi	SRSF1 ASF S	0.670829	0.663569	-0.07606	-0.33344	-0.26031	-0.32498	-0.23614	-0.23317	0.002461	0.130984
Q07960	Rho GTPase	ARHGAP1 C	0.743363	1.008087	0.913454	0.939812	0.48755	0.721906	0.664403	0.744459	0.397097	0.604717
Q08170	Serine/argi	SRSF4 SFRS	-0.45187	-0.29298	-1.12199	-0.62632	-0.28703	-0.74061	-0.7217	-0.78746	-0.76683	-0.75606

Q08209	Serine/thre	PPP3CA CA	-0.03063	-0.29298	0.323487	0.049328	0.294905	0.490881	-0.30035	-0.04654	0.060025	0.052151
Q08211	ATP-depen	DHX9 DDX9	-0.04967	0.145825	-0.43335	-0.56937	-0.23002	-0.34837	-0.25696	-0.47743	-0.54004	-0.26127
Q08257	Quinone o›	CRYZ	-0.18746	0.259781	-0.33662	-0.31411	-0.06075	-0.1375	0.030374	-0.10732	0.313541	0.36306
Q08378	Golgin subf	GOLGA3	-0.59104	-0.28974	-0.20945	-0.22127	-0.19231	-0.17403	-0.51303	-0.553	-0.20374	-0.04735
Q08379	Golgin subf	GOLGA2	-1.91754	-1.4761	-1.34427	-1.98751	-1.59991	-1.40054	-1.96546	-1.65113	-2.10073	-1.54137
Q08722	Leukocyte :	CD47 MER(	-0.37727	-0.43389	0.307127	0.077488	-0.19674	-0.20071	0.056909	0.151994	0.379901	0.02942
Q08752	Peptidyl-pr	PPID CYP4(	-0.24334	-0.16657	-0.07209	-0.02531	0.117564	0.188939	0.008683	0.044757	0.13493	0.03065
Q08881	Tyrosine-pı	ITK EMT LY	0.777608	0.477047	0.633525	0.354182	-0.28703	0.304334	0.384011	-0.36995	0.143492	-0.01781
Q08945	FACT comp	SSRP1 FACT	-0.85042	-0.57138	-0.92405	-0.3377	-1.04532	-0.18641	-0.38466	-0.60224	-0.91754	-0.77887
Q08999	Retinoblast	RBL2 RB2	-0.90613	-1.26459	-0.97679	-1.0126	-0.74193	-1.02203	-0.86343	-1.05842	-0.77357	-0.95677
Q08AF3	Schlafen fa	SLFN5	-0.15335	-0.10932	-0.36647	-0.22344	-0.37109	-0.37906	-0.22069	-0.05842	-0.62803	-0.47221
Q08AM6	Protein VA(	VAC14 TAX	-0.53182	-0.4983	-0.15453	-0.39451	-0.48791	-0.31798	-0.43214	-0.56814	-0.25776	-0.17383
Q08J23	RNA cytosi	NSUN2 SAK	0.332254	0.266189	0.123847	0.283733	0.116321	0.335284	0.1811	0.230495	0.259474	-0.10085
Q09028	Histone-bir	RBBP4 RBA	-0.77385	-0.50476	-0.28342	-0.52808	-0.62951	-0.62263	-0.59503	-0.50781	-0.34208	0.027946
Q09161	Nuclear ca	NCBP1 CBP	-0.2238	-0.03061	-0.34549	-0.29741	-0.25813	-0.2735	-0.29978	-0.0751	-0.21153	0.041271
Q09472	Histone ac	EP300 P30(	-1.89482	-2.05565	-2.07195	-1.56009	-2.55552	-2.91511	-1.9301	-2.88982	-1.57089	-1.38294
Q09666	Neuroblast	AHNAK PM	0.836501	0.944352	1.241743	1.137627	0.826352	0.765071	0.896622	0.879608	0.323813	0.258602
Q0VD83	Apolipopro	APOBR AP(	-0.83462	-0.79826	-0.2898	-0.3804	-0.7051	-0.54455	-0.71956	-0.85036	-0.96075	-1.15388
Q0VDF9	Heat shock	HSPA14 HS	-0.78629	-0.99222	-0.4021	-0.32979	-0.47063	-0.42968	-0.47422	-0.24257	-0.20754	-0.54783
Q10471	Polypeptid	GALNT2	0	-0.2185	-0.25061	0.182416	-0.42	-0.26961	0.117836	-0.00574	-0.59918	-0.54783
Q10567	AP-1 comp	AP1B1 ADT	-1.53086	-1.28405	-1.68726	-2.32329	-3.00697	-1.31158	-1.46768	-1.76994	-1.97484	-2.62106
Q10570	Cleavage a	CPSF1 CPSF	-0.91754	-0.78319	-0.92405	-0.90374	-0.49317	-1.16175	-0.83415	-0.78382	-0.86507	-0.53088
Q10589	Bone marr	BST2	0.20847	0.52661	0.288798	0.724366	0.258068	0.529253	0.475158	0.289507	0.108934	0.422761
Q10713	Mitochond	PMPCA INF	-0.58649	-0.37141	-0.4127	-0.9582	-0.83197	-0.55716	-0.60567	-0.17275	-0.34993	-0.46968
Q11201	CMP-N-ace	ST3GAL1 SI	-1.74507	-0.81714	-1.06378	-0.89129	-3.07635	-2.02203	-1.39808	-1.48543	-1.65748	-0.99118
Q12765	Secernin-1	SCRN1 KIA	-0.13327	0.053581	-0.07605	-0.55087	1.083082	1.133025	0.588369	0.673772	0.349942	0.182653
Q12769	Nuclear po	NUP160 KI	-0.76062	-0.82035	-1.39216	-1.47328	-2.64572	-1.04062	-1.39808	-1.51785	-1.46879	-1.44591
Q12770	Sterol regu	SCAP KIAA(	-2.76553	-2.20945	-1.6734	-1.89129	-1.71727	-3.79647	-2.16227	-1.24936	-1.9906	-1.34873
Q12778	Forkhead b	FOXO1 FKH	-1.89482	-2.12199	-1.81714	-3.32193	-1.79256	-1.81558	-1.55459	-2.14439	-1.31487	-0.83303
Q12788	Transducin	TBL3 SAZD	-1.06617	-1.13786	-1.62454	-1.31489	-1.63537	-1.51032	-1.23326	-1.54351	-1.2469	-1.32083
Q12792	Twinfilin-1	TWF1 PTK9	-4.08746	-3.4021	-1.43335	-1.8011	-3.79256	-1.95693	-1.58496	-2.1193	-1.73383	-1.73402
Q12802	A-kinase ar	AKAP13 BR	-1.34506	-1.17383	-0.65488	-0.94262	-0.98641	-0.58562	-0.75585	-0.76878	-0.8002	-0.51584
Q12824	SWI/SNF-re	SMARCB1 I	0.229304	0.510278	-0.17823	-0.12439	-0.14048	0.184425	0.122543	0.141661	0.281771	-0.09134

Q12830	Nucleosom	BPTF FAC1	-0.67565	-0.86605	-0.7176	-1.06413	-1	-1.31647	-1.28157	-1.03477	-0.69515	-1.36004
Q12846	Syntaxin-4	STX4 STX4/	-0.10126	-0.10464	-0.34672	-0.70623	0.236571	0.279887	-0.18781	-0.10668	0.000412	-0.41373
Q12849	G-rich sequ	GRSF1	-1.04933	-1.02359	-1.17383	-1.21613	-0.79256	-0.52869	-0.9301	-0.43812	-0.36864	-0.50952
Q12851	Mitogen-ac	MAP4K2 G(	-1.96997	-1.28336	-1.52833	-2.15307	-1.43022	-0.98375	-1.55357	-1.75378	-1.80052	-1.32981
Q12857	Nuclear fac	NFIA KIAA1	-0.03684	0.112475	-0.01963	-0.03814	0.154975	0.308753	0.248466	0.187627	0.517399	0.484162
Q12872	Splicing fac	SFSWAP SF	-3.00907	-1.83805	-2.01639	-1.55102	-1.85399	-2.84824	-2.62837	-3.07039	-2.45929	-1.9901
Q12873	Chromodoi	CHD3	0.714237	0.889592	0.48689	0.319417	0.498989	0.155278	0.455782	0.050626	0.652473	0.413709
Q12874	Splicing fac	SF3A3 SAP(	0.47587	0.431515	0.532014	0.319417	0.114333	0.381871	0.536406	0.581141	0.702614	0.73608
Q12882	Dihydropyr	DPYD	-0.00126	0.212888	0.114833	0.042431	0.021842	0.372415	-0.10008	0.083163	0.300586	0.209566
Q12888	TP53-bindi	TP53BP1	-0.93339	-0.83094	-0.45929	-0.59751	-0.66882	-0.48026	-0.65978	-0.64163	-0.60956	-0.54466
Q12893	Transmeml	TMEM115	-0.89482	-0.85896	-0.52576	-0.5881	-0.56649	-0.71348	-0.68012	-0.76783	-0.4119	-0.35437
Q12894	Interferon-	IFRD2	-0.5025	-0.44392	-0.85896	-1.40255	-0.81855	-0.85457	-0.82667	-0.53434	-0.78978	-1.06333
Q12899	Tripartite n	TRIM26 RN	0.24984	0.038474	0.130397	-0.38606	-0.34252	-0.04439	0.182377	-0.05842	-0.09248	0.273324
Q12904	Aminoacyl	AIMP1 EM/	-0.63743	-0.53703	-0.18265	-0.35364	-0.38074	-0.14353	-0.41163	-0.1635	0.238467	-0.01781
Q12905	Interleukin	ILF2 NF45 F	-0.14751	-0.26259	-1.36172	-0.56443	-1.10478	-0.44525	-0.36328	-0.48261	-0.2405	-0.78239
Q12906	Interleukin	ILF3 DRBF I	0.302439	0.504792	0.380031	0.313206	0.46346	0.469267	0.273165	0.478041	0.637674	0.813663
Q12907	Vesicular ir	LMAN2 C5(	0.902844	0.749389	0.332805	0.309328	0.539639	0.592231	0.356304	0.371448	0.177987	0.074727
Q12912	Inositol 1,4	IRAG2 JAW	0.308002	0.239492	0.490303	0.687449	0.454206	0.476906	0.755231	0.621396	-0.16143	0.338622
Q12913	Receptor-t	PTPRJ DEP1	-1.32209	-0.87567	-1.21103	-1.08371	-2.76017	-1.61211	-0.97859	-1.06069	-1.96561	-1.85688
Q12931	Heat shock	TRAP1 HSP	0.624818	0.702238	0.240108	0.067399	0.070777	0.286523	0.364274	0.241889	0.517399	0.458627
Q12933	TNF recept	TRAF2 TRA	-2.80986	-2.49297	-1.63784	-1.32287	-2.47358	-2.03669	-1.56167	-2.14306	-1.53825	-1.61416
Q12955	Ankyrin-3 (	ANK3	-2.80735	-2.05565	-2.48699	-3.0906	-2.39046	-2.95693	-1.69661	-1.82753	-1.3505	-2.06333
Q12959	Disks large	DLG1	0.685025	0.606416	1.116449	0.86213	1.102723	1.060397	0.925288	0.657241	1.137484	1.331543
Q12962	Transcripti	TAF10 TAF:	-0.71491	-0.46529	-0.38163	-0.90298	-0.25118	-0.39334	-0.45305	-0.2091	-0.14886	-0.0223
Q12972	Nuclear inh	PPP1R8 AR	0.384243	0.432091	0.645261	0.548461	0.357467	0.35641	0.467594	0.444883	0.414465	0.504183
Q12974	Protein tyr	PTP4A2 PR	-0.19438	0.363436	0.112475	-0.14505	0.065423	0.273018	0.26984	0.35811	-0.1913	0.060663
Q12979	Active brea	ABR	-2.57289	-1.49796	-1.80346	-2.84549	-3.07635	-2.11366	-1.7997	-1.93289	-2.06756	-1.92246
Q12982	BCL2/aden	BNIP2 NIP2	0.442403	0.074182	0.148399	0.014846	-0.08479	0.259531	-0.18012	0.288015	0.08591	-0.18624
Q12986	Transcripti	NFX1 NFX2	0.229304	0.821765	0.863081	1.049328	1.161638	0.683526	0.937264	0.666576	0.622195	0.480995
Q12996	Cleavage st	CSTF3	-1.01003	-0.9845	-0.8499	-0.94673	-1.27514	-0.9997	-1.09084	-1.22915	-1.00603	-0.82344
Q13009	T-lymphom	TIAM1	0.382656	0.40344	0.940862	0.831523	0.712906	0.780016	0.566878	0.717645	0.624789	0.681604
Q13011	Delta(3,5)-I	ECH1	-0.75575	-0.60985	-0.66535	-0.66739	-0.46145	-0.77624	-0.48382	-0.37639	-0.57512	-0.31258
Q13042	Cell divisioi	CDC16 ANA	0.116409	-0.2185	-0.33171	-0.50564	0.029444	0.037755	-0.15656	0.131245	-0.40564	-0.04952

Q13043	Serine/thre	STK4 KRS2	0.681627	0.735325	0.626308	0.280935	0.086588	0.250227	0.368243	0.34915	0.546743	0.419456
Q13045	Protein flig	FLII FLIL	-0.16876	-0.24807	0.181901	-0.47076	0.095172	0.162023	-0.01488	0.164445	-0.1568	-0.21655
Q13049	E3 ubiquiti	TRIM32 HT	-1.666	-1.58887	-0.90933	-1.10402	-1.03006	-1.37197	-1.66382	-1.61973	-1.55695	-1.51584
Q13057	Bifunctiona	COASY PSE	-0.81939	-1.04399	-0.65019	-0.9348	-1.10726	-0.68446	-1.12832	-2.16993	-1.21366	-0.84943
Q13077	TNF recept	TRAF1 EBI6	0.155031	0.641046	0.972444	1.027954	0.609541	0.652573	-0.00513	0.51056	0.06454	0.131052
Q13084	39S ribosor	MRPL28 M	1.265054	1.27919	1.213452	0.977877	0.954676	0.972414	1.079617	0.994264	0.973833	0.936674
Q13094	Lymphocyt	LCP2	1.415795	0.56354	0.99421	0.899544	0.54059	0.834779	0.714987	0.601566	0.856068	0.729348
Q13098	COP9 signa	GPS1 COPS	0.213707	0.165586	-0.22728	-0.3111	-0.24236	-0.40777	-0.19704	-0.34726	-0.14631	0.166972
Q13107	Ubiquitin c	USP4 UNP	-0.86139	-0.39183	-0.24136	-0.26053	-0.38074	-0.44448	-0.51019	-0.74846	-0.5824	-0.51784
Q13123	Protein Rec	IK RED RER	-0.47056	-0.64025	-0.00143	-0.47959	-0.00469	-0.14958	-0.34264	-0.1543	-0.31197	-0.01075
Q13126	S-methyl-5	MTAP MSA	-1.00254	-0.69195	-0.61819	-1.06446	-0.7408	-0.62922	-1.45578	-0.90768	-0.00447	0.369259
Q13131	5'-AMP-act	PRKAA1 AM	-2.89482	-3.72403	-2.4021	-4.03814	-3.27798	-4.1375	-2.89162	-2.848	NA	NA
Q13144	Translation	EIF2B5 EIF2	0.18315	0.34074	0.23672	0.13477	-0.01872	0.252917	0.137906	0.06073	0.066417	0.179265
Q13148	TAR DNA-b	TARDBP TD	0.395201	0.416986	0.800651	0.470106	0.675844	0.366468	0.38247	0.31279	0.387189	0.448901
Q13151	Heterogen	HNRNPA0 I	0.907546	0.755306	0.690145	0.791891	0.868164	0.691574	0.957707	0.684576	1.11351	1.184176
Q13153	Serine/thre	PAK1	-1.46855	-1.49796	-1.17383	-1.52356	-1.79256	-1.42968	-1.74723	-1.48543	-1.33852	-1.40621
Q13155	Aminoacyl	AIMP2 JTV1	0.220858	-0.04176	-0.29337	-0.16515	0.241475	-0.11051	0.248742	-0.07696	0.040445	0.025814
Q13162	Peroxiredo	PRDX4	0.701329	0.699418	0.82397	0.968288	0.014797	0.360954	0.498856	0.690193	0.667425	0.31305
Q13164	Mitogen-ac	MAPK7 BM	1.078818	1.058244	0.927538	0.719453	0.746463	0.766739	0.867312	0.834914	0.698255	0.916566
Q13177	Serine/thre	PAK2	0.110807	0.030861	0.445898	0	0.354283	-0.0902	0.204745	0.227291	0.467126	0.576084
Q13185	Chromobo	CBX3	1.171271	1.053581	1.352789	1.262217	1.354283	0.883384	1.31166	0.911463	1.084687	1.253311
Q13190	Syntaxin-5	STX5 STX5A	0.436099	0.259781	-0.0516	0.225972	-0.53382	0.208271	-0.04679	-0.2978	-0.55695	-0.52855
Q13200	26S protea	PSMD2 TRA	0.632659	0.69262	1.116711	0.914773	1.039297	0.807355	0.811715	0.935177	0.816754	0.841781
Q13206	Probable A	DDX10	-3.5446	-2.89187	-1.88753	-1.59756	-2.82647	-2.5127	-2.96963	-1.60224	-2.68156	-2.81737
Q13217	DnaJ homo	DNAJC3 P5	-0.03063	-0.0516	0.345135	0.471923	-0.19903	0.005454	-0.09516	-0.07641	-0.1913	-0.20407
Q13227	G protein p	GPS2	-0.16011	-0.27872	0.169058	0.067399	-0.29613	-0.05004	-0.25081	-0.03477	0.152003	-0.18886
Q13242	Serine/argi	SRSF9 SFRS	-0.9523	-0.65486	-1.03154	-1.14505	-1.11666	-1.1618	-0.90115	-0.38482	-0.83954	-0.42392
Q13243	Serine/argi	SRSF5 HRS	0.6203	0.787384	0.830236	0.619659	0.866368	0.576388	1.015957	0.885789	0.972976	1.112738
Q13247	Serine/argi	SRSF6 SFRS	1.231938	1.225096	1.332943	0.947858	1.350874	0.940594	1.081893	1.138179	1.711721	1.45804
Q13257	Mitotic spi	MAD2L1 M	-1.02445	-1.19153	-1.17383	-0.47917	-1.62263	-0.90484	-1.06274	-1.93289	-1.45003	-1.31531
Q13263	Transcripti	TRIM28 KA	-0.74659	-0.46253	0.03191	-0.40324	-0.47063	0.090032	0.038787	-0.02285	0.36209	0.202467
Q13283	Ras GTPase	G3BP1 G3B	-0.96408	-1.23676	-1.14768	-1.07731	-0.87933	-1.0723	-1.13955	-1.22239	-0.78978	-1.0268
Q13287	N-myc-inte	NMI	-0.37126	-0.22304	0.249978	0.114512	-0.01495	-0.1315	0.079617	0.573467	0.787006	0.860725

Q13303	Voltage-ga	KCNAB2 KC	2.810815	2.786031	1.63101	2.651364	1.482236	2.457443	2.417912	2.172467	1.860255	1.932043
Q13310	Polyadenyl	PABPC4 AP	0.211031	0.249248	0.05492	-0.1493	0.054683	0.233515	0.128861	-0.01567	-0.08149	-0.1169
Q13315	Serine-prot	ATM	0.371969	0.574527	-0.83616	-0.89723	0.164375	0.092649	-0.39808	-0.63743	0.099842	0.382659
Q13325	Interferon-	IFIT5 ISG58	0.408963	0.529314	-0.18709	0.160134	-0.54463	0.079727	0.045324	0.146841	-0.13845	-0.32082
Q13330	Metastasis	MTA1	0.883259	0.76094	0.566651	0.630243	1.040332	0.96123	1.113114	1.112832	1.199993	1.223024
Q13342	Nuclear bo	SP140 LYSF	-1.40296	-1.68589	-1.5656	-1.61667	-3.2076	-1.9783	-1.89162	-2.848	-1.91754	-1.71937
Q13347	Eukaryotic	EIF3I EIF3S	0.343587	0.45446	0.149828	0.180412	0.127221	0.224185	0.131866	0.287649	0.276545	0.197485
Q13356	RING-type	PPIL2	0.269489	0.382445	0.563897	0.148938	0.456668	0.500339	0.358992	0.681944	0.710767	0.345697
Q13363	C-terminal-	CTBP1 CTB	-0.20133	-0.07605	0.206872	0.615292	0	0.277534	0.681626	0.596765	0.206137	0.461377
Q13370	cGMP-inhil	PDE3B	-1.5025	-1.00782	-0.86605	-1.40255	-1.42999	-0.75899	-1.45305	-1.30485	-1.5025	-0.77887
Q13371	Phosducin-	PDCL PHLO	0.047697	-0.27399	0.23017	0.031027	0.31895	-0.16788	0.274077	0.039528	0.152003	0.334266
Q13404	Ubiquitin-c	UBE2V1 CR	0.309753	0.108863	-1	-0.23078	-0.51245	0.227069	0.299241	-0.35523	-0.36864	-0.49698
Q13405	39S riboso	MRPL49 C1	-0.1004	0	0.408198	0.363963	-0.05302	0.540568	0.282513	0.427862	0.487446	0.593786
Q13409	Cytoplasmi	DYNC1I2 D	-0.411	-0.25061	-0.25992	-0.39428	-0.62263	-0.1375	-0.50293	-0.2091	-0.05772	0.026311
Q13418	Integrin-lin	ILK ILK1 ILK	1.228003	0.95172	1.213382	1.216944	1.160404	1.263625	1.097412	1.186167	0.609113	0.590127
Q13422	DNA-bindir	IKZF1 IK1 Ik	-1.0777	-1.01568	-0.7176	-1.50564	-1.06075	-0.73151	-1.03103	-1.03477	-0.83954	-1.00888
Q13423	NAD(P) tra	NNT	0.734046	0.819515	0.417591	0.32947	0.029444	0.037784	0.297507	0.15658	0.095698	0.004176
Q13426	DNA repair	XRCC4	-0.65436	-0.30636	-0.40718	-0.54012	-0.47833	-0.11574	0.075414	-0.09648	-0.00343	-0.0773
Q13427	Peptidyl-pr	PPIG	-1.06193	-0.65486	-0.80462	-0.84831	-0.58986	-0.9698	-0.54709	-0.46623	-0.50711	-0.7414
Q13428	Treacle prc	TCOF1	-0.05905	-0.05364	-0.00135	-0.14507	0.28883	0.394398	0.11073	0.112636	0.297201	0.337258
Q13435	Splicing fac	SF3B2 SAP1	-0.21159	0.013116	0.295167	0.167664	0.285238	0.094962	-0.08158	0.07507	0.28352	0.46621
Q13439	Golgin subf	GOLGA4	-0.62803	-0.4021	0.155119	0.198904	0.239291	-0.28284	-0.12274	-0.1571	-0.1281	0.110703
Q13442	28 kDa hea	PDAP1 HAS	-0.22375	-0.15552	-0.49022	-0.45951	-0.33197	-0.50286	-0.13787	-0.31773	0.267611	0.44218
Q13451	Peptidyl-pr	FKBP5 AIG6	0.963079	1.058748	1.045771	1.079167	1.301854	0.952584	1.224585	1.261788	1.350996	1.374276
Q13459	Unconvent	MYO9B MY	-0.73526	-0.74506	-0.28345	-0.26053	-0.31451	-0.60977	-0.6477	-0.48543	-0.45003	-0.68339
Q13464	Rho-associ	ROCK1	-0.33502	-0.05386	-0.20757	-0.09085	-0.1488	-0.28664	-0.21786	-0.20582	-0.11045	-0.05314
Q13469	Nuclear fac	NFATC2 NF	0.070782	0.17379	0.032022	-0.28211	0.292941	0.277534	0.356331	0.363312	-0.36513	-0.31219
Q13485	Mothers ag	SMAD4 DP	-1.99761	-2.07419	-1.12635	-0.95763	-1.3433	-1.5422	-1.51263	-1.00159	-1.85957	-1.10393
Q13488	V-type prot	TCIRG1 ATI	-0.20133	0.193132	0.416986	-0.00629	-0.08421	0.084889	0.173332	-0.10692	-0.3505	-0.04952
Q13492	Phosphatid	PICALM CA	-0.833	-0.51049	-0.20084	-0.2961	-0.26719	-0.45687	-0.4255	-0.22499	-0.32077	-0.22956
Q13496	Myotubula	MTM1 CG2	-2.45388	-2.08691	-1.46529	-1.52356	-1.29613	-1.22422	-2.60718	-2.99367	-1.67243	-0.85688
Q13501	Sequestosc	SQSTM1 OI	-1.01597	-0.83141	-0.86693	-0.87357	-0.68803	-0.89016	-1.03506	-0.91353	-0.9193	-0.96257
Q13510	Acid ceram	ASAH1 ASA	1.781139	1.927025	0.828368	1.157325	0.703868	0.819792	1.069902	1.086115	-0.28011	-0.58718

Q13523	Serine/thre	PRPF4B KIA	-1.03448	-0.91783	-1.11652	-1.40896	-0.72644	-0.74061	-1.37435	-1.32639	-0.96747	-0.87924
Q13526	Peptidyl-pr	PIN1	-0.40475	-0.28538	-0.3433	-0.15345	-0.14062	-0.21476	-0.429	-0.20341	-0.02015	0.053009
Q13542	Eukaryotic	EIF4EBP2	0.280108	0.246696	0.083328	-0.22344	0.348454	-0.30293	-0.34514	-0.08854	0.397248	0.648451
Q13546	Receptor-ir	RIPK1 RIP F	-0.20007	-0.07081	0.005579	-0.10036	-0.30172	-0.08463	-0.11916	-0.21013	-0.26241	-0.18942
Q13547	Histone de	HDAC1 RPD	-0.40296	0.09067	-0.30507	-0.19438	0.236797	0.165956	0.168069	0.235025	-0.02519	0.208682
Q13555	Calcium/ca	CAMK2G C	-0.7256	-0.1978	-0.51457	-0.18015	0.266224	-0.2108	0.02726	-0.34581	0.083514	0.207375
Q13557	Calcium/ca	CAMK2D C	-0.74507	-0.39183	-0.60657	-0.46179	-0.17365	-0.05571	-0.0897	-0.02308	-0.18593	-0.03585
Q13561	Dynactin su	DCTN2 DCT	-0.05645	-0.24136	0.373022	0.320213	0.454068	0.652573	0.661349	0.673772	0.530515	0.70384
Q13564	NEDD8-act	NAE1 APPB	-0.29247	-0.04647	-0.39983	-0.82504	-0.84018	-0.81836	-0.64292	-1.01254	-0.61089	-0.71111
Q13573	SNW doma	SNW1 SKIIF	0.148789	0.051622	0.395898	0.140327	0.31895	0.435386	0.31166	0.053321	0.537028	0.320422
Q13574	Diacylglyce	DGKZ DAGI	-0.45187	-0.33662	0.038474	-0.0126	0.300954	0.221677	-0.02667	-0.04867	0.543512	0.695922
Q13576	Ras GTPase	IQGAP2	-0.00375	-0.07627	0.236803	-0.14252	-0.15697	-0.10819	-0.23409	-0.11524	-0.43721	-0.46996
Q13586	Stromal int	STIM1 GOK	-0.80735	-0.62783	-0.40883	-0.50355	-0.66918	-0.4837	-0.68012	-0.75052	-0.62399	-0.66925
Q13588	GRB2-relat	GRAP	1.003028	1.108863	0.69984	0.830467	0.931469	1.208271	0.709072	1.104869	1.490805	1.555154
Q13595	Transforme	TRA2A	0.756287	0.826914	1.043181	0.796561	0.585209	0.553388	0.842063	0.929924	0.904762	0.986351
Q13596	Sorting nex	SNX1	-1.53727	-1.28345	-1.44392	-0.86821	-1.39257	-1.34502	-1.39808	-1.24936	-1.31487	-1.29345
Q13601	KRR1 small	KRR1 HRB2	0.449478	0.206711	0.295421	0.445581	0.760338	0.81348	0.877898	0.645539	0.043266	0.515052
Q13613	Myotubula	MTMR1	-0.49964	-0.93821	-0.47069	-0.39428	-0.5231	-0.16175	-0.75585	-0.55102	-0.41818	-0.0223
Q13615	Myotubula	MTMR3 KIA	-0.96408	-1.28345	-1.20046	-1.14505	-1.39046	-1.48981	-1.15087	-1.51785	-1.4245	-1.29345
Q13616	Cullin-1 (CU	CUL1	-0.94104	-0.82091	-0.7508	-1.04495	-1.45779	-0.79348	-0.91653	-0.6759	-1.03346	-0.67374
Q13617	Cullin-2 (CU	CUL2	-1.18057	-0.43862	-0.43335	-0.24558	-0.58869	-0.62654	-0.6477	-0.48543	-0.4119	-0.47221
Q13618	Cullin-3 (CU	CUL3 KIAA(	0.057805	0.297557	0.125751	0.371596	-0.00373	0.066097	0.109328	0.000511	0.030012	0.062887
Q13619	Cullin-4A ((	CUL4A	-0.91365	-0.77791	-1.31339	-1.2118	-1.5729	-1.46091	-1.14336	-1.12515	-1.14199	-0.95502
Q13620	Cullin-4B ((	CUL4B KIAA	-0.86002	-1.17962	-0.82064	-0.77964	-1.42872	-0.81953	0.286323	0.060543	-0.7718	-0.58399
Q13627	Dual specif	DYRK1A DY	-0.0026	0.018524	0.238857	0.262817	0.080322	0.246177	0.007974	0.077268	0.436381	0.16695
Q13630	GDP-L-fuco	GFUS SDR4	0.872075	1.036949	0.45972	0.552707	0.545655	0.73594	0.679667	0.578466	0.546797	0.922667
Q13642	Four and a	FHL1 SLIM1	-0.22239	-0.77649	-0.53138	-0.55087	-0.49139	-0.73151	-0.3322	-0.63743	-0.49584	-0.41799
Q13685	Angio-asso	AAMP	-1.67102	-1.56786	-1.4181	-1.71151	-2.41022	-1.9532	-0.46713	-0.6644	-1.95976	-2.09818
Q13724	Mannosyl-(	MOGS GCS	1.136495	1.118558	0.39892	0.796181	0.309977	0.356649	0.952499	0.561749	0.076085	0.045242
Q13761	Runt-relate	RUNX3 AM	0.879969	1.221835	1.284002	0.984233	1.090085	1.066742	1.211388	1.469803	0.816754	0.843565
Q13765	Nascent po	NACA HSD4	1.026756	1.228243	0.359567	0.567201	0.387104	0.736433	0.501902	0.554649	0.921111	1.043679
Q13769	THO compl	THOC5 C22	-0.60942	-0.42286	-0.13523	0.102487	-0.20187	-0.43324	-0.28448	-0.32366	-0.18508	0.024739
Q13813	Spectrin al	SPTAN1 NE	0.073285	0.225542	-0.16506	0.055263	0.072079	0.107888	-0.10063	-0.05004	0.36459	0.330752

Q13823	Nucleolar G	GNL2 NGP1	0.873367	0.253253	-0.01568	0	0.422455	0.194011	0.257053	-0.09464	-0.1281	-0.40036
Q13825	Methylglut	AUH	-1.40296	-0.50348	-1.32193	-1.18728	-2.79256	-1.34395	-0.89162	-1.2091	-1.68753	-1.71937
Q13838	Spliceosom	DDX39B BA	1.867235	2.062566	1.800044	1.641169	1.931878	1.834496	1.827003	1.858994	1.78962	1.925436
Q13867	Bleomycin	BLMH	0.429108	0.516335	0.2886	0.42081	0.125338	0.859233	0.35488	0.581531	0.135734	0.229224
Q13868	Exosome co	EXOSC2 RR	-0.6227	-0.67862	-1.38154	-1.27932	-0.94719	-0.96566	-1.25798	-0.93951	-1.33448	-0.76205
Q13884	Beta-1-synt	SNTB1 SNT	-0.31699	0.488442	0.045286	-0.28085	0.329333	-0.42236	-0.26211	0.293011	-0.50827	0.283365
Q13895	Bystin	BYSL ENP1	0.034655	-0.03025	0.249874	0.146148	0.365368	0.183806	0.147465	0.16942	0.208584	0.086473
Q13905	Rap guanin	RAPGEF1 G	-2.45319	-1.93197	-1.5541	-1.75803	-1.07635	-1.66935	-3.01027	-1.64539	-1.67243	-2.08194
Q13951	Core-bindir	CBFB	-0.22239	-0.152	0.767827	0.783867	0.482236	0.435386	0.690171	0.410737	0.789736	0.833666
Q13952	Nuclear tra	NFYC	0.802079	0.802865	0.668883	0.894897	0.838406	1.042288	0.999637	0.821164	0.738362	0.93114
Q14004	Cyclin-depe	CDK13 CDC	-1.55497	-0.90202	-1.38163	-1.41923	-1.2076	-1.5366	-1.66382	-1.10692	-1.37474	-1.1589
Q14005	Pro-interle	IL16	-1.05099	-0.70714	-0.26301	-0.33048	0.107446	0.356485	0.081985	0.178092	-0.01743	-0.10392
Q14008	Cytoskelet	CKAP5 KIAA	-0.09398	-0.0699	-0.14707	0.143284	-0.15291	-0.37729	0.150467	0.241889	-0.38026	-0.17031
Q14011	Cold-induci	CIRBP A18H	-1.53727	-1.35371	-1.02359	-0.52356	-0.22487	-0.635	-0.70492	-0.79737	-0.75754	-0.77887
Q14019	Coactosin-l	COTL1 CLP	0.879969	0.869498	0.695824	0.809146	0.685489	0.656046	1.117836	1.031149	0.67925	0.503021
Q14103	Heterogene	HNRNPD A	1.242091	1.141468	0.969089	0.743225	1.007067	0.555912	1.190537	0.73465	1.188969	1.272738
Q14119	Vascular er	VEZF1 DB1	0.047697	-0.19153	-0.36146	-0.00873	-0.11715	0.20817	0.108743	-0.31547	-0.70288	-0.72536
Q14137	Ribosome b	BOP1 KIAA	-1.60942	-1.37151	-1.96153	-1.8011	-1.74193	-1.56866	-2.11718	-3.07039	-1.93546	-1.47836
Q14141	Septin-6	SEPTIN6 KI	-0.40296	-0.11353	-0.45873	-0.05765	0.01005	0.048363	-0.33379	-0.26107	-0.17325	0.03065
Q14142	Tripartite n	TRIM14 KIA	-0.96699	-1.25589	0.399356	0.043253	-1.13556	-1.48883	-0.97507	-1.20047	-0.22662	-0.91848
Q14146	Unhealthy	URB2 KIAA	-1.15335	-1.2185	-0.86605	-0.92663	-0.66918	-0.9783	-0.59266	-0.93289	-1.4245	-0.76377
Q14147	Probable A	DHX34 DDX	-3.06215	-3.58397	-1.54624	-2.3943	-3.19302	-1.77846	-1.87362	-2.18574	-1.96773	-1.77179
Q14149	MORC fam	MORC3 KIA	-0.25949	-0.07739	-0.60617	-0.57895	-0.40123	-0.18045	0.220785	-0.01636	-0.65654	-0.3709
Q14151	Scaffold att	SAFB2 KIAA	-0.12005	0.072314	0.503	0.255026	0.158799	0.123912	0.283908	0.569615	0.661475	0.424629
Q14152	Eukaryotic	EIF3A EIF3S	-0.01217	0.118315	0.226857	0.142847	0.159271	-0.12369	0.380085	0.18564	0.386468	0.273324
Q14155	Rho guanin	ARHGEF7 C	0.480222	0.488209	1.218488	0.875609	0.84717	0.785329	0.26984	-0.03477	0.822097	0.549117
Q14157	Ubiquitin-a	UBAP2L KIA	-0.60942	-0.45457	0.158617	-0.28325	-0.0594	0.043069	-0.378	-0.39232	-0.22396	-0.2507
Q14160	Protein scri	SCRIB CRIB	-1.06332	-1.18181	-0.94143	-0.86044	-1.01998	-0.74992	-1.43632	-0.92769	-1.19922	-1.06359
Q14161	ARF GTPase	GIT2 KIAA0	-0.22311	-0.25465	-0.09675	-0.22545	-0.17285	0.406817	-0.10928	0.083416	-0.44854	-0.45246
Q14165	Malectin	MLEC KIAA	0.560509	0.485427	-0.39183	-0.19444	-0.28703	-0.55254	0.094068	-0.79737	-1.03822	-1.19898
Q14166	Tubulin--ty	TTLL12 KIA	-0.97867	-0.22385	0.547563	0.440327	0.130907	0.245816	0.626383	0.591981	0.469585	0.648725
Q14186	Transcripti	TFDP1 DP1	-0.0786	0.056481	0.224236	0.11106	-0.09811	0.207984	-0.03404	-0.0715	0.090186	-0.09319
Q14197	Peptidyl-tR	MRPL58 DS	0.06936	0.078491	-0.157	-0.28198	-0.5279	-0.31131	-0.08284	-0.23474	0.044976	0.169187

Q14203	Dynactin su	DCTN1	-0.39497	-0.18709	-0.13478	-0.23816	-0.02248	0.021695	-0.313	-0.11309	0.139217	0.280628
Q14204	Cytoplasmi	DYNC1H1 D	0.35161	0.465798	0.25979	0.497745	0.302007	0.212994	0.340229	0.205056	0.099836	0.117811
Q14232	Translation	EIF2B1 EIF2	0.20909	0.363022	0.330722	0.114058	-0.0964	0.09131	0.007472	0.056385	0.354156	0.436667
Q14240	Eukaryotic	EIF4A2 DDX	0.3367	0.25473	0.353027	0.092777	0.01314	0.005775	0.21106	0.080424	0.513874	0.677839
Q14241	Elongin-A (	ELOA TCEB	-1.57975	-1.53027	-1.02909	-1.41389	-1.54308	-1.10653	-1.84285	-1.47678	-1.18065	-0.8046
Q14242	P-selectin g	SELPLG	1.588998	1.575836	1.081487	1.321928	1.300954	0.93002	1.200299	0.820382	0.981017	1.217326
Q14247	Src substra	CTTN EMS1	-1.03684	-1.38163	-1.07195	-1.48794	-0.71727	-1.38618	-1.68012	-1.53434	-1.15936	-1.97369
Q14254	Flotillin-2 (I	FLOT2 ESA1	0.249318	0.543879	0.218261	0.315656	0.595369	0.337997	0.247985	0.203792	0.748237	0.702601
Q14257	Reticulocal	RCN2 ERC5	-0.87442	-0.39623	-0.98467	-0.67113	-1.47654	-0.91873	-0.7741	-0.70831	-0.78165	-0.82833
Q14258	E3 ubiquitii	TRIM25 EFI	0.24984	0.291986	0.182799	0.329436	0.170938	0.370467	0.194718	0.25634	0.467984	0.406866
Q14289	Protein-tyr	PTK2B FAK2	0.121991	0.077938	0.001074	-0.03171	0.575013	-0.05004	0.079617	0.131245	0.338802	0.362078
Q14318	Peptidyl-pr	FKBP8 FKBI	1.697639	1.910525	1.603045	1.561778	1.65983	1.895185	1.762824	1.686278	1.463711	1.327229
Q14331	Protein FR(	FRG1	0.03592	0.144576	0.27275	0.171318	0.065423	0.125531	-0.08427	0.415037	0.073529	-0.12473
Q14344	Guanine nu	GNA13	0.290058	0.553427	-0.18709	0.411898	0.567425	0.921211	0.336182	0.321928	-0.18593	-0.57394
Q14353	Guanidinoa	GAMT	-0.18746	-0.31706	-0.86605	-0.67557	-0.97056	-0.55254	-0.81762	-0.71049	-0.41818	-0.29888
Q14376	UDP-glucos	GALE	-1.68536	-1.18265	-1.84488	-0.56937	-0.6574	-1.14958	-0.63176	-1.22239	-1.38702	-1.31531
Q14444	Caprin-1 (C	CAPRIN1 G	-0.38702	-0.14768	-0.37656	-0.62632	-0.269	-0.35789	-0.55459	-0.5764	-0.35652	-0.19898
Q14457	Beclin-1 (C	BECN1 GT1	-0.67503	-0.6757	-0.39677	-0.36573	-0.21289	-0.58735	-0.65157	-0.49043	-0.3654	-0.33033
Q14498	RNA-bindir	RBM39 HC(	-0.18871	-0.01568	0.126255	0.24285	0.072356	-0.00324	0.079633	-0.28033	0.103482	0.174834
Q14511	Enhancer o	NEDD9 CAS	-1.59104	-2.00782	-2.75697	-3.03906	-2.51245	-2.04439	-2.38368	-3.38113	-1.89984	-3.06333
Q14520	Hyaluronar	HABP2 HGI	-3.5025	-2.3363	-1.81642	-2.17557	-1.69302	-2	-2.55459	-1.80749	-2.11787	-1.95641
Q14527	Helicase-lik	HLTF HIP11	-1.19438	-0.95395	-0.93892	-1.23078	-0.24236	-0.81558	-1.09516	-1.24936	-1.2687	-0.9141
Q14558	Phosphorib	PRPSAP1	-0.39247	-0.54963	-1.10317	-0.82697	-0.83048	-0.85756	-0.92792	-0.77248	-0.71762	-0.64417
Q14562	ATP-depen	DHX8 DDX8	-1.70499	-1.5541	-1.88753	-3.0906	-2.69302	-3.04439	-1.18127	-1.22725	-2.45003	-3.13927
Q14566	DNA replica	MCM6	-0.40296	-0.38163	-1.01568	-0.73697	-1.33313	-1.34395	-0.782	-1.18286	-1.2916	-0.96502
Q14571	Inositol 1,4	ITPR2	-2.2073	-0.74853	-1.20437	-1.25011	-1.0162	-1.0085	-1.48134	-1.97728	-1.29498	-1.07263
Q14573	Inositol 1,4	ITPR3	0.202801	0.302985	-0.028	-0.53207	0.217337	-0.0917	0.220451	0.079072	-0.16891	0.010505
Q14643	Inositol 1,4	ITPR1 INSP	-0.88947	-0.96317	-0.77668	-1.4165	-1.49366	-1.56331	-2.09317	-1.26585	-1.76875	-1.8772
Q14644	Ras GTPase	RASA3	-0.49486	-0.49986	-0.96723	-0.75521	-1.22708	-1.00478	-1.28729	-1.1643	-0.95309	-0.99547
Q14653	Interferon	IRF3	0.526247	0.779895	-0.25821	-0.26824	-0.56931	-0.5606	0.147627	-0.38647	-0.16074	-0.24759
Q14657	EKC/KEOPS	LAGE3 DXS	-2.06193	-1.81714	-1.061	-1.46829	-1.38091	-0.68733	-0.65441	-0.62831	-0.82346	-0.66012
Q14669	E3 ubiquitii	TRIP12 KIA	-0.77145	-0.81714	-0.75002	-0.85681	-1.28137	-1.75899	-1.35819	-1.13179	-1.3012	-1.01781
Q14671	Pumilio hoi	PUM1 KIAA	-4.08746	-2.38163	-3.24598	-2.98751	-2.79256	-3.45943	-3.38466	-2.76783	-2.23502	-2.00888

Q14676	Mediator o	MDC1 KIAA	-1.27811	-0.87813	-0.40952	-0.65615	-0.65432	-0.93648	-1.09516	-1.58814	-0.66493	-0.46634
Q14677	Clathrin int	CLINT1 ENT	0.10557	0.095719	0.647223	0.183358	0.199579	0.30224	0.134358	0.217719	0.382345	0.244348
Q14678	KN motif a	KANK1 ANK	-3.37126	-1.91667	-2.02359	-1.26053	-0.87199	-1.31647	-1.43911	-0.85834	-1.45003	-1.30433
Q14683	Structural r	SMC1A DXS	0.406735	0.414985	0.462872	0.324072	0.458531	0.451012	0.4825	0.530515	0.592758	0.495561
Q14684	Ribosomal	RRP1B KIAA	-0.27277	-0.06786	-0.26927	-0.32193	-0.14048	-0.35789	-0.16674	-0.30861	-0.17524	-0.29345
Q14690	Protein RRI	PDCD11 KIA	-0.71835	-0.63051	-0.90933	-0.68157	-0.64572	-0.74061	-0.47422	-0.54265	-0.67996	-1
Q14692	Ribosome l	BMS1 BMS	-1.24905	-0.85482	-0.44592	-0.4813	-0.83749	-0.51827	-0.3389	-0.5566	-0.43118	-0.54021
Q14694	Ubiquitin c	USP10 KIAA	-3.57289	-2.46529	-3.53138	-3.84549	-2.37719	-2.54081	-3.3322	-3.27684	-3.30319	-4.26127
Q14696	LRP chaper	MESD KIAA	-0.17372	-0.38672	0.33311	-0.17014	0.10743	0.182024	0.022038	-0.04217	0.210385	-0.00829
Q14697	Neutral alp	GANAB G2A	0.615347	0.749953	0.277126	0.225484	0.100527	0.151074	0.345029	0.132815	-0.03775	-0.10212
Q14699	Raftlin (Cel	RFTN1 KIAA	0.402616	0.265839	0.401689	0.377697	0.410165	-0.07751	0.476266	0.401749	-0.0192	0.027652
Q14738	Serine/thre	PPP2R5D	-0.79226	-0.48549	-0.58364	-0.72086	-1.61342	-0.99604	-0.94824	-0.47759	-1.05137	-0.96707
Q14739	Delta(14)-s	LBR	1.777524	1.617622	0.61973	0.945673	0.276604	0.495421	0.578125	0.670179	0.126316	-0.05642
Q14746	Conserved	COG2 LDLC	-2.80735	-3.44392	-3.88753	-3.45317	NA	NA	NA	NA	-4.16993	-3.39453
Q14761	Protein tyr	PTPRCAP L	0.088176	0.797293	0.141044	-0.15899	-0.269	0.079727	0.186878	-0.09464	-0.33852	-0.03585
Q14764	Major vaul	MVP LRP	0.52264	0.413757	0.431515	0.193429	0.154975	0.259387	0.226771	0.246722	0.578663	0.549117
Q14765	Signal trans	STAT4	-1.14734	-1.03095	-0.87684	-0.86069	-1.12241	-0.87236	-0.63001	-0.63647	-1.31868	-1.41622
Q14789	Golgin subf	GOLGB1	-1.30986	-1.05857	-1.13379	-1.02531	-0.83088	-0.92183	-1.13564	-0.83523	-0.86966	-0.97979
Q14790	Caspase-8 (	CASP8 MCH	-0.35735	-0.86838	-0.49712	-0.42764	-0.94177	-0.59323	-0.89416	-0.98721	-0.78311	-0.86509
Q14839	Chromodo	CHD4	0.414303	0.427707	0.431454	0.399497	0.475813	0.588564	0.560738	0.192645	0.168877	0.509082
Q14847	LIM and SH	LASP1 MLN	0.648766	0.410274	0.416124	0.321926	0.516095	0.360386	0.341643	0.14406	0.112574	0.379862
Q14919	Dr1-associa	DRAP1	0.158197	0.053332	0.206042	-0.1336	0.324337	0.159587	0.117836	0.384664	0.442405	0.38922
Q14966	Zinc finger	ZNF638 NP	-0.20133	-0.27395	0.112475	0.037153	-0.30343	-0.31741	-0.16801	-0.13989	-0.01421	-0.06959
Q14974	Importin su	KPNB1 NTF	0.177233	0.352219	0.452109	0.544349	0.306978	0.461023	0.552427	0.500068	0.301033	0.442056
Q14978	Nucleolar a	NOLC1 KIAA	0.269949	0.401455	0.049352	0.030975	0.406865	0.193599	0.323921	0.274183	0.337566	0.31843
Q14980	Nuclear mi	NUMA1 NM	-0.38392	-0.30006	-0.12241	-0.27588	-0.03767	-0.16175	-0.13502	-0.14758	0.152253	0.132329
Q14BN4	Sarcolemm	SLMAP KIA	-0.85042	-0.84488	-0.54839	-0.79021	-0.51245	-0.73151	-0.73867	-0.59358	-0.93546	-1.11991
Q14C86	GTPase-act	GAPVD1 GA	-1.70499	-1.30256	-1.1264	-1.90302	-1.47063	-0.72247	-1.18536	-1.43812	-1.46773	-1.29676
Q14CW9	Ataxin-7-lik	ATXN7L3	-3.37126	-2.69849	-1.19153	-2.63604	-1.84503	-1.65208	-1.81762	-1.6735	-1.78165	-1.58054
Q15005	Signal pept	SPCS2 KIAA	0.029689	0.004727	0.186323	0.044985	-0.23087	-0.19858	0.207458	-0.31586	-0.50557	-0.31973
Q15006	ER membra	EMC2 KIAA	-2.94062	-2.15634	-2.48699	-3.38606	NA	NA	-2.96963	-2.88982	-2.93546	-3.54137
Q15007	Pre-mRNA-	WTAP KIAA	-0.18772	-0.11912	-0.11418	-0.02593	-0.19359	-0.33471	-0.16858	-0.31315	-0.12117	-0.17949
Q15008	26S protea	PSMD6 KIA	0.839892	0.799535	0.27275	0.533888	0.559958	0.431339	0	0.35811	0.411497	0.442435

Q15014	Mortality fa	MORF4L2 K	-2.53727	-1.88753	-2.5541	-2.61577	-1.62263	-1.52083	-2.16227	-1.46949	-3.1281	-2.30433
Q15018	BRISC comp	ABRAXAS2	-0.64814	-0.63879	-0.71646	-1.21613	-0.60806	-0.52869	-0.77495	-0.47055	-0.55166	-0.54622
Q15019	Septin-2 (N	SEPTIN2 DI	-0.22948	-0.39696	-0.16955	-0.08172	-0.19903	0.14543	-0.29649	-0.35569	-0.14868	0.014081
Q15020	Squamous	SART3 KIAA	0.024045	0.046047	0.079643	0.024875	-0.15697	0.169925	0.040358	0.299595	0.032631	0.090064
Q15022	Polycomb p	SUZ12 CHE	-0.63246	-0.33475	-0.9571	-0.81607	-1.14647	-0.68256	-0.902	-0.83574	-0.3757	-0.7125
Q15024	Exosome co	EXOSC7 KIA	-1.07357	-0.60489	-1.04687	-0.75982	-0.69166	-0.99601	-1.26096	-0.97228	-1.02838	-0.47919
Q15025	TNFAIP3-in	TNIP1 KIAA	0.344377	0.166862	0.892785	0.851477	0.699579	0.6386	0.322756	0.498454	0.882164	0.70384
Q15027	Arf-GAP wi	ACAP1 CEN	0.324351	0.288798	-0.07605	-0.34564	-0.47063	0.133677	-0.07346	-0.42979	-0.3505	-0.59384
Q15029	116 kDa U5	EFTUD2 KIA	0.495109	0.548103	0.890432	0.981667	1.007645	1.07194	0.767595	0.916274	1.172768	1.090413
Q15042	Rab3 GTPa	RAB3GAP1	0.372668	0.574746	0.8635	0.783268	0.948115	0.903595	0.558567	0.610061	0.905129	0.891146
Q15046	Lysine--tRN	KARS1 KAR	0.165949	0.183203	0.33311	0.248857	0.508612	0.3723	0.476012	0.478416	0.445804	0.334001
Q15047	Histone-lys	SETDB1 ESI	-1.55497	-1.39183	-0.98449	-1.63604	-1.14048	-1.41504	-1.13955	-1.34792	-0.65006	-0.94785
Q15050	Ribosome b	RRS1 KIAA0	-0.33258	0.01165	-0.57719	-0.38457	-0.269	-0.16175	-0.45305	-0.27684	-0.38702	-0.32636
Q15052	Rho guanin	ARHGEF6 C	-0.0747	0.203328	-0.17383	-0.35364	-0.38065	-0.37245	-0.27453	-0.23187	-0.56432	-0.20465
Q15056	Eukaryotic	EIF4H KIAA	0.319027	0.95362	1.221835	1.212502	1.368754	1.037755	1.459712	1.042311	1.295281	1.110703
Q15057	Arf-GAP wi	ACAP2 CEN	-1.13869	-1.08445	-0.82402	-1.13124	-0.96857	-1.0333	-1.24473	-1.16993	-1.11274	-0.93934
Q15059	Bromodom	BRD3 KIAA0	0.291615	0.286619	0.710977	0.794796	1.348454	1.410933	0.243868	0.363662	0.510869	0.608202
Q15061	WD repeat	WDR43 KIA	-0.34902	-0.29463	-0.6893	-0.36733	-0.37627	-0.23635	-0.45721	-0.52986	-0.40465	-0.21422
Q15075	Early endos	EEA1 ZFYVE	-0.43576	-0.517	-0.09675	-0.42764	-0.62263	-0.59318	-0.35819	-0.56789	-0.2916	0.243729
Q15084	Protein dis	PDIA6 ERP5	0.155828	0.285602	0.464211	0.477823	-0.11091	0.021695	-0.01012	0.200774	-0.32075	-0.12307
Q15102	Platelet-act	PAFAH1B3	-1.82873	-1.68589	-1.12199	-0.92663	-2.89947	-1.0902	-1.83577	-1.72935	-1.58496	-1.77887
Q15118	[Pyruvate c	PDK1 PDHK	-0.18057	0.097975	-1.02359	-1.20163	-0.27798	-0.73151	-0.782	-0.93289	-0.53629	-0.79414
Q15120	[Pyruvate c	PDK3 PDHK	-1.36548	-0.90037	-1.46815	-1.55985	-1.68505	-0.80551	-1.06547	-0.90316	-0.93727	-1.27636
Q15121	Astrocytic p	PEA15	0.138605	0.351262	0.680507	0.512062	0.31895	0.439422	0.456638	0.51056	0.742612	0.75718
Q15149	Plectin (PC	PLEC PLEC1	-0.15074	-0.09259	0.414062	0.21482	0.192833	0.110424	0.218001	0.246722	0.365899	0.39902
Q15154	Pericentrio	PCM1	-1	-0.69849	-0.4761	-0.69574	-0.91341	-0.62654	-1.06654	-1.28936	-1.21399	-1.35908
Q15170	Transcripti	TCEAL1 SIIF	-1.02445	-0.77649	-1.06378	-1.73697	-0.68105	-0.80599	-1.17377	-0.922	-1.96585	-1.99916
Q15172	Serine/thre	PPP2R5A	0.734539	0.744743	1.151613	0.981058	0.788104	1.112953	0.814645	1	1.214287	0.637114
Q15181	Inorganic p	PPA1 IOPPI	0.710309	0.52355	0.489405	0.338968	0.516725	0.299709	0.392333	0.305871	0.811941	0.978931
Q15185	Prostagland	PTGES3 P23	0.171271	0.264168	0.854481	0.743225	0.81794	0.865499	0.638526	0.407678	0.858956	0.801019
Q15208	Serine/thre	STK38 NDR	0.329184	0.416986	0.189718	-0.01894	0.258068	0.475242	-0.04152	0.326501	0.181404	0.081724
Q15233	Non-POU d	NONO NRB	-0.34792	-0.09675	0.21092	-0.22154	0.253767	0.356485	0.26984	0.303491	0.497782	0.320157
Q15257	Serine/thre	PTPA PPP2	-0.08181	0.304633	0.150097	0.746231	0.389057	0.486535	0.76317	0.709017	0.44631	0.538001

Q15269	Periodic try	PWP2 PWP	-2.3987	-2.35716	-3.24598	-3.32193	-3.01998	-3.09095	-3.05209	-3.33342	-2.35754	-1.97589
Q15276	Rab GTPase	RABEP1 RA	-0.01217	-0.53754	0.006484	-0.13472	-0.05302	-0.40612	-0.09516	-0.03477	0.197939	0.008824
Q15283	Ras GTPase	RASA2 GAP	-0.15049	-0.04423	0.008902	-0.59224	0.035326	0.0555	-0.0327	-0.13727	-0.09713	-0.16155
Q15286	Ras-related	RAB35 RAB	1.141356	1.030861	0.91983	0.64235	0.739937	0.877447	0.893872	0.817136	0.711279	0.573113
Q15287	RNA-bindir	RNPS1 LDC	0.744507	0.861549	0.880573	0.550835	0.942631	0.680172	0.870114	0.981464	1.223029	1.214627
Q15291	Retinoblast	RBBP5 RBC	0.716207	0.288798	-0.09777	-0.08579	1.562478	1.548063	1.26984	1.014242	0.056806	0.084935
Q15293	Reticulocal	RCN1 RCN	-0.61728	-0.41898	-0.61065	-0.7475	-1.22865	-1.01955	-0.71416	-0.68421	-0.69824	-0.93964
Q15311	RalA-bindir	RALBP1 RLI	-0.18746	-0.2185	-0.4761	-0.32979	-0.06853	-0.51301	-0.378	-0.79737	-0.74169	-0.00443
Q15326	Zinc finger	ZMYND11 I	-0.60018	-0.54209	-0.67977	-0.73529	-0.37821	-0.50017	-0.63937	-0.56764	-0.43721	-0.80642
Q15334	Lethal(2) gi	LLGL1 DLG4	-1.84329	-1.14084	-1.0994	-1.37255	-2.36268	-1.17485	-1.03437	-1.18699	-1.47212	-1.15459
Q15345	Leucine-ric	LRRC41 MU	-3.03684	-4.62449	-2.28345	-2.8011	-2.69302	-3.95693	-3.55459	-3.02308	-2.16993	-3.1008
Q15363	Transmeml	TMED2 RN	-0.25096	-0.58301	-0.82743	-0.18015	-0.68105	0.029208	-0.7214	0.067114	-0.9445	-1.0268
Q15365	Poly(rC)-bir	PCBP1	0.310934	0.039707	0.130397	-0.152	-0.08713	0.212994	0.136573	0.531031	0.249944	0.199599
Q15366	Poly(rC)-bir	PCBP2	0.380892	0.421717	0.312941	0.228219	-0.58442	-0.17529	-0.06859	-0.14179	-0.12038	-0.24885
Q15369	Elongin-C (I	ELOC TCEB	-2.12815	-1.82602	-1.78495	-1.60965	-1.58028	-1.47742	-2.80866	-1.7286	-1.45685	-1.27385
Q15370	Elongin-B (I	ELOB TCEB	1.442804	1.471434	1.558731	1.407175	1.45537	1.725444	1.693339	1.752341	1.4975	1.565657
Q15388	Mitochond	TOMM20 K	0.763394	0.474243	0.744743	0.209792	0.648015	0.82905	0.500694	0.415037	0.658492	0.186547
Q15393	Splicing fac	SF3B3 KIAA	-0.0183	0.189718	-0.1305	-0.19444	0.041378	-0.15577	-0.25691	-0.22909	0.069042	0.371017
Q15404	Ras suppre	RSU1 RSP1	-1.04351	-1.26654	-0.53703	-0.23816	-1.77439	-1.21056	-0.41845	-0.61973	-2.12224	-1.36859
Q15418	Ribosomal	RPS6KA1 M	-1.33018	-0.65498	-1.0097	-1.4394	-0.73936	-2.02425	-1.38751	-2.44964	-1.09769	-1.49505
Q15424	Scaffold att	SAFB HAP H	0.280108	0.378512	0.112475	0.091148	0.354283	0.184425	0.06502	0.284815	0.393664	0.356531
Q15427	Splicing fac	SF3B4 SAP4	1.100987	0.96446	0.718619	0.914459	0.901297	0.773408	0.925073	0.924492	0.829441	0.616915
Q15428	Splicing fac	SF3A2 SAP6	-0.0332	0.484649	0.448351	0.878697	0.73969	0.481922	0.589988	0.559874	0.349942	0.625376
Q15435	Protein pho	PPP1R7 SD	0.234465	0.502042	0.391927	0.432284	0.216301	0.048363	0.387926	0.275386	0.411497	0.690191
Q15436	Protein tra	SEC23A	-0.04307	-0.00782	-0.88753	-1.02531	-1	-0.80599	-0.44606	-0.90046	-0.67243	-0.82517
Q15437	Protein tra	SEC23B	0.671236	0.772399	0.92805	0.580775	0.838879	0.889297	0.456638	0.817136	-0.00946	-0.20407
Q15438	Cytohesin-:	CYTH1 D17	-0.83229	-1.13056	-0.14955	-0.7381	-1.86458	-0.05176	-0.13211	-0.16107	-0.26002	-0.14962
Q15459	Splicing fac	SF3A1 SAP:	0.580916	0.643276	0.437219	0.273451	0.405712	0.506353	0.368842	0.490326	0.499915	0.792422
Q15477	Helicase SK	SKIV2L DD)	-0.05302	0.208232	-0.25331	-0.40843	0.109013	0.068417	0.123576	-0.05574	0.346996	0.514008
Q15527	Surfeit locu	SURF2	0.116409	0.375509	0.298341	-0.18728	0.416895	0.41913	0.654865	0.222392	0.463711	0.081724
Q15528	Mediator o	MED22 SUI	0.727234	0.790547	1.055458	0.626185	0.895942	0.717157	0.743092	0.905363	0.767752	0.853396
Q15532	Protein SS)	SS18 SSXT S	2.248565	2.206711	2.388227	1.922694	2.18799	2.186827	2.379102	2.168652	2.508313	2.234355
Q15542	Transcriptio	TAF5 TAF2I	-0.30677	-0.46682	-0.2978	-0.39941	-0.37728	-0.28421	-0.4636	-0.24739	-0.08007	-0.18697

Q15545	Transcripti	TAF7 TAF2I	-0.9523	-1.09675	-0.99222	-1.3377	-0.9852	-1.37197	-1.08427	-0.82753	-1	-1.03585
Q15554	Telomeric r	TERF2 TRBI	-0.13619	-0.04536	-0.01908	-0.46907	-0.19573	-0.17387	0.142779	-0.20869	0.238266	-0.14558
Q15555	Microtubul	MAPRE2 RI	0.684331	0.687654	0.980152	0.828659	0.823785	0.58506	0.923061	0.864034	0.579913	0.87814
Q15560	Transcripti	TCEA2	-0.1669	-0.45992	-0.05971	-0.20887	-0.00746	0.016302	-0.4957	-0.24257	-0.09248	-0.26127
Q15628	Tumor nec	TRADD	-0.01374	-0.06703	0.33894	0.219007	0.222595	0.137239	0.291591	0.275156	-0.43715	-0.6595
Q15629	Translocati	TRAM1 TRA	0.032665	0.14094	0.327417	-0.2447	-0.43965	-0.17704	0.225737	-0.00503	-0.59667	-0.37384
Q15631	Translin (E	TSN	0.102359	0.305418	-0.02451	0.86824	0.962996	0.762155	0.654154	0.815722	0.450945	0.14952
Q15637	Splicing fac	SF1 ZFM1 Z	-0.24125	-0.19006	-0.17631	-0.36746	-0.36075	-0.4119	-0.25691	-0.15758	0.192019	0.256899
Q15642	Cdc42-inte	TRIP10 CIP	-3.19438	-2.38163	-2.32193	-3.84549	NA	NA	-3.3322	-2.93289	-2.23502	-3.0268
Q15646	2'-5'-oligoa	OASL TRIP1	-0.71491	-1.1305	-1.06378	-1.2909	-1.37109	-1.16175	-1.60039	-1.26303	-1.88235	-2.32636
Q15648	Mediator o	MED1 ARC	-1.35566	-1.11353	-1.30256	-1.3377	-0.91341	-1.17403	-1.73016	-1.29078	-1.10764	-1.2507
Q15650	Activating	TRIP4	-1.04854	-1.05058	-0.84866	-0.481	-0.69523	-0.90431	-1.05653	-1.04025	-1.37743	-1.06318
Q15651	High mobil	HMGN3 TR	0.597035	0.59274	-0.20495	0.15451	1.519028	1.70044	-0.01543	-0.28962	1.320041	1.223024
Q15653	NF-kappa-E	NFKBIB IKB	1.672893	1.888249	1.773198	1.66615	1.626594	1.572162	1.606466	1.745863	1.312008	1.35972
Q15691	Microtubul	MAPRE1	0.886542	0.574527	0.917984	0.778483	0.966141	0.573991	0.724635	0.246722	0.597201	0.885486
Q15696	U2 small n	ZRSR2 U2A	0.171271	0.363436	0.27275	0.294061	0.564994	0.31755	0.532874	0.61891	0.327575	0.106599
Q15697	Zinc finger	ZNF174 ZS	-2.68536	-1.93147	-2.42286	-3.67557	-2.35199	-1.83494	-1.41163	-2.72935	-2.04793	-1.73402
Q15717	ELAV-like p	ELAVL1 HU	0.088435	-0.31221	-0.04757	-0.23816	0.062459	0.048363	-0.06825	-0.08246	0.023383	0.11867
Q15750	TGF-beta-a	TAB1 MAP	-3.89482	-3.72403	-3.36146	-4.14505	-3.51245	-2	-3.3322	-3.27684	-2.28011	-3.44184
Q15773	Myeloid le	MLF2	-0.76553	-0.57138	-1.26459	-0.82312	-0.81855	-0.86449	-0.94973	-1.34792	-0.77357	-0.84888
Q15785	Mitochond	TOMM34 U	-1.01217	-0.97679	-0.59474	-0.63604	-0.7051	-0.79647	-1.34514	-1.33342	-0.96277	-0.98241
Q15796	Mothers ag	SMAD2 MA	-2.26348	-2.54777	-1.87949	-1.95748	-2.1163	-2.18727	-2.14237	-2.00529	-2.09713	-1.62622
Q15814	Tubulin-spe	TBCC	-0.44361	0.087004	-0.16506	-0.47045	-0.27798	-0.42968	-0.10063	-0.20249	-0.07748	-0.52855
Q15819	Ubiquitin-c	UBE2V2 MI	1.241889	1.092962	1.375641	1.542863	1.553527	1.505868	1.3623	1.37811	1.787957	1.935127
Q15833	Syntaxin-bi	STXBP2 UN	-0.38711	-0.34827	-0.51457	-0.32193	-0.54463	0.058894	-0.62372	-0.55063	-0.99365	-0.97915
Q15836	Vesicle-ass	VAMP3 SYE	-3.43539	-2.60064	-1.96153	-2.0126	-2.55552	-2.91511	-2.9301	-2.16993	-2.23502	-1.58054
Q15848	Adiponecti	ADIPOQ AC	1.690145	1.471434	1.416986	0.349267	0.7175	1.328471	1.880039	0.941583	0.619129	1.019778
Q15853	Upstream s	USF2 BHLH	-2.50619	-2.42635	-2.91039	-1.93063	-0.77973	-1.26303	-2.25298	-2.43388	-0.91754	-0.9141
Q15906	Vacuolar pi	VPS72 TCFI	-3.30986	-1.87317	-2.10512	-2.9386	-3.69302	-1.75899	-3.38466	-1.78746	-2.25739	-2.37145
Q15907	Ras-relatec	RAB11B YP	-0.29947	0.004953	0.105491	0.01227	0.048434	-0.20107	-0.22544	-0.28832	-0.25263	0.005111
Q15942	Zyxin (Zyxi	ZYX	-0.27277	-0.35506	0.390459	-0.25865	-0.33209	-0.08599	-0.24938	-0.36234	-0.00472	0.209693
Q16134	Electron tr	ETFDH	-0.21534	-0.25061	-0.58301	-0.22344	-0.74193	0.016302	-0.41163	-0.65535	-0.55003	-0.79414
Q16181	Septin-7 (C	SEPTIN7 CD	-0.13993	0.095745	0.152411	0.006259	0.14828	0.155278	0.015267	-0.13444	0.189695	0.090064

Q16186	Proteasom	ADRM1 GP	-1	-1.14138	-0.84488	-0.7245	-0.57755	-0.25196	-0.18536	-0.7189	-0.58496	-0.71937
Q16204	Coiled-coil	CCDC6 D10	-1.60249	-1.27623	-1.10557	-1.58065	-0.91511	-1.43044	-1.05794	-0.93494	-1.51726	-1.08
Q16254	Transcripti	E2F4	-0.86675	-0.83427	-0.21941	-0.2663	-0.11181	-0.1545	-0.55014	-0.33766	-0.21873	0.011581
Q16401	26S protea	PSMD5 KIA	0.485852	0.429275	0.7314	0.647325	0.566307	0.810512	0.463066	0.727078	0.941163	1.116431
Q16512	Serine/thre	PKN1 PAK1	-0.41722	-0.18507	-0.29556	-0.18702	-0.23087	-0.57277	-0.41052	-0.5876	-0.39073	-0.30926
Q16514	Transcripti	TAF12 TAF1	0.430035	0.538899	0.536398	0.544235	0.501493	0.658123	0.474435	0.64333	0.780446	0.47296
Q16531	DNA dama	DDB1 XAP1	0.455795	0.654643	0.172676	0.259617	0.346395	0.247911	0.201905	0.019101	0.43291	0.348239
Q16537	Serine/thre	PPP2R5E	-2.89482	-2.57719	-3.24598	-3.32193	NA	NA	-3.61599	-2.27684	-3.45003	-3.76377
Q16539	Mitogen-ac	MAPK14 CS	0.540882	0.61705	-0.02618	-0.10407	-0.46004	-0.29491	-0.10947	-0.12683	-0.41143	-0.09878
Q16543	Hsp90 co-c	CDC37 CDC	1.024045	0.915702	1.175978	1.13179	1.124601	1.005454	0.921145	1.011405	1.258203	1.194304
Q16555	Dihydropyr	DPYSL2 CRI	-0.63743	-0.39696	-0.11775	-0.34564	-0.63413	-0.52083	-0.43911	-0.6735	-0.45003	-0.52218
Q16560	U11/U12 sı	SNRNP35 H	-3.03684	-3.72403	-2.57719	-1.84549	-2.74193	-3.04439	-3.18536	-2.93289	-3.3505	-2.30433
Q16563	Synaptoph	SYPL1 SYPL	0.144101	0.030861	-0.03953	0.977877	0.739937	0.439422	0.08445	1.421464	-0.03339	0.783129
Q16566	Calcium/ca	CAMK4 CAI	-0.22302	-0.30896	0.037224	0.397684	-0.00879	0.194438	0.417193	0.256642	0.375067	0.480043
Q16576	Histone-bir	RBBP7 RBA	0.333608	0.356539	0.23017	0.260844	0.174874	0.458287	0.299241	0.56642	0.553184	0.543056
Q16594	Transcripti	TAF9 TAF2(	-1.38702	-0.73697	-0.83094	-1.06413	-0.56649	-0.68684	-0.62385	-0.6644	-0.96277	-0.53495
Q16595	Frataxin, m	FXN FRDA X	1.546253	1.686034	0.112493	0.543067	0.289536	0.519391	0.370222	0.236769	0.244053	0.313217
Q16611	Bcl-2 homc	BAK1 BAK E	-2.11872	-2.15019	-2.69716	-2.68228	-2.95606	-2.28114	-2.13885	-2.5561	-1.39941	-1.13927
Q16629	Serine/argi	SRSF7 SFRS	-0.08746	0.108863	0.165586	-0.17661	-0.2076	-0.44448	-0.07346	-0.39985	-0.16993	0.29873
Q16630	Cleavage ai	CPSF6 CFIN	-0.49028	-0.41017	-0.578	-0.44965	-0.46454	-0.58723	-0.4193	-0.61586	-0.41358	-0.14203
Q16637	Survival mc	SMN1 SMN	-0.94062	-1.16506	-0.8519	-0.7794	-0.81855	-0.82523	-1.03103	-1.55102	-1.31487	-1.22979
Q16643	Drebrin (De	DBN1 D0S1	-2.47758	-1.469	-1.46545	-0.89197	-1.13251	-0.8933	-0.69214	-0.68117	-1.40204	-1.11799
Q16644	MAP kinase	MAPKAPK3	-0.61869	-0.58301	-0.53138	-0.50564	-0.64572	-0.50524	-0.36476	-0.55943	-0.28011	-0.41209
Q16656	Nuclear res	NRF1	-0.35566	-0.55984	-0.50348	-0.48794	-0.50188	-0.35789	-0.30035	-0.41504	-0.22396	-0.53495
Q16666	Gamma-int	IFI16 IFNGI	0.514573	0.165586	0.510189	0.501498	0.154975	0.096281	0.269722	0.20884	-0.05282	-0.24544
Q16698	2,4-dienoyl	DECR1 DEC	0.381307	0.63101	0.571047	0.543409	0.06614	0.335723	0.406977	0.351236	0.661478	0.258722
Q16718	NADH deh	NDUFA5	1.930141	2.002921	2.0925	1.957026	2.287308	1.735325	1.976793	1.903834	2.401716	2.123963
Q16740	ATP-depen	CLPP	-0.06707	-0.18709	0.162105	-0.03814	-0.1487	-0.18021	0.122543	0.217477	0.009399	0.232472
Q16773	Kynurenine	KYAT1 CCB	-0.26546	0.038474	-0.55984	-0.27563	-0.87199	-0.35091	-0.6477	-0.62855	-0.22948	-0.14906
Q16774	Guanylate	GUK1 GMK	0.233097	0.298975	0.454059	0.176397	0.466317	0.408229	0.637983	0.553764	0.564188	0.334228
Q16775	Hydroxyacy	HAGH GLO	-0.50368	-0.46554	-0.01633	-0.4386	-0.04534	-0.08543	-0.09305	-0.0444	0.140784	0.093464
Q16777	Histone H2	H2AC20 H2	-1.74236	-0.84149	0.045988	0.237327	0.35868	-0.30531	0.327709	0.46664	-0.65974	0.652943
Q16778	Histone H2	H2BC21 H2	-1.03684	-1.23676	1.424268	1.359081	1.18799	1.386058	1.584963	1.268273	1.130629	1.161047

Q16795	NADH dehy	NDUFA9 NI	0.996582	1.191439	0.961242	0.931943	0.567198	0.932221	1.072083	0.996036	0.855108	0.959546
Q16822	Phosphoen	PCK2 PEPC	-1.76524	-2.23644	-0.29776	-0.38606	-1.99154	-2.44028	-0.04152	0.028345	-2.09755	-2.09051
Q16831	Uridine pho	UPP1 UP	0.523035	0.566651	0.390459	0.225972	0.161638	0.295456	0.26559	0.340133	0.206137	0.455403
Q16836	Hydroxyacy	HADH HAD	0.38809	0.550431	0.860936	0.181558	0.749107	1.095157	0.522838	0.812078	1.119116	0.79593
Q16851	UTP--gluco	UGP2 UGP1	1.013777	0.655971	0.477047	0.547006	0.589912	0.649093	0.397587	0.340133	0.848521	1.139112
Q16854	Deoxyguan	DGUOK DG	-2.06193	-2.03953	-1.75002	-1.7794	-3.69302	-1.79647	-2.09516	-2.88982	-1.7028	-1.70487
Q16864	V-type prot	ATP6V1F AT	-0.75527	-0.59474	-0.96153	-0.70594	-1.37109	-1.1375	-0.58811	-0.53269	-0.9445	-0.84093
Q16875	6-phosphof	PFKFB3	-2.10516	-1.69616	-2.15645	-2.66233	-1.74926	-1.65065	-2.26023	-2.35213	-2.6018	-1.5353
Q16881	Thioredoxi	TXNRD1 GF	1.06089	0.955142	0.836925	0.813154	0.929368	1.031382	0.951714	0.844345	0.527355	0.590178
Q16891	MICOS com	IMMT HMF	0.299939	0.625965	0.343262	0.439171	0.331666	0.340888	0.464603	0.559718	0.139781	0.254373
Q17R31	Putative de	TATDN3	-2.03684	-2.38163	-1.4021	-1.54171	-2.64572	-1.65208	-1.96963	-1.63743	-1.47603	-1.1589
Q1KMD3	Heterogene	HNRNPUL2	0.27001	0.491081	0.424451	0.294061	0.575013	0.679634	0.686479	0.704729	0.54686	0.419456
Q27J81	Inverted fo	INF2 C14or	-0.8797	-0.81206	-0.49351	-0.78772	-0.08475	0.000683	-0.30904	-0.18616	-0.04325	0.158979
Q29RF7	Sister chro	PDS5A KIA	-0.68116	-0.83967	-0.83094	-0.48276	-0.62136	-0.62654	-0.90115	-0.74897	-0.7489	-0.56737
Q2KHT3	Protein CLE	CLEC16A KI	-0.02445	-0.10512	0.015513	-0.17306	0.072512	0.079727	-0.10063	0.303491	0.09134	0.052151
Q2M2I8	AP2-associ	AAK1 KIAA1	-1.52507	-1.34474	-1.35426	-1.49332	-1.09806	-1.03243	-1.34801	-1.10235	-1.01898	-1.38294
Q2NL82	Pre-rRNA-p	TSR1 KIAA1	0.503272	0.467881	0.433568	0.316213	0.446466	0.836992	0.883443	0.826638	0.210016	-0.13601
Q2PZI1	Probable C	DPY19L1 G	-0.73495	-1.46529	-1.33171	-0.41086	-0.87199	-0.59318	-1.20881	0.207595	-0.45649	-0.42392
Q2TAA2	Isoamyl ace	IAH1	1.567374	1.633751	0.800934	1.234105	1.13149	1.700455	1.098968	1.01394	1.38135	1.123358
Q2TAL8	Glutamine-	QRICH1	-1.12663	-1.27399	-1.81714	-1.67557	-1.66918	-2.11366	-1.74723	-1.93289	-1.73383	-1.28264
Q2TAY7	WD40 repe	SMU1	0.60874	0.327734	0.894883	0.674146	0.766409	0.944291	0.797087	1.083416	1.164677	1.021959
Q2TBE0	CWF19-like	CWF19L2	-0.9523	-1.32193	-0.88753	-0.87971	-0.69302	-1.33015	-0.91073	-0.73888	-0.67996	-1.03585
Q32MZ4	Leucine-ric	LRRFIP1 GC	-0.28011	-0.18709	0.329705	-0.20287	0.161638	-0.05094	-0.56212	-0.01728	0.060025	-0.1008
Q32NC0	UPF0711 p	C18orf21 X	-1.78629	-1.50901	-1.5656	-1.61667	-1.57755	-1.61813	-1.96963	-1.58496	-1.46297	-1.66223
Q32P44	Echinoderm	EML3	-1.242	-0.85271	-0.31221	-0.3377	-0.87611	-0.92244	-0.18536	-0.26303	-0.69194	-0.5468
Q3B726	DNA-direct	POLR1F TW	-0.51978	-0.10512	-0.33171	-0.19444	-0.42	0.155278	-0.25691	-0.14439	-0.38087	-1.11032
Q3B7J2	Glucose-fru	GFOD2 UN	-1.82873	-2.03953	-1.94642	-3.14505	-2.59991	-1.75899	-2.11718	-2.848	-1.95361	-1.45391
Q3B7T1	Erythroid d	EDRF1 C10	0.944386	1.240108	1.77468	1.672179	1.669214	1.477206	1.741563	1.820382	1.659984	1.679178
Q3KQU3	MAP7 dom	MAP7D1 KI	-2.57289	-1.84488	-1.5212	-1.29944	-1.81855	-1.91511	-2.04156	-1.48864	-1.89556	-1.1589
Q3KQV9	UDP-N-ace	UAP1L1	-1.1669	-1.63656	-3.72403	-4.84549	-2.64572	-1.93587	-1.58496	-1.53434	-1.5025	-1.84093
Q3LXA3	Triokinase/	TKFC DAK	-1.47755	-0.93768	-1.17824	-2.21415	-0.87363	-0.79749	-1.73447	-2.79248	-0.9163	-0.6335
Q3MHD2	Protein LSM	LSM12	0.229304	0.199938	1.20502	1.034093	1.134797	0.989029	0.942598	0.982722	1.456858	1.482579
Q3SXM5	Inactive hy	HSDL1 SDR	-1.32496	-0.91667	-0.44392	-0.71621	-0.75442	-1.27621	-1.39808	-1.09464	-0.96277	-0.93934

Q3YEC7	Rab-like pr	RABL6 C9o	-0.09392	-0.12624	0.308355	0.308759	0.010881	0.039335	0.248466	0.246907	0.266089	0.348838
Q3ZCM7	Tubulin bet	TUBB8	0.646137	0.695415	0.884887	0.622587	0.878116	0.783455	1.036522	0.747077	0.63278	0.678701
Q3ZCQ8	Mitochond	TIMM50 TI	-1.39286	-1.40072	-0.82437	-0.66562	-0.75535	-0.97475	-1.07113	-0.81727	-0.66327	-0.92986
Q460N5	Protein mo	PARP14 BA	-0.11346	-0.51457	-0.05971	-0.26806	-0.41009	-0.24349	-0.51019	-0.97728	-0.36257	-0.22461
Q49A26	Putative ox	GLYR1 HIBI	0.974858	0.895148	0.790547	0.785929	0.761284	0.958594	0.877633	0.784497	0.969024	0.617409
Q49AR2	UPF0489 p	C5orf22	-0.51978	-0.7632	-0.53138	-0.59756	-0.38074	-0.85457	-0.44606	-0.48543	-0.55003	-0.52218
Q4G0F5	Vacuolar pı	VPS26B	-0.09565	0.194546	-0.50567	-0.24969	-0.24774	-0.27397	-0.37918	-0.16356	-1.12508	-0.81209
Q4G0I0	Protein CCS	CCSMST1 C	0.218924	-0.29298	0.034673	-0.0973	0.072512	-0.10776	0.108376	-0.06439	0.185556	-0.1589
Q4G0J3	La-related	LARP7 HDC	0.03592	0.206711	0.540081	0.268252	0.072512	0.189226	-0.30666	-0.2091	0.446516	-0.03132
Q4G0N4	NAD kinase	NADK2 C5c	-1.41772	-1.70552	-1.42757	-2.01282	-1.35775	-0.65945	-0.6866	-0.77858	-1.80809	-0.89867
Q4G163	F-box only	FBXO43 EN	0.203839	0.738868	1.056822	1.39097	2.223166	1.889505	1.061147	1.448979	1.560539	1.48379
Q4G176	Malonate--	ACSF3 PSE(	-0.87682	-1.25035	-0.94454	-1.41031	0.871762	0.964035	-0.85119	-1.03797	0.814075	0.751934
Q4KMP7	TBC1 doma	TBC1D10B	-0.83954	-0.36146	0.220163	0.209792	-0.15697	-0.21785	-0.27537	-0.32623	0.368321	0.351712
Q4KMQ2	Anoctamin	ANO6 TME	0.059379	-0.19153	-0.13478	0.182416	-0.10007	0.203533	0.117836	0.303491	-0.44361	-0.61402
Q4LE39	AT-rich inte	ARID4B BR(	-1.32822	-1.15197	-0.70865	-0.35981	-0.6755	-0.74482	-1.0155	-1.33936	-0.69873	-0.45305
Q4V328	GRIP1-asso	GRIPAP1 KI	-1.5025	-1.26459	-0.83377	-1.0906	-1.29613	-1.07677	-0.86343	-0.86876	-0.7028	-0.99118
Q4VC31	Coiled-coil	CCDC58	0.371969	0.451612	0.137504	0.354182	0.539639	-0.08439	0.598541	0.126008	0.473931	0.590851
Q52LJ0	Protein FAI	FAM98B	-0.14852	-0.75659	0.101614	0	-0.19903	0.352211	0.301824	0.199138	0.457381	0.284267
Q53EL6	Programme	PDCD4 H73	-0.16561	-0.22458	-0.06378	-0.26053	-0.25118	-0.26961	-0.30666	-0.39985	-0.71049	-0.30981
Q53ET0	CREB-regul	CRTC2 TOR	-1.89482	-1.4021	-2.5541	-3.32193	-1.66918	-1.87447	-1.61599	-1.56789	-2.39941	-1.70487
Q53F19	Nuclear ca	NCBP3 C17	-1.17779	-1.22554	-1.5427	-1.45317	-1.29589	-1.41158	-1.42313	-1.36705	-1.47734	-1.26127
Q53GL7	Protein mo	PARP10	-0.91509	-0.96103	-1.1639	-1.19101	-1.55762	-1.63469	-0.69075	-0.78199	-1.46632	-1.62609
Q53GQ0	Very-long-c	HSD17B12	-0.37912	-0.65486	-0.34651	-0.7794	-0.53382	-0.2962	-0.46007	-0.6644	-1.01898	-1.87299
Q53GS7	Nucleopori	GLE1 GLE1I	-3.80735	-2.44392	NA	NA	-3.2076	-4.04439	-2.89162	-3.27684	-2.06756	-3.21945
Q53GS9	U4/U6.U5 t	USP39 CGI-	0.905643	0.950081	0.970487	0.835589	0.648966	0.689755	0.880304	0.838806	0.975332	0.932043
Q53H12	Acylglycerc	AGK MULK	-0.53727	-0.78319	-0.58301	-0.73697	-1.12418	-0.89465	-0.9898	-0.95491	-1.2687	-1.09134
Q53H47	Histone-lys	SETMAR	0.36257	0.610726	0.137504	0.618034	0.051139	0.203533	0.332124	0.506536	0.304855	0.709264
Q53QV2	Protein LBH	LBH	-1.04814	-0.68596	-1.52259	-1.41978	-1.54003	-1.50758	-1.32081	-1.53549	-1.92797	-1.78929
Q53QZ3	Rho GTPas	ARHGAP15	-0.56957	-0.24788	-0.45937	-0.46136	-0.29941	-0.31673	-0.41907	-0.40842	-0.29749	-0.42726
Q562E7	WD repeat	WDR81	-1.01217	-1.4761	-0.97679	-0.89129	-0.06075	-0.4975	-0.61599	-0.60224	0.069042	-0.13441
Q562R1	Beta-actin-	ACTBL2	1.406448	1.102579	1.276633	1.750234	1.685141	1.541584	1.113289	1.760401	1.332912	1.790095
Q567U6	Coiled-coil	CCDC93	-0.53621	-0.64947	-0.42692	-0.26361	-0.19717	-0.21782	-0.37859	-0.91707	-0.18671	-0.10322
Q56VL3	OCIA doma	OCIAD2	-1.70499	-2.20945	-2.32193	-2.98751	-2.74193	-3.40054	NA	NA	-2.3505	-1.69052

Q5BJF6	Outer dens	ODF2	-1.09882	-0.62638	-0.24302	-0.00861	-0.41283	-0.50711	-0.6256	-0.47412	-0.43394	-0.40651
Q5BKU9	Oxidoreduc	OXLD1 C17	0.053549	0.419903	0.58756	0.572362	0.570012	0.703799	0.595158	0.648429	1.069042	0.991122
Q5C9Z4	Nucleolar N	NOM1 C7o	-0.46855	-0.46529	-0.69849	-0.23816	-0.66918	-0.38618	-0.60817	-0.22909	-0.60634	-1.04495
Q5EBM0	UMP-CMP	CMPK2	0.506062	0.26628	0.101614	0.120294	-0.27798	0.490881	0.074768	-0.02891	0.770518	1.052151
Q5EG05	Caspase re	CARD16 CC	-0.0646	-0.16522	-0.0473	-0.34356	-0.22755	-0.1745	-0.12443	-0.18497	-0.24587	-0.34762
Q5F1R6	DnaJ homo	DNAJC21 D	-0.89482	-0.76983	-0.4761	-0.49676	-0.53382	-0.93587	-0.5697	-0.45372	-0.2687	-0.10555
Q5GLZ8	Probable E	HERC4 KIA	0.42711	0.285602	0.468619	-0.05759	-0.02248	0.415037	0.411195	0.340133	0.323813	0.320157
Q5H9R7	Serine/thre	PPP6R3 C1	-1.30549	-1.52529	-0.5252	-1.00005	-1.22634	-1.30653	-0.8442	-1.27192	-0.74001	-0.71574
Q5HYI8	Rab-like pr	RABL3	1.552881	1.565506	1.533183	1.364367	1.505104	1.4738	1.264612	1.386574	1.349781	1.432695
Q5HYJ3	Protein FAI	FAM76B	-0.28011	-0.08017	-2.26459	-2.84549	-1.26747	-1.44809	-1.39212	-1.67085	-1.4209	-1.10818
Q5HYW2	NHS-like pr	NHSL2	-1.56393	-1.17201	-0.47651	-0.77683	-0.58054	-0.88852	-0.84233	-0.50602	-2.45164	-1.6028
Q5J8M3	ER membra	EMC4 TME	-1.28622	-0.77362	-1.53837	-1.64746	-2.16937	-1.55397	-1.45858	-1.77925	-2.22401	-1.72669
Q5JPI9	EEF1A lysin	EEF1AKMT	-0.5285	-0.35147	-0.5427	-0.23816	-0.66918	-0.37197	-0.23868	-0.43812	-1.01898	-1.0726
Q5JRA6	Transport a	MIA3 KIAA	-0.75527	-0.43335	-0.31706	-0.26806	-0.5231	0.154002	-0.45305	-0.55943	-0.81444	-0.51584
Q5JRX3	Presequenc	PITRM1 KIA	0.385953	0.275973	0.295167	-0.18015	-0.32949	-0.20134	0.76433	0.565752	0.168877	-0.2402
Q5JS54	Proteasom	PSMG4 C6c	-1.1004	-1.5427	-1.85896	-1.0906	-0.97056	-1.52083	-1.91073	-1.18286	-1.39941	-1.2402
Q5JSH3	WD repeat	WDR44	-0.12487	-0.32914	-0.31221	-0.62632	-0.48054	-0.23195	-0.53996	-0.42764	-0.23685	-0.31401
Q5JSL3	Dedicator c	DOCK11 ZI	-0.30986	-0.4761	0.157901	0.097024	0.029444	-0.0614	-0.14172	0.055091	-0.07756	0.075852
Q5JSP0	FYVE, RhoG	FGD3 ZFYV	-0.18057	-0.13906	0.199938	-0.03814	0.200987	0.286523	0	-0.17638	0.422092	0.284267
Q5JSZ5	Protein PRI	PRRC2B BA	-3.98793	-4.28345	-1.41244	-3.84549	-3.14048	-1.81558	-1.9898	-1.61973	-1.848	-2.1008
Q5JTD0	Tight juncti	TJAP1 PILT	-3.55861	-2.92089	-2.2461	-3.17557	-3.14048	-2.02203	-1.9898	-2	-2.76553	-2.38004
Q5JTH9	RRP12-like	RRP12 KIA	0.195964	0.023496	-0.21708	-0.4278	-0.09862	0.021686	-0.00031	0.02834	-0.21395	-0.18925
Q5JTJ3	Cytochrom	COA6 C1or	-1.54089	-1.15879	-1.38878	-1.74253	-2.03885	-1.67667	-1.61688	-1.54197	-1.76573	-1.04537
Q5JTV8	Torsin-1A-i	TOR1AIP1 I	-0.24022	-0.0056	-0.41209	-0.40255	-0.1144	0.040353	-0.43219	-0.64636	-0.38762	-0.64895
Q5JTZ9	Alanine--tR	AARS2 AAR	-0.818	-0.81714	-0.31221	0.593302	-0.62263	-0.23704	-0.3322	-0.71989	-0.5025	-0.62763
Q5JVF3	PCI domair	PCID2 HT0	0.45547	0.48963	-0.25558	0.207168	-0.23256	0.233904	0.229653	0.17345	-0.41911	-0.41293
Q5JVS0	Intracellula	HABP4	-0.36344	-0.16069	-0.08017	-0.45317	0.128008	0.105353	0.098853	-0.12553	0.032631	1.037134
Q5JXB2	Putative ub	UBE2NL	-0.22239	0.01165	-1.49796	-0.7794	-0.66918	-0.50524	-0.21474	-0.39232	-1.24616	-0.60054
Q5K651	Sterile alph	SAMD9 C7c	0.223066	0.186266	0.169379	-0.12159	0.103787	0.202885	0.088947	0.192967	0.220041	0.186325
Q5MIZ7	Serine/thre	PPP4R3B K	-3.25096	-3.53138	-3.62449	-3.32193	-1.69302	-3.65208	-3.18536	-3.16993	-2.97199	-2.34873
Q5QJ74	Tubulin-spe	TBCEL LRR	1.680721	1.659678	1.655971	1.241973	1.484896	1.529253	1.67102	1.51056	1.646494	1.458627
Q5QJE6	Deoxynucle	DNTTIP2 EI	-0.88359	-0.65486	-0.88753	-0.71621	-0.35199	-0.44448	-0.42694	-0.1671	-0.53629	-0.61402
Q5QNW6	Histone H2	H2BC18 HI	0.410579	1.086079	2.460469	2.122771	1.924746	1.596422	2.356149	2.434677	1.608127	1.534533

Q5R372	Rab GTPase	RABGAP1L	-0.98793	-1.12069	-0.67963	-0.8011	-1.10842	-0.86449	-0.86828	-0.65535	-1.25051	-1.64549
Q5RKV6	Exosome c	EXOSC6 MT	-0.9732	-0.71916	-0.16403	-0.27225	-0.05353	-0.1026	-0.39519	-0.09271	0.188534	0.287003
Q5SRE5	Nucleopori	NUP188 KIA	-1	-0.90004	-0.5541	-0.70594	-1.04532	-0.71348	-1.21046	-0.94386	-0.89107	-0.89752
Q5SRN2	Testis-expr	TSBP1 C6or	1.324351	1.533361	1.3725	1.446832	1.597304	1.321928	2.047801	1.446886	1.760812	1.884892
Q5SSJ5	Heterochro	HP1BP3	0.836501	0.742414	0.733584	0.772895	1.445464	0.930243	1.154389	0.933691	0.894836	0.852932
Q5SW79	Centrosom	CEP170 FA	-1.26922	-0.9391	-0.98904	-0.65094	-1.15974	-0.87447	-1.42094	-1.88577	-1.00554	-1.26236
Q5SY16	Polynucleo	NOL9	0.155031	0.253253	0.323487	0.462849	0.666874	0.666412	0.467779	0.321928	0.702614	1.073337
Q5T011	KICSTOR cc	SZT2 C1orf	-4.08746	-4.00782	-1.7632	-2.71621	-3.3702	-3.36123	-3.049	-3.11028	-3.14031	-2.91198
Q5T013	Putative hy	HYI HT036	1.949103	2.081487	1.189718	1.421296	1.757639	1.577658	1.560888	1.528532	1.599038	1.429349
Q5T0F9	Coiled-coil	CC2D1B KIA	-1.127	-1.1866	-1.08558	-1.02165	0.981288	1.404755	1.117836	1.152003	-1.1818	-0.48181
Q5T1C6	Acyl-coenz	THEM4 CTM	-0.17471	-0.04543	-0.40302	-0.39313	0.143756	0.181292	-0.59516	-0.69295	-0.04733	0.08851
Q5T1M5	FK506-bind	FKBP15 KIA	-0.34775	-0.20805	-0.01183	-0.35364	-0.16528	-0.28465	0.061055	-0.05111	-0.13069	-0.13927
Q5T200	Zinc finger	ZC3H13 KIA	-2.14639	-1.75673	-1.36146	-1.47045	-2.47063	-2.34502	-2.80866	-1.97728	-1.9906	-1.54137
Q5T2T1	MAGUK p5	MPP7	-0.53566	-0.46698	-0.41897	-0.38534	-0.01691	-0.2996	-0.28829	-0.17971	0.08216	0.117177
Q5T3I0	G patch do	GPATCH4 C	-1.51978	-1.84488	-2.05565	-3.03814	-2.84503	-2.91511	-1.71329	-3.07039	-1.86507	-1.8893
Q5T440	Putative tra	IBA57 C1or	0.160465	0.034673	-1.21773	-0.90699	-1.91077	-0.99185	-0.80246	-0.84539	-1.03592	-0.93162
Q5T4S7	E3 ubiquiti	UBR4 KIAA	-0.68536	-0.45457	-0.26929	-0.41086	-0.58869	-0.79647	-0.41886	-0.76783	-0.62803	-0.59384
Q5T5Y3	Calmodulin	CAMSAP1	-1.91572	-1.0873	-0.71801	-0.912	-1.29972	-0.82545	-0.96921	-0.86694	-0.67541	-0.90559
Q5T601	Adhesion G	ADGRF1 GP	-0.68536	-0.6734	-0.20046	-0.51457	-0.76702	-0.45943	-0.37137	-0.33342	-0.30902	1.118877
Q5T653	39S riboso	MRPL2 CGI	-1.0025	-0.90812	-1.10864	-1.07204	-1.16531	-1.00515	-0.96775	-1.18497	-1.06824	-0.98137
Q5T6J7	Probable gl	IDNK C9orf	0.906082	1.038474	0.375509	0.471923	0.079567	-0.1375	0.352302	0.162271	-0.23502	-0.08663
Q5T6V5	Queuosine	C9orf64	-1.52861	-0.97024	-1.72952	-2.37773	-1.61465	-1.96559	-1.19333	-1.16428	-1.22504	-1.05912
Q5T8P6	RNA-bindin	RBM26 C13	-0.03641	0.01995	0.075	0.246017	0.127831	0.235563	-0.11676	0.22874	0.228429	0.527482
Q5T9A4	ATPase fan	ATAD3B KIA	1.106091	1.218195	1.235103	1.218372	1.295866	1.20401	1.136802	1.069697	1.249631	1.10958
Q5T9C2	Protein FAI	FAM102A C	-2.89482	-1.5541	-2.54975	-2.53998	-2.04174	-2.14072	-3.25712	-2.73891	-2.26857	-1.93031
Q5TAX3	Terminal ur	TUT4 KIAA	-2.60942	-1.34156	-0.63051	-1.2909	-0.81855	-0.91511	-1.46713	-1.37737	-1.15936	-0.94785
Q5TBB1	Ribonuclea	RNASEH2B	1.194797	1.197874	1.515366	1.368001	1.191621	1.046871	1.233693	1.163631	1.109735	1.108762
Q5TDH0	Protein DD	DDI2	-0.11944	0.13593	0.32842	0.458291	0.226454	0.285938	-0.00322	0.402508	0.53743	0.53361
Q5TEJ8	Protein TH	THEMIS2 C	-1.85334	-1.72129	-1.80716	-2.03032	-1.40375	-1.76969	-1.58504	-1.625	-1.62583	-1.46298
Q5TFE4	5'-nucleoti	NT5DC1 NT	1.855603	1.75898	1.248262	1.631881	1.633125	1.619137	1.564113	1.629616	1.328727	1.322314
Q5THJ4	Vacuolar pr	VPS13D KIA	-2.85042	-2.48699	-1.97679	-1.65567	-3.07635	-2.1375	-2.16227	-1.60224	-2.1913	-2.26127
Q5TYW1	Zinc finger	ZNF658	1.610994	1.687787	1.458723	1.47869	1.726517	1.797956	1.623661	1.557995	1.721323	1.463449
Q5TZA2	Rootletin (	CROCC KIA	-0.42742	-0.31706	-0.59822	-0.49849	-0.64929	-0.41504	-0.38355	-0.3919	-0.36561	-0.10708

Q5VIR6	Vacuolar pı	VPS53 PP1:	-1.70499	-1.12199	-2.10356	-1.7248	0.28273	0.570316	-1.90613	-1.67709	-0.03339	0.276981
Q5VSL9	Striatin-int	STRIP1 FAN	-0.74394	-0.42294	-0.41685	-0.25853	-0.23987	-0.43394	-0.84192	-0.42269	-0.61651	-0.72745
Q5VT06	Centrosom	CEP350 CA	-1.21714	-0.56398	-0.67105	-0.62713	-1.42624	-0.59335	-0.59268	-0.78915	-0.82559	-0.5929
Q5VT52	Regulation	RPRD2 KIA	-0.341	-0.06786	0.111816	0.042491	-0.01495	-0.02203	-0.04316	-0.10076	0.06454	0.059257
Q5VTL8	Pre-mRNA-	PRPF38B	-0.15335	-0.19153	-1.75241	-0.97644	-1.69222	-1.55575	0.113114	0.005714	-1.47472	-1.79914
Q5VTR2	E3 ubiquitiı	RNF20 BRE	-0.90119	-0.75487	-0.51055	-0.60358	-0.46036	-0.71348	-0.92198	-0.52892	-0.64693	-0.50324
Q5VTU8	ATP syntha	ATP5F1EP2	0.760972	0.726715	0.666024	0.777212	0.53601	0.849226	1.231755	1.144674	0.543505	0.425472
Q5VU43	Myomegali	PDE4DIP CI	-2.80735	-2.13906	-1.73697	-1.61667	-2.74193	-2.06711	-2.9301	-2.30485	-1.76553	-1.64829
Q5VV17	OTU domai	OTUD1 DU	-1.53727	-0.96914	-0.20495	-0.20163	-0.42999	-0.32329	-0.39136	-0.1193	-0.3445	-0.04495
Q5VV52	Zinc finger	ZNF691	-2.72489	-2.34156	-3.48699	-3.0906	-1.31451	-2.95693	-3.18536	-3.51785	-3.25739	-3.1008
Q5VW32	BRO1 dom	BROX BROI	-2.85042	-1.60064	-0.57719	-0.65567	-0.23359	-1.11366	-0.70492	-0.56789	-1.62803	-0.7121
Q5VWQ0	Lysine-spe	RSBN1	-1.24293	-1.06856	-0.71881	-1.00005	-1.05651	-0.8471	-0.76525	-1.27791	-1.13778	-0.68163
Q5VYK3	Proteasom	ECPAS ECM	-1.64689	-1.96153	-2.07195	-1.98751	-3.79256	-3.79647	-2.05209	-2.80735	-1.81444	-2.32636
Q5VZ89	DENN dom	DENND4C (	-2.17646	-1.37565	-0.32193	-0.21613	-0.54463	-0.52869	-2.11429	-1.7948	-0.35652	-0.25597
Q5VZL5	Zinc finger	ZMYM4 KIA	0.850016	0.756331	0.351262	0.693669	0.638492	0.669851	0.661349	0.659345	0.753839	0.831181
Q5W0V3	Protein FAI	FAM160B1	0.03592	0.01165	-0.13906	-0.03171	-0.63413	-0.45943	-0.32577	-0.42269	-0.88235	-0.81735
Q5W111	SPRY doma	SPRYD7 C1	0.572788	0.740082	0.507538	0.542527	0.739937	0.624491	0.693339	0.842899	0.520689	-0.01334
Q63HN8	E3 ubiquitiı	RNF213 AL	-0.4718	-0.32352	-0.37679	-0.36996	-0.53382	-0.23704	-0.20881	-0.41183	-0.50959	-0.3431
Q641Q2	WASH com	WASHC2A	-3.72489	-2.53138	-1.73697	-2.67557	-2.53078	-1.67366	-1.25824	-1.77957	-2.55855	-2.54229
Q658Y4	Protein FAI	FAM91A1	-2.68536	-1.02359	-0.16069	0.646363	0.014797	0.174774	-0.00513	0.039528	-0.52268	-0.32082
Q66K14	TBC1 doma	TBC1D9B K	-1.07464	-1.03953	-1.09675	-1.82312	-1.37109	-0.91511	-1.17377	-1.50155	-1.10764	-1.40621
Q66K74	Microtubul	MAP1S BPY	-0.76922	-0.62866	-0.07023	-0.2125	-0.3246	-0.16481	-0.69105	-1.07501	-0.35489	-0.21272
Q66K89	Transcripti	E4F1 E4F	-0.30986	-0.18265	-0.31221	-0.38606	-0.29613	-0.2962	-0.31937	-0.39985	-0.49584	-0.16882
Q66LE6	Serine/thre	PPP2R2D K	-0.31739	-0.01963	0.019365	0	-0.7051	-0.14958	-0.23868	-0.31194	-0.02857	-0.03132
Q66PJ3	ADP-ribosy	ARL6IP4	0.523035	0.369485	0.485427	0.236659	0.498119	0.381871	0.239827	0.34915	0.762203	0.811143
Q676U5	Autophagy	ATG16L1 A	-0.67451	-0.75062	-0.2095	-0.30717	-0.38714	-0.27846	-0.36731	-0.33755	-0.39705	-0.49537
Q684P5	Rap1 GTPa	RAP1GAP2	-3.58014	-3.94642	-2.15634	-2.67557	-1.69302	-1.7809	-3.20437	-3.74756	-3.5025	-3.1788
Q68CP9	AT-rich inte	ARID2 BAF	0.819428	0.790547	0.668291	0.209792	1.007417	0.801096	0.690171	0.871343	0.985786	0.75718
Q68CQ4	Digestive o	DIEXF C1or	-1.45187	-1.05565	-1.63656	-1.24558	-1.39046	-0.76827	-1.23266	-1.26303	-0.75754	-1.03585
Q68CZ6	HAUS augn	HAUS3 C4c	-1.02445	-0.92405	-0.15634	-0.32979	-0.1487	-0.37906	-0.37137	-0.31907	0.018737	-0.24544
Q68D20	Protein PM	PMS2CL PN	-1.74507	-1.44392	-1.77649	-1.89129	-2.51245	-1.47454	-2.96963	-2.04654	-1.46297	-2.41799
Q68DK7	Male-speci	MSL1 MSL1	-1.1669	-1.12199	-1.11353	-1.32193	-1.10806	-0.87447	-0.4957	-1.03477	-1.03822	-0.7121
Q68E01	Integrator (	INTS3 C1or	-1.32746	-1.64238	-1.49269	-1.31819	-0.83996	-1.83491	-0.85285	-1.17732	-1.19633	-0.64533

Q68EM7	Rho GTPas	ARHGAP17	-0.53727	-0.17823	-0.45874	-0.40055	-0.44667	-0.52177	-0.67319	-0.58241	-0.42845	-0.45854
Q69YN2	CWF19-like	CWF19L1	0.80427	0.790249	0.72733	0.556954	0.905242	0.621541	0.851344	0.475343	0.736422	0.753605
Q69YN4	Protein viri	VIRMA KIA	-3.13993	-1.68589	-1.88753	-1.71621	-2.64572	-3.04439	-3.18536	-3.07039	-2.28011	-1.50324
Q6DD87	Zinc finger	ZNF787	-1.941	-2.56233	-2.73373	-2.39346	-2.50019	-2.39842	-0.01543	-0.53434	-2.79471	-2.54971
Q6DD88	Atlastin-3 (	ATL3	0.029995	0.007777	0.042266	-0.3377	-0.23359	-0.2962	-0.02581	0.167378	-0.78978	-0.69052
Q6F5E8	Capping pr	CARMIL2 LI	-1.68077	-1.53106	-1.30758	-1.46181	-1.5978	-2.27819	-1.63232	-1.3323	-1.51587	-1.07619
Q6FI81	Anamorsin	CIAPIN1 CU	-0.7027	-0.57719	-0.48699	-0.38841	-0.75759	-0.66235	-0.55498	-0.71989	-0.52384	-0.32423
Q6GMV2	SET and MY	SMYD5 RAI	-0.27277	-0.09675	0.348202	-0.03171	-0.03753	-0.11677	0.022811	0.064667	0.20861	0.208834
Q6GMV3	Putative pe	PTRHD1 C2	-0.90613	-0.74348	-0.6672	-0.66558	-0.7051	-0.76827	-0.39808	-0.81741	-0.68753	-0.63448
Q6GPH4	XIAP-assoc	XAF1 BIRC4	-0.65641	-0.15634	-2.71284	-1.52672	-0.26006	-0.37197	-1.93063	-2.22017	-0.02377	-0.01334
Q6GYQ0	Ral GTPase	RALGAPA1	-3.89482	-3.44392	-2.53138	-3.45317	-2.84503	-1.93587	-1.94973	-3.72935	-3.39941	-3.1788
Q6I9Y2	THO compl	THOC7 NIF	1.109451	1.220533	1.374186	1.31281	1.228872	1.27255	1.256742	1.304043	1.534649	1.242125
Q6IA17	Single Ig IL-	SIGIRR UNC	-0.35797	-0.262	-0.43643	-0.94465	-0.7211	-0.62908	-0.66547	-1.0086	-0.83794	-0.87301
Q6IAA8	Ragulator c	LAMTOR1 (	-1.90618	-1.92742	-1.97647	-3.52537	-2.26724	-1.57204	-1.64237	-1.56879	-1.8505	-2.00987
Q6IBS0	Twinfilin-2	TWF2 PTK9	0.157886	0.116077	-0.38303	-0.29408	-0.33313	-0.36966	-0.37365	-0.45372	-0.4154	-0.28803
Q6ICL3	Transport a	TANGO2 C2	0.329184	0.295167	0.027039	0.666263	0.086588	0.373458	0.131912	0.177538	0.349942	-0.20918
Q6IN85	Serine/thre	PPP4R3A K	0.815989	1.009715	0.923616	0.646363	0.869326	0.977974	0.752232	0.75743	0.88977	0.67609
Q6IQ49	Replication	SDE2 C1orf	0.760299	0.82918	1.158308	0.745346	0.721921	0.811348	1.050231	1.035351	1.224479	1.191235
Q6JBY9	CapZ-intera	RCSD1 CAP	-0.1975	-0.13431	-0.80832	-0.97328	-0.54459	-0.8596	-0.64888	-0.65402	-0.66263	-0.48679
Q6K0P9	Pyrin and H	PYHIN1 IFIX	-1.292	-1.08825	-1.88753	-1.8011	-0.49139	-0.86449	-3.09516	-2.02308	-1.97293	-1.10835
Q6KC79	Nipped-B-li	NIPBL IDN3	-0.77307	-0.66834	-0.79667	-0.9629	-0.87728	-0.92102	-0.37606	-0.56789	-0.9445	-0.39453
Q6L8Q7	2',5'-phosp	PDE12	-1.60321	-1.20146	-1.18711	-1.69804	-1.38809	-1.34597	-1.07142	-1.31935	-1.07628	-1.2734
Q6MZP7	Protein lin-	LIN54 CXCD	-3.5025	-3.77649	-2.30256	-4.14505	-3.89947	-4.34395	-4.3322	-1.80735	-4.08746	-3.13927
Q6N069	N-alpha-ac	NAA16 NAR	-1.74507	-1.90202	-1.46529	-1.15899	-1.04532	-1.24998	-1.5697	-1.97728	-1.73383	-0.89752
Q6NTE8	MRN comp	MRNIP C5o	-2.53727	-2.17383	-1.77649	-3.45317	-3.42999	-3.28951	-2.16227	-2.93289	-1.78165	-1.77887
Q6NUK1	Calcium-bir	SLC25A24 A	0.121991	-0.03953	-0.35147	-0.30633	-1.12418	-0.11366	-0.27537	-0.5764	-1.04793	-1.22979
Q6NUM9	All-trans-re	RETSAT PPS	-0.80735	-0.35646	-0.61849	-0.82312	-1.35199	-0.93587	-0.18536	-0.52607	-1.83112	-1.38294
Q6NUQ4	Transmeml	TMEM214	-0.11346	-0.35147	-0.66102	-0.97513	-0.269	-0.90484	-0.5175	-0.37737	-1.24616	-0.98241
Q6NVY1	3-hydroxyis	HIBCH	1.26	1.554276	0.850161	1.296617	0.96233	1.084889	0.971584	1.036741	1.236468	1.29693
Q6NXR4	TELO2-inte	TTI2 C8orf4	-0.91238	-0.41665	-1.13204	-0.31675	-0.94581	-0.94825	1.336182	1.429988	-0.22722	-0.48926
Q6NYC8	Phostensin	PPP1R18 H	-0.16011	-0.23217	0.46028	0.481592	0.255211	0.082663	0.030128	0.074998	-0.2687	0.284043
Q6NZY4	Zinc finger	ZCCHC8	-0.5639	-0.36604	-0.2276	-0.61667	-0.5231	-0.54455	-0.4885	-0.56789	-0.87485	-0.63831
Q6P158	Putative AT	DHX57	-0.73329	-0.8142	-0.74987	-0.59008	-0.43684	-0.78704	-0.8721	-0.79607	-0.80277	-1.06493

Q6P179	Endoplasm	ERAP2 LRA	-0.10093	-0.16869	-0.32127	-0.14645	0.10304	0.197506	-0.58904	-0.25135	-0.34344	-0.58127
Q6P1J9	Parafibrom	CDC73 C1o	-2.85042	-2.08844	-1.20046	-0.45317	-1.26006	-1	-1.42531	-1.51785	-1.2916	-1.34873
Q6P1K2	Polyamine-	PMF1	-0.76553	-0.72403	-0.66102	-0.5881	-0.85845	-0.77761	-0.9898	-0.71989	-1.03822	-0.49698
Q6P1L5	Protein FAI	FAM117B /	-3.89482	-3.94642	NA	NA	-3.59991	-3	-4.23266	-4.07039	-2.1281	-3.21945
Q6P1L8	39S ribosor	MRPL14 M	-1.09515	-0.52892	-0.10509	0.138416	-0.40887	-0.65269	-0.57539	-0.99133	-0.43828	-0.28717
Q6P1N0	Coiled-coil	CC2D1A AK	-0.01217	0.003894	0.003894	0.288936	-0.10806	-0.05571	0.209177	0.136462	-0.4892	0.052151
Q6P1N9	Putative de	TATDN1 CD	1.083481	0.957845	0.705531	0.634833	0.821465	0.714817	0.799534	0.661264	1.304752	1.031616
Q6P1R4	tRNA-dihyd	DUS1L	0.014548	0.072049	0.5712	0.392717	0.361426	0.412407	0.30137	0.489704	0.511494	0.387728
Q6P2E9	Enhancer o	EDC4 HEDL	-0.62005	-0.06644	-0.16506	-0.46179	-0.29613	-0.0902	-0.45305	-0.1635	-0.18593	-0.1788
Q6P2H3	Centrosom	CEP85 CCD	-1.45187	-1.4021	-1.5427	-1.02531	-1.01495	-1.26303	-0.9898	-1.23581	-1.02857	-1.27191
Q6P2Q9	Pre-mRNA-	PRPF8 PRP(	0.348356	0.329705	-0.152	-0.0973	-0.13231	0.090032	0.005107	-0.2978	0.09134	0.186547
Q6P3X3	Tetratricop	TTC27	-1.02445	-1	-1.2185	-1.67557	-1	-0.90484	-1.81762	-1.40742	-1.31487	-1.27191
Q6P4R8	Nuclear fac	NFRKB INO	-0.92219	-0.39232	0.018313	-0.22715	-0.18704	-0.28544	0.040641	-0.1018	0.241119	-0.04391
Q6P587	Acylpyruva	FAHD1 C16	-0.26874	0.270319	0.07045	0.26311	0.369211	0.546192	0.615862	0.647332	0.371044	0.017832
Q6P589	Tumor neci	TNFAIP8L2	-0.94062	-0.63051	-0.44923	-0.36976	-0.66918	-0.48215	-0.42531	-0.74846	-0.89984	-1.26127
Q6P5X5	UPF0545 p	C22orf39	-3.08746	-3.83094	-2.2276	-1.67557	-4.14048	-1.83494	-3.28157	-1.69188	-2.1913	-1.76377
Q6P6C2	RNA demet	ALKBH5 AB	-0.36817	-0.39003	0.035149	0.039041	0.255874	0.221503	0.101072	0.214631	0.062713	0.16894
Q6P996	Pyridoxal-d	PDXDC1 KI/	-0.9523	-1.15634	-0.76983	-0.52356	-0.72955	-1.27621	-0.7217	-0.87925	-1.14886	-0.64137
Q6PCB8	Embigin	EMB	0.4932	0.52661	0.854481	1.13179	0.365871	0.227069	0.475158	0.644772	0.778786	0.619939
Q6PCE3	Glucose 1,6	PGM2L1 BI	-1.68536	-1.79681	-1.6269	-1.8928	-1.48942	-1.39823	-1.43818	-2.01376	-1.48442	-1.23477
Q6PD62	RNA polym	CTR9 KIAA(	-3.03684	-1.64874	-2.60064	-2.98751	0.054239	-1.36014	-1.02035	-1.27365	-1.74959	-1.77887
Q6PD74	Alpha- and	AAGAB	-1.4345	-1.16539	-1.26267	-1.35497	-0.56209	-0.58534	-0.94018	-0.78293	-1.62909	-1.4358
Q6PGP7	Tetratricop	TTC37 KIAA	-1.92834	-2.30064	-1.76764	-1.21879	-1.14048	-0.89465	-1.18536	-1.45372	-2.03903	-1.75862
Q6PI48	Aspartate--	DARS2	1.370797	1.567275	1.503356	1.424383	1.29326	1.6093	1.457113	1.281182	1.3186	1.097312
Q6PII3	Coiled-coil	CCDC174 C	-1.30986	-1.68589	-1.08844	-1.3377	-0.97056	-1.38618	-1.38466	-1.14439	-1.07748	-0.93934
Q6PIZ9	T-cell recep	TRAT1 TCR	-0.23328	-0.29734	-0.50094	-0.76744	-0.28562	-0.49504	-0.49799	-0.36748	-0.31358	-0.29957
Q6PJ69	Tripartite n	TRIM65	0.296893	0.008997	0.340892	0.405395	0.462484	0.728292	0.416065	0.490201	0.104192	0.106211
Q6PJG2	Mitotic dea	MIDEAS C1	-1.45187	-1.07195	-0.97679	-1.03814	-0.8055	-0.88452	-0.78836	-0.79309	-0.87368	-1.2507
Q6PJI9	GATOR con	WDR59 KIA	0.45635	0.486276	0.130128	0.10039	0.572079	0.632644	0.597599	0.266357	0.359188	0.311148
Q6PJT7	Zinc finger	ZC3H14	-0.67147	-0.39527	-0.69691	-0.64635	-0.72976	-0.84985	-0.8637	-0.75367	-0.82869	-0.71939
Q6PJW8	Consortin	CNST C1orf	-2.55148	-2.079	-2.31391	-3.00233	-0.84391	-0.98112	-1.00733	-1.84067	-1.38502	-2.4375
Q6PK04	Coiled-coil	CCDC137 c	1.719892	1.934185	2.182864	1.917722	1.895942	1.671568	1.761315	1.886906	1.816754	1.54609
Q6PKC3	Thioredoxi	TXNDC11 E	0.03592	-0.36146	-0.29776	-0.23816	-0.44004	-0.14958	-0.44606	0.039528	-0.09752	-0.08194

Q6PKG0	La-related	LARP1 KIAA	-0.73646	-1.21483	-1.27402	-0.89129	-1.07635	-1.17958	-0.31937	-0.46949	-0.87729	-0.54137
Q6PL18	ATPase fan	ATAD2 L16	-1.76553	-1.90202	-2.24598	-2.03814	-3.2076	-3.34395	-2.01027	-3.22239	-2.23502	-2.11991
Q6QNY0	Biogenesis	BLOC1S3 B	-1.08196	-1.04407	-1.6103	-1.77426	-1.01518	-1.29758	-1.39025	-1.30733	-1.10659	-1.05316
Q6QNY1	Biogenesis	BLOC1S2 B	-2.02916	-1.61355	-0.5656	-0.27563	-2.4855	-1.69028	-1.86707	-2.48864	-0.97062	-1.60011
Q6RFH5	WD repeat	WDR74 NS.	-0.68536	-0.69849	-0.1305	-0.30633	0.093574	-0.1315	-0.24473	-0.6735	0.222392	0.143125
Q6RW13	Type-1 ang	AGTRAP AT	-1.91373	-1.71984	-0.25526	-0.39428	-1.77798	-2.32008	-0.35165	-0.36257	-0.11784	-0.03585
Q6SPF0	Atherin (St	SAMD1	0.03592	0.079643	0.378512	0.031027	-0.10806	0.347923	0.159657	0.094182	0.273994	0.66808
Q6SZW1	NAD(+) hyc	SARM1 KIA	-1.27331	-0.71109	-1.03602	-0.5741	-1.05734	-0.82037	-0.75696	-0.50988	-0.85442	-0.53496
Q6UB98	Ankyrin rep	ANKRD12 A	-0.24377	0.034673	0.26628	0.15451	0.168271	0.352211	-0.11718	0.344648	0.382857	0.392739
Q6ULP2	Aftiphilin	AFTPH AFT	-1.84498	-1.53068	-1.72516	-2.49397	-2.55612	-2.66843	-2.72786	-2.35396	-1.7412	-1.78617
Q6UN15	Pre-mRNA	FIP1L1 FIP1	0.224123	0.253253	-0.37656	-0.2986	-0.88567	-0.45943	-0.378	-0.64636	-0.37474	-0.36004
Q6UUV7	CREB-regul	CRTC3 TOR	-2.94062	-2.53138	-1.97679	-3.38606	NA	NA	-2.03103	-1.848	-2.16993	-2.30433
Q6UUV9	CREB-regul	CRTC1 KIAA	0.05143	0.049962	0.187673	-0.04334	0.544601	0.488987	0.194773	0.414014	0.779348	0.546204
Q6UWE0	E3 ubiquitin	LRSAM1 TA	-1.60942	-1.23676	-0.44923	-0.73697	-1.22487	-1.01097	-0.84493	-0.68266	-0.78978	-0.86491
Q6UX04	Spliceosom	CWC27 SD(	-1.9019	-1.12952	-1.78458	-2.14612	-1.96652	-1.85416	-2.30866	-1.70383	-1.81999	-1.23839
Q6UXB8	Peptidase i	PI16 CRISP!	-1.37126	-1.2185	-0.93147	-0.92663	-1.89947	-1.85457	-1.26919	-1.37737	-1.68753	-2.2402
Q6UXH1	Protein dis	CRELD2 UN	0.154587	0.694965	1.000481	1.146873	0.927137	0.78837	0.959179	0.177866	0.828395	0.493529
Q6UXN9	WD repeat	WDR82 TM	0.960935	0.827232	0.229212	0.457799	0.557166	0.775877	0.673046	0.676267	0.495514	0.423256
Q6UXV4	MICOS com	APOOL CXo	0.889817	0.770115	1.370994	1.064403	0.973734	1.33931	1.204745	1.180067	1.014075	1.002211
Q6VEQ5	WAS protei	WASH2P FA	-0.34006	-0.35021	0.082622	-0.07826	-1.34843	-0.57154	0.67869	0.602158	0.185746	0.067667
Q6VMQ6	Activating t	ATF7IP MC.	-1.1004	-1.26459	-1.20046	-0.73697	-0.81855	-1.05571	-0.96963	-0.53434	-1.25739	-2.0268
Q6VN20	Ran-bindin	RANBP10 K	-3.08746	-2.08844	-1.13906	-3.14505	-1.74193	-1.30293	-2.16227	-1.82753	-1.39941	-1.6763
Q6VY07	Phosphofu	PACS1 KIAA	-1.1004	-0.96153	-0.91506	-1.19962	-1.01495	-0.95693	-1.04507	-1.01182	-0.95854	-1.03217
Q6WCQ1	Myosin pho	MPRIP KIAA	-1.04281	-1.37193	-0.64211	-0.64228	0.020613	-0.10825	-0.34207	-0.15288	-0.1546	-0.01752
Q6WKZ4	Rab11 fami	RAB11FIP1	-1.91754	-2.00782	-1.61252	-1.24558	-2.95606	-1.52083	-1.53673	-1.93554	-1.65748	-1.97369
Q6XQN6	Nicotinate	NAPRT FHII	-0.91211	-0.64269	-0.08638	0.00854	-0.46753	-0.3747	-0.03673	-0.32222	-1.05985	-1.07668
Q6Y2X3	DnaJ homo	DNAJC14 D	-0.82873	-0.62449	-0.37656	-1.23078	-1.39046	-1.28951	-0.83577	-0.95491	-1.46297	-0.75627
Q6Y7W6	GRB10-inte	GIGYF2 KIA	-1.60942	-1.02359	0.08672	-0.42905	-0.92749	-0.53349	-1.04152	-0.95491	-0.88235	-0.9141
Q6YN16	Hydroxyste	HSDL2 C9o	0.937566	0.682938	0.384789	0.220107	0.330134	0.495109	0.446706	0.619096	0.46457	0.212635
Q6YP21	Kynurenine	KYAT3 CCB	-0.02493	0.128084	-0.29596	-0.08863	-0.10613	0.169851	0.097159	-0.02127	-1.04216	-1.08188
Q6ZMI0	Protein pho	PPP1R21 C	0.082462	-0.2185	-0.27399	-0.72655	-0.37109	-0.26303	-0.37137	-0.36257	-0.23502	-0.01334
Q6ZMT4	Lysine-spec	KDM7A JHI	-1.53727	-1.83094	-1.66102	-1.91475	-1.71727	-1.34395	-1.46713	-1.1571	-1.57089	-1.51584
Q6ZMZ3	Nesprin-3 (	SYNE3 C14	-1.24593	-0.89609	-0.94642	-0.68407	-0.57789	-0.23704	-0.73867	-1.04654	-1.20211	-1

Q6ZNB6	NF-X1-type	NFXL1 OZF	0.239609	0.638544	0.298341	0.906054	0.31895	0.525461	0.641808	0.465664	0.523972	0.518551
Q6ZNJ1	Neurobeac	NBEAL2 KIA	-4.08746	-1.85896	-3.48699	-3.45317	NA	NA	NA	NA	-3.93546	-3.13927
Q6ZS11	Ras and Ra	RINL	-0.60942	-0.86605	0.837125	0.425973	0.96233	1.008174	0.51866	0.911463	0.714156	0.573113
Q6ZSR9	Uncharacte	0	-0.81479	-0.82758	-0.35096	-0.47811	-0.38074	0.177029	-0.04972	-0.2978	-0.20349	-0.09096
Q6ZSS7	Major facili	MFSD6 MM	-0.5025	-0.5541	-0.65486	-0.75803	-0.68105	-0.74977	-0.61599	-0.61973	-0.83954	-0.93088
Q6ZSZ5	Rho guanin	ARHGEF18	-0.35585	-0.12689	0.126461	0.193992	0.472715	-0.00105	0.217222	0.156113	0.138848	0.608441
Q6ZU80	Centrosom	CEP128 C14	-1.54028	-1.41487	-0.62164	-0.6558	-1.97122	-0.92751	-0.86891	-0.77096	-0.89684	-0.77092
Q6ZUM4	Rho GTPas	ARHGAP27	-1.2569	-0.72146	-0.31614	-0.20561	-0.42	-0.92773	-0.66105	-0.43969	-0.44428	-0.29325
Q6ZUT1	Uncharacte	NKAPD1 C1	0.029995	0.019365	0.196539	0.15451	-0.04532	0.217701	-0.0897	-0.1193	0.523972	0.662499
Q6ZVF9	G protein-r	GPRIN3 KIA	-1.62803	-1.68589	-1.26235	-1.26053	-1.2076	-1.41504	-1.52065	-1.63743	-1.39801	-1.23771
Q6ZVK8	8-oxo-dGD	NUDT18 M	-1.09227	-0.75781	-1.0104	-1.5247	-1.38502	-1.46754	-0.97356	-1.81876	-1.10707	-1.0537
Q6ZVM7	TOM1-like	TOM1L2	0.006049	0.064808	0.256521	0.114512	0.232977	-0.2962	-0.30035	0.061639	0.400824	0.409495
Q6ZW49	PAX-intera	PAXIP1 PAX	-1.60942	-2.30256	-3.36146	-1.96285	-3.27798	-2.06711	-1.83577	-2.848	-2.21299	-1.36004
Q702N8	Xin actin-bi	XIRP1 CMY	1.930141	2.050761	0.680507	0.968291	1.232977	1.568474	1.424598	1.061639	0.976232	0.728087
Q709C8	Vacuolar pr	VPS13C KIA	-0.42655	-0.49202	-0.53138	-0.5881	-1.14048	-0.78359	-0.73016	-0.48543	-0.65006	-0.85688
Q70J99	Protein unc	UNC13D	-0.33545	-0.37132	-0.28796	-0.39713	-0.62018	-0.47079	-0.48694	-0.59354	-0.68476	-0.59938
Q70Z53	Protein FRA	FRA10AC1	-1.02445	-1.01568	-1.88753	-1.36976	-2.59991	-1.04439	-1.42531	-1.53434	-1.74959	-1.54137
Q71SY5	Mediator o	MED25 ACI	-3.43539	-4.44392	-1.53138	-1.67557	-2.84503	-1.87447	-1.51019	-1.91119	-1.65748	-1.42986
Q71U36	Tubulin alp	TUBA1A TU	0.336401	-0.22886	-0.93423	-0.52738	-0.40675	-0.74009	-0.19017	-0.63055	-0.96052	-1.131
Q71UI9	Histone H2	H2AZ2 H2A	0.472419	0.75549	0.125779	0.511785	0.83834	0.512533	0.454912	0.433142	0.116973	0.476725
Q71UM5	40S ribosor	RPS27L	1.770518	2.023207	2.022248	1.656347	1.701583	1.959826	1.749192	1.86507	1.896164	1.550629
Q75N03	E3 ubiquiti	CBLL1 HAK	-1.46855	-1.27399	-0.6734	-1.06413	-0.91341	-1.26303	-1.29406	-1.24936	-0.74169	-0.51584
Q75N90	Fibrillin-3 [	FBN3 KIAA	-0.57289	0.09067	0.042266	-0.39428	-0.37109	-0.03877	-0.35819	0.005714	-0.02377	0.228701
Q75QN2	Integrator	INTS8 C8or	-0.70499	-0.63656	-0.76983	-0.25303	-0.39046	-0.37906	-0.5697	-0.51785	-0.3505	-0.33192
Q76N32	Centrosom	CEP68 KIAA	-1.26546	-1.18265	-1.7632	-1.32193	-1.35199	-1.81558	-1.71329	-2.80735	-1.81444	-1.58054
Q7KZ85	Transcripti	SUPT6H KIA	-0.86139	-0.8052	-1.06898	-0.81207	-0.99505	-0.56866	-0.88887	-0.74846	-0.80091	-0.96384
Q7KZF4	Staphyloco	SND1 TDRD	0.121991	0.474243	0.26254	0.201957	0.248136	0.201063	0.213164	0.144077	0.16246	0.170646
Q7KZI7	Serine/thre	MARK2 EM	-1.78041	-1.73749	-1.30578	-1.13236	-1.8428	-1.1682	-1.68723	-1.17016	-1.9349	-1.33245
Q7L014	Probable A	DDX46 KIA	0.277504	0.564016	0.369473	0.176878	0.207442	0.259387	-0.06275	0.002223	0.262286	0.564161
Q7L0Y3	tRNA meth	TRMT10C M	-0.62742	-0.47873	-0.3924	-0.62408	-0.40024	-0.5366	-0.7997	-0.94386	-0.93546	-0.65524
Q7L1Q6	Basic leucir	BZW1 BZAF	1.074788	1.015513	-0.28821	0.309328	0.554186	0.70753	0.792409	0.878433	-0.07748	-0.32082
Q7L1W4	Volume-reg	LRRC8D LR	-1.76553	-1.33171	-1.2276	-1.47045	-1.53382	-1.52083	-1.42531	-1.33342	-1.95361	-1.41799
Q7L2E3	ATP-depen	DHX30 DDX	-0.46019	-0.4761	0.068531	-0.0126	0.029444	0.090032	-0.45305	0.061639	-0.08746	-0.04495

Q7L2H7	Eukaryotic	EIF3M HFLI	-1.25096	-0.82402	-1.00782	-0.40255	-1.49139	-0.73151	-0.93988	-1	-1.28011	-0.93934
Q7L2J0	7SK snRNA	MEPCE BCD	-3.25096	-2.46529	-2.26083	-2.67779	-2.50377	-2.1351	-0.96963	-1.40742	-2.26625	-2.5867
Q7L3B6	Hsp90 co-c	CDC37L1 C	-1.08746	-0.79667	-0.4761	-0.52356	-0.87199	-0.68684	-0.81762	-0.93289	-0.87368	-0.44184
Q7L4I2	Arginine/se	RSRC2	-0.80735	-0.85261	-0.55586	-0.33231	-0.5375	-0.27621	-0.5283	-0.79607	-0.32281	-0.29345
Q7L592	Protein arg	NDUFAF7 C	-0.2949	-0.16506	-0.20495	-0.19444	-0.17365	-0.24998	-0.32577	-0.09464	-0.57791	-0.37145
Q7L5N1	COP9 signa	COPS6 CSN	0.347397	0.220163	0.112475	0.088535	0.226636	-0.05477	-0.11921	0.043058	0.322735	0.276981
Q7L7L0	Histone H2	H2AW HIST	0.543974	0.972444	1.738914	2.147448	2.027621	1.954196	2.16651	2.06301	1.191761	1.124978
Q7L9L4	MOB kinas	MOB1B MC	1.850016	1.92805	2.227674	2	2.15831	1.975197	2.08445	2.123382	2.356402	2.539254
Q7LBC6	Lysine-spec	KDM3B C5c	0.369386	0.688743	0.0912	0.266039	0.287275	0.228224	0.26153	0.224234	0.263399	0.138098
Q7LBR1	Charged m	CHMP1B C1	-1.666	-1.37151	-1.43335	-1.47045	-0.88567	-1.01097	-0.84493	-0.85834	-1.02857	-1.09134
Q7LDG7	RAS guanyl	RASGRP2 C	-0.43539	-0.19599	-1.33737	-0.82095	-1.53382	-0.21601	-0.83623	-1.21925	-0.766	-0.63579
Q7LG56	Ribonucleo	RRM2B P53	-1.87245	-1.34156	-0.38163	-0.84549	-1.19052	-0.66069	-0.06809	-0.25618	-0.57089	-0.99118
Q7RTP6	[F-actin]-m	MICAL3 KIA	-3.80735	-3.77649	-3.62449	-2.0126	-3.27798	-3.0902	-3.3322	-2.07039	-3.30319	-3.30433
Q7RTR2	NLR family	NLRC3 NOD	-3.68589	-3.95397	-3.36146	-3.84549	-3.59991	-3.40054	NA	NA	-4.25739	-4.1788
Q7RTV0	PHD finger	PHF5A	-0.25819	-0.17917	-0.20945	-0.36012	-0.14188	-0.43386	-0.53964	-0.2978	-0.03665	0.03065
Q7Z2K6	Endoplasm	ERMP1 FXN	-0.3769	-0.65057	-1.04689	-1.3316	-0.81996	-0.7972	-1.57114	-1.20129	-1.39484	-1.50286
Q7Z2T5	TRMT1-like	TRMT1L C1	-0.27277	-0.29298	0.148099	-0.42764	-0.40024	0.053638	-0.19704	0.131245	0.168877	0.305908
Q7Z2W4	Zinc finger	ZC3HAV1 Z	-0.48983	-0.53703	-0.84937	-0.86658	-1.10027	-0.97554	-0.83381	-0.92752	-1.05203	-1.05262
Q7Z333	Probable h	SETX ALS4	-0.08104	-0.24136	-0.16069	0.148863	0.036712	-0.10188	-0.17377	-0.10076	-0.14365	-0.08663
Q7Z392	Trafficking	TRAPPC11	-1.11346	-0.63051	-1.39901	-1.95892	-2.14197	-2.09717	-1.43858	-2.22289	-1.76705	-1.58548
Q7Z3B4	Nucleopori	NUP54	-0.15097	-0.34793	0.139302	-0.10963	-0.04043	-0.29547	-0.05686	-0.47896	0.139545	0.240048
Q7Z3D6	D-glutamat	DGLUCY C1	0.006049	-0.02756	-2.57719	-3.38606	-0.60274	-0.47115	0.383675	0.313838	-0.49584	-1.06333
Q7Z3J2	VPS35 endo	VPS35L C16	-0.44137	-0.55913	-1.12229	-1.09806	-1.79279	-0.93341	-0.67361	-0.91899	-1.16259	-1.04515
Q7Z3K3	Pogo trans	POGZ KIAA	-0.55497	-0.90933	-0.72172	-0.9366	-0.69302	-0.7304	-0.73016	-1.09464	-0.62544	-0.49075
Q7Z406	Myosin-14	MYH14 KIA	0.27733	0.264343	0.486517	0.612957	0.587361	0.717896	0.501387	0.643377	0.719778	1.075135
Q7Z417	Nuclear fra	NUFIP2 KIA	0.110807	-0.02359	0.69262	1.286367	0.680858	0.710493	-1.779	-1.8107	0.591235	0.385981
Q7Z422	SUZ domai	SZRD1 C1o	-2.98793	-1.52015	-1.94642	-1.23078	-2.79256	-2.06711	-1.89162	-2.80735	-1.95361	-1.38294
Q7Z434	Mitochond	MAVS IPS1	-0.69319	-0.64791	-0.70387	-0.74614	-0.6119	-0.78295	-1.35819	-0.60357	-1.10764	-0.87299
Q7Z460	CLIP-associ	CLASP1 KIA	-0.6002	-0.20889	-0.10658	-0.37156	-0.06944	-0.20553	-0.25712	-0.44535	-0.34669	-0.68536
Q7Z478	ATP-depen	DHX29 DDX	-3.26612	-2.78545	-2.47224	-2.59756	-1.49139	-1.5366	-2.69938	-3.12706	-1.3505	-1.45391
Q7Z4G1	COMM dor	COMMD6 I	-0.42917	-0.10551	-1.20579	-1.08735	-1.24342	-1.63641	-1.76715	-1.92719	-1.65323	-1.68018
Q7Z4H3	5'-deoxynu	HDDC2 C6c	-0.46019	-0.59474	-0.35646	-0.50564	-0.5231	-0.61813	-0.43219	-0.34066	-0.17524	-0.13441
Q7Z4H7	HAUS augn	HAUS6 DGT	0.620882	0.680507	0.994139	0.712931	0.863612	0.788496	0.779312	0.673772	0.797895	0.416143

Q7Z4H8	Protein O-g	POGLUT3 K	-0.71878	-0.60457	-0.24716	-0.56872	-1.14067	-0.84198	-0.3847	-0.46534	-0.51899	-0.399
Q7Z4Q2	HEAT repea	HEATR3	-2.37673	-1.51588	-2.03731	-1.94585	-2.77259	-3.40517	-1.71368	-2.36468	-2.22502	-1.61036
Q7Z4S6	Kinesin-like	KIF21A KIA	-2.98793	-1.30256	-1.19153	-1.20163	-2.74193	-1.50524	-1.30666	-1.63743	-1.4892	-1.56737
Q7Z4V5	Hepatoma-	HDGFL2 HD	1.103313	1.303181	0.461277	0.573941	0.641969	0.413696	0.253441	0.326411	0.086908	-0.26127
Q7Z4W1	L-xylulose r	DCXR SDR2	0.180846	0.144252	-0.41764	-0.23816	0.141554	-0.2115	0.04087	-0.16993	0.418285	0.530855
Q7Z569	BRCA1-asso	BRAP RNF5	-2.76553	-1.94642	-1.39183	-0.9386	-1.47063	-1.1375	-1.68012	-1.37737	-1.39941	-0.84888
Q7Z591	Microtubul	AKNA KIAA	-1.19785	-1.32752	-0.96413	-1.50733	-0.73913	-1.13841	-1.71329	-1.6735	-1.27685	-1.10022
Q7Z5K2	Wings apar	WAPL FOE	-0.46804	-0.37541	-0.37634	-0.4656	-0.41723	-0.45101	-0.63184	-0.6219	-0.25537	-0.32829
Q7Z5L9	Interferon	IRF2BP2	-0.89018	-0.87124	-0.59383	-0.67628	0.046194	0.174644	-0.99644	-0.80742	-0.26542	-0.34865
Q7Z5R6	Amyloid be	APBB1IP PI	-0.26546	-0.57138	0.44154	0.210689	0.318408	0.218458	0.162805	0.163187	0.301322	0.309837
Q7Z614	Sorting nex	SNX20 SLIC	-0.22948	-0.01963	-0.40726	-0.5787	-0.83173	-0.64351	-0.43219	-0.45372	-0.55695	-0.7713
Q7Z6B0	Coiled-coil	CCDC91 GC	-0.2608	-0.12487	0.019365	0.143195	-0.2076	-0.15565	0.113114	-0.06439	0.126316	-0.12473
Q7Z6E9	E3 ubiquiti	RBBP6 P2P	0.006049	-0.10093	-0.03953	-0.11078	0.14828	-0.11958	-0.06274	0.017074	-0.18593	-0.00888
Q7Z6I6	Rho GTPas	ARHGAP30	-0.34848	-0.50433	-0.34198	-0.58863	-0.26608	-0.19131	-0.29134	-0.28759	-0.40463	-0.26539
Q7Z6L1	Tectonin be	TECPR1 KIA	-1.02651	-1.14173	-1.16517	-1.22153	-0.95786	-0.92073	-1.31548	-1.16614	-0.54678	-0.71945
Q7Z6M1	Rab9 effect	RABEPK RA	-0.27277	0.049819	0.994139	1.828702	0.859519	0.514027	0.372201	-0.37737	0.696808	0.717361
Q7Z6M4	Transcripti	MTERF4 M	-1.19438	-1.26459	-1.26017	-1.40944	-1.93501	-1.29759	-1.8473	-1.82639	-1.61642	-2.00646
Q7Z6Z7	E3 ubiquiti	HUWE1 KIA	-0.72489	-0.45472	-0.61849	-0.59756	-0.50188	-0.70454	-0.53224	-0.53519	-0.55003	-0.46609
Q7Z7A1	Centriolin (	CNTRL CEP	-1.82873	-1.36146	-1.63438	-1.41146	-1.61127	-1.65999	-1.95312	-1.58773	-2.0287	-1.32041
Q7Z7F7	39S ribosor	MRPL55 UI	-1.54927	-0.89237	-0.51124	-0.14853	-0.36949	-0.64344	-0.30539	-1.07764	-0.50566	-0.49451
Q7Z7G8	Vacuolar pı	VPS13B CH	2.142729	2.197389	2.492373	2.186556	2.404308	2.531145	2.182377	2.335603	2.612206	2.13408
Q7Z7H5	Transmeml	TMED4 ERS	-1.59046	-1.59939	-1.65545	-0.74746	-0.9417	-0.1315	-1.76766	-1.31907	-1.83112	-1.58054
Q7Z7K0	COX assem	CMC1 C3or	0.799254	0.9501	0.22796	0.072831	0.123467	-0.37761	0.036617	0.240344	-0.0183	0.152784
Q7Z7K6	Centromer	CENPV PRR	0.004817	0.058271	-0.13901	-0.23341	0.356196	0.322569	0.041734	0.109411	0.7084	0.771547
Q86SX6	Glutaredox	GLRX5 C14	-0.97798	-1.34308	-0.93587	-1.11475	-1.08648	-1.01608	-1.71838	-1.31091	-0.88109	-1.10362
Q86TI0	TBC1 doma	TBC1D1 KIA	-0.6099	-0.64404	-0.72334	-0.50726	-0.89546	-0.70738	-0.84036	-0.63462	-0.65005	-0.89275
Q86TI2	Dipeptidyl	DPP9 DPRF	0.969368	1.049819	0.288798	0.126054	0.107446	0.308753	0.235488	0.232173	0.436099	0.31305
Q86TJ2	Transcripti	TADA2B AD	-1.45187	-1.34156	-1.53138	-1.63604	-0.76702	-1.31647	-0.88216	-0.95491	-1.30319	-1.70487
Q86TP1	Exopolyph	PRUNE1 PR	-1.53833	-1.37972	-1.03294	-1.34169	-1.55651	-1.73727	-1.35456	-1.52157	-1.01178	-1.19124
Q86TU7	Actin-histic	SETD3 C14	-2.06415	-1.75056	-1.81303	-1.15074	-2.23002	-2.36452	-1.34848	-1.80638	-1.66619	-1.11668
Q86TX2	Acyl-coenz	ACOT1 CTE	-0.51184	-0.21583	-0.60912	-0.62307	-0.88862	-0.64011	0.075495	-0.16962	0.06454	-0.0981
Q86U28	Iron-sulfur	ISCA2 HBLD	0.207697	0.109733	0.195266	0.24497	0.130347	0.13035	0.040049	0.017827	0.019007	0.208956
Q86U38	Nucleolar p	NOP9 C14c	0.603656	0.596566	-0.03771	-0.11437	0.27981	0.170571	0.310186	0.2421	-0.12191	-0.22554

Q86U42	Polyadenyl	PABPN1 PA	0.229304	0.144576	0.402309	0.198904	0.492844	0.398549	0.127235	0.294183	0.372796	0.573177
Q86U44	N6-adenos	METTL3 M	-0.97056	-0.77789	-0.76204	-0.53492	-0.81025	-0.77294	-0.54079	-1.01348	-0.89194	-0.6811
Q86U86	Protein pol	PBRM1 BAI	-1.46855	-1.27399	-1.14768	-1.2909	-1.15697	-1.07861	-0.87276	-0.93289	-0.81444	-0.98055
Q86UA1	Pre-mRNA-	PRPF39	-1.13993	-0.57138	-2.30256	-3.75803	-3.2076	-1.83494	-1.91073	-1.82753	-2.1281	-1.85688
Q86UE4	Protein LYF	MTDH AEG	0.539081	0.697125	0.405256	0.108706	0.3249	-0.03877	0.307295	-0.24936	0.206137	0.291517
Q86UE8	Serine/thre	TLK2	-0.88359	-0.88753	-1.08844	-0.4361	-1.69302	-1.0902	-1.29406	-0.88982	-0.86507	-0.94785
Q86UK7	E3 ubiquiti	ZNF598	-2.64889	-2.22806	-1.53059	-1.77078	-2.16821	-1.81609	-1.65342	-2.31914	-1.92672	-1.63174
Q86UL3	Glycerol-3-	GPAT4 AGF	-2.53727	-1.77649	-1.45457	-2.71621	-3.07635	-1.91511	-3.13955	-2	-3.45003	-2.41799
Q86UP2	Kinectin (C	KTN1 CG1 I	0.055103	0.265778	0.216811	-0.0013	0.251215	0.197501	-0.03394	0.051762	0.389479	0.31668
Q86UU0	B-cell CLL/l	BCL9L DLNI	-1.01217	-0.67963	-0.63656	-0.15899	-0.36151	-0.37906	-0.22666	-0.31194	-0.05772	0.056413
Q86UV5	Ubiquitin c	USP48 USP	-0.06104	-0.10414	0.258459	-0.058	-0.05329	0.148319	0.055691	-0.15318	0.241248	-0.05003
Q86UW6	NEDD4-bin	N4BP2 B3B	-3.03684	-2.38163	-2.12199	-3.38606	-3.35199	-3.18641	-3.61599	-3.72935	-3.30319	-2.34873
Q86UX7	Fermitin fa	FERMT3 KII	0.385219	0.550038	0.111448	0.076399	-0.16588	0.123248	0.15532	-0.02706	-0.15934	0.04857
Q86V48	Leucine zip	LUZP1	-0.46855	-0.29298	0.193132	-0.2986	-0.04532	-0.10188	-0.06274	0.02272	-0.54314	-0.60054
Q86V81	THO compl	ALYREF ALY	0.337628	0.408547	-0.0095	0.160401	0.365132	0.255291	0.181034	0.318017	-0.02676	-0.11164
Q86VM9	Zinc finger	ZC3H18 NH	-0.19478	0.001789	-0.48699	-0.0126	-0.27798	-0.72247	-0.30035	-0.2091	-0.45003	-0.28803
Q86VN1	Vacuolar pr	VPS36 C13	-1.21002	-1.3195	-1.75002	-1.3377	-0.67339	-1.44448	-1.18536	-1.71049	-1.4245	-1.32636
Q86VP3	Phosphofu	PACS2 KIAA	-2.60942	-1.1305	-1.32193	-1.96285	-0.69302	-1.81558	-1.11718	-1.61973	-2.1913	-0.99118
Q86VP6	Cullin-asso	CAND1 KIA	0.62032	0.828368	0.45446	0.299168	0.207442	0.23173	0.475158	0.506536	0.67925	0.625686
Q86VQ1	Glucocortic	GLCCI1	-0.83954	-1.20945	-0.50348	-1.18728	-1.09212	-1.1375	-1.01027	-1.1193	-0.88235	-0.93088
Q86VR2	Reticulopha	RETREG3 F	-1.32496	-1.4761	-1.57719	-1.11757	-1.37109	-1.33015	-1.81078	-1.70916	-2.16728	-1.79235
Q86VS8	Protein Ho	HOOK3	-0.7835	-0.71596	-0.67963	-0.06413	-0.2076	0.016302	-0.19704	-0.42269	-1.09752	-1.12956
Q86VU5	Catechol O	COMTD1 U	-0.49394	-0.64874	-0.84488	-0.38606	-0.88567	-0.50524	-0.313	-0.52607	-0.79796	-1.22979
Q86W42	THO compl	THOC6 WD	-0.35566	-0.52015	-0.70483	-0.51457	-0.53382	-0.35091	-0.33865	-0.26992	-0.54314	-0.18382
Q86W50	RNA N6-ad	METTL16 N	-1.22239	-0.83094	-0.39057	-0.74038	0.086588	0.074547	-0.66562	-0.84286	-0.73947	-0.12042
Q86WA6	Valacyclovi	BPHL MCN	-0.94062	-1.19153	-1.23676	-1.21613	-1.47063	-0.9783	-0.75585	-0.46949	-0.91754	-0.97369
Q86WB0	Nuclear-int	ZC3HC1 NII	-0.79679	-0.90202	-0.34853	-0.35431	-0.32379	-0.50524	-0.7388	-0.47166	-0.78165	-0.8893
Q86WJ1	Chromodo	CHD1L ALC	0.524323	0.388614	-0.44425	-0.10576	-0.01113	0.434117	0.263157	0.151552	0.468765	0.158587
Q86WN1	F-BAR and	FCHSD1 UN	-0.22449	-0.02492	-0.24598	-0.37788	-0.51785	-0.18802	-0.46007	-0.18938	-0.14886	-0.50324
Q86WR0	Coiled-coil	CCDC25	0.963311	1.051666	0.824296	0.599523	0.917164	0.872709	1.013733	1.059467	0.900362	0.853801
Q86WV1	Src kinase-	SKAP1 SCA	-0.74934	-0.80074	-1.0342	-0.90112	-1.04058	-0.7881	-0.90856	-1.07703	-1.22662	-1.07378
Q86WV6	Stimulator	STING1 ERI	-0.71491	-0.25992	0.137504	-0.31411	0.161638	0.110424	-1.94973	-3.80735	-0.40564	-0.12473
Q86X29	Lipolysis-st	LSR LISCH	-2.29498	-3.08208	-1.35147	-1.56009	-0.79256	-0.70454	-2.67715	-2.37929	-2.21698	-2.40626

Q86X53	Glutamate-	ERICH1 HSI	0.488887	0.474243	0.886471	0.4899	0.181447	0.669851	0.611993	0.577309	0.843274	0.631411
Q86X55	Histone-arg	CARM1 PRI	-1.1669	-1.2752	-0.91942	-0.79086	-1.32622	-1.29847	-0.87609	-1.19295	-1.63165	-1.15398
Q86X76	Deaminate	NIT1	-1.38702	-0.78991	-1.00782	-0.20163	-0.9852	-0.58496	-0.61599	-0.68266	-0.77357	-0.71937
Q86X95	Corepressc	CIR1 CIR	-3.37126	-1.60064	0.275973	0.349267	0.181447	0.304334	0.399607	0.326501	0.537028	0.57905
Q86XP3	ATP-depen	DDX42	-0.39497	-0.18709	-0.40528	-0.61918	-0.48097	-0.4765	-0.4575	-0.52623	-0.36257	-0.19236
Q86XR8	Centrosom	CEP57 KIAA	-0.58193	-0.62449	-0.93147	-0.98751	-0.59991	-0.60977	-0.92038	-0.6644	-1.04793	-0.61402
Q86XT2	Vacuolar pı	VPS37D WI	0.836501	1.019365	0.545434	0.626185	0.336725	0.506353	0.418869	0.35811	0.379237	-0.04495
Q86Y39	NADH dehy	NDUFA11	0.371969	0.532014	-0.03953	0.165737	-0.05302	-0.15565	-0.07346	0	-0.01898	0.147127
Q86YP4	Transcripti	GATAD2A	-0.16759	-0.09316	-0.04069	-0.3099	-0.18206	-0.07285	-0.03159	-0.00152	0.232165	0.04609
Q86YS7	C2 domain-	C2CD5 CDF	-0.63743	-0.59474	-1.20945	-0.74746	0.408774	0.30098	0.530721	0.484651	-0.95361	-0.83303
Q86YV0	RAS proteir	RASAL3	0.195776	0.338083	0.182472	-0.14611	-0.27798	0.021695	0.135912	-0.29078	-0.13327	0.069124
Q86YV5	Inactive tyr	PRAG1 SGK	-2.80486	-1.8625	-2.84205	-3.07272	-2.63733	-3.0315	-1.94447	-2.39734	-1.32976	-1.06779
Q8IU68	Transmeml	TMC8 EVEF	-3.72489	-3.28345	-2.03953	-1.91475	-3.27798	-1.83494	-2.9301	-1.86876	-2.28011	-1.90578
Q8IU81	Interferon	IRF2BP1	-2.98793	-1.52015	-1.03953	-0.98751	-0.22487	-0.96758	-0.9898	-1.02308	-1.01898	-1.03585
Q8IU85	Calcium/ca	CAMK1D C.	-1.43539	-0.91667	-1.08017	-0.66558	-0.95606	-1.07861	-0.83577	-2.22239	-1.36257	-0.86491
Q8IUE6	Histone H2	H2AC21 HI!	-0.3832	-0.69862	0.30862	0.346699	0.280421	0.873569	0.362691	0.38327	0.39003	0.147453
Q8IUF8	Ribosomal	RIOX2 MDI	0.514573	0.603045	-0.39696	-0.97513	0.014797	0.254814	0.299241	0.530515	-0.88235	-0.39453
Q8IUH3	RNA-bindir	RBM45 DRI	-1.85491	-1.4056	-0.9958	-1.87975	-0.85493	-1.04616	-1.12275	-1.87012	-1.00827	-0.90028
Q8IUI8	Cytokine re	CRLF3 CREI	0.124835	0.136527	-0.20048	-0.50389	-0.09342	-0.24225	-0.15638	-0.33201	0.068593	0.277731
Q8IUR0	Trafficking	TRAPPC5	0.63553	0.634901	0.384943	0.326502	0.660657	0.529894	0.345502	0.389254	0.453989	0.698146
Q8IUR7	Armadillo r	ARMC8 S86	-2.85042	-2.34156	-2.05565	-1.98751	-2.55552	-3.28951	-2.41163	-1.76783	-2.45003	-3.39453
Q8IV04	Carabin (TE	TBC1D10C	-1.20486	-1.35948	-1.26514	-1.07737	-1.65974	-1.14739	-0.96905	-1.11981	-1.76638	-0.99306
Q8IV38	Ankyrin rep	ANKMY2	-0.55497	-0.62449	-0.46529	-0.92663	-0.66918	-0.43706	-0.90115	-0.68266	-0.45003	-0.42392
Q8IV53	DENN dom	DENND1C I	0.028061	0.048404	0.136844	0.205189	-0.03138	0.061341	0.210077	0.116663	0.168247	0.028631
Q8IV63	Inactive sei	VRK3	-2.98793	-2.03953	-2.12199	-2.06413	-2.47063	-2	-3.09516	-2.76783	-2.58496	-2.2402
Q8IVB5	LIX1-like pr	LIX1L	0.302347	0.196395	1.022309	0.726167	0.724561	1.08229	0.754451	0.745082	1.145497	0.882281
Q8IVD9	NudC doma	NUDCD3 KI	-2.60942	-1.90202	-2.38163	-1.8011	-2.59991	-3.0902	-3.13955	-3.97728	-1.67243	-1.99118
Q8IVH4	Methylmal	MMAA	-0.37126	-0.17823	-0.64264	-0.44461	-1.15697	-0.42234	-0.35819	-0.62855	-0.32665	-0.58718
Q8IVT5	Kinase supı	KSR1 KSR	-1.45197	-1.37112	-1.34611	-1.9394	-1.61666	-1.60902	-1.57386	-1.83877	-1.99732	-1.84085
Q8IW45	ATP-depen	NAXD CARI	-1.30986	-1.33171	-0.64874	-1.61667	-1.95606	-1.5366	-1.15087	-1.18286	-1.32665	-1.04495
Q8IWB7	WD repeat	WDFY1 FEI	-0.51624	-0.1183	-0.05207	-0.50584	-0.34745	-0.2287	-0.24502	-0.17734	-0.28637	-0.45391
Q8IWI9	MAX gene-	MGA KIAA(	-3.80735	-2.2276	-2.20945	-3.75803	-2.89947	-2.79647	-2.89162	-3.33342	-3.16993	-2.34873
Q8IWJ2	GRIP and c	GCC2 KIAA(	-1.06878	-1.07685	-0.56645	-0.82364	-0.70023	-0.47865	-0.71534	-0.57712	-0.69032	-0.69318

Q8IWS0	PHD finger	PHF6 CENP	-0.22948	0.068531	0.083328	-0.2909	-0.21621	0.14543	-0.16227	-0.08854	-0.30902	-0.63448
Q8IWV7	E3 ubiquiti	UBR1	-0.67565	-0.5541	-0.48153	-0.15899	-0.13231	-0.5366	-0.37137	-0.17638	-0.51592	-0.07727
Q8IWV8	E3 ubiquiti	UBR2 C6or	0.716207	0.723647	0.70941	0.754423	0.584963	0.624491	0.608641	0.104869	0.139217	0.455403
Q8IWW6	Rho GTPas	ARHGAP12	-0.16011	-0.10093	-0.07195	-0.25303	-0.1487	-0.5366	-0.02061	-0.31194	-0.06263	-0.21945
Q8IWX8	Calcium ho	CHERP DAN	1.414658	1.685443	0.892367	1.163572	1.007716	1.324273	1.035784	1.147351	0.888526	0.771607
Q8IWY9	Codanin-1	CDAN1 UN	-1.80798	-1.99047	-1.64972	-1.91495	-2.57908	-2.3795	-1.90181	-2.52383	-1.96303	-1.78667
Q8IWZ3	Ankyrin rep	ANKHD1 KI	-1.74823	-1.64275	-1.41855	-1.75803	-1.19302	-1.26303	-1.46921	-1.63359	-1.51221	-1.79889
Q8IWZ8	SURP and G	SUGP1 SF4	-0.26948	-0.32267	0.046245	-0.31514	0.098507	-0.24386	-1.32227	-0.99933	-0.97214	-0.73645
Q8IX01	SURP and G	SUGP2 KIA	-0.69515	-0.2276	-0.18709	-0.12439	-0.31451	-0.02203	-0.25691	-0.61096	-0.29739	-0.65524
Q8IX12	Cell divisio	CCAR1 CAR	0.047256	0.003346	0.217504	0.204358	0.18799	0.100264	0.18077	0.088809	0.342525	0.135088
Q8IXB1	DnaJ homo	DNAJC10 E	-0.36344	-0.03953	-0.64874	-0.63604	-1.57755	-1.02203	-0.22069	-0.50967	-1.46297	-1.1589
Q8IXH7	Negative el	NELFCD NE	-0.6002	-1.01568	-1.19153	-1.56009	-1.06075	-1.18641	-1.23266	-1.37737	-1.39941	-1.76377
Q8IXI1	Mitochond	RHOT2 ARH	-0.00014	0.066417	-0.11689	-0.25782	-0.39704	0.000962	-0.07086	-0.03124	-0.30427	-0.47188
Q8IXI2	Mitochond	RHOT1 ARH	-1.72032	-1.24155	-1.51544	-1.31768	-0.98731	-1.31468	-1.09367	-0.97171	-2.11933	-0.88013
Q8IXM2	Chromatin	BAP18 C17	0.437802	0.625744	0.430625	0.221763	0.144603	0.553369	0.179978	0.216056	0.254879	0.421434
Q8IXM3	39S riboso	MRPL41 BN	1.572788	1.87907	1.518467	1.51426	1.676212	1.762961	1.690171	1.982722	1.848521	1.203941
Q8IXQ6	Protein mo	PARP9 BAL	-1.30986	-0.81714	-0.45992	-0.17306	-0.75442	-1.05571	-0.51019	-1.0115	-0.85651	-0.43584
Q8IXT5	RNA-bindir	RBM12B	-0.38295	-0.12715	-0.59808	-0.57878	-0.30441	-1.46281	-0.77093	-0.9444	-0.88856	-0.30496
Q8IY17	Patatin-like	PNPLA6 NT	-2.68536	-2.12199	-1.69849	-1.50564	-4.01495	-1.37197	-1.52484	-2.19592	-2.1913	-2.00888
Q8IY21	Probable A	DDX60	-0.04393	0.27262	0.0154	-0.16131	-0.09892	0.130264	-0.03583	-0.06371	0.209388	0.003372
Q8IY37	Probable A	DHX37 DDX	-3.57289	-1.68589	-1.49796	-1.5787	-1.49139	-1.50524	-1.66382	-1.61973	-1.37474	-1.44184
Q8IY67	Ribonucleo	RAVER1 KIA	-0.3999	-0.52997	-0.80441	-0.44324	-0.81309	-0.76578	-0.45796	-0.70916	-0.77495	-0.60575
Q8IY81	pre-rRNA 2	FTSJ3 SB92	0.480222	0.414062	0.600476	0.533888	0.792405	0.791656	0.71843	0.684498	0.696808	0.521637
Q8IYB3	Serine/argi	SRRM1 SRM	-0.34792	-0.30738	0.203328	0.037153	0.181447	0.105353	0.191366	0.131245	0.202044	0.323697
Q8IYB7	DIS3-like ex	DIS3L2 FAM	-0.55584	-0.21313	-0.09965	-0.47682	-0.12642	-0.10055	-1.03677	-0.83635	-0.26379	-0.18968
Q8IYB8	ATP-depen	SUPV3L1 SU	0.47587	1.040371	0.256521	0.392915	0.270452	0.652573	1.220198	1.177538	0.335069	-0.0726
Q8IYD1	Eukaryotic	GSPT2 ERF3	-2.5025	-1.64874	-0.79667	-1.96285	-1.62263	-1.22422	-1.94973	-0.63743	-0.848	-1.13927
Q8IYI6	Exocyst co	EXOC8	-1.37126	-0.91667	-2.08844	-1.50564	-0.90134	-0.48809	-2.85843	-2.50018	-2.02857	-1.82517
Q8IYJ3	Synaptotag	SYTL1 SLP1	-0.6246	-0.89445	-0.24812	-0.50377	-0.52894	-0.26734	-0.72697	-0.56366	-0.77902	-0.73327
Q8IYL3	UPF0688 p	C1orf174	-1.1669	-1.88753	-1.66102	-1.32193	-1.03006	-1.45943	-1.13955	-0.70116	-2.10764	-1.90578
Q8IYM9	E3 ubiquiti	TRIM22 RN	-0.72489	-0.50348	-0.36146	-0.42764	-1.2076	-0.78701	-0.83577	-0.922	-0.90866	-0.36573
Q8IYQ7	Threonine	THNSL1	-0.27271	-0.35897	-0.18857	-0.34439	-0.29833	0.103732	-0.02362	-0.10274	0.156532	0.11545
Q8IZ07	Ankyrin rep	ANKRD13A	-2.46211	-1.57267	-2.45121	-2.49812	-2.10539	-2.54323	-3.07401	-1.93315	-2.35183	-2.32884

Q8IZ57	Neurensin-	NRSN1 VM	1.229304	1.534708	1.179425	1.416605	1.303969	1.35435	1.134244	1.448985	1.384664	1.382591
Q8IZ69	tRNA (urac	TRMT2A H	-2.11561	-1.29048	-1.26083	-1.16954	-1.1163	-1.47967	-1.0106	-0.91716	-1.28999	-1.0931
Q8IZ83	Aldehyde d	ALDH16A1	0.139561	-0.20299	-0.37595	-0.33413	-0.4423	-0.50832	-0.51391	-0.53286	-0.46949	-0.08065
Q8IZD2	Inactive his	KMT2E ML	-0.78142	-0.63098	-0.31868	-0.52537	-0.10207	-0.16789	-0.41565	-0.38189	-0.3067	-0.21289
Q8IZD4	mRNA-deca	DCP1B	-2.89482	-2.03953	-1.31221	-1	-1.41009	-1.17403	-1.12832	-0.78746	-1.9906	-1.44184
Q8IZH2	5'-3' exorib	XRN1 SEP1	-0.754	-0.84779	-0.8843	-0.96035	-0.9393	-1.00102	-1.17715	-1.29956	-1.05128	-0.89028
Q8IZL8	Proline-, gl	PELP1 HMX	0.683224	0.708509	0.87514	0.575354	1.064473	0.793116	0.785252	0.775393	0.843274	0.751934
Q8IZP0	Abl interac	ABI1 SSH3E	-0.56616	-0.48154	-1.49256	-1.62441	-1.81879	-1.6603	-1.27644	-1.77711	-1.73076	-1.744
Q8IZQ5	Selenoprot	SELENOH C	0.485165	0.827219	0.253253	0.263034	0.652528	0.364785	0.582964	0.559001	0.678029	0.799342
Q8N0W3	L-fucose ki	FCSK FUK	-1.20832	-1.36146	-1.42286	-0.95067	-1.37109	-1.05571	-1.12832	-1.53434	-1.16024	-1.13937
Q8N0X4	Citramalyl-	CLYBL CLB	-0.33076	-0.52576	-0.80346	-1.1815	-0.5231	-0.0614	-0.52484	-0.37737	-0.04307	-0.0726
Q8N0X7	Spartin (Sp	SPART KIA	-1.11346	-1.03154	-0.64874	-0.46179	-1.2076	-0.94636	-1.12832	-1.88982	-1.11784	-0.73402
Q8N0Z3	Spindle and	SPICE1 CCD	-3.57289	-3.48699	NA	NA	-3.59991	-4.58496	-3.43911	-2.93289	-3.45003	-2.51584
Q8N0Z8	tRNA pseu	PUSL1	-2.46855	-1.7632	-0.78319	-1.06413	-1.07635	-1.52083	-3.09516	-1.37737	-0.98126	-0.8893
Q8N103	T-cell activ	TAGAP TAG	-2.66778	-2.39214	-0.77538	-1.50663	-0.76702	-1.38287	-0.93569	-1.09822	-2.40163	-2.31779
Q8N108	Mesoderm	MIER1 KIA	-0.52826	-0.56655	0.03859	-0.32315	-0.22679	-0.11431	-0.22061	0.168011	-0.04583	0.208948
Q8N122	Regulatory	RPTOR KIA	-1.34844	-0.66522	-0.61756	-0.74153	-0.80556	-0.79922	-0.53368	-1.42114	-0.68107	-0.64292
Q8N137	Centrobin (	CNTROB LI	-0.5285	-0.35646	-0.16944	-0.51457	-0.47063	-0.59318	-0.37137	-0.28379	-0.29739	-0.26127
Q8N163	Cell cycle a	CCAR2 DBC	-0.26546	-0.47069	-0.24598	-0.42206	-0.03286	0.010888	-0.26303	-0.42269	-0.34796	-0.27263
Q8N183	NADH dehy	NDUFAF2 N	0.076726	0.148099	0.216811	0.252542	0.43351	0.540568	0.26559	0.546282	0.770518	0.490476
Q8N1B4	Vacuolar pı	VPS52 SACI	-1.44741	-0.95556	-0.43709	-0.57883	-0.3537	-0.42456	-0.74838	-0.78489	-0.53708	-0.5268
Q8N1F7	Nuclear po	NUP93 KIA	-0.53944	-0.44662	-0.39183	-0.47917	-0.56649	-0.29456	-0.36556	-0.25618	-0.27439	-0.62361
Q8N1F8	Serine/thre	STK11IP KI	-3.5025	-3.57719	-3.44392	-3.32193	-2.55552	-1.77761	-3.38466	-3.39232	-3.04793	-3.39453
Q8N1G0	Zinc finger	ZNF687 KIA	-1.08746	-0.91667	-0.98449	-0.4361	-0.51245	-0.66069	-0.9301	-0.80735	-0.45003	-0.37718
Q8N1G2	Cap-specifi	CMTR1 FTS	-0.80735	-0.20945	-0.05565	-0.07731	-0.23359	-0.30968	-0.27537	0.056143	-0.22396	-0.18886
Q8N1G4	Leucine-ric	LRRC47 KIA	0.064765	0.203328	0.08827	0.317935	0.136585	0.194011	0.244954	0.194842	0.255336	0.324523
Q8N1K5	Protein THI	THEMIS C6	-0.10057	-0.32833	0.272202	0.350501	-0.12646	-0.25328	0.059094	0.166088	-0.11502	-0.23265
Q8N201	Integrator	INTS1 KIAA	-2.08769	-1.33154	-1.51111	-1.67611	-2.71914	-1.95802	-1.83577	-1.70855	-1.76553	-1.77576
Q8N257	Histone H2	H2BU1 HIS	-1.77881	-0.83334	-1.14619	-0.72968	-1.0311	-0.85603	0.350534	0.297602	-0.37301	-0.25587
Q8N2K0	Lysophospl	ABHD12 C2	-0.13993	-0.7112	-0.14336	-0.40255	-0.22487	-0.16175	-0.41163	-0.27684	-0.92647	-1.54137
Q8N335	Glycerol-3-	GPD1L KIA	-0.49394	0.176654	0.105585	-0.24646	-0.1015	-0.11477	0.339668	0.212003	0.012373	-0.13163
Q8N357	Solute carr	SLC35F6 C2	-0.8238	-1.32127	-1.32483	-0.55055	-0.49762	-0.31849	-1.14149	-0.721	-1.23481	-1.20458
Q8N392	Rho GTPas	ARHGAP18	-0.70499	-0.57138	0.133954	-0.14505	0.058299	-0.56866	0.235488	-0.12553	-0.18593	-0.13441

Q8N3C0	Activating s	ASCC3 HELI	-0.40296	-0.08844	-0.07195	-0.59756	-0.25118	-0.09603	-0.53222	-0.50967	-0.26303	-0.03585
Q8N3D4	EH domain	EHBP1L1	-1.13328	-1.11109	-0.21102	-0.48794	-0.51245	-0.46697	-0.83577	-0.9886	-0.52316	-0.65661
Q8N3P4	Vacuolar pı	VPS8 KIAA(	-4.19438	-2.34156	-2.26459	-3.45317	-2.64572	-3.72247	NA	NA	-3.55695	-3.30433
Q8N3U4	Cohesin sul	STAG2 SA2	0.254929	0.3266	-0.07605	0.031027	-0.10806	0.19878	-0.03103	-0.08854	0.218346	0.118877
Q8N3X1	Formin-bin	FNBP4 FBP	-1.05673	-0.98674	-0.89237	-1.20163	-0.65735	-0.8331	-1.1461	-1.04557	-0.50628	-0.59727
Q8N3Z6	Zinc finger	ZCCHC7 HS	-1.85042	-2.26459	-1.72403	-2.59756	-1.31451	-1.91511	-1.45305	-1.86876	-1.97199	-1.60726
Q8N488	RING1 and	RYBP DEDA	-3.13993	-3.24598	NA	NA	NA	NA	-3.89162	-3.88982	-1.83112	-3.06333
Q8N4C6	Ninein (hNi	NIN KIAA15	-0.05561	-0.27872	-1.5202	-1.68312	-0.74193	0.100264	-0.73867	-0.4459	-0.91058	-0.90781
Q8N4C8	Misshapen·	MINK1 B55	-0.03063	0.155119	0.2824	0.425973	0.194503	0.459432	0.332124	0.275386	0.304855	0.254898
Q8N4H5	Mitochond	TOMM5 C9	1.110807	1.170791	-0.10932	-0.45317	-0.51245	-0.4975	0.449163	-0.38482	-0.21299	-0.79414
Q8N4N3	Kelch-like p	KLHL36 C1(	-0.89482	-0.80346	-0.73716	-1	-0.71508	-0.48826	-0.76451	-0.52291	-0.31857	-0.58054
Q8N4P3	Guanosine-	HDDC3 ME	0.668091	0.823534	0.497445	0.463703	0.905559	0.799458	0.937037	0.765721	0.937293	0.874753
Q8N4Q1	Mitochond	CHCHD4 M	-0.52727	-0.40622	-0.49272	-0.81584	-1.05964	-0.17913	-0.64243	-0.06577	-0.45472	-1.01181
Q8N4S0	Coiled-coil	CCDC82 HT	0.29548	0.437742	0.563717	0.445169	0.522204	0.636158	0.02032	0.028345	0.582215	0.634237
Q8N4T8	Carbonyl re	CBR4 SDR4	-3.30986	-4.83094	-3.24598	-3.9386	NA	NA	-3.61599	-3.80735	-4.08746	-4.0268
Q8N554	Zinc finger	ZNF276 CEI	-0.78705	-0.86901	-0.66425	-0.61783	-0.16763	-0.42976	-0.10901	-0.15513	-0.36722	-0.19289
Q8N573	Oxidation r	OXR1 Nbla(	-0.79717	-0.55625	-0.92405	-0.56937	-0.56217	-0.50894	-0.47781	-0.53596	-0.58021	-0.70165
Q8N584	Tetratricop	TTC39C C18	0.856726	0.944352	0.419903	0.601593	0.31895	0.451461	0.356304	0.162271	0.164677	0.521637
Q8N5A5	Zinc finger	ZGPAT GPA	-0.94081	-0.44546	-0.33736	-0.60607	-0.4861	-0.50574	-0.50367	-0.29109	-0.21383	-0.39752
Q8N5F7	NF-kappa-E	NKAP	-1.31849	-1.40407	-1.31153	-1.4973	-1.12105	-1.12421	-0.99592	-1.09531	-0.34998	-0.64691
Q8N5J2	Ubiquitin c	MINDY1 FA	-1.16089	-1.0342	-1.57069	-1.70888	-1.05718	-0.86562	-1.01778	-1.09744	-0.92234	-0.58094
Q8N5K1	CDGSH iror	CISD2 CDG	-1.02198	-1.06821	-1.45457	-1.35364	-1.12418	-1.75899	-1.22906	-0.82265	-1.848	-0.97369
Q8N5N7	39S ribosor	MRPL50	-1.43539	-0.78991	-1.23676	-0.89129	-1.57755	-1.33015	-0.95964	-1.23581	-1.21299	-1.12956
Q8N5P1	Zinc finger	ZC3H8 ZC3	-2.94062	-1.64874	-1.5427	-1.69574	-2.51245	-1.65208	-1.52484	-1.78746	-2.06756	-1.45391
Q8N5V2	Ephexin-1 (	NGEF	1.110807	1.334352	1.575836	1.294061	1.4902	1.703799	1.562621	1.6282	1.830075	1.676411
Q8N684	Cleavage aı	CPSF7	-0.74797	-0.65425	-0.88522	-1.06628	-0.81321	-0.96446	-0.95095	-0.92826	-0.83232	-0.73903
Q8N6F7	Germinal c	GCSAM GA	-1.19438	-1.18265	-0.96914	-0.87971	-1.14048	-1.33015	-1.42531	-1.03477	-1.57089	-0.87299
Q8N6H7	ADP-ribosy	ARFGAP2 Z	-0.72135	0.157253	0.092179	0.2265	-0.5609	-0.6749	-0.89532	-0.92291	-0.92511	-0.90567
Q8N6M0	Deubiquitir	OTUD6B DI	0.160465	0.310969	0.422815	0.63429	0.67155	0.427281	0.282513	0.35811	0.848521	0.903937
Q8N6N3	UPF0690 p	C1orf52 BA	1.302399	1.450186	1.019365	1.003133	1.029444	0.84739	0.961111	1.072569	1.228441	1.275153
Q8N6R0	eEF1A lysin	EEF1AKNM	-1.95388	-1.17142	-1.21126	-1.20648	-2.18307	-1.60365	-1.26362	-1.34359	-1.48394	-1.12959
Q8N6S5	ADP-ribosy	ARL6IP6 PF	-1.579	-1.24785	-0.91705	-1.53081	-1.53753	-1.1681	-1.38504	-1.11183	-1.4137	-1.17568
Q8N6T3	ADP-ribosy	ARFGAP1 A	-0.14716	-0.13024	-0.00782	-0.27563	-0.23359	-0.14958	-0.34514	-0.69188	-0.3932	-0.08194

Q8N7H5	RNA polym	PAF1 PD2	-0.07464	-0.05374	0.357362	0.193429	0.194005	0.021695	0.098853	0.050626	0.293359	0.272291
Q8N806	Putative E3	UBR7 C14o	-0.37505	-0.33638	-0.18996	-0.18882	-0.35771	-0.31179	-0.21802	-0.35449	-0.10804	0.00705
Q8N8A2	Serine/thre	ANKRD44	0.4975	0.649549	0.648528	0.88243	0.737666	0.84948	0.684604	0.58612	0.409117	0.347768
Q8N8A6	ATP-depen	DDX51	0.047697	0.603045	0.422815	0.198904	0.45537	0.584963	0.364274	0.265894	0.584963	0.605468
Q8N960	Centrosom	CEP120 CC	-3.30986	-1.7632	-1.93147	-1.13124	-3.14048	-1.72247	-2.20881	-2.04654	-2.30319	-1.71937
Q8N9E0	Protein FAI	FAM133A	-2.02458	-1.60226	-0.90059	-1.37049	-0.69754	-0.56489	-1.24016	-1.01075	-1.04306	-1.29469
Q8N9N8	Probable R	EIF1AD	0.549153	0.75089	0.768565	0.530907	0.80743	0.769633	0.750426	0.59807	1.303149	1.344423
Q8N9T8	Protein KRI	KRI1	-0.67238	-0.65618	-0.27437	-0.36062	0.126401	-0.03561	0.172992	0.032776	-0.05399	-0.20261
Q8NAV1	Pre-mRNA-	PRPF38A	-0.12003	-0.38672	-0.03553	0.13179	-0.26006	0.021695	-0.19118	-0.22239	-0.14365	0.202019
Q8NB16	Mixed linea	MLKL	0.049649	-0.14757	0.069164	0.047989	-0.28137	-0.00491	-0.38689	-0.26303	-0.27963	-0.18517
Q8NB37	Glutamine	GATD1 PDD	-1.22448	-0.64114	-0.72611	-0.85497	-0.85249	-0.7614	-0.92809	-0.90368	-0.64084	-0.54506
Q8NB90	ATPase fan	SPATA5 AF	-2.36452	-2.25918	-2.50901	-1.87533	-3.42999	-3.45943	-3.18536	-2.15987	-2.30319	-2.48307
Q8NBJ5	Procollager	COLGALT1	-2.04525	-2.07419	-2.41562	-2.33709	-2.23425	-2.46037	-1.87753	-1.73575	-1.20211	-0.8893
Q8NBJ7	Inactive C-a	SUMF2 PSE	-0.03625	0.048643	-0.15131	-0.06877	0.039058	0.547036	0.360449	0.35647	-0.47899	-0.83374
Q8NBL1	Protein O-g	POGLUT1 C	-1.04132	-1.15777	-1.41253	-1.22347	-1.80291	-1.32962	-1.34573	-2.04634	-1.92311	-1.74072
Q8NBN7	Retinol deh	RDH13 SDR	-1.91754	-1.25526	-1.30256	-1.05107	-1.33313	-1.30293	-1.83577	-3.22239	-1.14886	-0.89752
Q8NBQ5	Estradiol 1	HSD17B11	0.36114	0.391229	-0.01745	-0.06356	-0.02302	-0.03885	0.024129	0.110881	-0.13762	-0.17681
Q8NBS9	Thioredoxi	TXNDC5 TL	0.725946	0.874381	0.152642	-0.04191	-0.07126	-0.07847	-0.12146	-0.23954	0.125203	0.113678
Q8NBU5	ATPase fan	ATAD1 FNP	-1.60942	-1.52015	-1.87317	-1.54171	-1.7956	-1.55775	-1.74032	-1.39044	-1.86191	-1.87299
Q8NBX0	Saccharopi	SCCPDH CG	1.317071	1.081487	0.994139	1.046294	1.24871	1.485036	1.596851	1.241889	1.335069	1.402816
Q8NC44	Reticulopha	RETREG2 C	-1.47373	-1.73841	-0.65459	-0.87579	0.005955	-0.15694	-1.15377	-0.96941	-0.77341	-0.76354
Q8NC51	Plasminoge	SERBP1 PA	0.934121	0.822576	0.27919	0.13179	0.432658	0.455452	0.307532	0.326501	0.693897	0.93436
Q8NCA5	Protein FAI	FAM98A	-1.78629	-1.53138	-1.87317	-1.86821	-1.47063	-1.52083	-1.34514	-1.80735	-1.67243	-1.12956
Q8NCG7	Diacylglyce	DAGLB	-0.22239	-0.12199	-0.39696	-0.04459	-0.09212	0	-0.22069	-0.69188	-0.21299	-0.38873
Q8NCN4	E3 ubiquiti	RNF169 KIA	-1.78508	-2.1636	-1.56782	-1.41156	0.058299	-0.21785	-1.70901	-1.58886	-1.58302	-1.44909
Q8NCN5	Pyruvate d	PDPR KIAA	-1.20297	-0.51214	-0.48207	-1.3274	-1.2781	-0.42169	-0.42388	-0.36047	-0.61243	-0.60949
Q8NCW5	NAD(P)H-h	NAXE AIBP	-0.57289	-0.54839	-0.39183	-0.66558	-0.53382	-0.37906	-0.52484	-0.58496	-0.37474	-0.12473
Q8NCX0	Coiled-coil	CCDC150	1.413521	1.478447	1.674412	1.120294	1.516431	1.624491	1.465928	1.492362	1.594361	1.155098
Q8ND04	Protein SM	SMG8 ABC	-1.77502	-2.1531	-1.32566	-1.75104	-1.58097	-1.62953	-1.7723	-1.76756	-1.25422	-1.54356
Q8ND24	RING finger	RNF214	-1.26553	-1.46964	-1.40283	-1.53081	-1.75623	-2.23474	-2.11208	-2.24297	-1.46122	-1.48222
Q8ND56	Protein LSM	LSM14A C1	0.993925	0.655971	1.675633	1.425973	1.652753	1.538689	1.458501	1.35811	1.66594	1.564161
Q8ND71	GTPase IM	GIMAP8 IA	0.057029	0.254843	0.48845	0.527334	0.626031	0.894994	0.513226	0.510801	0.000955	0.065712
Q8NDH3	Probable a	NPEPL1 KIA	0.029995	-0.03154	0.468619	0.512062	0.270452	0.562936	0.299241	0.152003	0.463711	0.382591

Q8NDT2	Putative RI	RBM15B O	-0.16011	-0.01963	-1.89346	-1.98004	-1.46127	-2.03547	-1.35149	-2.11332	-4.08746	-3.54137
Q8NDX1	PH and SEC	PSD4 EFA6I	-1.00091	-0.82739	-0.8273	-1.02972	-0.89927	-0.8267	-1.06833	-1.30172	-1.22438	-0.89209
Q8NDX5	Polyhomec	PHC3 EDR3	-1.63771	-1.49481	-0.60999	-0.57373	-1.09684	-0.50265	1.040358	0.846087	-1.08524	-0.41517
Q8NE71	ATP-bindin	ABCF1 ABC	-0.20832	0.481302	0.352274	0.294263	0.177303	0.272061	0.237033	0.199098	0.328606	0.435907
Q8NE86	Calcium un	MCU C10or	0.383248	0.22173	-0.52191	-0.01775	-0.61883	-0.4813	-0.2402	-0.70155	-1.03333	-1.2649
Q8NEB9	Phosphatid	PIK3C3 VPS	-2.49964	-2.93155	-1.01568	-1.71621	-0.83173	-0.65208	-1.30666	-1.61973	-2.70443	-2.71212
Q8NEJ9	Neuroguidi	NGDN C14o	-1.46855	-1.2276	-0.99222	-1.06413	-1.14048	-1.28951	-1.43911	-1.10692	-1.4119	-0.84888
Q8NEY8	Periphilin-1	PPHLN1 HS	-0.18057	0.357362	0.246696	0.589138	0.726517	0.5025	-1.6705	-1.46264	0.397248	0.515458
Q8NEZ2	Vacuolar pi	VPS37A HC	0.552265	0.718917	0.88013	0.95541	0.81794	0.797956	0.788227	0.82362	0.719892	0.730757
Q8NEZ4	Histone-lys	KMT2C HAI	-2.36312	-2.55793	-1.1305	-0.7794	-2.04174	-2.33179	-2.25298	-2.29248	-1.82607	-2.26471
Q8NEZ5	F-box only	FBXO22 FB	0.431612	0.468619	0.049819	-0.05759	0.207442	0.105353	0.204745	0.131245	0.121991	0.334266
Q8NF37	Lysophospl	LPCAT1 AY	-0.679	-0.37278	-2.0079	-0.81717	-1.6476	-0.94188	-0.20582	-0.80861	-1.43108	-1.16393
Q8NF50	Dedicator c	DOCK8	-0.53153	-0.48017	-0.07604	-0.26061	-0.20551	-0.24028	-0.30523	-0.27966	-0.13848	-0.11376
Q8NF91	Nesprin-1 (	SYNE1 C6o	-0.37912	-0.61849	-0.99893	-1.26178	-0.68105	-0.88452	-1.0681	-0.84286	-1.20247	-1.33279
Q8NF99	Zinc finger	ZNF397 ZN	1.009064	1.529314	0.819558	0.546827	0.977516	1.056268	1.077195	0.384664	0.556393	0.439174
Q8NFC6	Biorientati	BOD1L1 BC	-0.53727	-0.50801	-0.60064	-0.64916	-0.29538	-0.59318	-0.59781	-0.77007	-0.55142	-0.30281
Q8NFD5	AT-rich inte	ARID1B BA	-0.07801	0.087359	0.242062	0.256184	0.305473	0.02626	0.159526	0.302901	0.4832	0.587008
Q8NFH3	Nucleopori	NUP43	-0.91373	-0.66884	-1.05756	-0.83817	-0.46004	-0.86576	-1.24601	-1.06919	-0.6905	-0.54268
Q8NFH4	Nucleopori	NUP37	-0.46855	-0.12624	-0.7632	-1.56009	-1.14048	-1.42968	-0.56212	-0.71989	-1.4892	-0.96502
Q8NFH5	Nucleopori	NUP35 MP	-1.10942	-0.8868	-1.17975	-0.9284	-0.97476	-1.09057	-1.27389	-1.26592	-0.93743	-1.09887
Q8NFI3	Cytosolic e	ENGASE	0.057394	0.183871	-0.26011	-0.30909	-0.07989	0.317889	-0.17846	-0.03684	0.464035	0.466995
Q8NFJ8	Class E basi	BHLHE22 B	-0.28749	0.072245	-0.34651	-0.85681	-0.19903	-0.08439	-0.24473	-0.53434	-0.15936	0.0859
Q8NFU3	Thiosulfate	TSTD1 KAT	1.163174	1.358883	0.206711	0.411898	1.388772	0.804229	1.175598	1.146841	0.91504	0.948188
Q8NFW8	N-acylneur	CMAS	0.053549	0.23349	0.12683	0.091148	0.220266	-0.02203	0.278301	0.222392	0.301033	-0.04495
Q8NG11	Tetraspanii	TSPAN14 T	-1.25096	-1.02359	-1.15634	-0.7794	-0.79256	-0.77761	-1.22069	-1.55102	-0.97199	-0.87299
Q8NHG8	E3 ubiquitii	ZNRF2 RNF	-0.13327	-0.19153	-0.15634	-0.59756	-0.53382	-0.54455	-0.5697	-0.36995	-0.88235	-0.59384
Q8NHQ9	ATP-depen	DDX55 KIA	-1.10516	-0.57331	-0.41201	-0.42609	-0.26724	-0.23254	-0.53849	-0.81315	-0.18092	0.072889
Q8NHV1	GTPase IM	GIMAP7 IA	-0.06193	-0.10164	-0.23922	-0.24187	-0.04551	-0.14434	-0.04183	0.071818	-0.02511	-0.33406
Q8NHV4	Protein NEI	NEDD1	-1.42286	-1.25602	-1.04388	-0.94186	-1.53014	-1.51811	-1.97603	-2.04612	-1.94711	-1.55092
Q8NHZ8	Anaphase-p	CDC26 ANA	-0.24377	-0.01568	-0.63656	-0.72655	-0.24236	-0.79647	-0.69661	-0.85834	-0.60634	-0.37145
Q8NI08	Nuclear rec	NCOA7 ERA	-2.57289	-1.75391	-1.90004	-2.4693	-2.55552	-1.60145	-1.89162	-1.78746	-2.33621	-1.84093
Q8NI27	THO compl	THOC2 CXo	-0.86139	-0.43335	-0.79667	-1.23078	-0.41546	-0.44684	-0.4957	-0.75811	-0.67996	-0.41799
Q8NI36	WD repeat	WDR36	-1.08185	-0.73993	-0.80619	-0.8404	-0.45783	-0.33524	-0.48945	-0.20601	-0.15234	-0.34391

Q8NI60	Atypical kir	COQ8A AD	-2.64689	-2.00782	-1.87317	-3.52356	-3.59991	-1.93587	-1.71329	-2.80735	-1.58496	-2.2402
Q8TAD8	Smad nucle	SNIP1	-1.12974	-0.77135	-0.83086	-0.6523	-0.79759	-0.6526	-0.94581	-1.00767	-1.23021	-0.73076
Q8TAE8	Growth arr	GADD45GI	-3.89482	-2.5541	-2.28345	-3.38606	-3.07635	-3.18641	-3.23266	-3.58496	-3.5025	-3.13927
Q8TAF3	WD repeat	WDR48 KIA	-0.90613	-0.45457	-1.38163	-0.90298	-0.56649	-0.36491	-0.60817	-0.55102	-0.83954	-0.7414
Q8TAK5	GA-binding	GABPB2	-0.9523	-0.87317	-0.5541	-0.26053	-0.7051	-0.2962	-0.42531	-0.24257	-0.45649	-0.18886
Q8TAQ2	SWI/SNF cc	SMARCC2 E	-0.05561	-0.13478	0.405256	-0.05107	0.161638	-0.26303	-0.03626	0.110183	0.009399	0.0649
Q8TAT6	Nuclear prc	NPLOC4 KI/	0.431612	0.490986	0.534708	0.580775	0.45537	0.555519	0.395724	0.526546	0.961781	0.995568
Q8TB03	Uncharacte	CXorf38	-2.61896	-1.60746	-1.517	-1.32227	-0.94425	-1.36534	-1.04156	-1.60078	-2.11209	-1.90588
Q8TB24	Ras and Ra	RIN3	-0.1087	0.069617	-0.17405	-0.15095	0.204986	-0.15867	-0.01827	0.03237	0.318725	-0.05219
Q8TB37	Iron-sulfur	NUBPL C14	-0.56168	-0.52717	-0.34524	-0.46961	-0.45938	-0.08011	-0.37047	-0.27925	-0.30254	-0.54379
Q8TB72	Pumilio hoi	PUM2 KIAA	-0.25952	-0.4086	0.79111	0.982101	0.963671	0.756702	1.084938	1.109075	1.078493	0.977089
Q8TBA6	Golgin subf	GOLGA5 RE	-0.96452	-0.86297	-0.71764	-0.16995	-0.54251	-0.64739	-0.41362	-1.30472	-0.87953	-0.82974
Q8TBC4	NEDD8-act	UBA3 UBE1	-0.15335	-0.08017	-0.24598	-0.45317	-0.29613	-0.57679	-0.36476	-0.41504	-0.24616	-0.0726
Q8TBC5	Zinc finger	ZSCAN18 Z	-0.65641	-0.48699	-0.47069	-0.14505	-0.10007	-0.02203	-0.22069	-0.42269	0.327575	0.399465
Q8TBF2	Prostamide	PRXL2B C1	0.528937	0.635393	0.065636	-0.36814	3.83E-05	0.132731	0.108595	0.304586	0.47101	0.174946
Q8TBX8	Phosphatid	PIP4K2C PI	0.750642	1.02167	1.074098	0.780219	1.033082	1.194011	1.206641	1.233543	1.063508	0.986396
Q8TC05	Nuclear prc	MDM1	0.705095	0.58756	0.737745	0.681987	1.036712	1.021695	0.66458	1.112832	1.384664	1.396106
Q8TC07	TBC1 doma	TBC1D15	-2.82871	-1.96628	-1.96153	-1.73697	-1.75951	-1.71284	-2.11181	-1.67494	-1.92461	-1.63448
Q8TC12	Retinol deh	RDH11 ARS	-2.03684	-2.03953	-1.7112	-0.41086	-1.81855	-1.74061	-2.09516	-0.94386	-2.64268	-1.73402
Q8TC90	Coiled-coil	CCER1 C12	-1.45187	-0.91667	-0.33662	0.209792	-0.22487	-0.52869	-0.60039	-0.71049	-0.5639	-0.1788
Q8TCA0	Leucine-ric	LRRC20 UN	-1.18057	-1.02359	-1.50901	-1.8011	-0.88567	-1.72247	-1.08427	-1.6735	-0.81444	-1.1788
Q8TCC3	39S ribosor	MRPL30 M	-3.25096	-2.64874	-3.24598	-3.67557	-3.89947	-3.04439	-2.18536	-3.58496	-3.00946	-3.30433
Q8TCD5	5'(3')-deox	NT5C DNT1	1.247289	1.36192	0.133954	0.209792	0.731004	0.23173	0.588369	0.691605	0.490805	0.964155
Q8TCF1	AN1-type z	ZFAND1	-0.20832	-0.06378	0.057333	-0.05759	-0.09212	0.273018	-0.07346	-0.34792	0.446516	0.114796
Q8TCJ2	Dolichyl-di	STT3B SIMI	-0.4136	-0.62935	-0.84805	-0.81316	-1.42583	-0.97526	-1.18115	-0.90814	-0.84516	-0.72591
Q8TCS8	Polyribonu	PNPT1 PNP	-0.77588	-0.78658	-0.50171	-0.49402	-0.31451	-0.31638	-0.3646	-0.21938	-0.23742	-0.35797
Q8TCT9	Minor histc	HM13 H13	0.063387	-0.03678	-1.95743	-1.62423	-1.96388	-0.82369	-0.15677	-0.60052	-1.4107	-1.99584
Q8TCU6	Phosphatid	PREX1 KIA	-2.89482	-2.00782	-1.63656	-1.82312	-1.66918	-1.56866	-2.60583	-1.95491	-2.30319	-2.31568
Q8TD16	Protein bic	BICD2 KIAA	-0.54143	-0.41244	-1.24742	-1.03356	-0.40648	-0.42833	-0.49433	-0.53469	-1.36042	-1.56439
Q8TD19	Serine/thre	NEK9 KIAA	0.28235	0.295202	0.752581	0.611327	0.696848	0.693623	0.554089	0.704856	1.120271	0.920306
Q8TD55	Pleckstrin	PLEKHO2 P	-0.62234	-0.6771	0.093597	-0.07946	-0.6843	0.096455	-0.05568	-0.02769	-0.18326	-0.35496
Q8TDB6	E3 ubiquiti	DTX3L BBA	-0.13327	-0.08844	0.172522	0.160134	-0.05302	-0.0617	0.186878	0.13357	0.284629	0.095162
Q8TDD1	ATP-depen	DDX54	-1.72489	-1.69849	-2.34156	-2.81177	-2.51245	-1.89465	-2.72311	-1.56789	-2.06756	-2.32636

Q8TDH9	Biogenesis	BLOC1S5 M	-1.48214	-1.58701	0.256521	0.204358	1.076044	0.813587	-1.12976	-1.44896	-1.37262	-1.6692
Q8TDJ6	DmX-like p	DMXL2 KIA	-0.06827	0.023207	0.179425	0.120294	0.079567	0.31755	0.274077	0.375867	0.250407	0.435907
Q8TDN6	Ribosome b	BRIX1 BRIX	0.065184	0.91983	1.112475	1.57447	0.895942	1.517848	0.730813	1.008562	1.06454	0.831181
Q8TDP1	Ribonuclea	RNASEH2C	-4.08746	-2.30256	-2.44392	-3.03814	NA	NA	-2.89162	-2.76783	-3.25739	-3.0268
Q8TDX7	Serine/thre	NEK7	-1.21168	-0.95236	-0.78176	-0.66292	-0.27555	-0.98914	-0.87668	-0.85693	-0.97075	-0.56065
Q8TDZ2	[F-actin]-m	MICAL1 MI	-0.69125	-0.24046	-0.50072	-0.47292	-0.77153	-0.44821	-0.58496	-0.71989	-0.38544	-0.39186
Q8TE02	Elongator c	ELP5 C17or	-0.61869	-0.5427	-0.70483	-0.23078	-0.07635	-0.40054	-0.6477	-0.48543	-0.26303	-0.00888
Q8TE77	Protein pho	SSH3 SSH3I	-0.90743	-0.68536	-1.11331	-0.96565	-1.86002	-0.91144	-0.9672	-0.86796	-1.09266	-0.99764
Q8TEA8	D-aminoac	DTD1 C20o	0.299939	0	-0.15634	0.67021	0.609541	-0.05004	0.411195	0.490326	0.13493	0.287896
Q8TEB1	DDB1- and	DCAF11 WI	-0.95073	-0.94799	-0.71415	-0.94051	-1.26724	-0.84693	-0.81276	-0.8511	-0.95147	-1.00324
Q8TEM1	Nuclear po	NUP210 KI	0.355603	0.295167	-0.30026	-0.05433	-0.64572	-0.22422	0.122543	-0.14674	-0.51592	-0.58718
Q8TEQ6	Gem-assoc	GEMIN5	-0.70499	-0.83789	-0.89476	-0.60709	-0.74193	-0.38618	-1.01396	-0.87561	-0.68753	-0.63448
Q8TEQ8	GPI ethano	PIGO UNQ6	-2.85042	-1.44392	-1.13906	-0.26053	-2.79256	-2.26303	-1.60039	-3.16993	-1.5025	-1.60726
Q8TEU7	Rap guanin	RAPGEF6 P	-1.15335	-1.20945	-0.48699	-0.67557	-0.77973	-0.55254	-0.50293	-0.56789	-0.29739	-0.1788
Q8TEW0	Partitioning	PARD3 PAR	-0.82873	-0.41244	0.108863	-0.26053	0.043943	-0.11366	-0.3322	0.056143	-0.10257	-0.06333
Q8TEX9	Importin-4	IPO4 IMP4I	0.13553	0.190187	-0.11791	-0.35044	-0.76304	-0.75288	-0.43122	-0.39514	-0.47083	-0.2268
Q8TF01	Arginine/se	PNISR C6or	0.431612	0.675633	0.88013	0.716752	0.973734	0.759737	0.641808	0.874469	1.089126	1.396106
Q8TF05	Serine/thre	PPP4R1 ME	-0.84964	-0.64812	-0.66221	-1.07689	-1.22815	-1.00127	-1.79578	-1.00104	-0.85527	-0.95913
Q8TF42	Ubiquitin-a	UBASH3B K	-1.01217	-0.78991	-0.66102	-0.55087	-0.69302	-0.26961	-0.44606	-1.18286	-0.95361	-0.84093
Q8TF46	DIS3-like ex	DIS3L DIS3I	-2.53727	-3.24598	-1.93147	-1.9386	-1.53382	-1.87447	-2.20881	-1.78746	-1.31487	-1.95641
Q8TF68	Zinc finger	ZNF384 CA	1.376305	1.377791	1.26567	1.381095	1.497175	1.372999	1.422555	1.163522	1.011411	0.909891
Q8WTT2	Nucleolar c	NOC3L AD2	-1.5025	-2.34156	-1.17383	-1.3377	-0.7051	-1.0902	-0.88216	-0.85834	-1.58496	-0.78649
Q8WTW3	Conserved	COG1 KIAA	-1.87042	-1.06948	-0.87786	-1.23082	-0.67132	-0.71814	-1.18068	-0.87546	-1.14719	-1.06209
Q8WU79	Stromal me	SMAP2 SM	1.009608	1.056147	1.1004	1.008729	1.187048	1.126339	0.928867	1.114019	1.363482	1.442249
Q8WU90	Zinc finger	ZC3H15 DF	-0.24661	-0.09373	0.034673	-0.16672	0.316755	-0.01048	0.110045	0.039528	0.044437	-0.28264
Q8WUA2	Peptidyl-pr	PPIL4	-0.90613	-1.1305	-0.94573	-1.07634	-0.74416	-0.87566	-1.29406	-1.02308	-1.32665	-1.26127
Q8WUA4	General tra	GTF3C2 KIA	-0.40537	-0.4059	-0.3195	-0.45964	-0.32973	-0.50703	-0.75898	-0.83026	-0.7878	-0.65033
Q8WUA7	TBC1 doma	TBC1D22A	-1.76553	-1.61252	-2.50391	-2.58469	-3.69302	-1.47454	-1.96963	-3.02308	-2.31645	-2.61518
Q8WUD4	Coiled-coil	CCDC12	-0.42556	-0.15297	0.216563	0.381166	0.060694	-0.05143	-0.02126	0.122078	0.165762	0.194501
Q8WUF8	Cotranscrip	FAM172A (	0.217805	0.1859	0.286927	0.678115	0.345789	0.387759	0.380636	0.352411	0.496281	0.399995
Q8WUH6	Transmeml	TMEM263	-1.38702	-1.29298	-0.84488	-0.60709	-1	-0.84472	-0.6477	-0.56789	-0.1541	-0.35437
Q8WUI4	Histone de	HDAC7 HDA	-1.04933	-0.97679	-1.33171	-1.2909	-1.35199	-1.52083	-1.52484	-2.07039	-1.55695	-1.21945
Q8WUM4	Programme	PDCD6IP A	0.418065	0.3725	0.479846	0.319417	0.405712	0.443446	0.598541	0.344648	0.569161	0.593786

Q8WUQ7	Cactin (Rer	CACTIN C19	-0.50148	-0.60191	-0.28267	-0.42393	-0.26406	-0.25039	0.469347	0.528765	-0.38743	-0.1335
Q8WUR7	UPF0235 p	C15orf40	-2.53727	-2.02359	-1.52015	-1.03814	-2.95606	-1.37197	-1.53964	-1.82753	-1.3505	-1.28264
Q8WUW1	Protein BRI	BRK1 C3orf	0.491726	0.629653	-0.3977	-0.28397	-0.15726	-0.32195	-0.04994	-0.20825	-0.30008	-0.28894
Q8WUX9	Charged m	CHMP7	0.278595	0.265428	-0.02499	-0.11241	0.473133	0.436579	0.29291	0.039346	1.035841	0.729866
Q8WUZ0	B-cell CLL/l	BCL7C	-2.0607	-1.08104	-1.32376	-1.69611	-1.74565	-2.12832	-1.60851	-2.22945	-1.20984	-0.65199
Q8WVC0	RNA polym	LEO1 RDL	-1.2949	-0.91667	-0.73048	-0.5787	-0.72955	-0.44448	-0.84493	-0.50967	-0.68753	-0.56737
Q8WVJ2	NudC doma	NUDCD2	-1.25096	-1.09675	-0.94642	-0.5881	-0.87199	-0.74977	-0.97968	-1.0115	-0.78978	-0.77887
Q8WVK2	U4/U6.U5 s	SNRNP27	1.341196	0.984318	1.447329	1.385731	1.273531	1.087463	1.415037	1.346901	1.386468	1.365517
Q8WVM7	Cohesin sul	STAG1 SA1	0.501857	0.730221	0.826978	0.800958	0.344221	0.618979	0.649886	0.636279	0.825762	0.742188
Q8WVM8	Sec1 family	SCFD1 C14	-0.36892	-0.36647	-0.08306	-0.43453	-0.31451	-0.34243	-0.14475	-0.25566	-0.4871	-0.75627
Q8WVT3	Trafficking	TRAPPC12	-1.02445	-1.4761	-1.2276	-1.17306	-1.71727	-1.55254	-1.5697	-1.88982	-1.01898	-1.09134
Q8WVV9	Heterogen	HNRNPLL H	-0.67565	-0.5541	-1.14275	-1.18735	-1.04532	-1.35789	-1.29981	-1.89044	-1.46321	-1.62087
Q8WVY7	Ubiquitin-li	UBLCP1	0.419871	0.316876	0.543497	0.542682	0.328357	0.484449	0.55883	0.453601	0.736782	0.749327
Q8WW12	PEST prote	PCNP	0.032241	0.06666	0.696412	0.765502	0.900584	0.813587	0.761315	0.743533	1.036758	1.039983
Q8WWI1	LIM domai	LMO7 FBX2	-0.52953	-0.77489	-0.35331	-0.59283	-0.42004	-0.09781	-0.38389	-0.18939	0.2565	0.151068
Q8WWM7	Ataxin-2-lik	ATXN2L A2	-0.41596	-0.31231	0.009206	0.141504	0.021246	-0.23006	-0.3452	-0.12417	-0.3327	-0.29008
Q8WWP7	GTPase IM	GIMAP1 IM	-0.21337	-0.15727	-0.12238	-0.4386	-0.27722	0.163287	0.005941	0.033583	-0.4328	-0.20527
Q8WWQ0	PH-interact	PHIP DCAF	0.707516	0.526714	0.811705	0.565833	0.604658	0.573991	0.793732	0.834789	0.899857	0.962387
Q8WWV3	Reticulon-4	RTN4IP1 NI	-1.74507	-2.00782	-1.45457	-1.26053	-3.07635	-1.95693	-1.60039	-2.80735	-2.04793	-2.19898
Q8WWW0	Ras associa	RASSF5 NO	0.244516	0.117981	0.152701	0.272106	0.541686	0.380143	0.202789	0.286588	0.075724	0.533717
Q8WWY3	U4/U6 sma	PRPF31 PRI	0.597035	0.649475	-0.34156	-0.23078	0.321115	0.457409	0.303392	-0.05842	0.230452	-0.33192
Q8WX92	Negative el	NELFB COB	-0.1004	-0.06378	0.240108	0.288936	0.270452	0.095157	0.186878	0.157146	0.06454	0.102483
Q8WXA9	Splicing reg	SREK1 SFRS	0.045574	0.154589	-0.34744	-0.48998	-0.10372	-0.26045	-0.40783	-0.5898	-0.58787	-0.39768
Q8WXF1	Paraspeckl	PSPC1 PSP1	-0.411	-0.47069	-0.73291	-0.48794	-0.269	-0.16788	-0.76451	-0.60224	-0.43721	-0.3431
Q8WXG6	MAP kinase	MADD DEN	-1.28018	-0.85577	-0.47325	-0.47798	-0.29092	-0.45444	-0.65562	-0.52421	-0.48805	-0.30217
Q8WXH0	Nesprin-2 (	SYNE2 KIAA	-0.31237	-0.50337	-0.27427	-0.39582	-0.68853	-0.56971	-0.39645	-0.45372	-1.07018	-1.03639
Q8WXI9	Transcripti	GATAD2B K	-0.82504	-0.79238	-1.19616	-1.59806	-0.7974	-0.44249	-0.25691	0.094182	-0.95199	-1.24568
Q8WXX5	DnaJ homo	DNAJC9	0.819082	1.109185	0.686877	0.929714	0.693242	0.781962	0.855841	0.981885	0.774762	0.833462
Q8WYA6	Beta-caten	CTNNBL1 C	-0.36344	-0.70483	-1.08017	-1.18728	-1.26304	-1.14958	-1.09516	-1.27684	-1.24343	-0.35437
Q8WYH8	Inhibitor of	ING5	0.34337	0.296415	0.626428	0.369624	0.754681	0.639648	0.589765	0.538933	0.630978	0.629214
Q8WYJ6	Septin-1 (L	SEPTIN1 DI	0.441513	0.532258	0.60561	0.895977	0.750795	0.769387	0.380085	0.712718	0.685127	0.634265
Q8WYL5	Protein pho	SSH1 KIAA1	-3.13993	-2.15634	-1.72403	-2.0126	-2.95606	-3.04439	-1.9301	-1.31907	-1.52947	-1.59384
Q8WYP5	Protein ELY	AHCTF1 EL	-0.74507	-0.77649	-0.41764	-1.03332	-0.81362	-1.1275	-0.76729	-1.14974	-0.47603	-0.45998

Q8WYQ5	Microproce	DGCR8 C22	-1.69545	-1.97345	-1.15954	-1.58863	-1.61145	-1.35039	-0.96002	-1.07987	-0.95141	-1.19122
Q8WZ82	Esterase O\	OVCA2	0.409154	0.631008	0.134309	0.106771	0.293324	0.095622	0.52108	0.53409	0.027381	-0.09729
Q8WZA0	Protein LZI	LZIC	-0.67924	-0.63864	-0.52659	-0.60955	-0.40905	-0.46374	-0.63084	-0.68268	-0.42199	-0.27823
Q92499	ATP-depen	DDX1	0.352829	0.375509	0.42862	0.529549	0.422455	0.31755	0.235488	0.275386	0.540274	0.698396
Q92504	Zinc transp	SLC39A7 H	1.433857	1.284002	0.819558	1.176878	0.59976	0.90105	1.145851	1.144254	0.082462	-0.29888
Q92506	Estradiol 1	HSD17B8 F	0.014324	0.08948	0.425128	0.323878	0.381851	0.454852	-0.3493	0.151257	0.57168	0.524467
Q92520	Protein FAI	FAM3C ILE	-2.57289	-1.93147	-1.93147	-1.45317	-2.64572	-1.72247	-1.71329	-1.97728	-2.16993	-2.00888
Q92522	Histone H1	H1-10 H1F)	0.148582	0.185415	0.265715	-0.05596	0.207442	0.192459	0.50965	0.060188	0.040794	0.289879
Q92530	Proteasom	PSMF1	0.748939	0.821646	0.676524	0.714062	0.748109	0.728412	0.781624	0.882308	0.779884	0.855428
Q92538	Golgi-speci	GBF1 KIAA(	0.181998	0.320368	0.714171	0.765535	0.584963	0.169925	0.550447	0.308122	0.15624	0.069124
Q92541	RNA polym	RTF1 KIAA(	-0.31922	-0.28641	-0.12667	-0.33801	-0.05484	-0.12679	-0.56272	-0.42358	-0.30521	-0.21025
Q92552	28S ribosor	MRPS27 KI.	-1.0747	-0.58875	-1.08246	-1.2505	-1.77125	-1.07608	-0.66626	-1.37217	-1.426	-0.89486
Q92556	Engulfmen	ELMO1 KIA	-0.65641	-0.35147	0.193132	0.215206	-0.39046	0.33931	-0.25081	0.384664	0.456858	0.224919
Q92563	Testican-2	SPOCK2 KI/	-1.11346	-0.59474	-0.5656	-0.5787	-0.44004	-0.57679	-1.17377	-0.96605	-0.72601	-1.0268
Q92572	AP-3 comp	AP3S1 CLA	0.183066	0.229499	0.411079	0.093152	0.497869	0.250256	0.309655	0.599832	0.325762	0.169455
Q92575	UBX domai	UBXN4 KIA	-1.43539	-1.1305	-1.2185	-1.10402	-0.9417	-1.16222	-1.25789	-1.30485	-1.65748	-1.38294
Q92576	PHD finger	PHF3 KIAA(	-0.73315	-0.74348	-0.45457	-0.72655	-0.29613	-0.39422	-0.73016	-0.32623	-0.35652	-0.0726
Q92597	Protein ND	NDRG1 CAI	-1.40671	-0.96872	-1.69384	-1.3227	-1.98972	-1.40014	-1.75267	-2.05555	-2.20706	-2.03839
Q92598	Heat shock	HSPH1 HSP	0.082462	0.295167	-0.09562	-0.0172	0	0.275627	-0.07346	-0.22641	0.185556	0.110703
Q92600	CCR4-NOT	CNOT9 RCD	-1.85109	-1.45834	-0.66683	-0.69737	-1.38831	-0.72505	-0.95327	-1.21744	-1.4245	-1.47947
Q92608	Dedicator c	DOCK2 KIA	0.203214	0.246759	0.2824	0.252542	0.276604	0.676705	0.394092	0.34915	0.139217	0.305908
Q92609	TBC1 doma	TBC1D5 KI/	-0.45724	-0.57476	0.049516	-0.11944	-0.0836	-0.19174	-0.11084	-0.09943	-0.17388	-0.42465
Q92610	Zinc finger	ZNF592 KIA	-2.5099	-2.77947	-1.85896	-1.10402	-2.97122	-3.02631	-2.01027	-2.80735	-3.36018	-2.9601
Q92614	Unconvent	MYO18A C	-0.06193	-0.07368	0.139864	0.035338	-0.29613	-0.0902	-0.19704	-0.32623	-0.45649	-0.47087
Q92616	eIF-2-alpha	GCN1 GCN	0.105182	0.405256	0.369485	0.329436	-0.09212	0.115477	-0.01543	-0.02891	0.446516	0.174834
Q92619	Rho GTPas	ARHGAP45	0.275107	0.299828	0.249978	0.397684	0.466086	0.692817	0.396714	0.392749	0.40439	0.389364
Q92620	Pre-mRNA-	DHX38 DD)	-0.15725	-0.3282	-0.07452	-0.34672	-0.25784	-0.05571	-0.52693	-0.14228	-0.35993	-0.16938
Q92621	Nuclear po	NUP205 C7	-1	-1.01568	0.259781	-0.34564	-0.06853	-0.28951	-1.55459	-1.33342	-1.08746	-1.03585
Q92623	Tetratricop	TTC9 KIAA(	-3.03684	-2.5541	-2.02359	-2.9386	-2.74193	-1.66935	-2.16227	-2.1193	-1.91754	-1.79414
Q92625	Ankyrin rep	ANKS1A AN	-1.11346	-1.23676	-1.19153	-1.86821	-2.84503	-1.28951	-1.38466	-1.1193	-1.37474	-1.42986
Q92664	Transcripti	GTF3A	-1.1669	-1.5427	-0.83789	-1.26053	-0.69302	-0.86449	-1.13955	-1.6735	-1.59918	-1.00888
Q92665	28S ribosor	MRPS31 IM	0.050608	0.120486	0.077755	-0.01252	-0.37724	-0.04686	0.15994	0.274827	0.115442	0.095786
Q92685	Dol-P-Man	ALG3 NOT	-3.19438	-3.77649	-3.28345	-2.84549	-4.01495	-3.79647	-3.68012	-3.33342	-4.25739	-3.54137

Q92688	Acidic leuci	ANP32B AF	1.360211	1.309396	1.464386	1.339385	1.232977	1.268489	1.143537	1.369234	1.495829	1.054284
Q92696	Geranylger	RABGGTA	1.879969	2.005837	1.179425	1.187933	1.602211	1.224733	1.443531	1.457348	1.264213	1.327229
Q92733	Proline-rich	PRCC TPRC	-0.62803	-0.44923	-0.12199	-0.21613	-0.28703	0.100264	-0.47422	-0.24257	0.297201	0.073337
Q92734	Protein TF(	TFG	-0.5202	-0.1829	-0.72173	-0.52851	-0.77058	-0.3506	-0.45511	-0.47763	-0.62583	-0.17839
Q92769	Histone de	HDAC2	0.068052	-0.0699	-0.02574	-0.07167	0.250307	0.212801	0.057194	0.21224	0.298964	0.564596
Q92783	Signal trans	STAM STAN	1.566662	1.567966	1.676853	1.654356	1.513829	1.733683	1.646718	1.6282	1.562791	1.238112
Q92785	Zinc finger	DPF2 BAF4	-0.5639	-0.83789	-0.61849	-0.67557	-0.30491	-0.36318	-1.02061	-0.61096	-1.04793	-0.04039
Q92793	CREB-bindi	CREBBP CB	-0.30986	0.053581	0.12683	0.160134	0.28883	0	-0.0574	-0.18938	0.304855	0.654087
Q92794	Histone ace	KAT6A MO.	-1.17081	-1.04965	-2.00782	-2.71621	-1.22487	-1.65208	-1.91073	-1.60224	-1.57089	-1.6763
Q92797	Symplekin	SYMPK SPK	-0.02429	-0.04496	0.377526	0.33762	0.244567	0.097023	-0.07999	-0.31734	0.013523	0.017402
Q92800	Histone-lys	EZH1 KIAA(	-1.70499	-1.91667	-1.31221	-1.05107	-1.42999	-1.42968	-2.07346	-3.51785	-4.45003	-1.80957
Q92804	TATA-bindi	TAF15 RBP:	-1.00953	-0.58132	-0.50217	-0.47856	-0.71939	-0.69413	-0.056	0.129054	-0.47333	-0.24366
Q92805	Golgin subf	GOLGA1	-3.37126	-2.19153	-2.20945	-2.0126	-2.95606	-3	-1.9301	-2.09464	-2.14886	-1.73402
Q92828	Coronin-2A	CORO2A IR	-2.54938	-1.47565	-1.82602	-2.66562	-2.78251	-2.2463	-2.0717	-2.65086	-2.59249	-1.89107
Q92835	Phosphatid	INPP5D SHI	-0.67565	-0.66102	-0.16069	-0.0126	-0.57755	-0.32329	-0.22912	-0.24257	-0.63678	-0.83303
Q92841	Probable A	DDX17	0.40439	0.307822	0.370025	0.198904	0.531557	0.106015	0.422691	0.211592	0.353637	0.47782
Q92844	TRAF famil	TANK ITRAI	0.266282	0.153585	0.596098	0.494152	0.567533	0.296824	0.409304	0.321588	0.437886	0.287548
Q92851	Caspase-1C	CASP10 MC	-0.91754	-0.63656	-1.41244	-1.17306	-1.12418	-0.85457	-0.9301	-1.33342	-0.95361	-0.81735
Q92854	Semaphori	SEMA4D C:	-0.61869	-0.68589	0.332805	-0.17306	0.14828	0.212994	-0.30035	0.177538	0.197939	-0.04039
Q92878	DNA repair	RAD50	-0.47697	-0.31285	0.092836	0.15451	-0.09212	-0.15565	-0.31636	-0.49491	-0.28011	-0.02935
Q92882	Osteoclast-	OSTF1	-0.41752	0.020037	-0.29704	-0.34362	-0.70331	-0.60145	-0.63176	-0.46158	-0.57791	-0.93288
Q92888	Rho guanin	ARHGEF1	-0.16011	-0.08844	-0.10932	-0.46179	-0.26503	-0.26961	-0.18008	-0.20901	-0.39941	-0.2699
Q92890	Ubiquitin re	UFD1 UFD1	-0.36196	0.043505	0.902584	0.944959	0.347407	0.582688	0.902699	0.37418	0.57836	0.697697
Q92896	Golgi appa	GLG1 CFR1	-0.14064	-0.09042	-1.16273	-0.69589	-1.28934	-1.01056	-1.08443	-1.1855	-1.0109	-1.09384
Q92900	Regulator c	UPF1 KIAA(	0.110807	0.175978	0.314109	0.257798	0.342816	0.52166	0.31988	0.534473	0.250407	0.455403
Q92918	Mitogen-ac	MAP4K1 HI	-1.43137	-1.4928	-1.40765	-1.97222	-1.39804	-1.13841	-2.21912	-1.47098	-1.63941	-1.36881
Q92922	SWI/SNF cc	SMARCC1 E	0.963163	0.515742	1.162105	1.368829	1.342602	1.263945	1.175598	1.436348	1.323813	1.095322
Q92925	SWI/SNF-re	SMARCD2 I	-0.26577	-0.40726	-1.27504	-1.24496	-1.69382	-1.1745	-1.15468	-1.49837	-1.0305	-0.93734
Q92930	Ras-relatec	RAB8B	0.265054	0.249978	1.087004	0.875609	0.826352	1.120513	1.010196	1.123382	1.089126	0.894446
Q92945	Far upstrea	KHSRP FUB	-0.31268	-0.33199	0.079643	0.368829	-0.04591	-0.13879	-0.22737	-0.02814	0.195062	0.248856
Q92947	Glutaryl-Cc	GCDH	-0.38685	0.040191	0.110759	0.260891	0.101393	0.503902	0.511822	0.40017	0.055145	0.120223
Q92973	Transportir	TNPO1 KPN	-0.79314	-0.45457	-0.74102	-0.64738	-0.55552	-0.32329	0.141219	-0.10076	-0.49584	-0.72249
Q92974	Rho guanin	ARHGEF2 K	-0.70988	-0.69031	-0.23613	-0.60521	-0.62274	-0.43847	-0.61889	-0.56979	-0.65278	-0.20578

Q92979	Ribosomal	EMG1 C2F	-0.12003	0.042266	-0.85896	-0.26806	-0.49139	-0.06711	-0.34514	-0.6644	-0.31487	-0.1589
Q92989	Polyribonu	CLP1 HEAB	-0.78629	-0.93892	-0.88753	-0.72655	-1.45017	-1.2115	-1.07346	-1.04654	-1.06756	-1.36004
Q92993	Histone ac	KAT5 HTAT	-0.32496	-0.60064	-0.48153	-0.25303	0.107446	-0.28284	-0.19704	-0.27684	-0.71823	-0.41209
Q93008	Probable u	USP9X DFF	-1.25096	-1.03953	-0.52997	-0.55424	-0.26827	-0.54244	-1.13955	-1.65535	-0.66821	-0.62087
Q93009	Ubiquitin c	USP7 HAUS	-0.1635	0.277254	-0.41244	-0.60709	-0.39099	-0.33703	-0.41163	-0.38639	-0.17597	-0.2402
Q93033	Immunoglo	CD101 EWI	0.856726	0.801774	-0.16069	-0.05759	-0.10007	0.115477	1.758294	1.914504	0.778786	0.412823
Q93034	Cullin-5 (CU	CUL5 VACM	0.33082	0.445074	0.222702	0.298988	0.139093	0.338236	0.147006	0.211529	0.481381	0.501438
Q93052	Lipoma-pre	LPP	-2.0747	-1.63154	-1.498	-3.10661	-2.80556	-2.20465	-2.43221	-2.18339	-2.35053	-2.22067
Q93074	Mediator o	MED12 ARC	-1.9459	-1.44376	-1.49047	-2.11654	-2.95031	-2.32917	-2.13955	-1.86694	-1.61671	-1.73612
Q93084	Sarcoplasm	ATP2A3	0.697639	0.69984	0.609595	0.438287	0.377367	0.334984	0.800028	0.423745	0.04182	-0.08194
Q95604	HLA class I	HLA-C HLAC	-1.68536	-1.69849	-0.95395	-0.98751	-2.69302	-1.56866	-1.71329	-1.93289	-1.37474	-1.79414
Q95IE3	HLA class II	HLA-DRB1	1.317071	1.3266	0.223506	0.187933	-1.74193	-0.82523	0.78229	0.760812	-0.53629	-0.51584
Q969E4	Transcriptio	TCEAL3 MS	-0.27301	-0.02167	0.468619	0.280103	0.993758	0.662965	0.93248	0.622633	0.944027	0.896825
Q969E8	Pre-rRNA-p	TSR2	1.056467	1.332805	0.994139	1.460572	1.194503	2.008174	1.926538	1.899237	0.507484	-0.59384
Q969G3	SWI/SNF-re	SMARCE1 E	-0.20832	-0.25992	-0.08231	-0.4924	0.044123	-0.06699	-0.17483	-0.15558	0.110703	0.223894
Q969H8	Myeloid-de	MYDGF C19	1.366845	1.313164	1.218488	1.383329	1.474603	1.381247	1.455702	1.406612	1.057762	0.997786
Q969J2	Zinc finger	ZKSCAN4 Z	-2.85042	-3.13906	-3.6734	-3.0906	-2.42999	-2	-2.13955	-1.97728	-2.16993	-3.1008
Q969M3	Protein YIP	YIPF5 FING	-0.75527	-0.57719	0.87376	0.739472	0.48755	0.245625	-0.94973	-0.95491	-0.848	-0.72668
Q969M7	NEDD8-con	UBE2F NCE	-1.21569	-0.03953	-0.08112	0.149768	0.362474	0.057596	-0.06746	-0.01033	0.051598	0.13331
Q969N2	GPI transar	PIGT CGI-0	-1.74779	-1.95873	-2.32444	-1.68003	0.141554	0.236377	0.464075	0.261125	-0.03822	-0.1589
Q969P0	Immunoglo	IGSF8 CD81	-1.44684	-1.51218	-1.10979	-0.86649	-1.23097	-1.17387	-1.37838	-1.41659	-0.76127	-0.61472
Q969Q0	60S riboson	RPL36AL	-0.09029	-0.32611	-0.53721	-0.90666	-0.31748	-0.6703	-0.26434	-0.58506	-0.77902	-0.5998
Q969Q5	Ras-related	RAB24	-1.15335	-1.20945	-1.69849	-1.3377	-1.62263	-1.03317	-1.05209	-1.14439	-1.32665	-1.40621
Q969R8	KICSTOR co	ITFG2	-3.72489	-4.36146	-3.77649	-3.52356	-3.2076	-4.23704	-4.81762	-3.1193	-2.37474	-2.54137
Q969S3	Zinc finger	ZNF622 ZPI	-2.76142	-2.34115	-3.0342	-2.28085	-2.83422	-3.12558	-2.19708	-1.951	-2.44682	-2.81032
Q969S9	Ribosome-r	GFM2 EFG2	-2.85042	-2.34156	-3.4021	-3.45317	-2.59991	-2.83494	-1.96963	-3.88982	-2.1281	-2.08194
Q969W3	Protein FAI	FAM104A	-1.81299	-1.78102	-1.97679	-3.14505	-1.45017	-2.79647	-2.28998	-1.924	-2.08746	-2.51584
Q969X5	Endoplasm	ERGIC1 ERC	-1.55497	-1.48699	-1.66102	-1.71621	-1.51245	-1.44448	-1.74723	-1.53434	-2.1913	-2.0268
Q969X6	U3 small nu	UTP4 CIRH1	-1.01217	-0.5427	-0.0516	0.097024	-0.72955	-0.0902	-0.68834	-0.93289	-0.47603	-0.49075
Q969Y2	tRNA modi	GTPBP3 MT	-1.80471	-1.33442	-1.39361	-1.6899	-1.42915	-1.13301	-0.99253	-0.82258	-1.16891	-1.15304
Q969Z0	FAST kinase	TBRG4 CPR	0.376645	0.216811	0.220163	0.085247	0.200987	-0.17403	-0.17377	-0.47743	-0.51592	0.749304
Q96A19	Coiled-coil	CCDC102A	-1.60942	-1.37151	-0.0516	-1.30633	-1.07635	-1.17403	-1.37137	-1.39232	-1.25739	0
Q96A26	Protein FAI	FAM162A (	0.791683	0.90533	0.91983	0.826935	1.226636	1.117997	1.141219	1.099536	0.925007	1.135088

Q96A33	Coiled-coil	CCDC47 GK	-1.12663	-1.52015	-1.93147	-1.9386	-3.2076	-3.23704	-1.5697	-1.88982	-1.7028	-1.99118
Q96A35	39S ribosor	MRPL24	-0.00619	-0.05803	-0.30952	-0.17839	-0.44283	-0.09377	0.017528	-0.34067	-0.426	-0.2466
Q96A49	Synapse-as	SYAP1 PRO	-1.1236	-1.02394	-0.75039	-0.69175	-0.15454	-0.40272	-0.74295	-0.91951	-0.5793	-0.7161
Q96A65	Exocyst cor	EXOC4 KIA/	0.192645	-0.16069	0.226842	0.079322	0.014797	0.208271	0.251129	0.254993	0.230452	-0.1589
Q96AE4	Far upstrea	FUBP1	0.323765	0.345135	0.453019	0.00431	0.240294	-0.06711	0.030374	0.441499	0.840641	0.795549
Q96AG4	Leucine-ric	LRRC59 PR(	-0.07464	0.23017	0.396397	0.572362	0.449936	0.330645	0.290901	0.25634	-0.10257	0.344759
Q96AQ6	Pre-B-cell l(	PBXIP1 HPI	-0.82094	-0.63064	-0.67165	-0.51455	-0.68092	-0.42855	-0.88819	-0.7859	-1.22727	-0.94204
Q96AT1	Uncharacte	KIAA1143	-3.10374	-2.37622	-2.28929	-2.70136	-1.82941	-1.54692	-1.28847	-1.05004	-2.05279	-1.657
Q96AT9	Ribulose-pl	RPE HUSSY	-0.29101	0.097483	0.10649	-0.10872	0.195731	0.306408	0.044565	0.115487	0.598123	0.628322
Q96AX1	Vacuolar pı	VPS33A	-0.92411	-0.87268	-1.06178	-0.60823	-0.98889	-0.88385	-0.73774	-0.98685	-0.68379	-0.92542
Q96AZ6	Interferon-	ISG20 HEM	0.513538	0.584178	0.379252	0.490002	0.150677	0.500744	0.429553	0.606105	0.591368	0.565758
Q96B23	Uncharacte	C18orf25 A	0.064057	0.029653	-0.3761	-0.51979	-0.34182	-0.40612	-0.67715	-0.56734	0.072977	-0.08066
Q96B26	Exosome c(	EXOSC8 OII	-0.09768	-0.02667	-0.03573	0.127826	-0.19322	-0.19904	-0.07141	-0.44674	0.257482	0.392605
Q96B36	Proline-rich	AKT1S1 PR/	-0.07784	-0.15788	-0.25883	-0.35204	-0.42593	-0.16271	-0.48579	-0.28516	-0.04363	0.091781
Q96B45	BLOC-1-rel	BORCS7 C1	-2.94363	-2.55483	-2.24361	-3.22034	-2.42999	-3.1375	-1.85415	-4.27684	-2.78907	-2.10985
Q96B49	Mitochond	TOMM6 OI	-1.35566	-1.5656	-1.73697	-1.36976	-3.2076	-1.28951	-1.24473	-1.97728	-3.08746	-2.11991
Q96B97	SH3 domai	SH3KBP1 C	0.423707	0.510997	0.160184	0.136308	0.18697	0.360747	0.105952	0.233062	0.399567	0.377996
Q96BH1	E3 ubiquiti	RNF25	-1.08746	-1.18265	-1.31221	-1.45317	-1.24236	-1.35789	-1.11718	-1.27684	-1.14886	-0.83303
Q96BJ3	Axin intera	AIDA C1orf	-0.85342	-1.31043	-0.92028	-0.72001	-0.8201	-0.92663	-1.3322	-1.21953	-0.98963	-0.88158
Q96BK5	PIN2/TERF:	PINX1 LPTL	0.304855	0.393431	0.151613	-0.24558	0.383081	0.268489	0.278301	-0.10076	0.100164	0.217326
Q96BM9	ADP-ribosy	ARL8A ARL	1.312197	1.148099	1.367975	1.120294	0.813715	0.762961	0.888355	0.53842	1.030325	0.698396
Q96BN8	Ubiquitin tl	OTULIN FAI	-0.37405	-0.59434	0.110283	-0.39883	-0.31192	-0.12999	-0.77761	-1.77591	-0.58691	-0.04977
Q96BP3	Peptidylpr(	PPWD1 KIA	0.207802	0.030437	0.36321	0.568764	0.10046	0.5035	0.468329	0.327886	0.328142	0.651591
Q96BW5	Phosphotri	PTER	1.993925	1.862009	1.552098	1.835748	1.840956	1.860993	1.786745	1.860348	1.979822	2.004419
Q96BW9	Phosphatid	TAMM41 C	-0.1751	-0.10248	-0.14553	-0.13815	-1.05817	-0.46841	0.162964	-0.1844	-0.15285	-0.24792
Q96BY6	Dedicator (	DOCK10 KI/	-0.46855	-0.30256	-0.52015	-0.52356	-0.30529	0.058254	-0.18536	-0.22909	-0.29739	-0.19391
Q96BY7	Autophagy	ATG2B C14	-3.5025	-3.36146	NA	NA	NA	NA	-3.74723	-3.45372	-3.61353	-3.39453
Q96BZ8	Leukocyte	LENG1	-0.77801	-0.93581	-1.10534	-0.80259	-0.78626	-0.97079	-0.94787	-1.02163	-0.61177	-0.54081
Q96C01	Protein FAI	FAM136A	-0.17927	-0.22293	0.12769	0.048337	-0.23968	-0.22389	-0.11798	0.043243	-0.0339	0.021544
Q96C19	EF-hand do	EFHD2 SWS	0.771716	1.080612	-0.66352	-0.53816	-1.40778	-0.35709	-0.80616	-0.68916	-1.43376	-1.1576
Q96C23	Galactose r	GALM BLO(	0.336875	0.283325	0.305605	0.394999	0.305412	0.262919	0.148448	0.242498	0.231739	0.022569
Q96C86	m7GpppX (	DCPS DCS1	0.208003	0.234176	-0.01399	-0.16551	-0.17521	0.109784	-0.12483	0.058357	-0.17846	-0.17493
Q96C90	Protein pho	PPP1R14B	-3.5025	-2.46529	-3.62449	-3.0906	-3.59991	-2.2115	NA	NA	-3.45003	-1.87299

Q96CN7	Isochorism	ISOC1 CGI-1	-0.60989	-0.43408	-0.8575	-0.54182	-1.12719	-0.82354	-0.65603	-0.67761	-0.82221	-0.45043
Q96CN9	GRIP and co	GCC1	-2.80004	-2.65368	-1.6133	-1.2826	-2.34112	-2.34804	-1.74512	-2.18486	-1.66098	-1.47018
Q96CP2	FLYWCH fa	FLYWCH2	-0.36245	-0.49287	0.043576	-0.43973	-0.45462	-0.18466	-0.39337	0.073421	0.318814	0.356427
Q96CP6	Protein Ast	GRAMD1A	-0.20177	-0.22969	-0.1693	-0.1558	-0.46703	-0.21668	-0.37447	-0.34466	-1.1094	-0.74882
Q96CS2	HAUS augn	HAUS1 CCD	-1.11346	-1.08017	-1.09675	-0.92663	-0.83173	-1.01097	-1.16227	-0.9886	-0.68753	-0.18886
Q96CS3	FAS-associa	FAF2 ETEA	-0.30986	-0.33662	0.193132	-0.0973	-0.45332	-0.36096	0.00315	-0.37737	-0.45003	-0.44786
Q96CT7	Coiled-coil	CCDC124	-0.03063	0.087004	0.690206	0.359081	0.753234	0.606658	0.680628	0.61891	0.99054	0.937178
Q96CV9	Optineurin	OPTN FIP2	-0.38702	-0.24599	0.437285	0.079322	-0.19052	0.032421	-0.03103	0.146841	-0.32075	-0.36573
Q96CW1	AP-2 comp	AP2M1 CLA	0.622092	0.775623	0.541329	0.674392	0.415079	0.394712	0.305953	-0.56968	0.432565	0.477606
Q96CW6	Probable R	SLC7A6OS	-3.03684	-1.94642	-3.31303	-3.33154	-2.74193	-1.89465	-3.91808	-2.80735	-2.94134	-2.87857
Q96CX2	BTB/POZ do	KCTD12 C1	-1.13993	-0.84488	-2.00782	-1.69574	-2.59991	-1.85457	-1.63176	-1.6735	-1.20211	-0.84888
Q96D46	60S riboson	NMD3 CGI-	-0.40296	-0.14768	-0.69218	-0.89129	-0.54463	-0.95693	-0.5175	-1.07039	-0.85651	-0.40621
Q96D71	RalBP1-asso	REPS1	-0.20133	-0.49796	0.007777	-0.00629	0.276604	0.115477	-0.26919	-0.13808	0.073529	0.182653
Q96D96	Voltage-gat	HVCN1 VSC	0.020786	0.245081	-0.41483	-0.55216	0.245986	0.268275	-0.27124	-0.61454	-0.59932	-0.83685
Q96DA6	Mitochond	DNAJC19 T	-0.61869	-0.19599	-0.83094	-0.64582	-0.77973	-0.44448	-0.80863	-0.74846	-0.36864	-0.32082
Q96DB5	Regulator o	RMDN1 FA	1.216318	1.066671	0.70941	0.812721	0.694708	0.41913	0.915731	1.028345	0.88727	0.966421
Q96DF8	Splicing fac	ESS2 DGCR	0.081905	0.202827	0.752908	0.079432	0.183144	-0.1192	-0.08041	-0.0141	0.162017	0.807678
Q96DH6	RNA-bindin	MSI2	0.798669	0.875886	1.007777	1.286367	1.45537	1.095157	1	1.273018	1.46542	1.164999
Q96DI7	U5 small nu	SNRNP40 P	-0.60166	-0.73853	-0.90683	-1.57658	-1.34106	-1.97446	-1.10323	-0.79698	-0.6736	-0.57178
Q96DU3	SLAM famil	SLAMF6 KA	0.751631	0.945411	0.92394	0.842212	0.799171	0.733376	1.044192	1.110531	0.637124	0.475731
Q96E11	Ribosome-r	MRRF	0.317405	0.546319	0.407755	0.382366	0.186023	0.245264	0.183433	0.269491	0.379219	0.39607
Q96E39	RNA bindin	RBMXL1	-0.60116	-0.51829	-0.23061	-0.3943	-0.77036	-0.74735	-0.17913	-0.27637	0.102948	-0.16788
Q96E93	Killer cell le	KLRG1 CLE(	-0.92903	-1.18265	-1.08844	-1.05107	-1.26006	-1.42968	-2.07346	-1.65535	-1.52947	-1.2402
Q96EB1	Elongator c	ELP4 C11or	-2.68536	-0.97679	-0.20046	-0.01894	0.014797	-0.33015	-0.54709	-0.36995	-0.52947	-0.32082
Q96EB6	NAD-deper	SIRT1 SIR2L	-1.23661	-0.96914	-2.09476	-2.00884	-0.91341	-0.93587	-2.2387	-2.5866	-2.49145	-1.57185
Q96EE3	Nucleopori	SEH1L SEC1	-0.03063	0.079643	0.3266	0.329436	0.029444	0.165059	0.060121	0.241889	-0.05772	0.008824
Q96EI5	Transcriptio	TCEAL4 NP	-0.6002	0.112475	-0.0039	0.037153	0.079567	-0.15565	0.098853	-0.46949	0.073529	-0.10555
Q96EK7	Constitutiv	FAM120B (	-1.9184	-1.88928	-1.03745	-1.41575	-0.85294	-1.03324	-1.08951	-1.38976	-1.53889	-1.83385
Q96EK9	Protein KTI	KTI12 SBBI8	-1.06527	-0.94865	-1.7632	-1.2909	-1.20821	-0.81705	-1.34514	-1.22239	-1.02857	-1.1589
Q96EL3	39S riboson	MRPL53	-0.24044	-0.3937	-0.02316	0.188195	0.060376	-0.11764	0.168741	-0.10472	0.181679	-0.15349
Q96EP0	E3 ubiquitin	RNF31 ZIBF	-1.15335	-1.33171	-1.17383	-1.17306	-2.64572	-1.06711	-1.69661	-1.26303	-1.4892	-1.29345
Q96EP5	DAZ-associ	DAZAP1	1.12755	1.307822	1.437285	1.009378	1.45537	1.133025	1.017796	1.058894	1.390071	1.172872
Q96EQ0	Small gluta	SGTB SGT2	0.476021	0.448744	0.880772	0.525645	0.858762	0.738514	0.512598	0.665814	1.170597	0.700247

Q96ER3	Protein SA/	SAAL1	-3.80735	-3.6734	-3.28345	-3.14505	-3.27798	-3.65208	NA	NA	-3.3505	-3.76377
Q96EU6	Ribosomal	RRP36 C6o	-1.49822	-1.44454	-1.24332	-1.16974	-0.93124	-1.24021	-1.84391	-1.3034	-1.23481	-1.15748
Q96EV2	RNA-bindir	RBM33 PRI	-0.82585	-0.58707	-0.47074	-0.42057	-0.16285	-0.32526	-0.38096	-0.36661	-0.37516	-0.42423
Q96EY1	DnaJ homo	DNAJA3 HC	-0.62974	-0.64091	-0.70272	-1.23871	-0.51589	-0.63931	-0.37967	-1.63054	-0.1618	-0.28843
Q96EY4	Translation	TMA16 C4c	-0.2949	0.213452	0.186295	0.922694	0.388772	0.021695	0.186878	-0.20249	-0.01898	0.280628
Q96EY5	Multivesicu	MVB12A CI	-2.98793	-3.44392	-3.6734	-3.67557	-3.01495	-2.06711	-3.68012	-3.65535	-4.67243	-3.1008
Q96EY8	Corrinoid a	MMAB	0.083689	0.212961	-0.28555	-0.09486	0.179134	-0.0927	0.3279	0.479697	0.301961	0.273485
Q96F07	Cytoplasmi	CYFIP2 KIA/	0.215636	0.155892	-0.09435	-0.12244	-0.22805	-0.23526	-0.16928	-0.16782	-0.27373	-0.2761
Q96F15	GTPase IM/	GIMAP5 IA	0.015981	0.187657	-0.39515	-0.64457	-0.2076	-0.17744	0.032631	-0.06081	-0.1913	-0.44384
Q96F63	Coiled-coil	CCDC97	-0.9922	-1.09142	-1.31479	-1.17419	-1.15859	-1.59551	-1.3083	-0.93341	-0.81591	-0.87049
Q96F86	Enhancer o	EDC3 LSM1	-0.90779	-0.83789	-0.23639	0.004703	-0.38104	-0.39316	-0.60114	-0.79861	-0.13627	-0.27638
Q96FH0	BLOC-1-rel	BORCS8 MI	-2.50907	-2.00078	-1.33171	-1.13124	-1.09212	-1.12553	-1.41163	-1.76783	-2.53178	-2.48562
Q96FJ0	AMSH-like	STAMBPL1	-2.98793	-3.6734	-4.07195	-3.20163	-2.47063	-1.89465	-4.43911	-1.91119	-2.14886	-3.70487
Q96FJ2	Dynein ligh	DYNLL2 DL	-0.88359	-0.87317	-1.14768	-1.07731	-1.39046	-0.48215	-1.22069	-1.848	-1.15936	-1.04495
Q96FK6	WD repeat	WDR89 C1	-0.6002	-0.31221	-0.37892	-0.60709	-0.70617	-0.55858	-0.81762	-0.79826	-0.36864	-0.47684
Q96FS4	Signal-indu	SIPA1 SPA1	-1.2949	-1.08017	-0.93147	-0.95067	-1.02793	-0.773	-0.93928	-0.922	-0.9445	-0.33192
Q96FV2	Secernin-2	SCRN2	-0.0183	-0.16069	-1.03953	-0.64332	-0.16528	-0.70454	-0.91905	-1.55728	-0.42142	-0.29398
Q96FV9	THO compl	THOC1 HPI	-1.30986	-1.50901	-1.06378	-1.52356	-1.74193	-1.52083	-1.10613	-1.61973	-1.45003	-1
Q96FW1	Ubiquitin tl	OTUB1 OTE	-0.12234	-0.111	-1.14732	-1.0911	-1.15783	-1.26435	-1.14956	-0.99994	-1.38723	-1.00209
Q96FZ2	Abasic site	HMCES C3c	-1.72489	-2.32193	-2.00782	-1.71621	-1.55552	-2.18641	-2.35819	-2.93289	-1.73383	-1.8893
Q96FZ7	Charged m	CHMP6 VP:	-1.60093	-1.76618	-1.63661	-1.42157	-2.13454	-1.73383	-1.73166	-1.771	-1.90371	-1.28667
Q96G03	Phosphoglu	PGM2 MST	0.994873	1.144576	0.815132	0.731894	1.014797	0.925588	0.782841	0.817136	1.14988	1.106599
Q96G21	U3 small nu	IMP4 BXDC	-3.13993	-2.19153	-3.83094	-3.45317	NA	NA	-2.35819	-4.16993	-3.61353	-1.90578
Q96G28	Cilia- and fl	CFAP36 CC	-3.64689	-2.44392	-2.13906	-1.84549	-4.14048	-2.95693	-3.09516	-4.39232	-3.67243	-2.39453
Q96G46	tRNA-dihyc	DUS3L	-0.88359	-1.64874	0.087004	0.120294	-0.33313	0.155278	-0.46007	0.131245	0.494156	-0.16882
Q96GA3	Protein LT\	LTV1 C6orf	0.422595	0.408198	0.950418	1.223288	0.796692	0.989029	0.504305	0.427862	0.67925	0.582009
Q96GC9	Vacuole me	VMP1 TDC	-1.07464	-0.80346	-1.03154	-0.53261	-2.01495	-1.03317	-0.84493	-1.19592	-1.39941	-1.0726
Q96GD0	Pyridoxal p	PDXP CIN P	-0.18703	-0.15436	-0.09129	-0.1054	-0.04502	0.045848	-0.2453	-0.0649	-0.03147	-0.02911
Q96GF1	E3 ubiquiti	RNF185	-3.43539	-2.20945	-1.66102	-1.65567	-2.69302	-2.26303	-1.28157	-1.60224	-1.9906	-1.97369
Q96GG9	DCN1-like p	DCUN1D1 I	0.333327	0.03522	0.323272	0.361828	0.002602	0.449393	0.049528	0.321012	0.204456	0.207359
Q96GM5	SWI/SNF-re	SMARCD1 I	-0.62463	-0.66215	-0.69416	-0.73183	-0.76852	-0.76345	-0.90575	-0.68263	-0.86474	-0.80254
Q96GQ7	Probable A	DDX27 cPE	-1.20047	-1.48661	-0.65113	-0.50928	-0.97543	-0.69991	-0.71093	-1.28739	-1.24603	-0.52534
Q96GS4	BLOC-1-rel	BORCS6 C1	-1.74866	-1.33344	-2.07195	-2.7234	-1.82217	-1.43459	-2.892	-2.99228	-2.28011	-2.51562

Q96GX9	Methylthio	APIP CGI-2!	-0.71491	-0.48153	-0.29298	-0.31411	-0.53382	-0.46697	-0.61599	-0.54265	-0.20211	-0.1788
Q96GY3	Protein lin-	LIN37 MST	-0.96137	-1.01171	-1.22806	-1.25491	-1.22561	-1.42437	-1.14052	-1.4667	-1.32539	-1.14092
Q96HA1	Nuclear en	POM121 KI	-1.53727	-1.57719	-1.77649	-1.67557	-1.81855	-2.0902	-1.52484	-2.80735	-3.00946	-1.97369
Q96HC4	PDZ and LII	PDLIM5 EN	-0.5155	-0.34928	-0.04213	-0.39384	-0.04713	-0.11964	-0.27753	-0.70931	-0.16549	-0.08561
Q96HE7	ERO1-like p	ERO1A ERC	-0.33258	-0.53703	0.051111	0.189298	-0.20362	-0.15477	-0.03761	0.077686	-0.03025	-0.10318
Q96HI0	Sentrin-spe	SENP5 FKS(	1.229304	1.45446	1.321928	1.073373	1.339666	1.117997	1.122543	1.446886	1.208179	0.801019
Q96HN2	Adenosylh(	AHCYL2 KIA	-1.28011	-1.44392	-0.82402	-1.4361	-1.15697	-1.05571	-1.45305	-1.55102	-0.91754	-0.08194
Q96HS1	Serine/thre	PGAM5	0.047697	0.12683	0.451612	0.758136	0.657475	0.423211	0.696499	0.34915	0.520689	0.749304
Q96HY6	DDRGK dor	DDRGK1 C2	0.510324	0.839306	0.143384	-0.02633	0.45167	-0.06128	0.205802	-0.23229	-0.13444	0.538086
Q96HY7	Probable 2-	DHTKD1 KI.	-1.82873	-1.04757	-1.68589	-3.45317	-1.15697	-3.23704	-1.81762	-3.65535	-1.848	-1.40621
Q96I15	Selenocyste	SCLY SCL	0.523035	0.668291	-0.54966	0.203331	0.161638	0.283497	0.363491	0.115797	0.146692	-0.15624
Q96I24	Far upstrea	FUBP3 FBP	-0.65946	-0.67961	-0.43162	-0.60652	-0.64344	-0.34121	-0.65749	-0.28199	-0.35575	-0.10041
Q96I25	Splicing fac	RBM17 SPF	-0.36344	-0.50348	-0.4021	-0.03814	-0.51354	-0.61288	-0.60039	-0.21573	-0.38087	0.026311
Q96I51	RCC1-like G	RCC1L WBS	-1.18057	-0.81028	-1.11353	-0.95067	-1.39046	-1.18641	-1.09516	-0.59358	-1.46297	-1.21945
Q96I59	Probable as	NARS2	-0.51111	-0.41244	-0.88033	-0.23816	-1.01495	-0.4975	-0.17955	-0.49346	-0.65748	-0.7713
Q96I99	Succinate--	SUCLG2	0.160465	0.11967	0.225768	0.030437	0.213868	0.220001	0.197814	-0.14118	0.369176	0.190243
Q96II8	DISP compl	LRCH3	0.224123	0.072245	-0.34968	-0.25503	-0.27391	-0.41747	-0.25859	-0.42868	-0.55847	-0.24889
Q96IJ6	Mannose-1	GMPPA	-0.47697	-0.03154	-0.46529	-0.5787	-0.19052	-0.38618	-0.53222	-0.52607	-1.01898	-0.97369
Q96IU4	Protein ABI	ABHD14B (	-0.818	-0.55984	-1.00782	-0.64582	-0.7051	-0.68684	-0.65574	-0.86876	-0.57791	-0.60726
Q96IZ7	Serine/Argi	RSRC1 SRRI	-1.5606	-1.58596	-1.26089	-1.42905	-2.39681	-1.35817	-1.44824	-1.89546	-1.01428	-0.71132
Q96J01	THO compl	THOC3	-0.23661	-0.43862	-0.64874	-0.4361	-0.30529	-1.04439	-0.61599	-0.6735	-1.05772	-0.50952
Q96J02	E3 ubiquitii	ITCH	-1.14277	-1.27329	-1.26145	-1.66254	1.099559	1.253297	-1.0426	-1.50423	-1.48195	-1.38695
Q96JB2	Conserved	COG3 SEC3	0.299939	0.193132	0.123255	0.225972	-0.01495	-0.12553	-0.12832	-0.00574	0.04182	-0.21945
Q96JB5	CDK5 regul	CDK5RAP3	0.116409	0.075949	0.203328	0.061401	0.405712	0.22691	0.06502	0.136462	0.273994	0.127005
Q96JG6	Syndetin (C	VPS50 CCD	-1.89482	-1.30256	-0.13272	-0.5146	-1.76702	-3	-0.38848	-1.00018	-0.53885	0.147868
Q96JH7	Deubiquitir	VCPIP1 KIA	-0.48338	-0.49984	-0.45133	-0.4732	-0.59047	-0.78534	-0.59667	-0.69473	-0.77556	-0.83934
Q96JJ3	Engulfment	ELMO2 CEI	-1.61585	-1.15545	-0.83482	-0.90532	-1.22918	-0.73391	-0.53972	-0.67016	-1.42073	-1.29971
Q96JJ7	Protein dis	TMX3 KIAA	1.113611	0.737745	0.529314	0.555389	0.365871	0.498638	0.257053	0.39776	0.164677	0.79593
Q96JM3	Chromosor	CHAMP1 C	0.110807	-0.22475	-0.12263	-0.11475	0.051139	-0.00149	0.025356	0.005714	0.145944	0.045529
Q96JM7	Lethal(3)m	L3MBTL3 K	-1.61864	-1.67988	-1.5856	-1.29296	-1.05225	-1.40113	-1.60666	-1.27819	-2.58026	-2.21739
Q96JP5	E3 ubiquitii	ZFP91 ZNF	-3.03684	-2.2276	-1.70406	-1.64665	-2.13417	-2.22972	-1.67059	-3.07039	-1.78999	-1.41857
Q96JY6	PDZ and LII	PDLIM2 PP	-1.03119	-1.0083	-1.19153	-0.91786	-0.61123	-0.98911	-1.24977	-1.3122	-1.06507	-1.04005
Q96K17	Transcripti	BTF3L4	0.246044	0.122554	-0.2503	-0.26566	0.144571	0.220579	-0.25688	0.087925	0.341562	0.226984

Q96K21	Abscission/	ZFYVE19 AI	-3.20513	-2.30578	-1.97974	-2.99095	-3.55353	-2.78852	-2.13955	-3.1193	-3.62869	-2.54924
Q96K76	Ubiquitin c	USP47	-0.40296	-0.16944	0.301509	-0.15071	-0.07635	0.032421	0.32376	0.094731	0.15624	0.277835
Q96KG9	N-terminal	SCYL1 CVAI	-1.90695	-1.19025	-1.18945	-1.6056	-2.44665	-2.213	-1.37916	-1.85075	-1.8081	-1.73089
Q96KM6	Zinc finger	ZNF512B K	-1.26863	-1.06378	-0.5427	-0.82535	-0.64797	-0.28507	-0.22048	-0.77761	-0.51391	-0.29243
Q96KP4	Cytosolic n	CNDP2 CN2	0.787761	1.108686	0.777361	0.673884	0.832591	0.71674	0.893329	0.730059	0.905919	0.941879
Q96KQ7	Histone-lys	EHMT2 BAT	-0.74393	-0.55761	-0.11661	-0.61898	-0.4188	-0.50141	-0.3763	-0.50014	-0.2605	-0.51734
Q96KR1	Zinc finger	ZFR	0.519102	0.387696	0.588241	0.400084	0.729221	0.513636	0.371025	0.572312	0.600737	0.75293
Q96L73	Histone-lys	NSD1 ARA2	-1.15739	-0.23994	-0.01879	0.190409	-0.24969	-0.9658	-0.41266	-0.37149	-0.28175	-0.59186
Q96L91	E1A-bindin	EP400 CAG	-0.55497	0.09067	0.203328	-0.13124	-0.01495	0.282035	-0.13393	-0.01728	0.117652	0.224919
Q96L92	Sorting nex	SNX27 KIA	0.304855	0.27275	0.445898	0.605721	0.539639	0.236377	0.437877	0.592576	0.246438	0.458627
Q96LJ7	Dehydroge	DHRS1 SDR	-1.82913	-1.72562	-1.57046	-1.47845	-2.27047	-1.32312	-1.62678	-1.79708	-1.4515	-1.31792
Q96LT9	RNA-bindir	RNPC3 KIA	-1.30986	-0.80346	-1.97345	-2.06868	-0.69302	-0.55254	-0.73867	-0.54265	-0.67996	-0.41799
Q96M27	Protein PRI	PRRC1	0.846082	0.879443	0.572719	0.559833	0.823812	0.840499	0.911867	0.990616	0.906016	0.482815
Q96ME7	Zinc finger	ZNF512 KIA	-0.16674	-0.22339	-0.40531	-0.16515	-0.03767	-0.22434	-0.10954	-0.5763	-0.22464	0.056432
Q96MF7	E3 SUMO-p	NSMCE2 C8	-1.76553	-1.21262	-1.12964	-1.09507	-1.69906	-1.93723	-1.56309	-1.01348	-1.46274	-1.16858
Q96MM6	Heat shock	HSPA12B C	-1.98793	-2.36146	-2.19153	-2.67557	-4.01495	-3	-3.09516	-1.848	-1.65748	-2.1788
Q96MU7	YTH domai	YTHDC1 KIA	-0.7978	-1.29255	-0.93734	-0.80883	-0.3654	-0.45374	-0.65834	-0.85678	-0.42524	-0.87508
Q96MW1	Coiled-coil	CCDC43	-0.37912	0.087004	0.101614	0.171318	0.843031	0.386058	0.005107	0.611435	0.658492	0.746669
Q96MX6	WD repeat	WDR92	-0.85042	-1.06378	-1.66102	-1.13124	-0.7051	-1.14958	-1.11718	-0.93289	-0.71823	-1.59384
Q96N66	Lysophospl	MBOAT7 B	-0.58193	-0.73697	-0.84488	-0.92663	-1.27798	-1.03317	-1.42531	-1.16993	-1.30319	-1.21945
Q96N96	Spermatog	SPATA13	-1.45187	-1.37151	-0.77649	-1.10402	-0.9852	-0.87447	-0.90115	-0.88982	-1.38702	-1.55431
Q96NB3	Zinc finger	ZNF830 CC	-1.61704	-1.36097	-0.99214	-0.98435	-0.92251	-0.86576	-1.25453	-1.05356	-0.66159	-0.57233
Q96NC0	Zinc finger	ZMAT2	1.024045	1.213452	1.546769	1.255172	1.482236	1.31755	1.231136	1.091498	1.564386	1.339522
Q96P11	28S rRNA (	NSUN5 NSU	-0.41789	-0.17879	-0.08168	0.050148	-0.37178	-0.05782	-0.29851	-0.13194	0.029556	0.050188
Q96P16	Regulation	RPRD1A P1	-1.02445	-1.16506	-0.77649	-0.92663	-0.8055	-0.60977	-0.97968	-0.75811	-0.74169	-0.71937
Q96P48	Arf-GAP wi	ARAP1 CEN	-1.06193	-0.51457	-2.68726	-2.67779	-1.26006	-1.03317	-1.17377	-1.09464	-2.1478	-1.71358
Q96P70	Importin-9	IPO9 IMP9	-0.5478	-0.55866	-0.44058	-0.4133	-0.86359	-0.32968	-0.662	-0.86854	-1.01034	-0.73824
Q96PE3	Inositol pol	INPP4A	-1.75734	-1.67195	-1.30128	-1.41108	-1.63806	-1.28433	-1.19646	-0.68129	-0.93772	-0.78816
Q96PK6	RNA-bindir	RBM14 SIP	-0.25819	0.083971	0.228652	0.318356	-0.09622	0.130264	0.239827	0.066545	0.266856	0.717361
Q96PN7	Transcripti	TRERF1 BC	-2.53727	-3.57719	-1.49796	-1.9386	-3.27798	-3.40054	-0.81762	-1.1571	-2.00946	-1.87299
Q96PP8	Guanylate-	GBP5 UNQ	-0.35566	-0.25061	0.079643	0.165737	-1.22487	-1.14958	-0.47422	-0.97728	-0.52947	-0.53495
Q96PP9	Guanylate-	GBP4	-1.6916	-1.41561	-1.32274	-1.59215	-2.30663	-1.71379	-1.42957	-1.2257	-1.41674	-1.2661
Q96PU8	Protein qua	QKI HKQ	-0.81369	-0.2674	-0.29642	-0.02818	-0.37293	-0.4258	-0.51931	-0.35325	-0.46053	-0.4615

Q96PV6	Leukocyte	LENG8 KIAA	-0.30236	-0.41244	-0.03953	-0.62632	-0.24236	-0.01097	-0.04679	-0.4459	0.073529	0.118877
Q96PY6	Serine/thre	NEK1 KIAA	-0.9523	-0.58887	0.094327	0.006259	0.879867	0.23173	0.257053	0.432111	0.104556	0.186547
Q96PZ0	Pseudourid	PUS7 KIAA	0.280108	0.189718	0.600476	0.359081	0.306978	0.570316	0.648351	0.914504	0.99054	0.759796
Q96PZ2	Serine prot	FAM111A K	-0.85042	-0.60064	-0.11775	0.061401	-1.07635	-0.40054	-0.68834	-0.922	-0.65748	-0.23499
Q96Q05	Trafficking	TRAPPC9 K	-1.35566	-1.58887	-1.20945	-1.20163	-1.17365	-1.52083	-1.28157	-2.27684	-1.97199	-1.8893
Q96Q11	CCA tRNA n	TRNT1 CGI-	0.51881	0.253253	0.083328	-0.27563	0.354283	-0.10776	-0.29406	-0.53434	-0.44361	0.159066
Q96Q15	Serine/thre	SMG1 ATX	-1.09999	-1.26326	-0.7985	-1.4583	-0.89072	-1.00632	-1.01174	-1.23361	-0.98111	-0.81189
Q96QC0	Serine/thre	PPP1R10 C	-0.9535	-1.00612	-0.71215	-1.66755	-0.99504	-0.90974	-1.51983	-1.19045	-1.21256	-1.30288
Q96QK1	Vacuolar pr	VPS35 MEM	-0.10692	-0.05936	-0.42986	-0.42764	-0.88567	-0.77761	-0.31937	-0.68131	-0.18093	-0.31531
Q96QR8	Transcripti	PURB	-0.14627	-0.55939	-0.20928	-0.31495	-0.09456	-0.33784	-0.19487	-0.02506	-0.14824	-0.16875
Q96QT6	PHD finger	PHF12 KIAA	-3.57289	-3.4021	-1.87317	-3.26053	-2.84503	-2.79647	-1.76451	-2.97728	-1.61353	-2.28264
Q96RD9	Fc receptor	FCRL5 FCRH	0.987825	1.648528	0.70941	0.689785	1.303969	0.912708	0	-0.02308	0.588102	0.471451
Q96RF0	Sorting nex	SNX18 SH3	-1.88398	-2.25133	-1.29051	-1.47328	-1.65974	-3.68727	-1.67252	-1.32354	-2.21565	-2.03656
Q96RK0	Protein cap	CIC KIAA03	-2.85042	-2.20945	-1.20945	-1.67557	-1.15099	-1.04088	-1.60039	-1.16993	-1.20211	-1.95641
Q96RL1	BRCA1-A co	UIMC1 RAP	-1.5025	-0.81381	-0.74527	-1.06413	-1.29613	-1.2115	-1.3322	-1.60224	-0.92647	-0.71937
Q96RL7	Vacuolar pr	VPS13A CH	0.270089	0.338983	0.603045	0.646363	0.744383	0.451461	0.164229	0.584963	-0.24616	0.017594
Q96RN5	Mediator o	MED15 ARC	-1.46855	-1.36146	-1.35147	-1.30633	-1.71727	-1.33015	-1.08427	-1.46949	-0.78978	-0.75627
Q96RP9	Elongation	GFM1 EFG	-1.04906	-1.16593	-1.22245	-0.86089	-0.94845	-0.64203	-0.98017	-0.90368	-1.12158	-1.11512
Q96RQ3	Methylcrot	MCCC1 MC	-1.25096	-0.68589	-0.61849	-0.75803	-0.91341	-1.33015	-0.76451	-0.70116	-0.54314	-0.57394
Q96RQ9	L-amino-ac	IL4I1 FIG1 U	-1.80735	-1.01568	-0.5427	-0.92663	-1.29613	-1.05571	-0.90115	-1.05842	-1.15936	-1.13927
Q96RR1	Twinkle pro	TWNK C10o	-2.68536	-1.43335	NA	NA	-2.95606	-2.72247	-3.05209	-2.76783	-4.3505	-1.80957
Q96RR4	Calcium/ca	CAMKK2 CA	0.576857	0.499286	1.231831	1.085247	1.217071	1.179608	1.338207	1.241889	0.879603	0.692931
Q96RS0	Trimethylg	TGS1 HCA1	-3.35109	-3.34169	-2.44392	-3.38606	-3.21748	-3.1994	-3.3322	-3.80735	-3.3505	-3.06333
Q96RS6	NudC doma	NUDCD1 CI	-2.09443	-1.17299	-1.77417	-0.97163	-1.21789	-0.83041	-1.93698	-2.19592	-0.96962	-0.80438
Q96RT1	Erbin (Dens	ERBIN ERBI	-0.75527	-0.36146	-0.11353	0.061401	0.300954	0.069348	-0.11718	-0.2091	0.1133	0.143125
Q96RU3	Formin-bin	FNBP1 FBP	-0.41908	-0.37151	0.049455	0.093966	0.141995	0.060827	0.000326	-0.00574	0.27092	0.385106
Q96S19	Methyltran	METTL26 JI	-0.77588	-0.16944	0	0.507657	-0.03767	0.027068	-0.31937	-0.42269	-0.1541	0.139112
Q96S44	EKC/KEOPS	TP53RK C20	-0.909	-1.01497	-0.87035	-1.12072	-1.02822	-0.92998	-0.93419	-1.18298	-1.29304	-1.25374
Q96S55	ATPase WR	WRNIP1 W	0.26	0.179425	-0.152	-0.34997	-0.03046	-0.31452	-0.01752	-0.21518	0.063871	0.28418
Q96S66	Chloride ch	CLCC1 KIAA	0.518308	0.817606	0.979862	0.501356	0.706385	0.466876	0.533853	0.546005	0.62304	0.682946
Q96S97	Myeloid-as	MYADM UI	0.856726	0.665835	0.886471	1.249907	1.007417	0.980745	0.904843	2.103538	0.781531	0.73608
Q96S99	Pleckstrin h	PLEKHF1 AI	-0.51111	-0.14768	-0.12624	0.294061	-0.42999	-0.1989	-0.06274	-0.36995	-0.5639	-0.51584
Q96SB3	Neurabin-2	PPP1R9B P	-1.12869	-1.16721	-1.01568	-1.07731	-1.26006	-1.48981	-1.6477	-1.33342	-1.5025	-1.11032

Q96SB8	Structural r	SMC6 SMC	-1.2949	-1.24598	-0.94306	-1.28772	-2.16937	-1.63951	-1.87276	-2.05141	-1.64962	-1.7816
Q96SK2	Transmeml	TMEM209	-1.35811	-1.75109	-1.80065	-1.48764	-2.33212	-2.1775	-1.86608	-1.94781	-1.6597	-1.95132
Q96ST2	Protein IW!	IWS1 IWS1	-0.34023	-0.26929	0.119902	-0.14545	0.123693	-0.08998	-0.02908	0.223718	0.429402	0.275578
Q96ST3	Paired amp	SIN3A	-0.63743	-0.64264	-0.4761	-0.71621	-0.26006	-0.27621	-0.46713	-0.26303	-0.24616	-0.01334
Q96SU4	Oxysterol-k	OSBPL9 OR	1.009064	1.196539	1.358883	1.123177	1.419678	1.247928	1.441649	1.258734	1.277888	1.137101
Q96SW2	Protein cer	CRBN AD-0	0.596467	0.741327	0.936099	0.726585	0.609977	0.973156	0.580972	0.75853	0.963744	1.035677
Q96SZ5	2-aminoeth	ADO C10or	-1.55673	-2.127	-0.933	-1.28343	-1.39628	-1.44702	-0.93658	-1.39561	-1.00703	-0.82884
Q96T23	Remodeling	RSF1 HBXA	-1.70499	-1.574	-1.77947	-1.9034	-1.21997	-1.66977	-1.35532	-1.57643	-1.53456	-1.47631
Q96T37	RNA-bindir	RBM15 OT	-1.12935	-1.01795	-0.76577	-1.03426	-0.60859	-0.71309	-0.95468	-0.45372	-0.68207	-0.67155
Q96T51	RUN and FY	RUFY1 RAB	-0.93678	-0.99222	-1.34658	-0.95691	-1.02063	-1.45943	-1.4144	-1.18286	-1.71673	-0.84003
Q96T58	Msx2-inter	SPEN KIAA(	-0.818	-1.14768	-0.81028	-0.814	-1.16149	-0.91511	-1.04152	-0.81741	-1.02857	-0.62081
Q96T60	Bifunctiona	PNKP	-0.69844	-0.99515	-0.50901	-0.81207	-0.47502	-0.60374	-1.63176	-0.6475	-1.13046	-1.30944
Q96T76	MMS19 nu	MMS19 MI	0.876672	0.641046	-0.65676	-1.12046	0.161638	0.386058	0.628633	0.241889	-1.62796	-1.55931
Q96TA1	Protein Nib	NIBAN2 C9	-1.54525	-1.29048	-2.06057	-1.93208	-0.99184	-0.88223	-1.16391	-1.67477	-2.08509	-1.96458
Q96TC7	Regulator c	RMDN3 FA	0.560509	0.465798	0.210086	0.198904	0.306978	0.245625	0.376148	0.436348	-0.00946	-0.11991
Q99417	c-Myc-bind	MYCBP AM	0.024489	0.300015	0.092318	0.179966	0.110889	0.103454	0.379351	0.162652	0.251239	0.181298
Q99418	Cytohesin-2	CYTH2 ARN	-1.20832	-1.10512	-1.4761	-1.86821	-1.55552	-1.55254	-1.25691	-0.96605	-1.4245	-1.01781
Q99426	Tubulin-fol	TBCB CG22	-0.83954	-1.4021	-0.29152	-0.23281	-0.32597	0.024262	-0.56212	-0.60135	-0.56662	-0.27638
Q99436	Proteasom	PSMB7 Z	0.381307	0.445898	-0.20945	-0.36167	0.213868	0.427281	0.67423	0.7022	0.032631	-0.41799
Q99439	Calponin-2	CNN2	0.006049	0.363436	-0.05397	-0.41923	-0.31451	-0.56659	-0.08901	-0.17655	-0.31487	-0.1589
Q99442	Translocati	SEC62 TLO(	1.845392	1.934211	2.03594	2.276975	1.249355	1.792657	1.759627	1.158376	1.0926	1.316411
Q99447	Ethanolam	PCYT2	-0.25096	-0.12052	-0.69499	-0.8407	-0.77658	-0.49987	-0.48433	-0.26333	0.033786	-0.14836
Q99459	Cell divisior	CDC5L KIAA	-0.32496	-0.19599	-0.25992	-0.23078	-0.00746	-0.56058	-0.06274	-0.36257	-0.16463	0.047877
Q99460	26S protea	PSMD1	0.343635	0.339836	0.382188	0.393731	0.470053	0.368544	0.315856	0.29307	0.45265	0.479622
Q99471	Prefoldin sı	PFDN5 MM	-0.05683	-0.30412	-0.15702	-0.44122	-0.37913	-0.43673	-0.61656	-0.31705	-0.05636	-0.43215
Q99490	Arf-GAP wi	AGAP2 CEN	-0.67565	-0.64264	-0.65849	-0.95532	-0.85845	-1.1375	-0.87276	-1.2091	-1.39941	-0.93934
Q99496	E3 ubiquitir	RNF2 BAP1	0.45391	0.695031	0.482639	0.171318	0.783791	0.662965	0.515085	0.770913	0.751041	0.573113
Q99497	Parkinson c	PARK7	0.533997	0.401588	0.632176	0.270889	0.352404	0.322802	0.517218	0.588367	0.607001	0.595168
Q99536	Synaptic ve	VAT1	-0.75527	-0.40726	0.046047	0.198904	0.107446	0.120513	-0.16227	-0.36995	0.168877	0.75718
Q99543	DnaJ homo	DNAJC2 MI	0.953805	0.92805	0.384498	0.344335	0.875821	-0.02203	0.638526	0.078003	0.312468	0.461843
Q99547	M-phase pl	MPHOSPH(	-0.21068	-0.16908	-0.24732	-0.74755	-0.23012	-0.39724	-0.50128	-0.45427	0.203345	-0.08211
Q99549	M-phase pl	MPHOSPH8	-1.55497	-1.80346	-1.73697	-1.48794	-0.98258	-1.33705	-1.53964	-0.99289	-1.37474	-1.11749
Q99567	Nuclear po	NUP88	-0.3081	-0.2276	-0.07131	0.027108	-0.02204	0.04581	0.044084	-0.23119	-0.02139	0.01617

Q99570	Phosphoin	PIK3R4 VPS	1.650147	1.851243	1.804009	2.017147	1.648015	1.902512	1.756781	1.935609	1.577083	1.552139
Q99575	Ribonuclea	POP1 KIAA	-0.68536	-0.18709	-0.43335	0.299168	-0.19903	-0.37197	-0.42531	-0.58496	-0.53629	-0.10555
Q99576	TSC22 dom	TSC22D3 D	-1.91754	-1.91667	-1.83906	-2.25865	-1.64459	-2.14415	-1.66245	-1.82283	-1.7709	-1.22003
Q99590	Protein SC/	SCAF11 CA:	-0.81852	-0.51814	-0.64032	-0.70725	-0.56754	-0.67001	-0.83132	-0.68124	-0.61847	-0.58761
Q99598	Translin-as:	TSNAX TRA	-0.19836	-0.15899	-1.02172	-0.41089	-0.54565	-0.19558	-0.10075	-0.01373	-0.1544	-0.56044
Q99613	Eukaryotic	EIF3C EIF3S	-0.45978	-0.21517	-0.09891	-0.13205	-0.48727	-0.37197	-0.41163	-0.07039	-0.02873	-0.16883
Q99614	Tetratricop	TTC1 TPR1	0.229304	0.457303	0.422815	0.220599	0.051139	0.169925	0.025356	0.222392	0.510796	0.355175
Q99615	DnaJ homo	DNAJC7 TP	-0.8525	-0.55093	-1.5656	-1.48794	-0.53271	-1.1989	-1.51019	-1.1193	-1.4245	-1.2507
Q99622	Protein C1(	C12orf57 C	-1.62803	-1.97679	-0.6672	-1.21613	-1.53382	-1.07861	-1.69661	-1.69188	-0.81444	-0.84093
Q99623	Prohibitin-:	PHB2 BAP I	0.299595	0.764652	0.54276	0.439912	0.47155	0.656046	0.585489	0.568306	0.517558	0.472082
Q99627	COP9 signa	COPS8 CSN	-0.73816	-0.05866	0.075588	-0.69481	-0.61163	-0.18206	-0.00437	-0.18187	-0.24997	-0.03098
Q99638	Cell cycle c	RAD9A	-3.75212	-2.42286	-3.04813	-3.18453	-2.99885	-2.65208	-2.48254	-2.84859	-2.99463	-2.87454
Q99700	Ataxin-2 (S	ATXN2 ATX	-0.02738	0.039173	0.506661	0.346548	0.211656	0.271677	0.213535	0.365922	0.420159	0.518256
Q99704	Docking pr	DOK1	-3.65493	-3.6777	-2.63417	-3.33431	-2.55552	-4.04439	-3.96963	-3.88982	-3.67243	-4.0268
Q99714	3-hydroxya	HSD17B10	0.414517	0.374994	0.276712	0.485427	0.298954	0.624491	0.371409	0.324244	0.437461	0.342173
Q99729	Heterogen	HNRNPAB /	0.465491	0.754887	0.538479	0.54333	0.685489	0.153875	0.837683	0.71619	0.4824	1.055028
Q99733	Nucleosom	NAP1L4 NA	-1.17204	-1.42969	-0.44857	-0.46612	-0.4634	-0.44919	-0.2799	-0.53415	-0.0803	0.233504
Q99747	Gamma-so	NAPG SNAI	-1.02549	-0.78498	0.331249	0.407327	0.547737	0.332554	0.19299	0.118345	0.302234	0.295387
Q99757	Thioredoxi	TXN2 TRX2	-0.05563	0.092439	-0.24703	-0.2339	-0.25694	-0.73063	-0.46401	-0.39267	0.166563	0.146501
Q99759	Mitogen-ac	MAP3K3 M	-0.45666	-0.3174	-0.17246	0.062902	-0.36892	-0.46403	-0.47088	-1.00374	-0.29716	-0.32321
Q99766	ATP syntha	DMAC2L AT	1.099536	1.01744	1.015513	1.171318	0.822152	0.895185	0.843442	0.743819	1.306762	1.163024
Q99767	Amyloid-be	APBA2 MIN	-2.32889	-2.63154	-2.47614	-2.59706	-2.12719	-1.60604	-1.73587	-2.06959	-1.38908	-1.50133
Q99795	Cell surface	GPA33	-1.02628	-0.93609	-0.66471	-0.53365	-0.75565	-0.76162	-0.41765	-0.90168	-0.88632	-0.64686
Q99798	Aconitate h	ACO2	-0.76327	-0.58909	-0.24471	-0.40858	-0.26962	-0.04995	-0.50362	-0.37545	0.097284	-0.24932
Q99807	5-demetho	COQ7	-0.03063	-0.04757	-1.03154	-1.11757	-0.92749	-1.22422	-1.31937	-1.26303	-0.76553	-0.43584
Q99816	Tumor susc	TSG101	1.634613	1.668291	1.810693	1.397684	1.722015	1.514027	1.699652	1.419325	1.467126	1.448933
Q99828	Calcium an	CIB1 CIB KI	-1.1751	-1.06242	-1.09288	-0.82057	-0.85068	-0.72665	-1.10811	-0.97412	-0.8495	-0.93645
Q99829	Copine-1 ((	CPNE1 CPN	-0.31739	-0.50901	-0.90933	-0.81051	-0.46127	-0.25828	-0.03179	-0.05625	-0.3238	-0.4624
Q99832	T-complex	CCT7 CCTH	1.010986	0.81693	0.546257	0.673006	0.524755	0.687058	0.622308	0.514603	0.868929	0.840199
Q99836	Myeloid di	MYD88	-0.06849	0.13193	-0.07757	-0.38745	-0.48769	-0.46571	-0.09993	-0.35937	-0.45855	-0.46682
Q99848	Probable rI	EBNA1BP2	0.121991	-0.28345	0.49712	0.097024	0.416895	0.165059	0.39183	0.273468	0.415037	0.432632
Q99873	Protein arg	PRMT1 HM	1.640458	1.738914	1.612002	1.803766	1.616833	1.743508	1.746145	1.719688	1.88727	1.75718
Q99933	BAG family	BAG1 HAP	-1.68497	-1.59604	-1.59591	-1.91475	-1.79764	-1.91511	-1.97859	-1.85793	-1.81169	-1.52725

Q99952	Tyrosine-pr	PTPN18 BD	-0.54416	-0.26417	0.090735	0.037813	-0.3359	-0.42633	0.004124	-0.02095	0.071362	0.011841
Q99961	Endophilin-	SH3GL1 CN	-0.64049	-0.8189	-0.6112	-0.50161	-0.48243	-1.3224	-0.50781	-0.37223	-0.46432	-0.82517
Q99986	Serine/thre	VRK1	0.26	0.396397	0.165586	-0.20887	0.022139	0.217701	-0.01543	0.182591	0.050951	0.243729
Q99996	A-kinase ar	AKAP9 AKA	-0.71491	-0.36647	-0.39696	-0.39428	-0.56649	-0.27621	-0.42531	-0.25222	-0.4245	-0.21945
Q9BPW8	Protein Nip	NIPSNAP1	-0.15963	-0.27813	-0.59432	-0.04635	-0.12216	0.234727	-0.21804	0.294138	-0.10265	-0.35164
Q9BPX5	Actin-relate	ARPC5L	0.408963	0.697437	0.114636	0.108706	0.096414	0.023561	-0.12832	0.335603	-0.10257	-0.05437
Q9BPZ3	Polyadenyl	PAIP2 PAIP	-2.80735	-2.48699	NA	NA	-3.07635	-2.75899	NA	NA	-3.79796	-3.0268
Q9BQ04	RNA-bindir	RBM4B RBI	-2.68536	-2.13906	-2.36146	-1.59756	-2.84503	-2.87447	-1.782	-1.6735	-1.7028	-1.52855
Q9BQ39	ATP-depen	DDX50	-0.6002	-0.27872	-0.69849	0.067399	-0.24236	-0.52869	-0.85415	-0.78746	-0.25739	-0.78649
Q9BQ52	Zinc phospl	ELAC2 HPC	0.097236	0.18649	0.534708	0.349267	0.575013	0.23173	0.292554	0.146841	0.210218	0.536968
Q9BQ61	Telomerase	TRIR C19or	0.027314	-0.27872	0.130397	0.171318	0.312976	-0.03317	-0.33865	-0.12553	0.360998	0.269657
Q9BQ67	Glutamate-	GRWD1 GR	-0.67535	-0.80126	-0.68961	-0.73791	-0.61266	-0.45395	-0.94018	-1.22066	-0.49874	-0.34731
Q9BQ69	ADP-ribose	MACROD1	-2.98793	-1.90202	-1.33171	-1.86821	-2.59991	-1.68684	-1.41163	-1.43812	-1.67243	-2.1589
Q9BQA1	Methylosoi	WDR77 ME	-0.87245	-0.73048	-1.24598	-0.95067	-0.71727	-0.9783	-1.01027	-0.6735	-1.33852	-0.71937
Q9BQB6	Vitamin K e	VKORC1 VK	-1.80735	-2.26459	-3.6734	-4.84549	-3.51245	-3.0902	-3.28157	-4.39232	-3.21299	-3.1008
Q9BQC6	Ribosomal	MRPL57 M	1.009064	0.890683	1.27919	1.247267	1.124601	1.019001	1.171061	1.315042	1.004707	1.180702
Q9BQE3	Tubulin alp	TUBA1C TU	0.40439	0.345135	0.448758	-0.11757	0	0.110424	0.02032	0.141661	-0.37474	-0.43584
Q9BQE5	Apolipopro	APOL2	-0.94062	-1.01568	-0.8519	-1.02531	-0.84503	-0.26303	-1.09516	-1.27684	-1.61353	-1.73402
Q9BQG0	Myb-bindir	MYBBP1A I	-1.62986	-1.79232	-1.24598	-1.8011	-0.39046	-1.26303	-1.85462	-1.58312	-1.4245	-1.41799
Q9BQS8	FYVE and c	FYCO1 ZFY\	-0.87174	-1.11128	-0.06886	-0.30459	-0.39542	-0.18523	-0.30356	-0.19354	-0.15301	-0.23693
Q9BRA2	Thioredoxi	TXNDC17 T	-1.45187	-0.83789	-0.5427	-0.72655	-0.50188	-0.25162	-0.68834	-0.62855	-0.68753	-0.56083
Q9BRD0	BUD13 hor	BUD13	-0.79859	-0.88065	-0.84473	-0.68685	-0.18213	-0.58324	-0.74494	-0.50351	-0.62674	-0.53209
Q9BRJ6	Uncharacte	C7orf50 FP	-0.05561	0.263034	-0.42286	-0.61667	-0.51245	-0.86449	-0.26919	-0.922	-0.66493	-0.3375
Q9BRL6	Serine/argi	SRSF8 SFRS	-0.17372	-0.50901	0.23017	0.193429	-0.19903	0.005454	-0.378	-0.26992	0.073529	0.098355
Q9BRP8	Partner of \	PYM1 PYM	0.675038	0.58756	0.737745	0.801968	0.753234	0.969626	0.352402	0.53842	0.808701	0.986662
Q9BRQ0	Pygopus hc	PYGO2 PP7	-1.34433	-1.12751	-1.15813	-1.08695	-0.78156	-1.03019	-1.49738	-1.02338	-0.88559	-0.65297
Q9BRQ6	MICOS com	CHCHD6 CH	-0.23661	-0.45457	0.12683	-0.16601	-0.35199	-0.26303	-0.26303	-0.59358	-0.19669	0.056413
Q9BRR6	ADP-depen	ADPGK PSE	-2.81804	-3.63906	-2.08844	-2.0126	-2.74193	-2	-3.33867	-2.78327	-3.30998	-3.88632
Q9BRR9	Rho GTPas	ARHGAP9	-0.51978	-0.5541	-0.41244	-0.37588	-0.55552	-0.54455	-0.32577	-0.34066	-0.74169	-0.29047
Q9BRS2	Serine/thre	RIOK1 RIO1	-0.65641	-0.50901	0.015513	0.108706	-0.16528	0.090032	-0.87276	-0.87925	0.270089	0.31305
Q9BRT2	Ubiquinol-c	UQCC2 C6c	-1.56157	-1.62345	-1.42975	-1.42934	-1.73609	-1.49001	-1.13698	-1.27825	-1.21837	-1.11228
Q9BRT3	Migration a	MIEN1 C17	-2.68536	-3.24598	-2.69849	-2.89129	-1.57755	-3.58496	-3.13955	-4.07039	-2.28011	-2.13927
Q9BRT6	Protein LLP	LLPH C12or	1.693897	1.654733	1.910525	1.909397	1.925608	2.027068	1.776328	1.946048	2.142424	2.150121

Q9BRU9	rRNA-proce	UTP23 C8o	-0.65641	-1.5427	-1.02359	-0.51457	-0.87199	-0.89465	-0.89162	-0.922	-1.1913	-0.70487
Q9BRV8	Suppressor	SIKE1 SIKE	-1.03958	-1.05041	-0.90236	-0.7505	-1.04036	-1.55817	-0.72667	-0.43148	-0.8382	-0.8044
Q9BRZ2	E3 ubiquiti	TRIM56 RN	-0.21534	-0.29069	-0.49665	-0.31169	-0.56649	-1.23704	-0.39613	-0.57696	-0.54678	-0.44075
Q9BS26	Endoplasm	ERP44 KIAA	0.293381	0.378721	-0.43055	-0.03366	-0.29804	-0.76706	0.051763	-0.24302	-0.50788	-0.36822
Q9BSB4	Autophagy	ATG101 C1	-0.5285	-0.59474	-0.41244	-0.2909	-0.32379	-0.57679	-0.53222	-0.83773	-1.04793	-0.54783
Q9BSC4	Nucleolar p	NOL10	-1.72156	-1.54597	-2.22081	-2.43063	-1.93222	-2.31314	-0.47422	-0.69188	-2.02282	-1.95733
Q9BSD7	Cancer-rela	NTPCR C1o	0.443257	0.628713	0.751707	0.7507	-0.10007	0.194011	-0.50293	-0.68266	0.323813	0.232472
Q9BSE5	Agmatinase	AGMAT	0.110807	0.151613	-0.15634	-0.15899	-0.68105	-0.1375	-1.10613	-1.18286	0.625255	0.239986
Q9BSF4	Mitochond	TIMM29 c1	0.723568	0.625965	0.291986	0.268252	0.411314	0.184425	0.661349	0.061639	-0.10257	0.435907
Q9BSH5	Haloacid de	HDHD3 C9o	0.329184	-0.10093	-0.33662	-0.38606	-0.89947	-0.2115	-0.11718	-0.33342	-0.64268	-0.2143
Q9BSJ8	Extended s	ESYT1 FAM	0.52308	0.552937	0.742535	0.376359	0.621672	0.438385	0.533486	0.338081	-0.11764	-0.07843
Q9BSL1	Ubiquitin-a	UBAC1 GBD	-1.5675	-1.80449	-1.561	-1.29057	-1.75798	-1.46897	-1.34607	-2.24603	-1.69694	-1.36022
Q9BSQ5	Cerebral ca	CCM2 C7or	-0.53727	-0.68589	-0.26459	-0.18015	-0.62263	-0.31647	-0.70492	-0.35523	-0.50919	-0.56737
Q9BSW2	EF-hand ca	CRACR2A E	-0.82873	-0.65486	-0.64264	-0.28325	-0.1161	-1.03317	-1.08427	-0.69188	-0.83954	-0.69768
Q9BT09	Protein car	CNPY3 CTG	0.108696	0.034724	0.352533	0.283447	0.368521	0.178829	0.181524	0.121973	0.221118	0.371012
Q9BT22	Chitobiosyl	ALG1 HMA	1.289938	1.405643	0.809317	0.753205	0.405504	0.5979	1.147298	0.548282	-0.2618	0.063229
Q9BT23	LIM domai	LIMD2 SB1	-0.04184	0.400615	-0.38686	-0.2662	-0.36674	-0.32516	-0.13639	-0.04273	-0.32501	-0.16571
Q9BT40	Inositol pol	INPP5K PPS	1.807355	1.91983	0.665835	0.467393	0.508612	0.467359	0.808816	0.270648	0.273994	0.090064
Q9BT78	COP9 signa	COPS4 CSN	-0.11405	0.291986	0.210086	0.304257	0.3249	0.41913	0.085434	0.284815	0.285644	0.150071
Q9BTA9	WW domai	WAC KIAA1	0.73928	1.078101	1.383867	1.476447	1.312284	1.350468	1.287464	1.20813	1.472215	1.446788
Q9BTC0	Death-indu	DIDO1 C20	-1.41908	-1.41244	-1.19285	-1.20561	-1.30556	-1	-1.30196	-1.39357	-0.93546	-1.00888
Q9BTD8	RNA-bindir	RBM42	-1.25808	-0.99178	-1.65755	-1.66921	-1.4364	-1.60701	-1.39165	-1.8649	-1.3816	-0.92413
Q9BTE1	Dynactin su	DCTN5	1.121636	1.236924	1.258271	0.927761	1.300468	1.359107	0.999265	1.294817	1.501256	1.319422
Q9BTE3	Mini-chrom	MCMBP C1	-0.67076	-0.58656	-0.58301	-0.71621	0.006927	-0.15135	-0.9898	-1.0115	-0.9445	-1.11991
Q9BTE6	Alanyl-tRN	AARSD1	-1.13586	-1.21483	-0.49231	-0.71393	-1.01472	-1.27409	-1.23769	-1.20311	-0.80596	-0.96464
Q9BTT0	Acidic leuci	ANP32E	-0.71505	-0.74226	-0.11982	-0.29037	-0.39257	-1.07286	-0.99598	-1.56619	-0.73378	-0.14786
Q9BTV4	Transmeml	TMEM43 U	0.334001	0.711793	-0.72403	-0.24558	-0.55552	-0.39334	-0.63971	-0.61973	-0.38702	-0.47221
Q9BTW9	Tubulin-spe	TBCD KIAA	-1.03064	-0.42206	-0.49339	-0.79911	-0.80269	-0.61613	-0.67391	-0.82251	-0.66872	-0.90987
Q9BTY7	Protein HG	HGH1 C8or	0.131806	-0.03386	-0.87554	-0.28077	0.017945	0.027955	0.010187	0.01573	-0.24097	-0.03488
Q9BTZ2	Dehydroge	DHRS4 SDR	1.355481	1.053581	1.360402	1.356634	1.449936	1.092597	1.338207	1.159711	1.069042	1.209693
Q9BU02	Thiamine-t	THTPA	-0.74507	-0.93147	-0.84488	-0.40255	-0.7051	-0.67807	-0.97968	-1.39232	-0.43721	-0.54137
Q9BU23	Lipase mat	LMF2 TMEI	0.734612	0.489405	0.835724	0.747572	0.543402	0.714294	0.683876	0.722259	0.411401	0.243555
Q9BU76	Multiple m	MMTAG2 C	-1.64689	-1.48699	-1.35147	-1.65567	-1.53382	-1.47454	-1.42531	-1.45372	-1.20211	-1.03585

Q9BUE0	Mediator o	MED18	-1.94062	-1.33171	-1.53138	-0.66558	-1.19052	-1.2115	-1.68012	-1.07039	-0.97199	-1.70487
Q9BUE6	Iron-sulfur	ISCA1 HBLD	-1.60942	-1.18265	-0.94642	-0.7794	-0.92749	-0.85457	-0.58496	-0.9886	-0.74959	-0.86491
Q9BUH6	Protein PA)	PAXX C9orf	-0.52749	0.037994	-1.07244	-1.42905	-1.13427	-1.4386	-1.06818	-1.24827	-1.19922	-1.01417
Q9BUJ2	Heterogen	HNRNPUL1	0.711948	0.798076	0.744604	0.575262	0.695204	0.656517	0.593277	0.55961	0.696829	0.689729
Q9BUK0	Coiled-coil-	CHCHD7	-0.82873	-1.2185	-1.16506	-1.45317	-0.76702	-0.84472	-1.09516	-0.90046	-0.78978	-0.80183
Q9BUL8	Programm	PDCD10 CC	-0.35497	-0.13776	-0.7287	-0.57968	-1.06075	-0.80602	-0.33351	-0.38295	-0.74577	-0.48042
Q9BUL9	Ribonuclea	RPP25	-0.45867	-0.39699	-0.91701	-0.79221	-0.67228	-0.80767	-1.08179	-0.81871	-0.62051	-0.48125
Q9BUP3	Oxidoredu	HTATIP2 CC	-0.38702	-0.33171	-0.4021	0.073373	-0.71727	-0.43706	-0.27537	-0.6735	-0.38702	-0.51584
Q9BUQ8	Probable A	DDX23	0.185849	0.342063	0.537237	0.287758	0.610001	0.439422	0.536173	0.518575	0.431481	0.664746
Q9BUR4	Telomeras	WRAP53 TC	-2.60942	-1.6734	-0.99222	-0.73697	-1.71727	-1.47454	-1.69661	-2.07039	-0.52268	-1.31531
Q9BUR5	MICOS con	APOO FAM	-1.666	-1.84488	-1.44392	-1.40255	-1.49139	-1.81558	-1.782	-1.71049	-1.76553	-1.32636
Q9BUT1	3-hydroxyb	BDH2 DHRS	-0.90613	-1.08017	0.087004	-0.18015	0.330824	0.406817	-0.11718	0.51056	0.078003	-0.52218
Q9BV20	Methylthio	MRI1 MRD	-0.60942	-0.75659	-1.29834	-0.85681	-0.71727	-0.67807	-0.62385	-1.04654	-0.38087	-0.35437
Q9BV38	WD repeat	WDR18	0.756154	0.891624	0.293959	0.196031	0.397927	0.456074	0.529551	0.282446	0.970468	1.090645
Q9BV40	Vesicle-ass	VAMP8	1.102277	0.980913	1.022947	0.86467	1.208312	1.135554	0.920378	1.013964	0.977337	0.745136
Q9BV44	THUMP do	THUMPD3	-1.87245	-1.35147	-0.53703	-0.91475	-0.63413	-0.68684	-0.66382	-0.73888	-0.7028	-0.69052
Q9BV57	1,2-dihydro	ADI1 MTCB	1.006049	1.10343	0.298341	0.425973	0.570012	1.005454	0.543443	0.406424	1.204092	1.081724
Q9BV68	E3 ubiquiti	RNF126	-0.69515	-0.7632	-1.34156	-1.03814	-1.26006	-1.75899	-0.92038	-1.39232	-1.1913	-0.90578
Q9BV73	Centrosom	CEP250 CEI	-1.44282	-1.22645	-1.2276	-1.1087	-0.32796	-0.83798	-1.25144	-1.02609	-1.27505	-1.14014
Q9BV79	Enoyl-[acyl	MECR NBR	-2.80735	-1.91667	-1.03953	-1.73697	-2.69302	-1.03317	-1.23266	-1.56789	-1.07748	-1.29345
Q9BVC4	Target of ra	MLST8 GBL	-3.00643	-2.71373	-2.59518	-2.44925	-2.56486	-2.48237	-2.83873	-2.56036	-1.41798	-1.14297
Q9BVC5	Ashwin	C2orf49	1.211091	1.468619	1.20502	1.193429	0.822152	0.977974	1.027867	1.069844	1.014075	1.194304
Q9BVC6	Transmeml	TMEM109	-0.69515	-0.60064	-1.44489	-1.12214	-2.37276	-1.4783	-1.7997	-3.07039	-3.39941	-2.00888
Q9BVG4	Protein PBI	PBDC1 CXo	-0.65225	-0.41635	-1.18682	-0.97328	-1.60646	-1.03356	-0.99023	-0.8444	-1.13595	-0.73771
Q9BVI4	Nucleolar c	NOC4L	0.886542	0.978394	0.584963	0.64235	0.775127	0.944291	1.015267	1.058894	0.79518	1.203941
Q9BVJ6	U3 small nu	UTP14A SD	-0.06795	-0.00717	0.017223	-0.22047	0.128874	0.117661	-0.03995	0.1349	0.321009	0.174876
Q9BVK6	Transmeml	TMED9 GP2	-0.9523	-1.09675	-1.2185	-0.4361	-1.37109	-0.76827	-1.42439	-0.84822	-1.83112	-1.44184
Q9BVL2	Nucleopori	NUP58 KIA	0.396898	0.441167	0.881228	0.898115	0.992089	0.800185	0.612256	0.728153	0.684055	0.798766
Q9BVL4	Protein ade	SELENOO S	-1.03288	-1.2305	-1.35584	-2.50049	-1.63251	-2.10231	-1.35261	-1.15041	-2.45766	-1.64856
Q9BVM4	Gamma-glu	GGACT A2L	-2.66613	-2.89216	-2.29669	-2.3274	-1.56687	-2.411	-2.12941	-1.59796	-2.86303	-3.3279
Q9BVP2	Guanine nu	GNL3 E2IG3	-0.59208	-0.41037	-0.2382	-0.40843	-0.33179	-0.48659	-0.71368	-0.46212	-0.43721	-0.36809
Q9BVS4	Serine/thre	RIOK2 RIO2	-0.78629	-1.14768	-0.92405	-1.24558	-0.68105	-0.77761	-1.18536	-1.63743	-0.848	-1.21945
Q9BVS5	tRNA (ader	TRMT61B	-3.08746	-1.83094	-0.52015	-0.56009	-0.48097	-0.96758	-0.63971	-1.04654	-1.05772	-0.39453

Q9BVT8	Transmeml	TMUB1 C7c	0.244734	0.259781	2.350497	1.082288	1.161638	0.490881	0.407343	0.167378	0.514102	1.192368
Q9BW27	Nuclear po	NUP85 NUI	-0.71491	-0.78991	-2.2167	-2.38012	-0.89947	-1.1989	-1.44152	-1.50666	-2.10778	-1.79676
Q9BW60	Elongation	ELOVL1 SS(	-1.25096	-1.35147	-1.7112	-1.82312	-3.07635	-3.1375	-1.96963	-1.97728	-3.00946	-3.34873
Q9BW61	DET1- and	DDA1 C19c	-0.69006	-0.06981	-0.1041	-0.02391	-0.42289	-0.40608	0.093718	0.13788	-0.05203	0.307155
Q9BW71	HIRA-intera	HIRIP3	-0.38702	-0.95395	-0.30738	-0.36167	-0.26006	-0.15565	0.050273	-0.26303	-0.07251	-0.2143
Q9BW85	Splicing fac	YJU2 CCDC	0.407459	0.637464	-0.00521	0.051921	0.282523	0.324131	0.25267	0.095965	0.12357	0.10795
Q9BWD1	Acetyl-CoA	ACAT2 ACT	-0.20798	-0.25565	0.627223	0.539816	0.49238	0.242311	0.953583	1.097403	0.973148	1.246068
Q9BWF3	RNA-bindir	RBM4 RBM	-0.75213	-0.76276	-0.29459	-0.28486	-0.32554	-0.33843	-0.59174	-0.34728	-0.27951	-0.2643
Q9BWH2	FUN14 don	FUNDC2 H(	0.213738	-0.01888	-0.35277	-0.60456	-0.52633	-0.68895	-0.43897	-0.55987	-0.63172	-0.45222
Q9BWJ5	Splicing fac	SF3B5 SF3E	0.809384	0.927841	1.157495	0.872492	1.155392	1.164159	0.852252	1.096303	1.324135	1.358176
Q9BWM7	Sideroflexir	SFXN3	-0.02538	-0.39026	-0.61644	-0.82156	-0.76409	-1.05746	-0.67744	-0.89754	-1.08702	-0.77074
Q9BWS9	Chitinase d	CHID1 GL0(	0.556393	0.58756	0.499286	0.674146	0.348454	0.369234	0.546949	0.270648	0.500835	0.487322
Q9BWU0	Kanadaptir	SLC4A1AP I	-0.24377	-0.07195	0.075949	-0.23816	0.141554	0.23173	0.286713	0.192645	0.13493	-0.0223
Q9BWW4	Single-strar	SSBP3 SSDI	-0.5025	-0.0039	-1.10512	-0.47045	-0.21621	-0.74977	-0.57731	-0.26303	-0.83112	-0.41209
Q9BX40	Protein LSN	LSM14B C2	-2.46855	-1.93147	-1.6734	-1.59756	-2.55552	-1.65208	-2.07346	-1.848	-2.10764	-1.64829
Q9BX68	Histidine tr	HINT2	1.331594	1.357362	0.790547	1.079322	1.31895	0.96123	1.198071	1.042311	1.17724	0.941291
Q9BXI6	TBC1 doma	TBC1D10A	0.003037	0.21564	0.210086	-0.00629	0.447251	0.321791	0.105561	0.058252	0.310377	0.304276
Q9BXJ9	N-alpha-ac	NAA15 GA1	0.26916	0.536574	0.036089	0.058903	-0.01512	0.077256	0.144788	-0.09303	0.142191	0.176777
Q9BXK5	Bcl-2-like p	BCL2L13 M	-1.14211	-1.12516	-0.72275	-0.37195	-1.09789	-0.42147	-0.89859	-0.23873	-0.99318	-0.61901
Q9BXL7	Caspase re	CARD11 CA	-0.78131	-1.13917	-0.56103	-0.68619	-0.41009	-0.59318	-0.97016	-0.86362	-1.05302	-0.61824
Q9BXP5	Serrate RN	SRRT ARS2	0.523035	0.882246	1.112058	0.766421	0.796692	0.760711	0.733893	0.677218	1.297839	1.075438
Q9BXS5	AP-1 comp	AP1M1 CLT	0.897421	1.074525	0.74418	0.559281	0.703297	0.73149	0.760829	0.569532	0.564664	0.59313
Q9BXV9	EKC/KEOPS	GON7 C14c	-2.6137	-2.04543	-0.87317	-1.76178	-1.22487	-1.088	-1.9301	-1.48543	-1.92661	-1.69591
Q9BXW7	Haloacid de	HDHD5 CE(	-0.01444	-0.0004	0.294558	0.497712	0.144227	0.225185	0.388164	0.182891	-0.14586	-0.20614
Q9BY11	Protein kin	PACSIN1 KI	-1.34023	-1.12199	-1.2276	-1.11757	-0.85845	-0.74061	-2.13955	-1.848	-1.76553	-1.84093
Q9BY32	Inosine trip	ITPA C20or	-1.05552	-0.95599	-0.59474	-0.66558	-0.31451	-0.52869	-0.46713	-0.47743	-0.30319	-0.50324
Q9BY42	Replication	RTF2 C20or	2.389428	2.213452	2.239282	1.909397	2.628906	2.440429	2.442591	2.516576	2.504163	2.132062
Q9BY43	Charged m	CHMP4A C	-0.2949	-0.36647	-0.05565	0.430447	-0.10007	-0.0902	0.122543	-0.34066	-0.53629	0.389364
Q9BY44	Eukaryotic	EIF2A CDA(	0.217579	0.03322	0.623117	0.574038	0.419332	0.606233	0.153634	0.290358	0.703765	0.60185
Q9BY49	Peroxisom	PECR SDR2	-1.2949	-0.96914	-0.58887	-0.72655	-0.8055	-0.70454	-1.23266	-1.0115	-0.95361	-1.44184
Q9BY77	Polymerase	POLDIP3 KI	-0.42721	-0.53703	-0.53703	-0.66558	-0.39046	-0.60977	-0.69661	-0.54265	-0.43084	-0.01334
Q9BYB4	Guanine nu	GNB1L GY2	-0.22948	-0.21397	-0.00782	-0.59756	-0.32379	-0.35789	-0.11165	-0.05842	-0.09248	0.419456
Q9BYC8	39S ribosor	MRPL32 HS	-0.38333	-0.59264	-0.52717	-0.22541	-0.48595	-0.41504	-0.25817	-0.39263	-0.11679	-0.41562

Q9BYD2	39S ribosor	MRPL9	0.445032	0.295167	0.704633	0.780219	0.694708	0.514027	0.661349	0.61891	0.806007	0.67364
Q9BYD3	39S ribosor	MRPL4 CD/	-1.75821	-1.30433	-1.02664	-1.3626	-1.65783	-1.04171	-1.58333	-0.84455	-1.04118	-0.81191
Q9BYD6	39S ribosor	MRPL1 BM	-1.03684	-0.81028	-1.10512	-0.9386	-0.88567	-0.88452	-1.02061	-0.79737	-0.78978	-0.73402
Q9BYG3	MKI67 FHA	NIFK MKI67	-0.14662	-0.49246	-0.03154	-0.50564	0.328373	0.085183	-0.28157	-0.50967	-0.38087	-0.03585
Q9BYK8	Helicase wi	HELZ2 KIAA	-0.90613	-1.43335	-0.90933	-1.56009	-1.26006	-0.64351	-0.84493	-1.04654	-1.4245	-0.80183
Q9BYM8	RanBP-type	RBCK1 C20	-0.37519	-0.63656	0.008028	-0.01132	0.110133	-0.10156	-0.10224	-0.27687	0.044882	0.114095
Q9BYN8	28S ribosor	MRPS26 C2	0	-0.07605	-0.09259	-0.19444	-0.00746	-0.1375	-0.00513	0.192645	0.17724	-0.13441
Q9BYV9	Transcriptio	BACH2	-2.60942	-1.46529	-0.96153	-0.47045	-0.97056	-1.07861	-1.28157	-1.2091	-0.66493	-0.84888
Q9BYX4	Interferon-	IFIH1 MDA!	-3.98793	-2.42286	-2.44392	-3.75803	-1.74193	-1.89465	-2.07346	-1.78746	-2.32665	-1.64829
Q9BZ23	Pantothena	PANK2 C20	-1.03448	-0.93032	-0.13401	-0.24465	-0.7076	-0.54598	-0.75815	-1.07491	-0.84196	-0.61116
Q9BZ29	Dedicator c	DOCK9 KIA	-0.53086	-0.31464	-0.1704	-0.19189	-0.07637	-0.09981	-0.05859	0.049482	-0.03267	-0.04688
Q9BZ67	FERM dom	FRMD8 FKS	-0.22948	-0.32193	-0.68589	-1.17306	-0.56649	-1.0902	-0.71329	-0.69188	-1.07748	-0.97369
Q9BZ95	Histone-lys	NSD3 WHS	-0.38398	-1.22685	-0.3498	-0.54217	-1.32888	-0.51739	-0.6245	-0.32897	-0.39839	-0.3038
Q9BZD3	Putative GI	GCOM2 GR	-0.79418	-0.63656	-0.57043	-1.00523	-0.53809	-0.76357	-0.76819	-0.75052	-0.47724	-0.41799
Q9BZE4	Nucleolar G	GTPBP4 CR	0.02838	0.401123	0.323955	-0.08552	-0.06853	-0.68684	-0.73016	-0.75811	-1.00946	-0.47221
Q9BZE9	Tether con	ASPSCR1 A	-0.22239	0.216811	-0.50901	-0.55834	-0.33313	-0.71348	-0.60817	-0.6642	-0.43063	-0.29888
Q9BZF1	Oxysterol-b	OSBPL8 KIA	0.309048	0.285772	-0.00526	0.011633	-0.09319	-0.17095	0.126748	-0.51154	-0.32958	-0.26445
Q9BZG8	2-(3-amino	DPH1 DPH2	-0.65641	-0.19599	-1.6531	-1.61054	-1.45837	-1.98201	-1.18269	-1.71403	-0.36864	-0.40621
Q9BZH6	WD repeat	WDR11 BR	-1.13993	-0.75659	-0.96914	-1	-1.33313	-1.33015	-1	-0.97728	-1.13845	-0.84888
Q9BZI7	Regulator c	UPF3B REN	1.221067	1.248229	1.515224	1.140214	1.543526	1.494183	1.440704	1.417894	1.497987	1.393451
Q9BZJ0	Crooked ne	CRNKL1 CR	0.433372	0.46537	0.473274	0.306486	0.506015	0.551685	0.442575	0.46671	0.422821	0.114225
Q9BZK7	F-box-like/	TBL1XR1 IR	-1.34794	-1.0947	0.137504	-0.25303	0.258068	0.053638	0.040358	-0.1571	-0.1913	0.052151
Q9BZL1	Ubiquitin-li	UBL5	-1.59104	-1.64874	-1.80346	-1.47045	-2.74193	-1.56866	-1.42531	-1.86876	-1.31487	-1.76377
Q9BZL4	Protein pho	PPP1R12C	-1.60684	-1.40826	-1.6357	-2.01996	-1.00724	-1.1658	-1.3661	-1.68762	-2.26297	-1.58481
Q9BZL6	Serine/thre	PRKD2 PKD	0.053549	-0.02359	0.155119	-0.152	0.18799	-0.18021	0.050273	-0.11309	0.242458	0.382591
Q9BZQ8	Protein Nib	NIBAN1 C1	0.32853	0.453551	0.196716	0.261888	-0.29788	-0.24349	0.045324	-0.20243	-0.83629	-0.82935
Q9BZV1	UBX domai	UBXN6 UBX	0.142574	0.057427	-0.04209	0.167254	-3.35199	-1.9783	-0.00808	-0.61203	-1.38702	-1.71937
Q9BZZ5	Apoptosis i	API5 MIG8	0.221786	0.291986	0.12269	0.131233	-0.08174	0.120513	-0.00455	0.088809	0.144476	0.352657
Q9C000	NACHT, LRI	NLRP1 CAR	-0.77588	-1.31221	-0.7632	-1.59756	-1.49139	-0.81558	-0.63971	-1.05842	-0.66493	-0.12473
Q9C035	Tripartite n	TRIM5 RNF	-0.12663	0.061076	-0.1305	-0.08394	-0.21621	0.032421	-0.25691	-0.36995	-0.2687	-0.08194
Q9C0B0	RING finger	UNK KIAA1	-2.39552	-2.07121	-2.06898	-2.98751	-2.23425	-1.85039	-3.14592	-2.22319	-1.86018	-1.90607
Q9C0B1	Alpha-keto	FTO KIAA17	-1.30698	-1.2066	-1.07117	-0.99151	-1.04327	-0.74692	-0.97053	-1.03234	-0.99213	-0.68936
Q9C0C9	(E3-indepe	UBE2O KIA	0.218652	0.355407	0.682799	0.748476	0.84675	0.769387	0.786323	0.776114	0.851174	0.894458

Q9C0D9	Ethanolam	SELENOI EF	-0.43539	-1.13906	-1.19153	0.143195	-0.71727	-0.22422	-0.53964	0.688056	-0.71049	-0.40036
Q9C0E8	Endoplasm	LNPK KIAA1	-1.35566	-1.10512	-0.55984	-1.89129	-1.33313	-0.39334	-0.9301	-1.09464	-1.4245	-1.20918
Q9C0G0	Zinc finger	ZNF407 KIA	-0.98793	-0.87317	-1.03906	-0.50465	-0.89535	-1.31918	-1.17206	-2.07039	-1.70035	-1.46988
Q9C0J8	pre-mRNA	WDR33 WI	-0.69438	-0.43622	-1.17056	-2.31177	-1.70681	-2.3979	-1.74468	-1.29823	-2.23614	-1.64595
Q9C0K0	B-cell lymp	BCL11B CTI	-0.8913	-0.97679	-0.42071	-0.41221	-0.70855	-0.2371	-0.45628	-0.56573	-0.51829	-0.29748
Q9GZL7	Ribosome l	WDR12	-2.37673	-1.83683	-0.38672	-1.10402	-0.79256	-0.44448	-1.87471	-1.95134	-1.80029	-1.70617
Q9GZM5	Protein YIP	YIPF3 C6or	-0.68536	-1.12199	-0.69218	0.378512	-0.74193	-0.31647	-1.01027	0.596367	-0.65006	-0.60054
Q9GZP4	PITH doma	PITHD1 C1c	-1.93464	-1.7146	-2.71853	-1.99527	-2.11702	-1.71954	-2.32079	-2.06083	-1.33049	-1.46598
Q9GZR1	Sentrin-spe	SENP6 KIAA	0.234465	0.213452	0.440162	0.791135	0.48755	0.398549	0.376148	0.36257	0.43958	0.631411
Q9GZR2	RNA exonu	REXO4 PMC	-0.88359	-1.07195	-1.5656	-1.71621	-0.77973	-1.23704	-1.74723	-1.22239	-1.45003	-1.55431
Q9GZR7	ATP-depen	DDX24	0.260213	0.263298	-0.01635	-0.15309	-0.03702	-0.07976	-0.09525	-0.37471	-0.32249	0.05152
Q9GZS3	WD repeat	WDR61	-0.32108	-0.10248	0.003946	0.035286	0.28578	0.083554	0.008647	0.289567	0.613671	0.673477
Q9GZT3	SRA stem-l	SLIRP C14o	-1.25096	-0.96153	-1.06378	-1.03814	-1.24236	-1.12553	-1.11718	-1	-1.01898	-0.75627
Q9GZT8	NIF3-like p	NIF3L1 ALS	-1.31017	-0.91265	-1.52314	-1.33446	-0.21206	-0.01729	-0.06124	-0.05472	-0.39231	-0.483
Q9GZT9	Egl nine ho	EGLN1 C1o	1.068078	0.972444	1.378512	1.296617	1.655116	1.319741	1.489805	1.315042	1.377423	1.316608
Q9GZU8	PSME3-inte	PSME3IP1 (	-1.98793	-1.57719	-1.81714	-2.84549	-2.95606	-3.23704	-2.18536	-3.1193	-1.78165	-0.99118
Q9GZY8	Mitochond	MFF C2orf3	-2.85042	-2.12199	-1.62449	-1.82312	-1.84503	-1.34395	-1.63176	-1.74846	-1.52947	-2.2402
Q9GZZ1	N-alpha-ac	NAA50 MA	1.065184	1.11067	1.014057	1.096908	1.116584	1.268489	1.231136	1.33787	1.214287	1.102483
Q9GZZ9	Ubiquitin-li	UBA5 UBE1	-0.4783	-0.61099	-0.7211	-0.54197	-0.56151	-0.33837	-0.47067	-0.30843	-0.77157	-0.62862
Q9H000	Probable E	MKRN2 RN	-4.19438	-4.13906	-2.44392	-3.52356	-3.51245	-3.79647	-3.61599	-3.22239	NA	NA
Q9H008	Phospholys	LHPP	1.133088	1.253253	-0.4021	0.439912	0.258068	1.308753	0.794139	0.36257	0.477322	-0.27727
Q9H019	Mitochond	MTFR1L FA	-0.64117	-0.46477	-0.54624	-0.77428	-0.38407	-0.41622	-0.31382	-0.40673	-0.41531	-0.17693
Q9H078	Caseinolyti	CLPB HSP78	-2.98793	-1.60064	-1.80346	-1.82312	-1.14048	-1.44448	-1.55459	-1.36257	-1.30319	-1.63448
Q9H081	Protein MIS	MIS12	-1.12663	-0.6734	-1.25526	-1.24558	-1.01495	-1.2115	-1.12832	-0.76783	-1.16993	-0.53495
Q9H098	Protein FAI	FAM107B (	0.562149	0.693869	1.174307	1.198901	1.053394	1.118908	1.084019	1.291115	1.187136	1.07293
Q9H0A0	RNA cytidir	NAT10 ALP	0.254929	0.357362	-0.06165	0.086241	0.256082	0.189226	0.200299	0.227291	-0.21847	-0.24544
Q9H0B6	Kinesin ligh	KLC2	-0.93622	-0.90225	-0.61094	-1.07737	-0.66041	-0.56602	-0.80644	-1.03519	-0.61117	-0.69629
Q9H0C8	Integrin-lin	ILKAP	0.902844	0.860936	0.90116	1.049328	0.867693	0.947128	0.968973	0.716207	1.044108	1.0649
Q9H0D6	5'-3' exorib	XRN2	-0.13599	0.187397	-0.1505	0.067399	0.087727	0.295741	0.032364	0.004135	0.036543	-0.0711
Q9H0E2	Toll-interac	TOLLIP	-0.93339	-1.03354	-1.14068	-1.00355	-1.10027	-0.88569	-1.56903	-1.64929	-1.44903	-1.16232
Q9H0E3	Histone de	SAP130	0.77279	0.966019	1.175205	0.807327	1.175941	1.177553	1.046524	1.156205	1.45819	1.216648
Q9H0E9	Bromodom	BRD8 SMAI	-1.20832	-0.18265	0.034673	-0.02531	0.086588	0.19878	0.045324	0.088809	-0.3932	-0.26658
Q9H0F6	Sharpin (Sh	SHARPIN SI	-1.57289	-1.78991	-1.62449	-1.63604	-1.57755	-1.58496	-1.39808	-1.10692	-1.06756	-1.22979

Q9H0G5	Nuclear spe	NSRP1 CCD	-0.57289	-1.17383	-0.6672	-0.64582	-0.5231	-0.91511	-0.59266	-0.56789	-0.4119	-0.38873
Q9H0H0	Integrator	INTS2 KIAA	-0.34509	-0.16745	-0.21834	-0.20327	-0.18992	-0.11668	-0.33459	-0.16731	-0.25577	-0.01965
Q9H0L4	Cleavage st	CSTF2T KIA	-1.51737	-1.26877	-1.00782	-1.30633	-1.51245	-1.41504	-1.04152	-1.26303	-0.89984	-0.97369
Q9H0P0	Cytosolic 5	NT5C3A NT	-3.30986	-2.15634	-1.87317	-3.14505	-3.2076	-3.18641	-2.18536	-2.04654	-1.78165	-1.70487
Q9H0R1	AP-5 comp	AP5M1 C14	0.727234	0.998049	0.695031	0.720564	0.83054	0.676705	0.808816	0.596367	0.556393	0.396106
Q9H0R4	Haloacid de	HDHD2	0.007979	0.287874	-0.72872	-0.80004	-0.52135	-0.35901	-0.64144	-1.18629	-0.40945	-0.1513
Q9H0S4	Probable A	DDX47	-0.37126	-0.24136	-0.26459	-0.51457	-0.32379	-0.17403	-0.35165	-0.63743	-0.39941	0.03065
Q9H0U4	Ras-relatec	RAB1B	1.688265	1.8927	0.850325	0.432074	0.327327	0.08383	0.645447	0.30408	0.291371	0.503031
Q9H0U9	Testis-spec	TSPYL1 TSP	-0.47002	-0.48076	0.084478	-0.43563	-0.17345	-0.13082	-0.33151	-0.06389	-0.11805	0.012142
Q9H0W9	Ester hydro	C11orf54 L	-0.02586	-0.09087	0.431779	0.338617	0.560203	0.836587	0.300532	0.379584	0.69138	0.262577
Q9H147	Deoxynucle	DNTTIP1 C2	-2.58488	-2.46099	-1.75012	-2.18721	-2.53079	-1.97905	-2.15527	-2.10839	-2.24638	-1.49428
Q9H1B7	Probable E3	IRF2BPL C1	-3.03684	-4.44392	-3.57719	-2.75803	-2.69302	-4.1375	NA	NA	-3.93546	-1.92246
Q9H1E3	Nuclear ub	NUCKS1 NU	0.530837	0.495229	0.133954	0.230087	0.181447	0.248919	0.110512	-0.05601	0.194082	0.50772
Q9H1E5	Thioredoxi	TMX4 KIAA	-0.24747	-0.15794	-1.13743	-1.08949	-1.24861	-1.8816	-0.72368	-1.17761	-1.52908	-1.66393
Q9H1I8	Activating s	ASCC2 ASC	-1.13278	-0.99257	-1.09991	-0.96844	-0.79175	-1.37649	-0.2177	0.130866	-1.58076	-1.05241
Q9H1J1	Regulator c	UPF3A REN	-0.31739	-0.39696	-0.4814	-0.84356	-1.57708	-1.6881	-1.49512	-0.99828	-0.6943	-0.70239
Q9H1K1	Iron-sulfur	ISCU NIFUN	0.160465	0.462972	0.310969	0.067399	0.84717	0.703799	0.584963	0.581141	1.244449	0.833666
Q9H1Y0	Autophagy	ATG5 APG5	-0.07464	0.042266	0.23763	0.245872	0.479165	0.495682	0.284736	0.289567	0.160465	0.155098
Q9H223	EH domain	EHD4 HCA1	-1.19438	-0.95395	-1.08017	-1.23606	-1.09212	-1.04439	-1.31937	-1.13179	-1.51592	-1.05411
Q9H269	Vacuolar pr	VPS16	1.381307	1.36192	1.427171	1.428306	1.362983	1.642106	1.350297	1.369234	1.360998	1.510807
Q9H270	Vacuolar pr	VPS11 RNF	-1.74557	-1.62548	-1.53098	-1.49197	-0.90725	-1.08125	-0.86451	-1.18281	-0.90309	-0.61885
Q9H299	SH3 domai	SH3BGRL3	-0.09392	0.279079	-0.79412	-0.34756	-0.51128	-0.82526	-0.60817	-0.64764	-0.86046	-0.63507
Q9H2G2	STE20-like	SLK KIAA02	-0.69046	-0.7492	-0.69391	-0.58102	-0.47167	-0.4241	-0.67394	-0.36802	-0.83283	-0.66678
Q9H2G4	Testis-spec	TSPYL2 CDA	0.187331	0.11967	0.360402	0.108706	0.492844	0.435386	0.434096	0.389042	0.285644	0.767616
Q9H2H8	Peptidyl-pr	PPIL3	-0.10616	-8.43E-05	0.599726	0.53461	1.14053	0.864741	-0.56484	-1.03477	0.586307	0.686841
Q9H2J4	Phosducin-	PDCL3 PhLI	-2.98793	-2.0834	-2.66149	-2.35721	-2.85399	-2.82917	-2.30933	-1.31449	-1.70525	-2.19172
Q9H2K0	Translation	MTIF3 DC3	1.541894	1.465798	1.721284	1.57447	1.768594	1.575826	1.78229	1.717948	1.628309	1.601099
Q9H2K8	Serine/thre	TAOK3 DPK	-0.35767	-0.25801	-0.63981	-0.76773	-0.53381	-0.59184	-0.35495	-0.55603	-0.49627	-0.5444
Q9H2M9	Rab3 GTPa	RAB3GAP2	-0.42986	-0.58656	-0.11041	-0.46549	-0.81355	-0.37307	-0.68041	-0.53442	-0.47156	-0.39238
Q9H2P0	Activity-de	ADNP ADN	0.206634	0.261757	-0.19623	0.257935	0.093462	0.137175	0.431166	0.11367	0.352313	-0.00425
Q9H2U1	ATP-depen	DHX36 DDX	-1.1669	-1.20945	-1.18265	-1.0126	-0.95606	-1.66935	-1.22069	-1.76783	-0.97199	-0.7713
Q9H2U2	Inorganic p	PPA2 HSPC	-0.0899	-0.01071	-0.28345	0.031249	-0.83173	-0.08439	0.034501	0.259042	0.281771	0.262297
Q9H2W6	39S ribosor	MRPL46 C1	0.338802	0.534708	0.921889	1.503238	0.614406	0.868495	0.536406	0.344648	0.556393	-0.00443

Q9H307	Pinin (140 l	PNN DRS M	0.612977	0.675633	0.856636	0.791135	0.981288	0.539144	0.383925	0.85561	0.857585	0.740483
Q9H330	Transmeml	TMEM245	0.802748	1.052925	0.604774	0.846075	0.3759	0.013778	0.929745	0.671178	0.027955	0.27832
Q9H334	Forkhead b	FOXP1 HSP	-1.25096	-0.8519	-0.53703	-0.82312	-0.36965	-0.28451	-0.73201	-0.61641	-0.47603	-0.44786
Q9H3K2	Growth hoi	GHITM DEI	1.152306	0.815132	0.448758	0.363963	0.306978	0.402689	0.749192	0.719688	0.460289	-0.1008
Q9H3K6	BolA-like pi	BOLA2 BOL	1.178473	1.325356	1.199399	1.140034	1.075379	1.029886	0.906169	0.906978	1.4756	1.291579
Q9H3N1	Thioredoxi	TMX1 TMX	0.067728	0.09299	-0.25256	-0.63026	-0.72765	-0.43044	-0.46739	-0.54834	-0.78528	-0.98839
Q9H3P2	Negative el	NELFA WH!	-0.5025	-0.53138	-0.79765	-0.76109	-0.22487	-0.40054	-0.74723	-0.87852	-0.54314	-0.0223
Q9H3P7	Golgi resid	ACBD3 GCI	0.361333	0.353913	-0.26696	-0.42164	-0.23276	-0.4057	-0.3762	-0.41109	-0.40884	-0.20308
Q9H3S4	Thiamin py	TPK1	-0.89623	-0.75254	-2.86041	-1.87467	-1.43054	-0.91038	-1.1777	-2.12016	-1.06689	-1.58548
Q9H3S7	Tyrosine-pi	PTPN23 KI/	-0.99673	-0.63462	-0.66827	-0.95509	-0.90054	-0.44893	-0.90323	-0.87274	-0.64637	-1.01305
Q9H3U1	Protein un	UNC45A SI	-0.37126	-0.57138	-0.13478	0.334419	0.114333	-0.34395	-0.0574	-0.39985	-0.26303	0.170908
Q9H3Z4	DnaJ homo	DNAJC5 CL	1.713747	1.677186	1.087912	1.545638	0.98571	1.953325	1.555424	1.787508	0.514415	0.418807
Q9H400	Lck-interac	LIME1 LIMI	-3.57289	-2.44392	-1.18265	-2.75803	-1.87199	-1.65208	-1.9898	-1.56789	-2.00946	-1.46609
Q9H410	Kinetochor	DSN1 C20o	-0.37912	-0.45457	-0.37656	-0.26053	-0.269	-0.23061	-0.46713	-0.41504	-0.08246	-0.29888
Q9H444	Charged m	CHMP4B C.	-0.1526	-0.23733	0.190561	-0.02449	-0.01386	-0.30489	-0.15952	-0.13243	0.164869	-0.14866
Q9H4A3	Serine/thre	WNK1 HSN	-0.35618	-0.13118	0.199938	0.333921	-0.24548	-0.11958	0.209313	-0.0115	0.018737	0.052151
Q9H4A4	Aminopept	RNPEP APB	-0.1959	0.119184	-0.34459	-0.56931	-0.27768	-0.41593	-0.18636	-0.39993	-0.43148	-0.06436
Q9H4A5	Golgi phos	GOLPH3L G	-2.76553	-2.34156	-1.94642	-1.26053	-1.35199	-2	-1.87276	-3.72935	-2.21299	-2.06333
Q9H4A6	Golgi phos	GOLPH3 GI	-0.1004	-0.22304	-0.51457	-0.64582	-0.6574	-0.39334	-0.9026	-1.10507	-0.78978	-0.3431
Q9H4E7	Differential	DEF6 IBP	0.569436	0.551851	0.365861	0.317013	0.371631	0.24401	0.410472	0.236516	0.140201	-0.04137
Q9H4G4	Golgi-assoc	GLIPR2 C9c	-1.31693	-1.21927	-1.25959	-1.19753	-1.17326	-1.28654	-1.43208	-1.29187	-1.50374	-1.98658
Q9H4L5	Oxysterol-l	OSBPL3 KI/	-0.73724	-0.94932	-0.45999	-0.47974	-0.34883	-0.39217	-0.50911	-0.69566	-1.11179	-1.21433
Q9H4L7	SWI/SNF-re	SMARCAD1	-0.2949	-0.30738	-0.44923	-0.27563	-0.40024	-0.27621	-0.46007	-0.43812	-0.55695	-0.30433
Q9H4M9	EH domain	EHD1 PAST	0.893085	1.205274	0.882246	1.102877	0.981288	1	1.146079	1.172467	0.787006	0.860725
Q9H4X1	Regulator c	RGCC C13o	-3.25096	-2.19153	-2.48699	-1.61667	-2.59991	-3.65208	-3.96963	-3.16993	-3.30319	-2.21945
Q9H4Z3	mRNA (2'-C	PCIF1 C20c	0.734313	0.768615	0.46392	0.157191	0.545655	0.555442	0.435034	0.425378	0.537013	0.174749
Q9H501	ESF1 homo	ESF1 ABTAI	-1.44081	-1.53891	-1.45196	-0.85431	-1.22059	-1.45103	-1.13788	-1.02027	-1.02142	-0.71475
Q9H583	HEAT repea	HEATR1 BA	-1.59104	-2.28345	-1.52015	-1.5787	-1.84503	-1.85457	-1.87276	-1.78746	-1.55695	-1.56737
Q9H5N1	Rab GTPase	RABEP2 RA	-1.2949	-1.43335	-2.25926	-2.39204	-1.94036	-2.05058	-1.76023	-2.55141	-1.60693	-2.244
Q9H6A0	DENN dom	DENND2D	-2.2075	-1.825	-1.60805	-1.58175	-1.16444	-1.11443	-0.39541	-1.24897	-1.08123	-0.81703
Q9H6F5	Coiled-coil	CCDC86 CY	-0.73923	-0.6656	-0.91878	-1.12955	-0.46091	-0.52831	-0.52388	-1.1888	-1.2393	-0.61067
Q9H6K4	Optic atrop	OPA3	-3.13993	-2.07195	-1.83094	-2.98751	-2.89947	-1.26303	-1.60039	-1.63743	-1.55695	-1.71937
Q9H6R4	Nucleolar p	NOL6	-0.20133	-0.2276	-0.93147	-1.71621	-0.68105	-0.4975	-1.09516	-0.78746	-0.59918	-0.69768

Q9H6S0	3'-5' RNA h	YTHDC2	-1.04933	-1.24598	-1.52015	-1.41923	-1.14048	-1.27621	-1.45305	-1.34792	-1.4119	-1.41799
Q9H6S1	5-azacytidi	AZI2 NAP1	-1.26546	0.210086	-0.62449	0.043253	-0.07635	0.037755	-0.5697	-0.40742	-0.35652	-0.69052
Q9H6S3	Epidermal g	EPS8L2 EPS	-4.30986	-2.19153	-3.20945	-3.32193	NA	NA	NA	NA	-3.79796	-3.76377
Q9H6T3	RNA polym	RPAP3	-0.5025	-0.487	-0.39165	-0.12486	-0.28774	-0.24717	-0.22845	-0.5856	-0.07786	-0.03979
Q9H6U6	Breast carc	BCAS3	0.088176	-0.01568	0.451612	0.252542	0.619256	0.415037	0.278301	0.110183	0.477322	0.422761
Q9H6W3	Ribosomal	RIOX1 C14o	-0.60251	-0.62	-2.21918	-1.34988	-1.63915	-1.1532	-0.72259	-1.03481	-2.02046	-1.44641
Q9H6Y2	WD repeat	WDR55	0.4932	0.381508	0.148099	0.037153	-0.04532	0.105353	-0.08427	0.136462	0.494156	0.665292
Q9H6Y7	E3 ubiquitir	RNF167 LP3	-1.96408	-1.72403	-2.07195	-3.38606	-3.35199	-2.72247	-1.91073	-3.16993	-3.61353	-2.39453
Q9H6Z4	Ran-binding	RANBP3	-0.9523	-1.07477	-1.36146	-1	-1.37109	-1.39982	-1.34514	-1.60297	-1.2687	-1.12065
Q9H773	dCTP pyrop	DCTPP1 XT	-3.25096	-2.38163	-1.88753	-1.61667	-3.35199	-2.95693	-1.96963	-3.72935	-3.86507	-2.11991
Q9H7C9	Mth938 do	AAMDC C1	0.698505	1.04341	-0.39205	-0.48387	-0.16821	-0.43111	-0.24478	-0.66329	0.02192	-0.32317
Q9H7D7	WD repeat	WDR26 CD	-1.28011	-1.03154	-1.6734	-1.98751	-2.59991	-1.85457	-1.68012	-1.63743	-1.25739	-1.54137
Q9H7J1	Protein pho	PPP1R3E	-0.63617	-0.65343	-1.11006	-0.67797	-0.87598	-1.06761	-0.71265	-1.38577	-0.67962	-0.73218
Q9H7L9	Sin3 histon	SUDS3 SAP	-0.89482	-0.90933	-0.35147	-0.47045	-0.62263	-0.26961	-0.46007	-0.88982	-0.46297	-0.28434
Q9H7M9	V-type imm	VSIR C10or	-1.32496	-0.95395	-0.61849	-0.36167	-1.27798	-0.91511	-0.03626	-0.16993	-1.13845	-1.08194
Q9H7N4	Splicing fac	SCAF1 SFRS	-2.64689	-1.4761	-1.11806	-1.10428	-1.20027	-1.44287	-1.28211	-1.50666	-1.07748	-1.80957
Q9H7X3	Zinc finger	ZNF696	-1.96408	-1.90202	-3.62449	-3.32193	-2.79256	-3.65208	-1.74723	-1.72935	-3.04793	-2.00888
Q9H7Z3	Nuclear exo	NRDE2 C14	-3.89482	-4.94642	-4.13906	-3.45317	-4.01495	-3.34395	-4.55459	-3.88982	NA	NA
Q9H7Z7	Prostagland	PTGES2 C9o	0.337838	0.471261	0.176068	0.159249	0.183649	0.45328	0.556104	0.421006	-0.14821	-0.21163
Q9H814	Phosphoryl	PHAX RNUX	0.073616	-0.03769	-0.18838	-0.16687	0.230642	0.335043	0.145658	0.146925	0.050707	-0.11972
Q9H832	Ubiquitin-c	UBE2Z HOY	-1.09219	-0.63963	-0.64484	-0.8484	-1.24537	-0.75315	-0.66894	-0.946	-0.92636	-0.41432
Q9H845	Complex I a	ACAD9	0.159322	0.320651	0.274275	0.170791	0.081898	0.531525	0.215233	0.382856	-0.03812	0.211381
Q9H869	YY1-associa	YY1AP1 HC	-2.85042	-3.72403	-3.4021	-3.59756	-3.07635	-3.23704	-3.89162	-3.33342	-3.93546	-3.8893
Q9H8H0	Nucleolar p	NOL11 L14	-1.22225	-1.1187	-0.98449	-0.90886	-1.16531	-1.33507	-1.17631	-0.94922	-1.22772	-0.86291
Q9H8H2	Probable A	DDX31	-2.94062	-2.24598	-1.44392	-3.0906	-1.45017	-1.41504	-1.83577	-1.2091	-1.54314	-1.45391
Q9H8H3	Methyltran	METTL7A P	1.402098	1.562696	0.871631	0.812721	0.923649	0.775785	0.926538	0.807355	0.776035	0.738735
Q9H8U3	AN1-type z	ZFAND3 TE	-0.36344	-1.04757	-0.48153	-0.64582	-0.53382	-0.51301	-0.782	-0.34792	-0.38702	-0.69768
Q9H8W4	Pleckstrin h	PLEKHF2 ZF	0.005733	-0.02956	-0.87317	-1.18728	-1.06075	-1.50524	-1.05209	-1.6735	-1.3505	-1.49075
Q9H8Y5	Ankyrin rep	ANKZF1 ZN	-2.98793	-3.88753	-2.08844	-3.84549	-4.42999	-3.1375	-3.68012	-3.80735	-3.16993	-2.51584
Q9H8Y8	Golgi reass	GORASP2 G	-0.55168	-0.73119	-0.19999	-0.02834	-0.38273	-0.55094	-0.42767	-0.36921	-0.48722	-0.23969
Q9H910	Jupiter mic	JPT2 C16or	-1.11898	-0.87148	-0.84724	-1.15554	-0.91109	-0.95147	-1.19816	-0.85254	-0.81639	-0.68563
Q9H930	Nuclear bo	SP140L	-1.39341	-1.35646	-0.9269	-0.83909	-1.0495	-0.78809	-1.34297	-0.89679	-0.97246	-0.64271
Q9H936	Mitochond	SLC25A22 G	-2.91918	-2.33442	-2.15206	-2.22034	-3.57772	-1.98915	-2.07411	-2.27791	-2.02473	-2.61188

Q9H939	Proline-ser	PSTPIP2	0.3541	0.295251	0.526846	0.584762	0.532602	0.415358	0.610045	0.27461	0.672937	0.62226
Q9H993	Damage-cc	ARMT1 C6c	0.719892	0.558731	1.179425	2.272153	2.221861	1.555519	1.677432	1.911463	0.959358	1.017594
Q9H9A5	CCR4-NOT	CNOT10	-0.36344	-0.16506	-0.64928	-0.33578	-0.03006	-0.20519	-0.38466	-0.06439	0.108934	0.039289
Q9H9A6	Leucine-ric	LRRC40	-0.32972	-0.16658	-0.52924	-0.43634	-0.22871	-0.15281	-0.31615	-0.38162	-0.29892	-0.16217
Q9H9B1	Histone-lys	EHMT1 EUI	-0.88359	-1.45457	-0.91667	-1.10402	-0.91341	-0.73151	-1.64026	-1.93041	-0.44361	-1.1589
Q9H9B4	Sideroflexi	SFXN1	0.488887	0.326868	0.216811	0.586712	0.18799	0.588601	0.131912	0.152003	-0.37474	-0.46609
Q9H9E3	Conserved	COG4	-1.34286	-0.86741	-0.98507	-0.80829	-1.19766	-0.72891	-0.87921	-1.16717	-0.99134	-0.82661
Q9H9J2	39S ribosoı	MRPL44	-0.411	-1	-0.79667	-0.92663	-0.8055	-0.60977	-0.22069	-1.14439	-2.43546	-2.34172
Q9H9L3	Interferon-	ISG20L2 HS	-1.59463	-1.0362	-2.00476	-1.65014	-1.78729	-1.57277	-1.79477	-1.84067	-1.55962	-1.31661
Q9H9L4	KAT8 regul	KANSL2 C1	-2.49396	-2.50374	-2.26156	-1.62402	-2.28934	-1.911	-0.67195	-0.75811	-2.09502	-2.10657
Q9H9P8	L-2-hydrox	L2HGDH C1	-0.70499	-0.25992	-1.39183	-1.05107	-2.74193	-0.52869	-0.94973	-3.1193	-0.58496	-0.64137
Q9H9Q2	COP9 signa	COPS7B CS	0.176645	0.638544	-0.49795	-0.85916	-0.5488	-0.73857	-0.73185	-0.55393	-0.43287	-0.15389
Q9H9Q4	Non-homol	NHEJ1 XLF	-0.656	-0.58229	-0.75724	-0.69421	-0.64132	-1.01114	-1.11735	-0.8794	-0.68371	-0.54067
Q9H9Y2	Ribosome	RPF1 BXDC	-0.34947	-0.61713	-0.27479	-0.49147	-0.23075	-0.11545	-0.24018	-0.30511	-0.1064	-0.01083
Q9HA64	Ketosamin	FN3KRP	1.141356	1.241758	0.890683	1.126054	1.526792	1.908347	1.776328	1.622633	1.40617	1.241859
Q9HA65	TBC1 doma	TBC1D17	-0.59463	-0.17123	-0.75265	-0.80325	-0.71293	-0.69863	-0.29674	-0.54434	-0.90673	-0.70644
Q9HA77	Probable c	CARS2 OK/	0.176645	0.23349	0.623437	0.568138	0.220266	0.326293	0.797087	0.790904	0.874469	0.751934
Q9HAB8	Phosphopa	PPCS COAB	0.501787	0.597902	0.90116	0.15451	0.559958	0.189226	0.5115	0.237039	0.546743	0.785698
Q9HAF1	Chromatin	MEAF6 C1c	0.462734	0.381508	0.59274	0.646363	0.879867	0.690316	0.677432	0.561879	0.929964	0.945893
Q9HAJ7	Histone de	SAP30L NS	0.285091	0.558731	-0.49796	-0.0906	-0.05302	-0.40777	-0.11165	-0.2091	-0.12296	0.368948
Q9HAU5	Regulator c	UPF2 KIAA	-0.06426	0.053786	0.403777	0.405429	0.282134	0.34253	0.163188	0.398021	0.480392	0.564009
Q9HAV4	Exportin-5	XPO5 KIAA	-0.71183	-0.30324	-0.61776	-0.93261	-0.83058	-0.68953	-0.70823	-0.6547	-0.93588	-0.62509
Q9HAV7	GrpE prote	GRPEL1 GR	0.684373	0.595346	0.692934	0.477679	0.522308	0.594082	0.673147	0.821993	0.968374	0.895469
Q9HB07	MYG1 exor	MYG1 C12c	0.62629	0.776363	0.544444	0.641017	0.508952	0.791642	0.754174	0.597197	0.833974	0.97664
Q9HB19	Pleckstrin	PLEKHA2 T	-0.3646	-0.57069	-0.47925	-0.71261	-0.46051	-0.7847	-0.5251	-0.6219	-0.78641	-0.82126
Q9HB21	Pleckstrin	PLEKHA1 T	-3.25096	-2.34156	-2.50901	-1.9386	-3.14048	-1.68684	-3.38466	-3.65535	-3.3505	-2.28264
Q9HB58	Sp110 nucl	SP110	-1.28011	-1.2185	-0.09259	-0.79021	-1.04532	-1.04439	-1.28157	-1.05395	-0.5025	-0.42986
Q9HB71	Calcyclin-bi	CACYBP S1	0.730891	0.751707	0.909488	0.826935	0.460783	0.938599	0.550447	0.790904	0.480704	0.833666
Q9HB90	Ras-relatec	RRAGC	-0.85042	-0.64264	-0.93147	-1.18728	-1.14048	-0.80599	-0.71329	-0.64636	-1.00946	-0.65524
Q9HBG7	T-lymphoc	LY9 CDABP	-1.04933	-0.93892	-0.94642	-0.69574	-0.87199	-0.58496	-1.18536	-1.09464	-0.90866	-1.03585
Q9HBH0	Rho-relate	RHOF ARHI	-0.86139	-0.68589	-1.80346	-1.96285	-2.79256	-1.5366	-1.9301	-2.80735	-1.95361	-2.1788
Q9HBH5	Retinol del	RDH14 PAN	-1.13832	-1.03704	-1.32536	-1.38606	-1.82695	-1.48948	-1.78619	-1.78558	-1.33852	-1.27191
Q9HBL7	Plasminoge	PLGRKT C9	1.254822	1.132138	0.681821	0.595951	0.545556	0.711446	0.785891	0.656812	0.718484	0.283893

Q9HC16	DNA dC->d	APOBEC3G	0.382334	1.053071	-0.26111	0.452801	-0.60229	-0.38146	0.090869	-0.62847	-0.83039	-0.99096
Q9HC35	Echinoderm	EML4 C2or	0.379743	0.451148	0.152706	0.066332	-0.28876	-0.0809	0.307813	0.160415	-0.15295	0.029814
Q9HC36	rRNA meth	MRM3 RNM	-2.64689	-2.36146	-1.15634	-1.45317	-1.76702	-1.11366	-1.9301	-1.93289	-1.2916	-1.04495
Q9HC38	Glyoxalase	GLOD4 C17	0.314636	0.256521	0.357362	0.006259	0.517851	0.347923	0.470165	0.212688	0.727912	0.754321
Q9HCC0	Methylcrot	MCCC2 MC	0.784662	0.443033	0.360402	0.349267	0.065423	0.268489	0.460361	0.094182	0.273994	0.521637
Q9HCD5	Nuclear rec	NCOA5 KIA	0.085111	0.202512	0.338697	0.153276	0.001245	0.095339	0.409238	0.324156	0.220227	0.20657
Q9HCE5	N6-adenos	METTL14 K	0.285108	0.464136	0.121102	0.238435	0.071888	0.277534	0.001631	0.218409	0.139115	0.285885
Q9HCG8	Pre-mRNA-	CWC22 KIA	-1.24093	-0.32812	-0.61657	-0.71115	-0.18334	-0.62617	-0.97624	-0.77474	-0.41902	-0.50272
Q9HCH5	Synaptotag	SYTL2 KIAA	0.902844	1.034673	1.277583	1.097024	1.068972	1.247928	1.195839	0.932613	0.285644	0.73608
Q9HCI7	E3 ubiquiti	MSL2 KIAA	0.376645	0.369485	0.288798	0.359081	0.460783	0.254814	0.426503	0.217477	-0.08746	-0.20918
Q9HCK8	Chromodo	CHD8 HELS	-1.09463	-1.06398	-0.9372	-0.92737	-0.81534	-0.78969	-0.84804	-1.1626	-0.73378	-1.08664
Q9HCN4	GPN-loop G	GPN1 MBD	-1.05618	-0.57829	-0.0872	-0.12831	-0.0333	-0.21923	-0.34838	-0.374	-0.22233	-0.20605
Q9HCN8	Stromal cel	SDF2L1 UN	-0.12003	0.049819	-0.11775	-0.26806	0.043943	-0.39334	0.209177	-0.27684	-0.67996	-0.60054
Q9HCS7	Pre-mRNA-	XAB2 HCNF	0.501787	0.416921	0.019365	0.061401	-0.07635	0.095157	0.173332	0.067114	0.108934	0.209693
Q9HCU5	Prolactin re	PREB SEC12	-0.53727	-0.60657	-0.94642	-0.63604	-0.95606	-1.14958	-0.63971	-0.922	-1.11784	-0.7713
Q9HCU9	Breast canc	BRMS1	0.147244	0.3118	-0.41129	-0.61194	-0.37431	-0.44602	-0.36043	-0.01841	-0.25849	-0.06246
Q9HD15	Steroid rec	SRA1 PP768	0.212342	0.301824	0.50593	0.536103	0.549825	0.452965	0.422676	0.745599	0.826978	0.732231
Q9HD20	Manganese	ATP13A1 A	1.159965	0.910808	0.348319	0.317672	-0.10815	-0.29351	0.649451	0.057424	-0.6094	0.042797
Q9HD33	39S ribosor	MRPL47 NC	-1.34023	-1.46529	-1.6734	-1.23078	-4.01495	-1.75899	-1.9301	-1.30485	-1.38702	-2.1788
Q9HD34	LYR motif-c	LYRM4 C6o	0.564614	0.521187	0.826171	0.507657	0.81794	0.822885	0.910297	0.877588	1.106747	1.325464
Q9HD42	Charged m	CHMP1A C	-0.62125	-0.66731	-0.24349	-0.09834	-0.06733	-0.13224	-0.16151	-0.10266	-0.13865	-0.17006
Q9HD45	Transmeml	TM9SF3 SM	0.609008	0.665835	-0.4021	-0.11078	-1.19052	0.005454	0.191366	-0.13808	-0.75754	-1.12956
Q9HDC9	Adipocyte p	APMAP C20	1.042709	1.164748	0.174184	0.281711	-0.57329	0.064642	0.463212	0.031189	-1.38634	-1.23612
Q9NNW7	Thioredoxi	TXNRD2 KIA	0.969368	0.797293	-1.52848	-1.59582	0.739937	0.791656	0.696499	0.498454	1.556393	1.439174
Q9NP31	SH2 domai	SH2D2A SC	-0.77588	-0.05971	-0.15634	0.334419	-0.19052	-0.31647	-0.13955	-0.2978	-0.20754	0.021959
Q9NP61	ADP-ribosy	ARFGAP3 A	-1.54623	-1.63043	-0.80745	-0.25994	-2.26721	-1.71009	-1.54929	-1.40035	-1.80394	-1.64822
Q9NP64	Nucleolar p	ZCCHC17 P	-1.46855	-1.42286	-1.23676	-1.32193	-1.49139	-1.17403	-1.34514	-0.85834	-0.66493	-1.37145
Q9NP72	Ras-relatec	RAB18	-2.16921	-1.99901	-1.9954	-2.80176	-2.00284	-2.15085	-2.42198	-1.70155	-2.33269	-2.6398
Q9NP77	RNA polym	SSU72 HSP	0.825369	1.00967	0.458666	0.747354	0.501847	0.362597	0.638702	0.351728	0.745189	0.832304
Q9NP79	Vacuolar pi	VTA1 C6orf	0.484561	0.27919	0.723647	0.498806	0.7175	0.895185	0.348289	0.353637	0.889817	0.855843
Q9NP80	Calcium-inc	PNPLA8 IPL	-1.72489	-1.09675	-0.39696	-0.06413	-0.91341	-0.20519	-0.59266	-0.59358	-0.62803	-1.1589
Q9NP81	Serine--tRN	SARS2 SARS	0.068353	0.263034	0.320368	0.163219	-0.25118	-0.20889	0.333502	0.317546	-0.03422	-0.16932
Q9NP97	Dynein ligh	DYNLRB1 B	0.965785	0.906613	0.736818	0.432493	0.669448	0.845237	0.745635	0.937495	1.106679	0.867425

Q9NPA8	Transcripti	ENY2 DC6	0.741807	0.540081	1.280796	0.948926	1.330824	1.08231	1.012733	0.726624	1.463711	1.424411
Q9NPD3	Exosome c	EXOSC4 RR	0.837961	0.894258	0.14012	0.440194	0.58865	0.431043	0.42261	0.264006	0.833717	0.858249
Q9NPE3	H/ACA ribo	NOP10 NO	0.081098	0.369387	0.488903	0.144133	0.606901	0.704505	0.683025	0.714657	0.911569	1.228044
Q9NPF4	Probable t	OSGEP GCF	-0.44973	-0.39249	-0.36365	-0.35759	-0.20375	-0.47308	-0.38956	-0.31838	-0.28964	-0.06296
Q9NPF5	DNA methy	DMAP1 KIA	-0.1809	-0.06209	-0.74828	-0.61956	-0.27377	-0.67903	-0.50338	-0.83755	-0.92834	-0.51305
Q9NPG3	Ubinuclein	UBN1	0.187331	0.357362	0.67319	0.273451	0.7175	-0.00548	0.802963	0.498454	0.562791	0.622815
Q9NPH0	Lysophospl	ACP6 ACPL	-1.26546	-0.49246	-0.31221	-0.56937	-0.59991	-0.60145	-0.33865	-0.30485	-0.43084	-0.09134
Q9NPH2	Inositol-3-p	ISYNA1 INC	0.133088	0.27919	-0.0516	0.012491	-0.03767	0.356485	0.449163	0.494395	0.206137	0.738735
Q9NPI1	Bromodom	BRD7 BP75	-0.69515	-0.74348	0.310969	0.38813	0.174874	0.140481	-0.15656	0.212545	0.415037	0
Q9NPI6	mRNA-deca	DCP1A SMI	-0.46855	-0.10124	-0.64874	-0.9682	-0.61123	-0.33015	-0.86343	-0.63743	-0.43721	-0.38294
Q9NPQ8	Synembryn	RIC8A	-0.32684	-0.62314	-0.06439	-0.66438	-0.60115	-0.38466	-0.02191	-0.03091	-0.11036	-0.25387
Q9NQ29	Putative RN	LUC7L LUC	-0.5606	-0.35543	-1.88753	-1.4361	-0.86649	-0.81905	-0.63604	-0.49667	-0.58839	-0.71871
Q9NQ48	Leucine zip	LZTFL1	-3.25096	-1.91667	-1.72403	-1.7794	-2.47063	-1.58496	-1.41163	-1.76783	-1.61353	-1.3375
Q9NQ55	Suppressor	PPAN BXDC	-2.39411	-2.30039	-2.05822	-2.01732	-2.16687	-2.79891	-2.47859	-1.99567	-2.10756	-1.73726
Q9NQ89	Protein C12	C12orf4	-2.68536	-1.63656	-1.11353	-1.07731	-1.22487	-1.22422	-1.06274	-1.19592	-1.37474	-1.46609
Q9NQC1	E3 ubiquiti	JADE2 KIAA	-1.2309	-1.16976	-0.62419	-0.6946	-0.55876	-0.42706	-0.73266	-0.62988	-0.61874	-0.69671
Q9NQC3	Reticulon-4	RTN4 KIAA	-0.51205	-0.35755	-0.42354	-0.53093	-0.84899	-0.77627	-0.69513	-1.03749	-1.1597	-0.87802
Q9NQC7	Ubiquitin c	CYLD CYLD	-1.21193	-1.05576	0.956459	1.040206	-1.16473	-0.16653	-0.57572	-0.64556	-0.47279	-0.33388
Q9NQG5	Regulation	RPRD1B C2	-1.16369	-0.7042	-0.32371	-0.74084	-0.29193	-0.29847	-0.74211	-0.35735	-0.1035	0.135043
Q9NQG6	Mitochond	MIEF1 MID	-3.08746	-2.24598	-3.57719	-3.9386	-3.27798	-3.34395	-3.81762	-4.07039	-3.86507	-3.21945
Q9NQH7	Xaa-Pro am	XPNPEP3	-3.5025	-2.32193	-3.6734	-3.26053	-4.01495	-3.18641	-4.05209	-3.65535	-2.52947	-2.28264
Q9NQP4	Prefoldin s	PFDN4 PFD	0.059887	0.18763	0.164944	-0.28155	-0.14917	-0.1512	-0.03187	-0.04776	0.303315	0.040366
Q9NQR4	Omega-am	NIT2 CUA0	-0.1016	-0.11334	-0.50349	-0.06997	-0.77833	-0.92546	-0.20221	-0.29586	-0.19563	0.034852
Q9NQS1	Cell death	AVEN	-1.33066	-1.28402	-1.23539	-1.1186	-0.92811	-1.37908	-1.23507	-1.26303	-0.82961	-1.07969
Q9NQT4	Exosome c	EXOSC5 CM	0.812541	0.996096	0.915702	0.666263	0.800967	0.895185	0.486157	0.684498	1.344383	1.251185
Q9NQT5	Exosome c	EXOSC3 RR	0.214644	0.158325	0.28987	-0.45412	-0.04492	0.056419	0.316893	0.233991	0.552278	0.638678
Q9NQT8	Kinesin-like	KIF13B GAK	-0.48274	-0.40857	-0.33281	-0.5252	-0.65269	-0.46501	-0.49815	-0.43289	-0.71619	-0.63425
Q9NQW6	Anillin	ANLN	-1.60942	-1.50901	-1.4021	-1.40255	-1.45017	-1.1989	-3.05209	-1.65535	-1.38702	-1.71937
Q9NQW7	Xaa-Pro am	XPNPEP1 X	-0.51111	-0.50348	-1.17566	-0.8353	-1.26569	-1.16909	-1.22494	-1.15265	-0.92383	-1.13481
Q9NQX3	Gephyrin [I	GPHN GPH	-1.34023	-1.11353	-0.94642	-0.56937	-2.23565	-2.45487	-1.53964	-0.62855	-0.18057	-0.45391
Q9NQZ2	Something	UTP3 CRLZ	-3.11703	-2.30719	-1.8007	-1.45124	-2.07074	-2.34735	-1.19899	-1.77684	-1.47252	-1.55757
Q9NQZ5	StAR-relate	STARD7 GT	-0.68536	-0.42809	0.314109	0.247267	0.703868	0.871485	0.315776	0.207595	0.504163	1.015407
Q9NR09	Baculoviral	BIRC6 KIAA	-1.46855	-0.88033	-1.82239	-2.42905	-1.9855	-2.26042	-2.76545	-2.49837	-2.63938	-1.92696

Q9NR12	PDZ and LII	PDLIM7 EN	-0.35549	-0.23145	-0.32662	-0.49728	0.003613	0.258924	-1.3322	-1.08246	-1.22396	-1.00888
Q9NR28	Diablo hor	DIABLO SM	-0.17646	-0.01455	-0.2898	-0.25114	-0.23798	0.029208	-0.47259	-0.32932	-0.56417	-0.47631
Q9NR30	Nucleolar F	DDX21	0.553272	0.55143	-0.14454	-0.38434	0.133317	0.058884	0.162494	-0.1357	-0.10314	-0.03656
Q9NR31	GTP-bindin	SAR1A SAR	0.089482	0.519558	-0.97057	-0.64819	-0.52013	-0.49214	-0.82266	-0.51738	-0.90263	-0.6314
Q9NR45	Sialic acid s	NANS SAS	-0.30986	0.236803	-0.48515	-0.90559	0.213868	0.090032	-0.8493	-0.49079	-0.08579	-0.06428
Q9NR46	Endophilin-	SH3GLB2 K	-0.10692	0.320368	0.482639	0.397684	0.400087	0	0.186878	0.375867	0.606798	0.518551
Q9NR50	Translation	EIF2B3	-0.13971	-0.14931	-0.07165	-0.07615	-0.34373	-0.02137	-0.30683	-0.2545	0.092127	-0.02198
Q9NR56	Muscleblin	MBNL1 EXI	-0.0927	0.098224	0.469515	0.267697	0.270241	0.228156	0.330514	0.164517	0.658492	0.455403
Q9NRF9	DNA polym	POLE3 CHR	-1.87245	-1.75002	-1.88393	-2.23863	-2.13086	-2.15741	-2.63604	-2.451	-2.17525	-2.53405
Q9NRG0	Chromatin	CHRAC1 CH	-3.37126	-2.50901	-3.20945	-4.03814	NA	NA	-3.68012	-3.39232	-2.93546	-3.70487
Q9NRG9	Aladin (Adr	AAAS ADR/	-0.31739	0.038474	0.256521	0.319417	-0.269	0.268489	0.177861	-0.31907	-0.10764	0.008824
Q9NRL3	Striatin-4 (i	STRN4 ZIN	-0.53705	-0.7408	-0.5276	-0.64378	-0.56742	-0.79528	-0.59758	-0.83105	-0.72143	-0.38567
Q9NRN7	L-aminoadi	AASDHPPT	0.972461	1.301509	1.23349	1.190684	1.194503	0.932886	1.094068	1.053387	1.016408	1.041441
Q9NRR5	Ubiquilin-4	UBQLN4 C1	1.96782	1.856636	-1.47687	-1.08469	0.770775	1.222392	-1.24672	-1.51803	-1.34253	-1.62803
Q9NRR8	CDC42 sma	CDC42SE1 S	0.029995	0.226842	0.744743	0.516454	0.534513	0.540568	0.67423	0.709221	0.281771	-0.08663
Q9NRS6	Sorting nex	SNX15	-1.06193	-1.57719	-1.96153	-1.0126	-1.03006	-0.93587	-1.30666	-0.95491	-0.89107	-2.00888
Q9NRV9	Heme-bind	HEBP1 HBF	0.076726	0.27919	0.440162	0.299168	0.519028	0.343623	0.045324	-0.25676	-0.52947	-0.2143
Q9NRW3	DNA dC->d	APOBEC3C	0.107973	0.005773	-1.42818	-0.55436	0.348454	0.431339	-0.93376	-0.5854	-1.56621	-1.71945
Q9NRX1	RNA-bindir	PNO1	-0.89482	-0.73697	-1.18265	-1.52356	-1.07635	-1.03317	-0.782	-0.71989	-0.5025	-0.29888
Q9NRX4	14 kDa phc	PHPT1 PHP	-1.07727	-1.16459	-1.12164	-0.62632	-1.41703	-0.8037	-1.41977	-1.26205	-0.82251	-0.54028
Q9NRY5	Protein FAI	FAM114A2	-0.47354	-0.30179	-0.49246	-0.68326	-0.55552	-0.30968	-0.17955	-0.71989	-0.21847	-0.1738
Q9NRY6	Phospholip	PLSCR3	0.133088	0.09067	0.23349	0.344335	-0.16528	0.570316	0.06502	0.648429	-0.01898	-0.58054
Q9NS23	Ras associa	RASSF1 RD/	0.887099	0.874044	0.69149	0.439904	0.477372	0.58428	0.639812	0.541275	-0.10861	-0.05705
Q9NS69	Mitochond	TOMM22 T	-0.48543	-0.23217	-3.57719	-1.91475	-2.31025	-2.34129	-1.63176	-2.69188	-1.86303	-2.62803
Q9NS86	LanC-like p	LANCL2 GP	-2.16613	-1.55138	-1.50421	-1.24642	-2.07977	-3.14475	-2.60181	-2.78867	-1.55124	-1.45518
Q9NSD9	Phenylalan	FARSB FAR	-0.70499	-0.35974	-0.6159	-0.82116	-0.63413	-0.83494	-0.69876	-0.80997	-0.82298	-0.42812
Q9NSE4	Isoleucine-	IARS2	0.436099	0.67319	0.742414	0.685891	0.405712	0.850424	0.667804	0.774264	0.734134	0.549117
Q9NSI8	SAM doma	SAMSN1 H/	0.693673	0.699181	0.719773	0.696649	0.428935	0.197563	0.479589	0.423113	0.073566	0.351192
Q9NSU2	Three-prim	TREX1	-1.05354	-1.17418	-1.19384	-0.96254	-2.01998	-2.1375	-1.1938	-1.99619	-1.52441	-0.8045
Q9NT62	Ubiquitin-li	ATG3 APG3	-0.03086	-0.22832	1.257792	1.017911	1.066228	1.058079	-0.03126	0.094093	-0.21586	-0.47901
Q9NTI5	Sister chroi	PDS5B APR	-0.18746	-0.24136	-0.03939	-0.35444	-0.01	-0.34975	-0.38482	-0.04834	-0.30887	-0.12152
Q9NTJ5	Phosphatid	SACM1L KI/	-0.00607	-0.07605	-0.13914	-0.35846	-0.88567	-0.635	-0.5934	-0.57214	-1.18014	-0.48217
Q9NTK5	Obg-like AT	OLA1 GTPB	1.200784	1.205271	1.297546	1.226702	1.114267	1.090448	1.193604	1.218979	1.461913	1.207563

Q9NTM9	Copper hor	CUTC CGI-3	-0.60853	-0.18333	-0.01573	-0.08963	-0.36205	-0.43068	-0.38801	-0.64817	-0.11483	-0.10555
Q9NTW7	Zinc finger	ZFP64 ZNF3	-0.03063	0.199938	0.540081	0.533888	0.460783	0.736966	0.426503	0.131245	0.540274	0.499895
Q9NTX5	Ethylmalon	ECHDC1	-0.18258	-0.01421	-0.13918	-0.06994	-0.25538	0.019105	-0.22681	-0.09567	0.114583	0.099016
Q9NTZ6	RNA-bindir	RBM12 KIA	0.805021	0.821138	0.62221	0.293862	0.416368	0.365683	0.973342	0.668287	0.903647	0.918028
Q9NU22	Midasin (D	MDN1 KIAA	-0.43241	-0.44792	-0.42464	-0.47811	-0.23958	-0.36534	-0.35947	-0.66763	-0.27761	-0.59579
Q9NUB1	Acetyl-coer	ACSS1 ACA	0.644721	0.769357	-0.21624	-0.01638	-0.56393	0.064358	0.214128	-0.04123	-0.20044	-0.36118
Q9NUG6	p53 and DN	PDRG1 C20	1.334001	1.066671	1.206711	1.392915	1.572515	1.431339	1.323973	1.342392	1.804658	1.598178
Q9NUJ1	Palmitoyl-p	ABHD10	0.842312	0.73925	0.407659	0.770725	0.79969	0.856564	0.670788	0.522164	0.746301	0.663928
Q9NUL3	Double-stra	STAU2	0.40439	0.196539	0.445898	0.568138	0.498119	0.720477	0.500694	0.5025	0.473931	0.442435
Q9NUL5	Shiftless an	SHFL C19or	0.774067	0.87376	0.702238	0.88922	1.128008	1.013598	1.052741	-0.2978	1.310568	1.253043
Q9NUP1	Biogenesis	BLOC1S4 C	-0.41908	-0.48153	-0.68589	-0.39428	-0.47063	-0.42968	-0.53222	-0.36995	-0.05282	0.186547
Q9NUP9	Protein lin-	LIN7C MAL	0.788177	0.875886	0.850161	0.916061	1.213868	0.769387	0.831992	0.880701	1.030325	0.984427
Q9NUQ2	1-acyl-sn-g	AGPAT5	0.138605	0.275973	0.003894	-0.4361	0.007417	0.160177	0.239827	0.289507	-0.25739	-0.18382
Q9NUQ3	Gamma-ta:	TXLNG CXo	-2.16587	-1.73334	-1.3082	-1.94889	-1.29659	-0.85173	-1.1563	-1.84067	-1.51693	-1.01918
Q9NUQ8	ATP-bindin	ABCF3	-1.59533	-1.20367	-0.43335	-0.24558	-0.33313	-0.69567	0.079617	-0.36257	-1.08746	-0.40621
Q9NUQ9	CYFIP-relat	CYRIB CYRI	0.410263	0.638291	0.562005	0.358409	0.514459	0.466725	0.495003	0.40929	0.518849	0.554364
Q9NUU7	ATP-depen	DDX19A DI	-0.90136	-1.02936	-0.9707	-0.75662	-1.13427	-0.69293	-0.92789	-0.92925	-0.31721	-0.04541
Q9NUV9	GTPase IM/	GIMAP4 IA	-0.44476	-0.05335	0.697234	0.683672	0.662466	0.196638	0.600269	0.474687	0.646051	0.690739
Q9NUW8	Tyrosyl-DN	TDP1	-2.68536	-1.42286	-0.26929	-0.69574	-0.8055	-0.45194	-0.75585	-0.59358	-0.33852	-0.27727
Q9NV06	DDB1- and	DCAF13 WI	-0.9523	-1.7112	-1.6734	-0.83426	-1.89947	-1.16175	-1.4957	-0.43812	-0.89984	-1.03585
Q9NV70	Exocyst cor	EXOC1 SEC	0.316743	0.326352	0.61329	0.434947	0.192479	0.456813	0.263438	0.13422	0.366467	0.246179
Q9NVA2	Septin-11	SEPTIN11 S	-0.92521	-0.55578	-0.30348	-0.28489	-0.53126	-0.51675	-0.52321	-0.38339	-0.871	-0.97228
Q9NVC6	Mediator o	MED17 ARC	-1.62803	-1.5541	-0.78991	-0.73697	-0.33313	-0.26303	-0.85415	-0.13808	-0.07251	0.0859
Q9NVE7	4'-phospho	PANK4	-0.50899	-0.21248	-0.47889	-0.56783	-0.59482	-0.80244	-0.3405	-0.71564	-0.63934	-0.5997
Q9NVF7	F-box only	FBXO28 CE	-1.64689	-1.62449	-1.5427	-2.71621	-3.42999	-2.02203	-1.6477	-1.71049	-2.06756	-1.51584
Q9NVH1	DnaJ homo	DNAJC11	-3.03684	-3.53138	NA	NA	-3.69302	-3.1375	-3.81762	-3.97728	-4.00946	-2.08194
Q9NVH2	Integrator (	INTS7 C1or	-2.72489	-2.02359	-1.68589	-1.96285	-1.22487	-1.52083	-1.53964	-1.93289	-1.91754	-1.64829
Q9NVI7	ATPase fan	ATAD3A	-0.93772	-0.77284	-1.14265	-1.38559	-1.51307	-1.09869	-1.30866	-1.22338	-1.46174	-1.60393
Q9NVK5	FGFR1 onco	FGFR1OP2	-0.08104	-0.16506	0.566287	0.536136	0.423333	0.383118	0.399926	0.244281	0.57411	0.548482
Q9NVM6	DnaJ homo	DNAJC17	-0.33915	0.040166	1.148893	0.734756	0.778339	0.982744	1.133999	1.047607	0.916401	1.160057
Q9NVM9	Integrator (	INTS13 ASU	0.275107	0.375509	0.437285	0.407175	0.48755	0.533035	0.471473	0.680932	0.40439	0.375786
Q9NVP1	ATP-depen	DDX18 cPE	0.794071	0.762104	0.578401	0.267857	1.136441	1.194011	0.936732	0.747705	0.726827	0.70384
Q9NVR2	Integrator (	INTS10 C8o	-0.04307	0.199938	-0.02756	0.15451	0.207442	-0.07861	-0.13393	-0.07039	-0.18593	0.228701

Q9NVS2	39S ribosor	MRPS18A	-0.19438	0.007777	-0.13906	-0.0973	-0.51245	-0.38618	0.155069	-0.16993	-0.09248	-0.01781
Q9NVU0	DNA-direct	POLR3E KIA	-0.67565	-0.50901	-0.53703	-0.89129	-0.57755	-0.33015	-0.41845	-0.4459	-0.45003	-0.9141
Q9NVU7	Protein SD	SDAD1 NU	-0.65946	0.063029	-0.18411	-0.27006	-0.5518	-0.48312	-0.37021	-1.17884	-1.03333	-0.88855
Q9NVV4	Poly(A) RN	MTPAP PAI	0.012073	0.445898	0.288798	0.879024	-0.24236	0.360747	-0.3322	0.241889	-0.50919	0.213514
Q9NVW2	E3 ubiquiti	RLIM RNF1	-1.37126	-1.23676	-1.03154	-0.82312	-1.31451	-0.85457	-0.74723	-0.9886	-1.31487	-0.50324
Q9NVX2	Notchless p	NLE1 HUSS	-0.59104	-0.32193	-1.10512	-1.10402	-0.5231	-0.90484	-0.9301	-0.922	0.13493	0.348239
Q9NVZ3	Adaptin ea	NECAP2	-0.411	0.155119	0.108863	-0.13813	-0.34955	-0.10776	-0.38466	-0.1571	0.338802	0.269657
Q9NW13	RNA-bindir	RBM28	-0.54609	-0.14768	-0.66944	-0.78992	-0.03767	-0.20519	-0.40484	-0.36257	-0.37642	-0.78328
Q9NW64	Pre-mRNA-	RBM22 ZC3	-2.28766	-0.45508	-1.59361	-0.85128	-2.01495	-1.76542	-1.26481	-1.1981	-1.6237	-1.34185
Q9NW82	WD repeat	WDR70	-0.88359	-0.80346	-0.00782	-0.05759	-0.09212	0.105353	-0.46007	-0.14439	0.082462	0.309483
Q9NWB6	Arginine ar	ARGLU1	-0.05561	0.137504	0.008902	0.126054	-0.07575	-0.35318	-0.30666	-0.04654	-0.16116	0.429349
Q9NWH9	SAFB-like t	SLTM MET	0.326751	0.226842	0.272944	0.088198	0.128008	0.386058	0.18294	0.465273	0.578663	0.485998
Q9NWQ8	Phosphopr	PAG1 CBP I	-0.92494	-1.37324	-0.96153	-1.29562	-1.49207	-1.084	-1.33392	-1.8051	-1.86507	-1.47836
Q9NWT8	Aurora kina	AURKAIP1	-0.88359	-0.98449	-0.47069	-1.02531	-1.03006	-0.92545	-0.73016	-1.19592	0.540274	0.190431
Q9NWU1	3-oxoacyl-[	OXSM	0.491797	0.631617	0.085185	0.139528	0.355683	0.445416	0.400912	0.287484	0.520377	0.46668
Q9NWU2	Glucose-in	GID8 C20o	-2.68536	-1.90202	-1.94642	-1.8011	-1.55552	-1.87447	-1.782	-1.93289	-2.30319	-2.1589
Q9NWU5	39S ribosor	MRPL22 M	-0.88359	-1.07195	-0.53138	-0.32979	-1.45017	-0.80599	-0.83577	-0.90046	-0.92647	-1.32636
Q9NWV4	CXXC motif	CZIB C1orf	0.388915	0.345135	0.323487	0.209792	0.405712	0.364997	0.274077	0.39776	0.357322	0.665292
Q9NWV8	BRISC and I	BABAM1 C	-1.73724	-1.77984	0.01165	-0.25303	-0.23505	-0.12002	-0.19704	-0.07641	0.004707	-0.27191
Q9NWW6	Nicotinami	NMRK1 C9	-0.47697	-0.43862	-0.25061	-0.49676	-0.54463	-0.1315	-0.56212	-0.56789	-0.66493	-0.54783
Q9NWY4	Histone PA	HPF1 C4orf	0.319501	0.419903	0.637987	0.354933	0.293209	0.671273	0.178775	0.473507	0.680751	0.36306
Q9NWZ3	Interleukin	IRAK4	0.568707	0.723647	0.90533	0.875609	0.943119	0.82905	0.727727	0.85561	0.553184	0.442435
Q9NX14	NADH deh	NDUFB11 U	0.956931	0.744743	0.163619	0.383329	-0.19052	0.832123	0.776328	0.251539	-0.4635	-0.59162
Q9NX24	H/ACA ribo	NHP2 NOL	-0.1706	0.077363	-0.06141	-0.1632	-0.18063	-0.04027	0.047942	-0.0374	0.111212	0.021168
Q9NX40	OCIA doma	OCIAD1 AS	0.356256	0.105544	0.054168	-0.11539	-0.15132	0.076988	-0.08627	-0.07624	-0.24731	-0.2466
Q9NX46	ADP-ribose	ADPRS ADP	-0.42043	-0.48457	-0.30421	-0.81855	-0.30875	-0.07514	-0.35018	-0.09385	0.188612	-0.11033
Q9NX55	Huntingtin-	HYPK C15o	-1.35566	-0.88753	-0.50164	-0.58838	-0.17864	-0.48758	-0.27323	-0.34947	-0.08559	-0.00385
Q9NX58	Cell growth	LYAR PNAS	0.529689	0.121019	0.282175	0.103019	0.453941	0.049958	0.230583	0.136678	0.036266	-0.1277
Q9NX63	MICOS com	CHCHD3 M	0.431199	0.708609	0.645302	0.593302	0.638492	0.533311	0.213595	0.577289	0.706076	0.650052
Q9NX70	Mediator o	MED29 IXL	-1.57289	-1.11353	-1.24598	-1.7794	-1.66918	-1.58496	-1.12832	-1.46949	-1.81444	-1.16882
Q9NX74	tRNA-dihyd	DUS2 DUS2	1.079597	1.206711	1.536053	1.344335	1.779466	1.337149	1.65811	1.592576	1.411497	1.550629
Q9NX76	CKLF-like M	CMTM6 CK	2.411589	2.307106	1.325365	0.807042	0.940111	1.022137	1.20707	0.683939	0.947374	0.696811
Q9NXA8	NAD-deper	SIRT5 SIR2L	-0.44361	-0.42286	-0.43335	-0.26806	-0.34252	-0.54455	-0.44606	-0.34792	-0.21299	-0.83303

Q9NXC5	GATOR con	MIOS	-0.75876	-0.8329	-0.82206	-1.04784	-0.69561	-0.63227	-0.98547	-1.01994	-0.71346	-0.6623
Q9NXE4	Sphingomy	SMPD4 KIA	-0.28011	0.23349	-1.92605	-1.35792	-0.39046	-0.95693	-1.6453	-1.99223	-1.58496	-0.80957
Q9NXF1	Testis-expr	TEX10 L18	-1.37126	-1.28345	-0.79667	-1.67557	-0.79256	-1.22422	-0.95964	-1.04654	-1	-1.21945
Q9NXG2	THUMP do	THUMPD1	-0.64748	-0.55483	-0.65965	-0.57956	-0.33313	-0.54455	-0.31937	-0.39232	-0.51901	-0.07674
Q9NXH9	tRNA (guar	TRMT1	-1.12663	-1.53138	-0.82402	-0.98751	-1.07635	-1.5366	-0.80863	-0.79737	-1.67243	-1.11032
Q9NXR1	Nuclear dis	NDE1 NUD	0.956931	0.259781	0.685364	0.816288	0.18799	0.686925	0.356304	0.51056	0.121991	1.681939
Q9NXV6	CDKN2A-in	CDKN2AIP	-0.45216	-0.37559	-0.13291	-0.21805	-0.06853	-0.09603	-0.34839	-0.30221	-0.27391	-0.04975
Q9NY12	H/ACA ribo	GAR1 NOL/	1.508194	1.472839	0.58756	0.885829	0.935363	1.19162	1.079617	1.328782	1.039528	1.008824
Q9NY27	Serine/thre	PPP4R2 SBI	-0.05258	-0.09419	0.234502	0.321918	0.527307	0.427268	0.130952	0.223968	0.06108	0.062657
Q9NY33	Dipeptidyl	DPP3	0.275307	0.33504	-0.44392	-0.46179	-0.51245	-0.60977	-0.37137	-0.31194	-0.62076	-0.52855
Q9NY35	Claudin dor	CLDND1 C3	-0.12003	-0.90202	-1.4021	-0.87971	-1.10806	-0.33703	-1	-1.10692	-1.88235	-1.69052
Q9NY93	Probable A	DDX56 DD)	-1.89623	-1.30758	-1.54525	-2.23833	-2.05618	-2.07903	-1.46505	-1.41886	-1.57096	-1.76416
Q9NYB0	Telomeric r	TERF2IP DF	-0.22239	-0.33171	0.133954	0.018696	0.029444	0.130532	-0.16227	0.078003	-0.1541	0.302324
Q9NYF8	Bcl-2-assoc	BCLAF1 BTI	-0.47838	-0.20619	0.240108	0.162141	0.255598	0.304334	0.093423	0.138649	0.467598	0.418891
Q9NYH9	U3 small nu	UTP6 C17o	0.408652	0.366464	-0.13906	-0.41923	-0.27798	0.448127	0.079617	0.126894	0.254365	0.056413
Q9NYJ1	Cytochrom	COA4 CHCH	-2.0025	-1.96859	-1.25151	-2.03584	-0.63413	-0.35789	-1.71134	-1.65546	-1.79805	-1.39754
Q9NYL9	Tropomodu	TMOD3	0.665652	0.656102	0.743567	0.783811	0.957863	0.647478	0.567766	0.742118	1.107953	0.887427
Q9NYM9	BET1-like p	BET1L GS1!	0.020044	-0.12744	-0.33692	-0.27512	-0.83033	-0.3052	-0.35385	-0.42631	-0.2378	-0.16693
Q9NYU2	UDP-glucos	UGGT1 GT	0.397341	0.556081	0.249978	0.38813	0.043943	0.110424	0.464075	-0.04654	-0.06263	-0.28264
Q9NYV4	Cyclin-depe	CDK12 CRK	-1.43	-1.1582	-2.13906	-1.30633	-1.39947	-0.65423	-1.41163	-1.34792	-2.06756	-1.22979
Q9NYZ3	G2 and S pl	GTSE1	-1.59104	-3.24598	-2.83094	-1.86821	NA	NA	-3.61599	-3.51785	-3.73383	-0.72668
Q9NZ01	Very-long-c	TECR GPSN	1.552936	1.495214	1.389662	1.454055	1.415368	1.44368	1.484096	1.386851	1.182672	1.290287
Q9NZ08	Endoplasm	ERAP1 APP	0.573139	0.862763	0.165976	0.104728	-0.40011	-0.4975	-0.18592	-0.37197	0.163465	0.120956
Q9NZ09	Ubiquitin-a	UBAP1 NAC	-0.9466	-0.93649	-1.09432	-0.81312	-1.31915	-1.04894	-1.79085	-1.48365	-1.07594	-0.84172
Q9NZ32	Actin-relate	ACTR10 AC	-3.25096	-1.99222	-2.46529	-1.86821	-3.35199	-3.23704	-2.16227	-3.27684	-2.25739	-1.64829
Q9NZ45	CDGSH iror	CISD1 C10c	1.039396	1.124004	1.356186	1.104119	1.254868	1.022495	1.344339	1.234732	1.412426	1.295836
Q9NZ63	Telomere l	C9orf78 HC	1.121991	1.309396	0.28017	0.068181	0.573794	0.516691	0.438352	0.571602	0.790908	0.943929
Q9NZA1	Chloride in	CLIC5	-1.46855	-1.02359	-0.53703	-0.25303	-1.03006	-1.52083	-0.59266	-0.6644	-2.3505	-1.27191
Q9NZB2	Constitutiv	FAM120A (	0.14358	0.190814	0.794288	0.597226	0.699523	0.501426	0.548036	0.599948	0.684113	0.4129
Q9NZD2	Glycolipid t	GLTP	-1.22302	-0.84658	-0.73355	-0.59333	-1.31262	-1.04303	-0.91125	-0.74186	-1.01059	-0.91311
Q9NZI7	Upstream-l	UBP1 LBP1	0.077538	0.125033	0.14252	0.584738	-0.04639	0.184417	-0.01296	0.064252	0.070154	-0.01649
Q9NZJ7	Mitochond	MTCH1 PS/	-1.04933	-0.73697	-0.63656	-0.30633	-0.16528	-0.66069	0.010196	-0.31194	-0.60634	-0.42392
Q9NZJ9	Diphospho	NUDT4 DIP	-1.07464	-1.10113	-1.82702	-1.41495	-1.25415	-1.56866	-1.49124	-1.61588	-1.69727	-1.7017

Q9NZL4	Hsp70-binc	HSPBP1 HS	-1.28857	-0.93504	-1.16044	-0.83848	-1.96477	-1.65044	-2.1477	-1.675	-1.15548	-1.12338
Q9NZL9	Methionine	MAT2B TGI	-0.9523	-1.03953	-0.85896	0.012491	-0.88567	-0.48981	-1	-0.79737	-0.87368	-0.42392
Q9NZM3	Intersectin	ITSN2 KIAA	-0.47697	-0.65486	-0.33662	-0.63604	-0.7051	-0.98911	-0.84493	-0.46949	-0.38702	-0.50952
Q9NZN8	CCR4-NOT	CNOT2 CDC	-2.53727	-1.33171	-1.5427	-1.71621	-1.47063	-0.95693	-1.53964	-1.23581	-1.4892	-1.40621
Q9NZQ3	NCK-intera	NCKIPSD AI	-3.43539	-2.13906	-2.46529	-3.14505	-4.01495	-3.0902	-2.11718	-2.65535	-3.73383	-2.41799
Q9NZT2	Opioid grov	OGFR	0.03592	0.269518	0.027039	0.263034	0.007417	0.048363	0.218001	-0.10692	0.037233	0.017594
Q9NZZ3	Charged m	CHMP5 C9c	-0.08178	-0.32226	0.050807	0.020037	0.129387	0.054109	-0.01971	0.117941	0.375685	0.572723
Q9P013	Spliceosom	CWC15 C11	-0.60989	-0.47069	-0.44392	-0.20887	-0.39046	-0.18641	-0.1452	-0.12553	-0.54714	-0.2717
Q9P016	Thymocyte	THYN1 THY	0.902844	0.620143	0.280438	0.39087	0.795319	0.617885	0.523439	0.337548	0.731112	0.726135
Q9P031	Thyroid tra	CCDC59 BR	-0.53993	-0.70868	-0.53319	-0.65263	-0.56906	-0.50576	-0.66802	-0.86265	-0.60004	-0.31958
Q9P032	NADH dehy	NDUFAF4 C	1.102362	1.248338	0.92805	1.105795	1.294905	1.06413	-0.9175	-1.04907	-1.38329	-1.40474
Q9P0J0	NADH dehy	NDUFA13 C	2.909313	3.074561	0.236803	0.746967	0.867693	0.410933	1.65811	0.833292	-0.10764	0.114796
Q9P0J1	[Pyruvate c	PDP1 PDP I	-1.91265	-0.7276	-0.7279	-0.89148	-1.58665	-0.70131	-1.13176	-0.99277	-1.12914	-1.3135
Q9P0L0	Vesicle-ass	VAPA VAP3	1.269528	1.201569	1.515742	1.492132	1.224729	1.106238	1.328054	1.064275	1.40439	1.417801
Q9P0P8	Mitochond	MTRES1 C6	-1.80735	-1.41244	-1.27399	-1.11757	-1.55552	-1	-0.88216	-1.18286	-0.9906	-1.3375
Q9P0U4	CXXC-type	CXXC1 CFP1	-1.06449	-1.1305	-1.10084	-1.14026	-0.86319	-1.00669	-1.35827	-1.0936	-0.25739	-0.4013
Q9P107	GEM-intera	GMIP	-0.57906	-0.64874	-0.27083	-0.24558	-0.68105	-0.28685	-0.38565	-0.58496	-0.57104	-0.65958
Q9P1F3	Costars fan	ABRACL C6	1.091024	1.072245	1.627228	1.707179	1.626499	1.475242	1.487983	1.63743	1.717027	1.523177
Q9P1W8	Signal-regu	SIRPG SIRP	-3.29376	-3.5893	-2.24326	-2.14452	-1.10806	-0.31647	-2.82898	-2.17154	-0.72601	-1.08194
Q9P1Y6	PHD and RI	PHRF1 KIAA	-0.97281	-1.34699	-0.15962	-0.33441	-0.16598	-0.25332	0.045247	-0.25626	-0.13784	-0.14478
Q9P1Z0	Zinc finger	ZBTB4 KIAA	-3.72489	-2.36146	-2.26459	-2.9386	-2.95606	-2.87447	-3.23266	-3.80735	-2.37474	-1.69052
Q9P1Z2	Calcium-bir	CALCOCO1	-3.03684	-3.24598	-3.27058	-3.61577	-3.59991	-3.04439	-2.80511	-3.04829	-2.69146	-3.62726
Q9P219	Protein Da	CCDC88C D	-0.51277	-0.52669	-0.25385	-0.14347	-0.15118	-0.29578	-0.08126	-0.07501	-1.18057	-1.0726
Q9P241	Probable p	ATP10D AT	-1.18057	-1.50901	-1.31221	-0.87971	-1.04532	-1.58496	-1.34514	-1.33342	-1.00946	-1.34873
Q9P246	Stromal int	STIM2 KIAA	-0.94095	-0.78632	-0.57228	-0.92643	-1.49199	-1.43632	-1.88539	-1.73085	-1.98416	-1.92655
Q9P253	Vacuolar pı	VPS18 KIAA	-3.13993	-2.28345	-2.34156	-1.89129	-0.44337	-0.44686	-2.23266	-1.97728	-2.14886	-3.34873
Q9P258	Protein RC(	RCC2 KIAA1	0.275107	0.116077	0.052573	0.304257	0.158137	0.112777	0.360295	0.432111	0.297201	0.234295
Q9P265	Disco-inter	DIP2B KIAA	-0.28749	-0.51457	-1.03953	-0.59756	-0.19052	-1.22422	-0.6477	-0.77761	-0.50919	-0.29888
Q9P270	SLAIN moti	SLAIN2 KIA	-3.02916	-2.574	-2.26472	-2.10409	-2.33617	-2.17156	-2.53972	-3.2077	-2.46491	-2.3551
Q9P275	Ubiquitin c	USP36 KIAA	-0.89262	-1.02716	-0.37406	-0.79021	-0.61929	-0.72168	-0.84375	-0.66929	-0.89178	-0.71515
Q9P289	Serine/thre	STK26 MAS	0.10726	0.349011	-1.11353	-1.35364	-0.70894	-0.25092	-1.55459	-0.75811	-0.57791	-1.55431
Q9P2A4	ABI gene fa	ABI3 NESH	0.520353	0.611609	0.736344	0.685396	0.36124	0.514082	0.724409	0.850754	0.680295	0.577531
Q9P2D3	HEAT repea	HEATR5B K	0.616935	0.90741	1.021288	1.058392	0.875821	1.140481	1.002556	0.659345	0.920032	0.770213

Q9P2D7	Dynein hea	DNAH1 DH	-3.30986	-3.24598	-3.44392	-2.98751	-3.69302	-3.79647	-3.23266	-3.51785	NA	NA
Q9P2E9	Ribosome-l	RRBP1 KIA/	-0.39497	-0.38259	-0.40153	-0.46179	-0.56649	-0.26303	-0.39808	-0.49755	-0.5507	-0.67513
Q9P2I0	Cleavage a	CPSF2 CPSF	-1.15335	-1.41244	-1.11353	-1.47045	-0.9417	-1.44448	-0.30786	0.005612	-1.39941	-0.93934
Q9P2J5	Leucine--tR	LARS1 KIAA	-0.56391	-0.6323	-0.65931	-0.9148	-0.56045	-0.65855	-1.04898	-0.53519	-0.53599	-0.55221
Q9P2K3	REST corep	RCOR3 KIA.	0.116409	0.220163	0.23349	0.349267	0.394441	0.402689	0.507907	0.486244	0.581816	0.614168
Q9P2K5	Myelin exp	MYEF2 KIA.	-0.06193	0.038474	-0.62449	-0.25303	-0.49139	-0.28951	-0.25081	-0.09464	-0.40564	-0.53495
Q9P2N5	RNA-bindir	RBM27 KIA	-0.62017	-0.96841	-0.58331	-0.75491	-0.76902	-0.58701	-0.67527	-0.67825	-0.84461	-0.81737
Q9P2N6	KAT8 regul	KANSL3 KI/	-0.04933	-0.18265	-0.07605	0.031027	-0.30529	-0.28284	-0.10613	-0.11309	-0.04307	0.102483
Q9P2P6	StAR-relate	STARD9 KI/	0.488887	0.345135	0.742414	0.875609	0.748815	0.595851	0.557416	0.506536	0.193823	0.295128
Q9P2R3	Rabankyrin	ANKFY1 AN	-0.38702	-0.1305	-0.68589	-0.62632	-0.14079	-0.60145	-0.45305	-0.58496	-0.91754	-0.54783
Q9P2R6	Arginine-gl	RERE ARG /	-1.92627	-1.54331	-1.3597	-1.2075	-1.12368	-0.99878	-1.1445	-1.1502	-1.07739	-1.24248
Q9P2R7	Succinate--	SUCLA2	0.254929	0.21005	0.108863	-0.23526	-0.05302	0.435386	0.252766	0.490326	0.234465	-0.13927
Q9P2T1	GMP reduc	GMPR2	0.280108	0.23017	-0.30738	0.097024	0.439006	0.277534	0.26559	0.289507	0.069042	-0.22979
Q9P2W9	Syntaxin-1	STX18 GIG	-0.94455	-0.60643	-0.62517	-0.81506	-1.39149	-0.93409	-0.82937	-1.02805	-1.19116	-0.83355
Q9UBB4	Ataxin-10 (	ATXN10 SC	-0.85308	-0.94127	-1.30513	-1.3305	-1.91511	-1.48525	-1.30345	-0.74345	-1.56042	-1.33245
Q9UBB5	Methyl-Cp	MBD2	0.138605	0.09067	0.133954	0.273451	0.128862	0.222392	0.209007	-0.34066	-0.14365	0.060663
Q9UBB9	Tuftelin-int	TFIP11 STIF	-0.90426	-1.04617	-0.87317	-0.60709	-1	-1.0902	-1.24473	-1.2091	-1.10764	-0.84093
Q9UBC2	Epidermal	EPS15L1 EF	-1.27558	-0.90441	-0.29845	0.016127	-0.57703	-0.97618	-0.90213	-1.03164	-1.15755	-1.34972
Q9UBE0	SUMO-acti	SAE1 AOS1	-0.48543	-0.05971	-0.39296	-0.37788	0.014797	0.203533	0.050273	0.011405	-0.07251	-0.31531
Q9UBF2	Coatomer s	COPG2	-0.22315	-0.64049	-0.23316	-0.70436	-0.76647	-0.54163	-0.51317	-0.35716	-0.63062	-0.54406
Q9UBI9	Headcase p	HECA HDC	-1.18589	-1.33382	-1.35882	-1.6865	-2.81499	-1.30805	-1.36178	-2.07744	-1.36691	-1.55484
Q9UBL3	Set1/Ash2	ASH2L ASH	-0.29579	0.204583	-0.37784	-0.61379	-0.50192	0.11516	0.048418	-0.84352	-0.56111	0.070363
Q9UBN7	Histone de	HDAC6 KIA	-0.30986	-0.43862	-0.31706	-0.36976	-0.62263	-0.52083	-0.40484	-0.40742	-0.89984	-0.7414
Q9UBQ0	Vacuolar p	VPS29 DC1	1.335154	1.270017	1.089166	0.909397	1.120149	1.190423	1.076522	0.849167	1.201155	1.035965
Q9UBQ5	Eukaryotic	EIF3K EIF3S	-0.06594	-0.05849	-0.03121	-0.09572	-0.19428	-0.27572	-0.32719	-0.24048	-0.25832	0.504791
Q9UBQ7	Glyoxylate	GRHPR GLX	-1.49497	-0.66292	-0.4206	-0.38008	-0.67785	-0.51059	-0.49195	-0.53684	-0.22122	-0.20932
Q9UBS0	Ribosomal	RPS6KB2 S	0.097503	-0.04382	-0.89237	-0.69385	-0.77439	-0.00917	-0.92191	-0.65818	-0.37963	-0.17721
Q9UBS4	DnaJ homo	DNAJB11 E	-0.46461	-0.36719	-1.52015	-0.91475	-2.41901	-1.50258	-1.31937	-1.83169	-1.86507	-1.8893
Q9UBS8	E3 ubiquiti	RNF14 ARA	-1.04933	-1.02359	-0.50348	-0.92663	-1.03006	-1.01097	-1.25691	-1.26303	-0.97199	-0.55431
Q9UBT2	SUMO-acti	UBA2 SAE2	1.19304	1.154401	1.253279	1.149944	1.079868	1.400621	1.424598	1.240128	1.291928	1.230588
Q9UBU8	Mortality f	MORF4L1 M	-0.88559	-1.18002	-1.4761	-1.40255	-0.16598	-0.64625	-0.74408	-0.50793	-1.79796	-2.0268
Q9UBU9	Nuclear RN	NXF1 TAP	-1.57289	-1	-1.2276	-0.72655	-1.12418	-0.90484	-1.08427	-1.46949	-1.28011	-1.00888
Q9UBV8	Peflin (PEF	PEF1 ABP3	-1.15708	-0.20168	-0.25802	-0.31489	-0.21688	-0.27814	0.010428	0.014351	-0.04314	-0.049

Q9UBW5	Bridging int	BIN2 BRAP:	0.26	0.216811	-0.68046	-0.20163	-0.07397	-0.37197	-0.34142	-0.29078	-0.62572	-0.20874
Q9UBW7	Zinc finger	ZMYM2 FIN	-1.82873	-3.57719	-2.26459	-2.63604	-3.07635	-3.34395	-3.05209	-2.93289	-3.1281	-1.73402
Q9UBW8	COP9 signa	COPS7A CS	-1.85042	-1.77649	-1.7112	-2.84549	-2.47063	-1.635	-1.71329	-1.27684	-1.28011	-1.77887
Q9UDY8	Mucosa-as:	MALT1 MLT	0.187331	0.363436	-0.20046	-0.15899	-0.44004	-0.54455	-0.5175	-0.56789	-0.5639	-0.54783
Q9UEE9	Craniofacia	CFDP1 BCN	-0.06827	-0.40726	-0.09259	0.006259	0.161638	0.084889	-0.51019	0.033947	0.238467	0.409495
Q9UER7	Death dom	DAXX BING	-1.18748	-1.38339	-1.01657	-1.09819	-1.24236	-0.83494	-1.18536	-1.035	-1.10372	-0.88064
Q9UEW8	STE20/SPS:	STK39 SPAI	0.475295	0.449437	0.154338	0.276906	0.087301	0.874401	0.290774	0.244178	0.250592	-0.07637
Q9UEY8	Gamma-ad	ADD3 ADDI	0.281342	0.257097	0.534999	0.64235	0.843031	0.676373	0.990996	0.995625	1.020461	0.997786
Q9UFC0	Leucine-ric	LRWD1 CEI	-1.55497	-1.33171	-1.35147	-1.17306	-1.29613	-1.83494	-1.74723	-1.36257	-0.98126	-1.14906
Q9UFG5	UPF0449 p	C19orf25	-3.5025	-2.50901	-2.03953	-1.59756	-2.59991	-2.83494	-3.68012	-3.22239	-2.39941	-1.74882
Q9UFW8	CGG triplet	CGGBP1 CC	-0.55031	-0.83789	-0.25526	-0.57721	-0.5231	-0.5366	-0.43219	-0.61096	-0.14365	-0.23038
Q9UG22	GTPase IM	GIMAP2 IM	-0.28018	-0.25751	-0.42726	-0.42417	-1.13501	-0.66935	-0.63971	-0.71989	-1.07748	-1.04495
Q9UG63	ATP-bindin	ABCF2 HUS	0.213707	0.310969	0.118692	-0.09823	0.245577	0.223384	0.244153	0.419325	0.189695	-0.10721
Q9UGI8	Testin (TES	TES	1.082462	0.984318	1.025851	1.209792	1.04885	1.172352	1.057665	1.123382	1.22037	1.130475
Q9UGJ1	Gamma-tul	TUBGCP4 7	-0.75527	-0.45457	-1.88825	-1.94585	-2.06754	-2.26758	-2.17381	-2.37255	-0.20211	-0.27191
Q9UGM6	Tryptophar	WARS2	-0.70763	-0.65193	-0.30562	-0.34055	-0.91688	-0.58171	-1.04934	-0.96193	-0.69568	-0.60943
Q9UGN4	CMRF35-lik	CD300A CN	0.076726	0.186295	0.332805	0.257798	-0.19052	0.208271	0.074768	0.039528	-0.50919	-0.12956
Q9UGP4	LIM domaiı	LIMD1	-0.88785	-1.29298	-1.30256	-1.26053	-1.31451	-0.95693	-0.7997	-1.42269	-1.43721	-0.82517
Q9UGP8	Translocati	SEC63 SEC6	-0.57176	-0.65096	-1.41473	-1.12367	-1.08119	-1.12351	-0.16566	-0.71299	-0.88088	-0.99672
Q9UGU0	Transcripti	TCF20 KIAA	-0.83954	-0.54839	-0.33662	-0.0973	-0.04532	-0.35091	-0.26303	-0.38482	-0.31487	-0.06796
Q9UGV2	Protein ND	NDRG3	-0.96079	-0.72587	-0.4352	-0.14073	-0.72624	-0.62356	-0.64592	-0.52102	-0.47985	-0.3273
Q9UH62	Armadillo r	ARMCX3 AI	-1.58425	-2.38178	-0.0516	-0.44461	-0.10007	-0.22422	-0.23266	-0.07641	-2.64539	-2.35155
Q9UH65	Switch-ass	SWAP70 KI	-0.82873	-0.7112	-0.20495	0.299168	-0.59991	0.150363	-0.12832	-0.27684	0.104556	0.127005
Q9UH99	SUN domai	SUN2 FRIG	0.440573	0.411133	0.157321	0.433084	-0.38567	-0.36792	0.244153	-0.1155	-1.06365	-1.11032
Q9UHA2	SS18-like p	SS18L2	-1.46855	-1.5541	-1.16506	-0.92663	-1.07635	-1.12553	-1.63176	-0.54265	-1.52947	-0.84093
Q9UHA3	Probable ri	RSL24D1 C:	-0.23193	-0.17535	-0.32756	-0.24847	-0.64489	-0.61364	-0.56994	-0.28855	-0.45929	-0.08922
Q9UHA4	Ragulator c	LAMTOR3 I	-0.5639	-0.92405	-1.78991	-0.95067	-1.57755	-1.18641	-0.80863	-1.45372	-0.88235	-0.70487
Q9UHB6	LIM domaiı	LIMA1 EPLI	-0.86139	-0.37242	0.342576	0.378212	-0.10477	-0.20519	-0.45305	-0.22577	-0.16463	0.017594
Q9UHB9	Signal reco	SRP68	-0.30236	-0.38672	-0.34149	-0.07174	-0.45895	-0.37138	-0.39731	-0.71296	-0.59918	-0.69768
Q9UHD1	Cysteine ar	CHORDC1 (	0.646922	0.593761	0.730572	0.77102	0.738919	0.716521	0.735246	0.798972	0.888932	0.980057
Q9UHD2	Serine/thre	TBK1 NAK	-0.69515	-0.90202	-0.58301	-0.27563	-0.76702	-1.17403	-0.7997	-0.72935	-0.95361	-0.71937
Q9UHD8	Septin-9 (M	SEPTIN9 KI.	0.226807	0.127049	0.302947	-0.06594	0.2539	0.555519	0.521787	0.478047	0.417202	0.378436
Q9UHG3	Prenylcyste	PCYOX1 KIA	-0.19095	-0.27325	-0.29678	-0.40886	-0.50771	-0.14958	-0.06587	-0.22249	-0.40564	-0.41209

Q9UHJ6	Sedoheptu	SHPK CARK	0.275107	0.26628	-1.43763	-1.93261	0.258068	0.334984	0.500694	0.415037	0.018737	-0.05411
Q9UHL4	Dipeptidyl	DPP7 DPP2	-0.5649	-0.44893	-0.78705	-0.79594	-1.82286	-1.1366	-0.66382	-1.12419	-1.02177	-0.82653
Q9UHP3	Ubiquitin c	USP25 USP	-0.57289	-0.57719	0.169058	0.097024	0.114333	0.135515	-0.28157	0.088809	0.320041	0.131052
Q9UHQ9	NADH-cyto	CYB5R1 NC	-0.84968	-1.27316	-1.24143	-0.99766	-1.74926	-1.31174	-1.23718	-1.03453	-1.51485	-1.75158
Q9UHR5	SAP30-binc	SAP30BP H	-1.68536	-1.13906	-1.24598	-1.23078	-2.64572	-1.91511	-1.23236	-1.38717	-1.01898	-1.59384
Q9UHV9	Prefoldin sı	PFDN2 PFD	0.572857	0.538714	0.793008	0.518683	0.812591	0.383563	0.644635	0.748213	0.886831	0.857914
Q9UHW9	Solute carr	SLC12A6 K(	-1.29775	-1.62876	-1.8868	-1.73289	-0.1161	0.212994	0.150467	-0.20249	-0.90425	-1.23342
Q9UHX1	Poly(U)-bin	PUF60 FIR I	0.059379	0.220163	0.057095	0.067399	-0.12418	0.074547	0.338342	-0.12553	-0.24284	-0.14906
Q9UHY1	Nuclear rec	NRBP1 BCC	-0.29272	-0.2532	-0.36146	-0.3377	-0.21206	-0.25603	-0.20291	-0.50155	-0.13845	-0.34535
Q9UHY7	Enolase-ph	ENOPH1 M	-1.27228	-0.66388	-1.26012	-1.16938	-1.33638	-1.3073	-1.2713	-1.27412	-2.11382	-2.19285
Q9UI08	Ena/VASP-l	EVL RNB6	-0.04933	-0.63656	-0.28409	-0.55951	-0.39377	-0.1375	-0.41489	-0.19594	-0.09455	-0.25022
Q9UI09	NADH dehy	NDUFA12 D	0.348875	0.758055	0.475797	0.468367	0.304457	0.53255	0.188239	0.42731	0.55864	0.507517
Q9UI10	Translation	EIF2B4 EIF2	0.090696	0.00458	-0.25058	-0.08866	-0.35565	-0.13559	0.063701	-0.44807	-0.30584	-0.47254
Q9UI12	V-type prot	ATP6V1H C	0.183777	0.141512	-0.29252	-0.00256	-0.06182	0.075377	-0.16716	0.030082	-0.15148	-0.26862
Q9UI30	Multifuncti	TRMT112 A	-1.28011	-1.25526	-0.30256	-0.18728	-0.27798	0.304334	-0.22069	0.061639	0.293359	0.122947
Q9UIA0	Cytohesin-4	CYTH4 CYT4	-0.58193	-0.4761	-0.7112	-0.79021	-1	-0.66935	-0.56212	-0.76783	-1.08746	-0.71937
Q9UIA9	Exportin-7	XPO7 KIAA(	-0.33258	-0.18265	-0.15634	-0.11757	-0.12418	-0.33015	-0.18536	0.050626	0.095759	0.047877
Q9UIC8	Leucine car	LCMT1 LCM	-1.80549	-1.63174	-1.48154	-2.36658	-0.269	-0.78701	-2.15468	-2.23311	-2.11094	-2.06428
Q9UID3	Vacuolar pı	VPS51 ANG	-2.76553	-3.83094	-2.35304	-2.3683	-2.7469	-2.8016	-2.06311	-2.65751	-3.45003	-3.44184
Q9UIF9	Bromodom	BAZ2A KIA/	-1.74851	-1.06733	-1.04757	-1.23078	-1.47063	-0.92545	-2.21192	-2.05787	-1.83976	-2.21621
Q9UIG0	Tyrosine-pı	BAZ1B WB!	-0.47697	-0.34651	-0.72403	-0.53261	-0.54463	-0.5366	-0.74887	-0.58886	-0.7327	-0.88112
Q9UII2	ATPase inh	ATP5IF1 AT	-0.08746	0.151728	0.540081	0.4899	0.411441	0.393625	0.352302	0.465664	0.625644	0.602557
Q9UIJ7	GTP:AMP p	AK3 AK3L1	-0.04373	-0.07735	0.544082	0.660337	0.662182	0.565266	0.989732	0.545045	0.8459	0.650939
Q9UIL8	PHD finger	PHF11 BCA	0.160465	0.236803	0.658443	0.593302	0.476903	0.343623	0.529334	0.51056	0.202044	0.468255
Q9UIQ6	Leucyl-cyst	LNPEP OTA	-0.23816	-0.2262	-0.45451	-0.97592	-0.36151	-0.11376	-0.41346	-0.45483	0.069205	-0.00339
Q9UIS9	Methyl-Cp(	MBD1 CXX(	-0.83954	-0.67963	-1.72403	-1.67557	-1.59991	-2.06711	-0.97968	-1.36257	-1.81444	-1.69052
Q9UIV1	CCR4-NOT	CNOT7 CAF	-0.34023	-0.65486	-0.35147	-0.39428	-0.39046	-0.2115	-0.62385	-0.40742	-0.45649	-0.52855
Q9UJ41	Rab5 GDP/	RABGEF1 R	-1.15335	-1.14399	-1.03156	-1.17334	-0.6655	-0.78163	-1.62301	-1.20191	-1.15997	-1.14629
Q9UJ70	N-acetyl-D-	NAGK	0.431612	0.414062	-0.29776	-0.55087	-0.5231	-0.60302	-0.87276	-0.50967	-0.71049	-0.47836
Q9UJA5	tRNA (ader	TRMT6 KIA	-0.40426	-0.32822	-0.34661	-0.26974	-0.01925	0.159762	-0.15499	0.210376	0.171262	0.15341
Q9UJC3	Protein Ho	HOOK1	-1.57289	-0.92405	-0.85896	-0.96285	-0.14048	-0.67807	-0.76451	-1.08246	-0.36864	-0.51584
Q9UJU2	Lymphoid e	LEF1	-2.0377	-1.69816	-1.32134	-1.66671	-1.36981	-1.23606	-0.62363	-0.78294	-0.69363	-0.7762
Q9UJU6	Drebrin-like	DBNL CMA	-0.74507	-0.6245	-0.53138	-0.69574	-0.45678	-0.46385	-0.63176	-0.65535	-0.29739	-0.51584

Q9UJW0	Dynactin su	DCTN4	-0.8858	-0.62539	-0.40551	-0.46799	-0.65194	-0.28821	-0.44799	-0.63054	-0.32274	-0.16399
Q9UJX3	Anaphase-p	ANAPC7 AF	-0.45187	-0.26459	-0.15421	-0.3601	-0.71727	-0.10188	-0.35165	-0.17638	-0.5639	-0.08663
Q9UJY4	ADP-ribosy	GGA2 KIAA	-0.818	-0.36146	0.042266	0.507657	-0.53382	-0.5366	-0.80863	-0.95491	-0.75754	-0.14906
Q9UJY5	ADP-ribosy	GGA1	1.119203	1.155119	1.518467	1.190684	1.330824	1.147899	1.173332	1.308122	1.591235	1.219228
Q9UJZ1	Stomatin-li	STOML2 SL	-0.63743	-0.46371	-0.52015	-0.71621	-0.25118	-0.14353	-0.59266	-0.61096	-0.43879	-0.81735
Q9UK41	Vacuolar pı	VPS28	-1.44741	-0.98964	-1.5784	-1.79478	-1.76304	-1.90779	-1.64654	-0.92564	-1.64848	-1.59484
Q9UK45	U6 snRNA-a	LSM7	0.780099	0.91951	0.897065	0.830467	0.903913	0.887557	0.761315	0.820382	1.014075	0.916963
Q9UK53	Inhibitor of	ING1	-0.74507	-0.52576	-0.10932	-0.51457	-0.36151	-0.78701	-0.53222	-0.71049	-0.49584	-0.36004
Q9UK59	Lariat debr	DBR1	-1.35566	-1.03953	-1.42286	-1.71621	-1.62263	-1.58496	-1.63176	-1.50155	-1.25739	-1.20918
Q9UK61	Protein TAS	TASOR C3o	-2.35705	-2.28291	-2.95334	-2.02401	-2.4769	-2.73479	-1.33727	-2.30541	-2.33151	-2.22121
Q9UK76	Jupiter mic	JPT1 ARM2	-0.19438	0.061076	0.075949	0.079322	-0.41009	-0.26303	0.010196	-0.1571	-0.00472	-0.26127
Q9UK99	F-box only	FBXO3 FBX	-1.1439	-0.46489	-0.3998	-0.20019	-0.89894	-1.32105	-0.57098	-0.70794	-0.94585	-1.01119
Q9UKA4	A-kinase ar	AKAP11 AK	-0.69515	-0.06786	0.735405	0.974689	-0.23359	-0.07285	-0.02061	-0.31907	0.266174	0.039289
Q9UKA8	Calcipressi	RCAN3 DSC	-0.87245	-0.93147	-0.7632	-0.59756	-0.42	-0.28951	-0.61599	-0.90046	-0.41818	-0.04039
Q9UKD1	Glucocortic	GMEB2 KIA	-0.6668	-0.6915	-0.50357	-0.52879	-0.56603	-0.34743	-0.39645	-0.55684	-0.10628	-0.19822
Q9UKD2	mRNA turn	MRTO4 C1	1.163174	1.2824	1.036575	1.326937	0.855414	1.277534	1.198071	1.042311	1.329452	1.372371
Q9UKE5	TRAF2 and	TNIK KIAA0	-1.17763	-0.84582	-0.65486	-0.70594	-1.14048	-0.71348	-1.51019	-1.05842	-0.93546	-0.7713
Q9UKF6	Cleavage a	CPSF3 CPSF	-1.28011	-1.09675	-1.25526	-1.67557	-1.22487	-1.0902	-1.58496	-1.19592	-1.30319	-0.84888
Q9UKF7	Cytoplasmi	PITPNC1	0.371969	0.618366	-0.40726	-0.89129	-0.53382	-1.17403	-0.50293	-0.52607	-0.80618	-0.63448
Q9UKG1	DCC-intera	APPL1 APP	0.213707	0.532014	0.507538	0.329436	0.336725	0.510195	0.155069	0.067114	0.553184	0.670863
Q9UKG9	Peroxisoma	CROT COT	-1.53727	-0.65486	-0.08017	0.622115	-0.84503	-0.80599	-1.10613	-1.97728	-1.2916	-1.16882
Q9UKI8	Serine/thre	TLK1 KIAA0	-2.76553	-1.39183	-1.02359	-1.30633	-1.24236	-1.07861	-1.31937	-1.29078	-1.00946	-0.71937
Q9UKJ3	G patch do	GPATCH8 G	-0.32476	-0.0798	-0.33284	-0.32189	-0.10031	-0.2581	-0.37792	0.016784	0.290707	0.076537
Q9UKK3	Protein mo	PARP4 ADP	0.989171	1.113457	1.308255	1.068386	1.012437	1.152971	1.08747	1.074605	1.193219	1.153511
Q9UKK9	ADP-sugar	NUDT5 NU	0.501787	0.553427	0.474243	0.037153	0.18799	-0.22422	-0.12274	0.152003	0.304855	0.896825
Q9UKM9	RNA-bindir	RALY HNRP	0.976751	1.060708	1.007777	1.140352	1.134797	1.027068	0.775632	0.669052	0.643356	0.651707
Q9UKN8	General tra	GTF3C4	0.224123	0.332805	0.027039	-0.45317	0.276604	-0.11958	0.186878	0.094182	0.281771	-0.08194
Q9UKT9	Zinc finger	IKZF3 ZNFN	0.211201	0.18253	0.100744	-0.09088	0.084136	0.272852	0.225173	0.275065	0.22964	0.178934
Q9UKU7	Isobutyryl-	ACAD8 ARC	-1.80735	-1.16506	-0.87317	-1.4361	-1.41009	-1.50524	-1.87276	-1.82753	-1.7028	-1.03585
Q9UKV3	Apoptotic c	ACIN1 ACIN	-0.33943	-0.09259	0.019365	0.421296	-0.06075	-0.21887	-0.01027	-0.02843	0.327575	0.250314
Q9UKV8	Protein arg	AGO2 EIF2	1.038363	1.065901	0.761764	0.741973	0.753254	0.893001	0.996348	0.72316	1.112733	1.063832
Q9UKX7	Nuclear po	NUP50 NPA	-0.13327	0.130397	0.238196	0.373605	0.107446	0.122533	-0.16811	-0.02843	0.377413	0.393177
Q9UKY7	Protein CD	CDV3 H41	0.44053	0.512758	0.570836	0.393885	0.409507	0.459214	0.524521	0.530206	0.691159	0.97472

Q9UKZ1	CCR4-NOT	CNOT11 C2	-0.78629	-0.50901	-0.4021	-0.55087	-0.19052	-0.40777	-0.84493	-0.46949	-0.65006	-0.66223
Q9UL15	BAG family	BAG5 KIAA	-0.10692	-0.1782	-0.3971	-0.28325	-0.12138	-0.45656	-0.16722	-0.12339	-0.2486	-0.07977
Q9UL17	T-box trans	TBX21 TBET	-2.77502	-1.59504	-1.85812	-1.4397	-2.34206	-2.07058	-1.3567	-1.46983	-2.52767	-1.76471
Q9UL18	Protein arg	AGO1 EIF2(	-1.34023	-1.15634	-1.16212	-0.96799	-1.56653	-1.11138	-1.5327	-1.32169	-1.15548	-0.925
Q9UL25	Ras-relatec	RAB21 KIAA	0.285091	-0.23676	-0.12199	-0.15899	-0.24236	-0.41504	-0.27537	-0.95491	-1.11469	-1.24036
Q9UL26	Ras-relatec	RAB22A RA	1.686383	1.484034	1.497906	1.15451	1.327865	1.467359	1.467779	1.406424	1.141356	1.133071
Q9UL33	Trafficking	TRAPPC2L I	-1.40296	-0.96153	-1.5541	-1.05107	-2.59991	-1.34395	-1.23266	-0.95491	-1.74959	-1.37145
Q9UL40	Zinc finger	ZNF346 JAZ	-1.30986	-1.53138	-0.16506	-0.71621	-0.33313	-0.69567	-0.4957	-0.10692	0.06454	0.094215
Q9UL46	Proteasom	PSME2	1.180052	1.149015	1.219133	0.946186	0.799724	0.838054	1.16389	1.217779	1.331454	1.303036
Q9ULA0	Aspartyl an	DNPEP ASP	0.680435	0.955786	-0.80578	-0.98966	-0.33743	-0.42712	-0.39316	-0.82446	-0.56063	-0.58238
Q9ULC4	Malignant	MCTS1 MC	1.129367	1.115915	0.999389	0.985399	0.894618	1.142548	1.157299	1.16804	1.029458	0.905603
Q9ULC5	Long-chain	ACSL5 ACS!	-1.40296	-1.3423	-1.41641	-2.44181	-2.42776	-2.08945	-1.59218	-1.39122	-1.4119	-1.84093
Q9ULD4	Bromodom	BRPF3 KIAA	-1.12663	-1.66102	-1.52015	-2.8011	-2.69302	-1.93587	-1.52484	-1.76783	-1.76553	-1.16882
Q9ULG1	Chromatin-	INO80 INO8	0.690145	0.819558	-0.16069	0.538214	-0.06075	-0.03877	0.500694	-0.1193	0.046393	-0.40036
Q9ULH1	Arf-GAP wi	ASAP1 DDE	-1.40811	-1.43713	-0.93511	-1.31111	-0.98427	-1.27008	-1.25534	-1.11663	-1.59508	-1.31964
Q9ULK4	Mediator o	MED23 AR(	-1.70486	-1.51834	-1.87112	-2.36188	-2.00934	-2.25262	-1.51322	-1.35614	-1.66009	-1.7002
Q9ULR0	Pre-mRNA-	ISY1 KIAA1	-0.16061	-0.14194	0.268171	0.106585	-0.05149	0.062979	0.077485	0.190044	0.259356	0.441045
Q9ULT8	E3 ubiquiti	HECTD1 KIA	-1.21371	-0.87437	-0.77361	-0.87131	-1.14412	-1.02541	-1.16793	-1.04819	-1.20841	-0.69316
Q9ULU4	Protein kin	ZMYND8 KI	-1.04933	-1.17383	-1.82219	-1.95195	-1.59991	-1.05571	-1.58496	-1.09464	-0.96277	-1.1589
Q9ULV3	Cip1-intera	CIZ1 LSFR1	-1.85135	-2.28193	-1.24209	-2.06667	-2.10432	-2.13911	-1.82766	-1.74259	-3.79796	-3.70487
Q9ULV4	Coronin-1C	CORO1C CF	-1.30285	-0.59145	-0.71764	-0.8001	1.18799	1.360747	-0.76467	-0.64057	1.147754	1.054284
Q9ULW3	Activator o	ABT1	-1.30929	-1.18122	-1.35327	-1.25028	-0.83642	-1.23573	-1.19119	-1.45114	-1.24644	-1.27035
Q9ULX6	A-kinase ar	AKAP8L NA	-1.13128	-1.00098	-0.87056	-0.85175	-0.9067	-1.03223	-0.42602	-0.79352	-1.23799	-0.70338
Q9ULZ3	Apoptosis-a	PYCARD AS	0.044426	0.470458	-0.2014	0.40199	0.00933	-0.21028	-0.20608	-0.47213	-0.62785	0.293648
Q9UM00	Calcium loa	TMCO1 TM	-1.74507	-1.81714	-2.64874	-3.52356	-3.01495	-3.72247	-1.48134	-3.58496	-3.45003	-4.34873
Q9UM54	Unconvent	MYO6 KIAA	2.189991	3.113376	3.492026	4.331618	3.014797	2.451461	2.71843	0.633745	2.377423	2.384287
Q9UMR2	ATP-depen	DDX19B DE	-0.62185	-0.81371	0.967917	0.636291	0.463268	0.129399	1.141492	0.640989	0.648312	0.463627
Q9UMS0	NFU1 iron-	NFU1 HIRIF	-0.41908	-0.1305	0.269518	0.198904	0.072512	-0.01097	-0.04152	0.141661	0.323813	0.493622
Q9UMS4	Pre-mRNA-	PRPF19 NM	0.506121	0.652271	0.624455	0.601001	0.766171	0.743293	0.669286	0.790642	0.816744	0.909837
Q9UMX5	Neudesin ((	NENF CIR2	-0.26943	-0.27231	-0.71858	-0.55522	-0.72676	-0.95013	-0.70278	-0.83676	-0.44094	-0.17197
Q9UMY1	Nucleolar p	NOL7 C6or	-1.2949	-0.93892	-0.59474	-1.5787	-1	-0.35091	-0.76451	-0.90046	-0.71823	-0.75627
Q9UMY4	Sorting nex	SNX12	1.533542	1.263034	1.383004	1.316901	1.100527	1.189226	1.193604	1.251539	1.732716	1.596716
Q9UMZ2	Synergin ga	SYNRG AP1	-1.32219	-1.46529	-0.72374	-1.28053	-0.91348	-1.52083	-1.52484	-1.848	-1.32665	-0.69932

Q9UN37	Vacuolar pı	VPS4A VPS	-0.04933	-0.0843	0.158617	0.241973	-0.15697	0.165059	0.186878	0.172467	-0.3445	-0.25597
Q9UN86	Ras GTPase	G3BP2 KIA	-0.41908	-0.46529	-0.35147	-0.07731	-0.32379	0.079727	-0.75585	-0.30485	-0.23502	-0.2507
Q9UNE7	E3 ubiquitiı	STUB1 CHII	-1.25096	-1.4021	-1.61252	-0.85681	-1.57755	-1.48981	-0.77323	-0.96605	-1.97199	-1.27191
Q9UNF0	Protein kin	PACSIN2	0.181998	-0.17383	0.130397	-0.38839	-0.03388	0.595851	0.145851	-0.10365	0.756633	0.245238
Q9UNF1	Melanoma	MAGED2 B	-0.87245	-0.88753	-0.49796	-0.55855	-0.81855	-0.26874	-1	-0.24257	-0.29739	-0.06333
Q9UNH7	Sorting nex	SNX6	0.039714	-0.13725	0.130397	0.073373	-0.16528	-0.07015	0.054093	-0.1193	0.078016	-0.03132
Q9UNL2	Translocon	SSR3 TRAP	-0.92659	-1.17889	1.246696	1.15451	0.476903	0.727095	1.348289	0.810623	0.13493	0.251185
Q9UNM6	26S protea	PSMD13	-0.06827	0.786031	-0.20495	-0.30633	-0.4828	-0.52869	-0.69297	-0.53434	-0.5639	-0.62716
Q9UNN5	FAS-associa	FAF1 UBXD	-0.45738	0.058466	-0.35224	-0.51393	-0.0532	-0.3621	-0.2456	-0.47055	0.020086	0.027109
Q9UNP9	Peptidyl-pr	PPIE CYP33	-0.52146	-0.29621	-0.32205	-0.53177	-0.44649	-0.50107	-0.22555	-0.15308	-0.01688	-0.17591
Q9UNQ2	Probable d	DIMT1 DIM	0.44977	0.431792	0.378153	0.062527	0.529224	0.253593	0.168436	0.095233	0.458163	0.546342
Q9UNS2	COP9 signa	COPS3 CSN	0.353079	0.475645	0.570854	0.289886	0.486926	0.583415	0.335903	0.621751	0.575114	0.734927
Q9UNX4	WD repeat	WDR3	-0.90613	-1.14768	-1.08331	-1.35801	-0.83173	-0.56058	-0.44743	-0.83907	-1.08746	-0.72668
Q9UNZ2	NSFL1 cofa	NSFL1C UB	0.029995	-0.04354	-0.08844	-0.09457	0.120035	-0.15565	-0.07346	-0.26303	0.162386	0.355175
Q9UNZ5	Leydig cell	C19orf53 H	0.997877	0.868778	1.444444	1.45135	1.826897	1.761363	1.357915	1.448453	1.429478	1.216617
Q9UP83	Conserved	COG5 GOL	-2.4265	-2.53704	-1.1305	-0.67557	-1.51245	-0.86449	-1.13955	-1.69188	-1.51592	-1.01781
Q9UPN3	Microtubul	MACF1 ABI	-0.5639	-0.4012	-0.21738	-0.18015	-0.35199	-0.35091	-0.43219	-0.46158	-0.45649	-0.62425
Q9UPN6	SR-related	SCAF8 CCA	-0.57519	-0.80185	-0.2112	-0.35412	-0.5605	-0.43856	-0.67755	-0.40813	-0.4362	-0.12357
Q9UPN7	Serine/thre	PPP6R1 KIA	-0.81291	-0.98616	-1.03284	-0.83996	-0.79304	-1.30003	-1.20582	-1.22338	-1.08698	-0.69769
Q9UPN9	E3 ubiquitiı	TRIM33 KIA	-0.82873	-0.41764	0.443033	-0.11757	0.67155	0.250227	0.079617	0.232173	0.368321	0.515458
Q9UPP1	Histone lys	PHF8 KIAA1	-0.63743	-0.64874	-0.83789	-0.71621	-0.06853	-0.46697	-0.61599	-0.55943	-0.1281	-0.36004
Q9UPQ9	Trinucleoti	TNRC6B KIA	-1.89482	-1.77649	-1.83094	-1.67557	-1.33313	-1.91511	-2.75629	-2.91264	-1.68713	-2.03587
Q9UPR0	Inactive ph	PLCL2 KIAA	-3.14889	-3.34169	-3.11454	-3.39808	-3.96652	-3.26902	-2.93486	-2.79412	-2.60151	-3.49161
Q9UPT8	Zinc finger	ZC3H4 C19	-0.3239	-0.25866	-0.32316	-0.53509	-0.35364	-0.51278	-0.5346	-0.63419	-0.34818	-0.22611
Q9UPU5	Ubiquitin c	USP24 KIA	-1.59582	-0.99423	-1.28477	-1.69449	-0.77349	-0.61815	-1.07258	-1.31829	-1.08389	-1.24679
Q9UPU7	TBC1 doma	TBC1D2B K	-2.35441	-2.06898	-2.76764	-2.73712	-1.22372	-1.50657	-1.63898	-1.85715	-2.19711	-2.22016
Q9UPW0	Forkhead b	FOXJ3 KIAA	-2.64689	-3.44392	-2.36146	-3.9386	-2.59991	-4.23704	-3.96963	-3.33342	-1.89984	-3.13927
Q9UPW5	Cytosolic c	AGTPBP1 C	-1.87673	-1.32058	-0.82048	-0.79098	-0.87747	-1.65844	-0.88782	-0.82821	-1.26911	-1.3429
Q9UQ35	Serine/argi	SRRM2 KIA	-0.72489	-0.99222	-0.74759	-0.79057	-0.6271	-0.90072	-0.61233	-0.59358	-0.49584	-0.5039
Q9UQ80	Proliferatio	PA2G4 EBP	0.723269	1.040298	0.447287	0.306953	0.841872	0.68862	0.699315	0.644356	0.966606	0.843021
Q9UQE7	Structural r	SMC3 BAM	-0.20133	-0.05816	0.174035	0.176851	0.184147	0.181968	0.145851	0.178825	0.385995	0.241528
Q9UQN3	Charged m	CHMP2B C	-1.26686	-1.16244	-0.803	-1.19703	-0.77979	-0.94028	-0.83646	-0.38113	-0.93049	-1.15941
Q9UQR1	Zinc finger	ZNF148 ZBI	-0.34858	-0.24155	-0.8799	-1.73712	-0.57287	-0.85491	-0.97972	-0.86944	-0.71936	-0.35168

Q9Y217	Myotubula	MTMR6	-2.46894	-2.49281	-1.40534	-1.80708	-1.21129	-2.40343	-1.87447	-2.21795	-2.46773	-2.24828
Q9Y223	Bifunctiona	GNE GLCNE	0.822859	0.890683	0.603045	0.349267	0.766409	0.551796	0.882816	0.959358	0.393664	0.833666
Q9Y224	RNA transc	RTRAF C14	0.726862	0.914525	1.158491	1.064966	1.086421	0.996137	0.825057	1.077134	1.280898	1.130481
Q9Y228	TRAF3-inte	TRAF3IP3 T	-0.2501	-0.05976	0.264887	-0.03929	-0.01765	-0.00906	0.159657	-0.04649	-0.15106	0.02764
Q9Y230	RuvB-like 2	RUVBL2 IN	0.387904	0.375509	0.425227	0.228271	0.497096	0.406138	0.43721	0.393383	0.595779	0.609077
Q9Y237	Peptidyl-pr	PIN4	0.488887	0.566651	0.375509	0.085247	0.766409	1.058894	0.733893	0.733527	0.222392	0.098355
Q9Y262	Eukaryotic	EIF3L EIF3E	0.937244	1.119199	0.47087	0.452883	0.303112	0.364815	0.446558	0.384032	0.397705	0.572337
Q9Y263	Phospholip	PLAA PLAP	-0.12003	0.169058	-0.06507	0.126454	0.025569	-0.43706	-0.23378	-0.69951	-0.2636	-0.07043
Q9Y265	RuvB-like 1	RUVBL1 IN	-0.06589	-0.07604	-0.08452	-0.19444	0.087471	-0.00151	-0.04819	0.016352	0.055495	0.260847
Q9Y266	Nuclear mi	NUDC	0.657318	0.892785	0.326644	0.563026	0.559958	0.934622	0.721536	0.669605	0.825601	0.967933
Q9Y277	Voltage-de	VDAC3	1.738177	1.647283	0.349022	0.875609	-0.68988	0.593581	1.303392	0.839704	-0.38278	-0.9441
Q9Y282	Endoplasm	ERGIC3 C2	-0.43539	-0.57138	-0.37656	-0.44461	-0.76702	-0.70454	-0.43911	-0.43812	-1.3505	-1.21945
Q9Y285	Phenylalan	FARSA FAR	0.192645	0.307544	0.123255	-0.24382	-0.06853	0.010888	0.06502	0.197646	0.436099	0.316608
Q9Y295	Developme	DRG1 NEDI	-0.83954	-0.82402	-0.58887	-0.55781	-0.71727	-0.51301	-0.74723	-0.6735	-0.50441	-0.47968
Q9Y2B0	Protein car	CNPY2 MS	-0.55667	-0.48848	-0.31773	-0.37428	-0.14436	-0.41518	-0.57459	-0.43028	-0.24896	-0.38883
Q9Y2D0	Carbonic a	CA5B	1.096657	1.143933	1.200885	1.210088	1.381114	1.481667	1.200247	1.402183	1.026572	1.043493
Q9Y2D9	Zinc finger	ZNF652 KIA	0.254929	0.521187	0.603045	0.329436	0.735478	0.330645	0.323973	0.017074	0.500835	0.458627
Q9Y2G2	Caspase re	CARD8 KIA	-0.3677	0.079404	0.267758	0.359356	0.293046	0.363167	-0.03998	-0.00796	0.087095	-0.00834
Q9Y2H0	Disks large	DLGAP4 DA	0.658622	0.556262	-0.73048	-0.28325	-0.4086	0.323503	-0.02154	-0.1901	-0.25471	-0.15945
Q9Y2H6	Fibronectir	FNDC3A FN	-2.76553	-1.44392	-2.70272	-3.49875	-3.66473	-2.17156	-1.55459	-2.848	-2.06756	-2.37145
Q9Y2I1	Nischarin (I	NISCH IRAS	-1.28011	-1.38163	-1.05565	-0.5881	-1.42999	-1.31647	-1.07346	-1.19592	-0.74959	-0.54137
Q9Y2I8	WD repeat	WDR37 KIA	0.399803	0.414062	0.776958	0.658336	0.809478	0.564159	0.776328	0.561879	0.390071	0.409495
Q9Y2K7	Lysine-spec	KDM2A CX	-1.43987	-0.96113	-1.655	-1.46721	-1.48563	-1.38015	-1.1055	-0.92302	-1.06756	-1.1589
Q9Y2L1	Exosome c	DIS3 KIAA1	-0.06193	0.210086	0.112362	0.220318	-0.00463	-0.04868	0.253432	0.35736	-0.20754	-0.12956
Q9Y2L5	Trafficking	TRAPPC8 K	-0.27277	-0.06786	0.039083	0.30827	-0.37109	-1.0902	-0.03653	-0.09822	-0.82276	-0.30981
Q9Y2L9	Leucine-ric	LRCH1 CHD	-0.77588	0.083328	-1.1305	-1.21613	-0.76702	-0.94636	-0.90115	-1.33342	-0.62076	-0.54783
Q9Y2P8	RNA 3'-terr	RCL1 RNAC	-1.48543	-1.16506	-1.7632	-1.23078	-1.74193	-0.77761	-0.88216	-1.48543	-1.08746	-1.60726
Q9Y2Q3	Glutathion	GSTK1 HDC	0.969681	0.822702	0.944352	0.672017	0.454548	0.888025	0.601348	0.71217	0.460325	0.37562
Q9Y2Q5	Ragulator c	LAMTOR2 I	-0.98563	-0.61511	-1.18726	-0.66885	-1.17209	-0.74319	-0.9106	-1.36603	-1.27202	-1.03908
Q9Y2Q9	28S ribosor	MRPS28 M	-0.37126	-0.24598	-0.48153	-0.44461	-0.54463	-0.11958	-0.41163	-0.29078	-0.46949	-0.1788
Q9Y2R2	Tyrosine-pı	PTPN22 PT	1.458329	1.484034	1.701039	1.496585	1.607101	1.568474	1.622	1.624491	1.338802	1.396106
Q9Y2R5	28S ribosor	MRPS17 RI	0.297304	0.213021	0.267979	0.14315	0.287789	0.177345	0.272132	0.27454	0.362542	0.003919
Q9Y2S6	Translation	TMA7 CCD	0.131698	0.35344	1.099068	0.788717	1.74086	0.899496	0.96276	0.85545	1.954431	2.351685

Q9Y2T2	AP-3 comp	AP3M1	1.438338	1.609448	0.779231	0.837505	0.879867	1.128033	1.120192	0.911463	0.792461	0.880091
Q9Y2U5	Mitogen-ac	MAP3K2 M	-1.70499	-1.58887	-1.81714	-1.73697	-0.97056	-2.02203	-1.45305	-2	-1.83112	-1.74882
Q9Y2U8	Inner nucle	LEMD3 MA	-1.33147	-1.62069	-1.2752	-1.20192	-2.15478	-1.72586	-1.43063	-1.44021	-1.67368	-1.29885
Q9Y2V2	Calcium-re	CARHSP1	-3.30986	-2.4021	-1.42286	-1.96285	-2.07635	-2.0902	-1.52484	-1.55102	-1.9906	-1.30433
Q9Y2W1	Thyroid ho	THRAP3 BC	0.478033	0.482639	0.784829	0.547038	0.588856	0.500538	0.43429	0.493058	1.078949	1.064956
Q9Y2W2	WW domai	WBP11 NP	-0.01435	-0.02971	0.745388	0.834848	0.842017	0.938859	0.634016	0.945416	1.015828	1.054537
Q9Y2W6	Tudor and	TDRKH TDF	-1.40065	-0.60807	-0.79329	-0.80821	-0.72137	-0.51405	-0.83191	-0.54366	-0.90576	-0.94636
Q9Y2X3	Nucleolar p	NOP58 NO	-0.09019	0.263034	0.832753	0.4899	0.899933	0.551796	0.478834	0.312739	0.89743	0.711968
Q9Y2X7	ARF GTPas	GIT1	-1.06193	-1.02359	-0.91697	-0.76808	-0.81855	-0.4975	-1.25696	-1.35838	-0.66182	-0.84093
Q9Y2Y0	ADP-ribosy	ARL2BP BA	0.741807	0.756331	-1.31635	-1.56868	-1.16377	-1.5985	-2.16573	-1.85838	0.756633	0.656896
Q9Y2Y9	Krueppel-li	KLF13 BTEE	-2.57289	-1.96153	-2.17383	-3.20163	-3.35199	-2	-3.3322	-3.80735	-3.5025	-2.2402
Q9Y2Z0	Protein SG	SUGT1	-0.79679	-0.70483	-0.31706	-0.0707	-0.4855	-0.60126	-0.64703	-0.77305	-0.43549	-0.19898
Q9Y2Z9	Ubiquinone	COQ6 CGI-	0.552645	0.52061	-0.66551	0.056828	0.370305	-0.04985	0.158212	0.311486	-0.77857	0.103612
Q9Y303	N-acetylglu	AMDHD2 C	-3.19438	-2.30256	-2.05565	-2.9386	-3.79256	-3.34395	NA	NA	-3.16993	-4.1008
Q9Y305	Acyl-coenz	ACOT9 CGI	-0.09508	0.115053	0.039567	-0.13607	0.258738	0.100417	-0.00499	-0.04946	-0.434	-0.52615
Q9Y314	Nitric oxide	NOSIP CGI-	0.268257	0.213448	0.151613	-0.0443	0.208074	0.263945	0.081208	0.032494	0.470533	0.573113
Q9Y315	Deoxyribos	DERA CGI-2	0.418065	0.675633	0.158617	0.765535	0.48755	0.053638	0.286713	0.380272	0.375607	0.47782
Q9Y320	Thioredoxi	TMX2 TXNI	-1.48873	-1.31966	-0.74261	-1.65041	-0.47543	-0.73406	-0.86659	-0.72066	-0.67572	-0.99607
Q9Y324	rRNA-proce	FCF1 C14or	-0.72489	-0.7112	-1.11353	-2.03814	-1.37109	-0.79647	-0.70492	-1.23581	-1.2916	-1.73402
Q9Y333	U6 snRNA-a	LSM2 C6or	0.165878	0.363436	1.239713	1.140226	1.154975	1.367812	0.727727	1.270648	1.381156	1.230588
Q9Y371	Endophilin-	SH3GLB1 K	-0.62609	-0.72236	-0.27811	-0.11169	-0.47456	-0.39155	0.248466	0.518575	-0.4068	-0.26278
Q9Y376	Calcium-bi	CAB39 MO	0.763191	0.786232	0.522493	0.865316	0.492844	1.138553	0.897877	0.971087	0.413046	0.196845
Q9Y383	Putative RI	LUC7L2 CG	0.021353	0.584904	0.310489	0.447027	0.366544	0.048738	0.180168	-0.18842	0.429016	0.570002
Q9Y394	Dehydroge	DHRS7 DHI	-0.73595	-0.58158	-0.30757	-0.7447	-1.50525	-0.71654	-0.60486	-0.58284	-0.27931	-0.59905
Q9Y399	28S riboso	MRPS2 CGI	-0.96094	-1.05676	-0.30083	0.005366	-0.58544	-0.38806	-0.36864	-0.34051	-0.38874	-1.06779
Q9Y3A2	Probable U	UTP11 UTP	-0.18979	-0.58887	-0.74522	-0.9164	-0.19903	-0.30219	-0.75386	-0.83951	-0.64565	-0.62936
Q9Y3A3	MOB-like p	MOB4 MOI	2.209245	2.348934	1.940453	2.065008	1.656091	1.827602	1.966141	1.973367	1.934975	1.810138
Q9Y3A4	Ribosomal	RRP7A CGI-	-0.13451	0.099317	-0.47591	-0.31224	0.006943	-0.20232	-0.43602	-0.21159	-0.35477	-0.18186
Q9Y3A5	Ribosome r	SBDS CGI-9	0.548126	0.968464	0.697437	0.689785	0.757639	0.90105	0.648351	0.655715	0.91504	1.364319
Q9Y3B2	Exosome co	EXOSC1 CS	-0.51978	-0.52576	-0.64874	-0.70594	-0.33313	-0.56058	-0.82667	-0.78746	-0.4892	-0.30433
Q9Y3B3	Transmemb	TMED7 CGI	0.317233	-0.07242	0.181857	0.916088	0.805649	0.408082	0.050062	1.48052	0.502319	0.84331
Q9Y3B7	39S riboso	MRPL11 CC	-0.07008	-0.17978	0.351249	0.354549	0.283287	0.492415	0.310062	0.458625	0.585644	0.59829
Q9Y3B8	Oligoribon	REXO2 SFN	-2.0332	-1.1325	-1.25241	-1.68453	-1.67874	-1.19863	-1.8367	-0.86325	-1.04118	-1.2017

Q9Y3C0	WASH com	WASHC3 A	-1.1669	-1.34156	-1.08017	-2.98751	-2.84503	-2.48981	-2.07346	-1.71049	-1.55695	-1.0726
Q9Y3C1	Nucleolar p	NOP16 CGI	1.267574	1.414062	0.638544	0.294061	1.110894	0.673282	0.303392	0.611435	1.080234	0.936674
Q9Y3C6	Peptidyl-pr	PPIL1 CYPL	1.2151	1.13861	1.362001	0.938704	1.369215	1.43202	1.338641	1.401991	1.459449	1.341885
Q9Y3C8	Ubiquitin-fo	UFC1 CGI-1	0.367277	0.357362	0.45446	0.055377	-0.02248	0.110424	0.336182	0.067114	0.242458	0.442435
Q9Y3D0	Cytosolic ir	CIAO2B CIA	-0.51769	-0.53156	-0.16722	-0.45317	-0.34206	-0.31798	-0.52458	-0.25089	-0.39055	-0.40313
Q9Y3D3	28S ribosor	MRPS16 RF	-3.37126	-3.44392	-1.7901	-2.91031	-2.68505	-2.52425	-1.92029	-1.37012	-1.6018	-2.34102
Q9Y3E5	Peptidyl-tR	PTRH2 BIT1	-1.51487	-1.55138	-1.61316	-1.31472	-1.80743	-1.27645	-2.3192	-1.46827	-1.72856	-1.73402
Q9Y3E7	Charged m	CHMP3 CG	0.213707	0.443033	0.550768	0.085247	0.300954	0.548063	-0.1452	0.197646	0.581816	0.637114
Q9Y3F4	Serine-thre	STRAP MAV	-0.73668	-0.5148	-0.74522	-0.70797	-0.04532	0.120513	-0.22069	0.222392	-0.57875	-0.5201
Q9Y3I0	RNA-splicir	RTCB C22o	0.484144	0.471596	0.622103	0.52877	0.534513	0.268489	0.522227	0.490049	0.739792	0.711968
Q9Y3L3	SH3 domai	SH3BP1	0.077561	0.181325	0.231355	0.258061	0.146019	0.128988	0.324682	0.261488	0.112714	0.208343
Q9Y3P8	Signaling th	SIT1 SIT	0.523035	0.798709	0.968852	0.895977	0.503375	0.710493	0.989732	0.861924	0.655501	0.634265
Q9Y3P9	Rab GTPase	RABGAP1 H	-0.28572	-0.44933	-0.1509	-0.12507	-0.38424	-0.27796	-0.2601	-0.39687	-0.47309	-0.44121
Q9Y3Q8	TSC22 dom	TSC22D4 TI	-0.63743	-0.50901	-0.5909	-0.20402	-0.54511	-0.42236	-0.55459	-0.69188	-0.3484	-0.53612
Q9Y3S2	Zinc finger	ZNF330 NC	-2.09393	-1.36648	-1.34914	-1.87277	-2.39628	-2.1682	-1.74224	-1.73827	-1.94762	-1.53816
Q9Y3T9	Nucleolar c	NOC2L NIR	-1.90994	-1.38393	-3.24598	-1.59756	-2.89947	-2.1375	-3.28157	-1.72935	-3.32684	-2.69912
Q9Y3U8	60S ribosor	RPL36	0.652077	0.740082	0.59934	0.13954	0.539639	0.356485	0.344264	0.011405	0.168877	0.28061
Q9Y3X0	Coiled-coil	CCDC9	-0.32849	-0.1393	-0.35702	-0.13108	-0.03404	-0.3491	-0.16326	-0.26282	-0.25583	-0.03941
Q9Y3Y2	Chromatin	CHTOP C1c	-0.54563	-0.49687	-0.78319	-1.48794	-0.88567	-0.68684	-0.65941	-0.48721	-0.42432	-0.46325
Q9Y3Z3	Deoxynucle	SAMHD1 M	0.775811	0.932143	0.269518	0.392115	0.34675	0.689504	0.699276	0.506615	0.234465	0.191275
Q9Y421	Protein FAI	FAM32A O	1.173012	1.427121	1.429432	1.128722	1.417691	1.136728	1.277681	0.991611	1.467031	1.651739
Q9Y450	HBS1-like p	HBS1L HBS	-1.92045	-2.15624	-1.62289	-2.24076	-1.53753	-1.98088	-1.79586	-1.65931	-1.91075	-1.47884
Q9Y478	5'-AMP-act	PRKAB1 AM	-1.25096	-1.02359	-1.24598	-1.0906	-0.51245	-0.67807	-0.76451	-0.80735	-1	-0.79414
Q9Y490	Talin-1	TLN1 KIAA1	0.313013	0.335249	0.17761	0.186394	0.086588	0.13378	0.24846	0.241839	0.063018	0.121547
Q9Y4A5	Transforma	TRRAP PAF	-0.25819	-0.01963	-0.44392	-0.11078	-0.17521	-0.02329	-0.01543	-0.0115	-0.00055	-0.26942
Q9Y4B6	DDB1- and	DCAF1 KIA/	-0.0485	0.119938	0.960831	0.466208	0.543943	0.211675	0.454134	0.346857	0.527614	0.993567
Q9Y4C1	Lysine-spec	KDM3A JHI	-2.38156	-2.08596	-2.35096	-2.06964	-2.29717	-2.02631	-0.93988	-1.13179	-1.849	-2.00789
Q9Y4C8	Probable R	RBM19 KIA	-3.57289	-3.62449	-4.07195	-3.03814	NA	NA	-2.9301	-2.88982	-2.32665	-2.04495
Q9Y4E1	WASH com	WASHC2C I	-1.17985	-1.28311	-1.25295	-0.04096	-1.04435	-0.39154	-1.07604	-1.10502	-0.91195	-0.66735
Q9Y4E8	Ubiquitin c	USP15 KIA/	0.012073	0.123255	-0.18709	0.055377	0.036712	-0.01097	0.025356	-0.16993	0.13493	0.03065
Q9Y4F9	Rho family-	RIPOR2 C6c	-0.68407	-0.46301	-0.43928	-0.41821	-0.51674	-0.6864	-0.39599	-0.36455	-0.61105	-0.8444
Q9Y4H4	G-protein-s	GPSM3 AG.	-2.32889	-2.01124	-1.68956	-2.26053	-2.8931	-1.98201	-2.34777	-2.4439	-1.79202	-1.70415
Q9Y4I1	Unconvent	MYO5A MY	-0.54609	-0.32193	-0.30738	-0.82312	-0.22487	-0.60145	-0.43911	-0.78746	-0.79796	-0.27727

Q9Y4K1	Beta/gamn	CRYBG1 AII	0.36607	0.289054	0.886886	0.791678	0.406522	0.526574	0.512054	0.373483	0.421243	0.308522
Q9Y4L1	Hypoxia up	HYOU1 GRI	0.26	0.275722	0.425721	0.516454	-0.04639	0.084889	0.307532	0.017074	0.189192	-0.05554
Q9Y4P8	WD repeat	WIPI2 CGI-!	-0.92903	-1.15634	-1.02359	-0.85681	-0.74193	-0.55254	-0.9301	-0.69188	-0.90866	-0.6763
Q9Y4W2	Ribosomal	LAS1L MST	-0.39497	0.034673	-0.28102	-0.52497	-0.18206	-0.33703	-0.45443	-0.48723	-0.22396	-0.01334
Q9Y4W6	AFG3-like p	AFG3L2	0.730984	0.911455	0.78137	0.689256	0.519579	0.935745	0.92075	0.529185	0.992193	0.605468
Q9Y4X5	E3 ubiquiti	ARIH1 ARI I	0.642619	0.599175	0.958688	0.83042	0.755235	0.831365	0.888577	0.688756	1.107726	1.124833
Q9Y4Z0	U6 snRNA-a	LSM4	-0.0739	0.114411	0.3725	0.171318	-0.23359	0.624491	-0.28157	0.335603	0.612977	0.596716
Q9Y508	E3 ubiquiti	RNF114 ZN	-1.19438	-0.93892	-0.99222	-0.91475	-0.66918	-0.635	-0.7997	-0.96605	-0.85651	-0.46609
Q9Y512	Sorting anc	SAMM50 S	-0.12172	0.152194	-0.5961	-1.70915	-1.33309	-0.81575	-0.22871	-0.92599	-0.1066	0.193411
Q9Y520	Protein PRI	PRRC2C BA	-0.83954	-0.59474	-0.28483	-0.51584	-0.30182	-0.27631	-0.53964	-0.56789	-0.19174	-0.3393
Q9Y530	ADP-ribose	OARD1 C6c	-0.51978	-0.92405	1.030861	0.984233	1.036712	1.453458	0.730813	1.072569	1.344383	1.276981
Q9Y570	Protein pho	PPME1 PM	-0.64702	-0.66021	-0.34515	-0.58402	-0.33358	-0.63991	-0.30907	-0.28288	-0.16598	-0.29184
Q9Y580	RNA-bindir	RBM7	-0.69515	-0.59474	-0.55984	0.114512	-0.39046	-0.31647	-0.70492	-0.80735	-0.45649	-0.66925
Q9Y5A7	NEDD8 ulti	NUB1 NYRI	-0.40296	-0.53703	-2.43155	-2.44423	-0.57755	-0.67807	-2.19366	-2.27791	-2.01372	-2.36981
Q9Y5A9	YTH domai	YTHDF2 HG	0.431612	0.638544	0.743165	1.066548	1.145826	1.360383	1.31166	1.072569	-0.03822	-0.07727
Q9Y5B0	RNA polym	CTDP1 FCP	-0.26812	-0.16572	-0.16186	-0.4583	0.036694	-0.39329	-0.12545	-0.14316	-0.35963	0.099414
Q9Y5B6	PAX3- and	PAXBP1 C2	-1.28011	-1.27399	-1.26459	-1.7794	-4.01495	-1.60145	-1.9898	-1.43812	-1.37474	-1.2402
Q9Y5B9	FACT comp	SUPT16H F	0.181998	-0.11775	-0.31221	-0.0707	0.405712	0.386058	-0.30666	-0.05842	0.147754	0.039289
Q9Y5J1	U3 small nu	UTP18 WD	-0.666	-0.96153	-1.20046	-1.84549	-1.10806	-0.90484	-1.45305	-0.85834	-0.74169	-0.60726
Q9Y5J6	Mitochond	TIMM10B I	-1.32526	-1.01264	-1.47175	-1.70867	-1.04874	-1.02272	-1.2739	-1.00836	-0.72407	-0.68307
Q9Y5J7	Mitochond	TIMM9 TIN	1.422082	1.524427	1.591776	1.598996	1.598604	1.583105	1.507798	1.547118	1.850669	1.739099
Q9Y5K5	Ubiquitin c	UCHL5 UCH	0.24669	0.836846	0.408832	0.646029	0.771696	0.691527	0.94834	0.697806	0.336781	0.295705
Q9Y5K6	CD2-associ	CD2AP	0.371969	0.513013	1.133954	1.024875	0.899933	0.566631	0.767339	0.655715	1.164677	1.260451
Q9Y5L0	Transportir	TNPO3 IPO	-0.48093	-0.32161	-0.15275	-0.01834	-0.05642	-0.14452	0.030989	-0.0397	0.505365	0.46579
Q9Y5L4	Mitochond	TIMM13 TI	-0.70195	-0.67878	-0.72495	-0.95374	-1.46769	-0.65261	-0.53943	-0.57129	-0.49246	-0.55663
Q9Y5M8	Signal reco	SRPRB PSE(	0.953805	0.915702	1.662144	1.371256	1.772952	1.743508	1.198071	0.880701	1.262248	1.155098
Q9Y5P4	Ceramide t	CERT1 CERT	-1.1242	-1.28018	-0.152	-0.39428	-1.61561	-0.77098	-1.29147	-1.12777	-0.23502	-0.68339
Q9Y5P6	Mannose-1	GMPPB	0.880898	0.989736	0.917982	0.851017	1.192512	0.953068	0.880434	0.641238	0.607638	0.718766
Q9Y5S1	Transient r	TRPV2 VRL	-1.48543	-1.57719	-1.45457	-1.35364	-3.69302	-1.79647	-2.38466	-1.30485	-1.89984	-1.80957
Q9Y5S9	RNA-bindir	RBM8A RBI	-0.6548	-0.64093	-0.74645	-0.65621	-0.70135	-0.39334	-0.63289	-0.78739	-0.53629	-0.64829
Q9Y5T5	Ubiquitin c	USP16 MST	-1.64889	-1.32768	-0.81616	-1.29033	-1.40523	-1.77627	-2.18611	-1.22276	-0.57851	-1.02483
Q9Y5V0	Zinc finger	ZNF706 HS	-1.75377	-1.34577	-1.19484	-1.40776	-1.50796	-1.4243	-1.36921	-1.27149	-0.86735	-1.28752
Q9Y5X1	Sorting nex	SNX9 SH3P	-3.13993	-2.24598	-2.32612	-1.89178	-1.31451	-2.02203	-1.66382	-2	-1.93546	-1.74882

Q9Y5X3	Sorting nex	SNX5	0.006049	-0.14336	-0.42809	-0.79021	-0.41009	-0.05004	-0.30035	-1.0115	-0.4119	-0.87299
Q9Y5Y2	Cytosolic F	NUBP2	-0.28751	-0.28221	-0.28741	-0.00486	-0.07274	0.260354	0.015267	0.137101	-0.10637	0.059789
Q9Y5Z4	Heme-bind	HEBP2 C6o	-0.01563	-0.21841	0.368712	0.390762	0.257623	0.112154	0.20346	0.235113	0.529657	0.520832
Q9Y5Z9	UbiA preny	UBIAD1 TEI	-1.35566	-1.12199	-1.61252	-1.4361	-1.14048	-1.27621	-1.45305	-1.6735	-1.76553	-1.74882
Q9Y606	tRNA pseu	PUS1 PP89	-1.02445	-1.01568	-0.89476	-0.52494	-1.01495	-0.97159	-0.73016	-0.77761	-1.02857	-0.7019
Q9Y608	Leucine-ric	LRRFIP2	0.14257	0.204465	0.197482	0.155334	0.140122	-0.06893	0.29853	0.052641	0.306466	0.427823
Q9Y617	Phosphose	PSAT1 PSA	-1.01217	-1.34156	-1.06378	-0.98751	-1.14048	-1	-1.38466	-1.23581	-0.89984	-0.84093
Q9Y624	Junctional	F11R JAM1	-0.72489	0.061076	-0.65486	-0.51457	-0.19903	-0.03877	-0.378	-0.50155	-0.39941	-1.18886
Q9Y657	Spindlin-1 (	SPIN1 OCR	-1.35766	-1.43581	-1.07676	-1.4344	-1.72561	-1.43044	-1.15216	-1.13359	-1.29456	-1.11378
Q9Y673	Dolichyl-ph	ALG5 HSPC	-0.64689	-0.7632	-0.87317	-0.62632	-1.07635	-0.84472	-0.93988	-1.16993	-1.31487	-1.32636
Q9Y676	28S riboso	MRPS18B C	-0.73495	-0.60064	-2.33953	-2.50093	-2.14197	-2.09547	-0.7997	-1.34792	-2.33269	-2.91974
Q9Y678	Coatomer s	COPG1 COI	0.716089	0.770341	0.457175	0.318777	0.51815	0.328929	0.400967	0.391661	0.139744	0.460747
Q9Y692	Glucocortic	GMEB1	-0.76553	-0.59474	-0.28821	-0.62632	-0.6574	-0.31647	-0.46007	-0.69188	-0.71823	-0.44184
Q9Y696	Chloride in	CLIC4	-0.62594	-0.62494	-0.8517	-0.9628	-0.37225	-0.34919	-1.07542	-0.8407	-1.06584	-0.67404
Q9Y697	Cysteine de	NFS1 NIFS	0.990879	0.714171	1.097975	0.865316	0.887927	1.257102	0.953206	0.874469	1.325695	1.221127
Q9Y6A4	Cilia- and fl	CFAP20 BU	-0.83868	-0.10246	-0.95141	-0.84567	-1.46857	-1.27095	-1.09636	-1.55258	-0.75537	-0.6036
Q9Y6A5	Transformi	TACC3 ERIC	-0.7912	-0.79667	-1.14768	-1.26053	-0.9417	-0.71895	-1.34496	-1.32318	-0.76553	-0.34295
Q9Y6B6	GTP-bindin	SAR1B SAR	0.543974	0.909488	-0.7176	-0.17306	-0.55552	-0.42968	-0.26919	-0.79737	-1.28011	-0.61402
Q9Y6D6	Brefeldin A	ARFGEF1 A	-0.53224	-0.47565	-1.29396	-1.27563	-1.26006	-1.02203	-1.1536	-1.20588	-1.7028	-1.65069
Q9Y6D9	Mitotic spi	MAD1L1 M	-0.06568	0.34233	-0.11416	-0.25139	0.381567	0.265955	0.268117	0.161102	0.203945	0.369811
Q9Y6E0	Serine/thre	STK24 MST	-1.49874	-1.12209	-1.55406	-1.8079	-1.16473	-0.82552	-1.00233	-1.0951	-1.52767	-1.25217
Q9Y6G9	Cytoplasmi	DYNC1LI1 D	-1.56728	-1.84519	-1.26899	-1.31464	-2.62281	-1.52117	-1.58627	-2.12988	-1.52953	-1.62816
Q9Y6I3	Epsin-1 (EH	EPN1	-2.09393	-2.6269	-2.78671	-2.07615	-1.59991	-1.22422	-1.19704	-1.55102	-2.83621	-2.31032
Q9Y6I4	Ubiquitin c	USP3	-1.89552	-1.92453	-1.43224	-1.73606	-0.269	0.016302	-0.02061	-0.04064	-0.24058	-0.1788
Q9Y6J0	Calcineurin	CABIN1 KIA	-0.25096	0.123255	0.564016	0.538214	0.766409	0.245625	0.411195	0.662965	0.559596	0.918058
Q9Y6K1	DNA (cytos	DNMT3A	-2.78784	-2.39341	-2.5006	-2.76164	-2.37347	-2.76612	-2.67896	-2.68153	-2.55523	-2.84646
Q9Y6K5	2'-5'-oligoa	OAS3 P/OK	0.160465	0.12683	0.069661	0.102877	-0.04532	-0.03877	0.235488	0.298844	-0.43084	-0.42392
Q9Y6K9	NF-kappa-E	IKBKG FIP3	0.032756	-0.21659	0.344717	0.093152	0.181596	0.419859	-0.29093	0.10587	0.046393	0.150534
Q9Y6M9	NADH deh	NDUFB9 LY	0.514573	0.193132	-0.69218	-0.04459	-0.88567	0.740241	0.348289	-0.02891	-0.97199	-1.0726
Q9Y6N5	Sulfide:qui	SQOR SQRI	1.319501	1.184581	1.168825	1.473074	0.762244	0.781018	1.057665	0.959358	0.583499	0.462562
Q9Y6R4	Mitogen-ac	MAP3K4 KI	-1.85042	-1.73697	-1.93147	-1.86821	-1.06075	-1.33015	-1.26919	-1.60224	-1.38702	-1.16882
Q9Y6V7	Probable A	DDX49	1.107997	1.057333	0.690206	0.407175	0.883903	0.90105	0.661349	0.222392	0.797895	1.108653
Q9Y6W5	Wiskott-Al	WASF2 WA	0.798669	0.342063	0.405256	0.354182	0.114333	0.475242	0.677432	0.567652	0.400824	0.462841

Q9Y6X4	Soluble lan	FAM169A I	-0.70723	-0.41245	-0.11704	-0.36282	0.149434	-0.16842	-0.32371	-0.23739	0.327575	0.030253
Q9Y6Y8	SEC23-inte	SEC23IP MS	0.187292	-0.06899	0.514292	0.459042	0.422988	0.050518	0.288473	0.36158	0.284175	0.632833
A6H8Y1	Transcripti	BDP1 KIAA	NA	NA	-3.57719	-3.67557	-2.95606	-4.34395	-3.74723	-3.16993	-3.21299	-3.44184
A8MSI8	LYR motif-c	LYRM9 C17	NA	NA	-2.10512	-3.14505	-2.51245	-2.95693	-2.9301	-1.80735	-3.25739	-3.21945
O60293	Zinc finger	ZFC3H1 CC	NA	NA	-3.6734	-3.52356	-3.59991	-3.65208	-3.61599	-3.02308	-3.16993	-3.26127
O75179	Ankyrin rep	ANKRD17 C	NA	NA	-3.62449	-3.38606	-3.79256	-4.34395	NA	NA	-3.84094	-3.45478
O95236	Apolipopro	APOL3	NA	NA	-1.93147	-2.84549	NA	NA	-2.89162	-2.848	-3.55695	-3.34873
P30456	HLA class I	HLA-A HLA	NA	NA	0.679735	0.676512	-1.26326	-0.04379	-1.85415	-1.93289	-2.80357	-2.75536
P30462	HLA class I	HLA-B HLAI	NA	NA	-2.15634	-1.9386	-1.43965	-1.71017	1.984051	1.874743	-3.67243	-2.6028
P32297	Neuronal a	CHRNA3 NA	NA	NA	-4.13906	-2.89129	-3.27798	-3.52083	-4.23266	-3.88982	-3.61353	-3.30433
P41743	Protein kin	PRKCI DXS1	NA	NA	-3.4021	-3.59756	NA	NA	-4.55459	-4.27684	-3.21299	-3.06333
P53539	Protein fos	FOSB G0S3	NA	NA	-3.57719	-3.67557	-2.69302	-3	-3.81762	-2.93289	NA	NA
P62699	Protein yip	YPEL5 CGI-	NA	NA	-3.62449	-3.14505	-4.01495	-3	-3.3322	-3.65535	-3.5025	-3.49075
P82921	28S ribosor	MRPS21 RI	NA	NA	-2.46529	-2.98751	-2.55552	-4.04439	-3.28157	-2.80735	-3.73383	-2.28264
Q03052	POU doma	POU3F1 O(	NA	NA	-4.07195	-3.38606	NA	NA	-1.48134	-1.36257	-3.45003	-3.95641
Q12983	BCL2/aden	BNIP3 NIP3	NA	NA	-3.4021	-3.26053	-4.01495	-1.68684	-2.05209	-2.72935	-3.00946	-3.70487
Q14493	Histone RN	SLBP HBP	NA	NA	-2.48699	-2.59756	-3.51245	-3.34395	-4.23266	-4.27684	-2.1913	-2.32636
Q4FZB7	Histone-lys	KMT5B SU\	NA	NA	-2.24598	-1.84549	-3.2076	-3.34395	-1.94973	-2.76783	-3.86507	-3.26127
Q5T3J3	Ligand-dep	LRIF1 C1orf	NA	NA	-4.28345	-4.38606	-3.27798	-4.23704	-3.43911	-2.97728	-2.3505	-3.1788
Q7L7X3	Serine/thre	TAOK1 KIA	NA	NA	-3.4021	-3.14505	-3.69302	-4.34395	-3.74723	-3.72935	-2.97199	-3.82517
Q7L8L6	FAST kinase	FASTKD5 K	NA	NA	-3.24598	-4.52356	-3.35199	-2.91511	-4.05209	-3.80735	-2.97199	-3.59384
Q8N228	Sex comb c	SCML4	NA	NA	-2.4021	-3.32193	-4.01495	-3.34395	NA	NA	-4.16993	-3.26127
Q8N668	COMM dor	COMMD1 (	NA	NA	-3.48699	-3.03814	-4.14048	-2.91511	NA	NA	-4.25739	-3.34873
Q8N8V4	Ankyrin rep	ANKS4B HA	NA	NA	-0.67963	-1.67557	-1.81855	-1.01097	-1	-1.09464	-1.13845	-0.58054
Q8N9B5	Junction-m	JMY	NA	NA	-3.94642	-3.45317	-3.79256	-4.04439	-2.03103	-3.07039	-4.55695	-3.64829
Q92817	Envoplakin	EVPL	NA	NA	-1.13906	-1.24558	-0.85845	-0.36491	-1.48134	-0.96605	-1.10764	-0.38294
Q96GM8	Target of E	TOE1	NA	NA	-3.4021	-3.0906	-3.69302	-3	-4.43911	-3.45372	NA	NA
Q96QU8	Exportin-6	XPO6 KIAA	NA	NA	-4.07195	-3.0906	NA	NA	-2.13955	-2.16993	-3.04793	-3.64829
Q96RT7	Gamma-tul	TUBGCP6 C	NA	NA	-3.10512	-3.26053	-2.95606	-3.72247	-2.9301	-3.97728	-3.39941	-3.21945
Q9BU61	NADH deh	NDUFAF3 C	NA	NA	-3.48699	-1.67557	-3.59991	-2.91511	-1.87276	-1.78746	-1.59918	-2.41799
Q9BWG4	Single-strar	SSBP4	NA	NA	-2.4021	-3.20163	-2.55552	-1.75899	-1.89162	-1.48543	-1.93546	-1.6763
Q9BX59	Tapasin-rel	TAPBPL	NA	NA	-3.72403	-3.32193	-4.14048	-4.58496	-3.01027	-3.45372	NA	NA
Q9C0B7	Transport a	TANGO6 KI	NA	NA	-3.53138	-3.20163	NA	NA	-3.28157	-3.33342	-4.45003	-4.64829

Q9H5V9	UPF0428 p	CXorf56	NA	NA	-3.36146	-3.9386	-3.07635	-2.95693	NA	NA	-2.76911	-3.6028
Q9HBF4	Zinc finger	ZFYVE1 DF(	NA	NA	-4.28345	-3.45317	-3.07635	-3.72247	-3.68012	-3.33342	-4.16993	-4.26127
Q9NPJ6	Mediator o	MED4 ARC:	NA	NA	-1.97679	-2.0906	-2.69302	-2.23704	-3.28157	-3.22239	-2.30319	-2.1788
Q9NR81	Rho guanin	ARHGEF3	NA	NA	-3.24598	-3.0906	-3.51245	-3	-3.28157	-3.22239	-4.45003	-3.82517
Q9UEU0	Vesicle trar	VTI1B VTI1	NA	NA	-1.85896	-1.91475	-1.22487	-1.81558	-1.91073	-1.80735	-1.59918	-1.64829
Q9UHQ4	B-cell recep	BCAP29 BA	NA	NA	-2.44392	-1.47045	-3.51245	-2.91511	-3.89162	-0.94386	-3.30319	-3.34873
Q9Y3E1	Hepatoma-	HDGFL3 HD	NA	NA	-3.32193	-3.52356	NA	NA	-4.43911	-4.39232	-3.21299	-3.44184
Q9Y679	Lipid dropl	AUP1	NA	NA	-3.88753	-4.14505	NA	NA	-2.09516	-2.97728	-4.45003	-3.49075
P30479	HLA class I	HLA-B HLAI	NA	NA	NA	NA	-4.27798	-2.65208	2.829115	2.619842	-3.08746	-2.46609
Q8N3J5	Protein pho	PPM1K PP2	NA	NA	NA	NA	-3.07635	-3.95693	-4.43911	-3.88982	-4.3505	-3.64829
Q9BXB4	Oxysterol-b	OSBPL11 O	NA	NA	NA	NA	-3.59991	-4.1375	-3.38466	-3.58496	-3.08746	-3.82517

Supplemental Table 2. Log2/median normalized TMT reporter intensities of phosphorylation sites of 24 h IL-7 stimulation of human primary T cell phosphoproteome

	pSite.in.Mas	Accessions	Protein names	Gene names	Control_1	Primed_1	Control_2	Primed_2	Control_3	Primed_3	Control_4	Primed_4	Control_5	Primed_5
1	S-10_P2935(	P29350	Tyrosine-protein	PTPN6 HCP I	0.3877	0.0660	-0.4241	-0.0636	0.0480	-0.3520	0.5108	0.2048	-0.5643	-0.7534
2	S-10_P4652	P46527	Cyclin-dependen	CDKN1B KIP	0.9955	1.0225	0.8536	1.0541	0.9147	0.9608	0.8809	0.9423	0.9497	0.8876
3	S-100_P231	P23193	Transcription elo	TCEA1 GTF2	0.3692	0.7076	-0.0250	0.1227	0.1352	0.1377	0.1701	0.1673	0.0593	0.1108
4	S-100_Q142	Q14258	E3 ubiquitin/ISG1	TRIM25 EFP	0.4366	0.4932	0.7220	0.5039	0.3317	0.5325	0.4503	0.6691	0.8362	0.7243
5	S-100_Q96R	Q96RR4	Calcium/calmodu	CAMKK2 CAI	-0.5370	-0.4022	-0.1959	-0.1797	0.1039	0.0119	-0.3232	-0.2298	-0.4409	-0.4994
6	S-100_Q9H3	Q9H307	Pinin (140 kDa nu	PNN DRS ME	-0.9820	-1.8046	-1.0664	0.5201	-0.2301	-0.7645	-0.8276	-0.3033	-1.7487	-1.0780
7	S-1004_O95	O95104	SR-related and C	SCAF4 KIAA1	-1.8329	-0.6752	0.9098	0.8737	0.1643	-0.4914	-0.0897	-1.0657	-0.4094	-0.2773
8	S-1004_Q9U	Q9UKV3	Apoptotic chrom	ACIN1 ACINU	0.2462	0.3432	0.1020	0.1960	0.0805	0.0497	0.2166	0.0626	0.1749	0.2740
9	S-1008_Q6Z	Q6ZU80	Centrosomal pro	CEP128 C14	-1.5175	-1.3496	-1.0108	-0.3826	-0.9092	-1.0032	-0.5514	-0.6494	-1.4675	-1.3722
10	S-101_Q9P1	Q9P1Y6	PHD and RING fir	PHRF1 KIAA1	-1.7948	-1.6646	-2.2140	-1.9562	-2.3192	-2.0392	-2.0953	-2.4564	-1.8671	-1.6630
11	S-1010_Q5V	Q5VUA4	Zinc finger protei	ZNF318 HRIH	0.6918	0.6389	1.3509	1.0627	1.2056	1.0683	0.9329	1.0235	1.4124	1.1749
12	S-1014_Q9U	Q9UKJ3	G patch domain-	GPATCH8 GI	0.3385	0.5274	0.3340	0.2313	0.2411	0.0059	0.2043	0.0227	0.2922	0.2015
13	S-1014_Q9U	Q9UQ35	Serine/arginine r	SRRM2 KIAA	0.8110	0.8722	1.1219	0.9233	0.9513	0.9213	0.8852	0.8350	0.9246	0.8821
14	S-1017_O95	O95785	Protein Wiz (Wid	WIZ ZNF803	0.8355	0.8122	0.7872	0.6896	0.6436	0.6163	0.7481	0.5236	0.8179	0.8309
15	S-102_O753	O75351	Vacuolar protein	VPS4B SKD1	0.7985	0.9098	0.3556	0.7094	0.3505	0.5092	0.5140	0.6981	0.0646	-0.0841
16	S-102_P170	P17096	High mobility grc	HMGA1 HM	-0.5715	0.0932	-0.0841	0.0177	0.1022	-0.2961	-0.0262	-0.0498	-0.3905	0.2358
17	S-102_P541	P54105	Methylosome su	CLNS1A CLC	-0.7823	-0.8143	-0.8873	-0.9528	-0.8194	-0.7848	-0.8912	-1.0278	-0.6888	-0.6711
18	S-102_Q9GZ	Q9GZT3	SRA stem-loop-ir	SLIRP C14orf	0.3918	0.3327	0.1214	0.1913	0.0426	0.1266	0.1503	0.2285	0.2535	0.1479
19	S-1021_P13	P13612	Integrin alpha-4	ITGA4 CD49	1.2154	1.3498	0.0844	0.0846	-0.2606	-0.0377	0.1303	-0.0247	-0.3974	-0.5837
20	S-1028_Q04	Q04637	Eukaryotic transl	EIF4G1 EIF4	-0.2696	-0.3490	0.2316	-0.5498	0.1310	-0.3108	0.2344	0.1590	0.0698	0.0419
21	S-1029_Q4L	Q4LE39	AT-rich interactiv	ARID4B BRC	-0.4804	-0.6489	-0.1745	-0.3621	-0.3874	-0.2780	-0.5066	-0.5076	-0.2571	-0.0578
22	S-103_P164	P16402	Histone H1.3 (His	H1-3 H1F3 H	0.8151	0.9250	1.9450	0.9929	1.5398	1.5175	1.8212	1.4004	2.3400	2.3520
23	S-103_Q9Y6	Q9Y6W5	Wiskott-Aldrich s	WASF2 WAV	1.1501	1.1636	1.5493	1.3567	0.9137	1.2537	1.0716	1.2677	1.3039	0.9456
24	S-1031_O95	O95466	Formin-like prote	FMNL1 C17c	0.6670	0.7598	1.0993	0.9941	0.6543	1.1979	1.0795	1.3414	0.7320	0.3690
25	S-1033_Q99	Q99683	Mitogen-activate	MAP3K5 ASI	-0.2458	-0.2326	-0.0907	-0.3735	-0.4514	-0.5124	-0.6480	-0.5317	-0.9883	-0.9783
26	S-1034_Q9B	Q9BSJ8	Extended synapt	ESYT1 FAM6	-0.8233	-0.7500	-1.0603	-0.9547	-1.1171	-1.3501	-1.0547	-1.3044	-1.4815	-1.3419
27	S-1039_Q92	Q92835	Phosphatidylinos	INPP5D SHIF	0.6474	0.7721	0.5842	0.5469	0.5877	0.4549	0.6485	0.7499	0.1793	0.3047
28	S-104_O151	O15173	Membrane-assoc	PGRMC2 DG	0.4577	0.4298	0.3597	0.3332	0.2076	0.2067	0.3240	0.2017	-0.0380	-0.0481
29	S-104_O431	O43159	Ribosomal RNA-p	RRP8 KIAA04	-1.9123	-2.2655	-2.3198	-2.1322	-2.5585	-2.2284	-2.2848	-2.4314	-2.6556	-1.9707
30	S-104_P319	P31943	Heterogeneous r	HNRNPH1 H	-0.9089	-0.9967	-0.8719	-0.8594	-1.2829	-0.9911	-1.0382	-1.2244	-1.2416	-1.3008
31	S-104_P490	P49006	MARCKS-related	MARCKSL1 M	-0.7885	-0.4726	1.1123	0.8746	0.8051	0.1550	1.3626	0.8938	1.0309	0.4979
32	S-104_Q151	Q15121	Astrocytic phosp	PEA15	0.6254	0.5563	1.7599	1.2583	1.3813	0.9881	1.5793	1.3765	0.8464	0.4805
33	S-104_Q8N2	Q8N201	Integrator compl	INTS1 KIAA1	-1.6687	-1.5630	-2.0533	-1.5655	-1.7746	-1.4260	-1.6178	-1.2606	-1.7663	-1.8359
34	S-1045_Q7Z	Q7Z3B3	KAT8 regulatory	KANSL1 CEN	-0.3116	-0.4051	-0.4157	-0.3193	-0.4364	-0.2654	-0.2228	-0.3669	-0.1932	-0.0299
35	S-105_P053	P05387	60S acidic ribosо	RPLP2 D11S	-0.2077	-0.4347	-0.1923	-0.9120	-0.2540	-0.4441	-0.3363	-0.4273	0.0423	-0.1778
36	S-105_P561	P56181	NADH dehydroge	NDUFV3	-2.3641	-2.2339	-1.1616	-1.8691	-1.0785	-1.5124	-1.0144	-1.0466	-1.1321	-1.1110
37	S-105_Q8IX	Q8IXQ4	GPALPP motifs-c	GPALPP1 KIA	0.5630	0.5799	0.5224	0.5871	0.3629	0.5461	0.3982	0.4171	0.8927	1.0532

38	S-105_Q9Y6	Q9Y6K1	DNA (cytosine-5)	DNMT3A	-0.0551	-0.1719	-0.1363	-0.1409	0.0930	-0.2647	0.0369	-0.1239	-0.4159	-0.1741
39	S-1052_Q7Z	Q7Z6E9	E3 ubiquitin-prot	RBBP6 P2PR	0.7261	0.8722	1.1363	1.3407	1.5580	1.1157	1.4254	1.2226	1.2220	1.4154
40	S-1057_Q8N	Q8N1G0	Zinc finger protei	ZNF687 KIAA	-0.7948	-0.7740	-0.3853	-0.4468	-0.5396	-0.3425	-0.3812	-0.4171	-0.3452	-0.2252
41	S-106_P245:	P24534	Elongation factor	EEF1B2 EEF1	-0.5742	-0.4407	-1.0253	-0.6692	-0.6654	-0.5274	-0.7470	-0.5273	-0.5876	-0.3378
42	S-106_P485!	P48556	26S proteasome	PSMD8	0.0995	0.0350	0.2065	-0.0197	0.2856	-0.2162	0.4020	0.0831	0.2730	0.4591
43	S-106_P490:	P49023	Paxillin	PXN	0.5234	0.4955	0.5171	0.5275	0.3592	0.4324	0.7945	0.7403	0.0599	-0.0052
44	S-106_Q5TD	Q5TDH0	Protein DDI1 hor	DDI2	0.1329	0.1159	-0.3991	-0.5087	-0.3330	-0.3653	-0.3528	-0.1345	-0.2727	0.0837
45	S-1061_P46:	P46100	Transcriptional re	ATRX RAD54	0.5196	0.3275	0.6194	0.9465	0.9721	0.8297	0.9302	0.8630	0.8413	0.7801
46	S-1067_Q9U	Q9UQE7	Structural mainte	SMC3 BAM I	-0.9433	-1.3448	-0.0052	0.0455	-0.5158	0.2778	-0.2737	0.0362	0.3310	0.2778
47	S-107_P525!	P52597	Heterogeneous r	HNRNPF HN	-0.7304	-0.6062	-0.5860	-0.6357	-0.5105	-0.4706	-0.5554	-0.5743	-0.7841	-0.8779
48	S-107_Q9UK	Q9UKY7	Protein CDV3 ho	CDV3 H41	0.2666	0.4039	0.2236	0.1598	0.3302	0.3990	0.1503	0.7154	0.4904	0.3217
49	S-1072_Q6P	Q6PD62	RNA polymerase	CTR9 KIAA01	-4.3183	-4.2391	-2.6735	-2.2712	-2.6026	-2.5295	-2.1176	-1.9758	-2.4930	-2.2914
50	S-1073_P27!	P27816	Microtubule-asso	MAP4	1.7052	1.5861	1.4320	1.3456	0.7525	0.8783	1.0026	0.7367	0.3788	0.4805
51	S-1074_Q9U	Q9UQE7	Structural mainte	SMC3 BAM I	-0.2893	-0.4803	-1.0686	-0.6834	-0.9991	-1.1736	-0.3055	-0.3729	-0.7206	-0.4564
52	S-1075_Q9U	Q9UMZ2	Synergin gamma	SYNRG AP1G	0.3241	0.0099	0.5857	0.7711	0.7721	0.9392	0.3762	0.8359	0.4241	0.1479
53	S-1076_Q9U	Q9UKJ3	G patch domain-	GPATCH8 GF	-0.4905	-0.4422	0.0094	-0.1797	0.2096	0.2049	-0.1707	0.0569	0.4501	-0.0299
54	S-1078_O95	O95248	Myotubularin-re	SBF1 MTMR	-0.5291	-0.5403	-0.9127	-0.7627	-0.8522	-0.5533	-0.7579	-0.7177	-0.6053	-0.6427
55	S-108_O751	O75152	Zinc finger CCCH	ZC3H11A KIA	0.7007	0.4662	0.8414	0.8521	0.6600	0.6919	0.8153	0.9239	0.5387	0.3340
56	S-108_Q148	Q14839	Chromodomain-l	CHD4	0.6940	0.5927	-0.1627	0.4951	0.6162	0.7725	0.1930	0.2985	-0.0982	0.0090
57	S-108_Q96L	Q96LT9	RNA-binding regi	RNPC3 KIAA	-0.5415	-0.5531	-0.7311	-0.4403	-0.7465	-0.6466	-0.6532	-0.6414	-0.8397	-0.6257
58	S-108_Q9H6	Q9H6Z4	Ran-binding prot	RANBP3	0.6567	0.8920	0.4657	0.1614	0.2016	0.0454	0.0955	0.1342	0.4319	0.1868
59	S-108_Q9Y4	Q9Y478	5'-AMP-activated	PRKAB1 AMI	1.2500	1.0628	0.3636	0.8688	0.7699	0.9699	1.1596	0.8613	0.0859	0.1030
60	S-1082_Q70	Q70J99	Protein unc-13 h	UNC13D	-0.9468	-1.2209	-1.5427	-1.6029	-0.7714	-1.0701	-1.1722	-1.0785	-1.3669	-1.9309
61	S-1083_Q9U	Q9UQ35	Serine/arginine r	SRRM2 KIAA	1.4350	1.7536	1.6961	1.7838	1.7745	1.5194	1.8614	1.6939	1.6050	1.6102
62	S-109_Q8ND	Q8NDT2	Putative RNA-bin	RBM15B OT	-0.0780	-0.1516	-0.0243	-0.2499	-0.1100	-0.1804	-0.0196	-0.2865	-0.2997	-0.2013
63	S-109_Q8NE	Q8NE71	ATP-binding cass	ABCF1 ABC5	0.4000	0.2156	0.5829	0.4397	0.6460	0.6734	0.4254	0.5161	0.5556	0.4412
64	S-109_Q96T	Q96T37	RNA-binding pro	RBM15 OTT	-3.6630	-3.1188	-2.5892	-2.7687	-2.6831	-2.8995	-2.1751	-2.3281	-2.8296	-3.2358
65	S-109_Q9BL	Q9BUQ8	Probable ATP-de	DDX23	0.3385	0.5189	0.2944	0.1975	0.3606	-0.0608	0.0623	0.0599	0.3018	0.2159
66	S-1093_Q9Y	Q9Y4F3	Meiosis regulato	MARF1 KIAA	-0.6487	-0.4528	-0.6970	-0.5369	-0.8357	-0.7645	-0.7318	-0.9939	-0.6556	-0.8245
67	S-1094_Q12	Q12888	TP53-binding pro	TP53BP1	0.8882	0.7085	0.9772	0.7432	1.0136	1.0897	0.8544	1.0743	1.1626	1.2467
68	S-1095_O95	O95248	Myotubularin-re	SBF1 MTMR	-0.4854	-0.3920	-0.4241	-0.7036	-0.4871	-0.3053	-0.8253	-0.3552	-0.2112	-0.2695
69	S-11_P1875	P18754	Regulator of chro	RCC1 CHC1	-0.3712	-0.1958	-0.4046	-0.4493	-0.4804	-0.3927	-0.4212	-0.3665	-0.3082	-0.4890
70	S-11_Q9268	Q92685	Dol-P-Man:Man(	ALG3 NOT N	1.1997	1.1072	0.7372	1.1983	1.2856	1.2985	1.1249	1.2311	0.5538	0.6384
71	S-110_Q134	Q13425	Beta-2-syntrophi	SNTB2 D16S	-0.1294	-0.1816	0.0136	-0.0361	-0.2032	-0.2048	-0.1528	-0.2867	-0.6618	-0.6570
72	S-1103_Q9U	Q9UK61	Protein TASOR (C	TASOR C3or	0.8732	0.8535	0.7980	0.6775	0.8342	0.9320	0.9022	0.9446	0.8642	0.7204
73	S-111_Q8W	Q8WWM7	Ataxin-2-like pro	ATXN2L A2D	-0.8137	-0.8699	-0.8680	-0.7417	-0.7250	-0.6954	-0.6789	-0.6514	-0.8814	-0.7383
74	S-1111_Q13	Q13428	Treacle protein (T	TCOF1	-0.3071	-0.1865	-0.0555	-0.0975	0.0987	0.1348	-0.0664	-0.0309	-0.5040	-0.5251
75	S-1112_Q08	Q08999	Retinoblastoma-	RBL2 RB2	-0.3026	-0.2248	-0.1947	-0.4812	-0.4539	-0.2943	-0.4086	-0.2958	-0.1740	-0.4267
76	S-1113_Q92	Q92794	Histone acetyltra	KAT6A MOZ	-1.0145	-0.9005	0.4045	0.4683	0.1887	0.3659	0.1781	0.1549	0.4624	0.3861

77	S-1114_Q5T	Q5T1M5	FK506-binding pr	FKBP15 KIAA	-1.2309	-1.0762	-1.2336	-1.3193	-1.1708	-1.2179	-1.2628	-1.4191	-1.2696	-1.1027
78	S-112_O950	O95049	Tight junction pr	TJP3 ZO3	-0.4332	-0.1033	-0.3211	-0.5113	-0.5639	-0.2708	-0.1064	-0.2120	-0.2355	-0.1264
79	S-112_Q9NV	Q9NVM6	DnaJ homolog su	DNAJC17	-4.1163	-4.8237	-2.9292	-2.0461	-2.9399	-3.6601	-2.4377	-2.4733	-3.4471	-3.9555
80	S-1124_Q9U	Q9UQ35	Serine/arginine r	SRRM2 KIAA	1.0126	0.9961	0.4351	-0.0143	0.3228	0.2126	0.7231	0.5394	0.5270	0.5124
81	S-113_O608	O60841	Eukaryotic transl	EIF5B IF2 KIA	0.1501	0.1849	0.2652	0.0605	0.1426	0.1287	-0.0949	-0.0939	0.2089	0.0870
82	S-113_Q151	Q15185	Prostaglandin E s	PTGES3 P23	0.3904	0.4296	-1.0126	-0.6579	-0.4092	-1.0515	-0.2722	-1.0561	-0.9832	-1.0247
83	S-113_Q167	Q16778	Histone H2B type	H2BC21 H2E	-0.5475	0.1562	0.4037	1.1219	1.4882	1.1409	0.9981	1.3498	0.0686	0.4563
84	S-113_Q9BR	Q9BRR9	Rho GTPase-activ	ARHGAP9	-0.5110	-0.6559	-0.6685	-0.5894	-1.4948	-1.1703	-0.9082	-0.9848	-1.0927	-0.9090
85	S-113_Q9H1	Q9H1E3	Nuclear ubiquito	NUCKS1 NU	-0.1464	-0.1624	-0.1940	-0.4997	0.1266	-0.3061	-0.4862	-0.3863	0.1668	0.1632
86	S-1132_Q9U	Q9UQ35	Serine/arginine r	SRRM2 KIAA	-0.2433	-0.4052	0.3952	0.5207	0.3061	0.3380	0.5148	0.7565	0.3504	0.2886
87	S-114_P313	P31323	cAMP-dependen	PRKAR2B	-0.6802	-0.7721	-0.6194	-0.7269	-1.3423	-1.1939	-0.8579	-0.8058	-0.9961	-1.1648
88	S-114_Q9NF	Q9NP64	Nucleolar protei	ZCCHC17 PS	-0.2479	0.1482	0.2987	0.7511	0.6705	0.4543	0.5226	0.0000	-0.1010	0.1268
89	S-114_Q9UI	Q9UNZ2	NSFL1 cofactor p	NSFL1C UBX	1.4774	1.3854	1.5251	1.5082	1.5951	1.6725	1.3232	1.4578	2.0451	2.0761
90	S-1140_P20	P20701	Integrin alpha-L (	ITGAL CD11	0.1895	0.1769	-0.4727	0.0369	-1.2784	-0.2870	-0.2381	-0.1667	-1.1122	-1.1910
91	S-1144_P78	P78312	Protein FAM193	FAM193A C	-0.0704	-0.1200	0.2414	-0.2165	-0.1360	0.0739	-0.1275	-0.1013	-0.1150	-0.2207
92	S-1145_Q14	Q14980	Nuclear mitotic a	NUMA1 NM	-2.2140	-2.2549	-2.0664	-1.9087	-1.7809	-2.4061	-2.0039	-2.2388	-2.4857	-2.1939
93	S-115_Q096	Q09666	Neuroblast differ	AHNAK PM2	-1.2711	-1.3852	-0.9744	-1.1003	-1.6015	-1.1018	-1.3628	-1.3281	-1.5837	-1.5703
94	S-1152_Q9U	Q9UQ35	Serine/arginine r	SRRM2 KIAA	-0.8622	-0.8759	0.0494	-0.0786	-0.0496	-0.1854	0.0078	0.0932	0.0499	0.1880
95	S-1155_O15	O15085	Rho guanine nuc	ARHGEF11 K	-1.7036	-1.6894	-1.5155	-1.6412	-1.6138	-1.2744	-1.8048	-1.7127	-1.3271	-1.0944
96	S-1157_Q5U	Q5UIP0	Telomere-associ	RIF1	-0.5213	-0.7128	-0.1971	0.0796	-0.2150	-0.0180	-0.2274	-0.1581	-0.2202	-0.1504
97	S-1158_P61	P61129	Zinc finger CCCH	ZC3H6 KIAA	-0.6544	-0.7816	-0.7863	-0.4228	-1.0028	-0.8829	-0.5029	-0.6323	-0.6393	-0.7297
98	S-116_Q151	Q15121	Astrocytic phosp	PEA15	0.1493	0.1210	1.0459	0.4836	0.8395	0.0654	0.7772	0.3688	0.3049	-0.0511
99	S-116_Q153	Q15311	RalA-binding pro	RALBP1 RLIP	-0.2915	-0.3350	-0.5021	0.0106	0.3184	-0.6621	0.5172	-0.0529	-0.4268	-0.2993
100	S-116_Q8N3	Q8N3X1	Formin-binding p	FNBP4 FBP3	-0.4255	-0.8438	-0.4496	-0.4521	-0.5851	-0.7303	-0.5801	-0.5781	-0.3206	-0.1974
101	S-116_Q9H4	Q9H4Z3	mRNA (2'-O-met	PCIF1 C20or	0.9791	0.7083	0.8969	0.6356	0.8415	1.0155	1.0302	0.7403	1.2254	0.9884
102	S-116_Q9UI	Q9UH99	SUN domain-con	SUN2 FRIGG	-0.0441	0.2317	0.1566	0.4292	-0.0727	-0.1973	0.2981	0.0377	-0.1799	0.0306
103	S-1165_P20	P20701	Integrin alpha-L (	ITGAL CD11	0.5347	0.4452	-0.4932	0.4186	-0.1381	-0.7051	0.4617	-0.3948	-1.3109	-0.9936
104	S-117_P368	P36871	Phosphoglucomu	PGM1	-0.1767	-0.1479	-0.4611	-0.2352	-0.5831	-0.2424	-0.4828	-0.3261	-0.3436	-0.3200
105	S-117_Q128	Q12846	Syntaxin-4 (Rena	STX4 STX4A	0.5095	0.6439	0.2485	0.3308	0.5822	0.9023	0.8409	0.9792	0.4737	0.3502
106	S-117_Q5QJ	Q5QJE6	Deoxynucleotidy	DNTTIP2 ERI	-0.6123	-0.7256	-0.9445	-0.7387	-1.0364	-0.6249	-0.6258	-0.8465	-0.9987	-0.9808
107	S-1176_Q9U	Q9UKV3	Apoptotic chrom	ACIN1 ACIN	-3.0857	-2.5728	-1.4511	-0.8822	-1.1179	-1.5820	-1.2350	-1.0216	-1.3370	-1.0013
108	S-1179_Q7Z	Q7Z6E9	E3 ubiquitin-prot	RBBP6 P2PR	0.2470	0.1789	0.2432	0.3137	0.1659	0.2279	0.1550	0.2595	0.3007	0.2628
109	S-1179_Q9N	Q9NRY4	Rho GTPase-activ	ARHGAP35	0.1783	0.3981	-0.0350	0.1373	-0.2193	-0.1537	-0.2078	-0.1079	-0.1696	0.0982
110	S-118_Q86U	Q86UU0	B-cell CLL/lymph	BCL9L DLNB	0.2133	0.5046	0.5647	0.6575	0.7094	0.4928	0.7519	0.6749	0.3880	0.5628
111	S-118_Q8W	Q8WYH8	Inhibitor of grow	ING5	0.2546	0.0839	-0.1019	-0.3507	0.1478	-0.0701	-0.1162	-0.2865	0.2458	0.3989
112	S-118_Q929	Q92934	Bcl2-associated a	BAD BBC6 B	0.2485	0.2392	0.1992	0.2053	0.2473	0.2581	0.2541	0.4166	0.1622	0.2607
113	S-1184_O14	O14497	AT-rich interacti	ARID1A BAF	-0.0553	-0.1603	0.1992	0.1819	0.2442	0.5013	0.4264	0.4291	0.1423	0.1256
114	S-119_O147	O14737	Programmed cell	PDCD5 TFAR	-0.5688	-0.7020	-0.7349	-0.7963	-0.5998	-0.3905	-0.6098	-0.4690	-0.5245	-0.3657
115	S-119_P558	P55884	Eukaryotic transl	EIF3B EIF3S	-2.2652	-2.2443	-1.2635	-1.5708	-1.1708	-0.9589	-1.3429	-1.6205	-1.2633	-0.8498

116	S-119_Q8W'	Q8WW12	PEST proteolytic	PCNP	-0.4406	-0.2852	-0.4102	-0.6551	-0.3588	-0.0842	-0.3594	-0.1378	-0.1711	-0.0944
117	S-1195_Q29	Q29RF7	Sister chromatid	PDS5A KIAA(	-1.2140	-1.8598	-0.9028	-1.2082	-1.3284	-1.2424	-1.8603	-1.5743	-1.0571	-0.8291
118	S-12_P3561:	P35611	Alpha-adducin (E	ADD1 ADDA	-0.5501	-0.7797	-0.1406	-0.1657	-0.3146	0.1171	-0.1547	0.0613	-0.0625	-0.2297
119	S-12_Q1543	Q15435	Protein phospha	PPP1R7 SDS:	-1.5742	-1.0553	-0.3436	-1.0233	-0.8791	-1.0119	-1.1176	-1.1211	-0.7575	-0.8429
120	S-12_Q9UKT	Q9UKT5	F-box only protei	FBXO4 FBX4	-1.0036	-0.7988	-1.3409	-1.3507	-1.7250	-1.9162	-1.1722	-1.5473	-1.7886	-1.5049
121	S-120_O952	O95218	Zinc finger Ran-b	ZRANB2 ZIS	-0.1849	0.4106	0.4335	-0.0452	0.0373	0.1722	-0.0183	0.1942	0.3403	0.4506
122	S-120_P226'	P22670	MHC class II regu	RFX1	-0.3319	-0.4437	-0.1314	-0.1302	-0.2829	-0.2076	-0.2319	-0.1736	-0.3029	-0.0524
123	S-120_Q32N	Q32MZ4	Leucine-rich repe	LRRFIP1 GCF	-0.8576	-0.9752	-0.7164	-0.8803	-1.1118	-1.1059	-1.1448	-1.0398	-1.2112	-0.4617
124	S-1202_Q9P	Q9P1Y6	PHD and RING fir	PHRF1 KIAA:	0.1735	0.1149	0.1697	0.1373	-0.0401	0.1762	0.0026	0.1848	0.1174	0.2788
125	S-121_P062:	P06239	Tyrosine-protein	LCK	-0.0704	-0.2721	-0.4554	-0.2951	-0.2150	0.0511	-0.0705	0.1522	-0.3585	-0.6468
126	S-121_Q9H7	Q9H7D7	WD repeat-conta	WDR26 CDW	-0.3595	0.0692	0.0824	-0.0748	-0.0824	-0.1097	-0.0196	-0.1718	-0.0941	-0.1490
127	S-121_Q9NF	Q9NR30	Nucleolar RNA h	DDX21	0.5639	0.5751	0.1029	0.1998	0.5637	0.3110	0.4956	0.2982	0.0784	0.0169
128	S-121_Q9Y5	Q9Y5J1	U3 small nucleol	UTP18 WDR	0.3484	0.6858	0.6558	1.1598	1.1684	1.1246	0.9424	1.0555	0.7429	1.0898
129	S-1217_Q13	Q13813	Spectrin alpha ch	SPTAN1 NEA	-0.6681	-0.4676	-0.3174	-0.3725	-0.1703	-0.0909	-0.4385	-0.3581	0.4033	0.4251
130	S-1219_Q9U	Q9UQ35	Serine/arginine r	SRRM2 KIAA	2.1922	2.0888	1.7899	2.0464	1.9599	1.6012	2.3727	1.8264	1.7167	1.9426
131	S-122_P112'	P11274	Breakpoint clust	BCR BCR1 D:	0.8171	0.9900	1.1500	1.0247	0.8670	0.8991	0.7975	0.9518	0.7035	0.4814
132	S-122_Q018	Q01831	DNA repair prote	XPC XPCC	-2.4018	-2.5338	-2.5518	-2.5550	-2.3192	-2.3219	-2.5103	-2.1479	-2.0223	-1.9211
133	S-122_Q5VZ	Q5VZL5	Zinc finger MYM-	ZMYM4 KIA/	-0.3162	-0.2641	-0.1365	-0.1979	-0.2728	-0.2147	-0.3728	-0.3673	-0.2050	-0.3016
134	S-122_Q8W:	Q8WXI9	Transcriptional r	GATAD2B KI	-0.9226	-0.7074	-0.7331	-0.8370	-0.7403	-0.6692	-1.2381	-0.9549	-0.1579	-0.1362
135	S-122_Q995	Q99576	TSC22 domain fa	TSC22D3 DS	-0.3049	-0.0727	0.1472	0.4644	0.3155	0.2997	0.3438	0.6748	0.0155	0.3653
136	S-1222_Q96	Q96T58	Msx2-interacting	SPEN KIAA0!	0.4485	0.4313	0.6670	0.7191	0.6222	0.7319	0.3941	0.4639	0.7003	0.6084
137	S-1225_Q7Z	Q7Z401	C-myc promoter-	DENND4A IF	-0.2015	-0.0879	-0.3881	-0.0994	0.0174	-0.2388	-0.3846	-0.2939	-0.4250	-0.4423
138	S-1228_Q9B	Q9BYW2	Histone-lysine N-	SETD2 HIF1	-0.3456	-0.1690	-0.4397	-0.2165	-0.2150	0.0425	-0.0967	0.0788	-0.1052	-0.2710
139	S-124_P220:	P22087	rRNA 2'-O-methy	FBL FIB1 FLR	0.2621	0.2560	0.2555	0.2716	1.2317	1.0338	0.8774	0.8012	0.1246	0.0605
140	S-1246_Q6F	Q6F5E8	Capping protein,	CARMIL2 LR	0.1213	0.0088	0.4755	0.2493	0.3036	0.5810	0.1884	0.1103	0.4783	0.5507
141	S-125_P067	P06748	Nucleophosmin (	NPM1 NPM	-0.8110	-0.7011	-0.7886	-0.8491	-0.8991	-0.6829	-0.8077	-0.6580	-0.4094	-0.3358
142	S-125_P558	P55884	Eukaryotic transl	EIF3B EIF3S!	-4.2827	-4.1188	-2.7845	-1.9911	-2.7936	-2.4061	-2.5478	-2.4818	-2.8766	-2.6149
143	S-125_Q997	Q99733	Nucleosome asse	NAP1L4 NAF	0.1687	0.3424	-0.0774	-0.5420	0.0918	-0.1487	-0.2895	0.0672	0.3647	0.6695
144	S-125_Q9GZ	Q9GZT9	Egl nine homolog	EGLN1 C1ori	0.7913	0.7778	0.8237	0.6230	0.5785	0.2900	0.5811	0.3771	0.4652	0.2788
145	S-125_Q9N(	Q9NQP4	Prefoldin subuni	PFDN4 PFD4	0.3821	0.1311	0.3415	0.2130	0.1226	0.3636	0.1303	0.2114	0.5279	0.0318
146	S-1257_Q96	Q96BY6	Dedicator of cyto	DOCK10 KIA	0.9475	1.0479	0.8622	0.7763	1.4058	1.5232	1.0869	1.1855	1.4095	1.1386
147	S-126_Q68D	Q68DK7	Male-specific letl	MSL1 MSL1L	-0.0590	-0.0302	-0.2385	-0.0692	-0.4166	-0.3388	-0.1884	-0.3338	-0.1814	-0.2587
148	S-126_Q8N5	Q8N5C8	TGF-beta-activat	TAB3 MAP3I	-1.0742	-1.0033	-0.6718	-0.7358	-1.0439	-0.6978	-1.0842	-0.5630	-0.6495	-0.5837
149	S-1267_Q9B	Q9BQG0	Myb-binding pro	MYBBP1A P:	0.4459	0.1492	0.2494	-0.0215	0.8364	0.7373	0.7025	0.7004	0.4718	0.0293
150	S-1268_Q3T	Q3T8J9	GON-4-like prote	GON4L GON	-1.4406	-1.4834	-1.3922	-1.4204	-1.2129	-1.3520	-1.5863	-1.4439	-1.4391	-1.5232
151	S-127_Q5T8	Q5T8P6	RNA-binding pro	RBM26 C13(	-0.1265	-0.0961	0.0156	-0.0655	-0.3330	-0.0453	-0.1722	-0.1194	-0.0210	-0.0578
152	S-1272_Q56	Q562E7	WD repeat-conta	WDR81	-0.1325	-0.0078	-0.1983	0.0053	-0.0746	-0.0180	-0.0289	-0.1926	0.2535	0.3360
153	S-128_Q5JSI	Q5JSP0	FYVE, RhoGEF an	FGD3 ZFYVE	0.1879	0.1592	-0.5065	-0.2670	-0.2628	-0.3539	-0.5066	-0.6159	-0.5189	-0.3104
154	S-128_Q96T	Q96T37	RNA-binding pro	RBM15 OTT	1.0260	1.0415	1.1229	0.8293	0.7828	1.0141	0.8802	0.9391	0.8948	0.7290

155	S-1287_Q96	Q96T58	Msx2-interacting	SPEN KIAA0!	0.5209	0.5517	0.7246	0.5891	0.6670	0.8447	0.5750	0.6910	0.8392	0.8112
156	S-129_O754	O75475	PC4 and SFRS1-ir	PSIP1 DFS70	1.0206	1.1745	0.6664	1.0291	1.0788	0.7039	1.2800	0.9271	0.8377	0.8800
157	S-129_O755	O75533	Splicing factor 3E	SF3B1 SAP1!	-0.3782	-0.5761	-0.7038	-0.8855	-0.4897	-0.7051	-0.5196	-0.4356	-0.4875	-0.5895
158	S-129_Q96J'	Q96JY6	PDZ and LIM don	PDLIM2 PP6	-0.0422	-0.1176	-0.1511	-0.3644	-0.3682	-0.2672	-0.3561	-0.1996	-0.5452	-0.4146
159	S-1290_Q13	Q13459	Unconventional	MYO9B MYF	1.1468	0.7752	1.1181	0.8112	1.0073	0.8983	1.2816	1.1895	0.5863	0.2978
160	S-1295_Q9B	Q9BV73	Centrosome-assc	CEP250 CEP:	-0.4880	-0.2042	0.1127	0.1502	0.0046	0.2215	-0.0569	-0.0185	-0.0249	-0.2267
161	S-130_Q49A	Q49A26	Putative oxidore	GLYR1 HIBDI	0.1933	0.2087	0.4630	0.4826	1.0826	0.7796	0.6743	0.7414	0.5743	0.5153
162	S-130_Q9UI(	Q9UI08	Ena/VASP-like pr	EVL RNB6	-0.3387	-0.5877	-0.0785	-0.2144	-0.0401	0.2228	-0.1591	0.0407	0.2524	0.1102
163	S-1305_Q29	Q29RF7	Sister chromatid	PDS5A KIAA(	0.7007	0.3726	0.9613	0.8233	0.9485	0.8879	0.8219	0.9621	0.9850	0.5820
164	S-131;154_C	Q8N4S0;Q8	Coiled-coil doma	CCDC82 HT0	1.1990	1.2041	1.3546	1.2790	1.4530	1.4052	1.2405	1.4206	1.6536	1.4633
165	S-1315_Q6F	Q6F5E8	Capping protein,	CARMIL2 LR	0.3081	0.3862	0.3047	0.1227	0.1741	0.2481	0.0882	0.0932	0.2912	0.4066
166	S-132_O751	O75152	Zinc finger CCCH	ZC3H11A KI/	-0.2415	-0.1739	-0.1487	-0.4228	-0.3874	-0.2888	-0.1106	-0.3033	-0.1711	-0.3807
167	S-132_P049:	P04920	Anion exchange	SLC4A2 AE2	-2.3183	-1.8719	-1.8284	-1.6412	-1.7746	-1.1603	-1.4034	-1.5428	-2.1378	-1.9114
168	S-132_Q7Z2	Q7Z2E3	Aprataxin (EC 3.6	APTX AXA1	-1.3183	-1.5663	-1.7279	-1.3237	-1.2874	-1.7670	-0.8626	-1.6732	-1.8343	-1.6835
169	S-132_Q8W'	Q8WVK2	U4/U6.U5 small	SNRNP27	-0.6687	-0.9361	0.6112	-0.0342	0.4816	-0.4320	0.8852	-0.0899	-0.3602	-0.3184
170	S-132_Q9H9	Q9H981	Actin-related prc	ACTR8 ARP8	-1.3595	-1.1260	-0.9771	-1.2755	-1.0326	-1.1973	-1.0981	-1.2753	-1.4077	-1.1362
171	S-132_Q9Y2	Q9Y2K6	Ubiquitin carbox	USP20 KIAA:	-1.1932	-1.0033	-0.5876	-0.3416	-0.8064	-0.7295	-0.8299	-1.0657	-0.7730	-0.7952
172	S-1322_Q14	Q14669	E3 ubiquitin-prot	TRIP12 KIAA	-1.1524	-1.3732	-1.4947	-1.3326	-1.3377	-1.1973	-1.3594	-1.2569	-1.5720	-1.3329
173	S-1327_Q8N	Q8N201	Integrator compl	INTS1 KIAA1	0.7681	0.9198	0.0290	0.5386	0.2128	0.1945	0.5017	0.3219	-0.0078	0.1019
174	S-1328_P46:	P46100	Transcriptional r	ATRX RAD54	0.1879	-0.0565	-0.2939	0.2037	0.3520	0.5314	0.0384	0.3923	0.2643	0.4173
175	S-1328_Q13	Q13464	Rho-associated p	ROCK1	-0.5265	-0.8985	-0.5751	-0.3060	-0.8556	-0.5775	-0.4505	-0.7715	-0.8766	-0.3362
176	S-1328_Q7Z	Q7Z6E9	E3 ubiquitin-prot	RBBP6 P2PR	1.2138	1.1764	1.2690	0.8373	1.1461	1.1596	1.0136	0.9311	1.2187	1.0362
177	S-133_O754	O75494	Serine/arginine-r	SRSF10 FUSI	0.4288	0.4492	0.5709	0.6299	0.9234	0.1971	1.1196	0.4782	0.3248	0.6384
178	S-133_P296!	P29692	Elongation factoı	EEF1D EF1D	0.3877	0.4908	-0.4425	-0.4861	-0.0961	-0.6574	-0.0355	-0.3338	-0.4146	-0.1013
179	S-133_Q6NY	Q6NYC8	Phostensin (Prot	PPP1R18 HK	1.7970	1.9858	1.6515	1.4773	1.6017	1.2481	1.8703	1.5214	1.2270	1.1333
180	S-133_Q8NE	Q8NEY8	Periphilin-1 (CDC	PPHLN1 HSP	0.3936	0.3425	0.0878	0.2251	-0.0085	0.0843	0.2559	0.0302	0.0849	0.1154
181	S-1334_Q9N	Q9NTI5	Sister chromatid	PDS5B APRII	-0.8297	-1.1116	-1.0841	-0.7718	-1.0176	-0.6457	-0.9377	-0.8658	-0.9351	-1.0834
182	S-134_Q9U(	Q9UGY1	Nucleolar proteir	NOL12	-0.6658	-0.6489	-0.6536	-0.7036	-0.7281	-0.7394	-0.7339	-0.8911	-0.8553	-0.6447
183	S-1341_Q13	Q13464	Rho-associated p	ROCK1	0.2115	0.2808	1.3559	1.2198	1.3841	0.5478	1.4795	1.0008	0.5485	0.1185
184	S-1342_Q9U	Q9UIG0	Tyrosine-protein	BAZ1B WBS(	1.6613	1.7032	1.0947	1.5915	1.9297	1.4907	1.6393	1.7567	0.1353	0.1197
185	S-135_Q9UK	Q9UKM9	RNA-binding pro	RALY HNRPC	-1.5199	-1.7365	-2.0995	-2.2138	-2.4156	-2.1724	-1.9990	-2.2731	-2.3760	-2.4880
186	S-1350_Q13	Q13428	Treacle protein ('	TCOF1	0.1751	0.0802	0.0967	0.2972	0.3330	0.3546	0.4109	0.3584	-0.2203	0.0353
187	S-1358_Q9N	Q9NTI5	Sister chromatid	PDS5B APRII	0.0029	-0.1384	0.5526	0.4195	0.4836	0.5924	0.6907	0.4998	0.5127	0.8348
188	S-136_Q024	Q02446	Transcription fac	SP4	0.7881	0.8618	0.2092	0.3822	0.0228	0.3831	0.0257	0.4166	-0.1249	-0.4423
189	S-136_Q144	Q14498	RNA-binding pro	RBM39 HCC	0.1312	0.3160	-0.1552	0.1316	-0.1921	0.0745	-0.1783	0.1640	-0.0124	-0.2129
190	S-136_Q8IV(	Q8IV63	Inactive serine/tl	VRK3	0.2424	0.2799	0.1594	0.4119	0.3534	0.2543	0.3792	0.3414	0.3957	0.2724
191	S-136_Q8N9	Q8N9T8	Protein KRI1 hon	KRI1	0.3670	0.2907	0.5197	0.2164	-0.1224	-0.0309	-0.0420	-0.0990	-0.4878	-0.4317
192	S-136_Q8TB	Q8TB72	Pumilio homolog	PUM2 KIAA(	0.5529	0.4150	0.3377	0.2000	0.2357	0.1988	0.2756	0.1244	0.2013	-0.0848
193	S-137_O608	O60841	Eukaryotic transl	EIF5B IF2 KI/	-0.4430	-0.3114	-0.0697	-0.2021	0.2395	0.2266	0.0359	0.0599	0.2556	0.0179

194	S-137_Q005	Q00537	Cyclin-dependen	CDK17 PCTA	1.0945	0.8072	1.0234	0.7752	1.1911	1.2450	1.2470	1.1251	1.0568	0.5392
195	S-137_Q929	Q92974	Rho guanine nuc	ARHGEF2 KI/	-2.7454	-2.7930	-2.6536	-2.5975	-2.2739	-2.8344	-2.7867	-2.5607	-2.4497	-2.8268
196	S-1370_A6N	A6NC98	Coiled-coil doma	CCDC88B BR	-0.4357	-0.6594	-0.5095	-0.8181	-0.7039	-0.4706	-0.3611	-0.2551	-0.6930	-0.6670
197	S-1375_Q02	Q02880	DNA topoisomer	TOP2B	-0.1812	-0.2082	-0.7350	-0.5847	-0.6993	-0.7235	-0.5202	-0.7535	-0.8045	-0.7604
198	S-1378_Q13	Q13428	Treacle protein ('	TCOF1	1.3577	1.4260	0.8581	1.0483	0.3521	0.8116	0.9000	0.9616	0.3234	0.3756
199	S-138_O756	O75683	Surfeit locus prot	SURF6 SURF	-0.9573	-0.6699	-1.2165	-1.1128	-0.9378	-1.4542	-0.9155	-1.1278	-1.2172	-1.1706
200	S-138_O959	O95999	B-cell lymphoma	BCL10 CIPER	-0.6919	-1.0370	-0.0105	0.2964	0.1428	0.0894	0.1160	0.1508	-0.2052	-0.0985
201	S-138_Q5VT	Q5VTR2	E3 ubiquitin-prot	RNF20 BRE1	0.5372	0.7144	-0.2635	0.0813	-0.2366	-0.1454	-0.2597	-0.2424	-0.2949	-0.4635
202	S-138_Q96C	Q96C57	Protein CUSTOS	CUSTOS C12	-0.2015	-0.6507	2.2283	2.3992	1.9745	2.1890	2.3219	2.3090	1.8690	2.0459
203	S-138_Q9HA	Q9HAZ1	Dual specificity p	CLK4	-1.1444	-1.2183	-1.1971	-1.4444	-1.0213	-1.0826	-1.0302	-1.1996	-1.0409	-0.9834
204	S-1381_Q6F	Q6F5E8	Capping protein,	CARMIL2 LR	0.7118	0.5816	0.6802	0.6242	0.6330	0.7102	0.5802	0.6022	0.7297	0.5015
205	S-1387_Q7Z	Q7Z591	Microtubule orga	AKNA KIAA1	0.1799	0.5920	1.0347	1.0558	1.0953	0.9752	0.8936	0.9028	0.6655	1.0512
206	S-139_P497	P49736	DNA replication l	MCM2 BM2	0.2437	0.2196	0.1112	-0.0624	-0.4759	-0.4707	-0.0119	-0.2625	0.0715	-0.0978
207	S-139_Q6NY	Q6NYC8	Phostensin (Prot	PPP1R18 HK	-0.1973	-0.6334	0.4543	0.4644	0.3735	0.3612	0.5863	0.5352	0.0883	-0.0155
208	S-139_Q9Y2	Q9Y266	Nuclear migratio	NUDC	-0.2161	-0.1615	-0.6098	-0.3828	-0.6567	-0.1372	-0.4224	-0.2209	-0.2806	-0.4564
209	S-1395_Q7Z	Q7Z6Z7	E3 ubiquitin-prot	HUWE1 KIA/	-1.2224	-1.2209	-1.4684	-1.6863	-1.5970	-1.6787	-1.6258	-1.7077	-1.4820	-1.1193
210	S-14_P5158	P51580	Thiopurine S-me	TPMT	0.7628	0.8527	0.7811	0.8132	0.3287	0.6222	0.4456	0.5140	0.7722	0.2978
211	S-14_Q9H6Y	Q9H6Y2	WD repeat-conta	WDR55	-0.8831	-0.9160	-1.7092	-2.3161	-2.0681	-1.8641	-2.0534	-1.8141	-1.3254	-1.6605
212	S-140_P161	P16104	Histone H2AX (H	H2AX H2AF	0.0162	0.3257	0.9170	1.7695	1.6843	0.9654	1.8492	1.9203	0.4997	0.8751
213	S-140_P497	P49759	Dual specificity p	CLK1 CLK	-1.2224	-1.3060	-1.5673	-1.3016	-1.1462	-1.4706	-1.5066	-1.4397	-1.3669	-1.7212
214	S-140_Q8NE	Q8NE71	ATP-binding cass	ABCF1 ABC5	0.8203	0.5309	0.7419	0.6700	0.8394	1.0080	0.7451	0.8790	0.7851	0.7123
215	S-1400_Q02	Q02880	DNA topoisomer	TOP2B	0.7283	0.8782	0.3571	0.4292	0.3462	0.5034	0.5172	0.3677	0.6440	0.4823
216	S-1401_P21	P21359	Neurofibromin (I	NF1	-0.4162	-0.7092	1.3369	0.4463	1.4836	0.2556	1.4579	0.7854	0.5983	0.1766
217	S-1404_Q9U	Q9UQ35	Serine/arginine r	SRRM2 KIAA	0.5421	0.2836	0.7689	0.3958	0.5599	0.6193	0.5432	0.5603	0.5845	0.6368
218	S-141_P188	P18858	DNA ligase 1 (EC	LIG1	-0.0403	-0.2081	0.3063	-0.1147	0.0780	0.0554	-0.0624	0.1032	0.4802	0.2799
219	S-1410_Q9N	Q9NQT8	Kinesin-like prot	KIF13B GAKI	-0.3183	-0.2695	-0.0708	0.0317	-0.3169	-0.3182	-0.4189	-0.3968	-0.5547	-0.3216
220	S-1413_Q02	Q02880	DNA topoisomer	TOP2B	0.2084	0.2652	0.2414	0.1518	0.1394	0.0583	0.0783	0.0422	0.1944	0.2037
221	S-1413_Q9N	Q9NRL2	Bromodomain ac	BAZ1A ACF1	-0.2509	-0.1309	-0.4764	-0.3420	-0.4848	-0.2639	-0.0983	-0.4226	-0.6706	-0.4806
222	S-1413_Q9N	Q9NZM4	BRD4-interacting	BICRA GLTS	-0.1645	0.0242	-0.0211	-0.1109	-0.2193	-0.1388	-0.0705	0.0831	-0.0249	0.1019
223	S-142_P162	P16220	Cyclic AMP-respo	CREB1	0.2756	0.2504	-0.3475	-0.3215	-0.4794	-0.2162	0.0168	-0.1244	-0.3271	-0.1447
224	S-142_Q5SS	Q5SSJ5	Heterochromatir	HP1BP3	-2.3829	-2.2183	-1.9206	-2.1637	-2.1300	-2.2145	-2.0091	-2.0498	-2.2447	-1.8826
225	S-142_Q927	Q92785	Zinc finger prote	DPF2 BAF45	-0.3229	-0.2127	-0.3493	-0.2915	-0.3844	-0.3666	-0.2645	-0.4131	-0.3519	-0.2389
226	S-143_P352	P35236	Tyrosine-protein	PTPN7	-0.8921	-1.2496	-0.5565	-0.8244	-0.3192	-0.2816	-0.0502	-0.2865	-0.9080	-0.8132
227	S-143_Q5JT	Q5JTV8	Torsin-1A-intera	TOR1AIP1 L/	-0.1128	0.0932	-0.0785	0.0930	0.5760	0.9715	0.8696	0.6242	0.1053	0.2171
228	S-143_Q8NI	Q8NDX1	PH and SEC7 don	PSD4 EFA6B	1.0269	1.1915	0.8686	0.9410	0.7710	1.0768	0.8922	0.8697	0.5845	0.6016
229	S-144_Q135	Q13523	Serine/threonine	PRPF4B KIA/	-0.7274	-0.6472	-0.3812	-0.1858	-1.0785	-0.9134	-1.0013	-1.0216	-0.4893	-0.3674
230	S-144_Q96K	Q96K21	Abscission/NoCu	ZFYVE19 AN	0.3931	0.2649	0.6029	0.5560	0.3352	0.5004	0.1872	0.1838	0.5009	0.4717
231	S-144_Q9H1	Q9H1E3	Nuclear ubiquito	NUCKS1 NU	-0.2488	-0.4155	-0.7155	-1.0144	-0.4671	-0.4635	-0.7161	-0.7073	-0.1487	-0.1837
232	S-1448_Q14	Q14160	Protein scribble l	SCRIB CRIB1	-0.3618	-0.3294	-0.3185	-0.2394	-0.2366	-0.3350	-0.5746	-0.2939	-0.6252	-0.5607

233	S-1453_Q86	Q86U86	Protein polybron	PBRM1 BAF1	-3.0857	-3.1979	-3.0796	-2.4252	-2.9844	-2.8344	-2.6419	-3.2827	-3.3469	-3.3891
234	S-1459_P21	P21333	Filamin-A (FLN-A	FLNA FLN FL	0.3404	0.3057	0.4722	0.3825	0.2824	0.4079	0.1801	0.3702	0.4761	0.5730
235	S-146_O603	O60307	Microtubule-ass	MAST3 KIAA	1.0144	1.1077	0.8547	0.9722	0.9370	1.2784	1.2827	1.2760	0.9101	0.6475
236	S-146_Q134	Q13404	Ubiquitin-conjug	UBE2V1 CRC	-1.2394	-1.0279	-1.3977	-1.3461	-1.5970	-1.1372	-1.1988	-1.2939	-1.3803	-1.5721
237	S-146_Q148	Q14847	LIM and SH3 dom	LASP1 MLN5	-0.4177	-0.3978	-0.4440	-0.6083	-0.3553	-0.3788	-0.3462	-0.3818	-0.4782	-0.2141
238	S-146_Q96A	Q96AT1	Uncharacterized	KIAA1143	-2.2652	-2.7110	-0.6034	-1.9562	-0.8995	-2.2708	-1.0144	-2.1547	-1.6272	-2.1419
239	S-1461_Q13	Q13576	Ras GTPase-activ	IQGAP2	-0.4042	-0.2708	0.7416	-0.1597	0.4030	-0.7797	0.3530	-0.1513	-0.7335	-0.6387
240	S-1468_Q9U	Q9UIG0	Tyrosine-protein	BAZ1B WBS(	0.2146	0.0000	0.1688	-0.0805	-0.1159	0.0697	-0.0597	-0.1547	-0.3803	-0.1362
241	S-147_Q8IZI	Q8IZD4	mRNA-decapping	DCP1B	0.6312	0.8569	0.5410	0.5483	0.6222	0.8380	0.4934	0.6863	0.3597	0.4544
242	S-147_Q8W	Q8WW12	PEST proteolytic	PCNP	-1.1767	-0.8478	0.4045	-0.2499	-0.1482	-1.0985	0.2333	-0.6276	-0.7730	-0.9334
243	S-1475_Q14	Q14160	Protein scribble l	SCRIB CRIB1	-0.9260	-1.1903	-0.9285	-0.6607	-0.8927	-0.7197	-0.8579	-0.8437	-1.1066	-0.9758
244	S-148_O152	O15234	Protein CASC3 (C	CASC3 MLN5	-0.2650	-0.1792	-0.0871	-0.0099	-0.0350	0.0391	-0.2011	-0.2272	0.0362	0.0123
245	S-149_Q132	Q13283	Ras GTPase-activ	G3BP1 G3BF	-1.3004	-1.0950	-1.5549	-1.7447	-1.8064	-1.9106	-1.5941	-2.4733	-1.7444	-1.8638
246	S-149_Q6UE	Q6UB98	Ankyrin repeat d	ANKRD12 AI	-0.0609	0.0209	0.2581	0.0018	-0.1320	0.0838	0.0942	0.0759	-0.2037	-0.1866
247	S-149_Q8IVI	Q8IVM7	Deleted.	0	-2.0255	-2.2132	-2.0360	-2.3968	-2.1544	-2.1670	-2.1751	-1.8658	-2.3141	-2.6070
248	S-149_Q8N5	Q8N5F7	NF-kappa-B-activ	NKAP	0.4865	0.2671	-0.7227	-0.1657	-0.0611	-0.6834	0.4069	-0.7052	-0.6292	-0.3857
249	S-1494_Q00	Q00610	Clathrin heavy ch	CLTC CLH17	-0.3548	-0.1357	-0.8434	-0.8307	-0.9133	-0.8029	-0.6893	-0.8968	-1.0761	-0.9910
250	S-1499_Q9U	Q9UQ35	Serine/arginine r	SRRM2 KIAA	0.7744	0.8950	1.1692	1.1574	1.1436	1.0640	0.8445	1.0569	1.0065	1.1368
251	S-150_P062	P06239	Tyrosine-protein	LCK	1.2816	1.1646	0.6964	0.5820	0.8208	0.7020	1.0097	0.6758	0.5966	0.6776
252	S-150_Q6ZS	Q6ZSZ5	Rho guanine nuc	ARHGEF18 K	-0.5213	-0.7384	-0.5457	-0.7809	-0.5749	-0.3016	-0.6706	-0.6042	-0.5395	-0.6752
253	S-1507_P51	P51610	Host cell factor 1	HCFC1 HCF1	-1.0515	-1.4742	-1.2361	-1.0861	-1.3564	-1.3406	-1.3661	-1.0185	-1.2293	-1.0090
254	S-151_P404	P40425	Pre-B-cell leuken	PBX2 G17	-0.8622	-0.8438	-0.9506	-0.4252	-1.0477	-1.0858	-1.0382	-1.1650	-0.8296	-0.8154
255	S-151_Q96S	Q96S55	ATPase WRNIP1	WRNIP1 WH	-0.8105	-1.1878	-0.0382	-0.3038	0.0391	-0.7148	-0.0489	-0.6205	-0.1579	0.1709
256	S-1510_Q8W	Q8WWI1	LIM domain only	LMO7 FBX2(	-0.8426	-0.7440	-0.2824	-0.6806	0.1411	-0.4200	-0.2488	-0.3454	0.0104	-0.2087
257	S-152_P185	P18583	Protein SON (Bax	SON C21orf5	-0.3479	-0.4213	-0.1053	-0.1498	-0.2784	-0.0890	-0.0302	-0.0964	-0.0476	-0.0551
258	S-152_Q7L2	Q7L2J0	7SK snRNA meth	MEPCE BCDI	-0.9631	-1.2506	-0.1719	-0.4634	0.2562	0.0514	-0.1140	0.1927	0.1901	-0.2080
259	S-152_Q9UI	Q9UI08	Ena/VASP-like pr	EVL RNB6	-1.9749	-2.4169	-1.4713	-1.7186	-1.7129	-1.8238	-1.1634	-1.6538	-1.5151	-1.8498
260	S-1523_Q5S	Q5SRE5	Nucleoporin NUF	NUP188 KIA	-0.2287	-0.2158	-0.2140	-0.1283	-0.6365	-0.0499	-0.0911	-0.2049	-0.4181	-0.5875
261	S-1528_P51	P51531	Probable global t	SMARCA2 B/	-0.2245	0.0586	-0.5230	-0.0378	-0.5558	0.7898	-0.3379	-0.0882	-0.3029	-0.2464
262	S-153_O952	O95218	Zinc finger Ran-b	ZRANB2 ZIS	0.8863	0.9794	0.3072	0.5974	0.3462	0.1321	0.5343	0.1159	0.2069	0.3411
263	S-153_Q166	Q16666	Gamma-interfer	IFI16 IFNGIP	0.9457	0.8410	0.6558	0.2022	0.5334	0.5201	0.5837	0.7594	0.4774	0.7444
264	S-153_Q8N2	Q8N2F6	Armadillo repeat	ARMC10 SVI	-0.9123	-0.6078	-0.6569	-0.3170	-0.4717	-0.4340	-0.6118	-0.5652	-1.0329	-0.9163
265	S-153_Q96S	Q96S55	ATPase WRNIP1	WRNIP1 WH	0.4164	0.4557	0.1079	0.4384	0.2159	0.2406	0.5298	0.3605	0.2600	0.3248
266	S-153_Q9NY	Q9NYF8	Bcl-2-associated	BCLAF1 BTF	1.4225	1.3640	1.6909	1.5176	1.3331	1.4516	1.5076	1.5556	1.8597	1.7711
267	S-1535_Q14	Q14839	Chromodomain-l	CHD4	1.2736	1.1369	0.8676	0.7269	0.9592	0.8993	0.9029	0.8981	0.7640	0.5803
268	S-1537_Q9U	Q9UQ35	Serine/arginine r	SRRM2 KIAA	-4.3548	-3.1382	-2.1064	-2.8056	-2.6949	-2.1537	-2.8509	-2.8492	-1.9252	-1.9457
269	S-154_Q8N4	Q8N4S0	Coiled-coil doma	CCDC82 HT0	1.9129	1.8860	2.1272	1.9708	2.3140	2.2615	2.1362	2.0422	2.0987	2.0883
270	S-155_Q8TB	Q8TBF4	Zinc finger CCHC	ZCRB1	-2.0478	-2.0393	-2.3146	-2.5138	-2.2129	-2.4380	-2.0953	-2.8603	-2.6312	-1.9961
271	S-155_Q96I	Q96I25	Splicing factor 45	RBM17 SPF4	-1.3260	-1.3138	-1.0559	-1.0392	-1.1374	-0.9962	-1.0707	-0.9496	-1.0668	-1.2884

272	S-1552_Q02	Q02880	DNA topoisomer	TOP2B	0.3169	0.1108	0.1050	0.3685	0.3331	0.2692	0.2768	0.4039	0.0486	-0.1851
273	S-1565_Q12	Q12802	A-kinase anchor	AKAP13 BRX	0.2867	0.4921	0.0999	0.2084	0.4842	0.2649	0.4689	0.1759	-0.1308	-0.3322
274	S-1568_Q9Y	Q9Y520	Protein PRRC2C (	PRRC2C BAT	-2.1484	-2.1627	-1.9245	-2.0861	-1.7936	-2.2284	-1.4559	-1.8547	-2.0276	-2.1082
275	S-157_O754	O75420	GRB10-interactir	GIGYF1 CDS2	-0.8787	-0.7477	-0.3502	-0.3735	-0.1959	-0.4340	-0.2753	-0.3474	-0.8483	-0.7577
276	S-157_P103!	P10398	Serine/threonine	ARAF ARAF1	-0.0704	-0.1777	-0.0919	0.1112	-0.1401	0.0654	-0.0705	-0.0325	-0.0557	-0.1292
277	S-157_P234!	P23497	Nuclear autoanti	SP100	0.1607	0.1230	-0.1337	-0.0899	-0.2108	-0.4706	-0.2784	-0.5428	-0.2965	0.0406
278	S-157_Q927	Q92733	Proline-rich prot	PRCC TPRC	0.9652	1.1983	1.0016	0.8333	0.8114	0.8571	0.6422	0.7951	1.1162	0.9845
279	S-157_Q96S	Q96ST2	Protein IWS1 hor	IWS1 IWS1L	0.6658	0.6601	0.6023	0.6009	0.6054	0.6251	0.3911	0.8195	0.6163	0.4298
280	S-1579_Q69	Q69YN4	Protein virilizer h	VIRMA KIAA	0.3612	0.2551	1.1557	0.8804	0.9312	1.1402	0.8845	1.0377	1.3561	1.1368
281	S-158_O754	O75494	Serine/arginine-r	SRSF10 FUSI	-1.6179	-1.2549	-0.8834	-0.9562	-0.8723	-0.8912	-0.9935	-1.0000	-0.9178	-1.1533
282	S-1581_Q02	Q02880	DNA topoisomer	TOP2B	0.2546	0.1983	0.3693	0.0575	0.0780	0.3967	0.1850	0.2220	0.1956	0.2756
283	S-159_O757	O75791	GRB2-related ad	GRAP2 GADS	-3.2309	-3.6334	-3.6470	-3.0937	-2.9699	-3.6181	-3.3232	-3.5428	-3.6556	-3.4027
284	S-1590_Q8T	Q8TEU7	Rap guanine nuc	RAPGEF6 PD	-1.1726	-1.1021	-0.9087	-0.9876	-1.2829	-1.1209	-1.2975	-1.2316	-0.9602	-0.9334
285	S-1592_Q92	Q92608	Dedicator of cyto	DOCK2 KIAA	-1.5475	-1.6507	-1.9127	-1.7387	-1.0902	-2.1405	-1.7645	-1.7899	-1.8296	-2.1648
286	S-1596_Q04	Q04637	Eukaryotic transl	EIF4G1 EIF4I	0.1671	0.0607	-0.0084	0.2642	0.2519	0.4012	0.4971	0.1968	-0.4893	-0.3774
287	S-16_Q1320	Q13200	26S proteasome	PSMD2 TRAI	1.6330	1.6015	1.7559	1.5879	1.4823	1.6226	1.6559	1.7973	1.7402	1.3703
288	S-16_Q9BVG	Q9BVG9	Phosphatidylseri	PTDSS2 PSS2	0.2037	0.1896	0.1510	0.0846	-0.3169	0.0207	0.0685	-0.0817	-0.4181	-0.4670
289	S-16_Q9Y5B	Q9Y5B6	PAX3- and PAX7-	PAXBP1 C21	-0.4829	0.4206	0.6834	0.1944	0.5410	0.4080	0.6743	0.6986	0.9490	1.2412
290	S-160_O602	O60231	Pre-mRNA-splicir	DHX16 DBP2	1.1090	1.0436	0.5553	0.6331	0.6283	0.4119	0.6570	0.5249	0.4814	0.4521
291	S-160_O754	O75494	Serine/arginine-r	SRSF10 FUSI	-1.9892	-1.3197	-1.1686	-0.7269	-0.9449	-1.1871	-0.8002	-0.7689	-1.2885	-0.7686
292	S-160_Q8N5	Q8N554	Zinc finger protei	ZNF276 CEN	-1.7699	-1.6197	-1.3731	-1.0786	-1.2301	-1.4300	-1.5364	-1.3435	-1.4042	-1.2058
293	S-161_Q1KM	Q1KMD3	Heterogeneous r	HNRNPUL2 I	0.3183	0.6146	0.0104	0.3985	0.0210	0.0015	0.1712	0.1699	-0.4129	-0.0834
294	S-161_Q68D	Q68DK7	Male-specific letl	MSL1 MSL1L	-1.7095	-1.6858	-1.4655	-1.4886	-1.5053	-1.5954	-1.6582	-1.4818	-1.7487	-1.4511
295	S-161_Q9H1	Q9H147	Deoxynucleotidy	DNTTIP1 C20	1.6359	1.6848	1.3563	1.4696	1.0718	1.1422	1.2623	1.3546	1.1216	0.9104
296	S-1619_Q9U	Q9UKA4	A-kinase anchor	AKAP11 AKA	0.3385	0.3701	-0.0211	0.4619	0.3606	0.5488	0.2043	0.1834	0.0536	-0.0985
297	S-162_P296!	P29692	Elongation factor	EEF1D EF1D	-0.6589	0.1332	-0.2990	0.0000	-0.6376	-0.0515	-0.4847	-0.3051	-0.1993	-0.2757
298	S-164_O150	O15027	Protein transport	SEC16A KIAA	-1.0648	-1.3652	0.4894	0.6323	0.4494	0.4418	0.7362	0.5383	0.3722	0.7398
299	S-164_O608	O60841	Eukaryotic transl	EIF5B IF2 KIA	1.1419	0.9596	1.3135	1.2731	1.2112	1.2431	1.2827	1.3041	0.9389	0.8149
300	S-164_P506:	P50613	Cyclin-dependen	CDK7 CAK CA	1.8223	1.7781	1.0918	1.3116	0.9551	0.8652	1.0097	0.8781	0.9490	0.8068
301	S-164_Q9UF	Q9UFW8	CGG triplet repea	CGGBP1 CGC	-1.1206	-1.0902	1.3118	-0.2144	0.3592	-1.2042	0.1896	-1.1378	-0.2571	-0.2914
302	S-1644_Q9N	Q9NQT8	Kinesin-like prote	KIF13B GAKI	-0.6290	-0.8359	-1.2609	-1.2373	-1.0863	-0.9078	-1.2018	-0.9878	-1.1349	-1.1939
303	S-165_P518!	P51858	Hepatoma-derive	HDGF HMG1	0.8656	1.0649	1.0487	0.9433	0.6924	0.7475	0.6852	0.9167	1.0955	0.9297
304	S-165_Q8IW	Q8IWW6	Rho GTPase-activ	ARHGAP12	0.5397	0.7332	-0.1395	0.0745	0.2032	0.0739	0.4484	0.1453	-0.3974	-0.4009
305	S-165_Q96B	Q96B97	SH3 domain-cont	SH3KBP1 CIN	-0.6402	-0.4696	-0.1269	-0.5894	-0.3493	-0.3905	-0.1318	-0.3729	-0.3837	-0.2252
306	S-165_Q96G	Q96G03	Phosphoglucomu	PGM2 MSTP	1.0099	1.2940	0.0423	0.1454	0.1428	0.3320	0.3377	0.1089	0.3206	0.3950
307	S-165_Q96J(	Q96JC9	ELL-associated fa	EAF1	0.1831	-0.0189	0.2476	0.1851	-0.0844	0.1212	0.0232	-0.0689	0.1090	0.1374
308	S-165_Q9Y3	Q9Y3Q8	TSC22 domain fa	TSC22D4 TH	-0.9964	-0.5243	-1.5704	-0.8822	-1.9991	-0.9970	-1.4276	-1.3166	-1.0543	-0.9532
309	S-166_O603	O60341	Lysine-specific hi	KDM1A AOF	-0.6745	-0.4498	-0.3094	-0.3461	-0.6831	-0.4563	-0.7667	-0.7952	-0.1378	-0.0471
310	S-166_Q9NC	Q9NQG5	Regulation of nu	RPRD1B C20	-2.0478	-1.9989	-1.6903	-2.0488	-1.5423	-1.3071	-1.4452	-1.5972	-1.3370	-1.2177

311	S-1663_Q7Z	Q7Z333	Probable helicase	SETX ALS4 K	-1.0255	-1.1212	-0.9506	-0.9562	-1.0251	-1.0858	-0.8555	-0.7304	-1.0816	-0.9961
312	S-167_P538(	P53805	Calcipressin-1 (A	RCAN1 ADAI	-0.8361	-1.0302	-0.1121	-0.6978	-0.0157	-0.4200	-0.1361	-0.5120	-0.3304	-0.5012
313	S-168_Q014	Q01433	AMP deaminase	AMPD2	-0.1167	-0.5978	1.4830	1.0447	1.3309	0.6017	1.1975	0.9589	1.2298	0.8410
314	S-168_Q146	Q14676	Mediator of DNA	MDC1 KIAA(	-1.0553	-1.0347	-0.6751	-0.7299	-0.7434	-0.6834	-0.7623	-0.5295	-0.6053	-0.4284
315	S-1685_Q92	Q92608	Dedicator of cyto	DOCK2 KIAA	1.3877	1.3597	1.6782	1.6763	1.6900	1.4494	2.1867	1.9239	1.0328	1.1256
316	S-169_O435	O43598	2'-deoxynucleosi	DNPH1 C6or	0.3752	0.5274	-0.6291	-0.8888	-0.5612	-0.5775	-0.4648	-0.8492	-0.5357	-0.3757
317	S-169_Q9H4	Q9H4L4	Sentrin-specific p	SENP3 SSP3	-0.3548	-0.5258	-0.8415	-0.8056	-1.0902	-0.4380	-0.8276	-1.0372	-1.2509	-0.7907
318	S-1692_Q9Y	Q9Y2F5	Little elongation	ICE1 KIAA09	-0.8343	-0.6780	-0.6424	-0.6151	-0.3014	-0.6333	-0.6948	-0.5971	-0.7007	-0.3040
319	S-17_O0019	O00193	Small acidic prot	SMAP C11or	0.0685	0.5269	-0.3725	-0.3319	-0.3248	-0.7009	-0.3054	-0.6117	-0.2196	-0.3388
320	S-17_P3968	P39687	Acidic leucine-ric	ANP32A C15	-0.5768	-0.6966	-0.3977	0.0846	-0.0496	-0.2907	-0.0368	0.2088	-0.5189	-0.6876
321	S-17_P4945(	P49450	Histone H3-like c	CENPA	-2.1404	-1.7329	-2.0403	-2.1960	-2.0515	-1.8397	-2.1693	-2.1412	-2.3939	-2.1476
322	S-17_P5399	P53999	Activated RNA pc	SUB1 PC4 RI	-0.4211	-0.3114	0.1762	0.1630	-0.0805	-0.0030	-0.2427	-0.1667	0.3102	0.4450
323	S-17_Q96F1	Q96F15	GTPase IMAP fan	GIMAP5 IAN	0.1012	-0.0201	-0.0818	-0.2206	-0.2628	-0.0210	-0.1766	-0.1328	-0.2052	-0.1462
324	S-170_P095	P09543	2',3'-cyclic-nucle	CNP	0.0928	0.0723	0.1817	0.2252	-0.1360	0.3106	0.1562	0.3329	0.0883	-0.0325
325	S-170_Q8NI	Q8NHV1	GTPase IMAP fan	GIMAP7 IAN	-0.7274	-0.7110	-0.8247	-0.4275	-0.6251	-0.5798	-0.6439	-0.5675	-0.7465	-0.8874
326	S-170_Q96D	Q96D71	RalBP1-associate	REPS1	-0.6487	-1.1212	0.6126	0.5013	0.2795	0.6771	0.0722	0.3830	0.8053	0.9871
327	S-1705_Q92	Q92608	Dedicator of cyto	DOCK2 KIAA	0.8636	0.9273	1.4653	1.5471	1.2364	1.5968	1.3018	1.6296	1.1216	0.9422
328	S-171_O751	O75152	Zinc finger CCCH	ZC3H11A KI/	0.3037	0.4476	-0.1947	-0.0692	-0.0669	0.2023	-0.0502	0.2114	-0.1306	0.0740
329	S-171_Q8N	Q8N9T8	Protein KRI1 hon	KRI1	0.1230	-0.0738	-0.2104	0.0229	-0.6336	-0.6954	0.1550	0.1453	0.0871	0.1936
330	S-1714_P35	P35579	Myosin-9 (Cellula	MYH9	0.0180	0.1828	0.5229	0.2657	0.4856	0.7462	0.4436	0.8797	0.8821	0.4693
331	S-1721_Q15	Q15149	Plectin (PCN) (PL	PLEC PLEC1	-1.8523	-1.7664	-1.6835	-0.8056	-1.0251	-1.2388	-1.2138	-1.0154	-1.5263	-1.2788
332	S-1722_Q9U	Q9UKK3	Protein mono-AD	PARP4 ADPI	-0.3641	-0.7496	-0.3303	-0.4204	-0.5667	-0.6411	-0.0760	-0.0419	-0.4766	-0.6752
333	S-1729_Q9U	Q9UQ35	Serine/arginine r	SRRM2 KIAA	0.1911	0.0077	0.1734	0.2206	0.4333	0.3213	0.1952	0.2835	0.6086	0.6516
334	S-173_Q153	Q15365	Poly(rC)-binding	PCBP1	0.1134	-0.0819	-0.2271	-0.2031	-0.2133	0.1127	-0.0767	0.0870	-0.0219	0.0394
335	S-173_Q69Y	Q69YN4	Protein virilizer h	VIRMA KIAA	-0.2805	-0.1492	0.2200	0.2538	0.0462	0.0382	0.2155	0.2734	0.2370	0.2564
336	S-1731_Q9U	Q9UQ35	Serine/arginine r	SRRM2 KIAA	1.0962	0.9331	0.8771	0.9475	1.0444	1.0468	0.9572	1.1117	1.5093	1.5142
337	S-1732_Q9U	Q9UQ35	Serine/arginine r	SRRM2 KIAA	0.7272	0.7063	0.4680	0.6550	0.8219	0.7066	0.6711	0.6700	0.8105	0.7913
338	S-1736_Q92	Q92608	Dedicator of cyto	DOCK2 KIAA	1.2986	1.3862	1.4226	1.1968	1.1668	1.3543	1.3065	1.4853	1.2816	1.4915
339	S-174_P279	P27987	Inositol-trisphos	ITPKB	-3.0553	-2.9298	-2.7630	-2.8691	-2.6949	-2.8995	-3.2975	-2.5251	-2.3939	-2.0013
340	S-174_Q929	Q92974	Rho guanine nuc	ARHGEF2 KI/	-0.0628	-0.1394	-0.6486	-0.4713	-1.0139	-0.4061	-0.6872	-0.6159	-0.3939	-0.5214
341	S-174_Q96A	Q96A70	Antizyme inhibito	AZIN2 ADC K	0.6005	0.3007	0.5156	0.5713	-0.0573	0.2937	0.4215	0.2998	0.2975	0.1902
342	S-175_Q6PC	Q6PCE3	Glucose 1,6-bispl	PGM2L1 BM	1.1282	1.1731	0.2818	0.2925	0.4470	0.3495	0.5260	0.3801	0.2747	0.3970
343	S-175_Q7Z5	Q7Z5L9	Interferon regula	IRF2BP2	1.3248	1.6478	0.5425	0.7309	0.6797	0.4357	0.5226	0.3305	0.3546	0.5965
344	S-1757_Q14	Q14980	Nuclear mitotic a	NUMA1 NM	0.6738	0.8410	-0.1971	-0.1558	-0.0922	-0.3463	0.1421	-0.0123	-0.0026	-0.0418
345	S-176_Q134	Q13442	28 kDa heat- and	PDAP1 HASI	-0.2805	-0.4865	0.4642	-0.6384	0.0480	-0.2870	-0.0170	-0.1857	-0.4356	-0.6488
346	S-176_Q32P	Q32P44	Echinoderm micr	EML3	-0.2182	-0.4437	0.8788	0.1063	0.3243	-0.0561	0.4254	0.0407	-0.0356	-0.1720
347	S-177_O753	O75396	Vesicle-traffickin	SEC22B SEC	0.4929	0.2962	0.7137	-0.0287	0.5810	-0.2128	0.2617	-0.0061	-0.3045	-1.0431
348	S-177_O754	O75475	PC4 and SFRS1-ir	PSIP1 DFS70	-0.4645	-0.5177	-0.3145	-0.2371	-0.4058	-0.2765	-0.5130	-0.2881	-0.3158	-0.1439
349	S-177_Q096	Q09666	Neuroblast differ	AHNAK PM2	1.0911	0.9156	1.1243	0.9195	0.4908	0.6556	1.1064	0.7915	-0.2602	-0.0052

350	S-177_Q150	Q15024	Exosome comple	EXOSC7 KIA/	-0.6431	-0.3934	-0.7559	-1.0824	-1.2172	-0.9503	-1.2753	-1.1892	-0.7078	-0.3024
351	S-177_Q9NY	Q9NYF8	Bcl-2-associated	BCLAF1 BTF	1.7771	1.9233	1.9389	2.3201	2.0937	1.9638	2.1914	2.2111	2.0081	1.9671
352	S-178_Q8W	Q8WUA2	Peptidyl-prolyl ci	PPIL4	-2.1484	-2.0122	-2.2092	-2.1960	-2.2387	-1.9162	-2.4103	-2.1684	-2.4356	-1.7997
353	S-178_Q9BZ	Q9BZ23	Pantothenate kir	PANK2 C20o	-1.0478	-0.8578	-0.5110	-0.4961	-0.7069	-0.4624	-0.6726	-0.6395	-0.6783	-0.7404
354	S-179_O954	O95429	BAG family mole	BAG4 SODD	0.1263	0.0099	0.1909	0.3560	0.3273	0.3344	0.3712	0.3665	0.0052	0.0331
355	S-179_P027	P02794	Ferritin heavy ch	FTH1 FTH FT	0.9928	1.3292	1.0115	1.7368	1.3199	1.5478	1.3118	1.5865	0.8579	0.9179
356	S-179_Q6JB	Q6JBY9	CapZ-interacting	RCSD1 CAPZ	0.5089	0.5382	0.7323	0.3128	0.7769	0.2350	0.7310	0.4475	0.1248	0.0544
358	S-18_P2349	P23497	Nuclear autoanti	SP100	0.4499	0.5449	0.3902	0.3781	0.3578	0.5263	0.4398	0.4336	0.2080	0.2883
359	S-18_Q86YV	Q86YV0	RAS protein activ	RASAL3	0.0928	-0.1578	0.5496	0.5126	0.2658	0.5149	0.7558	0.4550	0.3647	0.2336
360	S-18_Q8N3X	Q8N3X1	Formin-binding p	FNBP4 FBP3	-0.8508	-0.7438	-0.8551	-0.8267	-0.9025	-1.0168	-0.8985	-1.0183	-0.8733	-0.9223
361	S-18_Q9300	Q93009	Ubiquitin carbox	USP7 HAUSF	-0.1325	0.0055	-1.1522	-1.4444	-0.9520	-1.1405	-0.9278	-0.9025	-0.9961	-1.0780
362	S-18_Q9H6F	Q9H6F5	Coiled-coil doma	CCDC86 CYC	0.5471	0.4460	0.2137	0.2819	0.3125	0.3774	0.4273	0.3207	0.1944	0.4823
363	S-180_O151	O15156	Zinc finger and B	ZBTB7B ZBTI	-0.8426	-1.0832	-1.9648	-1.4987	-1.4948	-1.2284	-1.1988	-1.2716	-1.1150	-1.1419
364	S-180_P0C1	P0C1Z6	TCF3 fusion partı	TFPT INO80I	-0.7394	-0.4943	-0.2572	-0.5012	-0.5027	-0.3577	-0.3462	-0.4564	-0.2633	-0.5875
365	S-180_Q6DN	Q6DN90	IQ motif and SEC	IQSEC1 ARF(	-2.2565	-2.7256	-1.0211	-1.5087	-1.4141	-1.9911	-1.1605	-1.1178	-1.7014	-2.2541
366	S-181_P525	P52594	Arf-GAP domain	AGFG1 HRB	0.7744	0.7385	0.9483	0.4606	0.9098	1.0454	0.7930	0.6585	0.9457	0.4887
367	S-181_Q929	Q92945	Far upstream ele	KHSRP FUBP	-0.7637	-0.6823	-0.4873	-0.7328	-0.4439	-0.4522	-0.6562	-0.6683	-0.3353	-0.6980
368	S-181_Q9BV	Q9BVG4	Protein PBDC1 (F	PBDC1 CXor	-2.4211	-2.5728	-1.1042	-0.9087	-0.5639	-0.9106	-1.0355	-0.6159	-1.0409	-0.9114
369	S-181_Q9BX	Q9BXB4	Oxysterol-bindin	OSBPL11 OR	0.7435	0.7491	0.4528	0.4199	0.0710	0.1932	0.1433	0.2454	0.1435	0.4047
370	S-181_Q9H1	Q9H1E3	Nuclear ubiquito	NUCKS1 NU(	1.4089	1.3137	1.3998	1.3315	1.1863	1.0824	1.3418	1.2665	1.1078	0.9714
371	S-1814_O95	O95613	Pericentrin (Kend	PCNT KIAA0	-1.1285	-0.9682	-0.5829	-0.2951	-0.3682	-0.6044	-0.4017	-0.4860	-1.1207	-1.0999
372	S-182_Q8TB	Q8TB72	Pumilio homolog	PUM2 KIAA(	0.2979	0.0328	0.0705	0.1835	0.0814	-0.0438	0.0510	0.3047	0.1162	-0.0524
373	S-182_Q9Y3	Q9Y3P8	Signaling thresho	SIT1 SIT	-0.0422	-0.2547	0.0203	0.2116	0.4617	0.6435	0.2008	0.4826	0.3740	0.3913
374	S-183_O001	O00148	ATP-dependent F	DDX39A DD)	-0.5661	-0.3019	-0.2189	-0.3529	-0.3192	-0.0030	-0.4189	-0.5251	-0.3452	-0.5856
375	S-183_Q8IZF	Q8IZP0	Abl interactor 1 (	ABI1 SSH3BF	1.1727	1.0355	1.1909	1.2068	1.3375	1.4570	0.9784	1.1928	1.2370	1.4717
376	S-183_Q96B	Q96B97	SH3 domain-cont	SH3KBP1 CII	0.3738	0.3752	0.5185	0.4292	0.3258	0.4586	0.3325	0.3877	0.4727	0.2171
377	S-1832_Q14	Q14573	Inositol 1,4,5-tris	ITPR3	-1.0972	-1.0234	-1.3277	-1.4252	-1.2172	-1.1017	-1.0678	-1.2102	-1.1992	-0.7512
378	S-184_O954	O95466	Formin-like prote	FMNL1 C17c	1.1863	1.1251	1.2564	1.3201	0.7807	0.9142	0.8517	0.9660	0.8551	0.4211
379	S-184_P193	P19338	Nucleolin (Protei	NCL	0.8233	0.7894	1.2361	0.3308	1.3317	0.6125	0.7810	0.8622	1.5534	1.5119
380	S-1847_Q14	Q14980	Nuclear mitotic a	NUMA1 NM	-1.2015	-0.9748	-1.0190	-0.7358	-0.9917	-0.8666	-0.6914	-0.8382	-0.9203	-0.9138
381	S-185_Q1KN	Q1KMD3	Heterogeneous r	HNRNPUL2 I	-0.0761	0.0828	0.0115	1.1194	0.9137	0.4510	0.2211	0.5737	-0.3158	0.7007
382	S-185_Q86X	Q86XP3	ATP-dependent F	DDX42	-0.2630	-0.2788	0.0991	0.0369	0.1124	0.4113	0.0397	0.4605	0.5061	0.4076
383	S-185_Q9H4	Q9H4A3	Serine/threonine	WNK1 HSN2	-0.7699	-1.2365	1.7759	1.5343	1.4065	1.6720	1.6452	1.5577	1.4524	1.4823
384	S-185_Q9UE	Q9UBE0	SUMO-activating	SAE1 AOS1 S	0.1378	-0.0313	0.2236	0.3712	0.7283	0.8339	0.6364	0.9093	0.3423	0.3891
385	S-1858_Q03	Q03164	Histone-lysine N-	KMT2A ALL1	-1.9468	-2.2235	-1.9325	-1.3507	-1.5639	-1.5599	-1.3897	-1.2496	-1.9653	-1.6109
386	S-1859_P27	P27708	CAD protein [Incl	CAD	1.0072	0.8002	1.4886	1.0299	1.5574	1.8883	1.7462	1.7042	1.3289	0.8009
387	S-186_P224	P22415	Upstream stimul	USF1 BHLHB	1.9215	2.0088	1.4389	1.7918	1.6791	1.3549	1.7990	1.6232	1.3818	1.3930
388	S-187_P325	P32519	ETS-related trans	ELF1	1.4980	1.5506	1.4288	1.5263	1.0892	1.4324	1.4186	1.3103	1.2805	1.2714
389	S-187_Q9UC	Q9UQN3	Charged multives	CHMP2B CG	0.3668	0.2662	0.8969	1.1292	0.8930	0.6716	0.5785	0.6360	0.7790	0.8031

390	S-1876_Q12	Q12802	A-kinase anchor	AKAP13 BRX	-1.7334	-2.0212	-1.7881	-1.4492	-1.7009	-1.9618	-1.5863	-2.0947	-2.0927	-1.7997
391	S-188_O952	O95218	Zinc finger Ran-b	ZRANB2 ZIS	0.6405	0.6817	0.6344	0.6595	0.4347	0.8612	0.4636	0.8546	0.6769	0.6301
392	S-188_P432	P43243	Matrin-3	MATR3 KIAA	-2.5213	-2.5794	-3.0796	-2.4156	-2.5158	-2.9561	-2.6338	-2.6732	-3.4426	-2.7297
393	S-189_P332	P33241	Lymphocyte-spe	LSP1 WP34	0.2988	0.3125	0.7913	0.7840	0.4717	0.3029	0.4381	0.5853	0.8623	0.9711
394	S-189_Q153	Q15365	Poly(rC)-binding	PCBP1	-0.6041	-0.7950	-0.9526	-1.4886	-1.0863	-0.4727	-1.0705	-0.7102	-0.6252	-0.7774
395	S-189_Q8IYL	Q8IYL3	UPF0688 protein	C1orf174	1.5025	1.3951	1.4124	0.8223	1.2064	1.3520	1.5149	1.5246	1.2896	1.3728
396	S-189_Q96E	Q96E09	Protein FAM122	FAM122A C	-0.1747	-0.3156	-0.2032	0.0018	-0.4043	-0.0090	-0.1810	0.0672	0.1576	-0.0538
397	S-19_O7517	O75179	Ankyrin repeat d	ANKRD17 G	-1.1808	-1.5306	-1.1453	-1.1090	-1.2829	-1.3634	-1.1318	-1.4314	-1.0679	-0.8638
398	S-19_P5399	P53999	Activated RNA p	SUB1 PC4 R	-0.3116	-0.0246	-0.1337	-0.2712	-0.3100	-0.0795	-0.4505	-0.5562	0.1162	0.1936
399	S-19_Q6PK0	Q6PK04	Coiled-coil doma	CCDC137 cP	-0.5931	-0.3434	-0.6569	-0.5138	-0.4117	-0.2726	-0.2365	-0.3908	-1.0543	-0.8874
400	S-19_Q9H1E	Q9H1E3	Nuclear ubiquito	NUCKS1 NU	-0.2959	-0.0738	-0.4439	-0.3394	-0.4464	-0.3916	-0.5193	-0.5339	-0.4278	-0.3006
401	S-19_Q9NU	Q9NUE0	Palmitoyltransfe	ZDHHC18	0.3169	0.0713	-0.0577	-0.1777	-0.0611	0.0852	0.1088	0.1188	-0.1507	-0.2664
402	S-19_Q9UN	Q9UNE7	E3 ubiquitin-pro	STUB1 CHIP	-0.1625	0.3142	0.3198	0.8668	0.6318	0.7576	0.5208	0.8689	0.3070	0.3881
403	S-190_O608	O60841	Eukaryotic transl	EIF5B IF2 KI	0.0233	-0.0461	0.1272	-0.1617	0.1871	-0.0165	-0.3728	-0.2298	0.2524	0.1800
404	S-190_Q129	Q12906	Interleukin enha	ILF3 DRBF M	-0.9820	-0.8378	-1.3068	-1.2842	-1.2044	-1.0732	-1.4242	-1.1378	-1.5189	-1.0378
405	S-190_Q153	Q15365	Poly(rC)-binding	PCBP1	0.4722	0.5258	0.6404	0.8333	0.5270	0.6134	0.4030	0.3900	0.4932	0.6117
406	S-192_Q8ND	Q8ND56	Protein LSM14 h	LSM14A C1	-0.6374	-0.3920	-0.8453	-0.8789	-0.8927	-0.7222	-0.5327	-0.7820	-0.6909	-0.6959
407	S-193_P220	P22059	Oxysterol-bindin	OSBP OSBP1	-0.7828	-0.8799	-0.7485	-0.8362	-0.5493	-0.7711	-0.9265	-0.8863	-0.4492	-0.1138
408	S-193_P422	P42229	Signal transduce	STAT5A STA	0.0550	-0.6948	-1.0105	-1.3689	-1.4897	-0.7620	-0.3528	-1.3204	-1.1292	-1.4336
409	S-193_Q1KN	Q1KMD3	Heterogeneous r	HNRNPUL2 I	-1.2565	-1.2762	0.8879	1.1013	1.2901	1.2937	1.0277	1.3671	0.7737	0.9429
410	S-193_Q8NA	Q8NAV1	Pre-mRNA-splicir	PRPF38A	0.0962	0.0744	-0.4129	-0.6137	-0.2562	-0.7124	-0.1649	-0.5207	-0.2696	-0.0039
411	S-194_O435	O43513	Mediator of RNA	MED7 ARC3	-0.1605	-0.3156	-0.2275	-0.3104	-0.1564	-0.0546	-0.3023	0.0107	0.0448	0.0642
412	S-194_O755	O75533	Splicing factor 3	SF3B1 SAP1	0.4813	0.5041	0.1118	0.2523	0.1544	0.3751	0.1620	0.5727	0.3196	-0.1925
413	S-194_P086	P08651	Nuclear factor 1	NFIC NFI	-3.3735	-3.7627	-2.3785	-1.8244	-2.2387	-2.3369	-2.2473	-2.1753	-2.6638	-2.0917
414	S-194_Q166	Q16629	Serine/arginine-r	SRSF7 SFRS7	1.0454	0.9244	0.6558	0.9326	0.6854	0.4180	0.8160	0.8797	0.6891	0.9001
415	S-194_Q6U	Q6UWD8	Transmembrane	C16orf54 UI	-0.7335	-0.7827	-0.7480	-0.8471	-1.0010	-0.8279	-0.7953	-0.8557	-1.0114	-1.0285
416	S-194_Q9BU	Q9BUJ2	Heterogeneous r	HNRNPUL1 I	0.5643	0.6796	0.2137	0.4371	0.4750	0.5107	0.3742	0.0540	0.4491	0.3482
417	S-1941_O94	O94915	Protein furry hor	FRYL AF4P1	-0.8201	-1.4258	0.5053	0.3588	-0.0746	0.5221	0.1076	0.3059	0.2513	0.1185
418	S-1943_P35	P35579	Myosin-9 (Cellula	MYH9	0.4352	0.7233	0.3522	0.5013	0.5966	0.1239	0.4869	0.0212	0.2425	0.5968
419	S-195_P432	P43243	Matrin-3	MATR3 KIAA	-0.7600	-0.6775	-0.4414	-0.6117	-0.5232	-0.6183	-0.6908	-0.6635	-0.4665	-0.2411
420	S-195_Q6NY	Q6NYC8	Phostensin (Prot	PPP1R18 HK	-0.5317	-0.4619	-0.1546	-0.4085	-0.2896	-0.1017	0.1112	-0.0231	-0.3922	-0.5645
421	S-196_Q081	Q08117	TLE family memb	TLE5 AES GR	NA	NA	-3.2991	-1.9911	-2.4240	-2.3071	-2.4382	-2.5163	-3.7400	-3.7383
422	S-196_Q166	Q16629	Serine/arginine-r	SRSF7 SFRS7	0.0894	0.0891	0.5723	0.5879	0.1725	0.2342	0.1243	0.3700	0.8722	1.0280
423	S-196_Q9BV	Q9BW71	HIRA-interacting	HIRIP3	-1.1767	-1.6752	-1.2165	-0.8338	-1.1139	-1.2318	-1.3330	-1.1684	-1.0170	-1.0090
424	S-196_Q9NY	Q9NYF8	Bcl-2-associated	BCLAF1 BTF	-0.3071	-0.3392	-0.1019	-0.1459	-0.0083	0.2593	-0.1693	0.0745	0.4510	0.3258
425	S-197_Q6PI	Q6PID6	Tetratricopeptid	TTC33	0.3484	0.3502	-0.0179	0.0678	0.3081	0.5086	0.4475	0.3795	0.3627	0.2639
426	S-197_Q96J	Q96JY6	PDZ and LIM dor	PDLIM2 PP6	-0.8105	-0.8882	-0.8118	-0.9020	-0.6166	-0.6504	-0.5941	-0.5562	-1.0436	-1.0431
427	S-197_Q9UF	Q9UP83	Conserved oligor	COG5 GOLT	0.3527	0.2504	-0.1935	-0.0692	-0.4240	-0.4522	-0.2911	-0.5033	-0.4338	-0.1998
428	S-198_Q5T2	Q5T200	Zinc finger CCCH	ZC3H13 KIA	1.7343	1.7788	1.8222	1.8751	1.8260	1.5899	1.7711	1.7657	1.4558	1.2857

429	S-199_Q079	Q07955	Serine/arginine-r	SRSF1 ASF S	1.2792	1.4606	1.0554	1.2364	0.9870	1.0086	0.9493	1.0976	1.2400	1.2451
430	S-199_Q146	Q14699	Raftlin (Cell migr	RFTN1 KIAA	-0.3829	-0.6248	0.0864	0.1112	-0.1646	-0.0499	-0.3594	-0.0216	-0.3652	-0.7127
431	S-20_O4356	O43566	Regulator of G-p	RGS14	-0.3595	-0.6594	-0.6258	-0.3529	-0.5053	-0.2943	-0.4594	-0.2244	-0.3502	-0.2132
432	S-20_P2484	P24844	Myosin regulato	MYL9 MLC2	1.7375	1.8163	0.5568	0.2068	-0.0688	1.2393	0.7081	0.8655	-0.1814	0.1066
433	S-20_P4120	P41208	Centrin-2 (Caltra	CETN2 CALT	1.3491	1.2279	0.2859	0.5471	1.1626	0.8603	1.0504	0.9215	0.4338	0.4933
434	S-20_P5256	P52566	Rho GDP-dissoci	ARHGDIB GD	0.5813	0.6503	0.3080	0.4052	0.1226	0.9134	0.5432	0.7247	0.7163	0.6920
435	S-20_Q1359	Q13595	Transformer-2 p	TRA2A	1.1468	1.1301	1.5371	1.7400	1.7115	1.1867	1.4961	1.4875	1.6782	1.7639
436	S-200_Q155	Q15555	Microtubule-ass	MAPRE2 RP	-1.4259	-0.6858	-1.0211	-0.7240	-1.0326	-0.7594	-0.6726	-0.2753	-1.0734	-1.1851
437	S-200_Q9Y3	Q9Y3E7	Charged multive	CHMP3 CGI1	-0.1849	-0.0011	-0.5751	-0.2394	-0.1340	0.4379	0.0052	0.3486	0.2943	0.3832
438	S-201_P202	P20290	Transcription fac	BTF3 NACB	0.0410	-0.0359	0.2379	0.1046	0.1610	0.5529	-0.2018	0.0903	0.4793	0.6185
439	S-201_P226	P22626	Heterogeneous r	HNRNPA2B1	0.4696	0.2671	0.8941	0.6242	0.6530	0.3933	0.7357	0.4727	0.8311	0.8932
440	S-202_P518	P51858	Hepatoma-deriv	HDGF HMG1	-0.2805	-0.4066	-0.1511	0.2701	0.1344	-0.0577	-0.2093	-0.5517	0.3758	-0.1677
441	S-202_Q86X	Q86X95	Corepressor inte	CIR1 CIR	-0.3829	-0.2496	-0.1019	-0.2373	-0.2409	-0.1193	-0.3379	-0.3338	0.0283	0.1421
442	S-2020_Q92	Q92614	Unconventional	MYO18A CD	0.0286	-0.0316	0.2261	0.1915	-0.0795	0.0849	-0.1415	0.0110	-0.3153	-0.3535
443	S-203_O435	O43566	Regulator of G-p	RGS14	0.5463	0.5891	0.9929	1.3602	1.3602	1.2756	1.3430	1.3603	0.9270	0.9117
444	S-203_O436	O43633	Charged multive	CHMP2A BC	-0.9235	-0.9632	-0.9520	-0.9221	-1.2229	-1.0878	-1.3983	-1.4522	-0.7795	-0.6483
445	S-203_P005	P00558	Phosphoglycerat	PGK1 PGKA	-0.7394	-0.7570	-0.5923	-0.4639	-0.6336	-0.6044	-0.8912	-0.8465	-0.2696	-0.2252
446	S-203_Q047	Q04726	Transducin-like e	TLE3 KIAA15	-0.3805	-0.1615	-0.5065	-0.7007	-0.5423	-0.7345	-0.4738	-0.6781	-0.6072	-0.4831
447	S-203_Q9BR	Q9BRR9	Rho GTPase-activ	ARHGAP9	-0.2161	-0.2748	0.0824	0.0863	-0.2431	-0.1922	-0.1332	-0.2772	-0.2248	-0.0431
448	S-203_Q9H8	Q9H8G2	Caspase activity	CAAP1 C9or	-0.1030	-0.1370	0.0290	-0.5317	0.1494	0.1932	0.0384	0.2285	0.3403	0.1444
449	S-203_Q9NY	Q9NYB0	Telomeric repeat	TERF2IP DRI	1.0233	0.9867	0.8243	0.9195	0.7093	0.7699	0.5459	0.7265	0.6955	0.7110
450	S-204_P279	P27987	Inositol-trisphos	ITPKB	0.3959	0.1098	0.3257	0.2553	0.4114	0.4180	0.3243	0.3759	0.1150	0.3113
451	S-204_P332	P33241	Lymphocyte-spe	LSP1 WP34	-0.5084	-0.3920	-0.5751	-0.2972	-0.1020	-1.1603	0.1172	-0.5163	-1.2447	-1.3525
452	S-204_Q86W	Q86WR0	Coiled-coil doma	CCDC25	-0.2805	-0.5597	-0.5534	-1.2885	-0.9168	-0.4830	-0.5592	-0.2032	-0.4356	-0.6347
453	S-2047_Q9U	Q9UPU5	Ubiquitin carbox	USP24 KIAA	1.8222	1.9728	1.7002	1.6991	1.5835	1.7043	1.6645	1.6917	1.6941	1.6454
454	S-205_Q68D	Q68DK7	Male-specific let	MSL1 MSL1L	-0.2372	-0.0133	-0.0620	-0.1777	0.1952	0.3797	-0.2319	0.0730	0.1257	-0.0524
455	S-205_Q96E	Q96EV2	RNA-binding pro	RBM33 PRR	1.1071	1.1397	0.0991	0.4502	0.4292	0.0936	0.5914	0.3629	0.0052	0.0910
456	S-2051_Q9Y	Q9Y4A5	Transformation/	TRRAP PAF4	-0.0459	0.1740	0.9709	0.9731	0.7882	1.0746	1.1924	1.2160	0.7691	0.5699
457	S-206_O754	O75475	PC4 and SFRS1-ir	PSIP1 DFS70	0.3932	0.2118	0.3257	0.4305	0.3317	0.4213	0.3408	0.2760	0.5261	0.4421
458	S-206_P193	P19338	Nucleolin (Protei	NCL	1.0697	1.1607	0.9297	0.5592	0.7305	0.7292	0.6937	0.9036	1.5079	1.5020
459	S-206_Q8W	Q8WYJ6	Septin-1 (LARP) (	SEPTIN1 DIF	-0.3049	-0.0680	-0.0285	-0.0053	-0.0382	0.4213	-0.5421	-0.0689	0.2091	-0.1110
460	S-206_Q96J	Q96JH8	Ras-associating a	RADIL KIAA1	-1.3548	-1.2081	-1.3922	-0.6834	-1.1060	-2.1471	-0.7083	-1.2032	-1.4640	-1.1619
461	S-207_P525	P52564	Dual specificity n	MAP2K6 ME	-1.0666	-1.0832	-0.6356	-0.5975	-0.8690	-0.3924	-0.5516	-0.2406	-0.7487	-0.6670
462	S-207_Q055	Q05519	Serine/arginine-r	SRSF11 SFRS	0.2890	0.4509	0.1743	0.2160	0.2238	0.4023	0.3315	0.3688	0.3133	0.4202
463	S-207_Q130	Q13094	Lymphocyte cytc	LCP2	-0.8799	-1.0374	-1.1334	-1.2830	-1.4725	-1.0463	-1.3699	-1.6794	-1.1144	-1.5892
464	S-207_Q141	Q14151	Scaffold attachm	SAFB2 KIAA	0.7315	1.0274	0.8703	1.1446	0.4361	1.1583	0.8852	0.9877	1.0859	0.7703
465	S-2071_Q9U	Q9UQ35	Serine/arginine r	SRRM2 KIAA	-0.8233	-0.5053	-0.6701	-0.7358	-0.7403	-1.0985	-0.8912	-0.8437	-0.9678	-0.7577
466	S-2073_P12	P12270	Nucleoprotein T	TPR	-1.5959	-1.8923	1.2498	1.4580	1.1855	1.1012	1.6645	1.4929	0.9724	1.1623
467	S-2077_Q9Y	Q9Y4A5	Transformation/	TRRAP PAF4	0.3052	0.2031	0.7872	0.6493	0.6005	0.7821	0.8746	0.7431	0.5207	0.5250

468	S-208_O948	O94880	PHD finger prote	PHF14 KIAA(	-1.5904	-1.5021	-1.6903	-1.7748	-1.7936	-1.6551	-1.5902	-1.6830	-1.3939	-1.3362
469	S-208_Q011	Q01130	Serine/arginine-r	SRSF2 SFRS2	1.8616	1.7868	1.6191	1.9298	1.6710	1.5630	1.6230	2.3952	2.0322	2.1233
470	S-209_Q8IYE	Q8IYB3	Serine/arginine r	SRRM1 SRM	-0.7854	-0.9361	-0.5488	-0.3148	-0.7683	-0.7222	-0.5459	-0.4294	-0.7163	-0.6208
471	S-209_Q8NA	Q8NAV1	Pre-mRNA-splicir	PRPF38A	-0.7905	-0.5792	-0.6916	-0.6020	-0.6962	-0.8463	-0.5406	-0.7635	-0.9758	-0.8202
472	S-2099_Q8N	Q8NF50	Dedicator of cytc	DOCK8	0.1312	0.0765	0.4589	0.3767	0.5649	0.4080	0.5689	0.5415	0.1353	0.0166
473	S-21_P0624	P06241	Tyrosine-protein	FYN	0.2666	0.4056	-0.2262	-0.2951	-0.3922	-0.5820	-0.1605	-0.0769	-0.6680	-0.6248
474	S-21_P4979	P49792	E3 SUMO-proteir	RANBP2 NUI	-0.2266	-0.1865	-0.1314	-0.2082	-0.2150	0.1226	-0.2659	-0.0529	0.0624	0.1054
475	S-21_P8336	P83369	U7 snRNA-associ	LSM11	-0.9022	-0.8123	-0.8247	-1.2842	-1.1544	-0.7027	-0.8985	-1.2102	-0.8935	-0.9482
476	S-21_Q86UU	Q86UU0	B-cell CLL/lymph	BCL9L DLNB	0.3456	0.1056	0.1566	0.0627	-0.0307	-0.0377	0.1220	-0.0077	0.1933	0.0115
477	S-21_Q8IUD	Q8IUD2	ELKS/Rab6-intera	ERC1 ELKS K	-0.8655	-0.5597	-1.5396	-0.9288	-1.1179	-1.3711	-1.0624	-1.4191	-1.5681	-1.4232
478	S-21_Q9Y4F	Q9Y4F9	Rho family-intera	RIPOR2 C6or	-0.7684	-0.7064	-0.6781	-0.5963	-0.9719	-0.8487	-0.6261	-0.9828	-0.9226	-0.8285
479	S-210_P783	P78347	General transcrip	GTF2I BAP13	-0.6151	-0.8618	-0.8566	-0.9154	-0.6138	-1.0240	-0.7491	-0.8547	-0.7707	-0.9657
480	S-210_Q9NZ	Q9NZM3	Intersectin-2 (SH	ITSN2 KIAA1	0.2208	-0.0541	0.0413	0.0627	0.0569	0.1749	-0.2457	0.0362	-0.0941	-0.0339
481	S-210_Q9UE	Q9UBB9	Tuftelin-interacti	TFIP11 STIP	0.4602	0.3259	0.4493	0.1736	0.1676	0.3191	0.2008	0.3646	0.4089	0.5172
482	S-2102_Q01	Q01082	Spectrin beta cha	SPTBN1 SPTI	1.0654	1.1005	1.0494	1.3754	1.4374	1.4548	1.2127	1.4336	1.7429	1.7744
483	S-211_O003	O00308	NEDD4-like E3 ul	WWP2	-1.1206	-1.0189	-1.1476	-1.0488	-1.2172	-1.1871	-0.8392	-0.9284	-1.4391	-1.5531
484	S-211_O954	O95466	Formin-like prote	FMNL1 C17c	-2.4905	-2.0212	-0.3772	-1.1167	-0.2497	-1.5425	-0.2643	-1.3319	-1.2540	-1.4267
485	S-211_Q132	Q13242	Serine/arginine-r	SRSF9 SFRS9	-2.0553	-1.8241	-2.6082	-2.3326	-2.6138	-2.4380	-2.0569	-2.3128	-2.0170	-2.0917
486	S-211_Q8TF	Q8TF01	Arginine/serine-r	PNISR C6orf	0.5741	0.5502	0.8576	0.6402	0.7817	0.8967	0.7129	0.7275	0.9073	0.9342
487	S-212_Q96B	Q96B36	Proline-rich AKT1	AKT1S1 PRA	0.0180	0.0744	0.0981	0.3223	-0.0139	0.1643	0.0820	0.0788	-0.0871	0.0179
488	S-212_Q9UF	Q9UFC0	Leucine-rich repe	LRWD1 CEN	1.5832	1.7739	1.3802	1.4463	1.5200	1.6279	0.9261	0.9053	1.8023	1.5020
489	S-2128_Q01	Q01082	Spectrin beta cha	SPTBN1 SPTI	0.1163	0.1230	0.1481	0.0897	0.1560	0.2655	0.2639	0.3158	0.6214	0.6590
490	S-213_Q6NY	Q6NYC8	Phostensin (Prot	PPP1R18 HK	-1.3502	-1.1652	0.5617	0.8142	0.2901	0.7292	0.4283	0.7513	0.2001	0.2171
491	S-213_Q928	Q92882	Osteoclast-stimu	OSTF1	-0.1830	-0.3042	-0.2670	-0.4266	-0.5451	-0.4007	-0.5839	-0.4995	-0.6795	-1.0722
492	S-2132_Q9U	Q9UQ35	Serine/arginine r	SRRM2 KIAA	-0.0520	-0.2000	-0.2539	-0.4168	-0.1615	-0.0730	-0.2719	-0.0862	-0.2623	-0.1420
493	S-214_O608	O60841	Eukaryotic transl	EIF5B IF2 KI/	-0.2394	-0.4990	0.9741	0.9354	0.8167	1.0619	0.6356	0.9061	0.9402	0.7305
494	S-214_Q137	Q13761	Runt-related trar	RUNX3 AML	-0.6318	-0.2522	-0.2522	-0.1597	-0.1219	-0.1113	-0.3412	-0.0262	-0.3158	-0.3072
495	S-214_Q6ZV	Q6ZVF9	G protein-regula	GPRIN3 KIA/	-0.7948	-0.8518	-0.9526	-0.9806	-1.1381	-0.8720	-0.9477	-1.1345	-1.4320	-1.3264
496	S-214_Q9H1	Q9H1E3	Nuclear ubiquito	NUCKS1 NU(	0.2224	0.2808	0.3440	0.5856	0.6600	0.6480	0.4215	0.6546	0.6947	0.6816
497	S-215_P498	P49841	Glycogen syntha:	GSK3B	-0.2287	-0.1677	-0.5595	-0.6056	-0.6194	-0.5820	-0.6935	-0.4946	-0.7730	-0.6129
498	S-215_Q145	Q14558	Phosphoribosyl p	PRPSAP1	-0.5135	-0.5994	-0.6735	-1.0899	-0.2829	-0.5403	-0.6582	-0.5317	-0.3436	-0.1910
499	S-2152_P21	P21333	Filamin-A (FLN-A	FLNA FLN FL	0.8863	0.8890	1.0187	1.1276	0.8710	0.9858	0.9592	1.1174	0.6963	0.7459
500	S-216_Q6JB	Q6JBY9	CapZ-interacting	RCSD1 CAPZ	-0.1952	-0.3294	-0.0774	-0.1637	-0.0688	0.0192	-0.1106	-0.0450	-0.4216	-0.6814
501	S-216_Q8ND	Q8ND56	Protein LSM14 h	LSM14A C19	0.8079	0.8192	0.6795	0.6527	0.8198	0.6678	0.8732	0.7440	0.7956	0.5759
502	S-216_Q9UK	Q9UKV3	Apoptotic chrom	ACIN1 ACINI	0.6996	0.6439	0.5582	0.8833	0.5981	0.4586	0.7302	0.7675	0.5033	0.5250
503	S-2169_Q01	Q01082	Spectrin beta cha	SPTBN1 SPTI	-0.1829	-0.0692	0.1697	0.1772	0.3763	0.3213	0.2487	0.2696	0.8001	0.6792
504	S-217_Q047	Q04726	Transducin-like e	TLE3 KIAA15	-0.2275	-0.3340	-0.0989	-0.1576	0.0486	-0.1357	-0.1130	0.1476	-0.3069	-0.1852
505	S-217_Q96F	Q96F15	GTPase IMAP far	GIMAP5 IAN	-0.6229	-0.5369	-0.8716	-0.7084	-0.7161	-0.3608	-0.4305	-0.4690	-0.8733	-0.6328
506	S-218_Q150	Q15019	Septin-2 (Neural	SEPTIN2 DIF	-0.0723	0.3485	-0.3068	0.2463	0.0192	0.2680	0.4293	0.4716	0.2226	-0.0971

507	S-218_Q5VV	Q5VVJ2	Histone H2A deu	MYSM1 KIAA	0.7755	0.9601	0.0923	0.2145	0.4086	-0.0670	0.4804	0.0152	0.0155	0.0368
508	S-219_Q9BV	Q9BWG6	Sodium channel	SCNM1	0.1815	0.1973	-0.1511	-0.5037	-0.5970	-0.5490	-0.5122	-0.5789	-0.1814	0.0654
509	S-220_Q96P	Q96PK6	RNA-binding pro	RBM14 SIP	-1.8010	-2.0808	-1.8699	-1.5814	-1.5970	-1.7320	-1.6379	-1.5789	-1.7357	-1.4939
510	S-221_O954	O95425	Supervillin (Arch	SVIL	-2.1404	-2.2183	-1.6735	-1.8434	-1.8129	-1.4340	-2.0196	-1.8996	-1.3736	-1.5049
511	S-221_Q7Z2	Q7Z2W4	Zinc finger CCCH-	ZC3HAV1 ZC	-0.4381	-0.6681	-0.8266	-0.7748	-0.8791	-0.5798	-0.5611	-0.7846	-0.8719	-0.9682
512	S-221_Q7Z5	Q7Z5K2	Wings apart-like	WAPL FOE K	-0.1030	-0.3032	0.7099	0.7071	0.6150	0.6308	0.6103	0.6729	0.8362	0.8403
513	S-222_Q7L4	Q7L4I2	Arginine/serine-r	RSRC2	-2.2652	-2.4528	-1.4511	-1.4252	-1.4439	-1.3444	-1.7867	-1.1446	-1.3635	-0.8874
514	S-222_Q8IZF	Q8IZP0	Abl interactor 1 (	ABI1 SSH3BF	-2.1808	-1.4228	0.2074	0.0524	0.2627	-0.3807	0.3772	0.1753	-0.2022	-0.1837
515	S-222_Q9NY	Q9NYF8	Bcl-2-associated	BCLAF1 BTF	0.4483	0.4643	-0.0154	0.3584	0.2300	-0.1903	0.1530	-0.1103	0.3084	0.6980
516	S-2229_Q9B	Q9BV73	Centrosome-asso	CEP250 CEP2	-0.8042	-0.7164	-0.9048	-0.6246	-0.7589	-0.9190	-0.8840	-0.7304	-0.8862	-0.8732
517	S-223_Q166	Q16629	Serine/arginine-r	SRSF7 SFRS7	-2.1808	-1.8398	0.3717	0.2099	0.1344	0.1762	0.5914	0.6652	0.8326	0.8068
518	S-224_O435	O43561	Linker for activat	LAT	2.0392	1.9165	0.9369	1.1693	1.3728	1.1178	1.5579	1.1611	0.9334	0.6671
519	S-224_O753	O75376	Nuclear receptor	NCOR1 KIAA	0.1671	0.2241	0.1575	0.0524	-0.0863	0.2518	0.0283	0.0961	0.2880	-0.0726
520	S-224_P118	P11831	Serum response	SRF	-0.7485	-0.8638	-0.4454	-0.5113	-0.8390	-0.4893	-0.7823	-0.5630	-0.2981	0.0242
521	S-224_Q135	Q13573	SNW domain-cor	SNW1 SKIIP	-0.4406	-0.2923	-0.3055	-0.1818	-0.1585	-0.2980	0.0771	-0.1178	-0.5585	-0.3088
522	S-225_Q150	Q15052	Rho guanine nuc	ARHGEF6 CC	1.9076	1.7935	1.8972	2.1870	1.7613	1.7712	2.0635	2.0425	1.2458	1.2059
523	S-225_Q8IZF	Q8IZP0	Abl interactor 1 (	ABI1 SSH3BF	-1.7823	-1.3589	-1.7141	-1.7094	-1.4691	-1.5687	-1.2566	-1.6065	-1.7976	-1.6149
524	S-226_P082	P08238	Heat shock prote	HSP90AB1 H	0.1425	0.8892	0.5727	0.5384	0.5445	0.3779	0.6822	0.2968	0.5226	0.7447
525	S-226_P172	P17252	Protein kinase C	PRKCA PKCA	-0.2587	-0.2055	-0.4032	-0.3126	0.0515	0.2100	-0.1162	-0.2442	0.0723	0.0356
526	S-226_Q084	Q08495	Dematin (Demat	DMTN DMT	-1.0292	-1.4195	-0.9773	-1.1286	-0.5492	-0.5708	-1.2746	-1.1339	-1.4271	-1.6167
527	S-227_O602	O60256	Phosphoribosyl p	PRPSAP2	-0.8475	-0.8020	-0.8503	-1.0020	-0.9993	-0.7550	-0.7770	-0.7741	-0.4571	-0.2750
528	S-227_P527	P52701	DNA mismatch re	MSH6 GTBP	0.3183	0.1671	-0.0642	0.1145	0.4630	0.2342	0.0039	0.1699	0.2816	0.2379
529	S-227_Q53G	Q53GL0	Pleckstrin homol	PLEKHO1 CK	-1.2479	-1.0808	-0.7954	-0.7299	-0.6451	-0.2690	-0.7711	-0.4356	-0.9178	-0.6407
530	S-227_Q9BV	Q9BW71	HIRA-interacting	HIRIP3	-0.3595	-0.2378	-0.3316	-0.4444	-0.3611	-0.2636	-0.3561	-0.3185	-0.3271	-0.2679
531	S-2272_Q9U	Q9UQ35	Serine/arginine r	SRRM2 KIAA	-1.2266	-1.2183	-1.1453	-1.5761	-1.3754	-1.4542	-1.3931	-1.2569	-1.1521	-1.2358
532	S-228_Q1KN	Q1KMD3	Heterogeneous r	HNRNPUL2 I	0.7617	1.5813	1.2024	0.8482	0.8619	0.9646	0.7450	0.7648	0.9477	1.0406
533	S-228_Q6P4	Q6P4R8	Nuclear factor re	NFRKB INO8	0.5434	0.4646	0.7176	0.7060	0.8229	0.6163	0.6489	0.4694	0.5565	0.7321
534	S-228_Q86Y	Q86YV0	RAS protein activ	RASAL3	-0.2674	-0.5116	-0.8229	-0.5761	-0.4068	-0.9823	-0.6460	-0.9727	-0.9007	-0.5419
535	S-228_Q8NE	Q8NE71	ATP-binding cass	ABCF1 ABC5	-0.6572	-0.8091	-0.1971	-0.4787	-0.5079	-0.8424	-0.4684	-0.8223	-0.5226	-0.7818
536	S-229_Q130	Q13033	Striatin-3 (Cell cy	STRN3 GS2N	0.0392	0.0828	0.1716	0.2448	-0.0065	0.1184	-0.0994	-0.0482	0.2192	0.2466
537	S-229_Q96P	Q96P48	Arf-GAP with Rho	ARAP1 CENT	-1.9191	-1.7002	-2.1757	-1.8498	-2.0065	-1.6787	-2.0953	-2.1615	-1.8766	-1.7383
538	S-23_Q9261	Q92619	Rho GTPase-activ	ARHGAP45 H	1.4780	1.4829	0.6815	0.9548	0.8690	0.7629	0.8718	0.8282	0.7858	0.6549
539	S-23_Q9NSI	Q9NSI8	SAM domain-cor	SAMSN1 HA	0.5655	0.7009	0.1147	-0.1518	0.1610	-0.5124	0.2009	-0.5251	-0.5856	-0.7974
540	S-230_Q7Z4	Q7Z417	Nuclear fragile X	NUFIP2 KIAA	0.5359	0.2002	1.1334	0.5879	1.0037	1.0418	1.0187	0.9823	1.0391	0.7110
541	S-230_Q96B	Q96B97	SH3 domain-cont	SH3KBP1 CIN	0.6707	0.6670	0.7198	1.2832	0.8943	0.4360	0.8350	0.6292	-0.4452	-0.4667
542	S-230_Q96S	Q96S82	Ubiquitin-like pro	UBL7 BMSCU	-0.5581	-0.8241	-0.7755	-0.9841	-0.8097	-0.6067	-0.9328	-0.6805	-0.8695	-0.8268
543	S-231_P017	P01732	T-cell surface gly	CD8A MAL	-0.8622	-1.1627	1.9805	0.8263	1.8584	-0.6668	2.5122	0.8030	-0.1652	-0.3476
544	S-231_P079	P07900	Heat shock prote	HSP90AA1 H	-0.1305	0.8354	0.4581	0.2037	0.5296	0.0497	0.6547	-0.0231	0.1506	0.9335
545	S-231_P226	P22626	Heterogeneous r	HNRNPA2B1	-1.7454	-1.6334	-1.0190	-0.7477	-1.1259	-1.2145	-0.9704	-1.1178	-1.0409	-0.8521

546	S-231_Q130	Q13009	T-lymphoma inva	TIAM1	-1.0441	-0.9857	-0.2485	-0.4713	-0.2086	-0.0890	-0.7125	-0.6371	-0.7184	-0.7490
547	S-231_Q4VX	Q4VX76	Synaptotagmin-li	SYTL3 SLP3	-0.2982	-0.5794	-0.3055	-0.0324	-0.2874	-0.3238	-0.1928	-0.0980	-0.5301	-0.3925
548	S-231_Q8IZF	Q8IZP0	Abl interactor 1 (	ABI1 SSH3BF	-1.3971	-1.3225	-0.6486	-0.7123	-0.2941	-0.9303	-0.3249	-0.6182	-1.1150	-0.9457
549	S-232_P273	P27348	14-3-3 protein th	YWHAQ	1.2515	1.1725	0.5773	0.8010	0.3918	0.3670	0.5521	0.4438	0.2315	0.2281
550	S-232_P356	P35659	Protein DEK	DEK	0.8355	0.5993	0.5207	0.4213	0.6612	0.7283	0.3561	0.7173	0.4270	0.4487
551	S-232_Q9UJ	Q9UJU6	Drebrin-like prot	DBNL CMAP	-0.2015	-0.5147	-0.5549	-0.3371	-0.5612	-0.4706	-0.6830	-0.5562	-0.2524	-0.3757
552	S-2322_Q9B	Q9BV73	Centrosome-asso	CEP250 CEP	-0.6773	-0.8027	-0.4171	-0.4737	-0.4117	-0.8133	-0.6582	-0.5996	-0.7685	-0.8544
553	S-233_O960	O96019	Actin-like protei	ACTL6A BAF	0.4531	0.3106	0.6962	0.6851	0.7765	0.8229	0.6090	0.7188	0.9459	0.5834
554	S-233_P079	P07910	Heterogeneous r	HNRNPC HN	1.6452	1.8291	0.7287	0.7619	0.8999	0.4950	1.0024	0.7922	0.6969	0.7303
555	S-233_Q166	Q16629	Serine/arginine-r	SRSF7 SFRS7	-0.4187	-0.6112	0.4778	0.6414	1.0364	0.6299	0.9192	1.0694	1.1162	1.5423
556	S-234_O435	O43516	WAS/WASL-inter	WIPF1 WASI	0.1607	0.3432	0.6411	0.8620	0.5773	0.0497	1.2688	0.6082	0.4951	0.3165
557	S-234_Q9H1	Q9H1E3	Nuclear ubiquito	NUCKS1 NU	0.1146	0.0713	0.5610	0.1357	0.4869	-0.1209	-0.1304	-0.3454	0.7873	1.0368
558	S-234_Q9H7	Q9H7L9	Sin3 histone dea	SUDS3 SAP4	-1.1484	-0.8300	-1.4227	-0.9187	-1.7434	-1.4340	-1.2535	-1.7228	-1.3974	-1.1648
559	S-236_O757	O75791	GRB2-related ad	GRAP2 GAD	-1.4213	-1.5663	-1.2937	-1.6443	-1.2740	-1.0810	-1.4765	-1.0981	-1.1020	-1.0821
560	S-236_P226	P22626	Heterogeneous r	HNRNPA2B1	-0.8010	-1.1455	-0.8359	-0.8855	-0.6860	-1.2110	-0.3396	-0.4294	-1.0899	-0.9987
561	S-236_Q6Y7	Q6Y7W6	GRB10-interactin	GIGYF2 KIAA	0.8303	0.9034	1.0664	1.0228	0.9571	1.0452	0.8944	0.9265	0.8968	0.8345
562	S-237_Q86T	Q86TI0	TBC1 domain fan	TBC1D1 KIA	0.0602	0.0995	0.2546	0.1260	-0.0102	0.3589	0.1374	0.1699	0.1198	-0.1433
563	S-237_Q9BT	Q9BTK6	PAXIP1-associate	PAGR1 C16o	-1.8329	-1.7969	-2.2385	-2.1637	-2.2651	-2.1871	-2.1751	-2.5251	-2.1493	-2.0484
564	S-237_Q9HC	Q9HCU9	Breast cancer me	BRMS1	-1.4603	-1.6717	-1.2509	-0.8594	-1.0630	-1.4061	-1.1693	-1.2496	-1.0927	-0.8521
566	S-2384_P02	P02751	Fibronectin (FN)	FN1 FN	-0.4336	-0.9294	-0.6606	-0.4338	-0.4757	0.0486	0.2225	0.0843	-0.5107	-0.5896
567	S-239_P518	P51858	Hepatoma-derive	HDGF HMG1	-0.5213	-0.4007	-1.3489	-1.5550	-1.1750	-1.1939	-1.0651	-1.1684	-1.0652	-1.0168
568	S-239_Q66P	Q66PJ3	ADP-ribosylation	ARL6IP4	-0.0292	-0.0438	-0.4046	-0.5867	-0.5290	-0.3711	-0.4792	-0.6562	-0.3939	-0.7085
569	S-24_P3324	P33241	Lymphocyte-spe	LSP1 WP34	-1.6402	-1.3253	-1.6470	-2.3598	-1.9917	-2.2565	-1.7513	-2.2460	-2.0223	-1.6228
570	S-24_Q1543	Q15435	Protein phospha	PPP1R7 SDS	-0.3641	-0.2937	-0.2250	-0.0287	-0.0630	-0.3634	0.2267	-0.2643	-0.2493	-0.2571
571	S-24_Q8IV5	Q8IV50	LysM and putati	LYSMD2	-1.2739	-1.4228	-0.3717	-0.2755	-0.3682	-0.3539	-0.6399	-0.5185	-0.4784	-0.3120
572	S-24_Q9Y2I	Q9Y2I8	WD repeat-conta	WDR37 KIA	-3.2827	-3.0347	-2.7917	-2.8434	-2.6026	-3.7926	-2.9082	-3.0000	-3.2696	-3.7555
573	S-240_Q6IQ	Q6IQ19	Centriole, cilia ar	CCSAP C1orf	-0.4779	-0.0473	0.1424	0.6242	0.9982	0.6173	0.6280	0.7630	0.2901	0.4768
574	S-240_Q7Z5	Q7Z5L9	Interferon regula	IRF2BP2	-2.6179	-2.2815	-2.4116	-1.9356	-2.0515	-2.1603	-2.1120	-1.9638	-1.8250	-1.7686
575	S-240_Q8W	Q8WU79	Stromal membra	SMAP2 SMA	-0.5501	-0.4696	-0.9265	-0.9666	-0.4948	-0.2654	-0.5727	-0.3689	-0.3535	-0.5760
576	S-240_Q9H1	Q9H1E3	Nuclear ubiquito	NUCKS1 NU	-2.2915	-2.4959	-3.0796	-2.1400	-2.0981	-3.3673	-2.4311	-2.4397	-2.7487	-2.2418
577	S-2409_P11	P11717	Cation-independ	IGF2R MPRI	0.4774	0.5653	0.2893	0.3685	0.1903	0.2203	0.3812	0.4438	0.0956	0.2925
578	S-241_O149	O14979	Heterogeneous r	HNRNPDL H	-0.5058	-0.2951	-0.7827	-0.5948	-0.5970	-0.1636	-0.6118	-0.3650	-0.2187	-0.4493
579	S-241_Q015	Q01543	Friend leukemia	FLI1	0.4787	0.5197	0.3685	0.2848	0.3433	0.4748	0.3346	0.5478	0.4154	-0.0712
580	S-242_Q5T2	Q5T200	Zinc finger CCCH	ZC3H13 KIA	1.6171	1.6368	1.5940	1.6890	1.3214	1.6565	1.4379	1.5961	1.6850	1.3905
581	S-242_Q9UI	Q9UI08	Ena/VASP-like pr	EVL RNB6	-0.6890	-0.3266	-0.5955	-0.8789	-1.0401	-0.6249	-0.4883	-0.5428	-0.3854	-0.7974
582	S-2426_Q9U	Q9UQ35	Serine/arginine r	SRRM2 KIAA	-0.5742	-0.8578	0.1185	-0.1032	0.0710	0.0824	-0.1504	-0.0325	0.1222	0.2639
583	S-243_P208	P20810	Calpastatin (Calp	CAST	0.6578	0.5358	0.4327	0.2921	0.3848	0.2985	0.3479	0.3712	-0.6393	-0.6488
584	S-243_Q53C	Q53QZ3	Rho GTPase-acti	ARHGAP15	-1.8148	-0.8105	-0.6530	-0.6232	-0.4214	-0.3622	-0.4822	-0.3703	-0.6184	-0.7293
585	S-243_Q928	Q92835	Phosphatidylino	INPP5D SHI	0.6534	0.5006	0.2660	0.2823	0.1501	0.5996	0.4516	0.4606	0.1575	0.2310

586	S-243_Q9UK	Q9UKV3	Apoptotic chrom	ACIN1 ACINU	0.5031	0.6754	0.8243	0.0897	0.3258	0.0236	0.4494	0.1713	0.3382	0.3522
587	S-243_Q9Y2	Q9Y2W1	Thyroid hormone	THRAP3 BCL	-0.9426	-0.7666	-1.2250	-1.1680	-1.0859	-1.2067	-0.9756	-0.9161	-1.2812	-1.3332
588	S-245_O438	O43815	Striatin	STRN	-0.2303	-0.2308	0.1520	0.1794	-0.2292	0.0188	-0.2373	-0.1099	-0.0468	-0.0193
589	S-245_O954	O95425	Supervillin (Arch	SVIL	0.8345	0.7616	0.9693	0.7804	1.0814	1.2894	1.0585	0.9343	0.9246	0.5845
590	S-247_Q8NE	Q8NB78	Lysine-specific hi	KDM1B AOF	0.5813	0.7550	0.6802	0.7711	0.5871	0.7690	0.7673	0.7275	0.8983	0.5854
591	S-247_Q9H3	Q9H3N1	Thioredoxin-rela	TMX1 TMX T	0.5782	0.6038	-0.4902	0.0613	-0.6116	-0.5742	0.0673	-0.3771	-1.1066	-0.9545
592	S-248_P1398	P13984	General transcrip	GTF2F2 RAP	-1.7394	-0.6095	-0.3636	-0.1302	-0.0420	-0.2636	-0.2033	-0.1979	-0.3685	-0.2633
593	S-248_Q9BR	Q9BRD0	BUD13 homolog	BUD13	-2.5213	-1.6541	-1.5245	-1.3237	-1.2784	-1.5082	-1.2879	-1.4397	-1.5915	-1.4759
594	S-248_Q9Y2	Q9Y2W1	Thyroid hormone	THRAP3 BCL	0.1799	0.1857	0.5061	0.7191	0.6150	0.3785	0.6356	0.7348	0.6009	0.5551
595	S-2484_P117	P11717	Cation-independ	IGF2R MPRI	-0.2674	-0.0416	-0.2311	-0.3921	-0.6509	-0.6906	-0.5402	-0.2569	-0.3652	-0.2464
596	S-2498_Q12	Q12802	A-kinase anchor	AKAP13 BRX	0.3766	0.2260	1.4489	1.6419	1.3756	1.4822	1.8718	1.6585	1.0705	1.2601
597	S-25_P1694	P16949	Stathmin (Leuke	STMN1 C1or	-0.2140	-0.2443	0.5575	0.3505	0.3243	0.6873	0.4683	0.4325	0.5618	0.7055
598	S-25_Q9NYL	Q9NYL9	Tropomodulin-3	TMOD3	0.6963	0.8154	0.5024	0.4332	0.2364	0.6241	0.3772	0.3993	0.6737	0.3723
599	S-250_P2578	P25788	Proteasome subu	PSMA3 HC8	1.4657	1.3084	0.9330	0.6885	1.0996	1.0425	1.1082	1.3323	1.0232	0.9987
600	S-250_P2935	P29350	Tyrosine-protein	PTPN6 HCP I	-0.1605	-0.2762	-0.4340	-0.1657	-0.5343	-0.3983	-0.3023	-0.4150	-0.9527	-1.0220
601	S-250_P5768	P57682	Krueppel-like fac	KLF3 BKLF	0.1979	0.3902	0.0219	0.1327	-0.1502	-0.1809	-0.0119	-0.1024	-0.0893	-0.0917
602	S-250_Q9UE	Q9UBI9	Headcase protein	HECA HDC	-2.8458	-2.8241	-1.8931	-2.2373	-1.9627	-2.1405	-2.1290	-1.6019	-2.0329	-2.2479
603	S-252_P3324	P33241	Lymphocyte-spe	LSP1 WP34	0.4134	0.3769	1.0611	0.5896	0.7970	0.5244	0.8934	0.7756	0.7092	0.5477
604	S-252_Q3KC	Q3KQU3	MAP7 domain-co	MAP7D1 KIA	-0.0329	-0.2017	-0.0179	-0.0824	-0.3447	-0.1049	-0.0856	-0.0785	-0.7999	-0.7297
605	S-253_O155	O15541	E3 ubiquitin-prot	RNF113A RN	-0.2209	-0.3562	-0.2068	-0.2001	-0.2970	-0.2117	-0.3011	-0.3923	-0.0803	-0.2224
606	S-254_Q8IXT	Q8IXT5	RNA-binding pro	RBM12B	-1.8075	-1.8592	-1.4305	-1.3404	-1.2997	-1.4562	-1.5537	-1.1997	-1.4380	-1.2769
607	S-255_P0823	P08238	Heat shock prote	HSP90AB1 H	0.1558	0.8539	0.6133	0.5312	0.5636	0.3922	0.9578	0.4166	0.2600	0.5178
608	S-255_P1753	P17535	Transcription fac	JUND	0.2711	0.2260	0.4397	0.1550	0.3890	0.5149	0.6052	0.7711	0.0091	0.2488
609	S-255_Q9UE	Q9UBC2	Epidermal growt	EPS15L1 EPS	-0.1108	0.1291	-0.0479	-0.0956	-0.3215	0.0396	-0.0104	0.0422	-0.3174	0.0293
610	S-256_Q96P	Q96PK6	RNA-binding pro	RBM14 SIP	-0.3923	-0.1928	-0.1349	0.0264	-0.2497	-0.0075	0.0270	0.3072	-0.0598	-0.0325
611	S-256_Q9HE	Q9HB58	Sp110 nuclear bo	SP110	1.4339	1.5820	0.7447	1.2863	1.1569	0.7541	1.4574	0.9542	0.5547	1.1209
612	S-257_Q130	Q13033	Striatin-3 (Cell cy	STRN3 GS2N	-1.2182	-1.4228	-0.2698	-0.6192	-0.5477	-0.0670	-0.7867	-0.3071	-0.2263	-0.1605
613	S-257_Q135	Q13523	Serine/threonine	PRPF4B KIAA	-0.6318	-0.5100	0.1716	0.5263	0.7138	0.2177	-0.0209	0.1753	-0.1278	0.5905
614	S-257_Q135	Q13586	Stromal interacti	STIM1 GOK	-0.1605	-0.0611	0.3522	0.5665	-0.1020	-0.0423	0.4934	0.4097	-0.1249	-0.2882
615	S-257_Q7Z2	Q7Z2W4	Zinc finger CCCH-	ZC3HAV1 ZC	-1.4170	-1.4546	-1.5293	-1.6455	-1.7589	-1.7406	-1.5005	-1.6065	-1.8254	-1.5361
616	S-257_Q96P	Q96PM5	RING finger and (	RCHY1 ARNI	-0.3296	-0.2196	-0.0447	0.0053	0.2946	0.3058	0.0359	0.1508	-0.0611	-0.0739
617	S-2578_Q15	Q15149	Plectin (PCN) (PL	PLEC PLEC1	-5.9398	-5.1382	-2.3569	-2.2799	-2.3284	-2.7823	-1.8792	-2.1684	-3.5452	-3.0325
618	S-2581_Q9U	Q9UQ35	Serine/arginine r	SRRM2 KIAA	1.3227	1.3662	1.4547	1.2709	1.2080	1.3184	1.2465	1.3158	1.2767	1.1600
619	S-259_P2262	P22626	Heterogeneous r	HNRNPA2B1	-1.5265	-1.6129	-1.0511	-1.4541	-1.3284	-1.1372	-1.7210	-1.8492	-1.5605	-1.3690
620	S-26_P6299	P62995	Transformer-2 pr	TRA2B SFRS1	-0.8233	-0.7183	0.3165	1.0098	0.4947	0.5730	0.6580	0.7977	1.0655	1.4553
621	S-26_Q0113	Q01130	Serine/arginine-r	SRSF2 SFRS2	1.6142	1.7823	0.9826	1.5556	0.9627	1.2368	1.4369	1.2492	0.7468	0.8701
622	S-260_P0791	P07910	Heterogeneous r	HNRNPC HN	0.4326	1.1452	-0.9833	-1.0415	-0.8656	-1.2494	-0.8985	-1.1345	-1.2172	-0.9457
623	S-260_Q63Z	Q63ZY6	Putative methylt	NSUN5P2 NS	1.2162	1.1567	1.4935	1.5689	1.5889	1.7484	1.6613	1.6671	1.6704	1.5841
624	S-260_Q8IYE	Q8IYB3	Serine/arginine r	SRRM1 SRM	0.3612	0.3363	0.3505	0.3657	0.3214	0.4136	0.2267	0.6212	0.4783	0.3482

625	S-260_Q9UK	Q9UKL0	REST corepressor	RCOR1 KIAA	-0.9964	-1.0622	-0.9628	-1.0269	-1.1100	-1.1703	-1.1008	-1.0882	-0.9755	-1.6188
626	S-261_O956	O95644	Nuclear factor of	NFATC1 NFA	0.6963	0.5809	0.8326	1.1764	1.3118	0.7682	0.9738	0.9916	0.8507	0.9903
627	S-261_Q8IYB	Q8IYB4	PEX5-related pro	PEX5L PEX5R	-0.3093	-0.1690	0.0834	-0.1283	-0.0824	0.1143	-0.1562	-0.0641	0.0723	0.2820
628	S-261_Q9NZ	Q9NZ63	Telomere length	C9orf78 HCA	1.1666	1.2170	0.5614	0.5626	0.6531	0.4319	0.5957	0.5049	0.5083	0.7722
629	S-262_Q9Y5	Q9Y5B6	PAX3- and PAX7-	PAXBP1 C21	-0.3995	-0.4665	-0.3731	-0.6720	-0.6138	-0.9303	-0.7667	-0.6466	-0.3854	-0.5271
630	S-263_P079(	P07900	Heat shock prote	HSP90AA1 H	0.1046	0.7225	0.5891	0.5653	0.6066	0.5107	0.7703	0.7051	0.5758	0.8567
631	S-263_P612	P61247	40S ribosomal pr	RPS3A FTE1	-0.3688	-0.2896	0.3595	-0.1558	-0.2023	0.5640	-0.1205	0.1004	0.3341	0.0166
632	S-264_O958	O95817	BAG family mole	BAG3 BIS	-0.0857	-0.0268	-0.6785	-0.2520	-0.5237	-0.5753	-0.3281	-0.5720	-0.0941	-0.1027
633	S-264_P279	P27987	Inositol-trisphos	ITPKB	-0.5265	-0.5370	-0.7471	-0.5655	-0.8522	-0.6434	-0.6218	-0.4818	-0.6556	-0.5569
634	S-264_Q148	Q14865	AT-rich interactiv	ARID5B DESI	-1.4703	-1.6129	-1.6339	-2.0636	-2.0824	-2.3219	-1.7125	-1.7952	-2.0329	-2.1590
635	S-2658_Q6K	Q6KC79	Nipped-B-like pro	NIPBL IDN3	-0.3049	-0.3920	-0.8585	-0.6056	-0.8423	-0.8531	-0.6582	-0.8658	-1.1235	-0.6347
636	S-266_Q150	Q15025	TNFAIP3-interact	TNIP1 KIAA0	1.4238	1.2237	1.5525	1.6796	1.8354	1.4721	1.9359	1.7000	1.6564	1.4703
637	S-2672_Q6K	Q6KC79	Nipped-B-like pro	NIPBL IDN3	-2.7454	-2.7329	-4.3840	-3.2373	-3.0139	-3.3983	-3.0842	-3.6159	-4.0065	-3.1881
638	S-268_O958	O95831	Apoptosis-induci	AIFM1 AIF P	0.1345	0.2532	-0.9567	-0.2415	-0.4389	-0.4320	-0.3119	-0.7002	-0.6909	-0.8614
639	S-268_Q9H0	Q9H0J9	Protein mono-AD	PARP12 ZC3	-1.0666	-1.2081	-0.8100	-0.9493	-1.3100	-0.9050	-0.9254	-0.8603	-1.5759	-1.4441
640	S-268_Q9NX	Q9NXV6	CDKN2A-interact	CDKN2AIP C	-2.7454	-2.4288	1.9717	1.3414	1.7008	2.2667	1.6993	2.1422	2.6451	2.3121
641	S-268_Q9NY	Q9NYF8	Bcl-2-associated	BCLAF1 BTF	-1.3971	-0.7740	-1.3356	-1.0053	-1.2344	-1.4260	-1.0065	-1.1857	-1.4462	-0.8898
642	S-269_Q8N4	Q8N4C6	Ninein (hNinein)	NIN KIAA156	-1.9398	-1.7589	-1.4829	-1.3016	-1.6831	-1.2780	-1.5103	-1.4648	-1.5528	-1.4267
643	S-27_O6067	O60678	Protein arginine	PRMT3 HRM	-0.6262	-0.6317	-0.3094	-0.6664	-0.9343	-0.7246	-0.8961	-0.7715	-0.2949	-0.3056
644	S-27_P0673	P06733	Alpha-enolase (E	ENO1 ENO1	-0.5501	-0.5354	-0.7176	-0.9053	-0.7069	-0.5167	-0.7867	-0.5339	-0.3974	-0.1027
645	S-27_P4973	P49736	DNA replication l	MCM2 BM2	1.0611	0.9366	0.6397	0.3209	0.0586	0.0838	0.5144	0.4348	0.5315	0.3812
646	S-27_Q9252	Q92522	Histone H1.10 (H	H1-10 H1FX	-0.0704	-0.2470	-0.4312	-0.3326	0.3006	-0.1049	0.1689	0.1494	-0.6292	-0.7106
647	S-27_Q9Y3Z	Q9Y3Z3	Deoxynucleoside	SAMHD1 MO	-0.0109	0.0000	-0.1769	0.0438	-0.1300	-0.1438	-0.1290	-0.2551	-0.1521	0.0356
648	S-2702_Q9U	Q9UQ35	Serine/arginine r	SRRM2 KIAA	NA	NA	-2.3049	-2.2862	-3.2441	-2.1810	-2.6298	-2.2698	-1.9791	-2.3990
649	S-271_Q008	Q00839	Heterogeneous r	HNRNPU C1	-0.3433	-0.3920	-1.6275	-1.5550	-1.6538	-0.5082	-1.1576	-0.6395	-1.2052	-1.0917
650	S-271_Q9BR	Q9BRD0	BUD13 homolog	BUD13	-1.6290	-1.1553	-1.4255	-1.4444	-1.6082	-1.4624	-1.4666	-1.7689	-1.8910	-1.3394
651	S-271_Q9UC	Q9UQ88	Cyclin-dependen	CDK11A CDC	-0.2937	-0.2735	-1.2385	-1.2248	-1.2475	-1.7320	-1.1461	-1.6586	-1.3568	-1.0299
652	S-272_Q135	Q13501	Sequestosome-1	SQSTM1 ORC	-0.0647	-0.2119	0.2128	-0.0544	-0.1462	0.0119	-0.2123	-0.2244	0.1471	0.0580
653	S-272_Q96D	Q96D71	RalBP1-associate	REPS1	1.9428	2.0875	0.3382	1.1170	0.7104	1.2500	1.0071	0.8580	0.4624	-0.0999
654	S-272_Q9H7	Q9H7M9	V-type immunog	VSIR C10orf5	-2.7948	-2.8319	-2.7917	-2.8056	-2.5158	-2.8238	-2.2597	-2.3204	-3.4712	-2.7297
655	S-273_Q131	Q13148	TAR DNA-binding	TARDBP TDP	-0.1973	-0.3350	0.3862	0.0846	0.1394	0.5232	-0.0342	0.2747	0.2237	0.3979
656	S-2739_Q6Z	Q6ZNJ1	Neurobeachin-lik	NBEAL2 KIAA	-1.0465	-0.9248	-0.3786	-0.5771	-0.6004	-0.9304	-0.6976	-0.7028	-1.1202	-1.5550
657	S-274_P170	P17010	Zinc finger X-chro	ZFX	0.3313	0.5419	-0.8931	-0.4987	-0.6308	-1.3866	-0.5196	-1.0031	-1.1264	-1.0971
658	S-274_Q8N2	Q8N228	Sex comb on mid	SCML4	-2.8329	-2.8319	-2.6146	-2.7328	-2.9273	-2.9106	-2.6747	-2.7228	-3.1150	-3.1306
659	S-275_O754	O75475	PC4 and SFRS1-ir	PSIP1 DFS70	0.0771	0.0312	0.2303	0.3985	0.2837	0.1643	0.2249	0.2521	0.3737	0.4850
660	S-275_P143	P14317	Hematopoietic li	HCLS1 HS1	-0.1128	-0.1979	-0.9669	-0.5012	-0.0515	-0.6621	0.1184	-0.4049	-0.8181	-0.5607
661	S-275_Q7Z2	Q7Z2W4	Zinc finger CCCH	ZC3HAV1 ZC	-0.0164	-0.0797	0.1678	-0.1597	-0.0766	0.0089	0.0039	-0.0529	0.0142	-0.4284
662	S-275_Q9NF	Q9NR45	Sialic acid syntha	NANS SAS	0.8253	1.1886	0.9634	1.2553	1.3721	1.4806	1.1730	1.3783	1.4159	1.3891
663	S-275_Q9UJ	Q9UJU6	Drebrin-like prot	DBNL CMAP	1.7408	1.8126	1.2833	0.9345	1.0806	1.4613	1.4389	1.4410	1.1162	0.8273

664	S-276_P497!	P49757	Protein numb ho	NUMB C14o	-0.6544	-0.6334	0.6085	0.0897	0.3549	-0.0285	0.2421	0.0672	-0.0078	-0.1040
665	S-276_Q9NF	Q9NRL3	Striatin-4 (Zinedi	STRN4 ZIN	-0.8523	-0.6966	-0.9166	-0.9187	-0.9449	-0.7295	-0.9452	-1.0340	-0.7976	-0.9961
666	S-277_O751	O75190	DnaJ homolog su	DNAJB6 HSJ:	-0.4905	-0.5483	-0.4976	-0.6083	-0.3970	-0.6480	-0.2304	-0.3968	-0.7142	-0.4441
667	S-277_P025	P02545	Prelamin-A/C [Cl	LMNA LMN1	-1.3735	-0.6403	0.3814	0.7405	0.3155	-0.0392	0.6221	0.3981	-0.9987	-0.6528
668	S-277_Q133	Q13342	Nuclear body prc	SP140 LYSP1	0.1687	-0.0416	1.0655	0.2906	0.5129	-0.2780	0.3812	-0.3166	0.7745	1.2510
669	S-277_Q135	Q13523	Serine/threonine	PRPF4B KIA/	0.2666	0.2998	-0.0544	-0.1777	-0.1080	-0.3539	-0.2319	-0.6182	-0.4233	0.2026
670	S-2774_O75	O75592	E3 ubiquitin-prot	MYCBP2 KIA	-2.3548	-1.9256	-0.7972	-1.3326	-1.2739	-1.1177	-1.5103	-1.9401	-1.1292	-1.0591
671	S-278_Q6IQ	Q6IQ49	Replication stres:	SDE2 C1orf5	-0.1890	-0.3790	-0.3515	-0.3126	-0.5263	-0.5360	-0.4720	-0.5540	-0.3652	-0.3168
672	S-2781_Q8V	Q8WXH0	Nesprin-2 (KASH	SYNE2 KIAA:	-1.8074	-1.6752	-1.5305	-1.6301	-2.0213	-1.7271	-1.6218	-1.6781	-3.0816	-2.0116
673	S-279_Q134	Q13428	Treacle protein ('	TCOF1	-0.0685	0.0066	0.3332	0.6091	0.2689	0.2190	0.0410	0.2620	0.4056	0.3693
674	S-279_Q9NF	Q9NPI1	Bromodomain-cc	BRD7 BP75 (	0.8902	0.8606	1.0433	0.9410	0.9731	0.7984	1.0882	0.9977	1.1084	0.9125
675	S-280_O147	O14745	Na(+)/H(+) excha	SLC9A3R1 N	-0.4854	-0.2522	-0.4074	-0.5867	-0.6831	-0.5555	-0.4828	-0.4294	-0.7078	-0.6569
676	S-283_Q8IZI	Q8IZD4	mRNA-decapping	DCP1B	-0.5187	-0.0507	-0.1406	-0.0692	0.1428	-0.1703	0.1445	-0.1926	-0.3702	-0.0312
677	S-283_Q9NZ	Q9NZQ7	Programmed cell	CD274 B7H1	-0.0237	-0.5728	0.1050	0.3533	0.4856	0.1841	0.4264	0.2233	0.0686	0.0970
678	S-283_Q9UL	Q9ULX6	A-kinase anchor	AKAP8L NAK	-1.2056	-1.4742	-1.0511	-0.6083	-0.7683	-0.8477	-1.0117	-1.0593	-1.1932	-0.6773
679	S-284_P619	P61978	Heterogeneous r	HNRNPK HN	1.1997	0.9939	1.0052	0.7363	1.0639	1.0418	1.2530	1.2543	1.0822	1.0728
680	S-284_Q6JB'	Q6JBY9	CapZ-interacting	RCSD1 CAPZ	0.4472	0.3632	0.7277	0.3037	0.4224	0.5978	0.6154	0.7191	0.2697	0.1548
681	S-284_Q9Y6	Q9Y624	Junctional adhes	F11R JAM1 J	-1.0337	-1.0103	0.4502	0.2256	0.6815	-0.4023	0.3497	0.0395	-0.0358	-0.1865
682	S-286_Q8TA	Q8TAQ2	SWI/SNF comple	SMARCC2 B/	-0.5634	-0.6214	-0.0200	-0.0215	-0.3353	-0.3615	-0.7491	-0.5383	-0.0422	-0.4546
683	S-2866_Q03	Q03164	Histone-lysine N-	KMT2A ALL1	-0.8921	-1.0879	-0.0841	-0.3529	-0.4615	0.0015	-0.2643	-0.0657	0.3738	0.3998
684	S-287_Q9GZ	Q9GZR7	ATP-dependent I	DDX24	0.2964	0.3701	-0.4903	0.2237	0.2301	-0.5599	0.1884	-0.3338	-0.5040	-0.1851
685	S-288_O948	O94888	UBX domain-con	UBXN7 KIAA	0.7839	0.6782	0.4505	0.5386	0.0355	0.5539	0.3540	0.4404	0.7538	0.7359
686	S-288_Q9UK	Q9UKM9	RNA-binding pro	RALY HNRPC	-0.3228	-0.3392	-0.3017	-0.4812	-0.5531	-0.4789	-0.3200	-0.5251	-0.5490	-0.7191
687	S-290_O948	O94880	PHD finger prote	PHF14 KIAA(	-0.6346	-0.8923	-0.3881	-0.5062	-0.5749	-0.3711	-0.7448	-0.6088	-0.0026	-0.2464
688	S-290_Q134	Q13427	Peptidyl-prolyl ci	PPIG	0.5309	0.7083	1.4665	1.4384	1.4928	1.2556	1.8664	1.6057	1.4932	1.9301
689	S-290_Q8TF	Q8TF01	Arginine/serine-r	PNISR C6orf	-0.0255	0.0231	0.2173	0.1309	-0.1159	-0.1161	-0.0502	-0.2827	0.2633	0.3268
690	S-290_Q9NY	Q9NYF8	Bcl-2-associated	BCLAF1 BTF	0.2317	0.2551	0.3870	0.2921	0.2658	0.0978	0.6000	0.2772	0.2447	0.2325
691	S-2900_P49	P49792	E3 SUMO-protein	RANBP2 NUI	0.9277	1.2222	-0.2019	0.2191	0.2032	-0.2494	0.2077	-0.2809	-0.3158	-0.2267
692	S-291_P161!	P16150	Leukosialin (GPL:	SPN CD43	-1.8921	-1.9256	0.0135	-0.5138	-0.9168	-1.4421	0.2366	-0.8686	-0.9426	-1.5798
693	S-291_Q133	Q13342	Nuclear body prc	SP140 LYSP1	0.9457	1.1617	0.9215	1.0728	0.8761	0.4434	1.1678	0.6585	0.5252	0.4997
694	S-292_Q031	Q03111	Protein ENL (YEA	MLLT1 ENL I	0.2485	0.2717	-0.2647	0.3477	-0.3470	-1.2816	0.2896	-0.1079	-0.4640	-0.1720
695	S-293_P085!	P08559	Pyruvate dehydr	PDHA1 PHE1	-0.2224	-0.1260	-0.0774	-0.3552	-0.1875	-0.2961	-0.2706	-0.1736	-0.1828	-0.6387
696	S-293_P783:	P78312	Protein FAM193/	FAM193A C4	-0.5904	-0.3993	-0.2824	-0.2415	-0.5053	-0.3692	-0.5233	-0.4212	-0.3045	-0.2664
697	S-294_Q96T	Q96T37	RNA-binding pro	RBM15 OTT	0.1113	0.0522	-0.3094	-0.2082	-0.3146	-0.3885	-0.1432	-0.4418	-0.2217	-0.2327
698	S-295_P365	P36578	60S ribosomal pr	RPL4 RPL1	0.1113	0.0797	-0.0200	-0.2165	-0.3706	-0.3482	-0.1106	-0.1822	-0.3239	-0.2028
699	S-295_Q86Y	Q86YS7	C2 domain-conta	C2CD5 CDP1	0.5753	0.4947	0.6126	0.2672	0.4737	0.8430	0.6154	0.7079	0.1494	-0.1895
700	S-295_Q9UK	Q9UKV3	Apoptotic chrom	ACIN1 ACINI	-0.3947	0.0077	-0.5735	-0.4492	-0.1646	-0.7100	-0.0801	-0.5903	-0.6721	-0.4406
701	S-295_Q9Y5	Q9Y5B6	PAX3- and PAX7-	PAXBP1 C21	-1.4357	-1.3032	-0.9206	-0.7870	-1.4141	-0.9134	-1.5785	-1.0980	-0.9729	-0.7621
702	S-2956_O95	O95613	Pericentrin (Kenc	PCNT KIAA0	-4.7823	-3.8882	-3.7845	-4.9631	-5.0902	-3.7124	-3.7426	-3.7329	-4.1843	-5.1764

703	S-296_Q96R	Q96RU3	Formin-binding p	FNBP1 FBP1	0.1063	0.0044	-0.0147	0.0712	-0.2044	-0.1438	-0.0275	-0.2921	-0.4268	-0.3525
704	S-297_Q9UI:	Q9UIS9	Methyl-CpG-binc	MBD1 CXXC:	-0.9123	-0.9791	-1.1087	-1.2799	-0.9808	-0.9589	-1.1722	-1.0466	-1.2509	-1.0889
705	S-297_Q9UC	Q9UQ35	Serine/arginine r	SRRM2 KIAA	-3.5331	-3.7067	-1.7967	-1.6536	-1.8124	-1.7187	-1.5067	-1.3351	-1.4645	-1.1931
706	S-298_Q6JB'	Q6JBY9	CapZ-interacting	RCSD1 CAPZ	2.1151	1.8416	2.1695	1.9672	2.1469	2.2550	2.1058	2.1834	2.2759	2.0403
707	S-298_Q86U	Q86UE4	Protein LYRIC (3D	MTDH AEG1	-0.3664	-0.3322	0.7328	0.4644	0.8343	0.6929	0.8710	1.1774	0.1588	0.0752
708	S-2986_Q8N	Q8NFC6	Biorientation of (	BOD1L1 BOD	-0.1444	-0.2964	-0.1326	-0.2994	-0.4364	-0.3369	-0.4452	-0.2958	-0.0571	-0.0260
709	S-299_P079:	P07910	Heterogeneous r	HNRNPC HN	-0.1167	0.0328	-1.2410	-1.6863	-0.9485	-1.0985	-1.1869	-1.4648	-1.2664	-1.6670
710	S-30_O7537	O75379	Vesicle-associate	VAMP4	-1.3355	-1.7469	-0.9133	-1.1398	-1.0174	-0.8572	-1.2528	-1.1556	-0.9187	-1.0983
711	S-300_O751	O75113	NEDD4-binding p	N4BP1 KIAA(	-0.3664	-0.3934	0.2747	0.0830	0.0604	0.0015	-0.0610	-0.1244	-0.0557	-0.0551
712	S-300_Q9P2	Q9P289	Serine/threonine	STK26 MASK	-0.6919	-0.6664	0.4770	-0.1263	0.2457	0.0221	0.1220	-0.1787	0.4755	0.4211
713	S-301_Q132	Q13247	Serine/arginine-r	SRSF6 SFRS6	0.8020	0.8995	0.7223	0.7170	0.9266	0.6870	0.6147	0.7898	1.1899	1.0771
714	S-3019_Q8N	Q8NFC6	Biorientation of (	BOD1L1 BOD	-0.2544	-0.1979	-0.0479	-0.2103	-0.2366	0.0338	-0.0911	-0.0434	-0.0236	-0.2898
715	S-3029_Q8N	Q8NFC6	Biorientation of (	BOD1L1 BOD	0.9936	1.0479	1.1448	1.3934	1.3857	1.3233	1.1896	1.3282	1.2963	1.5146
716	S-303_Q006	Q00613	Heat shock facto	HSF1 HSTF1	0.8135	0.8225	0.5878	0.6563	0.6152	0.3139	0.6521	0.4430	0.5481	0.7310
717	S-303_Q065	Q06546	GA-binding prote	GABPA E4TF	-2.4406	-2.8319	-0.4116	-0.4015	-0.5886	-0.6181	-0.4435	-0.3415	-0.8113	-0.3757
718	S-303_Q86V	Q86VQ1	Glucocorticoid-ir	GLCCI1	0.4604	0.2541	0.3926	0.0071	0.2856	0.2493	0.1643	0.3096	0.2922	0.2936
719	S-304_Q168	Q16822	Phosphoenolpyru	PCK2 PEPCK:	0.9791	0.8824	0.4589	0.4384	-0.0746	0.3034	0.6703	0.5911	0.4877	0.6193
720	S-304_Q6UI	Q6UN15	Pre-mRNA 3'-enc	FIP1L1 FIP1 I	-0.0329	-0.3392	0.1262	0.2657	0.1461	0.2393	0.0283	0.1174	0.3007	-0.0971
721	S-304_Q9P2	Q9P289	Serine/threonine	STK26 MASK	NA	NA	-3.7462	-3.7295	-4.0357	-3.9147	-3.4016	-3.0965	-4.0862	-3.6677
722	S-305_Q9BV	Q9BW71	HIRA-interacting	HIRIP3	-1.1128	-1.0808	-1.7210	-1.1051	-1.2562	-1.5909	-1.2722	-1.2790	-1.1784	-1.0944
723	S-306_P146:	P14625	Endoplasmin (94	HSP90B1 GR	-0.8329	-0.4619	-0.3264	-0.8244	-0.8064	-0.5146	-0.6603	-0.7304	-0.0802	-0.0484
724	S-307_P519	P51946	Cyclin-H (MO15-a	CCNH	0.4406	0.4106	0.5171	0.3781	0.2673	0.2985	0.2886	0.2363	0.3505	0.1362
725	S-308_Q96N	Q96MU7	YTH domain-cont	YTHDC1 KIA/	0.0339	-0.0588	-0.0653	-0.4420	0.0832	-0.1670	-0.1854	-0.3571	0.0561	0.1019
726	S-309_Q9H1	Q9H171	Z-DNA-binding p	ZBP1 C20orf	-0.8921	-0.7256	-0.5580	-0.7358	-0.9663	-1.1871	-0.8346	-0.9054	-0.9154	-1.2087
727	S-31_P6232	P62328	Thymosin beta-4	TMSB4X TB4	-0.2479	0.1261	0.2388	0.1518	0.2032	0.2342	0.0833	0.0686	0.5828	0.6084
728	S-31_Q9252	Q92522	Histone H1.10 (H	H1-10 H1FX	2.1731	2.1864	1.9151	2.3073	2.3162	2.0647	2.4174	2.3608	1.2810	1.3608
729	S-312_Q9BV	Q9BWU0	Kanadaptin (Hun	SLC4A1AP H	0.7354	0.4501	0.5086	0.1009	0.5348	0.6595	0.5763	0.8490	0.8223	0.5399
730	S-312_Q9H8	Q9H8G2	Caspase activity a	CAAP1 C9or	0.3863	0.1944	-0.4496	-0.3016	0.1936	0.3034	0.4522	0.2734	0.3668	0.5454
731	S-313_Q3KC	Q3KQU3	MAP7 domain-cc	MAP7D1 KIA	-3.7576	-3.8882	-3.4397	-4.6192	-4.4845	-5.0639	-3.4311	-3.4397	-5.7227	-3.7907
732	S-313_Q997	Q99795	Cell surface A33 a	GPA33	-0.3548	-0.4711	-0.2226	0.0980	0.1871	0.1496	0.2355	0.0525	-0.4111	-0.3993
733	S-314_P678	P67809	Y-box-binding pr	YBX1 NSEP1	-2.0182	-2.2549	-1.8100	-1.7870	-1.9414	-1.8829	-2.0787	-2.0247	-1.9755	-2.6070
734	S-315_O002	O00273	DNA fragmentati	DFFA DFF1 D	0.5297	0.5578	0.8355	0.6606	0.5847	0.7550	0.3792	0.5489	0.9684	0.9562
735	S-315_P495	P49585	Choline-phospha	PCYT1A CTP	0.3522	0.1342	-0.3583	-0.1415	-0.0024	-0.3966	0.3406	0.0945	-0.6690	-0.5757
736	S-315_Q9NY	Q9NYF8	Bcl-2-associated	BCLAF1 BTF	-0.0448	0.0067	0.3746	0.1776	0.1548	0.1633	0.2554	0.3324	0.1610	0.1935
737	S-315_Q9NZ	Q9NZT2	Opioid growth fa	OGFR	-1.3276	-1.0302	-0.7625	-0.7599	-1.4467	-1.1885	-1.0912	-1.0925	-0.9598	-1.0513
738	S-316_Q132	Q13247	Serine/arginine-r	SRSF6 SFRS6	1.1703	1.3533	1.4830	2.0610	1.6330	1.5123	1.5455	1.8130	1.8507	1.9396
739	S-3177_Q6Z	Q6ZRS2	Helicase SRCAP (	SRCAP KIAA(	-1.6630	-1.8359	-1.7701	-1.6920	-1.7872	-1.7721	-1.9377	-1.6635	-1.8483	-1.1939
740	S-319_O950	O95049	Tight junction pr	TJP3 ZO3	-3.3183	-3.9553	-3.1900	-3.3782	-3.1708	-3.1804	-3.1290	-2.8382	-2.7142	-2.8359
741	S-32_P3565	P35659	Protein DEK	DEK	0.0054	0.3052	0.0981	-0.1109	-0.1564	-0.0484	0.0648	0.0932	-0.1681	-0.2788

742	S-32_Q1352	Q13523	Serine/threonine	PRPF4B KIAA	0.3169	0.1867	0.4550	0.8804	0.8104	0.4960	0.3852	0.6594	0.5574	0.7227
743	S-32_Q6ZVF	Q6ZVF9	G protein-regulat	GPRIN3 KIAA	0.2177	0.1291	-0.0307	0.2877	0.1757	0.5620	0.4398	0.4848	-0.6072	-0.5122
744	S-32_Q9H0W	Q9H0W8	Protein SMG9 (P	SMG9 C19or	0.0619	0.1118	-0.0708	-0.1980	-0.2065	-0.1356	-0.0610	-0.1701	0.0347	0.1514
745	S-320_Q130	Q13043	Serine/threonine	STK4 KRS2 N	NA	NA	-1.0658	-1.5052	-1.8339	-1.4956	-1.8842	-1.6282	-0.7960	-1.1884
746	S-320_Q5VT	Q5VTL8	Pre-mRNA-splicir	PRPF38B	-1.2959	-1.0715	-1.0063	-1.0674	-1.0139	-1.1471	-0.6278	-1.1178	-0.9451	-1.0168
747	S-320_Q9Y2	Q9Y2W1	Thyroid hormone	THRAP3 BCL	-0.0590	-0.1104	0.1500	0.0403	-0.2562	-0.1736	-0.2289	-0.0689	0.3413	0.2957
748	S-321_Q8IXS	Q8IXS8	Protein FAM126I	FAM126B	-2.1484	-1.8201	-1.3225	-1.5343	-1.8589	-1.6692	-1.1261	-1.3669	-1.9203	-1.9507
749	S-324_O759	O75909	Cyclin-K	CCNK CPR4	-0.5661	-0.3561	-0.4298	-0.2605	-0.4117	-0.6763	-0.4276	-0.3435	-0.2711	-0.1433
750	S-324_P369	P36915	Guanine nucleoti	GNL1 HSR1	-1.2871	-1.5597	-1.7107	-1.7932	-1.3470	-1.5775	-1.4738	-1.4648	-1.5876	-1.4198
751	S-324_Q86V	Q86VZ1	P2Y purinocepto	P2RY8	-2.0182	-1.4347	-2.2534	-2.2627	-2.7683	-2.4624	-2.5327	-2.2388	-2.4930	-2.5760
752	S-325_P086	P08670	Vimentin	VIM	0.2786	0.0011	-0.5427	0.1438	-0.4564	-0.3615	-0.2335	-0.4501	-0.5263	-0.1778
753	S-325_Q140	Q14004	Cyclin-dependen	CDK13 CDC2	0.1509	0.3088	0.2467	0.3863	0.2254	0.4012	0.2832	0.2595	0.4124	0.6768
754	S-325_Q5T2	Q5T200	Zinc finger CCCH	ZC3H13 KIAA	-2.0553	-2.1827	-1.9648	-2.2206	-2.1060	-2.0639	-2.4523	-1.9343	-2.1550	-1.6794
755	S-327_O145	O14545	TRAF-type zinc fi	TRAFD1 FLN	0.8823	0.5735	-0.2497	0.3616	-0.0288	-0.3219	0.5226	-0.0577	-0.4784	-0.3297
756	S-328_O438	O43823	A-kinase anchor	AKAP8 AKAF	-1.1605	-1.1979	-0.9166	-0.3598	-1.2562	-1.6834	-1.3007	-1.7431	-1.5876	-1.2058
757	S-328_P322	P32249	G-protein couple	GPR183 EBI2	1.6738	1.5923	1.7128	1.6929	1.3073	1.7093	1.7216	1.8204	0.9980	0.9049
758	S-328_Q3B7	Q3B726	DNA-directed RN	POLR1F TWI	1.4942	1.6716	1.4634	1.5861	1.2635	1.6246	1.5199	1.5462	1.6340	1.4869
759	S-328_Q9Y6	Q9Y608	Leucine-rich repe	LRRFIP2	0.0877	0.2289	-0.2068	-0.1518	-0.3898	-0.3444	-0.3948	-0.1278	-0.1814	-0.0889
760	S-329_P515	P51531	Probable global t	SMARCA2 BA	-0.4357	-0.6385	-1.1616	-0.8466	-0.8995	-1.2816	-1.1461	-0.9758	-0.8044	-0.9114
761	S-329_Q154	Q15459	Splicing factor 3A	SF3A1 SAP1	0.0550	0.2854	-0.1616	-0.4204	-0.3802	-0.3164	-0.0039	-0.1667	-0.2022	-0.2058
762	S-329_Q6UU	Q6UUV7	CREB-regulated t	CRTC3 TORC	-0.4211	-0.3993	-0.2991	-0.5369	-0.5831	-0.2284	-0.6258	-0.3300	-0.1038	-0.2479
763	S-329_Q86X	Q86X27	Ras-specific guar	RALGPS2	0.1863	0.4484	0.4733	0.0745	0.6198	0.7020	0.4906	0.6261	0.7155	0.9022
764	S-33_P0049	P00491	Purine nucleosid	PNP NP	0.7734	0.8141	-0.0719	0.3533	-0.0515	0.8911	0.3921	0.1369	-0.0913	-0.3790
765	S-33_Q9252	Q92522	Histone H1.10 (H	H1-10 H1FX	-0.7154	-0.8438	-0.5735	-0.2268	0.0246	-0.4200	-0.2628	-0.3908	-0.8766	-0.6228
766	S-33_Q9Y3Z	Q9Y3Z3	Deoxynucleoside	SAMHD1 MO	0.4392	0.4900	0.3571	0.3699	0.0849	0.0810	0.1291	0.0407	-0.0052	-0.0645
767	S-330_P404	P40425	Pre-B-cell leuken	PBX2 G17	-1.2696	-1.2496	-1.9854	-1.5603	-1.3706	-1.6930	-1.1899	-1.5972	-1.8389	-1.6508
768	S-330_Q96S	Q96S94	Cyclin-L2 (Paneth	CCNL2 SB13	0.1113	0.1087	-0.0653	-0.1818	-0.4923	-0.2353	-0.4810	-0.3868	-0.2633	-0.2788
769	S-330_Q9BS	Q9BSI4	TERF1-interacting	TINF2 TIN2	-0.7607	-1.0715	-0.5396	-0.6301	-0.4539	-0.6978	-0.8002	-0.8883	-0.5414	-0.8685
770	S-331_P495	P49585	Choline-phospha	PCYT1A CTP	-2.0109	-2.0167	-1.7314	-1.3461	-1.4794	-2.1209	-1.7645	-1.8770	-1.8250	-1.8314
771	S-331_Q9UI	Q9UI08	Ena/VASP-like pr	EVL RNB6	-0.1186	-0.2470	1.0423	0.5238	0.8771	0.4790	0.8072	0.6022	0.6704	0.4259
772	S-331_Q9Y5	Q9Y5Y4	Prostaglandin D2	PTGDR2 CRT	-1.4703	-1.7440	-1.2609	-0.8338	-2.4439	-1.7926	-1.8415	-1.5972	-1.7575	-1.8591
773	S-332_P499	P49915	GMP synthase [g	GMPS	0.7370	0.7043	0.6938	0.5962	0.7249	0.8677	0.7726	0.8178	1.1435	0.8496
774	S-332_Q66P	Q66PJ3	ADP-ribosylation	ARL6IP4	1.1751	1.2284	0.0715	0.1325	0.4196	-0.2530	0.5923	0.0846	-0.0449	0.2314
775	S-333_P322	P32249	G-protein couple	GPR183 EBI2	0.1296	0.0744	-0.2019	-0.0378	-0.3032	0.1603	-0.0408	0.0903	-0.1887	-0.2449
776	S-333_Q6JB	Q6JBY9	CapZ-interacting	RCSD1 CAPZ	0.3696	0.8956	-0.0930	0.4199	0.5920	0.2406	0.3449	0.4550	0.3091	0.3910
777	S-334_P497	P49790	Nuclear pore cor	NUP153	-0.5877	-0.7570	-0.5876	-0.4762	-0.5290	-0.5820	-0.6685	-0.4212	-0.4802	-0.6856
778	S-335_Q5VS	Q5VSL9	Striatin-interacti	STRIP1 FAM	-0.1108	0.0220	0.4006	0.2568	0.0780	0.2100	0.0308	0.0362	0.1388	0.3762
779	S-335_Q8ND	Q8NDI1	EH domain-bindi	EHBP1 KIAA	-0.7515	-0.9425	-0.7107	-0.7123	-0.8522	-0.7222	-0.6893	-0.5949	-0.7335	-0.6752
780	S-3360_Q09	Q09666	Neuroblast differ	AHNAK PM2	-0.8329	-1.0622	1.3693	0.3449	0.6246	-0.7900	1.1833	-0.1581	-0.4604	-0.5104

781	S-338_Q9HC	Q9HCN4	GPN-loop GTPase	GPN1 MBDII	-0.5715	-0.0370	-1.2165	-0.9459	-1.2129	-1.4542	-0.7383	-1.3828	-1.1579	-1.1793
782	S-339_O957	O95747	Serine/threonine	OXSR1 KIAA	0.5924	0.6169	0.7452	0.6661	0.8384	0.7638	0.6847	0.7578	0.7766	0.6567
783	S-339_P086	P08651	Nuclear factor 1	NFIC NFI	-0.6773	-0.7403	-0.2991	-0.3416	-0.3946	-0.4300	-0.4738	-0.2865	-0.1667	-0.2773
784	S-339_P176	P17612	cAMP-dependen	PRKACA PKA	-0.8233	-0.8538	-0.3949	-0.3507	-0.4845	-0.4360	-0.7470	-0.4212	-0.4912	-0.4232
785	S-339_Q8NC	Q8NCN4	E3 ubiquitin-prot	RNF169 KIA	1.4735	1.6304	1.7188	1.5070	1.2372	1.5013	1.3397	1.4864	1.5578	1.3959
786	S-34_P1933	P19338	Nucleolin (Protei	NCL	-1.7637	-1.5435	-0.2863	-1.0786	0.0674	-0.4101	-0.2863	0.2518	0.9551	0.8862
787	S-340_O759	O75909	Cyclin-K	CCNK CPR4	0.1558	0.0044	0.2432	0.5039	0.3777	0.1880	0.3990	0.3426	0.1459	-0.0026
788	S-340_P683	P68363	Tubulin alpha-1B	TUBA1B	-0.2140	-0.2483	-0.6953	-0.2289	-0.1441	-0.2780	-0.0664	-0.4130	-0.7553	-0.6149
789	S-340_Q5VV	Q5VVJ2	Histone H2A deu	MYSM1 KIA	0.9569	0.8752	0.7787	0.6311	0.5847	0.9118	0.6422	0.5931	0.6688	0.4712
790	S-340_Q997	Q99759	Mitogen-activate	MAP3K3 MA	-0.0972	-0.1752	-0.0544	0.0947	0.0639	-0.0195	-0.3087	-0.0061	-0.0091	-0.0312
791	S-341_P287	P28715	DNA repair prote	ERCC5 ERCM	1.1403	0.9430	1.5843	1.2076	1.5045	0.9851	1.3515	0.9174	1.7107	1.8280
792	S-3412_Q09	Q09666	Neuroblast differ	AHNAK PM2	-0.7066	-0.5132	-1.5125	-1.0233	-1.4590	-1.6551	-1.3363	-1.5834	-2.2633	-2.0273
793	S-343_P322	P32249	G-protein couple	GPR183 EBI	-3.0704	-2.2030	-2.7073	-2.8822	-2.7809	-3.1537	-2.4311	-2.8826	-2.9858	-2.9407
794	S-3438_O75	O75592	E3 ubiquitin-prot	MYCBP2 KIA	-1.8393	-1.1627	-0.4539	-0.5655	-0.5749	-0.2852	0.0882	-0.1145	-0.7774	-0.8452
795	S-344_P820	P82094	TATA element m	TMF1 ARA1	1.7628	1.5449	1.6736	1.4358	0.9418	1.4068	1.2333	1.2588	1.4853	1.0549
796	S-345_A2A2	A2A288	Probable ribonu	ZC3H12D C6	0.6783	0.6734	0.5982	0.6102	0.3419	0.4981	0.3438	0.4280	0.2091	0.2115
797	S-345_Q9UI	Q9UI08	Ena/VASP-like pr	EVL RNB6	-0.9089	-0.8359	1.5226	1.1430	1.3440	0.4225	1.4929	1.2101	0.8098	0.3812
798	S-347_P126	P12694	2-oxoisovalerate	BCKDHA	-0.9123	-0.7892	-1.7809	-0.9053	-0.9099	-1.3257	-0.7383	-1.0185	-1.4146	-1.4511
799	S-347_P507	P50750	Cyclin-dependen	CDK9 CDC2L	-0.9468	-1.0902	-1.4713	-1.4109	-1.1100	-0.9388	-0.7867	-0.9727	-1.3436	-1.5683
800	S-347_Q8TA	Q8TAQ2	SWI/SNF comple	SMARCC2 B	0.2536	0.2277	0.4041	-0.0001	0.3440	0.4259	0.2647	0.5476	0.7337	0.7752
801	S-347_Q9NS	Q9NSI8	SAM domain-cor	SAMSN1 HA	0.0072	-0.0347	-0.1008	-0.0805	-0.1001	-0.2744	-0.2895	-0.0817	-0.2633	-0.2297
802	S-348_Q926	Q92610	Zinc finger protei	ZNF592 KIA	0.7359	0.7674	0.8817	0.8717	0.9796	0.9631	1.1003	1.1195	0.7813	0.8800
803	S-349_O759	O75995	SAM and SH3 do	SASH3 CXor	-1.7699	-2.0950	-2.0664	-2.1637	-1.9485	-2.3983	-2.3829	-2.1079	-2.0871	-1.9808
804	S-349_Q134	Q13428	Treacle protein (	TCOF1	0.5692	0.5358	0.6841	0.9685	0.7860	0.8791	0.5966	0.8108	1.1429	1.1168
805	S-349_Q135	Q13523	Serine/threonine	PRPF4B KIA	-2.9537	-2.9130	-1.6869	-1.9425	-1.6890	-2.3596	-1.7168	-1.5607	-1.9007	-1.2633
806	S-349_Q6IB	Q6IBS0	Twinfilin-2 (A6-r	TWF2 PTK9L	-0.3829	-0.3087	0.5639	0.6471	0.6623	0.7506	0.5459	0.7880	0.8529	0.2488
807	S-349_Q9UI	Q9UI08	Ena/VASP-like pr	EVL RNB6	-0.7668	-0.6231	-0.3409	-0.0470	-0.3611	-0.4380	-0.2411	-0.1462	-0.6013	-0.6447
808	S-35_Q8TAD	Q8TAD8	Smad nuclear-int	SNIP1	1.3856	1.6185	0.6283	0.5760	0.9833	0.5417	0.8553	0.2684	0.3112	0.6864
809	S-350_Q146	Q14684	Ribosomal RNA p	RRP1B KIAA	-0.3093	-0.5435	-0.3908	-0.3259	-0.4464	-0.0937	-0.4189	-0.4232	-0.2997	-0.3120
810	S-351_P161	P16150	Leukosialin (GPL	SPN CD43	0.3434	0.1749	1.6403	1.1239	1.1990	0.9905	1.2951	1.5264	0.8703	-0.0436
811	S-351_Q96N	Q96NB3	Zinc finger protei	ZNF830 CCD	-0.2739	-0.2313	-0.5185	-0.3968	-0.5667	-0.8774	-0.7275	-0.6953	-0.5624	-0.2282
812	S-351_Q9UN	Q9UMN6	Histone-lysine N-	KMT2B HRX	0.3183	0.4581	0.5432	0.8203	0.4155	0.4401	0.4841	0.1018	0.4529	0.4740
813	S-352_P381	P38159	RNA-binding mot	RBMX HNRP	-0.0934	0.0849	0.2146	0.3931	0.2032	0.2913	0.2155	0.1968	0.1842	0.1467
814	S-352_Q9UK	Q9UK58	Cyclin-L1 (Cyclin-	CCNL1 BM-0	0.9777	1.0424	0.6158	0.7884	0.9364	0.7068	0.7757	0.5456	0.9443	0.9149
815	S-353_Q9UQ	Q9UQ35	Serine/arginine r	SRRM2 KIAA	-1.5181	-1.1903	-1.7551	-1.6585	-1.3847	-1.7871	-1.4074	-1.7774	-1.6372	-1.2742
816	S-354_Q96K	Q96K21	Abscission/NoCu	ZFYVE19 AN	-1.2885	-1.2216	-0.6898	-0.9866	-0.9463	-0.7594	-1.1965	-1.0240	-0.7559	-0.9450
817	S-354_Q9UI	Q9UI08	Ena/VASP-like pr	EVL RNB6	-2.1011	-2.0167	-2.5892	-2.0197	-1.9844	-1.9852	-1.4702	-2.0882	-2.1321	-2.1706
818	S-355_P161	P16150	Leukosialin (GPL	SPN CD43	1.1285	1.0140	0.5521	0.5035	0.7791	0.7866	0.9388	0.9757	0.4215	0.0383
819	S-358_P356	P35611	Alpha-adducin (E	ADD1 ADDA	1.5221	1.9212	0.2128	0.0524	0.3462	0.2355	0.6881	0.3522	0.1634	0.0506

820	S-359_O957	O95747	Serine/threonine	OXSR1 KIAA1	-1.0515	-0.8902	-0.5275	-0.5037	-0.5210	-0.5533	-0.4103	-0.2244	-0.6112	-0.4795
821	S-359_Q154	Q15459	Splicing factor 3A	SF3A1 SAP11	-0.6374	-0.3253	-1.5704	-1.3416	-1.3850	-1.2424	-1.0328	-0.8410	-0.9858	-1.3362
822	S-359_Q9NC	Q9NQ55	Suppressor of SW	PPAN BXDC3	-0.1004	-0.1905	-0.4091	-0.1698	-0.1673	-0.3637	-0.3857	-0.5492	-0.1984	-0.2284
823	S-36_Q9BQ6	Q9BQ65	U6 snRNA phosp	USB1 C16orf	-2.9537	-3.6752	-3.2991	-2.7447	-2.7936	-3.0270	-2.5252	-3.2102	-3.1962	-3.1082
824	S-36_Q9BTL	Q9BTL3	RNA guanine-N7	RAMAC C15o	0.2455	0.1159	0.0187	-0.0215	-0.0805	0.1198	-0.1693	0.0332	0.0372	0.0518
825	S-360_Q165	Q16566	Calcium/calmodu	CAMK4 CAM	-0.1289	-0.0852	0.2325	0.2039	0.3222	0.4779	0.0077	0.2512	0.7381	0.6652
826	S-360_Q7Z5	Q7Z5L9	Interferon regula	IRF2BP2	0.5082	0.7868	0.1771	0.3308	0.3621	0.1171	0.0648	0.1032	0.1622	0.0970
827	S-361_O760	O76021	Ribosomal L1 do	RSL1D1 CAT	0.7596	0.4646	1.2312	1.1725	1.4816	1.4461	1.2907	1.4051	0.9039	0.9694
828	S-361_Q6P6	Q6P6C2	RNA demethylase	ALKBH5 ABH	-1.2098	-1.3993	-1.4713	-1.2457	-1.3706	-1.4624	-1.1120	-1.3513	-1.5837	-1.6794
829	S-362_P423	P42331	Rho GTPase-activ	ARHGAP25 K	0.7818	0.7746	1.0208	0.7721	0.5385	0.8179	1.0052	1.0332	0.5845	0.6393
830	S-362_P495	P49585	Choline-phospha	PCYT1A CTP	0.5434	0.4492	0.3886	0.4292	0.1428	0.4812	0.3540	0.4605	0.4395	0.2379
831	S-362_Q9UH	Q9UHB6	LIM domain and	LIMA1 EPLIN	0.3598	0.0880	0.4132	0.7223	0.3081	0.3106	0.0796	0.3256	-0.0223	0.3196
832	S-363_O144	O14497	AT-rich interactiv	ARID1A BAF	-0.9757	-1.1000	-1.0046	-1.4240	-1.0596	-1.1091	-1.1512	-0.9054	-1.0738	-0.9661
833	S-363_Q006	Q00613	Heat shock facto	HSF1 HSTF1	-2.0628	-1.9005	-1.8472	-1.8244	-2.5914	-1.7620	-2.1008	-2.1615	-2.8389	-2.8826
834	S-364_Q9Y2	Q9Y2W2	WW domain-bind	WBP11 NPW	-1.4406	-1.5927	-1.6668	-1.6029	-1.5053	-2.0331	-1.9730	-1.8437	-1.1608	-1.2388
835	S-366_P054	P05455	Lupus La protein	SSB	1.5924	1.6632	1.3342	1.3445	1.2762	1.0950	1.1546	1.1522	1.3182	0.9545
836	S-366_Q8N5	Q8N554	Zinc finger protei	ZNF276 CEN	-0.2056	-0.7514	-0.0436	-0.2842	0.1022	0.1736	-0.2535	-0.0216	0.0296	0.0934
837	S-368_Q135	Q13523	Serine/threonine	PRPF4B KIAA	-2.5634	-1.2470	-2.1900	-1.9911	-2.2301	-2.1471	-2.2784	-2.0625	-2.1321	-2.1138
838	S-368_Q9NC	Q9NQZ2	Something about	UTP3 CRLZ1	-1.3502	-1.5663	-0.6668	-0.4133	-0.5263	-0.5468	-0.7601	-0.5811	-0.3669	-0.3723
839	S-369_Q96S	Q96S94	Cyclin-L2 (Paneth	CCNL2 SB13	1.6624	1.5216	0.8124	0.9539	0.8548	0.4608	0.7937	0.6643	0.4811	0.3989
840	S-37_P0640	P06400	Retinoblastoma-	RB1	0.0995	-0.0797	-0.2422	-0.2648	-0.3826	-0.3182	-0.1176	-0.2790	-1.0223	-0.6711
841	S-37_P1640	P16402	Histone H1.3 (His	H1-3 H1F3 H	0.4649	0.6404	0.5884	0.5422	1.4842	1.4308	0.9220	1.1053	-0.0118	0.1502
842	S-37_Q9NQ	Q9NQZ2	Something about	UTP3 CRLZ1	-1.0644	-1.0335	-1.2022	-1.1641	-1.0785	-0.7178	-1.0868	-1.1356	-0.9606	-0.7609
843	S-37_Q9Y4F	Q9Y4F9	Rho family-intera	RIPOR2 C6or	1.4624	1.2430	1.1960	0.8996	0.9205	1.4080	1.3065	1.3540	1.3232	0.8918
844	S-370_O751	O75122	CLIP-associating	CLASP2 KIAA	1.5265	1.3248	1.7923	1.4811	1.5181	1.5939	1.5285	1.4929	1.4647	1.1426
845	S-370_Q5F1	Q5F1R6	DnaJ homolog su	DNAJC21 DN	0.5729	0.2744	0.1348	-0.2691	0.3243	0.2506	0.2725	0.0451	0.2826	0.2999
846	S-372_Q9H2	Q9H2G2	STE20-like serine	SLK KIAA020	-0.2587	-0.4943	1.2520	1.4318	1.3118	1.2821	1.3692	1.3016	1.4581	1.6824
847	S-372_Q9NS	Q9NSI8	SAM domain-con	SAMSN1 HA	-0.3876	-0.5116	-0.4326	-0.3944	-0.8194	-0.7519	-0.7470	-0.6635	-0.9326	-0.9556
848	S-375_Q134	Q13428	Treacle protein (	TCOF1	-0.0590	0.2184	0.0635	0.4913	-0.1020	0.2506	0.1666	0.4716	0.0219	-0.2013
849	S-375_Q142	Q14289	Protein-tyrosine	PTK2B FAK2	-0.4653	-0.7477	-0.4888	-0.8659	-0.7620	-0.7951	-0.5252	-0.6042	-0.5778	-0.5437
850	S-377_P062	P06239	Tyrosine-protein	LCK	0.1719	0.1026	-0.8719	-0.4861	-0.6713	-0.5381	-0.1943	-0.1412	-0.8862	-0.6468
851	S-377_Q8IXT	Q8IXT5	RNA-binding pro	RBM12B	0.4929	0.5127	0.2101	0.4951	0.7935	-0.0270	0.8101	0.2772	0.1114	0.3061
852	S-377_Q96S	Q96ST2	Protein IWS1 hor	IWS1 IWS1L	-0.1524	-0.3350	0.4108	0.4226	0.2719	0.1429	1.0359	0.3700	0.1899	0.5586
853	S-378_Q9H4	Q9H4A3	Serine/threonine	WNK1 HSN2	-0.3758	-0.2641	0.0544	0.2863	-0.0981	-0.3730	-0.0815	-0.0915	-0.2885	-0.4921
854	S-378_Q9NZ	Q9NZT2	Opioid growth fa	OGFR	-0.0628	0.2194	-0.8415	-0.5787	-0.7159	-0.6978	-0.1275	-0.4501	-0.8090	-0.8638
855	S-379_P423	P42331	Rho GTPase-activ	ARHGAP25 K	-2.1011	-2.1928	1.3000	-0.1637	0.8528	-1.3866	0.5557	-0.6854	-0.3618	-1.3394
856	S-38_O4331	O43314	Inositol hexakisp	PPIP5K2 HIS	-1.3340	-0.9489	-0.9120	-1.0844	-1.0273	-1.4492	-0.8212	-1.4643	-1.4171	-1.0297
857	S-38_Q0182	Q01826	DNA-binding pro	SATB1	-0.0336	-0.1874	-0.0450	-0.0352	0.0730	0.1758	0.0351	0.0190	0.4448	0.3247
858	S-380_O603	O60381	HMG box-contai	HBP1	-1.4162	-1.1188	-0.9225	-0.6891	-0.8423	-0.6341	-0.9527	-1.2496	-1.1094	-0.8874

859	S-380_Q9C0	Q9C0B5	Palmitoyltransfei	ZDHHC5 KIA	-0.3595	-0.5322	-0.2007	-0.4062	-0.2366	-0.5019	-0.4541	-0.5273	-0.5778	-0.3541
860	S-380_Q9HE	Q9HB58	Sp110 nuclear bo	SP110	0.6571	0.6487	0.8093	0.4777	0.7355	0.6104	0.6827	0.6435	0.6524	0.3812
861	S-381;446_C	Q13428;Q1	Treacle protein ('	TCOF1	0.8294	0.8072	1.3299	1.2501	1.4162	0.9268	1.5561	1.1679	0.8142	0.9825
862	S-381_Q96L'	Q96LT9	RNA-binding regi	RNPC3 KIAA	-0.4042	-0.5116	-0.8340	-0.8822	-0.7777	-0.7494	-0.8322	-0.5185	-0.7078	-0.3362
863	S-382_Q9H6	Q9H6Y2	WD repeat-conta	WDR55	0.6112	0.1886	0.3340	0.4747	0.1855	0.4080	0.0460	0.3795	1.0735	1.0512
864	S-384_Q134	Q13422	DNA-binding pro	IKZF1 IK1 IK/	-0.5187	-0.5927	0.2001	-0.2755	-0.2150	0.1389	0.2574	-0.1112	0.0871	0.0850
865	S-384_Q156	Q15696	U2 small nuclear	ZRSR2 U2AF	0.2332	0.3649	0.1396	0.5950	1.0083	1.0052	0.4748	0.3818	-0.0968	-0.1605
866	S-384_Q6P6	Q6P6C2	RNA demethylas	ALKBH5 ABH	-0.0685	0.0307	-0.1733	0.2935	-0.4794	-0.4320	-0.5516	-0.5185	-0.5528	-0.6327
867	S-384_Q8IU	Q8IU81	Interferon regula	IRF2BP1	-0.5370	-1.5370	0.7239	0.5001	0.8831	0.8783	0.6977	0.7348	0.8687	1.1250
868	S-384_Q9BS	Q9BSQ5	Cerebral caverno	CCM2 C7orf	0.4929	0.5927	0.3950	0.3849	0.5296	0.6037	0.4869	0.4661	0.3858	0.4703
869	S-385_Q8N5	Q8N5C8	TGF-beta-activat	TAB3 MAP3I	-0.8954	-0.6129	-0.2522	-0.3944	-0.3946	-0.2388	-0.5196	-0.4480	-0.1306	-0.0194
870	S-385_Q8NC	Q8NC44	Reticulophagy re	RETREG2 C2	-0.5959	-0.9214	-0.3172	-0.5867	-0.7342	-0.6504	-0.9704	-0.8166	-0.6972	-0.4724
871	S-385_Q9C0	Q9C0B0	RING finger prot	UNK KIAA17	-0.1932	-0.1739	0.0745	-0.1737	-0.0195	0.1019	-0.0435	-0.1909	0.1065	0.1374
872	S-385_Q9NY	Q9NYF8	Bcl-2-associated	BCLAF1 BTF	0.9643	1.5468	0.0575	0.1260	-0.1421	-0.0937	0.1861	-0.0593	0.0834	0.1444
873	S-385_Q9UE	Q9UEW8	STE20/SPS1-relat	STK39 SPAK	0.9513	0.7881	0.9380	0.7191	0.7115	0.6622	0.7787	0.9746	0.4932	-0.1138
874	S-386_Q018	Q01813	ATP-dependent 6	PFKP PFKF	0.1127	0.0159	-0.1063	0.2019	-0.0888	-0.1096	0.1333	0.0439	0.1323	0.0730
875	S-387_Q8N1	Q8N1F8	Serine/threonine	STK11IP KIA/	0.3696	0.2872	0.1510	-0.1420	0.3791	0.2317	0.4273	0.2128	0.3310	0.1880
876	S-387_Q996	Q99638	Cell cycle checkp	RAD9A	0.6312	0.7252	0.7372	0.5725	0.5039	0.6125	0.6530	0.6311	0.8289	0.6524
877	S-387_Q9Y6	Q9Y6K9	NF-kappa-B esse	IKBKG FIP3 N	0.3399	0.3794	-0.0479	0.5386	0.4361	0.1116	0.4841	0.0817	0.0000	0.2336
878	S-388_Q141	Q14141	Septin-6	SEPTIN6 KIA	0.0688	-0.1406	-0.1349	-0.3126	0.1577	-0.1520	-0.3846	0.0152	0.0499	-0.1648
879	S-388_Q9Y2	Q9Y2X7	ARF GTPase-activ	GIT1	0.8526	0.9104	0.8640	0.6184	0.7503	0.6669	0.6464	0.7889	0.7035	0.6185
880	S-389_Q8IYE	Q8IYB3	Serine/arginine r	SRRM1 SRM	-1.6607	-1.8170	-1.7554	-1.7739	-1.6711	-1.5385	-1.6612	-1.7327	-1.8046	-1.4708
881	S-39_B5ME1	B5ME19	Eukaryotic transl	EIF3CL	1.3053	1.2910	1.2591	1.0991	1.1085	1.2278	1.1077	1.1806	0.9347	0.4910
882	S-39_P0407!	P04075	Fructose-bisphos	ALDOA ALD/	0.6613	0.8273	0.9215	0.8101	0.8890	1.2292	1.0716	1.2696	1.4690	0.9721
883	S-39_P0997.	P09972	Fructose-bisphos	ALDOC ALDC	-0.0628	-0.3019	-0.1627	-0.4323	0.2128	0.1762	-0.0801	0.1861	0.4802	0.4805
884	S-39_Q9Y4H	Q9Y4H4	G-protein-signali	GPSM3 AGS	-2.7948	-2.5467	-2.3198	-2.3598	-2.2129	-2.2565	-2.0302	-2.1278	-2.3271	-2.5992
885	S-390_P025	P02545	Prelamin-A/C [Cl	LMNA LMN1	-0.6687	-0.5646	0.1238	-0.0555	-0.4072	-0.0743	-0.4416	-0.2545	-0.7521	-0.5678
886	S-392_P025	P02545	Prelamin-A/C [Cl	LMNA LMN1	0.3154	0.4765	1.2915	1.3747	0.9523	0.9993	1.3807	1.4578	0.2458	0.3248
887	S-392_Q995	Q99549	M-phase phosph	MPHOSPH8	-2.3183	-1.9172	-1.0907	-1.3416	-0.9308	-1.0090	-1.0489	-0.8382	-0.7400	-0.6168
888	S-393_Q135	Q13547	Histone deacetyl	HDAC1 RPD	0.0532	-0.0484	-0.4903	-0.5113	-0.3802	-0.2583	-0.1576	-0.2352	-0.1221	0.0825
889	S-393_Q8IYE	Q8IYB3	Serine/arginine r	SRRM1 SRM	0.8911	1.0339	0.0290	-0.0824	0.1626	-0.2601	0.4235	0.0197	-0.2981	0.2799
890	S-394_Q86V	Q86V48	Leucine zipper pı	LUZP1	0.4630	0.5472	1.0620	1.3379	1.2419	0.9692	1.2116	1.1376	0.4529	0.7071
891	S-394_Q927	Q92769	Histone deacetyl	HDAC2	-0.2849	-0.4213	-0.1792	-0.5189	-0.3564	0.0059	-0.3561	-0.0200	0.1634	0.2466
892	S-397_Q134	Q13427	Peptidyl-prolyl ci	PPIG	1.1098	1.2169	0.6517	0.8393	0.7909	0.7434	0.7907	0.7566	0.6965	0.5962
893	S-397_Q9NY	Q9NYF8	Bcl-2-associated	BCLAF1 BTF	0.6555	0.9021	-0.1627	0.0351	-0.1060	-0.2762	-0.1475	-0.1479	-0.2370	-0.0604
894	S-398_Q011	Q01167	Forkhead box pr	FOXK2 ILF IL	-0.0857	0.1769	-0.1816	-0.3237	-0.5477	-0.0969	-0.2659	0.0182	-0.2949	-0.1249
895	S-399_Q3KC	Q3KQU3	MAP7 domain-co	MAP7D1 KIA	-1.2098	-0.9404	-0.8247	-1.4492	-1.2344	-1.1017	-0.9654	-1.3988	-1.2478	-1.3624
896	S-399_Q7Z4	Q7Z4V5	Hepatoma-derive	HDGFL2 HD(	-0.8458	-0.9769	-1.1947	-1.2041	-1.3100	-1.3520	-1.2722	-1.4069	-1.4604	-1.5760
897	S-400_Q96S	Q96ST2	Protein IWS1 hoı	IWS1 IWS1L	0.1799	0.1906	0.3555	0.5856	0.1708	0.2840	0.0052	0.4131	0.4783	0.5681

898	S-402_Q134	Q13422	DNA-binding pro	IKZF1 IK1 IKA	-4.9398	-4.0622	-3.4511	-2.7932	-3.2739	-3.3369	-3.5941	-3.4397	-3.1493	-3.0220
899	S-403_Q5JSH	Q5JSH3	WD repeat-conta	WDR44	-0.4455	-0.4154	-0.6486	-0.3921	-0.9203	-0.5338	-0.7040	-0.7508	-0.5151	-0.5104
900	S-403_Q9UK	Q9UKG1	DCC-interacting p	APPL1 APPL	-0.6402	-0.4437	-0.6519	-0.8855	-0.9449	-0.4081	-0.8025	-0.9638	-0.5856	-0.4112
901	S-404_P2070	P20700	Lamin-B1	LMNB1 LMN	-1.8649	-1.5816	-0.6701	-0.7543	-0.9256	-0.6623	-0.8338	-0.7586	-0.8886	-0.8945
902	S-404_Q96A	Q96AD5	Patatin-like phos	PNPLA2 ATG	0.0962	-0.0867	0.0413	-0.1797	-0.0554	0.1019	-0.0884	0.0107	0.1796	0.0381
903	S-405_Q8WI	Q8WUI4	Histone deacetyl	HDAC7 HDA	-1.8329	-1.7403	-1.5125	-1.5138	-1.2695	-1.8884	-1.7733	-1.7952	-1.5954	-1.3925
904	S-405_Q9Y6	Q9Y6G9	Cytoplasmic dyn	DYNC1LI1 DI	-1.3782	-1.2261	-0.3502	-0.2248	-0.5504	-0.3331	0.0039	-0.4356	-0.3854	-0.3723
905	S-406_P2070	P20700	Lamin-B1	LMNB1 LMN	-0.6773	-0.6507	-0.1781	-0.0433	-0.1320	-0.0331	-0.0529	0.2167	-0.3957	-0.2773
906	S-406_P2358	P23588	Eukaryotic transl	EIF4B	-2.3364	-2.2727	-2.4173	-2.5548	-2.2744	-2.2066	-2.0474	-2.3234	-2.1359	-2.5535
907	S-406_Q135	Q13547	Histone deacetyl	HDAC1 RPD3	-0.2330	-0.3775	-0.0919	-0.1225	0.3462	0.3716	-0.0170	0.3207	0.3937	0.0394
908	S-406_Q6UV	Q6UWZ7	BRCA1-A comple	ABRAXAS1 A	-0.1747	-0.3253	-0.2888	-0.3259	-0.5370	-0.3463	-0.8048	-0.5011	-0.4303	-0.4163
909	S-406_Q9UE	Q9UBW5	Bridging integrat	BIN2 BRAP1	1.1063	0.9933	1.8118	1.3329	2.0797	1.0082	1.9099	1.5150	0.8976	0.9008
910	S-407_Q9UE	Q9UBB5	Methyl-CpG-binc	MBD2	-0.1308	-0.3615	-0.2852	-0.6643	-0.4784	-0.4387	-0.5100	-0.4022	-0.3415	-0.2869
911	S-408_Q9Y2	Q9Y2W1	Thyroid hormone	THRAP3 BCL	0.7657	1.0357	0.9359	1.1650	0.9546	0.8509	1.3245	1.0447	0.9264	0.9769
912	S-409_O435	O43516	WAS/WASL-inter	WIPF1 WASI	-2.9260	-3.1188	-0.9958	-1.0525	-1.5158	-1.3944	-0.9628	-0.9140	-1.8067	-1.7997
913	S-41_Q96EV	Q96EV2	RNA-binding pro	RBM33 PRR8	-1.6346	-2.2869	-1.7737	-2.0125	-2.2651	-1.9106	-1.9935	-2.3435	-1.8250	-1.8314
914	S-410_Q130	Q13043	Serine/threonine	STK4 KRS2 M	0.1671	0.4056	0.0290	0.1866	0.2795	0.3554	0.4176	0.5363	-0.1493	-0.3394
915	S-410_Q9UK	Q9UKV3	Apoptotic chrom	ACIN1 ACINU	0.5594	0.6117	0.7780	0.8273	0.4790	0.4629	0.4456	0.3256	0.8319	0.7665
916	S-410_Q9Y2	Q9Y2X7	ARF GTPase-activ	GIT1	-1.0515	-1.1953	-1.1360	-1.4252	-1.1060	-0.8584	-1.0144	-1.0278	-1.1608	-1.0618
917	S-411_Q8IY1	Q8IY17	Patatin-like phos	PNPLA6 NTE	-0.8556	-0.9967	-0.7176	-0.7447	-0.9133	-0.8829	-1.0870	-0.9401	-1.2142	-1.2819
918	S-412_Q9UC	Q9UQR1	Zinc finger protei	ZNF148 ZBP8	-0.4381	-0.5548	-0.5595	-0.4911	-1.0554	-0.6930	-0.7253	-0.6182	-0.7120	-0.6090
919	S-413_Q9P2	Q9P270	SLAIN motif-cont	SLAIN2 KIAA	-1.0716	-1.0205	-1.2608	-1.2140	-0.9481	-1.3598	-1.4197	-1.3462	-1.4333	-1.4576
920	S-414_Q8IYE	Q8IYB3	Serine/arginine r	SRRM1 SRM	-1.3502	-1.0599	-1.1269	-1.0488	-0.8556	-1.0890	-1.0815	-0.8686	-1.4111	-0.9334
921	S-415_O145	O14545	TRAF-type zinc fi	TRAFD1 FLN	1.3008	1.1857	0.8851	0.9205	0.5309	0.5620	0.8373	0.7827	0.6704	0.5348
922	S-415_Q134	Q13427	Peptidyl-prolyl ci	PPIG	-1.5959	-1.3476	0.8640	1.0421	0.6150	0.8061	0.9883	1.2350	0.5975	0.6622
923	S-415_Q9Y5	Q9Y5T5	Ubiquitin carbox	USP16 MSTF	0.2636	0.1760	0.4038	0.2854	0.2631	0.1892	0.1844	0.0499	0.2893	0.2004
924	S-417_P297	P29728	2'-5'-oligoadenyl	OAS2	-0.1890	-0.1333	-0.2863	-0.2670	-0.5721	-0.2371	-0.2033	-0.1598	-0.2617	-0.3313
925	S-417_Q6DN	Q6DN90	IQ motif and SEC	IQSEC1 ARFC	0.9597	1.2026	1.0580	1.4779	1.1158	1.1164	1.2228	1.3066	0.5758	0.7793
926	S-419_P1638	P16383	Intron Large com	GCFC2 C2orf	-0.4187	-0.6894	-0.5381	-0.6329	-0.7809	-0.5490	-0.5746	-0.6732	-0.4533	-0.2449
927	S-419_Q134	Q13428	Treacle protein (	TCOF1	1.0658	1.1076	0.8848	0.9245	0.8954	0.3165	0.7255	0.5939	0.8968	0.9205
928	S-42_O7582	O75821	Eukaryotic transl	EIF3G EIF3S4	-0.5058	-0.6454	-0.5065	-0.3898	-0.5184	-0.3924	-0.4068	-0.4376	-0.4320	-0.4867
929	S-42_Q8NHZ	Q8NHZ8	Anaphase-promo	CDC26 ANAF	0.5533	0.2689	0.3522	0.3280	0.3390	0.4012	0.2509	0.1563	0.3495	-0.0155
930	S-42_Q9Y3P	Q9Y3P9	Rab GTPase-activ	RABGAP1 HS	0.9652	0.7932	1.4065	1.2672	1.2543	1.0258	1.1591	0.8764	1.2578	1.1467
931	S-42_Q9Y5S	Q9Y5S9	RNA-binding pro	RBM8A RBM	0.2846	0.2031	0.1790	0.3767	0.6341	0.3106	0.6670	0.6172	0.3279	0.3319
932	S-421_Q135	Q13547	Histone deacetyl	HDAC1 RPD3	0.1879	0.0372	0.8777	0.7918	0.7678	0.5640	0.4273	0.5941	1.1418	1.0019
933	S-422_Q927	Q92769	Histone deacetyl	HDAC2	-0.6884	-0.3941	-0.3972	-0.2641	-0.3110	-0.7118	-0.5259	-0.5504	-0.1885	-0.1032
934	S-422_Q96S	Q96ST2	Protein IWS1 hor	IWS1 IWS1L	0.3154	0.4581	0.6749	0.6137	0.6294	0.5478	0.3951	0.5342	0.9463	1.0077
935	S-423_P5519	P55198	Protein AF-17 (AI	MLLT6 AF17	-0.0926	-0.5463	-0.0669	-0.0196	0.0907	0.1608	-0.1635	0.1086	0.3304	-0.1152
936	S-423_Q135	Q13547	Histone deacetyl	HDAC1 RPD3	0.2208	0.8047	0.1491	0.2921	0.3791	0.0338	0.0672	0.0031	0.3413	0.3693

937	S-424_Q032	Q03252	Lamin-B2	LMNB2 LMN	-0.8988	-0.5419	-0.0730	-0.1302	-0.1139	-0.0331	-0.3594	-0.0466	-0.2586	-0.3248
938	S-425_Q7Z3	Q7Z3K3	Pogo transposab	POGZ KIAA0	1.6527	1.7395	1.7296	1.8442	1.7294	1.8726	1.6719	1.7733	1.8920	1.7707
939	S-426_P479	P47974	mRNA decay acti	ZFP36L2 ERF	-2.7948	-2.5597	-1.6405	-1.2627	-1.7872	-1.4300	-1.8509	-1.6442	-1.6434	-1.8314
940	S-428_P061	P06127	T-cell surface gly	CD5 LEU1	-1.0628	-1.0461	0.3340	-0.1263	0.2286	-0.2441	0.1861	0.0831	0.1222	0.0000
941	S-428_Q148	Q14839	Chromodomain-l	CHD4	-1.8921	-1.9945	-2.0318	-2.7567	-2.8194	-2.6090	-2.1519	-2.9054	-2.9451	-2.2664
942	S-43_P0404	P04049	RAF proto-oncog	RAF1 RAF	0.0809	0.0713	0.2634	0.2731	0.4947	0.5138	0.5053	0.4505	0.0993	0.2204
943	S-431_O952	O95232	Luc7-like protein	LUC7L3 CRE	0.5801	0.7117	0.5031	0.8213	0.3273	0.5458	0.2233	0.7275	0.4066	0.2936
944	S-431_P356	P35611	Alpha-adducin (E	ADD1 ADDA	0.5838	0.7359	0.0239	0.1741	0.1790	-0.0149	0.1468	-0.1328	0.3028	0.6695
945	S-431_Q081	Q08170	Serine/arginine-r	SRSF4 SFRS4	-4.3923	-3.5021	-3.1430	-3.1400	-3.1875	-3.0639	-2.8230	-2.7534	-3.0490	-2.7555
946	S-433_P352	P35269	General transcrip	GTF2F1 RAP	1.3378	1.3106	1.6444	1.2426	1.1903	1.0096	1.3060	1.1665	1.1150	0.8149
947	S-434_Q055	Q05519	Serine/arginine-r	SRSF11 SFRS	1.0654	1.1612	0.6775	0.7501	0.8343	0.7655	0.8767	1.0008	0.7599	0.9760
948	S-436_A6NC	A6NC98	Coiled-coil doma	CCDC88B BR	-1.3410	-1.3618	-1.6275	-2.0488	-1.6480	-1.7569	-1.4452	-1.6490	-2.1435	-1.4476
949	S-437_P542	P54274	Telomeric repeat	TERF1 PIN2	0.2515	0.2634	0.1780	0.1129	-0.1938	0.1722	0.1850	0.0774	-0.0118	0.2607
950	S-438_O948	O94804	Serine/threonine	STK10 LOK	0.8110	0.7970	0.9704	0.8010	0.8240	0.9071	0.8781	1.0503	0.7422	0.7398
951	S-438_P086	P08670	Vimentin	VIM	-0.1788	-0.3775	-0.1087	-0.0692	-0.4819	-0.5210	-0.3445	-0.5317	-0.1335	-0.1292
952	S-439_O433	O43318	Mitogen-activate	MAP3K7 TAI	-0.5528	-0.4051	-0.2032	-0.2584	-0.4489	-0.3406	-0.4086	-0.5317	-0.4233	-0.3707
953	S-439_P061	P06127	T-cell surface gly	CD5 LEU1	-2.8993	-2.9251	-3.2039	-2.9477	-2.8463	-3.1647	-2.5685	-2.7868	-3.1292	-3.5723
954	S-439_Q5JPI	Q5JPB2	Zinc finger protei	ZNF831 C20	1.9071	1.7788	1.5589	1.6802	1.5963	1.3831	1.5667	1.3946	1.6465	1.7098
955	S-4396_Q15	Q15149	Plectin (PCN) (PL	PLEC PLEC1	-0.4981	-0.6858	-0.5735	-0.6920	-0.7968	-0.8774	-0.5327	-0.6182	-0.7931	-0.7907
956	S-44_P3523	P35236	Tyrosine-protein	PTPN7	-0.0876	-0.2815	-0.0021	-0.1980	0.2333	0.2113	-0.0052	0.1453	0.1727	0.0642
957	S-44_Q9ULL	Q9ULL5	Proline-rich prot	PRR12 KIAA	-0.7394	-0.6646	-1.0664	-1.1518	-1.1750	-0.9793	-1.1869	-1.3128	-0.9203	-1.2914
958	S-441_P234	P23443	Ribosomal protei	RPS6KB1 STI	-1.5161	-1.9989	-1.5215	-2.0937	-1.7434	-1.6787	-1.1840	-1.4069	-1.8343	-2.3362
959	S-442_Q134	Q13422	DNA-binding pro	IKZF1 IK1 IK	-1.3093	-1.5927	-0.9547	-0.7240	-1.1139	-0.7075	-0.9477	-0.7203	-1.2917	-1.1793
960	S-444_Q089	Q08945	FACT complex su	SSRP1 FACT	-0.4829	-0.5712	-0.5673	-0.8724	-0.9203	-0.5842	-0.7601	-0.6928	-0.5096	-0.4706
961	S-445_P850	P85037	Forkhead box pr	FOXK1 MNF	0.8416	0.9522	0.8331	0.8091	0.5981	0.8791	0.9843	0.6662	1.0296	1.2591
962	S-445_Q134	Q13422	DNA-binding pro	IKZF1 IK1 IK	-0.3507	-0.5546	-0.0322	0.2853	-0.1760	-0.0778	0.0799	0.0949	-0.2294	-0.2323
963	S-445_Q147	Q14764	Major vault prot	MVP LRP	0.1345	0.1200	-0.0382	0.2206	-0.4215	0.1402	0.3367	0.4336	0.1126	-0.2102
964	S-446_Q5SS	Q5SSJ5	Heterochromatir	HP1BP3	-0.4854	-0.7164	-0.7227	-0.7210	-0.2762	-0.3673	-0.4523	-0.5361	-0.6700	-0.9042
965	S-446_Q9BZ	Q9BZ67	FERM domain-co	FRMD8 FKSG	-1.1686	-1.5597	-0.1804	-0.8212	-0.5886	-0.7900	-0.2643	-0.6879	-0.9602	-1.1590
966	S-448_Q8IZI	Q8IZD4	mRNA-decapping	DCP1B	-0.6459	-0.7835	-0.5939	-0.4812	-0.7039	-0.4748	-0.6158	-0.5163	-0.7864	-0.6752
967	S-448_Q9H0	Q9H0D6	5'-3' exoribonucl	XRN2	-1.7154	-1.4589	-2.8509	-2.3060	-2.5914	-2.8133	-2.1693	-2.4069	-2.5226	-3.0325
968	S-449_Q055	Q05519	Serine/arginine-r	SRSF11 SFRS	0.6637	0.7602	1.0343	1.4999	1.0520	0.6254	1.3411	1.2492	1.0878	1.0565
969	S-450_Q8IYE	Q8IYB3	Serine/arginine r	SRRM1 SRM	1.1958	1.3773	0.4875	0.7721	0.8312	0.6366	0.6547	0.5727	0.5644	0.8236
970	S-452_Q8IYE	Q8IYB3	Serine/arginine r	SRRM1 SRM	0.9314	0.6559	0.8881	0.7747	1.1113	0.8784	0.8790	0.6903	0.7746	0.7785
971	S-453_Q86V	Q86VP3	Phosphofurin aci	PACS2 KIAA	0.7063	0.1077	0.6912	0.8263	1.0364	0.9470	1.0851	0.8419	0.6365	0.7031
972	S-453_Q8IU	Q8IU81	Interferon regula	IRF2BP1	-1.9820	-1.9682	-1.9048	-1.8498	-1.8723	-2.1081	-1.9527	-2.2977	-2.1667	-1.9211
973	S-453_Q9H7	Q9H7N4	Splicing factor, a	SCAF1 SFRS1	-2.0704	-1.6930	-2.0885	-2.4737	-1.8995	-2.1274	-1.5824	-1.7380	-1.9501	-1.6835
974	S-454_A2RU	A2RU30	Protein TESPA1 (	TESPA1 KIAA	-0.5450	-0.6007	-0.2133	-0.5631	0.2545	-0.0990	-0.4268	-0.4159	-0.0134	0.0223
975	S-455_O608	O60832	H/ACA ribonucle	DKC1 NOLA	-0.9191	-0.8478	-0.5065	-1.1757	-0.4117	-0.5621	-0.4993	-0.6635	-0.2202	-0.1793

976	S-456_Q055	Q05519	Serine/arginine-r	SRSF11 SFRS	1.5924	1.6047	0.4607	0.9456	0.9170	0.2099	1.1055	0.6184	0.4644	0.4733
977	S-456_Q081	Q08170	Serine/arginine-r	SRSF4 SFRS4	1.2689	1.2274	1.0834	1.1921	1.0884	0.9095	1.0697	1.1902	1.3546	1.2466
978	S-456_Q9UC	Q9UQ35	Serine/arginine r	SRRM2 KIAA	1.1767	1.5388	2.3114	2.5438	2.3199	2.2434	2.5572	2.4508	2.3385	2.5330
979	S-457_O151	O15117	FYN-binding prot	FYB1 FYB SL	-0.1767	0.1362	-0.2786	-0.5629	-0.5721	-0.2998	-0.4470	-0.5996	-0.3939	-0.4976
980	S-457_Q53E	Q53EL6	Programmed cell	PDCD4 H731	-0.4590	-0.8753	0.4419	-0.1444	0.3861	0.6170	0.6792	0.5064	0.1288	-0.4745
981	S-457_Q9BT	Q9BTC8	Metastasis-assoc	MTA3 KIAA1	-1.2915	-1.2681	-0.3881	-0.0730	-0.1340	0.0894	0.1480	-0.0231	0.0232	0.2852
982	S-458_Q081	Q08170	Serine/arginine-r	SRSF4 SFRS4	0.7074	0.6439	-0.0885	0.3435	0.3592	-0.2583	0.3128	0.1740	0.1005	0.1126
983	S-458_Q9UE	Q9UBW5	Bridging integrat	BIN2 BRAP1	0.9717	0.8167	0.5280	0.3194	0.2016	0.3739	0.1550	-0.0403	-0.4025	-0.4267
984	S-46_O9540	O95400	CD2 antigen cyto	CD2BP2 KIA	-0.2245	-0.1953	-1.2812	-1.0415	-1.0439	-1.2145	-0.8840	-0.6395	-0.9154	-0.7885
985	S-46_Q0244	Q02446	Transcription fac	SP4	0.0054	-0.4407	-0.1360	-0.0805	-0.4742	-0.1753	-0.3846	-0.2643	-0.0706	-0.1264
986	S-46_Q8WW	Q8WWP7	GTPase IMAP fan	GIMAP1 IMA	-0.4603	-0.4650	-0.3963	-0.4886	-0.1980	-0.1339	-0.5140	-0.5317	-0.3888	-0.6918
987	S-460_P511	P51178	1-phosphatidylin	PLCD1	-0.6658	-0.9835	-0.4932	-0.5472	-0.7652	-0.2708	-0.6258	-0.5585	-0.9178	-1.0090
988	S-460_Q081	Q08170	Serine/arginine-r	SRSF4 SFRS4	1.6183	1.7012	1.8199	1.8848	1.5944	1.5845	1.7239	1.7590	2.0605	2.0170
989	S-460_Q3KC	Q3KQU3	MAP7 domain-cc	MAP7D1 KIA	0.5946	0.7530	0.2352	0.1975	0.1903	0.4068	0.3852	0.2543	0.6630	0.4638
990	S-462_P552	P55201	Peregrin (Bromo	BRPF1 BR14	-0.8787	-0.9532	-0.4727	-0.5708	-0.5263	-0.8317	-0.7339	-0.8742	-0.6515	-0.4027
991	S-4626_Q15	Q15149	Plectin (PCN) (PL	PLEC PLEC1	-1.2739	-1.4258	-0.5215	-0.9528	-0.4043	-0.5820	-0.5289	-0.3748	-0.3602	-0.5104
992	S-463_O751	O75122	CLIP-associating	CLASP2 KIAA	-1.4653	-1.4169	-1.7773	-1.1400	-1.0176	-1.5731	-0.9082	-1.4232	-1.8959	-1.0591
993	S-463_P304	P30414	NK-tumor recogr	NKTR	-1.0780	-1.0785	-0.4815	-0.5708	-0.9064	-0.7544	-0.9180	-1.2280	-0.4111	-0.3008
994	S-464_Q5VL	Q5VUA4	Zinc finger prote	ZNF318 HRIH	-0.9928	-0.9967	-0.6210	-0.8370	-0.9133	-0.6067	-1.2350	-0.8968	-0.3991	-0.6732
995	S-464_Q86X	Q86X29	Lipolysis-stimulat	LSR LISCH	0.4272	0.2251	0.3415	0.1929	0.1936	0.4202	0.1735	0.4348	0.5627	0.5586
996	S-466_Q96E	Q96EP0	E3 ubiquitin-prot	RNF31 ZIBRA	0.5862	0.5742	0.8783	0.7532	1.1992	1.2882	1.0019	1.1665	0.8615	0.9780
997	S-466_Q9BV	Q9BWU0	Kanadaptin (Hun	SLC4A1AP H	-1.3099	-1.6509	-1.6243	-1.4131	-1.6040	-1.4382	-1.5899	-1.4236	-1.2589	-1.1299
998	S-466_Q9BX	Q9BXL7	Caspase recruitm	CARD11 CAF	-0.8787	-0.9425	-1.1828	-1.2755	-1.5053	-1.5864	-1.4919	-1.4606	-1.2540	-1.1221
999	S-467_Q96S	Q96S59	Ran-binding prot	RANBP9 RAN	1.4709	1.5049	1.4097	1.6155	1.4697	1.4543	1.5871	1.5209	1.2622	1.2165
1000	S-47_A6NK5	A6NK59	Ankyrin repeat a	ASB14	0.1428	0.0479	0.3382	0.4186	0.3462	0.5468	0.3682	0.4472	0.3505	0.5515
1001	S-47_Q96EB	Q96EB6	NAD-dependent	SIRT1 SIR2L1	-0.8201	-0.6112	-1.2509	-1.4252	-1.1421	-1.3482	-1.3897	-1.2939	-1.3174	-1.1447
1002	S-470_Q127	Q12778	Forkhead box pro	FOXO1 FKHF	-0.3365	-0.5483	-0.2165	-0.7065	-0.2584	-0.1537	-0.4259	-0.3128	0.3556	0.5124
1003	S-470_Q9UJ	Q9UJX6	Anaphase-promc	ANAPC2 APC	0.5421	0.6481	-0.0021	0.0627	-0.5804	-0.2406	-0.0475	-0.2155	-0.2917	-0.1576
1004	S-471_P432	P43243	Matrin-3	MATR3 KIAA	-1.0478	-0.6858	-1.2485	-0.8466	-0.3123	-0.8884	-0.3965	-0.6276	-1.6597	-0.9334
1005	S-473_Q132	Q13263	Transcription int	TRIM28 KAP	-0.7696	-0.9873	0.0272	-0.2855	-0.0411	-0.0932	-0.1063	-0.0046	0.0422	-0.0770
1006	S-4738_O14	O14686	Histone-lysine N-	KMT2D ALR	-1.8721	-1.8518	-2.0664	-1.7567	-1.6082	-2.2007	-2.1404	-2.2173	-1.9961	-1.6468
1007	S-474_Q130	Q13098	COP9 signalosom	GPS1 COPS1	-1.2158	-1.0779	-0.6073	-0.5715	-0.6922	-0.6621	-0.9288	-0.9024	-0.8145	-0.8125
1008	S-475_Q9BR	Q9BRR9	Rho GTPase-activ	ARHGAP9	0.3442	0.1915	0.0991	0.0745	-0.1381	0.1130	-0.0870	-0.0753	-0.0382	0.1350
1009	S-476_Q96JI	Q96JM3	Chromosome alig	CHAMP1 C1	0.7933	0.7022	0.1983	0.2538	0.3749	0.1496	0.6621	0.5880	0.3566	-0.1998
1010	S-477_Q141	Q14157	Ubiquitin-associa	UBAP2L KIA	-1.7948	-2.1431	-1.8434	-2.5446	-2.6596	-2.3219	-2.1233	-2.4480	-2.0329	-2.4867
1011	S-478_Q76N	Q76N32	Centrosomal pro	CEP68 KIAA	-0.7274	-0.7020	-1.1132	-0.9806	-1.1503	-0.7469	-0.9884	-0.7534	-0.6556	-0.8200
1012	S-48_A7E2V	A7E2V4	Zinc finger SWIM	ZSWIM8 KIA	-2.1167	-2.0484	-1.6735	-1.4204	-1.3706	-1.3983	-1.7601	-1.5517	-2.0276	-2.1590
1013	S-48_P6280	P62805	Histone H4	H4C1 H4/A H	-0.6068	-0.4036	-1.2165	-0.4109	-0.9099	-0.2512	-0.2690	-0.5630	-1.1873	-1.0511
1014	S-480_P178	P17844	Probable ATP-de	DDX5 G17P1	-1.5634	-1.6197	-0.9206	-1.2799	-1.4141	-1.3983	-0.8961	-1.1581	-1.4930	-1.5914

1015	S-480_Q8IV5	Q8IV53	DENN domain-co	DENND1C FA	-0.2501	-0.4959	-0.7648	-0.6523	-1.0942	-1.1603	-0.8093	-1.0721	-1.0598	-1.1590
1016	S-480_Q929	Q92945	Far upstream ele	KHSRP FUBP	0.5765	0.5548	0.6899	0.6368	0.5321	0.8389	0.5992	0.6891	0.9724	1.0580
1017	S-480_Q997	Q99767	Amyloid-beta A4	APBA2 MINT	0.1079	0.2336	-0.1166	0.0524	0.1192	-0.0060	0.1124	0.2246	0.5715	0.4497
1018	S-481_P5339	P53396	ATP-citrate synth	ACLY	1.2457	1.2631	1.0855	0.9422	0.5561	0.8250	0.9677	1.1756	1.0196	0.9482
1019	S-481_Q8IZL	Q8IZL8	Proline-, glutami	PELP1 HMX3	0.7510	0.7983	0.3481	0.3137	0.3806	0.5107	0.4040	0.3329	0.5033	0.6335
1020	S-482_Q156	Q15642	Cdc42-interacting	TRIP10 CIP4	-0.2415	-0.4896	0.1147	0.2701	-0.2215	0.2010	-0.1008	-0.1787	-0.0530	-0.0538
1021	S-483_P0612	P06127	T-cell surface gly	CD5 LEU1	0.9287	0.8464	-0.0410	0.0150	-0.0396	-0.0511	0.1310	0.2112	-0.4068	-0.4638
1022	S-483_P4256	P42568	Protein AF-9 (ALL	MLLT3 AF9 Y	-0.1305	-0.0313	-0.0468	-0.1597	-0.2322	0.0163	-0.1504	-0.0947	0.0785	0.0242
1023	S-483_Q055	Q05519	Serine/arginine-r	SRSF11 SFRS	-0.0200	-0.2081	-0.4713	-0.9322	-0.4742	-0.4727	-0.3948	-0.0882	-0.3871	-0.4441
1024	S-4849_O14	O14686	Histone-lysine N-	KMT2D ALR	-0.6773	-0.7384	-0.3840	-0.1341	-0.2044	-0.1290	-0.5364	-0.4294	-0.2758	-0.1490
1025	S-485_P0612	P06127	T-cell surface gly	CD5 LEU1	0.1831	0.2355	-0.0986	0.3051	0.1175	0.1483	0.1315	0.3841	-0.1066	-0.1193
1026	S-486_Q7KZ	Q7KZI7	Serine/threonine	MARK2 EMK	0.2455	0.3450	0.5787	0.7458	0.7338	0.4035	0.5697	0.8487	0.7714	0.8229
1027	S-488_Q9BC	Q9BQC3	2-(3-amino-3-car	DPH2 DPH2L	-0.8921	-1.0257	-0.8415	-0.7567	-0.8656	-0.7746	-0.7800	-0.8631	-1.1962	-0.9309
1028	S-49_P17026	P17026	Zinc finger protei	ZNF22 KOX1	0.3904	0.5304	0.7239	0.2053	0.9408	0.8205	0.8936	0.7191	0.3878	0.1891
1029	S-49_Q9UP8	Q9UP83	Conserved oligor	COG5 GOLTC	-1.5581	-1.1928	-0.3017	-0.5472	-0.2279	-0.2336	-0.2769	-0.2827	-0.3719	-0.1793
1030	S-49_Q9Y3T	Q9Y3T9	Nucleolar comple	NOC2L NIR	1.3030	1.2616	1.0849	1.1414	1.4783	1.4838	1.1770	1.2779	1.2442	1.0922
1031	S-490_Q9UH	Q9UHB6	LIM domain and	LIMA1 EPLIN	0.1847	-0.0011	0.8783	0.4734	0.4563	0.6047	0.4408	0.4427	0.3434	0.3093
1032	S-491_Q96P	Q96PN7	Transcriptional-r	TRERF1 BCA	-1.1605	-1.2762	-0.8547	-1.0053	-0.8522	-1.1504	-1.1928	-1.1112	-1.1992	-1.0405
1033	S-492_Q6UN	Q6UN15	Pre-mRNA 3'-enc	FIP1L1 FIP1	-0.1767	-0.1815	-0.6569	-0.5814	-0.7311	-0.6787	-0.9603	-0.8382	-0.7752	-0.6427
1034	S-494_O608	O60832	H/ACA ribonucle	DKC1 NOLA4	-0.5303	-0.7406	-0.3920	-0.5506	-0.4066	-0.3958	-0.6268	-0.5385	-0.5424	-0.2585
1035	S-495_Q9NF	Q9NRG9	Aladin (Adracalin	AAAS ADRAC	-0.4066	-0.1652	-1.3542	-1.2123	-0.8656	-1.4180	-0.8093	-1.2902	-1.1122	-1.1706
1036	S-495_Q9NF	Q9NRH2	SNF-related serir	SNRK KIAA0	-1.9892	-2.5338	-2.0533	-1.8181	-2.2919	-1.9162	-2.2535	-2.1615	-1.3837	-1.7043
1037	S-496_Q9NY	Q9NYF8	Bcl-2-associated	BCLAF1 BTF	0.2162	0.0818	0.3431	1.0264	0.7070	0.2876	0.8467	0.4880	0.3475	0.5480
1038	S-497_Q9C0	Q9C0K0	B-cell lymphoma	BCL11B CTIP	0.1767	0.2579	0.7565	0.5796	0.5785	0.4950	0.4369	0.5717	0.9341	0.8061
1039	S-498_O437	O43719	HIV Tat-specific f	HTATSF1	-0.6516	-0.1394	-0.4844	0.1309	-0.4117	-0.1520	-0.3948	-0.1547	-0.3452	-0.5177
1040	S-499_Q9H0	Q9H0D6	5'-3' exoribonucl	XRN2	-0.5097	-0.3850	-0.1679	0.0128	0.0434	-0.1551	-0.0594	-0.0218	-0.1201	0.1188
1041	S-5_P08670	P08670	Vimentin	VIM	-1.5475	-1.3336	-0.8173	-0.2457	-1.0746	-1.6090	-0.8579	-1.0154	-1.6515	-1.2571
1042	S-50_O9525	O95251	Histone acetyltra	KAT7 HBO1	-1.7948	-1.8964	-0.6275	-0.7358	-0.4264	-0.2336	-0.6000	-0.5562	-0.6112	-0.7169
1043	S-50_P1803	P18031	Tyrosine-protein	PTPN1 PTP1	-0.0459	-0.0145	-0.0021	-0.0918	-0.2540	-0.0090	-0.0732	-0.0561	-0.2853	-0.3362
1044	S-50_Q1326	Q13263	Transcription int	TRIM28 KAP	-1.4753	-1.5338	-1.0084	-1.4062	-1.1792	-0.8829	-1.1120	-0.9698	-0.5856	-0.4795
1045	S-50_Q1361	Q13610	Periodic tryptoph	PWP1	0.1729	-0.6752	-0.3758	-0.8056	-0.5612	-0.0529	-0.1463	-0.0660	-0.2108	-0.9284
1046	S-50_Q96AT	Q96AT1	Uncharacterized	KIAA1143	0.5508	0.3301	0.9137	0.7887	0.9418	1.1109	0.8131	0.8230	0.9571	0.8595
1047	S-50_Q9NW	Q9NWQ8	Phosphoprotein	PAG1 CBP PA	0.4538	0.5698	0.4535	0.0897	0.3946	0.9182	0.3601	0.6834	-0.0065	-0.0352
1048	S-501_P4267	P42679	Megakaryocyte-a	MATK CTK H	-0.4579	-0.4392	-0.1314	-0.3060	-0.4190	-0.2007	-0.2108	-0.0545	-1.0652	-0.9090
1049	S-501_Q132	Q13263	Transcription int	TRIM28 KAP	-1.4653	-1.2815	-0.5110	-0.5655	-0.5914	-0.3463	-0.9654	-0.3223	-0.3585	0.0801
1050	S-502_Q68E	Q68E01	Integrator compl	INTS3 C1orf	0.6601	0.7784	-0.3502	-0.3016	-0.0046	-0.1405	0.2110	-0.0325	-0.5022	-0.2192
1051	S-503_O759	O75925	E3 SUMO-protein	PIAS1 DDXBI	0.4406	0.3947	0.4482	0.1534	0.0918	0.3225	0.2970	0.1188	0.5198	0.4497
1052	S-503_Q9BY	Q9BY44	Eukaryotic transl	EIF2A CDA02	0.5120	0.4427	0.9747	0.8233	0.7316	0.9166	0.6637	0.6062	0.7729	0.6784
1053	S-504_P2358	P23588	Eukaryotic transl	EIF4B	-1.0972	-1.2815	-0.6421	-0.0269	0.0246	-0.5360	-0.0249	-0.1178	-0.9476	-0.6980

1054	S-506_Q3MI	Q3MII6	TBC1 domain fan	TBC1D25 OA	-0.0818	-0.3604	-0.2019	-0.3148	-0.3611	-0.4706	-0.1899	-0.5141	-0.5547	-0.6050
1055	S-506_Q9BY	Q9BY44	Eukaryotic transl	EIF2A CDA02	0.7250	0.6865	0.5002	0.4606	0.5155	0.4726	0.3479	0.5655	0.4462	0.4125
1056	S-507_Q96JI	Q96JM3	Chromosome ali	CHAMP1 C1	-1.3093	-1.3589	-1.0211	-0.9053	-0.8032	-1.3482	-0.9961	-0.7431	-1.3337	-1.2602
1057	S-5077_Q09	Q09666	Neuroblast differ	AHNAK PM2	1.1982	0.9994	1.3652	1.5013	1.1511	1.2481	1.4952	1.1480	0.3718	0.4028
1058	S-508_Q2VP	Q2VPK5	Cytoplasmic tRN	CTU2 C16orf	-1.0366	-1.2708	-0.6618	-0.7123	-1.1179	-0.4042	-1.1346	-1.0372	-0.6413	-0.6248
1059	S-51_P0867	P08670	Vimentin	VIM	0.1525	0.0964	-0.3502	0.1470	-0.4897	-0.2816	0.3692	0.0846	-0.8022	-1.3993
1060	S-51_Q1651	Q16514	Transcription init	TAF12 TAF1	0.5963	0.5059	0.7193	0.7325	0.2151	0.3759	0.5434	0.4625	0.6867	0.8148
1061	S-51_Q86YV	Q86YV0	RAS protein activ	RASAL3	-1.4520	-1.6002	0.0292	-0.6209	-0.1002	-0.7755	0.1485	-0.3737	-0.2548	-0.7480
1062	S-51_Q96SB	Q96SB4	SRSF protein kina	SRPK1	-0.0478	0.0723	-0.0566	-0.0071	-0.2129	0.0074	-0.1605	-0.1161	-0.2602	-0.2162
1063	S-51_Q9954	Q99549	M-phase phosph	MPHOSPH8	0.8027	0.6432	-0.2824	-0.1051	0.0727	-0.0764	-0.0091	-0.1079	-0.3652	-0.6149
1064	S-510_Q9BP	Q9BPZ7	Target of rapamy	MAPKAP1 M	1.2756	1.3186	1.2837	1.0712	1.3628	1.1516	1.2779	1.0860	1.0245	0.9193
1065	S-511_Q096	Q09666	Neuroblast differ	AHNAK PM2	0.2726	0.3632	0.3198	0.0796	0.0156	0.1033	0.0039	-0.0657	-0.8838	-0.9407
1066	S-512_Q135	Q13586	Stromal interacti	STIM1 GOK	0.7660	0.9782	0.0775	0.3463	-0.1523	-0.1001	0.2189	-0.0325	-0.3871	-0.3024
1067	S-512_Q9BX	Q9BXL7	Caspase recruitm	CARD11 CAF	0.9597	1.0290	1.0745	1.3265	1.1013	1.3927	1.3007	1.2595	1.2740	1.0636
1068	S-512_Q9NY	Q9NYF8	Bcl-2-associated	BCLAF1 BTF	1.4538	1.5649	1.5992	1.6752	1.3820	1.5815	1.3877	1.7242	2.0071	1.9851
1069	S-513_O608	O60832	H/ACA ribonucle	DKC1 NOLA	1.0817	0.7272	1.0499	1.0395	1.1610	0.9692	1.0161	0.7265	0.6971	0.6904
1070	S-514_O948	O94804	Serine/threonine	STK10 LOK	-0.9537	-0.8280	-0.8873	-0.7870	-0.9238	-0.6621	-0.7867	-0.5406	-1.1066	-0.9885
1071	S-515_Q6PJ	Q6PJT7	Zinc finger CCCH	ZC3H14	2.0171	1.9917	1.8536	1.7969	1.6341	1.6447	1.7941	1.7435	1.9115	1.7421
1072	S-515_Q96N	Q96MU7	YTH domain-cont	YTHDC1 KIA	-4.8329	-2.8638	-2.8063	-3.1878	-2.4742	-2.5555	-2.5863	-2.8382	-3.2822	-3.0645
1073	S-517_Q96R	Q96RU3	Formin-binding p	FNBP1 FBP1	-0.3035	-0.3803	-0.3145	-0.7924	-0.5285	-0.3896	-0.5107	-0.2800	-0.4016	-0.3866
1074	S-518_P295	P29590	Protein PML (Pro	PML MYL PP	-0.7707	-0.8372	-0.6923	-0.7244	-0.8416	-0.6760	-0.7723	-0.7802	-0.6856	-0.4724
1075	S-519_O005	O00567	Nucleolar protei	NOP56 NOL	0.7649	0.4064	0.1836	-0.4299	0.3462	0.5417	0.5181	0.4804	0.2922	0.2237
1076	S-52_Q8N9	Q8N9Q2	Protein SREK1IP1	SREK1IP1 P1	-1.3365	-1.1236	-0.4270	-0.8921	-0.3730	-1.1339	-0.9278	-0.9668	-0.0996	0.0481
1077	S-52_Q8TAD	Q8TAD8	Smad nuclear-int	SNIP1	-1.4804	-1.2081	-1.5923	-1.9087	-1.3802	-1.6044	-1.4523	-1.5789	-1.7357	-1.1533
1078	S-52_Q8TAP	Q8TAP8	Protein phospha	PPP1R35 C7	0.0215	-0.0692	-0.4511	-0.4861	-0.7620	-0.6112	-0.3495	-0.3415	-0.6272	-0.4406
1079	S-52_Q9Y2V	Q9Y2V2	Calcium-regulate	CARHSP1	0.7871	0.5898	0.5090	0.2313	0.6330	0.6993	0.7581	0.5182	0.4821	0.0343
1080	S-521_Q150	Q15057	Arf-GAP with coil	ACAP2 CENT	0.4942	0.2430	0.6112	0.4644	0.2810	0.6984	0.4673	0.5161	0.4415	0.7436
1081	S-521_Q6PK	Q6PKG0	La-related protei	LARP1 KIAA	-2.0628	-2.6681	-1.1246	-1.6692	-1.3423	-1.5040	-1.6338	-1.4946	-1.9451	-1.1969
1082	S-521_Q9C0	Q9C0E2	Exportin-4 (Exp4	XPO4 KIAA1	0.7348	0.5993	0.2494	0.5867	0.3635	0.5549	0.7216	0.6507	0.2794	0.3237
1083	S-522_O751	O75167	Phosphatase and	PHACTR2 C6	-0.6601	-0.4912	-0.4496	-0.0899	0.0462	0.0908	0.0372	-0.0387	-0.7531	-0.8993
1084	S-524_P332	P33240	Cleavage stimula	CSTF2	-0.7637	-0.7329	-1.0708	-1.1838	-1.1462	-0.4935	-0.9704	-0.6088	-0.4930	-0.4476
1085	S-525_Q9NF	Q9NPI6	mRNA-decapping	DCP1A SMIF	-0.7454	-0.6368	-1.0021	-1.1244	-1.0785	-1.2601	-1.2597	-1.6019	-0.9883	-0.7642
1086	S-526_Q7KY	Q7KYR7	Butyrophilin sub	BTN2A1 BT2	0.1477	0.3675	0.4830	0.6056	0.3735	0.6007	0.6662	0.6681	-0.1435	-0.3410
1087	S-527_P295	P29590	Protein PML (Pro	PML MYL PP	-0.4066	-0.4803	-0.7595	-0.3828	-0.5914	-0.7620	-0.6000	-0.6854	-0.6152	-0.2804
1088	S-528_Q9NF	Q9NPQ8	Synembryn-A (Pr	RIC8A	-1.3971	-1.4959	-1.1546	-1.4737	-1.3055	-1.0732	-1.3265	-0.9401	-1.3502	-1.0889
1089	S-529_Q5VT	Q5VTL8	Pre-mRNA-splicir	PRPF38B	-0.4480	-0.6282	0.3257	0.4670	0.7591	0.6650	0.8532	0.7061	0.1341	0.3861
1090	S-53_A7E2V	A7E2V4	Zinc finger SWIM	ZSWIM8 KIA	-2.6574	-2.5189	-2.7861	-3.1388	-2.7851	-3.0168	-3.0469	-2.7873	-3.0332	-3.0318
1091	S-53_O6086	O60869	Endothelial diffe	EDF1	NA	NA	-1.0818	-1.8244	-0.5290	-3.2636	-0.4311	-1.8547	-2.3403	-2.9910
1092	S-53_Q9BTA	Q9BTA9	WW domain-con	WAC KIAA1	0.1695	-0.2903	0.4217	0.3607	0.4907	0.6071	0.1810	0.5936	0.7305	0.5817

1093	S-530_Q6NY	Q6NYC8	Phostensin (Prot	PPP1R18 HK	0.4087	0.2735	0.8140	0.6313	0.4501	0.2593	0.8665	0.5525	0.3127	0.0880
1094	S-531_O433	O43353	Receptor-interac	RIPK2 CARDI	-1.3595	-1.7002	-1.1430	-1.0342	-1.1875	-1.5295	-1.1693	-1.3748	-1.6033	-1.3591
1095	S-531_Q9NY	Q9NYF8	Bcl-2-associated	BCLAF1 BTF	0.5297	0.4718	0.4853	0.3137	0.1887	0.1510	0.4157	0.2114	0.4115	0.4506
1096	S-534_Q8IW	Q8IWI9	MAX gene-associ	MGA KIAA05	-0.7916	-0.6966	-0.3238	-0.2123	-0.1219	-0.3145	-0.0104	-0.4232	-0.1221	-0.2710
1097	S-536_Q9UL	Q9ULM3	YEATS domain-cc	YEATS2 KIAA	-2.0182	-1.7552	-0.0307	-0.0599	0.1090	0.2406	0.6913	0.5236	0.3123	0.2358
1098	S-537_Q020	Q02040	A-kinase anchor	AKAP17A CX	0.2485	0.4089	0.5112	0.6796	0.5624	0.5076	0.5966	0.5214	0.3495	0.4487
1099	S-538_Q149	Q14978	Nucleolar and co	NOLC1 KIAA	1.2232	1.2251	0.4994	0.8233	0.9117	0.7958	0.9349	0.8453	0.5512	0.5507
1100	S-539_Q9H6	Q9H6Z4	Ran-binding prot	RANBP3	-0.1404	0.0000	-0.1269	-0.1617	-0.2150	0.0309	-0.1825	0.0015	-0.0748	0.0255
1101	S-539_Q9Y4	Q9Y4E1	WASH complex s	WASHC2C F/	-0.1651	-0.1181	0.0674	0.0128	-0.4445	-0.1896	-0.2279	-0.1140	0.0298	0.0024
1102	S-54_Q8TAD	Q8TAD8	Smad nuclear-int	SNIP1	-0.6402	-0.5516	-0.8247	-0.9806	-0.7069	-0.7197	-0.6768	-1.0372	-1.0196	-0.4493
1103	S-544_Q131	Q13144	Translation initia	EIF2B5 EIF2E	0.8745	0.5158	-0.7935	-0.6192	-0.4240	-0.9134	-0.0262	-0.4459	-0.7531	-0.8826
1104	S-544_Q9Y3	Q9Y3L3	SH3 domain-binc	SH3BP1	0.5627	0.4233	0.7844	0.5454	0.5879	0.6553	0.5523	0.7411	0.5888	0.4461
1105	S-5448_Q09	Q09666	Neuroblast differ	AHNAK PM2	-0.8074	-0.7778	-0.3582	-0.0071	-0.4691	-0.3463	-0.4630	-0.3532	-1.3337	-1.0039
1106	S-545_O602	O60216	Double-strand-bı	RAD21 HR21	-2.0432	-1.9185	-2.5271	-1.9190	-1.7005	-2.8171	-1.7867	-2.6779	-2.1353	-1.9144
1107	S-545_Q8W	Q8WYA6	Beta-catenin-like	CTNNBL1 C2	0.5862	1.0022	-0.3826	0.0351	-0.1482	0.2087	-0.1634	-0.0325	-0.2293	-0.2633
1108	S-546_Q134	Q13427	Peptidyl-prolyl ci	PPIG	0.5196	0.7352	0.3677	0.5263	0.8229	0.4445	0.4851	0.5520	0.3289	0.6532
1109	S-546_Q150	Q15022	Polycomb protei	SUZ12 CHET	0.2531	0.1592	0.3571	0.2221	0.0551	0.3449	0.3294	0.2285	0.3818	0.2215
1110	S-546_Q8TE	Q8TEH3	DENN domain-cc	DENND1A F/	-2.0553	-2.1777	-1.5642	-1.3644	-1.8723	-1.2816	-1.5252	-1.3948	-1.7270	-1.6427
1111	S-547_Q5JSI	Q5JSP0	FYVE, RhoGEF an	FGD3 ZFYVE	1.4272	1.3824	1.5808	1.6247	1.6716	1.7546	1.6837	1.6507	1.6214	1.2341
1112	S-547_Q8N2	Q8N2M8	CLK4-associating	CLASRP SFRS	1.3884	1.2989	1.2577	1.1936	1.1167	1.0251	1.1712	1.2272	1.2392	0.9576
1113	S-547_Q9H1	Q9H1B7	Probable E3 ubiq	IRF2BPL C14	0.4657	0.6678	0.9597	1.2221	0.7014	0.6222	0.4312	0.3438	0.7336	0.6760
1114	S-547_Q9NC	Q9NQC7	Ubiquitin carbox	CYLD CYLD1	-0.8655	-1.0507	-1.1019	-1.3104	-1.4539	-1.1636	-1.2535	-1.2716	-1.4893	-1.5307
1115	S-549_O953	O95365	Zinc finger and B	ZBTB7A FBI1	0.9513	1.0116	0.8337	0.7309	0.6957	0.7130	0.7915	0.6575	0.3698	0.4906
1116	S-549_Q9UI	Q9UHB7	AF4/FMR2 family	AFF4 AF5Q3	-3.1645	-2.4834	-2.4116	-2.2799	-2.1959	-2.4542	-2.2659	-2.1892	-2.2822	-2.4232
1117	S-55_Q96FS	Q96FS4	Signal-induced p	SIPA1 SPA1	0.1493	0.2763	0.3894	0.1598	0.4251	0.2215	0.3621	0.3970	0.1090	0.1490
1118	S-550_Q9Y3	Q9Y3L3	SH3 domain-binc	SH3BP1	0.4683	0.3862	0.2307	0.0455	0.2112	0.2164	0.3139	0.3818	0.2773	0.5088
1119	S-552_Q149	Q14966	Zinc finger protei	ZNF638 NP2	-0.1069	-0.3420	-0.0307	-0.2994	-0.2431	-0.2007	0.0709	-0.2698	-0.2022	-0.1735
1120	S-553_P3717	P37173	TGF-beta recepto	TGFBR2	-0.1128	-0.2081	0.1367	0.1309	0.2223	0.4046	0.3449	0.4415	0.7737	0.6024
1121	S-553_Q9NV	Q9NWH9	SAFB-like transcr	SLTM MET	1.1695	1.0723	0.5617	0.5126	0.9029	0.8685	0.9698	1.0686	0.7139	0.6401
1122	S-554_P278	P27824	Calnexin (IP90) (I	CANX	1.2606	1.7910	0.2191	0.4938	0.0780	-0.2076	0.6514	-0.0785	-0.5040	-0.4493
1123	S-554_Q150	Q15027	Arf-GAP with coil	ACAP1 CENT	0.7173	0.5502	1.1785	0.9326	0.8710	1.0668	1.1456	0.8946	0.7933	0.6647
1124	S-558_Q9Y2	Q9Y2K7	Lysine-specific de	KDM2A CXX	0.1096	0.1462	-0.0653	-0.0089	-0.1441	0.1762	-0.2753	-0.1345	-0.1579	0.0090
1125	S-56_O9575	O95758	Polypyrimidine tı	PTBP3 ROD1	-1.9537	-1.9682	-1.3436	-0.9528	-1.4240	-1.3035	-1.8048	-1.7027	-1.5954	-1.6070
1126	S-56_P1640	P16402	Histone H1.3 (His	H1-3 H1F3 H	-0.3414	-0.2529	-0.1910	-0.0887	0.0299	-0.0956	-0.1944	-0.0782	-0.1830	-0.0789
1127	S-56_Q5VZF	Q5VZF2	Muscleblind-like	MBNL2 MBL	-1.2015	-1.6299	-1.2140	-1.2842	-1.6308	-1.0240	-0.9987	-1.6538	-1.4111	-1.1706
1128	S-56_Q9Y5S	Q9Y5S9	RNA-binding pro	RBM8A RBM	-0.2805	-0.1640	-0.2875	-0.1128	-0.4717	0.0626	-0.2927	-0.0046	-0.0638	-0.0875
1129	S-560_O150	O15014	Zinc finger protei	ZNF609 KIA/	-1.1890	-1.1479	-0.9186	-0.7387	-0.6110	-1.0608	-0.5960	-0.5811	-0.4930	-0.3891
1130	S-560_Q149	Q14966	Zinc finger protei	ZNF638 NP2	-3.2827	-3.1878	-0.6356	-0.8954	-1.0902	-0.7544	-0.5364	-0.5926	-0.3786	-0.1954
1131	S-560_Q96F	Q96FV9	THO complex sul	THOC1 HPR1	-0.0478	0.3248	-0.1234	0.0644	-0.2695	-0.3711	-0.1519	-0.0915	-0.0422	0.2553

1132	S-560_Q9Y4	Q9Y4W2	Ribosomal bioge	LAS1L MSTP	-1.0515	-0.4498	-1.1269	-1.2248	-1.0251	-1.1177	-1.1290	-1.4818	-1.1992	-1.0378
1133	S-561_Q5JSI	Q5JSH3	WD repeat-conta	WDR44	0.8405	0.9418	-0.4713	-0.0975	-0.2023	-0.2907	-0.1707	0.0302	-0.3141	-0.4724
1134	S-562_Q8ND	Q8NDT2	Putative RNA-bir	RBM15B OT	-0.5135	-0.7797	0.3398	0.2627	0.4114	0.4158	0.0930	0.2696	0.1246	-0.0103
1135	S-563_P193	P19338	Nucleolin (Protei	NCL	1.3605	1.5742	0.9816	0.9465	0.7742	0.6781	0.7635	0.6261	0.6696	0.7015
1136	S-563_Q150	Q15059	Bromodomain-cc	BRD3 KIAA0	0.0108	-0.1566	-0.0997	-0.3304	-0.2453	-0.0331	-0.2659	-0.1753	0.0473	-0.1764
1137	S-563_Q5T5	Q5T5Y3	Calmodulin-regu	CAMSAP1	-0.5161	-0.6594	-0.4611	-0.6029	-0.5423	-0.4998	-0.3462	-0.4501	-0.5643	-0.6307
1138	S-564_P278	P27824	Calnexin (IP90) (I	CANX	1.1403	1.1442	-0.2647	0.4025	-0.1482	-0.4081	0.4484	-0.0466	-0.9080	-0.9018
1139	S-564_Q6PJI	Q6PJI9	GATOR complex	WDR59 KIAA	0.0304	0.2790	0.1185	0.0644	0.1952	0.4213	0.0882	0.0152	0.0735	0.0468
1140	S-564_Q9C0	Q9C0I1	Myotubularin-re	MTMR12 KIA	0.7173	0.7817	1.3934	1.0150	1.0771	0.7941	1.0906	0.8963	0.4261	0.2714
1141	S-564_Q9NF	Q9NRA8	Eukaryotic transl	EIF4ENIF1	-1.1726	-1.1382	-1.3569	-1.5189	-1.8064	-1.7173	-1.5516	-2.0372	-1.4391	-1.6267
1142	S-565_P295	P29590	Protein PML (Prc	PML MYL PP	-0.0934	-0.3032	-0.3596	-0.4228	-0.2851	-0.7645	-0.3846	-0.4733	-0.5096	-0.2602
1143	S-567_Q6VN	Q6VMQ6	Activating transc	ATF7IP MCA	-3.2652	-2.1431	-1.1757	-1.3735	-1.3284	-1.4624	-2.2848	-1.2388	-1.0844	-1.1306
1144	S-568_Q86U	Q86UE4	Protein LYRIC (3D	MTDH AEG1	-0.9573	-0.9026	-0.7737	-0.8724	-1.0942	-0.8477	-1.1751	-0.8940	-0.5022	-0.9114
1145	S-569_Q151	Q15111	Inactive phosphc	PLCL1	0.2424	0.4427	0.1946	0.3109	0.3228	0.5820	0.2939	0.2671	0.3165	0.2336
1146	S-569_Q926	Q92619	Rho GTPase-activ	ARHGAP45 I	-0.7761	-0.7237	-1.0349	-1.1315	-0.9531	-1.0796	-1.0447	-1.2051	-1.2219	-0.9689
1147	S-57_P2935(	P29350	Tyrosine-protein	PTPN6 HCP I	0.1612	-0.0064	-0.1264	0.1086	-0.1048	0.1105	0.1284	-0.1504	-0.0952	-0.1661
1148	S-57_Q8WU	Q8WUQ7	Cactin (Renal car	CACTIN C19	-4.8854	-4.2815	-3.4511	-4.3782	-4.7558	-4.6739	-3.2597	-3.5789	-3.2202	-2.6387
1149	S-572_Q166	Q16666	Gamma-interferc	IFI16 IFNGIP	0.0945	0.2079	-0.0774	0.1518	-0.4190	-0.1973	-0.7756	-0.7380	-0.2602	-0.4423
1150	S-573_O151	O15117	FYN-binding prot	FYB1 FYB SL	0.6077	0.5072	0.2590	0.3037	-0.0458	0.2961	0.2032	0.3570	0.5423	-0.0129
1151	S-573_Q147	Q14738	Serine/threonine	PPP2R5D	0.3793	0.2050	1.1877	0.9148	1.0814	1.1775	1.0942	1.0053	1.1071	0.9442
1152	S-575;588_P	P52272;P52	Heterogeneous r	HNRNPM HN	0.0000	-0.4184	0.1780	0.3209	0.4536	0.4001	0.2443	0.5415	0.1435	0.1339
1153	S-576_O145	O14526	F-BAR domain or	FCHO1 KIAA	-0.5634	-0.6948	-0.4626	0.0194	-0.2497	-0.2162	0.0833	-0.1013	-0.4111	-0.3281
1154	S-576_O150	O15014	Zinc finger protei	ZNF609 KIAA	0.0945	0.4900	0.4195	0.0863	0.5064	0.6809	0.2043	0.4370	0.7242	0.5454
1155	S-576_P260	P26038	Moesin (Membra	MSN	-0.2161	-0.4726	-0.4684	-0.8888	-0.4514	-0.0195	-0.3914	-0.1462	0.0078	-0.3024
1156	S-576_Q146	Q14694	Ubiquitin carbox	USP10 KIAA(	-0.3711	-0.6541	-0.7917	-0.6357	-0.8961	-0.8133	-0.8346	-0.7077	-0.8624	-1.3329
1157	S-5763_Q09	Q09666	Neuroblast differ	AHNAK PM2	1.6177	1.4924	1.7941	1.6561	1.5045	1.8238	1.5715	1.6628	1.6651	1.5151
1158	S-578_Q7LD	Q7LDG7	RAS guanyl-relea	RASGRP2 CD	-1.4114	-1.2896	-1.0930	-1.1205	-1.6138	-0.9589	-1.3965	-1.1753	-0.7954	-0.9707
1159	S-578_Q8N3	Q8N3F8	MICAL-like prote	MICALL1 KIA	0.5057	0.4378	0.5568	0.4567	0.7613	0.8595	0.4350	0.6853	0.7684	0.5802
1160	S-579_Q6UN	Q6UN15	Pre-mRNA 3'-enc	FIP1L1 FIP1 I	-1.2871	-1.2496	-0.7845	-0.6949	-1.1340	-0.5643	-1.0091	-0.7329	-0.6577	-0.8970
1161	S-58_P6017	P60174	Triosephosphate	TPI1 TPI	1.5343	1.3497	1.4771	1.3589	1.4003	1.4954	1.3913	1.5224	1.3241	1.3451
1162	S-58_Q9NU	Q9NUV9	GTPase IMAP fan	GIMAP4 IAN	1.2447	1.1886	1.6893	1.5294	1.5052	1.6207	1.6751	1.6891	1.7680	1.5392
1163	S-582_Q8IV	Q8IV53	DENN domain-cc	DENND1C FA	0.0126	0.2998	-0.4568	-0.7537	-0.8259	-1.2007	-0.5103	-0.9490	-0.9203	-0.7383
1164	S-583_P278	P27824	Calnexin (IP90) (I	CANX	1.1196	1.3662	0.3530	0.4159	0.1277	-0.1226	0.2421	-0.2809	-0.4893	-0.4027
1165	S-583_P497	P49756	RNA-binding pro	RBM25 RNP	0.2131	0.7212	-0.1269	0.2145	-0.3009	-0.5381	0.0334	-0.3377	-0.4008	-0.1793
1166	S-5830_Q09	Q09666	Neuroblast differ	AHNAK PM2	-0.5396	-0.8221	1.4323	0.6482	0.8456	-0.2494	1.1279	0.4074	-0.1873	-0.3690
1167	S-5841_Q09	Q09666	Neuroblast differ	AHNAK PM2	0.5221	0.7746	0.3958	0.4900	0.1005	0.4971	-0.0787	0.6863	-0.1507	-0.4885
1168	S-585_Q926	Q92608	Dedicator of cytc	DOCK2 KIAA	-2.4308	-2.3032	-1.8027	-1.8056	-2.0592	-1.9970	-1.6664	-2.0882	-1.6930	-1.7863
1169	S-586_P356	P35611	Alpha-adducin (E	ADD1 ADDA	0.3197	-0.1421	0.0941	0.5748	0.3605	0.1120	0.4050	0.4143	0.8025	0.9262
1170	S-587_Q7LD	Q7LDG7	RAS guanyl-relea	RASGRP2 CD	-0.8491	-0.5712	-0.4583	-0.4787	-0.3123	-0.4665	0.0065	-0.4439	-0.5701	-0.4458

1171	S-588_P518	P51825	AF4/FMR2 family	AFF1 AF4 FE	-2.7948	-3.5927	-2.7210	-2.7328	-2.4845	-2.4956	-2.7339	-2.1013	-2.8389	-2.7297
1172	S-59_P0804	P08047	Transcription fac	SP1 TSFP1	-0.6687	-0.1068	0.9093	1.3965	0.0265	-0.0060	-0.1022	-1.1479	-0.2233	-0.2373
1173	S-59_Q0083	Q00839	Heterogeneous r	HNRNPU C1	-0.3595	-0.5163	-0.3225	-0.7210	-0.5477	-0.8857	-0.5252	-0.1770	-0.6332	-0.3040
1174	S-59_Q8WU	Q8WUQ7	Cactin (Renal car	CACTIN C19	-1.3641	-1.3308	-1.0190	-0.7718	-0.9378	-1.1703	-0.7579	-0.9255	-1.2664	-1.0511
1175	S-59_Q9UBE	Q9UBB9	Tuftelin-interacti	TFIP11 STIP	-1.4504	-1.4347	-1.4171	-1.3552	-1.5105	-1.4789	-1.7557	-1.7228	-1.3939	-1.2358
1176	S-590_Q9H4	Q9H4E7	Differentially exp	DEF6 IBP	-0.8010	-0.8821	0.6344	0.1243	0.2191	-0.1307	0.3581	0.0932	-0.0775	-0.4027
1177	S-590_Q9NS	Q9NSK0	Kinesin light chai	KLC4 KNSL8	-0.2309	-0.2910	0.0010	0.0661	-0.1120	0.0540	-0.1943	-0.1029	-0.0104	-0.0325
1178	S-591_O005	O00567	Nucleolar proteir	NOP56 NOL	0.6897	1.4240	1.5275	1.5116	2.0501	1.7105	2.0061	2.0673	1.4336	1.1784
1179	S-592_O155	O15523	ATP-dependent I	DDX3Y DBY	-0.8523	-1.2549	-1.3382	-1.3148	-1.5158	-1.8666	-1.1148	-1.1513	-1.3045	-1.2977
1180	S-592_Q9UE	Q9UBT2	SUMO-activating	UBA2 SAE2	-2.0255	-1.9256	-1.6146	-1.8056	-1.6831	-1.8720	-2.0569	-1.9460	-1.6888	-1.9961
1181	S-5943_Q8N	Q8NF91	Nesprin-1 (Enapt	SYNE1 C6orf	-0.3251	-0.5306	-0.1557	-0.2627	-0.1708	-0.0561	-0.2927	-0.3610	-0.3190	-0.0903
1182	S-595_O608	O60841	Eukaryotic transl	EIF5B IF2 KIA	-0.9022	-0.6541	-0.5080	-0.8087	-0.3995	-0.6457	-0.7040	-0.6854	-0.6053	-0.8685
1183	S-596_Q9Y3	Q9Y3L3	SH3 domain-binc	SH3BP1	0.6475	0.6481	-0.1314	-0.0842	-0.2044	-0.3257	-0.1504	-0.2716	0.1978	0.3762
1184	S-597_Q9H4	Q9H4E7	Differentially exp	DEF6 IBP	-0.4728	-0.5633	0.1057	-0.0582	-0.1345	-0.0674	-0.3429	-0.0030	0.2779	0.0442
1185	S-598_Q029	Q02952	A-kinase anchor	AKAP12 AKA	-0.3228	-0.3128	-0.2773	-0.3898	0.2175	0.2266	-0.3829	-0.3185	0.0232	-0.0286
1186	S-6_P09651	P09651	Heterogeneous r	HNRNPA1 H	1.1362	1.1128	0.4957	0.9214	0.4183	0.5820	0.5610	0.5850	0.4491	0.5042
1187	S-6_Q9Y3Z3	Q9Y3Z3	Deoxynucleoside	SAMHD1 MC	-0.6041	-1.4928	0.0655	0.0053	0.1920	-0.4502	0.4739	0.2233	-0.6536	-0.6129
1188	S-60_O0050	O00505	Importin subunit	KPNA3 QIP2	0.3724	0.2616	0.4680	0.4025	0.3678	0.4001	0.0783	0.2479	0.2622	0.1078
1189	S-60_Q8N68	Q8N684	Cleavage and pol	CPSF7	1.6480	1.6127	0.5723	0.8192	0.7081	0.6193	1.0535	0.7367	0.6415	0.3573
1190	S-60_Q8N6F	Q8N6F7	Germinal center-	GCSAM GAL	NA	NA	NA	NA	-5.2919	-4.9676	-5.2597	-6.2977	-5.7227	-5.0013
1191	S-600_Q7Z4	Q7Z460	CLIP-associating	CLASP1 KIAA	1.4916	1.4427	1.5525	1.3802	1.2000	1.5002	1.4781	1.3225	1.1228	0.7321
1192	S-601_Q8N4	Q8N4C8	Misshapen-like k	MINK1 B55	-0.0666	-0.0611	0.8284	1.2045	0.5103	0.6892	0.7001	0.6350	-0.4111	-0.4215
1193	S-602_Q9BZ	Q9BZQ8	Protein Niban 1 (	NIBAN1 C1o	-0.8814	-0.8843	-0.3855	-0.3911	-0.9203	-0.6774	-0.8095	-0.7750	-1.4168	-1.4309
1194	S-604_Q154	Q15424	Scaffold attachm	SAFB HAP HI	1.5994	1.4896	1.3693	1.3073	1.3258	1.4241	1.3906	0.9660	1.2703	0.9342
1195	S-607_Q9NF	Q9NRH2	SNF-related serir	SNRK KIAA0	-1.5634	-1.3732	-1.4655	-1.9562	-1.5914	-1.4956	-1.6872	-1.4191	-1.4820	-1.7254
1196	S-608_P460	P46063	ATP-dependent I	RECQL RECC	-2.0780	-1.9989	-1.6372	-1.2799	-1.2829	-1.3711	-1.0065	-1.0625	-1.6638	-1.4198
1197	S-61_Q9H1E	Q9H1E3	Nuclear ubiquito	NUCKS1 NU	-0.0991	-0.2030	-0.0874	1.2290	0.9019	0.2023	0.2509	0.6281	-0.0263	0.5178
1198	S-61_Q9H40	Q9H400	Lck-interacting tr	LIME1 LIME	-1.3456	-1.1164	-0.6903	-0.5787	-0.6279	-0.5820	-0.5308	-0.6586	-0.9376	-0.6031
1199	S-61_Q9UH	Q9UH62	Armadillo repeat	ARMCX3 ALI	0.4955	0.1602	0.5968	0.5796	0.3214	0.5201	0.3662	0.3534	0.3848	0.3370
1200	S-611_Q9UK	Q9UKN8	General transcrip	GTF3C4	0.1213	-0.0067	-0.0752	-0.1439	-0.2851	-0.1017	-0.1913	0.1202	0.0473	0.2215
1201	S-614_Q9BY	Q9BYW2	Histone-lysine N-	SETD2 HIF1	-2.1089	-2.2869	-1.5155	-1.4156	-1.6713	-1.5167	-1.0815	-1.4860	-1.5376	-1.4795
1202	S-617_Q5H9	Q5H9R7	Serine/threonine	PPP6R3 C11	-0.5297	-0.7994	0.1279	0.1979	0.0154	0.1962	-0.3147	-0.0562	0.1392	-0.0212
1203	S-6175_Q8N	Q8NF91	Nesprin-1 (Enapt	SYNE1 C6orf	-0.2937	-0.2655	0.1547	0.1112	0.0901	0.0324	-0.1219	-0.1211	0.0599	0.1397
1204	S-619_O433	O43395	U4/U6 small nuc	PRPF3 HPRP	-0.5110	-0.4928	-0.3977	-0.5472	-0.3169	-0.2780	-0.5765	-0.2883	-0.5566	-0.7447
1205	S-619_P193	P19338	Nucleolin (Protei	NCL	-0.2035	-0.1272	-0.5351	-0.4936	-0.7620	-0.5082	-0.7019	-0.2370	-0.3652	-0.3152
1206	S-619_Q7KZ	Q7KZI7	Serine/threonine	MARK2 EMK	-1.3228	-1.3225	-1.3303	-1.4541	-1.8723	-1.3071	-1.2566	-1.3988	-1.3304	-1.2726
1207	S-62_Q6UU	Q6UUV7	CREB-regulated t	CRTC3 TORC	0.0000	0.0252	-1.2863	-0.3782	-1.2784	-0.3350	-0.3412	-0.4606	-0.2853	-0.9432
1208	S-62_Q8NFG	Q8NFG4	Folliculin (BHD sl	FLCN BHD	0.4552	0.7151	0.4077	-0.0397	0.2411	0.2791	0.4147	0.3232	0.4026	0.1834
1209	S-62_Q96E0	Q96E09	Protein FAM122	FAM122A C9	-0.9225	-1.0764	-0.7329	-0.7448	-0.5822	-0.6177	-0.6718	-0.6092	-0.7107	-0.8689

1210	S-62_Q9Y3C	Q9Y3Q8	TSC22 domain fa	TSC22D4 TH	-0.4129	-0.6174	-0.4595	-0.1270	-0.1064	-0.4461	-0.0499	-0.2681	-0.3410	-0.1374
1211	S-620_P4268	P42684	Tyrosine-protein	ABL2 ABLL A	-1.0366	-1.1333	-0.9486	-0.9597	-0.9378	-0.9881	-0.9781	-0.8410	-0.8766	-1.1504
1212	S-620_Q6PJ	Q6PJT7	Zinc finger CCCH	ZC3H14	0.0911	0.2689	0.4443	0.7028	0.2780	0.2368	0.4167	0.1439	-0.0706	-0.2013
1213	S-621_Q8N3	Q8N3F8	MICAL-like prote	MICALL1 KIA	0.5209	0.6196	-0.2398	-0.1013	-0.1544	-0.2565	-0.0355	-0.2352	-0.2157	0.0813
1214	S-623_P529	P52948	Nuclear pore cor	NUP98 ADAI	0.3356	0.3520	-0.0609	0.2508	0.2270	0.1722	0.1701	0.1549	0.1186	0.1902
1215	S-624_O954	O95466	Formin-like prote	FMNL1 C17c	1.9124	2.2308	1.1571	1.5772	1.5116	1.1212	1.6873	1.4291	0.9409	1.1823
1216	S-624_P527	P52756	RNA-binding pro	RBM5 H37 L	-0.3552	-0.2016	-0.4927	-0.5377	-0.3906	-0.4303	-0.5130	-0.6152	-0.3679	-0.2343
1217	S-624_Q53E	Q53ET0	CREB-regulated t	CRTC2 TORC	-0.0255	0.0490	0.3332	0.4226	-0.0176	0.3865	0.2065	0.2337	0.2643	0.3381
1218	S-625_Q7Z4	Q7Z4V5	Hepatoma-deriv	HDGFL2 HD	-3.7823	-3.3364	-2.9608	-2.6412	-3.7558	-2.8884	-3.1634	-2.9169	-2.9858	-2.6876
1219	S-625_Q9N	Q9NXH9	tRNA (guanine(2	TRMT1	-0.5959	-0.4574	-0.9730	-1.1479	-1.1219	-1.1017	-1.0302	-1.2753	-1.4967	-1.3591
1220	S-627_Q150	Q15025	TNFAIP3-interact	TNIP1 KIAA0	-1.1167	-1.2339	-0.8528	-1.4062	-1.4289	-0.7148	-1.0275	-0.7380	-0.9326	-1.1764
1221	S-628_Q8N9	Q8N9T8	Protein KRI1 hon	KRI1	-0.0246	-0.2002	-0.4431	-0.8049	-0.2344	-0.0610	-0.1583	-0.0315	-0.4642	-0.3449
1222	S-628_Q926	Q92625	Ankyrin repeat a	ANKS1A ANI	-1.4308	-1.2522	-1.1592	-1.6412	-1.1626	-1.0180	-1.1461	-1.1046	-0.9602	-0.8406
1223	S-63_Q1342	Q13422	DNA-binding pro	IKZF1 IK1 IK	-0.2569	-0.1675	-0.6742	-0.7767	-0.6799	-0.7689	-0.7145	-0.7111	-0.7013	-0.6358
1224	S-63_Q1344	Q13442	28 kDa heat- and	PDAP1 HASF	0.3130	0.1093	0.7446	0.4019	0.5854	0.1931	0.0447	0.0676	1.2974	1.2692
1225	S-63_Q6NXT	Q6NXT1	Ankyrin repeat d	ANKRD54 LI	-1.4869	-1.0945	-0.7662	-0.5453	-0.7755	-0.7386	-0.7876	-0.7926	-0.9117	-0.9165
1226	S-630_Q7Z6	Q7Z6I6	Rho GTPase-activ	ARHGAP30	0.4459	0.7589	-0.7684	-0.4444	-0.3055	-0.3539	-0.1634	-0.5163	-0.6152	-0.6109
1227	S-631_Q6PK	Q6PKG0	La-related protei	LARP1 KIAA	-0.4138	-0.5387	-0.6635	-0.2842	-0.7250	-0.6597	-0.6218	-0.6659	-0.2447	-0.0247
1228	S-631_Q928	Q92888	Rho guanine nuc	ARHGEF1	1.3675	1.2917	1.0280	0.6818	1.2128	1.0984	1.2896	1.2910	0.9876	0.5267
1229	S-633_Q020	Q02040	A-kinase anchor	AKAP17A CX	-0.8787	-0.4959	-0.6903	-0.4614	-0.0746	-0.9852	0.4254	-0.2624	-1.1755	-1.0378
1230	S-633_Q9Y5	Q9Y520	Protein PRRC2C (	PRRC2C BAT	-1.3410	-1.3703	-0.7827	-0.8954	-0.8690	-1.0953	-0.1693	-0.8714	-1.5338	-1.2479
1231	S-634_O146	O14617	AP-3 complex su	AP3D1 PRO	-0.9433	-1.0145	-0.4312	-0.6749	-0.6082	-0.5082	-0.9452	-0.6586	-0.4303	-0.1264
1232	S-635_O153	O15320	Melanoma inhibi	MIA2 CTAGE	0.2993	0.3528	0.1481	0.2448	-0.0477	0.1307	0.1631	0.0407	0.1435	0.1880
1233	S-635_Q9UF	Q9UPN7	Serine/threonine	PPP6R1 KIA	-2.8988	-2.6472	-2.2991	-2.2206	-2.5914	-2.5643	-2.7339	-2.4564	-2.4077	-2.2914
1234	S-637_Q018	Q01826	DNA-binding pro	SATB1	-0.6481	-0.3760	-0.7527	-0.3491	-0.5299	-0.5112	-0.7734	-0.6543	-0.2293	-0.0291
1235	S-638_Q8IYE	Q8IYB3	Serine/arginine r	SRRM1 SRM	-0.5904	-0.0926	-0.7279	-1.4015	-0.6279	-1.5643	-0.5289	-1.2280	-0.7819	0.1686
1236	S-638_Q9NV	Q9NW82	WD repeat-conta	WDR70	0.3212	0.3177	0.5815	0.5361	0.6353	0.8112	0.4475	0.6681	0.9537	0.8009
1237	S-638_Q9UF	Q9UPN7	Serine/threonine	PPP6R1 KIA	-2.0553	-2.0302	-1.6668	-1.5975	-2.0065	-1.7419	-1.9884	-1.8714	-1.9704	-1.5798
1238	S-639_O958	O95819	Mitogen-activate	MAP4K4 HG	-1.5528	-1.6507	-1.9445	-2.3237	-1.9203	-1.9050	-1.7912	-1.9401	-2.2696	-2.0699
1239	S-64_Q53QZ	Q53QZ3	Rho GTPase-activ	ARHGAP15 E	0.2084	0.3571	0.6085	1.1502	0.5129	0.9047	0.8639	0.4837	0.4163	0.1548
1240	S-64_Q9BPX	Q9BPX5	Actin-related pro	ARPC5L	0.2317	0.2680	0.4179	0.4811	0.3848	0.5559	0.1243	0.3830	0.1029	0.3350
1241	S-640_Q150	Q15052	Rho guanine nuc	ARHGEF6 CC	-0.4653	-0.3183	-0.9305	-1.0161	-0.6166	-0.9561	-0.5707	-0.8883	-0.9451	-0.8452
1242	S-640_Q9UC	Q9UGU0	Transcription fac	TCF20 KIAA	0.2651	0.2326	0.5374	0.3123	0.4279	0.3910	0.2704	0.2998	0.5125	0.4066
1243	S-642_O437	O43719	HIV Tat-specific f	HTATSF1	0.2193	0.4354	0.1845	-0.0197	-0.1564	0.0163	-0.0967	0.2141	0.4026	0.1571
1244	S-645_P542	P54259	Atrophin-1 (Dent	ATN1 D12S7	-0.1665	-0.4169	-0.1745	-0.1167	-0.1080	-0.1703	-0.1973	0.0242	0.0993	-0.1954
1245	S-647_Q926	Q92625	Ankyrin repeat a	ANKS1A ANI	-0.1584	-0.1504	-0.5035	-0.0507	-0.2279	-0.2128	-0.3478	-0.0705	-0.1696	-0.2192
1246	S-648_Q9BV	Q9BWT3	Poly(A) polymera	PAPOLG PAF	-0.8265	-1.0189	-0.6785	-0.6863	-0.6194	-0.6715	-0.7733	-0.5517	-0.5566	-0.5837
1247	S-648_Q9NY	Q9NYF8	Bcl-2-associated	BCLAF1 BTF	-0.4393	-0.1046	0.3257	0.5474	0.3609	0.0442	0.1336	0.1573	0.2157	0.3331
1248	S-65_P5270	P52701	DNA mismatch re	MSH6 GTBP	0.1847	0.7465	1.1914	1.6857	1.8172	1.4152	1.3936	1.5305	1.4386	1.4896

1249	S-65_Q1515	Q15154	Pericentriolar ma	PCM1	-0.7334	-0.6112	0.1900	-0.1244	-0.0883	-0.1736	-0.1389	-0.1650	-0.0276	-0.2418
1250	S-65_Q969E	Q969E4	Transcription elo	TCEAL3 MST	0.5081	0.7275	-0.3817	-0.0697	0.3588	0.0416	0.2004	0.1302	0.1717	0.2133
1251	S-65_Q96S5	Q96S55	ATPase WRNIP1	WRNIP1 WH	-0.3663	-0.3895	-0.0741	0.0291	0.0837	-0.2193	-0.4207	-0.1744	0.1289	0.1716
1252	S-652_Q7Z4	Q7Z417	Nuclear fragile X	NUFIP2 KIAA	-0.1464	-0.3239	0.5667	0.3890	0.1626	0.5128	0.0833	0.4650	0.3888	0.3812
1253	S-652_Q7Z4	Q7Z4V5	Hepatoma-derive	HDGFL2 HD(	-0.8721	-0.8319	-0.3436	-0.1677	-0.3850	-0.5798	-0.4017	-0.4946	-0.4042	-0.4078
1254	S-653_Q96R	Q96RL1	BRCA1-A comple	UIMC1 RAP8	-0.9643	-0.8678	-0.6852	-1.0786	-0.7373	-0.8829	-1.0489	-0.7952	-0.7378	-0.4994
1255	S-654_O602	O60281	Zinc finger protei	ZNF292 KIAA	-0.2458	-0.7219	-0.6602	-0.5629	-0.3898	-0.2007	-0.1781	-0.2262	-0.6763	-0.7169
1256	S-656_Q994	Q99490	Arf-GAP with GTI	AGAP2 CENT	-4.6402	-4.3253	-1.0318	-1.7507	-0.7159	-0.4300	-0.9704	-0.6112	-0.8067	-0.8291
1257	S-657_Q9H5	Q9H501	ESF1 homolog (A	ESF1 ABTAP	-1.2566	-1.4451	-1.2296	-1.3671	-1.2324	-0.8568	-1.3586	-1.4122	-0.9089	-0.7946
1258	S-657_Q9UK	Q9UKV3	Apoptotic chrom	ACIN1 ACINU	1.3633	1.2411	1.4226	1.4528	1.1201	1.0583	1.1918	1.2466	1.5056	1.3391
1259	S-658_Q8W	Q8WVC0	RNA polymerase	LEO1 RDL	-1.1325	-1.1603	-1.0126	-0.8087	-1.1462	-1.1570	-1.1605	-1.1244	-0.8273	-0.9859
1260	S-658_Q9NY	Q9NYF8	Bcl-2-associated	BCLAF1 BTF	-2.0403	-2.3420	-1.5185	-1.9356	-2.0746	-1.8397	-2.2411	-1.8112	-1.6232	-1.5012
1261	S-66_Q96B2	Q96B23	Uncharacterized	C18orf25 AR	0.2363	-0.0257	-0.3867	-0.3944	0.2064	-0.5577	-0.4435	-0.6781	-0.2680	-0.1054
1262	S-66_Q9H30	Q9H307	Pinin (140 kDa nu	PNN DRS ME	0.8068	0.7285	1.2906	1.1725	1.1960	1.2139	1.2693	1.4639	1.0328	0.7313
1263	S-660_Q9NF	Q9NPG3	Ubinuclein-1 (HIF	UBN1	-1.4211	-1.2681	-1.1733	-1.5975	-1.7190	-1.1703	-1.2535	-1.8603	-1.2052	-1.1533
1264	S-663_P166:	P16615	Sarcoplasmic/en	ATP2A2 ATP	-0.6179	-1.0100	-0.8229	-0.7094	-0.6801	-1.0392	-0.9131	-0.8742	-0.7142	-0.4493
1265	S-663_Q9H5	Q9H501	ESF1 homolog (A	ESF1 ABTAP	-0.2805	-0.4228	-0.2901	-0.3991	-0.3470	-0.1049	-0.3462	-0.2606	-0.0982	-0.0739
1266	S-664_Q7Z4	Q7Z4V5	Hepatoma-derive	HDGFL2 HD(	-0.1305	-1.3060	-0.5923	0.1788	-0.1159	-0.3769	-0.4242	-0.4212	-0.5778	-0.5456
1267	S-665_Q96T	Q96TA1	Protein Niban 2 (	NIBAN2 C9o	-0.6487	-0.6129	-0.1698	0.2613	-0.3493	-0.2726	-0.4068	-0.9284	-0.8181	-0.6050
1268	S-666_O002	O00267	Transcription elo	SUPT5H SPT	0.1453	0.1465	-0.3238	-0.4729	-0.1695	-0.1126	-0.1455	-0.1259	-0.0189	0.1185
1269	S-669_Q96S	Q96SB8	Structural mainte	SMC6 SMC6	-0.9055	-0.6403	-0.7524	-0.7840	-0.8129	-0.7874	-0.8888	-0.9939	-0.4985	-0.5085
1270	S-67_O1504	O15042	U2 snRNP-associ	U2SURP KIA	0.0706	0.0318	0.0605	0.0247	-0.3100	0.0683	0.0384	-0.0278	0.1005	0.2596
1271	S-67_O4376	O43768	Alpha-endosulfin	ENSA	1.6071	1.4370	0.9564	0.9576	0.7634	1.4806	1.1884	1.2646	1.3237	1.3155
1272	S-67_P4959(	P49590	Histidine--tRNA l	HARS2 HARS	-0.7730	-0.8985	-1.1269	-1.2972	-1.3423	-1.2852	-0.9909	-1.2280	-1.4146	-1.0431
1273	S-67_Q86VN	Q86VM9	Zinc finger CCCH	ZC3H18 NHN	-2.1135	-2.1853	-2.4820	-1.9566	-1.8613	-2.4166	-2.0212	-2.2037	-2.8469	-2.4331
1274	S-67_Q96FS	Q96FS4	Signal-induced p	SIPA1 SPA1	1.4103	1.5327	1.1118	1.1835	0.6623	1.4225	1.0875	1.1039	1.0871	0.8453
1275	S-67_Q9BXP	Q9BXP5	Serrate RNA effe	SRRT ARS2 A	-0.4066	-0.2842	-0.1406	-0.1697	-0.5423	-0.0090	-0.3544	-0.1822	0.0000	-0.0551
1276	S-670_P250!	P25098	Beta-adrenergic	GRK2 ADRBK	2.1735	2.0482	2.0451	2.1622	2.0992	2.2023	2.1564	2.1875	2.0886	1.7266
1277	S-670_Q96T	Q96T37	RNA-binding pro	RBM15 OTT	-0.2138	-0.3334	0.0780	-0.2764	-0.1032	0.1745	-0.0150	0.0369	0.0060	-0.2212
1278	S-670_Q9UF	Q9UPN7	Serine/threonine	PPP6R1 KIAA	-4.3183	-4.1578	-3.7419	-3.7094	-5.0902	-3.9446	-4.0516	-3.7127	-3.1843	-3.5607
1279	S-672_Q8N5	Q8N5C8	TGF-beta-activat	TAB3 MAP3I	NA	NA	-2.8970	-3.1717	-3.2387	-3.3219	-2.9477	-3.6928	-3.9451	-3.3362
1280	S-672_Q9C0	Q9C0C2	182 kDa tankyras	TNKS1BP1 K	-0.1245	-0.2937	-0.3251	-0.2062	-0.2851	0.0964	-0.1988	-0.0529	0.1494	-0.0090
1281	S-672_Q9Y2	Q9Y2W1	Thyroid hormone	THRAP3 BCL	0.1345	0.5033	0.6208	0.8929	0.9098	0.4748	0.6929	0.6681	0.6331	0.5888
1282	S-674_P783	P78347	General transcrip	GTF2I BAP1:	-0.4579	-0.7056	0.7163	0.5114	1.0355	0.2304	1.1148	0.7960	1.2965	1.6397
1283	S-674_Q96T	Q96T37	RNA-binding pro	RBM15 OTT	-1.2182	-1.3392	-1.8247	-1.5240	-1.5210	-1.2388	-1.6872	-1.7278	-1.5077	-1.5307
1284	S-675_Q8IYE	Q8IYB3	Serine/arginine r	SRRM1 SRM	1.2614	1.2804	1.6208	2.2456	2.2282	1.8251	2.0878	2.0280	1.3893	1.7153
1285	S-675_Q8TC	Q8TC07	TBC1 domain fan	TBC1D15	-0.0422	-0.1714	-0.2559	-0.4688	-0.4615	-0.3425	-0.5011	-0.5857	-0.2853	-0.1576
1286	S-677_P461(	P46100	Transcriptional r	ATRX RAD54	1.0436	1.1542	0.2001	0.3407	0.4590	-0.0469	0.3752	-0.0466	0.2567	0.3350
1287	S-678_P356!	P35658	Nuclear pore cor	NUP214 CAI	0.2177	0.3803	0.7447	0.4463	0.3932	0.2680	0.3438	0.3450	0.3908	0.3563

1288	S-678_Q4KM	Q4KMP7	TBC1 domain fan	TBC1D10B F	0.6112	0.6058	0.5794	0.6852	0.4934	0.4068	0.7286	0.4650	0.3112	0.3175
1289	S-678_Q9H0	Q9H0D6	5'-3' exoribonucl	XRN2	0.1719	0.1572	-0.0137	-0.2123	-0.1259	-0.1487	-0.1943	-0.1996	-0.1652	-0.1605
1290	S-680_Q9UK	Q9UKE5	TRAF2 and NCK-i	TNIK KIAA05	0.5334	0.3511	0.4489	0.3890	0.1968	0.4169	0.3621	0.2531	0.2535	-0.0155
1291	S-682_Q9Y2	Q9Y2W1	Thyroid hormone	THRAP3 BCL	1.0714	1.0388	1.5273	1.3965	1.3317	1.4456	1.1827	1.3402	1.5767	1.5334
1292	S-683_P154!	P15498	Proto-oncogene	VAV1 VAV	-0.3206	-0.1309	-0.6953	-0.6777	-0.6166	-0.6858	-0.6893	-0.6756	-0.9858	-0.7085
1293	S-684_Q150	Q15052	Rho guanine nuc	ARHGEF6 CC	-0.1245	-0.1188	-0.5611	-0.4614	-0.2150	-0.5061	-0.3023	-0.5926	-0.5096	-0.4406
1294	S-686_Q9BS	Q9BSJ8	Extended synapt	ESYT1 FAM6	0.0843	0.0912	0.4101	0.3065	-0.0120	0.5273	0.3170	0.3534	0.3320	0.0716
1295	S-687_P051(	P05164	Myeloperoxidase	MPO	-0.7007	-0.7552	-1.1292	-1.0937	-1.0288	-1.1339	-1.1634	-1.4775	-0.8886	-0.7997
1296	S-687_Q134	Q13427	Peptidyl-prolyl ci	PPIG	0.3598	0.4313	0.4381	0.7669	0.2270	0.1469	0.2077	0.1875	0.2270	0.4402
1297	S-688_Q9UK	Q9UKE5	TRAF2 and NCK-i	TNIK KIAA05	-1.9398	-1.9553	-1.2410	-1.6863	-1.3995	-1.6692	-1.7040	-1.5789	-1.6930	-1.9859
1298	S-69_Q7L2J(	Q7L2J0	7SK snRNA meth	MEPCE BCDI	-0.3595	-0.0565	0.6664	0.3008	0.4710	0.3693	-0.0236	0.2466	0.3888	0.4450
1299	S-69_Q9H6F	Q9H6F5	Coiled-coil doma	CCDC86 CYC	-1.0742	-0.9857	-1.0885	-1.0018	-0.8064	-0.8370	-0.5535	-0.9460	-1.4146	-0.9987
1300	S-690_Q9NY	Q9NYF8	Bcl-2-associated	BCLAF1 BTF	0.2628	0.3024	0.4905	0.3010	0.6332	0.2914	0.5870	0.4268	0.7596	0.7212
1301	S-691_Q129	Q12996	Cleavage stimula	CSTF3	-0.5422	-0.7440	-0.8931	-0.3439	-0.6480	-0.5999	-0.5459	-0.6323	-0.9628	-0.7106
1302	S-695_P515:	P51532	Transcription act	SMARCA4 B/	0.6974	0.5638	0.8112	0.4657	0.5000	0.7166	0.5923	0.5257	0.4357	0.6252
1303	S-695_Q047	Q04759	Protein kinase C	PRKCQ PRKC	-1.3456	-1.3905	-1.5215	-1.1558	-1.9844	-0.7670	-0.9402	-1.0434	-1.5338	-1.5419
1304	S-696_Q8IYE	Q8IYB3	Serine/arginine r	SRRM1 SRM	-0.6107	-0.5307	0.0908	0.1335	0.2055	-0.2108	0.3600	0.1837	-0.0178	0.2232
1305	S-696_Q929	Q92974	Rho guanine nuc	ARHGEF2 KI/	0.1378	0.0318	-0.1246	-0.1878	-0.1750	-0.1001	-0.2784	-0.2424	-0.4338	-0.1910
1306	S-698_Q149	Q14978	Nucleolar and co	NOLC1 KIAA	0.7387	0.5247	0.4188	0.3061	0.3261	0.5586	0.4332	0.3293	0.3892	0.3015
1307	S-698_Q9Y2	Q9Y2W1	Thyroid hormone	THRAP3 BCL	-1.2266	-1.0762	-1.4368	-0.8562	-1.2002	-0.9106	-1.0787	-0.7482	-1.2633	-1.5419
1308	S-699_Q155	Q15596	Nuclear receptor	NCOA2 BHLI	1.0269	0.9721	0.6775	0.8804	0.6716	0.6433	0.6447	0.6182	0.7767	0.8431
1309	S-7_O75995	O75995	SAM and SH3 do	SASH3 CXorf	-0.3161	-0.2788	0.8023	0.8040	1.0604	0.1854	0.8553	0.4771	0.2783	0.0837
1310	S-7_Q01105	Q01105	Protein SET (HLA	SET	0.5057	0.7661	-0.2660	0.0036	-0.4240	-0.2654	-0.1884	-0.2661	-0.4285	-0.4319
1311	S-7_Q09161	Q09161	Nuclear cap-bind	NCBP1 CBP8	-3.0635	-2.8296	-2.3334	-1.7938	-1.7002	-2.0201	-1.9131	-1.9593	-2.5005	-2.3448
1312	S-70_Q9H40	Q9H400	Lck-interacting tr	LIME1 LIME	-0.1952	-0.1021	-0.0468	0.0830	-0.1564	0.2876	0.0808	0.1955	-0.1122	-0.1778
1313	S-700_Q96T	Q96T37	RNA-binding pro	RBM15 OTT	0.2363	0.0901	0.1873	0.2627	0.2048	0.1171	0.1941	0.2415	0.0920	0.4269
1314	S-701_Q134	Q13428	Treacle protein (	TCOF1	0.0463	0.3336	0.3693	0.4696	0.3721	0.5860	0.2189	0.5520	0.8384	0.5751
1315	S-701_Q9U(	Q9UQ88	Cyclin-dependen	CDK11A CD(	-0.0329	0.1271	-0.3785	-0.2310	0.1090	-0.2834	-0.0801	0.0046	-0.4163	-0.4215
1316	S-706_O146	O14639	Actin-binding LIN	ABLIM1 ABL	0.4559	0.5850	0.2115	0.0271	0.1660	0.3547	0.3750	0.2651	0.7976	0.7791
1317	S-707_Q9UK	Q9UKE5	TRAF2 and NCK-i	TNIK KIAA05	0.1686	0.1375	0.4495	0.4245	-0.0111	0.2129	0.2907	0.1632	0.0671	0.1478
1318	S-709_Q146	Q14671	Pumilio homolog	PUM1 KIAA(	0.7488	0.6594	0.2155	0.2568	0.3890	0.1523	0.1896	0.1145	0.3133	0.3299
1319	S-710_Q9UK	Q9UKV3	Apoptotic chrom	ACIN1 ACINU	0.2239	0.1320	0.8149	1.1845	1.2750	1.1029	1.1296	1.2597	0.6891	0.6692
1320	S-713_Q8IYE	Q8IYB3	Serine/arginine r	SRRM1 SRM	-2.7334	-2.0997	-2.8063	-2.8954	-2.1792	-2.5643	-2.8048	-2.7952	-3.0065	-2.2977
1321	S-718_Q131	Q13144	Translation initia	EIF2B5 EIF2E	-2.0109	-2.8478	-1.6437	-1.5550	-1.7373	-1.7222	-1.6218	-2.2753	-2.5376	-2.1822
1322	S-718_Q8IXT	Q8IXT5	RNA-binding pro	RBM12B	-0.5715	-0.1248	-0.4991	-0.3461	-0.2717	-0.5338	-0.4792	-0.5903	-0.5022	-0.1124
1323	S-72_P0867(	P08670	Vimentin	VIM	-2.1089	-1.7110	-1.9979	-1.7094	-1.6831	-1.7620	-1.5980	-1.7027	-2.4857	-2.3492
1324	S-720_Q151	Q15149	Plectin (PCN) (PL	PLEC PLEC1	0.3255	0.3345	0.1771	0.3051	0.0301	0.1656	0.2981	0.2259	0.1944	0.3930
1325	S-723_P981	P98175	RNA-binding pro	RBM10 DXS8	1.4526	1.6227	0.7869	1.3928	1.2302	0.7137	1.4127	0.9057	0.8981	1.0135
1326	S-729_Q9UK	Q9UKV3	Apoptotic chrom	ACIN1 ACINU	2.0322	2.2913	1.6020	2.0120	1.7564	1.1543	1.9484	1.5267	1.4699	1.6405

1327	S-73_O1497	O14976	Cyclin-G-associat	GAK	0.2741	0.0984	0.1992	0.3152	0.3806	0.2876	0.8241	0.8697	-0.1221	-0.3410
1328	S-73_Q6JBY9	Q6JBY9	CapZ-interacting	RCSD1 CAPZ	0.3973	0.4987	-0.0896	0.0524	-0.0554	0.0697	0.0283	-0.0834	-0.3403	-0.1562
1329	S-73_Q9261	Q92619	Rho GTPase-activ	ARHGAP45 H	-1.6919	-1.6197	0.1622	-0.8244	-0.2044	-1.3145	-0.1461	-1.0403	-0.5720	-1.0064
1330	S-731_Q9Y2	Q9Y2K2	Serine/threonine	SIK3 KIAA09	-0.0837	0.1098	-0.0339	0.0947	0.1708	-0.0546	-0.1050	-0.3185	-0.2263	-0.1166
1331	S-733_Q32M	Q32MZ4	Leucine-rich repe	LRRFIP1 GCF	-0.4090	-0.1789	-1.5366	-1.5867	-1.7069	-1.7926	-1.5980	-1.6683	-1.7184	-1.7297
1332	S-735_Q7Z3	Q7Z3C6	Autophagy-relate	ATG9A APG9	-3.3548	-3.4896	-3.4171	-3.0636	-2.9414	-3.4461	-2.9781	-3.7329	-3.5452	-3.7212
1333	S-736_Q129	Q12965	Unconventional m	MYO1E MYO	-3.5742	-3.8085	-0.0031	0.0947	-0.1060	0.4368	0.2960	0.1861	-1.9105	-1.6918
1334	S-736_Q155	Q15596	Nuclear receptor	NCOA2 BHLH	0.7402	0.7829	0.2573	0.5974	0.5524	0.4368	0.3591	0.2898	0.4338	0.3030
1335	S-736_Q8TF	Q8TF01	Arginine/serine-r	PNISR C6orf	-1.5370	-1.3703	-1.2361	-1.3461	-1.0554	-1.1177	-0.9730	-0.9698	-1.2355	-1.1082
1336	S-736_Q96T	Q96T58	Msx2-interacting	SPEN KIAA09	-1.8921	-1.8923	-1.9730	-2.1797	-1.9203	-1.8720	-2.2597	-2.2032	-1.8343	-1.6548
1337	S-737_Q9P2	Q9P2G1	Ankyrin repeat a	ANKIB1 KIAA	-1.1325	-1.0010	-0.7317	-1.0932	-1.1199	-0.9114	-0.9308	-1.1110	-1.2530	-1.2021
1338	S-739_Q9UE	Q9UER7	Death domain-as	DAXX BING2	-0.3183	-0.6841	-0.1781	0.0403	-0.2739	-0.0469	-0.2033	-0.1079	-0.0422	0.0154
1339	S-74_Q96C1	Q96C19	EF-hand domain-	EFHD2 SWS1	0.6635	0.8323	-0.0631	0.2627	-0.4240	-0.4522	-0.4505	-0.2588	-1.1038	-1.1969
1340	S-74_Q96FS	Q96FS4	Signal-induced p	SIPA1 SPA1	-2.1288	-2.0177	-1.9788	-1.9768	-1.8664	-1.8840	-1.5737	-1.6995	-2.0357	-2.0322
1341	S-74_Q9BXP	Q9BXP5	Serrate RNA effe	SRRT ARS2 A	0.2193	0.1954	-0.5565	-0.1878	-0.4717	-0.7519	-0.1913	-0.4669	-0.6951	-0.6031
1342	S-74_Q9NP5	Q9NP56	cAMP-specific 3',	PDE7B	-1.1325	-1.0622	-1.5245	-1.7447	-1.3423	-1.3983	-1.6460	-1.5562	-1.7931	-1.3959
1343	S-740_Q8IYB	Q8IYB3	Serine/arginine r	SRRM1 SRM	-0.4114	-0.5179	0.4972	0.0661	0.2395	-0.2870	0.4302	0.0091	0.5180	0.5401
1344	S-742_Q9P2	Q9P275	Ubiquitin carbox	USP36 KIAA1	-0.4728	-0.3504	-0.2201	0.0980	0.2223	0.0454	0.0039	0.0302	-0.1594	-0.1533
1345	S-743_Q08J2	Q08J23	RNA cytosine C(5	NSUN2 SAKI	-1.1932	-1.1068	-1.3489	-1.0786	-1.2258	-1.2494	-2.3039	-2.1412	-2.5605	-2.1362
1346	S-75_Q96S5	Q96S55	ATPase WRNIP1	WRNIP1 WH	0.1279	-0.1128	0.5105	0.4371	0.5810	0.5760	0.6035	0.5065	0.5609	0.2237
1347	S-752_Q9UM	Q9UMZ2	Synergin gamma	SYNRG AP1G	0.9918	1.1108	1.1262	1.0762	1.0228	0.8758	1.0672	1.0996	0.6331	0.6573
1348	S-754_Q8IYB	Q8IYB3	Serine/arginine r	SRRM1 SRM	0.3356	0.4565	0.6958	0.9685	0.9398	0.7102	0.9013	0.7924	0.6498	1.0173
1349	S-759_Q66K	Q66K74	Microtubule-asso	MAP1S BPY2	-0.0857	-0.3183	0.2396	-0.3193	-0.0269	0.1656	-0.0289	-0.0915	-0.2293	-0.1334
1350	S-76_O1482	O14828	Secretory carrier	SCAMP3 C1o	0.1719	0.3319	0.3373	0.6707	0.9702	0.8179	0.5352	0.5299	0.0834	0.4487
1351	S-76_Q0143	Q01433	AMP deaminase	AMPD2	-1.4691	-1.5356	0.7142	-0.4931	0.4188	-1.2389	0.1068	-1.0023	-0.8424	-1.3122
1352	S-76_Q96E0	Q96E09	Protein FAM122A	FAM122A C9	-1.0628	-1.1260	-1.1639	-1.0674	-1.1875	-0.8995	-0.8626	-1.2716	-0.7752	-0.8429
1353	S-76_Q9UJ7	Q9UJ70	N-acetyl-D-gluco	NAGK	-0.5124	-0.6487	-0.6631	-0.6870	-0.7124	-0.5316	-0.8418	-0.8386	-0.5698	-0.6403
1354	S-763_Q8N4	Q8N4C8	Misshapen-like k	MINK1 B55 I	-0.4308	-0.4066	-0.0126	-0.0251	-0.2475	-0.2708	-0.2063	-0.4069	-0.9704	-0.5288
1355	S-766_P4324	P43243	Matrin-3	MATR3 KIAA	0.3255	0.9476	0.5134	0.8629	0.2519	0.9268	0.0918	0.8580	0.3758	0.3802
1356	S-769_Q8IYB	Q8IYB3	Serine/arginine r	SRRM1 SRM	0.3724	0.2717	0.2927	0.2478	0.4737	0.6076	0.2661	0.3426	0.4395	0.6679
1357	S-77_O6092	O60927	E3 ubiquitin-prot	PPP1R11 HC	-2.2309	-1.9467	-1.7004	-2.0125	-1.9699	-2.1145	-2.2350	-2.2388	-1.3871	-1.4061
1358	S-77_Q1506	Q15061	WD repeat-conta	WDR43 KIAA	-1.1524	-0.4819	-0.7471	-0.0197	-0.2002	-0.2110	-0.3379	-0.5033	-0.7487	-0.3008
1359	S-77_Q5T20	Q5T200	Zinc finger CCCH	ZC3H13 KIAA	0.8941	1.0776	-0.6275	-0.4565	-0.4640	-0.8802	-0.3363	-0.6830	-0.9252	-0.3690
1360	S-77_Q7Z5K	Q7Z5K2	Wings apart-like	WAPL FOE K	1.2927	1.3025	0.6558	0.8929	0.8861	0.4368	0.9410	0.6825	0.5143	0.7920
1361	S-771_Q134	Q13428	Treacle protein (T	TCOF1	-0.0219	0.0328	-0.1604	-0.1361	-0.2717	-0.2834	-0.3445	-0.1892	0.1029	0.0605
1362	S-772_Q7Z6	Q7Z6E9	E3 ubiquitin-prot	RBBP6 P2PR	-4.3548	-3.1777	-2.9206	-3.3782	-3.3850	-3.7320	-2.7168	-3.6159	-3.5759	-3.6070
1363	S-777_Q134	Q13428	Treacle protein (T	TCOF1	-0.7394	-0.3661	-0.4496	-0.3170	-0.5343	-0.3406	-0.5066	-0.4110	-0.0871	-0.1433
1364	S-779_Q9H2	Q9H2G2	STE20-like serine	SLK KIAA020	-0.3510	-0.3262	-0.5396	-0.2075	-0.4711	0.0637	-0.1392	-0.2395	-0.4555	-0.6134
1365	S-78_P13861	P13861	cAMP-dependen	PRKAR2A PK	-1.8988	-1.7969	-1.0490	-1.4204	-1.4289	-1.4624	-1.7557	-1.2977	-1.1579	-1.0917

1366	S-782_Q8TD	Q8TDD1	ATP-dependent I	DDX54	-0.3251	-0.3406	0.3055	0.2964	0.1741	0.2431	0.3232	0.2772	0.0000	0.1800
1367	S-782_Q929	Q92974	Rho guanine nuc	ARHGEF2 KI/	-2.9820	-2.0033	-0.9937	-1.1797	-1.1300	-0.8055	-1.5746	-0.9519	-1.2633	-1.4441
1368	S-787_Q927	Q92794	Histone acetyltra	KAT6A MOZ	-0.0422	-0.1467	-0.6339	-0.5761	-0.6308	-0.6272	-0.4103	-0.6659	-0.7597	-0.5934
1369	S-79_P2636	P26368	Splicing factor U	U2AF2 U2AF	1.4402	1.7053	0.9331	1.0362	1.1463	0.7046	1.1698	0.8906	0.8985	0.8945
1370	S-79_Q1677	Q16778	Histone H2B type	H2BC21 H2E	-1.0403	-1.2923	-0.3922	-0.0842	-0.0028	-0.9446	-0.3103	-0.6182	-0.6536	-0.6508
1371	S-792_P422	P42229	Signal transducer	STAT5A STA	-1.4259	-2.0302	-1.5035	-1.1919	-1.2044	-1.0701	-1.2442	-1.0340	-1.0598	-0.9910
1372	S-795_O753	O75362	Zinc finger protei	ZNF217 ZAB	0.0671	0.3016	0.6140	0.8492	0.5711	0.4844	0.4541	0.6468	0.6810	0.6143
1373	S-795_Q140	Q14005	Pro-interleukin-1	IL16	0.3966	0.1567	0.7922	-0.1724	0.5866	-0.4309	0.7590	0.2935	0.0418	-0.0242
1374	S-795_Q5T8	Q5T8P6	RNA-binding pro	RBM26 C13c	-0.1808	-0.1665	0.2833	0.3795	0.1855	0.1143	0.3149	0.3377	0.4811	0.5097
1375	S-797_Q8IYE	Q8IYB3	Serine/arginine r	SRRM1 SRM	0.2286	0.2392	0.1557	0.2804	-0.1300	0.0338	0.0000	0.1576	-0.1321	-0.2664
1376	S-799_O949	O94979	Protein transpor	SEC31A KIA/	0.6278	0.6057	0.9112	0.8332	0.6631	0.9389	0.5960	0.6817	0.7786	0.5373
1377	S-80_P5160	P51608	Methyl-CpG-binc	MECP2	1.8867	1.9127	0.7239	0.8122	0.5822	0.9158	1.1718	0.8781	-0.3635	0.0752
1378	S-80_Q1564	Q15649	Zinc finger HIT d	ZNHIT3 TRIP	-0.2674	-0.4574	-0.4439	-0.4936	-0.3778	-0.2231	-0.4594	-0.4522	-0.2187	-0.1306
1379	S-80_Q6IQ1	Q6IQ19	Centriole, cilia ar	CCSAP C1orf	-0.8921	-1.3790	-1.0796	-1.2331	-1.6422	-1.1242	-1.3071	-1.0980	-1.1696	-1.1648
1380	S-804_Q7L0	Q7L014	Probable ATP-de	DDX46 KIAA	-2.1325	-2.4051	-2.4742	-2.5240	-2.4742	-2.4220	-2.4738	-2.7741	-2.5003	-2.0645
1381	S-805_Q9BT	Q9BTC0	Death-inducer ol	DIDO1 C20o	-0.1167	0.0231	-0.2262	-0.1400	-0.3826	-0.3257	-0.3983	-0.4376	-0.2649	-0.0753
1382	S-808_Q154	Q15424	Scaffold attachm	SAFB HAP HI	-1.2394	-1.5147	-0.8340	-0.8757	-0.1139	-0.6457	-0.2319	-0.6490	-0.5528	-0.3976
1383	S-81_Q5VW	Q5VWQ0	Lysine-specific de	RSBN1	0.6463	0.6073	-0.2068	0.1866	0.1577	-0.0499	0.1397	0.2556	-0.0858	-0.1362
1384	S-810_Q8TE	Q8TEQ0	Sorting nexin-29	SNX29 RUNI	0.5801	0.7279	0.7353	0.9667	0.6945	0.8188	0.6145	0.9101	0.5915	0.5267
1385	S-819_P461	P46100	Transcriptional r	ATRX RAD54	0.8755	0.9487	0.9104	1.1038	0.9759	0.9955	0.9130	0.9862	1.0705	0.9301
1386	S-82_Q53GS	Q53GS9	U4/U6.U5 tri-snR	USP39 CGI-2	-0.6592	-0.8720	-1.0111	-0.4295	-0.7613	-0.9381	-0.4828	-0.7443	-1.2686	-0.9683
1387	S-82_Q9GZR	Q9GZR7	ATP-dependent I	DDX24	0.8466	-0.1164	0.0544	1.3123	0.8650	0.6366	0.6000	0.6872	-0.2571	-0.1837
1388	S-823_Q460	Q460N5	Protein mono-AI	PARP14 BAL	-0.1108	0.7739	-0.5766	-0.0599	-0.3730	-0.1871	-0.3628	-0.5607	-0.3702	0.0406
1389	S-825_P552	P55265	Double-stranded	ADAR ADAR	0.0000	-0.3197	-0.8604	-0.2972	-0.0028	-0.6158	0.3170	-0.3300	-1.0734	-1.4371
1390	S-829_O155	O15550	Lysine-specific de	KDM6A UTX	0.6440	0.6566	0.2599	0.5101	0.9580	0.9095	0.4128	0.2671	0.4849	0.5507
1391	S-829_Q9HC	Q9HCG8	Pre-mRNA-splicir	CWC22 KIAA	0.3241	-0.1966	0.5596	1.3931	1.4306	1.0236	0.9096	1.0444	0.4357	0.5033
1392	S-83_P1064	P10644	cAMP-dependen	PRKAR1A PK	-0.2245	-0.0867	-0.3133	-0.4204	-0.1959	-0.3145	-0.0981	-0.2827	-0.1165	-0.2373
1393	S-83_Q1497	Q14978	Nucleolar and co	NOLC1 KIAA	0.1719	0.0121	0.2747	0.2950	0.1676	0.2937	0.0894	0.3341	0.3206	0.5647
1394	S-83_Q8IV6	Q8IV63	Inactive serine/tl	VRK3	-0.6013	-0.5911	-0.2939	-0.2712	-0.6480	-0.4320	-0.2396	-0.4564	-0.1977	-0.2267
1395	S-8305_Q8N	Q8NF91	Nesprin-1 (Enapt	SYNE1 C6orf	0.0108	-0.1116	0.1030	-0.1439	-0.2695	-0.0685	-0.2319	-0.3128	-0.4839	-0.7841
1396	S-831_Q86Y	Q86YV0	RAS protein activ	RASAL3	-2.1404	-1.9945	-2.0403	-2.2123	-1.8324	-2.2424	-2.0039	-2.1412	-1.9806	-1.5837
1397	S-832_Q96S	Q96ST3	Paired amphipat	SIN3A	-0.0329	-0.2017	-0.3211	-0.4204	-0.0515	-0.0937	-0.2427	0.0496	-0.0789	-0.0739
1398	S-834_Q128	Q12872	Splicing factor, su	SFSWAP SFR	-4.6402	-3.6752	-4.0708	-5.1400	-3.3470	-3.5468	-3.3628	-3.1547	-3.5301	-3.5914
1399	S-834_Q995	Q99590	Protein SCAF11 (	SCAF11 CASI	-0.0534	-0.2483	1.0031	1.2183	1.3462	1.0483	0.8116	1.1508	0.9211	1.0255
1400	S-838_Q9Uk	Q9UKV3	Apoptotic chrom	ACIN1 ACINI	-0.1424	-0.1009	0.3661	0.0980	0.1141	-0.0826	0.1884	0.0599	0.0661	-0.1633
1401	S-84_O4356	O43561	Linker for activat	LAT	1.3125	1.1477	-0.0719	1.2598	1.2167	0.6864	1.4536	0.9455	0.0686	-0.0471
1402	S-844_Q6P2	Q6P2E9	Enhancer of mRI	EDC4 HEDLS	-0.5930	-0.5926	-0.7657	-0.7620	-0.7367	-0.6224	-1.0361	-0.9068	-0.9339	-0.9547
1403	S-844_Q8W	Q8WY36	HMG box transcr	BBX HBP2	0.9287	0.7771	1.3097	1.0988	1.4941	1.6394	1.1896	1.1117	1.5211	1.3212
1404	S-846_Q9P1	Q9P1Y6	PHD and RING fir	PHRF1 KIAA	0.9855	1.0554	-0.2262	0.2053	0.1806	-0.6067	0.4541	-0.0372	-0.4948	-0.0564

1405	S-85_P1117:	P11171	Protein 4.1 (P4.1	EPB41 E41P	-0.4653	-0.4007	0.8219	0.9242	0.7471	0.8196	1.3837	1.1821	0.8120	0.7703
1406	S-85_Q1518	Q15185	Prostaglandin E s	PTGES3 P23	-0.2394	-0.3364	0.0156	-0.0342	-0.1523	0.0236	0.1196	0.0846	-0.0476	-0.2237
1407	S-85_Q9UHI	Q9UHD8	Septin-9 (MLL se	SEPTIN9 KIA	1.5384	1.8851	1.9418	1.4900	1.4100	1.6988	1.6439	1.6886	1.2176	1.0154
1408	S-852_Q86V	Q86VM9	Zinc finger CCCH	ZC3H18 NHN	-1.7515	-1.5695	-0.8738	-0.6720	-0.6772	-1.4998	-0.5140	-1.1581	-0.8529	-0.6031
1409	S-857_Q86Y	Q86YV0	RAS protein activ	RASAL3	-0.1625	-0.3547	0.1827	-0.2041	-0.2193	-0.3827	0.1804	-0.0123	-0.6312	-0.2977
1410	S-857_Q928	Q92854	Semaphorin-4D (	SEMA4D C9	1.6800	1.9077	1.7799	1.6918	1.5748	1.8368	1.5932	1.5829	1.8264	1.5871
1411	S-86_P0511	P05114	Non-histone chr	HMGN1 HM	0.6647	0.7622	0.2263	0.7690	0.7580	0.4456	0.5108	0.6863	0.4558	0.7726
1412	S-86_Q3KQI	Q3KQU3	MAP7 domain-cc	MAP7D1 KIA	-0.5904	-0.4819	-0.3343	-0.5164	-0.2851	-0.0623	-0.3184	-0.1161	0.0723	0.1902
1413	S-860_Q96S	Q96ST3	Paired amphipat	SIN3A	-0.0278	0.1733	0.1889	0.4916	0.1724	0.3212	0.1231	0.1512	0.1462	0.0668
1414	S-861_Q9UI	Q9UMN6	Histone-lysine N-	KMT2B HRX	-0.0723	-0.0762	-0.0168	-0.1205	-0.3635	-0.0905	0.0447	-0.0785	-0.1873	-0.3056
1415	S-863_Q2KH	Q2KHT3	Protein CLEC16A	CLEC16A KIA	-3.1645	-2.9298	-2.2189	-2.1090	-2.2562	-2.1603	-2.0842	-1.9578	-2.2447	-2.0538
1416	S-864_P293	P29374	AT-rich interactiv	ARID4A RBB	0.9143	0.9238	0.9847	1.0000	0.8650	0.8196	0.9151	1.1046	0.9300	0.6343
1417	S-865_Q86Y	Q86YV0	RAS protein activ	RASAL3	0.3442	0.4139	-0.3731	-0.2041	-0.2562	-0.0858	0.3642	0.0362	-0.3109	-0.2237
1418	S-867_Q9P1	Q9P1Y6	PHD and RING fir	PHRF1 KIAA	1.6201	1.5982	1.1528	1.3829	1.0092	1.0126	1.1421	1.1159	0.9321	0.9721
1419	S-868_P783	P78332	RNA-binding pro	RBM6 DEF3	-0.1108	-0.1492	-0.2324	-0.0544	-0.1938	-0.2834	-0.1988	-0.1446	-0.1363	-0.0039
1420	S-868_Q86V	Q86VM9	Zinc finger CCCH	ZC3H18 NHN	1.9615	1.9467	2.1356	2.4683	2.2546	2.0360	2.1950	2.1404	1.9744	1.9142
1421	S-869_Q9Y2	Q9Y2K7	Lysine-specific de	KDM2A CXX	-0.2660	-0.1920	-0.3749	-0.2992	-0.5185	-0.2462	-0.3469	-0.4038	-0.4898	-0.5098
1422	S-87_P4123	P41236	Protein phospha	PPP1R2 IPP2	-2.3923	-2.6334	-3.0190	-5.0786	-3.4845	-2.9676	-2.6914	-2.6538	-2.9755	-2.4371
1423	S-87_Q1388	Q13884	Beta-1-syntrophi	SNTB1 SNT2	-0.2182	-0.2562	-0.1769	-0.5761	-0.6194	-0.3866	-0.2784	-0.3319	-0.2949	-0.6208
1424	S-87_Q8WW	Q8WW12	PEST proteolytic	PCNP	-2.3923	-1.8841	-0.2435	-0.6083	-0.0688	-1.3788	0.7041	-0.9939	-1.3013	-1.3427
1425	S-87_Q9UK7	Q9UK76	Jupiter microtub	JPT1 ARM2 I	1.1958	1.3354	0.7003	0.7039	0.5270	0.4865	0.6913	0.5193	0.3393	0.2510
1426	S-871_O149	O14974	Protein phospha	PPP1R12A M	-0.0182	0.3034	0.2970	0.1325	-0.0083	0.0810	0.0078	0.0287	0.0078	0.0703
1427	S-873_Q7Z6	Q7Z6E9	E3 ubiquitin-prot	RBBP6 P2PR	-0.4235	-0.3675	-0.9206	-0.8056	-0.8961	-0.9275	-0.8745	-0.8996	-0.8296	-0.6149
1428	S-874_Q8IYI	Q8IYB3	Serine/arginine r	SRRM1 SRM	1.1747	1.0125	0.7694	0.8777	0.7996	0.5857	0.9644	0.6148	0.6240	0.7971
1429	S-879_Q5T2	Q5T200	Zinc finger CCCH	ZC3H13 KIA	-1.6346	-1.6299	-0.1876	-0.5164	-0.4068	-0.2476	-0.4294	-0.5033	-0.0409	0.1174
1430	S-88_P0675	P06753	Tropomyosin alp	TPM3	0.2979	0.1602	0.2799	0.3931	0.1394	0.1074	0.3842	0.2141	0.2080	0.3462
1431	S-88_P3331	P33316	Deoxyuridine 5'-	DUT	0.9864	0.8348	0.9165	0.7679	0.8548	1.0037	1.0548	1.2022	1.5971	1.3401
1432	S-88_Q1315	Q13151	Heterogeneous r	HNRNPA0 H	-1.9608	-1.6858	-2.2092	-1.3282	-1.3850	-1.7469	-0.9935	-1.2716	-1.8296	-1.8732
1433	S-88_Q86UI	Q86UU0	B-cell CLL/lymph	BCL9L DLNB	0.9531	0.9337	1.0130	0.9938	1.0292	1.1362	0.9417	1.1110	1.2922	1.0734
1434	S-88_Q9NX	Q9NXG2	THUMP domain-	THUMPD1	-0.0666	-0.0359	-0.1912	-0.1459	-0.3377	-0.3108	-0.1120	0.1397	-0.0236	-0.3297
1435	S-882_O751	O75151	Lysine-specific de	PHF2 CENP-	-0.6687	-0.5451	-1.1292	-1.2082	-0.9954	-1.4542	-0.7823	-1.2496	-1.4426	-1.3657
1436	S-882_Q96T	Q96T23	Remodeling and	RSF1 HBXAP	-1.0403	-1.0370	-0.6768	-0.6806	-1.1421	-0.9134	-1.1461	-0.9054	-0.6867	-0.6589
1437	S-886_Q929	Q92974	Rho guanine nuc	ARHGEF2 KI	-2.0329	-1.3963	-1.7279	-2.2206	-1.6480	-1.6504	-1.6178	-1.5473	-1.6721	-1.5307
1438	S-886_Q9BX	Q9BXL7	Caspase recruitn	CARD11 CAF	-0.5795	-0.5116	-0.7881	-0.5814	-1.0288	-0.5381	-0.4276	-0.8085	-0.7707	-0.4284
1439	S-888_P529	P52948	Nuclear pore cor	NUP98 ADAI	0.1639	0.1492	0.7950	0.4305	0.5711	0.5539	0.4283	0.6967	0.7820	0.5259
1440	S-889_Q32P	Q32P44	Echinoderm micr	EML3	-0.7761	-0.6858	-0.9792	-1.1479	-0.9133	-1.0639	-1.0355	-0.9578	-0.8135	-0.9187
1441	S-89_P0511	P05114	Non-histone chr	HMGN1 HM	0.5765	0.4773	0.5702	0.6414	0.7405	0.7803	0.8227	0.7729	0.9273	1.0734
1442	S-89_Q9NR	Q9NR30	Nucleolar RNA h	DDX21	1.4505	1.4753	1.3575	1.4754	1.5480	1.2937	1.5618	1.3836	1.0200	1.0580
1443	S-890_Q9UI	Q9UHV7	Mediator of RNA	MED13 ARC	0.2515	0.6510	-0.1223	-0.0215	-0.2540	-0.1421	-0.1432	-0.1787	-0.1235	0.0192

1444	S-891_O150	O15027	Protein transpor	SEC16A KIAA	0.3299	0.3310	0.7670	0.6951	-0.2964	-0.1520	0.4906	0.4359	0.6939	0.8438
1445	S-891_P783	P78332	RNA-binding pro	RBM6 DEF3	0.0233	-0.3253	1.3612	1.6940	1.4690	1.5519	1.6633	1.6132	1.4709	1.4983
1446	S-893_Q86V	Q86VM9	Zinc finger CCCH	ZC3H18 NHN	0.1815	-0.0097	0.5787	0.9306	0.8225	0.4185	0.4817	0.4977	0.4033	0.4452
1447	S-9_Q9NYM	Q9NYM9	BET1-like protein	BET1L GS15	0.2424	-0.0416	0.3998	0.2523	0.2223	0.5325	0.1350	0.1563	0.3718	0.1857
1448	S-90_Q6PKG	Q6PKG0	La-related protei	LARP1 KIAA	-1.5475	-1.5827	-1.2559	-1.3689	-1.1421	-1.3905	-1.6706	-1.6395	-1.1094	-0.9783
1449	S-901_Q8IYE	Q8IYB3	Serine/arginine r	SRRM1 SRM	1.2651	1.0377	1.2923	2.3062	2.0931	1.6193	1.5745	1.9451	0.8864	1.3558
1450	S-906_P981	P98171	Rho GTPase-acti	ARHGAP4 KI	0.2409	0.2504	0.0555	0.0386	0.1073	0.3273	0.0397	-0.0278	-0.0530	-0.0168
1451	S-906_Q134	Q13428	Treacle protein (	TCOF1	-0.2652	-0.0427	-0.0588	0.1145	-0.1626	-0.0717	0.1015	0.2785	-0.4008	-0.5972
1452	S-907_P198	P19838	Nuclear factor N	NFKB1	-0.5291	-0.6984	-0.4829	-0.4275	-0.3730	-0.1553	-0.4594	-0.4130	-0.3190	-0.3104
1453	S-907_Q9P1	Q9P107	GEM-interacting	GMIP	-0.1788	-0.1248	-0.0533	-0.2863	-0.3470	-0.2601	-0.0719	-0.1079	-0.0302	-0.0498
1454	S-91_P1281	P12814	Alpha-actinin-1 (	ACTN1	-0.7214	-0.6966	-1.5245	-1.2373	-1.2344	-0.5446	-0.9204	-0.5834	-0.7707	-1.1193
1455	S-91_Q9H6F	Q9H6F5	Coiled-coil doma	CCDC86 CYC	0.5272	0.5809	-0.8008	-0.6083	-0.7342	-0.8584	-0.5122	-1.0721	-1.0276	-0.8268
1456	S-910_B5ME	B5ME19	Eukaryotic transl	EIF3CL	-0.3251	-0.0832	1.0715	1.3258	1.1968	1.0251	1.4762	1.5273	0.3898	0.5357
1457	S-913_P188	P18858	DNA ligase 1 (EC	LIG1	-1.0441	-1.2549	-0.9669	-0.9221	-1.1020	-1.1049	-1.1205	-1.2496	-0.8459	-1.0780
1458	S-914_Q9U0	Q9UQ35	Serine/arginine r	SRRM2 KIAA	1.6289	1.6318	2.3561	2.7142	2.5245	2.3825	2.7593	2.5262	2.4562	2.6475
1459	S-915_Q9P1	Q9P1Y6	PHD and RING fi	PHRF1 KIAA	-2.0628	-1.7816	-2.3569	-2.2542	-2.3946	-2.4956	-2.6258	-2.2679	-2.1493	-2.1138
1460	S-916_Q165	Q16512	Serine/threonine	PKN1 PAK1	-0.5007	-0.3949	-0.9245	-0.8118	-0.9308	-1.1973	-0.6977	-1.2316	-0.8647	-0.7276
1461	S-920_Q9BY	Q9BYX2	TBC1 domain fan	TBC1D2 PAR	-0.4504	-0.7608	-0.5565	-0.5369	-0.3123	-0.5887	-0.7557	-0.6347	-0.8600	-0.7796
1462	S-922_Q140	Q14005	Pro-interleukin-1	IL16	0.4247	0.6566	-0.2115	-0.1563	-0.0592	-0.1904	0.1800	-0.0573	-0.1806	0.0063
1463	S-923_Q6PJ	Q6PJG2	Mitotic deacetyl	MIDEAS C14	-5.4703	-5.5403	-5.9689	-4.3782	-5.1544	-5.9218	-5.8697	-5.7952	-5.2082	-4.0220
1464	S-927_Q9UK	Q9UK61	Protein TASOR (	TASOR C3or	-1.3502	-1.4022	-1.7141	-1.2584	-1.4742	-1.7926	-1.2048	-1.3552	-1.7663	-1.3657
1465	S-928_Q9Y2	Q9Y2W1	Thyroid hormone	THRAP3 BCL	-0.4018	-0.3211	-0.2710	-0.2670	-0.2387	-0.1620	-0.2706	-0.1615	-0.2217	-0.0848
1466	S-93_P3523	P35236	Tyrosine-protein	PTPN7	0.1190	-0.0505	0.7181	0.8451	0.5940	0.6923	0.5520	0.6392	0.5626	0.5336
1467	S-93_Q0966	Q09666	Neuroblast differ	AHNAK PM2	-1.6628	-1.1457	-1.2021	-1.1486	-1.8292	-1.5341	-1.4174	-1.4831	-1.9032	-2.2554
1468	S-930_P335	P33527	Multidrug resista	ABCC1 MRP	0.2801	0.6109	-0.3120	0.2848	0.1544	0.3944	0.5914	0.2973	-0.5395	-0.6327
1469	S-932_Q929	Q92974	Rho guanine nuc	ARHGEF2 KI	0.5184	0.4362	0.2414	0.5051	0.1774	0.1307	0.5181	-0.1013	-0.0065	0.1812
1470	S-936_Q9P1	Q9P1Y6	PHD and RING fi	PHRF1 KIAA	-0.4187	-0.5006	-0.1971	-0.1697	-0.2919	-0.5577	-0.5440	-0.3768	-0.2217	-0.2403
1471	S-938_O754	O75400	Pre-mRNA-proce	PRPF40A FB	0.5372	0.1954	0.7157	0.4266	0.6888	0.7121	0.4089	0.5819	0.7896	0.5655
1472	S-94_I3L1I5	I3L1I5	Rho guanine nuc	ARHGEF18 K	-1.1703	-1.2821	-1.1767	-1.0660	-1.0863	-0.8829	-1.3730	-1.2700	-1.1540	-1.1497
1473	S-94_P1858	P18583	Protein SON (Ba	SON C21orf	1.3605	1.0979	1.1233	0.9382	1.1436	0.9354	1.2725	1.2696	1.0058	0.6549
1474	S-94_Q0183	Q01831	DNA repair prote	XPC XPCC	0.4657	0.3835	0.5580	0.2587	0.6300	0.7950	0.4825	0.4801	0.5825	0.5574
1475	S-94_Q53EL	Q53EL6	Programmed cell	PDCD4 H731	2.5409	2.7684	1.9328	2.3808	2.1146	1.8192	2.4560	2.1265	1.5671	1.5299
1476	S-94_Q9NQ	Q9NQG6	Mitochondrial dy	MIEF1 MID5	-0.0761	-0.0703	-0.2572	-0.0674	-0.2717	-0.1888	-0.3039	-0.7203	-0.6252	-0.3942
1477	S-942_O607	O60763	General vesicula	USO1 VDP	-0.7516	-0.8116	-0.6753	-0.5038	-0.7123	-0.5197	-0.7136	-0.6255	-0.7455	-0.6964
1478	S-947_Q7Z3	Q7Z333	Probable helicas	SETX ALS4 K	-0.5634	-0.5861	-0.4511	-0.2352	0.0210	-0.3238	-0.1404	-0.3147	-0.3652	-0.0405
1479	S-948_P517	P51784	Ubiquitin carbox	USP11 UHX1	-1.3923	-1.3761	-1.7737	-1.5761	-1.5423	-1.8397	-1.8792	-1.3052	-1.6474	-1.5798
1480	S-95_Q96NY	Q96NY9	Crossover junctic	MUS81	-2.1890	-1.9088	-2.9127	-2.8434	-3.2562	-2.9332	-2.6914	-2.6065	-2.1493	-2.3492
1481	S-95_Q9BW	Q9BW61	DET1- and DDB1	DDA1 C19or	-2.7450	-2.7221	-2.4979	-2.1118	-2.2760	-2.5448	-1.8643	-1.8322	-2.4797	-2.5594
1482	S-952_O607	O60763	General vesicula	USO1 VDP	-0.8265	-0.8027	-0.5907	-0.8087	-0.6919	-0.4935	-1.0597	-0.9082	-0.7954	-1.0142

1483	S-953_O750	O75064	DENN domain-cc	DENND4B KI	-0.7823	-0.6699	-0.5411	-0.4299	-0.9699	-0.6457	-0.6480	-0.6708	-0.3837	-0.5251
1484	S-953_Q96C	Q96Q05	Trafficking protei	TRAPPC9 KIA	-0.5527	-0.6727	-0.5904	-0.6942	-0.8787	-0.5178	-0.5949	-0.4501	-1.1771	-1.0472
1485	S-954_Q9UC	Q9UQ35	Serine/arginine r	SRRM2 KIAA	-0.5634	-0.3533	-0.2978	0.1390	-0.2806	-0.2388	-0.4559	-0.1667	-0.0342	-0.1419
1486	S-956_Q5T1	Q5T1M5	FK506-binding pr	FKBP15 KIAA	-0.6013	-0.2106	-1.0752	-0.9947	-1.0981	-1.6044	-1.0925	-1.3669	-1.1696	-1.1998
1487	S-957_Q9UF	Q9UPU7	TBC1 domain fan	TBC1D2B KIA	1.4877	1.3472	1.6647	1.5885	1.4381	1.5013	1.4574	1.3865	1.2125	1.2020
1488	S-958_Q007	Q00722	1-phosphatidylin	PLCB2	-1.0818	-1.1677	-1.3277	-1.7210	-0.9343	-1.3944	-1.0842	-1.4029	-1.3702	-1.1166
1489	S-958_Q996	Q99683	Mitogen-activate	MAP3K5 ASK	-1.4406	-1.2261	-0.7737	-0.7687	-1.2258	-0.9618	-1.1928	-0.9197	-1.3141	-1.1910
1490	S-96_P0268(	P02686	Myelin basic prot	MBP	-0.1808	-0.2856	0.7701	0.1975	0.4670	0.1550	0.3469	0.2747	0.0611	-0.0484
1491	S-96_P5360:	P53602	Diphosphomeval	MVD MPD	-0.3274	-0.2762	-0.7384	-1.2584	-0.7465	-1.3634	-0.6059	-1.2316	-0.7909	-0.7361
1492	S-96_Q9ULL	Q9ULL5	Proline-rich prot	PRR12 KIAA1	1.0135	0.7661	1.0244	1.1005	1.1030	0.6594	1.2150	0.9303	0.9211	1.0716
1493	S-960_Q929	Q92974	Rho guanine nuc	ARHGEF2 KIA	0.0723	0.0734	-0.2032	-0.1818	-0.1854	-0.1939	-0.1884	-0.5098	-0.4586	-0.3264
1494	S-961_Q928	Q92834	X-linked retinitis	RPGR RP3 XL	-1.7761	-1.6472	-0.8970	-1.1919	-1.1060	-1.7519	-0.8093	-1.1112	-1.2981	-1.8685
1495	S-961_Q9Y4	Q9Y4E8	Ubiquitin carbox	USP15 KIAA(	-0.6601	-0.8759	-0.6585	-0.9356	-0.8522	-0.5599	-0.7125	-0.7127	-0.6454	-0.6691
1496	S-97_P6192	P61927	60S ribosomal pr	RPL37	-1.3049	-1.0347	-0.2398	0.3781	-0.0863	-0.4748	-0.0583	-0.0403	-0.0396	-0.0142
1497	S-97_Q96T3	Q96T37	RNA-binding prot	RBM15 OTT	-2.6745	-2.8007	-2.0533	-2.2627	-1.7009	-2.2565	-1.6789	-1.7077	-2.3271	-2.1249
1498	S-97_Q9C0K	Q9C0K0	B-cell lymphoma	BCL11B CTIP	0.7954	0.7504	1.2449	1.2782	1.3876	1.4302	1.2639	1.2460	1.6643	1.6804
1499	S-970_Q6PD	Q6PD62	RNA polymerase	CTR9 KIAA01	0.2681	0.6545	0.1233	0.1566	0.1936	-0.1161	-0.0569	0.1425	0.1102	0.0994
1500	S-973_P463	P46379	Large proline-ricl	BAG6 BAT3 (	-0.7334	-0.4881	0.1348	0.4305	-0.5776	-0.3350	-0.9858	-0.9758	-0.6252	-0.8109
1501	S-973_Q9P1	Q9P1Y6	PHD and RING fir	PHRF1 KIAA1	-0.2982	-0.1966	-0.6210	-0.2352	-0.4489	-0.9190	-0.6706	-0.8911	-0.6721	-0.2773
1502	S-974_Q140	Q14005	Pro-interleukin-1	IL16	-0.7547	-1.2410	1.6905	1.3277	1.7614	1.0263	1.9825	1.2737	1.2835	0.9280
1503	S-974_Q9UC	Q9UQ35	Serine/arginine r	SRRM2 KIAA	-0.2805	0.8097	0.9853	0.9391	0.8851	0.8895	0.8431	0.8822	0.9328	1.1048
1504	S-975_P085	P08575	Receptor-type ty	PTPRC CD45	0.5025	0.3208	0.0317	0.0307	-0.0806	0.2317	-0.0060	-0.0404	0.1377	0.1819
1505	S-98_Q1389	Q13895	Bystin	BYSL ENP1	0.1989	0.1432	0.1610	-0.2067	0.1495	0.1659	0.0812	0.1841	-0.5453	-0.5209
1506	S-98_Q1481	Q14814	Myocyte-specific	MEF2D	-0.1973	-0.2910	-0.2660	-0.1617	-0.2236	-0.1339	-0.2566	-0.2939	-0.2602	-0.1677
1507	S-98_Q9BSL	Q9BSL1	Ubiquitin-associa	UBAC1 GBDI	-0.4728	-0.5712	-0.3650	-0.5867	-0.1854	-0.1097	-0.4017	-0.5207	-0.4658	-0.4599
1508	S-98_Q9NS5	Q9NS56	E3 ubiquitin-prot	TOPORS LUN	1.0384	1.0393	0.8986	1.0871	1.1073	1.1436	0.8880	0.9327	0.9039	0.6968
1509	S-98_Q9UBE	Q9UBB9	Tuftelin-interacti	TFIP11 STIP	0.8192	1.0366	1.1739	0.9354	0.8436	1.0045	0.7885	0.9931	1.0505	0.7126
1510	S-981_Q8IX(	Q8IX03	Protein KIBRA (H	WWC1 KIAA	-2.1222	-1.5663	-0.0506	-0.4508	-0.2045	-0.8460	-0.1449	-0.4272	0.0962	0.5894
1511	S-983_Q083	Q08378	Golgin subfamily	GOLGA3	-0.5877	-0.6787	-0.4554	-0.4204	-0.1708	-0.2336	-0.3167	-0.3948	-0.2633	-0.2945
1512	S-985_Q994	Q99490	Arf-GAP with GTI	AGAP2 CENT	-0.7576	-0.9748	-1.0084	-1.5189	-1.1750	-1.0060	-1.4774	-1.2388	-1.1608	-1.0431
1513	S-988_Q86Y	Q86YV0	RAS protein activ	RASAL3	-0.7261	-1.0647	-0.0372	-0.2570	-0.7533	-0.2320	-0.3847	-0.3501	-0.4483	-0.4637
1514	S-989_Q5VZ	Q5VZ89	DENN domain-cc	DENND4C C9	-1.5528	-2.1188	-1.7954	-1.6863	-2.0902	-1.5252	-1.7778	-1.5880	-1.5643	-1.5344
1515	S-99_P1386	P13861	cAMP-dependen	PRKAR2A PK	-0.6183	-0.6617	-0.5277	-0.5881	-0.8121	-0.7848	-0.7982	-0.6746	-0.5567	-0.3783
1516	S-99_Q1318	Q13185	Chromobox prot	CBX3	1.9531	1.8307	1.9966	2.1382	1.9195	1.9745	1.9346	1.8835	1.4923	0.9152
1517	S-99_Q1476	Q14761	Protein tyrosine	PTPRCAP LP/	0.5594	1.1046	0.2191	0.2950	-0.2475	-0.1990	0.1409	0.0000	-0.1221	0.0752
1518	S-99_Q5D1E	Q5D1E8	Endoribonucleas	ZC3H12A M(	-0.2224	-0.2030	-0.3677	-0.4762	-0.3423	-0.7444	-0.5516	-0.4171	-0.4338	-0.4885
1519	S-99_Q5TAC	Q5TAQ9	DDB1- and CUL4	DCAF8 H326	-1.8137	-1.2496	-2.2287	-2.0197	-2.1139	-2.6272	-2.1988	-2.4232	-3.1725	-3.7043
1520	S-991_Q9P1	Q9P1Y6	PHD and RING fir	PHRF1 KIAA1	-0.1767	-0.0588	-0.8970	-0.3968	-0.3493	-0.8802	-0.1884	-0.5361	-1.0329	-0.7885
1521	S-993_Q6W(	Q6WCQ1	Myosin phospha	MPRIP KIAA(	0.6486	0.4435	0.8640	0.6207	0.6934	0.6929	0.4860	0.5320	0.3382	0.2270

1522	S-994_Q86Y	Q86YV0	RAS protein activ	RASAL3	1.0919	1.1020	1.4257	1.4312	1.2908	1.6356	1.4312	1.6222	1.0574	1.0337
1523	S-994_Q9UF	Q9UPS6	Histone-lysine N-	SETD1B KIAA	-1.3595	-1.0100	-0.8100	-0.3712	-0.8064	-0.7173	-0.9082	-1.2790	-0.8577	-0.6711
1524	S;S-100|107	Q86YP4	Transcriptional re	GATAD2A	-1.0403	-0.9682	-0.9486	-1.0599	-1.1100	-0.8370	-1.3039	-1.4946	-1.0571	-1.0511
1525	S;S-1012|10	O95785	Protein Wiz (Wid	WIZ ZNF803	-0.6459	-0.8759	-0.8509	-0.9187	-0.9520	-1.1905	-0.8792	-0.8940	-1.1902	-1.0834
1526	S;S-1017|10	Q7Z333	Probable helicase	SETX ALS4 K	-0.5187	-0.7110	-0.1223	-0.2185	-0.4264	-0.1081	-0.5196	-0.5540	-0.2385	-0.1822
1527	S;S-102|103	P17096	High mobility gro	HMGA1 HM	0.2861	0.1740	0.3390	0.1390	0.4442	0.4769	0.4137	0.2022	-0.0196	0.3155
1528	S;S-102|105	P05387	60S acidic riboso	RPLP2 D11S	1.1950	1.1347	0.6444	0.4226	0.8880	0.9283	0.9226	1.0613	0.9644	0.9655
1529	S;S-1023|10	Q01804	OTU domain-con	OTUD4 HIN-	0.9438	0.8697	1.1608	1.0334	0.9360	1.0803	0.9784	1.0788	0.9314	0.7883
1530	S;S-103|105	Q14839	Chromodomain-l	CHD4	0.4258	0.3467	0.8454	0.3712	0.3960	0.4169	0.3772	0.2620	0.6440	0.8517
1531	S;S-103|106	O60231	Pre-mRNA-splicir	DHX16 DBP2	-0.0876	-0.0634	-0.0031	-0.1899	-0.3192	-0.0670	-0.2959	-0.2679	0.0511	0.1925
1532	S;S-104|106	O43159	Ribosomal RNA-p	RRP8 KIAA0	0.7755	0.5227	1.2742	1.4246	1.2969	1.2406	1.3535	1.3152	1.1216	1.3881
1533	S;S-108|110	Q8WUQ7	Cactin (Renal car	CACTIN C19	-0.3116	-0.1802	-0.0479	0.2657	0.0246	-0.2816	-0.2123	-0.0169	-0.1536	-0.0378
1534	S;S-1085|10	O14647	Chromodomain-l	CHD2	-0.0422	-0.1578	0.5425	0.5616	0.3649	0.1389	1.0961	0.5819	-0.1464	-0.0862
1535	S;S-109|111	Q56P03	E2F-associated p	EAPP C14orf	-1.0780	-1.4711	0.7553	0.3449	0.4100	0.7524	-0.1233	0.4505	0.9396	0.9727
1536	S;S-109|111	Q86XP3	ATP-dependent R	DDX42	0.1130	-0.0011	-0.4626	-0.5787	-0.2606	-0.2744	-0.2911	-0.5585	-0.3485	-0.3378
1537	S;S-11|13_C	Q92685	Dol-P-Man:Man(	ALG3 NOT N	-1.6459	-1.7038	-1.1663	-0.6891	-0.7652	-0.7370	-0.7210	-0.7228	-1.3370	-1.0458
1538	S;S-112|115	Q9P287	BRCA2 and CDKN	BCCIP TOK1	-2.6977	-2.6823	-2.8434	-2.4252	-2.6365	-3.0764	-2.9884	-2.8274	-2.7057	-2.8544
1539	S;S-1122|11	Q9UQ35	Serine/arginine r	SRRM2 KIAA	-0.9892	-0.6524	-1.2710	-1.2123	-0.9991	-1.5731	-1.1605	-1.2352	-1.2293	-1.1504
1540	S;S-115|119	Q7Z309	Protein FAM122I	FAM122B	0.6312	0.4749	1.0680	1.1858	0.7025	0.8831	0.8489	0.9668	1.0429	0.9707
1541	S;S-116|118	P55081	Microfibrillar-ass	MFAP1	0.3154	0.2260	-0.2348	-0.1818	-0.0844	-0.3201	-0.2612	-0.3788	-0.3223	-0.2633
1542	S;S-116|120	Q6JBY9	CapZ-interacting	RCSD1 CAPZ	0.1146	0.0066	0.3652	0.1438	0.0867	0.1033	-0.0091	0.0555	0.0536	-0.1124
1543	S;S-1161|11	P46100	Transcriptional re	ATRX RAD54	0.1345	0.3007	0.2686	0.9694	0.6377	1.1348	0.9013	0.9808	0.5342	0.8229
1544	S;S-121|122	P41236	Protein phospha	PPP1R2 IPP2	0.4953	0.5272	0.8854	0.8182	0.7279	1.0257	0.5081	0.7242	1.0019	0.6110
1545	S;S-121|124	Q9Y5J1	U3 small nucleol	UTP18 WDR	0.2644	0.4428	0.6909	0.9691	0.5256	0.6808	0.6523	0.4513	0.3392	0.5406
1546	S;S-125|133	Q6NYC8	Phostensin (Prot	PPP1R18 HK	-0.5037	-0.3311	0.7194	0.0714	0.1461	0.3418	0.1418	0.2448	0.4885	0.2426
1547	S;S-126|129	Q96BR1	Serine/threonine	SGK3 CISK S	1.2470	1.1794	0.6350	1.0796	1.0814	0.7184	1.4010	0.7845	0.4007	0.6888
1548	S;S-1268|12	Q96T58	Msx2-interacting	SPEN KIAA0	0.0215	0.1532	0.3139	0.4106	0.4238	0.3022	0.5007	0.4727	0.3258	0.2724
1549	S;S-1281|12	Q8WWQ0	PH-interacting pr	PHIP DCAF1	0.5234	0.7312	0.2379	0.2114	0.0657	-0.0842	0.2222	-0.3377	-0.0026	0.0543
1550	S;S-1306|13	Q14160	Protein scribble I	SCRIB CRIB1	-0.9398	-0.7664	-0.3068	-0.7447	-0.4691	-0.4706	-0.5960	-0.2424	-0.8022	-0.6814
1551	S;S-131|137	O60341	Lysine-specific hi	KDM1A AOF	-0.4480	-0.3183	-1.3382	-1.2542	-1.1020	-1.3257	-1.0705	-1.1479	-1.3109	-1.2479
1552	S;S-131|138	O75446	Histone deacetyl	SAP30	-2.3093	-1.9510	-1.9048	-1.7932	-2.3850	-2.2565	-2.0569	-2.2679	-3.2324	-3.3624
1553	S;S-1315|13	Q6F5E8	Capping protein,	CARMIL2 LR	-0.8988	-0.6334	-0.7809	-0.9459	-0.5831	-0.5490	-0.7890	-0.6395	-0.5974	-0.3841
1554	S;S-132|133	P51858	Hepatoma-derive	HDGF HMG1	0.9726	1.2740	0.7144	0.9223	0.7977	1.0957	0.6069	0.9769	1.0410	0.8403
1555	S;S-134|143	Q8NDX1	PH and SEC7 dom	PSD4 EFA6B	1.3015	1.2826	1.1253	1.0880	0.7817	1.1442	1.1214	1.1713	0.4895	0.3792
1556	S;S-1348|13	P46100	Transcriptional re	ATRX RAD54	1.3527	1.6007	1.5804	1.7669	1.5804	1.6390	1.6322	1.5556	1.5516	1.4795
1557	S;S-135|138	Q15388	Mitochondrial im	TOMM20 KI	-1.9892	-1.9214	-0.0063	0.0036	0.7850	0.9707	0.7855	0.7774	0.1494	-0.0391
1558	S;S-136|138	Q9HAZ1	Dual specificity p	CLK4	-0.0311	0.1944	-0.1453	-0.0036	-0.0902	-0.0515	0.1941	0.0730	-0.0789	-0.1764
1559	S;S-1369|13	O14647	Chromodomain-l	CHD2	0.0995	0.1462	0.1538	0.2478	0.2064	0.1841	0.1148	0.1075	0.2337	0.2048
1560	S;S-14|15_C	Q12846	Syntaxin-4 (Rena	STX4 STX4A	0.4696	0.5002	1.0275	1.1590	1.1260	0.8927	1.0315	1.1881	0.7035	1.1554

1561	S;S-142|144	Q13523	Serine/threonine	PRPF4B KIAA	0.2132	0.1698	0.1495	0.1474	0.1361	0.0796	-0.0488	0.0014	0.2696	0.3274
1562	S;S-143|147	Q96E09	Protein FAM122A	FAM122A C9	-0.1625	-0.6334	0.6769	0.7288	0.3960	1.0612	0.4673	0.7836	0.8779	0.9063
1563	S;S-1432|14	Q7Z591	Microtubule orga	AKNA KIAA1	-0.8622	-0.9109	-0.7612	-0.7901	-1.6251	-0.7620	-0.5459	-0.5698	-1.6680	-1.2882
1564	S;S-1439|14	Q9UQ35	Serine/arginine r	SRRM2 KIAA	-1.1089	-0.8902	-0.3867	-0.3507	-0.3353	-0.5167	-0.2243	-0.3338	-0.2885	-0.2297
1565	S;S-145|148	Q86VQ1	Glucocorticoid-ir	GLCCI1	-0.5634	-0.9361	0.3013	0.5962	0.1692	0.1061	0.5662	0.3377	-0.1947	-0.3152
1566	S;S-145|153	P19338	Nucleolin (Protei	NCL	1.1739	1.1611	-0.0825	-0.1177	0.2932	0.0606	0.4146	0.1195	-0.0047	-0.0230
1567	S;S-1461|14	Q02880	DNA topoisomer	TOP2B	-0.3206	-0.2575	-0.8210	-0.7269	-0.7281	-1.0795	-0.9477	-0.5585	-0.7227	-0.5817
1568	S;S-1462|14	Q9UQ35	Serine/arginine r	SRRM2 KIAA	0.6195	0.5010	0.1900	-0.0730	0.7205	0.5013	0.5618	0.4815	1.0341	1.3896
1569	S;S-148|149	Q9Y388	RNA-binding mot	RBMX2 CGI-	0.1525	0.0713	0.0311	0.2221	0.1243	0.4971	0.0104	0.0137	0.2665	0.1042
1570	S;S-148|151	Q15185	Prostaglandin E s	PTGES3 P23	-0.9157	-1.2575	-2.4340	-3.3598	-2.2044	-2.7222	-1.6419	-2.2244	-2.4930	-2.4371
1571	S;S-15|17_A	A2AJT9	BCLAF1 and THR	BCLAF3 CXor	0.5247	0.6167	0.7201	0.6962	0.5748	0.5138	0.6221	0.6112	0.6052	0.4572
1572	S;S-15|17_C	Q9NZ63	Telomere length	C9orf78 HCA	-1.0628	-1.3448	-1.2019	-0.9493	-1.0746	-0.9388	-1.0039	-0.9431	-0.8624	-1.0780
1573	S;S-1512|15	P51531	Probable global t	SMARCA2 BA	0.1329	0.2826	0.1734	0.2993	0.3820	0.5149	0.0796	0.1928	0.1750	-0.0551
1574	S;S-1514|15	Q8NI27	THO complex sub	THOC2 CXor	1.3255	1.3782	1.6293	1.4792	1.5071	1.5417	1.3732	1.4951	1.7733	1.4749
1575	S;S-1517|15	Q9UQ35	Serine/arginine r	SRRM2 KIAA	-0.1706	0.0490	0.9762	0.7039	0.6424	0.4683	0.7496	0.7042	0.6737	0.9335
1576	S;S-1537|15	O43149	Zinc finger ZZ-typ	ZZEF1 KIAA0	-1.6123	-1.8241	-1.1223	-1.4688	-1.0824	-1.0240	-0.5122	-0.6854	-1.5798	-1.5381
1577	S;S-154|156	Q9BTC0	Death-inducer ob	DIDO1 C20o	0.1879	0.3432	0.6977	0.5629	0.6797	0.5221	0.3752	0.5011	1.1078	1.1508
1578	S;S-1541|15	Q9UQ35	Serine/arginine r	SRRM2 KIAA	1.1113	1.2279	1.0326	1.0947	1.5283	1.0119	1.3837	1.0302	1.4424	1.6663
1579	S;S-155|157	Q15287	RNA-binding pro	RNPS1 LDC2	1.2971	0.9109	1.6945	1.9015	2.1688	1.3705	2.5599	1.6468	1.5662	2.2684
1580	S;S-156|157	P28715	DNA repair prote	ERCC5 ERCM	0.0993	0.1191	0.1655	0.1109	0.1279	0.1705	-0.1420	0.1746	0.1880	0.3378
1581	S;S-156|159	Q7Z5K2	Wings apart-like	WAPL FOE K	0.3140	0.5668	0.1272	0.4463	0.0762	0.1590	0.2574	0.2722	0.0334	0.1732
1582	S;S-1561|15	Q9UQ35	Serine/arginine r	SRRM2 KIAA	-0.0719	-0.0041	0.4929	0.6231	0.8040	0.7607	0.8141	0.7903	0.8346	0.9719
1583	S;S-1568|15	P51531	Probable global t	SMARCA2 BA	1.4780	1.9346	0.9958	0.3971	0.9465	0.7030	1.0359	0.5299	0.8075	0.6467
1584	S;S-157|159	Q92733	Proline-rich prot	PRCC TPRC	1.4258	1.5753	1.2037	1.3294	1.0328	1.4236	1.1816	1.3841	1.4285	0.9994
1585	S;S-157|159	Q96ST2	Protein IWS1 hor	IWS1 IWS1L	0.5958	0.5675	0.5926	0.3904	0.2612	0.3728	0.2779	0.4946	0.6842	0.5250
1586	S;S-1570|15	P51532	Transcription act	SMARCA4 BA	0.7294	0.6124	0.1973	0.5520	0.4442	0.5978	0.6009	0.2233	-0.1321	-0.0339
1587	S;S-159|160	Q9BW71	HIRA-interacting	HIRIP3	0.9230	0.9045	1.0135	0.9354	0.8821	0.8579	0.8689	0.9463	1.1376	0.9402
1588	S;S-160|166	Q8N108	Mesoderm induc	MIER1 KIAA1	0.2949	0.2662	0.3838	0.3958	0.2566	0.4247	0.3961	0.4760	0.5070	0.5811
1589	S;S-1601|16	Q12873	Chromodomain-l	CHD3	0.6136	0.5297	0.2028	0.2191	0.3678	0.5478	0.5785	0.6301	0.6306	0.7181
1590	S;S-161|168	Q1KMD3	Heterogeneous r	HNRNPUL2 I	1.1958	1.8551	1.2405	0.9955	0.8831	1.1157	1.2443	1.0144	1.2220	1.0192
1591	S;S-1620|16	Q9UQ35	Serine/arginine r	SRRM2 KIAA	0.5297	0.6844	0.9832	1.0124	1.2278	1.1643	1.0771	1.0925	1.3576	1.4302
1592	S;S-1627|16	P51532	Transcription act	SMARCA4 BA	1.9177	2.2744	1.0037	0.7928	1.1494	0.9646	1.2827	0.8521	0.8319	0.8567
1593	S;S-167|168	P32519	ETS-related trans	ELF1	0.9745	0.6975	2.0262	1.9617	1.7580	2.0615	1.8329	2.0547	2.0458	2.0242
1594	S;S-17|19_P	P53999	Activated RNA po	SUB1 PC4 RF	0.9754	1.1920	1.1001	1.3144	0.8436	1.3285	0.7817	1.3195	1.2943	1.3014
1595	S;S-170|172	Q9NYV6	RNA polymerase	RRN3 TIFIA	0.0392	-0.0785	-0.4060	-0.2885	-0.6801	-0.7394	-0.8792	-0.6586	-0.3652	-0.6090
1596	S;S-1721|17	Q14980	Nuclear mitotic a	NUMA1 NM	-0.9964	-0.9489	-1.0021	-1.0599	-0.6223	-0.7246	-0.9527	-0.7741	-0.5077	-0.8970
1597	S;S-1727|17	Q9UQ35	Serine/arginine r	SRRM2 KIAA	0.9009	0.8655	0.7403	0.9110	0.9350	0.5978	0.7419	0.5890	0.6239	0.9131
1598	S;S-173|175	Q9NPG3	Ubinuclein-1 (HIF	UBN1	1.1926	1.3133	0.9932	0.9929	0.9465	0.9400	0.9981	0.8880	0.7948	0.7141
1599	S;S-1731|17	Q9UQ35	Serine/arginine r	SRRM2 KIAA	1.7954	1.8329	1.7531	1.9139	2.2309	2.1321	2.0049	2.1797	2.3725	2.4684

1600	S;S-174|175	Q7Z4V5	Hepatoma-deriv	HDGFL2 HD(	0.7162	0.6254	0.7315	0.8030	0.6530	0.7382	0.4147	0.6232	0.6001	0.6176
1601	S;S-176|180	Q9Y4C8	Probable RNA-bi	RBM19 KIAA	-0.7485	-0.8085	-0.5688	-0.7123	-0.6451	-0.5274	-0.9180	-0.8005	-0.1565	-0.0471
1602	S;S-1762|17	Q9UQ35	Serine/arginine r	SRRM2 KIAA	0.9051	0.3819	0.9729	1.1693	1.2399	0.9762	0.8968	1.2254	0.9764	1.1207
1603	S;S-181|183	Q16629	Serine/arginine-r	SRSF7 SFRS7	-0.2330	0.3580	0.3038	0.0142	0.1428	0.0324	0.3097	-0.0092	0.1102	0.3289
1604	S;S-181|183	Q96B97	SH3 domain-cont	SH3KBP1 CIN	0.9503	0.8397	0.6578	0.7093	0.4415	0.8644	0.5144	0.7576	0.4482	0.3113
1605	S;S-182|183	Q8ND56	Protein LSM14 h	LSM14A C19	0.6289	0.5949	0.9597	0.9576	0.9815	1.0389	1.1070	1.2703	0.6786	0.5169
1606	S;S-182|185	Q8NF99	Zinc finger protei	ZNF397 ZNF	0.6405	0.9003	0.7378	0.7147	0.6114	0.8710	0.5957	0.8039	0.9409	0.8503
1607	S;S-185|188	Q1KMD3	Heterogeneous r	HNRNPUL2 I	-0.5007	-0.7256	-0.0275	0.4528	0.1494	0.2113	0.0181	0.2428	-0.1080	-0.0794
1608	S;S-1866|18	Q9UQ35	Serine/arginine r	SRRM2 KIAA	-2.3641	-1.9130	-1.4171	-1.1479	-1.3192	-1.9162	-1.0144	-1.2138	-1.5003	-1.2510
1609	S;S-189|191	Q01130	Serine/arginine-r	SRSF2 SFRS2	1.4890	1.8286	0.4179	1.0515	1.0892	0.1984	1.3556	0.6399	0.7075	1.1078
1610	S;S-190|191	Q9UNF1	Melanoma-assoc	MAGED2 BC	-1.3274	-0.8985	-0.3731	-0.4936	-0.4564	-0.3788	-0.8070	-0.5098	-0.1769	0.0064
1611	S;S-190|193	P22059	Oxysterol-bindin	OSBP OSBP1	0.6041	0.4837	0.7787	0.5398	0.5219	0.5700	0.3911	0.5727	0.6289	0.7868
1612	S;S-193|194	Q8NAV1	Pre-mRNA-splicir	PRPF38A	0.1079	0.1412	-0.5782	-0.7299	-0.1812	-0.7051	-0.3023	-0.6347	-0.3125	-0.4061
1613	S;S-194|196	Q16629	Serine/arginine-r	SRSF7 SFRS7	1.0165	1.1169	0.9700	1.0581	0.7422	0.5637	1.0053	0.9463	1.2468	1.2391
1614	S;S-196|199	Q9BW71	HIRA-interacting	HIRIP3	-0.4430	-0.4051	-0.2385	0.0863	-0.2387	-0.0210	-0.1678	-0.0247	-0.3109	-0.2648
1615	S;S-20|23_C	Q13523	Serine/threonine	PRPF4B KIA	0.2105	0.2026	-0.6019	-0.6942	-0.6868	-0.6861	-0.6666	-0.7296	-0.5984	-0.4689
1616	S;S-2009|20	P18583	Protein SON (Ba	SON C21orf	0.2052	-0.0461	0.5374	-0.0071	0.6600	0.1198	-0.0026	0.0437	1.0822	1.0412
1617	S;S-202|213	Q92882	Osteoclast-stimu	OSTF1	-2.2224	-2.5274	-1.8547	-1.8434	-1.9991	-1.9050	-1.9033	-1.8166	-2.1784	-2.6387
1618	S;S-2029|20	P18583	Protein SON (Ba	SON C21orf	-0.0553	-0.0167	0.1069	-0.0599	0.1124	-0.2093	-0.0222	-0.2049	0.0549	0.0280
1619	S;S-204|206	Q9H5J8	TATA box-bindin	TAF1D JOSD	-0.8954	-0.8458	-0.9689	-0.6440	-0.7434	-0.8186	-1.0170	-0.7899	-0.4146	-0.4353
1620	S;S-204|214	Q96JM3	Chromosome ali	CHAMP1 C1	0.5284	0.4476	0.7265	0.8492	0.6739	0.7890	0.4408	0.4427	0.4755	0.4412
1621	S;S-2044|20	Q9UQ35	Serine/arginine r	SRRM2 KIAA	-0.0072	-0.0336	0.3546	0.1598	0.2871	-0.0195	0.2725	-0.1178	0.6137	0.7444
1622	S;S-206|209	Q96JY6	PDZ and LIM dor	PDLIM2 PP6	-1.2266	-0.9726	-0.3853	-0.6329	-0.1381	-0.4624	-0.4865	-0.2120	-1.0356	-0.8154
1623	S;S-209|213	Q6NYC8	Phostensin (Prot	PPP1R18 HK	-0.1344	0.0382	0.2816	0.9072	0.2795	0.5978	0.6555	0.5593	-0.3223	-0.0273
1624	S;S-210|216	Q09666	Neuroblast differ	AHNAK PM2	-0.3138	-0.3732	-0.8642	-0.7718	-1.0942	-1.1973	-0.8555	-1.1046	-1.8483	-1.5195
1625	S;S-210|216	Q8TBF4	Zinc finger CCHC	ZCRB1	-0.8105	-0.9857	-0.9567	-1.2755	-1.0326	-0.9247	-0.9578	-1.0657	-0.8647	-1.3993
1626	S;S-211|213	Q9BW85	Splicing factor YJ	YJU2 CCDC9	0.9115	0.7707	0.6756	0.7352	0.6877	0.5710	0.5662	0.5161	0.7684	0.7831
1627	S;S-211|216	Q13242	Serine/arginine-r	SRSF9 SFRS9	-0.8886	-0.7716	-1.2572	-1.1963	-1.2629	-1.4463	-1.0915	-1.6733	-1.5396	-1.3333
1628	S;S-2133|21	O75643	U5 small nuclear	SNRNP200 A	1.4500	1.4160	1.0843	1.0465	0.6728	1.0731	0.7864	0.9891	0.8762	0.7139
1629	S;S-215|217	Q16629	Serine/arginine-r	SRSF7 SFRS7	1.0714	1.1769	1.2974	1.7474	1.7272	1.2437	1.9588	1.5951	1.8765	1.9486
1630	S;S-215|220	Q96PK6	RNA-binding pro	RBM14 SIP	-0.8556	-0.7627	-0.1476	0.0212	0.1209	0.0015	0.3842	-0.0046	-0.3652	0.0115
1631	S;S-2152|21	P21333	Filamin-A (FLN-A	FLNA FLN FL	1.0550	1.0607	1.2664	1.2516	1.0101	1.3449	1.2939	1.4234	0.6704	0.4933
1632	S;S-217|220	Q96F15	GTPase IMAP fan	GIMAP5 IAN	-0.3711	-0.3833	-0.2685	-0.4228	-0.3238	-0.1323	-0.0897	-0.0450	-0.7575	-0.8452
1633	S;S-22|26_P	P52824	Diacylglycerol kir	DGKQ DAGK	0.4028	0.4022	0.3942	0.5879	-0.0844	-0.1145	0.0257	0.2658	-0.0052	-0.0155
1634	S;S-222|226	Q9BRD0	BUD13 homolog	BUD13	-0.3387	-0.6129	0.2028	0.1866	0.1141	0.2506	0.0257	0.0628	0.2248	0.4841
1635	S;S-223|234	Q9H1E3	Nuclear ubiquito	NUCKS1 NU	0.2816	0.2514	0.3902	0.1819	0.1192	0.1061	-0.0382	0.0091	0.5414	0.8359
1636	S;S-224|232	Q13573	SNW domain-cor	SNW1 SKIIP	-0.1050	-0.1865	0.1367	0.2508	0.2856	-0.0301	0.2574	0.0569	0.2215	0.2915
1637	S;S-225|227	Q16629	Serine/arginine-r	SRSF7 SFRS7	-0.6302	-0.5171	1.0894	1.2429	1.1056	1.0781	1.3734	1.4201	1.5631	1.8199
1638	S;S-225|228	Q92844	TRAF family mer	TANK ITRAF	1.0018	1.0372	1.3440	1.1788	0.8041	1.0971	0.6530	0.9589	0.8406	0.4307

1639	S;S-228|231	Q86YV0	RAS protein activ	RASAL3	-0.0991	-0.2549	-0.5095	-0.5113	-0.9520	-0.6574	-0.3611	-0.7637	-0.6742	-0.4795
1640	S;S-229|234	Q9H1E3	Nuclear ubiquito	NUCKS1 NU(	-0.2372	-0.2106	0.2476	0.1422	-0.2964	-0.0670	-0.4720	0.0889	0.4867	0.5620
1641	S;S-23|25_C	Q92619	Rho GTPase-activ	ARHGAP45 H	0.5633	0.7727	-0.2345	-0.2052	-0.0918	-0.5160	-0.1662	-0.5348	-0.4363	-0.1815
1642	S;S-232|234	Q9NYF3	Protein FAM53C	FAM53C C5c	-0.5715	-0.9510	-0.7935	-0.5012	-0.8423	-0.8638	-0.6399	-1.0915	-0.9129	-0.7534
1643	S;S-233|242	Q9Y6M7	Sodium bicarbon	SLC4A7 BT N	1.3484	1.1031	1.1780	1.7325	1.1201	1.1328	1.3159	1.0399	0.6777	0.7793
1644	S;S-234|240	Q9H1E3	Nuclear ubiquito	NUCKS1 NU(	0.2694	0.1776	0.3672	0.5183	0.0434	-0.0368	-0.1518	-0.0630	0.3673	0.6371
1645	S;S-236|243	Q13469	Nuclear factor of	NFATC2 NFA	1.2912	1.2726	0.7920	1.1874	0.7892	0.6771	1.0912	0.8230	0.5243	0.5725
1646	S;S-238|240	Q8NHQ1	Centrosomal pro	CEP70 BITE	0.1671	0.0901	0.5518	0.4025	0.2931	0.5700	0.2811	0.2008	0.5061	0.3175
1647	S;S-24|26_C	Q92466	DNA damage-bin	DDB2	1.9419	1.8015	1.5432	1.8253	1.7769	1.6139	2.2782	1.8647	1.3485	1.2336
1648	S;S-24|27_C	Q15435	Protein phospha	PPP1R7 SDS:	0.8725	1.1089	0.9422	1.0976	0.7991	0.7124	1.2287	0.9407	0.7182	0.6322
1649	S;S-248|253	Q9Y2W1	Thyroid hormone	THRAP3 BCL	0.2796	0.3140	0.3270	0.3231	0.5313	0.4976	0.6354	0.4848	0.3301	0.4402
1650	S;S-249|252	P0C1Z6	TCF3 fusion partı	TFPT INO80I	-3.2309	-3.3818	-4.4284	-3.8562	-4.8064	-5.1145	-3.8509	-4.8826	-4.1378	-3.7383
1651	S;S-251|253	O75494	Serine/arginine-r	SRSF10 FUSI	0.8979	1.3502	0.9148	1.0821	1.1013	0.4939	1.2843	0.7403	1.1558	1.3537
1652	S;S-253|256	O75494	Serine/arginine-r	SRSF10 FUSI	1.4670	1.6848	1.1025	1.1936	1.3656	0.8991	1.5199	1.3498	1.1628	1.0537
1653	S;S-255|259	P17535	Transcription fac	JUND	-0.1916	-0.3230	-0.1577	0.0224	-0.0956	0.1946	-0.0489	0.2172	-0.6323	-0.3493
1654	S;S-255|261	P08238	Heat shock prote	HSP90AB1 H	1.0953	1.5335	1.2285	1.1292	1.3728	1.1550	1.6043	1.1597	1.2203	1.2862
1655	S;S-258|260	Q86VR2	Reticulophagy re	RETREG3 FA	-0.2077	-0.2602	-0.3330	-0.6384	-0.8357	-0.7569	-0.6338	-0.7534	-0.9302	-0.8874
1656	S;S-258|261	Q13422	DNA-binding pro	IKZF1 IK1 IK/	-0.6516	-0.6163	-1.0447	-0.8118	-0.9627	-0.8747	-0.7361	-0.9818	-0.8296	-0.7318
1657	S;S-263|265	Q9NPI1	Bromodomain-co	BRD7 BP75 (	0.1079	0.2031	0.2885	0.3643	0.3404	0.2518	0.2768	0.1188	0.6663	0.4240
1658	S;S-266|271	Q6NY19	KN motif and anl	KANK3 ANKI	-0.0145	0.1392	0.1566	0.3971	0.0083	-0.1356	0.2077	-0.0419	-0.0598	0.0898
1659	S;S-27|31_C	O00418	Eukaryotic elong	EEF2K	-0.3735	-0.4302	-0.5006	-0.5814	-0.7069	-0.4956	-1.5029	-1.3988	-0.3223	-0.2479
1660	S;S-2702|27	Q9UQ35	Serine/arginine r	SRRM2 KIAA	1.0550	1.1949	0.5127	0.8243	0.9009	0.5128	1.1706	0.8187	0.6298	0.8624
1661	S;S-273|275	O75475	PC4 and SFRS1-ir	PSIP1 DFS70	-0.4254	-0.3894	-0.3339	-0.1576	-0.1320	-0.1604	-0.3060	-0.1428	-0.2119	-0.2070
1662	S;S-274|280	Q6KC79	Nipped-B-like pr	NIPBL IDN3 :	-0.2140	-0.2119	-0.0986	-0.0053	-0.1421	-0.0546	-0.2628	-0.1378	-0.1638	-0.2237
1663	S;S-276|278	Q8N5A5	Zinc finger CCCH-	ZGPAT GPAT	-2.1973	-2.6894	-2.3042	-2.8822	-2.4640	-2.2852	-2.3628	-2.8826	-2.3271	-2.5914
1664	S;S-277|279	Q13428	Treacle protein ('	TCOF1	-1.0000	-1.2815	-1.0974	-0.7597	-0.9772	-1.2179	-1.3330	-0.7978	-1.5759	-1.1419
1665	S;S-279|281	Q9UQ35	Serine/arginine r	SRRM2 KIAA	-2.1011	-2.2081	-2.0708	-1.4688	-1.1462	-1.5040	-1.0302	-1.6978	-1.9806	-2.1764
1666	S;S-28|34_P	P19338	Nucleolin (Protei	NCL	-0.5420	-0.5847	0.1263	-0.7540	0.0156	-0.5163	-0.0156	-0.2416	0.2954	0.1135
1667	S;S-281|287	Q2YD98	UV-stimulated sc	UVSSA KIAA:	-0.6318	-0.6770	-0.6098	-0.6164	-0.6683	-0.4461	-0.8276	-0.9025	-0.8296	-0.9936
1668	S;S-282|286	Q96JM3	Chromosome alig	CHAMP1 C1:	-0.3805	-0.1640	-0.5305	-0.6636	-0.3802	-0.5124	-0.4120	-0.6088	-0.2401	-0.3558
1669	S;S-29|31_C	Q9BVJ6	U3 small nucleol	UTP14A SDC	0.6171	0.9139	0.2677	0.2672	0.5309	0.4434	0.5181	0.2620	0.4680	0.4421
1670	S;S-29|34_C	Q9HCD5	Nuclear receptor	NCOA5 KIAA	0.9325	0.9766	1.3085	1.3560	1.5032	1.1984	1.4998	1.3605	1.2220	1.4707
1671	S;S-292|294	Q13523	Serine/threonine	PRPF4B KIA/	-1.2309	-1.4110	-2.5035	-1.8757	-2.2919	-2.6834	-1.9229	-2.0689	-2.4497	-2.6387
1672	S;S-295|297	Q9UQ35	Serine/arginine r	SRRM2 KIAA	0.8843	1.0436	0.7658	0.7815	0.6728	0.8677	0.8481	0.7458	0.7476	0.7274
1673	S;S-296|298	O00499	Myc box-depend	BIN1 AMPHI	0.4774	0.4139	-0.1337	-0.0711	0.0391	0.0583	0.4235	0.1397	0.0372	-0.2102
1674	S;S-299|307	Q14676	Mediator of DNA	MDC1 KIAA(	-0.4753	-0.4834	-0.1698	-0.4396	-0.2065	-0.3596	-0.4000	-0.5450	-0.4129	-0.2312
1675	S;S-30|32_C	Q7L4I2	Arginine/serine-r	RSRC2	1.4532	0.8428	1.6033	1.8263	1.9796	1.8579	1.8657	2.0329	1.5152	1.5850
1676	S;S-30|32_C	Q9Y2V2	Calcium-regulate	CARHSP1	0.6578	0.6892	-0.1360	-0.2499	-0.0345	-0.1973	-0.1078	-0.4314	-0.1094	-0.1376
1677	S;S-301|303	Q13247	Serine/arginine-r	SRSF6 SFRS6	-0.2471	0.0355	-0.1982	-0.0099	0.2868	-0.0704	-0.0382	-0.0491	0.1175	0.2318

1678	S;S-301|303	Q9NYV4	Cyclin-dependen	CDK12 CRK7	0.5716	0.4378	0.2299	0.6841	0.7525	0.3154	0.7450	0.6409	0.1471	0.1675
1679	S;S-302|303	Q13435	Splicing factor 3E	SF3B2 SAP14	-0.8475	-0.8508	-0.3847	-0.8309	-0.6859	-0.8933	-0.9183	-0.9173	-0.4807	-0.4763
1680	S;S-302|304	Q8TAQ2	SWI/SNF comple	SMARCC2 BA	1.6800	1.6135	1.3030	1.6038	1.5859	1.3670	1.4128	1.5134	1.4080	1.5223
1681	S;S-304|307	P05388	60S acidic riboso	RPLP0	-3.1325	-2.6611	-4.0190	-4.7567	-4.4845	-3.3827	-3.6419	-4.7952	-4.0927	-3.8638
1682	S;S-306|307	P35659	Protein DEK	DEK	-0.4114	-0.2721	-0.6002	-0.5037	-0.7220	-0.5977	-0.5233	-0.7354	-0.7120	-0.3674
1683	S;S-309|311	Q96SB4	SRSF protein kina	SRPK1	1.3731	1.3930	1.6772	1.4760	1.6808	1.6380	1.6747	1.7399	1.6688	1.1992
1684	S;S-312|314	Q13769	THO complex sub	THOC5 C22c	0.7089	0.6809	0.9258	0.8185	0.5908	0.7808	0.5834	0.7622	0.9921	0.8778
1685	S;S-312|318	O43586	Proline-serine-th	PSTPIP1 CD2	0.4499	0.3735	0.6815	0.3863	0.2704	0.5760	0.5627	0.4280	-0.0968	-0.2267
1686	S;S-313|321	Q8TF01	Arginine/serine-r	PNISR C6orf	-0.1186	0.0022	0.0280	-0.3991	-0.0176	-0.2059	-0.2879	-0.1770	0.3607	0.6541
1687	S;S-314|316	Q13247	Serine/arginine-r	SRSF6 SFRS6	0.2546	-0.0044	0.7841	1.3002	1.2434	1.0309	1.0635	1.1524	0.8969	1.0179
1688	S;S-320|321	Q9NY61	Protein AATF (Ap	AATF CHE1 D	0.9876	1.1271	0.9052	1.2606	0.7712	1.0814	0.9243	0.8971	1.0344	0.8919
1689	S;S-322|323	Q9UQ35	Serine/arginine r	SRRM2 KIAA	1.0081	1.3305	1.4288	1.4982	1.2916	1.6356	1.2838	1.4633	1.5494	1.6050
1690	S;S-328|330	P32249	G-protein couple	GPR183 EBI2	0.7835	0.8468	0.5246	0.3536	-0.0050	-0.0510	0.0069	-0.1756	-0.5040	-0.4781
1691	S;S-328|330	Q92922	SWI/SNF comple	SMARCC1 BA	0.7652	0.9230	0.5630	0.3455	0.5826	0.3769	0.5115	0.3531	0.7401	0.7923
1692	S;S-328|333	P32249	G-protein couple	GPR183 EBI2	-0.5959	-0.5532	0.1224	-0.5841	-0.0213	-0.6551	-0.1148	-0.8166	-0.6700	-0.7297
1693	S;S-33|37_C	Q9Y4F9	Rho family-intera	RIPOR2 C6or	-0.4956	-0.6437	-0.7773	-0.9736	-0.8859	-0.3238	-0.4774	-0.5540	-0.4967	-0.8154
1694	S;S-335|336	Q8IY81	pre-rRNA 2'-O-rib	FTSJ3 SB92	0.6918	0.8979	0.2037	0.5689	0.3095	0.7030	0.1689	0.1928	0.4577	0.1846
1695	S;S-343|347	O75676	Ribosomal protei	RPS6KA4 MS	-0.6890	-0.7056	-1.2509	-1.1518	-1.4389	-1.6787	-0.8721	-1.0403	-1.7270	-1.6918
1696	S;S-35|39_C	Q9Y4H4	G-protein-signali	GPSM3 AGS4	0.6486	0.6246	0.0229	0.6459	0.5436	0.3601	0.8824	0.6976	-0.2172	-0.0418
1697	S;S-351|353	Q9UQ35	Serine/arginine r	SRRM2 KIAA	0.1911	0.0033	-0.1499	0.3985	0.2395	-0.3071	0.1975	-0.1961	-0.2901	0.0934
1698	S;S-351|355	P16150	Leukosialin (GPL1	SPN CD43	0.6635	0.7842	0.7968	0.7049	0.8156	1.2692	0.9592	1.3759	0.7783	0.4525
1699	S;S-354|356	Q13523	Serine/threonine	PRPF4B KIAA	-1.2543	-1.2627	0.8517	0.2976	0.4465	0.3763	0.7661	0.7222	0.6911	0.8961
1700	S;S-364|365	Q9BZD3	Putative GRINL1E	GCOM2 GRII	0.4722	0.4362	0.4550	0.5039	0.2673	0.3990	0.3149	0.2454	0.5521	0.2259
1701	S;S-365|368	Q9NQZ2	Something about	UTP3 CRLZ1	-0.3782	-0.5147	0.0352	-0.1617	0.2473	0.3819	0.2133	0.2466	0.1669	0.0898
1702	S;S-366|368	Q13523	Serine/threonine	PRPF4B KIAA	0.0162	-0.1827	-0.1983	-0.0599	-0.0863	-0.5146	0.0193	-0.2716	-0.4060	-0.0207
1703	S;S-367|369	Q12874	Splicing factor 3A	SF3A3 SAP61	-0.3296	-0.3434	-0.4771	-0.3349	-0.1401	0.0309	-0.3812	-0.1892	-0.0196	-0.3476
1704	S;S-37|42_P	P16402	Histone H1.3 (His	H1-3 H1F3 H	-0.4755	-0.3534	-0.2591	-0.3454	-0.1021	0.1219	-0.1173	-0.2037	-0.9877	-0.7239
1705	S;S-370|371	Q9UEW8	STE20/SPS1-relat	STK39 SPAK	-0.6773	-0.5761	-0.5260	-0.4180	-0.7250	-0.4240	-0.2457	-0.3908	-0.7270	-0.7490
1706	S;S-378|379	Q8N554	Zinc finger protei	ZNF276 CEN	0.6918	0.8703	0.8811	0.8233	0.7914	0.9918	0.7160	0.7321	0.7414	0.8687
1707	S;S-379|382	Q8N554	Zinc finger protei	ZNF276 CEN	0.7829	0.9481	0.8748	0.9082	0.9608	1.0052	0.8929	0.9207	0.9328	0.6235
1708	S;S-380|382	Q9BVS4	Serine/threonine	RIOK2 RIO2	-0.2630	-0.4243	-0.0807	-0.2415	-0.1001	-0.2441	-0.3265	-0.4606	-0.2822	-0.1895
1709	S;S-382|384	P28715	DNA repair prote	ERCC5 ERCM	-1.1484	-0.8300	-0.9958	-0.9020	-1.1585	-1.0301	-1.1605	-1.4150	-1.0789	-1.0538
1710	S;S-382|386	Q96JM3	Chromosome alig	CHAMP1 C13	0.2224	0.2079	0.5009	0.5051	0.3360	0.4683	0.1930	0.2620	0.2676	0.3891
1711	S;S-383|384	Q15424	Scaffold attachm	SAFB HAP HI	0.8356	0.6838	0.5289	0.4307	0.7627	0.6031	0.8800	0.8327	0.6496	0.8703
1712	S;S-389|390	P55010	Eukaryotic transl	EIF5	-0.5213	-0.3646	-0.1189	-0.2268	-0.2896	-0.1736	-0.1722	-0.2846	-0.0249	-0.3184
1713	S;S-389|393	Q8IYB3	Serine/arginine r	SRRM1 SRM	0.9522	1.1164	0.0270	0.2099	0.1643	-0.0285	0.2939	0.1202	0.0586	0.2564
1714	S;S-391|393	Q8IYB3	Serine/arginine r	SRRM1 SRM	1.7797	1.5495	1.3017	1.1558	1.5951	1.3728	1.3397	1.2614	1.4746	1.4202
1715	S;S-392|394	Q86X95	Corepressor inte	CIR1 CIR	0.9191	0.6741	0.9948	1.1827	1.5436	1.1636	1.3206	1.3462	0.9314	1.0109
1716	S;S-398|400	Q96ST2	Protein IWS1 hor	IWS1 IWS1L	0.6242	0.5555	0.5632	0.7553	0.3258	0.5978	0.4215	0.6777	0.4251	0.4383

1717	S;S-409|410	P23497	Nuclear autoanti	SP100	-0.2761	-0.2222	0.0000	-0.1459	-0.2762	-0.2318	-0.3039	-0.2939	-0.0157	0.3196
1718	S;S-41|42_P	P19338	Nucleolin (Protei	NCL	-0.6123	-0.5505	-0.9840	-0.6840	-0.9894	-0.4675	-1.2176	-0.6650	-0.6029	-0.7088
1719	S;S-413|415	Q13427	Peptidyl-prolyl ci	PPIG	0.5838	0.6174	0.9580	1.2282	0.8821	1.3385	0.8561	1.2753	1.0097	0.8068
1720	S;S-416|420	P85037	Forkhead box pr	FOXK1 MNF	0.2378	0.3319	-0.0730	-0.1090	-0.6279	0.1469	0.4293	0.4074	0.2469	0.3812
1721	S;S-421|423	Q13547	Histone deacetyl	HDAC1 RPD3	0.4069	0.3467	0.6912	0.8243	0.5981	0.6528	0.5654	0.6092	0.7752	0.4795
1722	S;S-422|424	Q92769	Histone deacetyl	HDAC2	-0.2394	0.1210	0.3173	0.2979	0.2175	0.2380	-0.0117	0.0788	0.3918	0.3989
1723	S;S-422|424	Q9NTZ6	RNA-binding pro	RBM12 KIAA	0.4784	0.6441	0.1907	0.7180	0.2780	0.4683	0.3655	-0.0494	0.0187	-0.0863
1724	S;S-429|430	Q03468	DNA excision rep	ERCC6 CSB	-0.3183	-0.2158	0.1272	-0.0269	0.1676	0.4401	0.0820	0.1342	-0.0706	-0.0142
1725	S;S-429|431	Q8IYB3	Serine/arginine r	SRRM1 SRM	1.3696	1.3244	0.9853	1.4560	1.3820	0.9166	1.4326	1.2259	1.0901	1.0874
1726	S;S-431|437	Q13523	Serine/threonine	PRPF4B KIAA	1.3392	1.3441	1.2936	1.1598	1.0639	0.8895	1.1362	1.0053	1.2298	1.1908
1727	S;S-435|436	Q13435	Splicing factor 3E	SF3B2 SAP14	0.3052	0.1915	0.1557	0.1390	0.3932	0.1643	0.0308	0.2415	0.4299	0.2904
1728	S;S-44|47_C	O00178	GTP-binding prot	GTPBP1	-0.5895	-0.5207	-0.6530	-0.6609	-0.8198	-0.5196	-0.7579	-0.8660	-0.8561	-0.8167
1729	S;S-442|445	Q13422	DNA-binding pro	IKZF1 IK1 IKA	-0.7214	-1.5597	0.2028	0.0106	0.1260	0.5590	0.3540	0.6468	0.1990	-0.0672
1730	S;S-443|444	Q9BQF6	Sentrin-specific p	SENP7 KIAA1	0.0567	0.1281	0.1854	-0.1697	-0.0922	0.1157	0.2704	0.0031	0.1865	0.3713
1731	S;S-453|455	P29374	AT-rich interactiv	ARID4A RBB	0.7391	0.8728	0.1706	0.3822	0.2704	0.3922	0.4860	0.2823	0.0834	0.2093
1732	S;S-454|456	Q9UQ35	Serine/arginine r	SRRM2 KIAA	2.3335	2.1913	1.7974	2.4146	2.4432	1.7988	2.5994	2.0932	1.7657	2.0157
1733	S;S-455|457	Q7Z5L9	Interferon regula	IRF2BP2	-0.9678	-0.6984	-0.7542	-0.4987	-0.4691	-0.5775	-0.4684	-0.4480	-0.4515	-0.4777
1734	S;S-459|461	Q7Z5K2	Wings apart-like	WAPL FOE K	-0.3966	-0.4311	-0.3977	-0.4160	-0.4845	-0.3951	-0.5441	-0.5809	-0.3475	-0.2608
1735	S;S-460|462	P55201	Peregrin (Bromo	BRPF1 BR14	0.8476	0.9788	0.9580	0.8785	0.9088	1.1867	0.8263	0.8571	1.0232	1.1663
1736	S;S-463|465	Q8IYB3	Serine/arginine r	SRRM1 SRM	0.3299	0.3989	0.6181	1.1606	1.0444	1.0511	0.7581	0.9351	0.5732	0.6277
1737	S;S-472|478	Q76N32	Centrosomal pro	CEP68 KIAA(	-0.0972	-0.2145	-0.2201	-0.0880	-0.3970	-0.2961	-0.3728	-0.3052	-0.0530	-0.2072
1738	S;S-4752|47	Q9NU22	Midasin (Dynein-	MDN1 KIAA(	0.8476	0.9133	0.1510	0.0282	0.4947	0.2506	0.3702	0.1258	0.2114	0.1209
1739	S;S-479|480	Q96KC8	DnaJ homolog su	DNAJC1 HTJ1	0.2621	0.1402	0.2573	0.0712	-0.1421	-0.1323	-0.1576	0.0510	-0.4766	-0.8708
1740	S;S-479|480	Q96RK0	Protein capicua h	CIC KIAA030	0.2131	0.3025	0.0146	0.2892	0.0674	0.0207	-0.0289	-0.1650	0.1990	0.2756
1741	S;S-483|484	P42768	Wiskott-Aldrich s	WAS IMD2	-0.3433	-0.6752	-0.4074	-0.5498	-0.8522	-0.4220	-0.6541	-0.5207	-0.7954	-0.5683
1742	S;S-483|485	O75925	E3 SUMO-protein	PIAS1 DDXBI	-0.4603	-0.7797	-0.0479	-0.0730	0.1478	0.1102	-0.2033	0.1272	-0.0436	-0.1068
1743	S;S-483|492	Q96Q42	Alsin (Amyotropl	ALS2 ALS2CR	-1.1524	-1.1116	-1.0599	-1.0342	-0.8324	-0.7246	-1.3200	-1.1013	-1.1493	-0.8268
1744	S;S-484|486	Q9UQ35	Serine/arginine r	SRRM2 KIAA	1.3807	1.4027	1.2458	2.1478	2.2481	1.4602	2.2741	1.8814	1.2616	1.4698
1745	S;S-485|494	O60832	H/ACA ribonucle	DKC1 NOLA4	1.0063	0.9567	1.3681	1.3712	1.4590	1.4597	1.3586	1.4511	1.2627	1.2104
1746	S;S-487|491	O75038	1-phosphatidylin	PLCH2 KIAA(	0.4591	0.6131	0.7188	0.7006	0.0174	0.1590	0.2200	0.3365	0.2730	0.0568
1747	S;S-489|494	O94913	Pre-mRNA cleava	PCF11 KIAA(	-0.7394	-0.6403	-0.5396	-0.5472	-0.3055	-0.3257	-0.3249	-0.4314	-0.5040	-0.6650
1748	S;S-496|497	Q9C0K0	B-cell lymphoma	BCL11B CTIP	-1.2871	-1.3476	0.3257	0.1227	0.0192	0.9330	-0.4648	0.2428	0.9941	0.6442
1749	S;S-498|499	Q8TCJ2	Dolichyl-diphosp	STT3B SIMP	2.3179	1.8369	0.7022	0.8423	-0.5027	0.5948	0.8503	0.3389	-0.1165	-0.7818
1750	S;S-498|500	Q9H7N4	Splicing factor, a	SCAF1 SFRS1	0.3081	0.6022	0.2998	0.1687	0.2380	0.2490	0.2764	0.2896	0.3265	0.2861
1751	S;S-499|501	Q9H0D6	5'-3' exoribonucl	XRN2	0.3287	0.4055	0.1358	0.3008	-0.0727	0.2190	0.1596	0.1046	0.0846	0.2862
1752	S;S-502|514	Q9Y2X3	Nucleolar protein	NOP58 NOL5	0.8365	1.0328	1.2245	1.0472	1.0710	1.4352	1.0875	1.1167	0.9644	0.8345
1753	S;S-504|505	P29590	Protein PML (Pro	PML MYL PP	1.0593	1.1808	1.3165	1.2972	1.0867	1.0697	1.1964	1.1692	1.3393	1.2048
1754	S;S-508|510	Q9UQ35	Serine/arginine r	SRRM2 KIAA	-4.3183	-3.2391	-3.5955	-3.2542	-2.6949	-3.3673	-2.6998	-2.9638	-3.4285	-3.5012
1755	S;S-509|511	O94913	Pre-mRNA cleava	PCF11 KIAA(	0.7975	0.7151	0.9242	0.7575	0.7998	1.0569	0.8190	1.1046	0.8708	0.9335

1756	S;S-509|511	Q96B97	SH3 domain-cont	SH3KBP1 CIN	-0.4018	-0.2313	-0.2128	-0.3851	-0.2695	-0.2336	-0.3363	-0.3888	-0.5509	-0.3152
1757	S;S-518|527	P29590	Protein PML (Pro	PML MYL PP	0.8872	0.9033	0.2824	0.5867	0.5810	0.3531	0.5697	0.3865	0.6061	0.6590
1758	S;S-519|520	O00567	Nucleolar protei	NOP56 NOL	0.2084	0.0231	-0.1852	-0.5164	-0.2806	-0.0255	-0.1346	-0.1029	-0.3803	-0.0794
1759	S;S-52|53_P	P55081	Microfibrillar-ass	MFAP1	0.4774	0.1925	0.2546	-0.0711	0.1887	0.4280	0.1585	0.4694	0.4124	0.5294
1760	S;S-52|54_C	Q8TAD8	Smad nuclear-int	SNIP1	-0.1364	0.1710	-0.3251	-0.5087	0.1560	-0.2708	-0.0328	-0.3689	-0.2524	0.0886
1761	S;S-527|529	Q5VTL8	Pre-mRNA-splicir	PRPF38B	0.3626	-0.2456	0.3072	0.9530	0.8364	0.4390	1.0219	0.6967	0.1305	0.3812
1762	S;S-53|55_C	Q9NVC6	Mediator of RNA	MED17 ARC	-3.5317	-3.5663	0.1301	0.0541	0.9039	0.6612	-3.9278	-2.9878	-4.0706	-3.5307
1763	S;S-53|56_C	Q9P2R6	Arginine-glutami	RERE ARG A	-0.1050	-0.1164	0.3990	0.4836	-0.0326	0.2304	0.1303	0.2376	0.4830	0.3299
1764	S;S-532|534	Q86VM9	Zinc finger CCCH	ZC3H18 NHN	1.1525	1.2141	1.3025	1.3244	1.3309	1.4613	1.5249	1.6920	1.0410	0.7459
1765	S;S-533|539	Q9H6Z4	Ran-binding prot	RANBP3	0.2964	0.2560	0.0962	-0.0287	-0.1259	0.0382	-0.0939	0.0137	-0.0118	-0.0273
1766	S;S-54|58_C	Q9H1E3	Nuclear ubiquito	NUCKS1 NU	0.9587	0.7759	1.0239	1.8338	1.6341	1.4130	1.5636	1.6152	0.8282	1.1467
1767	S;S-544|546	Q13427	Peptidyl-prolyl ci	PPIG	2.0528	1.9033	2.1994	2.5373	2.5184	2.3288	2.4027	2.3935	2.1669	2.1543
1768	S;S-547|550	Q9H1C4	Protein unc-93 h	UNC93B1 UI	0.0688	0.0350	0.0665	-0.0397	-0.6742	-0.6158	-0.5824	-0.5789	-0.7292	-0.5779
1769	S;S-549|551	Q8IYB3	Serine/arginine r	SRRM1 SRM	0.2636	0.4484	0.4124	0.4515	0.5461	0.2481	0.3377	0.4572	0.2080	0.5612
1770	S;S-557|558	Q9Y5B6	PAX3- and PAX7-	PAXBP1 C21	-0.1994	-0.3547	-0.3475	-0.1878	-0.6654	-0.1956	-0.2959	-0.1496	-0.3552	-0.1662
1771	S;S-558|560	Q14966	Zinc finger protei	ZNF638 NP2	0.6760	0.5080	0.4358	0.3364	0.4710	0.7647	0.6364	0.6122	0.5697	0.3832
1772	S;S-560|562	Q8IYB3	Serine/arginine r	SRRM1 SRM	-0.0551	0.2151	0.2534	0.2279	0.4552	0.2095	0.2786	0.2595	0.1976	0.3096
1773	S;S-561|563	Q8TF01	Arginine/serine-r	PNISR C6orf	-0.2959	-0.2196	-0.3029	0.1975	0.4292	-0.3963	0.1136	-0.0031	-0.3568	-0.1619
1774	S;S-569|570	O00567	Nucleolar protei	NOP56 NOL	1.3731	1.2165	1.1725	1.0498	1.0867	1.2374	1.0491	1.1487	0.8793	0.3841
1775	S;S-573|575	P21580	Tumor necrosis f	TNFAIP3 OT	-0.2893	0.1422	0.1919	0.2130	0.4114	0.4302	0.0129	0.3653	0.1529	-0.0930
1776	S;S-576|578	O15014	Zinc finger protei	ZNF609 KIA	1.7228	1.7951	1.1896	1.0930	1.1494	1.2643	1.2481	1.1604	1.6340	1.3881
1777	S;S-576|580	Q8IZM8	Zinc finger protei	ZNF654	-0.7668	-0.8046	-0.7107	-0.6551	-0.8129	-0.7222	-0.7470	-0.9343	-0.6172	-0.6070
1778	S;S-58|61_C	Q9H1E3	Nuclear ubiquito	NUCKS1 NU	1.1175	1.0392	1.3055	2.2640	1.6633	1.6565	1.8091	1.8074	0.8860	1.0343
1779	S;S-590|597	Q9H4E7	Differentially exp	DEF6 IBP	0.8110	0.7557	1.8495	1.2130	1.5366	1.1570	1.5428	1.2415	1.2848	0.7165
1780	S;S-60|63_C	Q13442	28 kDa heat- and	PDAP1 HASF	0.2289	0.1458	0.3378	0.2622	0.3996	0.3135	0.3520	0.3007	0.4675	0.3491
1781	S;S-60|69_C	Q7L2J0	7SK snRNA meth	MEPCE BCDI	0.0843	0.0565	0.1780	0.3449	0.2380	0.3762	0.2110	0.4460	0.4007	0.4749
1782	S;S-601|604	Q15424	Scaffold attachm	SAFB HAP HI	1.0081	0.9855	1.0819	1.0779	1.3578	0.9677	1.7558	1.2428	0.9218	0.8438
1783	S;S-602|603	Q96RT1	Erbin (Densin-18	ERBIN ERBB	0.7261	0.7131	0.8541	0.8142	0.7688	1.0704	0.7566	0.7977	0.6963	0.2596
1784	S;S-61|63_C	Q9NXX6	Non-structural m	NSMCE4A C	-0.8787	-1.0715	-1.1592	-1.1919	-1.1100	-0.9823	-1.1547	-1.0403	-0.9401	-0.8382
1785	S;S-612|614	Q9H7N4	Splicing factor, a	SCAF1 SFRS1	0.2424	0.3459	-0.0371	0.2297	0.1124	-0.0407	0.4739	0.5352	-0.2649	-0.1807
1786	S;S-618|619	Q9Y6X4	Soluble lamin-as	FAM169A KI	-0.5904	-0.5163	-0.1499	-0.1818	-0.4240	-0.2583	-0.3897	-0.2996	0.4434	0.4192
1787	S;S-619|620	Q641Q2	WASH complex s	WASHC2A F	-0.5768	-0.6524	-0.6454	-0.8724	-0.9772	-0.7494	-1.0981	-0.7794	-0.8529	-0.7664
1788	S;S-62|64_C	O43159	Ribosomal RNA-p	RRP8 KIAA0	-0.2098	-0.1176	-0.5876	-0.6192	-0.6654	-0.5709	-0.6460	-0.5295	-0.5003	-0.4180
1789	S;S-626|628	Q8IYB3	Serine/arginine r	SRRM1 SRM	-0.0364	-0.6999	0.2011	0.3697	0.4880	0.0652	0.3175	0.2864	0.3582	0.6032
1790	S;S-626|631	Q9Y2K2	Serine/threonine	SIK3 KIAA09	-0.5661	-0.3434	-0.6869	-0.6978	-0.6919	-0.4441	-0.7318	-0.8770	-1.0598	-0.9581
1791	S;S-627|631	Q6PKG0	La-related protei	LARP1 KIAA	0.2379	0.0959	0.3611	0.4117	0.3085	0.5010	0.2311	0.2610	0.6098	0.6136
1792	S;S-63|65_P	P52701	DNA mismatch re	MSH6 GTBP	-0.8074	-0.5695	1.1325	1.1398	0.9945	1.2241	1.0585	1.1131	1.0296	1.1936
1793	S;S-636|638	Q8IYB3	Serine/arginine r	SRRM1 SRM	-0.0200	-0.0645	0.5425	0.4747	0.9796	-0.2961	0.7612	-0.1095	0.5351	0.4478
1794	S;S-640|641	Q3YEC7	Rab-like protein	RABL6 C9orf	0.8161	0.9749	1.2299	1.3505	0.6542	1.3397	0.9110	1.2246	0.8399	0.6475

1795	S;S-643|646	Q86X29	Lipolysis-stimulat	LSR LISCH	0.8586	0.9799	0.8628	1.2137	0.8093	1.6125	0.9876	0.9101	0.9477	0.6067
1796	S;S-650|653	Q9UKJ3	G patch domain-	GPATCH8 GI	0.3835	0.3380	0.5374	0.3849	0.5896	0.4467	0.4841	0.6380	0.6548	0.6293
1797	S;S-653|655	Q8IYB3	Serine/arginine r	SRRM1 SRM	1.3654	0.9407	1.5064	1.9929	2.1469	1.8923	1.8739	1.9920	1.6440	1.6732
1798	S;S-655|657	Q9UKV3	Apoptotic chrom	ACIN1 ACINI	1.2115	0.9389	0.8714	1.2613	1.1871	0.7664	1.2644	1.0122	0.9328	0.9994
1799	S;S-659|663	Q15334	Lethal(2) giant la	LLGL1 DLG4	-0.5161	-0.7589	-0.1223	-0.4252	-0.0083	-0.8967	0.1124	-0.3223	-0.8113	-0.9885
1800	S;S-66|68_C	Q9UJV9	Probable ATP-de	DDX41 ABS	-0.1464	0.0361	-0.0063	0.0264	-0.1917	0.0697	0.0967	0.2180	0.0372	-0.0917
1801	S;S-67|74_C	Q9BXP5	Serrate RNA effe	SRRT ARS2 A	0.9671	0.8824	1.0110	1.1349	1.0073	1.2171	1.0019	1.0000	1.0123	0.9288
1802	S;S-670|674	Q96T37	RNA-binding pro	RBM15 OTT	0.0427	0.2232	0.0474	0.0980	0.0064	0.0796	0.0697	0.0107	0.0686	0.0242
1803	S;S-672|673	Q9Y3T9	Nucleolar compl	NOC2L NIR	-0.9055	-0.7911	-0.5275	-0.8987	-0.9735	-0.6272	-0.6809	-0.8328	-0.6742	-0.4652
1804	S;S-673|677	Q9UEY8	Gamma-adducin	ADD3 ADDL	0.1735	0.3363	0.4466	0.5324	0.4196	0.5590	0.6255	1.0265	0.1053	0.0934
1805	S;S-678|680	Q9UKE5	TRAF2 and NCK-i	TNIK KIAA05	1.2794	1.1174	1.0223	1.1129	0.9302	0.9888	1.2612	1.1145	0.8997	0.7212
1806	S;S-678|681	Q8N163	Cell cycle and ap	CCAR2 DBC1	0.2756	0.3034	-0.9587	-1.2842	-0.6567	-0.6272	-0.5159	-0.5517	-0.5895	-0.4546
1807	S;S-683|685	Q8IYB3	Serine/arginine r	SRRM1 SRM	-1.3319	-1.2042	-0.4208	-0.3940	-0.3359	-0.5077	-0.4638	-0.3315	-0.5221	-0.1988
1808	S;S-683|686	Q8TC05	Nuclear protein I	MDM1	-0.1665	-0.1752	-0.3055	-0.6083	-0.0766	-0.1307	-0.1134	-0.0293	0.0524	0.2193
1809	S;S-688|689	P49959	Double-strand br	MRE11 HNG	-0.4284	-0.6231	-0.2547	-0.6551	-0.4742	-0.2888	-0.3511	-0.4130	-0.2997	-0.2043
1810	S;S-710|718	Q8IXT5	RNA-binding pro	RBM12B	0.2239	0.5166	0.1519	0.2493	0.4086	-0.0453	0.1964	0.0000	0.2348	0.5133
1811	S;S-725|727	Q96T58	Msx2-interacting	SPEN KIAA0	-0.1325	0.0995	-0.2609	-0.2103	-0.0439	-0.3294	-0.2228	-0.2921	-0.2649	-0.4078
1812	S;S-729|731	P46100	Transcriptional r	ATRX RAD54	0.5120	0.2606	0.7707	0.3546	0.7081	0.4939	0.6456	0.6082	0.7422	0.5965
1813	S;S-729|731	Q66K74	Microtubule-ass	MAP1S BPY2	-0.4554	-0.5100	-0.3004	-0.1128	-0.0139	-0.3257	-0.3728	-0.4273	-0.4622	-0.2804
1814	S;S-736|738	P98175	RNA-binding pro	RBM10 DXS8	0.7797	0.9889	-0.3422	0.1013	0.0355	-0.3788	0.2992	-0.0996	-0.2758	-0.0498
1815	S;S-737|739	Q9UER7	Death domain-as	DAXX BING2	0.2393	-0.0033	0.0585	0.1502	-0.1917	-0.0499	-0.1092	-0.0545	0.1692	-0.1096
1816	S;S-738|740	Q8IYB3	Serine/arginine r	SRRM1 SRM	1.3349	0.9273	2.0364	2.2679	2.2631	2.1500	2.1217	2.3170	1.8860	1.8428
1817	S;S-738|743	Q8IYB3	Serine/arginine r	SRRM1 SRM	0.8794	0.7616	1.2651	1.3265	1.0910	1.1736	1.1821	1.2121	1.1553	1.2820
1818	S;S-739|743	Q9Y6X9	ATPase MORC2 (	MORC2 KIAA	0.8068	0.3615	0.9231	1.3159	1.6180	1.3722	1.5135	1.3647	1.0097	1.0412
1819	S;S-74|76_C	O60930	Ribonuclease H1	RNASEH1 RN	-0.8361	-0.8538	-0.7737	-0.3259	-0.7129	-0.9078	-0.3216	-0.9758	-0.9252	-0.6670
1820	S;S-743|751	Q08J23	RNA cytosine C(5	NSUN2 SAKI	-0.4905	-0.5978	-0.4005	-0.5394	-0.6742	-0.4421	-1.5707	-1.6395	-1.4181	-1.4511
1821	S;S-744|745	Q13427	Peptidyl-prolyl ci	PPIG	0.6590	0.8884	0.8023	0.9214	0.6005	0.8505	0.5271	0.9463	0.9856	0.9871
1822	S;S-748|758	Q13627	Dual specificity t	DYRK1A DYR	-0.4930	-0.4865	-0.4482	-0.9255	-0.3922	-0.3313	-0.4068	-0.3090	-0.2309	-0.5779
1823	S;S-77|83_P	P10644	cAMP-dependen	PRKAR1A PK	1.2092	1.2899	1.0991	0.9603	1.0753	0.7211	0.9033	0.4382	0.9584	1.0568
1824	S;S-778|780	Q9UQ35	Serine/arginine r	SRRM2 KIAA	0.6289	0.7083	1.1123	1.1178	1.1952	0.9646	1.0955	1.0628	1.1732	1.2607
1825	S;S-779|782	Q8IWA0	WD repeat-conta	WDR75 UTP	0.3437	0.3237	-0.3938	-0.1511	-0.3054	-0.2404	-0.3714	-0.3657	-0.2845	-0.3519
1826	S;S-78|80_P	P13861	cAMP-dependen	PRKAR2A PK	0.0757	-0.1009	-0.0963	0.2130	-0.0650	-0.4727	0.2032	-0.1361	-0.2586	-0.0260
1827	S;S-781|782	O94880	PHD finger prote	PHF14 KIAA	0.0480	-0.3462	-0.3502	-0.8149	-0.3238	-0.2162	-0.4034	-0.3223	0.0586	0.1548
1828	S;S-787|790	Q86YV0	RAS protein activ	RASAL3	0.8695	0.7305	1.4634	1.6448	1.2064	1.6149	1.8890	1.8453	1.0078	1.2899
1829	S;S-79|81_C	Q5VUA4	Zinc finger prote	ZNF318 HRII	0.2492	0.3173	0.3506	0.3968	0.2860	0.3027	0.3432	0.4057	0.4679	0.6556
1830	S;S-797|802	Q8IYB3	Serine/arginine r	SRRM1 SRM	1.1245	1.1388	0.5228	0.8473	0.7326	0.7952	0.4964	0.8629	0.5281	0.4276
1831	S;S-80|82_C	Q15637	Splicing factor 1	SF1 ZFM1 ZN	1.0036	1.0665	-0.0787	0.1756	-0.2458	-0.0356	-0.3495	-0.1909	0.0285	0.4628
1832	S;S-805|809	Q9BTC0	Death-inducer ol	DIDO1 C20o	-0.8688	-0.5794	-0.7090	-1.0636	-0.9203	-0.9218	-0.8888	-0.9638	-0.7078	-0.7148
1833	S;S-807|808	Q9BZF1	Oxysterol-bindin	OSBPL8 KIAA	0.6544	0.6051	0.6458	0.3795	0.3081	0.4897	0.4607	0.4348	0.0956	0.3134

1834	S;S-816|818	Q99590	Protein SCAF11 (	SCAF11 CASI	0.9587	0.9273	1.3106	1.5641	1.6940	1.0483	1.8094	1.3096	0.8997	1.1919
1835	S;S-826|828	Q14687	Genetic suppress	GSE1 KIAA01	0.5946	0.7655	0.2334	0.2950	0.1361	-0.0561	0.0129	-0.0231	-0.0830	-0.1519
1836	S;S-8277|82	Q8NF91	Nesprin-1 (Enapt	SYNE1 C6orf	-0.2015	-0.3336	-0.2597	-0.2041	-0.1938	-0.1905	-0.2033	-0.3748	-0.6618	-0.6528
1837	S;S-83|85_P	P31323	cAMP-dependen	PRKAR2B	-1.1808	-1.2417	-1.2116	-1.2929	-1.8324	-1.9735	-1.5746	-1.3090	-1.4930	-1.3891
1838	S;S-837|839	Q96FS4	Signal-induced p	SIPA1 SPA1	0.6053	0.7746	0.4649	0.3265	0.5473	0.4247	0.8532	0.6002	0.2837	0.3593
1839	S;S-84|85_C	O15541	E3 ubiquitin-prot	RNF113A RN	-0.3041	-0.2336	-0.2515	-0.3502	-0.4194	-0.3519	-0.4856	-0.5342	-0.2127	-0.2541
1840	S;S-84|86_C	Q14739	Delta(14)-sterol r	LBR	0.0036	-0.0985	-0.1042	0.3685	0.6623	-0.4604	0.6929	-0.1294	-0.0899	0.0593
1841	S;S-84|88_C	Q13151	Heterogeneous r	HNRNPA0 H	0.5044	0.8734	0.2833	0.4213	0.6831	0.1603	0.9538	0.7549	0.1634	0.3522
1842	S;S-849|850	P46100	Transcriptional r	ATRX RAD54	1.3198	1.5853	1.1257	0.7575	0.8650	0.8205	0.8696	0.7907	1.1893	0.9159
1843	S;S-85|87_C	Q6UN15	Pre-mRNA 3'-enc	FIP1L1 FIP1	-0.5265	-0.3703	-0.2299	0.2687	-0.2874	-0.2231	-0.4276	-0.4733	0.0000	-0.1068
1844	S;S-86|88_C	Q9NXG2	THUMP domain-	THUMPD1	1.2809	1.3546	0.6497	0.7910	0.7301	0.8644	0.9829	0.8667	0.7738	0.5559
1845	S;S-86|89_P	P05114	Non-histone chro	HMGN1 HM	0.9726	1.1062	0.4604	1.3733	1.0408	1.0037	1.2122	1.2061	0.6009	0.8417
1846	S;S-860|861	Q6GYQ0	Ral GTPase-activ	RALGAPA1 G	1.6405	1.7596	0.9794	1.1112	1.1124	1.1280	1.2928	1.0795	0.9935	1.0703
1847	S;S-862|865	Q7Z6I6	Rho GTPase-activ	ARHGAP30	-0.2783	-0.3604	-0.7791	-0.7123	-0.7904	-0.7494	-0.5122	-0.7002	-1.0625	-0.9657
1848	S;S-862|871	O14974	Protein phospha	PPP1R12A M	0.6160	0.8794	0.3587	0.5483	0.1577	0.1143	0.0473	0.3771	0.0091	0.0630
1849	S;S-87|89_C	Q9C0C9	(E3-independent	UBE2O KIAA	-1.8655	-1.6095	-1.5305	-1.8056	-2.0439	-1.4665	-1.7339	-1.6635	-1.6353	-1.3104
1850	S;S-87|93_C	Q13523	Serine/threonine	PRPF4B KIAA	-1.8329	-2.3646	-1.6210	-1.4252	3.3606	3.2055	NA	NA	-2.0598	-2.0862
1851	S;S-871|876	Q9UQ35	Serine/arginine r	SRRM2 KIAA	-0.2982	-0.1714	-0.4539	-0.2755	-0.1259	-0.4280	-0.0746	-0.5339	-0.3255	-0.0418
1852	S;S-872|874	Q9Y5B0	RNA polymerase	CTDP1 FCP1	1.0945	1.1940	1.2307	0.8132	0.9370	0.8799	0.9431	1.2088	0.9280	0.6351
1853	S;S-875|876	Q9UQ35	Serine/arginine r	SRRM2 KIAA	0.1163	0.2089	0.1706	0.3643	0.0009	0.1348	-0.0774	0.0643	0.3060	0.1618
1854	S;S-883|884	Q01831	DNA repair prote	XPC XPCC	0.4929	0.4638	-0.2189	-0.2021	0.2380	0.2766	0.0685	0.0242	-0.4712	-0.4215
1855	S;S-89|90_C	Q14738	Serine/threonine	PPP2R5D	0.1926	0.1612	-0.2213	-0.1677	-0.2065	-0.3164	-0.1490	-0.4089	-0.3786	0.0000
1856	S;S-89|91_C	Q5VUA4	Zinc finger protei	ZNF318 HRII	0.3612	0.0077	0.2182	0.5796	0.4127	0.0950	0.1998	0.4268	0.2954	0.4307
1857	S;S-901|909	Q8IX07	Zinc finger protei	ZFPM1 FOG1	-0.7036	-0.5597	-0.8192	-0.7537	-0.9485	-0.9360	-0.4774	-0.9226	-0.9129	-0.8591
1858	S;S-91|93_C	Q9HCG8	Pre-mRNA-splicir	CWC22 KIAA	-0.3071	-0.2788	0.4823	0.4239	0.5436	0.8847	0.5415	0.8995	0.5288	-0.0181
1859	S;S-919|923	Q9P107	GEM-interacting	GMIP	1.2052	0.9636	1.2119	1.0558	0.5499	0.8661	0.7073	0.7986	0.7398	0.5410
1860	S;S-936|948	Q9P1Y6	PHD and RING fir	PHRF1 KIAA1	-0.7916	-0.8498	-0.6372	-0.4911	-1.0251	-0.5753	-0.7711	-0.7253	-0.7819	-0.6327
1861	S;S-94|98_I3	I3L1I5	Rho guanine nuc	ARHGEF18 K	-0.9749	-1.1727	-1.2534	-1.3282	-1.3470	-1.1736	-1.1751	-0.9969	-0.9476	-0.9987
1862	S;S-942|952	O60763	General vesicula	USO1 VDP	-0.8361	-1.0279	-0.6162	-0.6083	-0.8456	-0.5977	-0.8139	-0.7482	-0.7357	-1.0299
1863	S;S-946|948	O15042	U2 snRNP-associ	U2SURP KIA	0.8173	0.8941	1.2208	2.0130	2.1166	1.4800	1.9735	1.8465	1.0447	1.2349
1864	S;S-952|954	Q9UQ35	Serine/arginine r	SRRM2 KIAA	-0.7948	-1.3561	0.9132	0.2950	0.2597	0.9385	-0.6338	0.5717	0.8464	0.8229
1865	S;S-953|955	Q9H2P0	Activity-depende	ADNP ADNP	-0.0054	0.0628	-0.3462	-0.2123	-0.2129	-0.2690	-0.3880	-0.4171	-0.4077	-0.2710
1866	S;S-956|960	Q92974	Rho guanine nuc	ARHGEF2 KI	0.1671	-0.1080	0.3790	0.3322	0.3578	0.5055	0.1941	0.4085	0.4173	0.4231
1867	S;S;S-108|11	Q9HAZ1	Dual specificity p	CLK4	2.6910	2.1896	1.4077	2.3951	2.6309	1.8099	3.1450	2.4449	1.3469	1.6393
1868	S;S;S-109|11	P19484	Transcription fac	TFEB BHLHE	0.3140	0.4924	0.5829	0.5176	0.4361	0.5355	0.2682	0.4200	0.4299	0.3663
1869	S;S;S-12|13|	P45973	Chromobox prot	CBX5 HP1A	-0.1254	-0.2055	-0.2220	0.0098	-0.3322	-0.3606	-0.3837	-0.7265	0.0225	0.1452
1870	S;S;S-124|12	Q9H4L7	SWI/SNF-related	SMARCAD1	0.3140	0.0860	0.0464	-0.2627	0.0192	-0.0717	-0.0196	-0.1261	0.2046	0.2215
1871	S;S;S-140|14	Q9Y2U8	Inner nuclear me	LEMD3 MAN	-3.3004	-3.9553	-3.9048	-3.4936	-2.9699	-3.3219	-3.0732	-3.4232	-3.5759	-3.5456
1872	S;S;S-141|14	Q5QJE6	Deoxynucleotidy	DNTTIP2 ERI	0.9028	1.0665	1.1310	1.2130	0.7394	1.4960	1.1706	1.2015	0.9066	1.1362

1873	S;S;S-1478|1	Q9UQ35	Serine/arginine r	SRRM2 KIAA	0.8182	0.6146	0.5730	1.0955	1.1065	0.7112	1.4494	0.9526	0.6357	0.9449
1874	S;S;S-1512|1	P51531	Probable global t	SMARCA2 B/	0.7829	0.9978	0.6873	0.8403	0.3287	1.2042	0.6221	0.6526	0.3818	0.3452
1875	S;S;S-1522|1	Q02880	DNA topoisomer	TOP2B	0.2964	0.2002	0.0655	-0.0397	0.0498	0.1389	0.1573	-0.1161	-0.1122	-0.2373
1876	S;S;S-154|15	Q5JTV8	Torsin-1A-intera	TOR1AIP1 L/	-0.0385	-0.2326	-0.5923	-0.5841	-0.7009	-0.7197	-0.7210	-0.8492	-0.8135	-0.8755
1877	S;S;S-156|15	O75494	Serine/arginine-r	SRSF10 FUSI	-0.5850	-0.4169	0.7054	0.5422	0.8083	0.7030	1.0378	0.9053	0.6171	0.9097
1878	S;S;S-1579|1	Q9UQ35	Serine/arginine r	SRRM2 KIAA	-1.1484	-0.7459	-0.7124	-0.5576	-0.5290	-0.8344	-0.7125	-0.6371	-0.7400	-0.5381
1879	S;S;S-163|16	Q16629	Serine/arginine-r	SRSF7 SFRS7	0.9191	0.9441	0.5149	0.6918	0.7036	0.5800	0.8027	0.5530	0.5680	0.5507
1880	S;S;S-165|16	Q16629	Serine/arginine-r	SRSF7 SFRS7	1.2232	1.6813	0.9661	0.8531	1.1243	0.7328	1.3566	0.7854	0.8500	0.9819
1881	S;S;S-174|17	Q9Y4C8	Probable RNA-bi	RBM19 KIAA	-1.2479	-1.0055	-0.9206	-1.1128	-0.6567	-0.8029	-0.8485	-1.0721	-0.7752	-0.7863
1882	S;S;S-179|18	Q16629	Serine/arginine-r	SRSF7 SFRS7	2.0441	2.1592	1.1753	1.9006	1.6991	1.0936	1.8943	1.4505	1.3541	1.4919
1883	S;S;S-18|22|	Q9UHR5	SAP30-binding pi	SAP30BP HC	-2.2224	-2.6403	-3.2485	-3.2373	-3.7069	-3.0392	-2.9884	-3.1145	-3.0382	-3.0116
1884	S;S;S-192|19	Q8N4S0	Coiled-coil doma	CCDC82 HT0	-0.3525	-0.4452	-0.3436	-0.3349	-0.2431	0.0597	-0.6059	-0.1194	-0.0720	-0.2464
1885	S;S;S-193|19	Q8NAV1	Pre-mRNA-splicir	PRPF38A	-0.0895	-0.1009	0.2299	-0.1244	-0.0420	0.1984	-0.0302	0.3736	0.2986	0.1525
1886	S;S;S-2009|2	P18583	Protein SON (Ba	SON C21orf5	0.6077	0.5720	0.6451	0.4798	0.5785	0.5345	0.6431	0.5758	0.5983	0.6260
1887	S;S;S-2042|2	Q9UQ35	Serine/arginine r	SRRM2 KIAA	0.9587	1.0225	0.9450	0.8343	0.8041	0.7148	0.7665	0.7154	1.0219	1.1692
1888	S;S;S-218|22	P55198	Protein AF-17 (A	MLLT6 AF17	-0.3049	-0.5243	0.6418	0.7277	0.7014	0.7541	0.7403	0.8376	0.5758	0.9335
1889	S;S;S-224|22	Q86YV0	RAS protein activ	RASAL3	-1.2224	-1.0011	-0.0907	0.0541	-0.8032	-0.1753	-0.3330	-0.2388	-0.1977	0.1444
1890	S;S;S-229|23	Q9H1E3	Nuclear ubiquito	NUCKS1 NU	0.5801	0.4702	0.6841	0.9603	0.5698	0.6594	0.3762	0.4325	0.7131	0.7658
1891	S;S;S-230|23	P35659	Protein DEK	DEK	0.3640	0.5040	0.2804	0.2804	0.0242	0.2132	-0.0632	0.2739	0.1819	0.0530
1892	S;S;S-274|28	Q6KC79	Nipped-B-like pr	NIPBL IDN3	0.3904	0.1838	0.7534	0.4657	0.3904	0.4510	0.5053	0.6910	0.6923	0.6736
1893	S;S;S-296|29	P08240	Signal recognitio	SRPRA SRPR	0.1082	0.0184	-0.0570	-0.1900	-0.1081	-0.0866	-0.6796	-0.5936	-0.2957	-0.4168
1894	S;S;S-299|30	Q13247	Serine/arginine-r	SRSF6 SFRS6	-0.3388	-0.2771	-0.3004	-0.2007	-0.0895	-0.0628	-0.1123	0.0279	0.2792	0.2822
1895	S;S;S-30|32|	Q9Y2V2	Calcium-regulate	CARHSP1	1.0304	0.9922	0.8249	0.7627	0.6991	0.9993	0.7231	0.8963	0.8311	0.6598
1896	S;S;S-307|31	Q13769	THO complex sul	THOC5 C22c	-0.4374	-0.3655	-0.0426	-0.3093	-0.2825	-0.2777	-0.3671	-0.4721	-0.2387	-0.2853
1897	S;S;S-311|31	Q8TF01	Arginine/serine-r	PNISR C6orf	-1.2915	-1.3675	-1.3172	-1.7269	-1.0364	-1.1049	-0.9603	-0.8883	-1.2112	-0.9657
1898	S;S;S-316|32	Q9NY61	Protein AATF (Ap	AATF CHE1 [	0.6941	0.6010	0.4847	0.5445	0.6565	0.8302	0.6473	0.7123	0.5557	0.5548
1899	S;S;S-317|32	Q5C9Z4	Nucleolar MIF4G	NOM1 C7orf	0.4578	0.4098	0.4634	0.3294	0.3918	0.4554	0.2289	0.2298	0.3908	0.2946
1900	S;S;S-323|32	O43823	A-kinase anchor	AKAP8 AKAF	0.7129	0.8453	-0.1177	0.1788	-0.0883	-0.3905	0.2399	-0.2515	-0.3535	-0.0352
1901	S;S;S-330|33	Q9BW71	HIRA-interacting	HIRIP3	0.8764	0.8673	0.7896	0.8442	0.8720	0.6947	0.8409	0.6633	1.0455	1.0032
1902	S;S;S-331|33	Q14978	Nucleolar and co	NOLC1 KIAA	0.7744	0.9794	0.5553	0.8639	0.8240	0.9213	0.7001	0.8204	1.2315	1.0709
1903	S;S;S-332|33	Q9NYV4	Cyclin-dependen	CDK12 CRK7	0.2786	0.1311	0.1613	0.4580	0.5698	0.2913	0.5081	0.4902	0.3937	0.4018
1904	S;S;S-34|41|	P19338	Nucleolin (Protei	NCL	-1.7394	-1.8319	-1.7038	-1.8627	-1.9203	-2.0030	-2.0408	-1.7027	-1.3337	-1.4617
1905	S;S;S-352|35	P13746	HLA class I histo	HLA-A HLAA	1.8897	1.8251	-0.6437	-0.4762	-0.8589	-0.7444	-1.0382	-1.1446	0.9961	0.6168
1906	S;S;S-353|36	Q9Y2W2	WW domain-bin	WBP11 NPW	0.1263	0.1552	0.2055	0.0779	-0.0288	0.4258	0.0759	0.4639	0.4328	0.1409
1907	S;S;S-357|36	Q8N554	Zinc finger prote	ZNF276 CEN	0.1196	0.2146	-0.6569	-0.5215	-0.0805	-0.7670	-0.1447	-0.6562	-0.5395	-0.5934
1908	S;S;S-362|36	Q96ST2	Protein IWS1 hor	IWS1 IWS1L	0.9258	1.9366	2.0420	2.1886	1.9805	2.1043	2.0722	2.0946	1.9136	1.9264
1909	S;S;S-364|37	Q9BRD0	BUD13 homolog	BUD13	0.7744	0.7823	0.7099	0.4092	0.8333	0.4379	0.6953	0.4583	0.6907	0.9240
1910	S;S;S-365|36	Q12874	Splicing factor 3A	SF3A3 SAP6	-0.6290	-0.9682	-0.8137	-0.6973	-0.8194	-0.5621	-0.8255	-0.7767	-0.7954	-1.0131
1911	S;S;S-366|36	Q7Z4V5	Hepatoma-deriv	HDGFL2 HD	0.6089	0.7090	0.3790	0.3588	0.4496	0.3543	0.4711	0.4716	0.2215	0.4259

1912	S;S;S-378|37	Q8N554	Zinc finger protei	ZNF276 CEN	0.4903	0.5742	0.6154	0.6617	0.7416	0.9948	0.5424	0.8135	0.6120	0.4777
1913	S;S;S-389|39	Q8IYB3	Serine/arginine r	SRRM1 SRM	1.3918	1.5217	0.4765	0.6101	0.7671	0.3873	0.7232	0.5051	0.3555	0.4839
1914	S;S;S-397|39	Q01831	DNA repair prote	XPC XPCC	1.1934	1.1813	1.1538	1.5514	1.6347	1.6942	1.6297	1.5921	0.8355	1.4023
1915	S;S;S-407|40	P23497	Nuclear autoanti	SP100	0.0987	-0.0189	0.0615	0.1503	-0.2762	0.1589	-0.2155	-0.3223	-0.3093	-0.3624
1916	S;S;S-419|42	Q9P2I0	Cleavage and pol	CPSF2 CPSF1	0.0945	0.3186	0.3620	0.1772	0.1344	0.2481	-0.1050	0.2363	0.2943	0.1594
1917	S;S;S-420|42	Q9NTZ6	RNA-binding pro	RBM12 KIAA	0.8304	1.0490	-0.0522	0.8293	0.1774	0.6584	0.6179	0.1356	-0.1784	-0.1082
1918	S;S;S-431|43	Q13435	Splicing factor 3E	SF3B2 SAP14	1.4479	1.3546	1.3878	1.3044	1.5084	1.2260	1.3128	1.3004	1.4825	1.5281
1919	S;S;S-435|43	Q9UQ35	Serine/arginine r	SRRM2 KIAA	1.1246	1.2953	0.6002	0.7752	1.1056	0.5939	1.0378	0.6458	0.7194	0.8869
1920	S;S;S-441|44	Q5SSJ5	Heterochromatir	HP1BP3	-0.7080	-0.6618	-0.9848	-0.7618	-0.3789	-0.5695	-0.9553	-0.8870	-1.1171	-1.0041
1921	S;S;S-455|45	Q7Z5L9	Interferon regula	IRF2BP2	-0.1504	-0.0496	-0.0697	-0.1637	0.0373	0.1005	-0.0475	-0.0169	-0.1579	0.0531
1922	S;S;S-466|46	O75925	E3 SUMO-proteir	PIAS1 DDXBI	-1.0366	-1.4498	-1.2238	-1.2206	-1.6772	-1.3444	-1.5707	-1.2032	-1.4533	-1.1735
1923	S;S;S-470|47	Q5JSH3	WD repeat-conta	WDR44	0.7074	0.7218	0.3222	0.2529	0.4434	0.4412	0.3524	0.5103	0.4352	0.5302
1924	S;S;S-488|49	Q9C0K0	B-cell lymphoma	BCL11B CTIP	-0.4753	-0.3978	0.1547	-0.0125	0.1774	0.2876	-0.3412	-0.0061	0.7043	0.7883
1925	S;S;S-518|52	P29590	Protein PML (Pro	PML MYL PP	0.4644	0.5593	0.6756	0.4106	0.5723	0.7148	0.3180	0.4062	0.6273	0.5742
1926	S;S;S-529|53	Q9UPN7	Serine/threonine	PPP6R1 KIAA	0.4352	0.4288	0.4234	0.3166	0.3635	0.6809	0.4196	0.5161	0.5243	0.2809
1927	S;S;S-57|60|	Q13442	28 kDa heat- and	PDAP1 HASF	0.7670	0.6844	0.9165	0.7804	0.8751	1.1676	0.8725	1.2447	0.9273	0.8236
1928	S;S;S-591|59	O43290	U4/U6.U5 tri-snF	SART1	-0.0628	-0.0404	-0.5813	-0.8370	-0.7589	-0.9078	-0.5980	-0.9818	-0.7954	-0.8826
1929	S;S;S-603|60	Q05209	Tyrosine-protein	PTPN12	0.0251	0.1710	0.0331	-0.0089	-0.5316	-0.3145	-0.4720	-0.3204	-0.5320	-0.5251
1930	S;S;S-615|61	Q9Y6X4	Soluble lamin-as	FAM169A KI	-0.0459	-0.2443	-0.0105	-0.2648	-0.0195	0.0838	-0.2228	-0.0077	0.0956	-0.0299
1931	S;S;S-635|63	Q9Y6X4	Soluble lamin-as	FAM169A KI	-0.3491	-0.3950	-0.0335	-0.0642	-0.3461	-0.1505	-0.2760	-0.3001	0.0713	-0.1346
1932	S;S;S-645|64	Q8N2M8	CLK4-associating	CLASRP SFRS	0.2621	-0.0055	0.5120	0.9252	0.7249	0.7532	0.7216	0.7228	0.4299	0.4656
1933	S;S;S-671|67	Q08945	FACT complex su	SSRP1 FACT8	1.2833	1.1294	1.5715	1.6047	1.3827	1.4757	1.3188	1.4703	1.6893	1.8861
1934	S;S;S-69|73|	Q9H4L4	Sentrin-specific p	SENP3 SSP3	-2.3923	-2.6197	-2.6937	-2.7567	-2.8194	-2.6090	-2.6338	-3.1145	-3.0490	-2.4867
1935	S;S;S-723|73	P98175	RNA-binding pro	RBM10 DXS8	0.0000	0.0088	0.2083	-0.3394	-0.1120	0.0782	-0.1050	-0.1311	0.0993	0.2379
1936	S;S;S-73|75|	Q7KZ85	Transcription elo	SUPT6H KIAA	-0.0348	0.0671	0.1992	-0.0636	-0.0844	0.0133	0.0155	-0.0899	0.0536	-0.1633
1937	S;S;S-738|74	Q8IYB3	Serine/arginine r	SRRM1 SRM	0.5417	0.6552	0.5611	0.9774	0.7642	0.6945	1.2805	0.9319	0.4732	0.6574
1938	S;S;S-752|75	Q8IYB3	Serine/arginine r	SRRM1 SRM	-0.2221	-0.0169	-0.5036	-0.0287	-0.1929	-0.4494	-0.0049	-0.4004	-0.7244	-0.4763
1939	S;S;S-778|78	Q9UQ35	Serine/arginine r	SRRM2 KIAA	0.5570	0.3195	0.2520	0.5114	0.5636	0.3046	0.6111	0.3617	0.2665	0.3940
1940	S;S;S-795|79	Q8IYB3	Serine/arginine r	SRRM1 SRM	1.2247	1.1969	0.8885	1.2568	1.0444	1.2387	0.9123	1.2486	0.9457	0.9707
1941	S;S;S-807|80	Q9BZF1	Oxysterol-bindin	OSBPL8 KIAA	0.6336	0.5653	0.5745	0.3657	0.2961	0.5427	0.4888	0.3158	0.1186	0.1629
1942	S;S;S-860|86	Q6GYQ0	Ral GTPase-activa	RALGAPA1 G	-0.9573	-0.8985	-0.4961	-0.5734	-0.6596	-0.3944	-0.8816	-0.7559	-0.8044	-0.8222
1943	S;S;S-914|91	Q9P107	GEM-interacting	GMIP	0.7741	0.7716	0.8181	0.8228	0.2096	0.3573	0.3096	0.4031	0.6755	0.6355
1944	S;S;S-95|97|	P62995	Transformer-2 pr	TRA2B SFRS1	0.0757	0.4411	0.7608	0.2746	0.8292	0.5579	0.8710	0.9510	0.9987	1.1531
1945	S;S;S-972|97	Q9UQ35	Serine/arginine r	SRRM2 KIAA	0.6617	0.9506	0.9169	1.3975	1.3421	1.1311	1.3122	1.1768	0.9887	1.2315
1946	S;S;S-98|106	Q14814	Myocyte-specific	MEF2D	-0.4066	-0.1941	-0.2275	-0.2248	-0.3307	-0.0654	-0.3087	0.0918	-0.3485	-0.8382
1947	S;S;S-99|102	P17096	High mobility grc	HMGA1 HM	1.2378	1.7225	1.1891	1.1999	1.4875	1.5914	1.4659	1.4251	1.2153	1.2237
1948	S;S;T-2018|2	Q9UQ35	Serine/arginine r	SRRM2 KIAA	0.7606	0.8866	0.6391	0.7288	0.6210	0.6270	0.7604	0.7942	0.7131	0.8244
1949	S;S;T-2030|2	Q9UQ35	Serine/arginine r	SRRM2 KIAA	0.0642	0.0037	0.0359	-0.0327	0.0268	-0.0328	0.0891	0.0801	0.0721	0.0898
1950	S;S;T-288|29	Q9UKM9	RNA-binding pro	RALY HNRPC	-0.0403	-0.0727	-0.3004	0.0627	-0.2784	-0.3749	-0.3745	-0.2790	-0.3974	-0.4777

1951	S;S;T-461|48	P18887	DNA repair prote	XRCC1	-1.4703	-1.2261	-1.9365	-1.7328	-1.5158	-1.4461	-1.5631	-2.1278	-1.5528	-1.4903
1952	S;S;T-475|48	P18887	DNA repair prote	XRCC1	-0.7274	-0.6437	-1.0275	-0.7387	-0.9592	-0.9735	-1.0065	-1.0372	-1.1292	-0.9859
1953	S;T-108|110	P35251	Replication facto	RFC1 RFC14(	-1.5688	-1.5243	-2.1246	-2.0197	-1.7620	-1.9970	-1.9131	-2.1822	-2.0982	-1.9066
1954	S;T-114|118	Q96T60	Bifunctional poly	PNKP	0.4774	0.3623	1.3055	0.6572	0.9205	0.8505	0.8718	0.7862	0.5134	0.4478
1955	S;T-1376|13	Q14669	E3 ubiquitin-prot	TRIP12 KIAA	0.1079	-0.0891	-0.6785	-0.6083	-0.7069	-0.7394	-0.7040	-0.9788	-0.9351	-0.9859
1956	S;T-161|165	Q1KMD3	Heterogeneous r	HNRNPUL2 I	0.4617	0.5274	0.3340	0.5114	0.3974	0.2888	0.6841	0.4870	-0.1449	0.1606
1957	S;T-1925|19	Q9UQ35;Q9	Serine/arginine r	SRRM2 KIAA	-1.7154	-2.1234	-1.9420	-1.5376	-1.2987	-2.3350	-1.5463	-1.8484	-1.9992	-1.8273
1958	S;T-2020|20	Q9UQ35	Serine/arginine r	SRRM2 KIAA	-2.5742	-2.5927	-2.1246	-2.6192	-1.7069	-2.6181	-2.5478	-2.1822	-2.2385	-1.8970
1959	S;T-2032|20	Q9UQ35	Serine/arginine r	SRRM2 KIAA	-2.5602	-2.2271	-2.6990	-2.4209	-2.3503	-2.6107	-2.4502	-2.3209	-2.6504	-2.4835
1960	S;T-326|327	P24723	Protein kinase C	PRKCH PKCL	1.2749	1.2312	1.6857	1.0089	1.3192	1.1116	1.8496	1.4432	0.8435	0.2746
1961	S;T-329|331	Q14676	Mediator of DNA	MDC1 KIAA(	-0.4956	-0.6594	-0.1395	-0.6274	-0.4590	-0.2583	-0.2927	-0.3377	0.0091	-0.1166
1962	S;T-337|338	Q4G0J3	La-related protei	LARP7 HDCN	0.8489	1.0328	0.3252	0.5182	0.4522	0.3461	0.3506	0.4170	-0.0743	0.0893
1963	S;T-355|356	Q9Y2K1	Zinc finger and B	ZBTB1 KIAA(	-0.0274	0.0198	-0.5534	-0.3304	-0.4215	-0.3238	-0.4470	-0.8274	-0.7976	-0.8498
1964	S;T-356|358	Q13427	Peptidyl-prolyl ci	PPIG	0.0877	0.1532	-0.0285	-0.1283	-0.1421	-0.5820	-0.0144	-0.1598	-0.1977	-0.0418
1965	S;T-358|365	Q92994	Transcription fac	BRF1 BRF GT	-0.6318	-0.7477	-0.6275	-0.9221	-0.7311	-0.5040	-0.6809	-0.4212	-0.5133	-0.4706
1966	S;T-376|378	Q14676	Mediator of DNA	MDC1 KIAA(	0.3356	0.4222	0.0331	-0.0125	-1.0942	-1.0180	-0.1036	-0.1892	-0.1917	-0.1124
1967	S;T-385|389	P35269	General transcrip	GTF2F1 RAP	0.7029	0.8229	0.3232	0.4875	0.3692	0.1550	0.4767	0.2193	0.1400	0.0813
1968	S;T-397|402	Q9NYF8	Bcl-2-associated	BCLAF1 BTF	1.1403	0.9967	0.9537	0.9082	0.7580	0.7829	0.7915	0.9597	0.9777	0.8149
1969	S;T-402|406	Q8IYB3	Serine/arginine r	SRRM1 SRM	1.4890	1.5464	1.4950	1.4266	1.3806	1.5919	1.4432	1.5956	1.5858	1.3643
1970	S;T-420|426	Q9Y606	tRNA pseudouric	PUS1 PP898	-2.2056	-2.1979	-1.7630	-2.1878	-2.2562	-1.9106	-2.2168	-1.8658	-2.3206	-1.8591
1971	S;T-485|488	P18887	DNA repair prote	XRCC1	-0.4187	-0.3790	-0.6114	-0.3326	-0.6223	-0.6763	-0.6238	-1.0372	-0.9832	-0.4867
1972	S;T-492|494	Q6UN15	Pre-mRNA 3'-enc	FIP1L1 FIP1 I	-0.9678	-0.4881	-0.6852	-0.6777	-0.9238	-0.6480	-0.7535	-0.8911	-0.8459	-0.7752
1973	S;T-577|583	Q9UQ88	Cyclin-dependen	CDK11A CDC	0.1655	0.0639	0.1594	0.4567	0.0987	0.5117	0.4813	0.6458	0.5697	0.3613
1974	S;T-596|599	Q3YEC7	Rab-like protein	RABL6 C9orf	-1.2182	-1.1382	-1.4597	-1.3782	-1.6365	-1.4872	-1.6178	-1.9758	-1.8624	-1.4027
1975	S;T-710|711	Q8IX21	SMC5-SMC6 com	SLF2 C10orf(	0.5322	0.4484	0.0824	0.2221	0.0426	0.0177	0.1873	-0.1194	-0.2837	0.0293
1976	S;T-725|727	Q8IYB3	Serine/arginine r	SRRM1 SRM	-1.9055	-2.3818	-0.7056	-0.5734	-0.5721	-0.5019	-0.4311	-0.3052	-0.6993	-0.7555
1977	S;T-770|774	Q02446	Transcription fac	SP4	-0.7668	-1.0599	-1.2068	-1.4109	-1.6713	-1.3983	-1.7083	-1.6159	-1.4604	-1.3591
1978	S;T-790|793	Q4LE39	AT-rich interactiv	ARID4B BRC.	0.4955	0.6418	0.8308	0.7373	0.4670	0.8120	0.5618	0.4727	0.7729	0.5854
1979	S;T-808|810	Q9UQ35;Q9	Serine/arginine r	SRRM2 KIAA	-5.0553	-4.4169	-1.7701	-1.3016	0.0515	-0.7645	-0.9204	-1.2424	-1.4967	-1.3723
1980	S;T-855|856	Q9H0E3	Histone deacetyl	SAP130	0.2964	0.3221	0.1928	0.0490	0.2901	0.4434	0.1303	0.3402	0.5591	0.3009
1981	S;T-915|917	Q9P1Y6	PHD and RING fir	PHRF1 KIAA1	-0.7036	-0.3775	-0.4771	-1.1919	-1.2784	-1.1372	-1.1840	-1.0785	-1.0170	-0.4885
1982	S;T;S-105|1(	Q8NE71	ATP-binding cass	ABCF1 ABC5	0.9007	0.8471	1.0227	0.8733	0.8913	1.1003	0.6884	0.7307	0.9706	0.6183
1983	S;T;S-161|1(	Q1KMD3	Heterogeneous r	HNRNPUL2 I	0.7228	0.9766	0.3742	0.1646	0.2581	0.2864	0.2992	-0.1513	0.2192	0.2499
1984	S;T;S-1854|1	Q9UQ35	Serine/arginine r	SRRM2 KIAA	1.1879	1.6844	1.3415	1.1030	1.6454	1.0243	1.8890	1.4336	1.3133	1.4331
1985	S;T;S-2067|2	Q9UQ35	Serine/arginine r	SRRM2 KIAA	0.2934	0.5057	0.3974	0.4173	0.2871	0.3391	0.2520	0.4004	0.4173	0.5559
1986	S;T;S-889|89	Q9BTW9	Tubulin-specific c	TBCD KIAA0	-2.8329	-3.1382	-2.7559	-3.1558	-3.1060	-2.9106	-2.7339	-2.2753	-2.4930	-2.2851
1987	S;T;Y-113|1	P58335	Anthrax toxin rec	ANTXR2 CM	-0.2461	-0.2057	0.0769	-0.0399	-0.3028	-0.0102	-0.2054	-0.1825	-0.3193	-0.2201
1988	S;Y-53|54_C	Q9Y2W1	Thyroid hormone	THRAP3 BCL	1.6405	2.4112	1.3303	1.2906	1.3382	0.8935	1.5675	1.1848	1.3464	1.5334
1989	T-1003_Q9U	Q9UQ35	Serine/arginine r	SRRM2 KIAA	0.4041	0.2854	0.9693	0.9091	0.8861	0.8447	0.7496	0.7603	1.3258	1.3381

1990	T-101_Q9UK	Q9UKK3	Protein mono-AD	PARP4 ADPR	0.6148	0.5072	0.3612	0.6595	0.1903	0.4147	0.2399	0.4016	0.6704	0.7771
1991	T-103_Q9H8	Q9H8M2	Bromodomain-co	BRD9 UNQ3	0.6370	0.6432	0.7534	0.5151	0.5436	0.7975	0.7489	0.8655	0.8075	0.4211
1992	T-104_Q148	Q14847	LIM and SH3 don	LASP1 MLN5	0.8666	0.7425	0.7509	0.8901	0.3169	0.8035	0.6137	0.8290	0.5556	0.5142
1993	T-118_Q96T	Q96T60	Bifunctional poly	PNKP	0.3154	0.2744	0.6571	0.6539	0.5599	0.7093	0.4579	0.5310	-0.2022	0.1114
1994	T-122_P384	P38432	Coilin (p80-coilin	COIL CLN80	-0.0899	-0.0611	-0.1266	-0.1397	-0.0043	0.1125	-0.0305	0.0045	0.0187	-0.1599
1995	T-1220_O43	O43426	Synaptojanin-1 (I	SYNJ1 KIAA0	0.2115	0.2031	0.5829	0.7416	0.4684	0.5488	0.4147	0.3438	0.3454	0.1479
1996	T-1305_Q96	Q96T23	Remodeling and	RSF1 HBXAP	-1.8785	-1.7865	-2.2886	-2.1401	-2.1721	-2.6232	-2.2722	-2.5156	-2.2931	-2.3973
1997	T-134_Q7LD	Q7LDG7	RAS guanyl-relea	RASGRP2 CD	-0.0837	-0.0212	1.4014	2.0871	1.4717	1.8750	2.0065	2.0665	1.6175	1.4717
1998	T-135_Q9BR	Q9BRD0	BUD13 homolog	BUD13	-2.9964	-2.6197	-3.4284	-3.8822	-3.1544	-3.2353	-3.3103	-3.0123	-3.1150	-3.5307
1999	T-139_Q8W	Q8WW12	PEST proteolytic	PCNP	0.2255	0.3142	-0.2773	0.2553	-0.3055	0.1198	-0.3678	-0.0031	0.0091	0.1019
2000	T-139_Q96M	Q96MW1	Coiled-coil doma	CCDC43	-0.0292	-0.0576	0.0177	-0.1109	-0.2964	0.0554	-0.2675	-0.0785	0.0296	-0.0247
2001	T-1440_Q96	Q96BY6	Dedicator of cyto	DOCK10 KIA	0.5910	0.4492	-0.0863	0.1788	-0.1605	0.7515	0.4739	0.5043	0.0956	0.0468
2002	T-1468_Q7Z	Q7Z6E9	E3 ubiquitin-prot	RBBP6 P2PR	0.7413	0.6066	0.5787	0.9839	0.9049	0.7699	0.7520	0.8169	0.6298	0.5232
2003	T-148_Q96M	Q96MU7	YTH domain-cont	YTHDC1 KIA	-2.1167	-2.4407	-0.7297	-1.0197	-0.7250	-0.4002	-0.9987	-0.6205	-0.1887	-0.1490
2004	T-149_Q9H7	Q9H7E2	Tudor domain-co	TDRD3	-5.1167	-6.0997	-5.4742	-4.6192	-4.6138	-5.7519	-4.2350	-5.4232	-5.2082	-5.4301
2005	T-1492_Q9U	Q9UQ35	Serine/arginine r	SRRM2 KIAA	-0.7184	-0.6334	-1.4713	-0.9876	-1.0669	-1.3944	-1.0435	-0.8520	-1.2949	-1.0699
2006	T-169_Q131	Q13177	Serine/threonine	PAK2	-0.0329	-0.1104	0.1166	-0.0994	-0.0496	0.1945	-0.0157	0.1740	0.3320	-0.0958
2007	T-1733_Q9B	Q9BTC0	Death-inducer ol	DIDO1 C20o	-0.1128	-0.2042	-0.0952	0.0963	0.1806	-0.0438	-0.1722	-0.3808	0.1090	0.1721
2008	T-174_Q137	Q13765	Nascent polypep	NACA HSD4	0.6681	0.7983	-0.6034	-0.0397	-0.2279	-0.0060	-0.1795	-0.1211	-0.3871	-0.2587
2009	T-181_O438	O43829	Zinc finger and B	ZBTB14 ZFP	-1.0478	-1.1406	-0.6356	-0.7007	-0.5237	-0.3827	-1.0760	-0.8742	-0.3905	-0.2102
2010	T-183_P247	P24723	Protein kinase C	PRKCH PKCL	-0.6977	-0.9704	-1.7314	-0.8921	-1.8194	-1.4022	-1.1664	-1.2244	-1.6597	-1.9657
2011	T-195_Q155	Q15527	Surfeit locus prot	SURF2	-0.3595	-0.5274	-0.2262	-0.4015	0.1610	0.1630	0.0129	0.0889	0.0104	-0.5288
2012	T-206_Q96P	Q96PK6	RNA-binding pro	RBM14 SIP	0.1785	0.0412	0.1300	0.1365	0.1596	0.0986	0.0846	0.1091	0.2271	0.4837
2013	T-208_P421	P42167	Lamina-associate	TMPO LAP2	0.2455	0.2881	0.5582	0.6929	0.6222	0.7881	0.9116	0.6662	0.3341	0.3613
2014	T-2083_Q9U	Q9UMN6	Histone-lysine N-	KMT2B HRX	-0.9785	-0.8841	-0.7630	-0.9982	-0.8324	-1.0270	-0.7800	-0.9818	-0.6312	-0.8685
2015	T-21_Q9Y3Z	Q9Y3Z3	Deoxynucleoside	SAMHD1 MO	-0.0609	-0.1406	-0.2140	-0.2373	-0.4615	-0.2907	-0.4488	-0.3630	-0.5338	-0.2464
2016	T-211_O151	O15173	Membrane-asso	PGRMC2 DG	1.3682	1.5798	NA	NA	-0.0844	0.2937	-0.0856	0.3232	-0.2758	-0.3740
2017	T-215_Q7L1	Q7L190	Developmental p	DPPA4	-0.5768	-0.5532	-0.2250	-0.2542	-0.9308	-0.7027	-0.5459	-0.7127	-0.5817	-0.4336
2018	T-220_Q8IYE	Q8IYB3	Serine/arginine r	SRRM1 SRM	1.5679	1.6787	0.8915	0.8870	1.2533	0.8939	1.0718	0.8751	0.9250	0.9139
2019	T-223_Q9BX	Q9BXS5	AP-1 complex su	AP1M1 CLTN	-0.4779	-0.3114	-0.4815	-0.4062	-0.4264	-0.0577	-0.5177	-0.3319	-0.2187	-0.2147
2020	T-228_Q161	Q16181	Septin-7 (CDC10	SEPTIN7 CD	-0.9202	-1.1004	0.1267	-0.6862	-0.1487	-0.4519	-0.6240	-0.6399	0.8727	1.0087
2021	T-2320_Q01	Q01082	Spectrin beta cha	SPTBN1 SPT	-0.0685	0.3777	-0.1395	-0.1899	-0.3588	0.5396	0.2487	0.2923	0.5503	0.4393
2022	T-235_P176	P17676	CCAAT/enhancer	CEBPB TCF5	-0.3735	-0.3032	-0.1522	-0.2352	-0.1792	-0.0165	-0.3661	-0.4335	0.1126	0.0331
2023	T-242_O751	O75113	NEDD4-binding p	N4BP1 KIAA	1.3682	1.2127	1.2595	1.3435	1.0683	1.2055	1.1969	1.1432	1.0877	1.0071
2024	T-254_Q53G	Q53GL0	Pleckstrin homol	PLEKHO1 CK	1.0233	0.8991	0.4451	0.6044	0.3477	1.0915	0.4878	0.5172	0.2922	0.1947
2025	T-262_O757	O75791	GRB2-related ad	GRAP2 GAD	-2.4956	-2.5632	-1.9163	-1.9775	-1.7714	-1.8460	-2.0921	-1.9687	-1.8595	-2.0425
2026	T-267_P550	P55081	Microfibrillar-ass	MFAP1	-0.4728	-1.0832	-0.1733	-0.3782	-0.1300	-0.3053	-0.2879	-0.4989	-0.1392	0.1652
2027	T-271_P279	P27987	Inositol-trisphos	ITPKB	0.9634	0.8618	0.9429	0.9205	0.9852	1.2387	0.9511	1.1025	1.0852	0.8481
2028	T-280_O607	O60759	Cytohesin-intera	CYTIP PSCDE	0.5247	0.4214	1.3013	0.8122	1.4347	0.7093	1.6489	0.7098	1.2080	0.8388

2029	T-287_Q135	Q13555	Calcium/calmodu	CAMK2G CA	0.9937	0.7635	-0.1852	1.4715	-0.1482	1.2126	1.3677	1.2524	-0.1221	-0.4095
2030	T-287_Q135	Q13557	Calcium/calmodu	CAMK2D CA	0.7478	0.8428	0.4581	2.3965	1.2635	1.9358	2.5960	2.0889	0.3566	-0.9457
2031	T-291_P041	P04114	Apolipoprotein B	APOB	-1.4211	-1.3308	1.0755	-0.4228	0.6412	-1.3482	0.3232	-1.0785	-1.0463	-1.3723
2032	T-302_Q9BR	Q9BRQ0	Pygopus homolo	PYGO2 PP79	0.8735	0.8679	0.8077	0.5915	0.5649	0.4897	0.5750	0.6202	0.9497	0.6999
2033	T-309_Q86Y	Q86YS7	C2 domain-conta	C2CD5 CDP1	1.2029	1.2890	1.5244	1.2590	1.5593	1.1490	1.2907	1.1418	1.0723	0.7149
2034	T-314_Q8TB	Q8TB03	Uncharacterized	CXorf38	0.7445	1.0171	-0.2522	-0.0269	-0.0515	-0.2214	-0.1332	-0.4903	-0.5778	-0.3443
2035	T-328_Q925	Q92597	Protein NDRG1 (I	NDRG1 CAP4	-0.8954	-1.0122	0.2405	-0.0452	0.2348	-0.1471	0.4588	-0.1857	-0.1917	-0.3459
2036	T-33_P2798	P27987	Inositol-trisphos	ITPKB	-0.3735	-0.3142	-0.0952	0.0421	-0.2673	-0.0423	-0.1605	-0.1446	-0.1179	0.0518
2037	T-330_P0C7	P0C7T5	Ataxin-1-like (Br	ATXN1L BOA	-0.5317	-0.6334	-0.7366	-0.4517	-0.6365	-0.4810	-0.5863	-0.4669	-0.2790	-0.2162
2038	T-34_P6232	P62328	Thymosin beta-4	TMSB4X TB4	0.1542	-0.1188	0.2290	-0.0107	-0.1917	-0.1871	-0.1022	-0.0641	0.1830	0.1256
2039	T-358_Q134	Q13427	Peptidyl-prolyl ci	PPIG	-1.5634	-1.5532	-1.9689	-1.6301	-1.4794	-1.6692	-1.4276	-1.6879	-1.6888	-1.6959
2040	T-365_Q929	Q92994	Transcription fac	BRF1 BRF GT	-1.3049	-1.3675	-1.2509	-1.4787	-1.3146	-1.1274	-1.2198	-1.0850	-0.9401	-1.0405
2041	T-376_Q9C0	Q9C0K0	B-cell lymphoma	BCL11B CTIP	-0.5501	-0.3046	-0.6751	-0.7994	-0.8357	-0.9332	-0.6582	-0.9314	-0.6212	-0.6208
2042	T-393_Q9UK	Q9UKV3	Apoptotic chrom	ACIN1 ACINU	0.4852	0.8722	0.8314	0.8756	-0.0288	-0.2145	0.6849	0.7684	1.1899	0.9562
2043	T-394_P365	P36507	Dual specificity m	MAP2K2 ME	0.4155	0.1830	0.7241	0.8026	0.7404	0.8534	0.7923	0.8014	0.2988	0.1668
2044	T-398_O435	O43516	WAS/WASL-inter	WIPF1 WASI	-0.3876	-0.3905	-0.9425	-0.3552	-0.4439	-0.0623	-0.2457	-0.0705	-0.4111	-0.4441
2045	T-4030_Q15	Q15149	Plectin (PCN) (PL	PLEC PLEC1	-0.8854	-1.2522	0.1575	-0.9771	0.2333	-1.4220	0.3822	-0.0466	-0.9426	-0.9632
2046	T-4100_Q09	Q09666	Neuroblast differ	AHNAK PM2	-0.6179	-0.4711	-0.6735	-0.7036	-1.0746	-0.7874	-0.9935	-0.9939	-0.9961	-1.1193
2047	T-416_Q8IY	Q8IYB3	Serine/arginine r	SRRM1 SRM	-4.6402	-3.5274	-3.0533	-3.1878	-2.8064	-3.4956	-3.1988	-3.8166	-3.0816	-2.9910
2048	T-417_Q9C0	Q9C0K0	B-cell lymphoma	BCL11B CTIP	-0.1995	-0.1872	-0.2960	-0.1447	-0.0674	-0.0030	-0.3082	-0.1050	0.3128	0.3588
2049	T-418_Q9BR	Q9BRZ2	E3 ubiquitin-prot	TRIM56 RNF	1.2523	1.1056	1.1315	1.1534	1.3095	1.3291	1.2492	1.3365	1.2012	0.9213
2050	T-426_Q161	Q16181	Septin-7 (CDC10	SEPTIN7 CDC	0.8426	0.8818	0.8559	0.7846	0.4401	0.6260	0.6945	0.7449	0.5189	0.1721
2051	T-426_Q9Y6	Q9Y606	tRNA pseudouric	PUS1 PP898	-0.1994	-0.1212	-0.0211	-0.1838	-0.1320	-0.0392	-0.0651	-0.0185	-0.0316	-0.0312
2052	T-447_Q9P2	Q9P2N5	RNA-binding pro	RBM27 KIAA	-3.7576	-3.7477	-3.3409	-3.4347	-2.9133	-3.0515	-3.5631	-3.4903	-3.5003	-2.8922
2053	T-450_Q152	Q15233	Non-POU domain	NONO NRB5	-0.3342	-0.1467	-0.3369	-0.3735	-0.5667	-0.3866	-0.3265	-0.3435	-0.0449	-0.1376
2054	T-453_P188	P18887	DNA repair prote	XRCC1	1.4137	1.4476	1.5642	1.6201	1.3848	1.5948	1.3201	1.3800	1.3300	1.4577
2055	T-454_Q8N1	Q8N163	Cell cycle and ap	CCAR2 DBC1	-0.6516	-0.4635	-0.2584	-0.5343	-0.7558	-0.7569	-0.6830	-0.4397	-0.3320	-0.2464
2056	T-458_O608	O60832	H/ACA ribonucle	DKC1 NOLA4	0.8513	0.8548	0.4757	0.5624	0.8128	0.7773	0.5109	0.5978	0.7798	0.8048
2057	T-475_Q8TD	Q8TDZ2	[F-actin]-monoox	MICAL1 MIC	0.9362	0.9481	0.9921	1.0071	1.1453	0.8238	1.1315	0.7693	0.6137	0.6360
2058	T-4766_Q09	Q09666	Neuroblast differ	AHNAK PM2	0.4813	0.5396	-0.0708	0.0678	-0.1523	-0.1670	-0.1781	-0.3610	-0.4515	-0.3541
2059	T-51_Q5SSJ	Q5SSJ5	Heterochromatin	HP1BP3	1.0945	1.3824	1.5064	1.4580	1.9283	1.5514	1.6318	1.5161	1.2740	1.2978
2060	T-526_Q6PK	Q6PKG0	La-related protei	LARP1 KIAA	1.0419	1.0989	0.5546	0.8492	0.9068	0.9268	1.0289	0.7247	0.7918	0.8862
2061	T-528_Q9NF	Q9NPI6	mRNA-decapping	DCP1A SMIF	0.1395	0.2108	0.1697	0.2598	0.0119	0.2418	-0.0408	0.0875	0.0524	0.0331
2062	T-53_Q96NI	Q96NI6	Leucine-rich repe	LRFN5 C14o	-0.7214	-0.7038	0.0382	0.1804	0.6483	0.3485	0.2530	0.4946	0.1899	0.2883
2063	T-544_Q9BX	Q9BXP5	Serrate RNA effe	SRRT ARS2 A	0.1724	0.1715	0.0050	0.2541	0.0504	0.0613	0.0072	0.0592	0.1562	0.4309
2064	T-556_Q151	Q15111	Inactive phospho	PLCL1	-0.7668	-0.7514	-0.5907	-0.7358	-0.4539	-0.3983	-0.5785	-0.3709	-0.4622	-0.4180
2065	T-584_Q9UF	Q9UPR0	Inactive phospho	PLCL2 KIAA1	0.6284	0.5699	0.8257	0.5201	0.5494	0.9065	0.8467	0.9880	0.7769	0.5325
2066	T-614_Q8IY	Q8IYB3	Serine/arginine r	SRRM1 SRM	-0.5405	-0.6186	-0.3121	-0.2373	-0.2162	-0.3508	-0.3518	-0.3085	-0.3310	-0.3179
2067	T-620_Q2M	Q2M2I8	AP2-associated p	AAK1 KIAA1	0.5989	0.6002	0.9926	0.7995	1.1310	1.1333	0.6683	0.7717	0.8864	0.7424

2068	T-671_Q96C	Q96QT6	PHD finger prote	PHF12 KIAA1	0.3370	0.3327	-0.0436	0.0264	0.0083	-0.1033	0.0410	-0.1530	0.1281	0.1315
2069	T-682_Q9UK	Q9UKV3	Apoptotic chrom	ACIN1 ACINU	-0.2871	-0.3322	-0.7227	-0.5761	-0.6138	-0.3053	-0.5765	-0.4627	-0.5605	-0.4267
2070	T-692_Q9NY	Q9NYV4	Cyclin-dependen	CDK12 CRK7	-0.5823	-0.5745	-0.5110	-0.7507	-0.6194	-1.0180	-0.5707	-0.8826	-0.9755	-0.9309
2071	T-723_Q5VT	Q5VT52	Regulation of nu	RPRD2 KIAA	0.5765	0.7498	0.5603	0.6009	0.5181	0.4269	0.5126	0.5342	0.3647	0.6384
2072	T-748_Q134	Q13427	Peptidyl-prolyl ci	PPIG	-0.8721	-0.8221	-0.2092	-0.1283	-0.5184	-0.5061	-0.7275	-0.4543	-0.6192	-0.2898
2073	T-754_Q6GY	Q6GYQ0	Ral GTPase-activ	RALGAPA1 G	-1.8655	-2.0484	-0.0222	-0.6551	-0.1708	-1.1049	-0.4311	-0.8547	-0.4008	-0.7752
2074	T-754_Q9C0	Q9C0K0	B-cell lymphoma	BCL11B CTIP	0.0706	0.0143	-0.6114	-0.2289	-0.2762	-0.5909	-0.4883	-0.7177	-0.4268	-0.0971
2075	T-8_P45973	P45973	Chromobox prot	CBX5 HP1A	0.3752	0.5779	0.5164	0.4476	0.5736	0.7202	0.4244	0.5140	0.8127	0.5690
2076	T-84_Q9BW	Q9BW71	HIRA-interacting	HIRIP3	-1.9191	-2.3818	-2.3840	-2.8181	-2.1300	-2.3444	-2.4810	-2.1412	-1.9007	-2.2541
2077	T-848_Q9UC	Q9UQ35	Serine/arginine r	SRRM2 KIAA	-2.1726	-1.9639	-2.1155	-1.9020	-1.8927	-1.7271	-2.0732	-1.9878	-1.9501	-1.9066
2078	T-852_O954	O95425	Supervillin (Arch	SVIL	-0.3829	0.0436	-0.8642	-0.0018	-0.4439	1.0583	0.6985	0.5363	-0.4515	-0.3120
2079	T-866_Q9UC	Q9UQ35	Serine/arginine r	SRRM2 KIAA	-1.5422	-1.1928	-1.7489	-1.7627	-2.0364	-1.8133	-1.7601	-1.9939	-1.9302	-1.9066
2080	T-888_Q76F	Q76FK4	Nucleolar protei	NOL8 C9orf3	0.3569	0.3115	0.3156	0.2130	0.3621	0.4313	0.2189	0.1230	0.3788	0.2401
2081	T-899_Q9HC	Q9HC35	Echinoderm micr	EML4 C2orf2	-2.7948	-2.4650	-2.5095	-2.6749	-3.1708	-2.2145	-2.9180	-2.7637	-2.3939	-1.8222
2082	T-925_Q6PD	Q6PD62	RNA polymerase	CTR9 KIAA01	0.9708	0.9003	1.5378	1.2305	1.4523	1.1341	1.3336	1.3402	1.4154	1.3752
2083	T-947_Q140	Q14005	Pro-interleukin-1	IL16	0.5692	-0.0438	0.4273	0.1866	0.4842	0.3296	0.8109	0.9542	-0.0329	0.3782
2084	T-953_Q128	Q12802	A-kinase anchor	AKAP13 BRX	-0.3296	-0.4619	0.2137	0.0813	-0.0844	0.2087	-0.1988	0.0889	0.1773	0.0318
2085	T-99_Q0966	Q09666	Neuroblast differ	AHNAK PM2	1.1387	1.0734	1.5695	1.2993	1.0657	1.0059	1.2250	1.1039	0.4075	-0.0524
2086	T;S-101|102	Q9GZT3	SRA stem-loop-ir	SLIRP C14orf	1.8212	1.7697	1.0433	1.2006	0.8477	0.9377	1.3065	1.2134	0.7668	0.6143
2087	T;S-1010|10	Q96L91	E1A-binding prot	EP400 CAGH	0.4205	0.6425	-0.8738	-0.4468	-0.6654	-0.5468	-0.5140	-0.9578	-0.8250	-0.4476
2088	T;S-108|109	Q8NE71	ATP-binding cass	ABCF1 ABC5	0.5838	0.4876	1.0448	0.5592	0.9731	1.1603	0.8241	0.9295	0.9804	0.6655
2089	T;S-1171|11	Q13017	Rho GTPase-activ	ARHGAP5 RH	0.1312	0.1808	0.5432	0.4489	0.5142	0.5590	0.2949	0.2272	0.3607	0.3206
2090	T;S-1186|11	Q9Y3R5	Protein dopey-2	DOP1B C21o	-0.7979	-0.7969	-0.9628	-0.9597	-0.8423	-0.7721	-1.0435	-1.0031	-0.8838	-0.8041
2091	T;S-12|14_C	Q9NZC7	WW domain-con	WWOX FOR	-1.0478	-1.2923	-1.1200	-1.0674	-1.5970	-1.2214	-1.5289	-1.6300	-1.5226	-1.3790
2092	T;S-1305|13	Q96T23	Remodeling and	RSF1 HBXAP	-1.1564	-0.6559	-0.5065	-0.8181	-1.0213	-1.0060	-0.7756	-0.7767	-0.4444	-0.5232
2093	T;S-14|17_C	Q13761	Runt-related trar	RUNX3 AML	-0.5877	-0.7969	-0.6618	-1.1167	-0.5694	-0.4002	-0.2706	-0.4606	-0.7487	-0.7469
2094	T;S-147|162	P29692	Elongation factor	EEF1D EF1D	0.0708	0.3007	0.3704	0.2014	0.1121	0.4083	0.0566	0.3054	0.3278	0.1185
2095	T;S-159|163	Q9BRD0	BUD13 homolog	BUD13	0.1523	0.2321	-0.0266	-0.1503	0.0827	-0.0078	0.1759	-0.1643	-0.1216	0.0031
2096	T;S-165|168	Q1KMD3	Heterogeneous r	HNRNPUL2 I	0.9239	1.1612	0.8896	0.8462	0.6774	0.7725	1.0808	0.7933	0.6180	0.8214
2097	T;S-175|177	P33241	Lymphocyte-spec	LSP1 WP34	-0.1384	-0.0622	-0.5907	-0.1858	-0.0535	-0.4260	-0.2365	-0.2496	-0.4129	-0.3345
2098	T;S-202|204	Q9H1E3	Nuclear ubiquito	NUCKS1 NU	1.0427	0.9476	0.0784	1.2083	0.8323	0.6622	0.8049	0.9383	0.5547	0.5905
2099	T;S-258|260	Q63ZY6	Putative methylt	NSUN5P2 NS	1.6242	1.8116	1.5178	1.8182	1.8260	1.9063	1.7527	1.6536	1.5148	1.6409
2100	T;S-263|265	Q5T200	Zinc finger CCCH	ZC3H13 KIAA	-0.7080	-0.7254	-1.3253	-0.6152	-0.4249	-1.3450	-0.3698	-0.9468	-1.3698	-1.2197
2101	T;S-2738|27	Q9UQ35	Serine/arginine r	SRRM2 KIAA	-0.2783	0.0533	-0.2850	-0.4541	-0.1340	-0.3866	-0.0157	-0.1770	-0.1814	-0.1249
2102	T;S-376|381	Q9C0K0	B-cell lymphoma	BCL11B CTIP	0.3125	0.4517	-0.1534	0.2993	0.1871	0.0382	-0.0774	0.0347	0.2643	0.3742
2103	T;S-391|392	Q6GMV2	SET and MYND d	SMYD5 RAI1	-2.2739	-2.5728	-2.4340	-2.6749	-2.9414	-2.7721	-2.2784	-2.6442	-2.6972	-2.8638
2104	T;S-436|441	P85037	Forkhead box pr	FOXK1 MNF	0.4137	0.7550	0.3047	0.3726	0.1022	0.2506	0.5521	0.2633	0.4690	0.6872
2105	T;S-471|473	Q9NV70	Exocyst complex	EXOC1 SEC3	1.9909	2.1369	1.8947	2.1292	1.6460	1.9903	1.6816	1.7458	1.5432	1.4791
2106	T;S-553|554	Q9Y618	Nuclear receptor	NCOR2 CTG2	1.1021	1.0468	1.2573	1.8462	1.5064	1.5569	1.5702	1.5357	0.9232	1.0096

2107	T;S-581|583	Q8IYB3	Serine/arginine r	SRRM1 SRM	0.0368	0.2467	-0.1852	-0.0065	0.3719	-0.3402	0.5109	-0.0963	-0.3606	-0.1403
2108	T;S-609|616	Q8NDX5	Polyhomeotic-lik	PHC3 EDR3 I	0.2037	0.2944	-0.2311	0.0558	-0.1020	-0.2708	0.0422	-0.1412	-0.1681	-0.1306
2109	T;S-614|616	Q8IYB3	Serine/arginine r	SRRM1 SRM	0.9503	0.7810	0.6290	1.0652	1.1013	0.6231	1.1112	0.8630	0.4830	0.4431
2110	T;S-620|624	Q2M2I8	AP2-associated p	AAK1 KIAA1	2.1485	2.1747	1.9322	1.9205	2.0373	2.2459	2.1556	2.1563	1.8899	1.5485
2111	T;S-638|640	Q66K74	Microtubule-ass	MAP1S BPY2	0.5607	0.7319	-0.0599	-0.2103	-0.2651	-0.4360	-0.3265	-0.3242	-0.3386	-0.2087
2112	T;S-740|747	Q9ULL5	Proline-rich prot	PRR12 KIAA1	1.2286	1.2340	0.6478	0.6527	0.7828	0.6910	0.8618	0.4494	0.5261	0.5267
2113	T;S-84|87_C	Q9BW71	HIRA-interacting	HIRIP3	-0.5269	-0.3657	-0.6940	-0.4881	-0.7061	-0.8010	-0.5871	-0.9470	-0.8655	-0.8016
2114	T;S-872|874	Q8IYB3	Serine/arginine r	SRRM1 SRM	0.5384	0.6601	0.0218	0.2493	0.1839	-0.0180	0.4196	-0.0899	-0.1725	0.3319
2115	T;S-882|884	Q9NZM3	Intersectin-2 (SH	ITSN2 KIAA1	1.2957	1.2528	0.2970	0.6253	0.3749	0.9182	0.6857	0.6002	0.4222	0.3381
2116	T;S-888|890	Q76FK4	Nucleolar protei	NOL8 C9orf	-0.5161	-0.6282	-0.5275	-0.6467	-0.6308	-0.4340	-1.1810	-0.5972	-0.6618	-0.9859
2117	T;S-93|95_C	Q15111	Inactive phosphc	PLCL1	-0.1089	-0.1903	-0.0609	-0.0525	0.9982	1.3076	0.5415	0.7603	0.9810	0.6277
2118	T;S;S-120|1	Q8WXI9	Transcriptional r	GATAD2B KI	-1.3782	-1.6265	-1.1200	-1.2842	-1.5639	-1.1939	-1.6541	-1.4191	-1.1932	-1.0699
2119	T;S;S-120|1	Q8WXI9	Transcriptional r	GATAD2B KI	0.3583	0.2680	-0.2044	-0.1777	-0.5237	-0.5577	-0.4452	-0.2902	-0.3452	-0.3707
2120	T;S;S-138|1	Q8IXQ4	GPALPP motifs-c	GPALPP1 KIA	-0.2937	-0.2522	-0.5534	-0.8562	-0.8456	-0.6457	-0.7579	-0.7585	-0.4694	-0.4976
2121	T;S;S-151|1	Q9BTC0	Death-inducer ol	DIDO1 C20o	1.0215	1.0474	0.9580	0.8968	0.9674	0.9023	0.8234	0.8756	0.8486	0.6719
2122	T;S;S-202|2	Q9H1E3	Nuclear ubiquito	NUCKS1 NU	1.2160	1.0911	0.3997	0.8542	1.0228	0.6708	1.2249	1.0893	0.5575	0.6109
2123	T;S;S-2131|	O75643	U5 small nuclear	SNRNP200 A	1.2196	1.1922	0.3503	0.4968	0.0826	0.4050	0.2874	0.2706	0.3016	-0.0639
2124	T;S;S-58|61|	Q9NXX6	Non-structural m	NSMCE4A C	-2.5742	-2.3934	-2.3569	-2.6523	-2.7069	-2.4380	-2.5863	-2.4397	-2.3271	-2.3959
2125	T;S;S-793|7	Q8IYB3	Serine/arginine r	SRRM1 SRM	-0.0628	-0.1603	-0.3004	-0.1677	-0.2366	-0.2025	-0.3561	-0.2977	-0.3190	-0.2804
2126	T;T-190|195	Q15527	Surfeit locus prot	SURF2	0.3696	0.3692	0.4288	0.0880	0.6138	0.5549	0.6841	0.6882	0.2226	-0.1166
2127	T;T-207|211	O75533	Splicing factor 3	SF3B1 SAP1	1.1337	1.2917	0.9916	0.9456	0.7261	0.9007	0.9206	0.7782	0.8164	1.0179
2128	T;T-223|227	O75533	Splicing factor 3	SF3B1 SAP1	1.4955	1.8640	0.6126	0.8600	1.0613	0.8863	1.3002	0.8444	0.7178	0.9077
2129	T;T-230|231	Q8IWW6	Rho GTPase-activ	ARHGAP12	0.2068	-0.0257	-0.7314	-0.3782	-0.3564	-0.8291	-0.3265	-0.8274	-0.7184	-0.4319
2130	T;T-51|53_P	P17544	Cyclic AMP-depe	ATF7 ATFA	-0.5187	-0.4498	-0.4698	-0.4252	-0.1708	-0.3846	-0.1233	-0.4008	-0.3518	-0.6347
2131	T;T-572|574	Q8IYB3	Serine/arginine r	SRRM1 SRM	0.0282	0.2390	0.0234	0.1028	0.4903	-0.3712	0.7687	0.0860	-0.1400	0.2425
2132	T;T-897|899	Q9HC35	Echinoderm micr	EML4 C2orf	-1.4601	-1.5588	-1.1340	-1.2399	-1.5127	-1.3737	-1.5575	-1.3760	-0.9996	-0.8787
2133	T;T;T-223|2	O75533	Splicing factor 3	SF3B1 SAP1	0.6929	0.8938	-0.0328	0.4541	0.2223	0.3831	0.6017	0.4438	0.2665	0.2778
2134	Y-143_P161	P16104	Histone H2AX (H	H2AX H2AF	0.8745	1.0382	1.9374	2.4776	2.3287	1.4330	2.7359	2.6979	1.0993	1.4028
2135	Y-160_P325	P32519	ETS-related trans	ELF1	1.8011	1.6037	1.4443	1.4239	1.2597	1.2649	1.4417	1.2291	1.3128	1.4269
2136	Y-1874_P24	P24928	DNA-directed RN	POLR2A POL	0.8335	0.8323	0.8902	0.9812	0.8999	0.7881	0.9764	0.8495	1.0000	0.9877
2137	Y-260_P050	P05023	Sodium/potassiu	ATP1A1	-0.2959	0.0393	1.2923	0.0264	1.2254	-0.1356	-0.0039	-0.0657	0.9664	0.9694
2138	Y-269_Q8W	Q8WUB8	PHD finger prote	PHF10 BAF4	-2.2739	-2.1979	-1.9009	-1.9701	-1.8064	-1.7874	-2.0787	-1.9054	-1.8814	-2.1998
2139	Y-321_Q136	Q13627	Dual specificity t	DYRK1A DYR	0.7691	0.8354	-0.0941	0.2979	-0.2065	0.2985	0.0685	-0.0419	-0.4042	-0.2162
2140	Y-44_P0673	P06733	Alpha-enolase (E	ENO1 ENO1	-0.1089	-0.1541	-0.1121	-0.2457	-0.1360	0.0583	-0.0842	-0.2173	0.0968	0.0837
2141	Y-52_Q9UID	Q9UID6	Zinc finger protei	ZNF639 ZAS	0.8079	1.0171	1.0192	1.0412	1.0064	1.0704	0.8293	1.0810	0.9396	0.9125
2142	Y-571_O151	O15117	FYN-binding prot	FYB1 FYB SL	0.0126	-0.0055	-0.1212	0.0264	-0.3284	0.2556	0.1562	0.1875	-0.3837	-0.6031
2143	Y-694_P422	P42229	Signal transduce	STAT5A STA	1.9372	-1.3005	1.5797	-1.2248	-0.8161	1.9221	1.7885	-0.9788	1.7881	-1.9859
2144	Y-712_Q86Y	Q86YR7	Probable guanin	MCF2L2 DR	1.3668	1.3760	0.7756	0.8492	1.1725	1.1068	1.3211	1.2226	0.7249	0.3871
2145	Y-849_Q135	Q13523	Serine/threonine	PRPF4B KIA	0.6077	0.6810	0.4935	0.5139	0.2395	0.5909	0.2843	0.4672	0.5252	0.6549

2146	Y-88_Q1515	Q15154	Pericentriolar ma	PCM1	-0.2351	-0.3732	-0.4185	-0.9666	-0.5612	-0.3294	-0.5554	-0.0434	-0.6888	-0.4688
2147	Y;S-160|168	P32519	ETS-related trans	ELF1	0.8268	0.9171	0.4410	0.6909	0.1416	0.2044	0.5240	0.5287	0.3485	0.2857
2148	Y;S-1909|19	P24928;P24	DNA-directed RN	POLR2A POL	1.0619	0.9911	0.2729	0.4963	0.4790	0.2139	0.4118	0.1131	0.4774	0.3421
2149	Y;S;S-160|16	P32519	ETS-related trans	ELF1	-0.1849	-0.1541	-0.4185	-0.3552	-0.7620	-0.4872	-0.7756	-0.6395	-0.4802	-0.5721
2150	Y;T-400|401	Q86UW9	Probable E3 ubiq	DTX2 KIAA15	-1.0329	-1.9553	-1.7384	-1.5087	-1.7009	-1.3108	-2.0462	-1.7952	-2.2142	-1.2449

Supplemental Table 3.1. Log2/median normalized TMT reporter intensities of regulated proteins of 24 h IL-7 stimulation of human primary T cell total proteome

	Master.Prot	Description	Control_1	Primed_1	Control_2	Primed_2	Control_3	Primed_3	Control_4	Primed_4	Control_5	Primed_5	rank produ	log2 change
1	P00395	Cytochrom	-2.9879	-2.3416	-2.5541	-1.3377	-2.3520	-1.9151	-3.0521	-1.1444	-3.1699	-1.8893	0.0107	1.0976
2	Q8N960	Centrosom	-3.3099	-1.7632	-1.9315	-1.1312	-3.1405	-1.7225	-2.2088	-2.0465	-2.3032	-1.7194	0.0341	0.9022
3	Q9NVV4	Poly(A) RN	0.0121	0.4459	0.2888	0.8790	-0.2424	0.3607	-0.3322	0.2419	-0.5092	0.2135	0.0341	0.5848
4	Q9UM00	Calcium loa	-1.7451	-1.8171	-2.6487	-3.5236	-3.0150	-3.7225	-1.4813	-3.5850	-3.4500	-4.3487	0.0341	-0.9313
5	Q9Y3T9	Nucleolar c	-1.9099	-1.3839	-3.2460	-1.5976	-2.8995	-2.1375	-3.2816	-1.7294	-3.3268	-2.6991	0.0107	1.0233

Supplemental Table 3.2. Log2/median normalized TMT reporter intensities of regulated phosphorylation sites of 24 h IL-7 stimulation of human primary T cell phosphoproteome

	pSite.in.Mas	Master.Pro	Descriptior	Control_1	Primed_1	Control_2	Primed_2	Control_3	Primed_3	Control_4	Primed_4	Control_5	Primed_5	rank produ	log2 change
1	S-1401_P21	P21359	Neurofibro	-0.4162	-0.7092	1.3369	0.4463	1.4836	0.2556	1.4579	0.7854	0.5983	0.1766	0.0007	-0.7012
2	S-146_Q96A	Q96AT1	Uncharacte	-2.2652	-2.7110	-0.6034	-1.9562	-0.8995	-2.2708	-1.0144	-2.1547	-1.6272	-2.1419	0.0000	-0.9650
3	S-168_Q014	Q01433	AMP deam	-0.1167	-0.5978	1.4830	1.0447	1.3309	0.6017	1.1975	0.9589	1.2298	0.8410	0.0157	-0.4552
4	S-177_O753	O75396	Vesicle-tra	0.4929	0.2962	0.7137	-0.0287	0.5810	-0.2128	0.2617	-0.0061	-0.3045	-1.0431	0.0157	-0.5479
5	S-231_P017	P01732	T-cell surfa	-0.8622	-1.1627	1.9805	0.8263	1.8584	-0.6668	2.5122	0.8030	-0.1652	-0.3476	0.0062	-1.1743
6	S-379_P423	P42331	Rho GTPas	-2.1011	-2.1928	1.3000	-0.1637	0.8528	-1.3866	0.5557	-0.6854	-0.3618	-1.3394	0.0157	-1.2027
7	S-51_Q86YV	Q86YV0	RAS protei	-1.4520	-1.6002	0.0292	-0.6209	-0.1002	-0.7755	0.1485	-0.3737	-0.2548	-0.7480	0.0157	-0.4978
8	S-5830_Q09	Q09666	Neuroblast	-0.5396	-0.8221	1.4323	0.6482	0.8456	-0.2494	1.1279	0.4074	-0.1873	-0.3690	0.0111	-0.6128
9	S-625_Q7Z4	Q7Z4V5	Hepatoma-	-3.7823	-3.3364	-2.9608	-2.6412	-3.7558	-2.8884	-3.1634	-2.9169	-2.9858	-2.6876	0.0181	0.4355
10	S-76_Q0143	Q01433	AMP deam	-1.4691	-1.5356	0.7142	-0.4931	0.4188	-1.2389	0.1068	-1.0023	-0.8424	-1.3122	0.0322	-0.9021
11	S-974_Q140	Q14005	Pro-interle	-0.7547	-1.2410	1.6905	1.3277	1.7614	1.0263	1.9825	1.2737	1.2835	0.9280	0.0037	-0.5297
12	S;S-659|663	Q15334	Lethal(2) g	-0.5161	-0.7589	-0.1223	-0.4252	-0.0083	-0.8967	0.1124	-0.3223	-0.8113	-0.9885	0.0490	-0.4092
13	T-280_O607	O60759	Cytohesin-	0.5247	0.4214	1.3013	0.8122	1.4347	0.7093	1.6489	0.7098	1.2080	0.8388	0.0322	-0.5252
14	T-754_Q6GY	Q6GYQ0	Ral GTPase	-1.8655	-2.0484	-0.0222	-0.6551	-0.1708	-1.1049	-0.4311	-0.8547	-0.4008	-0.7752	0.0157	-0.5096

Supplemental Table 4.1. Raw Proteome discoverer output from the three replicates of Condition A

Checked	Confide	Sequence	Modifications	Contamin	Qvality PEP	Qvality q-va	Num	Num	Num	Master Pr	Master Protein I	Protein Ac	Num	Theo MHplus	Abundances	Abundances	Abundan	Abundan
FALSE	High	AEAESLYQSK		TRUE	0.00248	0.006854	1	1	1	P04264	Keratin, type II c	P04264	0	1125.54224	20021	5540.1	121.87	
FALSE	High	AGFAGDDAPF	1xLabel:13C(6	FALSE	0.0093099	0.006854	1	10	2	P60709	Actin, cytoplasm	A5A3E0; P	0	982.46841	222224	55269.7	61.42	50.51
FALSE	High	AGFAGDDAPRAVFPSIVGRPR		FALSE	0.208134	0.006854	1	9	1	P60709	Actin, cytoplasm	A5A3E0; P	1	2156.13595	40998	5349.2	52.81	
FALSE	High	AGLQFPVGR		FALSE	0.0195963	0.006854	2	15	1	Q16777; (	Histone H2A typ	Q16777; C	0	944.53123	2714467	503862.2	54.42	43.90
FALSE	High	AGLQFPVGRVHR		FALSE	0.156759	0.006854	1	12	1	Q16777	Histone H2A typ	Q16777; C	1	1336.75966	285714	57371.2	74	27.75
FALSE	High	AGYRDGFGAS	2xCarbamidoı	TRUE	3.35E-06	0.006854	1	1	9	sp	cRAP117 00000(	sp	1	2679.1522	6227066		30.05	
FALSE	High	AGYRDGFGAS	2xCarbamidoı	TRUE	1.96E-06	0.006854	1	1	12	sp	cRAP117 00000(	sp	2	3754.68202	8662712		66.1	
FALSE	High	AHVEHMQSLI	1xOxidation [I	FALSE	0.199724	0.006854	1	1	1	Q13029	PR domain zinc f	Q13029	0	2063.97025	4929521		50.4	
FALSE	High	ALAAAGYDVEKNNSR		FALSE	0.0388659	0.006854	1	5	1	P10412	Histone H1.4 OS	P16402; P	1	1578.78706	100211	40339.0	65	58.57
FALSE	High	AMGIMNSFVI	1xOxidation [I	FALSE	0.0085937	0.006854	2	14	2	Q16778; (	Histone H2B typ	Q16778; C	0	1759.8142	123953	35101.4	75.28	87.10
FALSE	High	AMGIMNSFVI	2xOxidation [I	FALSE	0.0018327	0.006854	2	14	2	Q16778; (	Histone H2B typ	Q16778; C	0	1775.80912	273366	61014.8	74.68	54.22
FALSE	High	AMGIMNSFVI	2xOxidation [I	FALSE	0.165343	0.006854	2	14	1	Q16778; (	Histone H2B typ	Q16778; C	0	1781.82925				
FALSE	High	ASGPPVSELITI	1xLabel:13C(6	FALSE	0.0529725	0.006854	1	3	2	P10412	Histone H1.4 OS	P16402; P	0	1204.68791	189156	61567.5	60.02	41.65
FALSE	High	AVKPKAAKPK		FALSE	0.186989	0.006854	2	3	1	P16401; P	Histone H1.5 OS	P16401; P	1	1037.68298	27457	15832.3	58.28	42.40
FALSE	High	AVTKYTSSK		FALSE	0.0847537	0.006854	2	12	1	Q16778; (	Histone H2B typ	Q16778; C	1	984.53604	26805	8365.2	19.39	42.38
FALSE	High	AYDNATAAVAK		TRUE	0.0012642	0.006854	1	1	3	sp	cRAP117 00000(	sp	0	1094.54766	8481269		38.8	
FALSE	High	AYDNATAAVA	1xCarbamidoı	TRUE	0.0124328	0.006854	1	1	2	sp	cRAP117 00000(	sp	1	3766.76871	313438		84.17	
FALSE	High	CDVDIRKDLYA	1xCarbamidoı	FALSE	0.175569	0.006854	1	2	1	P60709	Actin, cytoplasm	P60709; P	2	3101.50304				
FALSE	High	CELAAAMKR	1xCarbamidoı	TRUE	0.0605511	0.006854	1	1	2	cRAP078	Lysozyme C OS=	cRAP078	1	1049.52305	96483		58.33	
FALSE	High	CNKEVYFAER	1xCarbamidoı	FALSE	0.0015968	0.006854	1	1	4	P50238	Cysteine-rich prc	P50238	1	1315.60995	242991	31872.6	98.53	68.77
FALSE	High	CNKEVYFAERY	1xCarbamidoı	FALSE	0.0085937	0.006854	1	1	1	P50238	Cysteine-rich prc	P50238	2	1900.95856	15630	5005.5	120.38	
FALSE	High	CSQAVYAAEK	1xCarbamidoı	FALSE	0.0208968	0.006854	1	1	1	P21291	Cysteine and gly	P21291	0	1126.51974	50083		98.23	
FALSE	High	DGFGASGSCE	2xCarbamidoı	TRUE	0.0004086	0.006854	1	1	3	sp	cRAP117 00000(	sp	0	2231.92918	138686		37.31	
FALSE	High	DGQVINETSQHHDDLE		FALSE	0.0999876	0.006854	1	1	2	P08670	Vimentin OS=Hc	P08670	0	1836.79947				
FALSE	High	DIGFIKLD		FALSE	0.0717932	0.006854	1	1	1	P62273	40S ribosomal p	P62273	1	920.50876	55957	60636.6	93.8	42.14
FALSE	High	DIGFIKLD	1xLabel:13C(6	FALSE	0.0433158	0.006854	1	1	2	P62273	40S ribosomal p	P62273	1	926.52889				
FALSE	High	DNIQGITKPAIR		FALSE	0.0499101	0.006854	1	1	1	P62805	Histone H4 OS=I	P62805	0	1325.75357	47426		48.81	
FALSE	High	EKLQLISALTNI	2xLabel:13C(6	FALSE	0.231207	0.006854	1	1	1	O75071	EF-hand calcium	O75071	1	4321.26006				
FALSE	High	EIQTAVRLLLPGELAK		FALSE	0.0202745	0.006854	1	14	2	Q16778	Histone H2B typ	Q16778; C	1	1751.04255	1385639	377343.0	104.55	104.38
FALSE	High	EVQTAVRLLLPGELAK		FALSE	0.046153	0.006854	1	1	1	Q8N257	Histone H2B typ	Q8N257	1	1737.0269	53799	26140.8	99.03	27.11
FALSE	High	FFVAPFPEVFGK		TRUE	0.0489893	0.006854	1	1	1	cRAP013	Alpha-S1-casein	cRAP013	0	1384.72999	168258		45.02	
FALSE	High	FFVAPFPEVFGKEK		TRUE	0.0317443	0.006854	1	1	1	cRAP013	Alpha-S1-casein	cRAP013	1	1641.86754	313634		46.04	
FALSE	High	FSSCGGGGGS	1xCarbamidoı	TRUE	1.12E-05	0.006854	1	1	6	P04264	Keratin, type II c	P04264	0	1765.73471	531843		44.92	
FALSE	High	FSSSSGYGGGSSR		TRUE	0.0013912	0.006854	1	1	1	P35527	Keratin, type I cy	P35527	0	1235.52872	18525		88.41	
FALSE	High	FVNVVPTFGK		FALSE	0.246322	0.006854	1	1	1	P62861	40S ribosomal p	P62861	0	1107.61971		22128.2		129.91
FALSE	High	GAVHDVKDVLDSVL		FALSE	0.0144179	0.006854	1	1	1	P81605	Dermcidin OS=H	P81605	1	1466.78493	156044	16501.0		
FALSE	High	GFGGASGGGYSSSGGFGGGF		TRUE	0.246322	0.006854	1	1	1	P35527	Keratin, type I cy	P35527	0	4862.0799	36919		79.16	
FALSE	High	GGLYGGPSYC	1xCarbamidoı	TRUE	2.35E-05	0.006854	1	1	2	sp	cRAP117 00000(	sp	0	1727.78059	344903		63.81	
FALSE	High	GGLYGGPSYC	1xCarbamidoı	TRUE	0.0002336	0.006854	1	1	4	sp	cRAP117 00000(	sp	1	3593.60264	2521634		69.94	
FALSE	High	GGSGGSHGGGSGFGGESGGS		TRUE	3.21E-06	0.006854	1	1	1	P35527	Keratin, type I cy	P35527	0	3223.28157	7006		73.07	

FALSE	High	GGSGGSYGGGGSGGGYGGG:		TRUE	1.57E-06	0.006854	1	1	1	P35527	Keratin, type I cy	P35527	0	1791.72771				
FALSE	High	GGSISGGGYGSGGGK		TRUE	0.0004229	0.006854	1	1	3	P35908	Keratin, type II c	P35908	0	1197.54945	429455		80.26	
FALSE	High	GSLGGGFSSGGFSGGSFSR		TRUE	8.52E-06	0.006854	1	1	2	P13645	Keratin, type I cy	P13645	0	1707.77214	258308	7190.4	36.67	59.01
FALSE	High	GSSGGGCFGG	1xCarbamidoı	TRUE	0.0368815	0.006854	1	1	1	P13645	Keratin, type I cy	P13645	0	2342.98433	38311		62.82	
FALSE	High	GTDVQAWIR		TRUE	0.0543694	0.006854	1	1	1	cRAP078	Lysozyme C OS=	cRAP078	0	1045.54252	122815		55.84	
FALSE	High	GTGASGSFKLI	3xLabel:13C(6	FALSE	0.154538	0.006854	2	5	1	P16401; P	Histone H1.5 OS	P16401; P	2	1312.77176	52445	48797.8	137.31	127.49
FALSE	High	GYSLGNWVCA	1xCarbamidoı	TRUE	0.0021442	0.006854	1	1	1	cRAP078	Lysozyme C OS=	cRAP078	0	1325.63068	78526		66.56	
FALSE	High	GYTVLAAAATI	2xCarbamidoı	FALSE	0.0547751	0.006854	1	1	2	Q9P2S6	Ankyrin repeat a	Q9P2S6	0	3125.51428				
FALSE	High	HAVSEGTKAVTK		FALSE	0.0014791	0.006854	2	15	4	Q16778; (	Histone H2B typ	Q16778; (	1	1227.66918	2027029	377090.0	60.81	65.48
FALSE	High	HAVSEGTKAVTKYTSSK		FALSE	0.0003066	0.006854	2	12	10	Q16778; (	Histone H2B typ	Q16778; (	2	1793.9392	801608	322530.7	91.37	58.70
FALSE	High	HAVSEGTKAV	3xLabel:13C(6	FALSE	0.0139867	0.006854	2	12	4	Q16778; (	Histone H2B typ	Q16778; (	2	1811.99959				
FALSE	High	HGLDNYRGYS	1xCarbamidoı	TRUE	0.0488071	0.006854	1	1	2	cRAP078	Lysozyme C OS=	cRAP078	1	2181.02943	99002		85.4	
FALSE	High	HGLDNYRGYS	1xCarbamidoı	TRUE	0.0956824	0.006854	1	1	1	cRAP078	Lysozyme C OS=	cRAP078	2	3590.66182				
FALSE	High	HQGLPQEVLNENLLR		TRUE	0.061456	0.006854	1	1	1	cRAP013	Alpha-S1-casein	cRAP013	0	1759.94496	10911		82.56	
FALSE	High	HSGNITFDEIVNIAR		FALSE	0.0040327	0.006854	1	1	1	P30050	60S ribosomal p	P30050	0	1685.86056				
FALSE	High	KATGPPVSELI	2xLabel:13C(6	FALSE	0.0761627	0.006854	1	1	2	P16401	Histone H1.5 OS	P16401	1	1352.81865	34427	53720.5	68.92	80.08
FALSE	High	KGTVEGFEPAI	1xCarbamidoı	FALSE	0.0017041	0.006854	1	1	1	P37108	Signal recognitic	P37108	2	1952.04041	66307	23172.0		88.69
FALSE	High	KIEEIKDFLLTAR		FALSE	0.0113917	0.006854	1	1	2	P63173	60S ribosomal p	P63173	2	1575.91047	73116		12.06	
FALSE	High	KLPFQR		FALSE	0.111592	0.006854	1	5	1	P84243	Histone H3.3 OS	P84243; C	1	788.47773	78328	4456.7	41.54	67.25
FALSE	High	KTVTAMDVVYALKR		FALSE	0.0067315	0.006854	1	1	2	P62805	Histone H4 OS=I	P62805	2	1594.89852	75131	18065.0	69.36	49.18
FALSE	High	KVPQVSTPTLVEVSR		TRUE	0.002814	0.006854	2	2	2	cRAP087;	Serum albumin (	cRAP087;	1	1639.93775	36298		67.36	
FALSE	High	KYSQGNVSAVGVTYDGHTAL		TRUE	1.32E-07	0.006854	1	1	4	sp	cRAP117 00000(	sp	1	2324.16295	1164605		68.38	
FALSE	High	LALDIEIATYR		TRUE	0.0085937	0.006854	1	6	2	P04264	Keratin, type II c	P08729; P	0	1277.70998	10582		37.75	
FALSE	High	LGEHNIDVLEGNEQFINAAK		TRUE	4.93E-07	0.006854	1	1	3	cRAP112	Trypsin OS=Sus s	cRAP112	0	2211.10404	7712058		30.36	
FALSE	High	LGGSAVISLEG	1xLabel:13C(6	FALSE	0.0113917	0.006854	1	1	2	P23528	Cofilin-1 OS=Hoı	P23528	0	1346.79852	234457	43744.3	46.11	32.48
FALSE	High	IGRIEDVTPIPSDSTRR		FALSE	0.0269043	0.006854	1	1	1	P62263	40S ribosomal p	P62263	2	1912.02466				
FALSE	High	IHRHLK		FALSE	0.138266	0.006854	1	2	2	Q71UI9	Histone H2A.V C	Q71UI9; P	1	803.49987	276482		16.99	
FALSE	High	LKECCDKPLLE	2xCarbamidoı	TRUE	0.0224513	0.006854	1	1	1	cRAP087	Serum albumin (	cRAP087	1	1532.78111	13775		91.83	
FALSE	High	LLCGLLAER	1xCarbamidoı	FALSE	0.006095	0.006854	1	1	1	P14174	Macrophage mi	P14174	0	1044.58703	60850	130164.0	87.15	76.14
FALSE	High	IIIPEIQK	1xLabel:13C(6	FALSE	0.215364	0.006854	1	1	1	P55060	Exportin-2 OS=H	P55060	0	959.62312				
FALSE	High	LLLPGELAK		FALSE	0.105635	0.006854	2	15	3	Q16778; (	Histone H2B typ	Q16778; (	0	953.60299	852848		11.76	
FALSE	High	LLRKGNYAER		FALSE	0.0434779	0.006854	1	9	2	Q16777	Histone H2A typ	Q16777; (	2	1219.69058	131618	17218.2	54.7	36.76
FALSE	High	IITHPNFNGNTLDNDIMLIK		TRUE	0.0020716	0.006854	1	1	4	cRAP112	Trypsin OS=Sus s	cRAP112	0	2283.18018	3407499	11848.9	58.42	3.35
FALSE	High	IITHPNFNGNT	1xOxidation [I	TRUE	2.29E-05	0.006854	1	1	5	cRAP112	Trypsin OS=Sus s	cRAP112	0	2299.17509	1019261		55.4	
FALSE	High	IITHPNFNGNTLDNDIMLIKLS:		TRUE	0.0057335	0.006854	1	1	1	cRAP112	Trypsin OS=Sus s	cRAP112	1	3309.72599	56782		34.75	
FALSE	High	IMLKGDNITLL	1xLabel:13C(6	FALSE	0.002194	0.006854	1	1	1	P62304	Small nuclear rik	P62304	1	1751.96673	133169	182769.6	15.25	62.34
FALSE	High	IQVRLGEHNIDVLEGNEQFIN/		TRUE	0.15126	0.006854	1	1	1	cRAP112	Trypsin OS=Sus s	cRAP112	1	2707.4162	112527		39.29	
FALSE	High	LSAKPAPPKPEPRPK		FALSE	0.163014	0.006854	1	1	1	O00479	High mobility gr	O00479	0	1612.95334	45489		55.7	
FALSE	High	ISGLIYEETR		FALSE	0.0016591	0.006854	1	1	2	P62805	Histone H4 OS=I	P62805	0	1180.62083	185311	7845.3	15.46	108.96
FALSE	High	LVAASQAALGL		TRUE	0.0073214	0.006854	1	1	1	P02768	Serum albumin (	P02768	0	1013.59897				
FALSE	High	LVREIAQDFKTDLR		FALSE	0.0720589	0.006854	1	3	1	P84243	Histone H3.3 OS	P84243; P	2	1703.94389				
FALSE	High	LVSDLK	1xLabel:13C(6	FALSE	0.213174	0.006854	1	1	1	Q15528	Mediator of RN/	Q15528	0	680.42845		309728.3		67.06

FALSE	High	MVFTSSADGC	1xCarbamidor	TRUE	1.90E-06	0.006854	1	1	12	sp	cRAP117 00000(	sp	0	2691.23889	20475620	19905.4	29.95	
FALSE	High	MVFTSSADGC	1xCarbamidor	TRUE	8.45E-06	0.006854	1	1	9	sp	cRAP117 00000(	sp	0	2707.23381	494768	76106.5	28.22	54.26
FALSE	High	NDYFSDFSGVYSQISR		TRUE	5.21E-05	0.006854	1	1	3	sp	cRAP117 00000(	sp	0	1884.83988	1924722		40.08	
FALSE	High	NLCNIPCSALL	3xCarbamidor	TRUE	7.67E-05	0.006854	1	1	2	cRAP078	Lysozyme C OS=	cRAP078	0	2508.18911	431405	11727.5	54.47	
FALSE	High	NLCNIPCSALL	3xCarbamidor	TRUE	0.133844	0.006854	1	1	1	cRAP078	Lysozyme C OS=	cRAP078	1	2636.28407	80137			
FALSE	High	NLQTVNVDEN		FALSE	0.189612	0.006854	1	1	1	P62899	60S ribosomal p	P62899	0	1145.54331				
FALSE	High	NTDGSTDYGILQINSR		TRUE	0.0002206	0.006854	1	1	3	cRAP078	Lysozyme C OS=	cRAP078	0	1753.83513	393517		51.14	
FALSE	High	NVQALEIELQSQLALK		TRUE	0.00024	0.006854	1	1	4	P13645	Keratin, type I cy	P13645	0	1797.01164	153442	5233.2	75.72	38.02
FALSE	High	QVHPDTGISSK		FALSE	0.0207395	0.006854	2	15	1	Q16778; (	Histone H2B typ	Q16778; (	0	1168.59568	409722	63697.3	61.69	66.68
FALSE	High	RDTLLLELK		TRUE	0.0037499	0.006854	1	1	5	sp	cRAP117 00000(	sp	1	1100.66739	2377187		77.52	
FALSE	High	RDTLLLELKR		TRUE	0.0025966	0.006854	1	1	7	sp	cRAP117 00000(	sp	2	1256.7685	1705324		86.79	
FALSE	High	RGDLPFVVPRR		FALSE	0.138765	0.006854	1	1	2	P35579	Myosin-9 OS=Hc	P35579	2	1311.76441	50161		48.45	
FALSE	High	RHPYFYAPELLFFAKR		TRUE	0.0108419	0.006854	1	1	2	P02768	Serum albumin (	P02768	2	2055.09631	72454			
FALSE	High	RKASGPPVSELITK		FALSE	0.0223667	0.006854	1	2	1	P10412	Histone H1.4 OS	P16402; P	2	1482.86385	91270	19747.5	55.61	47.29
FALSE	High	RKPDTIEVQQI	1xOxidation [I	FALSE	0.0011576	0.006854	1	3	3	P35241	Radixin OS=Hom	P35241; P	1	1488.78389	64871		87.2	
FALSE	High	RKPDTIEVQQMKAQAR		FALSE	0.180568	0.006854	1	3	1	P35241	Radixin OS=Hom	P35241; P	2	1899.02289	41218		18.49	
FALSE	High	RTPPAGVFYQGWSATPIANG:		TRUE	0.0152044	0.006854	1	1	4	sp	cRAP117 00000(	sp	1	3239.62457	1593495	10592.4	37.27	17.82
FALSE	High	RTPPAGVFYQGWSATPIANG:		TRUE	0.0003348	0.006854	1	1	8	sp	cRAP117 00000(	sp	2	3610.80506	21548810		37.09	
FALSE	High	RVTIMPK		FALSE	0.0398971	0.006854	1	5	2	P84243	Histone H3.3 OS	P84243; C	1	844.50732	1085997	196631.5	58.49	56.41
FALSE	High	RVTIMPKDIQLAR		FALSE	0.149644	0.006854	1	5	1	P84243	Histone H3.3 OS	P84243; C	2	1540.89919	586693	148745.7	87.68	77.29
FALSE	High	RVTIMPKDIQI	1xOxidation [I	FALSE	0.0201218	0.006854	1	5	1	P84243	Histone H3.3 OS	P84243; C	2	1556.89411	170693	30635.3	72.39	60.41
FALSE	High	SAHSIAQLQK		FALSE	0.0717932	0.006854	1	1	1	Q9ULS5	Transmembrane	Q9ULS5	0	1082.59528	5427		4.29	
FALSE	High	SCQAQPTTM/	1xCarbamidor	TRUE	0.0032797	0.006854	1	1	2	cRAP016	Kappa-casein OS	cRAP016	0	1250.56162	72070		90.65	
FALSE	High	SGGGGGGGF(	1xCarbamidor	FALSE	0.0279361	0.006854	1	1	1	P13647	Keratin, type II c	P13647	1	2257.05269	26416	4687.2	62.93	83.86
FALSE	High	SGGGGGRFSS	1xCarbamidor	TRUE	0.0297804	0.006854	1	1	1	P04264	Keratin, type II c	P04264	2	3149.45658	20735		25.64	
FALSE	High	SGVSLAALKKALAAAGYDVEK		FALSE	0.0372982	0.006854	1	3	1	P10412	Histone H1.4 OS	P16402; P	2	2062.15428	65407	17325.8		
FALSE	High	SLDLDSIIAEVK		TRUE	0.0637766	0.006854	1	1	2	P04264	Keratin, type II c	P04264	0	1302.71512	7300		103.43	
FALSE	High	SIRDTPAKNA(	3xLabel:13C(6	FALSE	0.0535668	0.006854	1	1	2	P06748	Nucleophosmin	P06748	2	1346.78847	32288	140792.4	74.18	59.73
FALSE	High	SPAQILQWQ\	1xCarbamidor	TRUE	0.141288	0.006854	1	1	1	cRAP016	Kappa-casein OS	cRAP016	1	3211.63506				
FALSE	High	SSLLEKGLDGAK		FALSE	0.168891	0.006854	1	1	2	P81605	Dermcidin OS=H	P81605	1	1217.67359	325464		87.2	
FALSE	High	SSLLEKGLDGAKK		FALSE	0.0433158	0.006854	1	1	1	P81605	Dermcidin OS=H	P81605	2	1345.76856	26192		54.78	
FALSE	High	SSSSGSVGESSSKGPR		TRUE	0.222795	0.006854	1	1	1	P13645	Keratin, type I cy	P13645	1	1495.6983				
FALSE	High	STELLIR		FALSE	0.0433158	0.006854	1	5	1	P84243	Histone H3.3 OS	P84243; C	0	831.49344	1534981	133888.6	30.84	48.00
FALSE	High	STITSREIQTAVRLLLPGELAK		FALSE	0.161861	0.006854	1	13	2	Q16778	Histone H2B typ	Q16778; (	2	2396.38713	250218	76890.5	116.94	92.37
FALSE	High	STSSFSCLSR	1xCarbamidor	TRUE	0.0442971	0.006854	1	1	2	P35908	Keratin, type II c	P35908	0	1131.5099	305723		46.78	
FALSE	High	SYELPDGQVITIGNER		FALSE	0.0022279	0.006854	1	10	2	P60709	Actin, cytoplasm	A5A3E0; P	0	1790.89192	90747		42.84	
FALSE	High	THNLEPYFESFINNLR		TRUE	0.0560101	0.006854	1	1	1	P04264	Keratin, type II c	P04264	0	1993.97665	18590		64.23	
FALSE	High	TIGISVDPR		FALSE	0.0212953	0.006854	1	1	1	P26373	60S ribosomal p	P26373	0	957.53637	36001		43.47	
FALSE	High	TLNDMRQEYEQLIAK		TRUE	0.0371588	0.006854	1	1	1	P35527	Keratin, type I cy	P35527	1	1851.92692	38416			
FALSE	High	TLTSGGHAEH	2xCarbamidor	FALSE	0.125847	0.006854	1	1	1	P50238	Cysteine-rich pr(	P50238	0	3117.3764	46790	12042.4	112.87	
FALSE	High	TPGSRNLCNIF	3xCarbamidor	TRUE	0.103719	0.006854	1	1	1	cRAP078	Lysozyme C OS=	cRAP078	1	3006.44415	60354			
FALSE	High	TPPAGVFYQGWSATPIANGSI		TRUE	0.0398971	0.006854	1	1	2	sp	cRAP117 00000(	sp	0	3083.52346	622322		51.06	

FALSE	High	TPPAGVFYQGWSATPIANGSl		TRUE	0.0205057	0.006854	1	1	4	sp	cRAP117 00000(	sp	1	3454.70394	1136332		58.02	
FALSE	High	TPPAGVFYQGWSATPIANGSl		TRUE	0.0252357	0.006854	1	1	5	sp	cRAP117 00000(	sp	2	3582.79891	1269281		58.18	
FALSE	High	TVTAMDVVYALK		FALSE	0.0218657	0.006854	1	1	1	P62805	Histone H4 OS=l	P62805	0	1310.70245	132445	34616.5	18.23	
FALSE	High	TVTAMDVVYALKR		FALSE	0.0321038	0.006854	1	1	2	P62805	Histone H4 OS=l	P62805	1	1466.80356	38727	26423.3	47.04	19.76
FALSE	High	VATVSLPR		TRUE	0.0217833	0.006854	1	1	4	cRAP112	Trypsin OS=Sus s	cRAP112	0	842.50943	21581588		30.71	
FALSE	High	VERADGYEPP	1xLabel:13C(6	FALSE	0.134815	0.006854	1	1	1	P61247	40S ribosomal p	P61247	1	1680.81708	115304	98579.9	85.29	67.77
FALSE	High	VTIAQGGVLPNIQAVLLPK		FALSE	0.0128167	0.006854	1	10	4	Q16777	Histone H2A typ	Q16777; (	0	1931.16881	244625	39166.4	115.61	98.71
FALSE	High	VTIAQGGVLPNIQAVLLPKK		FALSE	0.02358	0.006854	1	10	1	Q16777	Histone H2A typ	Q16777; (	1	2059.26377	53372		36.17	
FALSE	High	VTIMPKDIQLAR		FALSE	0.179307	0.006854	1	5	1	P84243	Histone H3.3 OS	P84243; C	1	1384.79808	7734		88.88	
FALSE	High	VTIMPKDIQLA	1xOxidation [l	FALSE	0.129553	0.006854	1	5	1	P84243	Histone H3.3 OS	P84243; C	1	1400.793	78275	10603.1	88.37	84.07
FALSE	High	WVGDLPNGR	1xCarbamido	FALSE	0.0456394	0.006854	1	1	2	Q96DR8	Mucin-like prote	Q96DR8	1	1369.66813	69435		61.15	
FALSE	High	YADLTEDQLP	1xCarbamido	FALSE	0.0438037	0.006854	1	1	1	P18669	Phosphoglycera	P18669	1	2437.1954				
FALSE	High	YALYDATYETK		FALSE	0.0015907	0.006854	1	2	2	P23528	Cofilin-1 OS=Ho	P23528; C	0	1337.62597	140032	37581.3	60.32	54.83
FALSE	High	YEKDIAAYR		FALSE	0.0110923	0.006854	1	2	2	P26583	High mobility gr	P26583; P	1	1128.5684	399365	3144.5	68.84	
FALSE	High	YICDNQDTISS	1xCarbamido	TRUE	0.0003102	0.006854	1	1	1	cRAP087	Serum albumin (	cRAP087	0	1443.64203	20696		94.91	
FALSE	High	YLGYLEQLLR		TRUE	0.007238	0.006854	1	1	2	cRAP013	Alpha-S1-casein	cRAP013	0	1267.7045	44967	14635.8	96.97	77.53
FALSE	High	YRPGTVALR		FALSE	0.224306	0.006854	1	5	2	P84243	Histone H3.3 OS	P84243; C	0	1032.59489	162184	19325.8	40.38	37.11
FALSE	High	YRPGTVALREI	3xLabel:13C(6	FALSE	0.114481	0.006854	1	5	1	P84243	Histone H3.3 OS	P84243; C	1	1448.88304	167531	8276.1	34.44	70.35
FALSE	High	YSQGNVSAVGVTYDGHTALTI		TRUE	0.0512279	0.006854	1	1	2	sp	cRAP117 00000(	sp	0	2196.06798	1345676	7148.6	56.04	3.98

Abundance	Abundance F	Abundance	Abundanc	Abundance	Abundar	Precu	Quan Info	Quan Ch	Found	Found	Found	Found	Found	Found	Confid	Percolator q	Percolator P	XCorr b	Top Apex RT in min
131147	20021	12433	5540			1		Light	High	Peak F	Peak F	Peak F	Not Fo	Not Fc	High	0.008403	0.013350	3.84	66.16
416383	222224	112660	103472	55270	39419	2		Heavy	Peak F	Peak F	Peak F	High	Peak F	High	High	0.008403	0.031020	3.1	64
64110	40998	20062		5349		3		Light	Peak F	High	Peak F	Not Fo	Peak F	Not Fc	High	0.009259	0.164900	3.39	139.93
2714467	3938081	1124488	503862	557639	211506	4	NotUnique	Light	Peak F	High	Peak F	Peak F	Peak F	Peak F	High	0.009259	0.023860	3.13	122.24
285714	777584	200126	57371	75814	43269	5		Light	High	Peak F	Peak F	Peak F	Peak F	Peak F	High	0.008403	0.185400	2.51	94.63
6227066	7836148	4187765				6		Light	High	High	High	Not Fo	Not Fo	Not Fc	High	0.008403	0.000196	6.34	113.99
8662712	20929875	6206443				7		Light	High	High	High	Not Fo	Not Fo	Not Fc	High	0.009259	0.000061	6.5	139.94
9545468	4929521	3751901				8		Light	Peak F	High	Peak F	Not Fo	Not Fo	Not Fc	High	0.009259	0.159400	2.37	116.55
100211	129126	23246	49848	40339	11652	9		Light	Peak F	High	Peak F	Peak F	Peak F	Peak F	High	0.009259	0.041590	2.51	73.16
123953	348765	89028		71991	17115	10	NotUnique	Light	High	High	Peak F	Not Fo	Peak F	Peak F	High	0.008403	0.029480	4.22	205.02
273366	625788	128432	61015	99925	30798	11	NotUnique	Light	High	Peak F	High	Peak F	Peak F	Peak F	High	0.008403	0.011000	4.96	178.85
						11	Redundan	Heavy	Peak F	Peak F	Peak F	Peak F	High	Peak F	High	0.009259	0.136200	4.68	178.98
189156	302699	75620	61568	61808	25812	12		Heavy	Peak F	Peak F	Peak F	High	High	Peak F	High	0.008403	0.093700	2.4	117.49
13358	47495	27457	7366	18813	15832	13	NotUnique	Light	Peak F	High	Peak F	Peak F	Peak F	Peak F	High	0.009259	0.150500	3.03	12.95
34516	23855	26805	10864	4284	8365	14	NotUnique	Light	Peak F	High	Peak F	Peak F	Peak F	Peak F	High	0.009259	0.078640	2.24	21.3
10900042	8481269	4715281				15		Light	High	High	High	Not Fo	Not Fo	Not Fc	High	0.008403	0.008673	3.64	62.25
45372	561857	313438				16		Light	Peak F	High	High	Not Fo	Not Fo	Not Fc	High	0.009259	0.016490	5.8	200.54
						17	NoQuanVa	Light	High	Not Fo	Not Fo	Not Fo	Not Fo	Not Fc	High	0.008403	0.199300	3.04	
180220	96483	53876				18		Light	Peak F	High	High	Not Fo	Not Fo	Not Fc	High	0.009259	0.059760	2.76	62.16
788402	242991	88355	63342	31873	13918	19		Light	High	High	High	Peak F	Peak F	Peak F	High	0.008403	0.010090	3.01	69.55
82032	15630	5736	5005			20		Light	High	Peak F	Peak F	Peak F	Not Fo	Not Fc	High	0.008403	0.029440	3.69	118.44
209316	50083	35280				21		Light	Peak F	High	Peak F	Not Fo	Not Fo	Not Fc	High	0.009259	0.025180	3.29	50.67
138686	190650	87068				22		Light	High	High	High	Not Fo	Not Fo	Not Fc	High	0.009259	0.001062	7.44	123.03
							IndistinguishableCl		Not Fo	Not Fo	Not Fo	Not Fo	Not Fo	Not Fc	High	0.008403	0.140200	2.36	81.13
135202	55957	10578	82451	44594		23		Light	High	Peak F	Peak F	Peak F	Peak F	Not Fc	High	0.008403	0.113800	2.05	174.68
						23	Redundan	Heavy	Peak F	Peak F	Peak F	High	High	Not Fc	High	0.008403	0.082590	2.42	174.68
47426	55081	17960				24		Light	High	Peak F	Peak F	Not Fo	Not Fo	Not Fc	High	0.008403	0.090260	2.72	91.45
							InconsistentlyLabe		Not Fo	Not Fo	Not Fo	Not Fo	Not Fo	Not Fc	High	0.009259	0.180000	2.26	166.98
1385639	5690543	692908	377343	1033143	40429	25		Light	Peak F	High	High	Peak F	Peak F	Peak F	High	0.009259	0.024520	3.13	171.74
53799	200947	27954	34404	26141	19912	26		Light	High	Peak F	Peak F	Peak F	Peak F	Peak F	High	0.008403	0.085850	3.15	162.91
168258	174210	65358				27		Light	High	Peak F	Peak F	Not Fo	Not Fo	Not Fc	High	0.008403	0.089190	2.66	208.24
313989	313634	116116				28		Light	Peak F	High	Peak F	Not Fo	Not Fo	Not Fc	High	0.009259	0.035300	3.08	181.24
677341	531843	248219				29		Light	High	High	High	Not Fo	Not Fo	Not Fc	High	0.008403	0.000424	5.59	111.32
68079	18525	14575				30		Light	High	Peak F	Peak F	Not Fo	Not Fo	Not Fc	High	0.008403	0.009235	3.33	50.47
			107449		4557	31		Light	High	Not Fo	Not Fo	Peak F	Not Fo	Peak F	High	0.008403	0.247000	2.6	147.09
156044			16501			32		Light	High	Not Fo	Not Fo	Peak F	Not Fo	Not Fc	High	0.008403	0.040940	3.24	177.91
14492	36919	84898				33		Light	High	Peak F	Peak F	Not Fo	Not Fo	Not Fc	High	0.008403	0.247000	9.42	216.39
578775	344903	130493				34		Light	High	Peak F	High	Not Fo	Not Fo	Not Fc	High	0.008403	0.000679	5.03	104.85
4785959	2521634	954559				35		Light	High	High	High	Not Fo	Not Fo	Not Fc	High	0.009259	0.000677	7.12	186.96
22340	7005	7006				36		Light	High	Peak F	Peak F	Not Fo	Not Fo	Not Fc	High	0.008403	0.000190	8.7	78.31

						37	NoQuanVa	Light	High	Not Fo	Not Fo	Not Fo	Not Fo	Not Fc	High	0.008403	0.000121	5.77	
708358	429455	66048				38		Light	High	High	High	Not Fo	Not Fo	Not Fc	High	0.008403	0.004315	4.56	45.26
314657	258308	142989	11214		4611	39		Light	High	Peak Fc	High	Peak Fc	Not Fo	Peak F	High	0.008403	0.000355	5.99	140.92
81249	38311	23559				40		Light	Peak Fc	High	Peak Fc	Not Fo	Not Fo	Not Fc	High	0.009259	0.039910	4.09	157.34
219009	122815	68264				41		Light	Peak Fc	High	Peak Fc	Not Fo	Not Fo	Not Fc	High	0.009259	0.054750	3.01	122.96
25552	483616	52445	28816	361704	48798	42	NotUnique	Heavy	Peak Fc	Peak Fc	Peak Fc	Peak Fc	High	Peak F	High	0.009259	0.128500	1.04	38.34
148056	78526	33518				43		Light	High	Peak Fc	Peak Fc	Not Fo	Not Fo	Not Fc	High	0.008403	0.012180	4.25	147.85
						44	NoQuanVa	Light	High	High	Not Fo	Not Fo	Not Fo	Not Fc	High	0.008403	0.095850	2.2	
2790909	2027029	618584	655797	377090	143081	45	NotUnique	Light	High	Peak Fc	High	Peak Fc	Peak Fc	Peak F	High	0.008403	0.009605	4.01	24.59
801608	2958246	567347	322531	611066	182713	46	NotUnique	Light	High	High	High	Peak Fc	Peak Fc	Peak F	High	0.008403	0.003513	5.66	54.45
						46	Redundan	Heavy	Peak Fc	Peak Fc	Peak Fc	High	High	High	High	0.009259	0.018160	4.75	54.45
256435	99002	39028				47		Light	High	High	Peak Fc	Not Fo	Not Fo	Not Fc	High	0.009259	0.068890	3.12	142.13
						48	NoQuanVa	Light	Not Fo	High	Not Fo	Not Fo	Not Fo	Not Fc	High	0.009259	0.086910	5	
29334	10911	5358				49		Light	High	Peak Fc	Peak Fc	Not Fo	Not Fo	Not Fc	High	0.008403	0.103000	3.1	145
						50	NoQuanVa	Light	High	Not Fo	Not Fo	Not Fo	Not Fo	Not Fc	High	0.008403	0.018210	3.7	
34427	90445	26638	53721	172209	42856	51		Heavy	Peak Fc	Peak Fc	Peak Fc	Peak Fc	High	High	High	0.009259	0.072200	3.85	95.66
66307			48408		11092	52		Heavy	Peak Fc	Not Fo	Not Fo	High	Not Fo	Peak F	High	0.008403	0.010490	4.5	101.66
79944	73116	62721				53		Light	High	Peak Fc	High	Not Fo	Not Fo	Not Fc	High	0.008403	0.035260	2.92	144.77
78328	90980	36523	7475		2657	54		Light	High	Peak Fc	Peak Fc	Peak Fc	Not Fo	Peak F	High	0.008403	0.150100	2.02	72.88
75131	160719	37417	18065	28751	10143	55		Light	Peak Fc	High	High	Peak Fc	Peak Fc	Peak F	High	0.009259	0.010050	4.15	135.17
79489	36298	20395				56	NotUnique	Light	High	High	Peak Fc	Not Fo	Not Fo	Not Fc	High	0.008403	0.014480	3.52	118.13
2193594	1164605	462250				57		Light	High	High	High	Not Fo	Not Fo	Not Fc	High	0.008403	0.000025	6.67	103.03
10582	9956	18832				58		Light	High	Peak Fc	High	Not Fo	Not Fo	Not Fc	High	0.008403	0.029480	3.26	173.15
7819909	7712058	4292076				59		Light	High	High	High	Not Fo	Not Fo	Not Fc	High	0.008403	0.000057	6.35	151.39
242427	234457	88180	43744	46845	23977	60		Heavy	Peak Fc	Peak Fc	Peak Fc	High	High	Peak F	High	0.009259	0.015360	3.65	150.8
						61	NoQuanVa	Light	High	Not Fo	Not Fo	Not Fo	Not Fo	Not Fc	High	0.008403	0.060990	2.13	
276482	293969	210148				62		Light	High	Peak Fc	Peak Fc	Not Fo	Not Fo	Not Fc	High	0.008403	0.171400	2.31	13.27
46650	13775	7853				63		Light	High	Peak Fc	Peak Fc	Not Fo	Not Fo	Not Fc	High	0.008403	0.054270	2.79	59.65
130234	60850	12546	268225	130164	45178	64		Light	High	Peak Fc	Peak Fc	Peak Fc	Peak Fc	Peak F	High	0.008403	0.023660	2.87	140.77
						65	NoQuanVa	Heavy	Peak Fc	Peak Fc	Peak Fc	Peak Fc	High	Peak F	High	0.009259	0.169800	2.07	136.7
955794	852848	754764				66	NotUnique	Light	High	High	Peak Fc	Peak Fc	Peak Fc	Peak F	High	0.009259	0.094670	2.22	136.7
131618	252180	86351	17218	21065	9551	67		Light	High	Peak Fc	High	Peak Fc	Peak Fc	Peak F	High	0.008403	0.082730	2.43	37.97
3407499	5916807	1660899	11572		12133	68		Light	High	High	High	Peak Fc	Not Fo	Peak F	High	0.009259	0.003908	4.92	170.08
1019261	1207273	316331				69		Light	High	High	High	Not Fo	Not Fo	Not Fc	High	0.008403	0.000668	4.5	161.67
	72974	44183				70		Light	Not Fo	High	Peak Fc	Not Fo	Not Fo	Not Fc	High	0.009259	0.008847	4.4	187.17
119507	148393		182770	253721	53087	71		Heavy	Peak Fc	Peak Fc	Not Fo	High	Peak Fc	Peak F	High	0.008403	0.012340	3.38	167.62
209319	110152	112527				72		Light	Peak Fc	High	Peak Fc	Not Fo	Not Fo	Not Fc	High	0.009259	0.126400	2.23	160.97
71477	45489	20455				73		Light	Peak Fc	High	Peak Fc	Not Fo	Not Fo	Not Fc	High	0.009259	0.134500	2.48	45.15
229328	185311	171474	21788		2825	74		Light	High	Peak Fc	High	Peak Fc	Not Fo	Peak F	High	0.008403	0.010330	3.58	115.23
							IndistinguishableCl		Not Fo	Not Fo	Not Fo	Not Fo	Not Fo	Not Fc	High	0.008403	0.026600	3.33	155.7
						75	NoQuanVa	Light	High	Not Fo	Not Fo	Not Fo	Not Fo	Not Fc	High	0.008403	0.114000	3	
			893608	309728	307566	76		Heavy	Not Fo	Not Fo	Not Fo	Peak Fc	High	Peak F	High	0.009259	0.168200	1.06	12.36

22899508	20475620	12292065			19905	77		Light	High	High	High	Not Fo	Not Fo	Peak F	High	0.008403	0.000136	6.96	168.86
494768	582744	323550	172666	76107	69871	78		Light	High	High	High	Peak Fc	Peak Fc	Peak F	High	0.008403	0.000354	6.46	161.39
2403952	1924722	1002535				79		Light	High	Peak Fc	High	Not Fo	Not Fo	Not Fc	High	0.008403	0.001131	5.55	190.68
431405	768994	250102	11727			80		Light	High	Peak Fc	High	Peak Fc	Not Fo	Not Fc	High	0.008403	0.001449	4.8	182.22
	80137					81		Light	Not Fo	High	Not Fo	Not Fo	Not Fo	Not Fc	High	0.009259	0.114400	2.3	165.92
							IndistinguishableCl		Not Fo	Not Fo	Not Fo	Not Fo	Not Fo	Not Fc	High	0.008403	0.208900	2.63	95.47
530176	393517	163448				82		Light	High	Peak Fc	High	Not Fo	Not Fo	Not Fc	High	0.008403	0.002845	5.5	139.95
137791	484432	153442	5233	9500	5075	83		Light	High	Peak Fc	High	Peak Fc	Peak Fc	Peak F	High	0.008403	0.003002	5.33	195.01
578884	409722	125521	122297	63697	28072	84	NotUniqu	Light	High	Peak Fc	Peak Fc	Peak Fc	Peak Fc	Peak F	High	0.008403	0.051630	2.24	40.55
2377187	6228128	1348546				85		Light	High	High	High	Not Fo	Not Fo	Not Fc	High	0.009259	0.012770	3.61	122.66
4367816	1705324	608795				86		Light	High	High	High	Not Fo	Not Fo	Not Fc	High	0.009259	0.010940	3.84	102.08
71684		35100				87		Light	Peak Fc	High	High	Not Fo	Not Fo	Not Fc	High	0.009259	0.117900	3.92	100.36
	72454					88		Light	High	High	Not Fo	Not Fo	Not Fo	Not Fc	High	0.008403	0.034170	2.96	167.71
60231	138306		19747	31688	11867	89		Light	Peak Fc	High	Not Fo	Peak Fc	Peak Fc	Peak F	High	0.009259	0.026550	3.07	77.25
247699	57807	64871				90		Light	High	High	High	Not Fo	Not Fo	Not Fc	High	0.008403	0.008213	3.14	39.98
40367	55399	41218				91		Light	Peak Fc	High	Peak Fc	Not Fo	Not Fo	Not Fc	High	0.009259	0.146500	2.76	60.61
2171184	1593495	989839	12853	9017	10592	92		Light	Peak Fc	High	High	Peak Fc	Peak Fc	Peak F	High	0.009259	0.019450	3.44	139.68
21548810	24474535	11053491				93		Light	High	High	High	Not Fo	Not Fo	Not Fc	High	0.008403	0.003714	4.86	131.62
1504040	1085997	365105	305514	196632	84785	94		Light	Peak Fc	High	High	Peak Fc	Peak Fc	Peak F	High	0.009259	0.042520	1.8	54.57
586693	1413747	168212	148746	277727	38433	95		Light	Peak Fc	High	Peak Fc	Peak Fc	Peak Fc	Peak F	High	0.009259	0.125400	1.36	108.35
170693	467321	127986	30635	66973	21622	96		Light	Peak Fc	High	Peak Fc	Peak Fc	Peak Fc	Peak F	High	0.009259	0.024390	2.43	93.07
5265	5594					97		Light	Peak Fc	High	Not Fo	Not Fo	Not Fo	Not Fc	High	0.009259	0.068740	2.93	49.18
218478	72070	33014				98		Light	High	High	Peak Fc	Not Fo	Not Fo	Not Fc	High	0.008403	0.015950	3.3	50.17
26416	56783	16626	4687	12798	2259	99		Light	Peak Fc	High	Peak Fc	Peak Fc	Peak Fc	Peak F	High	0.009259	0.031810	5.28	123.46
16509	27475	20735				100		Light	Peak Fc	High	Peak Fc	Not Fo	Not Fo	Not Fc	High	0.009259	0.033480	3.7	131.5
	65407			17326		101		Light	Not Fo	High	Not Fo	Not Fo	Peak Fc	Not Fc	High	0.009259	0.040210	3.73	177.74
7300	29761	3800				102		Light	High	Peak Fc	High	Not Fo	Not Fo	Not Fc	High	0.008403	0.105500	2.93	199.6
56449	32288	8406	140792	153438	34345	103		Heavy	Peak Fc	Peak Fc	Peak Fc	Peak Fc	High	Peak F	High	0.009259	0.054080	3.79	23.61
						104	NoQuanVa	Light	Not Fo	High	Not Fo	Not Fo	Not Fo	Not Fc	High	0.009259	0.119500	3.36	
959801	325464	163114				105		Light	High	Peak Fc	High	Not Fo	Not Fo	Not Fc	High	0.008403	0.194300	1.87	92.33
46417	26192	14880				106		Light	High	Peak Fc	Peak Fc	Not Fo	Not Fo	Not Fc	High	0.008403	0.082590	2.18	75.29
						107	NoQuanVa	Light	High	Not Fo	Not Fo	Not Fo	Not Fo	Not Fc	High	0.008403	0.231600	1.45	
1540761	1534981	840631	133889	189729	65440	108		Light	High	Peak Fc	Peak Fc	Peak Fc	Peak Fc	Peak F	High	0.008403	0.082590	2.53	95.34
94048	1218627	250218	19563	196173	76890	109		Light	High	High	Peak Fc	Peak Fc	Peak Fc	Peak F	High	0.009259	0.152300	1.51	195.18
305723	404664	141628				110		Light	Peak Fc	High	High	Not Fo	Not Fo	Not Fc	High	0.009259	0.046360	2.82	81.74
124064	66377					111		Light	High	High	Not Fo	Not Fo	Not Fo	Not Fc	High	0.009259	0.004144	3.7	164.94
	30343	11389				112		Light	Not Fo	High	Peak Fc	Not Fo	Not Fo	Not Fc	High	0.009259	0.056060	4.37	197.08
56858	36001	23487				113		Light	High	Peak Fc	Peak Fc	Not Fo	Not Fo	Not Fc	High	0.008403	0.052550	2.57	105
38416						114		Light	High	Not Fo	Not Fo	Not Fo	Not Fo	Not Fc	High	0.008403	0.074880	2.16	156.15
186950	46790	12214	12042			115		Light	High	Peak Fc	Peak Fc	Peak Fc	Not Fo	Not Fc	High	0.008403	0.161600	1.24	97.25
60354						116		Light	High	Not Fo	Not Fo	Not Fo	Not Fo	Not Fc	High	0.008403	0.143400	2.71	162.9
714867	622322	217377				117		Light	High	High	Peak Fc	Not Fo	Not Fo	Not Fc	High	0.009259	0.060220	3.76	152.12

1731916	1136332	449612				118		Light	High	High	High	Not Fo	Not Fo	Not Fc	High	0.008403	0.051290	3.89	142.98
1880454	1269281	477432				119		Light	High	High	High	Not Fo	Not Fo	Not Fc	High	0.008403	0.058570	3.17	134.9
150775	116343		34616			120		Light	High	Peak Fo	Not Fo	Peak Fo	Not Fo	Not Fc	High	0.008403	0.053370	3.6	174.13
38727	57176	20634	30107	26423	20122	121		Light	High	High	Peak Fo	Peak Fo	Peak Fo	Peak F	High	0.008403	0.068230	2.85	155.53
23219206	21581588	12364321				122		Light	High	High	High	Not Fo	Not Fo	Not Fc	High	0.008403	0.053240	2.28	89.77
263272	115304	32557	163418	98580	31041	123		Heavy	Peak Fo	Peak Fo	Peak Fo	Peak Fo	High	Peak F	High	0.009259	0.115200	2.24	110.95
244625	1296031	128397	39166	168521	28853	124		Light	Peak Fo	High	High	Peak Fo	Peak Fo	Peak F	High	0.009259	0.016900	5.01	208.05
41089	69325					125		Light	High	Peak Fo	Not Fo	Not Fo	Not Fo	Not Fc	High	0.008403	0.056100	1.69	185.53
7469	31636	7734				126		Light	Peak Fo	High	Peak Fo	Not Fo	Not Fo	Not Fc	High	0.009259	0.145300	2.28	124.2
78275	202871	26547	10603	27596	4504	127		Light	Peak Fo	High	Peak Fo	Peak Fo	Peak Fo	Peak F	High	0.009259	0.111400	2.62	107.38
135917	69435	38618				128		Light	High	Peak Fo	High	Not Fo	Not Fo	Not Fc	High	0.008403	0.085330	2.79	132.23
						129	NoQuanVa	Heavy	Not Fo	Not Fo	Not Fo	Not Fo	High	Not Fc	High	0.009259	0.045880	3.29	
171941	140032	37473	37581	39541	10786	130		Light	High	High	Peak Fo	Peak Fo	Peak Fo	Peak F	High	0.008403	0.010060	3.48	117.64
928364	399365	240996	3145			131		Light	High	High	Peak Fo	Peak Fo	Not Fo	Not Fc	High	0.008403	0.034670	2.96	66.22
66440	20696	8914				132		Light	High	Peak Fo	Peak Fo	Not Fo	Not Fo	Not Fc	High	0.008403	0.003542	3.81	73.4
44967	203367	39556	14636	25989	3291	133		Light	High	Peak Fo	High	Peak Fo	Peak Fo	Peak F	High	0.008403	0.026420	2.84	195.01
202305	162184	83695	22100	19326	9968	134		Light	Peak Fo	High	High	Peak Fo	Peak Fo	Peak F	High	0.009259	0.175700	2.59	67.22
167531	260298	135771	8216	25166	8276	135		Heavy	Peak Fo	Peak Fo	Peak Fo	Peak Fo	High	Peak F	High	0.009259	0.100800	2.18	85.33
1984155	1345676	541102	7353	6950		136		Light	Peak Fo	High	Peak Fo	Peak Fo	Peak Fo	Not Fc	High	0.009259	0.052140	3.26	116.96

Supplemental Table 4.2. Raw Proteome discoverer output from the three replicates of Condition B

Checked	Confid	Sequence	Modifications	Contami	Qvality PEP	Qvality q-va	Numl	Numl	Num	Master Protei	Master Protei	Protein Acces	Num	Theo MH	Abundances	Abundar	Abundar	Abundan
FALSE	High	AAAAAAAAAPAAAATAPTTA		FALSE	0.061508	0.009903	1	1	1	P37108	Signal recogni	P37108	0	2368.2				
FALSE	High	RKPDTIEVQQMK		FALSE	0.058303	0.009903	1	3	2	P35241	Radixin OS=H	P35241; P260	1	1472.8	334783	1502	34.95	
FALSE	High	RKATGPPVSEI	3xLabel:13C(6	FALSE	0.213189	0.009903	1	1	1	P16401	Histone H1.5	P16401	2	1514.9	20857	37745	61.41	52.89
FALSE	High	RKASGPPVSELITK		FALSE	0.089855	0.009903	1	2	1	P16402	Histone H1.3	P16402; P104	2	1482.9	81008	23323	33.12	40.08
FALSE	High	RGDLPFVVPRR		FALSE	0.095214	0.009903	1	1	2	P35579	Myosin-9 OS=	P35579	2	1311.8	26984		25.89	
FALSE	High	RGAFSSVSMS	1xOxidation [	FALSE	0.209531	0.009903	1	1	1	P19013	Keratin, type	P19013	1	1442.7	195553			
FALSE	High	RDTLLLELKR		TRUE	0.190443	0.009903	1	1	2	cRAP117	000000|rLys-	cRAP117	2	1256.8	787090		66.37	
FALSE	High	RDTLLLELK		TRUE	0.149668	0.009903	1	1	4	cRAP117	000000|rLys-	cRAP117	1	1100.7	11516309		50.17	
FALSE	High	QVQSLTCEVD	1xCarbamido	FALSE	0.095214	0.009903	1	1	1	P08670	Vimentin OS=	P08670	1	2377.2	12020		92.23	
FALSE	High	RKPDTIEVQQI	1xOxidation [	FALSE	0.157091	0.009903	1	3	2	P35241	Radixin OS=H	P35241; P260	1	1488.8	51681		64.77	
FALSE	High	QVHPDTGISSK		FALSE	0.131682	0.009903	1	15	1	Q16778	Histone H2B t	Q16778; Q5Q	0	1168.6	96508	25493	67.53	68.1
FALSE	High	NLCNIPCSALL	3xCarbamido	TRUE	0.057018	0.009903	1	1	3	cRAP078	Lysozyme C O	cRAP078	0	2508.2	207152	7275	45.65	
FALSE	High	NDYFSDFSGVYSQISR		TRUE	0.079309	0.009903	1	1	4	cRAP117	000000|rLys-	cRAP117	0	1884.8	1373511		25.27	
FALSE	High	MVFTSSADG	1xCarbamido	TRUE	0.048778	0.009903	1	1	8	cRAP117	000000|rLys-	cRAP117	0	2707.2	302006	5463	37.06	
FALSE	High	MVFTSSADG	1xCarbamido	TRUE	0.028311	0.009903	1	1	12	cRAP117	000000|rLys-	cRAP117	0	2691.2	5280815		30.41	
FALSE	High	LSSPATLNSR		TRUE	0.157091	0.009903	1	1	2	cRAP112	Trypsin OS=S	cRAP112	0	1045.6	1476094		10.13	
FALSE	High	ISLPLPNFSSLNLRETNLDSLPL		FALSE	0.150329	0.009903	1	1	1	P08670	Vimentin OS=	P08670	2	3376.8	12572		65.93	
FALSE	High	ISGLIYEETR		FALSE	0.157783	0.009903	1	1	2	P62805	Histone H4 O	P62805	0	1180.6	148545	3833	50.78	75.35
FALSE	High	LSAKPAPPKPEPRPK		FALSE	0.214113	0.009903	1	1	1	O00479	High mobility	O00479	0	1613.0	34684	1847	53.32	
FALSE	High	NTDGSTDYGILQINSR		TRUE	0.073519	0.009903	1	1	2	cRAP078	Lysozyme C O	cRAP078	0	1753.8	146877		7.12	
FALSE	High	RTPPAGVFYQGWSATPIANGS		TRUE	0.044816	0.009903	1	1	4	cRAP117	000000|rLys-	cRAP117	1	3239.6	732045		23.04	
FALSE	High	RTPPAGVFYQGWSATPIANGS		TRUE	0.122674	0.009903	1	1	3	cRAP117	000000|rLys-	cRAP117	2	3610.8	469226		62.62	
FALSE	High	RVTIMPKDIQI	1xOxidation [	FALSE	0.206826	0.009903	1	5	2	P84243	Histone H3.3	P84243; Q16	2	1556.9	450540	55894	66.99	30.74
FALSE	High	YEKDIAAYRAK		FALSE	0.124316	0.009903	1	2	1	P26583	High mobility	P26583; P094	2	1327.7	137167		81.06	
FALSE	High	VTIAQGGVLPNIQAVLLPKKTE		FALSE	0.084043	0.009903	2	8	1	Q7L7L0; Q99	Histone H2A t	Q7L7L0; P049	2	2778.6	56325	21715	104.38	
FALSE	High	VTIAQGGVLPI	1xLabel:13C(6	FALSE	0.184721	0.009903	2	10	1	Q7L7L0; Q99	Histone H2A t	Q16777; Q7L	0	1937.2	246520	38289		
FALSE	High	VFLENVIRDAVTYTEHAK		FALSE	0.173004	0.009903	1	1	1	P62805	Histone H4 O	P62805	1	2105.1	11257	79386	26.42	13.09
FALSE	High	VDWPSAVVEGGSSGSGLLTVA		TRUE	0.038683	0.009903	1	1	4	cRAP117	000000|rLys-	cRAP117	0	2864.4	284304		23.6	
FALSE	High	VAPEEHPVLLTEAPLNPK		FALSE	0.192946	0.009903	1	2	1	P60709	Actin, cytopla	P60709; P632	0	1954.1	51707	3738	22.82	73.52
FALSE	High	TVSLGAGAKD	3xLabel:13C(6	FALSE	0.135224	0.009903	1	1	1	P06748	Nucleophosm	P06748	2	3674.0				
FALSE	High	TPPAGVFYQGWSATPIANGSL		TRUE	0.149668	0.009903	1	1	3	cRAP117	000000|rLys-	cRAP117	2	3582.8	355737		27.73	
FALSE	High	TPPAGVFYQGWSATPIANGSL		TRUE	0.127665	0.009903	1	1	3	cRAP117	000000|rLys-	cRAP117	1	3454.7	1309984		50.76	
FALSE	High	TLLIKTVETRDGQVINETSQHH		FALSE	0.213189	0.009903	1	1	1	P08670	Vimentin OS=	P08670	2	2991.5	3726		120.55	
FALSE	High	SYELPDGQVITIGNER		FALSE	0.188791	0.009903	1	10	1	P60709	Actin, cytopla	A5A3E0; P632	0	1790.9	46783			
FALSE	High	STITSREIQTAV	2xLabel:13C(6	FALSE	0.140708	0.009903	1	13	1	Q16778	Histone H2B t	Q16778; Q5Q	1	1473.8				
FALSE	High	STITSREIQTAVR		FALSE	0.191274	0.009903	1	13	2	Q16778	Histone H2B t	Q16778; Q5Q	1	1461.8	1035940	198732	63.46	66.43
FALSE	High	SRGSGGLGGA	1xCarbamido	FALSE	0.079309	0.009903	1	3	1	P04259	Keratin, type	P04259; P025	1	1667.8	143101		28.26	
FALSE	High	SPAQILQWQV	1xCarbamido	TRUE	0.055512	0.009903	1	1	1	cRAP016	Kappa-casein	cRAP016	1	3211.6	38041		45.53	
FALSE	High	SPAQILQWQVLSNTVPAK		TRUE	0.065179	0.009903	1	1	4	cRAP016	Kappa-casein	cRAP016	0	1980.1	338669		67.84	
FALSE	High	SGGGGGRFSS	1xCarbamido	TRUE	0.099994	0.009903	1	1	1	P04264	Keratin, type	P04264	1	2294.0	66971		40.4	

FALSE	High	SGGGGGGGLGSGGSIR		TRUE	0.073519	0.009903	1	1	2	P35527	Keratin, type	P35527	0	1232.6	96449		46.31	
FALSE	High	SCAAAGTECLI	2xCarbamido	TRUE	0.147703	0.009903	1	1	2	cRAP112	Trypsin OS=Su	cRAP112	0	1882.8	26480		115.05	
FALSE	High	IITHPNFNGNT	1xOxidation [	TRUE	0.096066	0.009903	1	1	3	cRAP112	Trypsin OS=Su	cRAP112	0	2299.2	85205		30.72	
FALSE	High	IITHPNFNGNTLDNDIMLIK		TRUE	0.032653	0.009903	1	1	2	cRAP112	Trypsin OS=Su	cRAP112	0	2283.2	730113	7723	23.5	19.24
FALSE	High	IIRSSEDPNEDIVER		FALSE	0.173763	0.009903	1	1	1	P01591	Immunoglobu	P01591	1	1771.9	63887		120.09	
FALSE	High	LLCGLLAER	1xCarbamido	FALSE	0.210440	0.009903	1	1	1	P14174	Macrophage	P14174	0	1050.6	25993	59316	60.3	53.89
FALSE	High	GGGGSFGYSYGGGSGGGFSA		TRUE	0.052153	0.009903	1	1	2	P35527	Keratin, type	P35527	0	2705.2	74580	10680	52.79	15.57
FALSE	High	FSSCGGGGGS	1xCarbamido	TRUE	0.028311	0.009903	1	1	2	P04264	Keratin, type	P04264	0	1765.7	171135		19.2	
FALSE	High	FGQGSGPIVLDDVR		FALSE	0.217846	0.009903	1	1	1	Q9UGM3	Deleted in ma	Q9UGM3	0	1459.8	199969			
FALSE	High	FFVAPFPEVFGKEK		TRUE	0.224524	0.009903	1	1	2	cRAP013	Alpha-S1-case	cRAP013	1	1641.9	43065		57.07	
FALSE	High	FESNFNTQATNR		TRUE	0.134627	0.009903	1	1	2	cRAP078	Lysozyme C O	cRAP078	0	1428.7	104491		58.06	
FALSE	High	EIQTAVRLLLP	2xLabel:13C(6	FALSE	0.156402	0.009903	1	14	1	Q16778	Histone H2B t	Q16778; Q5Q	1	1763.1	412612	98891	76.11	44.01
FALSE	High	DGFGASGSCE	2xCarbamido	TRUE	0.034448	0.009903	1	1	3	cRAP117	000000|rLys-	cRAP117	0	2231.9	118113		45.16	
FALSE	High	CNKEVYFAER	1xCarbamido	FALSE	0.092702	0.009903	1	1	2	P50238	Cysteine-rich	P50238	1	1315.6	153072	16589	62.43	39.68
FALSE	High	CKELGITALHI	1xCarbamido	FALSE	0.184721	0.009903	1	1	1	P62263	40S ribosoma	P62263	1	1382.8				
FALSE	High	AYDNATAAVA	1xCarbamido	TRUE	0.038856	0.009903	1	1	2	cRAP117	000000|rLys-	cRAP117	1	3766.8	234266		89.03	
FALSE	High	AYDNATAAVAK		TRUE	0.137635	0.009903	1	1	4	cRAP117	000000|rLys-	cRAP117	0	1094.5	5687050		17.84	
FALSE	High	AVFVDLEPTVI	1xLabel:13C(6	FALSE	0.115285	0.009903	1	3	1	Q71U36	Tubulin alpha	Q71U36; P68	0	1707.9		16314		53.59
FALSE	High	ATNYNAGDRSTDYGIFQINSR		TRUE	0.201514	0.009903	1	1	1	P61626	Lysozyme C O	P61626	1	2363.1	15340		133.98	
FALSE	High	AQAAAPASVPAQAPK		FALSE	0.219735	0.009903	1	1	1	P47914	60S ribosoma	P47914	0	1377.7	168317	57903	52.57	59.27
FALSE	High	AMGIMNSFVI	1xOxidation [	FALSE	0.196331	0.009903	1	14	2	Q16778	Histone H2B t	Q16778; Q5Q	0	1759.8	110048	32920	116.75	119.24
FALSE	High	AGYRDGFGAS	2xCarbamido	TRUE	0.034757	0.009903	1	1	8	cRAP117	000000|rLys-	cRAP117	2	3754.7	4393702		37.49	
FALSE	High	AGYRDGFGAS	2xCarbamido	TRUE	0.028311	0.009903	1	1	8	cRAP117	000000|rLys-	cRAP117	1	2679.2	5771841		28.24	
FALSE	High	AGFAGDDAPRAVFPSIVGRPR		FALSE	0.208626	0.009903	1	9	1	P60709	Actin, cytopla	A5A3E0; P632	1	2156.1	30625	4352	25.26	23.38
FALSE	High	AAFGGSGGRGSSSGGGYSSGS		TRUE	0.028311	0.009903	1	1	1	P35908	Keratin, type	P35908	1	2501.1	26302		62.04	
FALSE	High	GGLYGGPSYC	1xCarbamido	TRUE	0.073519	0.009903	1	1	4	cRAP117	000000|rLys-	cRAP117	0	1727.8	405943		40.87	
FALSE	High	YLGYLEQLLR		TRUE	0.213189	0.009903	1	1	2	cRAP013	Alpha-S1-case	cRAP013	0	1267.7	424405	9055	64.49	62.34
FALSE	High	GGLYGGPSYC	1xCarbamido	TRUE	0.051921	0.009903	1	1	3	cRAP117	000000|rLys-	cRAP117	1	3593.6	2244644		43.86	
FALSE	High	GGSISGGGYGSGGGK		TRUE	0.173004	0.009903	1	1	3	P35908	Keratin, type	P35908	0	1197.5	54486		130.49	
FALSE	High	LKECCDKPLLE	2xCarbamido	TRUE	0.097358	0.009903	1	1	1	cRAP087	Serum albumi	cRAP087	1	1532.8	28537		42.66	
FALSE	High	LGTRLVPAERR		FALSE	0.155032	0.009903	1	1	1	Q6NZI2	Caveolae-ass	Q6NZI2	2	1267.8	57586		16.57	
FALSE	High	LGEHNIDVLEGNEQFINAAK		TRUE	0.039909	0.009903	1	1	2	cRAP112	Trypsin OS=Su	cRAP112	0	2211.1	3283452		26.43	
FALSE	High	KYSQGNVSAVGVTYDGHTAL		TRUE	0.028311	0.009903	1	1	4	cRAP117	000000|rLys-	cRAP117	1	2324.2	1190312		30.49	
FALSE	High	KVPQVSTPTLVEVSR		TRUE	0.147054	0.009903	1	2	2	cRAP087	Serum albumi	cRAP087; P02	1	1639.9	41618		20.76	
FALSE	High	KTVTAMDVVYALKR		FALSE	0.138857	0.009903	1	1	1	P62805	Histone H4 O	P62805	2	1594.9	56835	12731	66.55	78.9
FALSE	High	KLHTDVVENG	1xCarbamido	FALSE	0.175290	0.009903	1	1	1	Q96J66	ATP-binding c	Q96J66	2	2824.5				
FALSE	High	KGTVEGFEPAI	1xCarbamido	FALSE	0.102701	0.009903	1	1	1	P37108	Signal recogni	P37108	2	1952.0				
FALSE	High	HLQLAIRNDEELNKLLGR		FALSE	0.163424	0.009903	1	3	1	Q7L7L0	Histone H2A t	Q7L7L0; P049	2	2132.2	19359	13576	130.31	
FALSE	High	HLQLAIRNDEI	3xLabel:13C(6	FALSE	0.146407	0.009903	1	7	1	Q99878	Histone H2A t	Q16777; Q99	2	2122.2	227957	15557		122.86
FALSE	High	HLQLAIRNDEELNKLLGK		FALSE	0.097793	0.009903	1	7	1	Q99878	Histone H2A t	Q16777; Q99	2	2104.2				
FALSE	High	HGGGGGGFGGGGFGSR		TRUE	0.169258	0.009903	1	1	2	P35908	Keratin, type	P35908	0	1320.6	96952		17.17	
FALSE	High	HAVSEGTKAV	3xLabel:13C(6	FALSE	0.100441	0.009903	1	12	1	Q16778	Histone H2B t	Q16778; Q5Q	2	1812.0				

FALSE	High	HAVSEGTKAVTKYTSSK		FALSE	0.051921	0.009903	1	12	9	Q16778	Histone H2B t	Q16778; Q5Q	2	1793.9	631313	227158	68.21	43.65
FALSE	High	HAVSEGTKAV	2xLabel:13C(6	FALSE	0.132266	0.009903	1	15	2	Q16778	Histone H2B t	Q16778; Q5Q	1	1239.7				
FALSE	High	HAVSEGTKAVTK		FALSE	0.082557	0.009903	1	15	6	Q16778	Histone H2B t	Q16778; Q5Q	1	1227.7	1225202	299464	50.69	65.3
FALSE	High	GYSLGNWVC/	1xCarbamidor	TRUE	0.143214	0.009903	1	1	2	cRAP078	Lysozyme C O	cRAP078	0	1325.6	36316		41.37	
FALSE	High	GSRGGSGGSYGGGGSGGGYG		TRUE	0.037662	0.009903	1	1	1	P35527	Keratin, type	P35527	1	2091.9	15878		104.2	
FALSE	High	GSLGGGFSSGGFSGGSFSR		TRUE	0.028311	0.009903	1	1	2	P13645	Keratin, type	P13645	0	1707.8	83687	4005	65.19	
FALSE	High	GGSGGSYGGGGSGGGYGGG		TRUE	0.149011	0.009903	1	1	1	P35527	Keratin, type	P35527	0	1791.7	38309		94.47	
FALSE	High	YSQGNVSAVGVTYDGHTALTI		TRUE	0.101339	0.009903	1	1	4	cRAP117	000000|rLys-	cRAP117	0	2196.1	1342856		18.47	

Abundance	Abundance	Abundance	Abundanc	Abundance	Abundan	Precur	Quan I	Quan Cha	Found	Found	Found	Found	Found	Found	Confide	Percolator q	Percolator PE	XCorr	Top Apex RT in min
							Indistinguishable		Not Fo	Not Fo	Not Fo	Not Fo	Not Fo	Not Fo	High	0.009524	0.03618	4.67	135.94
581858	334783	327129			1502	60		Light	High	Peak F	High	Not Fo	Not Fo	Peak F	High	0.009524	0.03428	3.31	57.03
20857	17872	51110	37745	35640	85035	59		Heavy	Peak F	Peak F	Peak F	Peak F	Peak F	High	High	0.009524	0.1418	3.43	79.08
62499	81008	119406	23323	16574	36672	58		Light	Peak F	Peak F	High	Peak F	Peak F	Peak F	High	0.009524	0.05484	3.6	76.54
22423		32474				57		Light	High	Not Fo	High	Not Fo	Not Fo	Not Fo	High	0.009524	0.0584	3.74	99.89
195553						56		Light	High	Not Fo	Not Fo	Not Fo	Not Fo	Not Fo	High	0.007576	0.1213	4.03	53.6
576288	787090	1930723				55		Light	High	Peak F	Peak F	Not Fo	Not Fo	Not Fo	High	0.007576	0.1995	3.25	101.54
5953859	11516309	17718209				54		Light	High	Peak F	High	Not Fo	Not Fo	Not Fo	High	0.009524	0.09604	3.22	121.82
4465	12020	34703				53		Light	Peak F	Peak F	High	Not Fo	Not Fo	Not Fo	High	0.009524	0.05838	2.91	139.48
86876	18902	51681				61		Light	High	Peak F	High	Not Fo	Not Fo	Not Fo	High	0.009524	0.101	3.23	38.64
96508	95490	280054	25493	15390	58541	52		Light	Peak F	Peak F	High	Peak F	Peak F	Peak F	High	0.009524	0.08341	1.75	39.03
207152	98600	272972	7275			50		Light	High	Peak F	High	Peak F	Not Fo	Not Fo	High	0.009524	0.0334	5.29	181.48
1360050	2066950	1373511				49		Light	High	Peak F	High	Not Fo	Not Fo	Not Fo	High	0.007576	0.05251	5.32	190
335388	302006	152236	5463			48		Light	High	Peak F	High	Peak F	Not Fo	Not Fo	High	0.009524	0.02817	6.23	156.83
4131378	5280815	7504595				47		Light	High	Peak F	High	Not Fo	Not Fo	Not Fo	High	0.009524	0.01554198	7.56	167.69
1581997	1476094	1291803				46		Light	High	Peak F	High	Not Fo	Not Fo	Not Fo	High	0.009524	0.1016	2.56	71.41
	7586	20835				45		Light	Not Fo	Peak F	High	Not Fo	Not Fo	Not Fo	High	0.009524	0.0964	2.62	201.62
51633	148545	167935	3833	3016	11279	44		Light	High	Peak F	High	Peak F	Peak F	Peak F	High	0.009524	0.1015	3.8	114.28
16254	34684	53311			1847	43		Light	Peak F	Peak F	High	Not Fo	Not Fo	Peak F	High	0.009524	0.1423	2.69	43.34
144278	146877	164154				51		Light	High	Peak F	High	Not Fo	Not Fo	Not Fo	High	0.009524	0.07917	5.34	138.84
863108	732045	537521				62		Light	High	Peak F	High	Not Fo	Not Fo	Not Fo	High	0.009524	0.02564	3.81	138.83
469226	1127767	372250				63		Light	High	Peak F	High	Not Fo	Not Fo	Not Fo	High	0.009524	0.0772	4.19	130.66
120048	670726	450540	31485	55894	58691	64		Light	High	Peak F	High	Peak F	Peak F	Peak F	High	0.009524	0.1369	3.3	92.33
270668	33031	137167				82		Light	High	Peak F	Peak F	Not Fo	Not Fo	Not Fo	High	0.007576	0.06907	4.24	53.86
7119	56325	158513			21715	81	NotUn	Light	Peak F	Peak F	High	Not Fo	Not Fo	Peak F	High	0.009524	0.0509	2.28	145.95
		246520			38289	80	NotUn	Heavy	Not Fo	Not Fo	Peak F	Not Fo	Not Fo	High	High	0.009524	0.1208	3.92	207.45
	13599	9318	79386	77639	97705	79		Light	Not Fo	Peak F	High	Peak F	Peak F	Peak F	High	0.009524	0.1123	2.77	192.03
337714	284304	207854				78		Light	High	Peak F	High	Not Fo	Not Fo	Not Fo	High	0.009524	0.02181	7.96	203.81
53953	34472	51707	2101		6650	77		Light	High	Peak F	Peak F	Peak F	Not Fo	Peak F	High	0.007576	0.1108	3.37	131.28
						76	NoQua	Heavy	Not Fo	Not Fo	Not Fo	Not Fo	Not Fo	High	High	0.009524	0.08569	3.97	
464485	355737	264266				75		Light	High	Peak F	High	Not Fo	Not Fo	Not Fo	High	0.009524	0.09595	3.63	133.96
1309984	1870732	595894				74		Light	High	Peak F	High	Not Fo	Not Fo	Not Fo	High	0.009524	0.08048	4.72	142.03
3237	27382	3726				73		Light	Peak F	Peak F	High	Not Fo	Not Fo	Not Fo	High	0.009524	0.1418	4.22	122.55
46783						72		Light	High	Not Fo	Not Fo	Not Fo	Not Fo	Not Fo	High	0.007576	0.1084	4.26	163.88
						71	Redun	Heavy	Peak F	Peak F	Peak F	Peak F	Peak F	High	High	0.009524	0.08965	3.4	82.05
223801	1035940	1184075	74905	198732	351093	71		Light	High	Peak F	High	Peak F	Peak F	Peak F	High	0.009524	0.1257	3.04	82.05
116862	175232					70		Light	High	Peak F	Not Fo	Not Fo	Not Fo	Not Fo	High	0.007576	0.1771	5.5	69.21
	27245	53116				69		Light	Not Fo	Peak F	High	Not Fo	Not Fo	Not Fo	High	0.009524	0.03249	4.24	170.24
461526	74464	338669				68		Light	High	Peak F	High	Not Fo	Not Fo	Not Fo	High	0.009524	0.0387	3.14	186.13
119497	58614	66971				67		Light	High	Peak F	Peak F	Not Fo	Not Fo	Not Fo	High	0.007576	0.05459	6.11	97.84

194898	96449	89827				66		Light	High	Peak Fo	High	Not Fo	Not Fo	Not Fo	High	0.009524	0.04932	4.79	56.14
26480	5102	103765				65		Light	Peak Fo	Peak Fo	High	Not Fo	Not Fo	Not Fo	High	0.009524	0.09442	5.27	142.86
85205	57951	109717				42		Light	High	Peak Fo	High	Not Fo	Not Fo	Not Fo	High	0.009524	0.05906	3.94	160.51
837812	730113	516965	8856	6735		41		Light	High	Peak Fo	High	Peak Fo	Peak Fo	Not Fo	High	0.009524	0.0182	3.5	169.23
223682	18247					40		Light	High	Peak Fo	Not Fo	Not Fo	Not Fo	Not Fo	High	0.007576	0.09923	4.8	84.06
29315	6373	25993	39708		88606	39		Heavy	Peak Fo	Peak Fo	Peak Fo	Peak Fo	Not Fo	High	High	0.009524	0.1396	2.29	139.75
93475	74580	26900		9563	11929	19		Light	High	Peak Fo	High	Not Fo	Peak Fo	Peak Fo	High	0.009524	0.03027	5.32	164.77
171135	173915	120921				18		Light	High	Peak Fo	High	Not Fo	Not Fo	Not Fo	High	0.009524	0.01554	5.81	110.29
199969						17		Light	High	Not Fo	Not Fo	Not Fo	Not Fo	Not Fo	High	0.007576	0.1267	4.54	141.34
56550	14176	43065				16		Light	High	Peak Fo	High	Not Fo	Not Fo	Not Fo	High	0.009524	0.1503	3.87	180.53
104491	44313	166246				15		Light	High	Peak Fo	High	Not Fo	Not Fo	Not Fo	High	0.009524	0.08536	4.02	82.68
180204	938439	412612	52530	137040	98891	14		Heavy	Peak Fo	Peak Fo	Peak Fo	Peak Fo	Peak Fo	High	High	0.009524	0.1005	3.4	170.87
201507	118113	83467				13		Light	High	Peak Fo	High	Not Fo	Not Fo	Not Fo	High	0.009524	0.01922	3.79	121.85
287237	75175	153072	16589	8605	20435	12		Light	High	Peak Fo	High	Peak Fo	Peak Fo	Peak Fo	High	0.009524	0.05666	3.6	68.51
						11	NoQua	Light	Not Fo	Not Fo	High	Not Fo	Not Fo	Not Fo	High	0.009524	0.1209	2.89	
40741	234266	497500				10		Light	High	Peak Fo	Peak Fo	Not Fo	Not Fo	Not Fo	High	0.007576	0.2278	4.58	199.81
5670736	7635451	5687050				9		Light	High	Peak Fo	High	Not Fo	Not Fo	Not Fo	High	0.009524	0.08756	4.1	60.89
			10948		24308	8		Heavy	Not Fo	Not Fo	Not Fo	Peak Fo	Not Fo	High	High	0.009524	0.07217	4.73	188.99
93306		2522				7		Light	High	Not Fo	Peak Fo	Not Fo	Not Fo	Not Fo	High	0.007576	0.1164	6.28	134.45
221806	64940	168317	57903	20431	84444	6		Light	High	Peak Fo	Peak Fo	Peak Fo	Peak Fo	Peak Fo	High	0.007576	0.1277	3.44	73.38
34022		355968	9603		112848	5		Light	High	Not Fo	High	Peak Fo	Not Fo	Peak Fo	High	0.009524	0.1293	5.02	204.76
4393702	7842554	4190097				4		Light	High	Peak Fo	High	Not Fo	Not Fo	Not Fo	High	0.009524	0.01944	7.14	138.67
4072672	7299123	5771841				3		Light	High	Peak Fo	High	Not Fo	Not Fo	Not Fo	High	0.007576	0.01420443	6.95	112.96
42744	26570	30625	3683		5142	2		Light	Peak Fo	Peak Fo	High	Peak Fo	Not Fo	Peak Fo	High	0.009524	0.1385	3.07	138.93
41375	9480	26302				1		Light	High	Peak Fo	Peak Fo	Not Fo	Not Fo	Not Fo	High	0.007576	0.0142	7.73	67.77
731630	405943	357046				20		Light	High	Peak Fo	High	Not Fo	Not Fo	Not Fo	High	0.009524	0.06091	4.57	103.93
259410		694343	9055	20691	6556	83		Light	High	Not Fo	High	Peak Fo	Peak Fo	Peak Fo	High	0.009524	0.1418	2.96	194.52
2244644	4262019	1995427				21		Light	High	Peak Fo	High	Not Fo	Not Fo	Not Fo	High	0.009524	0.03012	5.19	186.21
307411	8299	54486				23		Light	High	Peak Fo	High	Not Fo	Not Fo	Not Fo	High	0.009524	0.1124	4.62	43.78
40128	16041	28537				38		Light	High	Peak Fo	Peak Fo	Not Fo	Not Fo	Not Fo	High	0.007576	0.05304	2.87	58.29
65711	46955	57586				37		Light	Peak Fo	Peak Fo	High	Not Fo	Not Fo	Not Fo	High	0.009524	0.09985	2.07	58.7
3283452	3307397	1986920				36		Light	High	Peak Fo	High	Not Fo	Not Fo	Not Fo	High	0.007576	0.04276	5.99	150.14
943981	1709119	1190312				35		Light	High	Peak Fo	High	Not Fo	Not Fo	Not Fo	High	0.007576	0.0142	6.14	102.14
41618	32396	49470				34		Light	High	Peak Fo	High	Not Fo	Not Fo	Not Fo	High	0.007576	0.09083	4.41	117.15
21154	56835	100006	6782		23898	33		Light	Peak Fo	Peak Fo	High	Peak Fo	Not Fo	Peak Fo	High	0.009524	0.1084	3.99	134.04
							InconsistentlyLa		Not Fo	Not Fo	Not Fo	Not Fo	Not Fo	Not Fo	High	0.009524	0.1139	1.71	102.06
						32	NoQua	Heavy	Not Fo	Not Fo	Not Fo	Not Fo	Not Fo	High	High	0.009524	0.06356	4.82	
2498	19359	106012			13576	31		Light	Peak Fo	Peak Fo	High	Not Fo	Not Fo	Peak Fo	High	0.009524	0.1055	3.16	144.35
		227957	4122		58705	30		Heavy	Not Fo	Not Fo	Peak Fo	Peak Fo	Not Fo	High	High	0.009524	0.09345	3.01	141.3
						30	Redun	Light	Not Fo	Not Fo	High	Peak Fo	Not Fo	Peak Fo	High	0.009524	0.06003	2.59	141.31
96952	127734	95173				29		Light	High	Peak Fo	High	Not Fo	Not Fo	Not Fo	High	0.007576	0.1068	4.95	63.27
						28	Redun	Heavy	Peak Fo	Peak Fo	Peak Fo	Peak Fo	Peak Fo	High	High	0.009524	0.06197	5.4	52.99

163333	954217	631313	121061	313170	227158	28		Light	High	Peak Fo	High	Peak Fo	Peak Fo	Peak Fo	High	0.009524	0.0301	5.84	52.99
						27	Redund	Heavy	Peak Fo	Peak Fo	Peak Fo	High	Peak Fo	High	High	0.009524	0.08365	4.26	22.94
1225202	572051	1779299	299464	85267	443532	27		Light	High	Peak Fo	High	Peak Fo	Peak Fo	Peak Fo	High	0.009524	0.05004	4.75	22.94
47663	19352	36316				26		Light	High	Peak Fo	High	Not Fou	Not Fo	Not Fou	High	0.009524	0.0915	3.79	146.76
78340	12515	15878				25		Light	High	Peak Fo	Peak Fo	Not Fou	Not Fo	Not Fou	High	0.007576	0.01927	6.35	38.55
232213	81572	83687	4005			24		Light	High	Peak Fo	High	Peak Fo	Not Fo	Not Fou	High	0.009524	0.0525	5.75	139.83
148720	26765	38309				22		Light	High	Peak Fo	Peak Fo	Not Fou	Not Fo	Not Fou	High	0.007576	0.08377	5.39	47.83
1342856	1532910	1052667				84		Light	High	Peak Fo	High	Not Fou	Not Fo	Not Fou	High	0.007576	0.1046	3.53	115.91

Supplemental Table 4.3. Raw Proteome discoverer output from the three replicates of Condition C

Checke	Confid	Sequence	Modifications	Contami	Qvality PEP	Qval	Num	Num	Num	Master Pr	Master Protein	Protei	Nur	Theo M	Abundances	Abundan	Abunda	Abunda	Abundance I	Abundance F
FALSE	High	AAFGGSGGRGSSSGGGYSSG		TRUE	1.30E-05	0	1	1	1	P35908	Keratin, type II	P3590	1	2501	23168		52.5		15248	42876
FALSE	High	NTDGSTDYGILQINSR		TRUE	0.000719	0	1	1	4	cRAP078	Lysozyme C OS	cRAP0	0	1754	293812		30.79		293812	303813
FALSE	High	NVQALEIELQSQLALK		TRUE	0.041544	0	1	1	1	P13645	Keratin, type I (	P1364	0	1797						
FALSE	High	QVHPDTGISSK		FALSE	0.087527	0	2	15	2	Q16778; C	Histone H2B ty	Q1677	0	1169	154430	37923	99.82	75.22	154430	599198
FALSE	High	QVQSLTCEV	1xCarbamidom	FALSE	0.063161	0	1	1	1	P08670	Vimentin OS=H	P0867	1	2377	18431		105.38		18431	57463
FALSE	High	RDTLLLELK		TRUE	0.034554	0	1	1	10	cRAP117	000000|rLys-C	cRAP1	1	1101	25409181		26.29		25409181	32893649
FALSE	High	RDTLLLELKR		TRUE	0.014499	0	1	1	7	cRAP117	000000|rLys-C	cRAP1	2	1257	2083050		45.02		2975330	2083050
FALSE	High	RGDLPFVVPR		FALSE	0.039197	0	1	1	3	P35579	Myosin-9 OS=H	P3557	1	1156	223080		33.61		223080	311783
FALSE	High	RKASGPPVSELITK		FALSE	0.064184	0	2	2	1	P16402; P	Histone H1.3 O	P1640	2	1483	55839	16959	73.59	42.91	55839	113532
FALSE	High	RKPDTIEVQQMK		FALSE	0.003237	0	1	3	3	P35241	Radixin OS=Ho	P3524	1	1473	515492		24.71		590632	515492
FALSE	High	RKPDTIEVQ(	1xOxidation [M	FALSE	0.02616	0	1	3	2	P35241	Radixin OS=Ho	P3524	1	1489	45052		27.95		60166	45052
FALSE	High	RKPDTIEVQQMKAQAR		FALSE	0.030557	0	1	3	2	P35241	Radixin OS=Ho	P3524	2	1899	67966		14.5		67966	82354
FALSE	High	NMAINPSKE	2xCarbamidom	TRUE	0.024439	0	1	1	1	cRAP014	Alpha-S2-casei	cRAP0	2	2497	25146		113.94		8250	76644
FALSE	High	RKTVTAMDVVYALK		FALSE	0.001462	0	1	1	3	P62805	Histone H4 OS=	P6280	2	1595	21898	7103	120.09	92.65	21898	89281
FALSE	High	RTPPAGVFYQGWSATPIANG		TRUE	0.000154	0	1	1	14	cRAP117	000000|rLys-C	cRAP1	2	3611	17143618		27.03		17143618	25095979
FALSE	High	RVTIMPK	2xLabel:13C(6)	FALSE	0.158995	0	1	5	1	P84243	Histone H3.3 O	P8424	1	857	164674	38401	78.02	49.04	164674	351674
FALSE	High	RVTIMPKDIQLAR		FALSE	0.058097	0	1	5	2	P84243	Histone H3.3 O	P8424	2	1541	419746	100357	65.12	33.73	419746	864734
FALSE	High	RVTIMPKDI(	1xOxidation [M	FALSE	0.030557	0	1	5	2	P84243	Histone H3.3 O	P8424	2	1557	119908	24640	61.75	27.96	119908	222479
FALSE	High	SADTLWDIQ	2xLabel:13C(6)	FALSE	0.20043	0	1	1	1	P07195	L-lactate dehyc	P0719	2	1773	22009	55908				22009
FALSE	High	SCAAAGTEC	2xCarbamidom	TRUE	0.000128	0	1	1	2	cRAP112	Trypsin OS=Sus	cRAP1	0	1883	263061		48.5		99633	263061
FALSE	High	SCQAQPTTN	1xCarbamidom	TRUE	0.008706	0	1	1	2	cRAP016	Kappa-casein C	cRAP0	0	1251	66023		106		66023	261664
FALSE	High	SGDAAIVDN	1xCarbamidom	FALSE	0.022906	0	1	2	1	P68104	Elongation fact	P6810	0	2995						
FALSE	High	SGGGGGGG	1xCarbamidom	FALSE	0.036154	0	1	1	1	P13647	Keratin, type II	P1364	1	2257	14512		68.9		14512	34950
FALSE	High	SGGGGGGG	1xCarbamidom	TRUE	0.0001	0	1	1	1	P13645	Keratin, type I (	P1364	0	1550	22936		48.08		22936	45267
FALSE	High	SGGGGGGGLGSGGSIR		TRUE	0.000287	0	1	1	2	P35527	Keratin, type I (	P3552	0	1233	132472		22.57		132472	186149
FALSE	High	RTPPAGVFYQGWSATPIANG		TRUE	0.006866	0	1	1	6	cRAP117	000000|rLys-C	cRAP1	1	3240	1409932		52.57		1409932	1869592
FALSE	High	SGVSLAALK		FALSE	0.050747	0	2	3	1	P16402; P	Histone H1.3 O	P1640	0	846	14244	21348	107.61		14244	74293
FALSE	High	NMAINPSKE	2xCarbamidom	TRUE	0.144894	0	1	1	1	cRAP014	Alpha-S2-casei	cRAP0	1	2014	214409					214409
FALSE	High	NIETIINTFHQYSVK		FALSE	0.180284	0	1	1	1	P06702	Protein S100-A	P0670	0	1807						
FALSE	High	IITHPNFNGNTLDNDIMLIKLS		TRUE	0.0326	0	1	1	2	cRAP112	Trypsin OS=Sus	cRAP1	1	3310	624786		38.78		452225	972316
FALSE	High	IQVRLGEHNIDVLEGNEQFIN/		TRUE	0.048821	0	1	1	2	cRAP112	Trypsin OS=Sus	cRAP1	1	2707	27339		22.82		25500	38327
FALSE	High	LSAKPAPPKPEPKPK		FALSE	0.22784	0	1	1	1	P05204	Non-histone ch	P0520	0	1585	122333	11782	54.86	33.98	122333	233706
FALSE	High	LSAKPAPPKPEPKPR		FALSE	0.095674	0	1	1	1	Q15651	High mobility g	Q1565	0	1613	10597		122.83		10597	56689
FALSE	High	LSAKPAPPKPEPRPK		FALSE	0.196249	0	1	1	2	O00479	High mobility g	O0047	0	1613	36507	2595	119.3		36507	201872
FALSE	High	ISGLIYEETR		FALSE	0.019166	0	1	1	2	P62805	Histone H4 OS=	P6280	0	1181	231603	16336	56.48	68.88	197903	524449
FALSE	High	ISGLIYEETRGVLK		FALSE	0.125901	0	1	1	1	P62805	Histone H4 OS=	P6280	1	1578						
FALSE	High	ISLPLPNFSSLNLRETNLDSLPL		FALSE	0.074142	0	1	1	1	P08670	Vimentin OS=H	P0867	2	3377	20293		128.77			93783
FALSE	High	LSSPATLNSR		TRUE	0.111366	0	1	1	2	cRAP112	Trypsin OS=Sus	cRAP1	0	1046	1936401		22.76		1936401	2498953
FALSE	High	ISTVVSSKEV	3xLabel:13C(6)	FALSE	0.048821	0	1	1	1	P37108	Signal recognit	P3710	2	2532	7324	20367	102.92		2907	18452
FALSE	High	LTGKDVNFE	1xLabel:13C(6)	FALSE	0.114932	0	1	1	1	P62081	40S ribosomal	P6208	1	1790	41534		10.31		38610	44680

FALSE	High	NLQEAEEWYK		FALSE	0.018494	0	1	2	1	P08670	Vimentin OS=H	P0867	0	1310	17548		101.66		17548	51184
FALSE	High	ITIADCGQLE	1xCarbamidom	TRUE	0.063978	0	1	1	2	P62937	Peptidyl-prolyl	P6293	0	1120						
FALSE	High	MFLRGDSVIVVLRNPLIAGK		FALSE	0.164978	0	1	1	1	P62316	Small nuclear r	P6231	2	2198	40000	8558		93.27		40000
FALSE	High	MFLRGDSVI	3xLabel:13C(6)	FALSE	0.032917	0	1	1	1	P62316	Small nuclear r	P6231	2	2216						
FALSE	High	MKYLFFSWL	1xCarbamidom	FALSE	0.175403	0	1	1	1	Q5T7M9	Protein FAM69	Q5T7N	1	3799	8787	4621	106.8	34.99	7313	46839
FALSE	High	MIAGQVLDINLAAEPK		FALSE	0.052918	0	1	1	1	P07910	Heterogeneous	P0791	0	1683						
FALSE	High	MIAGQVLDI	1xLabel:13C(6)	FALSE	0.004677	0	1	1	1	P07910	Heterogeneous	P0791	0	1689	7689	29166	120.71		7689	45717
FALSE	High	MVFTSSADG	1xCarbamidom	TRUE	4.69E-05	0	1	1	17	cRAP117	000000|rLys-C	cRAP1	0	2691	10457346	27286	28.04	84.88	7625672	13533387
FALSE	High	MVFTSSADG	1xCarbamidom	TRUE	8.49E-05	0	1	1	11	cRAP117	000000|rLys-C	cRAP1	0	2707	259130	44180	17.25		259130	322097
FALSE	High	NDEELNKLLGR		FALSE	0.159978	0	1	3	1	Q93077	Histone H2A ty	Q7L7L	1	1301	13759	10828	123.52	17.56	13759	109257
FALSE	High	NDYFSDFSGVYSQISR		TRUE	0.000227	0	1	1	7	cRAP117	000000|rLys-C	cRAP1	0	1885	2601187		46.08		2601187	2744995
FALSE	High	NGLSLAALKI	3xLabel:13C(6)	FALSE	0.116023	0	1	1	1	P16401	Histone H1.5 O	P1640	2	2107	12269	34387				12269
FALSE	High	NLCNIPCSAL	3xCarbamidom	TRUE	0.00137	0	1	1	4	cRAP078	Lysozyme C OS	cRAP0	0	2508	628974	3636	40.41	48.55	476683	1034335
FALSE	High	LYAEERYPILI	1xCarbamidom	TRUE	0.15039	0	1	1	1	cRAP086	Ovalbumin OS=	cRAP0	2	2845						
FALSE	High	IITHPNFNGN	1xOxidation [M	TRUE	0.047119	0	1	1	4	cRAP112	Trypsin OS=Sus	cRAP1	0	2299	268084		47.46		268084	503479
FALSE	High	SGVSLAALKK		FALSE	0.016402	0	2	3	1	P16402; P	Histone H1.3 O	P1640	1	974	70160	29407	90.47	67.18	70160	171299
FALSE	High	SLDLDSIIAEVK		TRUE	0.011817	0	1	1	1	P04264	Keratin, type II	P0426	0	1303	39862		18.88		34853	45590
FALSE	High	VATVSLPR		TRUE	0.072965	0	1	1	3	cRAP112	Trypsin OS=Sus	cRAP1	0	843	18005166		15.03		18237082	18005166
FALSE	High	VCNYVNWI	1xCarbamidom	TRUE	0.063568	0	1	1	1	cRAP112	Trypsin OS=Sus	cRAP1	0	1794						
FALSE	High	VDWPSAVVEGGSSGSGLLTV		TRUE	0.000199	0	1	1	6	cRAP117	000000|rLys-C	cRAP1	0	2864	568199		14.41		511383	677496
FALSE	High	VDWPSAVV	1xCarbamidom	TRUE	0.077536	0	1	1	1	cRAP117	000000|rLys-C	cRAP1	1	4573						
FALSE	High	VERADGYEPPVQESV		FALSE	0.067565	0	1	1	1	P61247	40S ribosomal	P6124	1	1675	69997	56631	48.47	33.83	69997	90440
FALSE	High	VFLENVIR		FALSE	0.100334	0	1	1	1	P62805	Histone H4 OS=	P6280	0	990	25007	6471	119.61	96.19	25007	170334
FALSE	High	VFLENVIRDAVTYTEHAK		FALSE	0.058097	0	1	1	2	P62805	Histone H4 OS=	P6280	1	2105	43788	55063	50.25	23.41	43788	26060
FALSE	High	VHPAVVIR		FALSE	0.07438	0	1	1	1	P62829	60S ribosomal	P6282	0	891	91940	1635	16.42	62.71	91940	95732
FALSE	High	VLKQVHPDTGISSK		FALSE	0.063773	0	2	15	3	Q16778; C	Histone H2B ty	Q1677	1	1509	104612	25057	76.82	48.64	104612	256183
FALSE	High	VLPVPQKAVPYPQR		TRUE	0.203463	0	1	1	1	cRAP015	Beta-casein OS	cRAP0	1	1592	78015		120.08		22289	273067
FALSE	High	VTIAQGGVLPNIQAVLLPK		FALSE	0.000169	0	2	10	5	Q16777; C	Histone H2A ty	Q1677	0	1931	929054	263588	114.55	87.55	929054	4182895
FALSE	High	VAPEEHPVLLTEAPLNPK		FALSE	0.018919	0	1	2	1	P60709	Actin, cytoplas	P6070	0	1954	52653	10873	81.84	51.36	52653	130167
FALSE	High	VTIAQGGVL	1xLabel:13C(6)	FALSE	0.199828	0	2	10	2	Q16777; C	Histone H2A ty	Q1677	0	1937						
FALSE	High	VTIAQGGVLPNIQAVLLPKKTI		FALSE	0.06649	0	1	1	1	Q16777	Histone H2A ty	Q1677	2	2642	9298		92.86		9298	28893
FALSE	High	VTIMPKDIQLAR		FALSE	0.114571	0	1	5	2	P84243	Histone H3.3 O	P8424	1	1385	178059	37362	93.07	59.44	178059	591331
FALSE	High	WWCNDGR	1xCarbamidom	TRUE	0.124331	0	1	1	1	cRAP078	Lysozyme C OS	cRAP0	0	993	45752		30.13		36850	56804
FALSE	High	WWCNDGRT	1xCarbamidom	TRUE	0.059229	0	1	1	1	cRAP078	Lysozyme C OS	cRAP0	1	1492	150658		38.46		150658	166149
FALSE	High	YADLTEDQL	1xCarbamidom	FALSE	0.120866	0	1	1	2	P18669	Phosphoglycer	P1866	1	2437		49149		61.06		
FALSE	High	YALYDATYETK		FALSE	0.071348	0	1	2	2	P23528	Cofilin-1 OS=H	P2352	0	1338	88240	23967	59.79	47.23	88240	161864
FALSE	High	YALYDATYET	1xLabel:13C(6)	FALSE	0.180284	0	1	2	1	P23528	Cofilin-1 OS=H	P2352	0	1344						
FALSE	High	YEKDIAAYR		FALSE	0.042631	0	1	2	1	P26583	High mobility g	P2658	1	1129	324092	1500	16.33		324092	339278
FALSE	High	YEKDIAAYRAK		FALSE	0.205918	0	1	2	2	P26583	High mobility g	P2658	2	1328	121464	1853	19.89	71.35	157296	107648
FALSE	High	YLGYLEQLLR		TRUE	0.035574	0	1	1	3	cRAP013	Alpha-S1-casei	cRAP0	0	1268	338391	31279	159.2	34.6	338391	6035232
FALSE	High	YRPGTVALR		FALSE	0.151325	0	1	5	2	P84243	Histone H3.3 O	P8424	0	1033	265681	35127	80.63	76.01	265681	814862
FALSE	High	VTIAQGGVLPNIQAVLLPKKTI		FALSE	0.008	0	1	8	1	Q93077	Histone H2A ty	Q7L7L	2	2779	8664	2674	32.09		6877	10915

FALSE	High	SGVSLAALKI	3xLabel:13C(6)	FALSE	0.043185	0	2	3	1	P16402; P	Histone H1.3 O	P1640	2	2080	150952	37742				150952
FALSE	High	TYSLGSALRPSTSR		FALSE	0.049295	0	1	1	1	P08670	Vimentin OS=H	P0867	0	1496	38737		124.04		9911	151407
FALSE	High	TVTAMDVV'	1xOxidation [M	FALSE	0.100334	0	1	1	2	P62805	Histone H4 OS=	P6280	0	1327	54819	7653	95.46	102.9	54819	181129
FALSE	High	SISISVAR		TRUE	0.105212	0	1	1	1	P04264	Keratin, type II	P0426	0	832	338942		11.62		292698	338942
FALSE	High	SLVNLGGSK		TRUE	0.062155	0	1	1	4	P04264	Keratin, type II	P0426	0	874	930681		38.77		930681	1261590
FALSE	High	SLVNLGGSKSISISVAR		TRUE	0.032285	0	1	1	1	P04264	Keratin, type II	P0426	1	1688	23151		66.49		10170	44222
FALSE	High	SPAQILQWQVLSNTVPAK		TRUE	0.016086	0	1	1	4	cRAP016	Kappa-casein C	cRAP0	0	1980	157734		130.75		157734	1107873
FALSE	High	SPAQILQWC	1xCarbamidom	TRUE	0.006028	0	1	1	2	cRAP016	Kappa-casein C	cRAP0	1	3212	64485		113.87		21186	196281
FALSE	High	SPAQILQWC	1xCarbamidom	TRUE	0.008593	0	1	1	1	cRAP016	Kappa-casein C	cRAP0	1	3228	84172					84172
FALSE	High	SQRAGLQFPVGR		FALSE	0.092683	0	1	2	1	Q71UI9	Histone H2A.V	Q71UI	1	1316	47042	7174	44.05	48.06	47042	87394
FALSE	High	SRGSGGLGG	1xCarbamidom	FALSE	0.0004	0	1	3	1	P04259	Keratin, type II	P0425	1	1668	22980		117.04		14387	134761
FALSE	High	SSGPTSLFAVTVAPPGAR		FALSE	0.149925	0	1	1	1	Q00839	Heterogeneous	Q0083	0	1715						
FALSE	High	SSLLEKGLDGAKK		FALSE	0.102908	0	1	1	1	P81605	Dermcidin OS=	P8160	2	1346	46600		43.12		51542	46600
FALSE	High	STELLIR		FALSE	0.160472	0	1	5	2	P84243	Histone H3.3 O	P8424	0	831	800295	87946	66.12	85	800295	1746260
FALSE	High	TVTAMDVVYALKR		FALSE	0.134454	0	1	1	1	P62805	Histone H4 OS=	P6280	1	1467	25422	5768	82.14	54.53	25422	59816
FALSE	High	STITSREIQTAVR		FALSE	0.016998	0	1	13	4	Q16778	Histone H2B ty	Q1677	1	1462	407059	126464	81.99	45.71	407059	1162101
FALSE	High	STSSFSCLSR	1xCarbamidom	TRUE	0.090356	0	1	1	2	P35908	Keratin, type II	P3590	0	1132	121617		67.26		121617	293585
FALSE	High	SYELPDGQVITIGNER		FALSE	0.00212	0	1	10	2	P60709	Actin, cytoplas	A5A3E	0	1791	40516	8980	71.26	16.9	40516	72786
FALSE	High	TKVIPYVR		TRUE	0.086144	0	1	1	1	cRAP014	Alpha-S2-casei	cRAP0	1	976	38674		124.05		38674	224218
FALSE	High	TKVIPYVRYL		TRUE	0.192727	0	1	1	1	cRAP014	Alpha-S2-casei	cRAP0	2	1252	18842		112.25		18842	102964
FALSE	High	TIGISVDPR		FALSE	0.169598	0	1	1	1	P26373	60S ribosomal	P2637	0	958						
FALSE	High	TIPSWATLSASQLAR		FALSE	0.212169	0	1	1	1	Q5SSJ5	Heterochroma	Q5SSJ!	0	1602	37080	7440				37080
FALSE	High	TPPAGVFYQGWSATPIANGSI		TRUE	0.003083	0	1	1	3	cRAP117	000000|rLys-C	cRAP1	0	3084	563851		59.22		592212	563851
FALSE	High	TPPAGVFYQGWSATPIANGSI		TRUE	0.040484	0	1	1	4	cRAP117	000000|rLys-C	cRAP1	1	3455	1949843		57.03		2012182	1949843
FALSE	High	TPPAGVFYQGWSATPIANGSI		TRUE	0.012366	0	1	1	7	cRAP117	000000|rLys-C	cRAP1	2	3583	985990		65.62		985990	1041720
FALSE	High	TTLCVSILERI	1xCarbamidom	FALSE	0.160967	0	1	1	1	Q16401	26S proteasom	Q164C	2	2942						
FALSE	High	TVTAMDVV'	1xLabel:13C(6)	FALSE	0.22784	0	1	1	1	P62805	Histone H4 OS=	P6280	0	1317	52305	8404	114.31	101.07	52305	257357
FALSE	High	STITSREIQT/	2xLabel:13C(6)	FALSE	0.043325	0	1	13	1	Q16778	Histone H2B ty	Q1677	1	1474						
FALSE	High	IITHPNFNGNTLDNDIMLIK		TRUE	0.001531	0	1	1	5	cRAP112	Trypsin OS=Sus	cRAP1	0	2283	2062590		64.31		1578225	5001836
FALSE	High	LLRKGNYAER		FALSE	0.072732	0	2	9	2	Q16777; C	Histone H2A ty	Q1677	2	1220	90361	15202	56.03	56.49	90361	183926
FALSE	High	LIQENVVSVI	1xLabel:13C(6)	FALSE	0.204688	0	1	1	1	Q9P2D7	Dynein heavy c	Q9P2D	1	3796						
FALSE	High	AYDNATAA\	1xCarbamidom	TRUE	0.000203	0	1	1	2	cRAP117	000000|rLys-C	cRAP1	1	3767	476341		21.42		626947	476341
FALSE	High	AYDNATAA\	1xCarbamidom	TRUE	0.120866	0	1	1	1	cRAP117	000000|rLys-C	cRAP1	2	3923						
FALSE	High	CELAAAMKF	1xCarbamidom	TRUE	0.085054	0	1	1	1	cRAP078	Lysozyme C OS	cRAP0	1	1050	103086		37.32		117233	103086
FALSE	High	CNKEVYFAEI	1xCarbamidom	FALSE	0.036154	0	1	1	2	P50238	Cysteine-rich p	P5023	1	1316	202990	24628	46.97	19.64	292308	202990
FALSE	High	CSQAVYAAE	1xCarbamidom	FALSE	0.112071	0	1	1	2	P21291	Cysteine and gl	P2129	1	1652	164138		27.17		212245	164138
FALSE	High	DAEAWFNEK		TRUE	0.064805	0	1	4	1	P13645	Keratin, type I (	Q7Z3Y	0	1109	33988		28.42		26071	46027
FALSE	High	DAKRDTLLLELK		TRUE	0.020784	0	1	1	2	cRAP117	000000|rLys-C	cRAP1	2	1415	135205		26.84		163334	135205
FALSE	High	DGFGASGSC	2xCarbamidom	TRUE	1.15E-05	0	1	1	6	cRAP117	000000|rLys-C	cRAP1	0	2232	397312		37.64		397312	422952
FALSE	High	DGFGASGSC	2xCarbamidom	TRUE	0.001305	0	1	1	2	cRAP117	000000|rLys-C	cRAP1	1	3307	44814		21.74		44814	46690
FALSE	High	DGQVINETSQHHDDLE		FALSE	0.158017	0	1	1	2	P08670	Vimentin OS=H	P0867	0	1837						
FALSE	High	DNIQGITKPAIR		FALSE	0.025408	0	1	1	3	P62805	Histone H4 OS=	P6280	0	1326	335535	51605	88.63	72.41	335535	1016714

FALSE	High	AYDNATAAVAK		TRUE	0.004106	0	1	1	5	cRAP117	000000|rLys-C	cRAP1	0	1095	8386054	1424	39.14		8386054	11817950
FALSE	High	DTLLLELK		TRUE	0.14625	0	1	1	2	cRAP117	000000|rLys-C	cRAP1	0	945	136043	3517	53.04	41.76	136043	153569
FALSE	High	EGIHAQQKE	1xOxidation [M	TRUE	0.183605	0	1	1	1	cRAP013	Alpha-S1-casei	cRAP0	1	3224	29082					29082
FALSE	High	ELINSWVESQTNGIIR		TRUE	0.025823	0	1	1	1	cRAP086	Ovalbumin OS=	cRAP0	0	1859						
FALSE	High	EIQTAVRLLLPGELAK		FALSE	0.036037	0	1	14	5	Q16778	Histone H2B ty	Q1677	1	1751	851452	171849	116.74	90.96	851452	4633597
FALSE	High	EIQTAVRLLL	2xLabel:13C(6)	FALSE	0.015075	0	1	14	1	Q16778	Histone H2B ty	Q1677	1	1763						
FALSE	High	EVQTAVRLLLPGELAK		FALSE	0.175939	0	1	1	1	Q8N257	Histone H2B ty	Q8N25	1	1737	10422	11651	115.75	110.21	10422	57806
FALSE	High	EVQTAVRLLI	2xLabel:13C(6)	FALSE	0.172743	0	1	1	1	Q8N257	Histone H2B ty	Q8N25	1	1749						
FALSE	High	FESNFNTQATNR		TRUE	0.009351	0	1	1	2	cRAP078	Lysozyme C OS	cRAP0	0	1429	131975		38.79		133030	131975
FALSE	High	FFSDKIAK		TRUE	0.154638	0	1	1	1	cRAP016	Kappa-casein C	cRAP0	1	956	11642		96.16		11642	33455
FALSE	High	FFVAPFPEVFGK		TRUE	0.15321	0	1	1	2	cRAP013	Alpha-S1-casei	cRAP0	0	1385	459240		130.44		459240	3257098
FALSE	High	FFVAPFPEVFGKEK		TRUE	0.005854	0	1	1	2	cRAP013	Alpha-S1-casei	cRAP0	1	1642	204644		138.8		204644	1961150
FALSE	High	FLEQQNKILLAELEQLKGQGK		FALSE	0.004454	0	1	1	2	P08670	Vimentin OS=H	P0867	2	2427	61159					61159
FALSE	High	EGIHAQQKEPMIGVNQELAYI		TRUE	0.106214	0	1	1	1	cRAP013	Alpha-S1-casei	cRAP0	1	3208						
FALSE	High	FSSCGGGGG	1xCarbamidom	TRUE	0.000918	0	1	1	3	P04264	Keratin, type II	P0426	0	1766	134372		21.84		134372	177916
FALSE	High	AVKPKAAKPK		FALSE	0.049775	0	2	3	3	P16401; P	Histone H1.5 O	P1640	1	1038	98013	38421	93.39	72.95	98013	292145
FALSE	High	ASGQAFELIL	1xLabel:13C(6)	FALSE	0.021123	0	1	1	1	P16949	Stathmin OS=H	P1694	0	1395		30348		48.74		
FALSE	High	AGFAGDDAPR		FALSE	0.02133	0	1	10	1	P60709	Actin, cytoplas	A5A3E	0	976	159146	43474	73.45	66	159146	383623
FALSE	High	AGFAGDDAI	1xLabel:13C(6)	FALSE	0.020649	0	1	10	2	P60709	Actin, cytoplas	A5A3E	0	982						
FALSE	High	AGFAGDDAPRAVFPSIVGRPF		FALSE	0.092683	0	1	9	1	P60709	Actin, cytoplas	A5A3E	1	2156	22136	6101	92.88	42.23	22136	52190
FALSE	High	AGLQFPVGR		FALSE	0.016725	0	3	15	2	Q16777; C	Histone H2A ty	Q1677	0	945	2562606	508858	88.6	67.14	2562606	7816538
FALSE	High	AGLQFPVGRVHR		FALSE	0.097203	0	2	12	1	Q16777; C	Histone H2A ty	Q1677	1	1337						
FALSE	High	AGLQFPVGR	2xLabel:13C(6)	FALSE	0.16753	0	2	12	1	Q16777; C	Histone H2A ty	Q1677	1	1349	173277	39685	75.86	44.87	173277	427179
FALSE	High	AGYRDGFG/	2xCarbamidom	TRUE	3.08E-05	0	1	1	12	cRAP117	000000|rLys-C	cRAP1	1	2679	8177292		16.55		8177292	10001487
FALSE	High	AGYRDGFG/	2xCarbamidom	TRUE	8.80E-06	0	1	1	9	cRAP117	000000|rLys-C	cRAP1	2	3755	7345876		7.75		6967360	8104098
FALSE	High	AHVEHMQS	1xOxidation [M	FALSE	0.196842	0	1	1	1	Q13029	PR domain zinc	Q1302	0	2064	2943165		8.94		2943165	3335101
FALSE	High	AKAPKPKAAKPK		FALSE	0.007351	0	1	1	1	P16402	Histone H1.3 O	P1640	2	1235	30126	5585	106.03	56.51		79658
FALSE	High	AKAVKPKAAKPK		FALSE	0.05942	0	1	1	1	P10412	Histone H1.4 O	P1041	2	1237	22924	4392	110.68	73.24	22924	101131
FALSE	High	AVFVDLEPT	1xLabel:13C(6)	FALSE	0.02764	0	1	3	3	Q71U36	Tubulin alpha-1	Q71U3	0	1708	7443	37469	40.23	75.21	5555	9972
FALSE	High	ALAAAGYDVEK		FALSE	0.008849	0	3	5	1	P16402; P	Histone H1.3 O	P1640	0	1108	55986	22797	121.71	98.91	55986	339908
FALSE	High	ALAAAGYDV	2xLabel:13C(6)	FALSE	0.131545	0	3	5	1	P16402; P	Histone H1.3 O	P1640	1	1591						
FALSE	High	ALCSEKLDQ	2xCarbamidom	TRUE	0.015272	0	1	1	2	cRAP075	Alpha-lactalbu	cRAP0	2	1893	18699		145.14		18699	293387
FALSE	High	AMGIMNSFVNDIFER		FALSE	0.001904	0	2	14	3	Q16778; C	Histone H2B ty	Q1677	0	1744	236583	66805	67.61		236583	360602
FALSE	High	AMGIMNSF	1xLabel:13C(6)	FALSE	0.051075	0	2	14	1	Q16778; C	Histone H2B ty	Q1677	0	1750						
FALSE	High	AMGIMNSF	1xOxidation [M	FALSE	9.09E-05	0	2	14	5	Q16778; C	Histone H2B ty	Q1677	0	1760	355563	98456	135.89	121.31	247926	3592699
FALSE	High	AMGIMNSF	1xOxidation [M	FALSE	0.052578	0	2	14	2	Q16778; C	Histone H2B ty	Q1677	0	1766						
FALSE	High	AMGIMNSF	2xOxidation [M	FALSE	0.006822	0	2	14	2	Q16778; C	Histone H2B ty	Q1677	0	1776	123848	19662	105.63	135.82	123848	553199
FALSE	High	AMGIMNSF	2xOxidation [M	FALSE	0.009474	0	2	14	1	Q16778; C	Histone H2B ty	Q1677	0	1782						
FALSE	High	AMKPWIQPK		TRUE	0.147618	0	1	1	1	cRAP014	Alpha-S2-casei	cRAP0	0	1099	11935		127.02		11935	84374
FALSE	High	ASGPPVSELITK		FALSE	0.062355	0	2	3	1	P16402; P	Histone H1.3 O	P1640	0	1199						
FALSE	High	ASGPPVSELI	1xLabel:13C(6)	FALSE	0.102908	0	2	3	1	P16402; P	Histone H1.3 O	P1640	0	1205	180985	37835	104.45	80.58	180985	686499
FALSE	High	ALAAAGYDVEKNNSR		FALSE	0.196842	0	3	5	2	P16402; P	Histone H1.3 O	P1640	1	1579	72012	27957	102.75	75.76	72012	239097

FALSE	High	GAVHDVKDVLDSVL		FALSE	0.226497	0	1	1	1	P81605	Dermcidin OS=	P8160	1	1467	293617	7357	57.11		191344	450555
FALSE	High	GDEELDSLIK		FALSE	0.141333	0	1	2	1	Q71UI9	Histone H2A.V	Q71UI	0	1119	14224	9620	108.06		5201	38901
FALSE	High	GGLEPINFQTAADQAR		TRUE	0.005989	0	1	1	2	cRAP086	Ovalbumin OS=	cRAP0	0	1688	27849		64.82		27849	49954
FALSE	High	KGTVEGFEP.	1xCarbamidom	FALSE	0.003797	0	1	1	2	P37108	Signal recognit	P3710	2	1934						
FALSE	High	KGTVEGFEP.	1xCarbamidom	FALSE	0.000701	0	1	1	2	P37108	Signal recognit	P3710	2	1952	32665	31068	27.88		39889	26750
FALSE	High	KIEEIKDFLLTAR		FALSE	0.076551	0	1	1	1	P63173	60S ribosomal	P6317	2	1576	32120	3044	40.21		32120	40624
FALSE	High	KIIGLK		FALSE	0.215356	0	1	1	1	Q6IN85	Serine/threonii	Q6IN8	1	671	45555	33993	84.79	65.05	45555	97200
FALSE	High	KTVTAMDVVYALK		FALSE	0.012775	0	1	1	1	P62805	Histone H4 OS=	P6280	1	1439	11346	2288	76.79		11346	21791
FALSE	High	KTVTAMDVVYALKR		FALSE	0.032077	0	1	1	5	P62805	Histone H4 OS=	P6280	2	1595	88764	36117	101.78	67.33	88764	303079
FALSE	High	KTVTAMDV\	1xOxidation [M	FALSE	0.104548	0	1	1	1	P62805	Histone H4 OS=	P6280	2	1611	20111	5468	51.55	38.45	20111	37315
FALSE	High	KVHGSLAR		FALSE	0.166505	0	1	1	1	P62861	40S ribosomal	P6286	1	868	74936	83412	16.74	20.48	66535	84399
FALSE	High	KVPQVSTPTLVEVSR		TRUE	0.014036	0	1	2	4	cRAP087	Serum albumin	cRAP0	1	1640	185260		117.13		871682	185260
FALSE	High	KYSQGNVSAVGVTYDGHTAL		TRUE	0.000407	0	1	1	4	cRAP117	000000|rLys-C	cRAP1	1	2324	1580930		28.02		1580930	1856555
FALSE	High	LAPDYDALDVANKIGII		FALSE	0.002689	0	1	1	2	P62750	60S ribosomal	P6275	1	1801	218603	117227	29.89	53.66	270929	176383
FALSE	High	KATGPPVSE	2xLabel:13C(6)	FALSE	0.021608	0	1	1	2	P16401	Histone H1.5 O	P1640	1	1353	33945	45366	74.96	81.18	33945	64835
FALSE	High	LGEHNIDVLEGNEQFINAAK		TRUE	1.52E-05	0	1	1	4	cRAP112	Trypsin OS=Sus	cRAP1	0	2211	4406445		20.84		4406445	5378594
FALSE	High	LGGSAVISLE	1xLabel:13C(6)	FALSE	0.006476	0	1	1	1	P23528	Cofilin-1 OS=H	P2352	0	1347	61157	13108	63.76	63.2	61157	130659
FALSE	High	LGKDAVEDLESVGK		FALSE	0.002462	0	1	1	1	P81605	Dermcidin OS=	P8160	1	1460	165687		52.44		165687	183755
FALSE	High	IGLGGRGGSGGSYGR		TRUE	0.13572	0	1	1	1	P35527	Keratin, type I	P3552	1	1351	252094		39.69		252094	408383
FALSE	High	LGIKSLVSKGTLVQTK		FALSE	0.07438	0	1	1	1	Q02539	Histone H1.1 O	Q0253	2	1672	24656	5970	87.01	105.48	12033	50520
FALSE	High	IGRIEDVTPII	2xLabel:13C(6)	FALSE	0.065642	0	1	1	1	P62263	40S ribosomal	P6226	1	1768	39121	26261	40.16	27.37	39121	46093
FALSE	High	LLCGLLAER	1xCarbamidom	FALSE	0.098757	0	1	1	2	P14174	Macrophage m	P1417	0	1045						
FALSE	High	LLCGLLAER	1xCarbamidom	FALSE	0.053605	0	1	1	2	P14174	Macrophage m	P1417	0	1051	34677	85209	81.91	73.26	34677	55912
FALSE	High	LLDFGSLSNLQVTQPTVGMNI		FALSE	0.007795	0	1	1	1	P08708	40S ribosomal	P0870	1	2763						
FALSE	High	LLDFGSLSNL	2xLabel:13C(6)	FALSE	0.004601	0	1	1	1	P08708	40S ribosomal	P0870	1	2775	172097	99799				172097
FALSE	High	ILDKVGINYWLAHK		TRUE	0.090933	0	1	1	1	cRAP075	Alpha-lactalbu	cRAP0	1	1670	29708					29708
FALSE	High	LLLPGELAK		FALSE	0.219233	0	2	15	1	Q16778; (	Histone H2B ty	Q1677	0	954	2627716	612357	101.01	75.31	2627716	9988036
FALSE	High	LGEYGFQNALIVR		TRUE	0.045475	0	1	1	2	cRAP087	Serum albumin	cRAP0	0	1480	124285		105.04		323469	47754
FALSE	High	KASGPPVSELITKAVAASK		FALSE	0.087527	0	2	3	1	P16402; P	Histone H1.3 O	P1640	2	1854	9793	6783	114.04		3206	29915
FALSE	High	HQGLPQEVLNENLLR		TRUE	0.018079	0	1	1	2	cRAP013	Alpha-S1-casei	cRAP0	0	1760	47804	2263	10.36	42.51	42208	47804
FALSE	High	HPIKHQGLPQEVLNENLLR		TRUE	0.012816	0	1	1	3	cRAP013	Alpha-S1-casei	cRAP0	1	2235	42857		148.64		42857	533554
FALSE	High	GGLYGGPSY	1xCarbamidom	TRUE	0.001813	0	1	1	4	cRAP117	000000|rLys-C	cRAP1	0	1728	735480		52.98		735480	820584
FALSE	High	GGLYGGPSY	1xCarbamidom	TRUE	0.001267	0	1	1	4	cRAP117	000000|rLys-C	cRAP1	1	3594	3031392		72.23		3031392	5823678
FALSE	High	GGSGGSYGGGGSGGGYGGG		TRUE	0.000535	0	1	1	2	P35527	Keratin, type I	P3552	0	1792	66413		25.68		66413	93609
FALSE	High	GGSISGGGYGSGGGK		TRUE	0.007304	0	1	1	2	P35908	Keratin, type II	P3590	0	1198	46680		82.59		47234	46680
FALSE	High	GHYTEGAEL	1xLabel:13C(6)	FALSE	0.100016	0	1	5	1	Q13885	Tubulin beta-2	Q1388	0	1965						
FALSE	High	GSCGIGGGI	1xCarbamidom	FALSE	0.013153	0	1	2	1	P02533	Keratin, type I	P0253	0	1279	14203		71.97		12586	41901
FALSE	High	GSLGGGFSSGGFSGGSFSR		TRUE	0.000311	0	1	1	3	P13645	Keratin, type I	P1364	0	1708	92047	173055	43.97	29.67	91286	185222
FALSE	High	GTDVQAWIR		TRUE	0.063978	0	1	1	2	cRAP078	Lysozyme C OS	cRAP0	0	1046	164414		46.85		164414	176477
FALSE	High	GTGASGSFKLNK		FALSE	0.083177	0	4	5	1	P16401; P	Histone H1.5 O	P1640	1	1167						
FALSE	High	GTGASGSFK	2xLabel:13C(6)	FALSE	0.062355	0	4	5	1	P16401; P	Histone H1.5 O	P1640	1	1179	29971	20523	86.29	70.81	29971	82751
FALSE	High	GYSLGNWV(	1xCarbamidom	TRUE	0.055717	0	1	1	2	cRAP078	Lysozyme C OS	cRAP0	0	1326	48968		45.11		48968	72932

FALSE	High	HAVSEGTKAVTK	FALSE	0.003004	0	2	15	5	Q16778; C	Histone H2B ty	Q1677	1	1228						
FALSE	High	HAVSEGTKA 2xLabel:13C(6)	FALSE	0.005704	0	2	15	2	Q16778; C	Histone H2B ty	Q1677	1	1240	885742	211587	65.79	50.53	885742	1843801
FALSE	High	HAVSEGTKAVTKYTSSK	FALSE	0.000642	0	2	12	13	Q16778; C	Histone H2B ty	Q1677	2	1794						
FALSE	High	HAVSEGTKA 3xLabel:13C(6)	FALSE	0.001581	0	2	12	5	Q16778; C	Histone H2B ty	Q1677	2	1812	481919	168786	87.63	65.39	481919	1487311
FALSE	High	HFNAPSHIR	FALSE	0.052073	0	1	1	1	P61254	60S ribosomal	P6125	0	1079	25730	3716	28.02	43.01	25730	36803
FALSE	High	HGGGGGGFGGGGFGSR	TRUE	0.000153	0	1	1	1	P35908	Keratin, type II	P3590	0	1321	96535		46.57		96535	152190
FALSE	High	HGLDNYRGY 1xCarbamidom	TRUE	0.030955	0	1	1	2	cRAP078	Lysozyme C OS	cRAP0	1	2181	96497		46.92		96497	100397
FALSE	High	HGSSSGSSSHYGQHGSGSR	FALSE	0.021469	0	1	1	1	Q86YZ3	Hornerin OS=H	Q86YZ	0	1859	13039		79.16		13039	40302
FALSE	High	HGSSSGSSSRYGQHGSGSR	FALSE	0.061165	0	1	1	1	Q86YZ3	Hornerin OS=H	Q86YZ	1	1878	34149		12.64		28548	34149
FALSE	High	HIQKEDVPSER	TRUE	0.021123	0	1	1	1	cRAP013	Alpha-S1-casei	cRAP0	1	1338	44798		117.77		44798	215258
FALSE	High	HIQKEDVPSERYLGYLEQLLR	TRUE	0.103889	0	1	1	1	cRAP013	Alpha-S1-casei	cRAP0	2	2586						
FALSE	High	HLQLAIRNDEELNK	FALSE	0.18926	0	2	12	1	Q16777; C	Histone H2A ty	Q1677	1	1693	21207	11584	92.94		21207	73765
FALSE	High	HLQLAIRNDEELNKLLGK	FALSE	0.124331	0	1	7	3	Q16777	Histone H2A ty	Q1677	2	2104	207668	18197	118.37	129.22	61860	697159
FALSE	High	HLQLAIRNDEELNKLLGR	FALSE	0.005292	0	1	3	3	Q93077	Histone H2A ty	Q7L7L	2	2132	43796	6625	143.36	124.95	43796	485860
FALSE	High	YRPGTVALREIR	FALSE	0.112779	0	1	5	1	P84243	Histone H3.3 O	P8424	1	1431	112468	10540	40.39	55.76	112468	193060
FALSE	High	YSQGNVSAVGVTYDGHTALT	TRUE	0.000325	0	1	1	6	cRAP117	000000|rLys-C	cRAP1	0	2196	1920979		41.65		1920979	2432481

Abundance F	Abundanc	Abundanc	Abundanc	Precur	Quan I	Quan (	Found	Found	Found	Found	Found	Found	Confid	Perc	Percolator	XCorr	Top Apex RT in min
23168				1		Light	Peak F	High	Peak F	Not Fc	Not Fc	Not Fc	High	0	3.13E-06	7.29	67.8
163748				123		Light	High	High	Peak F	Not Fc	Not Fc	Not Fc	High	0	0.00028	6.09	139
				124	NoQua	Light	Not Fc	High	Not Fc	Not Fc	Not Fc	Not Fc	High	0	0.02643	4.26	
84944	37923	104363	26050	125	NotUn	Light	High	High	Peak F	Peak F	Peak F	Peak F	High	0	0.06103	2.52	39.1
3468				126		Light	Peak F	High	Peak F	Not Fc	Not Fc	Not Fc	High	0	0.0424	2.57	139
19315187				127		Light	High	High	Peak F	Not Fc	Not Fc	Not Fc	High	0	0.02148	2.61	122
1121212				128		Light	High	High	Peak F	Not Fc	Not Fc	Not Fc	High	0	0.008106	4.03	102
157247				129		Light	High	High	Peak F	Not Fc	Not Fc	Not Fc	High	0	0.02475	3.27	122
21055	12398	23198		130	NotUn	Light	Peak F	High	Peak F	Peak F	Peak F	Not Fc	High	0	0.04304	3.41	76.6
355040				131		Light	High	High	Peak F	Not Fc	Not Fc	Not Fc	High	0	0.001513	3.9	57.3
34292				132		Light	High	High	Peak F	Not Fc	Not Fc	Not Fc	High	0	0.01574	3.22	38.8
62438				133		Light	High	High	Peak F	Not Fc	Not Fc	Not Fc	High	0	0.01875	2.61	59.5
				122		Light	Peak F	High	Not Fc	Not Fc	Not Fc	Not Fc	High	0	0.01459	3.7	129
2556	3242	15560		134		Light	High	High	Peak F	Peak F	Peak F	Not Fc	High	0	0.000621	4.46	129
15344767				136		Light	High	High	Peak F	Not Fc	Not Fc	Not Fc	High	0	4.98E-05	4.69	131
57003	38401	56439	19169	137		Heavy	Peak F	Peak F	Peak F	Peak F	High	Peak F	High	0	0.1199	2.81	54
225447	100357	134489	66693	138		Light	High	High	Peak F	Peak F	Peak F	Peak F	High	0	0.03859	3.25	108
59008	24640	25794	14745	139		Light	High	High	Peak F	Peak F	Peak F	Peak F	High	0	0.01875	3.06	92.2
		55908		140		Heavy	Not Fc	Peak F	Not Fc	Not Fc	High	Not Fc	High	0	0.1559	2.71	197
302365				141		Light	Peak F	High	Peak F	Not Fc	Not Fc	Not Fc	High	0	4.05E-05	5.92	143
27581				142		Light	High	High	Peak F	Not Fc	Not Fc	Not Fc	High	0	0.004578	3.3	48.7
				143	NoQua	Light	Not Fc	High	Not Fc	Not Fc	Not Fc	Not Fc	High	0	0.01356	4.25	
9400				144		Light	Peak F	High	Peak F	Not Fc	Not Fc	Not Fc	High	0	0.02266	4.12	122
19373				145		Light	Peak F	High	Peak F	Not Fc	Not Fc	Not Fc	High	0	3.09E-05	5.64	56.1
124955				146		Light	High	High	Peak F	Not Fc	Not Fc	Not Fc	High	0	0.0001	4.68	56.3
548263				135		Light	High	High	Peak F	Not Fc	Not Fc	Not Fc	High	0	0.003503	2.94	139
10906		21348		147	NotUn	Light	Peak F	High	Peak F	Not Fc	Peak F	Not Fc	High	0	0.03315	3.16	102
				121		Light	Not Fc	High	Not Fc	Not Fc	Not Fc	Not Fc	High	0	0.108	2.88	118
				119	NoQua	Light	Not Fc	High	Not Fc	Not Fc	Not Fc	Not Fc	High	0	0.1382	2.61	
624786				98		Light	High	High	Peak F	Not Fc	Not Fc	Not Fc	High	0	0.02013	4.27	186
27339				99		Light	High	High	Peak F	Not Fc	Not Fc	Not Fc	High	0	0.03167	4.07	160
79432	11782	18234	9604	100		Light	Peak F	High	Peak F	Peak F	Peak F	Peak F	High	0	0.1803	1.77	40.2
3362				101		Light	Peak F	High	Peak F	Not Fc	Not Fc	Not Fc	High	0	0.06747	1.84	43.6
17022		2595		102		Light	High	High	Peak F	Not Fc	Peak F	Not Fc	High	0	0.152	2.13	43.6
231603	13043	43552	16336	103		Light	High	High	Peak F	Peak F	Peak F	Peak F	High	0	0.01109	3.74	114
				104	NoQua	Light	Not Fc	High	Not Fc	Not Fc	Not Fc	Not Fc	High	0	0.09213	3.13	
4391				105		Light	Not Fc	High	Peak F	Not Fc	Not Fc	Not Fc	High	0	0.05073	3.07	201
1593014				106		Light	High	High	Peak F	Not Fc	Not Fc	Not Fc	High	0	0.08015	2.41	71.4
		20367		107		Heavy	Peak F	Peak F	Not Fc	Not Fc	High	Not Fc	High	0	0.03166	3.14	159
				108		Heavy	Peak F	Peak F	Not Fc	Not Fc	High	Not Fc	High	0	0.08304	2.94	208

3536				120		Light	Peak F	High	Peak F	Not Fc	Not Fc	Not Fc	High	0	0.01065	3.17	123
					Indistinguisha		Not Fc	Not Fc	Not Fc	Not Fc	Not Fc	Not Fc	High	0	0.04301	3.19	131
	8558	28287	4309	110		Light	Not Fc	High	Not Fc	Peak F	Peak F	Peak F	High	0	0.1249	2.84	182
				110	Redun	Heavy	Not Fc	Peak F	Not Fc	Peak F	High	Peak F	High	0	0.02033	4.11	182
8787	3589	5948		111		Light	Peak F	High	Peak F	Peak F	Peak F	Not Fc	High	0	0.1337	2.25	123
				112	Redun	Light	Peak F	High	Peak F	Not Fc	Peak F	Not Fc	High	0	0.03466	3.2	172
3994		29166		112		Heavy	Peak F	Peak F	Peak F	Not Fc	High	Not Fc	High	0	0.002284	4	172
10457346		54587	13640	113		Light	High	High	Peak F	Not Fc	Peak F	Peak F	High	0	1.32E-05	6.33	167
230886		44180		114		Light	High	High	Peak F	Not Fc	Peak F	Not Fc	High	0	2.57E-05	7.23	157
12082		12267	9557	115		Light	Peak F	High	Peak F	Not Fc	Peak F	Peak F	High	0	0.1206	2.71	120
991696				116		Light	High	High	Peak F	Not Fc	Not Fc	Not Fc	High	0	7.71E-05	5.2	190
		34387		117		Heavy	Not Fc	Peak F	Not Fc	Not Fc	High	Not Fc	High	0	0.08393	2.97	168
628974	3519	7756	3636	118		Light	High	High	Peak F	Peak F	Peak F	Peak F	High	0	0.000577	5.4	181
				109	NoQua	Light	Not Fc	High	Not Fc	Not Fc	Not Fc	Not Fc	High	0	0.1125	2.92	
210242				97		Light	High	High	Peak F	Not Fc	Not Fc	Not Fc	High	0	0.03048	3.6	161
17588	29407	58444	13639	148	NotUn	Light	Peak F	High	Peak F	Peak F	Peak F	Peak F	High	0	0.009329	2.91	76.7
				150		Light	Peak F	High	Not Fc	Not Fc	Not Fc	Not Fc	High	0	0.006451	4.3	199
13783602				177		Light	Peak F	High	Peak F	Not Fc	Not Fc	Not Fc	High	0	0.04979	1.78	88.7
					Indistinguisha		Not Fc	Not Fc	Not Fc	Not Fc	Not Fc	Not Fc	High	0	0.04256	3.55	218
568199				178		Light	High	High	Peak F	Not Fc	Not Fc	Not Fc	High	0	6.65E-05	6.73	204
				179	NoQua	Light	Not Fc	High	Not Fc	Not Fc	Not Fc	Not Fc	High	0	0.05332	5.54	
29964	56631	75997	37600	180		Light	Peak F	High	Peak F	Peak F	Peak F	Peak F	High	0	0.04566	3.23	110
19336	4162	25009	6471	181		Light	Peak F	High	Peak F	Peak F	Peak F	Peak F	High	0	0.07128	2.88	141
73603	55063	76188	49442	182		Light	High	High	Peak F	Peak F	Peak F	Peak F	High	0	0.03848	3.57	191
69665	1635	3428	987	183		Light	Peak F	High	Peak F	Peak F	Peak F	Peak F	High	0	0.05088	2.9	58.9
52462	25057	42263	15723	184	NotUn	Light	High	High	Peak F	Peak F	Peak F	Peak F	High	0	0.04275	2.46	55.1
				185		Light	Peak F	High	Not Fc	Not Fc	Not Fc	Not Fc	High	0	0.1583	2.64	109
323708	127857	543409		186	NotUn	Light	High	High	Peak F	Peak F	Peak F	Not Fc	High	0	5.55E-05	4.58	207
21820	7432	15908		176		Light	Peak F	High	Peak F	Peak F	Peak F	Not Fc	High	0	0.01094	3.45	131
				186	Redun	Heavy	Peak F	Peak F	Peak F	High	High	Not Fc	High	0	0.1554	2.54	207
4078				188		Light	Peak F	High	Peak F	Not Fc	Not Fc	Not Fc	High	0	0.04484	2.48	157
91583	37362	79918	26061	189		Light	High	High	Peak F	Peak F	Peak F	Peak F	High	0	0.08271	2.48	123
				190		Light	Peak F	High	Not Fc	Not Fc	Not Fc	Not Fc	High	0	0.09064	2.25	88.7
72907				191		Light	Peak F	High	Peak F	Not Fc	Not Fc	Not Fc	High	0	0.03936	3.14	84.4
	49149	69316	15393	192		Heavy	Not Fc	Not Fc	Not Fc	High	High	Peak F	High	0	0.088	3.16	150
45422	23967	47511	20935	193		Light	High	High	Peak F	Peak F	Peak F	Peak F	High	0	0.04856	3.37	116
				193	Redun	Heavy	Peak F	Peak F	Peak F	Peak F	High	Peak F	High	0	0.138	3.04	116
246882		1500		194		Light	Peak F	High	Peak F	Not Fc	Peak F	Not Fc	High	0	0.02723	3.58	65
121464	1063	3229		195		Light	High	High	Peak F	Peak F	Peak F	Not Fc	High	0	0.1609	3.1	54.1
10337	24367	40152		196		Light	High	High	Peak F	Peak F	Peak F	Not Fc	High	0	0.02219	3.58	194
189252	35127	110507	31009	197		Light	Peak F	High	Peak F	Peak F	Peak F	Peak F	High	0	0.1131	2.12	66.2
		2674		187		Light	Peak F	High	Not Fc	Not Fc	Peak F	Not Fc	High	0	0.004162	2.68	146

		37742		149	NotUn	Heavy	Not Fc	Peak F	Not Fc	Not Fc	High	Not Fc	High	0	0.02762	4.39	177
				175		Light	Peak F	High	Not Fc	Not Fc	Not Fc	Not Fc	High	0	0.03207	1.8	98.9
24791	7653	24064	1862	173		Light	Peak F	High	Peak F	Peak F	Peak F	Peak F	High	0	0.07133	3.66	147
369769				151		Light	Peak F	High	Peak F	Not Fc	Not Fc	Not Fc	High	0	0.07522	1.97	88.6
552575				152		Light	High	High	Peak F	Not Fc	Not Fc	Not Fc	High	0	0.04151	2.07	85.4
23151				153		Light	Peak F	High	Peak F	Not Fc	Not Fc	Not Fc	High	0	0.01995	4.02	135
61459				154		Light	High	High	Peak F	Not Fc	Not Fc	Not Fc	High	0	0.009108	3.27	186
				155		Light	Peak F	High	Not Fc	Not Fc	Not Fc	Not Fc	High	0	0.00303	4.44	170
				156		Light	Not Fc	High	Not Fc	Not Fc	Not Fc	Not Fc	High	0	0.004504	4.57	164
39917	5587	13552	7174	157		Light	Peak F	High	Peak F	Peak F	Peak F	Peak F	High	0	0.06516	3.02	96.2
22980				158		Light	Peak F	High	Peak F	Not Fc	Not Fc	Not Fc	High	0	0.000146	4.84	69.3
				159	NoQua	Light	Not Fc	High	Not Fc	Not Fc	Not Fc	Not Fc	High	0	0.1119	2.22	
19980				160		Light	Peak F	High	Peak F	Not Fc	Not Fc	Not Fc	High	0	0.07319	2.65	74.4
466167	87946	242246	41164	161		Light	Peak F	High	Peak F	Peak F	Peak F	Peak F	High	0	0.1209	2.25	94.3
9186	3841	8662		174		Light	Peak F	High	Peak F	Peak F	Peak F	Not Fc	High	0	0.09912	3.19	154
235097	126464	227966	96848	162		Light	High	High	Peak F	Peak F	Peak F	Peak F	High	0	0.009689	3.12	82.1
83690				163		Light	High	High	Peak F	Not Fc	Not Fc	Not Fc	High	0	0.06342	3.1	80.7
12896	7964	10126		164		Light	High	High	Peak F	Peak F	Peak F	Not Fc	High	0	0.000942	3.51	164
14557				165		Light	Peak F	High	Peak F	Not Fc	Not Fc	Not Fc	High	0	0.05998	2.42	77.1
12856				166		Light	Peak F	High	Peak F	Not Fc	Not Fc	Not Fc	High	0	0.1491	3.35	133
				167	NoQua	Light	Not Fc	High	Not Fc	Not Fc	Not Fc	Not Fc	High	0	0.1289	2.3	
		7440		168		Light	Not Fc	High	Not Fc	Not Fc	Peak F	Not Fc	High	0	0.1661	2.29	171
136694				169		Light	High	High	Peak F	Not Fc	Not Fc	Not Fc	High	0	0.001429	4.84	151
509669				170		Light	High	High	Peak F	Not Fc	Not Fc	Not Fc	High	0	0.02567	2.39	142
179152				171		Light	High	High	Peak F	Not Fc	Not Fc	Not Fc	High	0	0.006794	3.46	134
					NoQuanValu		Not Fc	Not Fc	Not Fc	Not Fc	Not Fc	Not Fc	High	0	0.1213	2.6	
24291	8172	43122	8404	172		Heavy	Peak F	Peak F	Peak F	Peak F	High	Peak F	High	0	0.1803	3.25	173
				162	Redun	Heavy	Peak F	Peak F	Peak F	Peak F	High	Peak F	High	0	0.02771	3.46	82.1
2062590				96		Light	High	High	Peak F	Not Fc	Not Fc	Not Fc	High	0	0.000654	5.24	169
63723	15202	30280	10145	95	NotUn	Light	High	High	Peak F	Peak F	Peak F	Peak F	High	0	0.04962	3.22	36.9
					Inconsistently		Not Fc	Not Fc	Not Fc	Not Fc	Not Fc	Not Fc	High	0	0.1597	2.58	124
416384				23		Light	High	High	Peak F	Not Fc	Not Fc	Not Fc	High	0	6.81E-05	7.98	200
				24	NoQua	Light	Not Fc	High	Not Fc	Not Fc	Not Fc	Not Fc	High	0	0.08798	4.1	
52641				25		Light	Peak F	High	Peak F	Not Fc	Not Fc	Not Fc	High	0	0.0592	2.53	61.1
104562	24628	26939	18137	26		Light	High	High	Peak F	Peak F	Peak F	Peak F	High	0	0.02262	3.94	68.6
122036				27		Light	High	High	Peak F	Not Fc	Not Fc	Not Fc	High	0	0.0807	3.25	120
33988				28		Light	Peak F	High	Peak F	Not Fc	Not Fc	Not Fc	High	0	0.04354	2.74	127
93596				29		Light	High	High	Peak F	Not Fc	Not Fc	Not Fc	High	0	0.01214	3.57	116
191385				30		Light	High	High	Peak F	Not Fc	Not Fc	Not Fc	High	0	2.72E-06	7.62	122
30522				31		Light	High	High	Peak F	Not Fc	Not Fc	Not Fc	High	0	0.000547	5.6	148
					Indistinguisha		Not Fc	Not Fc	Not Fc	Not Fc	Not Fc	Not Fc	High	0	0.1189	2.62	79.9
168601	51605	131744	33410	32		Light	High	High	Peak F	Peak F	Peak F	Peak F	High	0	0.01521	3.71	90.3

5192565	1424			22		Light	High	High	Peak F	Peak F	Not Fc	Not Fc	High	0	0.001971	4.02	60.9
43830	4769	2594		33		Light	High	High	Peak F	Peak F	Peak F	Not Fc	High	0	0.109	2.6	157
				34		Light	Not Fc	High	Not Fc	Not Fc	Not Fc	Not Fc	High	0	0.1409	2.3	193
				36	NoQua	Light	Not Fc	High	Not Fc	Not Fc	Not Fc	Not Fc	High	0	0.0155	4.54	
448355	167250	732087	171849	37		Light	High	High	Peak F	Peak F	Peak F	Peak F	High	0	0.02255	3.01	171
				37	Redun	Heavy	Peak F	Peak F	Peak F	Peak F	High	Peak F	High	0	0.008486	3.92	171
6111	4103	33086		38		Light	Peak F	High	Peak F	Peak F	Peak F	Not Fc	High	0	0.1342	2.75	162
				38	Redun	Heavy	Peak F	Peak F	Peak F	Peak F	High	Not Fc	High	0	0.1315	3.29	162
59774				39		Light	High	High	Peak F	Not Fc	Not Fc	Not Fc	High	0	0.004956	4.05	82.5
3379				40		Light	Peak F	High	Peak F	Not Fc	Not Fc	Not Fc	High	0	0.1162	2.45	76.5
189930				41		Light	Peak F	High	Peak F	Not Fc	Not Fc	Not Fc	High	0	0.1148	3.14	208
96593				42		Light	Peak F	High	Peak F	Not Fc	Not Fc	Not Fc	High	0	0.00293	3.6	180
				43		Light	Not Fc	High	Not Fc	Not Fc	Not Fc	Not Fc	High	0	0.002157	5.03	175
				35	NoQua	Light	Not Fc	High	Not Fc	Not Fc	Not Fc	Not Fc	High	0	0.07597	3.45	
117270				44		Light	High	High	Peak F	Not Fc	Not Fc	Not Fc	High	0	0.000369	5.17	110
37378	38421	108148	29927	21	NotUn	Light	High	High	Peak F	Peak F	Peak F	Peak F	High	0	0.03236	3.33	11.5
	30348	49691	18072	19		Heavy	Not Fc	Not Fc	Not Fc	Peak F	High	Peak F	High	0	0.01237	3.06	165
87795	43474	98320	27340	2		Light	Peak F	High	Peak F	Peak F	Peak F	Peak F	High	0	0.01249	2.91	62.7
				2	Redun	Heavy	Peak F	Peak F	Peak F	High	High	Peak F	High	0	0.01206	3.54	62.7
4118	4484	8301		3		Light	Peak F	High	Peak F	Peak F	Peak F	Not Fc	High	0	0.06515	3.76	139
1309885	508858	1171630	319268	4	NotUn	Light	Peak F	High	Peak F	Peak F	Peak F	Peak F	High	0	0.009526	2.91	121
				5	Redun	Light	Peak F	High	Peak F	Peak F	Peak F	Peak F	High	0	0.06864	2.04	93.8
91618	39685	61483	24164	5	NotUn	Heavy	Peak F	Peak F	Peak F	Peak F	High	Peak F	High	0	0.127	2.65	93.8
7244246				6		Light	High	High	Peak F	Not Fc	Not Fc	Not Fc	High	0	8.24E-06	6.76	113
7345876				7		Light	High	High	Peak F	Not Fc	Not Fc	Not Fc	High	0	2.02E-06	7.78	138
2814656				8		Light	Peak F	High	Peak F	Not Fc	Not Fc	Not Fc	High	0	0.1528	2.44	115
11393	3658	8527		9		Light	Not Fc	High	Peak F	Peak F	Peak F	Not Fc	High	0	0.003782	3.55	11.7
9796	4392	12386	3401	10		Light	Peak F	High	Peak F	Peak F	Peak F	Peak F	High	0	0.03959	4.52	11.4
	37469	94931	21558	20		Heavy	Peak F	Peak F	Not Fc	High	High	Peak F	High	0	0.01676	3.05	189
28815	22797	99712	17269	11	NotUn	Light	Peak F	High	Peak F	Peak F	Peak F	Peak F	High	0	0.004668	3.48	87.1
				12	Redun	Heavy	Peak F	Peak F	Peak F	Peak F	High	Peak F	High	0	0.09676	2.12	72.2
16837				13		Light	High	High	Peak F	Not Fc	Not Fc	Not Fc	High	0	0.008592	3.56	169
63941		66805		14	NotUn	Light	High	High	Peak F	Not Fc	Peak F	Not Fc	High	0	0.000834	4.97	217
				14	Redun	Heavy	Peak F	Peak F	Peak F	Not Fc	High	Not Fc	High	0	0.03339	4.02	217
355563	98456	684411	72713	15	NotUn	Light	High	High	Peak F	Peak F	Peak F	Peak F	High	0	2.77E-05	5.91	205
				15	Redun	Heavy	Peak F	Peak F	Peak F	Peak F	High	Peak F	High	0	0.03449	4.5	204
72578	5611	147817	19662	16	NotUn	Light	Peak F	High	Peak F	Peak F	Peak F	Peak F	High	0	0.003479	5.31	178
				16	Redun	Heavy	Peak F	Peak F	Peak F	Peak F	High	Peak F	High	0	0.005035	4.94	177
6428				17		Light	Peak F	High	Peak F	Not Fc	Not Fc	Not Fc	High	0	0.1102	2.31	81.9
				18	Redun	Light	Peak F	High	Peak F	Peak F	Peak F	Peak F	High	0	0.04168	3.31	116
73082	37835	123708	30908	18	NotUn	Heavy	Peak F	Peak F	Peak F	Peak F	High	Peak F	High	0	0.07328	2.63	116
21454	27957	70600	15642	12	NotUn	Light	High	High	Peak F	Peak F	Peak F	Peak F	High	0	0.1528	2.13	72.2

		7357		45		Light	Peak F	High	Not Fc	Not Fc	Peak F	Not Fc	High	0	0.1793	2.86	177
		9620		46		Light	Peak F	High	Not Fc	Not Fc	Peak F	Not Fc	High	0	0.1047	2.69	131
11503				47		Light	High	High	Peak F	Not Fc	Not Fc	Not Fc	High	0	0.003015	4.33	143
				74	Redun	Light	High	High	Not Fc	Peak F	Not Fc	Not Fc	High	0	0.001804	4.65	101
	31068			74		Heavy	Peak F	Peak F	Not Fc	High	High	Not Fc	High	0	0.000273	4.62	101
16905		3044		75		Light	Peak F	High	Peak F	Not Fc	Peak F	Not Fc	High	0	0.05256	3.05	144
10898	33993	57485	12433	76		Light	Peak F	High	Peak F	Peak F	Peak F	Peak F	High	0	0.1688	2.23	65.7
3225		2288		77		Light	Peak F	High	Peak F	Not Fc	Peak F	Not Fc	High	0	0.007027	4.31	148
30845	21518	60623		78		Light	High	High	Peak F	Peak F	Peak F	Not Fc	High	0	0.01981	2.98	134
13684	4137	7227		79		Light	Peak F	High	Peak F	Peak F	Peak F	Not Fc	High	0	0.07466	2.68	114
	72094	96507		80		Light	Peak F	High	Not Fc	Peak F	Peak F	Not Fc	High	0	0.1264	2.61	12.9
61187				81		Light	High	High	Peak F	Not Fc	Not Fc	Not Fc	High	0	0.007829	3.88	117
1035574				82		Light	High	High	Peak F	Not Fc	Not Fc	Not Fc	High	0	0.000148	6.3	102
	146172	117227	40833	83		Light	High	High	Not Fc	Peak F	Peak F	Peak F	High	0	0.001226	4.66	209
10427	45366	128253	26463	73		Heavy	Peak F	Peak F	Peak F	High	High	Peak F	High	0	0.01267	3.93	94.6
3529360				84		Light	High	High	Peak F	Not Fc	Not Fc	Not Fc	High	0	3.74E-06	7.41	150
36988	13108	30984	9877	86		Heavy	Peak F	Peak F	Peak F	Peak F	High	Peak F	High	0	0.003285	3.45	150
53670				87		Light	Peak F	High	Peak F	Not Fc	Not Fc	Not Fc	High	0	0.001114	5.17	119
190017				88		Light	Peak F	High	Peak F	Not Fc	Not Fc	Not Fc	High	0	0.1003	2.84	62
	5970	33332	5783	89		Light	Peak F	High	Not Fc	Peak F	Peak F	Peak F	High	0	0.05081	3.22	115
19170	26261	32767	18614	90		Heavy	Peak F	Peak F	Peak F	Peak F	High	Peak F	High	0	0.0442	2.68	116
				91	Redun	Light	High	High	Peak F	Peak F	Peak F	Peak F	High	0	0.0699	2.33	140
4339	85209	134515	18563	91		Heavy	Peak F	Peak F	Peak F	High	High	Peak F	High	0	0.03526	2.8	140
				92	Redun	Light	Not Fc	High	Not Fc	Not Fc	Peak F	Not Fc	High	0	0.004052	3.98	197
		99799		92		Heavy	Not Fc	Peak F	Not Fc	Not Fc	High	Not Fc	High	0	0.002244	3.95	197
				93		Light	Not Fc	High	Not Fc	Not Fc	Not Fc	Not Fc	High	0	0.06377	2.58	147
1292379	612357	1656134	404076	94	NotUn	Light	Peak F	High	Peak F	Peak F	Peak F	Peak F	High	0	0.1724	2.29	136
				85		Light	High	High	Not Fc	Not Fc	Not Fc	Not Fc	High	0	0.0293	3.91	164
		6783		72	NotUn	Light	Peak F	High	Not Fc	Not Fc	Peak F	Not Fc	High	0	0.06119	2.71	134
51985		3086	1660	71		Light	Peak F	High	Peak F	Not Fc	Peak F	Peak F	High	0	0.01039	4.01	144
13337				70		Light	Peak F	High	Peak F	Not Fc	Not Fc	Not Fc	High	0	0.007053	3.89	121
235149				48		Light	High	High	Peak F	Not Fc	Not Fc	Not Fc	High	0	0.000791	5.08	104
1068967				49		Light	High	High	Peak F	Not Fc	Not Fc	Not Fc	High	0	0.000529	5.31	186
57893				50		Light	High	High	Peak F	Not Fc	Not Fc	Not Fc	High	0	0.000201	5.98	48
1475				51		Light	High	High	Peak F	Not Fc	Not Fc	Not Fc	High	0	0.003757	4.38	44.2
				52	NoQua	Heavy	Not Fc	Not Fc	Not Fc	Not Fc	High	Not Fc	High	0	0.07092	2.77	
14203				53		Light	Peak F	High	Peak F	Not Fc	Not Fc	Not Fc	High	0	0.007259	4.18	66.1
92047	173055	273536	164980	54		Light	High	High	Peak F	Peak F	Peak F	Peak F	High	0	0.00011	5.79	138
61978				55		Light	High	High	Peak F	Not Fc	Not Fc	Not Fc	High	0	0.043	3.4	122
				56	Redun	Light	Peak F	High	Peak F	Peak F	Peak F	Peak F	High	0	0.05763	3.13	54.9
13314	20523	49264	12352	56	NotUn	Heavy	Peak F	Peak F	Peak F	Peak F	High	Peak F	High	0	0.04166	3.46	54.9
27911				57		Light	High	High	Peak F	Not Fc	Not Fc	Not Fc	High	0	0.0368	3.04	147

				58	Redun	Light	High	High	Peak F	Peak F	Peak F	Peak F	High	0	0.001389	4.46	23.1
474476	211587	341776	116909	58	NotUn	Heavy	Peak F	Peak F	Peak F	High	High	Peak F	High	0	0.002851	4.49	23.1
				59	Redun	Light	High	High	Peak F	Peak F	Peak F	Peak F	High	0	0.000246	4.12	53.2
264281	168786	414969	128216	59	NotUn	Heavy	Peak F	Peak F	Peak F	High	High	Peak F	High	0	0.000678	5.02	53.2
21606	3716	6437	2864	60		Light	Peak F	High	Peak F	Peak F	Peak F	Peak F	High	0	0.03406	3.2	46.5
57585				61		Light	Peak F	High	Peak F	Not Fc	Not Fc	Not Fc	High	0	4.95E-05	5.78	63.4
35564				62		Light	High	High	Peak F	Not Fc	Not Fc	Not Fc	High	0	0.01903	4	141
9954				63		Light	Peak F	High	Peak F	Not Fc	Not Fc	Not Fc	High	0	0.0126	4.06	11.5
36745				64		Light	Peak F	High	Peak F	Not Fc	Not Fc	Not Fc	High	0	0.04088	2.56	11.8
15100				65		Light	Peak F	High	Peak F	Not Fc	Not Fc	Not Fc	High	0	0.01237	3.5	36.5
				66	NoQua	Light	Not Fc	High	Not Fc	Not Fc	Not Fc	Not Fc	High	0	0.07398	3.61	
12273		11584		67	NotUn	Light	Peak F	High	Peak F	Not Fc	Peak F	Not Fc	High	0	0.1457	2.21	91
	18197	126624	7893	68		Light	High	High	Not Fc	Peak F	Peak F	Peak F	High	0	0.09089	2.59	141
19619	6625	38041	2228	69		Light	Peak F	High	Peak F	Peak F	Peak F	Peak F	High	0	0.002624	3.49	145
91822	6947	15990		198		Light	Peak F	High	Peak F	Peak F	Peak F	Not Fc	High	0	0.08131	2.87	85.2
974569				199		Light	High	High	Peak F	Not Fc	Not Fc	Not Fc	High	0	0.000115	6.33	116

Supplemental Table 4.4. Raw Proteome discoverer output from the three replicates of Condition D

Checked	Confid	Sequence	Modifications	Contamii	Qvality PEP	Qvality q	Num	Num	Num	Master Pr	Master Protei	Protein A	Num	Theo MH	Abundan	Abundan	Abundan	Abundan	Abundan	Abundan
FALSE	High	AAAAAAAAAPAAAATAPTTAA		FALSE	0.006123	0.0064	1	1	1	P37108	Signal recogni	P37108	0	2368.2						
FALSE	High	RTPPAGVFYQGWSATPIANGSI		TRUE	9.45E-08	0.0064	1	1	3	cRAP117	000000|rLys-(	cRAP117	1	3239.6	1E+06		54.52		2E+06	1E+06
FALSE	High	RTPPAGVFYQGWSATPIANGSI		TRUE	0.000184	0.0064	1	1	3	cRAP117	000000|rLys-(	cRAP117	2	3610.8	2E+07		27.8		2E+07	3E+07
FALSE	High	RVTIMPK		FALSE	0.049925	0.0064	1	5	1	P84243	Histone H3.3 (	P84243; (	1	844.51	810816	169458	55	47.13	387548	1E+06
FALSE	High	RVTIMPKDIQLAR		FALSE	1.89E-06	0.0064	1	5	1	P84243	Histone H3.3 (	P84243; (	2	1540.9	802681	187820	41.96	36.09	382290	964873
FALSE	High	RVTIMPKDIC	1xOxidation [M	FALSE	4.71E-05	0.0064	1	5	2	P84243	Histone H3.3 (	P84243; (	2	1556.9	443413	73971	85.66	85.61	125552	1E+06
FALSE	High	RYQKSTELLIR		FALSE	0.004882	0.0064	1	5	1	P84243	Histone H3.3 (	P84243; (	2	1406.8	34500	7351.6	50.11	75.92		49965
FALSE	High	RKPDTIEVQC	1xOxidation [M	FALSE	1.74E-05	0.0064	1	3	1	P35241	Radixin OS=H	P35241; I	1	1488.8	45160		59.33		106120	37998
FALSE	High	SCAAAGTECL	2xCarbamidom	TRUE	7.32E-14	0.0064	1	1	3	cRAP112	Trypsin OS=Su	cRAP112	0	1882.8	402786		97.7		14361	820060
FALSE	High	SGDAAIVDM	1xCarbamidom	FALSE	8.97E-05	0.0064	1	2	1	P68104	Elongation fac	P68104; (	0	2995.4						
FALSE	High	SGDAAIVDM	1xCarbamidom	FALSE	0.004989	0.0064	1	2	1	P68104	Elongation fac	P68104; (	0	3007.4	54704	21022	82.29	79.43	8423.6	95233
FALSE	High	SGYRSGGGFSSGSAGIINYQR		TRUE	6.10E-05	0.0064	1	1	1	P04264	Keratin, type I	P04264	1	2121	42531					
FALSE	High	SIQADGLVW	1xLabel:13C(6)	FALSE	0.000146	0.0064	1	1	1	P24534	Elongation fac	P24534	0	1353.7						
FALSE	High	SISISVAR		TRUE	0.000928	0.0064	1	1	1	P04264	Keratin, type I	P04264	0	832.49	1E+06	7326.4	25.97		776951	1E+06
FALSE	High	SLVGLGGTK		TRUE	0.138859	0.0064	1	1	1	P35908	Keratin, type I	P35908	0	831.49	600521					
FALSE	High	SCQAQPTTM	1xCarbamidom	TRUE	1.06E-06	0.0064	1	1	1	cRAP016	Kappa-casein	cRAP016	0	1250.6	66648		22.1		79977	50911
FALSE	High	RKPDTIEVQQMK		FALSE	2.72E-05	0.0064	1	3	1	P35241	Radixin OS=H	P35241; I	1	1472.8	645745		28.2		826006	462377
FALSE	High	RKATGPPVSE	3xLabel:13C(6)	FALSE	0.017192	0.0064	1	1	1	P16401	Histone H1.5 (	P16401	2	1514.9	37529	59763	66.26	84.13	15643	69523
FALSE	High	RGDLPFVVPR		FALSE	0.000381	0.0064	1	1	2	P35579	Myosin-9 OS=	P35579	1	1155.7	239921		38.13		237130	239921
FALSE	High	LSSPATLNSR		TRUE	0.010985	0.0064	1	1	1	cRAP112	Trypsin OS=Su	cRAP112	0	1045.6	4E+06		33.86		2E+06	4E+06
FALSE	High	ITIADCGQLE	1xCarbamidom	TRUE	0.051914	0.0064	1	1	1	P62937	Peptidyl-proly	P62937	0	1119.5						
FALSE	High	IVGGYTCAAN	3xCarbamidom	TRUE	4.34E-05	0.0064	1	1	1	cRAP112	Trypsin OS=Su	cRAP112	0	4660.2	55311		115.86			175490
FALSE	High	LYAEERYPILP	1xCarbamidom	TRUE	0.000264	0.0064	1	1	1	cRAP086	Ovalbumin OS	cRAP086	2	2845.5	112792		80.34		27571	220342
FALSE	High	MDKNELVQK	1xAcetyl [N-Ter	FALSE	0.000261	0.0064	1	1	1	P63104	14-3-3 protein	P63104	2	1345.7	111364	42799	60.99	67.84	27002	111364
FALSE	High	MFLRGDSVIV	3xLabel:13C(6)	FALSE	0.021112	0.0064	1	1	1	P62316	Small nuclear	P62316	2	2216.3	64614	44992		77.06		
FALSE	High	MGCSSSALNI	1xCarbamidom	FALSE	0.114434	0.0064	1	1	1	Q6P6B1	Glutamate-ric	Q6P6B1	1	1649.7						
FALSE	High	MVFTSSADG	1xCarbamidom	TRUE	3.89E-17	0.0064	1	1	11	cRAP117	000000|rLys-(	cRAP117	0	2691.2	2E+07	29473	45.09	73.54	1E+07	2E+07
FALSE	High	MVFTSSADG	1xCarbamidom	TRUE	3.84E-14	0.0064	1	1	11	cRAP117	000000|rLys-(	cRAP117	0	2707.2	369829	63224	86.15	92.92	68021	369829
FALSE	High	NDYFSDFSGVYSQISR		TRUE	1.64E-10	0.0064	1	1	1	cRAP117	000000|rLys-(	cRAP117	0	1884.8	414028		25.27		340877	558256
FALSE	High	NLCNIPCSALI	3xCarbamidom	TRUE	3.52E-09	0.0064	1	1	1	cRAP078	Lysozyme C O	cRAP078	0	2508.2	343259		60.11		97953	441091
FALSE	High	NLQNVDMKIGV		FALSE	0.008616	0.0064	1	1	1	P16070	CD44 antigen	P16070	1	1230.7	50324		53.7		97788	50324
FALSE	High	NTDGSTDYGILQINSR		TRUE	4.05E-06	0.0064	1	1	1	cRAP078	Lysozyme C O	cRAP078	0	1753.8	41488		71.09		21067	92340
FALSE	High	QVEMLNK		FALSE	0.062429	0.0064	1	1	1	Q5U623	Activating tra	Q5U623	0	861.45	51638		77.77		3967.8	51638
FALSE	High	RDTLLLELK		TRUE	7.29E-06	0.0064	1	1	1	cRAP117	000000|rLys-(	cRAP117	1	1100.7	4E+06		0.92		4E+06	4E+06
FALSE	High	SLVNLGGSK		TRUE	0.037509	0.0064	1	1	1	P04264	Keratin, type I	P04264	0	874.5	2E+06		34.08		2E+06	2E+06
FALSE	High	SLYASSPGGVYATR		FALSE	3.06E-08	0.0064	1	1	2	P08670	Vimentin OS=	P08670	0	1428.7	164463		61.88			102872
FALSE	High	SPAQILQWQVLSNTVPAK		TRUE	2.37E-13	0.0064	1	1	2	cRAP016	Kappa-casein	cRAP016	0	1980.1	631638		42.09		347046	631638
FALSE	High	SPAQILQWQ	1xCarbamidom	TRUE	0.063035	0.0064	1	1	1	cRAP016	Kappa-casein	cRAP016	1	3211.6	74516		73.18		16697	74516
FALSE	High	TVSLGAGAKI	3xLabel:13C(6)	FALSE	2.33E-08	0.0064	1	1	1	P06748	Nucleophosm	P06748	2	3674						
FALSE	High	TVTAMDVVY	1xOxidation [M	FALSE	0.007665	0.0064	1	1	1	P62805	Histone H4 OS	P62805	0	1326.7	34389	6435.9	43.2	37.81	20331	50653

FALSE	High	VAPEEHPVLLTEAPLNPK		FALSE	0.000214	0.0064	1	2	2	P60709	Actin, cytopla	P60709;	0	1954.1	86415	6111.3	15.85	22.33	100284	72857
FALSE	High	VFLENVIR		FALSE	0.000352	0.0064	1	1	1	P62805	Histone H4 OS	P62805	0	989.58	12989	11860	107.58		9557.5	66504
FALSE	High	VLKQVHPDTGISSK		FALSE	0.001149	0.0064	2	15	2	O60814; Q	Histone H2B t	Q16778;	1	1508.8	153267	32517	54.01	53.67	123429	323803
FALSE	High	VSLAGACGV	1xCarbamidom	FALSE	2.96E-10	0.0064	1	1	1	P13647	Keratin, type I	P13647	0	1410.7	77931		50.04		86467	27306
FALSE	High	VTIAQGGVLPNIQAVLLPK		FALSE	2.63E-08	0.0064	1	10	1	Q16777	Histone H2A t	Q16777;	0	1931.2						
FALSE	High	VTIAQGGVLF	1xLabel:13C(6)	FALSE	0.033614	0.0064	1	10	1	Q16777	Histone H2A t	Q16777;	0	1937.2	48649	45094	134.88	28.45	7483.6	
FALSE	High	VTIMPK	1xLabel:13C(6)	FALSE	0.153498	0.0064	1	5	1	P84243	Histone H3.3 (	P84243;	0	694.43	368255	63757	66.06	61.63	66715	399275
FALSE	High	VTIMPKDIQLAR		FALSE	0.000645	0.0064	1	5	2	P84243	Histone H3.3 (	P84243;	1	1384.8	327986	68485	74.81	65.45	95474	610256
FALSE	High	VYQSLCPTSV	1xCarbamidom	FALSE	0.038261	0.0064	1	1	1	P14854	Cytochrome c	P14854	2	3170.5						
FALSE	High	WWCNDGR	1xCarbamidom	TRUE	0.008708	0.0064	1	1	1	cRAP078	Lysozyme C O	cRAP078	0	993.4	93515	21175	67.78	63.33	25024	142377
FALSE	High	WWCNDGRT	4xCarbamidom	TRUE	5.00E-05	0.0064	1	1	1	cRAP078	Lysozyme C O	cRAP078	2	3980.8	101383	18467	17.13			89766
FALSE	High	YADLTEDQLF	1xCarbamidom	FALSE	0.006189	0.0064	1	1	1	P18669	Phosphoglyce	P18669	1	2437.2		65622		49.18		
FALSE	High	YEKDIAAYRAK		FALSE	0.007665	0.0064	1	2	1	P26583	High mobility	P26583;	2	1327.7	280139		45.49		495810	208990
FALSE	High	TVSLGAGAKI	2xLabel:13C(6)	FALSE	0.010985	0.0064	1	1	1	P06748	Nucleophosm	P06748	1	2941.5	28538	45107	69.82	15.24	5866	28538
FALSE	High	ISGLIYEETR		FALSE	1.62E-06	0.0064	1	1	1	P62805	Histone H4 OS	P62805	0	1180.6	281704	5287.5	30.26	90.6	214824	281704
FALSE	High	TVETRDGQVINETSQHHDDLE		FALSE	1.62E-07	0.0064	1	1	1	P08670	Vimentin OS=	P08670	1	2423.1	28108		66.29		8040.7	28108
FALSE	High	TTAEPQHQSPPGISSEGFPWD(		FALSE	0.153498	0.0064	1	1	1	Q68DA7	Formin-1 OS=	Q68DA7	2	4839.3						
FALSE	High	SPAQILQWQ	1xCarbamidom	TRUE	1.19E-07	0.0064	1	1	1	cRAP016	Kappa-casein	cRAP016	1	3227.6	42978		94.3			19216
FALSE	High	SQRAGLQFPVGR		FALSE	0.077095	0.0064	1	2	1	Q71UI9	Histone H2A.\	Q71UI9;	1	1315.7	160048	24474	106.89	122.24	59686	429170
FALSE	High	SRGSGGLGG	1xCarbamidom	FALSE	2.98E-05	0.0064	1	3	1	P04259	Keratin, type I	P04259;	1	1667.8	74282		18.22		77658	74282
FALSE	High	SSLLEKGLDGAK		FALSE	0.129035	0.0064	1	1	1	P81605	Dermcidin OS	P81605	1	1217.7	503275		66.19		491479	1E+06
FALSE	High	SSLLEKGLDGAKK		FALSE	0.023143	0.0064	1	1	1	P81605	Dermcidin OS	P81605	2	1345.8	98817		53.69		43867	147776
FALSE	High	STELLIR		FALSE	0.00105	0.0064	1	5	3	P84243	Histone H3.3 (	P84243;	0	831.49	1E+06	79846	60.19	110.97	839521	3E+06
FALSE	High	STITSREIQTAVR		FALSE	0.000443	0.0064	1	13	2	O60814	Histone H2B t	Q16778;	1	1461.8	1E+06	387273	88.6	87.01	240609	3E+06
FALSE	High	STITSREIQTA	2xLabel:13C(6)	FALSE	5.00E-06	0.0064	1	13	1	O60814	Histone H2B t	Q16778;	1	1473.8						
FALSE	High	SYELPDGQVITIGNER		FALSE	2.72E-06	0.0064	1	10	1	P60709	Actin, cytopla	A5A3E0;	0	1790.9						
FALSE	High	THNLEPYFESFINNLR		TRUE	0.001149	0.0064	1	1	1	P04264	Keratin, type I	P04264	0	1994	16235		70.88		16235	30093
FALSE	High	TIGISVDPR		FALSE	0.001074	0.0064	1	1	1	P26373	60S ribosoma	P26373	0	957.54	74904	8054.6	43.88		54346	
FALSE	High	TIPSWATLSASQLAR		FALSE	6.34E-09	0.0064	1	1	1	Q5SSJ5	Heterochrom	Q5SSJ5	0	1601.9	47065		1.56			47588
FALSE	High	TNGKEPELLEPIPYEFMA		FALSE	0.000959	0.0064	1	1	1	P46778	60S ribosoma	P46778	1	2078	31901	15786				
FALSE	High	TPPAGVFYQGWSATPIANGSL(		TRUE	9.50E-05	0.0064	1	1	2	cRAP117	000000|rLys-(	cRAP117	0	3083.5	441727		13.5		529182	441727
FALSE	High	TPPAGVFYQGWSATPIANGSL(		TRUE	3.52E-06	0.0064	1	1	2	cRAP117	000000|rLys-(	cRAP117	1	3454.7	914063		22.21		1E+06	914063
FALSE	High	TTEGCLNPR	1xCarbamidom	FALSE	0.00028	0.0064	1	1	1	P05107	Integrin beta-	P05107	0	1047.5	28071		17.34		27828	37275
FALSE	High	YLGYLEQLLR		TRUE	2.59E-05	0.0064	1	1	1	cRAP013	Alpha-S1-case	cRAP013	0	1267.7	192268		50.54		105950	310113
FALSE	High	LSAKPAPPKPEPRPK		FALSE	0.018477	0.0064	1	1	1	O00479	High mobility	O00479	0	1613	50796	2517.2	92.09		26333	166184
FALSE	High	IQVRLGEHNIDVLEGNEQFINA		TRUE	0.00027	0.0064	1	1	1	cRAP112	Trypsin OS=Su	cRAP112	1	2707.4	188754		41.58		83010	188754
FALSE	High	AVFVDLEPTV	1xLabel:13C(6)	FALSE	0.000205	0.0064	1	3	1	Q71U36	Tubulin alpha	Q71U36;	0	1707.9		136020		66.66		
FALSE	High	AYDNATAAVAK		TRUE	1.31E-05	0.0064	1	1	1	cRAP117	000000|rLys-(	cRAP117	0	1094.5	1E+07	2371.4	38.47		9E+06	1E+07
FALSE	High	AYDNATAAV	1xCarbamidom	TRUE	1.50E-15	0.0064	1	1	2	cRAP117	000000|rLys-(	cRAP117	1	3766.8	771961		69.08		151660	771961
FALSE	High	AYDNATAAV	1xCarbamidom	TRUE	0.000731	0.0064	1	1	1	cRAP117	000000|rLys-(	cRAP117	2	3922.9	95746		38.68			126763
FALSE	High	CDVDIRKDLY	1xCarbamidom	FALSE	0.03262	0.0064	1	2	1	P60709	Actin, cytopla	P60709;	2	3117.5	28059		37.62		28059	13172
FALSE	High	CELAAAMKR	1xCarbamidom	TRUE	0.000928	0.0064	1	1	2	cRAP078	Lysozyme C O	cRAP078	1	1049.5	137898		51.21		51238	169473

FALSE	High	ASGQAFELILS	1xLabel:13C(6)	FALSE	8.36E-05	0.0064	1	1	1	P16949	Stathmin OS=	P16949	0	1394.8		92848		12.96		
FALSE	High	CNKEVYFAEF	1xCarbamidom	FALSE	4.24E-05	0.0064	1	1	2	P50238	Cysteine-rich	P50238	1	1315.6	384790	55910	26.88	47.62	342569	384790
FALSE	High	DGFGASGSCI	2xCarbamidom	TRUE	4.37E-15	0.0064	1	1	2	cRAP117	000000|rLys-(	cRAP117	0	2231.9	228507		27.69		194969	228507
FALSE	High	DGFGASGSCI	2xCarbamidom	TRUE	9.47E-13	0.0064	1	1	1	cRAP117	000000|rLys-(	cRAP117	1	3307.5	43314		47.53		38959	87599
FALSE	High	DIGFIKLD		FALSE	0.015992	0.0064	1	1	1	P62273	40S ribosoma	P62273	1	920.51	55823	41512	24.65	26.52		66572
FALSE	High	DIGFIKLD	1xLabel:13C(6)	FALSE	0.020266	0.0064	1	1	1	P62273	40S ribosoma	P62273	1	926.53						
FALSE	High	DNIQGITKPAIR		FALSE	0.144026	0.0064	1	1	1	P62805	Histone H4 OS	P62805	0	1325.8	48183	11665	68.05		15870	82228
FALSE	High	DTLLLELK		TRUE	0.00061	0.0064	1	1	1	cRAP117	000000|rLys-(	cRAP117	0	944.57	272519	14022	39.62		151182	272519
FALSE	High	CSQAVYAAEI	1xCarbamidom	FALSE	0.000352	0.0064	1	1	1	P21291	Cysteine and g	P21291	1	1651.8	188528		14.5		194518	147415
FALSE	High	ASGPPVSELITK		FALSE	0.0995	0.0064	1	3	1	P16402	Histone H1.3 (	P16402;	0	1198.7	172967	47868	36.92	32.26	164135	305247
FALSE	High	AQAAAPASVPAQAPK		FALSE	0.000544	0.0064	1	1	1	P47914	60S ribosoma	P47914	0	1377.7	555409	185552	46.95	57.59	200182	556051
FALSE	High	AMKPWIQPK		TRUE	0.031026	0.0064	1	1	2	cRAP014	Alpha-S2-case	cRAP014	0	1098.6	37123		64.81		13027	37123
FALSE	High	AAFGGSGGRGSSSGGGYSSGSS		TRUE	3.26E-15	0.0064	1	1	2	P35908	Keratin, type I	P35908	1	2501.1	67393		46.97		67393	64592
FALSE	High	ACGVSRPVIA	2xCarbamidom	FALSE	0.007998	0.0064	1	1	1	P55769	NHP2-like pro	P55769	2	3799	71598		33.73			91310
FALSE	High	AEAESLYQSK		TRUE	0.00016	0.0064	1	1	1	P04264	Keratin, type I	P04264	0	1125.5	31466		72.69		9936.6	31466
FALSE	High	AEFVEVTKLVTDLTK		TRUE	0.001257	0.0064	1	1	1	cRAP087	Serum albumi	cRAP087	1	1692.9	28214		5.87		29412	27066
FALSE	High	AGFAGDDAPR		FALSE	5.90E-07	0.0064	1	10	1	P60709	Actin, cytopla	A5A3E0;	0	976.45	295235	63361	71.55	64.16	295235	274361
FALSE	High	AGLQFPVGR		FALSE	1.31E-06	0.0064	2	15	1	Q16777; Q	Histone H2A t	Q16777;	0	944.53	3E+06	529758	59.89	64.05	1E+06	4E+06
FALSE	High	AGLQFPVGR	1xLabel:13C(6)	FALSE	0.011822	0.0064	2	15	1	Q16777; Q	Histone H2A t	Q16777;	0	950.55						
FALSE	High	AGYRDGFGA	2xCarbamidom	TRUE	8.62E-13	0.0064	1	1	3	cRAP117	000000|rLys-(	cRAP117	1	2679.2	9E+06		34.93		9E+06	9E+06
FALSE	High	AGYRDGFGA	2xCarbamidom	TRUE	8.56E-18	0.0064	1	1	2	cRAP117	000000|rLys-(	cRAP117	2	3754.7	2E+07		53.49		6E+06	2E+07
FALSE	High	AHVEHMQSI	1xOxidation [M	FALSE	0.058342	0.0064	1	1	1	Q13029	PR domain zir	Q13029	0	2064	3E+06		92.35		8461.4	4E+06
FALSE	High	ALAAAGYDVEKNNSR		FALSE	0.05091	0.0064	1	5	1	P16402	Histone H1.3 (	P16402;	1	1578.8	108281	44694	109.67	109.77	23195	383101
FALSE	High	ALCSEKLDQV	2xCarbamidom	TRUE	0.009181	0.0064	1	1	1	cRAP075	Alpha-lactalbu	cRAP075	2	1892.9	99944		71.23		19746	142121
FALSE	High	ALKAWSVAR		TRUE	0.001521	0.0064	1	1	1	cRAP087	Serum albumi	cRAP087	1	1001.6	84450		65.26		18032	103127
FALSE	High	AMGIMNSFV	1xOxidation [M	FALSE	0.003929	0.0064	2	14	1	O60814; Q	Histone H2B t	Q16778;	0	1759.8	130285	43204	82.37	48.43	18728	222347
FALSE	High	AMGIMNSFV	1xOxidation [M	FALSE	0.001027	0.0064	2	14	1	O60814; Q	Histone H2B t	Q16778;	0	1765.8						
FALSE	High	EIQTAVRLLLPGELAK		FALSE	0.019855	0.0064	1	14	1	O60814	Histone H2B t	Q16778;	1	1751	6E+06	1E+06	75.2	74.47	733002	8E+06
FALSE	High	EVQTAVRLLLPGELAK		FALSE	0.053979	0.0064	1	1	1	Q8N257	Histone H2B t	Q8N257	1	1737	149249	82898	71.62	71.95	21130	175303
FALSE	High	GGLYGGPSY(	1xCarbamidom	TRUE	3.56E-08	0.0064	1	1	1	cRAP117	000000|rLys-(	cRAP117	1	3593.6	4E+06		59.88		2E+06	7E+06
FALSE	High	GGSISGGGYGSGGGK		TRUE	3.40E-08	0.0064	1	1	1	P35908	Keratin, type I	P35908	0	1197.5	67203		110.32		340998	42398
FALSE	High	LAPDYDALDVANKIGII		FALSE	0.001778	0.0064	1	1	1	P62750	60S ribosoma	P62750	1	1801	49803	48001		43.24		
FALSE	High	LGEHNIDVLEGNEQFINAAK		TRUE	2.29E-15	0.0064	1	1	2	cRAP112	Trypsin OS=Su	cRAP112	0	2211.1	7E+06		47.1		5E+06	7E+06
FALSE	High	LGEHNIEVLEGNEQFINAAK		FALSE	1.21E-07	0.0064	1	3	1	P07477	Trypsin-1 OS=	P07477;	0	2225.1	25336		54.08		20919	25336
FALSE	High	IGLGGRGGSGGSYGR		TRUE	0.003601	0.0064	1	1	1	P35527	Keratin, type I	P35527	1	1350.7	32024		23.79		32024	25855
FALSE	High	IHRHLK		FALSE	0.081623	0.0064	1	2	1	Q71UI9	Histone H2A.\	Q71UI9;	1	803.5	771043	14850	30.83	9.79	1E+06	771043
FALSE	High	LKAEVEKQSK		FALSE	0.000847	0.0064	1	1	1	Q9UM00	Calcium load-a	Q9UM00	2	1159.7	10458		119.42		6412.4	10458
FALSE	High	LKTKENLEK		FALSE	0.025617	0.0064	1	1	1	Q6NZI2	Caveolae-assc	Q6NZI2	2	1102.6	45671		56.29		45671	16033
FALSE	High	LLDFGSLSNLQVTQPTVGMNFI		FALSE	5.62E-05	0.0064	1	1	1	P08708	40S ribosoma	P08708	1	2763.4	45069	17703	116.45	108.05	14025	144825
FALSE	High	LLDFGSLSNL(	2xLabel:13C(6)	FALSE	2.81E-05	0.0064	1	1	1	P08708	40S ribosoma	P08708	1	2775.5						
FALSE	High	LLDFGSLSNL(	1xOxidation [M	FALSE	0.013828	0.0064	1	1	1	P08708	40S ribosoma	P08708	2	3032.6						
FALSE	High	ILGADTSVDLI	2xLabel:13C(6)	FALSE	0.056668	0.0064	1	1	1	P25705	ATP synthase	P25705	1	2569.4	28057	22140	66.72	96.29	20391	28057

FALSE	High	LLLPGELAK		FALSE	0.102333	0.0064	2	15	1	O60814; Q	Histone H2B t	Q16778;	0	953.6	4E+06	805175	71.42	63.99	2E+06	8E+06
FALSE	High	IITHPNFNGNTLDNDIMLIK		TRUE	8.04E-13	0.0064	1	1	4	cRAP112	Trypsin OS=Su	cRAP112	0	2283.2	7E+06		31.31		4E+06	7E+06
FALSE	High	IITHPNFNGN	1xOxidation [M	TRUE	0.000246	0.0064	1	1	2	cRAP112	Trypsin OS=Su	cRAP112	0	2299.2	529976		39.59		313067	529976
FALSE	High	IITHPNFNGNTLDNDIMLIKLSSI		TRUE	2.78E-07	0.0064	1	1	1	cRAP112	Trypsin OS=Su	cRAP112	1	3309.7	2E+06	12323	24.99		1E+06	2E+06
FALSE	High	LAHYNKR		FALSE	0.002678	0.0064	2	15	1	O60814; Q	Histone H2B t	Q16778;	1	901.5	426349	94811	21.3	34.38	387307	574138
FALSE	High	LSAKPAPPKPEPKPK		FALSE	0.003228	0.0064	1	1	1	P05204	Non-histone c	P05204	0	1584.9	208000	22026	43.48	41.67	107420	278818
FALSE	High	LAADEDDDD	3xLabel:13C(6)	FALSE	3.36E-06	0.0064	1	1	1	P06748	Nucleophosm	P06748	2	4264.6	29454	70471				
FALSE	High	KTVTAMDVVYALKR		FALSE	9.32E-06	0.0064	1	1	1	P62805	Histone H4 OS	P62805	2	1594.9	38976	15328	51.65		36480	85754
FALSE	High	GSCGIGGGIG	1xCarbamidom	FALSE	1.09E-07	0.0064	1	2	1	P02533	Keratin, type I	P02533;	0	1278.6	53660		21.68		61101	39220
FALSE	High	GSGGGSSGGSIGGRGSSSGGVK		TRUE	0.020474	0.0064	1	1	1	P04264	Keratin, type I	P04264	1	1751.8	67188		39.14		98163	43775
FALSE	High	GSLGGGFSSGGFSGGSFSR		TRUE	2.39E-11	0.0064	1	1	1	P13645	Keratin, type I	P13645	0	1707.8	238125		13.4		248288	191505
FALSE	High	GTGASGSFKL	3xLabel:13C(6)	FALSE	0.140134	0.0064	2	5	1	P16401; P1	Histone H1.5 (	P16401;	2	1312.8	98762	77330	108.52	111.47	21426	341526
FALSE	High	HAVSEGTKAVTK		FALSE	8.81E-10	0.0064	2	15	3	O60814; Q	Histone H2B t	Q16778;	1	1227.7						
FALSE	High	HAVSEGTKAV	2xLabel:13C(6)	FALSE	9.23E-08	0.0064	2	15	2	O60814; Q	Histone H2B t	Q16778;	1	1239.7	2E+06	448778	22.4	26.06	1E+06	2E+06
FALSE	High	HAVSEGTKAV	3xLabel:13C(6)	FALSE	2.20E-05	0.0064	1	3	1	O60814	Histone H2B t	O60814;	2	1796	258319	30780	86.77	110.74		527826
FALSE	High	HAVSEGTKAVTKYTSSK		FALSE	2.72E-08	0.0064	1	12	3	Q8N257	Histone H2B t	Q16778;	2	1793.9	895498	226813	100.14	100.79	146279	2E+06
FALSE	High	HAVSEGTKAV	3xLabel:13C(6)	FALSE	9.22E-07	0.0064	1	12	2	Q8N257	Histone H2B t	Q16778;	2	1812						
FALSE	High	HGGGGGGFGGGGFGSR		TRUE	1.41E-07	0.0064	1	1	1	P35908	Keratin, type I	P35908	0	1320.6	47874		23.35		50704	31872
FALSE	High	HGLDNYRGY	1xCarbamidom	TRUE	0.005928	0.0064	1	1	1	cRAP078	Lysozyme C O	cRAP078	1	2181	63638		67.11		41409	149087
FALSE	High	KGTVEGFEPA	1xCarbamidom	FALSE	1.89E-07	0.0064	1	1	1	P37108	Signal recogni	P37108	2	1934	30619	41112	104.96	70.17	7206.9	99839
FALSE	High	KGTVEGFEPA	1xCarbamidom	FALSE	3.24E-05	0.0064	1	1	1	P37108	Signal recogni	P37108	2	1952						
FALSE	High	KIEEIKDFLLTAR		FALSE	8.56E-05	0.0064	1	1	1	P63173	60S ribosoma	P63173	2	1575.9	50938		48.08		36319	50938
FALSE	High	KIIGLK		FALSE	0.002425	0.0064	1	1	2	Q6IN85	Serine/threon	Q6IN85	1	671.48	89033	73974	74.78	71.62	30079	176551
FALSE	High	KYSQGNVSAVGVTYDGHTALT		TRUE	7.56E-08	0.0064	1	1	1	cRAP117	000000|rLys-(	cRAP117	1	2324.2	2E+06		16.75			2E+06
FALSE	High	YSQGNVSAVGVTYDGHTALTR		TRUE	1.12E-12	0.0064	1	1	2	cRAP117	000000|rLys-(	cRAP117	0	2196.1	118254		46.83		118254	210749

Abundan	Abundan	Abundan	Abundan	Precurso	Quan Infı	Quan Cha	Found in	Found in	Found in	Found in	Found in	Found in	Confiden	Percolatc	Percolatc	XCorr by	Top Apex RT in min
					IndistinguishableCl		Not Four	Not Four	Not Four	Not Four	Not Four	Not Four	High	0.0096	0.1209	3.45	136.91
520478				86		Light	Peak Fou	High	High	Not Four	Not Four	Not Four	High	0.0096	0.0082	4.36	139.62
2E+07				87		Light	Peak Fou	High	High	Not Four	Not Four	Not Four	High	0.0095	0.0218	2.68	131.43
810816	105655	277367	169458	88		Light	Peak Fou	Peak Fou	High	Peak Fou	Peak Fou	Peak Fou	High	0.0096	0.2053	1.7	57.14
802681	95773	205171	187820	89		Light	Peak Fou	High	Peak Fou	Peak Fou	Peak Fou	Peak Fou	High	0.0095	0.0024	3.84	109.5
443413	24360	179701	73971	90		Light	Peak Fou	High	High	Peak Fou	Peak Fou	Peak Fou	High	0.0096	0.0368	3.15	94.47
23822		13392	4035.6	91		Light	Not Four	High	Peak Fou	Not Four	Peak Fou	Peak Fou	High	0.0095	0.049	2.72	73.21
45160				85		Light	Peak Fou	Peak Fou	High	Not Four	Not Four	Not Four	High	0.0096	0.029	3.79	41.43
402786				92		Light	Peak Fou	High	High	Not Four	Not Four	Not Four	High	0.0096	0.0003	5.66	144.44
				94	Redunda	Light	Peak Fou	High	Peak Fou	Not Four	Peak Fou	Peak Fou	High	0.0095	0.0105	3.58	189.79
54704		39677	11138	94		Heavy	Peak Fou	Peak Fou	Peak Fou	Not Four	High	Peak Fou	High	0.0095	0.0493	3.1	189.79
42531				95		Light	Not Four	Not Four	High	Not Four	Not Four	Not Four	High	0.0096	0.0392	3.9	110.69
				96	NoQuan\	Heavy	Not Four	Not Four	Not Four	Not Four	High	Not Four	High	0.0095	0.0127	3.63	
1E+06		7326.4		97		Light	Peak Fou	High	Peak Fou	Not Four	Peak Fou	Not Four	High	0.0095	0.0258	2.29	91.63
600521				98		Light	Not Four	Not Four	High	Not Four	Not Four	Not Four	High	0.0096	0.2671	2.27	87.56
66648				93		Light	Peak Fou	Peak Fou	High	Not Four	Not Four	Not Four	High	0.0096	0.0147	3.44	51.29
645745				84		Light	Peak Fou	High	Peak Fou	Not Four	Not Four	Not Four	High	0.0095	0.0066	3.54	59.67
37529	30609	168241	59763	83		Heavy	Peak Fou	Peak Fou	Peak Fou	Peak Fou	High	Peak Fou	High	0.0095	0.0799	3.04	81.46
440483				82		Light	Peak Fou	High	High	Not Four	Not Four	Not Four	High	0.0095	0.0488	3.25	123.75
4E+06				69		Light	Peak Fou	High	Peak Fou	Not Four	Not Four	Not Four	High	0.0095	0.0672	2.09	74.01
					IndistinguishableCl		Not Four	Not Four	Not Four	Not Four	Not Four	Not Four	High	0.0096	0.2072	2.75	133.22
17433				70		Light	Not Four	Peak Fou	High	Not Four	Not Four	Not Four	High	0.0096	0.0361	4.85	189.33
112792				71		Light	Peak Fou	High	Peak Fou	Not Four	Not Four	Not Four	High	0.0095	0.0159	3.46	189.06
127981	6738.2	42799	43672	72		Light	Peak Fou	Peak Fou	High	Peak Fou	Peak Fou	Peak Fou	High	0.0096	0.0557	2.92	77.13
64614	6691.3	66838	44992	73		Heavy	Not Four	Not Four	Peak Fou	Peak Fou	Peak Fou	High	High	0.0096	0.1651	2.84	183.2
					InconsistentlyLabe		Not Four	Not Four	Not Four	Not Four	Not Four	Not Four	High	0.0096	0.2538	2.87	81.67
3E+07		52449	16562	75		Light	Peak Fou	High	High	Not Four	Peak Fou	Peak Fou	High	0.0095	#######	7.44	168.17
747191	11438	63224	148100	74		Light	Peak Fou	High	High	Peak Fou	Peak Fou	Peak Fou	High	0.0095	#######	6.29	159.69
414028				76		Light	Peak Fou	Peak Fou	High	Not Four	Not Four	Not Four	High	0.0096	0.0017	5.17	190.55
343259				77		Light	Peak Fou	Peak Fou	High	Not Four	Not Four	Not Four	High	0.0096	0.0037	5.83	181.57
34929				78		Light	Peak Fou	Peak Fou	High	Not Four	Not Four	Not Four	High	0.0096	0.1315	3.21	136.47
41488				79		Light	Peak Fou	High	Peak Fou	Not Four	Not Four	Not Four	High	0.0095	0.0032	4.53	140.75
56980				80		Light	Peak Fou	High	Peak Fou	Not Four	Not Four	Not Four	High	0.0095	0.1326	1.4	68.41
4E+06				81		Light	Peak Fou	High	Peak Fou	Not Four	Not Four	Not Four	High	0.0095	0.004	3.39	124.31
3E+06				99		Light	Peak Fou	Peak Fou	High	Not Four	Not Four	Not Four	High	0.0096	0.1911	2.27	88.67
262929				100		Light	Not Four	High	High	Not Four	Not Four	Not Four	High	0.0095	0.0005	3.84	106.52
862817				101		Light	Peak Fou	High	High	Not Four	Not Four	Not Four	High	0.0095	#######	6	186.65
118925				102		Light	Peak Fou	High	Peak Fou	Not Four	Not Four	Not Four	High	0.0095	0.1334	2.52	170.21
				121	NoQuan\	Heavy	Not Four	Not Four	Not Four	Not Four	High	Not Four	High	0.0095	0.0004	4.92	
34389		8464.9	4893.2	122		Light	Peak Fou	Peak Fou	High	Not Four	Peak Fou	Peak Fou	High	0.0096	0.128	2.9	148.36

86415	5211.8	7166.1		123		Light	Peak Fou	High	High	Peak Fou	Peak Fou	Not Four	High	0.0095	0.0147	3.53	132.65
12989		11860		124		Light	Peak Fou	High	Peak Fou	Not Four	Peak Fou	Not Four	High	0.0095	0.0178	2.48	143.58
153267	27233	69736	32517	125	NotUniq	Light	Peak Fou	High	Peak Fou	Peak Fou	Peak Fou	Peak Fou	High	0.0095	0.028	2.74	57.33
77931				126		Light	Peak Fou	Peak Fou	High	Not Four	Not Four	Not Four	High	0.0096	0.002	4.81	107.32
				127	Redunda	Light	Peak Fou	Not Four	High	Peak Fou	Not Four	Peak Fou	High	0.0096	0.006	4.67	207.89
316258	36775		55295	127		Heavy	Peak Fou	Not Four	Peak Fou	Peak Fou	Not Four	High	High	0.0096	0.1854	2.79	207.89
368255	15852	77028	63757	128		Heavy	Peak Fou	Peak Fou	Peak Fou	Peak Fou	High	Peak Fou	High	0.0095	0.1891	1.87	72.84
327986	26416	120033	68485	129		Light	Peak Fou	High	High	Peak Fou	Peak Fou	Peak Fou	High	0.0095	0.0225	2.58	124.76
				130	NoQuan\	Light	Not Four	High	Not Four	Not Four	Not Four	Not Four	High	0.0095	0.1096	3.14	
93515	5232.4	27028	21175	131		Light	Peak Fou	Peak Fou	High	Peak Fou	Peak Fou	Peak Fou	High	0.0096	0.1321	2.36	91.66
114504		18467		132		Light	Not Four	High	Peak Fou	Not Four	Peak Fou	Not Four	High	0.0095	0.0084	3.06	173.48
		94327	45652	133		Heavy	Not Four	Not Four	Not Four	Not Four	High	Peak Fou	High	0.0095	0.0537	3.37	151.19
280139				134		Light	Peak Fou	Peak Fou	High	Not Four	Not Four	Not Four	High	0.0096	0.1279	3.43	56.38
39734		50262	40481	120		Heavy	Peak Fou	Peak Fou	Peak Fou	Not Four	High	Peak Fou	High	0.0095	0.067	3.19	179.38
392375	3371.2	18370	5287.5	68		Light	Peak Fou	High	Peak Fou	Peak Fou	Peak Fou	Peak Fou	High	0.0095	0.0022	3.72	116.24
42786				119		Light	Peak Fou	Peak Fou	High	Not Four	Not Four	Not Four	High	0.0096	0.0093	4.78	83.41
				117	NoQuan\	Light	Not Four	Not Four	High	Not Four	Not Four	Not Four	High	0.0096	0.274	2.92	
96125				103		Light	Not Four	Peak Fou	High	Not Four	Not Four	Not Four	High	0.0096	0.0087	4.07	164.57
		90727	6601.8	104		Light	Peak Fou	High	Not Four	Not Four	Peak Fou	Peak Fou	High	0.0095	0.1442	2.62	98.61
54461				105		Light	Peak Fou	Peak Fou	High	Not Four	Not Four	Not Four	High	0.0096	0.033	3.96	71.54
503275				106		Light	Peak Fou	High	Peak Fou	Not Four	Not Four	Not Four	High	0.0095	0.1765	2.05	93.92
98817				107		Light	Peak Fou	Peak Fou	High	Not Four	Not Four	Not Four	High	0.0096	0.1688	2.39	76.8
1E+06	26069	325237	79846	108		Light	Peak Fou	High	High	Peak Fou	Peak Fou	Peak Fou	High	0.0095	0.027	2.29	97.63
1E+06	78427	821565	387273	109		Light	Peak Fou	High	High	Peak Fou	Peak Fou	Peak Fou	High	0.0095	0.0194	2.4	84.24
				109	Redunda	Heavy	Peak Fou	Peak Fou	Peak Fou	Peak Fou	Peak Fou	High	High	0.0096	0.0214	3.89	84.24
				110	NoQuan\	Light	Not Four	Not Four	High	Not Four	Not Four	Not Four	High	0.0096	0.0185	3.28	
5618.6				111		Light	Peak Fou	Peak Fou	High	Not Four	Not Four	Not Four	High	0.0096	0.0798	3.93	197.52
103238			8054.6	112		Light	Peak Fou	Not Four	High	Not Four	Not Four	Peak Fou	High	0.0096	0.0785	2.76	106.11
46548				113		Light	Not Four	Peak Fou	High	Not Four	Not Four	Not Four	High	0.0096	0.0042	4.67	171.94
31901			15786	114		Light	Not Four	Not Four	High	Not Four	Not Four	Peak Fou	High	0.0096	0.0764	4.14	205.17
409053				115		Light	Peak Fou	High	Peak Fou	Not Four	Not Four	Not Four	High	0.0095	0.0107	3.43	151.78
688514				116		Light	Peak Fou	High	Peak Fou	Not Four	Not Four	Not Four	High	0.0095	0.003	3.63	142.72
28071				118		Light	Peak Fou	High	Peak Fou	Not Four	Not Four	Not Four	High	0.0095	0.0163	2.94	51.44
192268				135		Light	Peak Fou	Peak Fou	High	Not Four	Not Four	Not Four	High	0.0096	0.0319	3.21	195.92
50796		2517.2		67		Light	Peak Fou	High	Peak Fou	Not Four	Peak Fou	Not Four	High	0.0095	0.0822	2.13	45.58
204233				65		Light	Peak Fou	Peak Fou	High	Not Four	Not Four	Not Four	High	0.0096	0.0561	3.91	160.47
	23911	147782	136020	18		Heavy	Not Four	Not Four	Not Four	Peak Fou	High	Peak Fou	High	0.0095	0.0145	4.47	189.76
2E+07		2371.4		19		Light	Peak Fou	High	Peak Fou	Not Four	Peak Fou	Not Four	High	0.0095	0.005	3.53	63.67
1E+06				20		Light	Peak Fou	High	Peak Fou	Not Four	Not Four	Not Four	High	0.0095	#######	6.67	199.82
72319				21		Light	Not Four	Peak Fou	High	Not Four	Not Four	Not Four	High	0.0096	0.0716	4.41	187.33
28612				22		Light	Peak Fou	Peak Fou	High	Not Four	Not Four	Not Four	High	0.0096	0.1842	3.21	162.26
137898				23		Light	Peak Fou	High	High	Not Four	Not Four	Not Four	High	0.0095	0.0258	3.15	63.75

		84695	101785	17		Heavy	Not Four	Not Four	Not Four	Not Four	Peak Fou	High	High	0.0096	0.0423	3.27	166.97
560069	20137	55910	57961	24		Light	Peak Fou	High	High	Peak Fou	Peak Fou	Peak Fou	High	0.0095	0.0282	3.42	71.13
328373				26		Light	Peak Fou	High	High	Not Four	Not Four	Not Four	High	0.0096	0.0001	7.67	123.18
43314				27		Light	Peak Fou	Peak Fou	High	Not Four	Not Four	Not Four	High	0.0096	0.0005	6.08	149.01
46809		50187	34337	28		Light	Not Four	Peak Fou	High	Not Four	Peak Fou	Peak Fou	High	0.0096	0.1537	2.03	176.4
				28	Redunda	Heavy	Not Four	Peak Fou	Peak Fou	Not Four	Peak Fou	High	High	0.0096	0.1634	2.16	176.4
48183		11665		29		Light	Peak Fou	High	Peak Fou	Not Four	Peak Fou	Not Four	High	0.0095	0.1842	2.73	92.47
355947		14022		30		Light	Peak Fou	High	Peak Fou	Not Four	Peak Fou	Not Four	High	0.0095	0.022	2.62	159.31
188528				25		Light	Peak Fou	Peak Fou	High	Not Four	Not Four	Not Four	High	0.0096	0.0598	3.1	121.72
172967	45522	78678	47868	16		Light	Peak Fou	High	Peak Fou	Peak Fou	Peak Fou	Peak Fou	High	0.0095	0.1597	2.67	118.37
555409	57664	185552	236314	15		Light	Peak Fou	Peak Fou	High	Peak Fou	Peak Fou	Peak Fou	High	0.0096	0.0665	3.24	75.28
61097				14		Light	Peak Fou	High	High	Not Four	Not Four	Not Four	High	0.0095	0.1461	2.15	84.38
139606				1		Light	Peak Fou	High	High	Not Four	Not Four	Not Four	High	0.0096	0.0001	7.1	69.36
56142				2		Light	Not Four	High	Peak Fou	Not Four	Not Four	Not Four	High	0.0095	0.0594	3.77	166.72
58041				3		Light	Peak Fou	Peak Fou	High	Not Four	Not Four	Not Four	High	0.0096	0.0495	3.31	68.09
				4		Light	Peak Fou	High	Not Four	Not Four	Not Four	Not Four	High	0.0095	0.029	2.54	188.39
885674	63361	59845	170279	5		Light	Peak Fou	Peak Fou	High	Peak Fou	Peak Fou	Peak Fou	High	0.0096	0.0128	3.35	65.6
3E+06	166753	806791	529758	6	NotUniqu	Light	Peak Fou	High	Peak Fou	Peak Fou	Peak Fou	Peak Fou	High	0.0095	0.002	2.87	123.36
				6	Redunda	Heavy	Peak Fou	Peak Fou	Peak Fou	Peak Fou	High	Peak Fou	High	0.0095	0.069	2.74	123.36
2E+07				7		Light	Peak Fou	Peak Fou	High	Not Four	Not Four	Not Four	High	0.0096	0.0005	5.76	113.7
2E+07				8		Light	Peak Fou	High	Peak Fou	Not Four	Not Four	Not Four	High	0.0095	#######	7.55	139.12
3E+06				9		Light	Peak Fou	Peak Fou	High	Not Four	Not Four	Not Four	High	0.0096	0.2138	2.89	115.65
108281	9088.7	156613	44694	10		Light	Peak Fou	High	Peak Fou	Peak Fou	Peak Fou	Peak Fou	High	0.0095	0.1227	2.25	74.34
99944				11		Light	Peak Fou	High	Peak Fou	Not Four	Not Four	Not Four	High	0.0095	0.0626	3.86	170.27
84450				12		Light	Peak Fou	High	Peak Fou	Not Four	Not Four	Not Four	High	0.0095	0.0312	3.03	80.65
130285		61730	30238	13	NotUniqu	Light	Peak Fou	High	Peak Fou	Not Four	Peak Fou	Peak Fou	High	0.0095	0.045	3.55	191.76
				13	Redunda	Heavy	Peak Fou	Peak Fou	Peak Fou	Not Four	High	Peak Fou	High	0.0095	0.0268	4.69	192.11
6E+06	174891	2E+06	1E+06	31		Light	Peak Fou	High	Peak Fou	Peak Fou	Peak Fou	Peak Fou	High	0.0095	0.0845	3.38	171.72
149249	12769	104830	82898	32		Light	Peak Fou	Peak Fou	High	Peak Fou	Peak Fou	Peak Fou	High	0.0096	0.2093	2.93	163.1
4E+06				33		Light	Peak Fou	High	Peak Fou	Not Four	Not Four	Not Four	High	0.0095	0.0005	5.14	185.97
67203				34		Light	Peak Fou	Peak Fou	High	Not Four	Not Four	Not Four	High	0.0096	0.0064	4.37	46.65
49803		65831	35001	51		Light	Not Four	Not Four	High	Not Four	Peak Fou	Peak Fou	High	0.0096	0.0888	3.48	209.83
1E+07				52		Light	Peak Fou	High	High	Not Four	Not Four	Not Four	High	0.0095	#######	5.29	151.32
54282				53		Light	Peak Fou	Peak Fou	High	Not Four	Not Four	Not Four	High	0.0096	0.0087	4.81	151.36
41502				54		Light	Peak Fou	Peak Fou	High	Not Four	Not Four	Not Four	High	0.0096	0.1059	3.61	64.31
590188	14850	16356	13442	55		Light	Peak Fou	Peak Fou	High	Peak Fou	Peak Fou	Peak Fou	High	0.0096	0.2328	1.86	13.81
64273				56		Light	Peak Fou	Peak Fou	High	Not Four	Not Four	Not Four	High	0.0096	0.0741	2.62	22.55
61638				57		Light	Peak Fou	Peak Fou	High	Not Four	Not Four	Not Four	High	0.0096	0.1734	2.6	26.18
	8026.9	75389	17703	58		Light	Peak Fou	High	Not Four	Peak Fou	Peak Fou	Peak Fou	High	0.0095	0.0088	3.06	196.88
				58	Redunda	Heavy	Peak Fou	Peak Fou	Not Four	Peak Fou	High	Peak Fou	High	0.0095	0.0067	3.93	196.88
				59	NoQuan\	Light	Not Four	High	Not Four	Not Four	Not Four	Not Four	High	0.0095	0.0734	2.56	
68955	9647.2		50811	60		Heavy	Peak Fou	Peak Fou	Peak Fou	Peak Fou	Not Four	High	High	0.0096	0.2123	3.09	188.46

4E+06	428059	2E+06	805175	61	NotUniqu	Light	Peak Fou	Peak Fou	High	Peak Fou	Peak Fou	Peak Fou	High	0.0096	0.2467	2.29	138.03
7E+06				62		Light	Peak Fou	High	High	Not Foun	Not Foun	Not Foun	High	0.0095	#######	4.58	169.57
727379				63		Light	Peak Fou	Peak Fou	High	Not Foun	Not Foun	Not Foun	High	0.0096	0.0549	3.08	161.36
2E+06		12323		64		Light	Peak Fou	High	Peak Fou	Not Foun	Peak Fou	Not Foun	High	0.0095	0.0011	3.49	186.17
426349	94811	154001	83038	50	NotUniqu	Light	Peak Fou	Peak Fou	High	Peak Fou	Peak Fou	Peak Fou	High	0.0096	0.0983	1.35	14.24
208000	10806	27051	22026	66		Light	Peak Fou	High	Peak Fou	Peak Fou	Peak Fou	Peak Fou	High	0.0095	0.0417	2.83	42.4
29454			70471	49		Heavy	Not Foun	Not Foun	Peak Fou	Not Foun	Not Foun	High	High	0.0096	0.0195	3.83	105.61
38976		15328		47		Light	Peak Fou	High	Peak Fou	Not Foun	Peak Fou	Not Foun	High	0.0095	0.0044	3.33	135.7
53660				35		Light	Peak Fou	High	Peak Fou	Not Foun	Not Foun	Not Foun	High	0.0095	0.0008	3.52	68.92
67188				36		Light	Peak Fou	High	Peak Fou	Not Foun	Not Foun	Not Foun	High	0.0095	0.0855	2.02	33.18
238125				37		Light	Peak Fou	High	Peak Fou	Not Foun	Not Foun	Not Foun	High	0.0095	#######	5.5	141.29
98762	18569	293045	77330	38	NotUniqu	Heavy	Peak Fou	Peak Fou	Peak Fou	Peak Fou	High	Peak Fou	High	0.0095	0.1822	1.16	40.04
				39	Redunda	Light	Peak Fou	High	High	Peak Fou	Peak Fou	Peak Fou	High	0.0095	0.0001	4.71	26.24
2E+06	300217	514389	448778	39	NotUniqu	Heavy	Peak Fou	Peak Fou	Peak Fou	Peak Fou	High	High	High	0.0095	0.0007	4.24	26.24
126421	7657.7	115740	30780	40		Heavy	Not Foun	Peak Fou	Peak Fou	Peak Fou	High	Peak Fou	High	0.0095	0.0061	4.58	57.41
895498	66568	719528	226813	41		Light	Peak Fou	High	High	Peak Fou	Peak Fou	Peak Fou	High	0.0095	0.0005	5.92	55.18
				41	Redunda	Heavy	Peak Fou	Peak Fou	Peak Fou	Peak Fou	High	High	High	0.0095	0.0098	4.5	55.18
47874				42		Light	Peak Fou	Peak Fou	High	Not Foun	Not Foun	Not Foun	High	0.0096	0.009	4.21	66.12
63638				43		Light	Peak Fou	Peak Fou	High	Not Foun	Not Foun	Not Foun	High	0.0096	0.1199	3.31	142.37
30619	10477	64759	41112	44		Light	Peak Fou	High	Peak Fou	Peak Fou	Peak Fou	Peak Fou	High	0.0095	0.001	4.33	102.46
				44	Redunda	Heavy	Peak Fou	Peak Fou	Peak Fou	Peak Fou	High	Peak Fou	High	0.0095	0.0071	4.59	102.46
91649				45		Light	Peak Fou	Peak Fou	High	Not Foun	Not Foun	Not Foun	High	0.0096	0.0425	2.87	145.6
89033	29120	147712	73974	46		Light	Peak Fou	High	Peak Fou	Peak Fou	Peak Fou	Peak Fou	High	0.0095	0.0374	2.07	69.03
2E+06				48		Light	Not Foun	High	Peak Fou	Not Foun	Not Foun	Not Foun	High	0.0095	0.0007	3.54	102.4
85941				136		Light	Peak Fou	High	High	Not Foun	Not Foun	Not Foun	High	0.0095	#######	5.87	116.8

Supplemental Table 4.5. Raw Proteome discoverer output from the three replicates of Condition E

Checked	Confid	Sequence	Modifications	Contamin	Qvality PEF	Qvality q-	Numl	Numl	Numl	Master Pr	Master Protein Des	Protein Ac	Numb	Theo MHp	Abundances	Abundanc	Abundanc	Abundanc
FALSE	High	AAAAAAAAAPAAAATAPTTAAT		FALSE	5.87E-07	0.00611	1	1	1	P37108	Signal recognition p	P37108	0	2368.21				
FALSE	High	RKATGPPVSE	3xLabel:13C(6) [	FALSE	1.31E-06	0.00611	1	1	1	P16401	Histone H1.5 OS=H	P16401	2	1514.94	88151	149552	49.17	66.27
FALSE	High	RKPDTIEVQQMK		FALSE	0.000286	0.00611	1	3	2	P35241	Radixin OS=Homo s	P35241; P	1	1472.79	1373099		32.66	
FALSE	High	RKPDTIEVQQ	1xOxidation [M1	FALSE	0.080761	0.00611	1	3	1	P35241	Radixin OS=Homo s	P35241; P	2	1915.02	40257		30.49	
FALSE	High	RLPDAHSDYA	2xLabel:13C(6) [	FALSE	0.002962	0.00611	1	1	1	Q96PK6	RNA-binding protei	Q96PK6	1	1312.68	6257	37414	54.63	29.22
FALSE	High	RSTITSREIQTAVR		FALSE	0.023255	0.00611	1	13	1	Q16778	Histone H2B type 2	Q16778; (	2	1617.9	88702	36744	68.69	34.23
FALSE	High	RTPPAGVFYQGWSATPIANGSL(		TRUE	4.81E-08	0.00611	1	1	7	cRAP117	000000|rLys-C rLys	cRAP117	2	3610.81	28231713		50.98	
FALSE	High	RVTIMPKDIQLAR		FALSE	1.16E-06	0.00611	1	5	2	P84243	Histone H3.3 OS=H	P84243; C	2	1540.9	445451	159922	101.82	66.99
FALSE	High	SAHSIAQLQK		FALSE	0.001217	0.00611	1	1	1	Q9ULS5	Transmembrane ar	Q9ULS5	0	1082.6	41504		11.92	
FALSE	High	SAINEVVTR	1xLabel:13C(6) [	FALSE	4.35E-05	0.00611	1	1	2	P62899	60S ribosomal prot	P62899	0	994.562	72233	75404	25.74	65.04
FALSE	High	SCQAQPTTM	1xCarbamidome	TRUE	0.00065	0.00611	1	1	1	cRAP016	Kappa-casein OS=B	cRAP016	0	1250.56	78666		36.16	
FALSE	High	SGDAAIVDM\	1xCarbamidome	FALSE	0.147458	0.00611	1	2	1	P68104	Elongation factor 1	P68104; C	0	2995.4	27486	23315	43.45	
FALSE	High	SGGGGGGGF	1xCarbamidome	FALSE	1.02E-06	0.00611	1	1	1	P13647	Keratin, type II cytc	P13647	1	2257.05				
FALSE	High	SGGGGGGGC	1xCarbamidome	TRUE	3.10E-13	0.00611	1	1	2	P13645	Keratin, type I cyto	P13645	0	1549.68	44797		6.46	
FALSE	High	SGGGGGGGLGSGGSIR		TRUE	2.91E-06	0.00611	1	1	1	P35527	Keratin, type I cyto	P35527	0	1232.6	569856		58.83	
FALSE	High	SGGGGGRFSS	1xCarbamidome	TRUE	4.04E-06	0.00611	1	1	1	P04264	Keratin, type II cytc	P04264	1	2293.98	380324		28.63	
FALSE	High	RKATGPPVSELITK		FALSE	2.82E-06	0.00611	1	1	1	P16401	Histone H1.5 OS=H	P16401	2	1496.88				
FALSE	High	RKASGPPVSELITK		FALSE	0.000105	0.00611	1	2	1	P10412	Histone H1.4 OS=H	P16402; P	2	1482.86	217116	63159	59.41	68.23
FALSE	High	RHPEYAVSVLLR		TRUE	9.72E-05	0.00611	1	1	1	cRAP087	Serum albumin OS=	cRAP087	1	1439.81				
FALSE	High	RGDLPFVVPRR		FALSE	6.67E-05	0.00611	1	1	1	P35579	Myosin-9 OS=Hom	P35579	2	1311.76	152399		23.97	
FALSE	High	MFGGPGTASRPSSSR		FALSE	0.10185	0.00611	1	1	1	P08670	Vimentin OS=Hom	P08670	0	1494.71	209453	17994	48.45	
FALSE	High	MSVQPTVSL(	1xLabel:13C(6) [	FALSE	0.003736	0.00611	1	1	1	P06748	Nucleophosmin OS	P06748	0	2233.24	11300	26265	40.45	70.12
FALSE	High	MVFTSSADG(	1xCarbamidome	TRUE	6.51E-21	0.00611	1	1	5	cRAP117	000000|rLys-C rLys	cRAP117	0	2691.24	7682254	18463	23.23	114.02
FALSE	High	MVFTSSADG(	1xCarbamidome	TRUE	5.98E-12	0.00611	1	1	3	cRAP117	000000|rLys-C rLys	cRAP117	0	2707.23	411272		54.64	
FALSE	High	NDEELNKLLGR		FALSE	0.003147	0.00611	1	3	1	Q93077	Histone H2A type 1	Q7L7L0; P	1	1300.69	45002	7031	40.38	8.16
FALSE	High	NDYFSDFSGVYSQISR		TRUE	2.91E-11	0.00611	1	1	5	cRAP117	000000|rLys-C rLys	cRAP117	0	1884.84	3432455		22.51	
FALSE	High	NGLSLAALKKALAAGGYDVEK		FALSE	0.13847	0.00611	1	1	1	P16401	Histone H1.5 OS=H	P16401	2	2089.17	25225	39174		46.42
FALSE	High	SLDLDSIIAEVK		TRUE	0.083977	0.00611	1	1	1	P04264	Keratin, type II cytc	P04264	0	1302.72	162864		47.73	
FALSE	High	NGLSLAALKK	3xLabel:13C(6) [	FALSE	0.096164	0.00611	1	1	1	P16401	Histone H1.5 OS=H	P16401	2	2107.23				
FALSE	High	NLKVPSGQN	2xLabel:13C(6) [	FALSE	0.114095	0.00611	1	1	1	Q9GZZ1	N-alpha-acetyltran	Q9GZZ1	2	1839.96				
FALSE	High	NTDGSTDYGILQINSR		TRUE	2.70E-09	0.00611	1	1	2	cRAP078	Lysozyme C OS=Ga	cRAP078	0	1753.84	427205		23.23	
FALSE	High	NVQALEIELQSQLALK		TRUE	0.002624	0.00611	1	1	1	P13645	Keratin, type I cyto	P13645	0	1797.01	33289		39.64	
FALSE	High	QVHPDTGISSK		FALSE	0.009242	0.00611	2	15	1	Q16778; (	Histone H2B type 2	Q16778; (	0	1168.6	421372	73655	76.8	88.69
FALSE	High	RDTLLLELK		TRUE	6.26E-06	0.00611	1	1	2	cRAP117	000000|rLys-C rLys	cRAP117	1	1100.67	3677032		22.54	
FALSE	High	RDTLLLELKR		TRUE	4.85E-06	0.00611	1	1	3	cRAP117	000000|rLys-C rLys	cRAP117	2	1256.77	3171991		82.52	
FALSE	High	RGDLPFVVPR		FALSE	0.002059	0.00611	1	1	2	P35579	Myosin-9 OS=Hom	P35579	1	1155.66	620731		34.31	
FALSE	High	NLCNIPCSALL	3xCarbamidome	TRUE	1.59E-06	0.00611	1	1	2	cRAP078	Lysozyme C OS=Ga	cRAP078	0	2508.19	592271	20274	22.66	
FALSE	High	SISISVAR		TRUE	0.066321	0.00611	1	1	1	P04264	Keratin, type II cytc	P04264	0	832.489	1539474		111.45	
FALSE	High	SLYNLGGSK		FALSE	0.080761	0.00611	1	1	1	P13647	Keratin, type II cytc	P13647	0	938.494	168494		53.93	
FALSE	High	SPAQILQWQVLSNTVPAK		TRUE	1.87E-10	0.00611	1	1	1	cRAP016	Kappa-casein OS=B	cRAP016	0	1980.09	171891		43.68	

FALSE	High	VLKQVHPDT(	2xLabel:13C(6) [	FALSE	0.010964	0.00611	2	15	1	Q16778; (	Histone H2B type 2	Q16778; (	1	1520.88	170994	56026	91.96	4.55
FALSE	High	VTIAQGGVLPNIQAVLLPK		FALSE	5.61E-08	0.00611	2	10	3	Q93077; (	Histone H2A type 1	Q16777; (	0	1931.17	1876839	275157	72.29	73.99
FALSE	High	VTIAQGGVLP	1xLabel:13C(6) [	FALSE	2.57E-07	0.00611	2	10	1	Q93077; (	Histone H2A type 1	Q16777; (	0	1937.19				
FALSE	High	VTIAQGGVLPNIQAVLLPKKTESI		FALSE	0.128731	0.00611	2	8	1	Q93077; (	Histone H2A type 1	Q7L7L0; P	2	2778.6	135344	54964	45.4	
FALSE	High	VTIAQGGVLP	3xLabel:13C(6) [	FALSE	0.107824	0.00611	2	8	1	Q93077; (	Histone H2A type 1	Q7L7L0; P	2	2796.66				
FALSE	High	VTIMPK		FALSE	0.042787	0.00611	1	5	1	P84243	Histone H3.3 OS=H	P84243; C	0	688.406	826033	202446	77.97	66.85
FALSE	High	VTIMPK	1xLabel:13C(6) [	FALSE	0.030163	0.00611	1	5	1	P84243	Histone H3.3 OS=H	P84243; C	0	694.426				
FALSE	High	VHRLLR		FALSE	0.066981	0.00611	2	12	1	Q93077; (	Histone H2A type 1	Q16777; (	1	793.516	93738	41516	81.88	13.67
FALSE	High	VTIMPKDIQLAR		FALSE	0.00069	0.00611	1	5	3	P84243	Histone H3.3 OS=H	P84243; C	1	1384.8	202584	65700	96.66	43.75
FALSE	High	YADLTEDQLP	1xCarbamidome	FALSE	1.46E-05	0.00611	1	1	1	P18669	Phosphoglycerate ı	P18669	1	2437.2	17391	162157		35.54
FALSE	High	YALYDATYETK		FALSE	2.04E-06	0.00611	1	2	1	P23528	Cofilin-1 OS=Homo	P23528; C	0	1337.63	98475	41837	24	54.1
FALSE	High	YALYDATYETI	1xLabel:13C(6) [	FALSE	0.001431	0.00611	1	2	1	P23528	Cofilin-1 OS=Homo	P23528; C	0	1343.65				
FALSE	High	YEKDIAAYR		FALSE	0.001089	0.00611	1	2	2	P26583	High mobility grou	P26583; P	1	1128.57	2575152	9209	43.6	11.49
FALSE	High	YGTCIYQGR	1xCarbamidome	FALSE	0.000213	0.00611	1	2	1	P59665	Neutrophil defensi	P59665; P	0	1117.51	44839		26.52	
FALSE	High	YQKSTELLIR		FALSE	0.002678	0.00611	1	5	1	P84243	Histone H3.3 OS=H	P84243; C	1	1250.71	71446	6769		
FALSE	High	YRPGTVALR	2xLabel:13C(6) [	FALSE	0.061871	0.00611	1	5	1	P84243	Histone H3.3 OS=H	P84243; C	0	1044.64	1675282	355079	70.98	75.59
FALSE	High	WWCNDGR	1xCarbamidome	TRUE	0.009525	0.00611	1	1	1	cRAP078	Lysozyme C OS=Ga	cRAP078	0	993.4	142655		21.41	
FALSE	High	LYAEERYPILPI	1xCarbamidome	TRUE	1.20E-05	0.00611	1	1	1	cRAP086	Ovalbumin OS=Gal	cRAP086	2	2845.46	17528		29.1	
FALSE	High	VHPAVVIR		FALSE	0.010854	0.00611	1	1	1	P62829	60S ribosomal prot	P62829	0	890.557	288681	17205	39.81	69.85
FALSE	High	VCNYVNWIQ	1xCarbamidome	TRUE	0.010532	0.00611	1	1	1	cRAP112	Trypsin OS=Sus scr	cRAP112	0	1793.86				
FALSE	High	SRGSGGLGG/	1xCarbamidome	FALSE	4.96E-08	0.00611	1	3	1	P04259	Keratin, type II cyt	P04259; P	1	1667.77	120815		32.87	
FALSE	High	SSLLEKGLDGAKK		FALSE	0.001004	0.00611	1	1	1	P81605	Dermcidin OS=Hon	P81605	2	1345.77	123742		55.41	
FALSE	High	STELLIR	1xLabel:13C(6) [	FALSE	0.034692	0.00611	1	5	1	P84243	Histone H3.3 OS=H	P84243; C	0	837.514	3621997	803670	54.88	79.89
FALSE	High	STELLIRK		FALSE	0.087309	0.00611	1	5	1	P84243	Histone H3.3 OS=H	P84243; C	1	959.588	25943	4394	80.73	23.81
FALSE	High	STITSREIQTAVR		FALSE	0.124073	0.00611	1	13	1	Q16778	Histone H2B type 2	Q16778; (	1	1461.8	2076987	654985	62.78	62.86
FALSE	High	STITSREIQTA'	2xLabel:13C(6) [	FALSE	8.77E-06	0.00611	1	13	3	Q16778	Histone H2B type 2	Q16778; (	1	1473.84				
FALSE	High	STITSREIQTA'	3xLabel:13C(6) [	FALSE	0.003698	0.00611	1	13	2	Q16778	Histone H2B type 2	Q16778; (	2	2414.45	281015	58976	69.17	74.14
FALSE	High	VERADGYEPPVQESV		FALSE	0.000125	0.00611	1	1	1	P61247	40S ribosomal prot	P61247	1	1674.8	158979	98215	33.24	69.34
FALSE	High	SYELPDGQVITIGNER		FALSE	5.84E-05	0.00611	1	10	1	A5A3E0	POTE ankyrin dom	A5A3E0; F	0	1790.89	94996	25392	23.83	8.16
FALSE	High	TPPAGVFYQGWSATPIANGSLG		TRUE	0.012121	0.00611	1	1	1	cRAP117	000000|rLys-C rLys	cRAP117	0	3083.52	798439		63.89	
FALSE	High	TPPAGVFYQGWSATPIANGSLG		TRUE	0.096164	0.00611	1	1	1	cRAP117	000000|rLys-C rLys	cRAP117	1	3454.7	59012		82.9	
FALSE	High	TPPAGVFYQGWSATPIANGSLG		TRUE	1.24E-05	0.00611	1	1	1	cRAP117	000000|rLys-C rLys	cRAP117	2	3582.8	323791		106.6	
FALSE	High	TTHFVEGGDAGNREDQINR		FALSE	0.000368	0.00611	1	1	1	P18124	60S ribosomal prot	P18124	1	2115.98	47278	35804	20.48	71.36
FALSE	High	TVTAMDVVYALK		FALSE	0.001111	0.00611	1	1	1	P62805	Histone H4 OS=Hoı	P62805	0	1310.7	169141	52642	77.63	66.48
FALSE	High	TVTAMDVVY.	1xLabel:13C(6) [	FALSE	0.000677	0.00611	1	1	1	P62805	Histone H4 OS=Hoı	P62805	0	1316.72				
FALSE	High	VATVSLPR		TRUE	0.082354	0.00611	1	1	1	cRAP112	Trypsin OS=Sus scr	cRAP112	0	842.509	50894856		35.77	
FALSE	High	SYSKLLCGLLA	1xCarbamidome	FALSE	2.99E-07	0.00611	1	1	1	P14174	Macrophage migra	P14174	1	1521.85	27840	35496	42.14	70.17
FALSE	High	LVNELTEFAK		TRUE	0.00409	0.00611	1	1	1	cRAP087	Serum albumin OS	cRAP087	0	1163.63	31826		69.7	
FALSE	High	LSFSLKTQD	1xLabel:13C(6) [	FALSE	0.052759	0.00611	1	1	1	Q9NWB6	Arginine and glutar	Q9NWB6	1	1044.57	19196	22770	20.96	75.42
FALSE	High	LSAKPAPPKPEPRPK		FALSE	0.000499	0.00611	1	1	1	O00479	High mobility grou	O00479	0	1612.95	62002	4151	140.71	83.45
FALSE	High	DGFGASGSCE	2xCarbamidome	TRUE	0.165314	0.00611	1	1	1	cRAP117	000000|rLys-C rLys	cRAP117	0	2231.93	104171		16.53	
FALSE	High	DGFGASGSCE	2xCarbamidome	TRUE	3.33E-08	0.00611	1	1	1	cRAP117	000000|rLys-C rLys	cRAP117	1	3307.46	66696		34.17	

FALSE	High	DNIQGITKPAIR		FALSE	0.000863	0.00611	1	1	1	P62805	Histone H4 OS=Hor	P62805	0	1325.75	245847	49643	69.35	59.16
FALSE	High	EIQTAVR		FALSE	0.051717	0.00611	1	14	1	Q16778	Histone H2B type 2	Q16778; (	0	816.457	264670	60205	73.74	84.52
FALSE	High	EIQTAVRLLLPGELAK		FALSE	0.044976	0.00611	1	14	1	Q16778	Histone H2B type 2	Q16778; (	1	1751.04	2098210	754029	78.99	56.11
FALSE	High	EIQTAVRLLLP	2xLabel:13C(6) [	FALSE	0.073209	0.00611	1	14	1	Q16778	Histone H2B type 2	Q16778; (	1	1763.08				
FALSE	High	ENAGEDPGLAR		FALSE	0.02898	0.00611	1	1	1	P81605	Dermcidin OS=Hon	P81605	0	1128.53	40932		32.07	
FALSE	High	DAVEDLESVGK		FALSE	0.000142	0.00611	1	1	1	P81605	Dermcidin OS=Hon	P81605	0	1161.56	38710		101.75	
FALSE	High	EVQTAVRLLLI	2xLabel:13C(6) [	FALSE	0.00177	0.00611	1	1	1	Q8N257	Histone H2B type 3	Q8N257	1	1749.07	65949	42605	51.38	77.59
FALSE	High	GFGRGGAESHTFK		FALSE	0.031081	0.00611	1	1	1	P50238	Cysteine-rich prote	P50238	1	1350.65	37316		38.82	
FALSE	High	GGGGGGYGSGGSSYGSGGGSY(		TRUE	1.27E-18	0.00611	1	1	2	P04264	Keratin, type II cytc	P04264	0	2383.95	34101		139.96	
FALSE	High	GGLEPINFQTAADQAR		TRUE	2.97E-06	0.00611	1	1	2	cRAP086	Ovalbumin OS=Gal	cRAP086	0	1687.84	45369		18.17	
FALSE	High	GGLYGGPSYC	1xCarbamidome	TRUE	3.46E-06	0.00611	1	1	2	cRAP117	000000|rLys-C rLys	cRAP117	1	3593.6	618670		3.35	
FALSE	High	GGSGGGGSISGGGYGSGGGSG(		TRUE	1.70E-10	0.00611	1	1	1	P35908	Keratin, type II cytc	P35908	0	1741.75	12793	3521	113.58	
FALSE	High	GGSGGSHGGGSGFGGESGGSY(		TRUE	6.41E-23	0.00611	1	1	3	P35527	Keratin, type I cyto	P35527	0	3223.28	57045	6738	90.48	
FALSE	High	GGSGGSHGGGSGFGGESGGSY(		TRUE	1.29E-06	0.00611	1	1	1	P35527	Keratin, type I cyto	P35527	1	3621.44	8383		116.99	
FALSE	High	FADLSEAANR		FALSE	0.000917	0.00611	1	1	1	P08670	Vimentin OS=Hom(	P08670	0	1093.53	60621		13.86	
FALSE	High	GIVFGR		FALSE	0.066981	0.00611	1	1	1	Q96S38	Ribosomal protein	Q96S38	0	648.383	65340		16.83	
FALSE	High	DAEAWFNEK		TRUE	0.006244	0.00611	1	4	1	P13645	Keratin, type I cyto	Q7Z3Y7; F	0	1109.49	47855		80.13	
FALSE	High	AYDNATAAVAK		TRUE	9.24E-05	0.00611	1	1	1	cRAP117	000000|rLys-C rLys	cRAP117	0	1094.55	30425970		20.41	
FALSE	High	AAFGGSGGRGSSSGGGYSSGSS(		TRUE	1.40E-10	0.00611	1	1	1	P35908	Keratin, type II cytc	P35908	1	2501.07	171962		88.8	
FALSE	High	AAFGGSGGRGSSSGGGYSSGSS(		TRUE	3.23E-06	0.00611	1	1	1	P35908	Keratin, type II cytc	P35908	2	3016.31	42101		37.86	
FALSE	High	AGLQFPVGR		FALSE	0.001388	0.00611	3	15	2	Q71UI9; C	Histone H2A.V OS=	Q16777; (	0	944.531	4912568	1455481	74.8	68.58
FALSE	High	AGYRDGFGAS	2xCarbamidome	TRUE	8.79E-08	0.00611	1	1	4	cRAP117	000000|rLys-C rLys	cRAP117	1	2679.15	14686726		27.74	
FALSE	High	AGYRDGFGAS	2xCarbamidome	TRUE	9.57E-12	0.00611	1	1	6	cRAP117	000000|rLys-C rLys	cRAP117	2	3754.68	18102221	7324	52.08	82.86
FALSE	High	ALAAGGYDVE	2xLabel:13C(6) [	FALSE	5.22E-05	0.00611	1	1	1	P16401	Histone H1.5 OS=H	P16401	1	1576.81	11111	30237		59.53
FALSE	High	ALCSEKLDQW	2xCarbamidome	TRUE	0.098971	0.00611	1	1	1	cRAP075	Alpha-lactalbumin	cRAP075	2	1892.92	69210		59.63	
FALSE	High	CCTKPESERM	3xCarbamidome	TRUE	0.005262	0.00611	1	1	1	cRAP087	Serum albumin OS=	cRAP087	1	2888.3	46044		47.03	
FALSE	High	ALKAWSVAR		TRUE	0.146147	0.00611	1	1	1	cRAP087	Serum albumin OS=	cRAP087	1	1001.59	116825		74.14	
FALSE	High	AMGIMNSFV	1xOxidation [M2	FALSE	1.04E-08	0.00611	2	14	1	Q16778; (	Histone H2B type 2	Q16778; (	0	1759.81	302034	101632	48.02	70.09
FALSE	High	AMGIMNSFV	2xOxidation [M2	FALSE	2.11E-05	0.00611	2	14	1	Q16778; (	Histone H2B type 2	Q16778; (	0	1775.81	329370	196940	76.15	63.79
FALSE	High	AMGIMNSFV	2xOxidation [M2	FALSE	1.54E-07	0.00611	2	14	2	Q16778; (	Histone H2B type 2	Q16778; (	0	1781.83				
FALSE	High	ASGPPVSELIT	1xLabel:13C(6) [	FALSE	0.001616	0.00611	1	3	2	P10412	Histone H1.4 OS=H	P16402; P	0	1204.69	420934	162170	86.68	69.82
FALSE	High	ATGPPVSELIT	1xLabel:13C(6) [	FALSE	0.009525	0.00611	1	1	1	P16401	Histone H1.5 OS=H	P16401	0	1218.7	79874	158533	27.18	70.82
FALSE	High	AVFVDLEPTV	1xLabel:13C(6) [	FALSE	0.000313	0.00611	1	3	1	Q71U36	Tubulin alpha-1A cl	Q71U36; I	0	1707.93	10377	71935		55.31
FALSE	High	AVKPKAAKPK		FALSE	0.000587	0.00611	2	3	1	P16401; P	Histone H1.5 OS=H	P16401; P	1	1037.68	58700	32148	56.31	65.73
FALSE	High	AMGIMNSFV	1xLabel:13C(6) [	FALSE	3.03E-06	0.00611	2	14	1	Q16778; (	Histone H2B type 2	Q16778; (	0	1749.84	272596	105449	65.04	52.41
FALSE	High	YRPGTVALREIR		FALSE	0.002992	0.00611	1	5	2	P84243	Histone H3.3 OS=H	P84243; C	1	1430.82	843531	151115	37.75	11.24
FALSE	High	GSCGIGGGIG	1xCarbamidome	FALSE	1.75E-06	0.00611	1	2	1	P02533	Keratin, type I cyto	P02533; P	0	1278.59	103476		51.84	
FALSE	High	GSRGGSGGSYGGGGSGGGYGG		TRUE	5.20E-21	0.00611	1	1	2	P35527	Keratin, type I cyto	P35527	1	2091.88	36168		123.43	
FALSE	High	LAADEDDDDI	3xLabel:13C(6) [	FALSE	3.78E-07	0.00611	1	1	1	P06748	Nucleophosmin OS	P06748	2	4264.61	6718	40173		64.78
FALSE	High	LAHYNKR		FALSE	0.054361	0.00611	2	15	1	Q16778; (	Histone H2B type 2	Q16778; (	1	901.5	81313		17.13	
FALSE	High	LAPDYDALDVANKIGII		FALSE	9.10E-07	0.00611	1	1	2	P62750	60S ribosomal prot	P62750	1	1800.97	188194	129452	43.68	16.53
FALSE	High	LGKDAVEDLESVGK		FALSE	7.77E-05	0.00611	1	1	1	P81605	Dermcidin OS=Hon	P81605	1	1459.76	120207		62.92	

FALSE	High	IGRIEDVTPIPS	3xLabel:13C(6) [	FALSE	0.027021	0.00611	1	1	1	P62263	40S ribosomal prot	P62263	2	1930.09				
FALSE	High	LGTRLVPAERR		FALSE	0.004049	0.00611	1	1	1	Q6NZI2	Caveolae-associate	Q6NZI2	2	1267.76	293029		40.3	
FALSE	High	IHRHLK		FALSE	0.032349	0.00611	1	2	1	Q71UI9	Histone H2A.V OS=	Q71UI9; P	1	803.5	68968	3592	25.87	
FALSE	High	KYSQGNVSAVGVTYDGHTALTR		TRUE	3.77E-15	0.00611	1	1	1	cRAP117	000000|rLys-C rLys	cRAP117	1	2324.16	1341189		3.45	
FALSE	High	LKEKYEK		FALSE	0.063742	0.00611	1	4	2	P26583	High mobility grou	O15347; F	2	937.535	77331		67	
FALSE	High	LLDFGSLSNLQVTQPTVGMNFK		FALSE	0.003661	0.00611	1	1	1	P08708	40S ribosomal prot	P08708	1	2763.45	37120	21571	70.84	27.17
FALSE	High	LLLPGELAK		FALSE	0.05887	0.00611	2	15	1	Q16778; (	Histone H2B type 2	Q16778; (	0	953.603	3932073	1443102	83.04	53.01
FALSE	High	LLQDSVDFSLADAINTEFKNTR		FALSE	0.054905	0.00611	1	1	1	P08670	Vimentin OS=Hom	P08670	1	2497.26	43181		28.3	
FALSE	High	LLRKGNYAER		FALSE	0.009719	0.00611	2	9	1	Q93077; (	Histone H2A type 1	Q16777; (	2	1219.69	106586	29222	78.1	76.8
FALSE	High	IITHPNFNGNTLDNDIMLIK		TRUE	4.31E-10	0.00611	1	1	2	cRAP112	Trypsin OS=Sus scr	cRAP112	0	2283.18	8038655		45.9	
FALSE	High	IITHPNFNGNTLDNDIMLIKLSSP		TRUE	3.95E-11	0.00611	1	1	2	cRAP112	Trypsin OS=Sus scr	cRAP112	1	3309.73	1063314	4921	51.71	23.38
FALSE	High	IMLKGDNITLI	1xLabel:13C(6) [	FALSE	0.007045	0.00611	1	1	1	P62304	Small nuclear ribor	P62304	1	1751.97	30625	148919	63.17	41.61
FALSE	High	LLCGLLAER	1xCarbamidome	FALSE	0.056011	0.00611	1	1	1	P14174	Macrophage migra	P14174	0	1050.61	19930	66363	22.99	44.7
FALSE	High	GSGSGQSPSSGQHGTGFGR		FALSE	0.053822	0.00611	1	1	1	Q86YZ3	Hornerin OS=Hom	Q86YZ3	0	1747.77	30851		34.11	
FALSE	High	KVPQVSTPTLVEVSR		TRUE	4.44E-05	0.00611	1	2	1	cRAP087	Serum albumin OS	cRAP087;	1	1639.94	110391		47.9	
FALSE	High	KLLEAQSHFR		FALSE	0.056571	0.00611	1	1	1	Q13813	Spectrin alpha chai	Q13813	1	1228.68	81624		36.9	
FALSE	High	GTDVQAWIR		TRUE	0.000174	0.00611	1	1	1	cRAP078	Lysozyme C OS=Ga	cRAP078	0	1045.54	160739		8.04	
FALSE	High	GTGASGSFKLNK		FALSE	0.005315	0.00611	2	5	1	P16401; P	Histone H1.5 OS=H	P16401; P	1	1166.62				
FALSE	High	GTGASGSFKL	2xLabel:13C(6) [	FALSE	0.003889	0.00611	2	5	1	P16401; P	Histone H1.5 OS=H	P16401; P	1	1178.66	99611	117865	103.45	75.73
FALSE	High	GYSLGNWVC	1xCarbamidome	TRUE	0.005478	0.00611	1	1	1	cRAP078	Lysozyme C OS=Ga	cRAP078	0	1325.63	98455		15.13	
FALSE	High	HAVSEGTKAVTK		FALSE	5.31E-09	0.00611	2	15	3	Q16778; (	Histone H2B type 2	Q16778; (	1	1227.67	2305143	490877	47.63	82.86
FALSE	High	HAVSEGTKAV	2xLabel:13C(6) [	FALSE	2.95E-08	0.00611	2	15	2	Q16778; (	Histone H2B type 2	Q16778; (	1	1239.71				
FALSE	High	HAVSEGTKAVTKYTSSK		FALSE	8.36E-10	0.00611	2	12	4	Q16778; (	Histone H2B type 2	Q16778; (	2	1793.94	665761	332061	111.4	72.58
FALSE	High	KIVITDCGQLS	1xCarbamidome	FALSE	0.00123	0.00611	1	1	1	P30405	Peptidyl-prolyl cis-t	P30405	1	1239.67				
FALSE	High	HAVSEGTKAV	3xLabel:13C(6) [	FALSE	1.57E-08	0.00611	2	12	3	Q16778; (	Histone H2B type 2	Q16778; (	2	1812				
FALSE	High	HGLDNYRGYS	1xCarbamidome	TRUE	0.061259	0.00611	1	1	1	cRAP078	Lysozyme C OS=Ga	cRAP078	1	2181.03	77169		19.17	
FALSE	High	HGSSSGSSSHYGQHGSGSR		FALSE	1.50E-09	0.00611	1	1	1	Q86YZ3	Hornerin OS=Hom	Q86YZ3	0	1858.78	6877		118.57	
FALSE	High	HGVQELEIELQSQLSK		TRUE	0.000387	0.00611	1	1	1	P35527	Keratin, type I cyto	P35527	0	1837.97	23375		59.84	
FALSE	High	HLQLAIRNDE	3xLabel:13C(6) [	FALSE	0.099923	0.00611	1	7	1	Q99878	Histone H2A type 1	Q16777; (	2	2122.25	81231	25551	85.57	61.36
FALSE	High	KALAAAGYDVEKNNSR		FALSE	0.000111	0.00611	1	5	1	P10412	Histone H1.4 OS=H	P16402; P	2	1706.88	61591	30305	48	21.76
FALSE	High	KATGPPVSELI	2xLabel:13C(6) [	FALSE	2.53E-05	0.00611	1	1	1	P16401	Histone H1.5 OS=H	P16401	1	1352.82	161972	270347	54.49	68.97
FALSE	High	KIEEIKDFLLTAR		FALSE	0.000541	0.00611	1	1	1	P63173	60S ribosomal prot	P63173	2	1575.91	89404	5795	46.79	
FALSE	High	HGGGGGGFGGGGFGSR		TRUE	6.78E-09	0.00611	1	1	1	P35908	Keratin, type II cyto	P35908	0	1320.58	67433		33.15	
FALSE	High	YSQGNVSAVGVTYDGHTALTR		TRUE	3.66E-10	0.00611	1	1	2	cRAP117	000000|rLys-C rLys	cRAP117	0	2196.07	2464041		22	

Abundance	Abundance	Abundance	Abundan	Abundanc	Abundanc	Precur	Quan In	Quan Ch	Found in S	Found in	Found in S	Found in S	Found in S	Found in S	Confide	Percolator	Percolator	XCorr by	Top Apex RT
							Indistinguishabl		Not Found	Not Fou	Not Found	Not Found	Not Found	Not Found	High	0.00952	0.01364	4.38	136.14
30200	91035	88151	54815	149552	259609	94		Heavy	Peak Four	Peak Fo	Peak Four	Peak Four	Peak Four	High	High	0.00952	0.01649	3.59	80.38
1299181	2264745	1373099				95		Light	Peak Four	High	High	Not Found	Not Found	Not Found	High	0.00848	0.01894	3.33	58.52
40257	62351	36133				96		Light	Peak Four	High	Peak Four	Not Found	Not Found	Not Found	High	0.00848	0.1143	2.28	41.32
4750	12943	6257	23832	43905	37414	97		Heavy	Peak Four	Peak Fo	Peak Four	Peak Four	Peak Four	High	High	0.00952	0.09798	2.97	51.15
21417	130767	88702		28704	47036	98		Light	Peak Four	High	Peak Four	Not Found	Peak Four	Peak Four	High	0.00848	0.0771	3.22	69.85
32720126	28231713	9967951				99		Light	Peak Four	High	High	Not Found	Not Found	Not Found	High	0.00848	0.00422	5.09	130.69
281172	1901984	445451	73502	315327	159922	100		Light	Peak Four	High	High	Peak Four	Peak Four	Peak Four	High	0.00848	0.01065	3.84	108.86
34395	41504	43374				101		Light	Peak Four	High	Peak Four	Not Found	Not Found	Not Found	High	0.00848	0.03013	3.44	48.99
48942	83319	72233	30620	75404	134645	102		Heavy	Peak Four	Peak Fo	Peak Four	Peak Four	High	High	High	0.00848	0.02152	3.2	80.29
106441	78666	49811				103		Light	Peak Four	Peak Fo	High	Not Found	Not Found	Not Found	High	0.00952	0.06933	3.4	49.95
11112	28247	27486			23315	104		Light	Peak Four	Peak Fo	High	Not Found	Not Found	Peak Four	High	0.00952	0.2333	3.13	189.58
						105	NoQua	Light	Not Found	Not Fou	High	Not Found	Not Found	Not Found	High	0.00952	0.01552	4.52	
44714	44797	49957				106		Light	Peak Four	High	High	Not Found	Not Found	Not Found	High	0.00952	0.00047	5.98	57.34
482257	569856	1332458				107		Light	Peak Four	Peak Fo	High	Not Found	Not Found	Not Found	High	0.00952	0.01984	3.7	57.76
439621	242114	380324				108		Light	Peak Four	High	Peak Four	Not Found	Not Found	Not Found	High	0.00848	0.00485	4.96	98.15
						94	Redund	Light	Peak Four	Peak Fo	High	Peak Four	Peak Four	Peak Four	High	0.00952	0.01967	3.7	80.38
78778	323331	217116	23908	63159	115896	93		Light	Peak Four	High	Peak Four	Peak Four	Peak Four	Peak Four	High	0.00848	0.01375	4.38	77.81
						92	NoQua	Light	Not Found	High	Not Found	Not Found	Not Found	Not Found	High	0.00848	0.01342	3.33	
168532	152399	103429				91		Light	Peak Four	Peak Fo	High	Not Found	Not Found	Not Found	High	0.00952	0.04101	3.52	100.83
122273	337022	209453		17994		76		Light	Peak Four	High	Peak Four	Not Found	Peak Four	Not Found	High	0.00848	0.1231	1.41	64.05
8420		15165		15248	45240	77		Heavy	Peak Four	Not Fou	Peak Four	Not Found	Peak Four	High	High	0.00952	0.1034	3.44	215.93
9415710	7682254	5860139	18463	10879	98717	78		Light	Peak Four	High	High	Peak Four	Peak Four	Peak Four	High	0.00952	7.46E-06	8.2	167.65
438605	411272	119939				79		Light	Peak Four	High	Peak Four	Not Found	Not Found	Not Found	High	0.00848	6.61E-05	8.45	156.06
	60364	33550		6636	7449	80		Light	Not Found	High	Peak Four	Not Found	Peak Four	Peak Four	High	0.00848	0.04083	2.9	121.18
3432455	3436577	2249297				81		Light	Peak Four	High	High	Not Found	Not Found	Not Found	High	0.00848	0.00104	4.92	190.1
	25225			55084	27860	82		Light	Not Found	High	Not Found	Not Found	Peak Four	Peak Four	High	0.00848	0.1361	3.11	167.75
212470	72087	162864				109		Light	Peak Four	High	Peak Four	Not Found	Not Found	Not Found	High	0.00848	0.1156	3.02	199.07
						82	Redund	Heavy	Not Found	Peak Fo	Not Found	Not Found	High	Peak Four	High	0.00848	0.121	2.52	167.75
						84	NoQua	Heavy	Not Found	Not Fou	Not Found	Not Found	Not Found	High	High	0.00952	0.2201	2.73	
435063	427205	278319				85		Light	Peak Four	High	High	Not Found	Not Found	Not Found	High	0.00848	0.00112	5.39	139.58
33289	35398	15226				86		Light	Peak Four	High	Peak Four	Not Found	Not Found	Not Found	High	0.00848	0.03851	3.3	194.76
35381	458288	421372	10430	73655	146867	87	NotUni	Light	Peak Four	High	Peak Four	Peak Four	Peak Four	Peak Four	High	0.00848	0.0576	1.75	40.68
3999452	3677032	2538715				88		Light	Peak Four	Peak Fo	High	Not Found	Not Found	Not Found	High	0.00952	0.02373	3.44	123.2
8952939	3171991	1747931				89		Light	Peak Four	High	High	Not Found	Not Found	Not Found	High	0.00848	0.00736	3.77	102.8
807565	620731	391961				90		Light	Peak Four	High	Peak Four	Not Found	Not Found	Not Found	High	0.00848	0.03564	2.98	122.73
509054	786892	592271			20274	83		Light	Peak Four	High	High	Not Found	Not Found	Peak Four	High	0.00848	0.00408	5.31	181.27
1539474	1078827	8365539				110		Light	Peak Four	Peak Fo	High	Not Found	Not Found	Not Found	High	0.00952	0.1956	1.86	90.19
168494	80287	264910				111		Light	Peak Four	Peak Fo	High	Not Found	Not Found	Not Found	High	0.00952	0.204	2.62	89.86
335326	171891	161784				112		Light	Peak Four	Peak Fo	High	Not Found	Not Found	Not Found	High	0.00952	0.00208	5	186.22

17876	344912	170994		54252	57859	130	NotUni	Heavy	Peak Four	Peak Fo	Peak Four	Not Founc	Peak Four	High	High	0.00952	0.1315	2.83	56.22
282511	2372299	1876839	52516	275157	416725	131	NotUni	Light	Peak Four	High	High	Peak Four	Peak Four	Peak Four	High	0.00952	0.00788	3.65	207.66
						131	Redunc	Heavy	Peak Four	Peak Fo	Peak Four	Peak Four	Peak Four	High	High	0.00952	0.01127	4.24	207.65
76720	135344	201955			54964	132	NotUni	Light	Peak Four	Peak Fo	High	Not Founc	Not Founc	Peak Four	High	0.00952	0.2263	1.43	146.3
						132	Redunc	Heavy	Peak Four	Peak Fo	Peak Four	Not Founc	Not Founc	High	High	0.00952	0.2175	1.79	146.3
179013	1460574	826033	42808	202446	260362	133		Light	Peak Four	High	Peak Four	Peak Four	Peak Four	Peak Four	High	0.00848	0.09367	2.05	71.22
						133	Redunc	Heavy	Peak Four	Peak Fo	Peak Four	Peak Four	High	Peak Four	High	0.00848	0.08381	1.88	71.22
35279	223049	93738		45743	37680	129	NotUni	Light	Peak Four	High	Peak Four	Not Founc	Peak Four	Peak Four	High	0.00848	0.1078	1.37	33.39
160603	867308	202584	56932	122857	65700	134		Light	Peak Four	High	High	Peak Four	Peak Four	Peak Four	High	0.00848	0.04168	2.82	123.79
		17391	84931	162157	180258	136		Heavy	Not Founc	Not Fou	Peak Four	Peak Four	Peak Four	High	High	0.00952	0.02887	2.99	150.51
86340	135545	98475	17978	41837	61766	137		Light	Peak Four	Peak Fo	High	Peak Four	Peak Four	Peak Four	High	0.00952	0.01824	3.19	117.29
						137	Redunc	Heavy	Peak Four	Peak Fo	Peak Four	Peak Four	Peak Four	High	High	0.00952	0.08292	2.96	117.29
1084485	2831901	2575152		8489	9990	138		Light	Peak Four	High	High	Not Founc	Peak Four	Peak Four	High	0.00848	0.04802	3.27	66.18
54114	31231	44839				139		Light	Peak Four	Peak Fo	High	Not Founc	Not Founc	Not Founc	High	0.00952	0.05365	3.11	80.1
	71446			6769		140		Light	Not Founc	High	Not Founc	Not Founc	Peak Four	Not Founc	High	0.00848	0.03872	2.6	84.53
496332	2892928	1675282	38296	355079	426217	141		Heavy	Peak Four	Peak Fo	Peak Four	Peak Four	High	Peak Four	High	0.00848	0.105	2.86	67.5
124547	142655	187582				135		Light	Peak Four	High	Peak Four	Not Founc	Not Founc	Not Founc	High	0.00848	0.05811	2.25	90.22
17528	22263	12186				75		Light	Peak Four	High	Peak Four	Not Founc	Not Founc	Not Founc	High	0.00848	0.00688	5.94	188.82
276048	534891	288681	2429	17900	17205	128		Light	Peak Four	High	Peak Four	Peak Four	Peak Four	Peak Four	High	0.00848	0.0607	2.61	60.46
							Indistinguishabl		Not Founc	Not Fou	Not Founc	Not Founc	Not Founc	Not Founc	High	0.00848	0.0601	3.61	218.29
120815	66204	130982				113		Light	Peak Four	Peak Fo	High	Not Founc	Not Founc	Not Founc	High	0.00952	0.00767	4.39	70.18
123742	270542	102015				114		Light	Peak Four	High	Peak Four	Not Founc	Not Founc	Not Founc	High	0.00848	0.02835	3.52	75.52
1514576	5341536	3621997	51866	803670	938390	115		Heavy	Peak Four	Peak Fo	Peak Four	Peak Four	High	Peak Four	High	0.00848	0.08756	2.37	96.25
8413	56916	25943		5209	3707	116		Light	Peak Four	High	Peak Four	Not Founc	Peak Four	Peak Four	High	0.00848	0.1172	2.33	65.78
655104	3071636	2076987	237862	654985	1053453	117		Light	Peak Four	High	Peak Four	Peak Four	Peak Four	Peak Four	High	0.00848	0.1315	2.7	83.03
						117	Redunc	Heavy	Peak Four	Peak Fo	Peak Four	Peak Four	High	High	High	0.00952	0.02565	2.93	83.03
76199	445707	281015	15207	58976	102566	118		Heavy	Peak Four	Peak Fo	Peak Four	Peak Four	High	High	High	0.00848	0.04301	2.42	194.79
88053	158979	176834	39676	98215	190031	127		Light	Peak Four	Peak Fo	High	Peak Four	Peak Four	Peak Four	High	0.00952	0.04747	2.9	110.52
114330	94996	70068	26902	23966		119		Light	Peak Four	High	Peak Four	Peak Four	Peak Four	Not Founc	High	0.00848	0.01138	3.71	164.12
1474205	798439	360974				121		Light	Peak Four	High	Peak Four	Not Founc	Not Founc	Not Founc	High	0.00848	0.06278	2.84	151.03
215379	59012	55755				122		Light	Peak Four	Peak Fo	High	Not Founc	Not Founc	Not Founc	High	0.00952	0.2117	2.53	141.96
1367592	323791	156024				123		Light	Peak Four	Peak Fo	High	Not Founc	Not Founc	Not Founc	High	0.00952	0.02775	3.31	133.89
36639	55645	47278	13437	35804	69578	124		Light	Peak Four	Peak Fo	High	Peak Four	Peak Four	Peak Four	High	0.00952	0.06081	3.6	67.59
36437	295974	169141	9972	52642	61163	125		Light	Peak Four	Peak Fo	High	Peak Four	Peak Four	Peak Four	High	0.00952	0.0783	3.49	173.94
						125	Redunc	Heavy	Peak Four	Peak Fo	Peak Four	Peak Four	Peak Four	High	High	0.00952	0.06998	3.52	173.94
79364512	50894856	40045632				126		Light	Peak Four	Peak Fo	High	Not Founc	Not Founc	Not Founc	High	0.00952	0.205	2.12	90.06
20445	27840	46228	26594	35496	93681	120		Heavy	Peak Four	Peak Fo	Peak Four	Peak Four	Peak Four	High	High	0.00952	0.01166	3.74	157.03
31826	49445	8078				74		Light	Peak Four	High	Peak Four	Not Founc	Not Founc	Not Founc	High	0.00848	0.04441	3	136.25
17230	19196	25511	18921	22770	68871	73		Heavy	Peak Four	Peak Fo	Peak Four	Peak Four	Peak Four	High	High	0.00952	0.1859	2.47	121.23
19158	560671	62002		8175	2108	72		Light	Peak Four	High	Peak Four	Not Founc	Peak Four	Peak Four	High	0.00848	0.02266	2.61	44.56
104171	114194	81868				20		Light	Peak Four	High	Peak Four	Not Founc	Not Founc	Not Founc	High	0.00848	0.1448	7.33	122.27
66696	87612	42758				21		Light	Peak Four	High	Peak Four	Not Founc	Not Founc	Not Founc	High	0.00848	0.00105	5.16	148.36

36359	245847	261209		31790	77521	22		Light	Peak Four	High	Peak Four	Not Founc	Peak Four	Peak Four	High	0.00848	0.02697	3.53	91.24
30968	302052	264670	11084	60205	117191	23		Light	Peak Four	High	Peak Four	Peak Four	Peak Four	Peak Four	High	0.00848	0.09922	1.67	49.02
802084	4756666	2098210	210735	816298	754029	24		Light	Peak Four	High	Peak Four	Peak Four	Peak Four	Peak Four	High	0.00848	0.09501	2.93	171.13
						24	Redunc	Heavy	Peak Four	Peak Fo	Peak Four	Peak Four	High	Peak Four	High	0.00848	0.1107	2.96	171.13
31265	58874	40932				25		Light	Peak Four	Peak Fo	High	Not Founc	Not Founc	Not Founc	High	0.00952	0.1631	2.95	57.95
38710	186511	32151				19		Light	Peak Four	Peak Fo	High	Not Founc	Not Founc	Not Founc	High	0.00952	0.04876	3.45	127.19
28419	91845	65949	17068	42605	95946	26		Heavy	Peak Four	Peak Fo	Peak Four	Peak Four	Peak Four	High	High	0.00952	0.08718	3.24	162.18
54886	37316	24758				28		Light	Peak Four	High	Peak Four	Not Founc	Not Founc	Not Founc	High	0.00848	0.08464	2.37	50.27
34101	7573	278643				29		Light	Peak Four	Peak Fo	High	Not Founc	Not Founc	Not Founc	High	0.00952	2.56E-05	6.96	70.1
38512	45369	55279				30		Light	Peak Four	High	High	Not Founc	Not Founc	Not Founc	High	0.00848	0.00621	4.31	143.88
618670	628580	589151				31		Light	Peak Four	High	High	Not Founc	Not Founc	Not Founc	High	0.00952	0.02066	4.59	185.88
12793	12536	85046			3521	32		Light	Peak Four	Peak Fo	High	Not Founc	Not Founc	Peak Four	High	0.00952	0.00204	5.36	54.28
51621	57045	232465			6738	33		Light	Peak Four	Peak Fo	High	Not Founc	Not Founc	Peak Four	High	0.00952	2.53E-06	9.57	76.99
5561	8383	50286				34		Light	Peak Four	Peak Fo	High	Not Founc	Not Founc	Not Founc	High	0.00952	0.01641	5.02	68.46
	54943	66886				27		Light	Not Founc	High	Peak Four	Not Founc	Not Founc	Not Founc	High	0.00848	0.02754	2.53	91.09
49605	69050	65340				35		Light	Peak Four	High	Peak Four	Not Founc	Not Founc	Not Founc	High	0.00848	0.1078	1.73	62.66
156517	47855	39743				18		Light	Peak Four	High	Peak Four	Not Founc	Not Founc	Not Founc	High	0.00848	0.0508	2.6	127.74
30425970	37655656	25032102				16		Light	Peak Four	High	Peak Four	Not Founc	Not Founc	Not Founc	High	0.00848	0.01319	3.43	62.26
171962	81458	510343				1		Light	Peak Four	High	Peak Four	Not Founc	Not Founc	Not Founc	High	0.00848	0.00018	6.68	68.21
42101	24173	54488				2		Light	Peak Four	Peak Fo	High	Not Founc	Not Founc	Not Founc	High	0.00952	0.02031	4.85	58.71
1384357	9011246	4912568	240201	1455481	1656935	3	NotUni	Light	Peak Four	High	High	Peak Four	Peak Four	Peak Four	High	0.00848	0.03141	2.53	122.19
14686726	16488444	9296027				4		Light	Peak Four	High	High	Not Founc	Not Founc	Not Founc	High	0.00848	0.00143	5.51	113.06
18102221	19988990	5914890		14332	3743	5		Light	Peak Four	High	High	Not Founc	Peak Four	Peak Four	High	0.00848	0.0001	7.03	138.36
		11111		19302	47367	6		Heavy	Not Founc	Not Fou	Peak Four	Not Founc	Peak Four	High	High	0.00952	0.03873	4.07	68.53
163264	58135	69210				7		Light	Peak Four	High	Peak Four	Not Founc	Not Founc	Not Founc	High	0.00848	0.1219	2.68	169.71
65061	32586					17		Light	Peak Four	High	Not Founc	Not Founc	Not Founc	Not Founc	High	0.00848	0.04815	3.05	160.17
116825	242797	45185				8		Light	Peak Four	High	Peak Four	Not Founc	Not Founc	Not Founc	High	0.00848	0.1388	2.97	79.4
108544	318130	302034	19869	101632	138674	10	NotUni	Light	Peak Four	Peak Fo	High	Peak Four	Peak Four	Peak Four	High	0.00952	0.00533	4.53	204.63
98637	637886	329370	39218	208327	196940	11	NotUni	Light	Peak Four	High	Peak Four	Peak Four	Peak Four	Peak Four	High	0.00848	0.00821	4.95	178.17
						11	Redunc	Heavy	Peak Four	Peak Fo	Peak Four	Peak Four	High	High	High	0.00848	0.00366	4.93	178.6
83123	879617	420934	25905	162170	191276	12		Heavy	Peak Four	Peak Fo	Peak Four	Peak Four	High	High	High	0.00848	0.03292	2.94	117.41
	97033	65748	21220	167379	158533	13		Heavy	Not Founc	Peak Fo	Peak Four	Peak Four	High	Peak Four	High	0.00848	0.05814	2.61	120.26
		10377	46994	71935	138917	14		Heavy	Not Founc	Not Fou	Peak Four	Peak Four	Peak Four	High	High	0.00952	0.05861	3.59	189.26
25770	92021	58700	10788	32148	52686	15	NotUni	Light	Peak Four	Peak Fo	High	Peak Four	Peak Four	Peak Four	High	0.00952	0.06776	3.17	13.2
126573	521283	272596	34002	105449	116103	9	NotUni	Heavy	Peak Four	Peak Fo	Peak Four	Peak Four	Peak Four	High	High	0.00952	0.02003	3.9	217.58
774311	1481763	843531		163640	139548	142		Light	Peak Four	Peak Fo	High	Not Founc	Peak Four	Peak Four	High	0.00952	0.09816	3.53	85.99
110889	33330	103476				36		Light	Peak Four	Peak Fo	High	Not Founc	Not Founc	Not Founc	High	0.00952	0.01759	3.56	67.46
20944	36168	239539				38		Light	Peak Four	High	High	Not Founc	Not Founc	Not Founc	High	0.00952	7.09E-06	8.12	39.72
		6718	39128	40173	110718	56		Heavy	Not Founc	Not Fou	Peak Four	Peak Four	Peak Four	High	High	0.00952	0.01232	4.12	106.39
	71996	91836				57	NotUni	Light	Not Founc	High	Peak Four	Not Founc	Not Founc	Not Founc	High	0.00848	0.1011	2.37	13.19
188194	254523	97759	104987	146812	129452	58		Light	Peak Four	High	High	Peak Four	Peak Four	Peak Four	High	0.00848	0.00301	4.83	209.61
77043	260738	120207				59		Light	Peak Four	High	Peak Four	Not Founc	Not Founc	Not Founc	High	0.00848	0.0125	3.57	119.43

						60	NoQua	Heavy	Not Founc	Not Fou	Not Founc	Not Founc	Not Founc	High	High	0.00952	0.1605	3.11	
293029	478152	219842				61		Light	Peak Four	High	Peak Four	Not Founc	Not Founc	Not Founc	High	0.00848	0.04421	2.32	60.58
49532	84463	68968		3592		62		Light	Peak Four	High	Peak Four	Not Founc	Peak Four	Not Founc	High	0.00848	0.08559	2.09	15.86
1341189	1269742	1354392				55		Light	Peak Four	Peak Fo	High	Not Founc	Not Founc	Not Founc	High	0.00952	0.00017	6.07	102.3
	46208	129417				63		Light	Not Founc	High	High	Not Founc	Not Founc	Not Founc	High	0.00848	0.1061	2.46	15.28
83746	37120	19576	27989	21571	16108	65		Light	Peak Four	High	Peak Four	Peak Four	Peak Four	Peak Four	High	0.00848	0.04286	3.83	196.33
1589770	9841646	3932073	459025	1601906	1443102	66	NotUni	Light	Peak Four	High	Peak Four	Peak Four	Peak Four	Peak Four	High	0.00848	0.1035	2.22	136.72
	52890	35254				67		Light	Not Founc	High	Peak Four	Not Founc	Not Founc	Not Founc	High	0.00848	0.1012	2.56	200.96
64564	290559	106586	4021	41646	29222	68	NotUni	Light	Peak Four	High	Peak Four	Peak Four	Peak Four	Peak Four	High	0.00848	0.05843	2.8	38.6
8038655	8363166	3052815				69		Light	Peak Four	High	High	Not Founc	Not Founc	Not Founc	High	0.00952	0.00253	5.06	169.02
1915259	1063314	682683		5815	4165	70		Light	Peak Four	High	High	Not Founc	Peak Four	Peak Four	High	0.00952	0.00145	4.56	186.05
	18940	49519	148919	245081	109343	71		Heavy	Not Founc	Peak Fo	Peak Four	Peak Four	Peak Four	High	High	0.00952	0.119	2.48	166.96
19878	29041	19930	38654	66363	99534	64		Heavy	Peak Four	Peak Fo	Peak Four	Peak Four	High	Peak Four	High	0.00848	0.1019	2.2	140.91
30851	19387	39817				37		Light	Peak Four	Peak Fo	High	Not Founc	Not Founc	Not Founc	High	0.00952	0.1867	1.86	43.5
110391	165200	58524				54		Light	Peak Four	High	Peak Four	Not Founc	Not Founc	Not Founc	High	0.00848	0.01044	4.36	117.71
81624	83586	39108				52		Light	Peak Four	High	Peak Four	Not Founc	Not Founc	Not Founc	High	0.00848	0.1021	2.62	64.37
160739	174273	148369				39		Light	Peak Four	Peak Fo	High	Not Founc	Not Founc	Not Founc	High	0.00952	0.05115	3.32	123.01
						40	Redunc	Light	Peak Four	Peak Fo	High	Peak Four	Peak Four	Peak Four	High	0.00952	0.1118	3.13	56.31
15265	284553	99611	14771	154705	117865	40	NotUni	Heavy	Peak Four	Peak Fo	Peak Four	Peak Four	High	Peak Four	High	0.00848	0.04372	3.37	56.31
78036	98455	105385				41		Light	Peak Four	High	Peak Four	Not Founc	Not Founc	Not Founc	High	0.00848	0.04873	3.1	147.29
842416	2446858	2305143	111339	490877	986358	42	NotUni	Light	Peak Four	Peak Fo	High	Peak Four	Peak Four	Peak Four	High	0.00952	0.00455	4.57	24.81
						42	Redunc	Heavy	Peak Four	Peak Fo	Peak Four	Peak Four	High	High	High	0.00952	0.00679	4.34	24.81
308671	3070234	665761	124186	659664	332061	43	NotUni	Light	Peak Four	High	High	Peak Four	Peak Four	Peak Four	High	0.00952	0.00295	4.04	54.45
						53	NoQua	Heavy	Not Founc	Not Fou	Not Founc	Not Founc	Not Founc	High	High	0.00952	0.08023	3.02	
						43	Redunc	Heavy	Peak Four	Peak Fo	Peak Four	Peak Four	High	High	High	0.00952	0.00586	4.57	54.45
77169	103999	74435				45		Light	Peak Four	High	Peak Four	Not Founc	Not Founc	Not Founc	High	0.00848	0.1047	3.45	141.5
6877	5432	46156				46		Light	Peak Four	Peak Fo	High	Not Founc	Not Founc	Not Founc	High	0.00952	0.00338	4.29	13.03
10382	23375	39580				47		Light	Peak Four	Peak Fo	High	Not Founc	Not Founc	Not Founc	High	0.00952	0.0616	3.15	160.83
36994	223582	81231	16055	40664		48		Heavy	Peak Four	Peak Fo	Peak Four	Peak Four	High	Not Founc	High	0.00848	0.1225	2.44	141.7
	87705	43253		25951	35390	49		Light	Not Founc	Peak Fo	High	Not Founc	Peak Four	Peak Four	High	0.00952	0.04611	3.75	59.35
46361	161972	166384	67495	270347	408771	50		Heavy	Peak Four	Peak Fo	Peak Four	Peak Four	High	Peak Four	High	0.00848	0.00871	4.29	95.73
89404	122953	43471		5795		51		Light	Peak Four	High	Peak Four	Not Founc	Peak Four	Not Founc	High	0.00848	0.02322	3.35	144.64
92260	46706	67433				44		Light	Peak Four	Peak Fo	High	Not Founc	Not Founc	Not Founc	High	0.00952	0.00482	5.01	64.82
2676664	2464041	1718845				143		Light	Peak Four	High	High	Not Founc	Not Founc	Not Founc	High	0.00848	0.00025	6.38	116.09

Supplemental Table 4.6. Raw Proteome discoverer output from the three replicates of Condition F

Checked	Confide	Sequence	Modificati	Contamina	Qvality PE	Qvality q-v	Num	Num	Num	Master Pr	Master Protei	Protein A	Numb	Theo MHp	Abundances	Abundanc	Abundanc	Abundanc	Abundance F
FALSE	High	AAFGGSGGRGSSSGG(		TRUE	2.26E-05	0.00661	1	1	1	P35908	Keratin, type II	P35908	1	2501.07	90327		57.98		216939
FALSE	High	RDTLLLELK		TRUE	0.04313	0.00661	1	1	3	cRAP117	000000|rLys-C	cRAP117	1	1100.67	19354741		27.09		19495408
FALSE	High	RDTLLLELKR		TRUE	0.23353	0.00661	1	1	1	cRAP117	000000|rLys-C	cRAP117	2	1256.77	230148		75.21		540757
FALSE	High	RGDLPFVVPR		FALSE	0.0566	0.00661	1	1	2	P35579	Myosin-9 OS=H	P35579	1	1155.66	286722	1591	7.12		286722
FALSE	High	RKPDTIEVQQMKAQA		FALSE	0.0014	0.00661	1	3	1	P35241	Radixin OS=Ho	P35241; F	2	1899.02	167958		21.89		188099
FALSE	High	RTPPAGVFYQGWSAT		TRUE	0.00957	0.00661	1	1	1	cRAP117	000000|rLys-C	cRAP117	1	3239.62	283951		21.65		283951
FALSE	High	RTPPAGVFYQGWSAT		TRUE	0.00282	0.00661	1	1	2	cRAP117	000000|rLys-C	cRAP117	2	3610.81	298296		88.44		760327
FALSE	High	QVNEVLK		FALSE	0.06523	0.00661	1	1	1	Q96RW7	Hemicentin-1 (	Q96RW7	0	829.478	134673		15.62		170787
FALSE	High	RVTIMPK	2xLabel:13	FALSE	0.2036	0.00661	1	5	1	P84243	Histone H3.3 C	P84243; (	1	856.548	256800	54841	39.3	28.76	256800
FALSE	High	RVTIMPKDIQLAR		FALSE	0.00369	0.00661	1	5	2	P84243	Histone H3.3 C	P84243; (	2	1540.9	343080	60507	72.02	66.26	726929
FALSE	High	RVTIMPKD	1xOxidatic	FALSE	0.01917	0.00661	1	5	1	P84243	Histone H3.3 C	P84243; (	2	1556.89	189925	26998	111.23	106.13	819422
FALSE	High	SAHSIAQLQK		FALSE	0.01025	0.00661	1	1	1	Q9ULS5	Transmembrar	Q9ULS5	0	1082.6	37576		15.27		31988
FALSE	High	SCAAAGTE	2xCarbami	TRUE	0.00022	0.00661	1	1	1	cRAP112	Trypsin OS=Su	cRAP112	0	1882.84	78370		46.08		58212
FALSE	High	SCQAQPTT	1xCarbami	TRUE	0.03596	0.00661	1	1	1	cRAP016	Kappa-casein (	cRAP016	0	1250.56	35977		28.08		51518
FALSE	High	SGFSSVSVSR		FALSE	0.00812	0.00661	1	1	1	P02538	Keratin, type II	P02538	0	1012.51	83772		76.58		72416
FALSE	High	RVTIMPK	1xOxidatic	FALSE	0.08194	0.00661	1	5	1	P84243	Histone H3.3 C	P84243; (	1	860.502	88404	12353	28.35	38.04	54905
FALSE	High	QSPSYGRHGSGSGR		FALSE	0.12715	0.00661	1	1	1	Q86YZ3	Hornerin OS=H	Q86YZ3	1	1432.67	69959		59.6		17806
FALSE	High	NTDGSTDYGILQINSR		TRUE	0.00067	0.00661	1	1	2	cRAP078	Lysozyme C OS	cRAP078	0	1753.84	115816		64.09		280897
FALSE	High	NLCNIPCS/	3xCarbami	TRUE	0.01022	0.00661	1	1	1	cRAP078	Lysozyme C OS	cRAP078	0	2508.19	107270		60.57		277131
FALSE	High	LLLPGELA	1xLabel:13	FALSE	0.24035	0.00661	1	15	1	Q16778	Histone H2B ty	Q16778; (	0	959.623	2628580	499077	46.88	41.84	3659916
FALSE	High	IITHPNFNGNTLDNDIN		TRUE	0.05624	0.00661	1	1	1	cRAP112	Trypsin OS=Su	cRAP112	0	2283.18	1493997		63.97		2710962
FALSE	High	IITHPNFN(	1xOxidatic	TRUE	0.0665	0.00661	1	1	1	cRAP112	Trypsin OS=Su	cRAP112	0	2299.18	216072		31.53		216072
FALSE	High	IMLKGDNI	1xLabel:13	FALSE	0.01612	0.00661	1	1	1	P62304	Small nuclear r	P62304	1	1751.97	65015	59293	66.89	85.66	108696
FALSE	High	LSAKPAPPKPEPKPK		FALSE	0.01974	0.00661	1	1	2	P05204	Non-histone cl	P05204	0	1584.95	229786	29282	35.59	37.16	366336
FALSE	High	LSAKPAPPKPEPRPK		FALSE	0.10941	0.00661	1	1	1	O00479	High mobility g	O00479	0	1612.95	110647	2511	34.71	7.85	110647
FALSE	High	ISGLIYEETR		FALSE	0.03027	0.00661	1	1	1	P62805	Histone H4 OS	P62805	0	1180.62	127584	4177	33.95	71.52	127584
FALSE	High	LTGKDVNFEFPEFQL		FALSE	0.13998	0.00661	1	1	1	P62081	40S ribosomal	P62081	1	1783.89	35458	61689	88.61	48.41	85417
FALSE	High	MIAGQVLDINLAAEPK		FALSE	0.00086	0.00661	1	1	1	P07910	Heterogeneou	P07910	0	1682.91	33872	21166	47.17	77.55	47912
FALSE	High	MPCTEDYI	1xCarbami	TRUE	0.02325	0.00661	1	1	1	cRAP087	Serum albumir	cRAP087	0	1724.83	123567	16028	92.92	20.62	
FALSE	High	MREIVHIQ	1xCarbami	FALSE	0.08679	0.00661	1	4	1	Q13885	Tubulin beta-2	Q13885; (	1	2122.1		9810		117.98	
FALSE	High	MSRFKFID	1xOxidatic	FALSE	0.11909	0.00661	1	1	1	Q6P1N9	Putative deoxy	Q6P1N9	2	2323.2					
FALSE	High	MVFTSSAI	1xCarbami	TRUE	1.16E-05	0.00661	1	1	7	cRAP117	000000|rLys-C	cRAP117	0	2691.24	9616511		42.67		11244193
FALSE	High	MVFTSSAI	1xCarbami	TRUE	1.41E-06	0.00661	1	1	6	cRAP117	000000|rLys-C	cRAP117	0	2707.23	195400		38.47		218311
FALSE	High	NDYFSDFSGVYSQISR		TRUE	0.00096	0.00661	1	1	1	cRAP117	000000|rLys-C	cRAP117	0	1884.84	380230		24.58		389549
FALSE	High	SGGGGGG	1xCarbami	TRUE	4.87E-05	0.00661	1	1	2	P13645	Keratin, type I	P13645	0	1549.68	56494		41.23		56494
FALSE	High	LLLPGELAK		FALSE	0.25812	0.00661	1	15	1	Q16778	Histone H2B ty	Q16778; (	0	953.603					
FALSE	High	SGGGGGGGLGSGGSIF		TRUE	2.11E-05	0.00661	1	1	1	P35527	Keratin, type I	P35527	0	1232.6	195864		29.77		171159
FALSE	High	SGYRSGGGFSSGSAGII		TRUE	0.21403	0.00661	1	1	1	P04264	Keratin, type II	P04264	1	2121.01	27433		41.14		27433
FALSE	High	VDWPSAVVEGGSSGS		TRUE	0.03107	0.00661	1	1	1	cRAP117	000000|rLys-C	cRAP117	0	2864.41	395539		62.03		817605
FALSE	High	VHKECCH(	3xCarbami	TRUE	0.0355	0.00661	1	1	1	cRAP087	Serum albumir	cRAP087	2	2612.17	160926		71.63		23429

FALSE	High	VHPAVVIR		FALSE	0.06957	0.00661	1	1	1	P62829	60S ribosomal	P62829	0	890.557	195711	2400	31.9		225760
FALSE	High	VLKQVHPDTGISSK		FALSE	0.0781	0.00661	1	15	1	Q16778	Histone H2B ty	Q16778; (	1	1508.84	113065	20657	34.52	45.34	161059
FALSE	High	VLPGVDALSNI		FALSE	0.14172	0.00661	1	1	1	P00558	Phosphoglycer	P00558	0	1097.62					
FALSE	High	VTIAQGGVLPNIQAVLI		FALSE	0.00224	0.00661	1	10	1	Q16777	Histone H2A ty	Q16777; (	0	1931.17	586752	218455	91.83		1528001
FALSE	High	VASLRETY(	2xCarbami	TRUE	0.00179	0.00661	1	1	1	cRAP087	Serum albumir	cRAP087	1	2004.85	26384		30.91		
FALSE	High	VTIAQGGVLPNIQAVLI		FALSE	0.07491	0.00661	1	10	1	Q16777	Histone H2A ty	Q16777; (	1	2059.26	23689	5230	121.25	74.42	183326
FALSE	High	VTIMPKDIQLAR		FALSE	0.02302	0.00661	1	5	2	P84243	Histone H3.3 C	P84243; (	1	1384.8	314119	50397	32.16	18	314119
FALSE	High	WWCNDG	1xCarbami	TRUE	0.23353	0.00661	1	1	1	cRAP078	Lysozyme C OS	cRAP078	1	1491.65	235539		138.94		2123806
FALSE	High	YALYDATY	1xLabel:13	FALSE	0.01433	0.00661	1	2	1	P23528	Cofilin-1 OS=H	P23528; (	0	1343.65	53030	17608	93.53	88.38	191504
FALSE	High	YEKDIAAYR		FALSE	0.02027	0.00661	1	2	2	P26583	High mobility g	P26583; F	1	1128.57	1186020		36.49		1934749
FALSE	High	YICDNQDT	1xCarbami	TRUE	0.00111	0.00661	1	1	2	cRAP087	Serum albumir	cRAP087	0	1443.64	1944094		91.54		137243
FALSE	High	YRPGTVALR		FALSE	0.0689	0.00661	1	5	1	P84243	Histone H3.3 C	P84243; (	0	1032.59	545248	59615	44.28	46.32	545248
FALSE	High	VTIAQGGVLPNIQAVLI		FALSE	0.05221	0.00661	1	1	1	Q16777	Histone H2A ty	Q16777	2	2641.54	20021		10.28		21533
FALSE	High	TVTAMDVVYALKR		FALSE	0.05272	0.00661	1	1	2	P62805	Histone H4 OS	P62805	1	1466.8	67519	13487	50.94	3.86	67519
FALSE	High	TVTAMDVVYALK		FALSE	0.07419	0.00661	1	1	1	P62805	Histone H4 OS	P62805	0	1310.7	51863	7787	55.53	52.62	66569
FALSE	High	TVETRDGQVINETSQH		FALSE	0.03027	0.00661	1	1	1	P08670	Vimentin OS=H	P08670	1	2423.11	21354		111.53		103619
FALSE	High	SLDLDSIIAEVK		TRUE	0.00902	0.00661	1	1	1	P04264	Keratin, type II	P04264	0	1302.72	61236		12.67		
FALSE	High	SLHTLFGD	1xCarbami	TRUE	0.01154	0.00661	1	1	1	cRAP087	Serum albumir	cRAP087	0	1419.69	86133		86.67		7245
FALSE	High	SLNNQFASFIDK		TRUE	0.01994	0.00661	1	1	1	P04264	Keratin, type II	P04264	0	1383.69	11170	3236	113.8		3675
FALSE	High	SLVNLGGSK		TRUE	0.11114	0.00661	1	1	1	P04264	Keratin, type II	P04264	0	874.499	1255966		69.43		3263988
FALSE	High	SPAQILQWQVLSNTVF		TRUE	0.04412	0.00661	1	1	2	cRAP016	Kappa-casein (	cRAP016	0	1980.09	138589		51.58		260141
FALSE	High	SQRAGLQFPVGR		FALSE	0.16215	0.00661	1	2	1	Q71UI9	Histone H2A.V	Q71UI9; F	1	1315.72	67667	12047	67.74	93.21	149714
FALSE	High	SSLLEKGLDGAKK		FALSE	0.01261	0.00661	1	1	2	P81605	Dermcidin OS=	P81605	2	1345.77	120338		80.49		417258
FALSE	High	STITSREIQTAVR		FALSE	0.14978	0.00661	1	13	1	Q16778	Histone H2B ty	Q16778; (	1	1461.8	996142	247516	98.45	98.51	3125733
FALSE	High	TAENATSGETLEENEA(		FALSE	0.02317	0.00661	1	1	1	Q9UQ80	Proliferation-a	Q9UQ80	0	1837.76					
FALSE	High	TCVADESH	2xCarbami	TRUE	0.03875	0.00661	1	1	1	cRAP087	Serum albumir	cRAP087	0	1463.59	213007		90.65		15623
FALSE	High	TIPSWATLSASQLAR		FALSE	0.13158	0.00661	1	1	1	Q5SSJ5	Heterochroma	Q5SSJ5	0	1601.86	14927		71.9		27738
FALSE	High	TPPAGVFYQGWSATP		TRUE	0.01911	0.00661	1	1	1	cRAP117	000000|rLys-C	cRAP117	0	3083.52	41729	3255	111.8		261807
FALSE	High	TPPAGVFYQGWSATP		TRUE	0.07785	0.00661	1	1	1	cRAP117	000000|rLys-C	cRAP117	1	3454.7	357966		40.92		423658
FALSE	High	TPPAGVFYQGWSATP		TRUE	0.01655	0.00661	1	1	1	cRAP117	000000|rLys-C	cRAP117	2	3582.8	52753		56.35		104495
FALSE	High	TTHFVEGGDAGNRED		FALSE	0.08142	0.00661	1	1	1	P18124	60S ribosomal	P18124	1	2115.98	19886	18779	58.06	31.01	41793
FALSE	High	SGGGGGR	1xCarbami	TRUE	5.78E-05	0.00661	1	1	2	P04264	Keratin, type II	P04264	1	2293.98	104624		71.63		303554
FALSE	High	LLDFGSLSI	1xOxidatic	FALSE	0.08326	0.00661	1	1	1	P08708	40S ribosomal	P08708	1	2779.44	17897	18677	87.99		37082
FALSE	High	LLDFGSLSI	2xLabel:13	FALSE	0.0337	0.00661	1	1	2	P08708	40S ribosomal	P08708	1	2775.49	27446	20406	48.44	27.82	39219
FALSE	High	LLDFGSLSNLQVTQPT\		FALSE	0.00917	0.00661	1	1	1	P08708	40S ribosomal	P08708	1	2763.45					
FALSE	High	CCTKPESEI	3xCarbami	TRUE	0.02317	0.00661	1	1	1	cRAP087	Serum albumir	cRAP087	1	2872.31	44770		57.24		
FALSE	High	CELAAAMI	1xCarbami	TRUE	0.12715	0.00661	1	1	1	cRAP078	Lysozyme C OS	cRAP078	1	1049.52	18947		80.49		60345
FALSE	High	CNKEVYFA	1xCarbami	FALSE	0.06136	0.00661	1	1	1	P50238	Cysteine-rich p	P50238	1	1315.61	308034	53080	103.13	74.47	1631005
FALSE	High	CSQAVYA/	1xCarbami	FALSE	0.00057	0.00661	1	1	3	P21291	Cysteine and g	P21291	1	1651.85	127791	3753	14.44		106430
FALSE	High	DAEAWFNEK		TRUE	0.05221	0.00661	1	4	1	P13645	Keratin, type I	Q7Z3Y7; I	0	1109.49	16999		77.95		16999
FALSE	High	DAFLGSFLYEYSR		TRUE	0.00778	0.00661	1	1	1	cRAP087	Serum albumir	cRAP087	0	1567.74	120773		115.46		11408
FALSE	High	AYDNATA/	1xCarbami	TRUE	0.00069	0.00661	1	1	1	cRAP117	000000|rLys-C	cRAP117	1	3766.77	75734		48.5		108271

FALSE	High	DAVEDLESVGK		FALSE	0.04572	0.00661	1	1	1	P81605	Dermcidin OS=	P81605	0	1161.56	23461		35.04		28206
FALSE	High	DGFGASG	2xCarbami	TRUE	0.01158	0.00661	1	1	1	cRAP117	000000|rLys-C	cRAP117	1	3307.46	48774		32.28		48774
FALSE	High	DGQVINETSQHHDDL		FALSE	0.24592	0.00661	1	1	1	P08670	Vimentin OS=	P08670	0	1836.8					
FALSE	High	DNIQGITKPAIR		FALSE	0.01196	0.00661	1	1	2	P62805	Histone H4 OS	P62805	0	1325.75	63037	5408	40.09	35.37	69027
FALSE	High	DNIQGITKPAIRR		FALSE	0.11045	0.00661	1	1	1	P62805	Histone H4 OS	P62805	1	1481.85	97908	16729	37.91	25	97908
FALSE	High	DTHKSEIAHR		TRUE	0.06316	0.00661	1	1	1	cRAP087	Serum albumir	cRAP087	1	1193.6	1041120				
FALSE	High	DTLLLELK		TRUE	0.05715	0.00661	1	1	1	cRAP117	000000|rLys-C	cRAP117	0	944.566	115357	4217	73.16	17.67	273393
FALSE	High	DGFGASG	2xCarbami	TRUE	7.67E-05	0.00661	1	1	2	cRAP117	000000|rLys-C	cRAP117	0	2231.93	258828		70.97		578893
FALSE	High	AYDNATAAVAK		TRUE	0.02094	0.00661	1	1	2	cRAP117	000000|rLys-C	cRAP117	0	1094.55	18712346		49.64		28097283
FALSE	High	AVFVDLEP	1xLabel:13	FALSE	0.01607	0.00661	1	3	2	Q71U36	Tubulin alpha-	Q71U36;	0	1707.93		51091		50.38	
FALSE	High	AVFPSIVGRPR		FALSE	0.09671	0.00661	1	9	1	A5A3E0	POTE ankyrin	A5A3E0;	0	1198.71	58828	14641	76.17	81.55	162925
FALSE	High	AAKPKTAKPKAAKPK		FALSE	0.21153	0.00661	1	1	1	P10412	Histone H1.4	P10412	2	1534.98	24822	8623	69.27		58332
FALSE	High	AEFVEVTK		TRUE	0.04244	0.00661	1	1	1	cRAP087	Serum albumir	cRAP087	0	922.488	224498		81.01		46166
FALSE	High	AGFADPDDFTLGAGP		FALSE	0.23829	0.00661	1	1	1	Q6ZRV2	Protein FAM8	Q6ZRV2	0	1606.75	71480		70.83		210441
FALSE	High	AGFAGDD.	1xLabel:13	FALSE	0.01778	0.00661	1	10	1	A5A3E0	POTE ankyrin	A5A3E0;	0	982.468	494633	143575	65.49	66.81	1054583
FALSE	High	AGFAGDDAPRAVFPS		FALSE	0.16463	0.00661	1	9	1	A5A3E0	POTE ankyrin	A5A3E0;	1	2156.14	225754	41290	59.16	52.65	443238
FALSE	High	AGLQFPVGR		FALSE	0.06057	0.00661	2	15	1	Q16777; C	Histone H2A ty	Q16777;	0	944.531	2123992	430367	29.28	23.61	2123992
FALSE	High	AGLQFPV	1xLabel:13	FALSE	0.05884	0.00661	2	15	1	Q16777; C	Histone H2A ty	Q16777;	0	950.551					
FALSE	High	AGLQFPVGRVHR		FALSE	0.11578	0.00661	1	12	1	Q16777	Histone H2A ty	Q16777;	1	1336.76	273415	56510	76.38	74.29	670676
FALSE	High	AGYRDGF	2xCarbami	TRUE	0.0001	0.00661	1	1	5	cRAP117	000000|rLys-C	cRAP117	1	2679.15	6080934		30.58		7750479
FALSE	High	AGYRDGF	2xCarbami	TRUE	1.81E-05	0.00661	1	1	4	cRAP117	000000|rLys-C	cRAP117	2	3754.68	5086647	4136	38.41		6551620
FALSE	High	ALKAWSVAR		TRUE	0.21466	0.00661	1	1	1	cRAP087	Serum albumir	cRAP087	1	1001.59	725413		70.69		99009
FALSE	High	AMGIMNSFVNDIFER		FALSE	0.06136	0.00661	1	14	1	Q16778	Histone H2B ty	Q16778;	0	1743.82	86303	22213	72.08	57.01	206389
FALSE	High	AMGIMNS	1xLabel:13	FALSE	0.14886	0.00661	1	14	1	Q16778	Histone H2B ty	Q16778;	0	1749.84					
FALSE	High	AMGIMNS	1xOxidatic	FALSE	0.00637	0.00661	1	14	2	Q16778	Histone H2B ty	Q16778;	0	1759.81	231113	100424	86.79		376023
FALSE	High	ASGQAFEL	1xLabel:13	FALSE	0.2048	0.00661	1	1	1	P16949	Stathmin OS=	P16949	0	1394.77		27107		44.08	
FALSE	High	EIQTAVRLLLPGELAK		FALSE	0.00192	0.00661	1	14	2	Q16778	Histone H2B ty	Q16778;	1	1751.04	1289803	290166	73.39	63.96	2747081
FALSE	High	EIQTAVRL	2xLabel:13	FALSE	0.01688	0.00661	1	14	3	Q16778	Histone H2B ty	Q16778;	1	1763.08					
FALSE	High	FESNFNTQATNR		TRUE	0.00556	0.00661	1	1	2	cRAP078	Lysozyme C O	cRAP078	0	1428.65	147556		113.01		731085
FALSE	High	FESNFNTQATNRNTD		TRUE	0.03189	0.00661	1	1	1	cRAP078	Lysozyme C O	cRAP078	1	3163.47	25765		76.86		47376
FALSE	High	HIATNAVLFFGR		TRUE	0.14841	0.00661	1	1	1	cRAP086	Ovalbumin OS	cRAP086	0	1345.74	10491	1570	109.26	4.72	58040
FALSE	High	KVHGSLAR		FALSE	0.18962	0.00661	1	1	1	P62861	40S ribosomal	P62861	1	867.516	50922	70158	41.46	43.4	25991
FALSE	High	KVPQVSTPTLVEVSR		TRUE	0.00429	0.00661	1	2	1	cRAP087	Serum albumir	cRAP087;	1	1639.94	735675		99.19		94480
FALSE	High	KYSQGNVSAVGVTYD		TRUE	0.00018	0.00661	1	1	1	cRAP117	000000|rLys-C	cRAP117	1	2324.16	455605		108.51		2254218
FALSE	High	LAPDYDALDVANKIGI		FALSE	0.04528	0.00661	1	1	1	P62750	60S ribosomal	P62750	1	1800.97	28014	37106	65.65	25.01	55724
FALSE	High	LFIGGLSFETTEESLR		FALSE	0.04055	0.00661	1	1	1	P22626	Heterogeneou	P22626	0	1798.92					
FALSE	High	LFTFHADI	1xCarbami	TRUE	0.09764	0.00661	1	1	1	cRAP087	Serum albumir	cRAP087	0	1907.92	67701		70.2		
FALSE	High	LGEHNIDVLEGNEQFI		TRUE	1.05E-05	0.00661	1	1	2	cRAP112	Trypsin OS=Su	cRAP112	0	2211.1	4547298		44.76		4877349
FALSE	High	LGEYGFQNALIVR		TRUE	0.03097	0.00661	1	1	1	cRAP087	Serum albumir	cRAP087	0	1479.8	452717		95.43		62108
FALSE	High	LGGSAVISLEGKPL		FALSE	0.02564	0.00661	1	1	1	P23528	Cofilin-1 OS=H	P23528	0	1340.78	96772	30148	74.5	69.14	170102
FALSE	High	LGGSAVISI	1xLabel:13	FALSE	0.03425	0.00661	1	1	1	P23528	Cofilin-1 OS=H	P23528	0	1346.8					
FALSE	High	IGLGGRGGSGGSYGR		TRUE	0.18737	0.00661	1	1	1	P35527	Keratin, type I	P35527	1	1350.69	576744		48.54		1073380

FALSE	High	IHRHLK		FALSE	0.21718	0.00661	1	2	1	Q71UI9	Histone H2A.V	Q71UI9; F	1	803.5	70028	2681	24.64	25.63	52997
FALSE	High	LKECCDKP	2xCarbami	TRUE	0.00379	0.00661	1	1	2	cRAP087	Serum albumir	cRAP087	1	1532.78	1762565	2991	71.91	32.44	303397
FALSE	High	LLCGLLAEF	1xCarbami	FALSE	0.04016	0.00661	1	1	1	P14174	Macrophage n	P14174	0	1050.61	5395	15754	106	112.39	25286
FALSE	High	HGSGLGHSSSHGQHG		FALSE	0.06195	0.00661	1	1	1	Q86YZ3	Hornerin OS=H	Q86YZ3	0	1885.84	27240		16.08		21980
FALSE	High	YRPGTVALREIRR		FALSE	0.24663	0.00661	1	5	1	P84243	Histone H3.3 C	P84243; (	2	1586.92	68552	7353	64.77		98593
FALSE	High	HGLDNYR(	1xCarbami	TRUE	0.02157	0.00661	1	1	1	cRAP078	Lysozyme C OS	cRAP078	1	2181.03					
FALSE	High	HAVSEGTK	3xLabel:13	FALSE	0.01452	0.00661	1	12	1	Q16778	Histone H2B ty	Q16778; (	2	1812					
FALSE	High	FKDLGEEHFK		TRUE	0.00109	0.00661	1	1	1	cRAP087	Serum albumir	cRAP087	1	1249.62	103708		94.32		12790
FALSE	High	FSSCGGGG	1xCarbami	TRUE	0.00011	0.00661	1	1	2	P04264	Keratin, type II	P04264	0	1765.73	61827		48.84		133002
FALSE	High	FVNVVPTFGK		FALSE	0.24875	0.00661	1	1	1	P62861	40S ribosomal	P62861	0	1107.62	43192	27981	36.32	22.31	49033
FALSE	High	GAVHDVKDVLDSVL		FALSE	0.04677	0.00661	1	1	1	P81605	Dermcidin OS=	P81605	1	1466.78					
FALSE	High	GFGRGGAESHTFK		FALSE	0.03199	0.00661	1	1	1	P50238	Cysteine-rich p	P50238	1	1350.65	25320	8542	58.16	53.55	63370
FALSE	High	GGLYGGPS	1xCarbami	TRUE	0.00014	0.00661	1	1	3	cRAP117	000000|rLys-C	cRAP117	0	1727.78	333247		92.3		1153182
FALSE	High	GGLYGGPS	1xCarbami	TRUE	0.00325	0.00661	1	1	2	cRAP117	000000|rLys-C	cRAP117	1	3593.6	1304975		93.39		3908541
FALSE	High	GGSGGSYGGGGSGGG		TRUE	2.25E-06	0.00661	1	1	2	P35527	Keratin, type I	P35527	0	1791.73	50267		28.88		37378
FALSE	High	GSCGIGGG	1xCarbami	FALSE	0.01299	0.00661	1	2	1	P02533	Keratin, type I	P02533; F	0	1278.59	130392		49.93		82394
FALSE	High	GSLGGGFSSGGFSGGS		TRUE	7.25E-05	0.00661	1	1	1	P13645	Keratin, type I	P13645	0	1707.77	141630		13.94		141630
FALSE	High	GTDVQAWIR		TRUE	0.0337	0.00661	1	1	1	cRAP078	Lysozyme C OS	cRAP078	0	1045.54	72458		56.51		168232
FALSE	High	GTGASGSFKLNK		FALSE	0.08247	0.00661	1	5	1	P10412	Histone H1.4 C	P16401; F	1	1166.62	84374	50139	80.95	75.26	209229
FALSE	High	GTGASGSF	2xLabel:13	FALSE	0.0665	0.00661	1	5	2	P10412	Histone H1.4 C	P16401; F	1	1178.66					
FALSE	High	GTGASGSF	3xLabel:13	FALSE	0.19943	0.00661	1	5	1	P10412	Histone H1.4 C	P16401; F	2	1312.77	185626	123750	52.35	45.88	196212
FALSE	High	HAVSEGTKAVTKYTSSI		FALSE	0.00073	0.00661	1	12	6	Q16778	Histone H2B ty	Q16778; (	2	1793.94	1321874	226060	49.99	58.83	1594055
FALSE	High	HGGGGGGFGGGGFGS		TRUE	0.00423	0.00661	1	1	1	P35908	Keratin, type II	P35908	0	1320.58	33923		38.05		57508
FALSE	High	YSQGNVSAVGVTYDG		TRUE	0.00019	0.00661	1	1	3	cRAP117	000000|rLys-C	cRAP117	0	2196.07	1549846		64.38		2795056

Abundance F	Abundance F	Abundan	Abundan	Abundan	Precur	Quan I	Quan Char	Found in S	Found in S	Found in S	Found in S	Found in S	Found in S	Confid	Percolator	Percolator	XCorr by S	Top Apex RT in m
90327	82748				1		Light	High	Peak Foun	Peak Foun	Not Found	Not Found	Not Found	High	0.00952	0.00022	7.51	67.25
11544733	19354741				86		Light	High	Peak Foun	Peak Foun	Not Found	Not Found	Not Found	High	0.00952	0.06392	3.14	122.18
230148	112179				87		Light	High	Peak Foun	Peak Foun	Not Found	Not Found	Not Found	High	0.00952	0.2299	2.15	101.71
293430	256193		1591		88		Light	High	High	Peak Foun	Not Found	Peak Foun	Not Found	High	0.00952	0.07847	2.78	121.72
167958	120407				89		Light	Peak Foun	High	Peak Foun	Not Found	Not Found	Not Found	High	0.00926	0.00434	2.79	59.35
365444	238983				90		Light	Peak Foun	High	Peak Foun	Not Found	Not Found	Not Found	High	0.00926	0.01887	2.54	138.35
298296	96198				91		Light	Peak Foun	High	Peak Foun	Not Found	Not Found	Not Found	High	0.00926	0.00741	4.5	130.18
129109	134673				85		Light	Peak Foun	High	Peak Foun	Not Found	Not Found	Not Found	High	0.00926	0.08222	1.91	56.6
323144	137613	54841	61461	33882	92		Heavy	Peak Foun	Peak Foun	Peak Foun	Peak Foun	High	Peak Foun	High	0.00926	0.1976	2.46	54.89
343080	151174	138606	60507	38711	94		Light	High	High	Peak Foun	Peak Foun	Peak Foun	Peak Foun	High	0.00926	0.00909	3.12	107.93
189925	73261	118682	26998	14819	95		Light	Peak Foun	High	Peak Foun	Peak Foun	Peak Foun	Peak Foun	High	0.00926	0.03213	2.91	92.11
43494	37576				96		Light	Peak Foun	High	Peak Foun	Not Found	Not Found	Not Found	High	0.00926	0.01986	3.74	47.78
139682	78370				97		Light	High	Peak Foun	Peak Foun	Not Found	Not Found	Not Found	High	0.00952	0.00119	5.51	142.49
35977	30221				98		Light	High	Peak Foun	Peak Foun	Not Found	Not Found	Not Found	High	0.00952	0.05569	3.37	48.74
263304	83772				99		Light	Peak Foun	High	Peak Foun	Not Found	Not Found	Not Found	High	0.00926	0.01663	3.74	80.46
98614	88404	6173	13990	12353	93		Light	Peak Foun	High	Peak Foun	Peak Foun	Peak Foun	Peak Foun	High	0.00926	0.09784	1.59	33.54
69959	79624				84		Light	Peak Foun	High	Peak Foun	Not Found	Not Found	Not Found	High	0.00926	0.1372	1.33	16.26
115816	89271				83		Light	High	Peak Foun	Peak Foun	Not Found	Not Found	Not Found	High	0.00952	0.00278	6.16	138.6
104841	107270				82		Light	High	Peak Foun	Peak Foun	Not Found	Not Found	Not Found	High	0.00952	0.02161	5.73	179.76
2628580	1295866	763062	499077	323659	68		Heavy	Peak Foun	Peak Foun	Peak Foun	High	Peak Foun	Peak Foun	High	0.00952	0.2352	2.21	135.64
1493997	651313				69		Light	Peak Foun	High	Peak Foun	Not Found	Not Found	Not Found	High	0.00926	0.07329	3.82	167.83
272338	140574				70		Light	High	Peak Foun	Peak Foun	Not Found	Not Found	Not Found	High	0.00952	0.08852	3.63	159.66
38888		156966	59293	24403	71		Heavy	Peak Foun	Peak Foun	Not Found	High	Peak Foun	Peak Foun	High	0.00952	0.03042	3.18	165.63
229786	188484	42715	20042	29282	72		Light	High	High	Peak Foun	Peak Foun	Peak Foun	Peak Foun	High	0.00926	0.03282	3.19	40.4
150492	73141	2511	2405	2795	73		Light	Peak Foun	High	Peak Foun	Peak Foun	Peak Foun	Peak Foun	High	0.00926	0.1222	2.92	43.58
203051	109114	10841	4177	2888	74		Light	High	Peak Foun	Peak Foun	Peak Foun	Peak Foun	Peak Foun	High	0.00952	0.04897	3.48	113.96
35458	9607	88128	43182		75		Light	Peak Foun	High	Peak Foun	Peak Foun	Peak Foun	Not Found	High	0.00926	0.1477	2.95	206.5
23946		35616	21166	4148	76		Light	High	Peak Foun	Not Found	Peak Foun	Peak Foun	Peak Foun	High	0.00952	0.00336	5.33	171.17
56218	271603		13839	18562	77		Light	Not Found	High	Peak Foun	Not Found	Peak Foun	Peak Foun	High	0.00926	0.03724	3.58	205.92
		58902	9810	6174	78		Heavy	Not Found	Not Found	Not Found	High	Peak Foun	Peak Foun	High	0.00952	0.1083	3.35	90.71
						InconsistentlyLab		Not Found	Not Found	Not Found	Not Found	Not Found	Not Found	High	0.00926	0.1303	1.69	81.18
9616511	4375277				79		Light	High	High	Peak Foun	Not Found	Not Found	Not Found	High	0.00926	0.00011	6.8	166.35
195400	95508				80		Light	High	High	Peak Foun	Not Found	Not Found	Not Found	High	0.00926	2.19E-05	7.52	155.31
380230	241612				81		Light	Peak Foun	High	Peak Foun	Not Found	Not Found	Not Found	High	0.00926	0.00323	3.93	188.7
89645	39479				100		Light	High	High	Peak Foun	Not Found	Not Found	Not Found	High	0.00926	0.00033	5.54	56.21
					68	Redund	Light	High	Peak Foun	Peak Foun	Peak Foun	Peak Foun	Peak Foun	High	0.00952	0.2486	1.92	135.64
295338	195864				101		Light	Peak Foun	High	Peak Foun	Not Found	Not Found	Not Found	High	0.00926	0.00017	4.51	56.55
47041	21890				103		Light	Peak Foun	High	Peak Foun	Not Found	Not Found	Not Found	High	0.00926	0.2055	2.66	110.22
395539	237462				122		Light	High	Peak Foun	Peak Foun	Not Found	Not Found	Not Found	High	0.00952	0.04993	4.28	202.33
160926	193108				123		Light	Peak Foun	High	Peak Foun	Not Found	Not Found	Not Found	High	0.00926	0.05149	3.02	83.68

195711	115351		2400		124		Light	Peak Foun	High	Peak Foun	Not Found	Peak Foun	Not Found	High	0.00926	0.08638	2.62	59.33
113065	80014	38181	20657	16732	125		Light	High	Peak Foun	Peak Foun	Peak Foun	Peak Foun	Peak Foun	High	0.00952	0.09993	3.08	55.31
						IndistinguishableC		Not Found	Not Found	Not Found	Not Found	Not Found	Not Found	High	0.00952	0.1569	2.83	190.6
586752	164949	218455			126		Light	High	Peak Foun	Peak Foun	Peak Foun	Not Found	Not Found	High	0.00952	0.00689	5.2	206.33
21128	32947				121		Light	Not Found	High	Peak Foun	Not Found	Not Found	Not Found	High	0.00926	0.00524	3.65	100.94
23689	22137	9387	2914		127		Light	Peak Foun	High	Peak Foun	Peak Foun	Peak Foun	Not Found	High	0.00926	0.09124	1.57	183.32
333377	172406	50397	54719	38151	129		Light	High	High	Peak Foun	Peak Foun	Peak Foun	Peak Foun	High	0.00926	0.03694	2.39	122.82
235539	90164				130		Light	High	Peak Foun	Peak Foun	Not Found	Not Found	Not Found	High	0.00952	0.23	2.22	84.17
53030	32680	64423	17608	13738	131		Heavy	Peak Foun	Peak Foun	Peak Foun	High	Peak Foun	Peak Foun	High	0.00952	0.02786	3.49	116.09
1186020	987063				132		Light	Peak Foun	High	Peak Foun	Not Found	Not Found	Not Found	High	0.00926	0.03352	2.77	64.97
1944094	3608354				133		Light	Peak Foun	High	Peak Foun	Not Found	Not Found	Not Found	High	0.00926	0.00362	3.96	72.15
584184	221110	64420	59615	22937	134		Light	Peak Foun	High	Peak Foun	Peak Foun	Peak Foun	Peak Foun	High	0.00926	0.08578	2.42	66.35
18615					128		Light	Peak Foun	High	Not Found	Not Found	Not Found	Not Found	High	0.00926	0.06937	1.49	155.62
76055	23273	13860	13124		120		Light	High	High	Peak Foun	Peak Foun	Peak Foun	Not Found	High	0.00926	0.06975	3.01	153.86
51863	17527	12228	7787	3859	119		Light	Peak Foun	High	Peak Foun	Peak Foun	Peak Foun	Peak Foun	High	0.00926	0.0907	3.57	172.4
21354	11283				118		Light	High	Peak Foun	Peak Foun	Not Found	Not Found	Not Found	High	0.00952	0.0489	3.64	81.28
66991	55974				104		Light	Not Found	High	Peak Foun	Not Found	Not Found	Not Found	High	0.00926	0.01803	4.34	197.6
86133	143622				105		Light	Peak Foun	High	Peak Foun	Not Found	Not Found	Not Found	High	0.00926	0.02176	3.37	130.56
33955			3236		106		Light	Peak Foun	High	Not Found	Not Found	Peak Foun	Not Found	High	0.00926	0.03314	3.47	153.75
1255966	934571				107		Light	High	Peak Foun	Peak Foun	Not Found	Not Found	Not Found	High	0.00952	0.1305	2.25	85.89
138589	96197				108		Light	High	Peak Foun	Peak Foun	Not Found	Not Found	Not Found	High	0.00952	0.06506	4.78	184.61
67667	38325	36397	12047	4813	109		Light	High	Peak Foun	Peak Foun	Peak Foun	Peak Foun	Peak Foun	High	0.00952	0.1738	2.68	96.2
111295	120338				110		Light	High	High	Peak Foun	Not Found	Not Found	Not Found	High	0.00926	0.02329	2.5	74.49
996142	329013	854416	247516	107353	111		Light	Peak Foun	High	Peak Foun	Peak Foun	Peak Foun	Peak Foun	High	0.00926	0.1554	1.97	81.99
						NoQuanValues		Not Found	Not Found	Not Found	Not Found	Not Found	Not Found	High	0.00952	0.04005	4.02	
213007	388176				112		Light	Peak Foun	High	Peak Foun	Not Found	Not Found	Not Found	High	0.00926	0.0551	2.23	34.3
14927	4965				113		Light	High	Peak Foun	Peak Foun	Not Found	Not Found	Not Found	High	0.00952	0.1483	3.36	169.77
39304	41729		3255		114		Light	Peak Foun	High	Peak Foun	Not Found	Peak Foun	Not Found	High	0.00926	0.03207	4.1	150.44
357966	172677				115		Light	Peak Foun	High	Peak Foun	Not Found	Not Found	Not Found	High	0.00926	0.09403	2.75	141.3
52753	34988				116		Light	Peak Foun	High	Peak Foun	Not Found	Not Found	Not Found	High	0.00926	0.02873	4.05	133.32
19886	14000	25011	18779	13229	117		Light	High	Peak Foun	Peak Foun	Peak Foun	Peak Foun	Peak Foun	High	0.00952	0.1033	3.51	66.54
90622	104624				102		Light	High	High	Peak Foun	Not Found	Not Found	Not Found	High	0.00926	0.00038	7.03	97.1
8637		18677			66		Light	High	Peak Foun	Not Found	Peak Foun	Not Found	Not Found	High	0.00952	0.1049	4.99	183.61
19207		24906	16719		67		Heavy	Peak Foun	Peak Foun	Not Found	High	High	Not Found	High	0.00952	0.05309	3.11	194.75
					67	Redunc	Light	High	Peak Foun	Not Found	Peak Foun	Peak Foun	Not Found	High	0.00952	0.01989	4.37	194.75
29144	68773				19		Light	Not Found	High	Peak Foun	Not Found	Not Found	Not Found	High	0.00926	0.03715	2.35	169.43
18947	14699				20		Light	High	Peak Foun	Peak Foun	Not Found	Not Found	Not Found	High	0.00952	0.1446	2.42	61.2
308034	294390	162349	53080	46588	21		Light	High	Peak Foun	Peak Foun	Peak Foun	Peak Foun	Peak Foun	High	0.00952	0.08342	3.49	68.68
127791	142490	3753			22		Light	High	High	Peak Foun	Peak Foun	Not Found	Not Found	High	0.00926	0.00218	3.34	119.83
47363	10860				23		Light	Peak Foun	High	Peak Foun	Not Found	Not Found	Not Found	High	0.00926	0.06928	3.13	126.59
120773	425803				24		Light	Peak Foun	High	Peak Foun	Not Found	Not Found	Not Found	High	0.00926	0.01609	2.01	209.55
52976					18		Light	High	Peak Foun	Not Found	Not Found	Not Found	Not Found	High	0.00952	0.00283	8.97	198.09

13340	23461				25		Light	High	Peak Foun	Peak Foun	Not Found	Not Found	Not Found	High	0.00952	0.06686	2.97	126.12
63068	32121				27		Light	High	Peak Foun	Peak Foun	Not Found	Not Found	Not Found	High	0.00952	0.0237	4.65	147.55
						IndistinguishableC		Not Found	Not Found	Not Found	Not Found	Not Found	Not Found	High	0.00952	0.2393	1.94	79.8
63037	29091		6983	4189	28		Light	High	High	Peak Foun	Not Found	Peak Foun	Peak Foun	High	0.00926	0.02239	3.11	90.12
126373	55974	18019	16729	10883	29		Light	Peak Foun	High	Peak Foun	Peak Foun	Peak Foun	Peak Foun	High	0.00926	0.123	1.99	75.4
1041120					30		Light	Not Found	High	Not Found	Not Found	Not Found	Not Found	High	0.00926	0.08016	1.5	12.63
115357	62017	4217	3358	4808	31		Light	High	Peak Foun	Peak Foun	Peak Foun	Peak Foun	Peak Foun	High	0.00952	0.0791	2.74	156.79
258828	133399				26		Light	High	High	Peak Foun	Not Found	Not Found	Not Found	High	0.00952	0.00054	7.68	121.21
18712346	9472472				17		Light	High	Peak Foun	Peak Foun	Not Found	Not Found	Not Found	High	0.00952	0.0371	3.74	61.04
		76291	51091	25084	16		Heavy	Not Found	Not Found	Not Found	High	High	Peak Foun	High	0.00952	0.03034	5.16	187.82
58828	39595	44951	14641	10103	15		Light	Peak Foun	High	Peak Foun	Peak Foun	Peak Foun	Peak Foun	High	0.00926	0.111	2.97	111.77
24822	15091	8623			2		Light	High	Peak Foun	Peak Foun	Peak Foun	Not Found	Not Found	High	0.00952	0.2127	3.23	12.53
224498	417943				3		Light	Peak Foun	High	Peak Foun	Not Found	Not Found	Not Found	High	0.00926	0.05906	2.6	82.79
71480	65476				4		Light	Peak Foun	High	Peak Foun	Not Found	Not Found	Not Found	High	0.00926	0.2237	2.82	130.31
494633	280920	324504	143575	87715	5		Heavy	Peak Foun	Peak Foun	Peak Foun	High	Peak Foun	Peak Foun	High	0.00952	0.03278	3.05	62.87
225754	134784	78145	41290	28181	6		Light	Peak Foun	High	Peak Foun	Peak Foun	Peak Foun	Peak Foun	High	0.00926	0.1674	1.32	138.38
2349152	1283064	438600	430367	278265	7	NotUni	Light	Peak Foun	High	Peak Foun	Peak Foun	Peak Foun	Peak Foun	High	0.00926	0.0777	3.01	120.98
					7	Redund	Heavy	Peak Foun	Peak Foun	Peak Foun	Peak Foun	High	Peak Foun	High	0.00926	0.07588	3.03	120.98
273415	140072	142741	56510	33423	8		Light	Peak Foun	High	Peak Foun	Peak Foun	Peak Foun	Peak Foun	High	0.00926	0.1275	2.39	93.72
6080934	4099810				9		Light	High	High	Peak Foun	Not Found	Not Found	Not Found	High	0.00952	0.00067	7.86	111.98
5086647	2864086		4136		10		Light	High	High	Peak Foun	Not Found	Peak Foun	Not Found	High	0.00926	0.00015	7.5	137.63
725413	776229				11		Light	Peak Foun	High	Peak Foun	Not Found	Not Found	Not Found	High	0.00926	0.206	2.63	78.33
86303	49231	46723	22213	16181	12		Light	High	Peak Foun	Peak Foun	Peak Foun	Peak Foun	Peak Foun	High	0.00952	0.08346	3.94	216.93
					12	Redund	Heavy	Peak Foun	Peak Foun	Peak Foun	Peak Foun	High	Peak Foun	High	0.00926	0.1549	3.07	216.93
231113	17064	100424			13		Light	High	Peak Foun	Peak Foun	Peak Foun	Not Found	Not Found	High	0.00952	0.01513	3.67	203.33
		48157	27107	21195	14		Heavy	Not Found	Not Found	Not Found	High	Peak Foun	Peak Foun	High	0.00952	0.2079	3.03	164.18
1289803	543884	560740	290166	142998	32		Light	Peak Foun	High	Peak Foun	Peak Foun	Peak Foun	Peak Foun	High	0.00926	0.00552	4.14	169.73
					32	Redund	Heavy	Peak Foun	Peak Foun	Peak Foun	High	High	Peak Foun	High	0.00926	0.02914	3.19	169.73
147556	75636				33		Light	High	High	Peak Foun	Not Found	Not Found	Not Found	High	0.00952	0.01368	4.1	82.39
14012					34		Light	High	Peak Foun	Not Found	Not Found	Not Found	Not Found	High	0.00952	0.05095	4.3	147.31
8485	10491	1518	1623		52		Light	High	Peak Foun	Peak Foun	Peak Foun	Peak Foun	Not Found	High	0.00952	0.1625	2.79	141.94
50922	64544	37232	70158	95903	53		Light	Peak Foun	High	Peak Foun	Peak Foun	Peak Foun	Peak Foun	High	0.00926	0.1871	2.53	13.8
735675	1839442				54		Light	High	Peak Foun	Peak Foun	Not Found	Not Found	Not Found	High	0.00952	0.01125	5.18	116.54
455605	296393				55		Light	High	Peak Foun	Peak Foun	Not Found	Not Found	Not Found	High	0.00952	0.00101	4.93	101.16
28014	13762	44367		31034	56		Light	High	Peak Foun	Peak Foun	Peak Foun	Not Found	Peak Foun	High	0.00952	0.06628	3.16	208.2
					57	NoQua	Light	Not Found	High	Not Found	Not Found	Not Found	Not Found	High	0.00926	0.05711	3.47	
39274	116702				58		Light	Not Found	High	Peak Foun	Not Found	Not Found	Not Found	High	0.00926	0.1118	3.25	155.94
4547298	1820489				59		Light	High	High	Peak Foun	Not Found	Not Found	Not Found	High	0.00926	0.0001	6.25	149.67
452717	1051039				60		Light	High	Peak Foun	Peak Foun	Not Found	Not Found	Not Found	High	0.00952	0.04976	4.62	163.36
96772	25096	46410	30148	7724	61		Light	High	Peak Foun	Peak Foun	Peak Foun	Peak Foun	Peak Foun	High	0.00952	0.04319	3.5	149.52
					61	Redund	Heavy	Peak Foun	Peak Foun	Peak Foun	High	Peak Foun	Peak Foun	High	0.00952	0.05371	3.22	149.52
576744	431117				62		Light	Peak Foun	High	Peak Foun	Not Found	Not Found	Not Found	High	0.00926	0.1852	1.95	62

87586	70028	2681	3552	2135	63		Light	Peak Foun	High	Peak Foun	Peak Foun	Peak Foun	Peak Foun	High	0.00926	0.208	2.2	14.53
1762565	2376169	2368	3778		64		Light	High	High	Peak Foun	Peak Foun	Peak Foun	Not Found	High	0.00926	0.00929	2.65	58.52
5395	3488	95091	15754	13331	65		Heavy	Peak Foun	Peak Foun	Peak Foun	High	Peak Foun	Peak Foun	High	0.00952	0.06052	2.47	139.87
30436	27240				51		Light	Peak Foun	High	Peak Foun	Not Found	Not Found	Not Found	High	0.00926	0.07895	2.17	12.53
68552	19005		7353		135		Light	Peak Foun	High	Peak Foun	Not Found	Peak Foun	Not Found	High	0.00926	0.2299	2.39	79.45
					50	NoQua	Light	High	Not Found	Not Found	Not Found	Not Found	Not Found	High	0.00952	0.0379	3.72	
					48	Redund	Heavy	Peak Foun	Peak Foun	Peak Foun	High	Peak Foun	Peak Foun	High	0.00952	0.02813	4.37	53.44
103708	229300				35		Light	Peak Foun	High	Peak Foun	Not Found	Not Found	Not Found	High	0.00926	0.00357	3.56	72.18
61827	60306				36		Light	High	High	Peak Foun	Not Found	Not Found	Not Found	High	0.00926	0.00061	5.66	109.64
43192	22576	30265	27981	19329	37		Light	High	Peak Foun	Peak Foun	Peak Foun	Peak Foun	Peak Foun	High	0.00952	0.2412	2.78	145.57
					38	NoQua	Light	Not Found	High	Not Found	Not Found	Not Found	Not Found	High	0.00926	0.06369	3.16	
25320	25038	12723		5735	39		Light	High	Peak Foun	Peak Foun	Peak Foun	Not Found	Peak Foun	High	0.00952	0.05103	2.84	49.35
333247	195905				40		Light	High	High	Peak Foun	Not Found	Not Found	Not Found	High	0.00926	0.00073	6.01	103.01
1304975	504186				41		Light	High	Peak Foun	Peak Foun	Not Found	Not Found	Not Found	High	0.00952	0.00912	5.57	184.36
50267	67079				42		Light	High	High	Peak Foun	Not Found	Not Found	Not Found	High	0.00926	3.14E-05	6.11	47.88
225789	130392				43		Light	High	Peak Foun	Peak Foun	Not Found	Not Found	Not Found	High	0.00952	0.02587	3.42	66.22
172917	133482				44		Light	Peak Foun	High	Peak Foun	Not Found	Not Found	Not Found	High	0.00926	0.00045	5.52	139.36
72458	64978				45		Light	Peak Foun	High	Peak Foun	Not Found	Not Found	Not Found	High	0.00926	0.04958	3.59	121.87
84374	36661	116166	50139	23636	46		Light	High	Peak Foun	Peak Foun	Peak Foun	Peak Foun	Peak Foun	High	0.00952	0.1042	3.14	55.16
					46	Redund	Heavy	Peak Foun	Peak Foun	Peak Foun	High	High	Peak Foun	High	0.00926	0.0856	3.28	55.16
185626	58231	123750	135632	48730	47		Heavy	Peak Foun	Peak Foun	Peak Foun	High	Peak Foun	Peak Foun	High	0.00952	0.2036	1.22	37.68
1321874	500573	471321	226060	152959	48		Light	High	High	Peak Foun	Peak Foun	Peak Foun	Peak Foun	High	0.00926	0.00263	5.15	53.44
33923	28922				49		Light	High	Peak Foun	Peak Foun	Not Found	Not Found	Not Found	High	0.00952	0.01111	4.82	63.63
1549846	657740				136		Light	High	High	Peak Foun	Not Found	Not Found	Not Found	High	0.00952	0.00106	6.68	114.95

Supplemental Table 5.1. Master protein identifications from CD3 SPPLATed primary human T cells with SILAC abundance ratio Light/Heavy > 1.

			Condition A			Condition B			Condition C			Condition D			Condition E			Condition F		
	Master.Prc	Master.Protein.	rep1	rep2	rep3	rep1	rep2	rep3	rep1	rep2	rep3	rep1	rep2	rep3	rep1	rep2	rep3	rep1	rep2	rep3
1	A5A3E0	POTE ankyrin dc	NA	NA	NA	NA	NA	NA	NA	NA	NA	NA	NA	NA	2.087	1.987	16.096	2.367	2.451	2.346
2	O00479	High mobility gr	16.125	15.473	14.320	13.989	15.082	4.851	15.156	6.282	14.055	14.685	6.045	15.632	14.226	6.100	4.879	5.462	5.968	4.710
3	O60814	Histone H2B typ	NA	NA	NA	NA	NA	NA	NA	NA	NA	2.042	2.166	2.167	NA	NA	NA	NA	NA	NA
4	P05107	Integrin beta-2 (	NA	NA	NA	NA	NA	NA	NA	NA	NA	14.764	15.186	14.777	NA	NA	NA	NA	NA	NA
5	P05204	Non-histone chr	NA	NA	NA	NA	NA	NA	3.376	3.680	3.048	3.313	3.366	3.239	NA	NA	NA	3.100	3.519	2.686
6	P08670	Vimentin OS=Hc	NA	NA	NA	12.124	14.379	13.705	14.170	15.762	11.809	12.973	14.779	15.385	16.900	4.227	17.676	16.661	14.382	13.462
7	P10412	Histone H1.4 OS	1.619	2.292	1.551	NA	NA	NA	2.384	3.029	1.526	NA	NA	NA	1.682	2.439	1.138	2.758	14.599	13.881
8	P16070	CD44 antigen O	NA	NA	NA	NA	NA	NA	NA	NA	NA	16.577	15.619	15.092	NA	NA	NA	NA	NA	NA
9	P16402	Histone H1.3 OS	NA	NA	NA	1.422	2.289	1.703	NA	NA	NA	1.889	1.912	1.853	NA	NA	NA	NA	NA	NA
10	P21291	Cysteine and gly	17.675	15.612	15.107	NA	NA	NA	17.695	17.325	16.897	17.570	17.170	17.524	NA	NA	NA	4.826	16.963	17.120
11	P23528	Cofilin-1 OS=Ho	2.470	2.212	1.976	NA	NA	NA	2.222	2.093	1.682	NA	NA	NA	NA	NA	NA	1.874	1.788	1.626
12	P26373	60S ribosomal p	15.795	15.136	14.520	NA	NA	NA	NA	NA	NA	NA	NA	NA	NA	NA	NA	NA	NA	NA
13	P26583	High mobility gr	8.206	18.607	17.879	18.046	15.012	17.066	14.138	7.821	18.783	18.919	17.673	18.096	20.049	8.382	8.010	20.884	20.178	19.913
14	P35241	Radixin OS=Hon	16.937	16.115	15.985	16.407	14.908	10.340	19.172	18.976	18.438	19.978	18.819	19.184	20.394	21.111	20.356	17.521	17.358	16.878
15	P35579	Myosin-9 OS=Hc	NA	NA	NA	NA	NA	NA	17.767	18.250	17.263	17.855	17.872	18.749	19.506	19.244	18.632	18.129	7.527	17.967
16	P47914	60S ribosomal p	NA	NA	NA	NA	NA	NA	NA	NA	NA	1.796	1.583	1.233	NA	NA	NA	NA	NA	NA
17	P50238	Cysteine-rich pr	5.125	15.022	13.576	4.114	3.127	2.905	3.569	2.914	2.527	4.088	2.783	3.272	15.744	15.187	14.596	2.556	8.316	2.126
18	P59665	Neutrophil defe	NA	NA	NA	NA	NA	NA	NA	NA	NA	NA	NA	NA	15.724	14.931	15.452	NA	NA	NA
19	P60709	Actin, cytoplasn	15.377	8.861	14.292	4.302	15.078	2.959	2.825	3.103	13.331	14.646	13.685	14.804	NA	NA	NA	NA	NA	NA
20	P61254	60S ribosomal p	NA	NA	NA	NA	NA	NA	2.792	2.515	2.915	NA	NA	NA	NA	NA	NA	NA	NA	NA
21	P62805	Histone H4 OS=	15.323	15.749	14.132	1.781	9.793	2.065	11.257	7.042	11.655	15.093	2.968	15.250	8.740	2.951	1.840	2.906	3.174	2.796
22	P62829	60S ribosomal p	NA	NA	NA	NA	NA	NA	5.813	4.803	6.141	NA	NA	NA	6.829	4.901	4.069	17.784	6.350	16.816
23	P63104	14-3-3 protein z	NA	NA	NA	NA	NA	NA	NA	NA	NA	2.003	1.380	1.551	NA	NA	NA	NA	NA	NA
24	P63173	60S ribosomal p	16.287	16.158	15.937	NA	NA	NA	14.971	3.738	14.045	15.148	15.636	16.484	16.448	4.407	15.408	NA	NA	NA
25	P68104	Elongation factc	NA	NA	NA	NA	NA	NA	NA	NA	NA	13.040	1.263	2.296	NA	NA	NA	NA	NA	NA
26	P81605	Dermcidin OS=H	19.505	18.312	17.406	NA	NA	NA	17.338	17.340	15.841	18.796	20.438	19.399	16.912	18.045	16.783	18.257	16.764	17.228
27	P84243	Histone H3.3 OS	12.867	13.202	12.917	1.931	3.585	2.940	4.017	4.556	3.708	3.490	3.848	3.873	4.317	3.647	2.596	14.612	14.616	14.214
28	Q13029	PR domain zinc	23.186	22.233	21.839	NA	NA	NA	21.489	21.669	21.425	13.047	22.057	21.413	NA	NA	NA	NA	NA	NA
29	Q13813	Spectrin alpha c	NA	NA	NA	NA	NA	NA	NA	NA	NA	NA	NA	NA	16.317	16.351	15.255	NA	NA	NA
30	Q15651	High mobility gr	NA	NA	NA	NA	NA	NA	13.371	15.791	11.715	NA	NA	NA	NA	NA	NA	NA	NA	NA
31	Q16777	Histone H2A typ	2.934	3.582	2.828	NA	NA	NA	13.183	14.818	11.994	NA	NA	NA	NA	NA	NA	7.185	17.539	17.332
32	Q16778	Histone H2B typ	1.984	2.462	2.814	1.921	2.679	2.149	2.173	2.662	1.488	NA	NA	NA	2.303	2.918	1.766	14.317	14.422	14.059
33	Q5SSJ5	Heterochromati	NA	NA	NA	NA	NA	NA	NA	NA	NA	NA	NA	NA	NA	NA	NA	14.760	13.866	12.278
34	Q5T7M9	Protein FAM69A	NA	NA	NA	NA	NA	NA	1.027	2.977	13.101	NA	NA	NA	NA	NA	NA	NA	NA	NA
35	Q5U623	Activating trans	NA	NA	NA	NA	NA	NA	NA	NA	NA	11.954	15.656	15.798	NA	NA	NA	NA	NA	NA
36	Q6NZI2	Caveolae-associ	NA	NA	NA	16.004	15.519	15.813	NA	NA	NA	15.479	13.969	15.912	18.161	18.867	17.746	NA	NA	NA
37	Q6ZRV2	Protein FAM83H	NA	NA	NA	NA	NA	NA	NA	NA	NA	NA	NA	NA	NA	NA	NA	17.683	16.125	15.999
38	Q71UI9	Histone H2A.V (	18.077	18.165	17.681	NA	NA	NA	3.074	2.689	2.476	6.194	5.559	5.456	15.596	4.555	16.074	4.240	4.624	5.082
39	Q7L7L0	Histone H2A typ	NA	NA	NA	11.286	14.241	2.965	NA	NA	NA	NA	NA	NA	NA	NA	NA	NA	NA	NA

40	Q86YZ3	Hornerin OS=Hc	NA	NA	NA	NA	NA	NA	14.801	15.744	14.788	NA	NA	NA	14.748	14.243	16.305	14.872	16.094	16.108
41	Q8N257	Histone H2B typ	NA	NA	NA	NA	NA	NA	NA	NA	NA	1.136	1.741	1.981	NA	NA	NA	NA	NA	NA
42	Q93077	Histone H2A typ	NA	NA	NA	NA	NA	NA	2.725	3.675	3.138	NA	NA	NA	NA	NA	NA	NA	NA	NA
43	Q96DR8	Mucin-like prote	17.052	16.083	15.237	NA	NA	NA	NA	NA	NA	NA	NA	NA	NA	NA	NA	NA	NA	NA
44	Q96RW7	Hemicentin-1 O	NA	NA	NA	NA	NA	NA	NA	NA	NA	NA	NA	NA	NA	NA	NA	17.382	16.978	17.039
45	Q96S38	Ribosomal prote	NA	NA	NA	NA	NA	NA	NA	NA	NA	NA	NA	NA	15.598	16.075	15.996	NA	NA	NA
46	Q99878	Histone H2A typ	NA	NA	NA	NA	NA	NA	NA	NA	NA	NA	NA	NA	1.204	2.459	16.310	NA	NA	NA
47	Q9ULS5	Transmembrane	NA	NA	NA	NA	NA	NA	NA	NA	NA	NA	NA	NA	15.070	15.341	15.405	14.965	15.409	15.198
48	Q9UM00	Calcium load-ac	NA	NA	NA	NA	NA	NA	NA	NA	NA	12.647	13.352	15.972	NA	NA	NA	NA	NA	NA

Supplemental Table 5.2. Master protein identifications from CD3 SPPLATed primary human T cells with SILAC abundance ratio Light/Heavy > 1 not present in controls

			Condition A			Condition B			Condition C			Condition D			Condition E			Condition F		
	Master.Pr	Master.Protei	rep1	rep2	rep3	rep1	rep2	rep3	rep1	rep2	rep3	rep1	rep2	rep3	rep1	rep2	rep3	rep1	rep2	rep3
	O60814	Histone H2B t	NA	NA	NA	NA	NA	NA	NA	NA	NA	2.042	2.166	2.167	NA	NA	NA	NA	NA	NA
1	P05107	Integrin beta-	NA	NA	NA	NA	NA	NA	NA	NA	NA	14.764	15.186	14.777	NA	NA	NA	NA	NA	NA
2	P16070	CD44 antigen	NA	NA	NA	NA	NA	NA	NA	NA	NA	16.577	15.619	15.092	NA	NA	NA	NA	NA	NA
3	P16402	Histone H1.3	NA	NA	NA	1.422	2.289	1.703	NA	NA	NA	1.889	1.912	1.853	NA	NA	NA	NA	NA	NA
4	P26373	60S ribosoma	15.795	15.136	14.520	NA	NA	NA	NA	NA	NA	NA	NA	NA	NA	NA	NA	NA	NA	NA
5	P47914	60S ribosoma	NA	NA	NA	NA	NA	NA	NA	NA	NA	1.796	1.583	1.233	NA	NA	NA	NA	NA	NA
6	P60709	Actin, cytopla	15.377	8.861	14.292	4.302	15.078	2.959	2.825	3.1029	13.3305	14.646	13.685	14.804	NA	NA	NA	NA	NA	NA
7	P61254	60S ribosoma	NA	NA	NA	NA	NA	NA	2.792	2.5153	2.9152	NA	NA	NA	NA	NA	NA	NA	NA	NA
8	P63104	14-3-3 protei	NA	NA	NA	NA	NA	NA	NA	NA	NA	2.003	1.380	1.551	NA	NA	NA	NA	NA	NA
9	P68104	Elongation fa	NA	NA	NA	NA	NA	NA	NA	NA	NA	13.040	1.263	2.296	NA	NA	NA	NA	NA	NA
10	Q13029	PR domain zir	23.186	22.233	21.839	NA	NA	NA	21.489	21.6693	21.4245	13.047	22.057	21.413	NA	NA	NA	NA	NA	NA
11	Q15651	High mobility	NA	NA	NA	NA	NA	NA	13.371	15.7908	11.7151	NA	NA	NA	NA	NA	NA	NA	NA	NA
12	Q5T7M9	Protein FAM6	NA	NA	NA	NA	NA	NA	1.027	2.9771	13.1012	NA	NA	NA	NA	NA	NA	NA	NA	NA
13	Q5U623	Activating tra	NA	NA	NA	NA	NA	NA	NA	NA	NA	11.954	15.656	15.798	NA	NA	NA	NA	NA	NA
14	Q7L7L0	Histone H2A t	NA	NA	NA	11.286	14.241	2.965	NA	NA	NA	NA	NA	NA	NA	NA	NA	NA	NA	NA
15	Q8N257	Histone H2B t	NA	NA	NA	NA	NA	NA	NA	NA	NA	1.136	1.741	1.981	NA	NA	NA	NA	NA	NA
16	Q93077	Histone H2A t	NA	NA	NA	NA	NA	NA	2.7247	3.6749	3.1383	NA	NA	NA	NA	NA	NA	NA	NA	NA
17	Q96DR8	Mucin-like pr	17.052	16.083	15.237	NA	NA	NA	NA	NA	NA	NA	NA	NA	NA	NA	NA	NA	NA	NA
18	Q9UM00	Calcium load-	NA	NA	NA	NA	NA	NA	NA	NA	NA	12.647	13.352	15.972	NA	NA	NA	NA	NA	NA

Supplemental Table 6. List of antibodies used in this work

Antigen	Conjugate	Application	Company	Cat.	Dilution
CD3, clone UCHT1 (mouse primary)		Stimulation for Immunoblotting	BioLegend	300402	10 μg/ml
Anti-mouse secondary		Stimulation for Immunoblotting	Biolegend	405301	30 μg/ml
pERK1/2		Immunoblotting	Cell Signaling	4370	1/1000
ERK1/2		Immunoblotting	Cell Signaling	9102	1/1000
p-CYTIP(T280)		Immunoblotting	MRC PPU reagents	SA512- bleed 4	1 μg/ml antibody and 10 μg/ml non-phospho peptide
CYTIP		Immunoblotting	Abcam	96623	1/1000
Cytohesin-1		Immunoblotting	Abcam	151732	1/1000
SNX27		Immunoblotting	Abcam	77799	1/1000
Anti-Sheep secondary	HRP	Immunoblotting	R&D Systems	HAF016	1/10000
Anti-rabbit secondary	HRP	Immunoblotting	Cell Signaling	9212	1/10000
Cytohesin-1		Immunoprecipitation	R&D Systems	AF4807	
Goat IgG		Immunoprecipitation	R&D Systems	AB-108-C	
CD3, clone OKT3		T cell stimulation	ebioscience	16-0037	see figures for concentrations used
CD28, clone CD28.2		T cell stimulation	BD biosciences	555726	see figures for concentrations used
IFNγ		IFNγ ELISA capture antibody	BD biosciences	551221	4 μg/ml
IFNγ	biotin	IFNγ ELISA detection antibody	BD biosciences	554550	1 μg/ml
CD3, clone UCHT1 (mouse primary)		SPPLAT	BD biosciences	555329	10 μg/ml
CD3, clone UCHT1	HRP	SPPLAT	Abcore	AC14-0024-14	10 μg/ml
Anti-mouse secondary	HRP	SPPLAT	Jackson Immuno Rese	115-035-071	50 μg/ml
CD3, clone UCHT1 (mouse primary)		Confocal microscopy	R&D Systems	MAB100	10 μg/ml
CD3, clone UCHT1	AF647	Confocal microscopy	BioLegend	300416	10 μg/ml
Anti-mouse secondary	AF647	Confocal microscopy	BioLegend	405322	30 μg/ml
LFA-1 (CD11a/CD18, clone m24)	AF488	Confocal microscopy	BioLegend	363403	10 μg/ml
Integrin αL (rabbit primary)		Confocal microscopy	Covalab	?	1/5000
anti-rabbit secondary	FITC	Confocal microscopy	Jackson Immuno Rese	?	
Cytohesin-1/2 (mouse primary)		Confocal microscopy	Santa Cruz	sc-166542	1/50
Anti-mouse secondary	CFL488	Confocal microscopy	Santa Cruz	?	1/50

β-actin, clone 2F1-1	AF488	Confocal microscopy	BioLegend	643812	5 μg/ml or 1/100
CYTIP	AF488 (using Lightning-Link® Rapid)	Confocal microscopy	ThermoFisher	PA5-52400	?
CD3, clone SK7	BV605	Flow cytometry	BioLegend	344836	?
CD4, clone OKT4	BV710	Flow cytometry	BioLegend	317439	?
CD8, clone?	BV785	Flow cytometry	BioLegend	301045	
pSTAT5(pY694)	AF647	Flow cytometry	BD biosciences	562076	1 test
CD3, clone UCHT1 (mouse primary)		Flow cytometry	BD biosciences	555329	10 μg/ml
Anti-mouse secondary	HRP	Flow cytometry	Jackson Immuno Rese	115-035-071	50 μg/ml
pERK	AF647(pT202/pY204)	Flow cytometry	BD biosciences	612593	1 test

Supplemental Table 7. Stimulation scheme and antibody conjugation state used in each experiment and condition

Experiment	Stimulation	No IL-7 prime, no TCR activation	No IL-7 prime, TCR activation	IL-7 prime, no TCR activation	IL-7 prime, TCR activation	Negative control	Negative control
**Condition**		**A**	**B**	**C**	**D**	**E**	**F**
**SPLATT**	IL-7 24h prime	-	-	+	+	-	+
	Anti-CD3 antibody	HRP	unconjugated	HRP	unconjugated	-	-
	Cross-linking antibody	-	HRP	-	HRP	HRP	HRP
**Immunofluorescence**	IL-7 24h prime	-	-	+	+	-	+
	Anti-CD3 antibody	AF647	unconjugated	AF647	unconjugated	-	-
	Cross-linking antibody	-	AF647	-	AF647	-	-
**Immunoprecipitation/western blot**	IL-7 24h prime	-	-	+	+	-	+
	Anti-CD3 antibody	+	+	+	+	-	-
	Cross-linking antibody	-	+	-	+	-	-
